# Rapid groundwater decline and some cases of recovery in aquifers globally

**DOI:** 10.1038/s41586-023-06879-8

**Published:** 2024-01-24

**Authors:** Scott Jasechko, Hansjörg Seybold, Debra Perrone, Ying Fan, Mohammad Shamsudduha, Richard G. Taylor, Othman Fallatah, James W. Kirchner

**Affiliations:** 1 Bren School of Environmental Science & Management, University of California, Santa Barbara, Santa Barbara, CA USA; 2 Department of Environmental Systems Sciences, ETH Zürich, Zürich, Switzerland; 3 Environmental Studies Program, University of California, Santa Barbara, Santa Barbara, CA USA; 4 Department of Earth and Planetary Sciences, Rutgers University, New Brunswick, NJ USA; 5 Institute for Risk and Disaster Reduction, University College London, London, UK; 6 Department of Geography, University College London, London, UK; 7 Department of Nuclear Engineering, Faculty of Engineering, King Abdulaziz University, Jeddah, Saudi Arabia; 8 Center for Training and Radiation Protection, Faculty of Engineering, King Abdulaziz University, Jeddah, Saudi Arabia; 9 Swiss Federal Research Institute WSL, Birmensdorf, Switzerland; 10 Department of Earth and Planetary Science, University of California, Berkeley, Berkeley, CA USA

**Keywords:** Hydrology, Environmental sciences

## Abstract

Groundwater resources are vital to ecosystems and livelihoods. Excessive groundwater withdrawals can cause groundwater levels to decline^1–10^, resulting in seawater intrusion^11^, land subsidence^12,13^, streamflow depletion^14–16^ and wells running dry^17^. However, the global pace and prevalence of local groundwater declines are poorly constrained, because in situ groundwater levels have not been synthesized at the global scale. Here we analyse in situ groundwater-level trends for 170,000 monitoring wells and 1,693 aquifer systems in countries that encompass approximately 75% of global groundwater withdrawals^18^. We show that rapid groundwater-level declines (>0.5 m year^−1^) are widespread in the twenty-first century, especially in dry regions with extensive croplands. Critically, we also show that groundwater-level declines have accelerated over the past four decades in 30% of the world’s regional aquifers. This widespread acceleration in groundwater-level deepening highlights an urgent need for more effective measures to address groundwater depletion. Our analysis also reveals specific cases in which depletion trends have reversed following policy changes, managed aquifer recharge and surface-water diversions, demonstrating the potential for depleted aquifer systems to recover.

## Abstract (web summary)

Analysis of about 170,000 monitoring wells and 1,693 aquifer systems worldwide shows that extensive and often accelerating groundwater declines are widespread in the twenty-first century, but that groundwater levels are recovering in some cases.

## Main

Groundwater is the primary water source for many homes, farms, industries and cities around the globe. Unsustainable groundwater withdrawals and changes in climate can cause groundwater levels to fall^1–10^, making groundwater resources less accessible^17^. Global maps of groundwater storage trends are available^7^ from the Gravity Recovery and Climate Experiment (GRACE) satellites, although at a resolution that is too coarse (>150,000 km^2^; ref. ^19^) to detect local changes and inform local management. Measuring multidecadal groundwater-level declines and managing their consequences—including seawater intrusion^11^, land subsidence^12,13^, streamflow depletion^14–16^ and wells running dry^17^—requires in situ groundwater-level measurements from networks of monitoring wells. Such monitoring-well networks have been used at local and regional scales to estimate groundwater recharge^20,21^, characterize streamflow depletion^14^, evaluate the risk of wells running dry^17^ and test whether surface-water diversions^22,23^ or market and policy interventions^24^ have succeeded in slowing groundwater losses. However, in situ groundwater-level observations have rarely been analysed at the global scale because we lack a global compilation of in situ groundwater-level time series.

Here we compile and analyse in situ measurements of groundwater-level trends in about 170,000 monitoring wells. The measurements provide new constraints on the prevalence of rapid and accelerating groundwater-level declines and their correlation with land use and climatic drivers. Furthermore, the measurements highlight individual cases in which groundwater levels have recovered following policy changes^25^ and inter-basin water transfers^26^.

## Local hotspots of groundwater-level changes

**Fig. 1:**
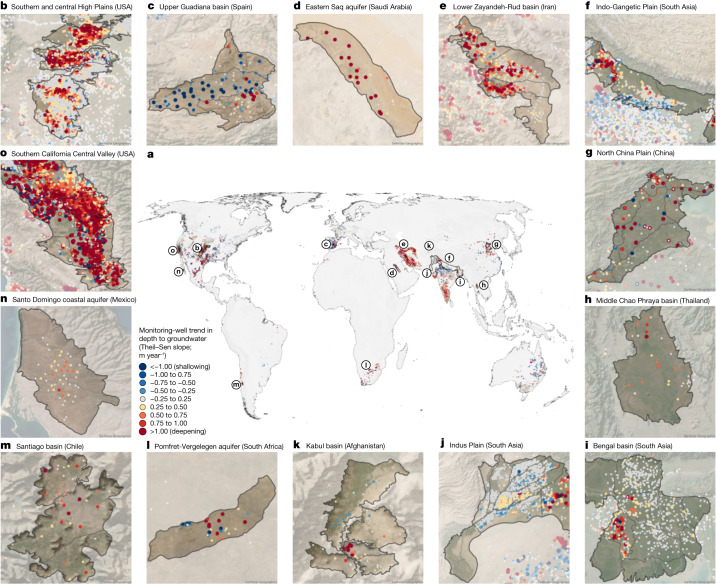
Twenty-first century groundwater-level trends in globally distributed monitoring wells. Each point represents one monitoring well, coloured to represent the Theil–Sen trend of annual median groundwater levels during the twenty-first century. Blue and red points indicate shallowing and deepening, respectively, of groundwater levels over time, with darker colours indicating faster rates. **a**, Spatial distributions of groundwater-level trends in globally distributed monitoring wells. **b**–**o**, Regional maps illustrating the substantial spatial variability in groundwater-level trends. Supplementary Notes 16 and 17 show monitoring wells and their groundwater-level trends at subcontinental scales (Supplementary Note 16) and in 207 individual aquifer systems (Supplementary Note 17). Background imagery shown in **b**–**o** from https://www.arcgis.com/home/item.html?id=10df2279f9684e4a9f6a7f08febac2a9. Source Data

We compiled and quality-controlled groundwater-level time series in monitoring wells from more than 40 countries (see Methods and Supplementary Notes 1 and 2). We calculated twenty-first century trends in depth to groundwater level for about 170,000 monitoring wells with time series that span at least 8 years using Theil–Sen robust regression (Fig. 1; analyses based on alternative regression techniques and on different quality-control thresholds yield similar results; see Supplementary Notes 3, 4, 5 and 6). Positive Theil–Sen slopes indicate deepening groundwater levels (red points in Fig. 1). Trends in groundwater levels often differ substantially from well to well, and local hotspots of groundwater decline can be found even in regions in which nearby groundwater levels are stable or rising, and vice versa (Fig. 1), highlighting the importance of analysing groundwater-level trends at the scales defined by the boundaries of individual aquifer systems.

**Fig. 2:**
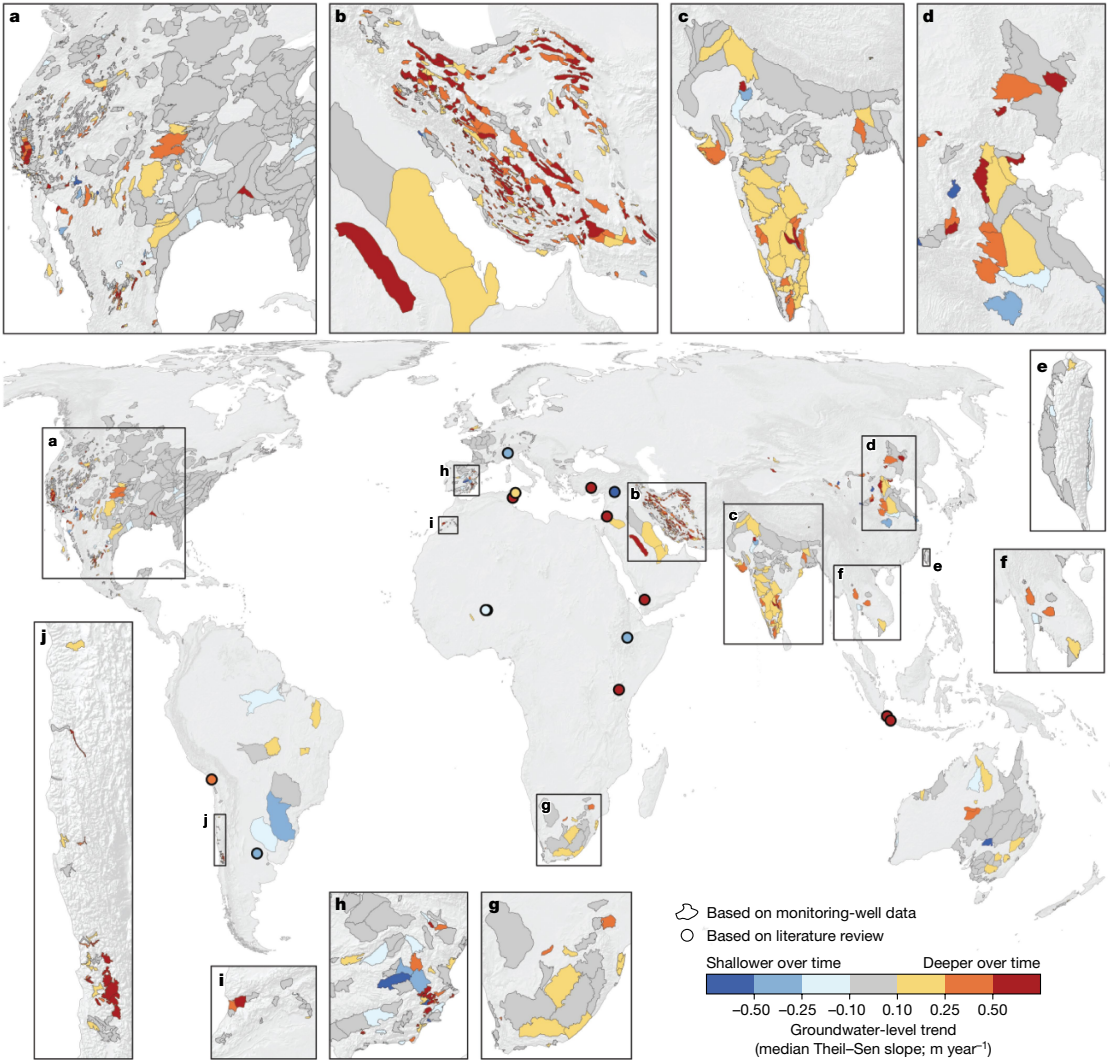
Twenty-first century trends in depth to groundwater in 1,693 globally distributed aquifer systems. Each polygon represents one aquifer system. Dark grey represents aquifer systems in which groundwater levels have been relatively stable (median Theil–Sen slope between −0.1 and 0.1 m year^−1^). Yellow, orange and red represent aquifer systems in which groundwater levels became deeper (median Theil–Sen slope >0.1 m year^−1^). Blue represents aquifer systems in which groundwater levels became shallower (median Theil–Sen slope of <−0.1 m year^−1^). Darker colours indicate faster rates. Circular points mark locations for which we lack monitoring-well data but groundwater-level trends have been documented in the literature, with colours indicating the average of the minimum and maximum literature values (Supplementary Note 15). Statistics describing the spatial variability of groundwater-level trends within individual aquifers are presented in Supplementary Note 23. Median Theil–Sen slopes for all 1,693 aquifer systems are tabulated in Supplementary Note 24. Source Data

To evaluate aquifer-scale groundwater-level trends, we manually delineated the boundaries of 1,693 aquifer systems—areas underlain by one or more aquifers—using maps and descriptions from 1,236 local and regional studies (see Methods and Supplementary Note 7). We calculated aquifer-scale groundwater-level trends as the median of the Theil–Sen slopes of all monitoring wells located within each aquifer system (Fig. 2). Most aquifer-scale groundwater-level trends range from −0.1 to 0.9 m year^−1^ (5th to 95th percentiles), in which negative values represent shallowing groundwater levels and positive values indicate deepening groundwater levels.

Groundwater levels became deeper over time at rates exceeding 0.1 m year^−1^ in 36% of the aquifer systems (617 of 1,693) and exceeding 0.5 m year^−1^ in 12% (210) of them. Aquifer systems that exhibit groundwater-level deepening and are too small to be detected by GRACE satellite observations (for example, southeastern Spain) highlight the value of in situ groundwater-level measurements to complement global-scale insights^5,7,9,19^ made possible by the GRACE (see Methods and Supplementary Note 8).

Groundwater levels became shallower over time faster than −0.1 m year^−1^ in 6% of the aquifer systems (97 of 1,693) and faster than −0.5 m year^−1^ in only 1% (13) of them. Some groundwater-shallowing trends may be explained by reductions in groundwater withdrawals, land-cover changes, managed aquifer recharge projects (for example, in Arizona’s East Salt River basin^22^) and inter-basin surface-water transfers (for example, the Wanjiazhai water diversion to China’s Taiyuan basin^26^).

## Accelerating groundwater-level declines

To place twenty-first century groundwater-level declines into context, we compared them with groundwater-level trends during the late twentieth century (1980–2000); this analysis was possible in 542 of the 1,693 delineated aquifer systems (see Methods and Supplementary Note 9).

**Fig. 3:**
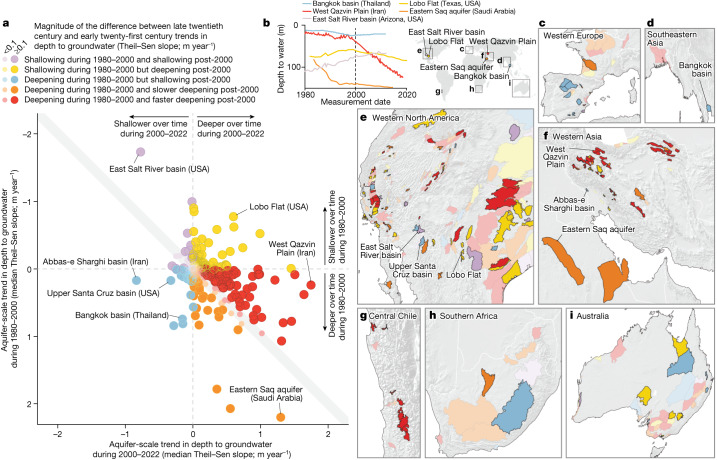
Comparison of aquifer-scale trends in depth to groundwater during the late twentieth and early twenty-first centuries. **a**, Scatter plot of aquifer-scale trends (median Theil–Sen slopes) during 2000–2022 (*x*-axis values) and during 1980–2000 (*y*-axis values). The colour of each point indicates one of the following categories of trends: (1) groundwater levels became shallower during 1980–2000 and continued to become shallower (purple points); (2) groundwater levels became shallower during 1980–2000 but have since become deeper (yellow points); (3) groundwater levels became deeper during 1980–2000 but have since become shallower (blue points); (4) groundwater levels became deeper during 1980–2000 and continued to become deeper but at a slower rate (that is, decelerated deepening; orange points); and (5) groundwater levels became deeper during 1980–2000 and continued to become deeper at a faster rate (that is, accelerated deepening; red points). The intensity of each colour scales with the absolute value (that is, magnitude) of the difference between the late twentieth and early twenty-first century trends in groundwater level (see legend). **b**, Examples of groundwater-level time series illustrating each of our five categories (see legend). **c**–**i**, Maps of aquifer systems categorized by their late twentieth and early twenty-first century trends in groundwater levels (colours correspond to categories in the legend). For an expanded version of this figure, see Supplementary Note 9. Source Data

**Extended Data Fig. 1:**
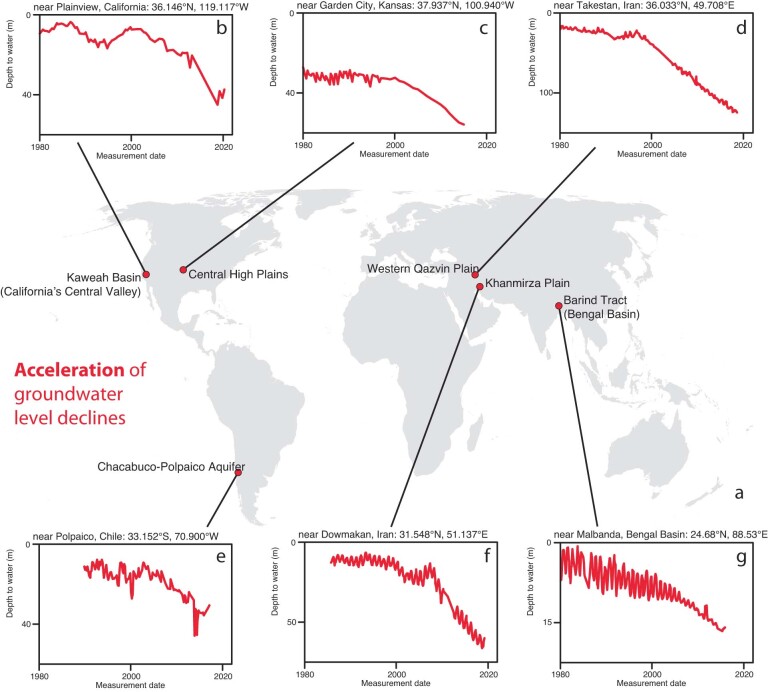
Illustrative examples of individual monitoring wells that record cases for which groundwater levels declined during late twentieth century and continued to decline at a faster rate in the early twenty-first century (that is, accelerated deepening). **a**, Global map depicting the locations of the six monitoring wells (that is, each point represents one monitoring well). The aquifer system that each monitoring well lies in is labelled next to each point. **b**–**g**, Measured groundwater-level variations over time for individual monitoring wells. Each panel presents groundwater-level data for a single monitoring well.

In 30% of these aquifer systems, groundwater-level declines accelerated, with early twenty-first century groundwater-level declines outpacing those of the late twentieth century (the red points in Fig. 3a; see the red time series in Fig. 3b and Extended Data Fig. 1 for illustrative examples). These cases of accelerating groundwater-level declines are more than twice as prevalent as one would expect from random fluctuations in the absence of any systematic trends in either time period (12.5%; *P*-value < 0.001 by the binomial test). Furthermore, among all cases in which groundwater levels declined in both the late twentieth and early twenty-first centuries, declines in the early twenty-first century outpaced those in the late twentieth century much more often than one would expect by chance (163 red points versus 107 orange points in Fig. 3a; *P*-value < 0.001 by the sign test). If we exclude cases in which groundwater-level trends changed by less than 0.1 m year^−1^ between these two periods (that is, considering only points lying outside the grey diagonal band in Fig. 3a), we find that accelerating declines (red points) outnumber decelerating declines (orange points) by a ratio of 5:2 (*P*-value < 0.001 by the sign test). In summary, groundwater-level declines have accelerated in a substantial share of the analysed aquifer systems.

To test for a potential statistical relationship between accelerating groundwater-level declines and climate variability, we analysed precipitation rates over the past four decades (Supplementary Note 10). We show that most (>80%) of the aquifer systems exhibiting accelerating groundwater-level declines also experienced a decline in precipitation over time (that is, lower average annual precipitation during the early twenty-first century than in the late twentieth century). Declines in precipitation can cause groundwater levels to fall as a result of both indirect impacts (for example, increased groundwater abstractions during droughts) and direct impacts (for example, reduced recharge rates during droughts; see ref. ^27^). Our finding—that early twenty-first century precipitation rates were lower than in the late twentieth century in most aquifer systems exhibiting accelerating groundwater-level declines—highlights a potential link between decadal-scale climate variability and accelerating groundwater-level declines. Accelerating groundwater-level declines, regardless of their potential drivers, are likely to also accelerate the consequences of those declines, including land subsidence^12,13^ and wells running dry^17^.

## Slowing and reversing groundwater-level declines

Many previous studies^1–10^ have highlighted groundwater losses, but the potential for slowing or reversing these losses has received less attention. Our analysis of groundwater levels suggests that long-term groundwater losses are neither universal nor inevitable. Specifically, in half (49%) of the 542 aquifer systems in our analysis, groundwater-level declines have decelerated (that is, slowed; orange in Fig. 3; 20%) or reversed (blue in Fig. 3; 16%), or groundwater levels have continued to rise (purple in Fig. 3; 13%).

**Extended Data Fig. 2:**
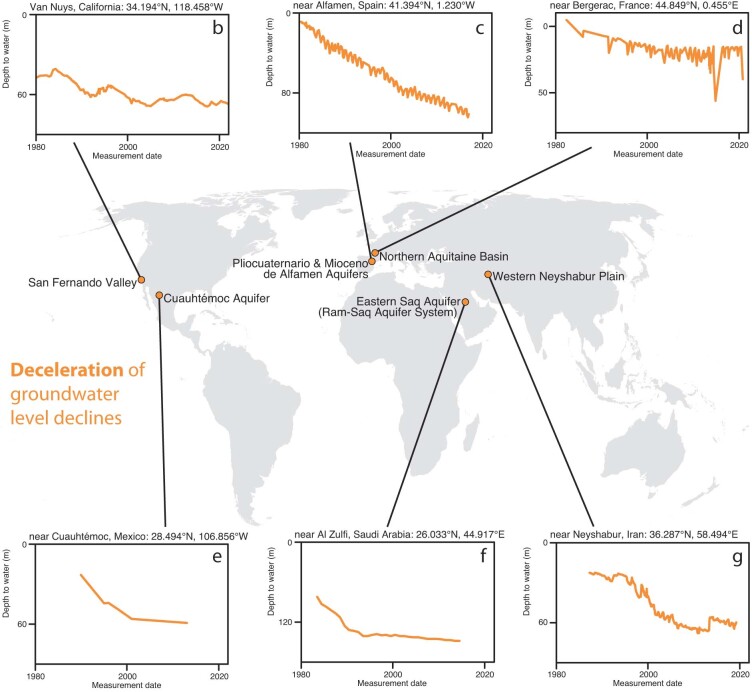
Illustrative examples of individual monitoring wells that record cases for which groundwater levels declined during late twentieth century and continued to decline but at a slower rate in the early twenty-first century (that is, decelerated deepening). **a**, Global map depicting the locations of the six monitoring wells (that is, each point represents one monitoring well). The aquifer system that each monitoring well lies in is labelled next to each point. **b**–**g**, Measured groundwater-level variations over time for individual monitoring wells. Each panel presents groundwater-level data for a single monitoring well.

In 20% of the aquifer systems, groundwater-level deepening has decelerated, as late twentieth century groundwater declines continued in the early twenty-first century, but at a slower rate (the orange points in Fig. 3a; see orange time series in Fig. 3b and Extended Data Fig. 2 for illustrative examples). Although these cases are outnumbered by those for which groundwater declines have accelerated, they demonstrate that it is possible to slow, and potentially even reverse, groundwater-level declines. For example, our analysis shows marked deceleration of groundwater-level deepening in the Eastern Saq aquifer of Saudi Arabia, possibly owing partly to policies designed to reduce agricultural water demands^28^ (see labelled orange point in Fig. 3a, which corresponds to the orange line in Fig. 3b).

**Extended Data Fig. 3:**
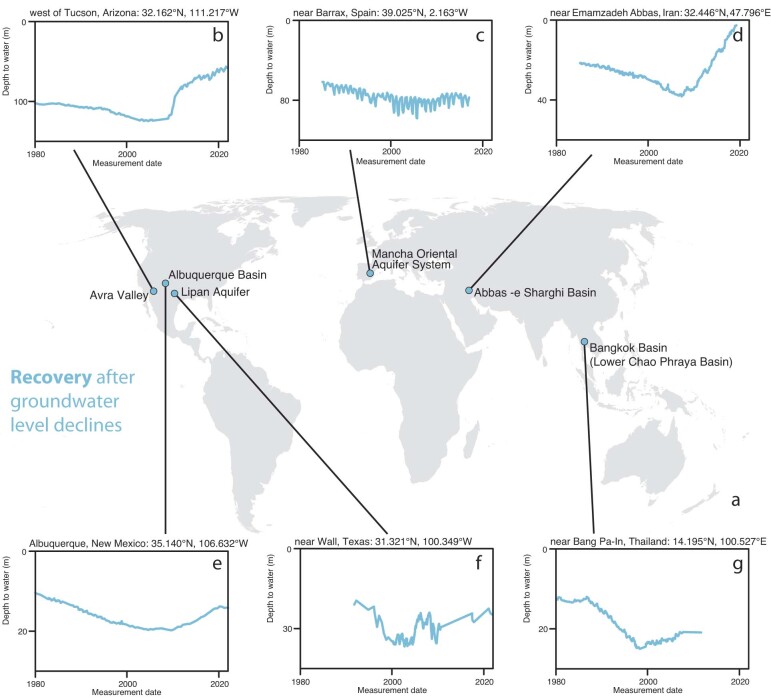
Illustrative examples of individual monitoring wells that record cases for which groundwater levels declined during late twentieth century but rose during the early twenty-first century (that is, cases of groundwater level recovery). **a**, Global map depicting the locations of the six monitoring wells (that is, each point represents one monitoring well). The aquifer system that each monitoring well lies in is labelled next to each point. **b**–**g**, Measured groundwater-level variations over time for individual monitoring wells. Each panel presents groundwater-level data for a single monitoring well.

In 16% of the aquifer systems, groundwater level declines reversed—defined as cases in which groundwater levels declined in the late twentieth century but rose in the early twenty-first century (the blue colours in Fig. 3; see blue time series in Fig. 3b and Extended Data Fig. 3 for examples). For example, in the Bangkok basin (Thailand), groundwater levels deepened during the late twentieth century but shallowed in the early twenty-first century (see labelled blue point in Fig. 3a); this reversal has been attributed^25^ to regulatory measures (groundwater pumping fees and licensing of wells). Another example is Iran’s Abbas-e Sharghi basin, in which twentieth century groundwater-level declines were reversed by the diversion of water to the basin from the Kharkeh Dam^29^. In other areas, groundwater deepening has been reversed following the implementation of managed aquifer recharge projects^22^ (for example, west of Tucson, Arizona; Extended Data Fig. 3). Recharge projects are sometimes only viable where excess surface waters are available, emphasizing the importance of coordinating groundwater and surface-water management^30^. Nevertheless, these examples illustrate that interventions of sufficient scope and scale can reverse declining groundwater trends.

**Extended Data Fig. 4:**
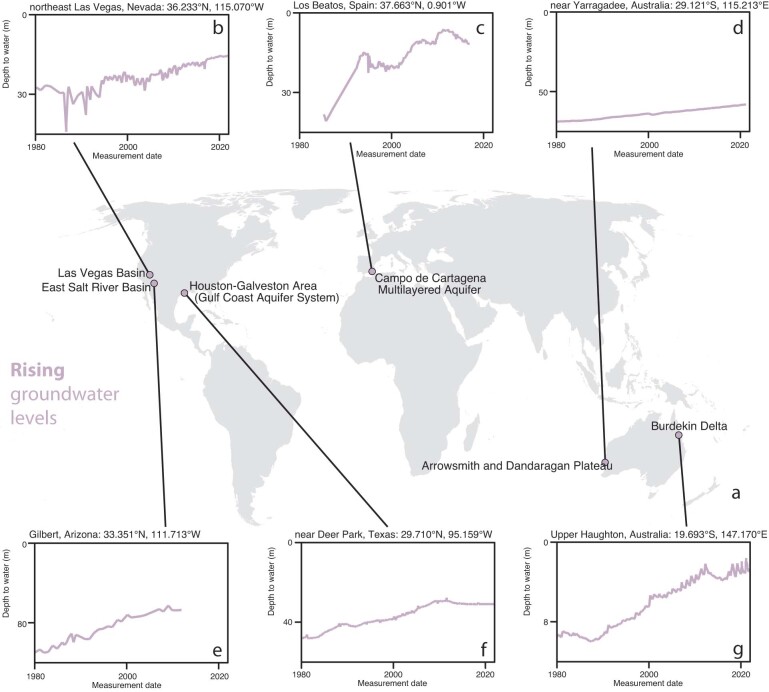
Illustrative examples of individual monitoring wells that record cases for which groundwater levels rose during late twentieth century, and continued to rise during the early twenty-first century. **a**, Global map depicting the locations of the six monitoring wells (that is, each point represents one monitoring well). The aquifer system that each monitoring well lies in is labelled next to each point. **b**–**g**, Measured groundwater-level variations over time for individual monitoring wells. Each panel presents groundwater-level data for a single monitoring well.

In a further 13% of the aquifer systems, groundwater levels rose in both the late twentieth and the early twenty-first centuries (purple colours in Fig. 3; see purple time series in Fig. 3b and Extended Data Fig. 4 for examples). Some of these cases indicate that aquifers that were heavily exploited before 1980 are recovering. Aquifer recovery can potentially ameliorate the consequences of groundwater pumping (for example, land subsidence^31^). In other cases, however, rising groundwater levels can be problematic. For example, rising groundwaters can lead to flooding of coastal cities^32^, waterlogging of farmlands^33^ and salinization of groundwaters and soils^34^. Rising groundwater levels may be driven by reductions in groundwater withdrawals^25^ or increases in recharge rates owing to land clearing^35,36^, irrigation^33^ or managed aquifer recharge^37^. Our aquifer-scale groundwater-level trends can help predict where rising groundwater levels may pose challenges.

Although these examples illustrate that groundwater declines can be slowed or reversed, several caveats must be kept in mind. In general, rates of groundwater-level shallowing are much slower than rates of groundwater-level decline. Of the aquifer systems in Fig. 3 with rising twenty-first century groundwater levels (blue and purple points), only 6% are rising faster than −0.2 m year^−1^. By contrast, of the aquifer systems with deepening twenty-first century groundwater levels (yellow, red and orange points in Fig. 3), 25% are falling faster than 0.2 m year^−1^. Furthermore, across these aquifer systems, the average rate of twenty-first century deepening (0.2 m year^−1^) exceeds the average rate of shallowing (−0.05 m year^−1^) by a factor of four. Thus, rapidly rising groundwater levels are rare, but they demonstrate that aquifer recovery is possible, especially following policy changes^25^, managed aquifer recharge^37^ and inter-basin surface water-transfers^26^.

## Groundwater declines in cultivated drylands

**Fig. 4:**
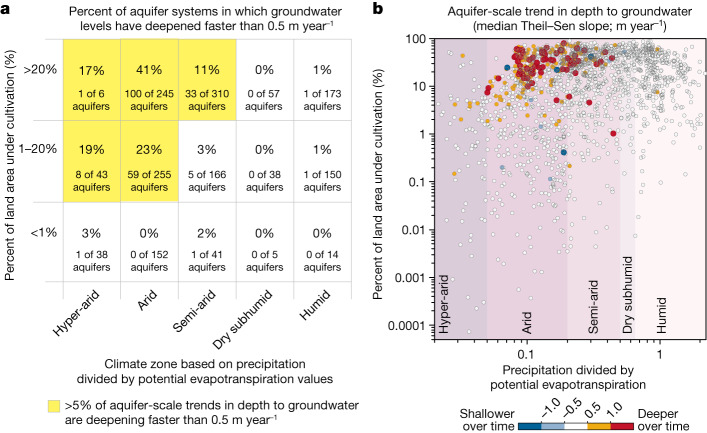
Twenty-first century aquifer-scale trends in depth to groundwater in the context of climate and cultivation. **a**, The percentage of aquifer systems with rapidly deepening groundwater (median Theil–Sen slope steeper than 0.5 m year^−1^) when categorized by climate conditions and cropland prevalence. Aquifer systems with rapidly deepening groundwater are most common in hyper-arid, arid and semi-arid climate zones (see categories on the *x* axis) and where a larger proportion of land is under cultivation (see categories on the *y* axis). **b**, Scatter plot of aquifer-scale average annual precipitation divided by potential evapotranspiration^39^, and the percentage of land area under cultivation^40^ (estimated for the year 2015). The colour of each point represents the twenty-first century aquifer-scale groundwater-level trend (median Theil–Sen slope). Blue and red points indicate shallowing and deepening, respectively, of groundwater, with darker colours indicating faster rates. Background shades represent climate zones classified by annual precipitation divided by potential evapotranspiration (that is, *x*-axis values). Several aquifer systems are absent from this plot because either no land is under cultivation (incompatible with the log scale of the *y* axis) or precipitation divided by evapotranspiration values fall outside the shown range of *x*-axis values. For alternative versions of this figure showing these aquifer systems, see Supplementary Note 11. Source Data

Many of the aquifer systems with declining twenty-first century groundwater levels (Fig. 2) underlie drylands, defined^38^ as areas in which average precipitation divided by potential evapotranspiration is less than 0.65. Rapidly deepening groundwater levels (faster than 0.5 m year^−1^) are found in 11%, 24% and 8% of aquifers in climate zones classified^38^ as hyper-arid, arid and semi-arid, respectively. Notably, aquifer systems with rapidly deepening groundwater levels are virtually absent (<1%) in humid and dry subhumid climate zones. Our 1,693 aquifer-scale groundwater-level trends exhibit a moderately strong rank correlation with precipitation divided by potential evapotranspiration^39^ (Spearman *ρ* = −0.40, *P*-value < 0.001; Supplementary Note 11 and Methods), implying that groundwater deepening is more common in drier climates (Fig. 4). As well as rapid groundwater-level declines, we also find that accelerating groundwater-level declines are more common in drier climates, especially underlying cultivated lands (Supplementary Note 9), probably reflecting greater reliance on groundwater for irrigation.

Irrigation is estimated to account for 70% of global groundwater withdrawals^18^. A lack of high-resolution, ground-truthed data quantifying groundwater withdrawals for irrigation precludes statistical tests of their correlation with groundwater-level changes over time. However, using high-resolution global land cover data^40^, we can test for statistical relationships between land-use patterns and groundwater trends (Fig. 4). Aquifer systems with rapidly deepening groundwater levels (>0.5 m year^−1^) are relatively common (17%) where more than one-fifth of the land surface is cultivated, but are virtually absent (0.8%) where cultivation accounts for <1% of the land surface. Across the 1,693 aquifer systems, rates of groundwater-level deepening are significantly correlated with the proportion of land under cultivation^40^ (Spearman *ρ* = 0.17, *P*-value < 0.001; Fig. 4). This statistical relationship becomes stronger when we account for the correlation between cultivation and climatic aridity (partial rank correlation coefficient = 0.32, *P*-value < 0.001; see Supplementary Note 11). Our analyses demonstrate that rapid groundwater declines are most common in cultivated drylands.

Groundwater losses from dryland aquifers pose management challenges. Aquifer recharge is typically slow in drylands^41^, meaning that depleted dryland aquifers will generally take longer to recover than aquifers in wetter climates^42^, except where recharge rates are artificially increased (for example, seepage from unlined canals in the Indus basin^33^). Moreover, groundwater is often the sole source of perennial drinking water for communities in drylands. As groundwater levels become deeper, shallower wells can run dry^17^, compromising local water access. Even where groundwater levels remain stable, groundwater withdrawals can deplete the flow of nearby streams by reducing natural seepage of groundwater to rivers, or even inducing streamwater leakage into underlying aquifers (see discussion of ‘capture’ by ref. ^43^). Indeed, leakage from surface waters may replenish pumped aquifers and stabilize groundwater levels at the expense of streamflow. The prevalence of rapid and accelerating groundwater declines in cultivated drylands suggests that, even if management strategies are in place, they have often been insufficient—either in concept or in implementation—to slow or reverse groundwater depletion.

## Depleting and recovering groundwater resources

Our analysis of groundwater-level measurements demonstrates that: (1) groundwater levels are declining rapidly (>0.5 m year^−1^) in many regions (Fig. 2); (2) groundwater declines are accelerating in many aquifer systems around the world (Fig. 3); and (3) both rapid and accelerating groundwater declines are particularly evident in aquifers underlying cultivated drylands (Fig. 4 and Supplementary Notes 9 and 11). Our analysis also identifies cases in which late twentieth century groundwater declines have been reversed in the early twenty-first century (blue points in Fig. 3). However, cases of rapidly rising groundwater levels remain outnumbered by cases of rapidly deepening groundwater levels.

Our results indicate that twenty-first century realities—including climatic trends, hydrogeologic conditions, groundwater withdrawal rates, land uses and management approaches—have resulted in widespread, rapid and accelerating groundwater-level declines. Nevertheless, the compiled in situ observations also capture numerous cases in which declines in groundwater levels have slowed, stopped or reversed following intervention (for example, implementation of regulatory measures^25^). Although our work represents the most extensive analysis of groundwater-level monitoring records so far, it does not cover the globe (see Methods section entitled ‘Limitations’). Further, analysed monitoring wells do not represent a randomized sample of global wells and we are only able to analyse groundwater level trends where monitoring data are available. Global maps of groundwater storage changes from GRACE satellite observations^7^ suggest that groundwater stores are declining in some regions in which monitoring data are not publicly available and, thus, cannot be evaluated here. GRACE data are also important for characterizing impacts of climate change and variability^9,19,44–46^ and evaluating global hydrologic models^47^. Evaluating such models is important because they are widely used to estimate groundwater depletion (see ref. ^6^ and Table 3 in ref. ^48^). Our compilation of monitoring-well data could facilitate future efforts to reconcile GRACE-based, model-based and piezometric-based groundwater time series (see refs. ^49,50^). Combining these diverse data products—and thus exploiting both the high spatial resolution of monitoring-well networks and the global coverage of GRACE^7,9,19^ and hydrologic models^2,3,6,16,48^—may yield new insights into the causes, consequences and spatial patterns of groundwater depletion.

Groundwater depletion can threaten ecosystems and economies. Specifically, groundwater depletion can damage infrastructure through land subsidence^12,13^, impair fluvial ecosystems through streamflow depletion^14–16^, jeopardize agricultural productivity^51^ and compromise water supplies as wells run dry^17^. Our methodologically consistent analysis of groundwater-level trends across 1,693 globally distributed aquifer systems demonstrates widespread, rapid and accelerating twenty-first century groundwater-level declines, particularly in cultivated drylands.

Our analysis also documents cases for which groundwater declines have slowed or reversed after: (1) the implementation of groundwater policies; (2) the alleviation of groundwater demand by means of surface-water transfers; or (3) the addition of groundwater storage following managed aquifer recharge projects. To address the growing problem of global groundwater depletion, these kinds of success stories would need to be replicated in dozens of aquifer systems with declining groundwater levels. Thus, our analysis illustrates the potential for depleted aquifers to recover, while demonstrating how much work remains to be done to protect groundwater resources. By documenting global hotspots of groundwater-level decline and recovery, this analysis can inform efforts to address rapid and accelerating groundwater depletion.

## Methods

### Delineating global aquifer systems based on literature review of local studies

For each country in our study, we consulted published accounts of local-scale studies^52–1288^ (Supplementary Note 7) to delineate 1,693 study areas, each underlain by one or more aquifers and/or low-permeability geologic formations that are, collectively, referred to as an ‘aquifer system’. Each aquifer system was delineated by consulting maps and reading descriptions within local-scale reports. Specific steps applied to delineate the boundaries of each aquifer system are detailed in Supplementary Note 7.

### Downloading groundwater-level data

Our study focuses on more than 40 countries for which we compiled monitoring-well data. We analysed groundwater-level time series derived from numerous data repositories (dataset-specific details are available in Supplementary Note 1; some of these datasets are described in refs. ^1289–1297^). The compiled groundwater-level databases span different time intervals and have different measurement frequencies (see heat map plot and global maps showing monitoring-well time series durations and measurement frequencies in Supplementary Note 12).

### Quality controlling groundwater-level time series

We completed five pre-processing steps before analysing groundwater-level data. First, we identified replicate groundwater-level measurements, defined as cases in which an identical measurement date and an identical groundwater-level measurement were reported from the same monitoring well; in these cases, we retain only one of these replicates. Second, we identified cases in which several groundwater-level measurements from the same monitoring well reported identical measurement dates. In these cases, we calculated the median among all groundwater-level measurements sharing the same measurement date and the adjacent points in the time series (that is, the median of the group of measurements with identical dates and the measurements immediately preceding and following the same-date measurements); we then kept only the single water-level measurement whose value was closest to this calculated median (Supplementary Note 2). Third, we excluded extreme values of depth to groundwater (that is, >1,000 m and <−1,000 m) and implausibly high groundwater elevations (that is, >8,000 m above sea level). Fourth, we excluded groundwater-level measurements with values of ‘999’, ‘−9,999’ or ‘0’, because some databases used these values as a code for missing measurements (see figures in Supplementary Note 2). Fifth, we excluded outlier values detected by a machine-learning algorithm^1298^ (based on an additive regression model^1299^; for details, see Supplementary Note 2.1). This algorithm was applied to each monitoring well with more than 15 groundwater-level measurements, yielding a prediction for each time step and its 99% confidence interval. We defined points to be outliers and excluded them if they fell outside the range defined by the predicted groundwater level ±0.75 times this confidence interval. If a large number of measurements within a monitoring well’s time series were classified as outliers, we excluded the entire time series from our analysis (in which a ‘large number of measurements’ is defined as cases for which there were at least five outliers identified by the machine-learning algorithm and for which these outliers comprise >1% of all measurements in the time series; for visualization, see schematics in Supplementary Note 2). Among the approximately 170,000 monitoring wells presented in Fig. 1, only about 12% had one or more outlier points removed by means of this machine-learning approach, highlighting that this machine-learning approach affected only a small proportion of consulted monitoring wells. Furthermore, a comparison of aquifer-scale trends in depth to groundwater with versus without the use of a machine-learning-based outlier-exclusion procedure suggests that our machine-learning approach had no substantial influence on our findings (see Supplementary Note 13).

### Flagging groundwater-level measurements based on rapid increases or decreases

After excluding potential outliers (through the steps outlined in the previous paragraph), we calculated each monitoring well’s annual median groundwater levels for each calendar year with at least one measurement. We then visually inspected plots of annual median groundwater levels over time. On visual inspection, we noted that a small number of monitoring wells show ‘spikes’ in their annual groundwater-level time series, in which a ‘spike’ is defined as a high-magnitude (absolute value > 20 m year^−1^) groundwater-level change followed directly by another high-magnitude groundwater-level change in the opposite direction (for example, a high-magnitude groundwater-level deepening trend between two adjacent points in the time series, directly followed by a high-magnitude groundwater-level shallowing trend between two adjacent points). We flagged these data points as potentially suspect. The first or last point in each time series was also flagged if it differed by more than 20 m year^−1^ from the second or next-to-last point. We compared groundwater-level trends with and without these flagged points and observed only trivial differences (Supplementary Note 5: ‘Similar aquifer-scale trends obtained with and without flagged measurements’). The results presented in the main text (for example, Fig. 1) derive from annual median groundwater-level time series that exclude the flagged measurements.

### Statistical analyses of twenty-first century groundwater-level trends (Figs. 1 and 2)

To evaluate groundwater-level trends since the year 2000, we excluded all previous measurements. Next, we excluded all monitoring wells for which the earliest and most recent annual medians were separated by fewer than 8 years. We calculated trends in annual median groundwater levels for all monitoring wells that met these minimum criteria for analysis (for a similar method, see ref. ^1288^).

Some data sources report groundwater levels as elevations (metres above sea level) and others report them as depth to groundwater (metres below the land surface, or below the top of the well). In cases in which both were reported, we used the depth to groundwater data. If groundwater levels were only reported as elevations, we reversed the signs of the calculated trends, to obtain trends in depth to groundwater.

Our results in the main text are based on Theil–Sen regression slopes^1300,1301^ but we also applied several different regression techniques, including ordinary least squares, iteratively reweighted least squares^1302–1304^ and RANSAC (or random sample consensus)^1305^, which yielded comparable results (Supplementary Note 3; for non-parametric regression techniques, see Supplementary Note 4 and ref. ^1306^). We present our results as trends in depth to groundwater, meaning that positive slopes represent groundwater levels becoming deeper over time. We calculated an aquifer-scale groundwater-level trend for each aquifer system by taking the median of the Theil–Sen slopes of all monitoring wells within its boundaries (Fig. 2).

### Comparing groundwater-level trends between the late twentieth and early twenty-first centuries (Fig. 3)

To contextualize twenty-first century trends in depth to groundwater, we identified monitoring wells with sufficient data during two periods: the late twentieth century (1980–2000) and the early twenty-first century (2000–2022). Here well time series are ‘sufficient’ if their earliest and latest annual medians are separated by at least 8 years within a given time interval (that is, 1980–2000 or 2000–2022). There are 45,911 monitoring wells in the compiled dataset with sufficient groundwater-level data for trend analyses during both periods. For these monitoring wells, we calculated Theil–Sen trends in depth to groundwater for the late twentieth century. Next, we grouped monitoring wells located within the same aquifer system and calculated aquifer-scale trends for the late twentieth century (medians of the Theil–Sen slopes for all wells in each system; that is, *y*-axis values presented in Fig. 3a). Only aquifer systems with at least five monitoring wells for both time periods (1980–2000 and 2000–2022) satisfying the aforementioned requirements were used to compare late twentieth century and early twenty-first century trends in depth to groundwater. Last, we assigned each aquifer system to one of five categories based on its late twentieth century and early twenty-first century trends in depth to groundwater: (1) groundwater levels became shallower during 1980–2000 and continued to become shallower (purple points in Fig. 3a); (2) groundwater levels became shallower during 1980–2000 but have since become deeper (yellow points in Fig. 3a); (3) groundwater levels became deeper during 1980–2000 but have since become shallower (blue points in Fig. 3a); (4) groundwater levels became deeper during 1980–2000 and continued to become deeper but at a slower rate (that is, decelerated deepening; orange circles in Fig. 3a); and (5) groundwater levels became deeper during 1980–2000 and continued to become deeper at a faster rate (that is, accelerated deepening; red circles in Fig. 3a). Further details are available in Supplementary Note 9.

### Geospatial analysis of potential explanatory variables (Fig. 4)

To test for statistical relationships between the spatial distributions of environmental conditions and groundwater-level trends, we downloaded two geospatial datasets: (1) long-term mean annual precipitation divided by potential evapotranspiration (the ‘CGIAR-CSI Global-Aridity and Global-PET Database’; ref. ^39^) and (2) the proportion of land area under cultivation (estimated for the year 2015; ref. ^40^). Next, we averaged each of these geospatial datasets over each of the 1,693 aquifer systems (Fig. 4). We calculated rank correlations between twenty-first century aquifer-scale groundwater-level trends and both of the potential explanatory variables (namely, (1) long-term mean annual precipitation divided by potential evapotranspiration and (2) the proportion of land area under cultivation). We also used multiple regression on the rank transforms of these explanatory variables to account for their covariation (Supplementary Note 11).

### Limitations

Our analyses are based on the best available measurements but nonetheless have limitations. Here we detail some of these limitations and evaluate how some may affect our main conclusions.

- Although we have used several steps, as outlined above, to detect and remove outliers, we cannot independently verify the accuracy of all groundwater-level time series. Nevertheless, our analysis is based on several layers of robust estimation (for example, Theil–Sen regression on annual medians), minimizing its sensitivity to unreliable data.
- Groundwater-level data from individual monitoring wells span different time intervals and have different measurement frequencies, as detailed in Supplementary Note 12. Furthermore, about 41% of the analysed monitoring wells have discontinuous time series of annual groundwater levels (for which ‘discontinuous’ time series are defined as those lacking a groundwater-level measurement for at least one of the calendar years that lie between the earliest and most recent twenty-first century groundwater-level measurements; for an example of a discontinuity in an annual groundwater-level time series, see Supplementary Fig. 3c).
- We could not obtain groundwater-level data for many countries around the globe and our conclusions are only directly applicable where we have data. GRACE satellite data^1307–1311^ suggest that groundwater storage has declined in some of the areas in which we lack monitoring-well data (Supplementary Note 8). Further, simulation results from a global model suggest that substantial groundwater depletion may have occurred in some of the countries in which we lack monitoring-well data, so groundwater-level deepening may be even more widespread than our results indicate (refs. ^16,1312^; Supplementary Note 14). We reviewed published and grey literature^20,427,802,1282,1313–1356^ to obtain groundwater-level trends for some of the countries in which we lack monitoring-well data (that is, point data in Fig. 2; details available in Supplementary Note 15).
- We highlight that monitoring wells are not distributed evenly across each aquifer system. Consequently, some locations within aquifer systems are not captured by compiled monitoring-well data (see discussion of Dhaka (Bangladesh) in Supplementary Note 15). The aquifer-scale trends that we present in the main text (Figs. 2–4) do not provide insights into the spatial patterns of groundwater-level trends within individual aquifer systems. The high variability in monitoring-well densities within aquifer systems, as well as the substantial variability in groundwater-level trends even among co-located monitoring wells, are presented in a suite of maps for individual aquifer systems in Supplementary Notes 16 and 17. Specifically, our analysis demonstrates that groundwater-level trends can vary greatly among wells within individual aquifer systems (Fig. 1 and Supplementary Notes 16 and 17), implying that local-scale groundwater-level declines may be even more widespread than our Fig. 2 suggests (Supplementary Note 18). Some of the variability in groundwater-level trends among co-located wells may be partly explained by differences in the depths of nearby monitoring wells, as shallow and deep aquifers can have different groundwater-level trends (see Supplementary Note 19).
- We stress that groundwater-level trends may differ between deeper and shallower wells (for example, ref. ^1357^) owing to, for example, differences in the depths of nearby wells used to extract groundwater and differences in storage coefficients between unconfined and confined aquifers (see, for example, refs. ^1358,1359^). Steep groundwater-level trends—both upward and downward—are more common in deeper wells than in shallower wells, possibly due in part to the greater prevalence of confined conditions at deeper depths (discussion and analyses available in Supplementary Note 19). 2D geologic data are available at the global scale^1360^, but an accurate high-resolution 3D hydrogeologic dataset remains unavailable for the globe, meaning that key hydrogeologic conditions (for example, whether the monitoring well captures unconfined versus confined conditions) cannot be ascribed for deep versus shallow wells at the global scale.
- We highlight that our approach to delineating boundaries for individual aquifer systems—although based on local-scale studies—potentially introduces inconsistencies, because local norms for delineating aquifer-system boundaries may differ. Further, some (16%) of the 170,000 monitoring wells fall outside the boundaries of the aquifer systems delineated here and, therefore, are excluded from our aquifer-scale statistical analyses. We present groundwater-level trends for monitoring wells both within and outside aquifer-system boundaries in a series of regional-scale maps (Supplementary Note 16).
- It is possible that some of monitoring-well-based time series may be truncated where the monitoring well itself has run dry (see ref. ^1361^), possibly excluding monitoring wells located in areas experiencing rapid groundwater depletion. We analysed monitoring-well depths and depth to groundwater data for 72,000 wells and conclude that it is possible that a small proportion of the groundwater-level time series was truncated owing to well desiccation (see Supplementary Note 20). Thus, rapid and accelerating twenty-first century groundwater-level deepening may be even more prevalent than our analysis indicates.
- Our main-text results are based on annual median groundwater levels. However, we acknowledge that trends in depth to groundwater can differ when based on measurements made during specific seasons (for example, long-term trends in pre-monsoon depth to groundwater can differ from long-term trends in post-monsoon depth to groundwater; see ref. ^1362^). We highlight that trends in season-specific groundwater levels may differ from trends in annual median groundwater levels (as presented in Fig. 1), especially where intra-annual groundwater-level variability is changing over time (for example, time series from the Bengal basin in Supplementary Note 21; see also the time series presented in refs. ^21,1363,1364^).
- The compiled groundwater-level time series do not allow us to infer trends over longer (for example, centennial-scale) time intervals. In some areas, substantial groundwater-level changes took place long before the four decades that we focus on here. For example, there is evidence^1365,1366^ that substantial accumulation occurred during the twentieth century in parts of South Asia and that groundwater levels were much deeper at the start of the twentieth century than they are today (see, specifically, Fig. 3b in ref. ^1365^). Some aquifer systems in our dataset, for example, may have been heavily depleted during the mid-twentieth century, but have exhibited relatively stable groundwater levels (or even shallowing groundwater-level trends) during the twenty-first century. Given the potential for such cases, we make no claim that stable twenty-first century groundwater levels necessarily imply a lack of previous or continuing disturbance.
- We do not make claims about aquifer-specific drivers behind rapid and accelerating groundwater declines (although we do make note of case studies in the literature that have identified important drivers; for example, ref. ^25^). We acknowledge that groundwater abstractions can perturb flow systems and, in many cases, deplete aquifers. Many of the aquifer systems exhibiting rapid groundwater-level declines are being accessed by wells, as evidenced by recorded well-completion events throughout the early twenty-first century (Supplementary Note 22; data described in refs. ^17,1367–1369^) and by regional-scale research^108,1370,1371^. Further, we acknowledge that climate variability and change can have both direct impacts on groundwater levels (such as through changes in groundwater recharge owing to, for example, changes in temporal variability in precipitation) and also indirect impacts on groundwater levels (for example, through changes in groundwater demand in response to climate variability, such as increased groundwater withdrawals during drier time intervals; see ref. ^27^). Available precipitation data^1372,1373^ suggest that most of the aquifer systems characterized as exhibiting accelerating groundwater-level declines (that is, red points in Fig. 3) are situated in areas in which early twenty-first century annual precipitation rates were lower than late twentieth century annual precipitation rates (Supplementary Note 10), highlighting that, at a minimum, we cannot rule out an influence of climate variability (direct or indirect) on groundwater-level changes over time.

## Online content

Any methods, additional references, Nature Portfolio reporting summaries, source data, extended data, supplementary information, acknowledgements, peer review information; details of author contributions and competing interests; and statements of data and code availability are available at 10.1038/s41586-023-06879-8.

### Supplementary information

Supplementary Information

Peer Review File

### Source data

Source Data Fig. 1–4

## Data Availability

Annual groundwater-level data are available for download in all cases for which we have received permission from a database manager to post data (data are available from Zenodo (10.5281/zenodo.10003697) and CUAHSI HydroShare (https://www.hydroshare.org/resource/da946dee3ada4a67860d057134916553/)); these datasets include groundwater-level data for: Afghanistan^1289^, Austria, Belgium, Brazil, Bulgaria, Canada (Alberta, British Columbia, Manitoba, Northwest Territories, Ontario, Prince Edward Island, Saskatchewan, Yukon), China^1290^, Croatia, Czech Republic, Denmark, France^1291^, Germany, Guam, Ireland, Israel, Italy, Latvia, Lithuania, New Zealand, Norway, Paraguay, Poland, Slovenia, Sweden, Switzerland and the USA (Groundwater Ambient Monitoring and Assessment Program, U.S. Geological Survey’s (USGS) National Water Information System and the Texas Water Development Board). The databases for which we have received written permission to post annual groundwater-level data encompass 59% of annual groundwater-level data analysed here (specifically, we received permission to post 66% (*n* = 4,170,802 of *n* = 6,314,793) of all annual ‘depth to groundwater’ data and 18% (*n* = 190,879 of *n* = 1,049,502) of all ‘groundwater elevation’ data). These datasets are specified in Supplementary Table 1 (see column entitled ‘Written permission received to post annual groundwater-level data’). Source data for each of the main-text figures are available here. Supplementary tables associated with this work are available at 10.5281/zenodo.10003697. Geospatial data for the 1,693 aquifer systems studied here are available from CUAHSI HydroShare (https://www.hydroshare.org/resource/73834f47b8b5459a8db4c999e6e3fef6/) and Zenodo (10.5281/zenodo.10003697). Source data are provided with this paper.

## Code availability

Analyses presented here do not depend on specific code; the approach can be reproduced following the procedures described in the Methods section.

## References

[1] Konikow LF, Kendy E (2005). Groundwater depletion: a global problem. Hydrol. J..

[2] Wada Y (2010). Global depletion of groundwater resources. Geophys. Res. Lett..

[3] Gleeson T, Wada Y, Bierkens MF, Van Beek LP (2012). Water balance of global aquifers revealed by groundwater footprint. Nature.

[4] Werner AD (2013). An initial inventory and indexation of groundwater mega-depletion cases. Water Resour. Manag..

[5] Famiglietti JS (2014). The global groundwater crisis. Nat. Clim. Change.

[6] Döll P, Müller Schmied H, Schuh C, Portmann FT, Eicker A (2014). Global‐scale assessment of groundwater depletion and related groundwater abstractions: combining hydrological modeling with information from well observations and GRACE satellites. Water Resour. Res..

[7] Richey AS (2015). Quantifying renewable groundwater stress with GRACE. Water Resour. Res..

[8] Alley, W. M. & Alley, R. *High and Dry: Meeting the Challenges of the World’s Growing Dependence on Groundwater* (Yale Univ. Press, 2017).

[9] Rodell M (2018). Emerging trends in global freshwater availability. Nature.

[10] Scanlon BR (2023). Global water resources and the role of groundwater in a resilient water future. Nature Rev. Earth Environ..

[11] Werner AD (2013). Seawater intrusion processes, investigation and management: recent advances and future challenges. Adv. Water Res..

[12] Shirzaei M (2021). Measuring, modelling and projecting coastal land subsidence. Nat. Rev. Earth Environ..

[13] Herrera-García G (2021). Mapping the global threat of land subsidence. Science.

[14] Barlow, P. M. & Leake, S. A. Streamflow depletion by wells—understanding and managing the effects of groundwater pumping on streamflow. U.S. Geological Survey Circular 1376. 10.3133/cir1376 (2012).

[15] Döll P (2012). Impact of water withdrawals from groundwater and surface water on continental water storage variations. J. Geodyn..

[16] de Graaf IE, Gleeson T, Sutanudjaja EH, Bierkens MF (2019). Environmental flow limits to global groundwater pumping. Nature.

[17] Jasechko S, Perrone D (2021). Global groundwater wells at risk of running dry. Science.

[18] Margat, J. & van der Gun, J. *Groundwater Around the World: A Geographic Synopsis* (CRC, 2013).

[19] Rodell M, Reager JT (2023). Water cycle science enabled by the GRACE and GRACE-FO satellite missions. Nat. Water.

[20] Cuthbert MO (2019). Observed controls on resilience of groundwater to climate variability in sub-Saharan Africa. Nature.

[21] Shamsudduha M (2022). The Bengal Water Machine: quantified freshwater capture in Bangladesh. Science.

[22] Scanlon BR, Reedy RC, Faunt CC, Pool D, Uhlman K (2016). Enhancing drought resilience with conjunctive use and managed aquifer recharge in California and Arizona. Environ. Res. Lett..

[23] Long D (2020). (2020). South-to-North Water Diversion stabilizing Beijing’s groundwater levels. Nat. Commun..

[24] Ayres AB, Meng KC, Plantinga AJ (2021). Do environmental markets improve on open access? Evidence from California groundwater rights. J. Political Econ..

[25] Buapeng, S. & Foster, S. Controlling groundwater abstraction and related environmental degradation in metropolitan Bangkok – Thailand. World Bank Case Profile Collection No. 20. https://documents1.worldbank.org/curated/en/750761468304831965/pdf/518250BRI0Box31GWMATE1CP1201Bangkok.pdf (World Bank, 2008).

[26] Tang W (2022). Land subsidence and rebound in the Taiyuan basin, northern China, in the context of inter-basin water transfer and groundwater management. Remote Sens. Environ..

[27] Taylor RG (2013). Groundwater and climate change. Nat. Clim. Change.

[28] Baig, M. B., Alotibi, Y., Straquadine, G. S. & Alataway, A. in *Water Policies in MENA Countries* (ed. Zekri, S.) 135–160 (Springer, 2020).

[29] Karimi H, Alimoradi S (2017). Impacts of water transfer from Karkheh Dam on rising of groundwater in Dasht-e-Abass Plain, Ilam Province. Res. Earth Sci..

[30] Winter, T. C., Harvey, J. W., Franke, O. L. & Alley, W. M. Ground water and surface water: a single resource. U.S. Geological Survey Circular 1139. 10.3133/cir1139 (1998).

[31] Li MG (2021). Effects of groundwater exploitation and recharge on land subsidence and infrastructure settlement patterns in Shanghai. Eng. Geol..

[32] Rotzoll K, Fletcher CH (2013). Assessment of groundwater inundation as a consequence of sea-level rise. Nat. Clim. Change.

[33] Qureshi AS, McCornick PG, Qadir M, Aslam Z (2008). Managing salinity and waterlogging in the Indus Basin of Pakistan. Agric. Water Manag..

[34] Foster SSD, Chilton PJ (2003). Groundwater: the processes and global significance of aquifer degradation. Philos. Trans. R. Soc. Lond. B Biol. Sci..

[35] Allison GB (1990). Land clearance and river salinisation in the western Murray Basin, Australia. J. Hydrol..

[36] Favreau G (2009). Land clearing, climate variability, and water resources increase in semiarid southwest Niger: a review. Water Resour. Res..

[37] Wendt DE, Van Loon AF, Scanlon BR, Hannah DM (2021). Managed aquifer recharge as a drought mitigation strategy in heavily-stressed aquifers. Environ. Res. Lett..

[38] Food and Agriculture Organization of the United Nations (FAO). Trees, forests and land use in drylands: the first global assessment. FAO Forestry Paper No. 184. https://www.fao.org/dryland-assessment/en/ (FAO, 2019).

[39] Zomer RJ, Trabucco A, Bossio DA, van Straaten O, Verchot LV (2008). Climate change mitigation: a spatial analysis of global land suitability for clean development mechanism afforestation and reforestation. Agric. Ecosyst. Environ..

[40] Buchhorn, M. et al. Copernicus Global Land Service: Land Cover 100m: collection 3: epoch 2015: Globe (V3.0.1). Zenodo. 10.5281/zenodo.3939038 (2020).

[41] Berghuijs WR, Luijendijk E, Moeck C, van der Velde Y, Allen ST (2022). Global recharge data set indicates strengthened groundwater connection to surface fluxes. Geophys. Res. Lett..

[42] Opie S, Taylor RG, Brierley CM, Shamsudduha M, Cuthbert MO (2020). Climate–groundwater dynamics inferred from GRACE and the role of hydraulic memory. Earth Syst. Dyn..

[43] Konikow LF, Leake SA (2014). Depletion and capture: revisiting “the source of water derived from wells”. Groundwater.

[44] Tapley BD (2019). Contributions of GRACE to understanding climate change. Nat. Clim. Change.

[45] Rodell M, Li B (2023). Changing intensity of hydroclimatic extreme events revealed by GRACE and GRACE-FO. Nat. Water.

[46] Liu PW (2022). Groundwater depletion in California’s Central Valley accelerates during megadrought. Nat. Commun..

[47] Scanlon BR (2018). Global models underestimate large decadal declining and rising water storage trends relative to GRACE satellite data. Proc. Natl Acad. Sci..

[48] Bierkens MF, Wada Y (2019). Non-renewable groundwater use and groundwater depletion: a review. Environ. Res. Lett..

[49] Li B (2019). Global GRACE data assimilation for groundwater and drought monitoring: advances and challenges. Water Resour. Res..

[50] Xu L (2023). From coarse resolution to practical solution: GRACE as a science communication and policymaking tool for sustainable groundwater management. J. Hydrol..

[51] Jain M (2021). Groundwater depletion will reduce cropping intensity in India. Sci. Adv..

[52] Abbasnejad A, Mirzaie A, Derakhshani R, Esmaeilzadeh E (2013). Arsenic in groundwaters of the alluvial aquifer of Bardsir plain, SE Iran. Environ. Earth Sci..

[53] Abiye, T. A. Groundwater need assessment ORASECOM. Africa Groundwater Network (AGWNET) report (2012).

[54] Abotalib AZ, Heggy E, Scabbia G, Mazzoni A (2019). Groundwater dynamics in fossil fractured carbonate aquifers in Eastern Arabian Peninsula: a preliminary investigation. J. Hydrol..

[55] Adams, G. P. & Bergman, D. L. Geohydrology of alluvium and terrace deposits of the Cimarron River from freedom to Guthrie, Oklahoma. U.S. Geological Survey Water-Resources Investigations Report 95-4066. https://pubs.usgs.gov/wri/1995/4066/report.pdf (1996).

[56] Adelana S, Xu Y, Vrbka PA (2010). A conceptual model for the development and management of the Cape Flats aquifer, South Africa. Water SA.

[57] Adinehvand R, Mozaffarizadeh J, Sajadi Z, Ansari A (2019). Identifying major factors affecting groundwater quality of the Galehdar plain, south of Fars province. Res. Earth Sci..

[58] Afshin, A. A. & Motlagh, K. S. The study of sharp decline in groundwater in Kohgiluyeh and Boyer province with special attention to the Calacho plain-Dehdasht-iran. International Journal of Research Publications. https://ijrp.org/paper-detail/67To (2018).

[59] Agarwal M, Gupta SK, Deshpande RD, Yadava MG (2006). Helium, radon and radiocarbon studies on a regional aquifer system of the North Gujarat–Cambay region, India. Chem. Geol..

[60] Aghlmand R, Abbasi A (2019). Application of MODFLOW with boundary conditions analyses based on limited available observations: a case study of Birjand plain in East Iran. Water.

[61] Ahmadi A, Aberoumand M (2009). Vulnerability of Khash-Plain aquifer, eastern Iran, to pollution using geographic information system (GIS). Geotech. Geol..

[62] Ahmadvand M, Karami E (2009). A social impact assessment of the floodwater spreading project on the Gareh-Bygone plain in Iran: a causal comparative approach. Environ. Impact Assess. Rev..

[63] Akhavan S (2010). Application of SWAT model to investigate nitrate leaching in Hamadan–Bahar Watershed, Iran. Agric. Ecosyst. Environ..

[64] Alatorre LC, Díaz RE, Miramontes S, Bravo LC, Sánchez E (2014). Spatial and temporal evolution of the static water level of the Cuauhtemoc Aquifer during the years 1973, 1991 and 2000: a geographical approach. J. Geogr. Inf. Syst..

[65] Alberta Environment. Cold Lake-Beaver River Basin. Groundwater quality state of the basin report. https://open.alberta.ca/dataset/1566ed51-e765-468d-99d5-cfb9f08be4d5/resource/e1317376-a2d4-4f93-8834-b95963c3daf7/download/2006-coldlake-beavergroundwaterreport-2006.pdf (2006).

[66] Aldaya, M. M. & Llamas, M. R. Water footprint analysis for the Guadiana river basin (vol. 3). https://waterfootprint.org/media/downloads/Report35-WaterFootprint-Guadiana.pdf (2008).

[67] Ali R (2012). Potential climate change impacts on groundwater resources of south-western Australia. J. Hydrol..

[68] Alimoradi J (2018). Data on corrosive water in the sources and distribution network of drinking water in north of Iran. Data Brief.

[69] Alizadeh MR, Nikoo MR, Rakhshandehroo GR (2017). Hydro-environmental management of groundwater resources: a fuzzy-based multi-objective compromise approach. J. Hydrol..

[70] Allander, K. K., Niswonger, R. G. & Jeton, A. E. Simulation of the Lower Walker River Basin hydrologic system, west-central Nevada, using PRMS and MODFLOW models. U.S. Geological Survey Scientific Investigations Report 2014-5190. https://pubs.usgs.gov/sir/2014/5190/pdf/sir2014-5190.pdf (2014).

[71] Alvarado JAC, Pačes T, Purtschert R (2013). Dating groundwater in the Bohemian Cretaceous Basin: understanding tracer variations in the subsurface. Appl. Geochem..

[72] Amin, M., Khan, M. R. & Jamil, A. in *Advances in Remote Sensing and Geo Informatics Applications. CAJG 2018. Advances in Science, Technology & Innovation* (eds El-Askary, H., Lee, S., Heggy, E. & Pradhan, B.) 299–304 (Springer, 2018).

[73] Amiri V, Rezaei M, Sohrabi N (2014). Groundwater quality assessment using entropy weighted water quality index (EWQI) in Lenjanat, Iran. Environ. Earth Sci..

[74] Amirkhizi MT, Delirhasannia R, Haghighatjou P, Majnooni Heris A (2019). Determining water quality of agricultural wells for use in pressurized irrigation systems of Sarab plain, Iran. Water Soil Sci..

[75] Amouzegari P, Panahi M, Mirnia SK, Daneshi A (2020). Estimation of preservation value of groundwater resources from the villagers’ perspective in Alashtar Watershed, Iran. Watershed Eng. Manag..

[76] Anand, A. V. S. S. Ground Water Brochure Nellore District, Andhra Pradesh. Central Ground Water Board, Ministry of Water Resources, Government of India. http://cgwb.gov.in/old_website/District_Profile/AP_districtProfiles.html (2009).

[77] Anderholm, S. K. Hydrogeology of the Socorro and La Jencia Basins, Socorro County, New Mexico. U.S. Geological Survey Water-Resources Investigations Report 84-4342. https://pubs.usgs.gov/wri/1984/4342/report.pdf (1984).

[78] Anders R, Mendez GO, Futa K, Danskin WR (2014). A geochemical approach to determine sources and movement of saline groundwater in a coastal aquifer. Groundwater.

[79] Andreu JM, Alcalá FJ, Vallejos A, Pulido-Bosch A (2011). Recharge to mountainous carbonated aquifers in SE Spain: different approaches and new challenges. J. Arid. Environ..

[80] Anning, D. W. Conceptual understanding and groundwater quality of selected basin-fill aquifers in the Southwestern United States. Section 7.—Conceptual understanding and groundwater quality of the basin-fill aquifer in the West Salt River Valley, Arizona. U.S. Geological Survey Professional Paper 1781. https://pubs.usgs.gov/pp/1781/pdf/pp1781_section7.pdf (2014).

[81] Ansari MA, Noble J, Deodhar A, Kumar US (2022). Isotope hydrogeochemical models for assessing the hydrological processes in a part of the largest continental flood basalts province of India. Geosci. Front..

[82] Arabameri A, Rezaei K, Cerda A, Lombardo L, Rodrigo-Comino J (2019). GIS-based groundwater potential mapping in Shahroud plain, Iran. A comparison among statistical (bivariate and multivariate), data mining and MCDM approaches. Sci. Total Environ..

[83] Arabameri A, Roy J, Saha S, Blaschke T, Ghorbanzadeh O, Tien Bui D (2019). Application of probabilistic and machine learning models for groundwater potentiality mapping in Damghan sedimentary plain, Iran. Remote Sens..

[84] Araneda M, Avendaño MS, Del Río GD (2010). Modelo estructural de la cuenca de Santiago, Chile y su relación con la hidrogeología. Rev. Geofís..

[85] Arasteh SM, Shoaei SM (2020). An assessment of the effects of excessive groundwater abstraction on the quality of groundwater resources of the Zanjan Plain, Iran. Environ. Earth Sci..

[86] Arauzo M, Martínez-Bastida JJ (2015). Environmental factors affecting diffuse nitrate pollution in the major aquifers of central Spain: groundwater vulnerability vs. groundwater pollution. Environ. Earth Sci..

[87] Aref F, Roosta R (2016). Assessment of groundwater quality and hydrochemical characteristics in Farashband plain, Iran. Arab. J. Geosci..

[88] Argamasilla Ruiz, M. & Andreo-Navarro, B. Resultados preliminares de la investigación hidrogeológica del acuífero aluvial del río Guadaiza (Marbella, España). https://riuma.uma.es/xmlui/handle/10630/8767 (2015).

[89] Arizona Department of Water Resources. The Groundwater Flow Model of the Willcox Basin. Arizona Department of Water Resources report. https://www.azwater.gov/sites/default/files/2022-12/Willcox_Report_2018.pdf (2018).

[90] Armengol S, Manzano M, Ayora C, Martínez S (2023). The origin of groundwater salinity in the Matanza-Riachuelo aquifer system, Argentina. Groundw. Sustain. Dev..

[91] Arrate I (1997). Groundwater pollution in Quaternary aquifer of Vitoria–Gasteiz (Basque Country, Spain). Influence of agricultural activities and water-resource management. Environ. Geol..

[92] Arreguín F, López-Pérez M, Galván R (2018). Acuíferos transfronterizos en México: análisis normativo hacia una estrategia de manejo. Tecnol. Cienc. Agua.

[93] Arthur, J. K. & Taylor, R. E. Ground-water flow analysis of the Mississippi embayment aquifer system, South-Central United States. U.S. Geological Survey Professional Paper 1416-1. https://pubs.usgs.gov/pp/1416i/report.pdf (1998).

[94] Aryafar A, Khosravi V, Hooshfar F (2019). GIS-based comparative characterization of groundwater quality of Tabas basin using multivariate statistical techniques and computational intelligence. Int. J. Environ. Sci. Technol..

[95] Aryafar A, Khosravi V, Zarepourfard H, Rooki R (2019). Evolving genetic programming and other AI-based models for estimating groundwater quality parameters of the Khezri plain, Eastern Iran. Environ. Earth Sci..

[96] Asadi F, Soltanian M, Mohmmadi A, Setareh P, Khezri SM (2015). Geographical zoning physicochemical quality change in groundwater catchment Gharehsou ten-year period 2003-2012. Biosci. Biotechnol. Res. Asia.

[97] Asadi N, Kaki M, Jamoor R (2016). Groundwater level decline and compensating withdrawal plan in Aleshtar plain, Lorestan province, Iran. J. Nat. Environ. Hazards.

[98] Asgharinia S, Petroselli A (2020). A comparison of statistical methods for evaluating missing data of monitoring wells in the Kazeroun Plain, Fars Province, Iran. Groundw. Sustain. Dev..

[99] Ashraf A, Ahmad Z (2008). Regional groundwater flow modelling of Upper Chaj Doab of Indus Basin, Pakistan using finite element model (Feflow) and geoinformatics. Geophys. J. Int..

[100] Ashraf, A., Ahmad, Z. & Akhter, G. in *Groundwater of South Asia* (ed. Mukherjee, A.) 593–611 (Springer, 2018).

[101] Ashworth, J. B. Bone Spring-Victorio Peak aquifer of the Dell Valley region of Texas. Texas Water Development Board report. https://www.twdb.texas.gov/publications/reports/numbered_reports/doc/R356/Chapter10.pdf (2001).

[102] Aucott, W. R. Hydrology of the Southeastern Coastal Plain aquifer system in South Carolina and parts of Georgia and North Carolina. U.S. Geological Survey Professional Paper 1410-E. https://pubs.usgs.gov/pp/1410e/report.pdf (1996).

[103] Australian Government. Sydney Basin bioregion assessment. https://www.bioregionalassessments.gov.au/assessments/sydney-basin-bioregion (2018).

[104] Avand M, Ekhtesasi MR (2020). The effect of geological formations on the quality and quantity of groundwater (case study: Imamzadeh Jafar Gachsaran plain). Sustain. Earth Rev..

[105] Awadh SM, Al-Mimar H, Yaseen ZM (2020). Groundwater availability and water demand sustainability over the upper mega aquifers of Arabian Peninsula and west region of Iraq. Environ. Dev. Sustain..

[106] Azizi F, Moghaddam AA, Nazemi A, Gorgij AD (2019). Introducing a novel method in evaluation of groundwater hydrochemical characteristics, GWQI_SI_ index: case study—Malekan Aquifer, Northwest of Iran. Arab. J. Geosci..

[107] Azma A (2021). Statistical modeling for spatial groundwater potential map based on GIS technique. Sustainability.

[108] Babaee S (2020). Land subsidence from interferometric SAR and groundwater patterns in the Qazvin plain, Iran. Int. J. Remote Sens..

[109] Bachand, P. A. M., Birt, K. S. & Bachand, S. M. Groundwater relationships to pumping, precipitation and geology in high-elevation basin, Sierra Valley, CA. Report to Feather River Land. https://aquadocs.org/handle/1834/41185 (2020).

[110] Bachman, L. J., Shedlock, R. J. & Phillips, P. J. Ground-water-quality assessment of the Delmarva Peninsula, Delaware, Maryland, and Virginia. U.S. Geological Survey Open-File Report 87-112. https://pubs.usgs.gov/of/1987/0112/report.pdf (1987).

[111] Bachman, S. Goleta groundwater basin groundwater management plan. Goleta Water District. https://www.goletawater.com/doc/1194/ (2010).

[112] Back, W. Geology and ground-water features of the Smith River Plain Del Norte County California. U.S. Geological Survey Water-Supply Paper 1254. https://pubs.usgs.gov/wsp/1254/report.pdf (1957).

[113] Back W (1983). Process and rate of dedolomitization: mass transfer and ^14^C dating in a regional carbonate aquifer. Geol. Soc. Am. Bull..

[114] Baghapour MA (2016). Optimization of DRASTIC method by artificial neural network, nitrate vulnerability index, and composite DRASTIC models to assess groundwater vulnerability for unconfined aquifer of Shiraz Plain, Iran. J. Environ. Health Sci. Eng..

[115] Bagheri R, Bagheri F, Eggenkamp HGM (2017). Origin of groundwater salinity in the Fasa Plain, southern Iran, hydrogeochemical and isotopic approaches. Environ. Earth Sci..

[116] Bagheri R, Nosrati A, Jafari H, Eggenkamp HGM, Mozafari M (2019). Overexploitation hazards and salinization risks in crucial declining aquifers, chemo-isotopic approaches. J. Hazard. Mater..

[117] Bahrami M, Khaksar E, Khaksar E (2020). Spatial variation assessment of groundwater quality using multivariate statistical analysis (case study: Fasa Plain, Iran). J. Groundw. Sci. Eng..

[118] Bai L (2016). Health risk assessment research on heavy metals ingestion through groundwater drinking pathway for the residents in Baotou, China. J. Environ. Health.

[119] Bal AA (1996). Valley fills and coastal cliffs buried beneath an alluvial plain: evidence from variation of permeabilities in gravel aquifers, Canterbury Plains, New Zealand. J. Hydrol. (New Zeal.).

[120] Balachandran, A. District groundwater brochure Tirunelveli district, Tamil Nadu. Central Ground Water Board Technical Report Series. http://cgwb.gov.in/old_website/District_Profile/TN_districtprofile.html (2009).

[121] Ballukraya PN, Kalimuthu R (2010). Quantitative hydrogeological and geomorphological analyses for groundwater potential assessment in hard rock terrains. Curr. Sci..

[122] Banejad H, Mohebzadeh H, Ghobadi MH, Heydari M (2014). Numerical simulation of groundwater flow and contamination transport in Nahavand Plain aquifer, west of Iran. J. Geol. Soc. India.

[123] Barati K, Koopaei JA, Azari A, Darvishi E, Yousefi A (2019). Ground water modeling to determine hydrodynamics coefficients in unconfined aquifer (case study: Kermanshah Plain). Iran. J. Soil Water Res..

[124] Barker, R. A. & Ardis, A. F. Hydrogeological framework of the Edwards-Trinity aquifer system, west-central Texas. U.S. Geological Survey Professional Paper 1421-B. https://pubs.usgs.gov/pp/1421b/report.pdf (1996).

[125] Barkmann, P. E. et al. ON-010 Colorado Groundwater Atlas. Geohydrology. Colorado Geological Survey. https://coloradogeologicalsurvey.org/water/colorado-groundwater-atlas/ (2020).

[126] Barnett, S., Harrington, N., Cook, P. & Simmons, C. T. in *Sustainable Groundwater Management. Global Issues in Water Policy*, Vol. 24 (eds Rinaudo, J.-D., Hollet, C., Barnett, S. & Montginoul, M.) 109–127 (Springer, 2020).

[127] Barnett, S. et al. A hydrostratigraphic model for the shallow aquifer systems of the Gambier Basin and South Western Murray Basin. Goyder Institute for Water Research Technical Report Series No. 15/15. https://goyderinstitute.org/report/a-hydrostratigraphic-model-for-the-shallow-aquifer-systems-of-the-gambier-basin-and-south-western-murray-basin/ (2015).

[128] Barnett, S., Simmons, C. T. & Nelson, R. in *Global Groundwater: Source, Scarcity, Sustainability, Security, and Solutions* (eds Mukherjee, A., Scanlon, B. R., Aureli, A., Langan, S., Guo, H. & McKenzie, A.) 35–46 (Elsevier, 2021).

[129] Barron O (2012). Climate change effects on water-dependent ecosystems in south-western Australia. J. Hydrol..

[130] Bartolino, J. R. & Cole, J. C. Ground-water resources of the Middle Rio Grande Basin. U.S. Geological Survey Water-Resources Circular 1222. https://pubs.usgs.gov/circ/2002/circ1222/pdf/circ1222.pdf (2002).

[131] Barzegar R, Moghaddam AA, Tziritis E (2017). Hydrogeochemical features of groundwater resources in Tabriz plain, northwest of Iran. Appl. Water Sci..

[132] Basharat, M. Groundwater Environment and Evaluation of Long-Term Sustainability of the Aquifer under Lahore, Punjab, Pakistan. International Waterlogging and Salinity Research Institute, Pakistan Water and Power Development Authority report. Project title: “Enhancing the groundwater management capacity in Asian cities through the development and application of groundwater sustainability index (GSII) in the context of global change” (2014).

[133] Baudron P (2014). Impacts of human activities on recharge in a multilayered semiarid aquifer (Campo de Cartagena, SE Spain). Hydrol. Process..

[134] Bauer-Gottwein P (2011). The Yucatán Peninsula karst aquifer, Mexico. Hydrol. J..

[135] Bayat-Varkeshi M, Farahani M, Ghabaei Sough M (2018). Effect of meteorological drought on groundwater resource (case study: Komijan Aquifer in Markazi Province). Iran Water Resour. Res..

[136] Bazrafshan O, Parandin F, Farokhzadeh B (2016). Assessment of hydro-meteorological drought effects on groundwater resources in Hormozgan region-South of Iran. Ecopersia.

[137] Beach, J. A. et al. Groundwater availability model for the Igneous and parts of the West Texas Bolsons (Wild Horse Flat, Michigan Flat, Ryan Flat and Lobo Flat) aquifers. Texas Water Development Board report. https://www.twdb.texas.gov/groundwater/models/gam/igbl/IGBL_Model_Report.pdf (2004).

[138] Beach, J. A., Burton, S. & Kolarik, B. Groundwater availability model for the Lipan Aquifer in Texas. Texas Water Development Board report. https://www.twdb.texas.gov/groundwater/models/gam/lipn/LIPN_Model_Report.pdf (2004).

[139] Beaudoin N, Gasparrini M, David ME, Lacombe O, Koehn D (2019). Bedding-parallel stylolites as a tool to unravel maximum burial depth in sedimentary basins: application to Middle Jurassic carbonate reservoirs in the Paris basin, France. GSA Bull..

[140] Beccaletto L, Hanot F, Serrano O, Marc S (2011). Overview of the subsurface structural pattern of the Paris Basin (France): insights from the reprocessing and interpretation of regional seismic lines. Mar. Pet. Geol..

[141] Becker, C. J., Runkle, D. & Rea, A. Digital data sets that describe aquifer characteristics of the Enid isolated terrace aquifer in northwestern Oklahoma. U.S. Geological Survey Open-File Report 96-450. https://pubs.usgs.gov/of/1996/ofr96-450/ (1997).

[142] Becker, C. J., Runkle, D. & Rea, A. Digital data sets that describe aquifer characteristics of the Elk City aquifer in western Oklahoma. U.S. Geological Survey Open-File Report 96-449. https://pubs.usgs.gov/of/1996/ofr96-449/ (1997).

[143] Becker, M. F. & Runkle, D. L. Hydrogeology, water quality, and geochemistry of the Rush Springs aquifer, western Oklahoma. U.S. Geological Survey Water-Resources Investigations Report 98-4081. https://pubs.usgs.gov/wri/1998/4081/report.pdf (1998).

[144] Bejarano MD (2012). Responses of riparian guilds to flow alterations in a Mediterranean stream. J. Veg. Sci..

[145] Bekesi G, McGuire M, Moiler D (2009). Groundwater allocation using a groundwater level response management method—Gnangara groundwater system, Western Australia. Water Resour. Manag..

[146] Bengtson S, Sallstedt T, Belivanova V, Whitehouse M (2017). Three-dimensional preservation of cellular and subcellular structures suggests 1.6 billion-year-old crown-group red algae. PLoS Biol..

[147] Berens, V., Alcoe, D. & Watt, E. Non-prescribed groundwater resources assessment — Eyre Peninsula natural resources management region. Technical Report DFW 2011/16. Science, Monitoring and Information Division, Department for Water. https://www.waterconnect.sa.gov.au/Content/Publications/DEW/EP_NRM_Non-prescribed_GW_Assessment_2011.pdf (2011).

[148] Berger, D. L. Hydrogeology and water resources of Ruby Valley, northeastern Nevada. U.S. Geological Survey Scientific Investigations Report 2005-5247. https://pubs.usgs.gov/sir/2005/5247/sir2005-5247.pdf (2006).

[149] Berger, D. L., Ross, W. C., Thodal, C. E. & Robledo, A. R. Hydrogeology and simulated effects of urban development on water resources of Spanish Springs Valley, Washoe County, West-Central Nevada. U.S. Geological Survey Water-Resources Investigations Report 96-4297. https://pubs.usgs.gov/wri/1996/4297/report.pdf (1997).

[150] Bernhard C (1992). Nitrate pollution of groundwater in the Alsatian Plain (France)—a multidisciplinary study of an agricultural area: the Central Ried of the Ill river. Environ. Geol. Water Sci..

[151] Bestland E (2017). Groundwater dependent pools in seasonal and permanent streams in the Clare Valley of South Australia. J. Hydrol. Reg. Stud..

[152] Betcher, R. N. Groundwater Availability Map Series - Dauphin Lake Area (62-O). Manitoba Department of Natural Resources map. https://www.gov.mb.ca/water/pubs/maps/water/1987_betcher_groundwater_availability_map_series_dauphin_lake.zip (1986).

[153] Betcher, R. N. Groundwater Availability Map Series - Neepawa Area (62-J). Manitoba Department of Natural Resources map. https://www.gov.mb.ca/water/pubs/maps/water/1988_betcher_groundwater_availability_map_series_neepawa.zip (1988).

[154] Betcher, R. N. Groundwater Availability Map Series - Selkirk Area (62-I). Manitoba Department of Natural Resources map. https://www.gov.mb.ca/water/pubs/maps/water/1986_betcher_groundwater_availability_map_series_selkirk.zip (1985).

[155] Betcher, R. N. Groundwater Availability Map Series - Virden Area (62-F). Manitoba Department of Natural Resources map. https://www.gov.mb.ca/water/pubs/maps/water/1983_betcher_groundwater_availability_map_series_virden.zip (1983).

[156] Betcher, R. N., Pupp, C. & Grove, G. Groundwater in Manitoba: hydrogeology, quality concerns, management. Environment Canada, National Hydrology Research Institute Report No. C2-93017. https://web.viu.ca/earle/geol304/hg-manitoba.pdf (1995).

[157] Beverly, C. et al. The Gippsland groundwater model. Technical report. Victoria State Government. https://www.parliament.vic.gov.au/images/stories/committees/EPC/Other_documents/G3_-_Gippsland_groundwater_model_report_June_2015_2.pdf (2015).

[158] Bexfield, L. M. & Anderholm, S. K. Predevelopment water-level map of the Santa Fe Group aquifer system in the middle Rio Grande basin between Cochiti Lake and San Acacia, New Mexico. U.S. Geological Survey Water-Resources Investigations Report 2000-4249. 10.3133/wri004249 (2000).

[159] Bhimani, S. A. *Study on Groundwater Salinization and Formulation of Management Strategies for the Coastal Aquifers of Mundra Region, Kutch District, Gujarat State*. PhD thesis, Maharaja Sayajirao University of Baroda (2013).

[160] Bhuiyan C, Singh RP, Flügel WA (2009). Modelling of ground water recharge-potential in the hard-rock Aravalli terrain, India: a GIS approach. Environ. Earth Sci..

[161] Bhunia GS, Keshavarzi A, Shit PK, Omran ESE, Bagherzadeh A (2018). Evaluation of groundwater quality and its suitability for drinking and irrigation using GIS and geostatistics techniques in semiarid region of Neyshabur, Iran. Appl. Water Sci..

[162] Bianco, E. Seismic interpretation of the Windsor-Kennetcook basin. Geological Survey of Canada Open File 7452. https://ftp.maps.canada.ca/pub/nrcan_rncan/publications/STPublications_PublicationsST/292/292763/of_7452.pdf (Geological Survey of Canada, 2013).

[163] Biteau JJ, Le Marrec A, Le Vot M, Masset JM (2006). The aquitaine basin. Pet. Geosci..

[164] Bjorklund, L. J. & McGreevy, L. J. Ground-water resources of Cache Valley, Utah and Idaho. Utah Department of Natural Resources, Division of Water Rights Technical Publication No. 36. https://waterrights.utah.gov/docSys/v920/w920/w920008y.pdf (1971).

[165] Bjorklund, L. J. Reconnaissance of ground water conditions in the Crow Flats area, Otero County, New Mexico. New Mexico State Engineer Office Technical Report No. 8. http://www.oteroswcd.org/PDF/NM%20OSE%20Reconnaissance%20of%20Ground%20Water%20Conditions%20in%20the%20Crow%20Flats%20Area%201957.pdf (1957).

[166] Blake S (2016). Compositional multivariate statistical analysis of thermal groundwater provenance: a hydrogeochemical case study from Ireland. Appl. Geochem..

[167] Bocanegra E, Da Silva GC, Custodio E, Manzano M, Montenegro S (2010). State of knowledge of coastal aquifer management in South America. Hydrol. J..

[168] Bonsor HC (2017). Hydrogeological typologies of the Indo-Gangetic basin alluvial aquifer, South Asia. Hydrol. J..

[169] Boonkaewwan S, Sonthiphand P, Chotpantarat S (2021). Mechanisms of arsenic contamination associated with hydrochemical characteristics in coastal alluvial aquifers using multivariate statistical technique and hydrogeochemical modeling: a case study in Rayong province, eastern Thailand. Environ. Geochem. Health.

[170] Bordbar M, Neshat A, Javadi S (2019). A new hybrid framework for optimization and modification of groundwater vulnerability in coastal aquifer. Environ. Sci. Pollut. Res..

[171] Borneuf, D. M. Hydrogeological map of the Oyen area, Alberta, NTS 72M. Alberta Energy Regulator map. https://static.ags.aer.ca/files/document/MAP/Map_120.pdf (2005).

[172] Boroghani M, Taie M, Mirnia SK (2013). Analysis of relationship between hydrogeological and climatological droughts using SWI and SPI indices in Sabzevar Plain. Iran. J. Rangeland Desert Res..

[173] Boswell, E. H. The Citronelle aquifers in Mississippi. U.S. Geological Survey Water-Resources Investigations Report 78-131. https://pubs.usgs.gov/wri/1978/0131/plate-1.pdf (1979).

[174] Bouchaou L (2008). Application of multiple isotopic and geochemical tracers for investigation of recharge, salinization, and residence time of water in the Souss–Massa aquifer, southwest of Morocco. J. Hydrol..

[175] Bradley, E. Summary of the ground-water resources of the Laramie River drainage basin, Wyoming, and the North Platte River drainage basin from Douglas, Wyoming, to the Wyoming-Nebraska state line. U.S. Geological Survey Open-File Report 55-17. https://pubs.usgs.gov/of/1955/0017/report.pdf (1955).

[176] Brahana, J. V. & Bradley, M. W. Preliminary delineation and description of the regional aquifers of Tennessee--the Highland Rim Aquifer System. U.S. Geological Survey Water-Resources Investigations Report 82-4054. https://pubs.usgs.gov/wri/wri824054/pdf/wrir_82-4054_a.pdf (1986).

[177] Brahana, J. V., Macy, J. A., Mulderink, D. & Zemo, D. Preliminary delineation and description of the regional aquifers of Tennessee--Cumberland plateau aquifer system. U.S. Geological Survey Water-Resources Investigations Open-File Report 82-338. https://pubs.usgs.gov/wri/wrir82-338/pdf/wrir_82-338_a.pdf (1986).

[178] Braun, C. L., Ramage, J. K. & Shah, S. D. Status of groundwater-level altitudes and long-term groundwater-level changes in the Chicot, Evangeline, and Jasper aquifers, Houston-Galveston region, Texas, 2019. U.S. Geological Survey Scientific Investigations Report 2019-5089. https://pubs.usgs.gov/sir/2019/5089/sir20195089.pdf (2019).

[179] Bredehoeft, J. D., Neuzil, C. E. & Milly, P. C. D. Regional flow in the Dakota aquifer: a study of the role of confining layers. U.S. Geological Survey Water-Supply Paper 2237. https://pubs.er.usgs.gov/publication/wsp2237 (1983).

[180] Bredehoeft, J. D. & Farvolden, R. N. Disposition of aquifers in intermontane basins of northern Nevada. International Association of Scientific Hydrology, Commission of Subterranean Waters, Publication no. 64, 197–212. https://iahs.info/uploads/dms/064017.pdf (1963).

[181] Bresciani E (2018). Using hydraulic head, chloride and electrical conductivity data to distinguish between mountain-front and mountain-block recharge to basin aquifers. Hydrol. Earth Syst. Sci..

[182] BRGM. L’aquifère des calcaires carbonifères. Presentation for an Interreg IVB NWE project for a better quality of surface and groundwater bodies in the Scheldt International River Basin District (IRBD). https://www.isc-cie.org/wp-content/uploads/PLEN_1701_pres-Parmentier_BRGM_Carbonifere.pdf (2013).

[183] Briar, D. W. & Dutton, D. M. Hydrogeology and aquifer sensitivity of the Bitterroot Valley, Ravalli County, Montana. U.S. Geological Survey Water-Resources Investigations Report 99-4219. https://pubs.usgs.gov/wri/1999/4219/report.pdf (1999).

[184] Briar, D. W. & Madison, J. P. Hydrogeology of the Helena Valley-fill aquifer system, west-central Montana. U.S. Geological Survey Water-Resources Investigations Report 92-4023. https://pubs.usgs.gov/wri/1992/4023/report.pdf (1992).

[185] Briceño Aguirre, A. D. *Funcionamiento Hidrogeológico y Geometría del Acuífero del Sector Norte y Centro de Santiago*. Thesis, Universidad de Chile (2020).

[186] Bright, D. J., Stamos, C. L., Martin, P. M. & Nash, D. B. Ground-water hydrology and quality in the Lompoc area, Santa Barbara County, California, 1987-88. U.S. Geological Survey Water-Resources Investigations Report 91-4172. https://pubs.usgs.gov/wri/1991/4172/report.pdf (1992).

[187] Brito-Castillo L, Méndez Rodríguez LC, Chávez López S, Acosta Vargas B (2010). Groundwater differentiation of the aquifer in the Vizcaino Biosphere Reserve, Baja California Peninsula, Mexico. Geofís. Int..

[188] Brockman, C. S. Physiographic regions of Ohio. State of Ohio, Department of Natural Resources, Division of Geological Survey map. https://www.epa.gov/sites/default/files/2016-04/documents/05_oh_rec4.pdf (1998).

[189] Brooks, H. K. Physiographic divisions of Florida. Report for the Florida Cooperative Extension Service, Institute of Food and Agricultural Sciences, University of Florida (1981).

[190] Brooks, L. E. & Mason, J. L. Hydrology and simulation of ground-water flow in Cedar Valley, Iron County, Utah. U.S. Geological Survey Scientific Investigations Report 2005-5170. https://pubs.usgs.gov/sir/2005/5170/PDF/SIR2005_5170.pdf (2005).

[191] Brooks, L. E. Evaluation of the groundwater flow model for southern Utah and Goshen Valleys, Utah, updated to conditions through 2011, with new projections and groundwater management simulations. U.S. Geological Survey Open-File Report 2013-1171. https://pubs.usgs.gov/of/2013/1171/pdf/ofr2013-1171.pdf (2013).

[192] Brown, C. R. & Macy, J. P. Groundwater, surface-water, and water-chemistry data from the C-aquifer Monitoring Program, northeastern Arizona, 2005–2011. U.S. Geological Survey Open-File Report 2012-1196. https://pubs.usgs.gov/of/2012/1196/of2012-1196.pdf (2012).

[193] Brown DM, Lloyd JW, Jacobson G (1990). Hydrogeological model for Amadeus Basin aquifers, central Australia. Aust. J. Earth Sci..

[194] Bruun, B., Jackson, K., Lake, P. & Walker, J. Texas aquifers study. Groundwater quantity, quality, flow, and contributions to surface water. Texas Water Development Board report. https://www.twdb.texas.gov/groundwater/docs/studies/TexasAquifersStudy_2016.pdf#page=89 (2016).

[195] Bugan RD (2016). Four decades of water recycling in Atlantis (Western Cape, South Africa): past, present and future. Water SA.

[196] Bujes Moreno, N. J. I. *Estudio de la propiedad del agua subterránea del acuίfero del Rίo Petorca en la Región de Valparaίso, Chile*. Thesis, Universidad de Chile (2015).

[197] Buono, A. The Southern Hills regional aquifer system of southeastern Louisiana and southwestern Mississippi. U.S. Geological Survey Water-Resources Investigations Report 83-4189. https://pubs.usgs.gov/wri/1983/4189/report.pdf (1983).

[198] Burbey, T. J. Hydrogeology and potential for ground-water development, carbonate-rock aquifers in southern Nevada and southeastern California. U.S. Geological Survey Water-Resources Investigations Report 95-4168. https://pubs.usgs.gov/wri/1995/4168/report.pdf (1997).

[199] Burgess WG (2010). Vulnerability of deep groundwater in the Bengal Aquifer System to contamination by arsenic. Nat. Geosci..

[200] Burns, E. R., Morgan, D. S., Peavler, R. S. & Kahle, S. C. Three-dimensional model of the geologic framework for the Columbia Plateau regional aquifer system, Idaho, Oregon, and Washington. U.S. Geological Survey Scientific Investigations Report 2010-5246. https://pubs.usgs.gov/sir/2010/5246/pdf/sir20105246.pdf (2011).

[201] Burns, E. R., Snyder, D. T., Haynes, J. V. & Waibel, M. S. Groundwater status and trends for the Columbia Plateau Regional Aquifer System, Washington, Oregon, and Idaho. U.S. Geological Survey Scientific Investigations Report 2012-5261. https://pubs.usgs.gov/sir/2012/5261/pdf/sir2012-5261.pdf (2012).

[202] Cai Y, Esaki T, Liu S, Mitani Y (2014). Effect of substitute water projects on tempo-spatial distribution of groundwater withdrawals in Chikugo-Saga plain, Japan. Water Resour. Manag..

[203] Calatrava J, Guillem A, Martínez-Granados D (2011). Analysis of alternatives to eliminate aquifer overdraft in the Guadalentín Valley, SE Spain. Econ. Agrar. Recur. Nat..

[204] Calf GE, McDonald PS, Jacobson G (1991). Recharge mechanism and groundwater age in the Ti‐Tree Basin, Northern Territory. Aust. J. Earth Sci..

[205] California Department of Water Resources. Basin Boundaries Description - Imperial Valley. Bulletin 118. https://water.ca.gov/-/media/DWR-Website/Web-Pages/Programs/Groundwater-Management/Bulletin-118/Files/2003-Basin-Descriptions/7_030_ImperialValley.pdf (2003).

[206] California Department of Water Resources. Borrego Valley - Ocotillo Wells Basin Boundaries. https://water.ca.gov/-/media/DWR-Website/Web-Pages/Programs/Groundwater-Management/Bulletin-118/Files/2016-Basin-Boundary-Descriptions/7_024_02_OcotilloWells.pdf (2016).

[207] California Department of Water Resources. California’s groundwater update 2013 - Chapter 10: North Lahontan Hydrologic Region. https://water.ca.gov/-/media/DWR-Website/Web-Pages/Programs/Groundwater-Management/Bulletin-118/Files/Statewide-Reports/GWU2013_Ch10_NorthLahontan_Final.pdf (2015).

[208] California Department of Water Resources. California’s groundwater update 2013 - Chapter 11: South Lahontan Hydrologic Region. https://water.ca.gov/-/media/DWR-Website/Web-Pages/Programs/Groundwater-Management/Data-and-Tools/Files/Statewide-Reports/California-Groundwater-Update-2013/California-Groundwater-Update-2013---Chapter-11---South-Lahontan.pdf (2015).

[209] California Department of Water Resources. California’s groundwater update 2013 - Chapter 12: Colorado River Hydrologic Region. https://water.ca.gov/-/media/DWR-Website/Web-Pages/Programs/Groundwater-Management/Bulletin-118/Files/Statewide-Reports/GWU2013_Ch12_ColoradoRiver_Final.pdf (2015).

[210] California Department of Water Resources. California’s groundwater update 2013 - Chapter 3: North Coast Hydrologic Region. https://water.ca.gov/-/media/DWR-Website/Web-Pages/Programs/Groundwater-Management/Bulletin-118/Files/Statewide-Reports/GWU2013_Ch3_NorthCoast_Final.pdf (2015).

[211] California Department of Water Resources. California’s groundwater update 2013 - Chapter 4: San Francisco Bay Hydrologic Region. https://cawaterlibrary.net/wp-content/uploads/2017/05/GWU2013_Ch4_SanFranciscoBay_Final.pdf (2015).

[212] California Department of Water Resources. California’s groundwater update 2013 - Chapter 5: Central Coast Hydrologic Region. https://cawaterlibrary.net/wp-content/uploads/2017/05/GWU2013_Ch5_CentralCoast_Final.pdf (2015).

[213] California Department of Water Resources. California’s groundwater update 2013 - Chapter 6: South Coast Hydrologic Region. https://cawaterlibrary.net/wp-content/uploads/2017/05/GWU2013_Ch6_SouthCoast_Final.pdf (2015).

[214] California Department of Water Resources. California’s groundwater update 2013 - Chapter 7: Sacramento River Hydrologic Region. https://water.ca.gov/-/media/DWR-Website/Web-Pages/Programs/Groundwater-Management/Bulletin-118/Files/Statewide-Reports/GWU2013_Ch7_SacramentoRiver_Final.pdf (2015).

[215] California Department of Water Resources. California’s groundwater update 2013 - Chapter 8: San Joaquin River Hydrologic Region. https://water.ca.gov/-/media/DWR-Website/Web-Pages/Programs/Groundwater-Management/Data-and-Tools/Files/Statewide-Reports/California-Groundwater-Update-2013/California-Groundwater-Update-2013---Chapter-8---San-Joaquin-River.pdf (2015).

[216] California Department of Water Resources. California’s groundwater update 2013 - Chapter 9: Tulare Lake Hydrologic Region. https://data.cnra.ca.gov/dataset/california-water-plan-groundwater-update-2013/resource/8a4ae915-b786-42e1-9abe-99a8fcc23349 (2015).

[217] Callegary, J. B. et al. San Pedro River Aquifer Binational Report: International Boundary and Water Commission. https://pubs.usgs.gov/publication/70191935 (2016).

[218] Camacho EAS (2010). Estimación del volumen promedio recibido por el humedal de la subcuenca del Río Blanco (bajo Papaloapan; Veracruz), a través del cálculo de un balance de aguas. Aqua-LAC.

[219] Campbell, E. E., Parker-Nance, T. & Bate, G. C. A compilation of information on the magnitude, nature and importance of coastal aquifers in Southern Africa. Water Research Commission Report No. 370/1/92. http://www.wrc.org.za/wp-content/uploads/mdocs/370-1-92.pdf (1992).

[220] Campos C (2011). Soil water retention and carbon pools in tropical forested wetlands and marshes of the Gulf of Mexico. Hydrol. Sci. J..

[221] Campos MN (2013). Sectorization of environmental risk and human consumption of manganese in groundwater extracted from the Sinaloa River Aquifer. WIT Trans. Ecol. Environ..

[222] Campos, M. N., Muñoz-Sevilla, P. & Le Bail, M. in *Advances in Environmental Monitoring and Assessment* (ed. Sarvajayakesavalu, S.) Ch. 1, 3–19 (IntechOpen, 2019).

[223] Camuñas Palencia C, Mejías Moreno M, Hornero Díaz JE, Ruíz Bermudo F, García Menéndez O (2022). Deep aquifers as strategic groundwater reservoir in Spain. Bol. Geol. Min..

[224] Cañez Araiza, D. A. *Caracterización hidrogeoquímica y situación actual de la intrusión marina en la porción costera del acuífero Caborca, Sonora, México*. MSc thesis, Universidad de Sonora (2018).

[225] Cantwell, C. A. & Fawler, A. P. G. in Proc. *Thirty-Ninth Workshop on Geothermal Reservoir Engineering*. SGP-TR-202. https://pangea.stanford.edu/ERE/pdf/IGAstandard/SGW/2014/Cantwell.pdf (2014).

[226] Cao S (2021). Determining the origin and fate of nitrate in the Nanyang Basin, Central China, using environmental isotopes and the Bayesian mixing model. Environ. Sci. Pollut. Res..

[227] Carceller-Layel T, Costa-Alandí C, Coloma-López P, García-Vera MÁ, San Román-Saldaña J (2007). Groundwater in the central sector of the Ebro Basin. Water Resour. Dev..

[228] Cardona A, Carrillo-Rivera JJ, Huizar-Alvarez R, Graniel-Castro E (2004). Salinization in coastal aquifers of arid zones: an example from Santo Domingo, Baja California Sur, Mexico. Environ. Geol..

[229] Cardoso PR (1993). Saline water intrusion in Mexico. WIT Trans. Ecol. Environ..

[230] Cardwell, G. T. Geology and ground water in the Santa Rosa and Petaluma Valley areas, Sonoma County, California. U.S. Geological Survey Water-Supply Paper 1427. https://pubs.usgs.gov/wsp/1427/report.pdf (1958).

[231] Carroll RW (2010). Mason Valley groundwater model: linking surface water and groundwater in the Walker River Basin, Nevada. J. Am. Water Resour. Assoc..

[232] Carroll, R. W. H., Pohll, G. & Rajagopal, S. South Lake Tahoe groundwater model. Desert Research Institute report. https://www.stpud.us/Phase%20I%20Report_revised_Feb_25_2016.pdf (2016).

[233] Carruth, R. L., Kahler, L. M. & Conway, B. D. Groundwater-storage change and land-surface elevation change in Tucson Basin and Avra Valley, south-central Arizona—2003–2016. U.S. Geological Survey Scientific Investigations Report 2018-5154. https://pubs.usgs.gov/sir/2018/5154/sir20185154.pdf (2018).

[234] Cartwright I (2012). Constraining groundwater flow, residence times, inter-aquifer mixing, and aquifer properties using environmental isotopes in the southeast Murray Basin, Australia. Appl. Geochem..

[235] Casado, M. The Tagus basin: groundwater and transboundary Aquifers. Presentation at the Workshop on Transboundary Water Resources Management in Western and Central Europe. https://www.researchgate.net/publication/341251820_The_Tagus_basin_Groundwater_and_Transboundary_Aquifers (2010).

[236] Central Ground Water Board. Aquifer mapping and ground water management Chennai Aquifer System. Central Ground Water Board report. http://cgwb.gov.in/cgwbpnm/public/uploads/documents/1699436014992103716file.pdf (2017).

[237] Central Ground Water Board. Aquifer systems of Chhattisgarh. Central Ground Water Board report. http://cgwb.gov.in/old_website/AQM/Chhattisgarh.pdf (2012).

[238] Central Ground Water Board. Aquifer systems of India. Central Ground Water Board report. http://cgwb.gov.in/cgwbpnm/public/uploads/documents/1687419512680023437file.pdf (2012).

[239] Central Ground Water Board. Aquifer systems of Karnataka. Central Ground Water Board report. http://cgwb.gov.in/old_website/AQM/Karnataka.pdf (2012).

[240] Central Ground Water Board. Aquifer systems of Kerala. Central Ground Water Board report. http://cgwb.gov.in/old_website/AQM/Kerala.pdf (2012).

[241] Central Ground Water Board. Aquifer systems of Madhya Pradesh. Central Ground Water Board report. http://cgwb.gov.in/old_website/AQM/Madhya%20Pradesh.pdf (2013).

[242] Central Ground Water Board. Aquifer systems of Tamilnadu and Puducherry. Central Ground Water Board report. http://cgwb.gov.in/cgwbpnm/publication-detail/670 (2012).

[243] Central Ground Water Board. Ground water information booklet Dharwad District, Karnataka. Central Ground Water Board report. http://cgwb.gov.in/old_website/District_Profile/Karnataka_districtprofile.html (2008).

[244] Central Ground Water Board. Ground water information booklet Haveri District, Karnataka. Central Ground Water Board report. http://cgwb.gov.in/old_website/District_Profile/Karnataka_districtprofile.html (2008).

[245] Central Ground Water Board. Ground water information booklet, Bhadrak District, Orissa. Central Ground Water Board report. http://cgwb.gov.in/old_website/District_Profile/Orissa/BHADRAK%20.pdf (2013).

[246] Central Ground Water Board. Ground water information Jaipur District, Rajasthan. Central Ground Water Board report. http://cgwb.gov.in/old_website/District_Profile/Rajasthan/Jaipur.pdf (2013).

[247] Central Ground Water Board. Pilot Project Report on Aquifer mapping in Lower Vellar watershed, Cuddalore District, Tamilnadu. Central Ground Water Board report. http://cgwb.gov.in/cgwbpnm/publication-detail/311 (2015).

[248] Cerón JC, Pulido-Bosch A (1996). Groundwater problems resulting from CO_2_ pollution and overexploitation in Alto Guadalentín aquifer (Murcia, Spain). Environ. Geol..

[249] Chalapathi Rao NV, Gibson SA, Pyle DM, Dickin AP (2004). Petrogenesis of Proterozoic lamproites and kimberlites from the Cuddapah Basin and Dharwar craton, southern India. J. Petrol..

[250] Chamanehpour E, Sayadi MH, Yousefi E (2020). The potential evaluation of groundwater pollution based on the intrinsic and the specific vulnerability index. Groundw. Sustain. Dev..

[251] Chang J, Wang G (2010). Major ions chemistry of groundwater in the arid region of Zhangye Basin, northwestern China. Environ. Earth Sci..

[252] Chapman JB, Thomas JM, Garner C (2022). Groundwater recharge timing based on ^14^C and ^2^H within Indian Wells Valley, California, USA. Appl. Geochem..

[253] Chastain-Howley, A., Dean, K. E. & Spear, A. A. Groundwater Availability Model for the Seymour Aquifer. Texas Water Development Board report. https://www.twdb.texas.gov/groundwater/models/gam/symr/symr.asp (2004).

[254] Chatterjee S, Biswal BP, Sinha UK, Patbhaje SD (2021). Isotope-geochemical assessment of thermal waters and their impact on surrounding potable water resources in the Tapi valley geothermal area, Maharashtra, India. Environ. Earth Sci..

[255] Chen CT, Hu JC, Lu CY, Lee JC, Chan YC (2007). Thirty-year land elevation change from subsidence to uplift following the termination of groundwater pumping and its geological implications in the Metropolitan Taipei Basin, Northern Taiwan. Eng. Geol..

[256] Chen WF, Liu TK (2003). Dissolved oxygen and nitrate of groundwater in Choshui Fan-Delta, western Taiwan. Environ. Geol..

[257] Chen Z, Wei W, Liu J, Wang Y, Chen J (2011). Identifying the recharge sources and age of groundwater in the Songnen Plain (Northeast China) using environmental isotopes. Hydrol. J..

[258] Cheraghi SAM, Nagafi B, Shajari S, Javan M (2020). The trend of changes in groundwater quantity and quality in the Sarvestan Plain of Fars Province. Watershed Manag. Res. J..

[259] Cherry, A. J. *A Multi-tracer Estimation of Groundwater Recharge in a Glaciofluvial Aquifer in Southeastern Manitoba*. MSc thesis, Univ. Ottawa (2000).

[260] Chica-Olmo M, Luque-Espinar JA, Rodriguez-Galiano V, Pardo-Igúzquiza E, Chica-Rivas L (2014). Categorical Indicator Kriging for assessing the risk of groundwater nitrate pollution: the case of Vega de Granada aquifer (SE Spain). Sci. Total Environ..

[261] Choubin B, Malekian A (2013). Relationship between fluctuations in the water table and aquifer salinization (case study: Aquifer Aspas-Fars Province). Desert Manag..

[262] Chowdari S (2017). Structural mapping based on potential field and remote sensing data, South Rewa Gondwana Basin, India. J. Earth Syst. Sci..

[263] Christenson, S. et al. Hydrogeology and simulation of groundwater flow in the Arbuckle-Simpson aquifer, south-central Oklahoma. U.S. Geological Survey Scientific Investigations Report 2011-5029. https://pubs.usgs.gov/sir/2011/5029/SIR2011-5029.pdf (2011).

[264] Chucuya S (2022). Hydrogeochemical characterization and identification of factors influencing groundwater quality in coastal aquifers, case: La Yarada, Tacna, Peru. Int. J. Environ. Res. Public Health.

[265] Cigna F, Tapete D (2021). Satellite InSAR survey of structurally-controlled land subsidence due to groundwater exploitation in the Aguascalientes Valley, Mexico. Remote Sens. Environ..

[266] City of Chilliwack. Groundwater Protection. https://www.chilliwack.com/main/page.cfm?id=205 (2021).

[267] Clark, B. R., Duncan, L. L. & Knierim, K. J. Groundwater availability in the Ozark Plateaus aquifer system. U.S. Geological Survey Professional Paper 1854. https://pubs.er.usgs.gov/publication/pp1854 (2019).

[268] Clark, W. Z. & Zisa, A. C. Physiographic map of Georgia. Georgia Department of Natural Resources. https://epd.georgia.gov/document/publication/sm-4-physiographic-map-georgia-12000000-1988/download (1976).

[269] Clauzon, G. et al. Genèse et évolution du piémont néogène subalpin du bas Dauphiné. Université d’Aix-Marseille II. https://hal-insu.archives-ouvertes.fr/file/index/docid/459143/filename/Clauzon1990.pdf (1990).

[270] Coes, A., Gellenbeck, D. J., Towne, D. C. & Freark, M. C. Ground water quality in the Upper Santa Cruz Basin. U.S. Geological Survey Water-Resources Investigations Report 00-4117. https://pubs.usgs.gov/wri/2000/4117/report.pdf (2002).

[271] Commission locale de l’eau Basse Vallee de l’Ain. Plan d’Aménagement et de Gestion Durable de la ressource en eau et des milieux aquatiques [PAGD]. https://www.gesteau.fr/sites/default/files/2-sage_pagd-adopte.pdf (2013).

[272] CONAGUA. Actualización de la disponibilidad media anual de agua en al acuifero Rio Fuerte (2501), estado de Sinaloa. Comisión Nacional del Agua report. https://www.gob.mx/cms/uploads/attachment/file/103330/DR_2501.pdf (2015).

[273] CONAGUA. Actualización de la disponibilidad media anual de agua en el acuífero Abrego (3215), estado de Zacatecas. https://sigagis.conagua.gob.mx/gas1/Edos_Acuiferos_18/zacatecas/DR_3215.pdf (2020).

[274] CONAGUA. Actualización de la disponibilidad media anual de agua en el acuífero Bajo Rio Bravo (2801), estado de Tamaulipas. https://sigagis.conagua.gob.mx/gas1/Edos_Acuiferos_18/tamaulipas/DR_2801.pdf (2020).

[275] CONAGUA. Actualización de la disponibilidad media anual de agua en el acuífero Cedros (3218), estado de Zacatecas. https://sigagis.conagua.gob.mx/gas1/Edos_Acuiferos_18/zacatecas/DR_3218.pdf (2020).

[276] CONAGUA. Actualización de la disponibilidad media anual de agua en el acuífero El Salvador (3219), estado de Zacatecas. https://sigagis.conagua.gob.mx/gas1/Edos_Acuiferos_18/zacatecas/DR_3219.pdf (2020).

[277] CONAGUA. Actualización de la disponibilidad media anual de agua en el acuífero Flores Magon-Villa Ahumada (0821), estado de Chihuahua. https://www.gob.mx/cms/uploads/attachment/file/103582/DR_0821.pdf (2015).

[278] CONAGUA. Actualización de la disponibilidad media anual de agua en el acuífero Guadalupe Garzarón (3212), estado de Zacatecas. https://sigagis.conagua.gob.mx/gas1/Edos_Acuiferos_18/zacatecas/DR_3220.pdf (2020).

[279] CONAGUA. Actualización de la disponibilidad media anual de agua en el acuífero Hidalgo (3202), estado de Zacatecas. https://www.gob.mx/cms/uploads/attachment/file/104507/DR_3202.pdf (2015).

[280] CONAGUA. Actualización de la disponibilidad media anual de agua en el acuífero Huatulco (2011), estado de Oaxaca. https://sigagis.conagua.gob.mx/gas1/Edos_Acuiferos_18/oaxaca/DR_2011.pdf (2020).

[281] CONAGUA. Actualización de la disponibilidad media anual de agua en el acuífero La Blanca (3228), estado de Zacatecas. https://www.gob.mx/cms/uploads/attachment/file/104536/DR_3228.pdf (2015).

[282] CONAGUA. Actualización de la disponibilidad media anual de agua en el acuífero Lampazos Villaldama (1901), estado de Nuevo León. https://sigagis.conagua.gob.mx/gas1/Edos_Acuiferos_18/nleon/DR_1901.pdf (2020).

[283] CONAGUA. Actualización de la disponibilidad media anual de agua en el acuífero Libres-Oriental (2102), estado de Puebla. https://sigagis.conagua.gob.mx/gas1/Edos_Acuiferos_18/puebla/DR_2102.pdf (2020).

[284] CONAGUA. Actualización de la disponibilidad media anual de agua en el acuífero Loreta (3229), estado de Zacatecas. https://sigagis.conagua.gob.mx/gas1/Edos_Acuiferos_18/zacatecas/DR_3229.pdf (2020).

[285] CONAGUA. Actualización de la disponibilidad media anual de agua en el acuífero Méndez San Fernando (2802), estado de Tamaulipas. https://sigagis.conagua.gob.mx/gas1/Edos_Acuiferos_18/tamaulipas/DR_2802.pdf (2020).

[286] CONAGUA. Actualización de la disponibilidad media anual de agua en el acuífero Navidad-Potosí-Raíces (1916), estado de Nuevo León. https://www.gob.mx/cms/uploads/attachment/file/103175/DR_1916.pdf (2015).

[287] CONAGUA. Actualización de la disponibilidad media anual de agua en el acuífero Ojocaliente (3212), estado de Zacatecas. https://sigagis.conagua.gob.mx/gas1/Edos_Acuiferos_18/zacatecas/DR_3212.pdf (2020).

[288] CONAGUA. Actualización de la disponibilidad media anual de agua en el acuífero Perote-Zalayeta (3004), estado de Veracruz. https://sigagis.conagua.gob.mx/gas1/Edos_Acuiferos_18/veracruz/DR_3004.pdf (2020).

[289] CONAGUA. Actualización de la disponibilidad media anual de agua en el acuífero Pino Suárez (3233), estado de Zacatecas. https://sigagis.conagua.gob.mx/gas1/Edos_Acuiferos_18/zacatecas/DR_3233.pdf (2020).

[290] CONAGUA. Actualización de la disponibilidad media anual de agua en el acuífero Poza Rica (3001), estado de Veracruz. https://sigagis.conagua.gob.mx/gas1/Edos_Acuiferos_18/veracruz/DR_3001.pdf (2020).

[291] CONAGUA. Actualización de la disponibilidad media anual de agua en el acuífero Puerto Madero (3224), estado de Zacatecas. https://sigagis.conagua.gob.mx/gas1/Edos_Acuiferos_18/zacatecas/DR_3224.pdf (2020).

[292] CONAGUA. Actualización de la disponibilidad media anual de agua en el acuífero Río Cañas (2513), estado de Sinaloa. https://sigagis.conagua.gob.mx/gas1/Edos_Acuiferos_18/sinaloa/DR_2513.pdf (2020).

[293] CONAGUA. Actualización de la disponibilidad media anual de agua en el acuífero Río Presidio (2509), estado de Sinaloa. https://sigagis.conagua.gob.mx/gas1/Edos_Acuiferos_18/sinaloa/DR_2509.pdf (2020).

[294] CONAGUA. Actualización de la disponibilidad media anual de agua en el acuífero Río Sinaloa (2502), estado de Sinaloa. https://sigagis.conagua.gob.mx/gas1/Edos_Acuiferos_18/sinaloa/DR_2502.pdf (2020).

[295] CONAGUA. Actualización de la disponibilidad media anual de agua en el acuífero Sabinas (3201), estado de Zacatecas. https://sigagis.conagua.gob.mx/gas1/Edos_Acuiferos_18/zacatecas/DR_3201.pdf (2020).

[296] CONAGUA. Actualización de la disponibilidad media anual de agua en el acuífero Sain Alto (3216), estado de Zacatecas. https://sigagis.conagua.gob.mx/gas1/Edos_Acuiferos_18/zacatecas/DR_3216.pdf (2020).

[297] CONAGUA. Actualización de la disponibilidad media anual de agua en el acuífero Sabinas-Parás (1902), estado de Nuevo León. https://sigagis.conagua.gob.mx/gas1/Edos_Acuiferos_18/nleon/DR_1902.pdf (2020).

[298] CONAGUA. Actualización de la disponibilidad media anual de agua en el acuífero San Felipe-Punta Estrella (0222), estado de Baja California. https://www.gob.mx/cms/uploads/attachment/file/103420/DR_0222.pdf (2015).

[299] CONAGUA. Actualización de la disponibilidad media anual de agua en el acuífero San José de Guaymas (2636), estado de Sonora. https://sigagis.conagua.gob.mx/gas1/Edos_Acuiferos_18/sonora/DR_2636.pdf (2020).

[300] CONAGUA. Actualización de la disponibilidad media anual de agua en el acuífero Valle de Canatlán (1002), estado de Durango. https://sigagis.conagua.gob.mx/gas1/Edos_Acuiferos_18/durango/DR_1002.pdf (2020).

[301] CONAGUA. Actualización de la disponibilidad media anual de agua en el acuífero Valle de Escuinapa (2511), estado de Sinaloa. https://sigagis.conagua.gob.mx/gas1/Edos_Acuiferos_18/sinaloa/DR_2511.pdf (2020).

[302] CONAGUA. Actualización de la disponibilidad media anual de agua en el acuífero Vanegas-Catorce (2401), estado de San Luis Potosi. https://sigagis.conagua.gob.mx/gas1/Edos_Acuiferos_18/sanluispotosi/DR_2401.pdf (2020).

[303] CONAGUA. Actualización de la disponibilidad media anual de agua en el acuífero Vicente Guerrero-Poanas (1004), estado de Durango. https://sigagis.conagua.gob.mx/gas1/Edos_Acuiferos_18/durango/DR_1004.pdf (2020).

[304] CONAGUA. Actualización de la disponibilidad media anual de agua en el acuífero Villa de Arriaga (2406), estado de San Luis Potosi. https://sigagis.conagua.gob.mx/gas1/Edos_Acuiferos_18/sanluispotosi/DR_2406.pdf (2020).

[305] CONAGUA. Actualización de la disponibilidad media anual de agua en el acuífero Villa García (3213), estado de Zacatecas. https://sigagis.conagua.gob.mx/gas1/Edos_Acuiferos_18/zacatecas/DR_3213.pdf (2020).

[306] CONAGUA. Actualización de la disponibilidad media anual de agua en el acuifero Orizaba-Córdoba (3007), estado de Veracruz. https://www.gob.mx/cms/uploads/attachment/file/104452/DR_3007.pdf (2015).

[307] CONAGUA. Acuíferos (nacional). https://sinav30.conagua.gob.mx:8080/SINA/?opcion=acuiferos (2021).

[308] CONAGUA. Aguas subterráneas/Acuíferos. https://sigagis.conagua.gob.mx/aprovechamientos/ (2021).

[309] Connecticut Department of Energy & Environmental Protection. Overview of the Ground Water Flow System in Connecticut. https://portal.ct.gov/DEEP/Aquifer-Protection-and-Groundwater/Ground-Water/Ground-Water-Flow-System-in-Connecticut (2021).

[310] Contoux C, Violette S, Vivona R, Goblet P, Patriarche D (2013). How basin model results enable the study of multi-layer aquifer response to pumping: the Paris Basin, France. Hydrol. J..

[311] Cook, P. G., Jolly, I. D., Leaney, F. W. J. Groundwater recharge in the Mallee region, and salinity implications for the Murray River: a review. CSIRO Land and Water report. https://publications.csiro.au/publications/publication/PIprocite:ef08494d-43a2-4dae-bda4-3d72a62e673f/SQ%22Groundwater%20recharge%20in%20the%20Mallee%20Region%2C%20and%20salinity%22/RP1/RS25/RORECENT/STsearch-by-keyword/LISEA/RI1/RT1 (2001).

[312] Courtois N (2010). Large‐scale mapping of hard‐rock aquifer properties applied to Burkina Faso. Groundwater.

[313] Cox, S. E. & Kahle, S. C. Hydrogeology, ground-water quality, and sources of nitrate in lowland glacial aquifers of Whatcom County, Washington, and British Columbia, Canada. U.S. Geological Survey Water-Resources Investigations Report 98-4195. https://pubs.er.usgs.gov/publication/wri984195 (1999).

[314] Craig, T. W. *Ground Water of the Uncompahgre Valley Montrose County, Colorado*. MSc thesis, Univ. Missouri-Rolla (1971).

[315] Cresswell RG, Jacobson G, Wischusen J, Fifield LK (1999). Ancient groundwaters in the Amadeus Basin, Central Australia: evidence from the radio-isotope ^36^Cl. J. Hydrol..

[316] Cresswell, R. & Gibson, D. Application of Airborne Geophysical Techniques to Groundwater Resource Issues in the Angas-Bremer Plains, South Australia. South Australia Salinity Mapping and Management Support Project Report No. DWLBC 2004/35, Land and Biodiversity Services Division, Department of Water, Land and Biodiversity Conservation. http://angasbremerwater.org.au/documents/abplains_summary.pdf (2004).

[317] Crosbie RS, Rachakonda PK (2021). Constraining probabilistic chloride mass-balance recharge estimates using baseflow and remotely sensed evapotranspiration: the Cambrian Limestone Aquifer in northern Australia. Hydrol. J..

[318] Crow, R. S. et al. The Colorado River and its deposits downstream from Grand Canyon in Arizona, California, and Nevada. U.S. Geological Survey Open-File Report 2018-1005. https://pubs.usgs.gov/of/2018/1005/ofr20181005.pdf (2018).

[319] Crowley, J. J., LaFave, J. I., Bergantino, R. N., Carstarphen, C. A. & Patton, T. W. Principle Aquifers of Montana. Montana Bureau of Mines and Geology Hydrogeologic Map 11. https://www.leg.mt.gov/content/Committees/Interim/2017-2018/Water-Policy/Meetings/Jan-2018/Exhibits/Jan9/Exhibit5.pdf (2017).

[320] Currell M, Banfield D, Cartwright I, Cendón DI (2017). Geochemical indicators of the origins and evolution of methane in groundwater: Gippsland Basin, Australia. Environ. Sci. Pollut. Res..

[321] Currell M, Cendón DI, Cheng X (2013). Analysis of environmental isotopes in groundwater to understand the response of a vulnerable coastal aquifer to pumping: Western Port Basin, south-eastern Australia. Hydrol. J..

[322] Currie, D. et al. Investigating the impact of climate change on groundwater resources: Aquifer characterisation. Report to the National Water Commission. https://publications.csiro.au/rpr/download?pid=csiro:EP202082&dsid=DS3 (2010).

[323] Custodio E (2016). Groundwater intensive use and mining in south-eastern peninsular Spain: hydrogeological, economic and social aspects. Sci. Total Environ..

[324] Cutshall I (1949). Urban settlement in Hokkaido. Econ. Geogr..

[325] Dadgar MA, Zeaieanfirouzabadi P, Dashti M, Porhemmat R (2017). Extracting of prospective groundwater potential zones using remote sensing data, GIS, and a probabilistic approach in Bojnourd basin, NE of Iran. Arab. J. Geosci..

[326] Dalmau, A. B., Gimena, E. C. & Vierbücher, C. L. Las aguas subterráneas en el delta del ebro. Revista de Obras Públicas, 3.36847. https://rac.es/ficheros/doc/00538.pdf (1997).

[327] Danis C (2014). Use of groundwater temperature data in geothermal exploration: the example of Sydney Basin, Australia. Hydrol. J..

[328] Dar FA (2011). Karstification in the Cuddapah Sedimentary Basin, southern India: implications for groundwater resources. Acta Carsologica.

[329] Das PP (2020). Saline contamination Mahanadi deltaic aquifers: a review. Proc. Indian Natl Sci. Acad..

[330] Das, S. & Prakash, I. in *Proc. 6th International Conference on Case Histories in Geotechnical Engineering.* https://core.ac.uk/download/pdf/229070665.pdf (2008).

[331] Daskin, W. R. Preliminary evaluation of the hydrogeologic system in Owens Valley, California. U.S. Geological Survey Water-Resources Investigations Report 88-4003. https://pubs.usgs.gov/wri/1988/4003/report.pdf (1988).

[332] Davidson, B. Kentucky Interagency Groundwater Monitoring Network: Annual Report July 2017–June 2018. http://www.uky.edu/KGS/water/gnet/itac17-18.pdf (2018).

[333] Davidson, W. A. & Yu, X. Perth region aquifer modelling system — PRAMS, hydrogeology and groundwater modelling. Western Australia Department of Water Hydrogeological Record Series HG20. https://www.wa.gov.au/system/files/2022-04/Perth-Region-Aquifer-Modelling-System-%28PRAMS%29-hydrogeology-and-groundwater-modelling.pdf (2006).

[334] Davies, H. & Hanley, P. T. State of the Watershed Report - 2010. Water Security Agency, Saskatchewan. Appendix A. https://www.wsask.ca/wp-content/uploads/2021/02/a_2010StateoftheWatershedReport.pdf (2010).

[335] Davies-Smith, A., Bolke, E. L. & Collins, C. A. Geohydrology and digital simulation of the ground-water flow system in the Umatilla Plateau and Horse Heaven Hills area, Oregon and Washington. U.S. Geological Survey Water-Resources Investigations Report 87-4268. https://pubs.usgs.gov/wri/1987/4268/report.pdf (1988).

[336] Davis, H. Hydrogeologic investigation and simulation of ground-water flow in the Upper Floridan aquifer of North-Central Florida and Southwestern Georgia and delineation of contributing areas for selected city of Tallahassee, Florida, water-supply wells. U.S. Geological Survey Water-Resources Investigations Report 95-4296. https://fl.water.usgs.gov/PDF_files/wri95_4296_davis.pdf (1996).

[337] Day JC (1978). International aquifer management: the Hueco Bolson on the Rio Grande River. Nat. Resour. J..

[338] de Caritat P (2019). Groundwater geochemistry, hydrogeology and potash mineral potential of the Lake Woods region, Northern Territory, Australia. Aust. J. Earth Sci..

[339] de la Losa A, Moreno L, Nunez EL (2010). Calidad química de las aguas subterráneas en una zona de actividad minera (Cuenca del Bierzo- León). Bol. Geol. Min..

[340] de Lourdes Corral-Bermudez M, Sánchez-Ortiz E, Álvarez-Bernal D, Gutiérrez-Montenegro MO, Cassio-Madrazo E (2019). Scenarios of availability of water due to overexploitation of the aquifer in the basin of Laguna de Santiaguillo, Durango, Mexico. PeerJ.

[341] De Melo MC, Paquete PC, Da Silva MM (2001). Evolution of the Aveiro Cretaceous aquifer (NW Portugal) during the Late Pleistocene and present day: evidence from chemical and isotopic data. Geol. Soc. Lond. Spec. Publ..

[342] de Montety V (2008). Origin of groundwater salinity and hydrogeochemical processes in a confined coastal aquifer: case of the Rhône delta (Southern France). Appl. Geochem..

[343] de Souza EL (2013). Síntese da hidrogeologia nas bacias sedimentares do Amazonas e do Solimões: Sistemas Aquíferos Içá-Solimões e Alter do Chão. Geol. USP Série Científica.

[344] Deeds, N. E. et al. Final conceptual model report for the High Plains Aquifer System groundwater availability model. Texas Water Development Board report. https://www.twdb.texas.gov/groundwater/models/gam/hpas/HPAS_GAM_Conceptual_Report.pdf (2015).

[345] Deolankar SB (1980). The Deccan basalts of Maharashtra, India—their potential as aquifers. Groundwater.

[346] Department of Environment and Water of the Government of South Australia. Lower Limestone Coast PWA Unconfined Aquifer. 2017 groundwater level and salinity status report. https://www.waterconnect.sa.gov.au/Content/Publications/DEW/Lower_Limestone_Coast_PWA_Unconfined_GSR_2017.pdf (2017).

[347] Department of Environment, Water and Natural Resources of the Government of South Australia. Booborowie Valley. Groundwater level and salinity status report. https://www.waterconnect.sa.gov.au/Content/Publications/DEW/Booborowie_Valley_Status_Report_2011.pdf (2011).

[348] Department of Science, IT, Innovation and the Arts. Mulgrave River basin hydrology - development of groundwater flow model for the Mulgrave River basin. Report prepared for the Department of Natural Resources and Mines for the Wet Tropics Draft Water Resource Plan. https://nla.gov.au/nla.obj-2742766628/view (2013).

[349] Department of Water Affairs and Forestry, South Africa. Vaal River system: large bulk water supply reconciliation strategy: groundwater assessment: dolomite aquifers. DWAF Report Number: P RSA C000/00/4406/06. https://www.dws.gov.za/iwrp/Vaal/documents/LargeBulkWater/06_Dolomitic%20Groundwater%20Assessment_Final.pdf (2006).

[350] Department of Water and Sanitation. Groundwater status report - Western Cape Region. Department of Water and Sanitation map. https://www.dws.gov.za/Groundwater/GroundwaterOffices/WC/Annual%20report_%20groundwater%20status%20A0%20-%20201503.pdf (2015).

[351] Deshpande, R. D. *Groundwater in and Around Cambay Basin, Gujarat: Some Geochemical and Isotopic Investigations*. PhD thesis, Physical Research Laboratory (2006).

[352] Dever L, Travi Y, Barbecot F, Marlin C, Gibert E (2001). Evidence for palaeowaters in the coastal aquifers of France. Geol. Soc. Lond. Spec. Publ..

[353] Dhar A (2015). Hydro-environmental assessment of a regional ground water aquifer: Hirakud command area (India). Environ. Earth Sci..

[354] Dhinagaran, V. District Groundwater Brochure Thanjavur District, Tamil Nadu. Central Ground Water Board, Ministry of Water Resources report. http://cgwb.gov.in/old_website/District_Profile/TN_districtprofile.html (2009).

[355] Díaz González, T. E. & Penas, Á. in *The Vegetation of the Iberian Peninsula* Vol. 12 (ed. Loidi, J.) 251–321 (Springer, 2017).

[356] Direccion General de Aguas. Analisis de disponibilidad de recursos hidricos subterraneous en el sector hidrogeologico de aprovechamiento comun Huasco Desembocadura, Cuencas Rio Huasco. Report No. 14593214. https://dga.mop.gob.cl/Decretos_Escacez/0303-2.pdf (2021).

[357] Direccion General de Aguas. Diagnóstico y Clasificación de Sectores Acuíferos, Volumen No. 2. Gobierno de Chile Ministerio de Obras Publicas report. https://snia.mop.gob.cl/sad/CQA5168v2.pdf (2009).

[358] Direccion General de Aguas. Inventario Nacional de acuiferos. Ministerio De Obras Públicas (Gobierno De Chile) report number 403. https://snia.mop.gob.cl/sad/SUB5748.pdf (2017).

[359] Direccion General de Aguas. Plan Nacional de Estudios Acuíferos. Report number 381. https://bibliotecadigital.ciren.cl/bitstream/handle/20.500.13082/32415/DGA_2015_actualizacion_plan_nacional_acuiferos.pdf?sequence=1&isAllowed=y (2015).

[360] Divine, D. & Sibray, S. S. An overview of secondary aquifers in Nebraska. Conservation and Survey Division, Educational Circular No. 26. https://core.ac.uk/download/pdf/127441451.pdf (2017).

[361] D’Lugosz, J. J. & McClaflin, R. G. Geohydrology of the Vamoosa-Ada aquifer east-central Oklahoma with a section on chemical quality of water. U.S. Geological Survey Circular 87. http://www.ogs.ou.edu/pubsscanned/Circulars/circular87mm.pdf (1986).

[362] Dong L, Guo Y, Tang W, Xu W, Fan Z (2022). Statistical evaluation of the influences of precipitation and river level fluctuations on groundwater in Yoshino River Basin, Japan. Water.

[363] Donoso, G., Lictevout, E. & Rinaudo, J.-D. in *Sustainable Groundwater Management*. (eds Rinaudo, J. D., Holley, C., Barnett, S. & Montginoul, M.) 481–509 (Springer, 2020).

[364] Dörfler, M. *Analysis of Aquifer-induced Soil Movements of Heterogeneous Subsoil in Urban Areas Based on Groundwater, Borehole and InSAR Data, a Case Study of Salzburg*. Masters thesis, Paris-Lodron-Univ. Salzburg (2021).

[365] Douglas AA, Osiensky JL, Keller CK (2007). Carbon-14 dating of ground water in the Palouse Basin of the Columbia River basalts. J. Hydrol..

[366] Downey, J. S. Geohydrology of the Madison and associated aquifers in parts of Montana, North Dakota, South Dakota, and Wyoming. U.S. Geological Survey Professional Paper 1273-G. https://pubs.usgs.gov/pp/1273g/report.pdf (1982).

[367] Doyle, W. W. Ground water in the Arica Area, Chile. Article number 170. Short Papers in Geology and Hydrology Articles 122–172. U.S. Geological Survey Professional Paper 475-D, D213–D215 (1964).

[368] Driscoll, D. G., Carter, J. M., Williamson, J. E. & Putnam, L. D. Hydrology of the Black Hills area, South Dakota. U.S. Geological Survey Water-Resources Investigations Report 2002-4094. https://pubs.usgs.gov/wri/wri024094/pdf/wri024094.pdf (2002).

[369] Duell Jr, L. F. W. Geohydrology of the Antelope Valley area, California, and design for a ground-water-quality monitoring network. U.S. Geological Survey Water-Resources Investigations Report 84-4081. https://pubs.usgs.gov/wri/1984/4081/report.pdf (1987).

[370] Dumont A, Salmoral G, Llamas MR (2013). The water footprint of a river basin with a special focus on groundwater: the case of Guadalquivir basin (Spain). Water Resour. Ind..

[371] Dunlop G, Palanichamy J, Kokkat A, James EJ, Palani S (2019). Simulation of saltwater intrusion into coastal aquifer of Nagapattinam in the lower cauvery basin using SEAWAT. Groundw. Sustain. Dev..

[372] Duque C, Calvache ML, Engesgaard P (2010). Investigating river–aquifer relations using water temperature in an anthropized environment (Motril-Salobreña aquifer). J. Hydrol..

[373] Duraiswami, R. A., Das, S. & Shaikh, T. Hydrogeological framework of aquifers from the Deccan Traps, India: some insights. *Mem. Geol. Soc. India*, 1–15 (2012).

[374] Dustin, J. D. *Hydrogeology of Utah Lake with Emphasis on Goshen Bay*. PhD dissertation, Brigham Young Univ. (1978).

[375] Dutta PK (2015). Resolving Kamthi-related problems in Gondwana stratigraphy of peninsular India. Indian J. Geosci..

[376] Ebadati N, Sepavandi S (2015). Role of geological structures and lithology in the quantitative and qualitative changes of Eshtehard aquifers. Iran. J. Ecohydrol..

[377] Ebrahim GY, Villholth KG, Boulos M (2019). Integrated hydrogeological modelling of hard-rock semi-arid terrain: supporting sustainable agricultural groundwater use in Hout catchment, Limpopo Province, South Africa. Hydrol. J..

[378] Ebrahimi Varzane S, Zarei H, TishehZan P, Akhondali AM (2019). Evaluation of groundwater-surface water interaction by using cluster analysis (case study: western part of Dezful-Andimeshk plain). Iran Water Resour. Res..

[379] Ebrahimi M, Kazemi H, Ehtashemi M, Rockaway TD (2016). Assessment of groundwater quantity and quality and saltwater intrusion in the Damghan basin, Iran. Geochemistry.

[380] Echogdali FZ (2023). Characterization and productivity of alluvial aquifers in sustainability oasis areas: a case study of the Tata watershed (southeast Morocco). Appl. Sci..

[381] Edalat A, Khodaparast M, Rajabi AM (2020). Scenarios to control land subsidence using numerical modeling of groundwater exploitation: Aliabad plain (in Iran) as a case study. Environ. Earth Sci..

[382] Ehya F, Saeedi F (2019). Assessment of groundwater quality in the Garmez area (Southeastern Khuzestan province, SW Iran) for drinking and irrigation uses. Carbonates Evaporites.

[383] Eimers, J. L., Daniel III, C. C. & Coble, R. W. Hydrogeology and simulation of ground-water flow at U.S. Marine Corps Air Station, Cherry Point, North Carolina, 1987-90. U.S. Geological Survey Water-Resources Investigations Report 94-4186. https://pubs.usgs.gov/wri/1994/4186/report.pdf (1994).

[384] El Mahdad, E. et al. in *The Souss‐Massa River Basin, Morocco* (eds Choukr-Allah, R., Ragab, R., Bouchaou, L. & Barceló, D.) 303–333 (Springer, 2017).

[385] Ellis, J. H. et al. Hydrogeology and simulation of groundwater flow and analysis of projected water use for the Canadian River alluvial aquifer, western and central Oklahoma. U.S. Geological Survey Scientific Investigations Report 2016-5180. https://pubs.usgs.gov/sir/2016/5180/sir20165180.pdf (2017).

[386] Emami S, Hemmati M, Arvanaghi H (2018). Performance evaluation of Imperialist Competitive and Genetic algorithm for estimating groundwater quality parameters (case study: Bostanabad plain). Hydrogeology.

[387] Erostate M (2018). Delayed nitrate dispersion within a coastal aquifer provides constraints on land-use evolution and nitrate contamination in the past. Sci. Total Environ..

[388] Eslamizadeh A, Samanirad S (2010). Land subsidence and fissuring due to ground water withdrawal in Yazd-Ardakan basin, central Iran. World Acad. Sci. Eng. Technol..

[389] Esmaeili-Vardanjani M, Rasa I, Yazdi M, Pazand K (2016). The hydrochemical assessment of groundwater resources in the Kadkan basin, Northeast of Iran. Carbonates Evaporites.

[390] Esteban E, Albiac J (2012). The problem of sustainable groundwater management: the case of La Mancha aquifers, Spain. Hydrol. J..

[391] Esteve P, Varela-Ortega C, Blanco-Gutiérrez I, Downing TE (2015). A hydro-economic model for the assessment of climate change impacts and adaptation in irrigated agriculture. Ecol. Econ..

[392] Evans, S. Baroota Groundwater Resource – Monitoring Review and Augmentation. Department of Water, Land and Biodiversity Conservation Report No. 2004/56. https://www.waterconnect.sa.gov.au/Content/Publications/DEW/dwlbc_report_2004_56.pdf (2004).

[393] Everett, R. R. et al. Geology, water-quality, hydrology, and geomechanics of the Cuyama Valley groundwater basin, California, 2008–12. U.S. Geological Survey Scientific Investigations Report 2013-5108. https://pubs.usgs.gov/sir/2013/5108/pdf/sir2013-5108.pdf (2013).

[394] Ezquerro, P. et al. Groundwater and subsidence modeling combining geological and multi-satellite SAR data over the alto Guadalentín Aquifer (SE Spain). *Geofluids*, 1359325. 10.1155/2017/1359325 (2017).

[395] Faghihi N, Kave F, Babazadeh H (2010). Prediction of aquifer reaction to different hydrological and management scenarios using visual MODFLOW model-case study of Qazvin plain. J. Water Sci. Res..

[396] Fallahi MM, Shabanlou S, Rajabi A, Yosefvand F, IzadBakhsh MA (2023). Effects of climate change on groundwater level variations affected by uncertainty (case study: Razan aquifer). Appl. Water Sci..

[397] Fang J, Ding YJ (2010). Assessment of groundwater contamination by NO_3_^−^ using geographical information system in the Zhangye Basin, Northwest China. Environ. Earth Sci..

[398] Faunt, C. C. et al. Hydrogeology, hydrologic effects of development, and simulation of groundwater flow in the Borrego Valley, San Diego County, California. U.S. Geological Survey Scientific Investigations Report 2015-5150. https://pubs.usgs.gov/sir/2015/5150/sir20155150.pdf (2015).

[399] Fayaji I, Sayadi MH, Mousazadeh H (2019). Potable groundwater analysis using multivariate Groundwater Quality Index technique. Glob. J. Environ. Sci. Manag..

[400] Feitosa, F. A., Diniz, J. A. O., Kirchheim, R. E., Kiang, C. H. & Feitosa, E. C. in *Groundwater Assessment, Modeling, and Management* (eds Thangarajan, M. & Singh, V. P.) 33–57 (Routledge, 2016).

[401] Fenelon, J. M. et al. Hydrogeologic atlas of aquifers in Indiana. U.S. Geological Survey Water-Resources Investigations Report 92-4142. https://pubs.er.usgs.gov/publication/wri924142 (1994).

[402] Fenneman, N. M. & Johnson, D. W. Physiographic divisions of the conterminous United States. U.S. Geological Survey map, 1:7,000,000 scale (1946).

[403] Ferguson GA, Betcher RN, Grasby SE (2007). Hydrogeology of the Winnipeg formation in Manitoba, Canada. Hydrol. J..

[404] Fernández‐Chacón F (2010). Isotopic composition (δ^18^O and δD) of precipitation and groundwater in a semi‐arid, mountainous area (Guadiana Menor basin, Southeast Spain). Hydrol. Process..

[405] Ferreira, A. L. Parnaiba Basin. Presentation at “Round 15 - Brazil: Oil and Gas Concessions”. http://www.anp.gov.br/images/Palestras/Seminario_tecnico_R15_P4/Ingles/06_Bacia_do_Parnaiba_R15_INGLES.pdf (2018).

[406] Ferris D, Lypka M, Ferguson G (2017). Hydrogeology of the Judith River formation in southwestern Saskatchewan, Canada. Hydrol. J..

[407] Fijani E, Moghaddam AA, Tsai FTC, Tayfur G (2017). Analysis and assessment of hydrochemical characteristics of Maragheh-Bonab plain aquifer, northwest of Iran. Water Resour. Manag..

[408] Fijani E, Nadiri AA, Moghaddam AA, Tsai FTC, Dixon B (2013). Optimization of DRASTIC method by supervised committee machine artificial intelligence to assess groundwater vulnerability for Maragheh–Bonab plain aquifer, Iran. J. Hydrol..

[409] Finch, S. T., Mccoy, A. & Melis, E. Geologic controls on ground-water flow in the Mimbres Basin, southwestern New Mexico. New Mexico Geological Society Guide Book, 59th Field Conference, 189–198. https://nmgs.nmt.edu/publications/guidebooks/downloads/59/59_p0189_p0198.pdf (2008).

[410] Fisher, C. A. Geology and water resources of the Bighorn Basin, Wyoming. U.S. Geological Survey Professional Paper 53. https://pubs.usgs.gov/pp/0053/report.pdf (1906).

[411] Fix, P. F., Nelson, W. B., Lofgren, B. E. & Butler, R. G. Ground water in the Escalante Valley, Beaver, Iron, and Washington Counties, Utah. Technical Publication 6. https://waterrights.utah.gov/docSys/v920/w920/w9200085.pdf (1950).

[412] Flint, L. E. et al. Geohydrology of Big Bear Valley, California: phase 1—geologic framework, recharge, and preliminary assessment of the source and age of groundwater. U.S. Geological Survey Scientific Investigations Report 2012-5100. https://pubs.usgs.gov/sir/2012/5100/pdf/sir20125100.pdf (2012).

[413] Flora, S. & Davis, T. Hydrologic Map Series (HMS), Water Level Change Map Series (WLCMS), and Basin Sweep Assessment Report ADWR Basins and Sub-Basins. Arizona Department of Water Resources Hydrology Division Field Services Section. https://www.azwater.gov/content/hms-wlcms-and-basin-sweep-assessment-report-2009 (2009).

[414] Florea, L. J., Hasenmueller, N. R., Branam, T. D., Frushour, S. S. & Powell, R. L. in *GSA Field Guide: Ancient Oceans, Orogenic Uplifts, and Glacial Ice: Geologic Crossroads in America’s Heartland* Vol. 51 (ed. Florea, L. J.) 95–112 (Geological Society of America, 2018).

[415] Flores-Márquez EL (2008). Numerical modeling of Etla Valley aquifer, Oax., Mexico: evolution and remediation scenarios. Geofís. Int..

[416] Fontes SL, Meju MA, Maurya VP, La Terra EF, Miquelutti LG (2019). Deep structure of Parecis Basin, Brazil from 3D magnetotelluric imaging. J. S. Am. Earth Sci..

[417] Fortin G, Van Der Kamp G, Cherry JA (1991). Hydrogeology and hydrochemistry of an aquifer-aquitard system within glacial deposits, Saskatchewan, Canada. J. Hydrol..

[418] Foster, S. Thailand: strengthening capacity in groundwater resources management. World Bank Case Profile Collection Number 1. https://documents1.worldbank.org/curated/en/521371468308952444/pdf/388010PAPER0TH1WMATE1CP10101PUBLIC1.pdf (2002).

[419] Foster, S., Garduño, H. & Tuinhof, A. Confronting the groundwater management challenge in the Deccan Traps Country of Maharashtra – India. World Bank Case Profile Collection Number 18 (2007).

[420] Fram, M. S. & Belitz, K. Groundwater quality in the Coastal Los Angeles Basin, California. U.S. Geological Survey Fact Sheet 2012-3096. https://pubs.er.usgs.gov/publication/70039952 (2008).

[421] Frei R (2020). The link between surface water and groundwater-based drinking water–strontium isotope spatial distribution patterns and their relationships to Danish sediments. Appl. Geochem..

[422] Frick, E. Quantitative analysis of groundwater flow in valley-fill deposits in Steptoe Valley, Nevada. Doctoral dissertation, Univ. Nevada (1985).

[423] Frimpter, M. H. & Gay, F. B. Chemical quality of ground water on Cape Cod, Massachusetts. U.S. Geological Survey Water-Resources Investigations Report 79-65. https://pubs.usgs.gov/wri/1979/0065/report.pdf (1979).

[424] Fuchs EH, King JP, Carroll KC (2019). Quantifying disconnection of groundwater from managed‐ephemeral surface water during drought and conjunctive agricultural use. Water Resour. Res..

[425] Fuentes-Arreazola MA, Ramírez-Hernández J, Vázquez-González R (2018). Hydrogeological properties estimation from groundwater level natural fluctuations analysis as a low-cost tool for the Mexicali Valley aquifer. Water.

[426] Fürst J, Bichler A, Konecny F (2015). Regional frequency analysis of extreme groundwater levels. Groundwater.

[427] Furuno K, Kagawa A, Kazaoka O, Kusuda T, Nirei H (2015). Groundwater management based on monitoring of land subsidence and groundwater levels in the Kanto Groundwater Basin, Central Japan. Proc. Int. Assoc. Hydrol. Sci..

[428] Gale, I. N. & Rutter, H. K. The Chalk aquifer of Yorkshire. British Geological Survey Research Report RR/06/04. http://nora.nerc.ac.uk/id/eprint/3700/1/RR06004.pdf (2006).

[429] Gan Y (2014). Hydrogeochemistry and arsenic contamination of groundwater in the Jianghan Plain, central China. J. Geochem. Explor..

[430] Gannett, M. W., Lite, K. E., La Marche, J. L., Fisher, B. J. & Polette, D. J. Ground-water hydrology of the upper Klamath Basin, Oregon and California. U.S. Geological Survey Scientific Investigations Report 2007-5050. https://pubs.usgs.gov/sir/2007/5050/pdf/sir20075050.pdf (2007).

[431] Gannett, M. W. & Breen, K. H. Groundwater levels, trends, and relations to pumping in the Bureau of Reclamation Klamath Project, Oregon and California. U.S. Geological Survey Open-File Report 2015-1145. https://pubs.usgs.gov/of/2015/1145/ofr20151145.pdf (2015).

[432] Gannett, M. W., Lite Jr, K. E., Morgan, D. S. & Collins, C. A. Ground-water hydrology of the upper Deschutes Basin, Oregon. U.S. Geological Survey Water-Resources Investigations Report 00-4162. https://pubs.usgs.gov/wri/wri004162/ (2001).

[433] Gao X, Wang Y, Li Y, Guo Q (2007). Enrichment of fluoride in groundwater under the impact of saline water intrusion at the salt lake area of Yuncheng basin, northern China. Environ. Geol..

[434] García-Meléndez, E., Ferrer Julià, M., Goy, J. L. & Zazo, C. Reconstrucción morfoestructural mediante modelos de elevación digital en un SIG del fondo de la cuenca sedimentaria de la Cubeta del Saltador (Cordilleras Béticas Orientales). https://digital.csic.es/handle/10261/247828 (2002).

[435] Gardner, P. M. & Kirby, S. Hydrogeologic and geochemical characterization of groundwater resources in Rush Valley, Tooele County, Utah. U.S. Geological Survey Scientific Investigations Report 2011-5068. https://pubs.usgs.gov/sir/2011/5068/pdf/sir20115068.pdf (2011).

[436] Gardner, P. M. & Masbruch, M. D. Hydrogeologic and geochemical characterization of groundwater resources in Deep Creek Valley and adjacent areas, Juab and Tooele Counties, Utah, and Elko and White Pine Counties, Nevada. U.S. Geological Survey Scientific Investigations Report 2015-5097. https://pubs.usgs.gov/sir/2015/5097/sir20155097.pdf (2015).

[437] Garduño, H. & Foster, S. Sustainable groundwater irrigation. Approaches to reconciling demand with resources. GW•MATE Strategic Overview Series Number 4, World Bank. https://openknowledge.worldbank.org/server/api/core/bitstreams/a6957092-3680-52cd-9707-91143c386175/content (2010).

[438] Garzon-Vidueira R (2020). Identification of nitrates origin in Limia river basin and pollution-determinant factors. Agric. Ecosyst. Environ..

[439] Gastmans D, Chang HK, Hutcheon I (2010). Stable isotopes (^2^H, ^18^O and ^13^C) in groundwaters from the northwestern portion of the Guarani Aquifer System (Brazil). Hydrol. J..

[440] Geological Survey of Alabama. Assessment of groundwater resources in Alabama, 2010-16. Geological Survey of Alabama Bulletin 186. https://www.gsa.state.al.us/img/Groundwater/docs/assessment/00_B186_StatewideAssessment_Print_Document.pdf (2018).

[441] George BG, Ray JS, Kumar S (2019). Geochemistry of carbonate formations of the Chhattisgarh Supergroup, central India: implications for Mesoproterozoic global events. Can. J. Earth Sci..

[442] George ME, Babu DS, Akhil T, Rafeeque MK (2018). Investigation on submarine groundwater discharge at Kozhikkode Coastal Aquifer, SW Western Ghats. J. Geol. Soc. India.

[443] Gerber RE, Howard K (2002). Hydrogeology of the Oak Ridges Moraine aquifer system: implications for protection and management from the Duffins Creek watershed. Can. J. Earth Sci..

[444] Ghadimi F, Ghomi M (2012). Statistical analysis of the hydrogeochemical evolution of groundwater in alluvial aquifer of Arak Mighan playa, Markazi province, Iran. J. Water Sci. Res..

[445] Ghafari S, Banihabib ME, Javadi S (2020). A framework to assess the impact of a hydraulic removing system of contaminate infiltration from a river into an aquifer (case study: Semnan aquifer). Groundw. Sustain. Dev..

[446] Ghafari S, Moradi H, Modares R (2018). Comparison of temporal and spatial changes of groundwater level in Isfahan-Borkhar, Najafabad and Chadegan Plains. Phys. Geogr. Res. Q..

[447] Ghanbari N, Rangzan K, Kabolizade M, Moradi P (2017). Improve the results of the DRASTIC model using artificial intelligence methods to assess groundwater vulnerability in Ramhormoz alluvial aquifer plain. J. Water Soil Conserv..

[448] Ghazavi R, Ebrahimi Z (2015). Assessing groundwater vulnerability to contamination in an arid environment using DRASTIC and GOD models. Int. J. Environ. Sci. Technol..

[449] Ghazaw YM, Ghumman AR, Al-Salamah I, Khan QU (2014). Investigations of impact of recharge wells on groundwater in Buraydah by numerical modeling. Arab. J. Sci. Eng..

[450] Ghazifard A, Moslehi A, Safaei H, Roostaei M (2016). Effects of groundwater withdrawal on land subsidence in Kashan Plain, Iran. Bull. Eng. Geol. Environ..

[451] Ghobadi A, Cheraghi M, Sobhanardakani S, Lorestani B, Merrikhpour H (2020). Hydrogeochemical characteristics, temporal, and spatial variations for evaluation of groundwater quality of Hamedan–Bahar Plain as a major agricultural region, West of Iran. Environ. Earth Sci..

[452] Gholami F, Malekian A (2018). Assessment of spatio-temporal oscillations and physico-chemical properties of Azna-Aligudarz basin. Desert Ecosyst. Eng. J..

[453] Gholami VCKW, Chau KW, Fadaee F, Torkaman J, Ghaffari A (2015). Modeling of groundwater level fluctuations using dendrochronology in alluvial aquifers. J. Hydrol..

[454] Ghoochanian, E., Etebari, B. & Akbarpour, A. Integrating groundwater management with WEAP and MODFLOW models (case study: Birjand Plain, east of Iran). MODFLOW and More, 2–5 (2013).

[455] Ghorbani H, Sadabad SM (2010). Annual changes in some qualitative parameters of groundwater in Shirvan Plain North East of Iran. World Acad. Eng. Technol..

[456] Gill, H. E. & Farlekas, G. M. Geohydrologic maps of the Potomac-Raritan-Magothy aquifer system in the New Jersey Coastal Plain. U.S. Geological Survey Hydrologic Atlas 557. https://pubs.er.usgs.gov/publication/ha557 (1976).

[457] Giménez-Forcada E (2014). Space/time development of seawater intrusion: a study case in Vinaroz coastal plain (Eastern Spain) using HFE-Diagram, and spatial distribution of hydrochemical facies. J. Hydrol..

[458] Giménez-Forcada E (2019). Use of the Hydrochemical Facies Diagram (HFE-D) for the evaluation of salinization by seawater intrusion in the coastal Oropesa Plain: comparative analysis with the coastal Vinaroz Plain, Spain. HydroResearch.

[459] Gingerich, S. B. The effects of withdrawals and drought on groundwater availability in the Northern Guam Lens Aquifer, Guam. U.S. Geological Survey Scientific Investigations Report 2013-5216. https://pubs.usgs.gov/sir/2013/5216/pdf/sir2013-5216.pdf (2013).

[460] Goderniaux P, Orban P, Rorive A, Brouyère S, Dassargues A (2023). Study of historical groundwater level changes in two Belgian chalk aquifers in the context of climate change impacts. Geol. Soc. Lond. Spec. Publ..

[461] Godfrey LV (2021). δ^13^C and ^14^C activity of groundwater DOC and DIC in the volcanically active and arid Loa Basin of northern Chile. J. Hydrol..

[462] Godfrey, L. & van Dyk, G. Reserve determination for the Pomfret-Vergelegen Dolomitic Aquifer, North West province. Report No ENV-P-C 2002 -031. https://scholar.ufs.ac.za/bitstream/handle/11660/7396/Tosca%20Reserve%20Report.pdf?sequence=6&isAllowed=y (2002).

[463] Golchin I, Moghaddam MA (2016). Hydro-geochemical characteristics and groundwater quality assessment in Iranshahr plain aquifer, Iran. Environ. Earth Sci..

[464] Golder Associates and Summit Environmental Consultants Ltd. Phase 2 Okanagan Water Supply and Demand Project: Groundwater Objectives 2 and 3 Basin Study. Report to Okanagan Basin Water Board. https://www.obwb.ca/wsd/about/project-reports (2009).

[465] Gomo M, Vermeulen D (2017). A transboundary aquifer of potential concern in Southern Africa. Water Policy.

[466] Gonçalves RD, Teramoto EH, Chang HK (2020). Regional groundwater modeling of the Guarani Aquifer System. Water.

[467] González-Trinidad J, Pacheco-Guerrero A, Júnez-Ferreira H, Bautista-Capetillo C, Hernández-Antonio A (2017). Identifying groundwater recharge sites through environmental stable isotopes in an alluvial aquifer. Water.

[468] Gopinath S (2018). Hydrochemical characteristics and salinity of groundwater in parts of Nagapattinam district of Tamil Nadu and the Union Territory of Puducherry, India. Carbonates Evaporites.

[469] Gordon, A. D., Carleton, G. B. & Rosman, R. Water-level conditions in the confined aquifers of the New Jersey Coastal Plain, 2013. U.S. Geological Survey Scientific Investigations Report 2019-5146. https://pubs.usgs.gov/sir/2019/5146/sir20195146.pdf (2021).

[470] Gordon, C. H. Geology and underground waters of the Wichita region, north-central Texas. U.S. Geological Survey Water-Supply Paper 317. https://pubs.usgs.gov/wsp/0317/report.pdf (1913).

[471] Goswami S, Dey S, Zakaulla S, Verma MB (2020). Active rifting and bimodal volcanism in Proterozoic Papaghni sub-basin, Cuddapah basin (Andhra Pradesh), India. J. Earth Syst. Sci..

[472] Goumehei E, Geravandi Y, Wanglin YAN (2016). A GIS-based study to investigate effect of water table changes on DRASTIC model: a case study of Kermanshah, Iran. Int. J. Environ. Geoinformatics.

[473] Government of Western Australia’s Department of Water. Northern Perth Basin: geology, hydrogeology and groundwater resources. Department of Water Hydrological Bulletin Series Report No. HB1. https://www.wa.gov.au/system/files/2022-04/Northern%20Perth%20Basin%20-%20geology%2C%20hydrogeology%20and%20groundwater%20resources.pdf (2017).

[474] Government of Western Australia’s Department of Water. West Canning Basin groundwater allocation limit report. Water resource allocation and planning report series, Report No. 52. https://www.wa.gov.au/system/files/2022-10/West-Canning-Basin-groundwater-allocation-limit-report.pdf (2012).

[475] Graham, W. G. & Campbell, L. J. Groundwater resources of Idaho. Idaho Department of Water Resources report. https://idwr.idaho.gov/wp-content/uploads/sites/2/publications/198108-MISC-GW-Resources-ID.pdf (1981).

[476] Grande JA, González A, Beltran R, Sánchez‐Rodas D (1996). Application of factor analysis to the study of contamination in the aquifer system of Ayamonte‐Huelva (Spain). Groundwater.

[477] Grasby SE, Betcher RN (2002). Regional hydrogeochemistry of the carbonate rock aquifer, southern Manitoba. Can. J. Earth Sci..

[478] Grasby SE, Chen Z, Hamblin AP, Wozniak PR, Sweet AR (2008). Regional characterization of the Paskapoo bedrock aquifer system, southern Alberta. Can. J. Earth Sci..

[479] Graves, R. P. Ground-water resources in Lajas Valley, Puerto Rico. U.S. Geological Survey Water-Resources Investigations Report 89-4182. https://pubs.usgs.gov/wri/1989/4182/report.pdf (1991).

[480] Gray, H. H. Map of Indiana showing physiographic divisions. Indiana Geological Survey Miscellaneous Map 69. https://scholarworks.iu.edu/dspace/bitstream/handle/2022/27232/SR61_A1b.pdf (2001).

[481] Great Barrier Reef Marine Park Authority. Plane Basin Assessment. Mackay Whitsunday Natural Resource Management Region report. https://elibrary.gbrmpa.gov.au/jspui/bitstream/11017/2902/2/Plane-Basin-assessment-2013.pdf (2013).

[482] Greenman, D. W., Bennett, G. D. & Swarzenski, W. V. Ground-water hydrology of the Punjab, West Pakistan, with emphasis on problems caused by canal irrigation. U.S. Geological Survey Water-Supply Paper 1608-H. https://pubs.usgs.gov/wsp/1608h/report.pdf (1967).

[483] Grenholm, O. H. M. The geodynamic evolution of a Paleoproterozoic orogenic system - a local to global perspective on the ca. 2.27-1.96 Ga Birimian Orogen in the Baoule Mossi domain of West Africa. Thesis, Univ. Western Australia (2019).

[484] Guerrero-Martínez L, Hernández-Marín M, Burbey TJ (2018). Estimation of natural groundwater recharge in the Aguascalientes semiarid valley, Mexico. Rev. Mex. Cienc. Geol..

[485] Güler C, Thyne GD (2004). Hydrologic and geologic factors controlling surface and groundwater chemistry in Indian Wells-Owens Valley area, southeastern California, USA. J. Hydrol..

[486] Gunnink JL, Pham HV, Oude Essink GH, Bierkens MF (2021). The three-dimensional groundwater salinity distribution and fresh groundwater volumes in the Mekong Delta, Vietnam, inferred from geostatistical analyses. Earth Syst. Sci. Data.

[487] Guo C, Shi J, Zhang Z, Zhang F (2019). Using tritium and radiocarbon to determine groundwater age and delineate the flow regime in the Taiyuan Basin, China. Arab. J. Geosci..

[488] Guo H, Wang Y (2005). Geochemical characteristics of shallow groundwater in Datong basin, northwestern China. J. Geochem. Explor..

[489] Guo H (2011). Hydrogeological and biogeochemical constrains of arsenic mobilization in shallow aquifers from the Hetao basin, Inner Mongolia. Environ. Pollut..

[490] Guo Q, Wang Y, Ma T, Ma R (2007). Geochemical processes controlling the elevated fluoride concentrations in groundwaters of the Taiyuan Basin, Northern China. J. Geochem. Explor..

[491] Gupta G, Erram VC, Kumar S (2012). Temporal geoelectric behaviour of dyke aquifers in northern Deccan Volcanic Province, India. J. Earth Syst. Sci..

[492] Gupta P, Sharma A, Joshi N (2015). Hydrochemical characterization of coastal groundwater in Porbandar Region, Gujarat, India. Int. J. Eng. Res. Gen. Sci..

[493] Gupta SK, Deshpande RD (2003). Origin of groundwater helium and temperature anomalies in the Cambay region of Gujarat, India. Chem. Geol..

[494] Gupte, P. R. Review of aquifer system of Deccan trap area, Gujarat state. Proceedings of the Fifth International Ground Water Congress (2012).

[495] Gutentag, E. D., Heimes, F. J., Krothe, N. C., Luckey, R. R. & Weeks, J. B. Geohydrology of the High Plains aquifer in parts of Colorado, Kansas, Nebraska, New Mexico, Oklahoma, South Dakota, Texas, and Wyoming. U.S. Geological Survey Professional Paper 1400-B. https://pubs.usgs.gov/pp/1400b/report.pdf (1984).

[496] Gxokwe S, Xu Y, Kanyerere T (2020). Scenarios analysis using water-sensitive urban design principles: a case study of the Cape Flats Aquifer in South Africa. Hydrogeol. J..

[497] Ha QK, Ngoc TDT, Le Vo P, Nguyen HQ, Dang DH (2022). Groundwater in Southern Vietnam: understanding geochemical processes to better preserve the critical water resource. Sci. Total Environ..

[498] Habermehl MA (2020). The evolving understanding of the Great Artesian Basin (Australia), from discovery to current hydrogeological interpretations. Hydrol. J..

[499] Hafezparast M (2021). Monitoring groundwater level changes of Mianrahan aquifer with GRACE satellite data. Iran. J. Irrig. Drain..

[500] Halford, K. J. & Barber, N. L. Analysis of ground-water flow in the Catahoula aquifer system in the vicinity of Laurel and Hattiesburg, Mississippi. U.S. Geological Survey Water-Resources Investigations Report 94-4219. https://pubs.usgs.gov/wri/1994/4219/report.pdf (1995).

[501] Hamid Reza, N. & Ferdows, S. N. Comparing vulnerability delineative of aquifer using drastic and fuzzy logic methods (case study: Gulgir Plain of Masjed Solieman, Iran). Proceedings of conference entitled “GIS Ostrava 2012 - Surface models for geosciences”. http://gisak.vsb.cz/GIS_Ostrava/GIS_Ova_2012/sbornik/papers/nassery.pdf (2012).

[502] Hamlin, H. Water resources of the Salinas Valley, California. U.S. Geological Survey Water-Supply and Irrigation Paper No. 89. https://pubs.usgs.gov/wsp/0089/report.pdf (1904).

[503] Hamlin, S. N. Ground-water quality in the Santa Rita, Buellton, and Los Olivos hydrologic subareas of the Santa Ynez River basin, Santa Barbara County, California. U.S. Geological Survey Water-Resources Investigations Report 84-4131. https://pubs.usgs.gov/wri/1984/4131/report.pdf (1985).

[504] Han DM, Song XF, Currell MJ, Yang JL, Xiao GQ (2014). Chemical and isotopic constraints on evolution of groundwater salinization in the coastal plain aquifer of Laizhou Bay, China. J. Hydrol..

[505] Han YL, Kuo MT, Fan KC, Chiang CJ, Lee YP (2006). Radon distribution in groundwater of Taiwan. Hydrol. J..

[506] Handman, E. H., Londquist, C. J. & Maurer, D. K. Ground-water resources of Honey Lake Valley, Lassen County, California, and Washoe County, Nevada. U.S. Geological Survey Water-Resources Investigations Report 90-4050. https://pubs.usgs.gov/wri/1990/4050/report.pdf (1990).

[507] Hanna, J. *Influence of Conceptual Model Uncertainty on Recharge Processes for the Wallal Aquifer System in the West Canning Basin, Western Australia*. MSc thesis, Univ. Western Australia (2014).

[508] Hanson RT (2015). Hydrologic framework of the Santa Clara Valley, California. Geosphere.

[509] Hanson, R. T. Aquifer-system compaction, Tucson Basin and Avra Valley, Arizona. U.S. Geological Survey Water-Resources Investigations Report 88-4172. https://pubs.usgs.gov/wri/1988/4172/report.pdf (1989).

[510] Hanson, R. T., Martin, P. & Koczot, K. M. Simulation of ground-water/surface-water flow in the Santa Clara-Calleguas ground-water basin, Ventura County, California. U.S. Geological Survey Water-Resources Investigations Report 2002-4136. https://pubs.usgs.gov/wri/wri024136/wrir024136.pdf (2002).

[511] Hanson, R. T., McLean, J. S. & Miller, R. S. Hydrogeologic framework and preliminary simulation of ground-water flow in the Mimbres Basin, Southwestern New Mexico. U.S. Geological Survey Water-Resources Investigations Report 94-4011. https://pubs.usgs.gov/wri/1994/4011/report.pdf (1994).

[512] Han-xue Q, Dong-yan L, Guan-qun L, Pi-hai N (1997). Saline water intrusion and its influence in the Laizhou area. Chin. J. Oceanol. Limnol..

[513] Hao L, Sun G, Liu Y, Qian H (2015). Integrated modeling of water supply and demand under management options and climate change scenarios in Chifeng City, China. J. Am. Water Resour. Assoc..

[514] Harden, S. L., Fine, J. M. & Spruill, T. B. Hydrogeology and ground-water quality of Brunswick County, North Carolina. U.S. Geological Survey Water-Resources Investigations Report 03-4051. https://pubs.usgs.gov/wri/2003/4051/wri20034051.pdf (2003).

[515] Harrill, J. R. & Prudic, D. E. Aquifer systems in the Great Basin region of Nevada, Utah, and adjacent states—summary report. U.S. Geological Survey Professional Paper 1409-A. https://pubs.usgs.gov/pp/1409a/report.pdf (1998).

[516] Harrington GA, Cook PG, Herczeg AL (2002). Spatial and temporal variability of ground water recharge in central Australia: a tracer approach. Groundwater.

[517] Harrington GA, Walker GR, Love AJ, Narayan KA (1999). A compartmental mixing-cell approach for the quantitative assessment of groundwater dynamics in the Otway Basin, South Australia. J. Hydrol..

[518] Harrington, G. A., Herczeg, A. L. & Cook, P. G. Groundwater sustainability and water quality in the Ti-Tree Basin, Central Australia. CSIRO report. http://hdl.handle.net/102.100.100/213199?index=1 (1999).

[519] Hart Jr, D. L. & Davis, R. E. Geohydrology of the Antlers aquifer (Cretaceous), southeastern Oklahoma. U.S. Geological Survey Circular 81. http://www.ogs.ou.edu/pubsscanned/Circulars/circular81mm.pdf (1981).

[520] Harte, P. T., Robinson Jr, G. R., Ayotte, J. D. & Flanagan, S. F. Framework for evaluating water quality of the New England crystalline rock aquifers. U.S. Geological Survey Open-File Report 2008-1282. https://pubs.usgs.gov/of/2008/1282/pdf/ofr2008-1282.pdf (2008).

[521] Hasan M, Shang Y, Akhter G, Jin W (2019). Application of VES and ERT for delineation of fresh-saline interface in alluvial aquifers of Lower Bari Doab, Pakistan. J. Appl. Geophys..

[522] Hashemi H, Berndtsson R, Kompani-Zare M (2012). Steady-state unconfined aquifer simulation of the Gareh-Bygone Plain, Iran. Open Hydrol. J..

[523] Hawley, J. W., Haase, C. S. & Lozinsky, R. P. An underground view of the Albuquerque Basin. Report No. CONF-9411293-TRN: IM9704%%261, 37–55. https://www.osti.gov/biblio/415630 (1995).

[524] Hawley, J. W. & Lozinsky, R. P. Hydrogeologic framework of the Mesjlla Basin in New Mexico and western Texas. New Mexico Bureau of Mines and Mineral Resources Open-File Report 323. https://geoinfo.nmt.edu/publications/openfile/downloads/300-399/323/ofr_323.pdf (1992).

[525] Hays, P. D., Knierim, K. J., Breaker, B., Westerman, D. A. & Clark, B. R. Hydrogeology and hydrologic conditions of the Ozark Plateaus aquifer system. U.S. Geological Survey Scientific Investigations Report 2016-5137. https://pubs.er.usgs.gov/publication/sir20165137 (2016).

[526] Hearne, G. A. et al. Colorado ground-water quality. U.S. Geological Survey Open-File Report 87-716. https://pubs.usgs.gov/of/1987/0716/report.pdf (1987).

[527] Heaton THE (1985). Isotopic and chemical aspects of nitrate in the groundwater of the Springbok Flats. Water SA.

[528] Heaton THE, Talma AS, Vogel JC (1986). Dissolved gas paleotemperatures and ^18^O variations derived from groundwater near Uitenhage, South Africa. Quat. Res..

[529] Hekmatnia H, Barzegari Banadkooki F, Moosavi V, Zare Chahouki A (2021). Evaluation of groundwater suitability for drinking, irrigation, and industrial purposes (case study: Yazd-Ardakan Aquifer, Yazd Province, Iran). ECOPERSIA.

[530] Helweg, O. J. & Labadie, J. W. A salinity management strategy for stream-aquifer systems. Colorado State University Hydrology Papers. https://mountainscholar.org/bitstream/handle/10217/61846/HydrologyPapers_n84.pdf?sequence=1 (1976).

[531] Hemmati F, Sajadi Z, Jamshidi AR (2014). Assessment of groundwater vulnerability in the Borazjan Aquifer of Bushehr, south of Iran, using GIS technique. Indian J. Fundam. Appl. Life Sci..

[532] Henry, R., Lindsay, K., Wolcott, B., Patten, S. & Baker, T. Walla Walla Basin Aquifer Recharge Strategic Plan. Walla Walla Basin Watershed Council report. https://wwbwc.org/index.php/recharge?highlight=WyJyZWNoYXJnZSIsInN0cmF0ZWdpYyIsInBsYW4iXQ== (2013).

[533] Herczeg AL, Dogramaci SS, Leaney FWJ (2001). Origin of dissolved salts in a large, semi-arid groundwater system: Murray Basin, Australia. Mar. Freshwater Res..

[534] Hernández F (2008). Pesticide residues and transformation products in groundwater from a Spanish agricultural region on the Mediterranean Coast. Int. J. Environ. Anal. Chem..

[535] Herrera-Barrientos J (2020). Determination of hydraulic transmissivity in coastal aquifer by optimal estimation of the Qe-T relationship using Kalman filter. Hidrobiológica.

[536] Herrera C (2021). Recharge and residence times of groundwater in hyper arid areas: the confined aquifer of Calama, Loa River Basin, Atacama Desert, Chile. Sci. Total Environ..

[537] Herrera E, Garfias J (2013). Characterizing a fractured aquifer in Mexico using geological attributes related to open-pit groundwater. Hydrol. J..

[538] Herrera MTA, Montenegro IF, Navar PR, Domínguez IRM, Vázquez RT (2001). Contenido de arsénico en el agua potable del valle del Guadiana, México. Tecnol. Cienc. Agua.

[539] Herrera, N. B. et al. Hydrogeologic framework and selected components of the groundwater budget for the upper Umatilla River Basin, Oregon. U.S. Geological Survey Scientific Investigations Report 2017-5020. https://pubs.usgs.gov/sir/2017/5020/sir20175020.pdf (2017).

[540] Herrera, N. B., Burns, E. R. & Conlon, T. D. Simulation of groundwater flow and the interaction of groundwater and surface water in the Willamette Basin and Central Willamette Subbasin, Oregon. U.S. Geological Survey Scientific Investigations Report 2014-5136. https://pubs.usgs.gov/sir/2014/5136/pdf/sir20145136.pdf (2014).

[541] Hidalgo MC, Cruz-Sanjulián J (2001). Groundwater composition, hydrochemical evolution and mass transfer in a regional detrital aquifer (Baza basin, southern Spain). Appl. Geochem..

[542] Hirata R, Foster S (2021). The Guarani Aquifer System–from regional reserves to local use. Q. J. Eng. Geol. Hydrogeol..

[543] Hirata R, Suhogusoff AV (2019). How much do we know about the groundwater quality and its impact on Brazilian society today?. Acta Limnol. Bras..

[544] Hoffman, S., Hunkeler, D. & Maurer, M. Approvisionnement en eau et assainissement des eaux usées durables en Suisse: défis et mesures possibles. PNR 61 – Synthèse thématique 3 dans le cadre du Programme national de recherche PNR 61. Gestion durable de l’eau. https://media.snf.ch/rWjOZoYQfS9iabW/nfp61_thematische_synthese_3_f.pdf (2014).

[545] Holland, M. *Hydrogeological Characterisation of Crystalline Basement Aquifers Within the Limpopo Province, South Africa*. PhD thesis, Univ. Pretoria (2011).

[546] Holmberg, M. J. Hydrogeologic characteristics and geospatial analysis of water-table changes in the alluvium of the lower Arkansas River Valley, southeastern Colorado, 2002, 2008, and 2015. U.S. Geological Survey Scientific Investigations Map 3378. https://pubs.usgs.gov/sim/3378/sim3378.pdf (2017).

[547] Holmes, W. F. & Thiros, S. A. Ground-water hydrology of Pahvant Valley and adjacent areas, Utah. U.S. Geological Survey Technical Publication No. 98. https://waterrights.utah.gov/docSys/v920/y920/y9200006.pdf (1990).

[548] Honarbakhsh A (2019). GIS-based assessment of groundwater quality for drinking purpose in northern part of Fars province, Marvdasht. J. Water Supply Res. Technol. AQUA.

[549] Hood, J. W. Characteristics of aquifers in the northern Uinta Basin area, Utah and Colorado. U.S. Geological Survey and Utah Department of Natural Resources, Division of Water Rights Technical Publication No. 53. https://waterrights.utah.gov/docSys/v920/w920/w920009f.pdf (1976).

[550] Hood, J. W. Hydrologic evaluation of Ashley Valley, northern Uinta Basin area, Utah. U.S. Geological Survey and Utah Department of Natural Resources, Division of Water Rights Technical Publication No. 54. https://pubs.usgs.gov/unnumbered/70043723/report.pdf (1977).

[551] Hosono T (2014). Different isotopic evolutionary trends of δ^34^S and δ^18^O compositions of dissolved sulfate in an anaerobic deltaic aquifer system. Appl. Geochem..

[552] Hosono T (2011). Multiple isotope (H, O, N, S and Sr) approach elucidates complex pollution causes in the shallow groundwaters of the Taipei urban area. J. Hydrol..

[553] Hosseini Poor H, Ghaioomeyan J, Ghasemi AR, Choopani S (2010). Investigating salt sources in Sarchahan aquifer in Hormozghan province using ion ratios. Watershed Eng. Manag..

[554] Hosseini M, Saremi A (2018). Assessment and estimating groundwater vulnerability to pollution using a modified DRASTIC and GODS models (case study: Malayer Plain of Iran). Civ. Eng. J..

[555] Hosseini SM, Parizi E, Ataie-Ashtiani B, Simmons CT (2019). Assessment of sustainable groundwater resources management using integrated environmental index: case studies across Iran. Sci. Total Environ..

[556] Hosseni MS, Jahanshahi R, Asadi N, Nasiri MA (2020). Qualitative study of groundwater resources in the Hassanabad-Dehchah, Northeast of Neyriz, Fars province. Hydrogeology.

[557] Hsieh, P. A. et al. Ground-water flow model for the Spokane valley-Rathdrum prairie aquifer, Spokane County, Washington, and Bonner and Kootenai Counties, Idaho. U.S. Geological Survey Scientific Investigations Report 2007-5044. https://pubs.usgs.gov/sir/2007/5044/pdf/sir20075044.pdf (2007).

[558] Hsu KC, Wang CH, Chen KC, Chen CT, Ma KW (2007). Climate-induced hydrological impacts on the groundwater system of the Pingtung Plain, Taiwan. Hydrol. J..

[559] Hsu SK (1998). Plan for a groundwater monitoring network in Taiwan. Hydrol. J..

[560] Huang Y (2012). Sources of groundwater pumpage in a layered aquifer system in the Upper Gulf Coastal Plain, USA. Hydrol. J..

[561] Huber E, Hendricks‐Franssen HJ, Kaiser HP, Stauffer F (2011). The role of prior model calibration on predictions with ensemble Kalman filter. Groundwater.

[562] Hudak PF (1999). Chloride and nitrate distributions in the Hickory aquifer, Central Texas, USA. Environ. Int..

[563] Huff, G. F. Simulation of ground-water flow in the basin-fill aquifer of the Tularosa Basin, south-central New Mexico, predevelopment through 2040. U.S. Geological Survey Scientific Investigations Report 2004-5197. https://pubs.usgs.gov/sir/2004/5197/pdf/sir20045197.pdf (2005).

[564] Hughes, J. L. Evaluation of ground-water quality in the Santa Maria Valley, California. U.S. Geological Survey, Water-Resources Investigations 76-128. https://pubs.usgs.gov/wri/1976/0128/report.pdf (1977).

[565] Hui Q, Li P (2011). Hydrochemical characteristics of groundwater in Yinchuan plain and their control factors. Asian J. Chem..

[566] Hunter, H. M. Nutrients and herbicides in groundwater flows to the Great Barrier Reef lagoon. Processes, fluxes and links to on-farm management. Report by individuals associated with the Australian Rivers Institute and Griffith University. https://www.qld.gov.au/__data/assets/pdf_file/0027/69066/rp51c-grounderwater-synthesis-great-barrier-reef.pdf (2012).

[567] Huntington, J. L., Minor, B., Bromley, M. & Morton, C. Reconnaissance investigation of phreatophyte vegetation vigor for selected hydrographic areas in Nevada. Division of Hydrologic Sciences, Desert Research Institute. http://www.conservationgateway.org/ConservationByGeography/NorthAmerica/UnitedStates/nevada/water/Documents/Final%20DRI-TNC%20spatiotemporal%20phreatophyte%20report_may31.pdf (2018).

[568] Hurlow HA (2014). Hydrogeologic studies and groundwater monitoring in Snake Valley and adjacent hydrographic areas, west-central Utah and east-central Nevada. Utah Geol. Surv. Bull..

[569] Hussain, S. D. et al. Surface water/groundwater relationship in Chaj Doab. Pakistan Institute of Nuclear Science & Technology Report No. PINSTECH/RIAD-122. https://inis.iaea.org/collection/NCLCollectionStore/_Public/22/031/22031202.pdf?r=1 (1990).

[570] Hussain Y (2017). Modelling the vulnerability of groundwater to contamination in an unconfined alluvial aquifer in Pakistan. Environ. Earth Sci..

[571] Hutchinson, R. D. & Klausing, R. L. Ground-water resources of Ramsey County, North Dakota. North Dakota State Water Commission Report. https://www.swc.nd.gov/info_edu/reports_and_publications/county_groundwater_studies/pdfs/Ramsey_Part_III.pdf (1980).

[572] Iepure S, Martinez-Hernandez V, Herrera S, Rasines-Ladero R, de Bustamante I (2013). Response of microcrustacean communities from the surface—groundwater interface to water contamination in urban river system of the Jarama basin (central Spain). Environ. Sci. Pollut. Res..

[573] Imes, J. L. & Emmett, L. F. Geohydrology of the Ozark Plateaus aquifer system in parts of Missouri, Arkansas, Oklahoma, and Kansas. U.S. Geological Survey Professional Paper 1414-D). https://pubs.usgs.gov/pp/1414d/report.pdf (1994).

[574] Instituto Mexicano de Tecnología del Agua (IMTA). Plan estatal hidrico 2040 de Chihuahua. Report (contract) number 060-207-E75-JCAS-PRODDER. https://www.nadb.org/uploads/files/1_plan_estatal_hdrico_de_chihuahua_2040_2018.pdf (2018).

[575] International Boundary and Water Commission (IBWC). Hydrogeological activities in the Conejos-Medanos/Mesilla Basin Aquifer, Chihuahua Phase I. International Boundary and Water Commission report. https://www.ibwc.gov/wp-content/uploads/2023/07/Final_report_English_Mesilla_ConejosMedanos_Study-2011.pdf (2011).

[576] International Hydrological Programme, Division of Water Sciences. Atlas of transboundary aquifers. Global maps, regional cooperation and local inventories. UNESCO Report SC-2009/WS/22. https://unesdoc.unesco.org/ark:/48223/pf0000192145 (2009).

[577] Izady A (2012). Application of “panel-data” modeling to predict groundwater levels in the Neishaboor Plain, Iran. Hydrol. J..

[578] Jabbari E, Fathi M, Moradi M (2020). Modeling groundwater quality and quantity to manage water resources in the Arak aquifer, Iran. Arab. J. Geosci..

[579] Jafari F, Javadi S, Golmohammadi G, Karimi N, Mohammadi K (2016). Numerical simulation of groundwater flow and aquifer-system compaction using simulation and InSAR technique: Saveh basin, Iran. Environ. Earth Sci..

[580] Jafari H, Shirafkan M, Bagheri R, Karami GH (2018). Assessing sustainability of the Bahabad aquifer, Central Iran. Appl. Ecol. Environ. Res..

[581] Jahanshahi A, Moghaddamnia A, Khosravi H (2015). Assessment of desertification density using IMDPA model (case study: Shahr-Babak plain, Kerman Province). J. Range Watershed Manag..

[582] Jaimes-Palomera LR (1989). Geoquimica isotopica del sistema hidrogeologico del valle de Cuerna Vaca, estado de Morelos, Mexico. Geofís. Int..

[583] Jain, A. K. & Nayak, K. M. Aquifer map and management plan, Porbandar District, Gujarat State. Central Ground Water Board report. http://cgwb.gov.in/cgwbpnm/publication-detail/1035 (2016).

[584] Jamshidzadeh Z, Mirbagheri SA (2011). Evaluation of groundwater quantity and quality in the Kashan Basin, Central Iran. Desalination.

[585] Janardhana MR, Khairy H (2019). Simulation of seawater intrusion in coastal aquifers: a case study on the Amol–Ghaemshahr coastal aquifer system, Northern Iran. Environ. Earth Sci..

[586] Jasrotia AS, Kumar A, Aasim M (2011). Morphometric analysis and hydrogeomorphology for delineating groundwater potential zones of Western Doon Valley, Uttarakhand, India. Int. J. Geomat. Geosci..

[587] Javadzadeh H, Ataie-Ashtiani B, Hosseini SM, Simmons CT (2020). Interaction of lake-groundwater levels using cross-correlation analysis: a case study of Lake Urmia Basin, Iran. Sci. Total Environ..

[588] Javanbakht M, Asadi V, Dabiri R (2020). Evaluation of hydrogeochemical characteristics and evolutionary process of groundwater in Jajarm Plain, Northeastern Iran. Environ. Water Eng..

[589] Javanmard Z, Asghari Moghaddam A (2016). Using statistical and hydrochemical models for qualitative analysis of groundwater resources (case study: Mehraban plain, in East Azerbaijan). Water Soil Sci..

[590] Javi ST, Malekmohammadi B, Mokhtari H (2014). Application of geographically weighted regression model to analysis of spatiotemporal varying relationships between groundwater quantity and land use changes (case study: Khanmirza Plain, Iran). Environ. Monit. Assess..

[591] Jawadi HA, Sagin J, Snow DD (2020). A detailed assessment of groundwater quality in the Kabul Basin, Afghanistan, and suitability for future development. Water.

[592] Jebreen H (2018). Recharge estimation in semi-arid karst catchments: Central West Bank, Palestine. Grundwasser.

[593] Jeddi TA (2023). Water resources status to global changes in the Taznakht plain, Draa basin, Morocco. Front. Sci. Eng..

[594] Jennings, S. P. Hydrogeology and groundwater assessment of the water distribution area of the town of Hodges water department, Franklin and Marion Counties, Alabama. Geological Survey of Alabama report. https://www.ogb.state.al.us/img/Groundwater/OFR/OFR1311.pdf (2013).

[595] Japan International Cooperation Agency (JICA) The study on the groundwater potential evaluation and management plan in the southeast Kalahari (Stampriet) Artesian Basin in the Republic of Namibia. https://openjicareport.jica.go.jp/pdf/11681699_01.PDF (2002).

[596] Jiménez-Martínez J, Aravena R, Candela L (2011). The role of leaky boreholes in the contamination of a regional confined aquifer. A case study: the Campo de Cartagena region, Spain. Water Air Soil Pollut..

[597] Jiráková H, Huneau F, Hrkal Z, Celle-Jeanton H, Le Coustumer P (2010). Carbon isotopes to constrain the origin and circulation pattern of groundwater in the north-western part of the Bohemian Cretaceous Basin (Czech Republic). Appl. Geochem..

[598] Jiráková H (2011). Geothermal assessment of the deep aquifers of the northwestern part of the Bohemian Cretaceous basin, Czech Republic. Geothermics.

[599] Jocson JMU, Jenson JW, Contractor DN (2002). Recharge and aquifer response: northern Guam lens aquifer, Guam, Mariana Islands. J. Hydrol..

[600] Johnson, G. C., Zimmerman, T. M., Lindsey, B. D. & Gross, E. L. Factors affecting groundwater quality in the Valley and Ridge aquifers, eastern United States, 1993–2002. U.S. Geological Survey Scientific Investigations Report 2011-5115. https://pubs.usgs.gov/sir/2011/5115/support/sir2011-5115.pdf (2011).

[601] Johnson, M. J. Ground-water conditions in the Eureka Area, Humboldt County, California. U.S. Geological Survey Water-Resources Investigations 78-127. https://pubs.usgs.gov/wri/1978/0127/report.pdf (1975).

[602] Jones, M. A. Geologic framework for the Puget Sound aquifer system, Washington and British Columbia. U.S. Geological Survey Professional Paper 1424-C. https://pubs.usgs.gov/pp/1424c/report.pdf (1999).

[603] Jordan, J. L. Aquifer parameter estimation from aquifer tests and specific-capacity data in Cedar Valley and the Cedar Pass Area, Utah County, Utah. Utah Geological Survey Special Study 146. https://ugspub.nr.utah.gov/publications/special_studies/ss-146/ss-146.pdf (2013).

[604] Jordan, J. L. et al. Characterization of the groundwater system in Ogden Valley, Weber County, Utah, with emphasis on groundwater–surface-water interaction and the groundwater budget. Utah Geological Survey Report Special Study 165. https://ugspub.nr.utah.gov/publications/special_studies/ss-165/ss-165.pdf (2019).

[605] Joshi SK (2021). Strongly heterogeneous patterns of groundwater depletion in northwestern India. J. Hydrol..

[606] Juran L (2017). Development and application of a multi-scalar, participant-driven water poverty index in post-tsunami India. Int. J. Water Resour. Dev..

[607] Kadlecová R, Olmer M (2011). Review of groundwater resources. Geol. Výzk. Mor. Slez..

[608] Kahle, S. C. et al. Hydrogeologic framework and hydrologic budget components of the Columbia Plateau Regional Aquifer System, Washington, Oregon, and Idaho. U.S. Geological Survey Scientific Investigations Report 2011-5124. https://pubs.usgs.gov/sir/2011/5124/pdf/sir20115124.pdf (2011).

[609] Kahle, S. C., Olsen, T. D. & Fasser, E. T. Hydrogeology of the Little Spokane River Basin, Spokane, Stevens, and Pend Oreille Counties, Washington. U.S. Geological Survey Scientific Investigations Report 2013-5124. https://pubs.usgs.gov/sir/2013/5124/pdf/sir20135124.pdf (2013).

[610] Kalantari N, Pawar NJ, Keshavarzi MR (2009). Water resource management in the intermountain Izeh Plain, Southwest of Iran. J. Mt. Sci..

[611] Kalantari N, Rangzan K, Thigale SS, Rahimi MH (2010). Site selection and cost-benefit analysis for artificial recharge in the Baghmalek plain, Khuzestan Province, southwest Iran. Hydrol. J..

[612] Kale VS, Bodas M, Chatterjee P, Pande K (2020). Emplacement history and evolution of the Deccan Volcanic Province, India. Episodes J. Int. Geosci..

[613] Kannan N, Joseph S, Sheela AM (2021). Characterization of groundwater in the shallow and deep aquifers of an agriculture-dominated tropical subhumid to semiarid region, India: a multivariate and GIS approach. J. Indian Soc. Remote Sens..

[614] Kansas Geological Survey. High Plains aquifer regions in Kansas. Kansas High Plains Aquifer Atlas. https://geokansas.ku.edu/kansas-high-plains-aquifer-atlas (2021).

[615] Kao YH, Liu CW, Wang PL, Liao CM (2015). Effect of sulfidogenesis cycling on the biogeochemical process in arsenic-enriched aquifers in the Lanyang Plain of Taiwan: evidence from a sulfur isotope study. J. Hydrol..

[616] Kapple, G. W., Mitten, H. T., Durbin, T. J. & Johnson, M. J. Analysis of the Carmel Valley alluvial ground-water basin, Monterey County, California. U.S. Geological Survey Water-Resources Investigations Report 83-4280. https://pubs.usgs.gov/wri/1983/4280/report.pdf (1984).

[617] Kar, G. et al. Integrated technologies to enhance productivity of seasonal deep waterlogged areas. Water Technology Centre for Eastern Region Research Bulletin 40. http://www.iiwm.res.in/pdf/Bulletin_40.pdf (2007).

[618] Kardan Moghaddam H, Dehghani M, Rahimzadeh Kivi Z, Kardan Moghaddam H, Hashemi SR (2017). Efficiency assessment of AHP and fuzzy logic methods in suitability mapping for artificial recharging (case study: Sarbisheh basin, Southern Khorasan, Iran). Water Harvest. Res..

[619] Kay, R. T. & Kraske, K. A. Ground-water levels in aquifers used for residential supply, Campton Township, Kane County, Illinois. U.S. Geological Survey Water-Resources Investigations Report 96-4009. https://pubs.usgs.gov/wri/1996/4009/report.pdf (1996).

[620] Kazmierczak J (2022). Groundwater arsenic content related to the sedimentology and stratigraphy of the Red River delta, Vietnam. Sci. Total Environ..

[621] Kelbe, B. E. & Germishuyse, T. Geohydrological studies of the primary coastal aquifer in Zululand. Water Research Commission Report No. K5/720/1/01. https://www.wrc.org.za/wp-content/uploads/mdocs/720-1-01.pdf (2001).

[622] Keller CK, Kamp GVD, Cherry JA (1986). Fracture permeability and groundwater flow in clayey till near Saskatoon, Saskatchewan. Can. Geotech. J..

[623] Kelley, V. A., Deeds, N. E., Fryar, D. G. & Nicot, J. P. Groundwater availability models for the Queen City and Sparta aquifers. Contract report to the Texas Water Development Board. https://www.twdb.texas.gov/groundwater/models/gam/qcsp/QCSP_Model_Report.pdf?d=29484 (2004).

[624] Kendy, E. Ground-water resources of the Gallatin Local Water Quality District, southwestern Montana. U.S. Geological Survey Fact Sheet 007-01. https://pubs.usgs.gov/fs/2001/0007/report.pdf (2001).

[625] Kennedy, J. R., Kahler, L. M. & Read, A. L. Aquifer storage change and storage properties, 2010–2017, in the Big Chino Subbasin, Yavapai County, Arizona. U.S. Geological Survey Scientific Investigations Report 2019-5060. https://pubs.usgs.gov/sir/2019/5060/sir20195060.pdf (2019).

[626] Kenny, S. Aquifers of the Capital Regional District. Capital Regional District report. https://www.env.gov.bc.ca/wsd/plan_protect_sustain/groundwater/aquifers/aquifers_crd/pdfs/aquif_crd.pdf, https://www.env.gov.bc.ca/wsd/plan_protect_sustain/groundwater/aquifers/aquifers_crd/pdfs/append_b.pdf (2004).

[627] Kent, R. & Belitz, K. Ground-water quality data in the Upper Santa Ana Watershed Study Unit, November 2006–March 2007: results from the California GAMA Program. U.S. Geological Survey Data Series 404. https://pubs.usgs.gov/ds/404/ds404.pdf (2009).

[628] Kernodle, J. M. Hydrogeology and steady-state simulation of ground-water flow in the San Juan Basin, New Mexico, Colorado, Arizona, and Utah. U.S. Geological Survey Water-Resources Investigations Report 95-4187. https://pubs.usgs.gov/wri/1995/4187/report.pdf (1996).

[629] Khair AM, Li C, Hu Q, Gao X, Wanga Y (2014). Fluoride and arsenic hydrogeochemistry of groundwater at Yuncheng Basin, Northern China. Geochem. Int..

[630] Khairy H, Janardhana MR (2013). Hydrogeochemical features of groundwater of semi-confined coastal aquifer in Amol–Ghaemshahr plain, Mazandaran Province, Northern Iran. Environ. Monit. Assess..

[631] Khalili Naft Chali A, Shahidi A (2021). Comparison of lazy algorithms and M5 model to estimate groundwater level (case study: Plain Neyshabur). J. Water Soil. Sci..

[632] Khashei-Siuki A, Sharifan H (2020). Comparison of AHP and FAHP methods in determining suitable areas for drinking water harvesting in Birjand aquifer. Iran. Groundw. Sustain. Dev..

[633] Khashei-Siuki A, Sarbazi M (2015). Evaluation of ANFIS, ANN, and geostatistical models to spatial distribution of groundwater quality (case study: Mashhad plain in Iran). Arab. J. Geosci..

[634] Khaska M (2013). Origin of groundwater salinity (current seawater vs. saline deep water) in a coastal karst aquifer based on Sr and Cl isotopes. Case study of the La Clape massif (southern France). Appl. Geochem..

[635] Khazai, E. & Riggi, M. G. Impact of urbanization on the Khash aquifer, an arid region of southeast Iran. International Association of Hydrological Sciences (IAHS) publication number 259, 211–218. https://iahs.info/uploads/dms/11462.211-217-259-Khazai.pdf (1999).

[636] Kheirandish M, Rahimi H, Kamaliardakani M, Salim R (2020). Obtaining the effect of sewage network on groundwater quality using MT3DMS code: case study on Bojnourd plain. Groundw. Sustain. Dev..

[637] Kheradpisheh Z, Talebi A, Rafati L, Ghaneian MT, Ehrampoush MH (2015). Groundwater quality assessment using artificial neural network: a case study of Bahabad plain, Yazd, Iran. Desert.

[638] Khodabakhshi N, Heidarzadeh N, Asadollahfardi G (2017). Vulnerability assessment of an aquifer using modified GIS‐based methods. J. Am. Water Works Assoc..

[639] Khosravi K, Bordbar M, Paryani S, Saco PM, Kazakis N (2021). New hybrid-based approach for improving the accuracy of coastal aquifer vulnerability assessment maps. Sci. Total Environ..

[640] Khosravi K, Nejad Roshan MH, Safari A (2017). Assessment of geostatistical methods for determining distribution patterns of groundwater resources in Sari-Neka coastal plain, northern Iran. Environ. Resour. Res..

[641] Kidd, R. E. & Lambeth, D. S. Hydrogeology and ground-water quality in the Black Belt area of west-central Alabama, and estimated water use for aquaculture, 1990. U.S. Geological Survey Water-Resources Investigations Report 94-4074. https://citeseerx.ist.psu.edu/viewdoc/download?doi=10.1.1.1015.2227&rep=rep1&type=pdf (1995).

[642] Kiran, D. A. & Ramaraju, H. K. The study of sea water intrusion using chemical indicators in the Coastal Region of Mangaluru. 52nd Annual Convention of Indian Water Works Association (IWWA) (2020).

[643] Knechtel, M. M. & Lohr, E. W. Geology and ground-water resources of the Valley of Gila River and San Simon Creek, Graham County, Arizona; with a section on the chemical character of the ground water. U.S. Geological Survey Water-Supply Paper 796-F. https://pubs.usgs.gov/wsp/0796f/report.pdf (1938).

[644] Knight, J. E., Gungle, B. & Kennedy, J. R. Assessing potential groundwater-level declines from future withdrawals in the Hualapai Valley, northwestern Arizona. U.S. Geological Survey Scientific Investigations Report 2021-5077. https://pubs.usgs.gov/sir/2021/5077/sir20215077.pdf (2021).

[645] Knochenmus, L. A. Regional evaluation of the hydrogeologic framework, hydraulic properties, and chemical characteristics of the intermediate aquifer system underlying southern west-central Florida. U.S. Geological Survey Scientific Investigations Report 2006-5013. https://pubs.usgs.gov/sir/2006/5013/pdf/2006-5013.pdf (2006).

[646] Koch U, Heinicke J (2007). Hydrological influences on long-term gas flow trends at locations in the Vogtland/NW Bohemian seismic region (German-Czech border). Ann. Geophys..

[647] Koci, J. Deep drainage potential of surface irrigated sugarcane in the Arriga Flats of far north Queensland. Report on improving application efficiency of furrow irrigated sugar cane using SIRMOD and implications for rising saline groundwater in the Arriga Basin of Far North Queensland funded by National Program for Sustainable Irrigation. http://27.111.91.222/xmlui/bitstream/handle/1/4125/JCU1101%20Final%20Report.pdf?sequence=1&isAllowed=y (2011).

[648] Kováč M, Sliva L, Sopkova B, Hlavata J, Škulová A (2008). Serravallian sequence stratigraphy of the northern Vienna Basin: high frequency cycles in the Sarmatian sedimentary record. Geol. Carpath..

[649] Kralik M (2014). Using ^18^O/^2^H, ^3^H/^3^He, ^85^Kr and CFCs to determine mean residence times and water origin in the Grazer and Leibnitzer Feld groundwater bodies (Austria). Appl. Geochem..

[650] Krauze P (2017). Microbiological and geochemical survey of CO_2_-dominated mofette and mineral waters of the Cheb Basin, Czech Republic. Front. Microbiol..

[651] Kulkarni H, Deolankar SB, Lalwani A, Joseph B, Pawar S (2000). Hydrogeological framework of the Deccan basalt groundwater systems, west-central India. Hydrol. J..

[652] Kumar A, Singh CK (2020). Arsenic enrichment in groundwater and associated health risk in Bari doab region of Indus basin, Punjab, India. Environ. Pollut..

[653] Kumar MD, Ghosh S, Patel A, Singh OP, Ravindranath R (2006). Rainwater harvesting in India: some critical issues for basin planning and research. Land Use Water Resour. Res..

[654] Kumar US, Sharma S, Navada SV, Deodhar AS (2009). Environmental isotopes investigation on recharge processes and hydrodynamics of the coastal sedimentary aquifers of Tiruvadanai, Tamilnadu State, India. J. Hydrol..

[655] Kumar VS, Amarender B, Dhakate R, Sankaran S, Kumar KR (2016). Assessment of groundwater quality for drinking and irrigation use in shallow hard rock aquifer of Pudunagaram, Palakkad District Kerala. Appl. Water Sci..

[656] Kuniansky, E. L., Bellino, J. C. & Dixon, J. Transmissivity of the Upper Floridan aquifer in Florida and parts of Georgia, South Carolina, and Alabama. U.S. Geological Survey Scientific Investigations Map 3204. https://pubs.usgs.gov/sim/3204/pdf/USGS_SIM-3204_Kuniansky_Web.pdf (2012).

[657] Kunkle, F. & Upson, J. E. Geology and ground water in Napa and Sonoma Valleys, Napa and Sonoma Counties, California. U.S. Geological Survey Water-Supply Paper 1495. https://pubs.usgs.gov/wsp/1495/report.pdf (1960).

[658] La Gal La Salle C, Marlin C, Savoye S, Fontes JC (1996). Geochemistry and ^14^C dating of groundwaters from Jurassic aquifers of North Aquitaine Basin (France). Appl. Geochem..

[659] La Rocque, G. A., Upson, J. E. & Worts Jr, G. F. Wells and water levels in principal ground-water basins in Santa Barbara County, California. U.S. Geological Survey Water-Supply Paper 1068. https://pubs.usgs.gov/wsp/1068/report.pdf (1950).

[660] Labus K, Bujok P, Klempa M, Porzer M, Matýsek D (2016). Preliminary geochemical modeling of water–rock–gas interactions controlling CO_2_ storage in the Badenian Aquifer within Czech Part of Vienna Basin. Environ. Earth Sci..

[661] LaFave, J. I. Potentiometric surface map of the southern part of the Flathead Lake area, Lake, Missoula, Sanders Counties, Montana. Montana Ground-Water Assessment Atlas No. 2, Part B, Map 4. Montana Bureau of Mines and Geology, A Department of Montana Tech of The University of Montana (2004).

[662] LaFave, J. I., Smith, L. N. & Patton, T. W. Ground-water resources of the Flathead Lake area: Flathead, Lake, Missoula, and Sanders Counties, Montana. Part A – descriptive overview and water-quality data. Montana Bureau of Mines and Geology. Montana Ground-Water Assessment Atlas 2. http://mbmg.mtech.edu/pdf/GWA_2.pdf (2004).

[663] LaFave, J. Quality and age of water in the Madison Aquifer, Cascade County, Montana. Montana American Water Resources Association Conference, Session 2. https://www.montanaawra.org/wp/ppts/2011/session2/5_LaFave_John_i.pdf (2011).

[664] Lalehzari R, Tabatabaei SH (2015). Simulating the impact of subsurface dam construction on the change of nitrate distribution. Environ. Earth Sci..

[665] Lambán, L. J. & Aragón, R. in *Groundwater and Saline Intrusion. Selected Papers from the 18th Salt Water Intrusion Meeting* (ed. Araguás, L.) 551–563 (2004).

[666] Lambert, P. M., Marston, T., Kimball, B. A. & Stolp, B. J. Assessment of groundwater/surface-water interaction and simulation of potential streamflow depletion induced by groundwater withdrawal, Uinta River near Roosevelt, Utah. U.S. Geological Survey Scientific Investigations Report 2011-5044. https://pubs.usgs.gov/sir/2011/5044/pdf/sir20115044.pdf (2011).

[667] LaMoreaux, P. E. et al. Reconnaissance of the geology and ground water of the Khorat Plateau, Thailand. U.S. Geological Survey Water-Supply Paper 1429. https://pubs.usgs.gov/wsp/1429/report.pdf (1958).

[668] Lancaster PJ, Dey S, Storey CD, Mitra A, Bhunia RK (2015). Contrasting crustal evolution processes in the Dharwar craton: insights from detrital zircon U–Pb and Hf isotopes. Gondwana Res..

[669] Land and Water Commissioner. Groundwater: Gunnedah Basin NSW, what water information can tell us. Presentation. https://www.industry.nsw.gov.au/__data/assets/pdf_file/0020/104852/gunnedah-groundwater-presentation.pdf (2019).

[670] Land, L. Overview of fresh and brackish water quality in New Mexico. Open-file report 583. https://geoinfo.nmt.edu/resources/water/amp/brochures/BWA/Estancia_Basin_FBWQNM.pdf (2016).

[671] Land, L. Overview of fresh and brackish water quality in New Mexico. Project Summary Report, New Mexico Bureau of Geology and Mineral Resources, Open-file Report 583. https://geoinfo.nmt.edu/resources/water/amp/brochures/BWA/Raton_Las_Vegas_Basin_FBWQNM.pdf (2016).

[672] Land, L. & Newton, B. T. Seasonal and long-term variations in hydraulic head in a karstic aquifer: Roswell artesian basin, New Mexico. New Mexico Bureau of Geology and Mineral Resources Open-File Report 503. https://geoinfo.nmt.edu/publications/openfile/downloads/500-599/503/ofr_503.pdf (2007).

[673] Land, M. et al. Ground-water quality of coastal aquifer systems in the West Coast Basin, Los Angeles County, California, 1999–2002. U.S. Geological Survey Scientific Investigations Report 2004-5067. https://pubs.usgs.gov/sir/2004/5067/sir2004-5067.pdf (2004).

[674] Laney, R. L. & Hahn, M. E. Hydrogeology of the eastern part of the Salt River Valley area, Maricopa and Pinal Counties, Arizona. U.S. Geological Survey Water-Resources Investigations Report 86-4147. https://pubs.er.usgs.gov/publication/wri864147 (1986).

[675] Langenheim, V. E., Duval, J. S., Wirt, L. & DeWitt, E. Preliminary report on geophysics of the Verde River headwaters region, Arizona. U.S. Geological Survey Open-File Report 00-403. https://pubs.usgs.gov/of/2000/0403/pdf/of00-403p.pdf (2000).

[676] Langeroudi SR, Turkamani SM (2016). Water quality assessment and hydrochemical characteristics of groundwater in Abhar Plain, Zanjan, Iran. J. Tethys.

[677] Langrudi MAO, Siuki AK, Javadi S, Hashemi SR (2016). Evaluation of vulnerability of aquifers by improved fuzzy drastic method: case study: Aastane Kochesfahan plain in Iran. Ain Shams Eng. J..

[678] Larque P (1981). La sédimentation et les paléoaltérations tertiaires de la plaine du Forez: nouvelles données. Essai de corrélations stratigraphiques. Sci. Géol. Bull. Mém..

[679] LaVanchy, G. T., Adamson, J. K. & Kerwin, M. W. in *Global Groundwater: Source, Scarcity, Sustainability, Security, and Solutions* (eds Mukherjee, A. et al.) 439–449 (Elsevier, 2021).

[680] Ledesma-Ruiz R, Pastén-Zapata E, Parra R, Harter T, Mahlknecht J (2015). Investigation of the geochemical evolution of groundwater under agricultural land: a case study in northeastern Mexico. J. Hydrol..

[681] Lee, S. *Investigating the Origin and Dynamics of Salinity in a Confined Aquifer System in Southeast Australia (Western Port Basin)*. BSc Thesis, RMIT Univ. (2015).

[682] Lee S, Currell M, Cendón DI (2016). Marine water from mid-Holocene sea level highstand trapped in a coastal aquifer: evidence from groundwater isotopes, and environmental significance. Sci. Total Environ..

[683] Lee, W. T. Water resources of Beaver Valley, Utah. U.S. Geological Survey Water-Supply Paper 217. https://pubs.usgs.gov/wsp/0217/report.pdf (1908).

[684] Leighton MM, Ekblaw GE, Horberg L (1948). Physiographic divisions of Illinois. J. Geol..

[685] Leonard, G. J., Watts, K. R. Leonard, G. J. & Watts, K. R. Hydrogeology and simulated effects of ground-water development on an unconfined aquifer in the Closed Basin Division, San Luis Valley, Colorado. U.S. Geological Survey Water-Resources Investigations Report 87-4284. https://pubs.usgs.gov/wri/1987/4284/report.pdf (1989).

[686] Leonard RB, Signor DC, Jorgensen DG, Helgesen JO (1983). Geohydrology and hydrochemistry of the Dakota Aquifer, central United States. J. Am. Water Resour. Assoc..

[687] Leonhard, L. Burton, K. & Milligan, N. in *Groundwater in the Coastal Zones of Asia-Pacific* (ed. Wetzelhuetter, C.) 359–378 (Springer, 2013).

[688] Leopold, R. Groundwater resource evaluation of the lower Dakota Aquifer in northwest Iowa. Iowa Geological and Water Survey Water Resources Investigation Report No. 1B. https://publications.iowa.gov/26582/1/WRI-1b.pdf (2008).

[689] Levi E, Goldman M, Tibor G, Herut B (2018). Delineation of subsea freshwater extension by marine geoelectromagnetic soundings (SE Mediterranean Sea). Water Resour. Manag..

[690] Lewis C, Ray D, Chiu KK (2007). Primary geologic sources of arsenic in the Chianan Plain (Blackfoot disease area) and the Lanyang Plain of Taiwan. Int. Geol. Rev..

[691] Li C, Gao X, Wang Y (2015). Hydrogeochemistry of high-fluoride groundwater at Yuncheng Basin, northern China. Sci. Total Environ..

[692] Li H, Zhan R, Lu Y, Zhou B, Wu J (2021). Spatiotemporal variation and periodic evolution characteristics of groundwater in the Xining area of China, eastern Qinghai–Tibet Plateau. Environ. Earth Sci..

[693] Li J, Wang Y, Xie X, Su C (2012). Hierarchical cluster analysis of arsenic and fluoride enrichments in groundwater from the Datong basin, Northern China. J. Geochem. Explor..

[694] Li XD, Liu CQ, Harue M, Li SL, Liu XL (2010). The use of environmental isotopic (C, Sr, S) and hydrochemical tracers to characterize anthropogenic effects on karst groundwater quality: a case study of the Shuicheng Basin, SW China. Appl. Geochem..

[695] Li Y, Wang D, Liu Y, Zheng Q, Sun G (2017). A predictive risk model of groundwater arsenic contamination in China applied to the Huai River Basin, with a focus on the region’s cluster of elevated cancer mortalities. Appl. Geochem..

[696] Liang CP, Jang CS, Liang CW, Chen JS (2016). Groundwater vulnerability assessment of the Pingtung Plain in Southern Taiwan. Int. J. Environ. Res. Public Health.

[697] Liang CP, Sun CC, Suk H, Wang SW, Chen JS (2021). A machine learning approach for spatial mapping of the health risk associated with arsenic-contaminated groundwater in Taiwan’s Lanyang Plain. Int. J. Environ. Res. Public Health.

[698] Liang K (2018). Investigation of the Yellow River buried fault in the Wuhai basin, northwestern Ordos Block, China, using deep/shallow seismic reflection and drilling techniques. J. Asian Earth Sci..

[699] Lindholm, G. F. Summary of the Snake River Plain regional aquifer-system analysis in Idaho and eastern Oregon. U.S. Geological Survey Professional Paper 1408-A. https://pubs.usgs.gov/pp/1408a/report.pdf (1996).

[700] Lithuanian Geological Survey and Latvian Environment, Geology and Meteorology Centre. Cross-border groundwater body characterization and status assessment. B-Solutions initiative report (2019).

[701] Liu J. & Zheng C. in *Integrated Groundwater Management* (eds Jakeman A. J., Barreteau O., Hunt R. J., Rinaudo J. D. & Ross A.) 455–475 (Springer, 2016).

[702] Liu CH, Pan YW, Liao JJ, Huang CT, Ouyang S (2004). Characterization of land subsidence in the Choshui River alluvial fan, Taiwan. Environ. Geol..

[703] Liu CW, Chen JF (1996). The simulation of geochemical reactions in the Heng-Chun limestone formation, Taiwan. Appl. Math. Model..

[704] Liu CW, Chou YL, Lin ST, Lin GJ, Jang CS (2010). Management of high groundwater level aquifer in the Taipei Basin. Water Resour. Manag..

[705] Liu J (2018). Study on the dynamic characteristics of groundwater in the valley plain of Lhasa City. Environ. Earth Sci..

[706] Liu S, Tang Z, Gao M, Hou G (2017). Evolutionary process of saline-water intrusion in Holocene and Late Pleistocene groundwater in southern Laizhou Bay. Sci. Total Environ..

[707] Llamas MR, Simpson ES, Alfaro PEM (1982). Ground‐water age distribution in Madrid Basin, Spain. Groundwater.

[708] Llopis-González A, Sánchez AL, Requena PM, Suárez-Varela MM (2014). Assessment of the microbiological quality of groundwater in three regions of the Valencian Community (Spain). Int. J. Environ. Res. Public Health.

[709] Lloyd JW, Jacobson G (1987). The hydrogeology of the Amadeus Basin, central Australia. J. Hydrol..

[710] Loeltz, O. J. & Eakin, T. E. Geology and water resources of Smith Valley, Lyon and Douglas Counties, Nevada. U.S. Geological Survey Water-Supply Paper 1228. https://pubs.usgs.gov/wsp/1228/report.pdf (1953).

[711] Londquist, C. J. & Livingston, R. K. Water-resources appraisal of the Wet Mountain Valley, in parts of Custer and Fremont Counties, Colorado. U.S. Geological Survey Water-Resources Investigations 78-1. from https://pubs.usgs.gov/wri/1978/0001/report.pdf (1978).

[712] Long, A. J., Thamke, J. N., Davis, K. W. & Bartos, T. T. Groundwater availability of the Williston Basin, United States and Canada. U.S. Geological Survey Professional Paper 1841. from https://pubs.usgs.gov/pp/1841/pp1841.pdf (2018).

[713] Lopes, T. J. Hydrologic evaluation of the Jungo area, southern Desert Valley, Nevada. U.S. Geological Survey Open-File Report 2010-1009. https://pubs.usgs.gov/of/2010/1009/pdf/ofr20101009.pdf (2010).

[714] Lopes, T. J. & Evetts. D. M. Ground-water pumpage and artificial recharge estimates for calendar year 2000 and average annual natural recharge and interbasin flow by hydrographic area, Nevada. U.S. Geological Survey Water-Resources Investigations Report 2004-5239. https://pubs.usgs.gov/sir/2004/5239/sir2004-5239.pdf (2005).

[715] López-Geta, J. A., Del Barrio Beato, V. & Vega Martin, L. Explotación de las Aguas Subterráneas En El Duero: Los Retos De La Cuenca. Conference paper. https://www.researchgate.net/profile/Leticia-Vega-Martin/publication/276938341_EXPLOTACION_DE_LAS_AGUAS_SUBTERRANEAS_EN_EL_DUERO_LOS_RETOS_DE_LA_CUENCA/links/555c6b3a08ae6aea08175a6e/EXPLOTACION-DE-LAS-AGUAS-SUBTERRANEAS-EN-EL-DUERO-LOS-RETOS-DE-LA-CUENCA.pdf (2006).

[716] Loris, P. *Hydrogeology of the Waipara Alluvial Basin*. MSc thesis, Univ. Canterbury (2000).

[717] Louisiana Department of Environmental Quality. Carrizo-Wilcox aquifer summary report 2007. Aquifer Sampling and Assessment Program (ASSET) Program. https://deq.louisiana.gov/assets/docs/Water/Triennial_reports/AquiferSummaries_2007-2009/02Carrizo-WilcoxAquiferSummary09.pdf (2007).

[718] Louisiana Department of Transportation and Development. Water Resources of Lafayette Parish. U.S. Geological Survey Fact Sheet 2010-3048. https://pubs.usgs.gov/fs/2010/3048/pdf/FS2010-3048.pdf (2011).

[719] Louisiana Department of Transportation and Development. Water Resources of Orleans Parish, Louisiana. U.S. Geological Survey Fact Sheet 2014-3017. https://pubs.usgs.gov/fs/2014/3017/pdf/fs2014-3017.pdf (2014).

[720] Louisiana Department of Transportation and Development. Water Resources of St. John the Baptist Parish, Louisiana. U.S. Geological Survey Fact Sheet 2014-3102. https://pubs.usgs.gov/fs/2014/3102/pdf/fs2014-3102.pdf (2014).

[721] Love AJ (1994). Groundwater residence time and palaeohydrology in the Otway Basin, South Australia: ^2^H, ^18^O and ^14^C data. J. Hydrol..

[722] Lu HY, Peng TR, Liu TK, Wang CH, Huang CC (2006). Study of stable isotopes for highly deformed aquifers in the Hsinchu-Miaoli area, Taiwan. Environ. Geol..

[723] Lu KL, Liu CW, Jang CS (2012). Using multivariate statistical methods to assess the groundwater quality in an arsenic-contaminated area of Southwestern Taiwan. Environ. Monit. Assess..

[724] Lü X, Han Z, Li H, Zheng Y, Liu J (2022). Influence of urbanization on groundwater chemistry at Lanzhou Valley basin in China. Minerals.

[725] Luckey, R. L. & Becker, M. F. Hydrogeology, water use, and simulation of flow in the High Plains aquifer in northwestern Oklahoma, southeastern Colorado, southwestern Kansas, northeastern New Mexico, and northwestern Texas. U.S. Geological Survey Water-Resources Investigations Report 99-4104. https://pubs.usgs.gov/wri/wri994104/pdf/wri994104.pdf (2003).

[726] Lund JR (1988). Regional water supply development in south Sweden. J. Urban Plan. Dev..

[727] Luo CY, Shen SL, Han J, Ye GL, Horpibulsuk S (2015). Hydrogeochemical environment of aquifer groundwater in Shanghai and potential hazards to underground infrastructures. Nat. Hazards.

[728] Lyke, W. L. & Coble, R. W. Regional study of the Castle Hayne Aquifer of eastern North Carolina. U.S. Geological Survey Open-File Report 87-571. https://pubs.usgs.gov/of/1987/0571/report.pdf (1987).

[729] Maathuis, H. The quality of natural groundwaters in Saskatchewan. Saskatchewan Research Council Publication No. 12012-1E08. https://www.wsask.ca/PageFiles/2978/The%20Quality%20of%20Natural%20Groundwaters%20in%20Saskatchewan,%20January%202008,%20Maathuis,%20H.,%20SRC%20pub.%20No.%2012012-1E08.pdf (2008).

[730] Maathuis, H. & Simpson, M. Groundwater resources of the prelate (72K) area, Saskatchewan. Saskatchewan Research Council Publication No. 11975-1E07. https://www.wsask.ca/wp-content/uploads/2021/08/Groundwater-Resources-Report-Prelate.pdf (2007).

[731] Maathuis, H. & Simpson, M. Hydrogeology of the Ribstone Creek Aquiferin Western Canada. Saskatchewan Research Council Publication No. 11500-1E02. https://www.wsask.ca/PageFiles/2978/Hydrogeology%20of%20the%20Ribstone%20Creek%20Aquifer%20in%20Western%20Canada,%20Maathuis,%20H.,%20and%20Simpson,%20M.,%202002,%20SRC%20Pub%20No%2011500-1E02.pdf (2002).

[732] MacDonald AM, Allen DJ (2001). Aquifer properties of the Chalk of England. Q. J. Eng. Geol. Hydrogeol..

[733] Macfarlane, P. A., Doveton, J. H. & Whittemore, D. O. User’s guide to the Dakota Aquifer in Kansas. Kansas Geological Survey, Technical Series 2. http://www.kgs.ku.edu/Publications/Bulletins/TS2/index.html (1998).

[734] Macfarlane, P. A. Revisions to the nomenclature for Kansas Aquifers. Kansas Geological Survey report. https://journals.ku.edu/cres/article/download/11815/11159 (2000).

[735] Machiwal D, Islam A, Kamble T (2019). Trends and probabilistic stability index for evaluating groundwater quality: the case of quaternary alluvial and quartzite aquifer system of India. J. Environ. Manag..

[736] Machkova, M., Velikov, B., Machkova, M., Dimitrov, D. & Neytchev, N. in *Natural Groundwater Quality* (eds Edmunds, W. M. & Shand, P.) 391–403 (Wiley, 2008).

[737] Mack TJ, Chornack MP, Taher MR (2013). Groundwater-level trends and implications for sustainable water use in the Kabul Basin, Afghanistan. Environ. Syst. Decis..

[738] Mack, T. J. Assessment of ground-water resources in the Seacoast region of New Hampshire. U.S. Geological Survey Scientific Investigations Report 2008-5222. https://pubs.usgs.gov/sir/2008/5222/pdf/sir2008-5222.pdf (2008).

[739] Maclear LGA (2001). The hydrogeology of the Uitenhage Artesian Basin with reference to the Table Mountain Group Aquifer. Water SA.

[740] Macphail, M. Hill, B., Carpenter, R. & McKellar, J. Cenozoic oil-shale deposits in southeastern-central Queensland: palynostratigraphic age determinations and correlations for the Biloela Formation (Biloela Basin) in GSQ Monto 5. Queensland Geological Record 2014/01. https://geoscience.data.qld.gov.au/report/cr089721 (2014).

[741] Madani K, Mariño MA (2009). System dynamics analysis for managing Iran’s Zayandeh-Rud river basin. Water Resour. Manag..

[742] Madison, J. P., LaFave, J. I., Patton, T. W., Smith, L. N. & Olson, J. N. Groundwater resources of the Middle Yellowstone River area: Treasure and Yellowstone counties, Montana Part A*—descriptive overview and water-quality data. Montana Bureau of Mines and Geology, Montana Ground-Water Assessment Atlas 3-A. http://mbmg.mtech.edu/pdf-publications/gwaa_3.pdf (2014).

[743] Magarey, P. & Deane, D. Willochra Basin Groundwater Monitoring Status Report 2005. Department of Water, Land and Biodiversity Conservation Report No. 2005/39. https://www.waterconnect.sa.gov.au/Content/Publications/DEW/ki_dwlbc_2005_39.pdf (2005).

[744] Magesh NS, Chandrasekar N, Soundranayagam JP (2012). Delineation of groundwater potential zones in Theni district, Tamil Nadu, using remote sensing, GIS and MIF techniques. Geosci. Front..

[745] Mahlknecht J (2023). Hydrochemical controls on arsenic contamination and its health risks in the Comarca Lagunera region (Mexico): implications of the scientific evidence for public health policy. Sci. Total Environ..

[746] Mahlknecht J, Merchán D, Rosner M, Meixner A, Ledesma-Ruiz R (2017). Assessing seawater intrusion in an arid coastal aquifer under high anthropogenic influence using major constituents, Sr and B isotopes in groundwater. Sci. Total Environ..

[747] Mahmoodlu M, Heshmatpour A, Jandaghi N, Zare A, Mehrabi H (2018). Hydrogeochemical assessment of groundwater quality: Seyedan-Farooq aquifer, Fars Province. Iran. J. Ecohydrol..

[748] Mahmoudzadeh E, Rezaian S, Ahmadi A (2013). Assessment of Meymeh Plain aquifer vulnerability in Esfahan using comparative method AVI, GODS, DRASTIC. J. Environ. Stud..

[749] Majola K, Xu Y, Kanyerere T (2022). Review: Assessment of climate change impacts on groundwater-dependent ecosystems in transboundary aquifer settings with reference to the Tuli-Karoo transboundary aquifer. Ecohydrol. Hydrobiol..

[750] Malakootian M, Nozari M (2020). GIS-based DRASTIC and composite DRASTIC indices for assessing groundwater vulnerability in the Baghin aquifer, Kerman, Iran. Nat. Hazards Earth Syst. Sci..

[751] Malekmohammadi B, Jahanishakib F (2017). Vulnerability assessment of wetland landscape ecosystem services using driver-pressure-state-impact-response (DPSIR) model. Ecol. Indic..

[752] Malenda, H. F. & Penn, C. A. Groundwater levels in the Denver Basin bedrock aquifers of Douglas County, Colorado, 2011–19. U.S. Geological Survey Scientific Investigations Report 2020–5076. https://pubs.usgs.gov/sir/2020/5076/sir20205076.pdf (2020).

[753] Mali N, Koroša A, Urbanc J (2023). Prevalence of pesticides in Krško-Brežice polje aquifer. Geologija.

[754] Mallory, M. J. Hydrogeology of the Southeastern Coastal Plain aquifer system in parts of eastern Mississippi and western Alabama. U.S. Geological Survey Professional Paper 1410-G. https://pubs.usgs.gov/pp/1410g/report.pdf (1993).

[755] Manjusree TM, Joseph S, Thomas J (2009). Hydrogeochemistry and groundwater quality in the coastal sandy clay aquifers of Alappuzha district, Kerala. J. Geol. Soc. India.

[756] Manning, A. H. Ground-water temperature, noble gas, and carbon isotope data from the Española Basin, New Mexico. U.S. Geological Survey Scientific Investigations Report 2008–5200. https://pubs.usgs.gov/sir/2008/5200/pdf/SIR08-5200.pdf (2009).

[757] Manning AH, Solomon DK (2005). An integrated environmental tracer approach to characterizing groundwater circulation in a mountain block. Water Resour. Res..

[758] Manz, R. P. Groundwater flow modeling of the Ojai basin using the USGS 3 dimensional MODFLOW model. MSc thesis, California State Univ. (1988).

[759] Marchildon, M. & Kassenaar, D. Analyzing low impact development strategies using continuous fully distributed coupled groundwater and surface water models. *J. Water Manag. Model.,* R246-17. 10.14796/JWMM.R246-17 (2013).

[760] Maroufpoor S, Fakheri-Fard A, Shiri J (2019). Study of the spatial distribution of groundwater quality using soft computing and geostatistical models. ISH J. Hydraul. Eng..

[761] Marques EA (2020). Analysis of groundwater and river stage fluctuations and their relationship with water use and climate variation effects on Alto Grande watershed, Northeastern Brazil. J. S. Am. Earth Sci..

[762] Marques, R. M. *Bacia do Parnaíba: Estado Atual do Conhecimento e Possibilidades Para a Produção de Gás Natural*. Thesis, Universidade Federal do Pará (2011).

[763] Marshall JS (2007). The geomorphology and physiographic provinces of Central America. Central Am. Geol. Resour. Hazards.

[764] Marshall, S. K., Fontaine, K., Kilgour, P. L. & Lewis, S. J. Regional hydrogeological characterisation of the Maryborough Basin, Queensland. Technical report for the National Collaboration Framework Regional Hydrogeology Project. Geoscience Australia Record 2015/14. https://wetlandinfo.des.qld.gov.au/resources/static/pdf/ecology/catchment-stories/gss/marshall-2015.pdf (2015).

[765] Marston, T. M. Water resources of Parowan Valley, Iron County, Utah. U.S. Geological Survey Scientific Investigations Report 2017-5033. 10.3133/sir20175033 (2017).

[766] Martin, P. Development and calibration of a two-dimensional digital model for the analysis of the ground-water flow system in the San Antonio Creek Valley, Santa Barbara County, California. U.S. Geological Survey Water-Resources Investigations Report 84-4340. https://pubs.usgs.gov/wri/1984/4340/report.pdf (1984).

[767] Martínez R (2009). The EU GeoCapacity project—saline aquifers storage capacity in group south countries. Energy Procedia.

[768] Martínez-Bastida JJ, Araúzo M, Valladolid M (2007). Caracterización hidroquímica de las aguas superficiales y subterráneas en la cuenca del Oja-Tirón. Procesos de contaminación. Limnetica.

[769] Martínez-Granados D, Calatrava J (2014). The role of desalinisation to address aquifer overdraft in SE Spain. J. Environ. Manag..

[770] Martínez-Retama S, Flores C, Castillo-Gurrola J (2007). Saline intrusion in Guaymas Valley, Mexico from time-domain electromagnetic soundings. Geofís. Int..

[771] Martínez-Santos P, Castaño-Castaño S, Hernández-Espriú A (2018). Revisiting groundwater overdraft based on the experience of the Mancha Occidental Aquifer, Spain. Hydrol. J..

[772] Marvin, R. F., Shafer, G. H. & Dale, O. C. Groundwater resources of Victoria and Calhoun Counties, Texas. https://www.twdb.texas.gov/publications/reports/bulletins/doc/Bull.htm/B6202.asp (1962).

[773] Mashburn, S. L., Ryter, D. W., Neel, C. R., Smith, S. J. & Correll, J. S. Hydrogeology and simulation of ground-water flow in the Central Oklahoma (Garber-Wellington) Aquifer, Oklahoma, 1987 to 2009, and simulation of avail-able water in storage, 2010–2059. U.S. Geological Survey Scientific Investigations Report 2013-5219. https://pubs.usgs.gov/sir/2013/5219/pdf/sir20135219_v2.0.pdf (2014).

[774] Masoumi M, Gharaie MHM, Ahmadzadeh H (2019). Assessment of groundwater quality for the irrigation of melon farms: a comparison between two arable plains in northeastern Iran. Environ. Earth Sci..

[775] Masterson, J. P. et al. Assessment of groundwater availability in the Northern Atlantic Coastal Plain aquifer system from Long Island, New York, to North Carolina. U.S. Geological Survey Professional Paper 1829. https://pubs.usgs.gov/pp/1829/pp1829.pdf (2016).

[776] Masterson, J. P. et al. Hydrogeology and hydrologic conditions of the Northern Atlantic Coastal Plain aquifer system from Long Island, New York, to North Carolina. U.S. Geological Survey Scientific Investigations Report 2013-5133. 10.3133/sir20135133 (2013).

[777] Masterson, J. P. & Walter, D. A. Hydrogeology and groundwater resources of the coastal aquifers of southeastern Massachusetts. U.S. Geological Survey Circular 1338. https://pubs.usgs.gov/circ/circ1338/pdf/circular%202009-1338_508.pdf (2009).

[778] Mathany, T. M., Wright, M. T., Beuttel, B. S. & Belitz, K. Groundwater-quality data in the Borrego Valley, Central Desert, and low-use basins of the Mojave and Sonoran Deserts study unit, 2008–2010: results from the California GAMA Program. U.S. Geological Survey Data Series 659. https://pubs.usgs.gov/ds/659/pdf/ds659.pdf (2012).

[779] Mather B (2022). Constraining the response of continental-scale groundwater flow to climate change. Sci. Rep..

[780] Matlock, W. G., Davis, P. R. & Roth, R. L. Groundwater in Little Chino Valley, Arizona: Tucson, University of Arizona, College of Agriculture, Agricultural Experiment Station, Technical Bulletin 201. https://repository.arizona.edu/bitstream/handle/10150/602177/TB178.pdf?sequence=1 (1973).

[781] Maurer, D. K. Geologic framework and hydrogeology of the middle Carson River Basin, Eagle, Dayton, and Churchill Valleys, West-Central Nevada. U.S. Geological Survey Scientific Investigations Report 2011-5055. https://pubs.usgs.gov/sir/2011/5055/pdf/sir20115055.pdf (2011).

[782] Maurer, D. K. & Thodal, C. E. Quantity and chemical quality of recharge, and updated water budgets, for the basin-fill aquifer in Eagle Valley, western Nevada. U.S. Geological Survey Water-Resources Investigations Report 99-4289. https://pubs.usgs.gov/wri/1999/4289/report.pdf (2000).

[783] Maxey, G. B. & Eakin, T. E. Ground water in White River Valley, White Pine, Nye, and Lincoln Counties, Nevada. U.S. Department of the Interior Water Resources Bulletin No. 8. https://www.nrc.gov/docs/ML0331/ML033140348.pdf (1949).

[784] Mayer A, Nguyen BT, Banton O (2016). Using radon-222 to study coastal groundwater/surface-water interaction in the Crau coastal aquifer (southeastern France). Hydrol. J..

[785] Mayo AL, Henderson RM, Tingey D, Webber W (2010). Chemical evolution of shallow playa groundwater in response to post-pluvial isostatic rebound, Honey Lake Basin, California–Nevada, USA. Hydrol. J..

[786] McGuire, V. L., Johnson, M. R., Schieffer, J. S., Stanton, J. S., Sebree, S. K. & Varstraeten, I. M. Water in storage and approaches to groundwater management, High Plains Aquifer, 2000. U.S. Geological Survey Circular 1243. https://pubs.usgs.gov/circ/2003/circ1243/pdf/C1243.pdf (2003).

[787] McLean, J. S. Saline ground-water resources of the Tularosa basin, New Mexico. U.S. Geological Survey OSW Report No. 561. https://pubs.usgs.gov/unnumbered/70139928/report.pdf (1970).

[788] Meinzer, O. E. Artesian water for irrigation in Little Bitterroot Valley, Montana. Water Supply Paper 400. https://pubs.usgs.gov/wsp/0400b/report.pdf (1916).

[789] Mejía-González MÁ, González-Hita L, Espinoza-Ayala J, González-Verdugo JA (2012). Determinación de las aportaciones de agua dulce a las lagunas costeras Chacahua y Salina Grande, Oaxaca, México, por medio de isótopos ambientales. Tecnol. Cienc. Agua.

[790] Mendez, G. O. & Christensen, A. H. Regional water table (1996) and water-level changes in the Mojave River, the Morongo, and the Fort Irwin ground-water basins, San Bernardino County, Calif., 38 pp. Accessed April 27, 2022 via https://pubs.usgs.gov/wri/1997/4160/report.pdf (1997).

[791] Mendez-Estrella R, Romo-Leon JR, Castellanos AE, Gandarilla-Aizpuro FJ, Hartfield K (2016). Analyzing landscape trends on agriculture, introduced exotic grasslands and riparian ecosystems in arid regions of Mexico. Remote Sens..

[792] Meng, A. & Harsh, J. F. Hydrogeologic framework of the Virginia coastal plain. U.S. Geological Survey Professional Paper 1404-C. https://pubs.usgs.gov/pp/pp1404-C/pdf/pp_1404-c.pdf (1988).

[793] Meng S (2015). Spatiotemporal evolution characteristics study on the precipitation infiltration recharge over the past 50 years in the North China Plain. J. Earth Sci..

[794] Meredith KT (2018). Evolution of dissolved inorganic carbon in groundwater recharged by cyclones and groundwater age estimations using the ^14^C statistical approach. Geochim. Cosmochim. Acta.

[795] Meredith K, Cendón DI, Pigois JP, Hollins S, Jacobsen G (2012). Using ^14^C and ^3^H to delineate a recharge ‘window’ into the Perth Basin aquifers, North Gnangara groundwater system, Western Australia. Sci. Total Environ..

[796] Miall AD (2013). Geoscience of climate and energy 13. The environmental hydrogeology of the Oil Sands, Lower Athabasca Area, Alberta. Geosci. Can..

[797] Michael HA, Voss CI (2009). Controls on groundwater flow in the Bengal Basin of India and Bangladesh: regional modeling analysis. Hydrol. J..

[798] Mihaylova, B. et al. in *Transboundary Aquifers: Challenges and the Way Forward Topic 3 Paper 11*, 110–107 (UNESCO, 2022).

[799] Miller, J. A. Ground Water Atlas of the United States: Segment 10, Illinois, Indiana, Kentucky, Ohio, Tennessee. U.S. Geological Survey Hydrologic Investigations Atlas 730-K. https://pubs.usgs.gov/ha/730k/report.pdf (1995).

[800] Miller, J. A. Ground Water Atlas of the United States: Segment 6, Alabama, Florida, Georgia, South Carolina. U.S. Geological Survey Hydrologic Investigations Atlas 730-G. https://pubs.usgs.gov/ha/730g/report.pdf (1990).

[801] Miller, J. A. & Appel, C. L. Ground Water Atlas of the United States: Segment 3, Kansas, Missouri, Nebraska. U.S. Geological Survey Hydrologic Investigations Atlas 730-D. https://pubs.usgs.gov/ha/730d/report.pdf (1997).

[802] Minderhoud PSJ (2017). Impacts of 25 years of groundwater extraction on subsidence in the Mekong delta, Vietnam. Environ. Res. Lett..

[803] Ministere de l’Ecologie, du Developpement Durable et de l’Energie. Hydrologie souterraine synthèse. BRGM report. https://professionnels.ofb.fr/sites/default/files/pdf/RE_Explore2070_Eaux_Sout_Synthese.pdf (2012).

[804] Ministerio de Medio Ambiente y Recursos Naturales. Informe de monitoreo de los Acuíferos de Zapotitán, Santa Ana y San Miguel. Ministerio de Medio Ambiente y Recursos Naturales report. http://rcc.marn.gob.sv/bitstream/handle/123456789/127/Acuiferos%20%20StaAna%2c%20SnMiguel%20y%20Zapotit%c3%a1n_2016.pdf?sequence=1&isAllowed=y (2016).

[805] Ministerio de Medio Ambiente y Recursos Naturales. Mapa Hidrogeológico de El Salvador. https://www.sica.int/documentos/mapa-hidrogeologico-de-el-salvador_1_128021.html (2021).

[806] Ministerio de Medio Ambiente y Recursos Naturales. Mapa Hidrogeológico de El Salvador. http://srt.snet.gob.sv/sihi/public/atlas (2023).

[807] Ministerio de Medio Ambiente y Recursos Naturales. Objetivos de Calidad de Agua, Ríos, Lagos y Embalses ZP1. http://srt.snet.gob.sv/sihi/public/atlas (2023).

[808] Ministerio de Medio Ambiente y Recursos Naturales Plan Nacional de Gestión Integrada del Recurso Hídrico de El Salvador, con énfasis en zonas prioritarias. Report by the Ministerio de Medio Ambiente y Recursos Naturales (MARN). http://rcc.marn.gob.sv/bitstream/handle/123456789/259/Resumen%20Ejecutivo%20PNGRH%202017.compressed.pdf?sequence=1&isAllowed=y (2017).

[809] Minnesota Department of Natural Resources Minnesota Groundwater Provinces 2021. Minnesota Department of Natural Resources map, 2 pp. Accessed April 14, 2021 from https://files.dnr.state.mn.us/waters/groundwater_section/mapping/provinces/2021-provinces.pdf (2021).

[810] Mirzaei R, Sakizadeh M (2016). Comparison of interpolation methods for the estimation of groundwater contamination in Andimeshk-Shush Plain, Southwest of Iran. Environ. Sci. Pollut. Res..

[811] Mirzavand M, Ghasemieh H, Sadatinejad SJ, Bagheri R (2020). Delineating the source and mechanism of groundwater salinization in crucial declining aquifer using multi-chemo-isotopes approaches. J. Hydrol..

[812] Miyakoshi A, Uchida Y, Sakura Y, Hayashi T (2003). Distribution of subsurface temperature in the Kanto Plain, Japan; estimation of regional groundwater flow system and surface warming. Phys. Chem. Earth A/B/C.

[813] Miyazaki, S., Hasegawa, S., Kayaki, T. & Osamu, W. in *Hydro-environments of Alluvial Fans in Japan, Monograph, 36th IAH Congress* (International Association for Hydro-Environment Engineering and Research, 2008).

[814] Tabari MMR, Kabiri Samani M (2019). Groundwater quality assessment using entropy weighted osculating value and set pair analysis methods (case study, SARAYAN plain). J. Environ. Sci. Technol..

[815] Mohammadi Z, Zare M, Sharifzade B (2012). Delineation of groundwater salinization in a coastal aquifer, Bousheher, South of Iran. Environ. Earth Sci..

[816] Mohammadzadeh-Habili J (2021). Influences of natural salinity sources and human actions on the Shapour River salinity during the recent streamflow reduction period. Environ. Monit. Assess..

[817] Mohammadzadeh-Habili J, Soltani M, Khalili D (2021). Effect of reservoir geometry on functionality of recharge dams influenced by sedimentation: case study of the Meymand recharge dam. Arab. J. Geosci..

[818] Mohammed N (2014). Isotopic and geochemical identification of main groundwater supply sources to an alluvial aquifer, the Allier River valley (France). J. Hydrol..

[819] Mohebbi Tafreshi G, Mohebbi Tafreshi A (2020). Statistical approaches and hydrochemical modeling of groundwater in the Golpayegan Plain aquifer, Iran. Model. Earth Syst. Environ..

[820] Mojarrad, M., Rakhshandehrou, G. R., Monadi, R. & Ghorbani, M. in *Proc. 2nd International Conference of Water Resources and Wetlands* (eds Gâştescu, P. & Marszelewski, W.) 336–343 (UNESCO, 2014).

[821] Mojiri H, Halabian A (2019). Evaluation of the effects of temporal variables of temperature, precipitation and water harvesting on groundwater resources in Mehrgerd basin of Semirom. J. Watershed Manag. Res..

[822] Mokhtar A, Aram S (2017). Systemic insights into agricultural groundwater management: case of Firuzabad Plain, Iran. Water Policy.

[823] Mokrik R, Mazeika J, Baublyt A, Martma T (2009). The groundwater age in the Middle-Upper Devonian aquifer system, Lithuania. Hydrol. J..

[824] Mora A, Mahlknecht J, Rosales-Lagarde L, Hernández-Antonio A (2017). Assessment of major ions and trace elements in groundwater supplied to the Monterrey metropolitan area, Nuevo León, Mexico. Environ. Monit. Assess..

[825] Morales, P., Casar, I., Cortes, A., Arizabalo, R. D. & Aravena, R. Environmental isotopes and geochemical investigation of groundwater in the north-western part of the State of Morelos, Mexico (IAEA-TECDOC-502). International Atomic Energy Agency (IAEA). https://inis.iaea.org/collection/NCLCollectionStore/_Public/21/031/21031083.pdf?r=1 (1989).

[826] Morales-Casique E (2012). Mixing of groundwaters with uncertain end-members: case study in the Tepalcingo-Axochiapan aquifer, Mexico. Hydrol. J..

[827] Moratilla, F. E. & Pérez, C. M. O. Aplicación de la tomografía remota térmica a la investigación de la hidrogeología y dinámica de flujos de las aguas subterráneas de la cuenca del río Júcar. Crisis y medio ambiente:¿ Oportunidad o retroceso? https://www.mapa.gob.es/ministerio/pags/biblioteca/revistas/pdf_AM%5CAMBIENTA_101.pdf (2012).

[828] Morell I (2003). Acuíferos detríticos costeros. Hidrogeol. Aguas Subterrán..

[829] Morikawa N (2008). Relationship between geological structure and helium isotopes in deep groundwater from the Osaka Basin: application to deep groundwater hydrology. Geochem. J..

[830] Morín, P. M. Aplicación de un modelo numérico para simular el flujo hidráulico del acuífero de Ojos Negros. MSc thesis, Ensenada Center for Scientific Research and Higher Education (2013).

[831] Morrison, R. B. Ground-water resources of the Big Sandy Valley, Mohave County, Arizona. U.S. Geological Survey Report. https://azmemory.azlibrary.gov/nodes/view/91763?keywords= (1940).

[832] Moslemi H (2019). Assessment of groundwater crisis in arid and semiarid areas (case study: Jaghin and Tokahor Plain). Irrig. Sci. Eng..

[833] Motagh M (2008). Land subsidence in Iran caused by widespread water reservoir overexploitation. Geophys. Res. Lett..

[834] Motevalli, A., Pourghasemi, H. R., Hashemi, H. & Gholami, V. in *Spatial Modeling in GIS and R for Earth and Environmental Sciences* (eds Pourghasemi, H. R. & Gokceoglu, C.) 547–571 (Elsevier, 2019).

[835] Moura, A. & Velho, J. L. in *Recursos Geologicos de Portugal* Ch. 57, 523–536 (Palimage, 2012).

[836] Mower, R. W. & Feltis, R. D. Ground-water hydrology of the Sevier Desert, Utah. U.S. Geological Survey Water-Supply Paper 1854. https://pubs.usgs.gov/wsp/1854/report.pdf (1968).

[837] Mthembu PP, Elumalai V, Brindha K, Li P (2020). Hydrogeochemical processes and trace metal contamination in groundwater: impact on human health in the Maputaland coastal aquifer, South Africa. Expos. Health.

[838] Muir, K. S. Ground-water reconnaissance of the Santa Barbara-Montecito area, Santa Barbara County, California. U.S. Geological Survey Water-Supply Paper 1859-A. https://pubs.usgs.gov/wsp/1859a/report.pdf (1968).

[839] Muir, M. A. K. & Martinez, A. A. Preliminary assessment of water resources including climate considerations for the Los Cabos and La Paz municipalities in the State of Baja California Sur, Mexico. International Water Association Water, Energy and Climate Conference. https://arctic.ucalgary.ca/sites/default/files/April18-IWA-FinalConferenceVersion-MAKMuir.pdf (2018).

[840] Mukherjee A, Fryar AE, Howell PD (2007). Regional hydrostratigraphy and groundwater flow modeling in the arsenic-affected areas of the western Bengal basin, West Bengal, India. Hydrol. J..

[841] Mukherjee A (2014). Revisiting the stratigraphy of the Mesoproterozoic Chhattisgarh Supergroup, Bastar craton, India based on subsurface lithoinformation. J. Earth Syst. Sci..

[842] Mukherjee A (2015). Groundwater systems of the Indian sub-continent. J. Hydrol. Reg. Stud..

[843] Municipio de El Llano. Atlas de Riesgos Naturales del Municipio de El Llano 2012. Report number 201010PP047745. http://rmgir.proyectomesoamerica.org/PDFMunicipales/2012/01010_El_Llano.pdf (2012).

[844] Munro-Stasiuk MJ, Manahan TK (2010). Investigating ancient Maya agricultural adaptation through ground penetrating radar (GPR) analysis of karst terrain, Northern Yucatán, Mexico. Acta Carsologica.

[845] Murray-Darling Basin Commission. Murray-Darling Basin groundwater: a resource for the future. Murray-Darling Basin Commission. https://catalogue.nla.gov.au/catalog/3024769 (1999).

[846] Musy S (2021). In-situ sampling for krypton-85 groundwater dating. J. Hydrol..

[847] Naderi M (2020). Assessment of water security under climate change for the large watershed of Dorudzan Dam in southern Iran. Hydrol. J..

[848] Naghibi SA, Vafakhah M, Hashemi H, Pradhan B, Alavi SJ (2018). Groundwater augmentation through the site selection of floodwater spreading using a data mining approach (case study: Mashhad Plain, Iran). Water.

[849] Nakai I (2023). Quality of the groundwater in Toyooka Basin. J. Groundwat. Hydrol..

[850] Nandakumaran P, Balakrishnan K (2020). Groundwater quality variations in Precambrian hard rock aquifers: a case study from Kerala, India. Appl. Water Sci..

[851] Naranjo, R. C., Welborn, T. L. & Rosen, M. R. The distribution and modeling of nitrate transport in the Carson Valley alluvial aquifer, Douglas County, Nevada. U.S. Geological Survey Scientific Investigations Report 2013–5136. https://pubs.usgs.gov/sir/2013/5136/pdf/sir2013-5136.pdf (2013).

[852] Naranjo-Fernández N, Guardiola-Albert C, Aguilera H, Serrano-Hidalgo C, Montero-González E (2020). Clustering groundwater level time series of the exploited Almonte-Marismas aquifer in Southwest Spain. Water.

[853] Narayan KA, Schleeberger C, Bristow KL (2007). Modelling seawater intrusion in the Burdekin Delta irrigation area, North Queensland, Australia. Agric. Water Manag..

[854] Nasiri A, Shirocova VA, Zareie S (2019). Zoning of groundwater quality for plain Garmsar in Iran. Water Resour..

[855] Nasiri M, Hamidi M, Kardan Moghaddam H (2019). Investigation of groundwater quantitative and qualitative variations trends (case study: Sari-Neka aquifer). J. Aquifer Qanat.

[856] Nath B, Jean JS, Lee MK, Yang HJ, Liu CC (2008). Geochemistry of high arsenic groundwater in Chia-Nan plain, Southwestern Taiwan: possible sources and reactive transport of arsenic. J. Contam. Hydrol..

[857] Nativ R, Weisbrod N (1994). Management of a multilayered coastal aquifer—an Israeli case study. Water Resour. Manag..

[858] Navarro, B. J. B. Estado y evolución de los procesos de intrusión marina en la unidad hidrogeológica 08.38 plana de Gandía-Denia (Valencia-Alicante, España). Tecnología De La Intrusión de Agua De Mar en Acuíferos Costeros: Países Mediterráneos. http://aguas.igme.es/igme/publica/tiac-01/Area%20V-17.pdf (2003).

[859] Nazari S, Ahmadi A (2019). Non-cooperative stability assessments of groundwater resources management based on the tradeoff between the economy and the environment. J. Hydrol..

[860] Nazari S, Ahmadi A, Rad SK, Ebrahimi B (2020). Application of non-cooperative dynamic game theory for groundwater conflict resolution. J. Environ. Manag..

[861] Negarash H, Shafiei N, Doraninejad MS (2016). Hydro-geomorphology effect of Nurabad Mamasani plain aquifer on the region’s water resources using GIS. Hydrogeomorphology.

[862] Neilson-Welch, L. & Allen, D. Groundwater and hydrogeological conditions in the Okanagan Basin, British Columbia: a state-of-the-basin report. Final report prepared for Objective 1 of the Phase 2 Groundwater Supply and Demand Project. https://www.obwb.ca/fileadmin/docs/water_supply_demand/water_supply_demand_final_report.pdf (2007).

[863] Nel, L. *The Geology of the Springbok Flats*. PhD dissertation, Univ. Free State (2012).

[864] Nell JP, Van Huyssteen CW (2014). Geology and groundwater regions to quantify primary salinity, sodicity and alkalinity in South African soils. S. Afr. J. Plant Soil.

[865] Nematollahi MJ, Ebrahimi P, Ebrahimi M (2016). Evaluating hydrogeochemical processes regulating groundwater quality in an unconfined aquifer. Environ. Process..

[866] Nematollahi MJ, Ebrahimi P, Razmara M, Ghasemi A (2016). Hydrogeochemical investigations and groundwater quality assessment of Torbat-Zaveh plain, Khorasan Razavi, Iran. Environ. Monit. Assess..

[867] Newcomb, R. C. Geology and ground-water resources of the Walla Walla River Basin, Washington-Oregon. Washington Division of Water Resources Water Supply Bulletin No. 21. https://apps.ecology.wa.gov/publications/documents/wsb21.pdf (1965).

[868] Nguyen TT (2015). Clustering spatio–seasonal hydrogeochemical data using self-organizing maps for groundwater quality assessment in the Red River Delta, Vietnam. J. Hydrol..

[869] Nickerson, E. L. & Myers, R. G. Geohydrology of the Mesilla ground-water basin, Dona Ana County, New Mexico, and El Paso County, Texas. U.S. Geological Survey Water-Resources Investigations Report 92-4156. https://pubs.usgs.gov/wri/1992/4156/report.pdf (1993).

[870] Nilzad M, Moradi H, Jalili K (2018). Estimation of temporal and spatial variations of the level of the aquifers in Bisotun plain of Kermanshah province with geostatistical methods. Irrig. Water Eng..

[871] Nishikawa, T. (ed.) Santa Barbara and Foothill groundwater basins geohydrology and optimal water resources management—developed using density dependent solute transport and optimization models. U.S. Geological Survey Scientific Investigations Report 2018-5059. https://pubs.usgs.gov/sir/2018/5059/sir20185059_.pdf (2018).

[872] Nitcheva O (2018). Hydrology models approach to estimation of the groundwater recharge: case study in the Bulgarian Danube watershed. Environ. Earth Sci..

[873] Nolan, S., Tan, P.-L. & Cox, M. Collaborative water planning: groundwater visualisation tool guide. Charles Darwin University. http://www.nespnorthern.edu.au/wp-content/uploads/2016/02/GVT_Griffith-Uni_13-May-2010-with-corrections1.pdf (2010).

[874] Noma Y, Kino Y, Goto H (2023). Ground water in the Kuzuryu River Basin, Fukui Prefecture [in Japanese]. Bull. Geol. Surv. Jpn..

[875] Norouzi H, Moghaddam AA (2020). Groundwater quality assessment using random forest method based on groundwater quality indices (case study: Miandoab plain aquifer, NW of Iran). Arab. J. Geosci..

[876] Nosrati K, Van Den Eeckhaut M (2012). Assessment of groundwater quality using multivariate statistical techniques in Hashtgerd Plain, Iran. Environ. Earth Sci..

[877] NSW Department of Planning and Environment. Lachlan alluvium groundwater resource description. NSW Department of Planning and Environment report. https://water.dpie.nsw.gov.au/__data/assets/pdf_file/0010/175969/Lachlan-alluvium-appendice-a-water-resource-description.pdf (2019).

[878] Nuñez Codoseo, J. *Evaluación de la disponibilidad de agua del Sector Acuífero Chacabuco-Polpaico: Factibilidad de entrega de nuevos derechos de aprovechamiento de agua provisionales*. Thesis, Universidad de Chile (2017).

[879] Nyambe IA (1999). Tectonic and climatic controls on sedimentation during deposition of the Sinakumbe Group and Karoo Supergroup, in the mid-Zambezi Valley Basin, southern Zambia. J. Afr. Earth. Sci..

[880] Nystrom, E. A. Ground-water quality in the Lake Champlain Basin, New York, 2004. U.S. Geological Survey Open-File Report 2006-1088. https://pubs.usgs.gov/of/2006/1088/pdf/Nystrom.OFR2006-1088.pdf (2006).

[881] Ojeda Olivares EA (2019). Climate change, land use/land cover change, and population growth as drivers of groundwater depletion in the central valleys, Oaxaca, Mexico. Remote Sens..

[882] Olcott, P. G. Ground Water Atlas of the United States: Segment 12, Connecticut, Maine, Massachusetts, New Hampshire, New York, Rhode Island, Vermont. U.S. Geological Survey Hydrologic Investigations Atlas 730-M. https://pubs.usgs.gov/ha/730m/report.pdf (1995).

[883] Olcott, P. G. Groundwater Atlas of the United States: Segment 9, Iowa, Michigan, Minnesota, Wisconsin. U.S. Geological Survey Hydrologic Investigations Atlas 730-J. https://pubs.usgs.gov/ha/730j/report.pdf (1992).

[884] Olmsted, F. H., Loeltz, O. J. & Irelan, B. Geohydrology of the Yuma area, Arizona and California. U.S. Geological Survey Professional Paper 486-H. https://pubs.usgs.gov/pp/0486h/report.pdf (1973).

[885] Ong’or BT, Long-Cang S (2009). Groundwater overdraft and the impact of artificial recharge on groundwater quality in a cone of depression, Jining, China. Water Int..

[886] Opluštil S (2005). The effect of paleotopography, tectonics and sediment supply on quality of coal seams in continental basins of central and western Bohemia (Westphalian), Czech Republic. Int. J. Coal Geol..

[887] Opluštil S, Lojka R, Pšenika J (2013). Late Variscan continental basins in western Bohemia: tectono-sedimentary, climate and biotic archives. Schriftreihe Dtsch. Ges. Geowiss..

[888] Orban P (2010). Regional transport modelling for nitrate trend assessment and forecasting in a chalk aquifer. J. Contam. Hydrol..

[889] Oregon Water Resources Department, Well Report Query. https://apps.wrd.state.or.us/apps/gw/well_log/Default.aspx (2021).

[890] Orehova, T. V. Groundwater in the watershed of Tundja River, Bulgaria. http://router.geology.bas.bg/~orehova/pdf/2006_Groundwater%20Tundja.pdf (2006).

[891] Oroji B (2019). Groundwater vulnerability assessment with using GIS in Hamadan–Bahar plain, Iran. Appl. Water Sci..

[892] Oroji B, Karimi ZF (2018). Application of DRASTIC model and GIS for evaluation of aquifer vulnerability: case study of Asadabad, Hamadan (western Iran). Geosci. J..

[893] Ortiz Letechipia J (2022). Aqueous arsenic speciation with hydrogeochemical modeling and correlation with fluorine in groundwater in a semiarid region of Mexico. Water.

[894] Osborn, N. I. Update of the hydrologic survey of the Tillman Terrace Groundwater Basin, southwestern Oklahoma. Oklahoma Water Resources Board Technical Report GW2002-1. https://www.owrb.ok.gov/studies/reports/reports_pdf/tillman_update.pdf (2002).

[895] Ossa-Valencia J, Betancur-Vargas T (2018). Hydrogeochemical characterization and identification of a system of regional flow. Case study: the aquifer on the Gulf of Urabá, Colombia. Rev. Fac. Ing. Univ. Antioquia.

[896] Othman A, Abotalib AZ (2019). Land subsidence triggered by groundwater withdrawal under hyper-arid conditions: case study from Central Saudi Arabia. Environ. Earth Sci..

[897] Othman A (2018). Use of geophysical and remote sensing data for assessment of aquifer depletion and related land deformation. Surv. Geophys..

[898] Owen DD, Raiber M, Cox ME (2015). Relationships between major ions in coal seam gas groundwaters: examples from the Surat and Clarence-Moreton basins. Int. J. Coal Geol..

[899] Oyarzún R (2014). Multi-method assessment of connectivity between surface water and shallow groundwater: the case of Limarí River basin, north-central Chile. Hydrol. J..

[900] Oyarzún R (2015). A hydrogeochemistry and isotopic approach for the assessment of surface water–groundwater dynamics in an arid basin: the Limarí watershed, North-Central Chile. Environ. Earth Sci..

[901] Pacheco-Martínez J (2013). Land subsidence and ground failure associated to groundwater exploitation in the Aguascalientes Valley, México. Eng. Geol..

[902] Padilla I, Irizarry C, Steele K (2011). Historical contamination of groundwater resources in the north coast karst aquifers of Puerto Rico. Rev. Dimens..

[903] Panahi MR, Mousavi SM, Rahimzadegan M (2017). Delineation of groundwater potential zones using remote sensing, GIS, and AHP technique in Tehran–Karaj plain, Iran. Environ. Earth Sci..

[904] Parent, M., Rivard, C., Lefebvre, R., Carrier, M.-A. & Séjourné, S. Hydrogeological systems of the Montérégie Est region, southern Québec: Fieldtrip Guidebook, GeoMontreal 2013 Conference. Geological Survey of Canada Open File 7605 (2014).

[905] Parizi E, Hosseini SM, Ataie-Ashtiani B, Simmons CT (2020). Normalized difference vegetation index as the dominant predicting factor of groundwater recharge in phreatic aquifers: case studies across Iran. Sci. Rep..

[906] Parks, K. & Andriashek, L. Preliminary investigation of potential, natural hydraulic pathways between the Scollard and Paskapoo formations in Alberta: implications for coalbed methane production. ERCB/AGS Open File Report 2009-16. https://static.ags.aer.ca/files/document/OFR/OFR_2009_16.pdf (2009).

[907] ParsiMehr M, Shayesteh K, Godini K (2020). The modeling and prediction of the quality of the groundwater resources in Tuyserkan plain using the optimized artificial neural network. J. Adv. Environ. Health Res..

[908] Parvaiz A (2021). Salinity enrichment, sources and its contribution to elevated groundwater arsenic and fluoride levels in Rachna Doab, Punjab Pakistan: Stable isotope (δ^2^H and δ^18^O) approach as an evidence. Environ. Pollut..

[909] Paschke, S. S. Groundwater availability of the Denver Basin aquifer system, Colorado. U.S. Geological Survey Professional Paper 1770. https://pubs.usgs.gov/pp/1770/contents/pp1770.pdf (2011).

[910] Pastén-Zapata E, Ledesma-Ruiz R, Harter T, Ramírez AI, Mahlknecht J (2014). Assessment of sources and fate of nitrate in shallow groundwater of an agricultural area by using a multi-tracer approach. Sci. Total Environ..

[911] Patenaude M, Baudron P, Labelle L, Masse-Dufresne J (2020). Evaluating bank-filtration occurrence in the Province of Quebec (Canada) with a GIS approach. Water.

[912] Pathak D (2011). Hydrogeology of shallow and deep aquifers in Nara Basin, West Japan. J. Nepal Geol. Soc..

[913] Paul, B., Raper, P., Simons, J., Stainer, G. & George, R. Weaber Plain aquifer test results. Government of Western Australia, Department of Agriculture and Food Resource Management Technical Report 367. https://library.dpird.wa.gov.au/cgi/viewcontent.cgi?article=1362&context=rmtr (2011).

[914] Payne BR, Quijano L, Latorre DC (1979). Environmental isotopes in a study of the origin of salinity of groundwater in the Mexicali Valley. J. Hydrol..

[915] Pazand K (2016). Geochemical and statistical evaluation of groundwater in Razan basin, Western Iran. Carbonates Evaporites.

[916] Pazand K, Javanshir AR (2014). Geochemistry and water quality assessment of groundwater around Mohammad Abad Area, Bam District, SE Iran. Water Qual. Expos. Health.

[917] Pazand K, Javanshir AR (2013). Hydrogeochemistry and arsenic contamination of groundwater in the Rayen area, southeastern Iran. Environ. Earth Sci..

[918] Pazand K, Khosravi D, Ghaderi MR, Rezvanianzadeh MR (2018). Identification of the hydrogeochemical processes and assessment of groundwater in a semi-arid region using major ion chemistry: a case study of Ardestan basin in Central Iran. Groundw. Sustain. Dev..

[919] Peeters, L., Batelaan, O. & Dassargues, A. Identification and quantification of sources of major solutes in a sandy, phreatic aquifer in Central Belgium through ionic ratios and geochemical mass-balance modelling. https://orbi.uliege.be/bitstream/2268/3587/1/publi159-2007.pdf (2007).

[920] Peña, L. C. B. et al. Identificación de áreas potenciales de recarga hídrica en el acuífero Cuauhtémoc (Chihuahua), mediante una evaluación espacial multi criterio. Estudios territoriales en México: Percepción remota y sistemas de información espacial, 339–362 (2016).

[921] Peng TR (2012). Using oxygen, hydrogen, and tritium isotopes to assess pond water’s contribution to groundwater and local precipitation in the pediment tableland areas of northwestern Taiwan. J. Hydrol..

[922] Perry, E., Velaquez-Oliman, V. & Socki, R. A. in *The Lowland Maya Area: Three Millennia at the Human-Wildland Interface* (eds Fedick, S., Allen, M., Jim?nez-Osornio, J. & Gomez-Pompa, A.) 115–138 (CRC, 2003).

[923] Peterson, S. M., Traylor, J. P. & Guira, M. Groundwater availability of the Northern High Plains aquifer in Colorado, Kansas, Nebraska, South Dakota, and Wyoming. U.S. Geological Survey Professional Paper 1864. https://pubs.usgs.gov/pp/1864/pp1864.pdf (2020).

[924] Pétré MA, Rivera A, Lefebvre R, Hendry MJ, Folnagy AJ (2016). A unified hydrogeological conceptual model of the Milk River transboundary aquifer, traversing Alberta (Canada) and Montana (USA). Hydrol. J..

[925] Pettifer, G. Bundaberg groundwater investigation, Australia – a case for the benefits of extensive use of geophysics in groundwater investigations. https://library.seg.org/doi/pdf/10.4133/1.2923407 (2004).

[926] Phiancharoen, C. *Interpretation of the Chemical Analyses of the Ground Water of the Khorat Plateau, Thailand*. MSc thesis, Univ. Arizona (1962).

[927] Phien-wej N, Giao PH, Nutalaya P (2006). Land subsidence in Bangkok, Thailand. Eng. Geol..

[928] Phillips FM, Bentley HW, Davis SN, Elmore D, Swanick GB (1986). Chlorine 36 dating of very old groundwater: 2. Milk River aquifer, Alberta, Canada. Water Resour. Res..

[929] Pimentel ET, Hamza VM (2014). Use of geothermal methods in outlining deep groundwater flow systems in Paleozoic interior basins of Brazil. Hydrol. J..

[930] Pinault JL, Doerfliger N, Ladouche B, Bakalowicz M (2004). Characterizing a coastal karst aquifer using an inverse modeling approach: the saline springs of Thau, southern France. Water Resour. Res..

[931] Pino E (2019). Factors affecting depletion and pollution by marine intrusion in the La Yarada’s coastal aquifer, Tacna., Peru. Tecnol. Cienc. Agua.

[932] Pino-Vargas E, Guevara-Pérez E, Avendaño-Jihuallanga C (2021). Historical evolution of the hydrogeological conceptualization and the use of Caplina aquifer on the northern edge of the Atacama Desert. Rev. Ing. UC.

[933] Pisani, J. Regional groundwater level analysis pre-summer 2020. Staff Report to the Regional District of Nanaimo. https://rdn-pub.escribemeetings.com/filestream.ashx?DocumentId=13450 (2020).

[934] Piyapong J, Thidarat B, Jaruwan C, Siriphan N, Passanan A (2019). Enhancing citizens’ sense of personal responsibility and risk perception for promoting public participation in sustainable groundwater resource management in Rayong Groundwater Basin, Thailand. Groundw. Sustain. Dev..

[935] Plume, R. W. Hydrogeologic framework and occurrence and movement of ground water in the upper Humboldt River basin, northeastern Nevada. U.S. Geological Survey Scientific Investigations Report 2009-5014. https://pubs.usgs.gov/sir/2009/5014/pdf/sir20095014.pdf (2009).

[936] Ponce VM, Pandey RP, Kumar S (1999). Groundwater recharge by channel infiltration in El Barbon basin, Baja California, Mexico. J. Hydrol..

[937] Poulsen, D. Culverden Basin hydrogeology. Environment Canterbury Regional Council Report No. R12/96. https://www.ecan.govt.nz/document/download?uri=1723844 (2012).

[938] Pourkhosravani M (2016). Qualitative analysis of Orzooiyeh plain groundwater resources using GIS techniques. Environ. Health Eng. Manag. J..

[939] Powell, W. J. Ground-water resources of the San Luis Valley, Colorado. U.S. Geological Survey Water-Supply Paper 1379. https://pubs.usgs.gov/wsp/1379/report.pdf (1958).

[940] Pratt, T. R. et al. Hydrogeology of the Northwest Florida Water Management District. Northwest Florida Water Management District, Water Resources Special Report, 96-4. (1996).

[941] Barraclough, J. T. & Marsh, O. T. Aquifers and quality of ground water along the Gulf Coast of western Florida. U.S. Geological Survey Report of Investigations No. 29. https://ufdcimages.uflib.ufl.edu/UF/00/00/12/16/00001/UF00001216.pdf (1962).

[942] Price, D. Ground water in Utah’s densely populated Wasatch Front area—the challenge and the choices. U.S. Geological Survey Water-Supply Paper 2232. https://pubs.usgs.gov/wsp/2232/report.pdf (1985).

[943] Priestley SC (2018). Use of U-isotopes in exploring groundwater flow and inter-aquifer leakage in the south-western margin of the Great Artesian Basin and Arckaringa Basin, central Australia. Appl. Geochem..

[944] Priestley SC (2020). A 35 ka record of groundwater recharge in south-west Australia using stable water isotopes. Sci. Total Environ..

[945] Priju, C., Sushanth, C. M. & Balan, V. Delineation of freshwater zones in the shallow coastal aquifers of Ernakulam-Chettuva region, Central Kerala, India using electrical resistivity methods. https://assets.researchsquare.com/files/rs-369371/v1/7e2408f9-0dd0-49c2-ab11-1a4457d52b58.pdf (2021).

[946] Prior, J. C., Boekhoff, J. L., Howes, M. R., Libra, R. D. & VanDorpe, P. E. Iowa’s groundwater basics. A geological guide to the occurrence, use, & vulnerability of Iowa’s aquifers. Iowa Department of Natural Resources report. https://s-iihr34.iihr.uiowa.edu/publications/uploads/2014-08-24_08-08-21_es-06.pdf (2003).

[947] Pulido-Bosch, A. *Principles of Karst Hydrogeology: Conceptual Models, Time Series Analysis, Hydrogeochemistry and Groundwater Exploitation* (Springer, 2020).

[948] Pulido-Bosch A (1999). Groundwater problems in the karstic aquifers of the Dobrich region, northeastern Bulgaria. Hydrol. Sci. J..

[949] Pulido-Bosch A, Morell I, Andreu JM (1995). Hydrogeochemical effects of groundwater mining of the Sierra de Crevillente Aquifer (Alicante, Spain). Environ. Geol..

[950] Pulido-Velazquez D, Ahlfeld D, Andreu J, Sahuquillo A (2008). Reducing the computational cost of unconfined groundwater flow in conjunctive-use models at basin scale assuming linear behaviour: the case of Adra-Campo de Dalías. J. Hydrol..

[951] Putthividhya A, Laonamsai J (2017). Hydrological assessment using stable isotope fingerprinting technique in the Upper Chao Phraya river basin. Lowl. Technol. Int..

[952] Qasemi M (2018). Health risk assessment of nitrate exposure in groundwater of rural areas of Gonabad and Bajestan, Iran. Environ. Earth Sci..

[953] Qasemi M, Afsharnia M, Zarei A, Farhang M, Allahdadi M (2018). Non-carcinogenic risk assessment to human health due to intake of fluoride in the groundwater in rural areas of Gonabad and Bajestan, Iran: a case study. Hum. Ecol. Risk Assess..

[954] Qasim A, Singh SP, Chandrashekhar AK (2022). Geochemical and isotope tracing of groundwater salinity in the coastal Gujarat alluvial plain, India. J. Contam. Hydrol..

[955] Qian K, Li J, Xie X, Wang Y (2017). Organic and inorganic colloids impacting total iodine behavior in groundwater from the Datong Basin, China. Sci. Total Environ..

[956] Qin D (2011). Assessing impact of irrigation water on groundwater recharge and quality in arid environment using CFCs, tritium and stable isotopes, in the Zhangye Basin, Northwest China. J. Hydrol..

[957] Quezadas JP, Heilweil VM, Silva AC, Araguas L, Ortega MDRS (2016). A multi-tracer approach to delineate groundwater dynamics in the Rio Actopan Basin, Veracruz State, Mexico. Hydrol. J..

[958] Radell, M. J. *Three-dimensional Groundwater Flow Model Use and Application: Bishop Basin, Owens Valley, California*. MSc thesis, Univ. Arizona. (1989).

[959] Radell, M. J., Lewis, M. E. & Watts, K. R. Hydrogeologic characteristics of the alluvial aquifer and adjacent deposits of the Fountain Creek valley, El Paso County, Colorado. U.S. Geological Survey Water-Resources Investigations Report 94-4129. https://pubs.er.usgs.gov/publication/wri944129 (1994).

[960] Radfar M, Van Camp M, Walraevens K (2013). Drought impacts on long-term hydrodynamic behavior of groundwater in the tertiary–quaternary aquifer system of Shahrekord Plain, Iran. Environ. Earth Sci..

[961] Rahbar A (2020). A hydrogeochemical analysis of groundwater using hierarchical clustering analysis and fuzzy C-mean clustering methods in Arak plain, Iran. Environ. Earth Sci..

[962] Rahimi S, Roodposhti MS, Abbaspour RA (2014). Using combined AHP–genetic algorithm in artificial groundwater recharge site selection of Gareh Bygone Plain, Iran. Environ. Earth Sci..

[963] Rahmati O, Samani AN, Mahmoodi N, Mahdavi M (2015). Assessment of the contribution of N-fertilizers to nitrate pollution of groundwater in western Iran (case study: Ghorveh–Dehgelan Aquifer). Water Qual. Expos. Health.

[964] Ramirez E, Robles E, Sainz M, Ayala R, Campoy E (2009). Microbiological quality of the Zacatepec aquifer, Morelos, Mexico. Rev. Int. Contam. Ambient..

[965] Randich, P. G. & Kuzniar, R. L. Ground-water resources of Towner County, North Dakota. North Dakota State Water Commission report. http://swc.state.nd.us/info_edu/reports_and_publications/county_groundwater_studies/pdfs/Towner_Part_III.pdf (1984).

[966] Rangel-Medina, M., Monreal, R., Minjarez, I., de la Cruz, L. & Oroz, L. The saline intrusion in the Costa de Hermosillo aquifer in Sonora, México; a challenge to restore. http://www.swim-site.nl/pdf/swim18/swim18_059.pdf (2004).

[967] Ransley, T. R. et al. Hydrogeological atlas of the Great Artesian Basin. Geoscience Australia. http://www.ga.gov.au/scientific-topics/water/groundwater/gab (2015).

[968] Ransley, T. R. & Smerdon, B. D. Hydrostratigraphy, hydrogeology and system conceptualisation of the Great Artesian Basin. A technical report to the Australian Government from the CSIRO Great Artesian Basin Water Resource Assessment. https://publications.csiro.au/rpr/download?pid=csiro:EP132693&dsid=DS5 (2012).

[969] Raper, G. P. et al. Groundwater trend analysis and salinity risk assessment for the south-west agricultural region of Western Australia, 2007–12. Government of Western Australia, Department of Agriculture and Food Resource Management Technical Report 388. https://library.dpird.wa.gov.au/cgi/viewcontent.cgi?article=1372&context=rmtr (2014).

[970] Raper, P., George, R. & Schoknecht, N. Preliminary soil and groundwater assessment of the Mantinea Development area, East Kimberley, Western Australia. Western Australian Agriculture Authority resource management technical report 389. https://www.agric.wa.gov.au/sites/gateway/files/Preliminary%20soil%20and%20groundwater%20assessment%20of%20the%20Mantinea%20Development%20area%2C%20East%20Kimberley%20-%20RMTR%20389%20%28PDF%204.2MB%29.pdf (2015).

[971] Rathfelder, K. & Gregory, L. Groundwater quality assessment and proposed objectives for the Osoyoos Aquifer. Water Science Series: WSS2019-06. https://a100.gov.bc.ca/pub/acat/documents/r57603/1_1571784531661_1784376098.pdf (2019).

[972] Rathore VS, Nathawat MS, Ray PC (2008). Influence of neotectonic activity on groundwater salinity and playa development in the Mendha river catchment, western India. Int. J. Remote Sens..

[973] Rattray G (2015). Geochemical evolution of groundwater in the Mud Lake area, Eastern Idaho, USA. Environ. Earth Sci..

[974] Ravenscroft P, McArthur JM, Rahman MS (2018). Identifying multiple deep aquifers in the Bengal Basin: implications for resource management. Hydrol. Process..

[975] Reichard, E. G. et al. Geohydrology, geochemistry, and ground-water simulation-optimization of the Central and West Coast Basins, Los Angeles County, California. U.S. Geological Survey Water-Resources Investigations Report 03-4065. https://pubs.usgs.gov/wri/wrir034065/wrir034065.pdf (2003).

[976] Reidel, S. P., Spane, F. A. & Johnson, V. G. Natural gas storage in basalt aquifers of the Columbia basin, Pacific Northwest USA: a guide to site characterization. Pacific Northwest National Lab (PNNL) Report No. PNNL-13962. https://www.pnnl.gov/main/publications/external/technical_reports/PNNL-13962.pdf (2002).

[977] Render, F. W. Aquifer capacity investigations 1980–1986. Manitoba Water Resources Hydrotechnical Services report. https://www.gov.mb.ca/water/pubs/water-science-management/groundwater/publication/1987_render_aquifer_capacity_investigations_1980_1986.pdf (1987).

[978] Render FW (1988). Water supply capacity of the Assiniboine Delta Aquifer. Can. Water Resour. J..

[979] Renken, R. A. et al. Geology and hydrogeology of the Caribbean islands aquifer system of the commonwealth of Puerto Rico and the US Virgin Islands. U.S. Geological Survey Professional Paper 1419. https://pubs.usgs.gov/pp/pp1419/pdf/BOOK.PDF (2002).

[980] Renken, R. A. Groundwater Atlas of the United States: Segment 5, Arkansas, Louisiana, Mississippi. U.S. Geological Survey Hydrologic Investigations Atlas 730-F. https://pubs.usgs.gov/ha/730f/report.pdf (1998).

[981] Retter A (2021). Application of the D-A-(C) index as a simple tool for microbial-ecological characterization and assessment of groundwater ecosystems—a case study of the Mur River Valley, Austria. Oesterr. Wasser- Abfallwirtsch..

[982] Reza AS (2011). A comparative study on arsenic and humic substances in alluvial aquifers of Bengal delta plain (NW Bangladesh), Chianan plain (SW Taiwan) and Lanyang plain (NE Taiwan): implication of arsenic mobilization mechanisms. Environ. Geochem. Health.

[983] Rezaei A, Hassani H (2018). Hydrogeochemistry study and groundwater quality assessment in the north of Isfahan, Iran. Environ. Geochem. Health.

[984] Rezaei A (2019). Evaluation of groundwater quality and heavy metal pollution indices in Bazman basin, southeastern Iran. Groundw. Sustain. Dev..

[985] Rezaei A, Hassani H, Tziritis E, Mousavi SBF, Jabbari N (2020). Hydrochemical characterization and evaluation of groundwater quality in Dalgan basin, SE Iran. Groundw. Sustain. Dev..

[986] Ribeiro L (2015). Evaluating piezometric trends using the Mann-Kendall test on the alluvial aquifers of the Elqui River basin, Chile. Hydrol. Sci. J..

[987] Richardson, G. B. Underground water in Sanpete and Central Sevier Valleys, Utah. U.S. Geological Survey Water-Supply and Irrigation Paper No. 199. https://pubs.usgs.gov/wsp/0199/report.pdf (1907).

[988] Rinehart, A., Koning, D. & Timmons, S. Hydrogeology of the San Agustin Plains. Presentation at the 62nd New Mexico Water Conference. https://geoinfo.nmt.edu/geoscience/research/documents/37/D2_07_Alex_Rinehart.pdf (2017).

[989] Rivard C, Michaud Y, Lefebvre R, Deblonde C, Rivera A (2008). Characterization of a regional aquifer system in the Maritimes Basin, Eastern Canada. Water Resour. Manag..

[990] Rivera-Hernández JR, Green-Ruiz C, Pelling-Salazar L, Trejo-Alduenda A (2017). Hydrochemistry of the Mocorito river coastal aquifer, Sinaloa, Mexico: water quality assessment for human consumption and agriculture suitability. Hidrobiológica.

[991] Roark, D. M., Holmes, W. F. & Shlosar, H. K. Hydrology of Heber and Round Valleys, Wasatch County, Utah, with emphasis on simulation of ground-water flow in Heber Valley. U.S. Geological Survey Technical Publication No. 101. https://waterrights.utah.gov/docSys/v920/y920/y9200009.pdf (1991).

[992] Robertson AJ (2022). Mesilla/Conejos-Médanos Basin: US-Mexico transboundary water resources. Water.

[993] Robins, N. S. & Ball, D. F. The Dumfries Basin aquifer. British Geological Survey Research Report RR/06/02. http://nora.nerc.ac.uk/id/eprint/3685/1/RR06002.pdf (2006).

[994] Robles E, Ramirez E, de Guadalupe Sáinz M, Duran A, González ME (2013). Bacteriological and physicochemical study on the water of an aquifer in Mexico. Univers. J. Environ. Res. Technol..

[995] Rodgers, K. D. Water-level trends and potentiometric surfaces in the Nacatoch Aquifer in northeastern and southwestern Arkansas and in the Tokio Aquifer in southwestern Arkansas, 2014–15. U.S. Geological Survey Scientific Investigations Report 2017-5090. https://pubs.usgs.gov/sir/2017/5090/sir20175090.pdf (2017).

[996] Rodrigo-Naharro J, Aracil E, del Villar LP (2018). Geophysical investigations in the Gañuelas-Mazarrón Tertiary basin (SE Spain): a natural analogue of a geological CO_2_ storage affected by anthropogenic leakages. J. Appl. Geophys..

[997] Rodríguez L, Vives L, Gomez A (2013). Conceptual and numerical modeling approach of the Guarani Aquifer System. Hydrol. Earth Syst. Sci..

[998] Rodriguez-Rodriguez M, Martos-Rosillo S, Pedrera A (2016). Hydrogeological behaviour of the Fuente-de-Piedra playa lake and tectonic origin of its basin (Malaga, southern Spain). J. Hydrol..

[999] Rojas R (2018). Groundwater resource assessment and conceptualization in the Pilbara Region, Western Australia. Earth Syst. Environ..

[1000] Roques C, Bour O, Aquilina L, Dewandel B (2016). High-yielding aquifers in crystalline basement: insights about the role of fault zones, exemplified by Armorican Massif, France. Hydrol. J..

[1001] Rosário de Jesus, M. Groundwater protection for public water-supply in Portugal. https://unece.org/fileadmin/DAM/env/water/meetings/groundwater01/portugal.pdf (2001).

[1002] Rose, T. P., Davisson, M. L., Smith, D. K. & Kenneally, J. M. Isotope hydrology investigation of regional groundwater flow in central Nevada. Hydrologic Resources Management Program and Underground Test Area Operable Unit FY 1997 Progress Report, Ch. 6. https://core.ac.uk/download/pdf/204554577.pdf#page=62 (1998).

[1003] Rose, T. P., Davisson, M. L., Hudson, G. B. & Varian, A. R. Environmental isotope investigation of groundwater flow in the Honey Lake Basin, California and Nevada. Department of Energy Report UCRL-ID-127978 ON: DE98051049. https://www.osti.gov/servlets/purl/620597 (1997).

[1004] Rostami AA, Isazadeh M, Shahabi M, Nozari H (2019). Evaluation of geostatistical techniques and their hybrid in modelling of groundwater quality index in the Marand Plain in Iran. Environ. Sci. Pollut. Res..

[1005] Rostkier‐Edelstein D (2014). Towards a high‐resolution climatography of seasonal precipitation over Israel. Int. J. Climatol..

[1006] Rotzoll K, Gingerich SB, Jenson JW, El-Kadi AI (2013). Estimating hydraulic properties from tidal attenuation in the Northern Guam Lens Aquifer, territory of Guam, USA. Hydrol. J..

[1007] Rouillard, J. & Maréchal, J.-C. in *Sustainable Groundwater Management: A Comparative Analysis of French and Australian Policies and Implications to Other Countries* (eds Rinaudo, J.-D., Holley, C., Barnett, S. & Montginoul, M.) 17–45 (Springer, 2020).

[1008] Rupérez-Moreno C, Pérez-Sánchez J, Senent-Aparicio J, del Pilar Flores-Asenjo M (2015). The economic value of conjoint local management in water resources: results from a contingent valuation in the Boquerón aquifer (Albacete, SE Spain). Sci. Total Environ..

[1009] Rupérez-Moreno C (2017). Sustainability of irrigated agriculture with overexploited aquifers: the case of Segura basin (SE, Spain). Agric. Water Manag..

[1010] Rushton KR, Rao SR (1988). Groundwater flow through a Miliolite limestone aquifer. Hydrol. Sci. J..

[1011] Rutulis, M. Aquifer maps of southern Manitoba. Manitoba Water Resources Branch map. https://www.gov.mb.ca/water/pubs/maps/water/1986_rutulis_bedrock_aquifers.pdf (1986).

[1012] Ruybal CJ, Hogue TS, McCray JE (2019). Assessment of groundwater depletion and implications for management in the Denver Basin Aquifer System. J. Am. Water Resour. Assoc..

[1013] Ryder, P. Ground Water Atlas of the United States: Segment 4, Oklahoma, Texas. U.S. Geological Survey Hydrologic Investigations Atlas 730-E. https://pubs.usgs.gov/ha/730e/report.pdf (1996).

[1014] Saadatmand A, Noorollahi Y, Yousefi H, Mohammadi A (2021). Investigation, modeling and analysis of qualitative parameters of groundwater resources in Kurdistan’s Kamyaran plain. Iran. J. Ecohydrol..

[1015] Sabzevari Y, Nasrolahi AH, Yonesi HA (2020). Investigation of temporal-spatial variations of groundwater resources quality in Borujerd-Dorood Plain. Irrig. Water Eng..

[1016] Sadeghfam S, Hassanzadeh Y, Nadiri AA, Khatibi R (2016). Mapping groundwater potential field using catastrophe fuzzy membership functions and Jenks optimization method: a case study of Maragheh-Bonab plain, Iran. Environ. Earth Sci..

[1017] Sadid, N. Surface-groundwater interaction in the Kabul region basin. Afghanistan Research and Evaluation Unit Report. https://reliefweb.int/sites/reliefweb.int/files/resources/2005-E-Surface-groundwater-interaction-in-the-Kabul-region-basin.pdf (2020).

[1018] Saeidi H, Lashkaripour G, Ghafoori M (2020). Evaluation of land subsidence in Kashmar-Bardaskan plain, NE Iran. Iran. J. Earth Sci..

[1019] Saffari A, Jan Ahmadi M, Raeati Shavazi M (2015). Site selection for suitable flood spreadingand artificial feeding through hybrid, AHP-Fuzzy Model Case Study: (Bushkan Plain, Bushehr Province). Hydrogeomorphology.

[1020] Saffi, M. H. National alarming on groundwater natural storage depletion and water quality deterioration of Kabul City and immediate response to the drinking water crises. Scientific Investigation Report in Afghanistan, DACAAR report (2019).

[1021] Saha D, Gor N (2020). A prolific aquifer system is in peril in arid Kachchh region of India. Groundw. Sustain. Dev..

[1022] Saha, D. & Ray, R. K. in *Groundwater Development and Management* (ed. Sikdar, P. K.) 19–42 (Springer, 2019).

[1023] Saha D, Shekhar S, Ali S, Vittala SS, Raju NJ (2016). Recent hydrogeological research in India. Proc. Indian Natl Sci. Acad..

[1024] Sahoo S, Dhar A, Kar A, Chakraborty D (2016). Index-based groundwater vulnerability mapping using quantitative parameters. Environ. Earth Sci..

[1025] Sahu JK, Das PP, Sahoo HK, Mohapatra PP, Sahoo S (2018). Geospatial analysis and hydrogeochemical investigation of a part of southern Mahanadi delta, Odisha, India. Himal. Geol..

[1026] Sahu S, Gogoi U, Nayak NC (2020). Patterns of groundwater chemistry: implications of groundwater flow and the relation with groundwater fluoride contamination in the phreatic aquifer of Odisha, India. Arab. J. Geosci..

[1027] Sajil Kumar PJ, James EJ (2016). Identification of hydrogeochemical processes in the Coimbatore district, Tamil Nadu, India. Hydrol. Sci. J..

[1028] Sakai A (2001). Land subsidence due to seasonal pumping of groundwater in Saga Plain, Japan. Lowl. Technol. Int..

[1029] Salehabadi G (2021). The effect of groundwater in plain settlement in Jovin. Sci. Res. Q. Geogr. Data.

[1030] Salehi H, Zeinivand H (2016). Evaluation and mapping of groundwater quality for rigation and drinking purposes in Kuhdasht region, Iran. Environ. Resour. Res..

[1031] Salemi, H. R. et al. Water management for sustainable irrigated agriculture in the Zayandeh Rud Basin, Esfahan Province, Iran. Report by Iranian Agricultural Engineering Research Institute, Esfahan Agricultural Research Center and the International Water Management Institute, Research Report Number 1 (2000).

[1032] Salinas Valley Basin Integrated Sustainability Plan. https://svbgsa.org/wp-content/uploads/2019/03/Valley-Wide-Integrated-Sustainability-Plan-optimized.pdf (2020).

[1033] Saltel M (2019). Paleoclimate variations and impact on groundwater recharge in multi-layer aquifer systems using a multi-tracer approach (northern Aquitaine basin, France). Hydrol. J..

[1034] Samantaray S, Rath A, Swain PC (2017). Conjunctive use of groundwater and surface water in a part of Hirakud Command Area. Int. J. Eng. Technol..

[1035] Samper, J. et al. Evaluació de los impactos del cambio climático e los acuíferos de la pla a de la galera y del aluvial de Tortosa. Estudios en la Zona no Saturada del Suelo. Vol. X, 359–364. http://zonanosaturada.com/zns11/publications/p359.pdf (2011).

[1036] Sanchez R, Eckstein G (2020). Groundwater management in the borderlands of Mexico and Texas: the beauty of the unknown, the negligence of the present, and the way forward. Water Resour. Res..

[1037] Sanchez R, Lopez V, Eckstein G (2016). Identifying and characterizing transboundary aquifers along the Mexico–US border: an initial assessment. J. Hydrol..

[1038] Sandberg, G. W. Ground-water resources of selected basins in southwestern Utah. U.S. Geological Survey Open Technical Publication 13. https://waterrights.utah.gov/docSys/v920/w920/w920008c.pdf (1966).

[1039] Sandiford M, Lawrie K, Brodie RS (2020). Hydrogeological implications of active tectonics in the Great Artesian Basin, Australia. Hydrol. J..

[1040] Sanford WE, Buapeng S (1996). Assessment of a groundwater flow model of the Bangkok Basin, Thailand, using carbon-14-based ages and paleohydrology. Hydrol. J..

[1041] Sanford, W. E., Pope, J. P., Selnick, D. L. & Stumvoll, R. F. Simulation of groundwater flow in the shallow aquifer system of the Delmarva Peninsula, Maryland and Delaware. U.S. Geological Survey Open-File Report 2012–1140. https://pubs.usgs.gov/of/2012/1140/pdf/OFR_2012-1140.pdf (2012).

[1042] Santha N, Sangkajan S, Saenton S (2022). Arsenic contamination in groundwater and potential health risk in Western Lampang Basin, Northern Thailand. Water.

[1043] Santoni S (2016). Strontium isotopes as tracers of water-rocks interactions, mixing processes and residence time indicator of groundwater within the granite-carbonate coastal aquifer of Bonifacio (Corsica, France). Sci. Total Environ..

[1044] Sanz D (2011). Modeling aquifer–river interactions under the influence of groundwater abstraction in the Mancha Oriental System (SE Spain). Hydrol. J..

[1045] Savoca, M. E., Sadorf, E. M. & Akers, K. K. Ground-water quality in the eastern part of the Silurian-Devonian and Upper Carbonate Aquifers in the eastern Iowa basins, Iowa and Minnesota, 1996. U.S. Geological Survey Water-Resources Investigations Report 98-4224. https://pubs.usgs.gov/wri/1998/wri984224/pdf/wri98-4224.pdf (1999).

[1046] Schoewe WH (1949). The geography of Kansas: Part II. Physical geography. Trans. Kans. Acad. Sci..

[1047] Schrader, G. P. Unconsolidated aquifer systems of Ripley County, Indiana. Indiana Department of Natural Resources, Division of Water report. https://www.in.gov/dnr/water/files/ripley_unconsolidated_text.pdf (2004).

[1048] Schult, J. Herbicides, pesticides and nutrients in the Tindall aquifer, Katherine Region. Northern Territory Government, Department of Land Resource Management report. https://landresources.nt.gov.au/__data/assets/pdf_file/0019/282160/GWQ-report.pdf (2016).

[1049] Schwennesen, A. T. & Forbes, R. H. Ground water in San Simon Valley, Arizona and New Mexico. U.S. Geological Survey Water Supply Paper 425-A. https://pubs.usgs.gov/wsp/0425a/report.pdf (1919).

[1050] Schwennesen, A. T. & Hare, R. F. Ground water in the Animas, Playas, Hachita, and San Luis Basins, New Mexico, with analyses of water and soil. U.S. Geological Survey Water-Supply Paper 422. https://pubs.usgs.gov/wsp/0422/report.pdf (1918).

[1051] Scibek, J. & Allen, D. M. Numerical groundwater flow model of the Abbotsford-Sumas aquifer, central Fraser Lowland of BC, Canada, and Washington State, US. Report prepared for Environment Canada. https://www.sfu.ca/personal/dallen/AB_Modeling_Report_Final.pdf (2005).

[1052] Scott, L., Hanson, C. & Cressy, C. Groundwater quality investigation of the mid-Waitaki valley. Environment Canterbury Regional Council Kaunihera Taiao ki Waitaha Report No. R12/71. http://citeseerx.ist.psu.edu/viewdoc/download?doi=10.1.1.799.6506&rep=rep1&type=pdf (2012).

[1053] Scott, T.-M., Nystrom, E. A. & Reddy, J. E. Groundwater quality in the Lake Champlain and Susquehanna River basins, New York, 2014. U.S. Geological Survey Open-File Report 2016-1153. https://pubs.usgs.gov/of/2016/1153/ofr20161153.pdf (2016).

[1054] Selck BJ (2018). Investigating anthropogenic and geogenic sources of groundwater contamination in a semi-arid alluvial basin, Goshen Valley, UT, USA. Water Air Soil Pollut..

[1055] Semeniuk V, Semeniuk CA (2006). Sedimentary fill of basin wetlands, central Swan Coastal Plain, southwestern Australia. Part 2: distribution of sediment types and their stratigraphy. J. R. Soc. West. Aust..

[1056] Senthilkumar, M. & Gnanasundar, D. Hydrogeological characterization and hydrological modeling for devising groundwater management strategies for Chennai aquifer system, Southern India. https://www.authorea.com/doi/full/10.22541/au.158990356.67099058 (2020).

[1057] Seraphin P, Gonçalvès J, Vallet-Coulomb C, Champollion C (2018). Multi-approach assessment of the spatial distribution of the specific yield: application to the Crau plain aquifer, France. Hydrol. J..

[1058] Serrat P, Lenoble JL (2007). La surexploitation des aquifères du Roussillon: une ressource patrimoniale en danger. Houille Blanche.

[1059] Serviço Geológico do Brasil. Aquífero Urucuia Caracterização hidrológica com base em dados secundários. Inistério de Minas e Energia Secretaria de Geologia, Mineração e Transformação Mineral Serviço Geológico do Brasil (CPRM) report. https://rigeo.cprm.gov.br/jspui/handle/doc/20922 (2019).

[1060] Shabani M (2012). Determining the most suitable interpolation method for groundwater chemical characteristics mapping. Watershed Eng. Manag..

[1061] Shah T (2014). Towards a managed aquifer recharge strategy for Gujarat, India: an economist’s dialogue with hydro-geologists. J. Hydrol..

[1062] Shahmohammadi-Kalalagh S, Taran F, Nasiri H (2020). Investigating groundwater level fluctuations via analyzing groundwater hydrograph: a case study of Naqadeh plain in north-west of Iran. Sustain. Water Resour. Manag..

[1063] Shalyari N, Alinejad A, Hashemi AHG, RadFard M, Dehghani M (2019). Health risk assessment of nitrate in groundwater resources of Iranshahr using Monte Carlo simulation and geographic information system (GIS). MethodsX.

[1064] Shams M (2012). Drinking water in Gonabad, Iran: fluoride levels in bottled, distribution network, point of use desalinator, and decentralized municipal desalination plant water. Fluoride.

[1065] Shamsudduha M (2007). Spatial variability and prediction modeling of groundwater arsenic distributions in the shallowest alluvial aquifers in Bangladesh. J. Spat. Hydrol..

[1066] Sharaf MA, Hussein MT (1996). Groundwater quality in the Saq aquifer, Saudi Arabia. Hydrol. Sci. J..

[1067] Sharpe, D. R. et al. in: *Canada’s Groundwater Resources*, (ed. Rivera, A.) 444–499 (Fitzhenry and Whiteside, 2013).

[1068] Shelton, J. L., Fram, M. S., Munday, C. M. & Belitz, K. Groundwater-quality data for the Sierra Nevada study unit, 2008. Results from the California GAMA program. U.S. Geological Survey Data Series 534. https://pubs.usgs.gov/ds/534/ds_534.pdf (2010).

[1069] Sheppard, G. M. *The Hydrogeology of the Kaikoura Plains, North Canterbury, New Zealand*. PhD dissertation, Univ. Canterbury (1995).

[1070] Shintani T (2022). Three-dimensional structure and sources of groundwater masses beneath the Osaka Plain, Southwest Japan. J. Hydrol. Reg. Stud..

[1071] Shterev KD (2004). The hydrogeothermal basin of Sofia graben (Bulgaria). Environ. Geol..

[1072] Shu LC, Liu PG, Ong’or BTI (2008). Environmental impact assessment using FORM and groundwater system reliability concept: case study Jining, China. Environ. Geol..

[1073] Siebenthal, C. E. Geology and water resources of the San Luis Valley, Colorado. U.S. Geological Survey Water-Supply Paper 240. https://pubs.usgs.gov/wsp/0240/report.pdf (1910). 10.1126/science.31.802.744-b 17736840

[1074] Sikandar P, Bakhsh A, Arshad M, Rana T (2010). The use of vertical electrical sounding resistivity method for the location of low salinity groundwater for irrigation in Chaj and Rachna Doabs. Environ. Earth Sci..

[1075] Silar, J. & Silar, J. in *Application of Tracers in Arid Zone Hydrology* (eds Adar, E. M. & Leibundgut, C.) 141–150 (IAHS, 1995).

[1076] Simonson BM, Schubel KA, Hassler SW (1993). Carbonate sedimentology of the early Precambrian Hamersley Group of western Australia. Precambrian Res..

[1077] Simpson, M. A. Geology and hydrostratigraphy of the Rosetown Area (72O), Saskatchewan. Saskatchewan Research Council Publication No. 10416-2C98. https://www.wsask.ca/wp-content/uploads/2021/08/Groundwater-Resources-Report-Rosetown.pdf (1998).

[1078] Singaraja C (2015). A study on the status of saltwater intrusion in the coastal hard rock aquifer of South India. Environ. Dev. Sustain..

[1079] Singh, J., Erenstein, O., Thorpe, W. R. & Varma, A. Crop-livestock interactions and livelihoods in the Gangetic Plains of Uttar Pradesh, India: a regional synthesis. International Livestock Research Institute (2007).

[1080] Singh, Y. & Dubey, D. P. in *Watershed Management for Sustainable Development* (eds Tiwari, R. N. & Pandey, G. P.) 122–134 (Excellent Publishing House, 2014).

[1081] Sinsakul S (2000). Late quaternary geology of the lower central plain, Thailand. J. Asian Earth Sci..

[1082] Sloan M, Gillies JA, Norum DI (1991). Using poor quality groundwater for irrigation in Saskatchewan, Canada. Can. Water Resour. J..

[1083] Smedley PL, Zhang M, Zhang G, Luo Z (2003). Mobilisation of arsenic and other trace elements in fluviolacustrine aquifers of the Huhhot Basin, Inner Mongolia. Appl. Geochem..

[1084] Smerdon, B. D. & Ramsley, T. R. Water resource assessment for the Surat region. A technical report to the Australian Government from the CSIRO Great Artesian Basin Water Resource Assessment. https://publications.csiro.au/rpr/download?pid=csiro:EP132644&dsid=DS4 (2012).

[1085] Smerdon, B. D., Ramsley, T. R., Radke, B. M., Kellett, J. R. Water resource assessment for the Great Artesian Basin. A technical report to the Australian Government from the CSIRO Great Artesian Basin Water Resource Assessment. https://publications.csiro.au/rpr/download?pid=csiro:EP132685&dsid=DS3 (2012).

[1086] Smit PJ (1978). Groundwater recharge in the dolomite of the Ghaap Plateau near Kuruman in the Northern Cape, Republic of South Africa. Water SA.

[1087] Smith, D. W., Buto, S. G. & Welborn, T. L. Groundwater-level change and evaluation of simulated water levels for irrigated areas in Lahontan Valley, Churchill County, west-central Nevada, 1992–2012. U.S. Geological Survey Scientific Investigations Report 2016-5045. https://pubs.usgs.gov/sir/2016/5045/sir20165045.pdf (2016).

[1088] Smith, K. *Assessing the Hydrogeologic Characteristics and Sources of Groundwater Recharge and Flow in the Elandsfontein Aquifer, West Coast, Western Cape, South Africa*. MSc thesis, Univ. Western Cape (2020).

[1089] Smith, L. N. Hydrologic framework of the Lolo-Bitterroot Area ground-water characterization study. Montana Bureau of Mines and Geology. Montana Ground-Water Assessment Atlas 4-B-02. http://mbmg.mtech.edu/pdf-publications/GWAA04B-02.pdf (2006).

[1090] Smith, L. N., LaFave, J. I. & Patton, T. W. Groundwater resources of the Lolo-Bitterroot area: Mineral, Missoula, and Ravalli counties, Montana. Montana Bureau of Mines and Geology. Montana Groundwater Assessment Atlas No. 4. http://www.mbmg.mtech.edu/pdf-publications/gwaa4a.pdf (2013).

[1091] Smith, L. N. Hydrogeologic framework of the southern part of the Flathead Lake Area, Flathead, Lake, Missoula, and Sanders counties, Montana. Montana Bureau of Mines and Geology. Montana Ground-Water Assessment Atlas 2-B-10. http://mbmggwic.mtech.edu/gwcpmaps/gwaa02map10untiled.pdf (2004).

[1092] Smith, M. L., Fontaine, K. & Lewis, S. J. Regional hydrogeological characterisation of the St Vincent Basin, South Australia. Technical Report for the National Collaboration Framework Regional Hydrogeology Project. Geoscience Australia Record 2015/16. https://d28rz98at9flks.cloudfront.net/78884/Rec2015_016.pdf (2015).

[1093] Smith, S. J. et al. Hydrogeology and model-simulated groundwater availability in the Salt Fork Red River aquifer, southwestern Oklahoma, 1980–2015. U.S. Geological Survey Scientific Investigations Report 2021-5003. https://pubs.usgs.gov/sir/2021/5003/sir20215003.pdf (2021).

[1094] Smith, S. J., Ellis, J. H., Wagner, D. L. & Peterson, S. M. Hydrogeology and simulated groundwater flow and availability in the North Fork Red River aquifer, southwest Oklahoma, 1980–2013. U.S. Geological Survey Scientific Investigations Report 2017-5098. https://pubs.usgs.gov/sir/2017/5098/sir20175098.pdf (2017).

[1095] Smolensky, D. A., Buxton, H. T. & Shernoff, P. K. Hydrologic framework of Long Island, New York. U.S. Geological Survey Hydrologic Atlas 709. https://pubs.usgs.gov/ha/709/plate-1.pdf (1990).

[1096] Sneed, M., Brandt, J. T. & Solt, M. Land subsidence, groundwater levels, and geology in the Coachella Valley, California, 1993–2010. U.S. Geological Survey Scientific Investigations Report 2014-5075. https://pubs.usgs.gov/sir/2014/5075/pdf/sir2014-5075.pdf (2014).

[1097] Sohrabi N, Chitsazan M, Amiri V, Nezhad TM (2013). Evaluation of groundwater resources in alluvial aquifer based on MODFLOW program, case study: Evan plain (Iran). Int. J. Agric. Crop Sci..

[1098] Soldo B, Mahmoudi Sivand S, Afrasiabian A, Đurin B (2020). Effect of sinkholes on groundwater resources in arid and semi-arid karst area in Abarkooh, Iran. Environments.

[1099] Soltani Mohammadi A, Sayadi Shahraki A, Naseri AA (2017). Simulation of groundwater quality parameters using ANN and ANN+ PSO models (case study: Ramhormoz Plain). Pollution.

[1100] Soltani S, Asghari Moghaddam A, Barzegar R, Kazemian N (2016). Evaluation of nitrate concentration and vulnerability of the groundwater by GODS and AVI methods (case study: Kordkandi-Duzduzan Plain, East Azarbaijan province). Iran. J. Ecohydrol..

[1101] Soltani S, Moghaddam AA, Barzegar R, Kazemian N, Tziritis E (2017). Hydrogeochemistry and water quality of the Kordkandi-Duzduzan plain, NW Iran: application of multivariate statistical analysis and PoS index. Environ. Monit. Assess..

[1102] Sorensen, J. P. et al. The influence of groundwater abstraction on interpreting climate controls and extreme recharge events from well hydrographs in semi-arid South Africa. *Hydrogeol. J.*, 1–15 (2021).

[1103] Souid F, Birkle P, Worrall F (2019). Water-rock interaction of the Jilh and Tawil aquifers in the Wadi Sirhan Basin, NW Saudi Arabia. E3S Web Conf..

[1104] South African Department of Water Affairs. Aquifer classification of South Africa. https://www.dws.gov.za/Groundwater/documents/Aquifer%20Classification.pdf (2012).

[1105] Squeo FA (2006). Groundwater dynamics in a coastal aquifer in north-central Chile: implications for groundwater recharge in an arid ecosystem. J. Arid. Environ..

[1106] Sreenivas A, Gowtham B, Vinodh K, Kumaresan K (2020). Aquifer mapping of hard rock terrain in parts of Dindigul district, Tamil Nadu. Int. J. Anal. Exp. Modal Anal..

[1107] Srivastava, M. & Poonia, O. P. Transboundary aquifers in Rajasthan, issues & management. Bhujal News, 28–36. https://hindi.indiawaterportal.org/articles/transboundary-aquifers-rajasthan-issues-management (2010).

[1108] Stamos, C. L., Christensen, A. H. & Langenheim, V. Preliminary hydrogeologic assessment near the boundary of the Antelope Valley and El Mirage Valley groundwater basins, California. U.S. Geological Survey Scientific Investigations Report 2017-5065. https://pubs.usgs.gov/sir/2017/5065/sir20175065.pdf (2017).

[1109] Standen AR, Kane JA (2023). The spatial distribution of radiological contaminants in the Hickory aquifer and other aquifers overlying the Llano Uplift, Central Texas. Austin Geol. Soc. Bull..

[1110] Stapinsky, M. et al. Groundwater resources assessment in the Carboniferous Maritimes Basin: preliminary results of the hydrogeological characterization, New Brunswick, Nova Scotia, and Prince Edward Island. Geological Survey of Canada Current Research Report 2002-D8. http://www.gov.pe.ca/photos/original/cle_WA10.pdf (2002).

[1111] State of New Mexico, Office of the State Engineer. Nutt-Hockett Basin Hydrographic Survey Report. https://www.ose.state.nm.us/HydroSurvey/legal_ose_hydro_nutt-hocket.php (1998).

[1112] Steinbrügge, G., Muñoz Pardo, J. F. & Fernández, B. Análisis probabilístico y optimización de los recursos de agua subterránea: el caso del acuífero Maipo-Mapocho, Chile. Ingenieria hidraulica en Mexico, XX, 85–97. https://repositorio.uc.cl/dspace/bitstreams/2172bd6b-172e-4233-806a-c9c2b0af5c13/download (2005).

[1113] Steinich B, Escolero O, Marín LE (1998). Salt-water intrusion and nitrate contamination in the Valley of Hermosillo and El Sahuaral coastal aquifers, Sonora, Mexico. Hydrol. J..

[1114] Stephenson, D. A. Hydrogeology of glacial deposits of the Mahomet Bedrock Valley in east-central Illinois. Illinois State Geological Survey Circular 409. https://www.ideals.illinois.edu/items/35335/bitstreams/112693/data.pdf (1967).

[1115] Stephenson, L. W. The ground-water resources of Mississippi. U.S. Geological Survey Water-Supply Paper 576. https://pubs.usgs.gov/wsp/0576/report.pdf (1941).

[1116] Steuer A, Helwig SL, Tezkan B (2008). Aquifer characterization in the Ouarzazate Basin (Morocco): a contribution by TEM and RMT data. Near Surf. Geophys..

[1117] Stolp BJ (2010). Age dating base flow at springs and gaining streams using helium‐3 and tritium: Fischa‐Dagnitz system, southern Vienna Basin, Austria. Water Resour. Res..

[1118] Story, J. & Lopez-Gunn, E. Comparing conflict in transboundary aquifer management: some insights from a comparative study between Spain and Australia. https://unesdoc.unesco.org/ark:/48223/pf0000190140 (2010).

[1119] Strom, E. W. & Mallory, M. J. Hydrogeology and simulation of ground-water flow in the Eutaw-McShan Aquifer and in the Tuscaloosa aquifer system in northeastern Mississippi. U.S. Geological Survey Water-Resources Investigations Report 94-4223. https://pubs.usgs.gov/wri/1994/4223/report.pdf (1995).

[1120] Subramanian, S. & Balasubramanian, A. Hydrochemical studies of Tiruchendur Coast, Tamilnadu, India. Regional Workshop on Environmental Aspects of Groundwater Development (1994).

[1121] Sun X (2021). Analysis and evaluation of the renewability of the deep groundwater in the Huaihe River Basin, China. Environ. Earth Sci..

[1122] Sun Y, Zhou J, Zho Y, Zeng Y, Chen Y (2019). Influencing factors of groundwater organic pollution around the Bosten Lake area of Xinjiang, China. E3S Web Conf..

[1123] Sureshjani MK, Amanipoor H, Battaleb-Looie S (2020). The effects of industrial wastewater on groundwater quality of the Boroujen aquifer, Southwest Iran. Nat. Resour. Res..

[1124] Sweetkind, D. S., Faunt, C. C. & Hanson, R. T. Construction of 3-D geologic framework and textural models for Cuyama Valley groundwater basin, California. U.S. Geological Survey Scientific Investigations Report 2013-5127. https://pubs.usgs.gov/sir/2013/5127/pdf/sir2013-5127.pdf (2013).

[1125] Szczucińska A, Dłużewski M, Kozłowski R, Niedzielski P (2019). Hydrochemical diversity of a large alluvial aquifer in an arid zone (Draa river, S Morocco). Ecol. Chem. Eng. S.

[1126] Szynkiewicz A, Medina MR, Modelska M, Monreal R, Pratt LM (2023). Sulfur isotopic study of sulfate in the aquifer of Costa de Hermosillo (Sonora, Mexico) in relation to upward intrusion of saline groundwater, irrigation pumping and land cultivation. Appl. Geochem..

[1127] Tafreshi GM, Nakhaei M, Lak R (2019). Land subsidence risk assessment using GIS fuzzy logic spatial modeling in Varamin aquifer, Iran. GeoJournal.

[1128] Tagma T, Hsissou Y, Bouchaou L, Bouragba L, Boutaleb S (2009). Groundwater nitrate pollution in Souss-Massa basin (south-west Morocco). Afr. J. Environ. Sci. Technol..

[1129] Taheri Zangi S, Vaezihir A (2020). Vulnerability of Shazand Plain subsidence caused by groundwater level reduction using weighting model and its validation analysis using radar interferometry. Iran. J. Ecohydrol..

[1130] Taheri K, Missimer TM, Amini V, Bahrami J, Omidipour R (2020). A GIS-expert-based approach for groundwater quality monitoring network design in an alluvial aquifer: a case study and a practical guide. Environ. Monit. Assess..

[1131] Talebi MS, Fatemi M (2020). Assessment of the quality and quantity of groundwater in Bahadoran plain using neural network methods, geostatistical and multivariate statistical analysis. J. Appl. Res. Water Wastewater.

[1132] Tanachaichoksirikun P, Seeboonruang U (2020). Distributions of groundwater age under climate change of Thailand’s Lower Chao Phraya basin. Water.

[1133] Tanaka, T. Groundwater resources, development and management in the Kanto Plain, Japan. https://core.ac.uk/download/pdf/76125416.pdf (2004).

[1134] Tanigawa K, Hyodo M, Sato H (2013). Holocene relative sea-level change and rate of sea-level rise from coastal deposits in the Toyooka Basin, western Japan. Holocene.

[1135] Taniguchi M (1994). Estimated recharge rates from groundwater temperatures in the Nara Basin, Japan. Appl. Hydrogeol..

[1136] Taucare M (2020). Connectivity of fractures and groundwater flows analyses into the Western Andean Front by means of a topological approach (Aconcagua Basin, Central Chile). Hydrol. J..

[1137] Tauchen, P. et al. Wind/Bighorn River Basin Water Plan Update Groundwater Study Level 1 (2008–2011). Groundwater Determination. Wyoming Water Development Commission Technical Memorandum. https://waterplan.state.wy.us/plan/bighorn/2010/gw-finalrept/gw-finalrept.pdf (2012).

[1138] Tavassoli S, Mohammadi F (2021). Critically assessment of groundwater quality based on WQI and its vulnerability to saltwater intrusion in a coastal city, Iran. Mod. Adv. Geogr. Environ. Earth Sci..

[1139] Taweesin K, Seeboonruang U, Saraphirom P (2018). The influence of climate variability effects on groundwater time series in the lower central plains of Thailand. Water.

[1140] Taylor CB (1989). Sources and flow of north Canterbury plains groundwater, New Zealand. J. Hydrol..

[1141] Taylor, C. J. & Nelson Jr, H. L. A compilation of provisional karst geospatial data for the Interior Low Plateaus physiographic region, central United States. U.S. Geological Survey Data Series 339. https://pubs.usgs.gov/ds/339/pdf/ds339_web.pdf (2008).

[1142] Taylor, G. C. & Ghosh, P. K. Artesian water in the Malabar coastal plain of southern Kerala, India. U.S. Geological Survey Water Supply Paper 1608-D. https://pubs.usgs.gov/wsp/1608d/report.pdf (1964).

[1143] Teng Y (2019). Risk assessment framework for nitrate contamination in groundwater for regional management. Sci. Total Environ..

[1144] Tezangi MF (2016). Studying the effects of drought on groundwater aquifers of Zarand, Kerman. Int. J. Pharm. Res. Allied Sci..

[1145] Thamke, J. N., LeCain, G. D., Ryter, D. W., Sando, R. & Long, A. J. Hydrogeologic framework of the uppermost principal aquifer systems in the Williston and Powder River structural basins, United States and Canada. U.S. Geological Survey Scientific Investigations Report 2014-5047. https://pubs.usgs.gov/sir/2014/5047/pdf/sir2014-5047.pdf (2014).

[1146] Thiros, S. A., Stolp, B. J., Hadley, H. K. & Steiger, J. I. Hydrology and simulation of ground-water flow in Juab Valley, Juab County, Utah. State of Utah Department of Natural Resources, Division of Water Rights Technical Publication No. 114. https://waterrights.utah.gov/docSys/v920/y920/y920000j.pdf (1996).

[1147] Thiros, S. A. Hydrogeology of shallow basin-fill deposits in areas of Salt Lake Valley, Salt Lake County, Utah. U.S. Geological Survey Water-Resources Investigations Report 03-4029. https://pubs.usgs.gov/wri/wri034029/pdf/wri034029.pdf (2003).

[1148] Thomas, H. E. Ground water in Tooele Valley, Tooele County, Utah. State of Utah Department of Natural Resources, Division of Water Rights Technical Publication No. 4. https://waterrights.utah.gov/docSys/v920/w920/w9200083.pdf (1946).

[1149] Thorleifson, L. H. et al. Hydrogeology and hydrogeochemistry of the Red River Valley/Interlake region of Manitoba. Manitoba Energy and Mines, Minerals Division Report of Activities, 172–185 (1998).

[1150] Tickell, S. J. Groundwater resources of the Oolloo Dolostone. Department of Infrastructure Planning and Environment, Natural Resources Division Report 17/2002. https://citeseerx.ist.psu.edu/viewdoc/download?doi=10.1.1.932.9762&rep=rep1&type=pdf (2002).

[1151] Tillman, F. D., Cordova, J. T., Leake, S. A., Thomas, B. E. & Callegary, J. B. Water availability and use pilot: methods development for a regional assessment of groundwater availability, southwest alluvial basins, Arizona. U.S. Geological Survey Scientific Investigations Report 2011-5071. https://pubs.usgs.gov/sir/2011/5071/sir2011-5071_text.pdf (2011).

[1152] Tillman, F. D., Garner, B. D. & Truini, M. Preliminary groundwater flow model of the basin-fill aquifers in Detrital, Hualapai, and Sacramento Valleys, Mohave County, northwestern Arizona. U.S. Geological Survey Scientific Investigations Report 2013-5122. http://pubs.usgs.gov/sir/2013/5122/ (2013).

[1153] Timms NE (2015). Sedimentary facies analysis, mineralogy and diagenesis of the Mesozoic aquifers of the central Perth Basin, Western Australia. Mar. Pet. Geol..

[1154] Tizro TA, Voudouris KS, Kamali M (2014). Comparative study of step drawdown and constant discharge tests to determine the aquifer transmissivity: the Kangavar aquifer case study, Iran. J. Water Resour. Hydraul. Eng..

[1155] Tokarsky, O. Hydrogeologic profile Alberta-Saskatchewan boundary. Report prepared for the Prairie Provinces Water Board. https://www.ppwb.ca/uploads/media/5c81764eb01c3/ppwb-report-78-no-maps-en.pdf?v1 (1985).

[1156] Tokarsky, O. Hydrogeologic profile Saskatchewan-Manitoba boundary. Report prepared for the Prairie Provinces Water Board. https://www.ppwb.ca/uploads/media/5c81764f23261/ppwb-report-79-no-maps-en.pdf?v1 (1985).

[1157] Tomás, R., Lopez-Sanchez, J. M., Delgado, J., Mallorquí Franquet, J. J. & Herrera García, G. in *Droughts: Causes, Effects and Predictions* (ed. Sánchez, J. M.) 253–276 (Nova Science, 2008).

[1158] Tomás R (2005). Mapping ground subsidence induced by aquifer overexploitation using advanced Differential SAR Interferometry: Vega Media of the Segura River (SE Spain) case study. Remote Sens. Environ..

[1159] Tomozawa Y, Onodera SI, Saito M (2019). Estimation of groundwater recharge and salinization in a coastal alluvial plain and Osaka megacity, Japan, using δ^18^O, δD, and Cl^−^. Geomate J..

[1160] Torak, L. J. & Painter, J. A. Geostatistical estimation of the bottom altitude and thickness of the Mississippi River Valley alluvial aquifer. U.S. Geological Survey Scientific Investigations Map 3426. https://pubs.er.usgs.gov/publication/sim3426 (2019).

[1161] Torkamanitombeki H, Rahnamarad J, Saadatkhah N (2018). Groundwater chemical indices changed due to water-level decline, Minab Plain, Iran. Environ. Earth Sci..

[1162] Torres-Martínez JA, Mora A, Knappett PS, Ornelas-Soto N, Mahlknecht J (2020). Tracking nitrate and sulfate sources in groundwater of an urbanized valley using a multi-tracer approach combined with a Bayesian isotope mixing model. Water Res..

[1163] Torres-Martínez JA (2021). Estimation of nitrate pollution sources and transformations in groundwater of an intensive livestock-agricultural area (Comarca Lagunera), combining major ions, stable isotopes and MixSIAR model. Environ. Pollut..

[1164] Torres-Martinez JA (2019). Constraining a density-dependent flow model with the transient electromagnetic method in a coastal aquifer in Mexico to assess seawater intrusion. Hydrol. J..

[1165] Torres-Rondon L, Carrière SD, Chalikakis K, Valles V (2013). An integrative geological and geophysical approach to characterize a superficial deltaic aquifer in the Camargue plain, France. C. R. Geosci..

[1166] Tosaki Y (2017). Deep incursion of seawater into the Hiroshima Granites during the Holocene transgression: evidence from ^36^Cl age of saline groundwater in the Hiroshima area, Japan. Geochem. J..

[1167] Tournoud MG, Payraudeau S, Cernesson F, Salles C (2006). Origins and quantification of nitrogen inputs into a coastal lagoon: application to the Thau lagoon (France). Ecol. Model..

[1168] Tran DA (2021). Groundwater quality evaluation and health risk assessment in coastal lowland areas of the Mekong Delta, Vietnam. Groundw. Sustain. Dev..

[1169] Trapp Jr, H. Hydrology of sand-and-gravel aquifer in central and southern Escambia County, Florida. U.S. Geological Survey Open-File Report 74-218. https://pubs.usgs.gov/of/1974/0218/report.pdf (1973).

[1170] Trapp Jr, H. & Horn, M. A. Ground water atlas of the United States: Segment 11, Delaware, Maryland, New Jersey, North Carolina, Pennsylvania, Virginia, West Virginia. U.S. Geological Survey Hydrologic Investigations Atlas 730-L. https://pubs.usgs.gov/ha/730l/report.pdf (1997).

[1171] Treu F (2017). Intrinsic vulnerability of the Isonzo/Soča high plain aquifer (NE Italy–W Slovenia). J. Maps.

[1172] Truong PV (2015). Hydrogeochemistry characteristics and salinity of groundwater in Quaternary sediments in the coastal zone of Ha Tinh province. Vietnam J. Earth Sci..

[1173] Tucci, P. Use of a three-dimensional model for the analysis of the ground-water flow system in Parker Valley, Arizona and California. U.S. Geological Survey Open-File Report 82-1006. https://pubs.usgs.gov/of/1982/1006/report.pdf (1982).

[1174] U.S. Geological Survey. National water summary 1984: hydrologic events, selected water-quality trends, and ground-water resources. U.S. Geological Survey Water-Supply Paper 2275. https://pubs.usgs.gov/wsp/2275/report.pdf (1984).

[1175] Umvoto Africa. The assessment of water availability in the Berg Catchment (WMA 19) by means of Water Resource Related Models. Department of Water Affairs and Forestry report. https://www.dws.gov.za/Documents/Other/WMA/19/Reports/Rep9-Vol5-GW%20Cape%20Flats%20Aquifer.pdf (2008).

[1176] United States Bureau of Reclamation. Final feasibility-level special study report. Odessa subarea special study. https://www.usbr.gov/pn/programs/eis/odessa/finaleis/final.pdf (2012).

[1177] University of Greenwich and Gujarat Institute of Desert Ecology. Ecosystem assessment of the coastal plain natural area of Kachchh District: planning for biodiversity and livelihoods into the future. Project presentation. https://gala.gre.ac.uk/id/eprint/16221/1/16221%20BARTLETT_Coastal_Plain_of_Kachchh_2016.pdf (2016).

[1178] Upson, J. E. & Thomasson, H. G. Geology and water resources of the Santa Ynez river basin, Santa Barbara County, California, Vol. 2. U.S. Geological Survey Water-Supply Report 1107. https://pubs.usgs.gov/wsp/1107/report.pdf (1951).

[1179] Urresti-Estala B, Gavilán PJ, Pérez IV, Cantos FC (2016). Assessment of hydrochemical trends in the highly anthropised Guadalhorce River basin (southern Spain) in terms of compliance with the European groundwater directive for 2015. Environ. Sci. Pollut. Res..

[1180] Urrutia J (2018). Hydrogeology and sustainable future groundwater abstraction from the Agua Verde aquifer in the Atacama Desert, northern Chile. Hydrol. J..

[1181] US Army Corps of Engineers. Water resources assessment of El Salvador. https://www.sam.usace.army.mil/Portals/46/docs/military/engineering/docs/WRA/ElSalvador/El%20Salvador%20WRA%20English.pdf (1998).

[1182] Uthman, W. & Beck J. Hydrogeology of the Upper Beaverhead Basin near Dillon, Montana. Montana Bureau of Mines and Geology Open-File Report 384. https://dnrc.mt.gov/_docs/water/Hydro_science_data/mbmg_open-file_report_384.pdf (1998).

[1183] Uugulu S, Wanke H (2020). Estimation of groundwater recharge in savannah aquifers along a precipitation gradient using chloride mass balance method and environmental isotopes, Namibia. Phys. Chem. Earth A/B/C.

[1184] Vaccaro, J. J., Hansen, A. J. & Jones, M. A. Hydrogeologic framework of the Puget Sound aquifer system, Washington and British Columbia. U.S. Geological Survey Professional Paper 1424-D. https://pubs.usgs.gov/pp/1424d/report.pdf (1998).

[1185] Vaccaro, J. J. et al. Groundwater availability of the Columbia Plateau Regional Aquifer System, Washington, Oregon, and Idaho. U.S. Geological Survey Professional Paper 1817. 10.3133/pp1817 (2015).

[1186] Vaezihir A, Tabarmayeh M (2015). Total vulnerability estimation for the Tabriz aquifer (Iran) by combining a new model with DRASTIC. Environ. Earth Sci..

[1187] Valin, Z. C. & McLaughlin, R. J. Locations and data for water wells of the Santa Rosa Valley, Sonoma County, California. U.S. Geological Survey Open File Report 2005-1318. https://pubs.usgs.gov/of/2005/1318/of2005-1318.pdf (2005).

[1188] van Geldern R (2014). Pleistocene paleo-groundwater as a pristine fresh water resource in southern Germany–evidence from stable and radiogenic isotopes. Sci. Total Environ..

[1189] Van Lam N, Van Hoan H, Duc Nhan D (2019). Investigation into groundwater resources in southern part of the Red River’s Delta Plain, Vietnam by the use of isotopic techniques. Water.

[1190] Varma, A. Groundwater resource and governance in Kerala. Status, issues and prospects. Forum for Policy Dialogue on Water Conflicts in India. Kerala Resource Centre report. https://www.soppecom.org/pdf/Groundwater-Resource-and-Governance-in-Kerala.pdf (2017).

[1191] Varma S, Michael K (2012). Impact of multi-purpose aquifer utilisation on a variable-density groundwater flow system in the Gippsland Basin, Australia. Hydrol. J..

[1192] Vazquez Sanchez E, Cortes A, Jaimes Palomera R, Fritz P, Aravena R (1989). Hidrogeologia isotopica de los valles de Cuautla y Yautepec, Mexico. Geofís. Int..

[1193] Vazquez JG, Grande JA, Barragán FJ, Ocaña JA, De La Torre ML (2005). Nitrate accumulation and other components of the groundwater in relation to cropping system in an aquifer in Southwestern Spain. Water Resour. Manag..

[1194] Vega-Granillo, E. L., Cirett-Galán, S., De la Parra-Velasco, M. L. & Zavala-Juárez, R. Hidrogeología de Sonora, México. Panorama de la geología de Sonora, México (ed. Calmus, T.) 267–298. Universidad Nacional Autónoma de México, Instituto de Geología, Boletín 118. https://boletin.geologia.unam.mx/index.php/boletin/issue/view/14/12 (2011).

[1195] Vergnes JP (2020). The AquiFR hydrometeorological modelling platform as a tool for improving groundwater resource monitoring over France: evaluation over a 60-year period. Hydrol. Earth Syst. Sci..

[1196] Vetrimurugan E, Elango L, Rajmohan N (2013). Sources of contaminants and groundwater quality in the coastal part of a river delta. Int. J. Environ. Sci. Technol..

[1197] Veve, T. D. & Taggart, B. E. Atlas of Ground-Water Resources in Puerto Rico and the U.S. Virgin Islands. U.S. Geological Survey Water-Resources Investigations Report 94-4198. https://pubs.usgs.gov/wri/1994/4198/report.pdf (1996).

[1198] Villanueva-Hernández H, Tovar-Cabañas R, Vargas-Castilleja R (2019). Classification of aquifers in the Mina field, Nuevo Leon, using geographic information systems. Tecnol. Cienc. Agua.

[1199] Villegas P, Paredes V, Betancur T, Ribeiro L (2013). Assessing the hydrochemistry of the Urabá Aquifer, Colombia by principal component analysis. J. Geochem. Explor..

[1200] Virbulis J, Bethers U, Saks T, Sennikovs J, Timuhins A (2013). Hydrogeological model of the Baltic Artesian Basin. Hydrol. J..

[1201] Vizintin G, Souvent P, Veselič M, Curk BC (2009). Determination of urban groundwater pollution in alluvial aquifer using linked process models considering urban water cycle. J. Hydrol..

[1202] Vogel JC, Talma AS, Heaton THE, Kronfeld J (1999). Evaluating the rate of migration of an uranium deposition front within the Uitenhage Aquifer. J. Geochem. Explor..

[1203] Vroblesky, D. A. & Fleck, W. B. Hydrogeologic Framework of the Coastal Plain of Maryland, Delaware, and the District of Columbia. U.S. Geological Survey Professional Paper 1404-E. https://pubs.usgs.gov/pp/1404e/report.pdf (1991).

[1204] Wacker, M. A., Cunningham, K. J. & Williams, J. H. Geologic and hydrogeologic frameworks of the Biscayne aquifer in central Miami-Dade County, Florida. U.S. Geological Survey Scientific Investigations Report 2014-5138. https://pubs.usgs.gov/sir/2014/5138/pdf/sir2014-5138.pdf (2014).

[1205] Wade, S. & Jigmond, M. Groundwater availability model of west Texas Bolsons (Presidio and Redford) Aquifer. Texas Water Development Board report. https://www.twdb.texas.gov/groundwater/models/gam/prbl/PRBL_ModelFinalReport.pdf (2013).

[1206] Wallace, J. & Lowe, M. Ground-water quality classification for the Principal Basin-fill Aquifer, Salt Lake Valley, Salt Lake County, Utah. Utah Geological Survey Open-File Report 560. https://ugspub.nr.utah.gov/publications/open_file_reports/ofr-560.pdf (2009).

[1207] Wang D, Yang C, Shao L (2021). The spatiotemporal evolution of hydrochemical characteristics and groundwater quality assessment in Urumqi, Northwest China. Arab. J. Geosci..

[1208] Wang L, Iwao Y (2000). Groundwater characteristics of the Saga Plain, Japan. J. Nepal Geol. Soc..

[1209] Wang SJ, Lee CH, Yeh CF, Choo YF, Tseng HW (2021). Evaluation of climate change impact on groundwater recharge in groundwater regions in Taiwan. Water.

[1210] Wang S (2009). Shallow groundwater dynamics in North China plain. J. Geog. Sci..

[1211] Washington State Department of Ecology. Puget Sound groundwater toxics loading analysis: direct discharge pathway. Publication No. 10-03-122. https://apps.ecology.wa.gov/publications/documents/1003122.pdf (2010).

[1212] Water and Marine Resources Division. Tasmanian Aquifer Framework. Groundwater Management Report Series. Report No. GW 2012/02. https://nre.tas.gov.au/Documents/Tasmanian%20Aquifer%20Framework.pdf (2012).

[1213] Watts, K. R. Hydrogeology and quality of ground water in the upper Arkansas River Basin from Buena Vista to Salida, Colorado, 2000–2003. U.S. Geological Survey Scientific Investigations Report 2005-5179. https://pubs.usgs.gov/sir/2005/5179/pdf/SIR2005-5179.pdf (2005).

[1214] Wei, M., Allen, D. M., Carmichael, V. & Ronneseth, K. State of understanding of the hydrogeology of the Grand Forks aquifer. Water Stewardship Division, BC Ministry of Environment Report. https://www.grandforks.ca/wp-content/uploads/reports/2010-Hydrogeology-Study-of-Grand-Forks-area.pdf (2010).

[1215] Weiss, J. S. Geohydrologic units of the coastal lowlands aquifer system, south-central United States. U.S. Geological Survey regional aquifer-system analysis. https://pubs.usgs.gov/pp/1416c/report.pdf (1990).

[1216] Welch, A. H., Sorey, M. L. & Olmsted, F. H. Hydrothermal system in Southern Grass Valley, Pershing County, Nevada. U.S. Geological Survey Open-File Report 81-915. https://www.osti.gov/servlets/purl/5119283-5mJ8YB/ (1981).

[1217] Welder, G. E. Geohydrologic framework of the Roswell ground-water basin, Chaves and Eddy Counties, New Mexico. New Mexico State Engineer Technical Report 42. https://www.ose.state.nm.us/Library/TechnicalReports/TechReport-042.pdf (1983).

[1218] Welder, G. E. Plan of study for the regional aquifer system analysis of the San Juan structural basin, New Mexico, Colorado, Arizona, and Utah. U.S. Geological Survey Water-Resources Investigations Report 85-4294. https://pubs.usgs.gov/wri/1985/4294/report.pdf (1986).

[1219] Wellman, T. P. Evaluation of groundwater levels in the South Platte River alluvial aquifer, Colorado, 1953–2012, and design of initial well networks for monitoring groundwater levels. U.S. Geological Survey Scientific Investigations Report 2015-5015. https://pubs.usgs.gov/sir/2015/5015/pdf/sir2015-5015.pdf (2015).

[1220] Welsh, W. D. Spatial and temporal water balance estimates using a GIS. Engineers Australia. https://openresearch-repository.anu.edu.au/bitstream/1885/43108/2/HYDRO2005_bowen2.pdf (2005).

[1221] Westjohn, D. B. & Weaver, T. L. Hydrogeologic framework of the Michigan Basin regional aquifer system. U.S. Geological Survey Professional Paper 1418. https://pubs.usgs.gov/pp/1418/report.pdf (1998).

[1222] Whitcomb, H. A. & Lowry, M. E. Ground-water resources and geology of the Wind River Basin area, central Wyoming. U.S. Geological Survey Hydrologic Atlas 270. https://pubs.usgs.gov/ha/270/report.pdf (1968).

[1223] White, P. A. & Reeves, R. R. The volume of groundwater in New Zealand 1994 to 2001. Statistics New Zealand, Client Report 2002/79. https://docs.niwa.co.nz/library/public/volume-of-groundwater-in-nz-2001%5B1%5D.pdf (2002).

[1224] White, W. N. Preliminary report on the ground-water supply of Mimbres Valley, New Mexico. U.S. Geological Survey Water Supply Paper 637. https://pubs.usgs.gov/wsp/0637B/report.pdf (1931).

[1225] Whitehead, E. J. & Lawrence, A. R. The Chalk aquifer of Lincolnshire. British Geological Survey Research Report RR/06/03. http://nora.nerc.ac.uk/id/eprint/3699/1/RR06003.pdf (2006).

[1226] Whitehead, R. L. Geohydrologic framework of the Snake River Plain regional aquifer system, Idaho and eastern Oregon. U.S. Geological Survey Professional Paper 1408-B. https://pubs.usgs.gov/pp/1408b/report.pdf (1992).

[1227] Whittlemore, D. O., Macfarlane, P. A. & Wilson, B. B. Water Resources of the Dakota Aquifer in Kansas. Kansas Geological Survey Bulletin 260. http://www.kgs.ku.edu/Publications/Bulletins/260/Bulletin_260_Dakota.pdf (2014).

[1228] Wildermuth Environmental. Chino Basin Optimum Basin Management Program. State of the Basin Report – 2004. Report prepared for Chino Basin Watermaster. http://www.cbwm.org/docs/engdocs/isob/ISOB_Final_FullVersion.pdf (2005).

[1229] Wilkes, P. Baseline assessment of groundwater characteristics in the Beetaloo Sub-basin, NT. GISERA Project Order. https://gisera.csiro.au/wp-content/uploads/2018/10/Water-16-Project-Order-1.pdf (2018).

[1230] Williams, L. J. & Kuniansky, E. L. Revised hydrogeologic framework of the Floridan aquifer system in Florida and parts of Georgia, Alabama, and South Carolina. U.S. Geological Survey Professional Paper 1807. https://pubs.usgs.gov/pp/1807/pdf/pp1807.pdf (2016).

[1231] Willmes M (2018). Mapping of bioavailable strontium isotope ratios in France for archaeological provenance studies. Appl. Geochem..

[1232] Wilson DD (1973). The significance of geology in some current water resource problems, Canterbury Plains, New Zealand. J. Hydrol. (New Zeal.).

[1233] Wilson, H. D. Ground-water appraisal of Santa Ynez River basin, Santa Barbara County, California, 1945-52. U.S. Geological Survey Water-Supply Paper 1467. https://pubs.usgs.gov/wsp/1467/report.pdf (1959).

[1234] Wilson JE, Brown S, Schreier H, Scovill D, Zubel M (2008). Arsenic in groundwater wells in Quaternary deposits in the Lower Fraser Valley of British Columbia. Can. Water Resour. J..

[1235] Wilson, J. T. Water-quality assessment of the Cambrian-Ordovician aquifer system in the northern Midwest, United States. U.S. Geological Survey Scientific Investigations Report 2011-5229. https://pubs.usgs.gov/sir/2011/5229/pdf/SIR20115229_web.pdf (2012).

[1236] Winner Jr, M. D. & Coble, R. W. Hydrogeologic framework of the North Carolina Coastal Plain aquifer system. U.S. Geological Survey Open-File Report 87-690. https://pubs.usgs.gov/of/1987/0690/report.pdf (1989).

[1237] Wolfgang, C. Hydrogeology of the Pilliga sandstone aquifer in the Western Coonamble embayment and its implications for water resource management. PhD thesis, Australia National Univ. (2000).

[1238] Wood, P. R. Geology and ground-water features of the Butte Valley region, Siskiyou County, California. U.S. Geological Survey Water-Supply Paper 1491. https://pubs.usgs.gov/wsp/1491/report.pdf (1960).

[1239] Wood, P. R. & Davis, G. H. Ground-water conditions in the Avenal-McKittrick Area Kings and Kern Counties California. U.S. Geological Survey Water-Supply Paper 1457. https://pubs.usgs.gov/wsp/1457/report.pdf (1959).

[1240] Woodman ND, Burgess WG, Ahmed KM, Zahid A (2019). A partially coupled hydro-mechanical analysis of the Bengal Aquifer System under hydrological loading. Hydrol. Earth Syst. Sci..

[1241] Woodward, D. G., Gannett, M. W. & Vaccaro, J. J. Hydrogeologic framework of the Willamette Lowland aquifer system, Oregon and Washington. U.S. Geological Survey Professional Paper 1424-B. https://pubs.usgs.gov/pp/1424b/report.pdf (1998).

[1242] Woolfenden, L. R. & Nishikawa, T. Simulation of groundwater and surface-water resources of the Santa Rosa Plain watershed, Sonoma County, California. U.S. Geological Survey Scientific Investigations Report 2014-5052. https://pubs.usgs.gov/sir/2014/5052/pdf/sir2014-5052.pdf (2014).

[1243] Worts, G. F. & Thomasson, H. G. Geology and ground-water resources of the Santa Maria Valley area, California. U.S. Geological Survey Water-Supply Paper 1000. https://pubs.usgs.gov/wsp/1000/report.pdf (1951).

[1244] Wright, P. R. Hydrogeology and water quality in the Snake River alluvial aquifer at Jackson Hole Airport, Jackson, Wyoming, water years 2011 and 2012. U.S. Geological Survey Scientific Investigations Report 2013-5184. https://pubs.usgs.gov/sir/2013/5184/pdf/sir2013-5184.pdf (2013).

[1245] Wurl J, Imaz-Lamadrid MA (2018). Coupled surface water and groundwater model to design managed aquifer recharge for the valley of Santo Domingo, BCS, Mexico. Sustain. Water Resour. Manag..

[1246] Xiao Y (2021). Hydrogeochemical constraints on groundwater resource sustainable development in the arid Golmud alluvial fan plain on Tibetan plateau. Environ. Earth Sci..

[1247] Xu N, Gong J, Yang G (2018). Using environmental isotopes along with major hydro-geochemical compositions to assess deep groundwater formation and evolution in eastern coastal China. J. Contam. Hydrol..

[1248] Xu YS, Shen SL, Ma L, Sun WJ, Yin ZY (2014). Evaluation of the blocking effect of retaining walls on groundwater seepage in aquifers with different insertion depths. Eng. Geol..

[1249] Xue Z, Du P, Li J, Su H (2017). Sparse graph regularization for robust crop mapping using hyperspectral remotely sensed imagery with very few in situ data. ISPRS J. Photogramm. Remote Sens..

[1250] Yamamoto S (1951). The groundwater hydrology of river valley (2) on the groundwater of Kinokawa valley. Geogr. Rev. Jpn..

[1251] Yang WQ, Shen L, Xiao H, Wang YZ (2013). Impact of shallow groundwater quality evolution in Kunming Urban by human activities. Adv. Mater. Res..

[1252] Yangouliba GI (2022). Modelling past and future land use and land cover dynamics in the Nakambe River Basin, West Africa. Model. Earth Syst. Environ..

[1253] Yazdi, Z. & Niroumand, H. Assessing land subsidence in Qazvin plain caused by groundwater level drop, using finite elements and finite difference methods. GeoTerrace-2020-043. https://eage.in.ua/wp-content/uploads/2020/12/GeoTerrace-2020-043.pdf (2020).

[1254] Yeh HF (2021). Spatiotemporal variation of the meteorological and groundwater droughts in central Taiwan. Front. Water.

[1255] Yeh HF, Lin HI, Lee CH, Hsu KC, Wu CS (2014). Identifying seasonal groundwater recharge using environmental stable isotopes. Water.

[1256] Yoneda M (2001). Groundwater deterioration caused by induced recharge: field survey and verification of the deterioration mechanism by stochastic numerical simulation. Water Air Soil Pollut..

[1257] Yonesi H (2020). Evaluating groundwater quality in Zayandehrood southern sub-basin aquifers. Desert Ecosyst. Eng. J..

[1258] Yoosefdoo I, Khashei Siuki A (2018). Determine the vulnerability of the aquifer using the standard drastic and data-based methods (case study: Kochisfahan Aquifer). Iran. J. Remote Sens. GIS.

[1259] Yoshioka Y (2020). Multiple‐indicator study of the response of groundwater recharge sources to highly turbid river water after a landslide in the Tedori River alluvial fan, Japan. Hydrol. Process..

[1260] Yoshioka Y, Yoshioka H (2022). Spatiotemporal variability of hydrogen stable isotopes at a local scale in shallow groundwater during the warm season in Tottori Prefecture, Japan. Hydrol. Res. Lett..

[1261] Young, H. L. Hydrogeology of the Cambrian-Ordovician aquifer system in the northern Midwest, United States. U.S. Geological Survey Professional Paper 1405-B. https://pubs.usgs.gov/pp/1405b/report.pdf (1992).

[1262] Young, H. W. Reconnaissance of ground-water resources in the Mountain Home plateau area, southwest Idaho. U.S. Geological Survey Water-Resources Investigations Report 77-108. https://pubs.usgs.gov/wri/1977/0108/report.pdf (1977).

[1263] Young, R. A. & Carpenter, C. H. Ground-water conditions and storage in the Central Sevier Valley, Utah. U.S. Geological Survey Water-Supply Paper 1787. https://pubs.usgs.gov/wsp/1787/report.pdf (1965).

[1264] Yu HL, Chu HJ (2012). Recharge signal identification based on groundwater level observations. Environ. Monit. Assess..

[1265] Yu HL, Chu HJ (2010). Understanding space–time patterns of groundwater system by empirical orthogonal functions: a case study in the Choshui River alluvial fan, Taiwan. J. Hydrol..

[1266] Yustres Á, Navarro V, Asensio L, Candel M, García B (2013). Groundwater resources in the Upper Guadiana Basin (Spain): a regional modelling analysis. Hydrol. J..

[1267] Zandi R, Ghahraman K, Asadi Z (2019). Monitoring the land subsidence and its associated landforms using remote sensing techniques in Feyzabad Plain (north-east Iran). J. Hydrosci. Environ..

[1268] Zare M, Koch M (2017). Computation of the irrigation water demand in the Miandarband plain, Iran, using FAO-56-and satellite-estimated crop coefficients. Interdiscip. Res. Rev..

[1269] Zarour, H., Aitchison-Earl, P., Scott, M., Peaver, L. & De Silva, J. Current state of the groundwater resource in the Orari-Temuka-Opihi-Pareora area. Environment Canterbury Regional Council Report No. R16/41. https://api.ecan.govt.nz/TrimPublicAPI/documents/download/2964277 (2018).

[1270] Zaryab A, Nassery HR, Alijani F (2021). Identifying sources of groundwater salinity and major hydrogeochemical processes in the Lower Kabul Basin aquifer, Afghanistan. Environ. Sci. Process. Impacts.

[1271] Zeng Y, Zhou Y, Zhou J, Jia R, Wu J (2018). Distribution and enrichment factors of high-arsenic groundwater in Inland Arid area of PR China: a case study of the Shihezi area, Xinjiang. Expos. Health.

[1272] Zhang B (2017). The renewability and quality of shallow groundwater in Sanjiang and Songnen Plain, Northeast China. J. Integr. Agric..

[1273] Zhang G, Deng W, Yang YS, Salama RB (2007). Evolution study of a regional groundwater system using hydrochemistry and stable isotopes in Songnen Plain, northeast China. Hydrol. Process..

[1274] Zhang H, Xu Y, Cheng S, Li Q, Yu H (2020). Application of the dual-isotope approach and Bayesian isotope mixing model to identify nitrate in groundwater of a multiple land-use area in Chengdu Plain, China. Sci. Total Environ..

[1275] Zhang H, Yang R, Wang Y, Ye R (2019). The evaluation and prediction of agriculture-related nitrate contamination in groundwater in Chengdu Plain, southwestern China. Hydrol. J..

[1276] Zhang, L., Stauffacher, M., Walker, G. R. & Dyce, P. Recharge estimation in the Liverpool Plains (NSW) for input groundwater models. CSIRO Technical Report 10/97 (1997).

[1277] Zhang Q (2012). Predicting the risk of arsenic contaminated groundwater in Shanxi Province, Northern China. Environ. Pollut..

[1278] Zhang W (2019). Using noble gases to trace groundwater evolution and assess helium accumulation in Weihe Basin, central China. Geochim. Cosmochim. Acta.

[1279] Zhang Y, Gable CW, Zyvoloski GA, Walter LM (2009). Hydrogeochemistry and gas compositions of the Uinta Basin: A regional-scale overview. AAPG Bull..

[1280] Zhang Y (2015). Land subsidence and uplift due to long-term groundwater extraction and artificial recharge in Shanghai, China. Hydrol. J..

[1281] Zhen, L. & Martin, P. Geohydrology, simulation of regional groundwater flow, and assessment of water-management strategies, Twentynine Palms area, California. U.S. Geological Survey Scientific Investigations Report 2010-5249. https://pubs.usgs.gov/sir/2010/5249/pdf/sir20105249.pdf (2011).

[1282] Zhong Y (2018). Groundwater depletion in the West Liaohe River Basin, China and its implications revealed by GRACE and in situ measurements. Remote Sens..

[1283] Zhou J, Hu BX, Cheng G, Wang G, Li X (2011). Development of a three‐dimensional watershed modelling system for water cycle in the middle part of the Heihe rivershed, in the west of China. Hydrol. Process..

[1284] Zhou Y, Wang Y, Li Y, Zwahlen F, Boillat J (2013). Hydrogeochemical characteristics of central Jianghan Plain, China. Environ. Earth Sci..

[1285] Zhou Z, Zhong J (2022). Role of atmospheric temperature and seismic activity in spring water hydrogeochemistry in Urumqi, China. Int. J. Environ. Res. Public Health.

[1286] Zhu GF, Li ZZ, Su YH, Ma JZ, Zhang YY (2007). Hydrogeochemical and isotope evidence of groundwater evolution and recharge in Minqin Basin, Northwest China. J. Hydrol..

[1287] Zulfic, D., Harrington, N. & Evans, S. Uley Basin groundwater modelling project, volume 2: groundwater flow model. DWLBC Report 2007/04, Department of Water, Land and Biodiversity Conservation. https://www.waterconnect.sa.gov.au/Content/Publications/DEW/ki_dwlbc_report_2007_04.pdf (2006).

[1288] GebreEgziabher M, Jasechko S, Perrone D (2022). Widespread and increased drilling of wells into fossil aquifers in the USA. Nat. Commun..

[1289] Taher, M. R., Chornack, M. P. & Mack, T. J. Groundwater levels in the Kabul Basin, Afghanistan, 2004–2013. U.S. Geological Survey Open-File Report 2013-1296. 10.3133/ofr20131296 (2014).

[1290] Gong H (2018). Long-term groundwater storage changes and land subsidence development in the North China Plain (1971–2015). Hydrol. J..

[1291] Winckel A, Ollagnier S, Gabillard S (2022). Managing groundwater resources using a national reference database: the French ADES concept. SN Appl. Sci..

[1292] Ascott MJ (2020). In situ observations and lumped parameter model reconstructions reveal intra‐annual to multidecadal variability in groundwater levels in sub‐Saharan Africa. Water Resour. Res..

[1293] Tao S (2020). Changes in China’s water resources in the early 21st century. Front. Ecol. Environ..

[1294] Adamson JK (2022). Significance of river infiltration to the Port-Au-Prince metropolitan region: a case study of two alluvial aquifers in Haiti. Hydrol. J..

[1295] Vongphachanh S, Gupta AD, Milne-Home W, Ball JE, Pavelic P (2017). Hydrogeological reconnaissance of Sukhuma District, Champasak Province, Southern Laos. J. Hydrol. (New Zeal.).

[1296] Fallatah OA (2020). Groundwater quality patterns and spatiotemporal change in depletion in the regions of the Arabian shield and Arabian shelf. Arab. J. Sci. Eng..

[1297] Hsu YJ (2020). Assessing seasonal and interannual water storage variations in Taiwan using geodetic and hydrological data. Earth Planet. Sci. Lett..

[1298] Taylor SJ, Letham B (2018). Forecasting at scale. Am. Stat..

[1299] Friedman JH, Stuetzle W (1981). Projection pursuit regression. J. Am. Stat. Assoc..

[1300] Theil H (1950). A rank-invariant method of linear and polynomial regression analysis. Indag. Math..

[1301] Sen PK (1968). Estimates of the regression coefficient based on Kendall’s tau. J. Am. Stat. Assoc..

[1302] Holland PW, Welsch RE (1977). Robust regression using iteratively reweighted least-squares. Commun. Stat. Theory Methods.

[1303] Kirchner JW (2019). Quantifying new water fractions and transit time distributions using ensemble hydrograph separation: theory and benchmark test. Hydrol. Earth Syst. Sci..

[1304] Kirchner JW, Knapp JLA (2020). Technical note: Calculation scripts for ensemble hydrograph separation. Hydrol. Earth Syst. Sci..

[1305] Fisher M, Bolles R (1981). Random sample consensus: a paradigm for model fitting with applications to image analysis and automated cartography. Commun. ACM.

[1306] Önöz B, Bayazit M (2012). Block bootstrap for Mann–Kendall trend test of serially dependent data. Hydrol. Process..

[1307] Shamsudduha M, Taylor RG (2020). Groundwater storage dynamics in the world’s large aquifer systems from GRACE: uncertainty and role of extreme precipitation. Earth Syst. Dyn..

[1308] Landerer FW, Swenson SC (2012). Accuracy of scaled GRACE terrestrial water storage estimates. Water Resour. Res..

[1309] Watkins MM, Wiese DN, Yuan D-N, Boening C, Landerer FW (2015). Improved methods for observing Earth’s time variable mass distribution with GRACE using spherical cap mascons. J. Geophys. Res. Solid Earth.

[1310] Wiese DN, Landerer FW, Watkins MM (2016). Quantifying and reducing leakage errors in the JPL RL05M GRACE mascon solution. Water Resour. Res..

[1311] Biancale, R. et al. 3 Years of Geoid Variations from GRACE and LAGEOS Data at 10-day Intervals from July 2002 to March 2005. CNES/GRGS data product (2006).

[1312] de Graaf ID, Sutanudjaja EH, Van Beek LPH, Bierkens MFP (2015). A high-resolution global-scale groundwater model. Hydrol. Earth Syst. Sci..

[1313] Duran-Llacer I (2020). Lessons to be learned: groundwater depletion in Chile’s Ligua and Petorca watersheds through an Interdisciplinary approach. Water.

[1314] Narvaez-Montoya C (2022). Predicting adverse scenarios for a transboundary coastal aquifer system in the Atacama Desert (Peru/Chile). Sci. Total Environ..

[1315] Oiro S, Comte JC, Soulsby C, MacDonald A, Mwakamba C (2020). Depletion of groundwater resources under rapid urbanisation in Africa: recent and future trends in the Nairobi Aquifer System, Kenya. Hydrol. J..

[1316] Castellazzi P, Garfias J, Martel R (2021). Assessing the efficiency of mitigation measures to reduce groundwater depletion and related land subsidence in Querétaro (Central Mexico) from decadal InSAR observations. Int. J. Appl. Earth Obs. Geoinf..

[1317] Nguyen M (2022). Assessment of long-term ground subsidence and groundwater depletion in Hanoi, Vietnam. Eng. Geol..

[1318] Bui LK (2021). Recent land deformation detected by Sentinel-1A InSAR data (2016–2020) over Hanoi, Vietnam, and the relationship with groundwater level change. GISci. Remote Sens..

[1319] Moshfika M, Biswas S, Mondal MS (2022). Assessing groundwater level declination in Dhaka city and identifying adaptation options for sustainable water supply. Sustainability.

[1320] Sohail MT (2022). Groundwater budgeting of Nari and Gaj formations and groundwater mapping of Karachi, Pakistan. Appl. Water Sci..

[1321] Dehghani F, Mohammadi Z, Zare M (2021). Assessment of groundwater depletion in a heterogeneous aquifer: historical reconnaissance and current situation. Environ. Earth Sci..

[1322] Gautam A, Rai SC, Rai SP (2020). Impact of anthropogenic activities on the alluvial aquifers of north-east Punjab, India. Environ. Monit. Assess..

[1323] Sajjad MM (2022). Impact of climate and land-use change on groundwater resources, study of Faisalabad district, Pakistan. Atmosphere.

[1324] Ouassanouan Y (2022). Multi-decadal analysis of water resources and agricultural change in a Mediterranean semiarid irrigated piedmont under water scarcity and human interaction. Sci. Total Environ..

[1325] Goode, D. J., Senior, L. A., Subah, A. & Jaber, A. Groundwater-level trends and forecasts, and salinity trends, in the Azraq, Dead Sea, Hammad, Jordan Side Valleys, Yarmouk, and Zarqa groundwater basins, Jordan. U.S. Geological Survey Open-File Report 2013-1061. http://pubs.usgs.gov/of/2013/1061/ (2013).

[1326] Naeem UA (2021). Impact of urbanization on groundwater levels in Rawalpindi City, Pakistan. Pure Appl. Geophys..

[1327] Snoussi M, Jerbi H, Tarhouni J (2022). Integrated groundwater flow modeling for managing a complex alluvial aquifer case of study Mio-Plio-Quaternary Plain of Kairouan (Central Tunisia). Water.

[1328] Zghibi A (2019). Implications of groundwater development and seawater intrusion for sustainability of a Mediterranean coastal aquifer in Tunisia. Environ. Monit. Assess..

[1329] Cotterman KA, Kendall AD, Basso B, Hyndman DW (2018). Groundwater depletion and climate change: future prospects of crop production in the Central High Plains Aquifer. Clim. Change.

[1330] Orhan O (2021). Monitoring of land subsidence due to excessive groundwater extraction using small baseline subset technique in Konya, Turkey. Environ. Monit. Assess..

[1331] Xia J (2019). Evaluating the dynamics of groundwater depletion for an arid land in the Tarim Basin, China. Water.

[1332] Custodio E (2017). Groundwater mining: benefits, problems and consequences in Spain. Sustain. Water Resour. Manag..

[1333] Taher TM (2016). Groundwater abstraction management in Sana’a Basin, Yemen: a local community approach. Hydrol. J..

[1334] Delinom, R. M. in *Groundwater and Subsurface Environments* (ed. Taniguchi, M.) 113–125 (Springer, 2011).

[1335] Taufiq A (2018). Impact of excessive groundwater pumping on rejuvenation processes in the Bandung basin (Indonesia) as determined by hydrogeochemistry and modeling. Hydrol. J..

[1336] Zaryab A, Nassery HR, Alijani F (2022). The effects of urbanization on the groundwater system of the Kabul shallow aquifers, Afghanistan. Hydrol. J..

[1337] Carrillo, M., Gomez, Y. A., Valle, S. & Prado, J. V. Behavior of groundwater levels in Texcoco Aquifer (1507) when they are lowered by excessive pumping from 1968 through 2014. 2016 ASABE Annual International Meeting. American Society of Agricultural and Biological Engineers. https://elibrary.asabe.org/abstract.asp?aid=47273 (2016).

[1338] Ojha C, Werth S, Shirzaei M (2019). Groundwater loss and aquifer system compaction in San Joaquin Valley during 2012–2015 drought. J. Geophys. Res. Solid Earth.

[1339] Noori R (2021). Anthropogenic depletion of Iran’s aquifers. Proc. Natl Acad. Sci..

[1340] Ashraf S, Nazemi A, AghaKouchak A (2021). Anthropogenic drought dominates groundwater depletion in Iran. Sci. Rep..

[1341] Saowiang K, Giao PH (2021). Numerical analysis of subsurface deformation induced by groundwater level changes in the Bangkok aquifer system. Acta Geotech..

[1342] Shi W (2020). Spatial-temporal evolution of land subsidence and rebound over Xi’an in western China revealed by SBAS-InSAR analysis. Remote Sens..

[1343] Sartirana D (2022). Data-driven decision management of urban underground infrastructure through groundwater-level time-series cluster analysis: the case of Milan (Italy). Hydrol. J..

[1344] Houspanossian J (2023). Agricultural expansion raises groundwater and increases flooding in the South American plains. Science.

[1345] Galanter, A. E. & Curry, L. T. S. Estimated 2016 groundwater level and drawdown from predevelopment to 2016 in the Santa Fe Group aquifer system in the Albuquerque area, central New Mexico. U.S. Geological Survey Scientific Investigations Map 3433. 10.3133/sim3433 (2019).

[1346] Hao Y, Xie Y, Ma J, Zhang W (2017). The critical role of local policy effects in arid watershed groundwater resources sustainability: a case study in the Minqin oasis, China. Sci. Total Environ..

[1347] Furi W, Razack M, Haile T, Abiye TA, Legesse D (2011). The hydrogeology of Adama-Wonji basin and assessment of groundwater level changes in Wonji wetland, Main Ethiopian Rift: results from 2D tomography and electrical sounding methods. Environ. Earth Sci..

[1348] Özel N, Bozdağ Ş, Baba A (2023). Effect of irrigation system on groundwater resources in Harran Plain (Southeastern Turkey). J. Food Sci. Eng..

[1349] Duran-Llacer I (2022). A new method to map groundwater-dependent ecosystem zones in semi-arid environments: a case study in Chile. Sci. Total Environ..

[1350] Pino E (2019). Factors affecting depletion and pollution by marine intrusion in the La Yarada’s coastal aquifer, Tacna, Peru. Tecnol. Cienc. Agua.

[1351] Vu TT, Tran NVT (2018). Assessment of urbanization impact on groundwater resources in Hanoi, Vietnam. J. Environ. Manag..

[1352] Roy SK, Zahid A (2021). Assessment of declining groundwater levels due to excessive pumping in the Dhaka District of Bangladesh. Environ. Earth Sci..

[1353] Taher T, Bruns B, Bamaga O, Al-Weshali A, Van Steenbergen F (2012). Local groundwater governance in Yemen: building on traditions and enabling communities to craft new rules. Hydrol. J..

[1354] Rybakov, V. Water crisis in Yemen: speculations, realities and mitigation actions. https://static1.squarespace.com/static/5eb18d627d53aa0e85b60c65/t/5eda46ed1c956a6bc14ae36c/1591363321836/Report-victor.pdf (2012).

[1355] Abidin, H. Z. et al. Land subsidence and groundwater extraction in Bandung Basin, Indonesia. IAHS publication 329, 145–156 (2009).

[1356] Livoreil B (2017). Systematic searching for environmental evidence using multiple tools and sources. Environ. Evid..

[1357] Malakar P (2021). Three decades of depth-dependent groundwater response to climate variability and human regime in the transboundary Indus-Ganges-Brahmaputra-Meghna mega river basin aquifers. Adv. Water Res..

[1358] Taylor, C. J. & Alley, W. M. Ground-water-level monitoring and the importance of long-term water-level data. U.S. Geological Survey Circular 1217 (2001). 10.1111/j.1745-6584.2001.tb02466.x 11708445

[1359] Russo TA, Lall U (2017). Depletion and response of deep groundwater to climate-induced pumping variability. Nat. Geosci..

[1360] Hartmann J, Moosdorf N (2012). The new global lithological map database GLiM: a representation of rock properties at the Earth surface. Geochem. Geophys. Geosyst..

[1361] Hora T, Srinivasan V, Basu NB (2019). The groundwater recovery paradox in South India. Geophys. Res. Lett..

[1362] Patle GT (2015). Time series analysis of groundwater levels and projection of future trend. J. Geol. Soc. India.

[1363] Shamsudduha M, Taylor RG, Ahmed KM, Zahid A (2011). The impact of intensive groundwater abstraction on recharge to a shallow regional aquifer system: evidence from Bangladesh. Hydrol. J..

[1364] Rushton KR, Zaman MA, Mehedi Hasan M (2023). Sustainable abstraction due to unconfined conditions in multi-layered aquifers: examples from northwest Bangladesh. Groundw. Sustain. Dev..

[1365] MacDonald AM (2016). Groundwater quality and depletion in the Indo-Gangetic Basin mapped from in situ observations. Nat. Geosci..

[1366] MacAllister DJ, Krishan G, Basharat M, Cuba D, MacDonald AM (2022). A century of groundwater accumulation in Pakistan and northwest India. Nat. Geosci..

[1367] Perrone D, Jasechko S (2017). Dry groundwater wells in the western United States. Environ. Res. Lett..

[1368] Perrone D, Jasechko S (2019). Deeper well drilling an unsustainable stopgap to groundwater depletion. Nat. Sustain..

[1369] Jasechko S, Perrone D (2017). Hydraulic fracturing near domestic groundwater wells. Proc. Natl Acad. Sci..

[1370] Mukherji A, Rawat S, Shah T (2013). Major insights from India’s minor irrigation censuses: 1986-87 to 2006-07. Econ. Political Wkly..

[1371] Laghari AN, Vanham D, Rauch W (2012). The Indus basin in the framework of current and future water resources management. Hydrol. Earth Syst. Sci..

[1372] Abatzoglou JT, Dobrowski SZ, Parks SA, Hegewisch KC (2018). TerraClimate, a high-resolution global dataset of monthly climate and climatic water balance from 1958–2015. Sci. Data.

[1373] Karger DN, Wilson AM, Mahony C, Zimmermann NE (2021). Global daily 1 km land surface precipitation based on cloud cover-informed downscaling. Sci. Data.

