# Supplementary material for: Rapid groundwater decline and some cases of recovery in aquifers globally
Source: Nature. 2024 Jan 24;625(7996):715–21. doi: 10.1038/s41586-023-06879-8 (PMC10808077; doi:10.1038/s41586-023-06879-8)
Supplement: Supplementary file 1 — Supplementary Information [file 41586_2023_6879_MOESM1_ESM.pdf]

nature portfolio

https://doi.org/10.1038/s41586-023-06879-8

Supplementary information

Rapid groundwater decline and some cases of recovery in aquifers globally

In the format provided by the authors and unedited

Supplementary Information

Rapid groundwater decline and some cases of recovery in aquifers globally

Scott Jasechko [1], Hansjörg Seybold [2], Debra Perrone [3], Ying Fan [4], Mohammad Shamsudduha [5], Richard G. Taylor [6], Othman Fallatah [7,8], James W. Kirchner [2,9,10]

[1] Bren School of Environmental Science & Management, University of California, Santa Barbara, California, 93106, USA
[2] Department of Environmental System Sciences, ETH Zürich, Universitätstrasse 16, CH-8092, Switzerland
[3] Environmental Studies Program, University of California, Santa Barbara, California, 93106, USA
[4] Department of Earth and Planetary Sciences, Rutgers University, New Brunswick, New Jersey, 08854, USA
[5] Institute for Risk and Disaster Reduction, University College London, London, WC1E 6BT, UK
[6] Department of Geography, University College London, London, WC1E 6BT, UK
[7] Department of Nuclear Engineering, Faculty of Engineering, King Abdulaziz University, P.O. Box 80204, Jeddah 21589, Saudi Arabia
[8] Centre for Training & Radiation Protection, Faculty of Engineering, King Abdulaziz University, P.O. Box 80204, Jeddah 21589, Saudi Arabia
[9] Swiss Federal Research Institute WSL, Birmensdorf, CH-8903, Switzerland
[10] Department of Earth and Planetary Science, University of California, Berkeley, California, 94720, USA

Table of contents

1 Groundwater level data sources 4
1.1 Afghanistan 19
1.2 Argentina 21
1.3 Australia 22
1.4 Austria 23
1.5 Bangladesh 24
1.6 Belgium: Brussels 25
1.7 Belgium: Wallonia 26
1.8 Bulgaria 27
1.9 Burkina Faso 28
1.10 Canada: Ontario 29
1.11 China: Gong et al. (2018) 30
1.12 China: Tao et al. (2020) 31
1.13 China: CIGEM 32
1.14 Colombia 33
1.15 Croatia 34
1.16 Czech Republic 35
1.17 Denmark 36
1.18 El Salvador 37
1.19 France and French Guiana 38
1.20 Haiti 39
1.21 Israel 40
1.22 Japan 41
1.23 Laos 42
1.24 Latvia 43
1.25 Lithuania 44
1.26 Morocco: Souss-Massa 45
1.27 Paraguay 48
1.28 Poland 49
1.29 Saudi Arabia 50
1.30 Slovenia 51
1.31 Switzerland 52
1.32 Taiwan 53
1.33 Thailand 54
1.34 United States of America: NWIS 55
1.35 United States of America: TWDB 59
1.36 United States of America: California DWR 64
1.37 United States of America: California GAMA 67
1.38 Vietnam 69
2 Groundwater level data pre-processing 70
2.1 Excluding potentially erroneous well water level measurements 71
2.2 Calculating annual median groundwater levels 76
2.3 Identifying records capturing 21st-century groundwater level trends 77
3 Different regression approaches yield similar groundwater level trends 79
4 Non-parametric regression approaches applied to groundwater level time series 84
5 Similar aquifer-scale trends obtained with and without flagged measurements 88
6 Sensitivity analysis of aquifer-scale trends to minimum length of time-series 90
7 Delineating aquifer system boundaries 95
8 21st century groundwater storage trends from GRACE satellites 603
9 21st century groundwater level trends compared to 1980-2000 groundwater level trends 611
10 Precipitation variability over four decades in aquifer systems 619
11 Rapid groundwater level deepening most common in cultivated areas and in drier climates 633
12 Spatial variations in monitoring well time series durations and measurement frequencies 638

13 Regression analyses if we include outliers identified by the machine learning algorithm..........643
14 Simulated groundwater depletion from a hydrologic model 645
15 Literature review of studies capturing groundwater trends via monitoring wells 647
15.1 Literature review of groundwater level deepening 647
15.2 Literature review of groundwater level shallowing 662
16 Spatial variability in groundwater level trends for individual sub-continents and continents..669
17 Spatial variability in groundwater level trends within individual aquifer systems 686
18 Hotspots of groundwater level deepening within some aquifer systems 895
18.1 Proportion of monitoring wells within a study area exhibiting relatively rapid rates of groundwater level deepening.........898
18.2 Hotspots of relatively rapid groundwater level deepening 900
19 Comparing groundwater-level trends in unconfined and confined aquifers..............................913
19.1 Groundwater level trends in deeper versus shallower wells..914
19.2 Groundwater level trends in wells that the USGS has identified as tapping unconfined or confined storage conditions......919
20 Potential for monitoring wells to have run dry 925
21 Intra-annual groundwater level variations...930
22 Well drilling time series in aquifer systems exhibiting rapid groundwater level deepening......932
23 Clustering of groundwater-level trends within individual aquifer systems................................958
23.1 Examples of aquifer systems exhibiting clustering of trends in depth to groundwater..960
23.2 Examples of aquifer systems that do not exhibit clustering of trends in depth to groundwater 965
24 Table summarising groundwater level trends for 1,693 study aquifer systems 969

1 GROUNDWATER LEVEL DATA SOURCES

Here we describe the piezometric data that we analyse in this study. Some of the databases that we analysed in this study were downloaded specifically for this project; we describe the download process for each of these databases in the following subsections (for example, subsection 1.1 presents (i) a hyperlink to a website where piezometric data for Afghanistan can be accessed, and (ii) a description of steps that we completed before analysing these data). Other databases that we analysed in this study are described in ref.[17] (i.e., Jasechko, S., & Perrone, D. Global groundwater wells at risk of running dry. *Science* **372,** 418–421 (2021)); for these databases, we provide links to the data source (Supplementary Table 1) and reference to the specific section within this reference (i.e., within ref.[17]) where the dataset is described.

Supplementary Table 1. Piezometric datasets

	Date the data were accessed	Link(s) to data sources	Dataset quality control steps *	Written permission received to post annual ground-water level data (see footnote: [0])	Estimated groundwater withdrawals (km^3/year) [Z]
**Afghanistan** [W]	**Dec-17-2020** [W]	**https://pubs.usgs.gov/of/2013/1296/** [W]	**Section 1.1 (i.e., below in this file)** [W]	**Yes** [W]	**7.12**
**Argentina**	**Jan-20-2023**	**http://datar.info/pt_BR/dataset/pozos-sifas**	**Section 1.2 (i.e., below in this file)**		**6.66**
**Australia**	**Dec-09-2021**	**http://www.bom.gov.au/water/groundwater/explorer/map.shtml**	**Section 1.3 (i.e., below in this file)**		**4.96**
**Austria**	**Apr-20-2023**	**https://www.eea.europa.eu/en/datahub/datahubitem-view/a4324714-784a-4763-b8b9-b8939ead87fe**	**Section 1.4 (i.e., below in this file)**	**Yes** [E]	**1.12**
**Bangladesh** [U]	**Jun-21-2021**	**http://www.hydrology.bwdb.gov.bd/index.php?pagetitle=ground_water-_hydrology&sub2=170&_subid=79&id=165**	**Section 1.5 (i.e., below in this file)**		**30.21**
**Belgium: Brussels**	**Aug-19-2019**	**https://geodata.environnement.brussels/client/view/b4231044-be68-4a1a-bf98-9e5397bb5324**	**Section 1.6 (i.e., below in this file)**	**Yes** [x]	**0.65** *(note: 0.65 km^3/year is the estimate for Belgium)*
**Belgium: Wallonia**	**Aug-02-2021**	**http://piezo.environnement.wallonie.be/login.do?time**	**Section 1.7 (i.e., below in this file)**	**Yes** [y]	**0.65** *(note: 0.65 km^3/year is the estimate for Belgium)*

	Date the data were accessed	Link(s) to data sources	Dataset quality control steps *	Written permission received to post annual ground-water level data (see footnote: [0])	Estimated groundwater withdrawals (km^3/year) [Z]
		**=1566500026975#**			
Brazil	Jul-30-2019	http://rimasweb.cprm.gov.br/layout/pesquisa_complexa.php?rimas=true	Supplementary Materials section S2.3 within Jasechko, S., Perrone, D. (2021) Science 372, 418–421	Yes [†]	10.06
**Bulgaria**	**Apr-20-2023**	**https://www.eea.europa.eu/en/datahub/datahubitem-view/a4324714-784a-4763-b8b9-b8939ead87fe**	**Section 1.8 (i.e., below in this file)**	**Yes [E]**	**0.58**
**Burkina Faso**	**Oct-20-2021**	**https://agupubs.onlinelibrary.wiley.com/doi/full/10.1029/2020WR028056**	**Section 1.9 (i.e., below in this file)**		**0.39**
Canada: Alberta [****]	Jul-02-2019	http://environment.alberta.ca/apps/GOWN/#	Supplementary Materials section S2.4 within Jasechko, S., Perrone, D. (2021) Science 372, 418–421	Yes [****]	1.87 *(note: 1.87 km^3/year is the estimate for all of Canada)*
Canada: British Columbia	Jul-09-2019	https://catalogue.data.gov.bc.ca/dataset/57c55f10-cf8e-40bb-aae0-2eff311f1685	Supplementary Materials section S2.5 within Jasechko, S., Perrone, D. (2021) Science 372, 418–421	Yes	1.87 *(note: 1.87 km^3/year is the estimate for all of Canada)*
Canada: Manitoba [zz]	Jan-06-2020	https://www.gov.mb.ca/iem/	Supplementary Materials section S2.6 within Jasechko, S., Perrone, D. (2021) Science 372, 418–421	Yes	1.87 *(note: 1.87 km^3/year is the estimate for all of Canada)*
Canada: New Brunswick [U]	Sep-24-2019	https://www2.gnb.ca/content/gnb/en/departments/elg.html	Supplementary Materials section S2.7 within Jasechko, S.,		1.87 *(note: 1.87 km^3/year is the estimate for all of Canada)*

	**Date the data were accessed**	**Link(s) to data sources**	**Dataset quality control steps ***	**Written permission received to post annual ground-water level data (see footnote: [0])**	**Estimated groundwater withdrawals (km^3/year) [z]**
			Perrone, D. (2021) Science 372, 418–421		
Canada: Northwest Territories	Nov-07-2019	https://www.maca.gov.nt.ca/en	Supplementary Materials section S2.9 within Jasechko, S., Perrone, D. (2021) Science 372, 418–421	Yes	1.87 *(note: 1.87 km^3/year is the estimate for all of Canada)*
Canada: Nova Scotia	Jul-02-2019	https://novascotia.ca/nse/groundwater/groundwaternetworkwells.asp	Supplementary Materials section S2.10 within Jasechko, S., Perrone, D. (2021) Science 372, 418–421		1.87 *(note: 1.87 km^3/year is the estimate for all of Canada)*
**Canada: Ontario**	**Jul-20-2021**	**https://www.ontario.ca/data/provincial-groundwater-monitoring-network**	**Section 1.10 (i.e., below in this file)**	**Yes**	**1.87** *(note: 1.87 km^3/year is the estimate for all of Canada)*
Canada: Prince Edward Island	Jul-03-2019	http://www.gov.pe.ca/groundwater/app.php?lang=E **	Supplementary Materials section S2.12 within Jasechko, S., Perrone, D. (2021) Science 372, 418–421	Yes ‡	1.87 *(note: 1.87 km^3/year is the estimate for all of Canada)*

	Date the data were accessed	Link(s) to data sources	Dataset quality control steps *	Written permission received to post annual ground-water level data (see footnote: [0])	Estimated groundwater withdrawals (km^3/year) [z]
Canada: Quebec [U]	Sep-17-2019	https://www.environnement.gouv.qc.ca/ministere/inter_en.htm	Supplementary Materials section S2.13 within Jasechko, S., Perrone, D. (2021) Science 372, 418–421		1.87 *(note: 1.87 km^3/year is the estimate for all of Canada)*
Canada: Saskatchewan [§]	Jul-10-2019 [§]	https://www.wsask.ca/water-info/ground-water/ (data provided to us via email) [§]	Supplementary Materials section S2.14 within Jasechko, S., Perrone, D. (2021) Science 372, 418–421	Yes	1.87 *(note: 1.87 km^3/year is the estimate for all of Canada)*
Canada: Yukon***	Aug-06-2019	https://yukon.ca/en/get-information-about-yukon-groundwater-and-wells (data source: Water Resources Branch)	Supplementary Materials section S2.15 within Jasechko, S., Perrone, D. (2021) Science 372, 418–421	Yes	1.87 *(note: 1.87 km^3/year is the estimate for all of Canada)*
Chile	Jan-28-2020	https://dga.mop.gob.cl/Paginas/default.aspx	Supplementary Materials section S2.16 within Jasechko, S., Perrone, D. (2021) Science 372, 418–421		0.98

	Date the data were accessed	Link(s) to data sources	Dataset quality control steps *	Written permission received to post annual ground-water level data (see footnote: [0])	Estimated groundwater withdrawals (km^3/year) [Z]
**China: Gong et al. (2018)**	**Jan-06-2021**	**https://link.springer.com/article/10.1007/s10040-018-1768-4**	**section 1.11 (i.e., below in this file)**	**Yes**	**111.95** *(note: 111.95 for all of China)*
**China: Tao et al. (2020)**	**Jan-18-2021**	**https://doi.org/10.1002/fee.2164**	**section 1.12 (i.e., below in this file)**		**111.95** *(note: 111.95 for all of China)*
**China: CIGEM**	**Sep-21-2020**	**http://jcgc.cigem.cn/share**	**section 1.13 (i.e., below in this file)**		**111.95** *(note: 111.95 for all of China)*
**Colombia [U]**	**Dec-18-2020**	**http://www.ideam.gov.co/**	**section 1.14 (i.e., below in this file)**		**1.75**
**Croatia**	**Sep-22-2020**	**voda.hr**	**section 1.15 (i.e., below in this file)**	**Yes**	**1.16**
**Czech Republic**	**Feb-08-2021**	**chmi.cz**	**Section 1.16 (i.e., see below in this file)**	**Yes**	**0.38**
**Denmark**	**Apr-20-2023**	**https://www.eea.europa.eu/en/datahub/datahubitem-view/a4324714-784a-4763-b8b9-b8939ead87fe**	**Section 1.17 (i.e., below in this file)**	**Yes [E]**	**0.65**
**El Salvador**	**Jan-22-2023**	**http://srt.snet.gob.sv/sihi/public/app/1/pozosmonitoreo**	**Section 1.18 (i.e., see below in this file)**		**0.58**
Estonia	Aug-30-2019 and Jul-11-2019	https://cr.eionet.europa.eu/sparql?query=PREFIX+rod%3A+%3Chttp%3A%2F%2Frod.eionet.europa.eu%2Fschema.rdf%23%3E%0D%0APREFIX+obl%3A+%3Chttp%3A%2F%2Frod.eionet.europa.eu%2Fobligations%2F%3E%0D	Supplementary Materials section S2.17 within Jasechko, S., Perrone, D. (2021) Science 372, 418–421		0.33

	Date the data were accessed	Link(s) to data sources	Dataset quality control steps *	Written permission received to post annual ground-water level data (see footnote: [0])	Estimated groundwater withdrawals (km^3/year) [z]
		%0ASELECT+DISTINCT+%3Fcountry%2C%3Freleased%2C%3Fdelivery++%0D%0AWHERE+%7B%3Fdelivery+rod%3Aobligation+%3Fobluri+%3B%0D%0Arod%3Areleased+%3Freleased+%3B%0D%0Arod%3Alocality+%3Flocality+.%0D%0A%3Flocality+rod%3Aloccode+%3Fcountry+.%0D%0AFILTER+%28%3Fobluri+in+%28obl%3A184%29%29%7D+%0D%0AORDER+BY+%3Fcountry+DESC%28%3Freleased%29&format=text%2Fhtml&nrOfHits=1000&execute=Execute and https://www.eea.europa.eu/data-and-maps/data/waterbase-water-quantity-11#tab-european-data			
**France and French Guiana ♠**	**Sep-30-2020 ♠**	**https://ades.eaufrance.fr/Recherche ♠**	**section 1.19 (i.e., below in this file) ♠**	**Yes ♠**	**5.72 *(note: 5.71 km³/year estimated for France and 0.01 km³/year estimated for French Guiana)***
Germany	Jan-24-2020	www.elwasweb.nrw.de	Supplementary Materials section	Yes [+]	5.83

	Date the data were accessed	Link(s) to data sources	Dataset quality control steps *	Written permission received to post annual ground-water level data (see footnote: [0])	Estimated groundwater withdrawals (km^3/year) [z]
			S2.19 within Jasechko, S., Perrone, D. (2021) Science 372, 418–421		
Guam	Sep-06-2019	https://guamhydrologicsurvey.uog.edu/index.php/data/bhdb/	Supplementary Materials section S2.20 within Jasechko, S., Perrone, D. (2021) Science 372, 418–421	Yes	0.02
**Haiti**	**Jan-19-2023**	**https://link.springer.com/article/10.1007/s10040-022-02488-3**	**section 1.20 (i.e., below in this file)**		**0.95**
India	Oct-01-2020	https://indiawris.gov.in/wris/#/groundWater [zzzzz]	Supplementary Materials section S6.1.2 within Jasechko, S., Perrone, D. (2021) Science 372, 418–421		251
Iran	Sep-22-2020	https://stu.wrm.ir/register.asp [>]	Supplementary Materials section S6.1.3 within Jasechko, S., Perrone, D. (2021) Science 372, 418–421		63.4
Ireland	Sep-04-2019 and Sep-05-2019	http://www.epa.ie/hydronet/#Groundwater and https://www.eea.europa.eu/data-and-maps	Supplementary Materials section S2.21 within Jasechko, S., Perrone, D. (2021) Science 372, 418–421	Yes	0.21
**Israel**	**Jun-17-2023**	**https://www.gov.il/he/departments/israel-geological-survey/govil-landing-page**	**section 1.21 (i.e., below in this file)**	**Yes**	**1.25**
Italy	Jul-11-2019	https://www.eea.europa.eu/data-and-maps/data/wat	Supplementary Materials section S2.22 within Jasechko, S.,	Yes [E]	10.4

	Date the data were accessed	Link(s) to data sources	Dataset quality control steps *	Written permission received to post annual ground-water level data (see footnote: [0])	Estimated groundwater withdrawals (km^3/year) [Z]
		erbase-water-quantity-11#tab-visualisations-produced	Perrone, D. (2021) Science 372, 418–421		
**Japan**	**Jan-26-2023**	**http://www1.river.go.jp/**	**section 1.22 (i.e., below in this file)**		**10.94**
**Laos**	**Jul-14-2021**	**https://www.hydrologynz.org.nz/journal/volume-56-2017 and https://opus.lib.uts.edu.au/handle/10453/135928**	**section 1.23 (i.e., below in this file)**		**0.34**
**Latvia**	**Apr-20-2023**	**https://www.eea.europa.eu/en/datahub/datahubitem-view/a4324714-784a-4763-b8b9-b8939ead87fe**	**section 1.24 (i.e., below in this file)**	**Yes [E]**	**0.11**
**Lithuania**	**Apr-20-2023**	**https://www.eea.europa.eu/en/datahub/datahubitem-view/a4324714-784a-4763-b8b9-b8939ead87fe**	**section 1.25 (i.e., below in this file)**	**Yes [E]**	**0.17**
Mexico	Jul-25-2019	https://datos.gob.mx/busca/dataset/red-piezometrica-mapas [<]	Supplementary Materials section S2.24 within Jasechko, S., Perrone, D. (2021) Science 372, 418–421		29.45
**Morocco: Souss-Massa**	**Jun-16-2023**	**http://www.abhsm.ma/index.php/partenariat-5/partenariat-4/piezometrie**	**section 1.26 (i.e., below in this file)**		**3.06 [y]**

	Date the data were accessed	Link(s) to data sources	Dataset quality control steps *	Written permission received to post annual ground-water level data (see footnote: ⁰)	Estimated groundwater withdrawals (km^3/year) [z]
New Zealand	Mar-26-2020	https://ecan.govt.nz/data/well-search/	Supplementary Materials section S2.25 within Jasechko, S., Perrone, D. (2021) Science 372, 418–421	Yes	0.80
Norway ^	Aug/Sep-2019	https://www2.nve.no/h/hd/plotreal/GRW/list.html ^	Supplementary Materials section S2.26 within Jasechko, S., Perrone, D. (2021) Science 372, 418–421	Yes ^	0.41
Pakistan	Sep-18-2020	irrigation.punjab.gov.pk	Supplementary Materials section S6.1.4 within Jasechko, S., Perrone, D. (2021) Science 372, 418–421		64.82
**Paraguay**	**Jan-05-2021**	**http://www.meteorologia.gov.py/nivel-pozo/**	**section 1.27 (i.e., below in this file)**	**Yes**	**0.25**
**Poland**	**Apr-20-2023**	**https://www.eea.europa.eu/en/datahub/datahubitem-view/a4324714-784a-4763-b8b9-b8939ead87fe**	**section 1.28 (i.e., below in this file)**	**Yes [E]**	**2.59**
Portugal	Feb-25-2020	https://snirh.apambi ente.pt	Supplementary Materials section S2.28 within Jasechko, S., Perrone, D. (2021) Science 372, 418–421		6.29
**Saudi Arabia [U]**	**Sep-30-2020**	**Fallatah, O.A. (2020). Groundwater quality patterns and spatiotemporal change in depletion in**	**section 1.29 (i.e., below in this file)**		**24.24**

	Date the data were accessed	Link(s) to data sources	Dataset quality control steps *	Written permission received to post annual ground-water level data (see footnote: [0])	Estimated groundwater withdrawals (km^3/year) [Z]
		**the regions of the Arabian shield and Arabian shelf. Arabian Journal for Science and Engineering, 45, 341-350**			
Sierra Leone	Feb-28-2020	https://nwrma.gov.sl/data/ground-water/ [zzzz]	Supplementary Materials section S2.29 within Jasechko, S., Perrone, D. (2021) Science 372, 418–421		0.11
**Slovenia**	**Apr-20-2023**	**https://www.eea.europa.eu/en/datahub/datahubitem-view/a4324714-784a-4763-b8b9-b8939ead87fe**	**section 1.30 (i.e., below in this file)**	**Yes [E]**	**0.19**
South Africa	Oct-01-2020	http://www3.dwa.gov.za/nganet/Security/WebLoginForm.aspx	Supplementary Materials section S2.30 within Jasechko, S., Perrone, D. (2021) Science 372, 418–421		3.14
Spain	Sep-17-2019	https://sig.mapama.gob.es/redes-seguimiento/?herramienta=Piezometros	Supplementary Materials section S2.31 within Jasechko, S., Perrone, D. (2021) Science 372, 418–421		5.70
Sweden ♦	Sep-12-2019	https://www.sgu.se/produkter/geologiska-data/oppna-data/grundvatten-oppna-data/grundvattennivaer-tidsserier ♦	Supplementary Materials section S2.32 within Jasechko, S., Perrone, D. (2021) Science 372, 418–421	Yes ♦	0.35

	Date the data were accessed	Link(s) to data sources	Dataset quality control steps *	Written permission received to post annual ground-water level data (see footnote: [0])	Estimated groundwater withdrawals (km^3/year) [Z]
**Switzerland**	**Apr-20-2023**	**https://www.eea.europa.eu/en/datahub/datahubitem-view/a4324714-784a-4763-b8b9-b8939ead87fe**	**section 1.31 (i.e., below in this file)**	**Yes [E]**	**0.79**
**Taiwan [U]**	**Mar-18-2021**	**Personal communication with Dr. Y.J. Hsu.<br><br>See: Hsu, Y.J., Fu, Y., Bürgmann, R., Hsu, S.Y., Lin, C.C., Tang, C.H., & Wu Y.M. Assessing seasonal and interannual water storage variations in Taiwan using geodetic and hydrological data. Earth and Planetary Science Letters 550, 116532 (2020).**	**section 1.32 (i.e., below in this file)**		**9.54**
**Thailand**	**Dec-30-2020**	**http://tgms.dgr.go.th/#/home**	**section 1.33 (i.e., below in this file)**		**10.74**
UK	August-2020	http://mapapps.bgs.ac.uk/groundwatertimeline/home.html	Supplementary Materials section S2.33 within Jasechko, S., Perrone, D. (2021) Science 372, 418–421		2.16
**USA: NWIS**	**May-25-2023**	**https://waterservices.usgs.gov/rest/GW-Levels-Service.html**	**section 1.34 (i.e., below in this file)**	**Yes**	**111.7** *(note: 111.7 for the entire USA)*

	Date the data were accessed	Link(s) to data sources	Dataset quality control steps *	Written permission received to post annual ground-water level data (see footnote: [0])	Estimated groundwater withdrawals (km^3/year) [z]
**USA: TWDB**	**May-29-2023**	**http://www.twdb.texas.gov/groundwater/data/gwdbrpt.asp**	**section 1.35 (i.e., below in this file)**	**Yes**	**111.7** *(note: 111.7 for the entire USA)*
**USA: California DWR**	**May-29-2023**	**https://data.ca.gov/dataset/periodic-groundwater-level-measurements and https://data.ca.gov/dataset/continuous-groundwater-level-measurements**	**section 1.36 (i.e., below in this file)**		**111.7** *(note: 111.7 for the entire USA)*
**USA: California GAMA**	**Jun-12-2023**	**http://geotracker.waterboards.ca.gov/gama/datadownload**	**section 1.37 (i.e., below in this file)**	**Yes**	**111.7** *(note: 111.7 for the entire USA)*
**Vietnam**	**Jan-22-2023**	**http://123.16.176.41/quantractainguyennuoc/public/**	**section 1.38 (i.e., below in this file)**		**1.63**

* ***bold and italicised*** formatting (with grey background shading) highlights that the data are described further in a supplementary subsection in the following pages

[0] we emailed database managers and requested permission to post annual groundwater level data. Values of "Yes" in this field correspond to datasets where we received permission from a database manager to annual post groundwater level data (see Data Availability statement)

*** a representative with PEI Environment stated in an email (received March 13, 2023) that this hyperlink is scheduled for decommissioning; the recommended (updated) webpages where PEI groundwater level data can be accessed are: https://www.princeedwardisland.ca/en/service/pei-water-registry#utm_source=redirect&utm_medium=url&utm_campaign=waterregistry and https://data.princeedwardisland.ca/Environment-and-Food/OD0038-Groundwater-Level-Monitoring/cgzy-bim6*

**** a representative with the Yukon Water Resources Branch requested via email (received March 15, 2023) that we note the following disclaimer (quoted directly from https://yukon.ca/sites/yukon.ca/files/20220610-hydrometric-conditions-report.pdf, accessed March 31, 2023): "The User understands and acknowledges that the use of the data is solely at their own risk. The User is solely responsible for confirming the accuracy, availability, suitability, reliability, usability, completeness or timeliness of the data. The User accepts the data "as is" and acknowledges that the Government of Yukon makes no warranties or representations (express or implied) with respect to the accuracy, availability, suitability, reliability, usability, completeness or timeliness of the data, including, without limitation, implied warranties for merchantability, fitness for a particular purpose, and non-infringement. In consideration of access to the data, the User also agrees that in no event will the Government of Yukon be liable (in tort or contract) or responsible whatsoever to the User or any other legal entity for the accuracy, availability, suitability, reliability, usability, completeness or timeliness of the data, including, without limitation, any loss of revenue*

or profit, or for direct, indirect, special, incidental, or consequential damages arising from or related to the data." (italicized text is direct quote from https://yukon.ca/sites/yukon.ca/files/20220610-hydrometric-conditions-report.pdf)

***** a representative with Alberta Environment and Protected Areas requested via email (received March 23, 2023) that we include the following disclaimer (the following text quoted directly from an email received from a representative with Alberta Environment and Protected Areas): "Please note that the information in this data set may not be fully checked. The Province disclaims all responsibility for its accuracy. The Province is continually cross checking the data to detect errors in order to improve the accuracy of the datasets. New data is being keyed regularly. Data provided is provisional and preliminary in nature. Data may be collected by several different sources and/or methods at various dates. This data may not been reviewed or edited for accuracy and may be subject to significant change when reviewed or corrected. Please exercise caution and carefully consider the provisional nature of the information provided. The Government of Alberta assumes no responsibility for the accuracy or completeness of this data and any use of it is therefore, entirely at your own risk."*

[W] At the request of the individual providing these data (email received March-14-2023), we wish to specifically cite USGS as the source of these data

[+] We cite the source as follows: © Land NRW, dl-de/by-2-0 (www.govdata.de/dl-de/by-2-0) https://www.elwasweb.nrw.de < January 24, 2020 >

[E] We acknowledge EEA as the original source of the material (i.e., data)

[†] We cite the Geological Survey of Brazil as the holder of the information (i.e., data). We acknowledge Valmor Jose Freddo Filho (Executive Coordinator of the Department of Hydrology with Serviço Geológico do Brasil (SGB)) for their support.

[§] We specifically acknowledge the Saskatchewan Water Security Agency for making these data available to use for analyses

‡ We provide the following specific attribution to the source of these data: Water and Air Monitoring Section of PEI Environment, Energy and Climate Action

[♠] A representative with ADES recommended the following article that provides further details pertaining to the ADES database: ref.[1291] (i.e., Winckel, A., Ollagnier, S., & Gabillard, S. Managing groundwater resources using a national reference database: the French ADES concept. SN Applied Sciences, 4, 217 (2022), available via: https://link.springer.com/article/10.1007/s42452-022-05082-0)

[♦] We acknowledge the source of these data to be SGU (Geological Survey of Sweden) and acknowledge the SGU copyright: "©Geological Survey of Sweden." (quoted from https://www.sgu.se/en/products/customer-services/copyright/, accessed June 20, 2023). We also quote the following disclaimer provided on the SGU sopyright webpage: "SGU disclaims liability for any loss, damage, injury or other adverse consequence that may arise from the use of our information, on its own or together with other information. Documents that have been scanned or interpreted using OCR may contain errors in the form of missing maps, appendices and pages, inverted pages and misspellings." (quoted from https://www.sgu.se/en/products/customer-services/copyright/, accessed June 20, 2023)

[^] The webpage used to access Norway groundwater level data in 2019 (i.e., cited in this supplementary table) is no longer active (personal communication with a representative of the Department of Hydrology, Norwegian Water Resources and Energy Directorates (NVE) on June 21, 2023). The updated webpage for accessing groundwater level data is https://sildre.nve.no/ (accessed June 21, 2023)

[x] We received an email March-16-2023 from a database manager with Environnement Brussels highlighting the following important components pertaining to the dataset: "We authorize you to publish a file of additional data on the annual median groundwater levels based on the data on our application. The use of the information downloaded from this application is under the responsibility of the user. Brussels Environment makes every effort to ensure that the information available on this application is correct, without guaranteeing its accuracy and completeness. Indeed, the information contained in this application may contain errors which, as soon as they have been brought to the attention of Brussels Environment, will be corrected as far as possible. In addition, Brussels Environment may make changes to this application at any time and without notice to improve it."

[y] at the request of a representative of the Service Public de Wallonie, we clarify that we have calculated annual median groundwater levels ourselves from Service Public de Wallonie data (specifically, we were informed that these data can be posted under the condition that we "…clearly indicate that you have calculated these values yourself from our daily data" (quoting email received March-23-2023 from piezometrie@spw.wallonie.be)

[Z] groundwater withdrawal estimates derived from Appendix 5 (field entitled "Estimated groundwater abstraction 2010" within ref.[18] (i.e., J. Margat, J. van der Gun, Groundwater Around the World: A Geographic Synopsis (CRC, 2013). Accessed June 14, 2023 via the website: https://www.un-igrac.org/sites/default/files/resources/files/Groundwater_around_world.pdf). The sum of groundwater withdrawals among all countries for which we have some piezometric data (i.e., the sum of values in the rightmost column of this table, counting Canada – which is

broken up into several provincial databases – just once in our calculation of the sum) is 806 km/^{3} year. The sum of estimated groundwater abstractions in Appendix 5 of ref.[18] is 982 km^3/year. Thus, the countries for which we compiled some piezometric groundwater level data represent 82.5% of estimated global groundwater abstractions (among the countries in Appendix 5 of ref.[18]; i.e., [810 km^3/year] divided by [982 km^3/year]). Values presented here are stimates for an entire country, but some datasets cover only a portion of the country.

[zz] we received a request to include the following statement alongside the Manitoba dataset (the following quoted from an email received July 15, 2023): "Note that the provided data may be subject to mechanical or human error, is largely unchecked, and is provided as-is; it comes with no implied or expressed guarantee as to the accuracy of the data and the values are subject to correction."

[zzz] we downloaded groundwater level data for Finland January 20, 2023 from https://wwwp2.ymparisto.fi/scripts/hearts/welcome.asp. However, we corresponded with an individual with the Finnish Environment Institute, who confirmed via email that the Finland dataset "contains many errors" (quoting email received June 28, 2023), prompting us to decide to exclude Finland groundwater level data from our analyses.

[zzzz] this website provides access to groundwater level data (as of November 1, 2023); however, groundwater level data were originally downloaded (on the date specified in the second column from the left) from https://www.salonewatersecurity.com/groundwater

[zzzzz] this website provides access to groundwater level data (as of November 1, 2023); however, groundwater level data were originally downloaded (on the date specified in the second column from the left) from https://www.indiawatertool.in

[>] this website provides access to groundwater level data (as of November 1, 2023); however, groundwater level data were originally downloaded (on the date specified in the second column from the left) from wrs.wrm.ir/amar

[<] this website provides access to groundwater level data (as of November 1, 2023); however, groundwater level data were originally downloaded (on the date specified in the second column from the left) from https://datos.gob.mx/busca/dataset/red-piezometrica-mapas/resource/cc203e00-3820-488a-8113-8243cb70853d

[U] data obtained via email communications and were not downloaded directly from website. Contacts associated with these databases include (i) gwh.bwdb@gmail.com (Bangladesh); (ii) nadine.caissielong@gnb.ca (Canada: New Brunswick); (iii) dina.daher@environnement.gouv.qc.ca (Canada: Quebec), (iv) avesga@ideam.gov.co (Colombia), (v) ofallatah@kau.edu.sa (Saudi Arabia), (vi) yaru@earth.sinica.edu.tw (Taiwan). These email addresses were active and the agencies/individuals were responsive at the time we requested access to the data.

1.1 AFGHANISTAN

Afghanistan groundwater level data were downloaded December 17, 2020 from https://pubs.usgs.gov/of/2013/1296. These data have been analysed and described in the following report: ref.[1289] (i.e., Taher, M.R., Chornack, M.P., & Mack, T.J. Groundwater levels in the Kabul Basin, Afghanistan, 2004–2013: US Geological Survey Open-File Report 2013-1296, 51 pp. http://dx.doi.org/10.3133/ofr20131296 (2014)). We acknowledge T.J. Mack for helping us to understand aspects of the data via email correspondence on December 17, 2020.

- *Well depths were obtained from the USGS report by matching the well identifier code listed under "Number" in the USGS report to the value listed under "GWM_ID" in the provided Excel files;*
- *We only analysed measurements if both of the following conditions were met: "Status" field states "Not pumping" and "WLCondition" field states "Static" – in this way we exclude measurements made when the well itself was being pumped or if the well was recorded to have been pumped recently (i.e., relative to that date the water level measurement was made);*
- *The recorded latitude and longitude values appear to be swapped for well ID 41; we swapped these values (i.e., replacing the recorded latitude with the recorded longitude, and replacing the recorded longitude with the recorded latitude);*
- *Water levels were interpreted as the measured water level minus the measuring point (i.e., assuming the measuring point has been measured as the "stick up" above the land surface). For n=25 rows in the dataset there was no numeric measuring point value (e.g., first two rows of data for GWM_ID "217" in the file entitled "Well 217_Data.xlsx"); we filled in these missing measuring point values with the measuring point value from the*

adjacent row in the dataset (choosing the closest measurement made in time to the row for which the measuring point value is missing) for that well's time series.

We excluded 7 measurements that have duplicate measurement dates.

1.2 ARGENTINA

Groundwater level observations for wells in Argentina were downloaded January 20, 2023 from http://datar.info/pt_BR/dataset/pozos-sifas. Static water levels (field entitled "NIVELE") with a value of zero were excluded from our analysis, as we could not confirm that these were not blank entries (as opposed to flowing artesian wells with water levels at the top of casing (or at land surface)). The longitude for one well ("F034") was recorded as "-5845", which was interpreted as a typo (i.e., the longitude was corrected to be -58.45, a longitude placing this well at a location very close (<10 km) to other monitoring wells in the dataset with similar well identifier codes (e.g., well "F035" has a recorded longitude of -58.36). n=14 records have well water levels that are deeper than the recorded well depth (e.g., one well water level record has a value of "NIVELE" of 41,720 m (and the well depth is only 172 m)); these n=14 records were excluded from analysis.

We also downloaded groundwater level time series data from http://www.bdh.acumar.gov.ar/bdh3/aguasubterranea_listado.php (accessed January 20, 2023) We identified the depths of these monitoring wells from the aforementioned point dataset (http://datar.info/pt_BR/dataset/pozos-sifas; see paragraph above) on the basis of monitoring well codes.

1.3 AUSTRALIA

We downloaded monitoring well water level data from Australia's Bureau of Meteorology on November 2, 2021 from http://www.bom.gov.au/water/groundwater/explorer/map.shtml.

1.4 AUSTRIA

Groundwater level data for Austria were downloaded from the European Environment Agency (EEA) webpage on April 20, 2023: https://www.eea.europa.eu/en/datahub/datahubitem-view/a4324714-784a-4763-b8b9-b8939ead87fe. Unique monitoring well locations were identified by matching site identifier codes to either (a) identifier codes within the most recent version of Waterbase (as of April 20, 2023) or, in the case no well location data were available, (b) identifier codes in an outdated (2018) version of the European Environment Agency Waterbase metadata* (*the 2018 Waterbase dataset metadata were used to locate wells, as the version available at the time we downloaded the Waterbase dataset (April-2023) had a confirmed issue (personal communication with the "EEA Enquiry Service" April 28, 2023) causing it to fail to report monitoring site locations for some sites). We excluded measurements if only a calendar year (no month and no day of measurement) was available for their measurement date. For measurements dates that lack information about the day of measurement (i.e., only a calendar year and month were available) we approximated the measurement date as the midpoint of the month. We deleted groundwater levels with a value of zero.

1.5 BANGLADESH

Bangladesh groundwater level time series data were made available to us from a monitoring network established and overseen by Bangladesh Water Development Board (BWDB; www.hydrology.bwdb.gov.bd). Data were provided to us at a monthly time step, though some monitoring wells may have measurements at finer temporal resolutions. These groundwater level data are described by ref.[21] (i.e., Shamsudduha, M., Taylor, R.G., Haq, M. I., Nowreen, S., Zahid, A., & Ahmed, K.M.U. The Bengal Water Machine: Quantified freshwater capture in Bangladesh. *Science* **377,** 1315-1319 (2022)); ref.[21] provides the following statement regarding data availability (the following grey italicised text is a direct quote from ref.[21]): *"We secured groundwater-level monitoring data from the BWDB and dry-season irrigation well information from the BADC; the latter was used to estimate groundwater abstraction. Original weekly monitored groundwater-level time-series data can be obtained from the BWDB (http://www.hydrology.bwdb.gov.bd/index.php) by making an "online data request" and payment. Processed monthly time-series data used in this paper along with codes written in R programming language can be made available upon request to the corresponding author for the purpose of reproducing or extending the analysis and creating visual illustrations…"* (quoted directly from ref.[21]).

1.6 BELGIUM: BRUSSELS

We downloaded Belgian groundwater level data from the Brussels Department of the Environment database on August 19, 2019 from https://geodata.environnement.brussels/client/view/b4231044-be68-4a1a-bf98-9e5397bb5324 (monitoring well construction metadata including well locations and depths were accessed via: https://geodata.environnement.brussels/client/view/22713aac-e03d-4a18-b0eb-c71f3ca55db1).

1.7 BELGIUM: WALLONIA

We downloaded Belgium (Wallonia) groundwater level time series August 2, 2021 from the Piezometric Network of the Department of Groundwater via:
http://piezo.environnement.wallonie.be/login.do?time=156650002697
5#

1.8 BULGARIA

Groundwater level data for Bulgaria were downloaded from the European Environment Agency (EEA) webpage on April 20, 2023: https://www.eea.europa.eu/en/datahub/datahubitem-view/a4324714-784a-4763-b8b9-b8939ead87fe. Unique monitoring well locations were identified by matching site identifier codes to either (a) identifier codes within the most recent version of Waterbase (as of April 20, 2023) or, in the case no well location data were available, (b) identifier codes in an outdated (2018) version of the European Environment Agency Waterbase metadata* (*the 2018 Waterbase dataset metadata were used to locate wells, as the version available at the time we downloaded the Waterbase dataset (April-2023) had a confirmed issue (personal communication with the "EEA Enquiry Service" April 28, 2023) causing it to fail to report monitoring site locations for some sites). We excluded measurements if only a calendar year (no month and no day of measurement) was available for their measurement date. For measurements dates that lack information about the day of measurement (i.e., only a calendar year and month were available) we approximated the measurement date as the midpoint of the month. We deleted groundwater levels with a value of zero as there was at least one station where zero values appeared to be outliers in the time series (e.g., station 'BG1G00000NQMP150'). We excluded seven stations—each containing the letter "J" within its station code (e.g., BG1G0000**J**3KMP311)—where we could not be certain that the reported units of measurement were in depth to water (rather than groundwater elevation); the excluded stations are: BG1G0000J3KMP311, BG1G0000J3KMP313, BG1G0000J3KMP314, BG1G0000J3KMP316, BG1G0000J3KMP318, BG1G0000J3KMP319, and BG1G0000J3KMP321.

1.9 BURKINA FASO

Groundwater level time series for monitoring wells in Burkina Faso were accessed and digitized by R.G. Taylor from display items published by ref.[1292] (i.e., Ascott et al. (2020): Ascott, M.J., Macdonald, D.M.J., Black, E., Verhoef, A., Nakohoun, P., Tirogo, J., Sandwidi, W.J.P., Bliefernicht, J., Sorensen, J.P.R., & Bossa, A.Y. In situ observations and lumped parameter model reconstructions reveal intra-annual to multidecadal variability in groundwater levels in sub-Saharan Africa. *Water Resources Research* **56,** e2020WR028056 (2020)).

1.10 CANADA: ONTARIO

We downloaded groundwater level data for Ontario July 20, 2021 from https://data.ontario.ca/dataset/provincial-groundwater-monitoring-network. Where groundwater levels were reported in absolute elevation, we calculated depth to groundwater on the basis of the reported groundwater surface elevation.

1.11 CHINA: GONG ET AL. (2018)

We downloaded groundwater level time series for the North China Plain were provided via email from Y. Pan received January 6, 2021 and are presented in ref.[1290] (i.e., Gong, H., Pan, Y., Zheng, L., Li, X., Zhu, L., Zhang, C., Huang, Z., Li, Z., Wang, H. & Zhou, C. Long-term groundwater storage changes and land subsidence development in the North China Plain (1971–2015). *Hydrogeology Journal* **26,** 1417-1427 (2018)).

1.12 CHINA: TAO ET AL. (2020)

We downloaded Chinese groundwater level data on January 18, 2021 via the "Supporting Information" appended to ref.[1293] (i.e., Tao, S., Zhang, H., Feng, Y., Zhu, J., Cai, Q., Xiong, X., Ma, S., Fang, L., Fang, W., Tian, D., Zhao, X., Fang, J. Changes in China's water resources in the early 21st century. *Frontiers in Ecology and the Environment* **18,** 188-193 (2020)).

1.13 CHINA: CIGEM

We downloaded groundwater level measurements for numerous monitoring wells across China September 21, 2020 from http://jcgc.cigem.cn/share

1.14 COLOMBIA

Groundwater level time series for Colombia were sent to us by A.M. Vesga Güiza with the Instituto de Hidrología, Meteorología y Estudios Ambientales (IDEAM; webpage: http://www.ideam.gov.co) on December 18, 2020. We excluded any records for which we could not identify a well location.

1.15 CROATIA

Groundwater level data for Croatia were provided to us via personal communication with D. Biondić on September 22, 2020. The dataset contains water level measurements for the years 2019-2020. We acknowledge Hrvatske vode as the source of the data.

1.16 CZECH REPUBLIC

Groundwater level data for Czechia were provided to us via personal communication with A. Lamačová (Czech Hydrometeorological Institute, Groundwater Department) on February 8, 2021. We acknowledge the Czech Hydrometeorological Institute for developing and providing these data.

1.17 DENMARK

Groundwater level data for Denmark were downloaded April 20, 2023 via https://www.eea.europa.eu/en/datahub/datahubitem-view/a4324714-784a-4763-b8b9-b8939ead87fe. Unique monitoring well locations were identified by matching site identifier codes to either (a) identifier codes within the most recent version of Waterbase (as of April 20, 2023) or, in the case no well location data were available, (b) identifier codes in an outdated (2018) version of the European Environment Agency Waterbase metadata* (*the 2018 Waterbase dataset metadata were used to locate wells, as the version available at the time we downloaded the Waterbase dataset (April-2023) had a confirmed issue (personal communication with the “EEA Enquiry Service” April 28, 2023) causing it to fail to report monitoring site locations for some sites). For measurements dates that lack information about the day of measurement (i.e., only a calendar year and month were available) we approximated the measurement date as the midpoint of the month. Many of the long-term (>7 year long) groundwater level time series for Denmark report only a calendar year for the measurement (no month or day value); we approximated the measurement date as the midpoint of the calendar year for these measurements. These monthly and annual groundwater level data may, in some cases, be averages of a series of measurements made at high frequency (e.g., see the following “Remarks” entry: “yearly average based on 2 manual measurements and 3450 data logger measurements”).

1.18 EL SALVADOR

Groundwater level time series were downloaded from http://srt.snet.gob.sv/sihi/public/app/1/pozosmonitoreo on January 22, 2023. We excluded n=1 record with an implausible recorded measurement date that postdates the date that the dataset was downloaded.

1.19 FRANCE AND FRENCH GUIANA

Groundwater level time series for France and French Guiana were downloaded September 30, 2020 from ADES via https://ades.eaufrance.fr/Recherche.

For measurements where we only have groundwater elevation (rather than depth to groundwater), we converted these values of 'groundwater level elevation' to 'depth to groundwater' on the basis of reference point elevation data provided in the database.

In our review of these data, we identified some suspicious data points; for example, the station BSS002LVTN recorded a well depth of 14.0 m (right hand side of the InfoTerre webpage linked to this well (website accessed in 2020)) but the piezometric time series (left hand side from ADES' webpage (accessed in 2020)) showed depth to groundwater measurements of 83 m (i.e., a recorded depth to groundwater measurement that is deeper than the recorded well depth). We emailed with a representative from ADES in 2020 and followed their instructions to correct issues that we identified.

A representative from ADES directed us to a recent publication that provides more information about the ADES database see ref.[1291] (i.e., Winckel, A., Ollagnier, S., & Gabillard, S. Managing groundwater resources using a national reference database: the French ADES concept. *SN Applied Sciences* **4,** 217 (2022)).

1.20 HAITI

Well water level data for two aquifer systems in Haiti (Cul-de-Sac Plain and Leogane Plain) were downloaded from the electronic supplementary information published by ref.[1294] (Adamson, J.K., Miner, W., Rochat, P.Y., Moliere, E., Piasecki, M., LaVanchy, G.T., Perez-Monforte, S., Rodriquez-Vera, M. Significance of river infiltration to the Port-Au-Prince metropolitan region: a case study of two alluvial aquifers in Haiti. *Hydrogeology Journal* **30,** 1367-1386 (2022)). Records with a statement reading "Flowing artesian well" were ascribed a static water level value of zero. Static water level data were converted from the units in the primary dataset (meters above sea level) to units of meters below land surface using the land surface elevation data provided in the primary dataset. For some wells, multiple water level measurements were made; these wells were identified by identical values of latitude, longitude, well depth and well identifier (well ID). One monitoring station (identified by its identical "Point ID" value) has a different recorded latitude than the other rows in the table with the identical "Point ID" value (see Table S3 on page 18 of the electronic supplement by ref.[1294]). As this latitude does not occur within Haiti, we replaced the stated latitude value (0.68250) with the stated latitude for other records with the identical "Point ID" ("ROU.02 (ROU.02)").

1.21 ISRAEL

Groundwater level data for Israel were received from the Israeli Hydrological Service. We thank and acknowledge the Israeli Hydrological Service, Israel Water Authority (i.e., the source of the data).

1.22 JAPAN

We downloaded groundwater level time series from http://www1.river.go.jp/cgi-bin/SrchSite.exe by searching for monitoring wells (search term: "地下水位・水質"). n=171 stations were excluded as we could not identify a latitude and longitude for the station. We excluded measurements with values that could not be confirmed to represent actual water level measurements (and instead potentially notation for missing measurements (e.g., values that were excluded: 9999.99, -99, -999). We excluded four stations where the recorded well locations were uncertain (stations that were excluded are: 506041286608030＃＃年, 506041286608040＃＃年, 506041286608050＃＃年, and 506041286608060＃＃年).

1.23 LAOS

Groundwater level time series for the Sukhuma District of Laos were contributed by M. Shamsudduha from the work published by ref.[1295] (i.e., Vongphachanh, S., Gupta, A.D., Milne-Home, W., Ball, J.E., & Pavelic, P. Hydrogeological reconnaissance of Sukhuma District, Champasak Province, Southern Laos. Journal of Hydrology (New Zealand), **56,** 79-96 (2017), data available via appendix 11 within: https://opus.lib.uts.edu.au/handle/10453/135928).

1.24 LATVIA

Groundwater level time series for Latvia were downloaded from the European Environment Agency on April 20, 2023 via https://www.eea.europa.eu/en/datahub/datahubitem-view/a4324714-784a-4763-b8b9-b8939ead87fe. Unique monitoring well locations were identified by matching site identifier codes to either (a) identifier codes within the most recent version of Waterbase (as of April 20, 2023) or, in the case no well location data were available, (b) identifier codes in an outdated (2018) version of the European Environment Agency Waterbase metadata* (*the 2018 Waterbase dataset metadata were used to locate wells, as the version available at the time we downloaded the Waterbase dataset (April-2023) had a confirmed issue (personal communication with the "EEA Enquiry Service" April 28, 2023) causing it to fail to report monitoring site locations for some sites). We excluded groundwater level measurements that reported only a calendar year (no month and no day of measurement). Some measurements report only a calendar year and month (no value for the day of measurement); these measurement dates were approximated by the midpoint of the month. We also excluded blank and zero values. We deleted one measurement with a value of "1118.28" meters, as all other measurements for this station had values of ~10 times less (i.e., the measurement preceding this value had a value of 118.23, and the following value was 118.22). Further, we deleted another measurement with a value of "309" meters, as all other measurements for this station had values of ~100 times less (e.g., the measurement preceding this one has a value of 3.16).

1.25 LITHUANIA

Lithuania groundwater level time series were downloaded April 20, 2023 via https://www.eea.europa.eu/en/datahub/datahubitem-view/a4324714-784a-4763-b8b9-b8939ead87fe. Unique monitoring well locations were identified by matching site identifier codes to either (a) identifier codes within the most recent version of Waterbase (as of April 20, 2023) or, in the case no well location data were available, (b) identifier codes in an outdated (2018) version of the European Environment Agency Waterbase metadata* (*the 2018 Waterbase dataset metadata were used to locate wells, as the version available at the time we downloaded the Waterbase dataset (April-2023) had a confirmed issue (personal communication with the “EEA Enquiry Service” April 28, 2023) causing it to fail to report monitoring site locations for some sites). Many measurement dates have only a calendar year (no month and no day of measurement) available; for these measurements, we approximated the measurement date as the midpoint of the year.

We excluded measurements if only a calendar year (no month and no day of measurement) was available for their measurement date. For measurements dates that lack information about the day of measurement (i.e., only a calendar year and month were available) we approximated the measurement date as the midpoint of the month. We deleted groundwater levels with a value of zero.

1.26 MOROCCO: SOUSS-MASSA

Morocco (Souss-Massa region) groundwater level time series were downloaded from http://www.abhsm.ma/index.php/partenariat-5/partenariat-4/piezometrie on June 16, 2023. Well locations ("Points d'eau" dataset) were downloaded June 16, 2023 via http://www.abhsm.ma/index.php/partenariat-5/partenariat-4/point-d-eau. We could not immediately identify metadata specifying the coordinate system that the "Points d'eau" dataset was projected in; we found a close correspondence between a map of monitoring wells obtained from the ABHSM website on June 19, 2023 (via http://www.abhsm.ma/index.php/photo-gallery-2/cartes-thematiques/event/Contrleetsuividesressourceseneau?lightbox=1) and the "Points d'eau" dataset projected in UTM coordinates; we highlight that there is some uncertainty in our estimates of monitoring well locations. We excluded all stations where we could not identify the well location within the "Points d'eau" dataset. Further, we excluded one monitoring well with a recorded location that appeared to be located ~50 km offshore. We also excluded a suite of monitoring wells that have recorded locations that fall outside of the Souss-Massa, Guelmim and Draa basins (as identified by georeferencing the following map from the ABHSM webpage: http://www.abhsm.ma/index.php/photo-gallery-2/cartes-thematiques/event/Contrleetsuividesressourceseneau?lightbox=1). We excluded measurements with recorded groundwater levels of precisely "0", "-1,00", or "-2.00" because we could not confirm that these values represented actual depth to groundwater measurements instead of blank entries or codes for suspicious measurements (based upon a visual inspection of groundwater level time series, these values appear to often be discrepancies compared to similar measurements made in the same well, suggesting that these values are likely flags for

missing/inadequate measurements in at least some cases). We excluded one measurement from the station with the identifying code of "203744"; the excluded measurement has a recorded value of "988.88" that appear to be an outlier, given that all other measurements for this monitoring well range from "6,95" to "9,00". Further, we excluded one measurement with a value of "889.4", as we could not confirm that this measurement was not a data entry error given that the measurement immediately before and after this one have values of 88.32 and 88.47 (suggesting that the decimal place may have been entered in the wrong location). Similarly, we excluded one value of "409", as the measurements immediately preceding and following it have values close to four (4.1 and 3.53), suggesting the decimal may have been entered in the wrong location. Similarly, again, we excluded one value of "402.2", as the measurements immediately preceding and following it have values close to forty (39.7 and 40.15). We excluded a recorded value of 377.01 (station ID 210923), as the preceding and following measurements (3.35 and 4.5) suggest a decimal place entry error may have taken place. We excluded a recorded value of 354.75 (station ID 200538), as the preceding and following measurements (34.29 and 36.27) suggest a decimal place entry error may have taken place. We excluded a recorded value of 304 (station ID 212952), as the following measurement (3.58) suggests a decimal place may have not been entered for this measurement, leading the recorded value to be a factor of ~100 times higher than all other measurements recorded for this station. We excluded three measurements for station ID 197511, as the preceding and following measurements were a factor of ~100 times lower in all cases, suggesting a decimal place entry error may have taken place (the three excluded measurements all have the same value of 267.85). We excluded a recorded value of 174 (station ID

214931), as the following measurement (17.23) suggests a decimal place may have not been entered for this measurement, leading the recorded value to be a factor of ~100 times higher than all other measurements recorded for this station.

1.27 PARAGUAY

We downloaded groundwater level time series for four monitoring wells in Paraguay on January 5, 2021 from www.meteorologia.gov.py/nivel-pozo/. The locations of these monitoring wells are imprecise, as only the city was provided (e.g., “Loma Pytá” under field “Ciudad”).

1.28 POLAND

Groundwater level data for Poland were accessed via https://www.eea.europa.eu/en/datahub/datahubitem-view/a4324714-784a-4763-b8b9-b8939ead87fe (download date: April 20, 2023). Unique monitoring well locations were identified by matching site identifier codes to either (a) identifier codes within the most recent version of Waterbase (as of April 20, 2023) or, in the case no well location data were available, (b) identifier codes in an outdated (2018) version of the European Environment Agency Waterbase metadata* (*the 2018 Waterbase dataset metadata were used to locate wells, as the version available at the time we downloaded the Waterbase dataset (April-2023) had a confirmed issue (personal communication with the "EEA Enquiry Service" April 28, 2023) causing it to fail to report monitoring site locations for some sites). Most measurement dates have only a calendar year (no month and no day of measurement) available; for these measurements, we approximated the measurement date as the midpoint of the year. Unlike many other EEA datasets compiled here, this dataset included a remark specifying that many measurements are reported in units of "depth to groundwater" (instead of groundwater elevation); we excluded measurements if the units of measurement were not specified in the 'remarks' column (we retained measurements with a remark of 'artesian aquifer' or 'depth to the groundwater level'). We excluded measurements with the remark "unreliable measurements due to a faulty manometer".

1.29 SAUDI ARABIA

Saudi Arabian groundwater level data were provided for analyses by O.A. Fallatah (see also ref.[1296]: Fallatah, O.A. Groundwater quality patterns and spatiotemporal change in depletion in the regions of the Arabian shield and Arabian shelf. *Arabian Journal for Science and Engineering* **45,** 341-350 (2020)). O.F. acknowledges the Ministry of Environment Water and Agriculture in Saudi Arabia for providing Saudi Arabian groundwater-level data.

We corrected one water level measurement with a value of "1933" meters, as the preceding water level measurement (of 191.3 meters) and the subsequent water level measurement in the time series (194.3 meters) suggest that a decimal place was absent; we therefore changed 1933 meters to 193.3 meters.

1.30 SLOVENIA

Slovenia groundwater time series were accessed April 20, 2023 via the European Environment Agency (EEA): https://www.eea.europa.eu/en/datahub/datahubitem-view/a4324714-784a-4763-b8b9-b8939ead87fe. Unique monitoring well locations were identified by matching site identifier codes to either (a) identifier codes within the most recent version of Waterbase (as of April 20, 2023) or, in the case no well location data were available, (b) identifier codes in an outdated (2018) version of the European Environment Agency Waterbase metadata* (*the 2018 Waterbase dataset metadata were used to locate wells, as the version available at the time we downloaded the Waterbase dataset (April-2023) had a confirmed issue (personal communication with the "EEA Enquiry Service" April 28, 2023) causing it to fail to report monitoring site locations for some sites). We excluded measurements if only a calendar year (no month and no day of measurement) was available for their measurement date. For measurements dates that lack information about the day of measurement (i.e., only a calendar year and month were available) we approximated the measurement date as the midpoint of the month. We excluded measurements that did not report the units of measurement in the field entitled "Remarks" (i.e., we retained measurements with a "Remarks" value of "water level in m asl - absolute values in meters above sea level").

1.31 SWITZERLAND

We downloaded groundwater level time series for Switzerland April 20, 2023 via https://www.eea.europa.eu/en/datahub/datahubitem-view/a4324714-784a-4763-b8b9-b8939ead87fe. Unique monitoring well locations were identified by matching site identifier codes to either (a) identifier codes within the most recent version of Waterbase (as of April 20, 2023) or, in the case no well location data were available, (b) identifier codes in an outdated (2018) version of the European Environment Agency Waterbase metadata* (*the 2018 Waterbase dataset metadata were used to locate wells, as the version available at the time we downloaded the Waterbase dataset (April-2023) had a confirmed issue (personal communication with the "EEA Enquiry Service" April 28, 2023) causing it to fail to report monitoring site locations for some sites). We deleted reported groudwater levels with a value of zero. We excluded measurements if only a calendar year (no month and no day of measurement) was available for their measurement date. For measurements dates that lack information about the day of measurement (i.e., only a calendar year and month were available) we approximated the measurement date as the midpoint of the month.

1.32 TAIWAN

Taiwan groundwater level data were provided to co-author R.G. Taylor via personal communication with Y.-J. Hsu; these data are also presented in ref.[1297] (Hsu, Y.J., Fu, Y., Bürgmann, R., Hsu, S.Y., Lin, C.C., Tang, C.H., & Wu Y.M. Assessing seasonal and interannual water storage variations in Taiwan using geodetic and hydrological data. *Earth and Planetary Science Letters* **550,** 116532 (2020)).

1.33 THAILAND

Thailand groundwater level monitoring data were downloaded December 8, 2020 from http://tgms.dgr.go.th/#/home.

We identified station (i.e., monitoring well) location data from the following webpage: http://tgms.dgr.go.th/#/report/station-status (accessed December 31, 2020); for 137 stations, we could not identify a well location from this website. For these 137 stations, we entered the station code into a search tool available at http://tgms.dgr.go.th/#/home (accessed October 29, 2021); we identified well location information for 119 of these 137 monitoring wells from this website.

1.34 UNITED STATES OF AMERICA: NWIS

We downloaded groundwater level data on May 25, 2023 from the United States Geological Survey REST portal (e.g., https://waterservices.usgs.gov/nwis/gwlevels/?format=rdb&countyCd=COUNTY&startDT=1800-01-01&endDT=2023-05-25&siteType=GW&siteStatus=all, where the word "COUNTY" was replaced with the corresponding county's FIPS code; e.g., "01009"). Second, we retrieved station information (e.g., locations, well depths) from the National Water Information System (https://waterdata.usgs.gov/nwis) and joined the groundwater level data to the station information via the field entitled "site_no". We completed several data cleaning steps prior to analysing these data:

First, we excluded the following site types (based on values in the Site Type Code heading "site_tp_cd"):

- *We excluded all rows in the dataset with a "site_tp_cd" value of "GW-MW" ("Multiple wells" – "A group of wells that are pumped through a single header and for which little or no data about the individual wells are available." quoting https://maps.waterdata.usgs.gov/mapper/help/sitetype.html, accessed May 29, 2023);*
- *We excluded all rows in the dataset with a "site_tp_cd" value of "GW-IW" ("Interconnected wells" – "Collector or drainage wells connected by an underground lateral.")*
- *We excluded all rows in the dataset with a "site_tp_cd" value of "GW-CR" ("Collector or Ranney type well" – "An infiltration gallery consisting of one or more underground laterals through which groundwater is collected and a vertical caisson from which groundwater is removed. Also known as a*

"horizontal well". These wells produce large yield with small drawdown.")

- *We excluded all rows in the dataset with a "site_tp_cd" value of "GW-TH" ("Test hole not completed as a well" – "An uncased hole (or one cased only temporarily) that was drilled for water, or for geologic or hydrogeologic testing. It may be equipped temporarily with a pump in order to make a pumping test, but if the hole is destroyed after testing is completed, it is still a test hole. A core hole drilled as a part of mining or quarrying exploration work should be in this class.")*

Second, we excluded the following water level measurements (based on data recorded in the field "lev_status_cd"):

- *We excluded all rows in the dataset with a "lev_status_cd" value of "2" (corresponding to "True value is below reported value due to local conditions")*
- *We excluded all rows in the dataset with a "lev_status_cd" value of "3" (corresponding to "True value is above reported value due to local conditions")*
- *We excluded all rows in the dataset with a "lev_status_cd" value of "6" (corresponding to "Measurement unable to be obtained due to local conditions")*
- *We excluded all rows in the dataset with a "lev_status_cd" value of "7" (corresponding to "Groundwater level affected by brackish or saline water")*
- *We excluded all rows in the dataset with a "lev_status_cd" value of "C" (corresponding to "Frozen")*
- *We excluded all rows in the dataset with a "lev_status_cd" value of "D" (corresponding to "Dry")*

- *We excluded all rows in the dataset with a "lev_status_cd" value of "O" (corresponding to "Obstructed")*
- *We excluded all rows in the dataset with a "lev_status_cd" value of "P" (corresponding to "Pumping")*

Third, we excluded one groundwater level measurement with a "lev_src_cd" value of "0" (implying this water level measurement was only accurate to the nearest foot: "Water level accuracy to nearest foot" quoting https://help.waterdata.usgs.gov/code/water_level_acc_cd_query?fmt=html). Fourth, we excluded groundwater level measurements with a Water-level Approval-Status Code of "P" – "Provisional data subject to revision". Fifth, upon examining recorded measurement dates within the dataset, we found a large number of measurements had a recorded date ("lev_dt" values) of January 1, 1900; we excluded these measurements from our compiled database (as we were uncertain if these recorded measurement dates in the dataset were accurate).

Dates that were reported to the nearest month were reported to the first day of the month. Similarly, dates that were reported to the nearest year were reported to the first day of the year. We downloaded station metadata (e.g., well depths, information about the types of aquifers (e.g., unconfined or confined) some of the monitoring well water level times series capture) on May 29, 2023 via https://waterdata.usgs.gov/nwis/inventory, and joined this station information to the well water level data via a join based on values in the field entitled "site_no".Two fields in the dataset provide information about the depths of wells – ("well_depth_va" and "hole_depth_va"). If a non-zero value was recorded for "well_depth_va" then we recorded this value as the estimated well depth; if no depth estimate was available via the field "well_depth_va"

but a non-zero value was recorded in the field “hole_depth_va”, we recorded this “hole_depth_va” as the estimate for the well depth.

Last, we excluded n=25 stations that lack decimal latitude and longitude values in the NWIS ‘Station Information’ portal (e.g., station number 485037104070301).

1.35 UNITED STATES OF AMERICA: TWDB

The Texas Water Development Board (TWDB) dataset was downloaded on May 29, 2023 via the website: http://www.twdb.texas.gov/groundwater/data/gwdbrpt.asp (specific link entitled "Full GWDB Database Download" – for direct download: http://www.twdb.texas.gov/groundwater/data/GWDBDownload.zip). A field is provided within the Texas Water Development Board database to highlight that the station is also contained within the 'National Water Information System' dataset described above (i.e., see 'NWIS' in the previous Supplementary Note 1.34); specifically, the Texas dataset contains a label entitled "USGSSiteNumber" that corresponds to the "site_no" for the corresponding monitoring well in the National Water Information System database. In order to avoid replication of sites that are within the Texas Water Development Board dataset and also within the National Water Information System, we identified these replicated sites (defined as sites where a non-blank value was listed in the field entitled "USGSSiteNumber" within the Texas Water Development Board database) and we excluded these time series from further analyses. We excluded measurements if the station identifier (field entitled "StateWellNumber") was not found in the well site information .csv file (entitled "WellMain.txt"). Measurement dates lacking daily resolution (i.e., year and month of measurement were recorded, but the "MeasurementDay" value is zero) were ascribed a measurement date of the middle of the month; Measurement dates lacking monthly resolution (i.e., the year the measurement was taken is available, but the "MeasurementMonth" value is zero) were ascribed to the beginning of the calendar year. We excluded all measurements where the value under the field entitled "Status" was "No measurement" or "Questionable". Further, we excluded all measurements with any one of the following values in the field entitled

‘Remarks’: “Access to well temporarily blocked due to winterization or debris”, or “Access to well temporarily blocked due to winterization or debris; Unable to insert tape into well”, or “Access to well temporarily blocked due to winterization or debris; Unable to insert tape into well; Well deleted from observation Program”, or “Accurately reflect water-level conditions; Inconsistent or spotty tape mark due to oil or gasoline”, or “Accurately reflect water-level conditions; Inconsistent or spotty tape mark due to oil or gasoline; Well or wells pumping nearby”, or “Accurately reflect water-level conditions; Well or wells pumping nearby”, or “Accurately reflect water-level conditions; Well pumped recently”, or “Airline tubing leaking air”, or “Airline tubing possibly leaking air”, or “Deleted as observation Well due to hazards to measure”, or “Deleted as observation Well due to owner request”, or “Deleted as observation Well due to owner request; Unable to reach water level with available measuring equipment”, or “Deleted as observation Well due to owner request; Well deleted from observation Program”, or “Deviation due to recompletion in different zone”, or “Discontinued - no reason stated (outside source)”, or “Inconsistent or spotty tape mark due to oil or gasoline”, or “Inconsistent or spotty tape mark due to oil or gasoline; Tape does not fall free in well”, or “Inconsistent or spotty tape mark due to oil or gasoline; Well deleted from observation Program”, or “Inconsistent or spotty tape mark due to oil or gasoline; Well pumped recently”, or “Inconsistent or spotty tape mark due to wet or leaking casing”, or “Inconsistent or spotty tape mark due to wet or leaking casing; Pumping-level measurement”, or “Inconsistent or spotty tape mark due to wet or leaking casing; Pumping-level measurement; Tape does not fall free in well”, or “Inconsistent or spotty tape mark due to wet or leaking casing; Tape does not fall free in well”, or “Inconsistent or spotty tape mark due to wet or leaking casing; Tape hangs before reaching water level”, or

“Inconsistent or spotty tape mark due to wet or leaking casing; Tape hangs before reaching water level; Well deleted from observation Program”, or “Inconsistent or spotty tape mark due to wet or leaking casing; Well deleted from observation Program”, or “Inconsistent or spotty tape mark due to wet or leaking casing; Well pumped recently”, or “Inconsistent or spotty tape mark due to wet or leaking casing; Well pumping”, or “Inconsistent or spotty tape mark due to wet or leaking casing; Wet or leaking casing”, or “measurement affected by atmospheric pressure”, or “Measurement before well completion”, or “Measurement deleted after review”, or “Measurement deleted after review; Measurement may be from incorrect well”, or “Measurement may be from incorrect well”, or “measurement may reflect perched water table”, or “No measurement due to COVID-19”, or “Observation well not measured due to admin decision”, or “Observation well not measured due to admin decision; Well deleted from observation Program”, or “Observation well not measured due to admin decision; Well site temporarily inaccessible due to impassable roads, locked gate, etc.”, or “Pumping-level measurement”, or “Pumping-level measurement; Tape does not fall free in well”, or “Pumping-level measurement; Tape hangs before reaching water level”, or “Pumping-level measurement; Uncertain of reason for questionable measurement”, or “Pumping-level measurement; Well pumped recently”, or “Tape does not fall free in well”, or “Tape does not fall free in well; Uncertain of reason for no measurement”, or “Tape does not fall free in well; Well or wells pumping nearby”, or “Tape does not fall free in well; Well pumped recently”, or “Tape hangs before reaching water level”, or “Tape hangs before reaching water level; Well apparently bridged or caved”, or “Tape hangs before reaching water level; Well apparently bridged or caved; Well deleted from observation Program”, or “Tape hangs before reaching water level;

Well deleted from observation Program", or "Tape hangs before reaching water level; Well pumping; Wet or leaking casing", or "Tape hangs before reaching water level; Well site temporarily inaccessible due to vicious animals", or "Tape hangs before reaching water level; Wet or leaking casing", or "Unable to insert tape into well", or "Unable to insert tape into well; Well deleted from observation Program", or "Unable to insert tape into well; Well site temporarily inaccessible due to vicious animals", or "Unable to locate well", or "Unable to locate well; Well deleted from observation Program", or "Unable to locate well; Well deleted from observation Program; Well destroyed", or "Unable to locate well; Well destroyed", or "Unable to locate well; Well site temporarily inaccessible due to impassable roads, locked gate, etc.", or "Unable to reach water level with available measuring equipment", or "Unable to reach water level with available measuring equipment; Well pumping", or "Uncertain of reason for no measurement", or "Uncertain of reason for questionable measurement", or "Uncertain of reason for questionable measurement; Well pumped recently", or "Well apparently bridged or caved", or "Well apparently bridged or caved; Well deleted from observation Program", or "Well apparently dry, well depth reached without water level", or "Well apparently dry, well depth reached without water level; Well deleted from observation Program", or "Well deleted from observation Program", or "Well deleted from observation Program; Well destroyed", or "Well deleted from observation Program; Well pumped recently", or "Well deleted from observation Program; Well pumping", or "Well deleted from observation Program; Well site temporarily inaccessible due to impassable roads, locked gate, etc.", or "Well deleted from observation Program; Wet or leaking casing", or "Well destroyed", or "Well or wells pumping nearby", or "Well or wells pumping nearby; Well pumped recently", or "Well or wells pumping

nearby; Well pumping”, or “Well pumped recently”, or “Well pumping”, or “Well pumping; Well site temporarily inaccessible due to vicious animals”, or “Well pumping; Wet or leaking casing”, or “Well site temporarily inaccessible due to impassable roads, locked gate, etc.”, or “Well site temporarily inaccessible due to impassable roads, locked gate, etc.; Well site temporarily inaccessible due to vicious animals”, or “Well site temporarily inaccessible due to vicious animals”, or “Wet or leaking casing”.

We received an email from a TWDB representative (March 15, 2023) requesting that we include the following statement in our work (the following italicised text is a direct quote from the email from a TWDB representative): “*GWDB DISCLAIMER: Except where noted, all of the information provided in the Texas Water Development Board (TWDB) Groundwater Database (http://www.twdb.texas.gov/groundwater/data/gwdbrpt.asp) is believed to be accurate and reliable; however, the TWDB assumes no responsibility for any errors appearing in rules or otherwise. Further, TWDB assumes no responsibility for the use of the information provided. PLEASE NOTE that users of these data are responsible for checking the accuracy, completeness, currency and/or suitability of all information themselves. TWDB makes no guarantees or warranties as to the accuracy, completeness, currency, or suitability of the information provided via the Groundwater Database (GWDB). TWDB specifically disclaims any and all liability for any claims or damages that may result from providing GWDB data or the information it contains.”*

1.36 UNITED STATES OF AMERICA: CALIFORNIA DWR

We downloaded Department of Water Resources (DWR) data from two repositories: (a) the ‘Periodic Groundwater Level Measurements’ database; and, (b) the ‘Continuous Groundwater Level Measurements’ database.

(a) First, we downloaded the "Periodic Groundwater Level Measurements" database from the California Department of Water Resources May 29, 2023 via https://data.ca.gov/dataset/periodic-groundwater-level-measurements. We excluded measurements where the water level accuracy was reported to be uncertain to the nearest foot (i.e., "wlm_acc_desc" values of "1 Ft" were excluded). Next, we excluded measurements with a quality control flag of "Questionable", "Provisional" or "Missing" (i.e., measurements with any of these three values in the field entitled "wlm_qa_desc" were excluded). We excluded one measurement with a recorded date (field entitled “msmt_date”) with a value of “1”. We found some stations in this “Periodic Groundwater Level Measurements” database (i.e., (a) described in this paragraph) may be replicates of stations within the “National Water Information System” (i.e., dataset “NWIS” described in Supplementary Note 1.34). We identified these stations based on matching values of “swn” (within the “National Water Information System” database) and “station_nm” (within the DWR dataset); specifically, we added a “0” to the beginning of the “swn” string and another zero between the third and the fourth characters in values of “swn” (i.e., a “swn” value of ‘16S22E27K002S’ became ‘016S022E27K002S’ – where underlined zeros represent the added characters). We then searched the “National Water Information System” for these adjusted “swn” values (i.e., with the additional zeros added), and we excluded any stations in the “Periodic

Groundwater Level Measurements” where we found the adjusted “swn” value within the “National Water Information System” database (i.e., we retained the time series from the “National Water Information System” and excluded the time series from the “Periodic Groundwater Level Measurements” database).

(b) We downloaded "Continuous Groundwater Level Measurements" database (specifically, the dataset entitled: "Groundwater Levels - Monthly Mean") from the California Department of Water Resources May 29, 2023 via https://data.ca.gov/dataset/continuous-groundwater-level-measurements. We excluded all monthly groundwater level data if any one of the following quality codes was reported (i.e., values in the field entitled "GSE_WSE_QC"): “15” (corresponding to “Provisional measurement”; see file entitled "gwl-quality_codes.csv" available for download via https://data.ca.gov/dataset/continuous-groundwater-level-measurements), or "70" (corresponding to "Estimated Data"), or “120” (corresponding to “Poor measurement”), or "130" (corresponding to the quality code "Estimate"), or "151" (corresponding to "'Data Missing'"), or “170” (corresponding to “Unreliable Data”), or “175” (corresponding to “Water level below sensor”), or “201” (corresponding to “Data not recorded”), or "255" (corresponding to “No data exists”). The majority of monitoring wells within the "Continuous Groundwater Level Measurements" database also appear within the “Periodic Groundwater Level Measurements” database (i.e., “(a)” described above). We excluded all stations within the "Continuous Groundwater Level Measurements" database that also appear within the “Periodic Groundwater Level Measurements” database (i.e., retaining data from the “Periodic Groundwater Level Measurements” database where sites are found in both databases). Replicated stations within the "Continuous Groundwater Level

Measurements" database were identified where a unique value under the heading “STATION” (in the continuous database) has an identical match in the field entitled (“continuous_data_station_number” within the “Periodic Groundwater Level Measurements” database). We preferentially retained data from the periodic groundwater level database (i.e., preferred over data from the continuous database) because these “Periodic Groundwater Level” time series tend to be longer than those within the “Continuous Groundwater Level” database.

1.37 UNITED STATES OF AMERICA: CALIFORNIA GAMA

We downloaded Groundwater Ambient Monitoring and Assessment Program (GAMA) data on June 12, 2023 from the following website: http://geotracker.waterboards.ca.gov/gama/datadownload. We excluded measurements that did not report both groundwater elevation and depth to water data (as the formatting of the primary database made it challenging to interpret which units a given measurement was reported in, for cases where only one measurement (i.e., either depth to water, or groundwater elevation) was reported). We excluded n=24 measurements where the recorded depth to water was "-999". We also excluded the following measurements, as many were deemed implausible and/or were highly inconsistent with other measurements recorded within the same monitoring well: (i) depth to water of 11150 ft. in station 04S11W24A004S, (ii) depth to water of "10300" in station 04S11W13F002S, (iii) depth to water of "7400" in station 04S10W25D001S, (iv) depth to water values of "6768" and 6754 in station 27S23E33J001M, (v) depth to water values ranging from "35392" to "90349" in several stations sharing the following beginning characters within their station identifier: "Environmental Monitoring Well T0606500029", (vi) depth to water values ranging from "40420" to "40420" in several stations sharing the following beginning characters within their station identifier: "Environmental Monitoring Well T0603783488", (vii) depth to water value "99999" in station "Environmental Monitoring Well SL206063824", (viii) depth to water value "87368" in station "Environmental Monitoring Well T0603704496", and (ix) depth to water value "57065" in station "Environmental Monitoring Well T0611100180". We excluded any well identifiers beginning with "DWR", to avoid potentially replicating sites contained in the dataset "United States of America: California DWR" described in the preceding section (i.e., Supplementary Note 1.36).

It remains possible that some of the monitoring wells in this GAMA database are replicates of wells within the NWIS database (see “United States of America: NWIS” subsection above). To estimate the potential prevalence of such cases (where a GAMA well may be a replicate of a monitoring well stored in the NWIS database), we completed a geospatial analysis and identified any unique GAMA monitoring well that is located within 10 m of one or more NWIS wells (based on the recorded latitudes and longitudes in each database); our geospatial analyses identified n=182 GAMA wells that are located within 10 m of at least one NWIS well. These n=182 wells account for 0.14% of all unique wells in the GAMA dataset (n=128,686 wells); therefore, even if a subset of these n=182 wells are replicates of wells within the NWIS dataset, our main findings are not sensitive to the inclusion of these wells. Further, we contacted a representative who manages the GAMA dataset (WB.GAMA@waterboards.ca.gov; correspondence June 28, 2023); they confirmed that (quote from email) “The depth to water file only includes the GeoTracker dataset (WB_CLEANUP) and DWR dataset because those are the only two datasets that report depth to water data.” (the dataset ‘WB_CLEANUP’ referenced in this email is described in GAMA documentation available via: https://gamagroundwater.waterboards.ca.gov/gama/GAMA_Data_Descriptions.pdf accessed July 4, 2023); given this information provided by a representative who manages the GAMA dataset, it appears that any widespread replication of NWIS sites in the GAMA database is unlikely.

1.38 VIETNAM

We downloaded groundwater level time series from http://123.16.176.41/quantractainguyennuoc/public/ January 22, 2023. Location data (latitude, longitude) for each well were retrieved from the map on the right-hand side of the webpage: http://123.16.176.41/quantractainguyennuoc/public/

2 GROUNDWATER LEVEL DATA PRE-PROCESSING

We completed a series of steps to quality control our piezometric dataset and analyse 21st century groundwater level trends. The following subsections detail steps we applied to:

i. *exclude potentially erroneous well water level measurements (see Supplementary Note 2.1 entitled "Excluding potentially erroneous well water level measurements"); and,*

ii. *calculate annual median groundwater levels (see Supplementary Note 2.2 – "Calculating annual median groundwater levels"); and,*

iii. *identify monitoring wells with a time series spanning a sufficient range of calendar years to characterize groundwater level trends for the 21st century (see Supplementary Note 2.3 – "Identifying records capturing 21st-century groundwater level trends").*

2.1 EXCLUDING POTENTIALLY ERRONEOUS WELL WATER LEVEL MEASUREMENTS

As detailed in the main text methods, we excluded (i) extreme values of depth to groundwater (i.e., >1,000 m and <−1,000 m) and implausibly high groundwater elevations (i.e., >8,000 m above sea level), and (ii) groundwater-level measurements with values of ‘999’, ‘-9999’, or ‘0’, because some databases used these values as a code for missing measurements (see figures on the following pages).

Further, we also excluded outlier values detected by a machine-learning algorithm (see ref.[1298]: Taylor, S.J., & Letham, B. Forecasting at scale. *The American Statistician* **72,** 37–45 (2018)). The algorithm is entitled “Prophet”, and is specifically designed to automate time series forecasting (for details: https://facebook.github.io/prophet/docs/quick_start.html#python-api; accessed July 25, 2023). It is based on an additive regression model (see ref.[1299]; Friedman, J.H., & Stuetzle, W. Projection pursuit regression. *Journal of the American statistical Association* **76,** 817–823 (1981)), where non-linear trends are fit with yearly, weekly, and daily seasonality, plus holiday effects (ref.[1298]). For our purposes, we do not use Prophet to forecast time series but, rather, we use Prophet to determine the confidence interval of the model, which is then compared to the actual measurements in our time series. The main advantage of Prophet over other machine-learning tools for time series forecasting is that it automates the forecast performance evaluation when determining an optimal model. Prophet only requires the time series (e.g., measurement date and groundwater level) and the number of iteration steps to optimize the model. In our case, we used 300 Monte-Carlo steps and 1000 uncertainty iterations (for further details for these parameters, see the Prophet manual:

https://facebook.github.io/prophet/docs/quick_start.html#python-api; accessed July 25, 2023). For each groundwater monitoring station, we first trained the machine-learning algorithm (i.e., Prophet) using the corresponding groundwater level (or groundwater elevation) time series, creating a prediction of the depth to groundwater time series (or groundwater elevation time series) over the same timespan Supplementary Figs. 1 and 2 depict a groundwater level time series (dark blue colours in supplementary figures to follow) together with the model prediction (violet in supplementary figures to follow). Excluded groundwater level values (i.e., those removed from the time series before applying the machine learning) are depicted as green points in Supplementary Figs. 1 and 2.

In addition to outputting a model prediction (which is shown as a dark purple line in Supplementary Figs. 1 and 2), Prophet also provides lower and upper confidence bounds for each prediction. Specifically, we use the 99% confidence bounds to calculate a band around the model prediction that allows us to classify the groundwater measurement values. We defined outliers as well water level measurements that fall outside of the range specified as plus (or minus) 0.75 times the 99% confidence interval (offset from the machine learning prediction; that is, the predicted value from the machine learning algorithm (dark purple line) plus-or-minus 0.75 times the 99 percent confidence interval; see light purple shading in Supplementary Figs. 1 and 2). These points (which fall outside of the range specified by the prediction plus or minus 0.75 times the 99% confidence interval) are depicted as orange circles in Supplementary Figs. 1 and 2.

This approach (i.e., machine-learning-based outlier detection method) was applied to each monitoring well with more than 15 groundwater-

level measurements, yielding a prediction for each time step and its 99% confidence interval; we defined points to be outliers and excluded them if they fell outside of the range defined by the predicted groundwater level ±0.75 times this confidence interval (see figures on the following pages). Later in this Supplementary Note 2 we compare (A) the aquifer-scale trends in depth to groundwater presented in the main text (which are based on groundwater level time series after we excluded measurements *with* the application of the machine learning algorithm), versus (B) aquifer-scale trends in depth to groundwater calculated *without* applying the machine learning algorithm to detect and exclude potential outlier measurements; these two aquifer-scale trends in depth to groundwater (i.e., (A) and (B) described in this paragraph) are similar for most aquifer systems.

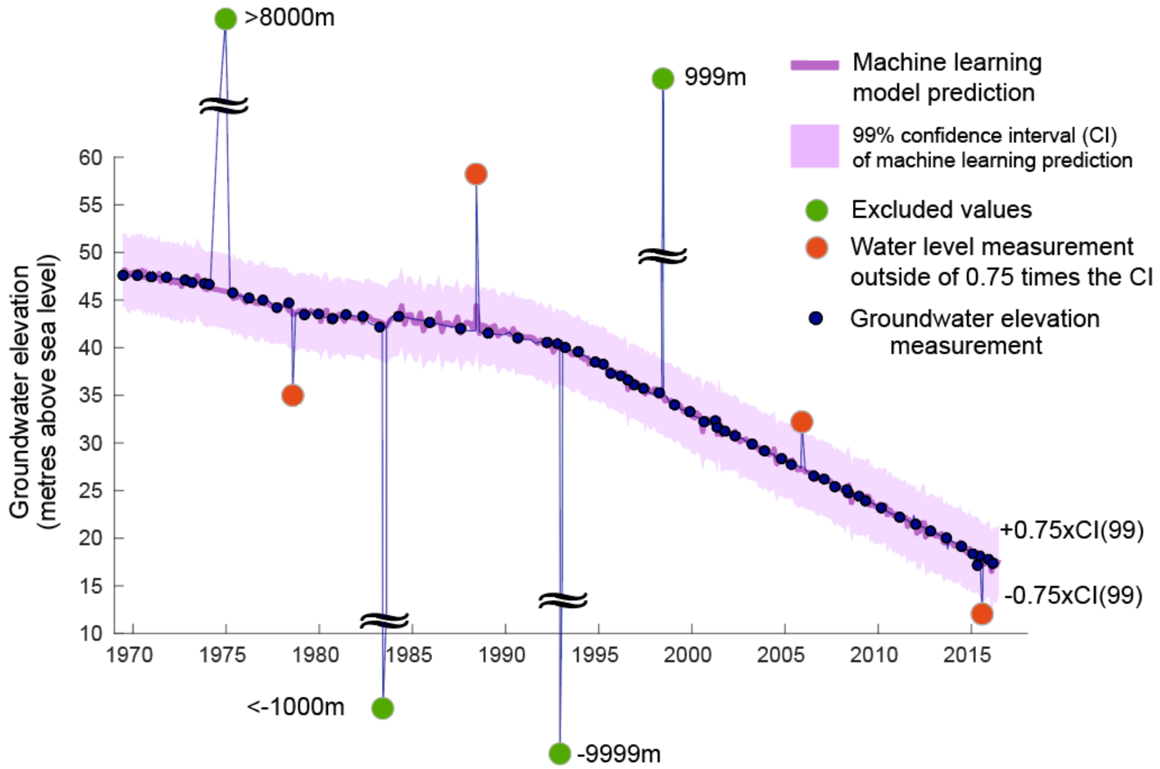

Supplementary Fig. 1. Schematic of a groundwater level time series and how outlier values were excluded (this figure differs from the one below as it plots a time series in units of <u>groundwater elevation</u> (not depth to groundwater). The dark blue circles and line represent the groundwater level time series (note: points within this time series were generated by us for illustrative purposes). Green circles represent points that were excluded because their groundwater elevation value exceeded 8000m (label ">8000m"), was less than 1000m below sea level (see label "<-1000m"), or were exactly 999 or -9999 (see labels "999m" and "-9999m"). The thick purple line represents the predicted groundwater level time series (based on a machine learning algorithm: see ref.[1298] Taylor, S.J., & Letham, B. Forecasting at scale. *The American Statistician* **72,** 37–45 (2018)). The light purple shaded area represents an offset from the prediction (i.e., a constant offset along the y-axis away from the purple line) of a magnitude of the 99% confidence interval (CI) multiplied by a factor of 0.75 (i.e., we calculated the 99% confidence interval, multiplied by a factor of 0.75, and then plotted this offset above (top of light purple shading; see label "+0.75xCI(99)", corresponding to an addition of 0.75 times the 99% confidence interval from the machine learning model prediction) and below (bottom of light purple shading; see label "-0.75xCI(99)", corresponding to a subtraction of 0.75 times the 99% confidence interval from the machine learning model prediction). The red circles represent points that fall outside of the light purple shading (i.e., the measurement was flagged as an outlier by the machine learning algorithm and our threshold for outliers of 0.75 times the 99% confidence interval).

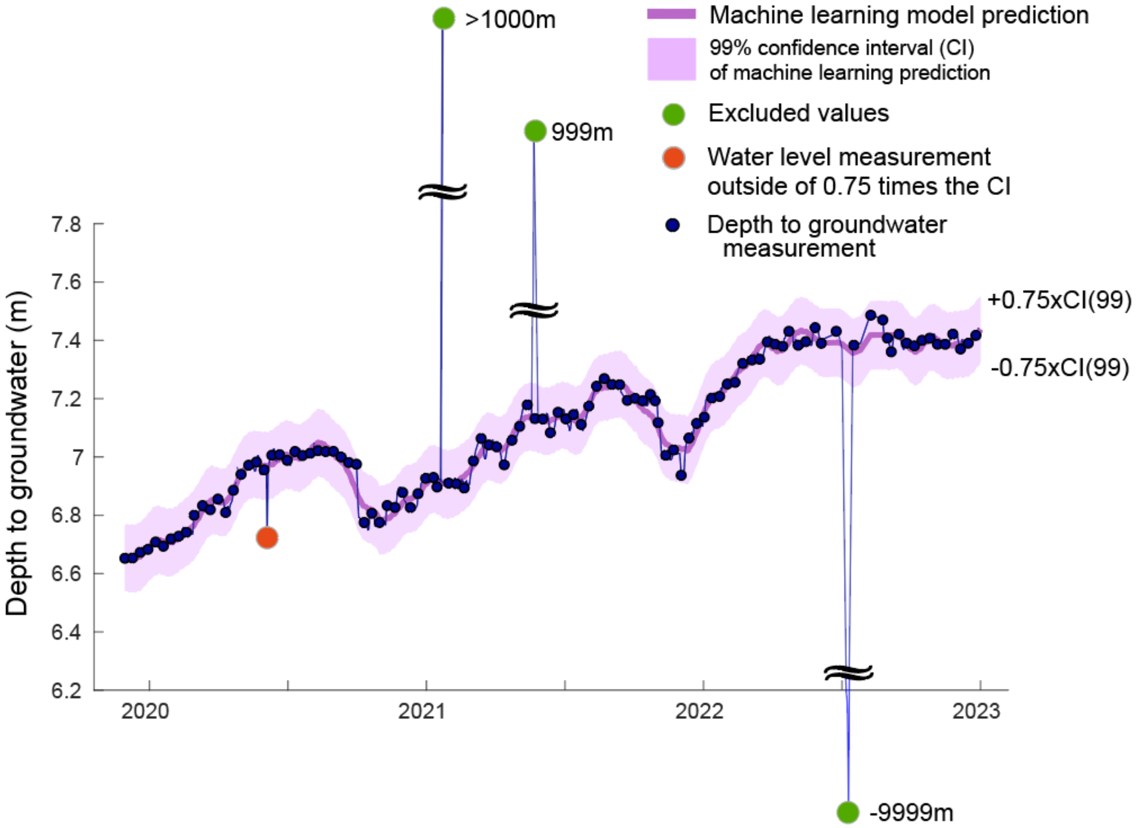

Supplementary Fig. 2. Schematic of a groundwater level time series and how outlier values were excluded (this figure differs from the one above as it plots a time series in units of <u>depth to groundwater</u> (not groundwater elevation). The dark blue circles and line represent the groundwater level time series (note: points within this time series were generated by us for illustrative purposes). Green circles represent points that were excluded because their depth to groundwater values exceeded 1000m (label ">1000m"), or were exactly 999 or -9999 (see labels "999m" and "-9999m"). The thick purple line represents the predicted groundwater level time series (based on a machine learning algorithm: see ref.[1298]: Taylor, S.J., & Letham, B. Forecasting at scale. *The American Statistician* **72,** 37–45 (2018)). The light purple shaded area represents an offset from the prediction (i.e., a constant offset along the y-axis away from the purple line) of a magnitude of the 99% confidence interval (CI) multiplied by a factor of 0.75 (i.e., we calculated the 99% confidence interval, multiplied by a factor of 0.75, and then plotted this offset above (top of light purple shading; see label "+0.75xCI(99)", corresponding to an addition of 0.75 times the 99% confidence interval from the machine learning model prediction) and below (bottom of light purple shading; see label "-0.75xCI(99)", corresponding to a subtraction of 0.75 times the 99% confidence interval from the machine learning model prediction). The red circle represents a point that falls outside of the light purple shading (i.e., the measurement was flagged as an outlier by the machine learning algorithm and our threshold for outliers of 0.75 times the 99% confidence interval).

2.2 CALCULATING ANNUAL MEDIAN GROUNDWATER LEVELS

We calculated annual median groundwater levels (i.e., the median of all groundwater level measurements made for a given station, for a given year). The following figure presents a walkthrough of the steps applied to calculate annual median groundwater levels.

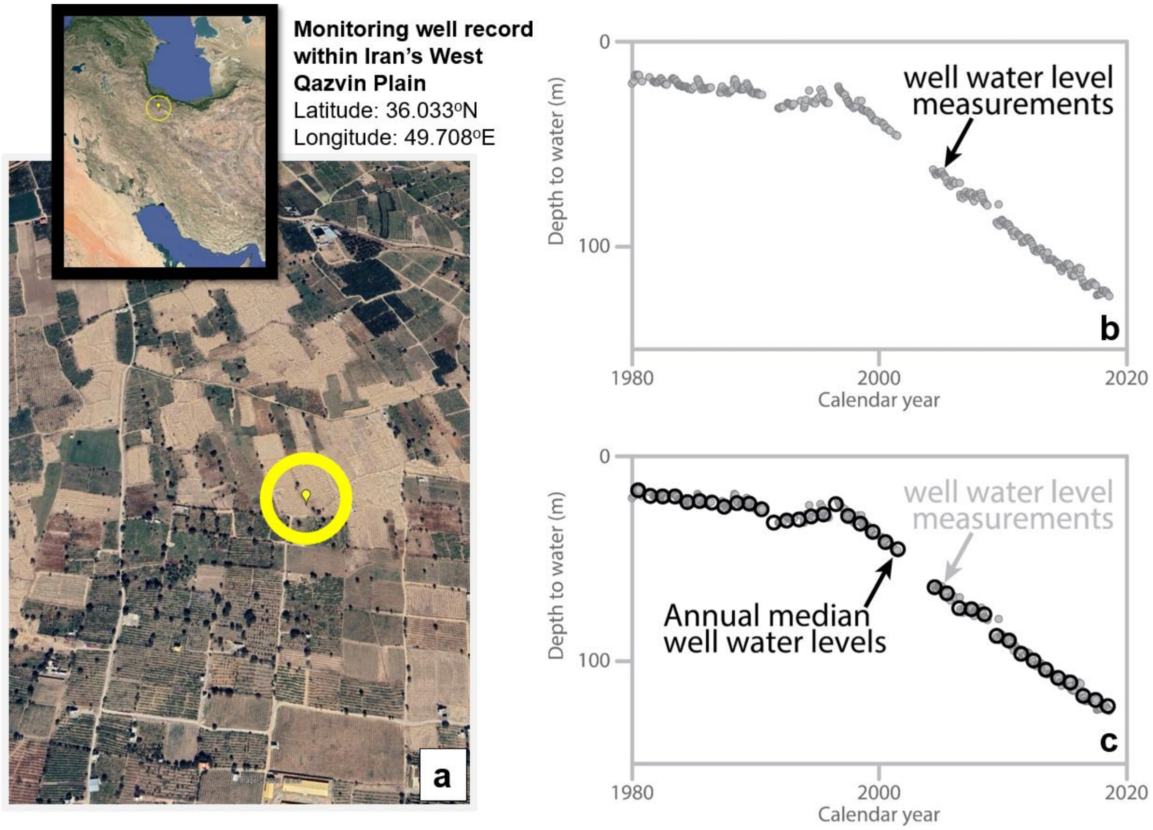

Supplementary Fig. 3. Calculation of annual median groundwater levels from well water level time series. (a) Location of a monitoring well in northern Iran (monitoring well is located within the boundaries of the West Qazvin Plain aquifer system). (b) Time series of groundwater levels recorded at this monitoring well. Each grey circle represents one recorded well water level measurements (units on y-axis are meters below the land surface). X-axis values represent the measurement date. (c) Calculation of annual median groundwater levels. Each black circle represents the median well water level for a given calendar year. The original time series of well water level measurements are presented as grey circles displayed behind the black circles. The black circles are equally spaced (one point per calendar year) with the exception of missing values (for calendar years during which no well water level measurements were made). The figure exemplifies the discontinuous nature of 21st-century groundwater level time series evident in (see gap in groundwater level measurements for the year 2003, for sample, in panels b and c). Such discontinuities occur in 41% of the monitoring wells presented in main text Fig. 1. Satellite imagery in panel a from https://www.arcgis.com/home/item.html?id=10df2279f9684e4a9f6a7f08febac2a9

2.3 IDENTIFYING RECORDS CAPTURING 21ST-CENTURY GROUNDWATER LEVEL TRENDS

We identified monitoring wells that capture 21st century trends in groundwater levels following a suite of criteria.

First, we excluded monitoring wells with just one water level measurement (rendering these wells unhelpful for examining changes in groundwater levels over time, as they have just a single snapshot of the groundwater level at a single point in time), *or* reporting groundwater level measurements that were all made during the same calendar year (rendering these monitoring wells unhelpful for examining changes in groundwater levels over multiple calendar years). This step – i.e., excluding monitoring wells where the water level time series takes place entirely within just one calendar year – excluded ~672 thousand monitoring wells (see figure below).

Second, we excluded monitoring wells that lack water level measurements made in two different calendar years during the 21st century (rendering these monitoring wells unhelpful for examining changes in groundwater levels over multiple calendar years in the 21st century). This step excluded ~219 thousand monitoring wells.

Third, we excluded monitoring wells where the earliest and latest calendar year (in the 21st century) where the monitoring well time series reports at least one measurement are separated by less than 8 years. This step – i.e., excluding monitoring wells where the 21st century time series spans fewer than 8 calendar years – excluded a further ~170 thousand wells (Supplementary Fig. 4; note: we present alternate results based on less-stringent thresholds (minimum of 5 years or minimum of 3 years separating the earliest and most-recent calendar years in a time series) in Supplementary Note 6).

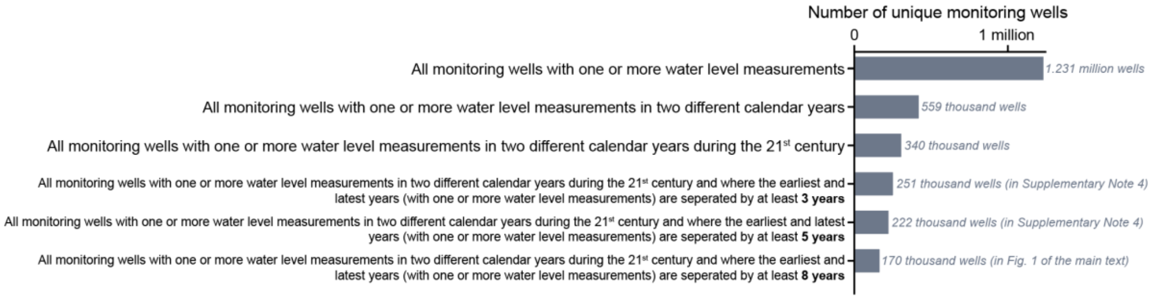

Supplementary Fig. 4. Monitoring wells in the compiled database that meet a suite of different criteria. (top row) There are 1.23 million monitoring wells in our dataset that report at least one well water level measurement. (second row). But, of these 1.23 million wells, only ~45% (559 thousand wells) report at least one water level measurement in two different calendar years. (third row) Furthermore, only 28% (340 thousand wells) report at least one water level measurement in two different calendar years during the 21st century. (fourth row) Of these monitoring wells, only 251 thousand report at least one well water level measurement in at least two different 21st-century calendar years that are separated by at least 3 years. (fifth row) Of these monitoring wells, only 222 thousand report at least one well water level measurement in at least two different 21st-century calendar years that are separated by at least 5 years. (bottom row) Of these monitoring wells, only 170 thousand report at least one well water level measurement in at least two different 21st-century calendar years that are separated by at least 8 years (results for these monitoring wells are presented in main text Fig. 1, and these 170 thousand monitoring wells form the foundation of the analyses presented in the main text). For a comparison of aquifer-scale trends in depth to groundwater presented in the main text versus results that are based on a less-stringent criteria (i.e., the earliest and most-recent 21st century calendar years with at least one groundwater level measurement must be separated by at least 3 years, or at least 5 years, see Supplementary Note 6. We stress that many of these monitoring wells do not have a complete time series of annual groundwater levels (that is, one or more calendar years between the earliest and most-recent calendar years in the time series lack a groundwater level measurement; ~41% of the monitoring wells have such discontinuities.

3 DIFFERENT REGRESSION APPROACHES YIELD SIMILAR GROUNDWATER LEVEL TRENDS

We compared 21st century trends in depth to groundwater obtained from different regression techniques. Specifically, we compared trends of depth to groundwater over time using (i) Theil-Sen robust regression (basis of main text results) versus trends obtained from (ii) RANdom SAmple Consensus (RANSAC) regression, (iii) iteratively reweighted least squares regression, or (iv) ordinary least squares regression.

(i) We calculated Theil-Sen robust regressions for each monitoring well with sufficient data for analyses (see main text methods for definitions of "sufficient data for analyses"). In cases where just two data points were available, we calculated the slope (change in groundwater level over time) from these two data points. For further reading on this regression approach see ref.[1300]: Theil, H. A rank-invariant method of linear and polynomial regression analysis. *Indagationes mathematicae* **12,** 386-392 (1950).

(ii) We calculated regression slopes for each monitoring well following RANdom SAmple Consensus (RANSAC) linear regression (ref.[1305]). RANSAC excludes outliers (defined as data points that are far from the other data points) by randomly sampling the data and categorising points as inliers or outliers, and then determining a median absolute deviation to select points as inliers with each iteration of the trained

model. The process iterates, retraining the model with a new set of inliers, until the model fit ceases to change substantially.

(iii) We calculated the slope of the regression determined via iteratively reweighted least squares for each monitoring well with sufficient data for analyses. We used the method described by Kirchner (ref.[1303]: Kirchner, J.W. Quantifying new water fractions and transit time distributions using ensemble hydrograph separation: theory and benchmark test. *Hydrology and Earth System Sciences* **23,** 303–349 (2019)) and code provided by ref.[1304]: Kirchner, J.W. & Knapp, J.L.A. Technical note: Calculation scripts for ensemble hydrograph separation. *Hydrology and Earth System Sciences* **24,** 5539–5558 (2020). In cases where just two data points were available, we calculated the slope manually (change in groundwater level over time). In rare cases (a few stations among the ~170,000 that we analysed) this code did not converge on a solution. We do not present results for these monitoring wells in the plot below.

(iv) We calculated ordinary linear regression slopes for each monitoring well meeting our minimum criteria for statistical analyses.

We report Theil-Sen slopes in the main text of this work; results obtained with three other regression approaches (iteratively reweighted least squares, ordinary least squares, RANSAC) are generally similar to those reported in the main text (Theil-Sen) for most of monitoring wells in our dataset (see figures on following pages).

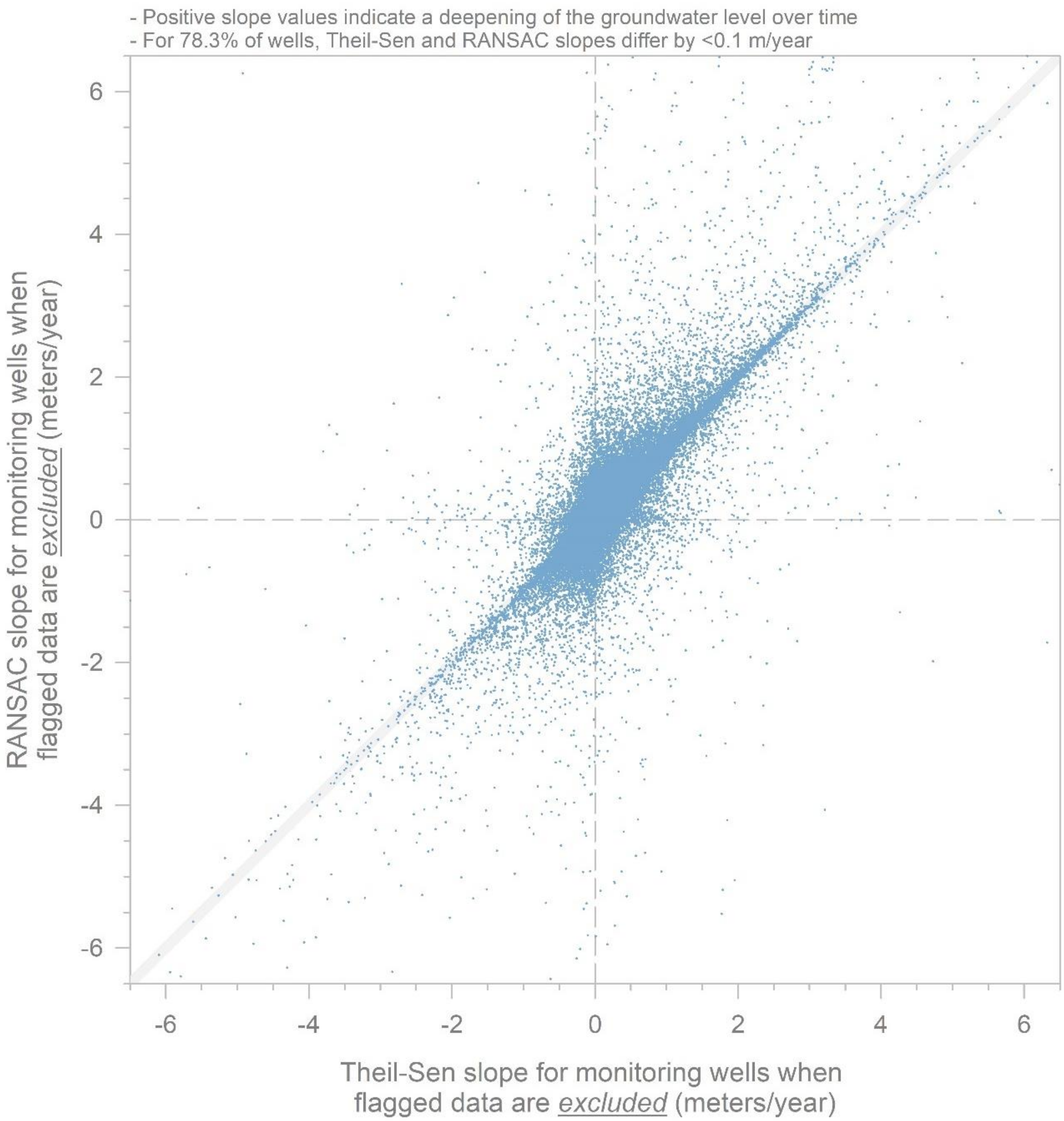

Supplementary Fig. 5. Comparison of slope coefficients describing groundwater level trends over time when determined with Theil-Sen and RANSAC regression approaches. Each data point represents one monitoring well. Points within the light grey shaded area have x-axis and y-axis values that differ by less than 0.1 m/year. 78.3% of monitoring wells have RANSAC and Theil-Sen slopes that differ by less than 0.1 m/year.

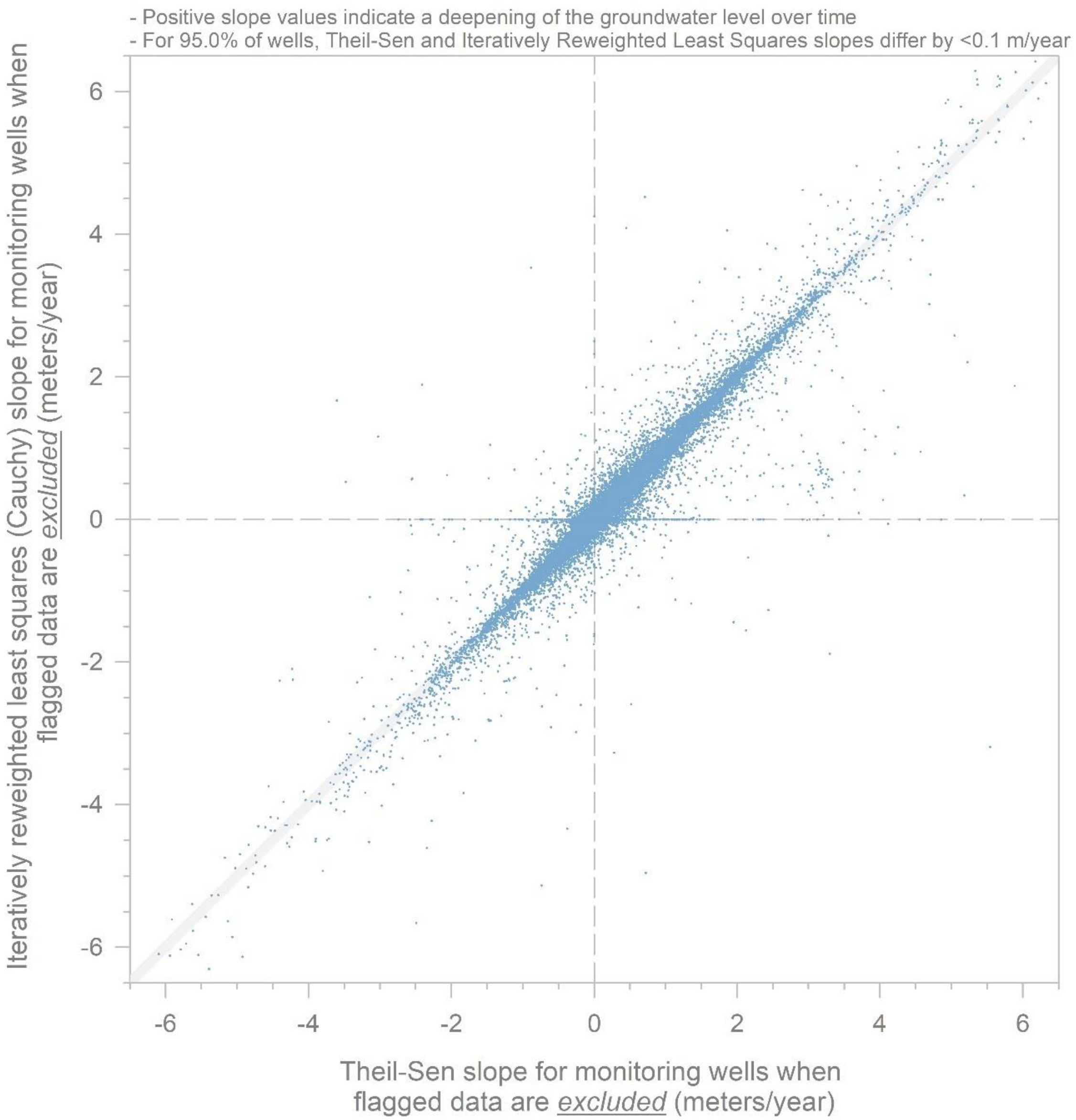

Supplementary Fig. 6. Comparison of slope coefficients describing groundwater level trends over time when determined with Theil-Sen and iteratively reweighted least squares (Cauchy weighting scheme) regression approaches. Each data point represents one monitoring well. Points within the light grey shaded area have x-axis and y-axis values that differ by less than 0.1 m/year. 95.0% of monitoring wells have iteratively reweighted least squares and Theil-Sen slopes that differ by less than 0.1 m/year.

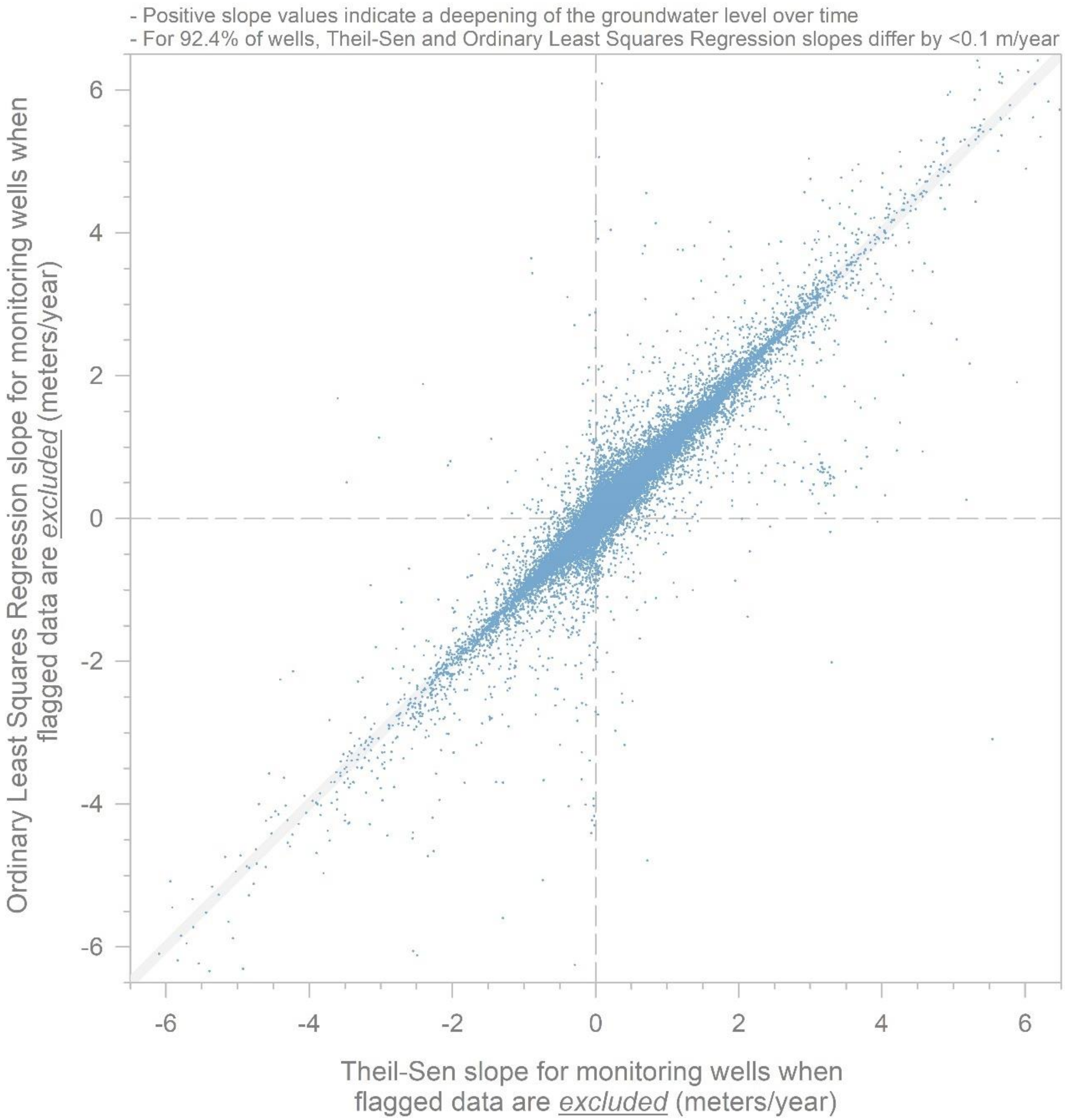

Supplementary Fig. 7. Comparison of slope coefficients describing groundwater level trends over time when determined with Theil-Sen and ordinary least squares regression approaches. Each data point represents one monitoring well. Points within the light grey shaded area have x-axis and y-axis values that differ by less than 0.1 m/year. 92.4% of monitoring wells have ordinary least squares and Theil-Sen slopes that differ by less than 0.1 m/year.

4 NON-PARAMETRIC REGRESSION APPROACHES APPLIED TO GROUNDWATER LEVEL TIME SERIES

We analysed 21st century changes over time in depth to groundwater via three approaches, all of which involve non-parametric regression techniques. Specifically, we characterized changes over time in annual median depths to groundwater – for each monitoring well with adequate data for analyses – by calculating: (i) Spearman rank correlation coefficients; and, (ii) Mann-Kendall correlation coefficients.

We cannot straightforwardly compare a correlation coefficient from a non-parametric regression (e.g., Spearman ρ) against the Theil-Sen slopes presented in our main text. Instead, we report on the proportion of monitoring wells with consistent signs in trends determined via Theil-Sen versus nonparametric regression (e.g., the sign (positive versus negative) of Spearman ρ values and the sign (positive versus negative) of Theil-Sen slopes). We also present a plot comparing Mann-Kendall and Spearman rank correlation coefficients. These results are presented in the following pages.

Supplementary Table 2. Comparison of signs of Theil-Sen slopes (21st century trends in depth to groundwater) versus Spearman rank correlation coefficients. The percentages in the table refer to the proportion of monitoring wells falling within a given category (e.g., 61% of monitoring wells have positive Theil-Sen slopes and *also* positive Spearman ρ values)

	**Negative Theil-Sen slope**	**Positive Theil-Sen slope**
**Positive Spearman ρ**	1%	61%
**Negative Spearman ρ**	37%	2%

* we excluded 1% of monitoring wells with undefined or zero slopes (and/or correlation coefficients)

Supplementary Table 3. Comparison of signs of Theil-Sen slopes (21st century trends in depth to groundwater) versus Mann-Kendall correlation coefficients (tau). The percentages in the table refer to the proportion of monitoring wells falling within a given category (e.g., 62% of monitoring wells have positive Theil-Sen slopes and *also* positive Kendall τ values)

	**Negative Theil-Sen slope**	**Positive Theil-Sen slope**
**Positive Kendall τ ** **	0.02%	62%
**Negative Kendall τ ** **	38%	0.03%

* we excluded 1.8% of monitoring wells with undefined or zero slopes (and/or correlation coefficients)

** these values based on regression analyses without bootstrapping

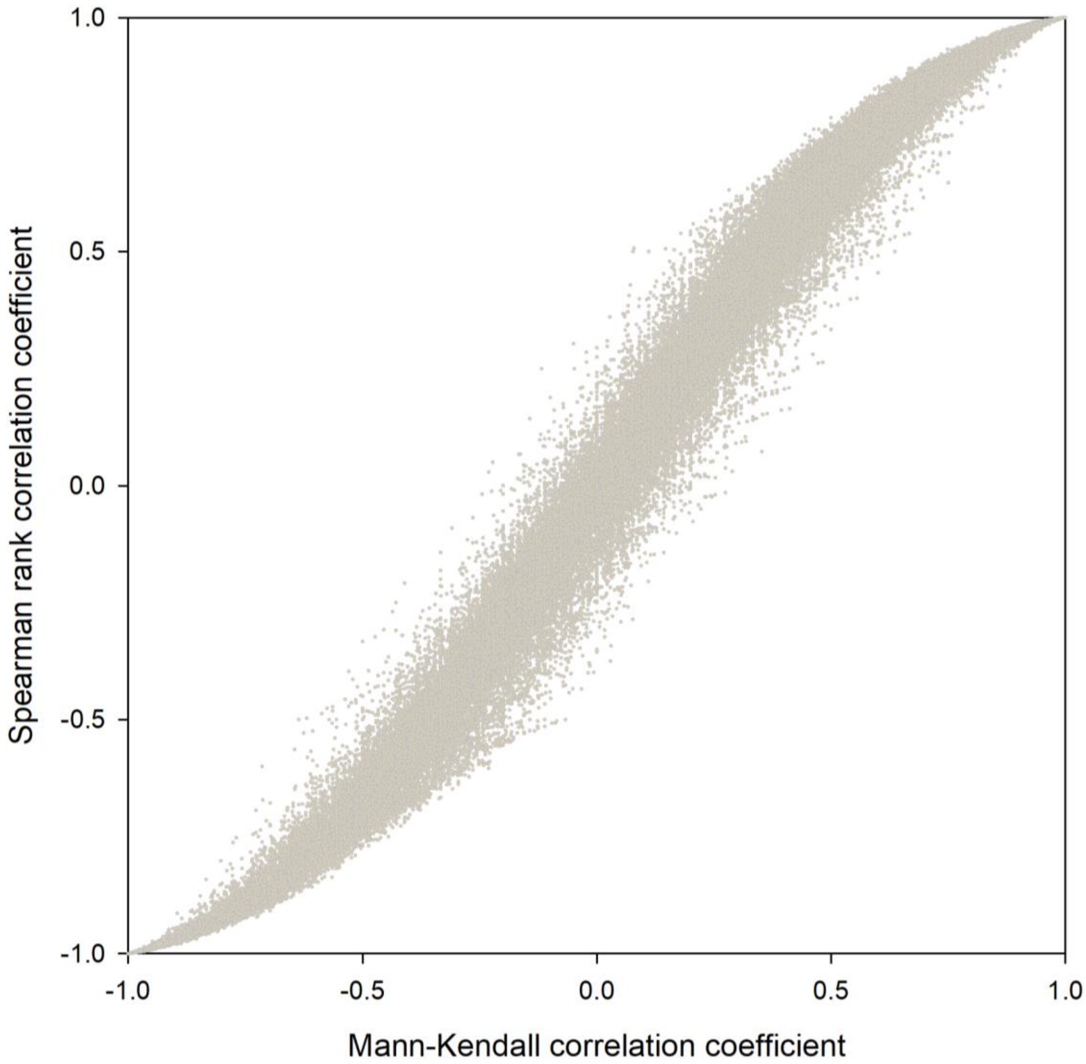

Supplementary Fig. 8. Comparison of correlation coefficients based on Mann-Kendall and Spearman rank regressions. Each data point represents one monitoring well and its 21st century variability in depth to groundwater (expressed as non-parametric correlation coefficients). Positive values indicate deepening groundwater levels over time, whereas negative values indicate shallowing of groundwater levels over time. Outputs of the two regression techniques agree in the majority of cases.

We also calculated Mann-Kendall correlation coefficients following a block bootstrapping approach (see figure below). We only completed this bootstrapping approach for stations with more than 15 unique calendar years during the 21st century with at least one water level measurement. In the overwhelming majority of cases, the non-bootstrapped Mann-Kendall correlation coefficient matched the bootstrapped Mann-Kendall correlation coefficient within 0.1 (99.88% of monitoring wells; see figure below). For further details on the bootstrapping approach see ref.[1306] (i.e., Önöz, B., & Bayazit M. Block bootstrap for Mann-Kendall trend test of serially dependent data. *Hydrological Processes* **26,** 3552-3560 (2012)).

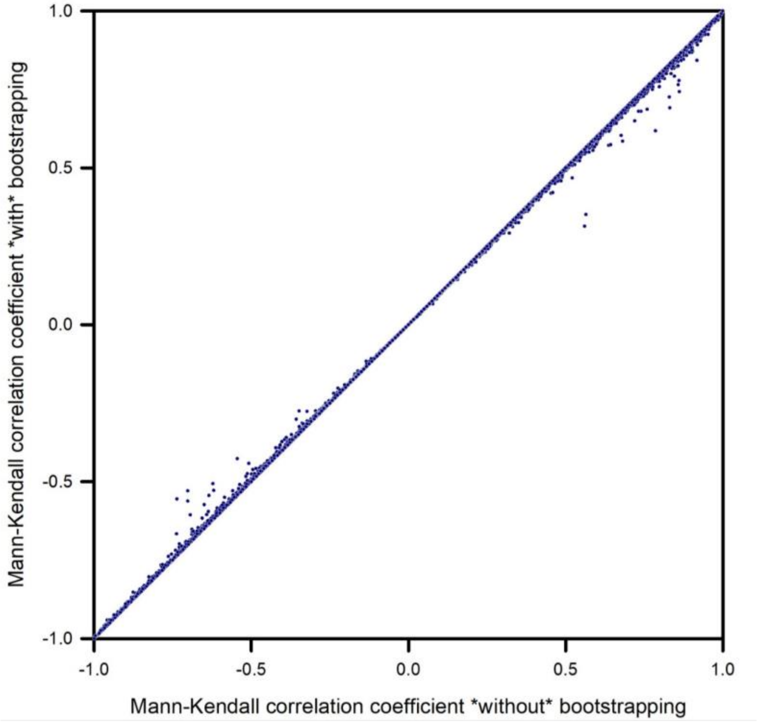

Supplementary Fig. 9. Comparison of Mann-Kendall correlation coefficients without bootstrapping (x-axis values) versus with bootstrapping (y-axis values). Each data point represents one monitoring well and its 21st century variability in depth to groundwater (expressed as non-parametric correlation coefficients). Positive values indicate deepening groundwater levels over time, whereas negative values indicate shallowing of groundwater levels over time. Mann-Kendall correlation coefficients determined with versus without bootstrapping closely match one another in the vast majority of cases.

5 SIMILAR AQUIFER-SCALE TRENDS OBTAINED WITH AND WITHOUT FLAGGED MEASUREMENTS

We compared aquifer-scale trends in depth to groundwater (median of the Theil-Sen slopes of all monitoring wells within the boundary of an aquifer system) for two scenarios:

(i) *<u>Excluding</u> annual median groundwater levels that were flagged on the basis of rapid increases (or decreases) in groundwater levels over time ('rapid' defined as a change of more than 20 m/y; see main text Methods section entitled "Flagging groundwater-level measurements based on rapid increases or decreases")*

(ii) *<u>Including</u> annual median groundwater levels that were flagged on the basis of rapid increases (or decreases) in groundwater levels over time ('rapid' defined as a change of more than 20 m/y; see main text Methods section entitled "Flagging groundwater-level measurements based on rapid increases or decreases")*

Aquifer-scale groundwater-level trends are largely insensitive to our decision to exclude groundwater level measurements that were flagged on the basis of rapid increases (or decreases) in groundwater levels over time (see the following figure).

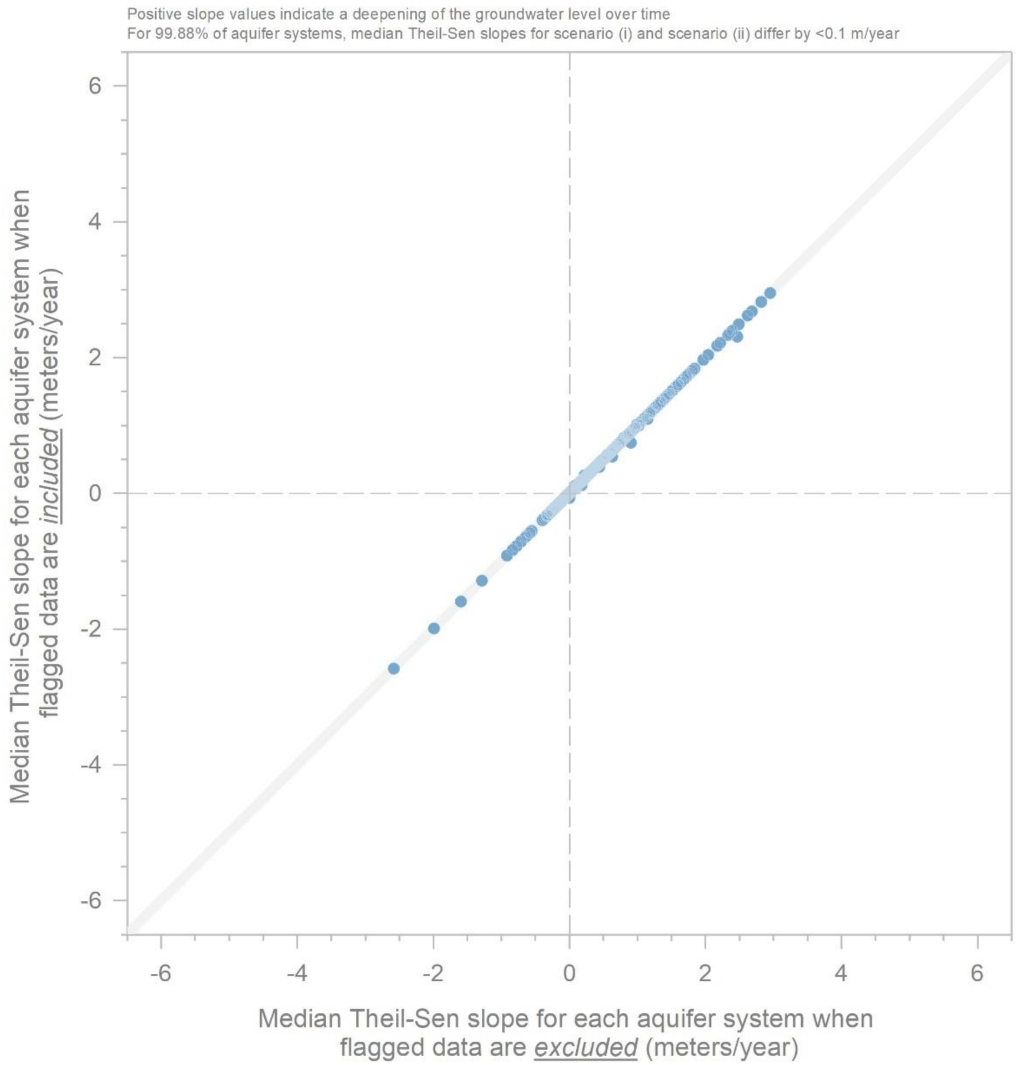

Supplementary Fig. 10. Aquifer-scale trends in depth to groundwater (median Theil-Sen slopes) after excluding flagged data points (x-axis value) versus including flagged data points (y-axis value). Each circle represents one aquifer system. Points within the light grey area shaded area have x-axis and y-axis values that differ by less than 0.1 m/year. 99.5% of aquifer-scale trends in depth to groundwater differ by less than 0.1 m/year under scenario (i) versus scenario (ii) (for explanation of scenario (i) and (ii) see text above the figure).

6 SENSITIVITY ANALYSIS OF AQUIFER-SCALE TRENDS TO MINIMUM LENGTH OF TIME-SERIES

Our main text (e.g., Figs. 1 and 2) focuses on groundwater level trends during the early 21st century. One of the criteria used to exclude monitoring wells with insufficiently long time series is that the first and the most-recent calendar year in the time series must be separated by eight years. Here, we present groundwater level trends for a variety of other time intervals, and apply a less stringent threshold for exclusion of monitoring wells (with respect to the temporal offset between the earliest and most-recent groundwater level measurements for a given time interval). The following table details these additional trend analyses and the minimum temporal offset between the earliest and most-recent groundwater level measurements for a given time interval (in order for a well to be included in the analysis).

Supplementary Table 4. Additional trends in depth to groundwater presented in this section

Time interval	Minimum temporal offset between the earliest and most-recent measurements in the time series	Number of monitoring wells meeting criteria for analyses	Figure where these results are presented
2000-2022	8 years	169,717	*main text Figs.
2000-2022	5 years ("alternate scenario A")	221,521 (pink in map below)	Fig. below
2000-2022	3 years ("alternate scenario B")	256,407 (yellow in map below)	Fig. below

* for context, this row of the table provides information about the number of wells meeting our criteria for analyses as presented in the main text

There are noteworthy differences in the spatial patterns and densities of monitoring wells meeting these three different criteria (i.e., minimum temporal offsets between the earliest and most-recent 21st century measurements in the time series of (i) 3 years, (ii) 5 years, or (iii) 8 years). For example, there are areas in South Asia (specifically, west central and eastern portions of South Asia) where many monitoring wells have adequate data for analyses of groundwater level trends over 5-7 years, but not for >8 year-long time intervals (i.e., red points in the following figure). Groundwater supplies play a key role in this region. Because the analyses on our main text are based solely on monitoring wells where the minimum temporal offset between the earliest and most-recent 21st century measurement is at least 8 years, many monitoring wells in these regions are excluded from our statistical analyses presented in the main text. However, our comparison of aquifer-scale trends in depth to groundwater using different thresholds for the minimum temporal offset between the earliest and most-recent 21st century measurement (Supplementary Figs. 12 and 13) suggest that, for the great majority of our studied aquifer systems, median aquifer-scale Theil-Sen slopes are largely unchanged when we reduce the stringency of our filtering criteria.

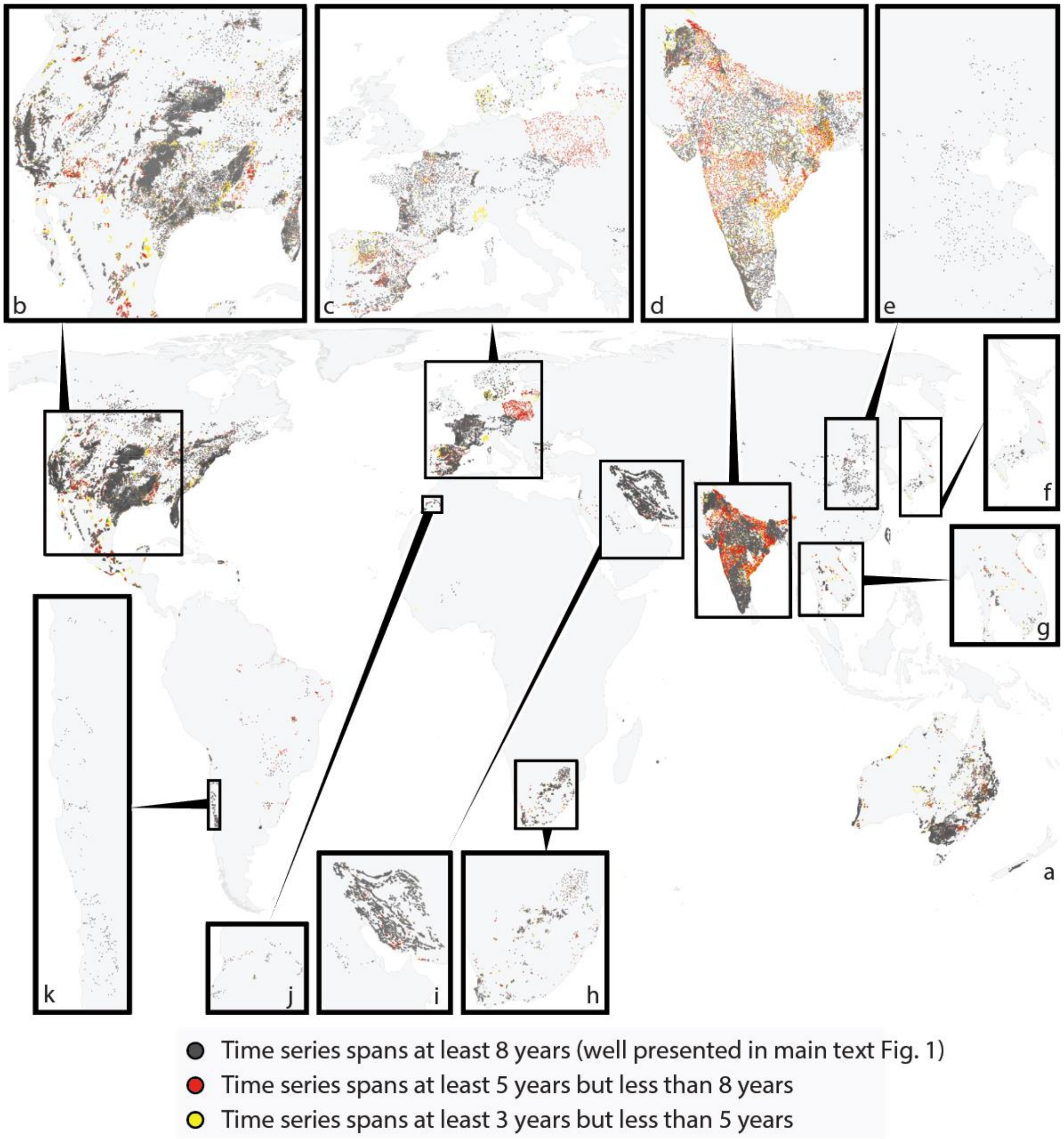

Supplementary Fig. 11. Groundwater monitoring wells meeting varying criteria for analyses. Dark grey points represent the n=169,717 monitoring wells meeting our criteria for analyses in the main text (i.e., these are the ~170 thousand points plotted in Fig. 2 and that form the basis for the aquifer-scale statistics presented in main text Fig. 2; these monitoring wells have 21st century groundwater level time series with at least 8 years between the earliest and most-recent 21st-century annual median groundwater level). Pink points represent monitoring wells that did not have an adequate number of years for inclusion in our main text Fig. 1, but do have groundwater level time series with at least 5 years separating the earliest and most-recent groundwater level measurement in the 21st century (see "scenario A" in supplementary table above). Yellow points represent monitoring wells that did not have an adequate number of years for inclusion in our main text Fig. 1 nor enough for inclusion in our "alternative scenario A", but do have groundwater level time series with at least 3 years separating the earliest and most-recent groundwater level measurement in the 21st century (see "scenario B" in supplementary table above). **a)** Global map. **b-k)** Maps for individual regions. Overall, including groundwater level time series that span fewer years than the sites presented in main text (i.e., the locations with pink and yellow in this map that do not have grey points) does not add considerably to the spatial coverage of monitoring wells; nevertheless, the few regions that are not represented in our main text results but could have been, if we had made our minimum criteria for a monitoring well to be studied less stringent (e.g., lowering the minimum number of years separating the earliest and most-recent annual median groundwater level from 8 years to either 5 or 3 years), include Poland, Lithuania and northern Italy.

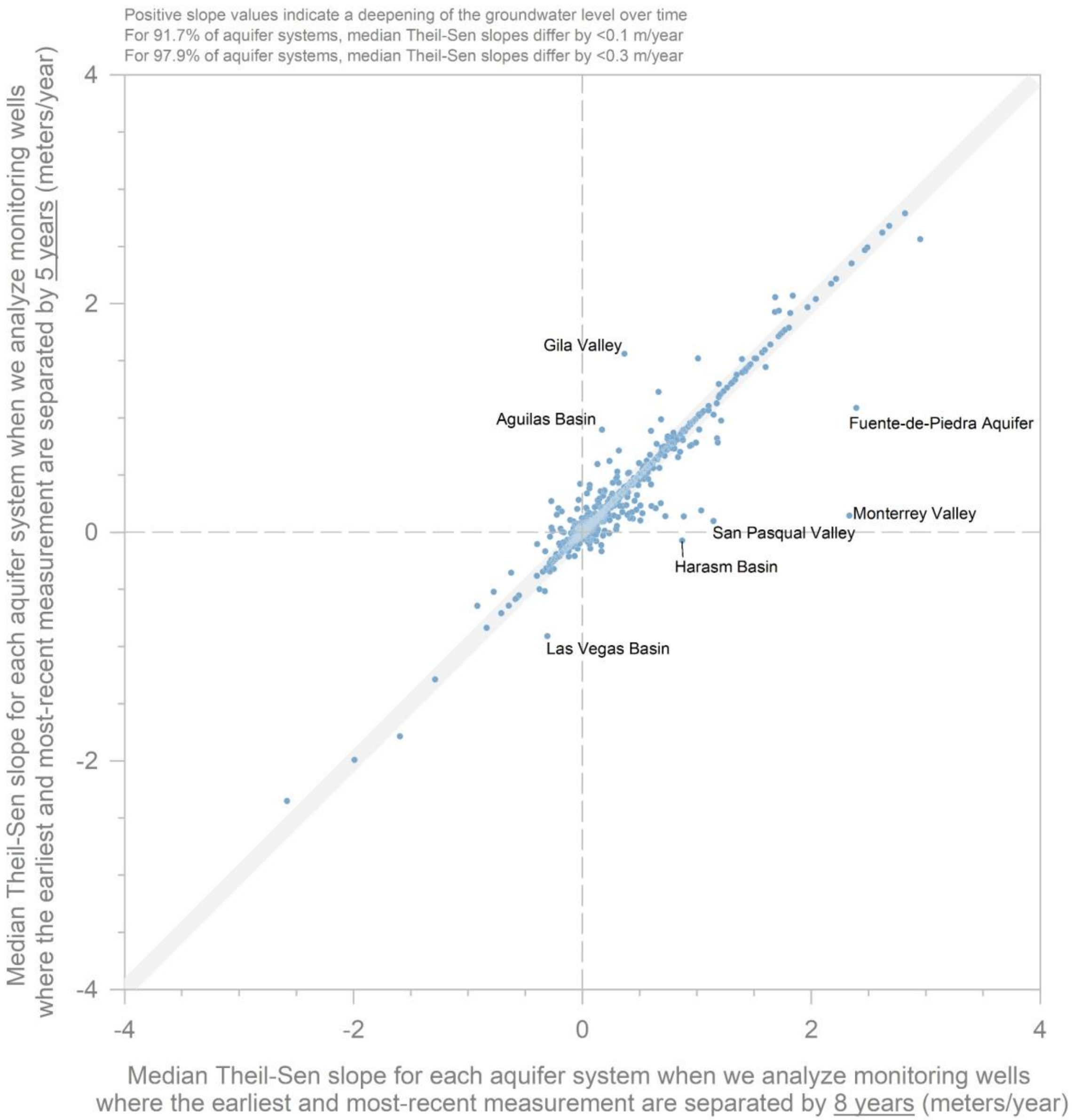

Supplementary Fig. 12. Sensitivity of aquifer-scale trends in depth to groundwater to the selection of a minimum number of years separating the earliest and most-recent annual median groundwater level (5 years versus 8 years presented here). Each circle represents one aquifer system and its 21st century trends in depth to groundwater (median Theil-Sen slope of monitoring wells within the boundaries of the aquifer system). The x-axis values are the aquifer-scale trends in depth to groundwater presented in the main text (e.g., Fig. 2). Points on the y-axis are aquifer-scale trends in depth to groundwater if we reduce the stringency of our criteria to include a monitoring well in our analyses, specifically by reducing the minimum temporal offset between the earliest and most-recent calendar year with at least one measurement from 8 years (criteria for inclusion in the main text) to, instead, just 5 years. Points within the light grey area shaded area have x-axis and the y-axis values that differ by less than 0.1 m/year. Overall, the great majority of aquifer-scale trends in depth to groundwater do not change substantially when we reduce our minimum temporal offset from 8 years to 3 years, as evidenced by the 91.7% of aquifer-scale trends in depth to groundwater that differ by less than 0.1 m/year (points within the light grey diagonal band) and the 97.9% of aquifer-scale trends in depth to groundwater that differ by less than 0.3 m/year.

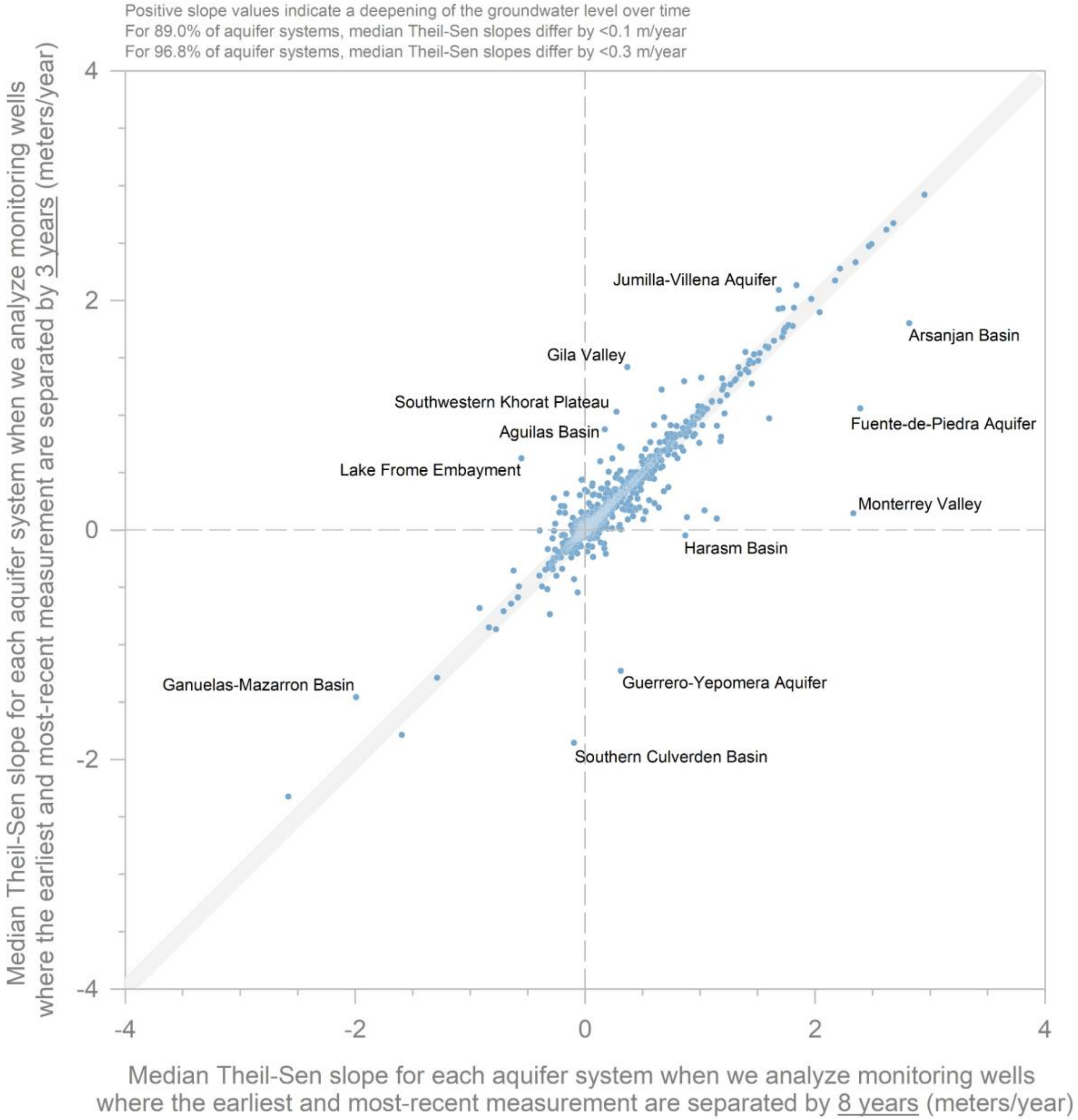

Supplementary Fig. 13. Sensitivity of aquifer-scale trends in depth to groundwater to the selection of a minimum number of years separating the earliest and most-recent annual median groundwater level (3 years versus 8 years presented here). Each circle represents one aquifer system and its 21st century trends in depth to groundwater (median Theil-Sen slope of monitoring wells within the boundaries of the aquifer system). The x-axis values are the aquifer-scale trends in depth to groundwater presented in the main text (e.g., Fig. 2). Points on the y-axis are aquifer-scale trends in depth to groundwater if we reduce the stringency of our criteria to include a monitoring well in our analyses, specifically by reducing the minimum temporal offset between the earliest and most-recent calendar year with at least one measurement from 8 years (criteria for inclusion in the main text) to, instead, just 3 years. Points within the light grey area shaded area have x-axis and the y-axis values that differ by less than 0.1 m/year. Overall, the great majority of aquifer-scale trends in depth to groundwater do not change substantially when we reduce our minimum temporal offset from 8 years to 3 years, as evidenced by the 89% of aquifer-scale trends in depth to groundwater that differ by less than 0.1 m/year (points within the light grey diagonal band) and the 96.8% of aquifer-scale trends in depth to groundwater that differ by less than 0.3 m/year.

7 DELINEATING AQUIFER SYSTEM BOUNDARIES

We consulted maps and descriptions in over 1,000 local- and regional-scale studies to outline the boundaries of 1,693 aquifer systems (total number of unique references in table below is n=1,236; these full citations are provided in alphabetical order via refs.[52-1287]). The specific steps we took to outline the boundary of each aquifer system are presented in the following table.

Supplementary Table 5. Literature sources consulted to delineate study area boundaries

Aquifer system	Broader aquifer system	Reference A	Reference B	Reference C	Steps to delineate
**Abadeh Eghlid Basin**	-	Hosseini, S. M., Parizi, E., Ataie-Ashtiani, B., Simmons, C. T. (2019). Assessment of sustainable groundwater resources management using integrated environmental index: Case studies across Iran. Science of the Total Environment, 676, 792-810.	-	-	Approximated from Fig. 6 by Hosseini et al. (2019)
**Abadeh Tashk Basin**	-	Hosseini, S. M., Parizi, E., Ataie-Ashtiani, B., Simmons, C. T. (2019). Assessment of sustainable groundwater resources management using integrated environmental index: Case studies across Iran. Science of the Total Environment, 676, 792-810.	-	-	Approximated from Fig. 6 by Hosseini et al. (2019)
**Abarkooh Plain**	-	Ghazavi, R., Ebrahimi, Z. (2015). Assessing groundwater vulnerability to contamination in an arid environment using DRASTIC and GOD models. International Journal of Environmental Science and Technology, 12, 2909-2918.	Soldo, B., Mahmoudi Sivand, S., Afrasiabian, A., Đurin, B. (2020). Effect of Sinkholes on Groundwater Resources in Arid and Semi-Arid Karst Area in Abarkooh, Iran. Environments, 7, 26.	-	Approximated from Fig. 1 by Ghazavi and Ebrahimi (2015) and Fig. 1 by Soldo et al. (2020)
**Abbas -e Sharghi Basin**	-	Hosseini, S. M., Parizi, E., Ataie-Ashtiani, B., Simmons, C. T. (2019). Assessment of sustainable groundwater resources management using	-	-	Approximated from Fig. 6 by Hosseini et al. (2019)

Aquifer system	Broader aquifer system	Reference A	Reference B	Reference C	Steps to delineate
		integrated environmental index: Case studies across Iran. Science of the Total Environment, 676, 792-810.			
**Abbotsford-Sumas Aquifer**	Puget Sound Lowland	Scibek, J., Allen, D. M. (2005). Numerical groundwater flow model of the Abbotsford–Sumas aquifer, central Fraser Lowland of BC, Canada, and Washington State, US. Report prepared for Environment Canada, Vancouver, 203 (accessed February 9, 2021 via https://www.sfu.ca/personal/dallen/AB_Modeling_Report_Final.pdf)	Cox, S. E., Kahle, S. C. (1999). Hydrogeology, ground-water quality, and sources of nitrate in lowland glacial aquifers of Whatcom County, Washington, and British Columbia, Canada (Vol. 98, No. 4195). US Department of the Interior, US Geological Survey. https://pubs.er.usgs.gov/publication/wri984195	Vaccaro, J. J., Hansen, A. J., Jones, M. A. (1998). Hydrogeologic framework of the Puget Sound aquifer system, Washington and British Columbia. U.S. Geological Survey Professional Paper 1424-D, 87 pp. Accessed March 11, 2021 from https://pubs.usgs.gov/pp/1424d/report.pdf	Broader Puget Sound Lowland approximated from Vaccaro et al. (1998). Abbotsford-Sumas aquifer approximated from Map 4 of Scibek and Allen (2005) and Plate 2 of Cox and Kahle (1999) (the latter reference led to our extending the southern boundary of the aquifer ~10 km farther south than delineated by Scibek and Allen (2005)
**Abdan-Dayer Coastal Plain**	-	Mohammadi, Z., Zare, M., Sharifzade, B. (2012). Delineation of groundwater salinization in a coastal aquifer, Bousheher, South of Iran. Environmental Earth Sciences, 67, 1473-1484.	-	-	Approximated from Fig. 1 by Mohammadi et al. (2012)
**Abhar Plain**	-	Langeroudi, S. R., Turkamani, S. M. (2016). Water Quality Assessment and Hydrochemical Characteristics of Groundwater in Abhar Plain, Zanjan, Iran. Journal of Tethys, 4, 209-220.	-	-	Approximated from Fig. 1 by Langeroudi and Turkamani (2016)
**Abrego Aquifer**	-	CONAGUA (2020o). Actualización de la disponibilidad media anual de agua en el acuífero Abrego (3215), Estado de Zacatecas. 28 pp. Accessed	-	-	Approximated from Fig. 1 by CONAGUA (2020o)

Aquifer system	Broader aquifer system	Reference A	Reference B	Reference C	Steps to delineate
		March 7, 2022 via https://sigagis.conagua.gob.mx/gas1/Edos_Acuiferos_18/zacatecas/DR_3215.pdf			
**Actopan Aquifer**	Coastal Plain of Veracruz	Quezadas, J. P., Heilweil, V. M., Silva, A. C., Araguas, L., Ortega, M. D. R. S. (2016). A multi-tracer approach to delineate groundwater dynamics in the Rio Actopan Basin, Veracruz State, Mexico. Hydrogeology Journal, 24, 1953-1966.	-	-	Approximated from Fig. 1 by Quezadas et al. (2016).
**Agua Verde Aquifer**	-	Urrutia, J., Jódar, J., Medina, A., Herrera, C., Chong, G., Urqueta, H., Luque, J. A. (2018). Hydrogeology and sustainable future groundwater abstraction from the Agua Verde aquifer in the Atacama Desert, northern Chile. Hydrogeology Journal, 26, 1989-2007.	-	-	Approximated from Fig. 1 by Urrutia et al. (2018).
**Aguadilla-Hatillo Region**	Puerto Rico North Coast Karst Aquifer	Padilla, I., Irizarry, C., Steele, K. (2011). Historical contamination of groundwater resources in the north coast karst aquifers of Puerto Rico. Dimensión Ingeniería y Agrimensura, 25, 7-12.	Veve, T.D., Taggart, B.E. (1996). Atlas of Ground-Water Resources in Puerto Rico and the U.S. Virgin Islands. U.S. Geological Survey Water Resources Investigations Report 94-4198, 151 pp. Accessed May 22, 2021 from https://pubs.usgs.gov/wri/1994/4198/report.pdf	-	Approximated from Fig. 1 by Padilla et al. (2011) and Fig. 2.1.1.B-1 by Veve and Taggart (1996).
**Aguanaval Aquifer**	-	CONAGUA (2021a). Acuíferos (nacional). Webpage accessed March 8, 2022 from	-	-	Approximated from aquifer boundaries defined by CONAGUA (2021a) web portal, physiography

Aquifer system	Broader aquifer system	Reference A	Reference B	Reference C	Steps to delineate
		http://sina.conagua.gob.mx/sina/tema.php?tema=acuiferos&ver=mapa&o=1&n=nacional			and drainage patterns, and well completion data.
**Aguascalientes Valley**	-	Pacheco-Martínez, J., Hernandez-Marín, M., Burbey, T. J., González-Cervantes, N., Ortíz-Lozano, J. Á., Zermeño-De-Leon, M. E., Solís-Pinto, A. (2013). Land subsidence and ground failure associated to groundwater exploitation in the Aguascalientes Valley, México. Engineering Geology, 164, 172-186.	Guerrero-Martínez, L., Hernández-Marín, M., Burbey, T. J. (2018). Estimation of natural groundwater recharge in the Aguascalientes semiarid valley, Mexico. Revista Mexicana de Ciencias Geológicas, 35, 268-278.	-	Approximated from Fig. 1 by Pacheco-Martínez et al. (2013) and Fig. 1 by Guerrero-Martínez et al. (2018).
**Aguilas Basin**	-	Martínez-Granados, D., Calatrava, J. (2014). The role of desalinisation to address aquifer overdraft in SE Spain. Journal of Environmental Management,144,247-257.	-	-	Approximated from Fig. 2 by Martínez-Granados and Calatrava (2014)
**Ahar-Varzeghan Basin**	-	Hosseini, S. M., Parizi, E., Ataie-Ashtiani, B., Simmons, C. T. (2019). Assessment of sustainable groundwater resources management using integrated environmental index: Case studies across Iran. Science of the Total Environment, 676, 792-810.	-	-	Approximated from Fig. 6 by Hosseini et al. (2019)
**Ahmadi Basin**	-	Hosseini, S. M., Parizi, E., Ataie-Ashtiani, B., Simmons, C. T. (2019). Assessment of sustainable groundwater resources management using	-	-	Approximated from Fig. 6 by Hosseini et al. (2019)

Aquifer system	Broader aquifer system	Reference A	Reference B	Reference C	Steps to delineate
		integrated environmental index: Case studies across Iran. Science of the Total Environment, 676, 792-810.			
**Alabama Coastal Lowlands**	Gulf Coast Regional Aquifer System	Mallory, M. J. (1993). Hydrogeology of the Southeastern Coastal Plain aquifer system in parts of eastern Mississippi and western Alabama. U.S. Geological Survey Professional Paper 1410-G, 66 pp. Accessed March 31, 2021 from https://pubs.usgs.gov/pp/1410g/report.pdf	Trapp Jr., H. (1973). Hydrology of sand-and-gravel aquifer in central and southern Escambia County, Florida. U.S. Geological Survey Open-File Report 74-218, 69 pp. Accessed March 31, 2021 from https://pubs.usgs.gov/of/1974/0218/report.pdf	Pratt, T. R., Richards, C. J., Milla, K. A., Wagner, J. R., Johnson, J. L., Curry, R. J. (1996). Hydrogeology of the Northwest Florida Water Management District. Northwest Florida Water Management District, Water Resources Special Report, 96-4. *and,* Barraclough, J.T., Marsh, O.T. (1962). Aquifers and quality of ground water along the Gulf Coast of western Florida. U.S. Geological Survey Report of Investigations No. 62, 41 pp. Accessed March 31, 2021 from https://ufdcimages.uflib.ufl.edu/UF/00/00/12/16/00001/UF00001216.pdf	Eastern margin approximated along the Conecuh and Escambia Rivers. This area was not included in Floridan Aquifer System, as some local hydrogeological reports in its eastern margins (e.g., Cantonment in northwest Florida) suggest the Floridan aquifer itself may be quite deep as "(t)he Pensacola Clay has been reached by only a few wells in the area" (quote from Trapp (1973)), and the Pensacola Clay overlies what has been deemed the "Floridan Aquifer System" (see Fig. 1 of Pratt et al. (1996)). The subarea contains an area that has been delineated as being part of both the Floridan and the Gulf Coastal Plain (see overlapping areas mapped in Fig. 1 of Mallory (1993)). Fig. 8 of Barraclough and Marsh (1962) also shows that the Upper Floridan Aquifer is more than 300 m deep in Baldwin County (Alabama). As the primary units of the Floridan do not immediately appear to be widely accessed, the subarea was included in the Gulf Coast Regional Aquifer rather than the Floridan Aquifer System.
**Albuquerque Basin**	Middle Rio Grande	Bexfield, L. M., Anderholm, S. K. (2000). Predevelopment water-level map of the Santa Fe Group	Bartolino, J.R., Cole, J.C. (2002). Ground-water resources of the Middle Rio Grande Basin. U.S.	Hawley, J. W., Haase, C.S., Lozinsky, R.P. (1995). An Underground View of the Albuquerque Basin. Report	Approximated from map by Bexfield and Anderholm (2000), Fig. 1 by Hawley et al. (1995) and

Aquifer system	Broader aquifer system	Reference A	Reference B	Reference C	Steps to delineate
		aquifer system in the middle Rio Grande basin between Cochiti Lake and San Acacia, New Mexico. U.S. Geological Survey Water-Resources Investigations Report 00–4249, 1 sheet. Accessed February 17, 2021 from https://doi.org/10.3133/wri004249	Geological Survey Water-Resources Circular 1222, 145 pp. Accessed November 28, 2021 from https://pubs.usgs.gov/circ/2002/circ1222/pdf/circ1222.pdf	No. CONF-9411293-TRN: IM9704%%261. 37-55. Accessed November 28, 2021 from https://nmwrri.nmsu.edu/wp-content/uploads/2015/watcon/proc39/Hawley.pdf	Fig. 2.1 by Bartolino and Cole (2002)
**Aledo Aquifer**	-	Calatrava, J., Guillem, A., Martínez-Granados, D. (2011). Analysis of alternatives to eliminate aquifer overdraft in the Guadalentín Valley, SE Spain. Economía Agraria y Recursos Naturales-Agricultural and Resource Economics, 11, 33-62.	-	-	Approximated from Map 2 by Calatrava et al. (2011)
**Aleshtar Plain**	-	Amouzegari, P., Panahi, M., Mirnia, S. K., Daneshi, A. (2020). Estimation of preservation value of groundwater resources from the villagers' perspective in Alashtar Watershed, Iran. Watershed Engineering and Management, 12, 57-71.	Asadi, N., Kaki, M., Jamoor, R. (2016). Groundwater level decline and compensating withdrawal plan in Aleshtar plain, Lorestan province, Iran. Journal of Natural Environmental Hazards, 5, 107-124.	-	Approximated from Fig. 1 by Amouzegari et al. (2020) and Fig. 1 by Asadi et al. (2016)
**Aliabad Plain**	-	Jafari, F., Javadi, S., Golmohammadi, G., Karimi, N., Mohammadi, K. (2016). Numerical simulation of groundwater flow and aquifer-system compaction using simulation and InSAR technique: Saveh basin, Iran. Environmental Earth Sciences, 75, 833.	Edalat, A., Khodaparast, M., Rajabi, A. M. (2020). Scenarios to control land subsidence using numerical modeling of groundwater exploitation: Aliabad plain (in Iran) as a case study. Environmental Earth Sciences, 79, 1-12.	-	Approximated from Fig. 1 by Jafari et al. (2016) and Fig. 1 by Edalat et al. (2020)

Aquifer system	Broader aquifer system	Reference A	Reference B	Reference C	Steps to delineate
**Allah Marvdasht Basin**	-	Hosseini, S. M., Parizi, E., Ataie-Ashtiani, B., Simmons, C. T. (2019). Assessment of sustainable groundwater resources management using integrated environmental index: Case studies across Iran. Science of the Total Environment, 676, 792-810.	-	-	Approximated from Fig. 6 by Hosseini et al. (2019)
**Allier Basin**	-	Mohammed, N., Celle-Jeanton, H., Huneau, F., Le Coustumer, P., Lavastre, V., Bertrand, G., Charrier, G., Clauzet, M.L., (2014). Isotopic and geochemical identification of main groundwater supply sources to an alluvial aquifer, the Allier River valley (France). Journal of Hydrology, 508, 181-196.	-	-	Approximated from Fig. 1 by Mohammed et al. (2014)
**Almanzora Basin**	-	Morell, I. (2003). Acuíferos detríticos costeros. Hidrogeología y Aguas Subterráneas, 1, 31-44.	-	-	Approximated from Fig. 5 by Morell (2003)
**Almonte-Marismas Aquifer**	Guadalquivir Basin	Naranjo-Fernández, N., Guardiola-Albert, C., Aguilera, H., Serrano-Hidalgo, C., Montero-González, E. (2020). Clustering Groundwater Level Time Series of the Exploited Almonte-Marismas Aquifer in Southwest Spain. Water, 12, 1063.	-	-	Approximated from Fig. 1 by Naranjo-Fernández et al. (2020)
**Alsatian Aquifer and**	-	Bernhard, C., Carbiener, R., Cloots, A. R., Froehlicher, R., Schenck, C., Zilliox, L.	-	-	Approximated from Fig. 1 by Bernhard et al. (1992)

Aquifer system	Broader aquifer system	Reference A	Reference B	Reference C	Steps to delineate
**Upper Rhine Valley**		(1992). Nitrate pollution of groundwater in the alsatian plain (France)—A multidisciplinary study of an agricultural area: The Central Ried of the Ill river. Environmental Geology and Water Sciences, 20, 125-137.			
**Alturas Warm Springs Valley**	-	California Department of Water Resources (2015i). California's groundwater update 2013 - Chapter 7: Sacramento River Hydrologic Region. California Department of Water Resources Report, 126 pp. Accessed April 27, 2022 via https://water.ca.gov/-/media/DWR-Website/Web-Pages/Programs/Groundwater-Management/Bulletin-118/Files/Statewide-Reports/GWU2013_Ch7_SacramentoRiver_Final.pdf	-	-	Approximated from Fig. 7-2 by California Department of Water Resources (2015i)
**Amargosa Desert**	-	Burbey, T. J. (1997). Hydrogeology and potential for ground-water development, carbonate-rock aquifers in southern Nevada and southeastern California. US Geological Survey Water-Resources Investigations Report 95-4168, 70 pp. Accessed March 10, 2021 from https://pubs.usgs.gov/wri/1995/4168/report.pdf	-	-	Approximated from Fig. 14 of Burbey (1997)

Aquifer system	Broader aquifer system	Reference A	Reference B	Reference C	Steps to delineate
**Amazonas Basin and Alter de Chao Aquifer System**	Amazon Aquifer System	Hirata, R., Suhogusoff, A. V. (2019). How much do we know about the groundwater quality and its impact on Brazilian society today? Acta Limnologica Brasiliensia, 31, e109.	Pimentel, E. T., Hamza, V. M. (2014). Use of geothermal methods in outlining deep groundwater flow systems in Paleozoic interior basins of Brazil. Hydrogeology Journal, 22, 107-128.	de Souza, E. L., Galvão, P. H. F., da Silva Pinheiro, C. D. S., Baessa, M. P. M., Demétrio, J. G. A., Brito, W. R. R. (2013). Síntese da hidrogeologia nas bacias sedimentares do Amazonas e do Solimões: Sistemas Aquíferos Içá-Solimões e Alter do Chão. Geologia USP. Série Científica, 13, 107-117.	Alter de Chao Aquifer System approximated from Fig. 2 by Hirata and Suhogusoff (2019), Fig. 1 by Pimentel and Hamza (2014), and Fig. 1 by de Souza et al. (2013).
**Amol-Ghaemshahr Plain**	-	Janardhana, M. R., Khairy, H. (2019). Simulation of seawater intrusion in coastal aquifers: a case study on the Amol–Ghaemshahr coastal aquifer system, Northern Iran. Environmental Earth Sciences, 78, 1-19.	-	-	Approximated from Fig. 1 by Janardhana and Khairy (2019)
**Anarak Basin**	-	Hosseini, S. M., Parizi, E., Ataie-Ashtiani, B., Simmons, C. T. (2019). Assessment of sustainable groundwater resources management using integrated environmental index: Case studies across Iran. Science of the Total Environment, 676, 792-810.	-	-	Approximated from Fig. 6 by Hosseini et al. (2019)
**Anderson Valley**	-	California Department of Water Resources (2015j). California's groundwater update 2013 - Chapter 3: North Coast Hydrologic Region. California Department of Water Resources Report, 80 pp. Accessed April 27, 2022 via	-	-	Approximated from Fig. 3-2 by California Department of Water Resources (2015j)

Aquifer system	Broader aquifer system	Reference A	Reference B	Reference C	Steps to delineate
		https://water.ca.gov/-/media/DWR-Website/Web-Pages/Programs/Groundwater-Management/Bulletin-118/Files/Statewide-Reports/GWU2013_Ch3_NorthCoast_Final.pdf			
**Angas-Bremer Plains and Murray Estuary Aquifer System**	Murray Basin	Herczeg, A. L., Dogramaci, S. S., Leaney, F. W. J. (2001). Origin of dissolved salts in a large, semi-arid groundwater system: Murray Basin, Australia. Marine and Freshwater Research, 52, 41-52.	Cresswell, R., Gibson, D. (2004). Application of Airborne Geophysical Techniques to Groundwater Resource Issues in the Angas-Bremer Plains, South Australia. South Australia Salinity Mapping and Management Support Project Report No. DWLBC 2004/35, Land and Biodiversity Services Division Department of Water, Land and Biodiversity Conservation, 50 pp. Accessed July 21, 2021 from http://angasbremerwater.org.au/documents/abplains_summary.pdf	-	Approximated from Fig. 1 by Herczeg et al. (2001), and Fig. 1 by Cresswell and Gibson (2004)
**Animas Valley**	-	Schwennesen, A.T., Hare, R.F. (1918). Ground water in the Animas, Playas, Hachita, and San Luis Basins, New Mexico, with analyses of water and soil. U.S. Geological Survey Water Supply Paper 422, 157 pp. Accessed March 29, 2021 from https://pubs.usgs.gov/wsp/0422/report.pdf	-	-	Approximated from Plate II of Schwennesen and Hare (1918).

Aquifer system	Broader aquifer system	Reference A	Reference B	Reference C	Steps to delineate
**Antelope Basin**	Sacramento Valley of the Central Valley **	California Department of Water Resources (2015i). California's groundwater update 2013 - Chapter 7: Sacramento River Hydrologic Region. California Department of Water Resources Report, 126 pp. Accessed April 27, 2022 via https://water.ca.gov/-/media/DWR-Website/Web-Pages/Programs/Groundwater-Management/Bulletin-118/Files/Statewide-Reports/GWU2013_Ch7_SacramentoRiver_Final.pdf	-	-	Approximated from Fig. 7-2 by California Department of Water Resources (2015i)
**Antelope Plain and Lost Hills**	Tulare Basin of the Central Valley **	California Department of Water Resources (2015c). California's groundwater update 2013 - Chapter 9: Tulare Lake Hydrologic Region. California Department of Water Resources Report, 80 pp. Accessed April 27, 2022 via https://data.cnra.ca.gov/dataset/california-water-plan-groundwater-update-2013/resource/8a4ae915-b786-42e1-9abe-99a8fcc23349	Wood, P.R., Davis, G.H. (1959). Ground-Water conditions in the Avenal-McKittrick Area Kings and Kern Counties California. US Geological Survey Water-Supply Paper 1457, 144 pp. Accessed April 28, 2022 via https://pubs.usgs.gov/wsp/1457/report.pdf	-	Approximated from Fig. 9-2 by California Department of Water Resources (2015c) and Fig. 2 by Wood and Davis (1959)
**Antelope Valley**	-	Stamos, C. L., Christensen, A. H., Langenheim, V. (2017). Preliminary hydrogeologic assessment near the boundary of the Antelope Valley and El Mirage Valley groundwater basins, California. U.S. Geological Survey Scientific	Duell Jr., L.F.W. (1987). Geohydrology of the Antelope Valley area, California, and design for a ground-water-quality monitoring network. US Geological Survey Water-Resources Investigations Report 84-4081, 76 pp.	-	Approximated from Fig. 7 of Stamos et al. (2017) and Plate 1 by Duell Jr. (1987)

Aquifer system	Broader aquifer system	Reference A	Reference B	Reference C	Steps to delineate
		Investigations Report 2017–5065, 56 pp. Accessed March 20, 2021 from https://pubs.usgs.gov/sir/2017/5065/sir20175065.pdf	Accessed April 18, 2022 via https://pubs.usgs.gov/wri/1984/4081/report.pdf		
**Antelope Valley near Topaz Lake**	-	Lopes, T.J., Evetts. D.M. (2005). Ground-Water pumpage and artificial recharge estimates for calendar year 2000 and average annual natural recharge and interbasin flow by hydrographic area, Nevada. Accessed April 25, 2022 via https://pubs.usgs.gov/sir/2004/5239/sir2004-5239.pdf	Harrill, J.R., Prudic, D.E. (1998). Aquifer systems in the Great Basin region of Nevada, Utah, and adjacent states; summary report. US Geological Survey Professional Paper 1409-A, 75 pp. Accessed April 26, 2022 via https://pubs.usgs.gov/pp/1409a/report.pdf	-	Approximated from Fig. 1 by Lopes and Evetts (2005), and Fig. 2 by Harrill and Prudic (1998). Well completion records, physiography and drainage were used to delinate areas.
**Antigonish Basin**	Maritimes Basin	Stapinsky, M. Michaud, Y., Morin, R.H., Butler, K.E., Deblonde, C., Chi, G, Thériault, T., Castonguay, S., Boisvert, V., Julien, H.P., Conohan, D., Hulsman, B., Carrier, C. Marion, J., Boisvert, É. (2002). Groundwater resources assessment in the Carboniferous Maritimes Basin: preliminary results of the hydrogeological characterization, New Brunswick, Nova Scotia, and Prince Edward Island. Geological Survey of Canada Current Research Report 2002-D8, 14 pp. Accessed May 5, 2021 from http://www.gov.pe.ca/photos/original/cle_WA10.pdf	Rivard, C., Michaud, Y., Lefebvre, R., Deblonde, C., Rivera, A. (2008). Characterization of a regional aquifer system in the Maritimes Basin, Eastern Canada. Water Resources Management, 22, 1649-1675.	Bianco, E. (2013). Seismic Interpretation of the Windsor-Kennetcook Basin. Geological Survey of Canada Open File 7452, 31 pp. Accessed May 5, 2021 from https://ftp.maps.canada.ca/pub/nrcan_rncan/publications/STPublications_PublicationsST/292/292763/of_7452.pdf	Approximated from Fig. 2 of Rivard et al. (2008) and Figs. 1 and 3 of Stapinsky et al. (2002). Area bounds approximated from Fig. 1 of Bianco (2013).

**Aquifer system**	**Broader aquifer system**	**Reference A**	**Reference B**	**Reference C**	**Steps to delineate**
**Antlers Aquifer**	-	Hart Jr., Davis, R.E. (1981). Geohydrology of the Antlers aquifer (Cretaceous), southeastern Oklahoma. U.S. Geological Survey Circular 81, 38 pp. Accessed April 5, 2021 from http://www.ogs.ou.edu/pubsscanned/Circulars/circular81mm.pdf	-	-	Approximated from Fig. 1 of Hart and Davis (1981)
**Apalachicola Delta**	Floridan Aquifer System	Davis, H. (1996). Hydrogeologic Investigation and Simulation of Ground-Water Flow in the Upper Floridan Aquifer of North-Central Florida and Southwestern Georgia and Delineation of Contributing Areas for Selected City of Tallahassee, Florida, Water-Supply Wells. U.S. Geological Survey Water-Resources investigations Report 95-4296, 61 pp. Accessed March 31, 2021 from https://fl.water.usgs.gov/PDF_files/wri95_4296_davis.pdf	Clark, W. Z., Zisa, A. C. (1976) Physiographic map of Georgia: Georgia Department of Natural Resources, 1 sheet. Accessed March 31, 2021 from https://epd.georgia.gov/document/publication/sm-4-physiographic-map-georgia-12000000-1988/download	Brooks, H. K. (1981). Physiographic divisions of Florida. Report for the Florida Cooperative Extension Service Institute of Food and Agricultural Sciences, University of Florida, 12 pp.	Approximated from Fig. 2 of Davis (1996) and the physiographic provinces for Georgia and Florida by Clark and Zisa (1976) and Brooks (1981)
**Aqda Basin**	-	Hosseini, S. M., Parizi, E., Ataie-Ashtiani, B., Simmons, C. T. (2019). Assessment of sustainable groundwater resources management using integrated environmental index: Case studies across Iran. Science of the Total Environment, 676, 792-810.	-	-	Approximated from Fig. 6 by Hosseini et al. (2019)

Aquifer system	Broader aquifer system	Reference A	Reference B	Reference C	Steps to delineate
**Arak Plain**	-	Jabbari, E., Fathi, M., Moradi, M. (2020). Modeling groundwater quality and quantity to manage water resources in the Arak aquifer, Iran. Arabian Journal of Geosciences, 13, 1-16.	Ghadimi, F., Ghomi, M. (2012). Statistical Analysis of the Hydrogeochemical Evolution of Groundwater in Alluvial Aquifer of Arak Mighan Playa, Markazi Province, Iran. Journal of Water Sciences Research, 4, 31-45.	Rahbar, A., Vadiati, M., Talkhabi, M., Nadiri, A. A., Nakhaei, M., Rahimian, M. (2020). A hydrogeochemical analysis of groundwater using hierarchical clustering analysis and fuzzy C-mean clustering methods in Arak plain, Iran. Environmental Earth Sciences, 79, 1-17.	Approximated from Fig. 1 by Jabbari et al. (2020), Fig. 1 by Ghadimi and Ghomi (2012) and Fig, 2 by Rahbar et al. (2020)
**Arbon Valley**	Eastern Snake River Plain	Graham, W.G., Campbell, L.J. (1981). Groundwater resources of Idaho. Idaho Department of Water Resources Report, 61 pp. Accessed March 23, 2021 from https://idwr.idaho.gov/files /publications/198108-MISC-GW-Resources-ID.pdf	-	-	Approximated from Plate 1 of Graham and Campbell (1981).
**Arbuckle-Simpson Aquifer**	-	Christenson, S., Osborn, N.I., Neel, C.R., Faith, J.R., Blome, C.D., Puckette, James, Pantea, M.P. (2011). Hydrogeology and simulation of groundwater flow in the Arbuckle-Simpson aquifer, south-central Oklahoma: U.S. Geological Survey Scientific Investigations Report 2011–5029, 104 pp. Accessed April 5, 2021 from https://pubs.usgs.gov/sir/2011/5029/SIR2011-5029.pdf	-	-	Approximated from Fig. 1 of Christenson et al. (2011)
**Arckaringa Basin and Southwestern Great**	Great Artesian Basin	Ransley, T.R., Radke, B.M., Feitz, A.J., Kellett, J.R., Owens, R., Bell, J. and Stewart, G. (2015).	Ransley, T.R., Smerdon, B.D. (2012a). Hydrostratigraphy, hydrogeology and system	Priestley, S. C., Payne, T. E., Harrison, J. J., Post, V. E., Shand, P., Love, A. J., Wohling, D. L. (2018). Use	Approximated from Map 1 on page 9 of report by Ransley et al. (2015), Fig. 2.3 by Ransley and

Aquifer system	Broader aquifer system	Reference A	Reference B	Reference C	Steps to delineate
**Artesian Basin**		Hydrogeological Atlas of the Great Artesian Basin, Geoscience Australia. Canberra, 141 pp. Accessed July 19, 2021 from http://www.ga.gov.au/scientific-topics/water/groundwater/gab	conceptualisation of the Great Artesian Basin. A technical report to the Australian Government from the CSIRO Great Artesian Basin Water Resource Assessment, 324 pp. Accessed July 20, 2021 from https://publications.csiro.au/rpr/download?pid=csiro:EP132693&dsid=DS5	of U-isotopes in exploring groundwater flow and inter-aquifer leakage in the south-western margin of the Great Artesian Basin and Arckaringa Basin, central Australia. Applied Geochemistry, 98, 331-344.	Smerdon (2012), and Fig. 1 by Priestly et al. (2018).
**Ardestand Basin**	-	Pazand, K., Khosravi, D., Ghaderi, M. R., Rezvanianzadeh, M. R. (2018). Identification of the hydrogeochemical processes and assessment of groundwater in a semi-arid region using major ion chemistry: A case study of Ardestan basin in Central Iran. Groundwater for Sustainable Development, 6, 245-254.	-	-	Approximated from Fig. 1 by Pazand et al. (2018)
**Arecibo-Manati and Vega Baja-Toa Baja Regions**	Puerto Rico North Coast Karst Aquifer	Padilla, I., Irizarry, C., Steele, K. (2011). Historical contamination of groundwater resources in the north coast karst aquifers of Puerto Rico. Dimensión Ingeniería y Agrimensura, 25, 7-12	Veve, T.D., Taggart, B.E. (1996). Atlas of Ground-Water Resources in Puerto Rico and the U.S. Virgin Islands. U.S. Geological Survey Water Resources Investigations Report 94-4198, 151 pp. Accessed May 22, 2021 from https://pubs.usgs.gov/wri/1994/4198/report.pdf	-	Approximated from Fig. 1 by Padilla et al. (2011) and Figs. 2.1.2.E-1 and 2.1.3.D-3 by Veve and Taggart (1996).
**Arriga Flats**	-	Koci, J. (2011). Deep drainage potential of surface irrigated sugarcane in the Arriga Flats of far north Queensland. Report	-	-	Approximated from Fig. 1 by Koci (2011)

Aquifer system	Broader aquifer system	Reference A	Reference B	Reference C	Steps to delineate
		on Improving application efficiency of furrow irrigated sugar cane using SIRMOD and implications for rising saline groundwater in the Arriga Basin of Far North Queensland funded by National Program for Sustainable Irrigation, 20 pp. Accessed May 8, 2023 via http://27.111.91.222/xmlui/bitstream/handle/1/4125/JCU1101%20Final%20Report.pdf?sequence=1&isAllowed=y			
**Arrowsmith and Dandaragan Plateau**	Perth Basin	Priestley, S.C., Meredith, K.T., Treble, P.C., Cendón, D.I., Griffiths, A.D., Hollins, S.E., Baker, A., Pigois, J.P. (2020). A 35 ka record of groundwater recharge in south-west Australia usingstablewater isotopes. Science of the Total Environment, 717, 135105.	Raper, G.P., Speed, R., Simons, J.A., Killen, A.L., Blake, A., Ryder, A.T., Smith, R.H., Stainer, G., Bourke, L. (2014). Groundwater trend analysis and salinity risk assessment for the south-west agricultural region of Western Australia, 2007–12. Government of Western Australia Department of Agriculture and Food Resource Management Technical Report 388, 148 pp. Accessed July 26, 2021 from https://researchlibrary.agric.wa.gov.au/cgi/viewcontent.cgi?article=1372&context=rmtr	Government of Western Australia's Department of Water (2017). Northern Perth Basin: Geology, hydrogeology and groundwater resources. Department of Water Hydrological Bulletin Series Report No. HB1, 395 pp. Accessed July 25, 2021 from https://www.water.wa.gov.au/__data/assets/pdf_file/0011/8993/111005.pdf	Approximated from Fig. 1 by Priestley et al. (2021), Fig. 1 by Raper et al. (2014) and Fig. 5 by Government of Western Australia's Department of Water (2017)
**Arsanjan Basin**	-	Hosseini, S. M., Parizi, E., Ataie-Ashtiani, B., Simmons, C. T. (2019).	-	-	Approximated from Fig. 6 by Hosseini et al. (2019)

Aquifer system	Broader aquifer system	Reference A	Reference B	Reference C	Steps to delineate
		Assessment of sustainable groundwater resources management using integrated environmental index: Case studies across Iran. Science of the Total Environment, 676, 792-810.			
**Asadabad Plain**	-	Oroji, B., Karimi, Z. F. (2018). Application of DRASTIC model and GIS for evaluation of aquifer vulnerability: case study of Asadabad, Hamadan (western Iran). Geosciences Journal, 22, 843-855.	-	-	Approximated from Fig. 1 by Oroji and Karimi (2018)
**Asahikawa Basin**	-	Cutshall, I. (1949). Urban Settlement in Hokkaido. Economic Geography, 25, 17-22.	-	-	Approximated from descriptions within the text of Cutshall (1949)
**Ascoy-Soplamo Aquifer**	-	Custodio, E., Andreu-Rodes, J.M., Aragón, R., Estrela, T., Ferrer, J., García-Aróstegui, J.L., Manzano, M., Rodríguez-Hernández, L., Sahuquillo, A., Del Villar, A. (2016). Groundwater intensive use and mining in south-eastern peninsular Spain: Hydrogeological, economic and social aspects. Science of the Total Environment, 559, 302-316.	-	-	Approximated from Fig. 1 by Custodio et al. (2016)
**Ashland Flats**	-	Zarour, H., Aitchison-Earl, P., Scott, M., Peaver, L., De Silva, J. (2018). Current state of the groundwater resource in the Orari-Temuka-Opihi-Pareora area. Environment	-	-	Approximated from Fig. 2-3 by Zarour et al.(2018).

Aquifer system	Broader aquifer system	Reference A	Reference B	Reference C	Steps to delineate
		Canterbury Regional Council Report No. R16/41, 56 pp. Accessed October 20, 2021 from https://api.ecan.govt.nz/TrimPublicAPI/documents/download/2964277			
**Ashley Valley**	Uinta Basin	Hood, J. W. (1976). Characteristics of aquifers in the northern Uinta Basin area, Utah and Colorado. U.S. Geological Survey and Utah Department of Natural Resources, Division of Water Rights Technical Publication 53, 71 pp. Accessed March 7, 2021 from https://waterrights.utah.gov/docSys/v920/w920/w920009f.pdf	Zhang, Y., Gable, C. W., Zyvoloski, G. A., Walter, L. M. (2009). Hydrogeochemistry and gas compositions of the Uinta Basin: A regional-scale overview. AAPG Bulletin, 93(8), 1087-1118.	Hood, J. W. (1977). Hydrologic evaluation of Ashley Valley, northern Uinta Basin area, Utah. State of Utah Department of Natural Resources, Division of Water Rights Technical Publication 54, 71 pp. Accessed March 7, 2021 from https://pubs.usgs.gov/unnumbered/70043723/report.pdf	Northern Uinta Basin outline from Plates 1-3 of Hood (1976) and Fig. 1 by Zhang et al. (2009). Ashley Valley outline approximated from Fig. 1 of Hood (1977) and extended to northern margin of Uinta Basin
**Aspas Plain**	-	Choubin, B., Malekian, A. (2013). Relationship between Fluctuations in the Water Table and Aquifer Salinization (Case Study: Aquifer Aspas-Fars Province). Desert Management, 1, 13-26.	Naderi, M. (2020). Assessment of water security under climate change for the large watershed of Dorudzan Dam in southern Iran. Hydrogeology Journal, 28, 1553-1574.	-	Approximated from Fig. 1 by Choubin and Malekian (2013) and Fig. 13 by Naderi (2020).
**Assiniboine Delta**	-	Render, F.W. (1988). Water supply capacity of the Assiniboine Delta Aquifer. Canadian Water Resources Journal, 13, 16-34.	Render, F.W. (1987). Aquifer capacity investigations 1980-1986. Manitoba Water Resources Hydrotechnical Services Report, 162 pp. Accessed May 5, 2021 from https://www.gov.mb.ca/water/pubs/water-science-management/groundwater/publication/1987_render_a	Betcher, R.N. (1988). Groundwater Availability Map Series - Neepawa Area (62-J). Manitoba Department of Natural Resoureces map. Accessed May 5, 2021 from https://www.gov.mb.ca/water/pubs/maps/water/1988_betcher_groundwater_availability_map_series_neepawa.zip	Approximated from Fig. 1 of Render (1988) and map on page 74 of Render (1987) and Fig. 6 by Betcher (1988).

Aquifer system	Broader aquifer system	Reference A	Reference B	Reference C	Steps to delineate
			quifer_capacity_investigations_1980_1986.pdf		
**Astanea-Kochesfahan Plain**	-	Langrudi, M. A. O., Siuki, A. K., Javadi, S., Hashemi, S. R. (2016). Evaluation of vulnerability of aquifers by improved fuzzy drastic method: Case study: Aastane Kochesfahan plain in Iran. Ain Shams Engineering Journal, 7, 11-20.	Yoosefdoo, I., Khashei Siuki, A. (2018). Determine the Vulnerability of the Aquifer Using the Standard Drastic and Data-Based Methods (Case Study: Kochisfahan Aquifer). Iranian Journal of Remote Sensing & GIS, 9, 99-116.	-	Approximated from Fig. 1 by Langrudi et al. (2016) and Fig. 1 by Yoosefdoo et al. (2018)
**Astian Aquifer**	-	Dever, L., Travi, Y., Barbecot, F., Marlin, C., Gibert, E. (2001). Evidence for palaeowaters in the coastal aquifers of France. Geological Society, London, Special Publications, 189, 93-106.	-	-	Approximated from Figs. 7 and 8 by Dever et al. (2001) and drainage and topography
**Ataiyah Basin**	-	Parizi, E., Hosseini, S. M., Ataie-Ashtiani, B., Simmons, C. T. (2020). Normalized difference vegetation index as the dominant predicting factor of groundwater recharge in phreatic aquifers: case studies across Iran. Scientific Reports, 10, 17473.	-	-	Approximated from Fig. 2 by Parizi et al. (2020)
**Atlantis Aquifer**	-	Bugan, R.D., Jovanovic, N., Israel, S., Tredoux, G., Genthe, B., Steyn, M., Allpass, D., Bishop, R., Marinus, V. (2016). Four decades of water recycling in Atlantis (Western Cape, South Africa): Past, present and future. Water SA, 42, 577-594.	LaVanchy, G. T., Adamson, J. K., Kerwin, M. W. (2021). Integrating groundwater for water security in Cape Town, South Africa. In: Global Groundwater, Source, Scarcity, Sustainability, Security, and Solutions. pp. 439-449. Elsevier.	-	Approximated from Fig. 4 by Bugan et al. (2016) and Fig. 32.4 by LaVanchy et al. (2021)

Aquifer system	Broader aquifer system	Reference A	Reference B	Reference C	Steps to delineate
**Aveiro Quaternary and Cretaceous Aquifer System**	-	De Melo, M. C., Paquete, P. C., Da Silva, M. M. (2001). Evolution of the Aveiro Cretaceous aquifer (NW Portugal) during the Late Pleistocene and present day: evidence from chemical and isotopic data. Geological Society, London, Special Publications, 189, 139-154.	Rosario de Jesus, M. (2001). Groundwater Protection for Public Water-Supply in Portugal. Proceedings of a Workshop on the protection of groundwaters used as a source of drinking water supply, Budapest, November 2001. 11 pp. Accessed October 20, 2021 from https://unece.org/fileadmin/DAM/env/water/meetings/groundwater01/portugal.pdf	-	Approximated from Fig. 2 by Rosário de Jesus (2001), Fig. 2 by De Melo et al. (2001)
**Avra Valley**	Tucson Basin and Avra Valley	Flora, S., Davis, T. (2009). Hydrologic Map Series (HMS), Water Level Change Map Series (WLCMS), and Basin Sweep Assessment Report ADWR Basins and Sub-Basins. Arizona Department of Water Resources Hydrology Division Field Services Section https://new.azwater.gov/sites/default/files/HMSWLCMSBasinSweepAssessmentReport2009.pdf	Carruth, R.L., Kahler, L.M., Conway, B.D. (2018). Groundwater-storage change and land-surface elevation change in Tucson Basin and Avra Valley, south-central Arizona—2003–2016: U.S. Geological Survey Scientific Investigations Report 2018–5154, 34 pp. Accessed April 1, 2021 from https://pubs.usgs.gov/sir/2018/5154/sir20185154.pdf	Hanson, R.T. (1989). Aquifer-system compaction, Tucson Basin and Avra Valley, Arizona. Water-Resources Investigations Report 88-4172, 75 pp. Accessed April 15, 2021 from https://pubs.usgs.gov/wri/1988/4172/report.pdf	Approximated from Fig. 1 of Flora and Davis (2009). See also Fig. 1 of Carruth et al. (2018). Broader Tucson Basin and Avra Valley outline from Fig. 1 of Hanson (1989).
**Ayamonte-Huelva Coastal Aquifer**	-	Grande, J. A., Gonzalez, A., Beltran, R., Sánchez-Rodas, D. (1996). Application of factor Analysis to the study of contamination in the aquifer system of Ayamonte-Huelva (Spain). Groundwater, 34, 155-161. Accessed May 1, 2023 via https://ngwa.onlinelibrary.	-	-	Approximated from Fig. 1 by Grande et al. (1996)

Aquifer system	Broader aquifer system	Reference A	Reference B	Reference C	Steps to delineate
		wiley.com/doi/epdf/10.1111/j.1745-6584.1996.tb01875.x			
**Azapa Aquifer**	-	Doyle, W.W. (1964). Ground water in the Arica Area, Chile. Article number 170 in: Short Papers in Geology and Hydrology Articles 122-172. U.S. Geological Survey Research Professional Paper 475-D, pp. D213-D215.	Direccion General de Aguas (2009). Diagnóstico y Clasificación de Sectores Acuíferos, Volumen No. 2. Gobierno de Chile Ministerio de Obras Publicas Report, 384 pp. Accessed May 16, 2021 from https://snia.mop.gob.cl/sad/CQA5168v2.pdf	Direccion General de Aguas (2015). Plan Nacional de Estudios Acuíferos. Report number 381, Santiago, Chile, 44 pp. Accessed May 16, 2021 from http://bibliotecadigital.ciren.cl/bitstream/handle/123456789/32415/DGA_2015_actualizacion_plan_nacional_acuiferos.pdf?sequence=1&isAllowed=y	Approximated from Fig. 170.1 by Doyle (1964), map presented in Table 1.1 on page 19 by Direccion General de Aguas (2009), and Fig. 2a by Direccion General de Aguas (2015)
**Azghand Basin**	-	Parizi, E., Hosseini, S. M., Ataie-Ashtiani, B., Simmons, C. T. (2020). Normalized difference vegetation index as the dominant predicting factor of groundwater recharge in phreatic aquifers: case studies across Iran. Scientific Reports, 10, 17473.	-	-	Approximated from Fig. 2 by Parizi et al. (2020)
**Azna-Aligudarz Basin**	-	Gholami, F., Malekian, A. (2018). Assessment of Spatio-temporal Oscillations and Physico-chemical Properties of Azna-Aligudarz Basin. Desert Ecosystem Engineering Journal, 7, 57-70.	-	-	Approximated from Fig. 1 by Gholami and Malekian (2018)
**Bacon Terrace**	Floridan Aquifer System	Williams, L.J., Kuniansky, E.L. (2016). Revised hydrogeologic framework of the Floridan aquifer system in Florida and parts of Georgia, Alabama, and South Carolina. U.S.	Clark, W. Z., Zisa, A. C. (1976) Physiographic map of Georgia: Georgia Department of Natural Resources, 1 sheet. Accessed March 31, 2021 from	-	Approximated from physiographic provinces of Georgia outlined by Clark and Zisa (1976). Broader Florida Aquifer System from Williams and Kuniansky (2016).

Aquifer system	Broader aquifer system	Reference A	Reference B	Reference C	Steps to delineate
		Geological Survey Professional Paper 1807, 140 pp. Accessed March 31, 2021 from https://pubs.usgs.gov/pp/1807/pdf/pp1807.pdf	https://epd.georgia.gov/document/publication/sm-4-physiographic-map-georgia-12000000-1988/download		
**Bad-Khaledabad Basin**	-	Nazari, S., Ahmadi, A., Rad, S. K., Ebrahimi, B. (2020). Application of non-cooperative dynamic game theory for groundwater conflict resolution. Journal of Environmental Management, 270, 110889.	Nazari, S., Ahmadi, A. (2019). Non-cooperative stability assessments of groundwater resources management based on the tradeoff between the economy and the environment. Journal of Hydrology, 578, 124075.	-	Approximated from Fig. 2 by Nazari et al. (2020) and Fig. 2 by Nazari and Ahmadi (2019)
**Baghan Basin**	-	Parizi, E., Hosseini, S. M., Ataie-Ashtiani, B., Simmons, C. T. (2020). Normalized difference vegetation index as the dominant predicting factor of groundwater recharge in phreatic aquifers: case studies across Iran. Scientific Reports, 10, 17473.	-	-	Approximated from tabulated coordinates in the Supplementary Information within Parizi et al. (2020) and physiographic and drainage patterns
**Baghmalek Plain**	-	Kalantari, N., Rangzan, K., Thigale, S. S., Rahimi, M. H. (2010). Site selection and cost-benefit analysis for artificial recharge in the Baghmalek plain, Khuzestan Province, southwest Iran. Hydrogeology journal, 18, 761-773.	-	-	Approximated from Fig. 1 by Kalantari et al. (2010)
**Bahabad Plain**	-	Jafari, H., Shirafkan, M., Bagheri, R., Karami, G. H. (2018). Assessing sustainability of the Bahabad aquifer, Central	Kheradpisheh, Z., Talebi, A., Rafati, L., Ghaneian, M. T., Ehrampoush, M. H. (2015). Groundwater quality assessment using	-	Approximated from Figs. 1 and 2 by Jafari et al. (2018), Fig. 2 by Kheradpisheh et al. (2015)

Aquifer system	Broader aquifer system	Reference A	Reference B	Reference C	Steps to delineate
		Iran. Applied Ecology and Environmental Research, 16, 2585-2602.	artificial neural network: A case study of Bahabad plain, Yazd, Iran. Desert, 20, 65-71.		
**Bahadoran Basin**	-	Talebi, M. S., Fatemi, M. (2020). Assessment of the quality and quantity of groundwater in Bahadoran plain using neural network methods, geostatistical and multivariate statistical analysis. Journal of Applied Research in Water and Wastewater, 7, 144-151.	-	-	Approximated from Fig. 1 by Talebi and Fatemi (2020)
**Bajestan Basin**	-	Qasemi, M., Afsharnia, M., Farhang, M., Bakhshizadeh, A., Allahdadi, M., Zarei, A. (2018a). Health risk assessment of nitrate exposure in groundwater of rural areas of Gonabad and Bajestan, Iran. Environmental Earth Sciences, 77, 1-9.	-	-	Approximated from Fig. 1 by Qasemi et al. (2018a)
**Bajo Guadalhorce Valley Alluvial Aquifer**	-	Morell, I. (2003). Acuíferos detríticos costeros. Hidrogeología y Aguas Subterráneas, 1, 31-44.	Urresti-Estala, B., Gavilán, P. J., Pérez, I. V., Cantos, F. C. (2016). Assessment of hydrochemical trends in the highly anthropised Guadalhorce River basin (southern Spain) in terms of compliance with the European groundwater directive for 2015. Environmental Science and Pollution Research, 23, 15990-16005.	-	Approximated from Fig. 5 by Morell (2003) and Fig. 1 by Urresti-Estala et al. (2016)
**Bajo Rio Bravo**	-	CONAGUA (2020cw). Actualización de la disponibilidad media anual de agua en el acuífero Bajo	-	-	Approximated from physiography, well completion data, and Fig. 1 by CONAGUA (2020cw)

Aquifer system	Broader aquifer system	Reference A	Reference B	Reference C	Steps to delineate
		Rio Bravo (2801), estado de Tamaulipas. 43 pp. Accessed March 8, 2022 via https://sigagis.conagua.gob.mx/gas1/Edos_Acuiferos_18/tamaulipas/DR_2801.pdf			
**Baladeh Basin**	-	Parizi, E., Hosseini, S. M., Ataie-Ashtiani, B., Simmons, C. T. (2020). Normalized difference vegetation index as the dominant predicting factor of groundwater recharge in phreatic aquifers: case studies across Iran. Scientific Reports, 10, 17473.	-	-	Approximated from tabulated coordinates in the Supplementary Information within Parizi et al. (2020) and physiographic and drainage patterns
**Balcones Fault Zone**	Edwards-Trinity Aquifer System	Bruun, B., Jackson, K., Lake, P., Walker, J. (2016). Texas Aquifers Study. Texas Water Development Board Report. 336 pp. Accessed April 1, 2021 from https://www.twdb.texas.gov/groundwater/docs/studies/TexasAquifersStudy_2016.pdf#page=89	Barker, R. A., Ardis, A. F. (1996). Hydrogeological framework of the Edwards-Trinity aquifer system, west-central Texas. U.S. Geological Survey Professional Paper 1421-B, 76 pp. Accessed April 1, 2021 from https://pubs.usgs.gov/pp/1421b/report.pdf	-	Approximated from Fig. 6-6 by Bruun et al. (2016) and Fig. 3 by Barker and Ardis (1996).
**Bam and Rahmatabad Basins**	-	Hosseini, S. M., Parizi, E., Ataie-Ashtiani, B., Simmons, C. T. (2019). Assessment of sustainable groundwater resources management using integrated environmental index: Case studies across Iran. Science of the Total Environment, 676, 792-810.	Parizi, E., Hosseini, S. M., Ataie-Ashtiani, B., Simmons, C. T. (2020). Normalized difference vegetation index as the dominant predicting factor of groundwater recharge in phreatic aquifers: case studies across Iran. Scientific Reports, 10, 17473.	Maroufpoor, S., Fakheri-Fard, A., Shiri, J. (2019). Study of the spatial distribution of groundwater quality using soft computing and geostatistical models. ISH Journal of Hydraulic Engineering, 25, 232-238.	Approximated from Fig. 1 by Maroufpoor et al. (2019), Fig. 6 by Hosseini et al. (2019) and Fig. 2 by Parizi et al. (2020)
**Bandan Basin**	-	Hosseini, S. M., Parizi, E., Ataie-Ashtiani, B.,	-	-	Approximated from Fig. 6 by Hosseini et al. (2019)

Aquifer system	Broader aquifer system	Reference A	Reference B	Reference C	Steps to delineate
		Simmons, C. T. (2019). Assessment of sustainable groundwater resources management using integrated environmental index: Case studies across Iran. Science of the Total Environment, 676, 792-810.			
**Bandar Ganaveh Basin**	-	Hosseini, S. M., Parizi, E., Ataie-Ashtiani, B., Simmons, C. T. (2019). Assessment of sustainable groundwater resources management using integrated environmental index: Case studies across Iran. Science of the Total Environment, 676, 792-810.	-	-	Approximated from Fig. 6 by Hosseini et al. (2019)
**Bandar Lengeh Basin**	-	Bazrafshan, O., Parandin, F., Farokhzadeh, B. (2016). Assessment of hydro-meteorological drought effects on groundwater resources in Hormozgan region-South of Iran. Ecopersia, 4, 1569-1584.	-	-	Approximated from map on page 1571 of Bazrafshan et al. (2016)
**Bangkok Basin**	Lower Chao Phraya Basin	Sanford, W. E., Buapeng, S. (1996). Assessment of a groundwater flow model of the Bangkok Basin, Thailand, using carbon-14-based ages and paleohydrology. Hydrogeology Journal, 4, 26-40.	Phien-wej, N., Giao, P. H., Nutalaya, P. (2006). Land subsidence in Bangkok, Thailand. Engineering Geology, 82, 187-201.	Tanachaichoksirikun, P., Seeboonruang, U. (2020). Distributions of Groundwater Age under Climate Change of Thailand’s Lower Chao Phraya Basin. Water, 12, 3474.	Approximated from Fig. 1 by Sanford and Buapeng (1996), Fig. 1 by Phien-wej et al. (2006), and Fig. 1 by Tanachaichoksirikun and Seeboonruang (2020). The northern boundary of the subarea was approximated on the basis of the northern limit of the Bangkok Clay formation as mapped by Tanachaichoksirikun and Seeboonruang (2020) and also the prevelence of deep wells in Thailand's well completion dataset.

Aquifer system	Broader aquifer system	Reference A	Reference B	Reference C	Steps to delineate
**Baoju Uplift**	Weihe Basin	Zhang, W., Li, Y., Zhao, F., Han, W., Li, Y., Wang, Y., Holland, G. Zhou, Z. (2019). Using noble gases to trace groundwater evolution and assess helium accumulation in Weihe Basin, central China. Geochimica et Cosmochimica Acta, 251, 229-246.	-	-	Approximated from Fig. 1 by Zhang et al. (2019)
**Baotao Plain**	-	Bai, L., Wang, Y., Guo, Y., Zhou, Y., Liu, L., Yan, Z., Li, F., Xie, X. (2016). Health risk assessment research on heavy metals ingestion through groundwater drinking pathway for the residents in Baotou, China. Journal of Environmental Health, 78, 84-91.	-	-	Approximated from Fig. 1 by Bai et al. (2016); western boundary defined by Hetao Plain aquifer, and eastern boundary defined by the Hohhot Basin
**Baradwar Basin**	Chhattisgarh Basin	Central Ground Water Board (2012e). Aquifer Systems of Chhattisgarh. Central Ground Water Board Report, 73 pp. Accessed July 13, 2021 from http://cgwb.gov.in/AQM/Chhattisgarh.pdf	George, B.G., Ray, J.S., Kumar, S. (2019). Geochemistry of carbonate formations of the Chhattisgarh Supergroup, central India: implications for Mesoproterozoic global events. Canadian Journal of Earth Sciences, 56, 335-346.	Mukherjee, A., Ray, R. K., Tewari, D., Ingle, V. K., Sahoo, B. K., Khan, M. W. Y. (2014). Revisiting the stratigraphy of the Mesoproterozoic Chhattisgarh Supergroup, Bastar craton, India based on subsurface lithoinformation. Journal of Earth System Science, 123, 617-632.	Approximated from Plate IV by Central Ground Water Board (2012e), Fig. 1 by George et al. (2019), and Figs. 1 and 2 by Mukherjee et al. (2014)
**Bardsir Plain**	-	Abbasnejad, A., Mirzaie, A., Derakhshani, R., Esmaeilzadeh, E. (2013). Arsenic in groundwaters of the alluvial aquifer of Bardsir plain, SE Iran.	Hosseini, S. M., Parizi, E., Ataie-Ashtiani, B., Simmons, C. T. (2019). Assessment of sustainable groundwater resources management using integrated environmental	-	Approximated from Fig. 1 by Abbasnejad et al. (2013); "Ghariyatolarab basin defined in Approximated from Fig. 6 by Hosseini et al. (2019) included in Bardsir Plain

Aquifer system	Broader aquifer system	Reference A	Reference B	Reference C	Steps to delineate
		Environmental Earth Sciences, 69, 2549-2557.	index: Case studies across Iran. Science of the Total Environment, 676, 792-810.		
**Bari Doab**	Indus Basin	Hasan, M., Shang, Y., Akhter, G., Jin, W. (2019). Application of VES and ERT for delineation of fresh-saline interface in alluvial aquifers of Lower Bari Doab, Pakistan. Journal of Applied Geophysics, 164, 200-213.	Kumar, A., Singh, C. K. (2020). Arsenic enrichment in groundwater and associated health risk in Bari doab region of Indus basin, Punjab, India. Environmental Pollution, 256, 113324.	Basharat, M. (2014). Groundwater Environment and Evaluation of Long-Term Sustainability of the Aquifer under Lahore, Punjab, Pakistan. International Waterlogging and Salinity Research Institute, Pakistan Water and Power Development Authority Report (Project title: "Enhancing the groundwater management capacity in Asian cities through the development and application of groundwater sustainability index (GSII) in the context of global change"), 80 pp.	Approximated from Fig. 1 by Hasan et al. (2019), Fig. 1.1 by Basharat (2014), and Fig. 1 by Kumar and Singh (2020)
**Barind Tract and Central Floodplains**	Bengal Basin	Shamsudduha, M. (2007). Spatial Variability and Prediction Modeling of Groundwater Arsenic Distributions in the Shallowest Alluvial Aquifers in Bangladesh. Journal of Spatial Hydrology, 7, 33-46.	Ravenscroft, P., McArthur, J. M., Rahman, M. S. (2018). Identifying multiple deep aquifers in the Bengal Basin: Implications for resource management. Hydrological Processes, 32, 3615-3632.	Michael, H. A., Voss, C. I. (2009). Controls on groundwater flow in the Bengal Basin of India and Bangladesh: regional modeling analysis. Hydrogeology Journal, 17, 1561-1577.	Approximated from Fig. 1 by Shamsudduha (2007), Fig. 1 by Ravenscroft et al. (2018), and Fig. 1 by Michael and Voss (2009)
**Basht Basin**	-	Hosseini, S. M., Parizi, E., Ataie-Ashtiani, B., Simmons, C. T. (2019). Assessment of sustainable groundwater resources management using integrated environmental index: Case studies across Iran. Science of the Total	-	-	Approximated from Fig. 6 by Hosseini et al. (2019)

Aquifer system	Broader aquifer system	Reference A	Reference B	Reference C	Steps to delineate
		Environment, 676, 792-810.			
**Bastam Plain**	-	Bagheri, R., Nosrati, A., Jafari, H., Eggenkamp, H. G. M., Mozafari, M. (2019). Overexploitation hazards and salinization risks in crucial declining aquifers, chemo-isotopic approaches. Journal of hazardous materials, 369, 150-163.	-	-	Approximated from Fig. 1 by Bagheri et al. (2019)
**Bavanat Basin**	-	Hosseini, S. M., Parizi, E., Ataie-Ashtiani, B., Simmons, C. T. (2019). Assessment of sustainable groundwater resources management using integrated environmental index: Case studies across Iran. Science of the Total Environment, 676, 792-810.	-	-	Approximated from Fig. 6 by Hosseini et al. (2019)
**Bayazeh Basin**	-	Hosseini, S. M., Parizi, E., Ataie-Ashtiani, B., Simmons, C. T. (2019). Assessment of sustainable groundwater resources management using integrated environmental index: Case studies across Iran. Science of the Total Environment, 676, 792-810.	-	-	Approximated from Fig. 6 by Hosseini et al. (2019)
**Baza Basin**	-	Hidalgo, M. C., Cruz-Sanjulián, J. (2001). Groundwater composition, hydrochemical evolution and mass transfer in a regional detrital aquifer (Baza basin, southern Spain). Applied	-	-	Approximated from Fig. 1 by Hidalgo and Cruz-Sanjulián (2001)

Aquifer system	Broader aquifer system	Reference A	Reference B	Reference C	Steps to delineate
		Geochemistry, 16, 745-758.			
**Bazman Basin**	Jazmurian Depression	Rezaei, A., Hassani, H., Hassani, S., Jabbari, N., Mousavi, S. B. F., Rezaei, S. (2019). Evaluation of groundwater quality and heavy metal pollution indices in Bazman basin, southeastern Iran. Groundwater for Sustainable Development, 9, 100245.	-	-	Approximated from Figs. 1 and 2 by Rezaei et al. (2019)
**Bear Lake Valley**	-	Graham, W.G., Campbell, L.J. (1981). Groundwater resources of Idaho. Idaho Department of Water Resources Report, 61 pp. Accessed March 23, 2021 from https://idwr.idaho.gov/files/publications/198108-MISC-GW-Resources-ID.pdf	-	-	Approximated from Plate 1 of Graham and Campbell (1981).
**Bear River Bay**	-	Price, D. (1985). Ground Water in Utah's Densely Populated Wasatch Front Area the Challenge and the Choices. U.S. Geological Survey Water-Supply Paper 2232, 78 pp. Accessed March 27, 2021 from https://pubs.usgs.gov/wsp/2232/report.pdf	-	-	Approximated from Fig. 1 of Price (1985)
**Beaver Valley**	-	Sandberg, G. W. (1966). Ground-water resources of selected basins in southwestern Utah. US Geological Survey Open File Report. 66 pp. Accessed March 7, 2021 from https://cicwcd.org/wp-	Lee, W.T. (1908). Water resources of Beaver Valley, Utah. U.S. Geological Survey Water Supply Paper 217, 60 pp. Accessed April 6, 2021 from https://pubs.usgs.gov/wsp/0217/report.pdf	-	Approximated from the map on page 49 of Sandberg (1966) and plate 1 by Lee (1908)

Aquifer system	Broader aquifer system	Reference A	Reference B	Reference C	Steps to delineate
		content/uploads/2018/09/1966-tech-pub-13-ground-water-resources-of-selected-basins-in-southwestern-utah-by-g-w-sandburg-usgs.pdf			
**Bedford-Coldwater Valley**	-	California Department of Water Resources (2015e). California's groundwater update 2013 - Chapter 6: South Coast Hydrologic Region. California Department of Water Resources Report, 84 pp. Accessed April 27, 2022 via https://cawaterlibrary.net/wp-content/uploads/2017/05/GWU2013_Ch6_SouthCoast_Final.pdf	-	-	Approximated from Fig. 6-2 by California Department of Water Resources (2015e)
**Behbahan Plain**	-	Ehya, F., Saeedi, F. (2019). Assessment of groundwater quality in the Garmez area (Southeastern Khuzestan province, SW Iran) for drinking and irrigation uses. Carbonates and Evaporites, 34, 1443-1454.	-	-	Approximated from Fig. 1 by Ehya and Saeedi (2019)
**Behshar Basin**	-	Tavassoli, S., Mohammadi, F. (2021). Critically Assessment of Groundwater Quality Based on WQI and Its Vulnerability to Saltwater Intrusion in a Coastal City, Iran. Modern Advances in Geography, Environment and Earth Sciences, 2, 126-138.	-	-	Approximated from Fig. 1 by Tavassoli and Mohammadi (2021)
**Bekan Basin**	-	Hosseini, S. M., Parizi, E., Ataie-Ashtiani, B., Simmons, C. T. (2019).	-	-	Approximated from Fig. 6 by Hosseini et al. (2019)

Aquifer system	Broader aquifer system	Reference A	Reference B	Reference C	Steps to delineate
		Assessment of sustainable groundwater resources management using integrated environmental index: Case studies across Iran. Science of the Total Environment, 676, 792-810.			
**Bellefontaine Upland and Mad River Interlobate Plain**	Central Lowland Till Plain	Brockman, S. (1998). Physiographic regions of Ohio. Ohio Department of Natural Resources, Division of Geological Survey map. Accessed April 12, 20221 from https://www.epa.state.oh.us/portals/27/SIP/Nonattain/F2-physiographic_regions_of_Ohio.pdf	Fenneman, N.M., Johnson, D.W. (1946). Physiographic divisions of the conterminous United States. U.S. Geological Survey map, 1:7,000,000 Scale.	-	Broader Till Plain physiographic region approximated from Fenneman and Johnson (1946). Bellefontaine Upland and Mad River Interlobate Plain areas approximated from the map by Brockman (1998).
**Benkersandstein Aquifer System**	-	van Geldern, R., Baier, A., Subert, H. L., Kowol, S., Balk, L., Barth, J. A. (2014). Pleistocene paleo-groundwater as a pristine fresh water resource in southern Germany–evidence from stable and radiogenic isotopes. Science of the Total Environment, 496, 107-115.	-	-	Approximated from Fig. 1 by van Geldern et al. (2014)
**Beram Basin**	-	Hosseini, S. M., Parizi, E., Ataie-Ashtiani, B., Simmons, C. T. (2019). Assessment of sustainable groundwater resources management using integrated environmental index: Case studies across Iran. Science of the Total	-	-	Approximated from Fig. 6 by Hosseini et al. (2019); western margin extended south of Qaemyeh and eastern margin is northwest of Mahdiyeh

Aquifer system	Broader aquifer system	Reference A	Reference B	Reference C	Steps to delineate
		Environment, 676, 792-810.			
**Beryl-Enterprise Subarea**	Escalante Valley	Sandberg, G. W. (1964). Ground-water resources of selected basins in southwestern Utah. US Geological Survey Open File Report. 66 pp. Accessed March 7, 2021 from https://cicwcd.org/wp-content/uploads/2018/09/1966-tech-pub-13-ground-water-resources-of-selected-basins-in-southwestern-utah-by-g-w-sandburg-usgs.pdf	Fix, P. F., Nelson, W. B., Lofgren, B. E., Butler, R. G. (1950). Ground water in the Escalante Valley, Beaver, Iron, and Washington Counties, Utah Technical Publication 6. 102 pp. Accessed March 7, 2021 from https://waterrights.utah.gov/docSys/v920/w920/w9200085.pdf	-	Approximated from Fig. 1 of Fix et al. (1950) with subareas ("Districts") approximated from the map on page 49 of Sandberg (1966).
**Beyza-Zarghan Plain**	-	Hosseini, S. M., Parizi, E., Ataie-Ashtiani, B., Simmons, C. T. (2019). Assessment of sustainable groundwater resources management using integrated environmental index: Case studies across Iran. Science of the Total Environment, 676, 792-810.	Mojarrad, M., Rakhshandehrou, G. R., Monadi, R., Ghorbani, M. (2014). Investigating wellhead protection area (WHPA) assessment methods, case study: Beyza-Zarghan drinking wells. Proceedings of the 2nd International Conference of Water resources and wetands, 11-13 September, Tulceea, Romania (eds. P. Gâştescu, W. Marszelewski). 336-343. Accessed June 21, 2021 from https://www.limnology.ro/water2014/proceedings/45_Morteza.pdf	-	Approximated from Fig. 6 by Hosseini et al. (2019) and Fig. 1 by Mojarrad et al. (2014)
**Bhuj Sandstone Aquifer System**	-	Saha, D., Gor, N. (2020). A prolific aquifer system is in peril in arid Kachchh region of India. Groundwater for	-	-	Approximated from Fig. 6 by Saha and Gor (2020)

Aquifer system	Broader aquifer system	Reference A	Reference B	Reference C	Steps to delineate
		Sustainable Development, 11, 100394.			
**Bierzo Basin**	-	Díaz González, T.E., Penas, Á. (2017). The high mountain area of northwestern Spain: The Cantabrian range, the Galician-Leonese mountains and the Bierzo trench. The Vegetation of the Iberian Peninsula, 1, 251-321.	de la Losa, A., Moreno, L., Nunez, E.L. (2010). Calidad química de las aguas subterráneas en una zona de actividad minera (Cuenca del Bierzo- León). Boletín Geológico y Minero, 121, 103-122.	-	Approximated from Fig. 1 and descriptions by Díaz González and Penas (2017), and Fig. 1 by de la Losa (2010)
**Big Bear Valley**	-	Flint, L. E., Brandt, J., Christensen, A. H.,Flint, A.L., Hevesi, J.A., Jachens, R., Kulongoski, J.T., Martin, P., Sneed, M. (2012). Geohydrology of Big Bear Valley, California: phase 1--Geologic framework, recharge, and preliminary assessment of the source and age of groundwater. US Geological Survey Scientific Investigations Report 2012-5100, 122 pp. Accessed March 14, 2021 from https://pubs.usgs.gov/sir/2012/5100/pdf/sir20125100.pdf	-	-	Approximated from Fig. 1 of Flint et al. (2012)
**Big Chino Valley**	-	Langenheim, V. E., Duval, J. S., Wirt, L., DeWitt, E. (2000). Preliminary report on geophysics of the Verde River headwaters region, Arizona: US Geological Survey Open-File Report 00-403, 28 pp. Accessed March 12, 2021 from https://pubs.usgs.gov/of/2000/0403/pdf/of00-403p.pdf	Kennedy, J.R., Kahler, L.M., Read, A.L. (2019). Aquifer storage change and storage properties, 2010–2017, in the Big Chino Subbasin, Yavapai County, Arizona: U.S. Geological Survey Scientific Investigations Report 2019–5060, 39 pp. Accessed November 29, 2021 from	-	Approximated from map on pp. 12 of Langenheim et al. (2000) and Fig. 2 by Kennedy et al. (2019)

Aquifer system	Broader aquifer system	Reference A	Reference B	Reference C	Steps to delineate
			https://pubs.usgs.gov/sir/2019/5060/sir20195060.pdf		
**Big Lost River Valley**	Eastern Snake River Plain	Graham, W.G., Campbell, L.J. (1981). Groundwater resources of Idaho. Idaho Department of Water Resources Report, 61 pp. Accessed March 23, 2021 from https://idwr.idaho.gov/files/publications/198108-MISC-GW-Resources-ID.pdf	-	-	Approximated from Plate 1 of Graham and Campbell (1981).
**Big Sandy Valley**	-	Morrison, R.B. (1940). Ground-water resources of the Big Sandy Valley, Mohave County, Arizona. U.S. Geological Survey Report. 8 pp. Accessed April 15, 2021 from https://azmemory.azlibrary.gov/digital/collection/statepubs/id/6455/	-	-	Approximated from "Map of Big Sandy Valley" on page 8 of Morrison (1940).
**Big Valley**	-	California Department of Water Resources (2015j). California's groundwater update 2013 - Chapter 3: North Coast Hydrologic Region. California Department of Water Resources Report, 80 pp. Accessed April 27, 2022 via https://water.ca.gov/-/media/DWR-Website/Web-Pages/Programs/Groundwater-Management/Bulletin-118/Files/Statewide-Reports/GWU2013_Ch3_NorthCoast_Final.pdf	-	-	Approximated from Fig. 3-2 by California Department of Water Resources (2015j)
**Big Valley in northern California**	-	California Department of Water Resources (2015i). California's groundwater	-	-	Approximated from Fig. 7-2 by California Department of Water Resources (2015i)

Aquifer system	Broader aquifer system	Reference A	Reference B	Reference C	Steps to delineate
		update 2013 - Chapter 7: Sacramento River Hydrologic Region. California Department of Water Resources Report, 126 pp. Accessed April 27, 2022 via https://water.ca.gov/-/media/DWR-Website/Web-Pages/Programs/Groundwater-Management/Bulletin-118/Files/Statewide-Reports/GWU2013_Ch7_SacramentoRiver_Final.pdf			
**Bighorn Basin**	-	Tauchen, P, Bartos, T.T., Clarey, K.E., Quillinan, S.A., Hallberg, L.L., Clark, M.L., Thompson, M., Gribb, N., Worman, B., Gracias, T. (2012). Wind/Bighorn River Basin Water Plan Update Groundwater Study Level 1 (2008–2011). Groundwater Determination. Wyoming Water Development Commission Technical Memorandum, 397 pp. Accessed March 29, 2021 from https://waterplan.state.wy.us/plan/bighorn/2010/gw-finalrept/gw-finalrept.pdf	Fisher, C.A. (1906). Geology and water resources of the Bighorn Basin, Wyoming. US Geological Survey Professional Paper 53, 97 pp. Accessed November 29, 2021 from https://pubs.usgs.gov/pp/0053/report.pdf	-	Approximate from maps in Chapter 3 of Tauchen et al. (2012) and Plate XII by Fisher (1906)
**Biloela Basin**	-	Macphail, M. Hill, B., Carpenter, R., McKellar, J. (2014). Cenozoic oil-shale deposits in southeastern-central Queensland: palynostratigraphic age determinations and correlations for the Biloela Formation (Biloela Basin) in	-	-	Approximated from Fig. 1 by Macphail et al. (2014)

Aquifer system	Broader aquifer system	Reference A	Reference B	Reference C	Steps to delineate
		GSQ Monto 5. Queensland Geological Record 2014/01, 24 pp. Accessed October 8, 2021 from https://geoscience.data.qld.gov.au/report/cr089721			
**Bimorgh Basin**	-	Hosseini, S. M., Parizi, E., Ataie-Ashtiani, B., Simmons, C. T. (2019). Assessment of sustainable groundwater resources management using integrated environmental index: Case studies across Iran. Science of the Total Environment, 676, 792-810.	-	-	Approximated from Fig. 6 by Hosseini et al. (2019)
**Birjand Aquifer**	-	Aghlmand, R., Abbasi, A. (2019). Application of MODFLOW with boundary conditions analyses based on limited available observations: A case study of Birjand plain in East Iran. Water, 11, 1904.	Ghoochanian, E., Etebari, B., Akbarpour, A. (2013). Integrating groundwater management with WEAP and MODFLOW models (Case study: Birjand Plain, east of Iran). MODFLOW and More, 2-5.	Khashei-Siuki, A., & Sharifan, H. (2020). Comparison of AHP and FAHP methods in determining suitable areas for drinking water harvesting in Birjand aquifer. Iran. Groundwater for Sustainable Development, 10, 100328.	Approximated from Fig. 2 by Aghlmand Abbasi, A. (2019), Fig. 1 by Ghoochanian et al. (2013) and Fig. 1 by Khashei-Siuki and Sharifan (2020).
**Biscayne Aquifer**	-	Wacker, M.A., Cunningham, K.J., Williams, J.H. (2014). Geologic and hydrogeologic frameworks of the Biscayne aquifer in central Miami-Dade County, Florida: U.S. Geological Survey Scientific Investigations Report 2014–5138, 66 pp. Accessed March 31, 2021 from https://pubs.usgs.gov/sir/2014/5138/pdf/sir2014-5138.pdf	-	-	Approximated from Fig. 1 of Wacker et al. (2014).

Aquifer system	Broader aquifer system	Reference A	Reference B	Reference C	Steps to delineate
**Bishop Basin**	-	Radell (1989). Three-dimensional groundwater flow model use and application : Bishop Basin, Owens Valley, California. MSc Thesis, University of Arizona, 150 pp. Accessed April 27, 2022 via https://repository.arizona.edu/bitstream/handle/10150/192011/azu_td_hy_e9791_1989_192_sip1_w.pdf?sequence=1	-	-	Approximated from Fig. 2.1 of Radell (1989)
**Biyarjomand Basin**	-	Hosseini, S. M., Parizi, E., Ataie-Ashtiani, B., Simmons, C. T. (2019). Assessment of sustainable groundwater resources management using integrated environmental index: Case studies across Iran. Science of the Total Environment, 676, 792-810.	-	-	Approximated from Fig. 6 by Hosseini et al. (2019)
**Black Hills Uplift**	-	Driscoll, D.G., Carter, J.M., Williamson, J.E., Putnam, L.D. (2002). Hydrology of the Black Hills area, South Dakota. US Geological Survey Water-Resources Investigations Report 2002-4094, 158 pp. Accessed February 16, 2021 from https://pubs.usgs.gov/wri/wri024094/pdf/wri024094.pdf	Back, W., Hanshaw, B. B., Plummer, L. N., Rahn, P. H., Rightmire, C. T., Rubin, M. (1983). Process and rate of dedolomitization: mass transfer and 14C dating in a regional carbonate aquifer. Geological Society of America Bulletin, 94, 1415-1429.	-	Approximated from Fig. 2 of Driscoll et al. (2002) and Fig. 1 by Back et al. (1983)
**Black Warrior River Aquifer System (Eutaw and**	-	Kidd, R. E., Lambeth, D. S. (1995). Hydrogeology and ground-water quality in the Black Belt area of west-central Alabama, and	Strom, E. W., Mallory, M. J. (1995). Hydrogeology and simulation of ground-water flow in the Eutaw-McShan Aquifer and in the	Miller, J.A. (1990). Ground Water Atlas of the United States: Segment 6, Alabama, Florida, Georgia, South Carolina. U.S.	Mississippi portion approximated from Fig. 8 of Strom and Mallory (1995). Alabama portion approximated from Fig. 3 of Kidd and Lambeth (1990) and Fig. 73

Aquifer system	Broader aquifer system	Reference A	Reference B	Reference C	Steps to delineate
**McShan Formations and Tuscaloosa Group)**		estimated water use for aquaculture, 1990 (Vol. 94, No. 4074). US Department of the Interior, US Geological Survey. Accessed February 10, 2021 via https://citeseerx.ist.psu.edu/viewdoc/download?doi=10.1.1.1015.2227&rep=rep1&type=pdf	Tuscaloosa aquifer system in northeastern Mississippi. US Geological Survey Water-Resources Investigations Report 94-4223, 89 pp. Accessed November 29, 2021 from https://pubs.usgs.gov/wri/1994/4223/report.pdf	Geological Survey Hydrologic Atlas 730-G, 30 pp. Accessed April 5, 2021 from https://www.nrc.gov/docs/ML1706/ML17060B027.pdf	by Miller (1990) for easternmost extent. Best estimates were made to connect these two separate delineations in the northwest portion of the aquifer's extent within Alabama.
**Blok -e Sharra Basin**	Razan Basin	Hosseini, S. M., Parizi, E., Ataie-Ashtiani, B., Simmons, C. T. (2019). Assessment of sustainable groundwater resources management using integrated environmental index: Case studies across Iran. Science of the Total Environment, 676, 792-810.	-	-	Approximated from Fig. 6 by Hosseini et al. (2019)
**Blue Creek Valley**	-	Lopes, T.J., Evetts. D.M. (2005). Ground-Water pumpage and artificial recharge estimates for calendar year 2000 and average annual natural recharge and interbasin flow by hydrographic area, Nevada. Accessed April 25, 2022 via https://pubs.usgs.gov/sir/2004/5239/sir2004-5239.pdf	Harrill, J.R., Prudic, D.E. (1998). Aquifer systems in the Great Basin region of Nevada, Utah, and adjacent states; summary report. US Geological Survey Professional Paper 1409-A, 75 pp. Accessed April 26, 2022 via https://pubs.usgs.gov/pp/1409a/report.pdf	-	Approximated from Fig. 1 by Lopes and Evetts (2005), and Fig. 2 by Harrill and Prudic (1998). Well completion records, physiography and drainage were used to delinate areas.
**Blue Mountains and Clearwater Embayment**	Columbia Plateau Regional Aquifer System	Kahle, S.C., Morgan, D.S., Welch, W.B., Ely, S.R., Vaccaro, J.J., Orzol, L.L. (2011). Hydrogeologic Framework and Hydrologic Budget Components of the Columbia Plateau Regional	Graham, W.G., Campbell, L.J. (1981). Groundwater resources of Idaho. Idaho Department of Water Resources Report, 61 pp. Accessed March 23, 2021 from	-	Physiographic Provinces (Blue Mountains, Clearwater Embayment) approximated from Fig. 1 of Kahle et al. (2011). Idaho portion approximated following Plate 1 of Graham and Campbell (1981; see "Clearwater Uplands

Aquifer system	Broader aquifer system	Reference A	Reference B	Reference C	Steps to delineate
		Aquifer System, Washington, Oregon, and Idaho. US Geological Survey Scientific Investigations Report 2011–5124, 80 pp. Accessed February 16, 2021 from https://pubs.usgs.gov/sir/2011/5124/pdf/sir20115124.pdf	https://idwr.idaho.gov/files/publications/198108-MISC-GW-Resources-ID.pdf		(Mussel Shell Basalt Subsection"). Columbia River Aquifer System approximated from Figs. 1 and 10 of Kahle et al. (2011).
**Blue River Basin**	-	Barkmann, P. E., Broes, L.D., Palkovic, M.J., Hopkins, J.C., Bird, K.S., Sebol, L.A., Fitzgerald. F.S. (2020). ON-010 Colorado Groundwater Atlas. Geohydrology. Colorado Geological Survey, Golden, CO. ON-010 Colorado Groundwater Atlas. Accessed March 29, 2021 from https://coloradogeologicalsurvey.org/water/colorado-groundwater-atlas/	-	-	Approximated from Fig. 12-01 of Barkmann et al. (2020).
**Bluffton Till Plain**	Central Lowland Till Plain	Gray, H.H. (2001). Map of Indiana showing physiographic divisions. Indiana Geological Survey Miscellaneous Map 69, 15 pp. Accessed April 12, 2021 from https://igws.indiana.edu/ReferenceDocs/Maps/PhysiographicRegions.pdf	Brockman, S. (1998). Physiographic regions of Ohio. Ohio Department of Natural Resources, Division of Geological Survey map. Accessed April 12, 20221 from https://www.epa.state.oh.us/portals/27/SIP/Nonattain/F2-physiographic_regions_of_Ohio.pdf	Fenneman, N.M., Johnson, D.W. (1946). Physiographic divisions of the conterminous United States. U.S. Geological Survey map, 1:7,000,000 Scale.	Broader Till Plain physiographic region approximated from Fenneman and Johnson (1946). Bluffton Till Plain approximated for Indiana from map by Gray (2001) and for Ohio following the approximate border of the "Central Ohio Clayey Till Plain" defined in map by Brockman (1998).
**Boein-Miandasht Aquifer**	-	Yonesi, H., Arshia, A., Torabipoudeh, H., Shahinejad, B., Sayedipour,	-	-	Approximated from Fig. 6 by Hosseini et al. (2019)

Aquifer system	Broader aquifer system	Reference A	Reference B	Reference C	Steps to delineate
		M., Vahdatpour, N. (2020). Evaluating Groundwater Quality in Zayandehrood Southern Sub-Basin Aquifers. Desert Ecosystem Engineering Journal, 9, 103-115.			
**Boise Valley and Homedale Murphy Area**	Western Snake River Plain	Graham, W.G., Campbell, L.J. (1981). Groundwater resources of Idaho. Idaho Department of Water Resources Report, 61 pp. Accessed March 23, 2021 from https://idwr.idaho.gov/files/publications/198108-MISC-GW-Resources-ID.pdf	Lindholm, G. F. (1996). Summary of the Snake River Plain regional aquifer-system analysis in Idaho and eastern Oregon. U.S Geological Survey Professional Paper 1408-A. 59 pp. Accessed March 1, 2021 via https://pubs.usgs.gov/pp/1408a/report.pdf	-	Approximated from Plate 1 of Graham and Campbell (1981). Zones "30" and "32" merged into one; locations of completed wells were used to guide the western boundary of the area. Broader portions of the Snake River Plain (i.e., Central, Western and Eastern) were approximated from Fig. 1 of Lindholm (1996).
**Bojnourd Plain**	-	Kheirandish, M., Rahimi, H., Kamaliardakani, M., Salim, R. (2020). Obtaining the effect of sewage network on groundwater quality using MT3DMS code: Case study on Bojnourd plain. Groundwater for Sustainable Development, 11, 100439.	Dadgar, M. A., Zeaieanfirouzabadi, P., Dashti, M., Porhemmat, R. (2017). Extracting of prospective groundwater potential zones using remote sensing data, GIS, and a probabilistic approach in Bojnourd basin, NE of Iran. Arabian Journal of Geosciences, 10(5), 114.	-	Approximated from Fig. 1 by Kheirandish et al. (2020) and Fig. 1 by Dadgar et al. (2017)
**Bone Spring-Victorio Peak Aquifer and Dell Valley**	-	Ashworth, J. (2001). Bone Spring-Victorio Peak aquifer of the Dell Valley region of Texas. Texas Water Development Board Report, Chapter 10, 18 pp. Accessed April 27, 2022 via https://www.twdb.texas.gov/publications/reports/numbered_reports/doc/R356/Chapter10.pdf	-	-	Approximated from Fig. 10-2 of Ashworth (2001)

Aquifer system	Broader aquifer system	Reference A	Reference B	Reference C	Steps to delineate
**Bonifacio Coastal Aquifer**	-	Santoni, S., Huneau, F., Garel, E., Aquilina, L., Vergnaud-Ayraud, V., Labasque, T., Celle-Jeanton, H. (2016). Strontium isotopes as tracers of water-rocks interactions, mixing processes and residence time indicator of groundwater within the granite-carbonate coastal aquifer of Bonifacio (Corsica, France). Science of the Total Environment, 573, 233-246.	-	-	Approximated from Fig. 1 by Santoni et al. (2016)
**Booborowie Valley**	-	Department of Environment, Water and Natural Resources (2011). Booborowie Valley groundwater level and salinity status report. Department of Environment, Water and Natural Resources Report, 6 pp. Accessed May 7, 2023 via https://www.waterconnect.sa.gov.au/Content/Publications/DEW/Booborowie_Valley_Status_Report_2011.pdf	-	-	Approximated from Fig. 2 by Department of Environment, Water and Natural Resources (2011)
**Boqueron Aquifer**	-	Rupérez-Moreno, C., Pérez-Sánchez, J., Senent-Aparicio, J., del Pilar Flores-Asenjo, M. (2015). The economic value of conjoint local management in water resources: Results from a contingent valuation in the Boquerón aquifer (Albacete, SE Spain). Science of the	-	-	Approximated from Fig. 1 by Rupérez-Moreno et al. (2015)

Aquifer system	Broader aquifer system	Reference A	Reference B	Reference C	Steps to delineate
		Total Environment, 532, 255-264.			
**Borazjan Aquifer**	-	Hemmati, F., Sajadi, Z., Jamshidi, A.R. (2014). Assessment of groundwater vulnerability in the Borazjan Aquifer of Bushehr, south of Iran, using GIS technique. Indian Journal of Fundamental and Applied Life Sciences, 4, 415-425.	-	-	Approximated from Fig. 1 by Hemmati et al. (2014)
**Boroujen Aquifer**	-	Sureshjani, M. K., Amanipoor, H., Battaleb-Looie, S. (2020). The effects of industrial wastewater on groundwater quality of the Boroujen aquifer, Southwest Iran. Natural Resources Research, 29, 3719-3741.	-	-	Approximated from Fig. 1 by Sureshjani et al. (2020)
**Borujerd - Doroud Plain**	-	Sabzevari, Y., Nasrolahi, A. H., Yonesi, H. A. (2020). Investigation of temporal-spatial variations of groundwater resources quality in Borujerd-Dorood Plain. Irrigation and Water Engineering, 11, 150-167.	-	-	Approximated from Fig. 1 by Sabzevari et al. (2020)
**Bosrouyeh Basin**	-	Hosseini, S. M., Parizi, E., Ataie-Ashtiani, B., Simmons, C. T. (2019). Assessment of sustainable groundwater resources management using integrated environmental index: Case studies across Iran. Science of the Total Environment, 676, 792-810.	-	-	Approximated from Fig. 6 by Hosseini et al. (2019)
**Bostanabad Basin**	-	Emami, S., Hemmati, M., Arvanaghi, H. (2018).	-	-	Approximated from Fig. 2 by Emami et al. (2019)

Aquifer system	Broader aquifer system	Reference A	Reference B	Reference C	Steps to delineate
		Performance evaluation of Imperialist Competitive and Genetic algorithm for estimating groundwater quality parameters (case study: Bostanabad plain). Hydrogeology, 2, 44-53.			
**Brahmani Delta and Northern Utkal Coastal Plain**	Utkal Coastal Plain	Kar, G., Sahoo, N., Kumar, A., Das, M., Roychoudhury, S., Chandra, D. (2007). Intergrated Technologies to enhance productivity of seasonal deep waterlogged areas. Water Technology Centre for Eastern Region Research Bulletin No. 40, 56 pp., Accessed July 14, 2021 from http://www.iiwm.res.in/pdf/Bulletin_40.pdf	Sahu, S., Gogoi, U., Nayak, N. C. (2020). Patterns of groundwater chemistry: implications of groundwater flow and the relation with groundwater fluoride contamination in the phreatic aquifer of Odisha, India. Arabian Journal of Geosciences, 13, 1-19.	Central Ground Water Board (2013c). Ground water information booklet, Bhadrak District, Orissa. Centeal Ground Water Board Report, 33 pp. Accessed July 14, 2021 from http://cgwb.gov.in/District_Profile/Orissa/BHADRAK%20.pdf	Approximated from Fig. 1 by Sahu et al. (2020), and Plate IV by Central Ground Water Board (2013c). Location of broader system detailed in section 5.0 and Fig. 4 by Kar et al. (2007).
**Bridgeport Valley**	-	California Department of Water Resources (2015d). California's groundwater update 2013 - Chapter 10: North Lahontan Hydrologic Region. California Department of Water Resources Report, 72 pp. Accessed April 27, 2022 via https://water.ca.gov/-/media/DWR-Website/Web-Pages/Programs/Groundwater-Management/Bulletin-118/Files/Statewide-Reports/GWU2013_Ch10_NorthLahontan_Final.pdf	-	-	Approximated from Fig. 10-2 by California Department of Water Resources (2015d)
**Broome-Wallal Aquifer System in West**	-	Government of Western Australia's Department of Water (2012). West Canning Basin groundwater allocation limit report.	Hanna, J. (2014). Influence of Conceptual Model Uncertainty on Recharge Processes for the Wallal Aquifer System in the West	Meredith, K. T., Han, L. F., Cendón, D. I., Crawford, J., Hankin, S., Peterson, M., Hollins, S. E. (2018). Evolution of dissolved	Approximated from Fig. 1 by Hanna (2014) and Fig. 1 by Meredith et al. (2018). West Canning Basin boundary approximated from Fig. 1 by

Aquifer system	Broader aquifer system	Reference A	Reference B	Reference C	Steps to delineate
**Canning Basin**		Water resource allocation and planning report series, Report No. 52, 61 pp. Accessed October 4, 2021 via https://www.water.wa.gov.au/__data/assets/pdf_file/0003/4998/103786.pdf	Canning Basin, Western Australia. MSc Thesis, University of Western Australia, 92 pp. Accessed October 4, 2021 from https://api.research-repository.uwa.edu.au/ws/portalfiles/portal/4541657/Hanna_Jonathan_2014.pdf	inorganic carbon in groundwater recharged by cyclones and groundwater age estimations using the 14C statistical approach. Geochimica et Cosmochimica Acta, 220, 483-498.	Government of Western Australia's Department of Water (2012).
**Bruneau-Grandview Area**	Western Snake River Plain	Graham, W.G., Campbell, L.J. (1981). Groundwater resources of Idaho. Idaho Department of Water Resources Report, 61 pp. Accessed March 23, 2021 from https://idwr.idaho.gov/files/publications/198108-MISC-GW-Resources-ID.pdf	Lindholm, G. F. (1996). Summary of the Snake River Plain regional aquifer-system analysis in Idaho and eastern Oregon. U.S Geological Survey Professional Paper 1408-A. 59 pp. Accessed March 1, 2021 via https://pubs.usgs.gov/pp/1408a/report.pdf	-	Approximated from Plate 1 of Graham and Campbell (1981). Broader portions of the Snake River Plain (i.e., Central, Western and Eastern) were approximated from Fig. 1 of Lindholm (1996).
**Brussels Sands**	-	Peeters, L., Batelaan, O., Dassargues, A. (2007). Identification and quantification of sources of major solutes in a sandy, phreatic aquifer in Central Belgium through ionic ratios and geochemical mass-balance modelling. In XXXV IAH Congress, International Association of Hydrogeologists, 10 pp. Accessed April 24, 2023 via https://orbi.uliege.be/bitstream/2268/3587/1/publi159-2007.pdf	-	-	Approximated from Fig. 1 by Peeters et al. (2007)
**Buena Vista Valley in northern Nevada**	-	Welch, A. H., Sorey, M. L., Olmsted, F. H. (1981). Hydrothermal system in Southern Grass Valley, Pershing County, Nevada.	-	-	Approximated from Fig. 2 of Welch et al. (1981)

Aquifer system	Broader aquifer system	Reference A	Reference B	Reference C	Steps to delineate
		U.S. Geological Survey Open-File Report 81-915, 200 pp. Accessed March 22, 2021 from https://www.osti.gov/servlets/purl/5119283-5mJ8YB/			
**Bukan Basin**	-	Hosseini, S. M., Parizi, E., Ataie-Ashtiani, B., Simmons, C. T. (2019). Assessment of sustainable groundwater resources management using integrated environmental index: Case studies across Iran. Science of the Total Environment, 676, 792-810.	-	-	Approximated from Fig. 6 by Hosseini et al. (2019)
**Bundaberg Aquifer**	-	Pettifer, G. (2004). Bundaberg Groundwater Investigation, Australia – A Case For The Benefits Of Extensive Use Of Geophysics In Groundwater Investigations. Conference Proceedings, 17th EEGS Symposium on the Application of Geophysics to Engineering and Environmental Problems, February, 2004. Accessed September 27, 2021 via https://library.seg.org/doi/pdf/10.4133/1.2923407	-	-	Approximated from Fig. 1 by Pettifer (2004).
**Burdekin Delta**	-	Narayan, K. A., Schleeberger, C., Bristow, K. L. (2007). Modelling seawater intrusion in the Burdekin Delta irrigation area, North Queensland, Australia. Agricultural	-	-	Approximated from Fig. 1 by Narayan et al. (2007)

Aquifer system	Broader aquifer system	Reference A	Reference B	Reference C	Steps to delineate
		Water Management, 89, 217-228.			
**Bushkan Plain**	-	Saffari, A., Jan Ahmadi, M., Raeati Shavazi, M. (2015). Site Selection for suitable flood spreadingand artificial feeding through hybrid, AHP-Fuzzy Model Case Study: (Bushkan Plain, Bushehr Province). Hydrogeomorphology, 1, 81-97.	-	-	Approximated from Fig. 1 by Saffari et al. (2015)
**Butte Basin**	Sacramento Valley of the Central Valley **	California Department of Water Resources (2015i). California's groundwater update 2013 - Chapter 7: Sacramento River Hydrologic Region. California Department of Water Resources Report, 126 pp. Accessed April 27, 2022 via https://water.ca.gov/-/media/DWR-Website/Web-Pages/Programs/Groundwater-Management/Bulletin-118/Files/Statewide-Reports/GWU2013_Ch7_SacramentoRiver_Final.pdf	-	-	Approximated from Fig. 7-2 by California Department of Water Resources (2015i)
**Butte Valley**	Upper Klamath Basin	Wood, P. R. (1960). Geology and ground-water features of the Butte Valley region, Siskiyou County, California. Geological Survey Water-Supply Paper 1491, 155 pp. Accessed March 20, 2021 from https://pdfs.semanticscholar.org/a448/a58a3c1ac120d400d26f75b74512f0a868a4.pdf	-	-	Approximated from Fig. 1 of Wood (1960)

Aquifer system	Broader aquifer system	Reference A	Reference B	Reference C	Steps to delineate
**Cache Valley**	-	Graham, W.G., Campbell, L.J. (1981). Groundwater resources of Idaho. Idaho Department of Water Resources Report, 61 pp. Accessed March 23, 2021 from https://idwr.idaho.gov/files/publications/198108-MISC-GW-Resources-ID.pdf	Bjorklund, L. J., McGreevy, L. J. (1971). Ground-water resources of Cache Valley, Utah and Idaho. Utah Department of Natural Resources, Division of Water Rights Technical Publication No. 36, 85 pp. Accessed March 27, 2021 from https://waterrights.utah.gov/docSys/v920/w920/w920008y.pdf	-	Approximated from Plate 1 of Graham and Campbell (1981) and Fig. 1 of Bjorklund and McGreevy (1971).
**Cactus Flat**	-	Lopes, T.J., Evetts. D.M. (2005). Ground-Water pumpage and artificial recharge estimates for calendar year 2000 and average annual natural recharge and interbasin flow by hydrographic area, Nevada. Accessed April 25, 2022 via https://pubs.usgs.gov/sir/2004/5239/sir2004-5239.pdf	Harrill, J.R., Prudic, D.E. (1998). Aquifer systems in the Great Basin region of Nevada, Utah, and adjacent states; summary report. US Geological Survey Professional Paper 1409-A, 75 pp. Accessed April 26, 2022 via https://pubs.usgs.gov/pp/1409a/report.pdf	-	Approximated from Fig. 1 by Lopes and Evetts (2005), and Fig. 2 by Harrill and Prudic (1998). Well completion records, physiography and drainage were used to delinate areas.
**Cadenas Marchigue y Yerbas Buenas**	-	Direccion General de Aguas (2017). Inventario Nacional de acuiferos. Ministerio De Obras Públicas (Gobierno De Chile) report number 403, 60 pp. Accessed May 17, 2021 from https://snia.mop.gob.cl/sad/SUB5748.pdf	-	-	Approximated from map on page 42 by Direccion General de Aguas (2017).
**Cahuilla Valley**	-	California Department of Water Resources (2015e). California's groundwater update 2013 - Chapter 6: South Coast Hydrologic Region. California Department of Water	-	-	Approximated from Fig. 6-2 by California Department of Water Resources (2015e)

Aquifer system	Broader aquifer system	Reference A	Reference B	Reference C	Steps to delineate
		Resources Report, 84 pp. Accessed April 27, 2022 via https://cawaterlibrary.net/wp-content/uploads/2017/05/GWU2013_Ch6_SouthCoast_Final.pdf			
**Calama Basin**	-	Herrera, C., Godfrey, L., Urrutia, J., Custodio, E., Jordan, T., Jódar, J., Delgado, K., Barrenechea, F. (2021). Recharge and residence times of groundwater in hyper arid areas: The confined aquifer of Calama, Loa River Basin, Atacama Desert, Chile. Science of The Total Environment, 752, 141847.	Godfrey, L. V., Herrera, C., Burr, G. S., Houston, J., Aguirre, I., Jordan, T. E. (2021). δ13C and 14C activity of groundwater DOC and DIC in the volcanically active and arid Loa Basin of northern Chile. Journal of Hydrology, 595, 125987.	-	Approximated from Fig. 1 by Herrera et al. (2021) and Fig. 1 by Godfrey et al. (2021)
**Calera Aquifer**	-	González-Trinidad, J., Pacheco-Guerrero, A., Júnez-Ferreira, H., Bautista-Capetillo, C., & Hernández-Antonio, A. (2017). Identifying groundwater recharge sites through environmental stable isotopes in an alluvial aquifer. Water, 9, 569.	-	-	Approxiamted from Fig. 1 by González-Trinidad et al. (2017).
**California Wash**	-	Lopes, T.J., Evetts. D.M. (2005). Ground-Water pumpage and artificial recharge estimates for calendar year 2000 and average annual natural recharge and interbasin flow by hydrographic area, Nevada. Accessed April 25, 2022 via	Harrill, J.R., Prudic, D.E. (1998). Aquifer systems in the Great Basin region of Nevada, Utah, and adjacent states; summary report. US Geological Survey Professional Paper 1409-A, 75 pp. Accessed April 26, 2022 via https://pubs.usgs.gov/pp/1409a/report.pdf	-	Approximated from Fig. 1 by Lopes and Evetts (2005), and Fig. 2 by Harrill and Prudic (1998). Well completion records, physiography and drainage were used to delinate areas.

Aquifer system	Broader aquifer system	Reference A	Reference B	Reference C	Steps to delineate
		https://pubs.usgs.gov/sir/2004/5239/sir2004-5239.pdf			
**Camargue Plain**	-	Torres-Rondon, L., Carrière, S. D., Chalikakis, K., Valles, V. (2013). An integrative geological and geophysical approach to characterize a superficial deltaic aquifer in the Camargue plain, France. Comptes Rendus Geoscience, 345, 241-250.	de Montety, V., Radakovitch, O., Vallet-Coulomb, C., Blavoux, B., Hermitte, D., Valles, V. (2008). Origin of groundwater salinity and hydrogeochemical processes in a confined coastal aquifer: case of the Rhône delta (Southern France). Applied Geochemistry, 23, 2337-2349.	-	Approximated from Fig. 1 by Torres-Rondon et al. (2013) and Fig. 1 by de Montety et al. (2008).
**Camas Prairie**	-	Graham, W.G., Campbell, L.J. (1981). Groundwater resources of Idaho. Idaho Department of Water Resources Report, 61 pp. Accessed March 23, 2021 from https://idwr.idaho.gov/files/publications/198108-MISC-GW-Resources-ID.pdf	-	-	Approximated from Plate 1 of Graham and Campbell (1981).
**Campina de Faro and Sao Joao da Venda Aquifers**	-	Moura, A., Velho, J.L. (2012). Aguas Subterraneas. Chapter 57 in Recursos Geologicos de Portugal, pp. 523-536. Accessed May 1, 2023 via https://dspace.uevora.pt/rdpc/bitstream/10174/7918/1/2011_Aveiro.pdf	-	-	Approximated from Fig. 57.8 by Moura and Velho (2012)
**Campo de Belchite and Cubeta de Azuara Aquifers**	-	Carceller-Layel, T., Costa-Alandí, C., Coloma-López, P., García-Vera, M. Á., & San Román-Saldaña, J. (2007). Groundwater in the central sector of the Ebro	-	-	Approximated from Fig. 3 by Carceller-Layel et al. (2007).

Aquifer system	Broader aquifer system	Reference A	Reference B	Reference C	Steps to delineate
		Basin. Water Resources Development, 23, 165-187.			
**Campo de Carinena Aquifer**	-	Carceller-Layel, T., Costa-Alandí, C., Coloma-López, P., García-Vera, M. Á., & San Román-Saldaña, J. (2007). Groundwater in the central sector of the Ebro Basin. Water Resources Development, 23, 165-187.	-	-	Approximated from Fig. 3 by Carceller-Layel et al. (2007).
**Campo de Cartagena Multilayered Aquifer**	-	Baudron, P., Barbecot, F., Aróstegui, J. L. G., Leduc, C., Travi, Y., Martinez-Vicente, D. (2014). Impacts of human activities on recharge in a multilayered semiarid aquifer (Campo de Cartagena, SE Spain). Hydrological Processes, 28, 2223-2236.	Jiménez-Martínez, J., Aravena, R., & Candela, L. (2011). The role of leaky boreholes in the contamination of a regional confined aquifer. A case study: the Campo de Cartagena region, Spain. Water, Air, & Soil Pollution, 215, 311-327.	-	Approximated from Fig. 1 by Baudron et al. (2014) and Fig. 1 by Jiménez-Martínez et al. (2011).
**Campo de Dalias Coastal Aquifer**	-	Andreu, J. M., Alcalá, F. J., Vallejos, A., Pulido-Bosch, A. (2011). Recharge to mountainous carbonated aquifers in SE Spain: Different approaches and new challenges. Journal of Arid Environments, 75, 1262-1270.	Pulido-Velazquez, D., Ahlfeld, D., Andreu, J., Sahuquillo, A. (2008). Reducing the computational cost of unconfined groundwater flow in conjunctive-use models at basin scale assuming linear behaviour: The case of Adra-Campo de Dalías. Journal of hydrology, 353, 159-174.	-	Approximated from Fig. 1 by Andreu et al. (2011) and Fig. 1 by Pulido-Velazquez et al. (2008)
**Campo De Montiel Aquifer**	Upper Guadiana Basin	Martínez-Santos, P., Castaño-Castaño, S., Hernández-Espriú, A. (2018). Revisiting groundwater overdraft based on the experience of the Mancha Occidental Aquifer, Spain.	-	-	Approximated from Fig. 1 by Martínez-Santos et al. (2018)

Aquifer system	Broader aquifer system	Reference A	Reference B	Reference C	Steps to delineate
		Hydrogeology Journal, 26, 1083-1097.			
**Campo Mina Aquifer**	-	Villanueva-Hernández, H., Tovar-Cabañas, R., Vargas-Castilleja, R. (2019). Classification of aquifers in the Mina field, Nuevo Leon, using geographic information systems. Tecnología y ciencias del agua, 10, 96-123.	-	-	Approximated from Fig. 1 by Villanueva-Hernández et al. (2019)
**Campos de Nijar Aquifer**	-	Morell, I. (2003). Acuíferos detríticos costeros. Hidrogeología y Aguas Subterráneas, 1, 31-44.	-	-	Approximated from Fig. 5 by Morell (2003)
**Canadian River Alluvial Aquifer**	-	Ellis, J.H., Mashburn, S.L., Graves, G.M., Peterson, S.M., Smith, S.J., Fuhrig, L.T., Wagner, D.L., Sanford, J.E. (2017). Hydrogeology and simulation of groundwater flow and analysis of projected water use for the Canadian River alluvial aquifer, western and central Oklahoma. U.S. Geological Survey Scientific Investigations Report 2016–5180, 64 pp. Accessed April 5, 2021 from https://pubs.usgs.gov/sir/2016/5180/sir20165180.pdf	-	-	Approximated from Fig. 1 of Ellis et al. (2017).
**Canatlan Valley**	-	CONAGUA (2020g). Actualización de la disponibilidad media anual de agua en el acuífero Valle de Canatlan (1002), Estado de Durango. 40 pp. Accessed January 24, 2022 via	-	-	Approximated from Fig. 1 by CONAGUA (2020g) and well completion data

Aquifer system	Broader aquifer system	Reference A	Reference B	Reference C	Steps to delineate
		https://sigagis.conagua.gob.mx/gas1/Edos_Acuiferos_18/durango/DR_1002.pdf			
**Canning Basin**	-	Government of Western Australia's Department of Water (2012). West Canning Basin groundwater allocation limit report. Water resource allocation and planning report series, Report No. 52, 61 pp. Accessed October 4, 2021 via https://www.water.wa.gov.au/__data/assets/pdf_file/0003/4998/103786.pdf	-	-	Approximated from Fig. 1 by Government of Western Australia's Department of Water (2012).
**Cape Cod Aquifer**	-	Frimpter, M.H., Gay, F.B. (1979). Chemical quality of ground water on Cape Cod, Massachusetts. U.S. Geological Survey Water-Resources Investigations Report 79-65, 20 pp. Accessed April 14, 2021 from https://pubs.usgs.gov/wri/1979/0065/report.pdf	Masterson, J.P., Walter, D.A. (2009). Hydrogeology and groundwater resources of the coastal aquifers of southeastern Massachusetts. U.S. Geological Survey Circular 1338, 16 pp. Accessed April 14, 2021 from https://pubs.usgs.gov/circ/circ1338/pdf/circular%202009-1338_508.pdf	-	Approximated from map on title page of Frimpter and Gay (1979) and Fig. 1 of Masterson and Walter (2009).
**Cape Flats Aquifer**	-	Adelana, S., Xu, Y., Vrbka, P. (2010). A conceptual model for the development and management of the Cape Flats aquifer, South Africa. Water SA, 36(4).	Umvoto Africa (2008). The assessment of water availability in the Berg Catchment (WMA 19) by means of Water Resource Related Models. Department of Water Affairs and Forestry Report, 153 pp. Accessed October 8, 2021 from https://www.dws.gov.za/Documents/Other/WMA/19/Reports/Rep9-Vol5-	Gxokwe, S., Xu, Y., Kanyerere, T. (2020). Scenarios analysis using water-sensitive urban design principles: a case study of the Cape Flats Aquifer in South Africa. Hydrogeology Journal, 28, https://doi.org/10.1007/s10040-020-02188-w	Approximated from Fig. 1 by Adelana et al. (2010), and Fig. 1.3 by Umvoto Africa (2008) and Fig. 1 by Gxokwe et al. (2020)

Aquifer system	Broader aquifer system	Reference A	Reference B	Reference C	Steps to delineate
			GW%20Cape%20Flats%20 Aquifer.pdf		
**Cara Sucia-San Pedro Aquifer**	-	Ministerio de Medio Ambiente y Recursos Natural (2021). Mapa Hidrogeológico de El Salvador, 1 pp. Accessed Februar 7, 2023 via https://www.sica.int/documentos/mapa-hidrogeologico-de-el-salvador_1_128021.html	-	-	Approximated from map by Ministerio de Medio Ambiente y Recursos Natural (2021)
**Carman Sand and Southern Carbonate Rock Aquifers**	Manitoba Carbonate Aquifer System	Betcher, R.N., Pupp, C., Grove, G. (1995). Groundwater in Manitoba: hydrogeology, quality concerns, management. Environment Canada, National Hydrology Research Institute Report No. C2-93017, 53 pp. Accessed May5, 2021 from https://web.viu.ca/earle/geol304/hg-manitoba.pdf	Ferguson, G.A., Betcher, R.N., Grasby, S.E. (2007). Hydrogeology of the Winnipeg formation in Manitoba, Canada. Hydrogeology Journal, 15, 573.	-	Approximated from Fig. 7 of Betcher et al. (1995); western boundary of area follows the Red River because saline water is common to the west (see Fig. 11 by Ferguson et al. (2007)) and the Carman Sand "..forms a valuable fresh water aquifer east of the Red River" (quote Betcher et al., 1995). Fluoride concentrations can be high in this area (see Fig. 16 by Betcher et al. 1995).
**Carmel Valley**	-	Kapple, G.W., Mitten, H.T., Durbin, T.J., Johnson, M.J. (1984). Analysis of the Carmel Valley alluvial ground-water basin, Monterey County, California US Geological Survey Water-Resources Investigations Report 83-4280, 50 pp. Accessed May 8, 2023 via https://pubs.usgs.gov/wri/1983/4280/report.pdf	-	-	Approximated from Fig. 1 by Kapple et al. (1984)
**Carpinteria Basin**	-	La Rocque, G.A., Upson, J. E., Worts Jr., G. F. (1950). Wells and water levels in principal ground-water	-	-	Approximated from Plate 1 of La Rocque et al. (1968)

Aquifer system	Broader aquifer system	Reference A	Reference B	Reference C	Steps to delineate
		basins in Santa Barbara County, California – Part 1. Carpenteria, Goleta and Santa Ynez Valleys, 1930-41. U.S. Geological Survey Water-SupplyPaper 1068. 465 pp. Accessed March 20, 2021 from https://pubs.usgs.gov/wsp/1068/report.pdf			
**Carson Valley**	Carson River Basin	Maurer, D. K. (2011). Geologic framework and hydrogeology of the middle Carson River Basin, Eagle, Dayton, and Churchill Valleys, West-Central Nevada. U. S. Geological Survey Scientific Investigations Report 2011–5055. 74 pp. Accessed March 10, 2021 from https://pubs.usgs.gov/sir/2011/5055/pdf/sir20115055.pdf	Naranjo, R.C., Welborn, T.L., Rosen, M.R . (2013). The distribution and modeling of nitrate transport in the Carson Valley alluvial aquifer, Douglas County, Nevada. US Geological Survey Scientific Investigations Report 2013–5136, 51 pp. Accessed April 11, 2022 via https://pubs.usgs.gov/sir/2013/5136/pdf/sir2013-5136.pdf	-	Approximated from Fig. 1 of Maurer et al. (2011) and Fig. 3 by Naranjo et al. (2013)
**Castellon Plain**	-	Hernández, F., Marín, J. M., Pozo, Ó. J., Sancho, J. V., López, F. J., Morell, I. (2008). Pesticide residues and transformation products in groundwater from a Spanish agricultural region on the Mediterranean Coast. International Journal of Environmental and Analytical Chemistry, 88, 409-424.	-	-	Approximated from Fig. 1 by Hernández et al. (2008)
**Castle Hayne Aquifer**	-	Lyke, W.L., Coble, R.W. (1987). Regional study of the Castle Hayne Aquifer of	Eimers, J.L. Daniel III, C.C., Coble, R.W. (1994). Hydrogeology and	Winner Jr., M.D., Coble, R.W. (1989). Hydrogeologic framework of the North	Approximated from Fig. 1 of Lyke and Coble (1987) and Fig. 14 of Winner Jr. and Coble (1989).

Aquifer system	Broader aquifer system	Reference A	Reference B	Reference C	Steps to delineate
		eastern North Carolina. U.S. Geological Survey Open-File Report 87-571, 2 pp. Accessed April 1, 2021 from https://pubs.usgs.gov/of/1987/0571/report.pdf	simulation of ground-water flow at U.S. Marine Corps Air Station, Cherry Point, North Carolina, 1987-90. Water-Resources Investigations Report 94-4186, 81 pp. Accessed April 1, 2021 from https://pubs.usgs.gov/wri/1994/4186/report.pdf	Carolina Coastal Plain aquifer system. U.S. Geological Survey Report 87-690, 167 pp. Accessed April 1, 2021 from https://pubs.usgs.gov/of/1987/0690/report.pdf	Eastern limit of Castle Hayne is extended farther than shown in Fig. 14 of Winner Jr and Coble (1989) as local reports near Cherry Point detail the importance of the Castle Hayne Aquifer -- see Eimers et al. (1994).
**Catahoula Area**	Gulf Coast Regional Aquifer System	Weiss, J. S. (1990). Geohydrologic units of the coastal lowlands aquifer system, south-central United States. U.S. Geological Survey Regional aquifer-systems analysis. 42 pp. Accessed March 30, 2021 from https://pubs.usgs.gov/pp/1416c/report.pdf	Halford, K. J., Barber, N. L. (1995). Analysis of ground-water flow in the Catahoula aquifer system in the vicinity of Laurel and Hattiesburg, Mississippi. U.S. Geological Survey Water-Resources Investigations Report 94-4219, 78 pp. Accessed March 31, 2021 from https://pubs.usgs.gov/wri/1994/4219/report.pdf	Kuniansky, E. L., Bellino, J. C., Dixon, J. (2012). Transmissivity of the upper Floridan aquifer in Florida and parts of Georgia, South Carolina, and Alabama. U.S. Geological Survey Scientific Investigations Map 3204, 1 sheet, scale 1:100,000, Accessed March 31, 2021 from https://pubs.usgs.gov/sim/3204/pdf/USGS_SIM-3204_Kuniansky_Web.pdf	Northern margin of Gulf Coast Regional Aquifer System approximated from Fig. 2 of Weiss (1990). Hydrostratigraphy for central portion of subarea detailed by Halford and Barber (1995). Eastern margin approximated along portions of the Chickasawhay River.
**Cave Valley**	-	Lopes, T.J., Evetts. D.M. (2005). Ground-Water pumpage and artificial recharge estimates for calendar year 2000 and average annual natural recharge and interbasin flow by hydrographic area, Nevada. Accessed April 25, 2022 via https://pubs.usgs.gov/sir/2004/5239/sir2004-5239.pdf	Harrill, J.R., Prudic, D.E. (1998). Aquifer systems in the Great Basin region of Nevada, Utah, and adjacent states; summary report. US Geological Survey Professional Paper 1409-A, 75 pp. Accessed April 26, 2022 via https://pubs.usgs.gov/pp/1409a/report.pdf	-	Approximated from Fig. 1 by Lopes and Evetts (2005), and Fig. 2 by Harrill and Prudic (1998). Well completion records, physiography and drainage were used to delinate areas.
**Cedar Valley**	-	Brooks, L. E., Mason, J. L. (2005). Hydrology and simulation of ground-water flow in Cedar Valley, Iron County, Utah (No. 2005-	-	-	Approximated from Fig. 1 of Brooks et al. (2005)

Aquifer system	Broader aquifer system	Reference A	Reference B	Reference C	Steps to delineate
		5170). US Geological Survey Scientific Investigations Report 2005-5170, 127 pp. Accessed March 7, 2021 from https://pubs.usgs.gov/sir/2005/5170/PDF/SIR2005_5170.pdf			
**Cedar Valley near Utah Lake**	-	Jordan, J. L. (2013). Aquifer Parameter Estimation from Aquifer Tests and Specific-capacity Data in Cedar Valley and the Cedar Pass Area, Utah County, Utah. Utah Geological Survey Special Study 146.	-	-	Approximated from Fig. 1 of Jordan (2013)
**Central Adelaide Plains**	St Vincent Basin	Smith, M. L., Fontaine, K., Lewis, S. J. (2015). Regional Hydrogeological Characterisation of the St Vincent Basin, South Australia: Technical Report for the National Collaboration Framework Regional Hydrogeology Project. Geoscience Australia Record 2015/16, 150 pp. Accessed July 27, 2021 from https://d28rz98at9flks.cloudfront.net/78884/Rec2015_016.pdf	Bresciani, E., Cranswick, R. H., Banks, E. W., Batlle-Aguilar, J., Cook, P. G., Batelaan, O. (2018). Using hydraulic head, chloride and electrical conductivity data to distinguish between mountain-front and mountain-block recharge to basin aquifers. Hydrology and Earth System Sciences, 22, 1629-1648.	-	Approximated from Fig. 2.2 by Smith et al. (2015), and Fig. 2 by Bresciani et al. (2018)
**Central Allegheny Plateau**	Appalachian Plateaus	Trapp Jr., H., Horn, M.A. (1997). Ground water atlas of the United States: Segment 11. U.S. Geological Survey Hydrologic Investigations Atlas 730-L, 26 pp. Accessed April 13, 2021	-	-	Broader Appalachian Plateau Aquifer System approximated from Fig. 3 of Trapp Jr. and Horn (1997)

Aquifer system	Broader aquifer system	Reference A	Reference B	Reference C	Steps to delineate
		from https://pubs.usgs.gov/ha/730l/report.pdf			
**Central Alluvial-Laucustrine Plain**	North China Plain	Meng, S., Liu, J., Zhang, Z., Lei, T., Qian, Y., Li, Y., Fei, Y. (2015). Spatiotemporal evolution characteristics study on the precipitation infiltration recharge over the past 50 years in the North China Plain. Journal of Earth Science, 26, 416-424.	Cao, G., Zheng, C., Scanlon, B. R., Liu, J., & Li, W. (2013). Use of flow modeling to assess sustainability of groundwater resources in the North China Plain. Water Resources Research, 49, 159-175.	Wang, S., Song, X., Wang, Q., Xiao, G., Liu, C., Liu, J. (2009). Shallow groundwater dynamics in North China plain. Journal of Geographical Sciences, 19, 175-188.	Approximated from Fig. 1 by Meng et al. (2015), Fig. 1 by Cao et al. (2013) and Fig. 6 by Wang et al. (2009).
**Central Amarkantak Plateau**	Amarkantak Plateau	Deolankar, S. B. (1980). The Deccan basalts of Maharashtra, India—their potential as aquifers. Groundwater, 18(5), 434-437.	Kale, V. S., Bodas, M., Chatterjee, P., Pande, K. (2020). Emplacement history and evolution of the Deccan Volcanic Province, India. Episodes, 43, 278-299.	Duraiswami, R. A., Das, S., Shaikh, T. (2012). Hydrogeological framework of aquifers from the Deccan Traps, India: some insights. Memoir Geological Society of India, 1-15.	Approximated from Fig. 1 by Deolankar (1980), Fig. 1 by Duraiswami et al. (2012), and Fig. 1 by Kale et al. (2016). Subarea based on topography and drainage patterns.
**Central Basin**	Los Angeles Basin**	Land, M., Reichard, E.G., Crawford, S.M., Everett, R.R., Newhouse, M.W., Williams, C.F. (2004). Ground-water Quality of Coastal Aquifer Systems in the West Coast Basin, Los Angeles County, California, 1999–2002. U.S. Geological Survey Scientific Investigations Report 2004-5067, 88 pp. Accessed February 15, 2021 from https://pubs.usgs.gov/sir/2004/5067/sir2004-5067.pdf	Fram, M. S., Belitz, K. (2008). Groundwater quality in the Coastal Los Angeles Basin, California; United States Geological Survey Fact Sheet 2012-3096. Accessed February 14, 2021 from https://pubs.er.usgs.gov/publication/70039952.	Reichard, E. G., Land, M., Crawford, S.M., Johnson, T.D., Everett, R.R., Kulshan, T.V., Ponti, D.J., Halford, K.L., Johnson, T.A., Paybins, K.S., Nishikawa, T. (2003). Geohydrology, Geochemistry, and Ground-Water Simulation Optimization of the Central and West Coast Basins, Los Angeles County, California. USGS Numbered Series, 2003–4065. Accessed November 28, 2021 from https://pubs.usgs.gov/wri/wrir034065/wrir034065.pdf	Approximated from Fig. 1 by Land et al. (2004), Fig. 6 by Reichard et al. (2003) and map on page 1 of Fram and Belitz (2008)
**Central Bohemian**	Bohemian	Alvarado, J. C., Pačes, T., Purtschert, R. (2013). Dating groundwater in the	Jiráková, H., Huneau, F., Hrkal, Z., Celle-Jeanton, H., Le Coustumer, P. (2010).	Jiráková, H., Procházka, M., Dědeček, P., Kobr, M., Hrkal, Z., Huneau, F., Le	Approximated from Fig. 1 by Jiráková et al. (2010), Fig. 1 by

Aquifer system	Broader aquifer system	Reference A	Reference B	Reference C	Steps to delineate
**Cretaceuos Basin**	Cretaceuos Basin	Bohemian Cretaceous Basin: Understanding tracer variations in the subsurface. Applied Geochemistry, 29, 189-198.	Carbon isotopes to constrain the origin and circulation pattern of groundwater in the north-western part of the Bohemian Cretaceous Basin (Czech Republic). Applied Geochemistry, 25, 1265-1279.	Coustumer, P. (2011). Geothermal assessment of the deep aquifers of the northwestern part of the Bohemian Cretaceous basin, Czech Republic. Geothermics, 40, 112-124.	Jiráková et al. (2011), and Fig. 1 by Alvarado et al. (2013)
**Central Caborca Aquifer**	Caborca Aquifer System	Cañez Araiza, D.A. (2018). Caracterización hidrogeoquímica y situación actual de la intrusión marina en la porción costera del acuífero Caborca, Sonora, México. Universidad de Sonora M.Sc. Thesis, 175 pp. Accessed May 6, 2021 from http://www.repositorioinstitucional.uson.mx/bitstream/handle/unison/1935/canezaraizadaynaangelicam.pdf?sequence=1	Vega-Granillo, E.L., Cirett-Galán, S, De la Parra-Velasco, M.L., Zavala-Juárez, R. (2011). Hidrogeología de Sonora, México. in: Calmus, Thierry, ed., Panorama de la geología de Sonora, México, Universidad Nacional Autónoma de México, Instituto de Geología, Boletín 118, 267–298. Accessed May 6, 2021 from	-	Approximated from Fig. 3 by Cañez Araiza (2018) and from Fig. 5 of Vega-Granillo et al. (2011).
**Central Cambay Basin**	Cambay Basin	Kumar, M. D., Ghosh, S., Patel, A., Singh, O. P., Ravindranath, R. (2006). Rainwater harvesting in India: some critical issues for basin planning and research. Land Use and Water Resources Research, 6, 1-17.	Deshpande, R.D. (2006). Groundwater in and Around Cambay Basin, Gujarat: Some Geochemical andIsotopic Investigations, Physical Research Laboratory, Ahmedabad, PhD Thesis. Accessed July 12, 2021 from https://shodhganga.inflibnet.ac.in/handle/10603/58203	Agarwal, M., Gupta, S. K., Deshpande, R. D., Yadava, M. G. (2006). Helium, radon and radiocarbon studies on a regional aquifer system of the North Gujarat–Cambay region, India. Chemical Geology, 228, 209-232.	Approximated from Fig. 6 by Kumar et al. (2006), Fig. 4.1 by Deshpande (2006), and Fig. 1 by Agarwal et al. (2006). West, Central and East subareas defined by two identified faults (see Fig. 4.1 by Deshpande (2006))
**Central Canterbury Plains**	-	Taylor, C. B., Wilson, D. D., Brown, L. J., Stewart, M. K., Burden, R. J., Brailsford, G. W. (1989). Sources and flow of north Canterbury	Bal, A. A. (1996). Valley fills and coastal cliffs buried beneath an alluvial plain: evidence from variation of permeabilities in gravel	Wilson, D. D. (1973). The significance of geology in some current water resource problems, Canterbury Plains, New	Approximated from Fig. 1 by Taylor et al. (1989), Figs. 1 and 2 by Bal (1996) and Fig. 1 by Wilson (1973). Physiography and drinage

Aquifer system	Broader aquifer system	Reference A	Reference B	Reference C	Steps to delineate
		plains groundwater, New Zealand. Journal of hydrology, 106, 311-340.	aquifers, Canterbury Plains, New Zealand. Journal of Hydrology (New Zealand), 1-27.	Zealand. Journal of Hydrology (New Zealand), 103-118.	features were applied to approximate subareas
**Central Cape Breton**	Maritimes Basin	Stapinsky, M. Michaud, Y., Morin, R.H., Butler, K.E., Deblonde, C., Chi, G, Thériault, T., Castonguay, S., Boisvert, V., Julien, H.P., Conohan, D., Hulsman, B., Carrier, C. Marion, J., Boisvert, É. (2002). Groundwater resources assessment in the Carboniferous Maritimes Basin: preliminary results of the hydrogeological characterization, New Brunswick, Nova Scotia, and Prince Edward Island. Geological Survey of Canada Current Research Report 2002-D8, 14 pp. Accessed May 5, 2021 from http://www.gov.pe.ca/photos/original/cle_WA10.pdf	Rivard, C., Michaud, Y., Lefebvre, R., Deblonde, C., Rivera, A. (2008). Characterization of a regional aquifer system in the Maritimes Basin, Eastern Canada. Water Resources Management, 22, 1649-1675.	Bianco, E. (2013). Seismic Interpretation of the Windsor-Kennetcook Basin. Geological Survey of Canada Open File 7452, 31 pp. Accessed May 5, 2021 from https://ftp.maps.canada.ca/pub/nrcan_rncan/publications/STPublications_PublicationsST/292/292763/of_7452.pdf	Approximated from Fig. 2 of Rivard et al. (2008) and Figs. 1 and 3 of Stapinsky et al. (2002). Area bounds approximated from Fig. 1 of Bianco (2013).
**Central Carrizo-Wilcox**	Carrizo-Wilcox	Huang, Y., Scanlon, B.R., Nicot, J.P., Reedy, R.C., Dutton, A.R., Kelley, V.A., Deeds, N E. (2012). Sources of groundwater pumpage in a layered aquifer system in the Upper Gulf Coastal Plain, USA. Hydrogeology Journal, 20(4), 783-796.	Kelley, V.A., Deeds, N.E., Fryar, D.G., Nicot, J.P. (2004). Groundwater availability models for the Queen City and Sparta aquifers. Contract report to the Texas Water Development Board, Austin, TX, 867 pp. Accessed February 24, 2022 from https://www.twdb.texas.gov/groundwater/models/gam/qcsp/QCSP_Model_Report.pdf?d=29484	-	Approximated from Fig. 2.2 by Kelley et al. (2004). Texas portion approximated from Fig. 1 of Huang et al. (2012). Eastern and western boundaries approximated by Texas' 12th Groundwater Management Area (GMA) (accessed February 11, 2021 from https://www.twdb.texas.gov/groundwater/management_areas/gma12.asp)

Aquifer system	Broader aquifer system	Reference A	Reference B	Reference C	Steps to delineate
**Central Chikugo-Saga Plain**	Chikugo-Saga Plain	Wang, L., Iwao, Y. (2000). Groundwater characteristics of the Saga Plain, Japan. Journal of Nepal Geological Society, 22, 343-350.	Sakai, A. (2001). Land subsidence due to seasonal pumping of groundwater in Saga Plain, Japan. Lowland Technology International, 3(1, June), 25-40. Accessed April 13, 2023 via https://cot.unhas.ac.id/journals/index.php/ialt_lti/article/view/287	Cai, Y., Esaki, T., Liu, S., Mitani, Y. (2014). Effect of substitute water projects on tempo-spatial distribution of groundwater withdrawals in Chikugo-Saga plain, Japan. Water Resources Management, 28, 4645-4663.	Approximated from Fig. 1 by Wang and Iwao (2000), Fig. 1 by Sakai (2001), and Fig. 1 by Cai et al. (2014)
**Central Coastal Deccan Traps**	-	Ansari, M.A., Noble, J., Deodhar, A., Kumar, U.S. (2022). Isotope hydrogeochemical models for assessing the hydrological processes in a part of the largest continental flood basalts province of India. Geoscience Frontiers, 13, 101336.	-	-	Approximated from Fig. 1 by Ansari et al. (2022)
**Central Colusa Basin**	Sacramento Valley of the Central Valley **	California Department of Water Resources (2015i). California's groundwater update 2013 - Chapter 7: Sacramento River Hydrologic Region. California Department of Water Resources Report, 126 pp. Accessed April 27, 2022 via https://water.ca.gov/-/media/DWR-Website/Web-Pages/Programs/Groundwater-Management/Bulletin-118/Files/Statewide-Reports/GWU2013_Ch7_SacramentoRiver_Final.pdf	-	-	Approximated from Fig. 7-2 by California Department of Water Resources (2015i). Divided from Northern Colusa Basin on the basis of GCID canal and divided from Southern Colusa Basin along Salt Creek and south of the city of Colusa
**Central Coromandel Fractured**	Coromandel	Central Ground Water Board (2012b). Aquifer Systems of Tamilnadu and	-	-	Approximated from Plate III by Central Ground Water Board (2012b)

Aquifer system	Broader aquifer system	Reference A	Reference B	Reference C	Steps to delineate
**Charnockite and Gneiss Aquifers**	Coastal Plain	Puducherry. Central Ground Water Board Report, 78 pp. Accessed June 23, 2021 from http://cgwb.gov.in/AQM/TamilNadu.pdf			
**Central Culverden Basin**	Culverden Basin	Poulsen, D. (2012). Culverden Basin hydrogeology. Environment Canterbury Regional Council Report No. R12/96, 104 pp. Accessed October 20, 2021 from https://www.ecan.govt.nz/document/download?uri=1723844	-	-	Approximated from Fig. 2-1 by Environment Canterbury Regional Council (2012). Subareas based on drainage and physiography.
**Central Cumberland Plateau and Sequatchie Valley**	Appalachian Plateaus	Brahana, J.V., Macy, J.A., Mulderink, D., Zemo, D. (1986). Preliminary delineation and description of the regional aquifers of Tennessee-Cumberland plateau aquifer system. U.S. Geological Survey, Water-Resources Investigations Report 82-338, 29 pp. Accessed April 13, 2021 from https://pubs.usgs.gov/wri/wrir82-338/pdf/wrir_82-338_a.pdf	-	-	Approximated from Fig. 2 by Brahana et al. (1986)
**Central Dezful-Andimeshk Plain**	Dezful-Andimeshk Plain	Hosseini, S. M., Parizi, E., Ataie-Ashtiani, B., Simmons, C. T. (2019). Assessment of sustainable groundwater resources management using integrated environmental index: Case studies across Iran. Science of the Total	-	-	Approximated from Fig. 6 by Hosseini et al. (2019)

Aquifer system	Broader aquifer system	Reference A	Reference B	Reference C	Steps to delineate
		Environment, 676, 792-810.			
**Central Dharwar Craton Banded Gneiss Complex**	Dharwar Craton Banded Gneiss Complex	Lancaster, P. J., Dey, S., Storey, C. D., Mitra, A., Bhunia, R. K. (2015). Contrasting crustal evolution processes in the Dharwar craton: insights from detrital zircon U–Pb and Hf isotopes. Gondwana Research, 28, 1361-1372.	Central Ground Water Board (2012c). Aquifer Systems of Karnataka. Central Ground Water Board Report, 75 pp. Accessed June 30, 2021 from http://cgwb.gov.in/AQM/Karnataka.pdf	-	Dharwar Craton geology approximated from Fig. 1 by Lancaster et al. (2015); hydrogeologic boundaries for Karnataka approximated from Plate III by Central Ground Water Board (2012c)
**Central Eromanga Depocenter**	Great Artesian Basin	Ransley, T.R., Smerdon, B.D. (2012). Hydrostratigraphy, hydrogeology and system conceptualisation of the Great Artesian Basin. A technical report to the Australian Government from the CSIRO Great Artesian Basin Water Resource Assessment, 324 pp. Accessed July 20, 2021 from https://publications.csiro.au/rpr/download?pid=csiro:EP132693&dsid=DS5	Ransley, T.R., Radke, B.M., Feitz, A.J., Kellett, J.R., Owens, R., Bell, J. and Stewart, G. (2015). Hydrogeological Atlas of the Great Artesian Basin, Geoscience Australia. Canberra, 141 pp. Accessed July 19, 2021 from http://www.ga.gov.au/scientific-topics/water/groundwater/gab	Smerdon, B.D., Ramsley, T.R., Radke, B.M., Kellett, J.R. (2012). Water Resource Assessment for the Great Artesian Basin. A technical report to the Australian Government from the CSIRO Great Artesian Basin Water Resource Assessment, 56 pp. Accessed July 20, 2021 from https://publications.csiro.au/rpr/download?pid=csiro:EP132685&dsid=DS3	Approximated from Fig. 2.3 by Ransley and Smerdon (2012), Fig. 1.4 by Smerdon et al. (2012), and Map 4 by Ransley et al. (2015)
**Central Eucla Basin**	Eucla Basin	Berens, V., Alcoe, D., Watt, E. (2011). Non-prescribed groundwater resources assessment — Eyre Peninsula natural resources management region. Science, Monitoring and Information Division Department for Water, 110 pp. Accessed October 6, 2021 from https://www.waterconnect.sa.gov.au/Content/Publications/DEW/EP_NRM_Non-	-	-	Approximated from Fig. 4 by Berens et al. (2011)

Aquifer system	Broader aquifer system	Reference A	Reference B	Reference C	Steps to delineate
		prescribed_GW_Assessment_2011.pdf			
**Central Guadalquivir Basin**	Guadalquivir Basin	Dumont, A., Salmoral, G., Llamas, M. R. (2013). The water footprint of a river basin with a special focus on groundwater: The case of Guadalquivir basin (Spain). Water Resources and Industry, 1, 60-76.	-	-	Broader basin boundaries approximated from Fig. 1 by Dumont et al. (2013)
**Central High Plains****	High Plains	Gutentag, E.D., Heimes, F.J., Krothe, N.C., Luckey, R.R., Weeks, J.B. (1984). Geohydrology of the High Plains aquifer in parts of Colorado, Kansas, Nebraska, New Mexico, Oklahoma, South Dakota, Texas, and Wyoming (No. 1400-B). Accessed February 10, 2021 from https://pubs.usgs.gov/pp/1400b/report.pdf	Luckey, R.L., Becker, M.F. (2003). Hydrogeology, water use, and simulation of flow in the High Plains aquifer in northwestern Oklahoma, southeastern Colorado, southwestern Kansas, northeastern New Mexico, and northwestern Texas. Water-Resources Investigations Report 99-4104, 73 pp. Accessed February 15, 2022 from https://pubs.usgs.gov/wri/wri994104/pdf/wri994104.pdf	McGuire, V.L., Johnson, M.R., Schieffer, J.S., Stanton, J.S., Sebree, S.K., Varstraeten (2003). Water in storage and approaches to groundwater management, High Plains Aquifer, 2000. US Geological Survey Circular 1243, 56 pp. Accessed February 15, 2022 from https://pubs.usgs.gov/circ/2003/circ1243/pdf/C1243.pdf	Approximated from Fig. 1 of Gutentag et al. (1984), Fig. 18 by McGuire et al. (2003), and Fig. 1 by Luckey and Becker (2003). Central subarea distinguished from North-central and South-central High Plains on the basis of the Arkansas and Canadian Rivers
**Central Huaihe Plain**	Huaihe River Basin	Sun, X., Lin, J., Gu, W., Min, X., Han, J., Dai, Y., Liu, P., Wang, S (2021). Analysis and evaluation of the renewability of the deep groundwater in the Huaihe River Basin, China. Environmental Earth Sciences, 80, 1-13.	Liu J., Zheng C. (2016). Towards Integrated Groundwater Management in China. In: Integrated Groundwater Management (eds. Jakeman A.J., Barreteau O., Hunt R.J., Rinaudo J.D., Ross A.). Springer, Cham. pp. 455-475	Li, Y., Wang, D., Liu, Y., Zheng, Q., Sun, G. (2017). A predictive risk model of groundwater arsenic contamination in China applied to the Huai River Basin, with a focus on the region's cluster of elevated cancer mortalities. Applied Geochemistry, 77, 178-183.	Approximated from Fig. 1 by Sun et al. (2021), Fig. 1 by Li et al. (2017), and Fig. 18.5 by Liu and Zheng (2016)
**Central Kabul Basin**	Kabul Basin	Zaryab, A., Nassery, H. R., Alijani, F. (2021). Identifying sources of groundwater salinity and	Sadid, N. (2020). Surface-groundwater interaction in the Kabul region basin. Afghanistan Research and	Saffi, M.H. (2019). National alarming on groundwater natural storage depletion and water quality	Approximated from Fig. 1 by Zaryab et al. (2021), Fig. 12 by Saffi (2019), and Fig. 3.2 by Sadid (2020). Southern boundary

Aquifer system	Broader aquifer system	Reference A	Reference B	Reference C	Steps to delineate
		major hydrogeochemical processes in the Lower Kabul Basin aquifer, Afghanistan. Environmental Science: Processes & Impacts, 23, 1589-1599.	Evaluation Unit Report, 58 pp. Accessed January 27, 2022 from https://reliefweb.int/sites/reliefweb.int/files/resources/2005-E-Surface-groundwater-interaction-in-the-Kabul-region-basin.pdf	deterioration of Kabul City and immediate response to the drinking water crises. Scientific INvestigation Report in Afghanistan, DACAAR Report, 88 pp.	defined along the Lower Kabul River
**Central Kachchh Coastal Plain**	Kachchh Coastal Plain	Saha, D., Gor, N. (2020). A prolific aquifer system is in peril in arid Kachchh region of India. Groundwater for Sustainable Development, 11, 100394.	Bhimani, S.A. (2013). Study on Groundwater Salinization and Formulation of Management Strategies for the Coastal Aquifers of Mundra Region, Kutch District, Gujarat State. Ph.D. Thesis, The Maharaja Sayajirao University of Baroda. 380 pp. Accessed July 5, 2021 from http://okgw.hmlibrary.ac.in:8080/jspui/handle/1/457	University of Greenwich and Gujarat Institute of Desert Ecology (2016). Ecosystem Assessment of the Coastal Plain Natural Area of Kachchh District: Planning for Biodiversity and Livelihoods into the Future. Project Presentation, 70 pp. Accessed July 5, 2021 from https://gala.gre.ac.uk/id/eprint/16221/1/16221%20BARTLETT_Coastal_Plain_of_Kachchh_2016.pdf	Approximated from Fig. 1 by Saha and Gor (2020) and Fig. 3.2 and descriptions of hydrogeologic setting in Chapter III by Bhimani (2013). Extent of broader aquifer system (region) approximated following the "Natural Character Area map" (page 4) by University of Greenwich and Gujarat Institute of Desert Ecology (2016)
**Central Karoo Basin**	Main Karoo Basin	Nyambe, I. A. (1999). Tectonic and climatic controls on sedimentation during deposition of the Sinakumbe Group and Karoo Supergroup, in the mid-Zambezi Valley Basin, southern Zambia. Journal of African Earth Sciences, 28, 443-463.	-	-	Broader aquifer system boundary (i.e., Main Karoo Basin) approximated from Fig. 1 by Nyambe (1999). Physiography (drainage patterns, geology) and well construction data were considered while digitizing subarea
**Central Kerala Coastal Aquifers**	Malabar Coastal Plain	Priju, C., Sushanth, C. M., Balan, V. (2021). Delineation of freshwater zones in the shallow coastal aquifers of Ernakulam-Chettuva region, Central Kerala, India using electrical resistivity methods. Accessed June	Central Ground Water Board (2012a). Aquifer Systems of Kerala. Central Ground Water Board Report, 72 pp. Accessed June 23, 2021 from http://cgwb.gov.in/AQM/Kerala.pdf	-	Approximated from Fig. 2 by Priju et al. (2021) and Plate III by Central Ground Water Board (2012a); northern limit extended to southern margin defined for the Kozhikkode Coastal Aquifer

Aquifer system	Broader aquifer system	Reference A	Reference B	Reference C	Steps to delineate
		23, 2021 from https://assets.researchsquare.com/files/rs-369371/v1/7e2408f9-0dd0-49c2-ab11-1a4457d52b58.pdf			
**Central Lake Area**	Floridan Aquifer System	Williams, L.J., Kuniansky, E.L. (2016). Revised hydrogeologic framework of the Floridan aquifer system in Florida and parts of Georgia, Alabama, and South Carolina. U.S. Geological Survey Professional Paper 1807, 140 pp. Accessed March 31, 2021 from https://pubs.usgs.gov/pp/1807/pdf/pp1807.pdf	Brooks, H. K. (1981). Physiographic divisions of Florida. Report for the Florida Cooperative Extension Service Institute of Food and Agricultural Sciences, University of Florida, 12 pp.	-	Approximated from physiographic provinces of Florida outlined by Brooks (1981). Broader Florida Aquifer System from Williams and Kuniansky (2016).
**Central Littoral Plain**	North China Plain	Meng, S., Liu, J., Zhang, Z., Lei, T., Qian, Y., Li, Y., Fei, Y. (2015). Spatiotemporal evolution characteristics study on the precipitation infiltration recharge over the past 50 years in the North China Plain. Journal of Earth Science, 26, 416-424.	Cao, G., Zheng, C., Scanlon, B. R., Liu, J., & Li, W. (2013). Use of flow modeling to assess sustainability of groundwater resources in the North China Plain. Water Resources Research, 49, 159-175.	Wang, S., Song, X., Wang, Q., Xiao, G., Liu, C., Liu, J. (2009). Shallow groundwater dynamics in North China plain. Journal of Geographical Sciences, 19, 175-188.	Approximated from Fig. 1 by Meng et al. (2015), Fig. 1 by Cao et al. (2013) and Fig. 6 by Wang et al. (2009). Northeastern boundary approximated near Sanhe Island.
**Central Malabar Khondalite and Charnockite Aquifers**	Malabar Coastal Plain	Central Ground Water Board (2012a). Aquifer Systems of Kerala. Central Ground Water Board Report, 72 pp. Accessed June 23, 2021 from http://cgwb.gov.in/AQM/Kerala.pdf	Varma, A. (2017). Groundwater Resource and Governance in Kerala, Status, Issues and Prospects. Forum for Policy Dialogue on Water Conflicts in India, Pune, Maharashtra, India. Kerala Resource Centre Report, 118 pp. Accessed June 23, 2021 from https://www.soppecom.org	-	Approximated from Plate III by Central Ground Water Board (2012a). Easternmost margin of Malabar Coastal Plain approximated along the ~76m topographic contour as detailed for the "lowland" and "midland" physiographic zones in Fig. 1.2 by Varma (2017)

Aquifer system	Broader aquifer system	Reference A	Reference B	Reference C	Steps to delineate
			/pdf/Groundwater-Resource-and-Governance-in-Kerala.pdf		
**Central Mallee**	Murray Basin	Herczeg, A. L., Dogramaci, S. S., Leaney, F. W. J. (2001). Origin of dissolved salts in a large, semi-arid groundwater system: Murray Basin, Australia. Marine and Freshwater Research, 52, 41-52.	Cook, P. G., Jolly, I. D., Leaney, F. W. J. (2001). Groundwater recharge in the Mallee region, and salinity implications for the Murray River: A review. CSIRO Land and Water Report, 133 pp. Accessed July 21, 2021 from https://publications.csiro.au/rpr/download?pid=procite:ef08494d-43a2-4dae-bda4-3d72a62e673f&dsid=DS1	Murray-Darling Basin Commission (1999). Murray-Darling Basin Groundwater - a resource for the future, Murray-Darling Basin Commission, Canberra. Accessed July 21, 2021 from https://www.mdba.gov.au/sites/default/files/archived/mdbc-GW-reports/2173_GW_a_resource_for_the_future.pdf	Approximated from Fig. 1 by Herczeg et al. (2001), Fig. 1.1 by Cook et al. (2001), and Fig. 6 by Murray-Darling Basin Commission (1999)
**Central Maputaland Coastal Plain**	Maputaland Coastal Plain	Mthembu, P. P., Elumalai, V., Brindha, K., Li, P. (2020). Hydrogeochemical processes and trace metal contamination in groundwater: impact on human health in the Maputaland coastal aquifer, South Africa. Exposure and Health, 12(3), 403-426.	-	-	Approximated from Fig. 1 by Mthembu et al. (2020)
**Central Mashad Plain**	Mashad-Narimani Basin	Khashei-Siuki, A., Sarbazi, M. (2015). Evaluation of ANFIS, ANN, and geostatistical models to spatial distribution of groundwater quality (case study: Mashhad plain in Iran). Arabian Journal of Geosciences, 8, 903-912.	Naghibi, S. A., Vafakhah, M., Hashemi, H., Pradhan, B., & Alavi, S. J. (2018). Groundwater augmentation through the site selection of floodwater spreading using a data mining approach (case study: Mashhad Plain, Iran). Water, 10, 1405.	Hosseini, S. M., Parizi, E., Ataie-Ashtiani, B., Simmons, C. T. (2019). Assessment of sustainable groundwater resources management using integrated environmental index: Case studies across Iran. Science of the Total Environment, 676, 792-810.	Approximated from Fig. 2 by Khashei-Siuki and Sarbazi (2015), Fig. 2 by Naghibi et al. (2018), and Fig. 6 by Hosseini et al. (2019)
**Central Minnesota Surficial and**	-	Minnesota Department of Natural Resources (2021). Minnesota Groundwater	-	-	Approximated from map by the Minnesota Department of Natural Resources (2021)

Aquifer system	Broader aquifer system	Reference A	Reference B	Reference C	Steps to delineate
**Buried Sand and Gravel Aquifers**		Provinces 2021. Minnesota Department of Natural Resources map, 2 pp. Accessed April 14, 2021 from https://files.dnr.state.mn.us/waters/groundwater_section/provinces/2021-provinces.pdf			
**Central Mississippi Embayment**	Mississippi Embayment	Arthur, J.K., Taylor, R.E. (1998). Ground-water flow analysis of the Mississippi embayment aquifer system, South-Central United States. US Geological Survey Professional Paper 1416-1, 59 pp. Accessed February 10, 2021 from https://pubs.usgs.gov/pp/1416i/report.pdf	Renken, R.A. (1998). Groundwater Atlas of the United States: Arkansas, Louisiana, Mississippi (HA-730-F). US Geological Survey Hydrologic Investigations Atlas 730-F. 30 pp. Accessed November 28, 2021 fromhttps://pubs.usgs.gov/ha/730f/report.pdf	-	Approximated from Fig. 2 of Arthur and Taylor (1998) and Figs. 8-10 and Fig. 65 by Renken (1998)
**Central Nallamalai Basin**	Cuddapah Sedimentary Basin	Dar, F. A., Perrin, J., Riotte, J., Gebauer, H. D., Narayana, A. C., Shakeel, A. (2011). Karstification in the Cuddapah Sedimentary Basin, southern India: implications for groundwater resources. Acta Carsologica, 40, 457-472.	Saha, D., Ray, R. K. (2019). Groundwater resources of India: potential, challenges and management. In Groundwater Development and Management. Springer, Cham (ed. Sikdar, P.K.), pp. 19-42.	Chalapathi Rao, N. V., Gibson, S. A., Pyle, D. M., Dickin, A. P. (2004). Petrogenesis of Proterozoic lamproites and kimberlites from the Cuddapah Basin and Dharwar craton, southern India. Journal of Petrology, 45, 907-948.	Approximated from Fig. 1 by Dar et al. (2011), Fig. 2.2 by Saha and Ray (2019), and Fig. 1 by Chalapathi Rao et al. (2004)
**Central Otway Basin**	Otway Basin	Barnett, S., Simmons, C. T., Nelson, R. (2021). Groundwater resources in Australia—their occurrence, management, and future challenges. In: Global Groundwater Source, Scarcity, Sustainability, Security, and Solutions (eds. Mukherjee, A., Scanlon, B.R., Aureli, A.,	-	-	Approximated from Fig. 1 by Barnett et al. (2021). Divide between Central and Eastern Otway Basins approximated along the Hopkins River and Salt Creek

Aquifer system	Broader aquifer system	Reference A	Reference B	Reference C	Steps to delineate
		Langan, S., Guo, H., McKenzie, A.), Elsevier, pp. 35-46.			
**Central Parana Basin and Torres Syncline**	Guarani Aquifer System	Hirata, R., Foster, S. (2021). The Guarani Aquifer System–from regional reserves to local use. Quarterly Journal of Engineering Geology and Hydrogeology, 54, qjegh2020-091.	Gonçalves, R. D., Teramoto, E. H., Chang, H. K. (2020). Regional Groundwater Modeling of the Guarani Aquifer System. Water, 12, 2323.	Rodríguez, L., Vives, L., Gomez, A. (2013). Conceptual and numerical modeling approach of the Guarani Aquifer System. Hydrology and Earth System Sciences, 17, 295-314.	Approximated from Fig. 2 by Gonçalves et al. (2020) and Fig. 1 by Hirata and Foster (2021), who describe the three regions of the Guarani Aquifer System as delimited by two major arches: (i) the Ponta Grossa Arch (quote Hirata and Foster (2021): "which forces groundwater to flow from east to west in São Paulo State"), and (ii) the Asunción–Rio Grande (quote Hirata and Foster (2021): "..which divides the portion south of the Ponta Grossa Arch into two semi-independent sedimentary basins"). The locations of these aforementioned arches were approximated from Fig. 2 by Rodríguez et al. (2013). Subarea name derives from discussion in section 2 of publication by Rodríguez et al. (2013).
**Central Paris Basin**	Paris Basin	Beaudoin, N., Gasparrini, M., David, M. E., Lacombe, O., Koehn, D. (2019). Bedding-parallel stylolites as a tool to unravel maximum burial depth in sedimentary basins: Application to Middle Jurassic carbonate reservoirs in the Paris basin, France. GSA Bulletin, 131, 1239-1254.	Contoux, C., Violette, S., Vivona, R., Goblet, P., Patriarche, D. (2013). How basin model results enable the study of multi-layer aquifer response to pumping: the Paris Basin, France. Hydrogeology Journal, 21, 545-557.	Beccaletto, L., Hanot, F., Serrano, O., Marc, S. (2011). Overview of the subsurface structural pattern of the Paris Basin (France): Insights from the reprocessing and interpretation of regional seismic lines. Marine and Petroleum Geology, 28, 861-879.	Approximated from Fig. 1 by Beaudoin et al. (2019), Fig. 1 by Contoux et al. (2013), and Fig. 1 by Beccaletto et al. (2011)
**Central Perth Basin**	Perth Basin	Ali, R., McFarlane, D., Varma, S., Dawes, W., Emelyanova, I., Hodgson, G., Charles, S. (2012).	Timms, N. E., Olierook, H. K., Wilson, M. E., Delle Piane, C., Hamilton, P. J., Cope, P., Stütenbecker, L.	-	Approximated from Fig. 1b by Ali et al. (2012) and Fig. 1 by Timms et al. (2015)

Aquifer system	Broader aquifer system	Reference A	Reference B	Reference C	Steps to delineate
		Potential climate change impacts on groundwater resources of south-western Australia. Journal of Hydrology, 475, 456-472.	(2015). Sedimentary facies analysis, mineralogy and diagenesis of the Mesozoic aquifers of the central Perth Basin, Western Australia. Marine and Petroleum Geology, 60, 54-78.		
**Central Piedmont Upland**	Piedmont Plateau	Miller, J.A. (1990). Ground Water Atlas of the United States: Segment 6, Alabama, Florida, Georgia, South Carolina. U.S. Geological Survey Hydrologic Atlas 730-G, 30 pp. Accessed April 5, 2021 from https://www.nrc.gov/docs/ML1706/ML17060B027.pdf	-	-	Approximated from Fig. 3 of Miller (1990). Northeastern margin of area approximated along the Hardware and James Rivers.
**Central Pilbara Granite-Greenstone Terrane**	Pilbara Craton	Rojas, R., Commander, P., McFarlane, D., Ali, R., Dawes, W., Barron, O., Hodgson, G., Charles, S.(2018). Groundwater resource assessment and conceptualization in the Pilbara Region, Western Australia. Earth Systems and Environment, 2(2), 345-365.	-	-	Approximated from Fig. 3 by Rojas et al. (2018). Subareas delineated on the basis of the Shaw and Yule Rivers.
**Central Plain**	Central Snake River Plain	Graham, W.G., Campbell, L.J. (1981). Groundwater resources of Idaho. Idaho Department of Water Resources Report, 61 pp. Accessed March 23, 2021 from https://idwr.idaho.gov/files/publications/198108-MISC-GW-Resources-ID.pdf	Lindholm, G. F. (1996). Summary of the Snake River Plain regional aquifer-system analysis in Idaho and eastern Oregon. U.S Geological Survey Professional Paper 1408-A. 59 pp. Accessed March 1, 2021 via https://pubs.usgs.gov/pp/1408a/report.pdf	-	Approximated from Plate 1 of Graham and Campbell (1981). Broader portions of the Snake River Plain (i.e., Central, Western and Eastern) were approximated from Fig. 1 of Lindholm (1996); "Eastern" portion as defined by Lindholm (1996) was split into two portions (entitled "Central" and "Eastern" Snake River Plain, respectively) at Craters of the Moon National Monument.

Aquifer system	Broader aquifer system	Reference A	Reference B	Reference C	Steps to delineate
**Central Prince Edward Island**	Prince Edward Island	Stapinsky, M. Michaud, Y., Morin, R.H., Butler, K.E., Deblonde, C., Chi, G, Thériault, T., Castonguay, S., Boisvert, V., Julien, H.P., Conohan, D., Hulsman, B., Carrier, C. Marion, J., Boisvert, É. (2002). Groundwater resources assessment in the Carboniferous Maritimes Basin: preliminary results of the hydrogeological characterization, New Brunswick, Nova Scotia, and Prince Edward Island. Geological Survey of Canada Current Research Report 2002-D8, 14 pp. Accessed May 5, 2021 from http://www.gov.pe.ca/photos/original/cle_WA10.pdf	-	-	Approximated from Figs. 1 and 3 of Stapinsky et al. (2002).
**Central Razan Plain**	Razan Basin	Pazand, K. (2016). Geochemical and statistical evaluation of groundwater in Razan basin, Western Iran. Carbonates and Evaporites, 31, 179-185.	-	-	Approximated from Fig. 1 by Pazand (2016)
**Central Santiago Basin**	Santiago Basin	Araneda, M., Avendaño, M. S., Del Río, G. D. (2010). Modelo estructural de la cuenca de Santiago, Chile y su relación con la hidrogeología. Revista Geofísica, 62, 29-48.	Steinbrügge, G., Muñoz Pardo, J. F., Fernández, B. (2005). Análisis probabilístico y optimización de los recursos de agua subterránea: el caso del acuífero Maipo-Mapocho, Chile. Ingenieria hidraulica en Mexico, XX, 85-97. Accessed May 15, 2021 from	-	Approximated from Figs. 2 and 7 by Araneda et al. (2010). Southern boundary of subarea approximated along the Rio Maipo and northwestern boundary approximated along the Rio Mapocho. See also Illustration 3 by Steinbrügge et al. (2005).

Aquifer system	Broader aquifer system	Reference A	Reference B	Reference C	Steps to delineate
			https://repositorio.uc.cl/xmlui/bitstream/handle/11534/28539/An%C3%A1lisis%20probabil%C3%ADstico%20y%20optimizaci%C3%B3n%20de%20los%20recursos%20de%20agua%20subterr%C3%A1nea%20el%20caso%20del%20acu%C3%ADfero%20Maipo-Mapocho,%20Chile.pdf?sequence=1		
**Central Sevier Valley**	-	Young, R. A., Carpenter, C. H. (1965). Ground-water conditions and storage in the Central Sevier Valley, Utah. US Geological Survey Water Supply Paper 1787. 106 pp. Accessed March 7, 2021 from https://pubs.usgs.gov/wsp/1787/report.pdf	-	-	Approximated from Plate 1 of Young and Carpenter (1965)
**Central Souss Basin**	Souss-Chtouka Basin	Garduño, H., Foster, S. (2010). Sustainable Groundwater Irrigation. Approaches to Reconciling Demand with Resources. GW•MATE Strategic Overview Series No. 4 World Bank (Washington DC, USA). Accessed 05/01/2023 via https://openknowledge.worldbank.org/server/api/core/bitstreams/a6957092-3680-52cd-9707-91143c386175/content	Tagma, T., Hsissou, Y., Bouchaou, L., Bouragba, L., Boutaleb, S. (2009). Groundwater nitrate pollution in Souss-Massa basin (south-west Morocco). African Journal of Environmental Science and Technology, 3, 301-309.	Bouchaou, L., Michelot, J.L., Vengosh, A., Hsissou, Y., Qurtobi, M., Gaye, C.B., Bullen, T.D., Zuppi, G. M. (2008). Application of multiple isotopic and geochemical tracers for investigation of recharge, salinization, and residence time of water in the Souss–Massa aquifer, southwest of Morocco. Journal of Hydrology, 352, 267-287.	Broader aquifer system boundaries approximated from Box 5 by Garduño and Foster (2010), Fig. 1 by Bouchaou et al. (2008), and Fig. 1 by Tagma et al. (2009). Southern limit approximated along the Massa River.
**Central Urucuia Aquifer**	Urucuia Aquifer System	Feitosa, F. A., Diniz, J. A. O., Kirchheim, R. E., Kiang, C. H., Feitosa, E. C. (2016). Assessment of Groundwater	Serviço Geológico do Brasil (2019). Aquífero Urucuia Caracterização hidrológica com base em dados	Marques, E.A., Junior, G.C.S., Eger, G.Z., Ilambwetsi, A.M., Raphael, P., Generoso, T.N., Oliveira,	Approximated from Fig. 3.1 by Feitosa et al. (2016; see also description of basin and Urucuia Aquifer in section 3.5.3.9), Fig. 1

Aquifer system	Broader aquifer system	Reference A	Reference B	Reference C	Steps to delineate
		Resources in Brazil: Current Status of Knowledge. in: Groundwater Assessment, Modeling, and Management (eds. Thangarajan, M., Singh, V.P.), pp. 33-57.	secundários. Inistério de Minas e Energia Secretaria de Geologia, Mineração e Transformação Mineral Serviço Geológico do Brasil (CPRM) Report, 419 pp. Accessed May 18, 2021 from https://rigeo.cprm.gov.br/jspui/handle/doc/20922	J., Júnior, J.N. (2020). Analysis of groundwater and river stage fluctuations and their relationship with water use and climate variation effects on Alto Grande watershed, Northeastern Brazil. Journal of South American Earth Sciences, 103, 102723.	by Serviço Geológico do Brasil (2019) and Fig. 1 by Marques et al. (2019).
**Central Vienna Basin**	Vienna Basin	Kováč, M. I. C. H. A. L., Sliva, L., Sopkova, B., Hlavata, J., & Škulová, A. D. R. I. A. N. A. (2008). Serravallian sequence stratigraphy of the northern Vienna Basin: high frequency cycles in the Sarmatian sedimentary record. Geologica Carpathica, 59, 545-561.	-	-	Approximated from Fig. 1 by Kováč et al. (2008)
**Central Wabash and Bloomington Ridged Plain**	Central Lowland Till Plain	Stephenson, D.A. (1967). Hydrogeology of glacial deposits of the Mahomet Bedrock Valley in east-central Illinois. Illinois State Geological Survey Circular 409, 56 pp. Accessed November 29, 2021 from https://www.ideals.illinois.edu/bitstream/handle/2142/35125/hydrogeologyofgl409step.pdf?sequence=2	Leighton, M. M., Ekblaw, G. E., Horberg, L. (1948). Physiographic divisions of Illinois. The Journal of Geology, 56, 16-33.	Gray, H.H. (2001). Map of Indiana showing physiographic divisions. Indiana Geological Survey Miscellaneous Map 69, 15 pp. Accessed April 12, 2021 from https://igws.indiana.edu/ReferenceDocs/Maps/PhysiographicRegions.pdf	Bloomington Ridged Plain approximated from Figs. 1 and 2 of Leighton et al. (1948) and Fig. 1 by Stephenson (1967); "Central Wabash Valley" delineated by Gray (2001) was included for the Indiana portion of the subarea
**Central Willamette Valley**	Willamette Valley	Woodward, D.G., Gannett, M.W., Vaccaro, J.J. (1998). Hydrogeologic framework of the Willamette Lowland aquifer system, Oregon and Washington. US Geological Survey Professional Paper 1424-B, 92 pp. Accessed	Herrera, N.B., Burns, E.R., Conlon, T.D. (2014). Simulation of groundwater flow and the interaction of groundwater and surface water in the Willamette Basin and Central Willamette Subbasin,	-	Approximated from Fig. 2 of Woodward et al. (1998) and Fig. 1 by Herrera et al. (2014). Basin extent was broadened to include foothills of surrounding mountains, as a considerable number of wells exist in these areas. The Waldo and Salem Hills were used to

Aquifer system	Broader aquifer system	Reference A	Reference B	Reference C	Steps to delineate
		February 16, 2021 from https://pubs.usgs.gov/pp/1424b/report.pdf	Oregon. US Geological Survey Scientific Investigations Report 2014–5136, 174 pp. Accessed February 16, 2021 from https://pubs.usgs.gov/sir/2014/5136/pdf/sir20145136.pdf		divide this Central Willamette Valley from the Southern Willamette Valley to the south. The Chehalem Mountains were used as an approximate divide between this central portion of the aquifer system and the Tualatin Basin to the north.
**Chacabuco-Polpaico Aquifer**	-	Nuñez Codoseo, J. (2017). Evaluación de la disponibilidad de agua del sector acuífero Chacabuco-Polpaico: factibilidad de entrega de nuevos derechos de aprovechamiento de agua provisionales. Universidad de Chile Thesis, 138 pp. Accessed May 15, 2021 from http://repositorio.uchile.cl/handle/2250/144210	-	-	Approximated from Fig. 6 by Nuñez Codoseo (2017)
**Chachaua Aquifer**	-	CONAGUA (2021a). Acuíferos (nacional). Webpage accessed March 8, 2022 from http://sina.conagua.gob.mx/sina/tema.php?tema=acuiferos&ver=mapa&o=1&n=nacional	-	-	Approximated from aquifer boundaries defined by CONAGUA (2021a) web portal, physiography and drainage patterns, and well completion data.
**Chadegan Plain**	-	Ghafari, S., Moradi, H., Modares, R. (2018). Comparison of temporal and spatial changes of groundwater level in Isfahan-Borkhar, Najafabad and Chadegan Plains. Physical Geography Research Quarterly, 50, 141-160.	Yonesi, H., Arshia, A., Torabipoudeh, H., Shahinejad, B., Sayedipour, M., Vahdatpour, N. (2020). Evaluating Groundwater Quality in Zayandehrood Southern Sub-Basin Aquifers. Desert Ecosystem Engineering Journal, 9, 103-115.	-	Approximated from Fig. 1 by Ghafari et al. (2018) and Fig. 1 by Yonesi et al. (2020)

Aquifer system	Broader aquifer system	Reference A	Reference B	Reference C	Steps to delineate
**Chahak -e Shahriari Basin**	-	Hosseini, S. M., Parizi, E., Ataie-Ashtiani, B., Simmons, C. T. (2019). Assessment of sustainable groundwater resources management using integrated environmental index: Case studies across Iran. Science of the Total Environment, 676, 792-810.	-	-	Approximated from Fig. 6 by Hosseini et al. (2019)
**Chahak Musaviyeh Basin**	-	Hosseini, S. M., Parizi, E., Ataie-Ashtiani, B., Simmons, C. T. (2019). Assessment of sustainable groundwater resources management using integrated environmental index: Case studies across Iran. Science of the Total Environment, 676, 792-810.	-	-	Approximated from Fig. 6 by Hosseini et al. (2019)
**Chahghah Basin**	-	Parizi, E., Hosseini, S. M., Ataie-Ashtiani, B., Simmons, C. T. (2020). Normalized difference vegetation index as the dominant predicting factor of groundwater recharge in phreatic aquifers: case studies across Iran. Scientific Reports, 10, 17473.	-	-	Approximated from tabulated coordinates in the Supplementary Information within Parizi et al. (2020) and physiographic and drainage patterns
**Chaj Doab**	Indus Basin	Hussain, S. D., Sajjad, M. I., Akram, W., Ahmad, M., Rafiq, M., & Tariq, J. A. (1990). Surface water/groundwater relationship in Chaj Doab. Pakistan Inst. of Nuclear Science and Technology,	Ashraf, A., Ahmad, Z. (2008). Regional groundwater flow modelling of Upper Chaj Doab of Indus Basin, Pakistan using finite element model (Feflow) and geoinformatics. Geophysical	Ashraf, A., Ahmad, Z., Akhter, G. (2018). Monitoring groundwater flow dynamics and vulnerability to climate change in Chaj Doab, Indus Basin, through modeling approach. In: Groundwater	Approximated from Fig. 2 by Ashraf and Ahmad (2008), Fig. 1 by Hussain et al. (1990), and Fig. 35.1 by Ashraf et al. (2018)

Aquifer system	Broader aquifer system	Reference A	Reference B	Reference C	Steps to delineate
		Report No. PINSTECH/RIAD--122, 74 pp. Accessed July 10, 2021 from https://inis.iaea.org/collection/NCLCollectionStore/_Public/22/031/22031202.pdf?r=1	Journal International, 173, 17-24.	of South Asia (ed. A. Mukherjee), Springer, Singapore, pp. 593-611.	
**Chaleant Valley**	-	Daskin, W.R. (1988). Preliminary evaluation of the hydrogeologic system in Owens Valley, California. US Geological Survey Water-Resources Investigations Report 88-4003, 81 pp. Accessed April 27, 2022 via https://pubs.usgs.gov/wri/1988/4003/report.pdf	-	-	Approximated from Fig. 1 by Daskin (1988)
**Cheb Basin**	-	Kadlecová, R., Olmer, M. (2011). Review of Groundwater Resources. Geologické výzkumy na Moravě a ve Slezsku. Proceedings of The Open Congress ČGS and SGS, September 22–25, 2011, Monínec 18, pp. 31-34. Accessed October 21, 2021 from https://journals.muni.cz/gvms/article/view/1679	Krauze, P., Kämpf, H., Horn, F., Liu, Q., Voropaev, A., Wagner, D., Alawi, M. (2017). Microbiological and geochemical survey of CO2-dominated mofette and mineral waters of the Cheb Basin, Czech Republic. Frontiers in Microbiology, 8, 2446.	Koch, U., Heinicke, J. (2007). Hydrological influences on long-term gas flow trends at locations in the Vogtland/NW Bohemian seismic region (German-Czech border). Annals of Geophysics, 60, 557-568. Accessed October 21, 2021 from https://www.earth-prints.org/bitstream/2122/3869/1/7%20koch.pdf	Approximated from Fig. 1 by Kadlecová and Olmer (2011), Fig. 1 by Krauze et al. (2017), and Fig. 1 by Koch and Heinicke (2007)
**Chehelkhaneh Aquifer**	-	Yonesi, H., Arshia, A., Torabipoudeh, H., Shahinejad, B., Sayedipour, M., Vahdatpour, N. (2020). Evaluating Groundwater Quality in Zayandehrood Southern Sub-Basin Aquifers. Desert Ecosystem	-	-	Approximated from Fig. 1 by Yonesi et al. (2020)

Aquifer system	Broader aquifer system	Reference A	Reference B	Reference C	Steps to delineate
		Engineering Journal, 9, 103-115.			
**Chengdu Plain**	-	Zhang, H., Xu, Y., Cheng, S., Li, Q., Yu, H. (2020). Application of the dual-isotope approach and Bayesian isotope mixing model to identify nitrate in groundwater of a multiple land-use area in Chengdu Plain, China. Science of The Total Environment, 717, 137134.	Zhang, H., Yang, R., Wang, Y., Ye, R. (2019). The evaluation and prediction of agriculture-related nitrate contamination in groundwater in Chengdu Plain, southwestern China. Hydrogeology Journal, 27, 785-799.	-	Approximated from Fig. 1 by Zhang et al. (2020) and Fig. 1 by Zhang et al. (2019)
**Chennai Coastal Aquifer**	Coromandel Coastal Plain	Central Ground Water Board (2017a). Aquifer mapping and ground water management Chennai Aquifer System. Central Ground Water Board Report, 56 pp. Accessed June 24, 2021 from http://cgwb.gov.in/AQM/NAQUIM_REPORT/TAMILNADU/chennai%20Aquifer%20system.pdf	Senthilkumar, M., Gnanasundar, D. (2020). Hydrogeological characterization and hydrological modeling for devising groundwater management strategies for Chennai aquifer system, Southern India. Authorea Preprints. Accessed June 24, 2021 from https://www.authorea.com/doi/full/10.22541/au.158990356.67099058	-	Approximated from Fig. 10 and descriptions in text by Central Ground Water Board (2017a) and following hydrogeologic descriptions by Senthilkumar and Gnanasundar (2020).
**Chicago Valley**	-	Lopes, T.J., Evetts. D.M. (2005). Ground-Water pumpage and artificial recharge estimates for calendar year 2000 and average annual natural recharge and interbasin flow by hydrographic area, Nevada. Accessed April 25, 2022 via https://pubs.usgs.gov/sir/2004/5239/sir2004-5239.pdf	Harrill, J.R., Prudic, D.E. (1998). Aquifer systems in the Great Basin region of Nevada, Utah, and adjacent states; summary report. US Geological Survey Professional Paper 1409-A, 75 pp. Accessed April 26, 2022 via https://pubs.usgs.gov/pp/1409a/report.pdf	-	Approximated from Fig. 1 by Lopes and Evetts (2005), and Fig. 2 by Harrill and Prudic (1998). Well completion records, physiography and drainage were used to delinate areas.

**Aquifer system**	**Broader aquifer system**	**Reference A**	**Reference B**	**Reference C**	**Steps to delineate**
**Chicxulub Sedimentary Basin**	Yucatan Peninsula	Bauer-Gottwein, P., Gondwe, B. R., Charvet, G., Marín, L. E., Rebolledo-Vieyra, M., Merediz-Alonso, G. (2011). the Yucatán Peninsula karst aquifer, Mexico. Hydrogeology Journal, 19, 507-524.	Munro-Stasiuk, M.J., Manahan, T. K. (2010). Investigating ancient Maya agricultural adaptation through ground penetrating radar (GPR) analysis ofkarst terrain, Northern Yucatán, Mexico. Acta Carsologica, 39, 123-36.	-	Approximated from Fig. 2 by Bauer-Gottwein et al. (2011), and from Fig. 2 by Munro-Stasiuk and Manahan (2010).
**Chimbarongo and San Fernando Aquifers**	Chilean Central Valley	Direccion General de Aguas (2017). Inventario Nacional de acuiferos. Ministerio De Obras Públicas (Gobierno De Chile) report number 403, 60 pp. Accessed May 17, 2021 from https://snia.mop.gob.cl/sad/SUB5748.pdf	-	-	Approximated from map on page 42 by Direccion General de Aguas (2017). Central Valley approximated between Biobio Aquifer (~38 degrees south) to Chimbarongo and San Fernando Aquifers (~34.5 degrees south).
**Chino Basin**	Upper Santa Ana Basin**	California Department of Water Resources (2015e). California's groundwater update 2013 - Chapter 6: South Coast Hydrologic Region. California Department of Water Resources Report, 84 pp. Accessed April 27, 2022 via https://cawaterlibrary.net/wp-content/uploads/2017/05/GWU2013_Ch6_SouthCoast_Final.pdf	Kent, R., Belitz, K. (2009). Ground-water quality data in the Upper Santa Ana Watershed Study Unit, November 2006 to March 2007: Results from the California GAMA Program: U.S. Geological Survey Data Series 404, 116 pp. Accessed March 21, 2021 from https://pubs.usgs.gov/ds/404/ds404.pdf	Wildermuth Environmental (2005). Chino Basin Optimum Basin Management Program State of the Basin Report - 2004. Report prepared for Chino Basin Watermaster. 643 pp. Accessed April 12, 2022 via http://www.cbwm.org/docs/engdocs/isob/ISOB_Final_FullVersion.pdf	Approximated from Fig. 6-2 by California Department of Water Resources (2015e), Fig. 2 of Kent and Belitz (2009) and Fig. 2-1 by Wildermuth Environmental (2005)
**Chittagong Coast Aquifer**	-	Ravenscroft, P., McArthur, J. M., Rahman, M. S. (2018). Identifying multiple deep aquifers in the Bengal Basin: Implications for resource management. Hydrological Processes, 32, 3615-3632.	-	-	Approximated from Fig. 1 by Ravenscroft et al. (2018)

Aquifer system	Broader aquifer system	Reference A	Reference B	Reference C	Steps to delineate
**Cholqui Aquifer**	-	Direccion General de Aguas (2017). Inventario Nacional de acuiferos. Ministerio De Obras Públicas (Gobierno De Chile) report number 403, 60 pp. Accessed May 17, 2021 from https://snia.mop.gob.cl/sad/SUB5748.pdf	-	-	Approximated from map on page 42 by Direccion General de Aguas (2017).
**Choshui Alluvial Fan**	-	Chen, W. F., Liu, T. K. (2003). Dissolved oxygen and nitrate of groundwater in Choshui Fan-Delta, western Taiwan. Environmental Geology, 44, 731-737.	Liu, C. H., Pan, Y. W., Liao, J. J., Huang, C. T., Ouyang, S. (2004). Characterization of land subsidence in the Choshui River alluvial fan, Taiwan. Environmental Geology, 45, 1154-1166.	Yu, H. L., Chu, H. J. (2010). Understanding space–time patterns of groundwater system by empirical orthogonal functions: a case study in the Choshui River alluvial fan, Taiwan. Journal of Hydrology, 381, 239-247.	Approximated from Figs. 1 and 2 by Chen and Liu (2003), Fig. 1 by Liu et al. (2004) and Fig. 1 by Yu and Chu (2010)
**Chowchilla Basin**	San Joaquin Basin of the Central Valley **	California Department of Water Resources (2015h). California's groundwater update 2013 - Chapter 8: San Joaquin River Hydrologic Region. California Department of Water Resources Report, 122 pp. Accessed April 28, 2022 via https://water.ca.gov/-/media/DWR-Website/Web-Pages/Programs/Groundwater-Management/Data-and-Tools/Files/Statewide-Reports/California-Groundwater-Update-2013/California-Groundwater-Update-2013---Chapter-8---San-Joaquin-River.pdf	-	-	Approximated from Fig. 8-2 by California Department of Water Resources (2015h)
**Chupaderos Aquifer**	-	Ortiz Letechipia, J., González-Trinidad, J.,	-	-	Approximated from Fig. 1 by Ortiz Letechipia et al. (2022)

Aquifer system	Broader aquifer system	Reference A	Reference B	Reference C	Steps to delineate
		Júnez-Ferreira, H. E., Bautista-Capetillo, C., Robles-Rovelo, C. O., Contreras Rodríguez, A. R., Dávila-Hernández, S. (2022). Aqueous arsenic speciation with hydrogeochemical modeling and correlation with fluorine in groundwater in a semiarid region of Mexico. Water, 14, 519.			
**Churchill Valley**	Carson River Basin	Maurer, D. K. (2011). Geologic framework and hydrogeology of the middle Carson River Basin, Eagle, Dayton, and Churchill Valleys, West-Central Nevada. U. S. Geological Survey Scientific Investigations Report 2011–5055. 74 pp. Accessed March 10, 2021 from https://pubs.usgs.gov/sir/2011/5055/pdf/sir20115055.pdf	-	-	Approximated from Fig. 1 of Maurer et al. (2011).
**Cienega Prieta-Moroleon Aquifer**	-	CONAGUA (2021a). Acuíferos (nacional). Webpage accessed March 8, 2022 from http://sina.conagua.gob.mx/sina/tema.php?tema=acuiferos&ver=mapa&o=1&n=nacional	-	-	Approximated from aquifer boundaries defined by CONAGUA (2021a) web portal, physiography and drainage patterns, and well completion data.
**Cimarron Basin**	-	Adams, G.P., Bergman, D.L. (1996). Geohydrology of alluvium and terrace deposits of the Cimarron River from freedom to Guthrie, Oklahoma. U.S.	-	-	Approximated from Fig. 1 of Adams and Bergman (1996).

Aquifer system	Broader aquifer system	Reference A	Reference B	Reference C	Steps to delineate
		Geological Survey Water-Resources Investigations Report 95-4066, 63 pp. Accessed April 5, 2021 from https://pubs.usgs.gov/wri/1995/4066/report.pdf			
**Cingla-Cuchillo Aquifer**	-	Rupérez-Moreno, C., Senent-Aparicio, J., Martinez-Vicente, D., García-Aróstegui, J. L., Calvo-Rubio, F. C., Pérez-Sánchez, J. (2017). Sustainability of irrigated agriculture with overexploited aquifers: The case of Segura basin (SE, Spain). Agricultural Water Management, 182, 67-76.	-	-	Approximated from Fig. 1 by Rupérez-Moreno et al. (2017)
**Clarence-Moreton Basin and Cecil Plains**	Great Artesian Basin	Smerdon, B.D., Ramsley, T.R. (2012). Water Resource Assessment for the Surat Region. A technical report to the Australian Government from the CSIRO Great Artesian Basin Water Resource Assessment, 142 pp. Accessed July 20, 2021 from https://publications.csiro.au/rpr/download?pid=csiro:EP132644&dsid=DS4	Ransley, T.R., Smerdon, B.D. (2012). Hydrostratigraphy, hydrogeology and system conceptualisation of the Great Artesian Basin. A technical report to the Australian Government from the CSIRO Great Artesian Basin Water Resource Assessment, 324 pp. Accessed July 20, 2021 from https://publications.csiro.au/rpr/download?pid=csiro:EP132693&dsid=DS5	Owen, D. D., Raiber, M., Cox, M. E. (2015). Relationships between major ions in coal seam gas groundwaters: Examples from the Surat and Clarence-Moreton basins. International Journal of Coal Geology, 137, 77-91.	Approximated from Fig. 5.2 by Smerdon and Ransley (2012), Fig. 2.3 by Ransley and Smerdon (2012), and Fig. 1 by Owen et al. (2015)
**Clayton Basin**	-	California Department of Water Resources (2015g). California's groundwater update 2013 - Chapter 4: San Francisco Bay Hydrologic Region. California Department of	-	-	Approximated from Fig. 4-2 by California Department of Water Resources (2015g)

Aquifer system	Broader aquifer system	Reference A	Reference B	Reference C	Steps to delineate
		Water Resources Report, 78 pp. Accessed April 27, 2022 via https://cawaterlibrary.net/wp-content/uploads/2017/05/GWU2013_Ch4_SanFranciscoBay_Final.pdf			
**Coachella Valley**	-	Sneed, M., Brandt, J.T., Solt, M. (2014). Land subsidence, groundwater levels, and geology in the Coachella Valley, California, 1993–2010: U.S. Geological Survey, Scientific Investigations Report 2014–5075, 62 pp. Accessed February 15, 2021 from https://pubs.usgs.gov/sir/2014/5075/pdf/sir2014-5075.pdf	-	-	Approximated from Figs. 1 and 2 of Sneed et al. (2014)
**Coal Valley**	-	Lopes, T.J., Evetts. D.M. (2005). Ground-Water pumpage and artificial recharge estimates for calendar year 2000 and average annual natural recharge and interbasin flow by hydrographic area, Nevada. Accessed April 25, 2022 via https://pubs.usgs.gov/sir/2004/5239/sir2004-5239.pdf	Harrill, J.R., Prudic, D.E. (1998). Aquifer systems in the Great Basin region of Nevada, Utah, and adjacent states; summary report. US Geological Survey Professional Paper 1409-A, 75 pp. Accessed April 26, 2022 via https://pubs.usgs.gov/pp/1409a/report.pdf	-	Approximated from Fig. 1 by Lopes and Evetts (2005), and Fig. 2 by Harrill and Prudic (1998). Well completion records, physiography and drainage were used to delinate areas.
**Coastal Aquifer**	-	Jebreen, H., Wohnlich, S., Wisotzky, F., Banning, A., Niedermayr, A., & Ghanem, M. (2018). Recharge estimation in semi-arid karst catchments: Central	Nativ, R., Weisbrod, N. (1994). Management of a multilayered Coastal aquifer—An Israeli case study. Water Resources Management, 8, 297-311.	Levi, E., Goldman, M., Tibor, G., Herut, B. (2018). Delineation of subsea freshwater extension by marine geoelectromagnetic soundings (SE Mediterranean Sea). Water	Approximated from Fig. 1 by Jebreen et al. (2018), Fig. 4 by Nativ and Weisbrod (1994), and Fig. 1 by Levi et al. (2018)

Aquifer system	Broader aquifer system	Reference A	Reference B	Reference C	Steps to delineate
		West Bank, Palestine. Grundwasser, 23, 91-101.		Resources Management, 32, 3765-3779.	
**Coastal Caborca Aquifer**	Caborca Aquifer System	Cañez Araiza, D.A. (2018). Caracterización hidrogeoquímica y situación actual de la intrusión marina en la porción costera del acuífero Caborca, Sonora, México. Universidad de Sonora M.Sc. Thesis, 175 pp. Accessed May 6, 2021 from http://www.repositorioinstitucional.uson.mx/bitstream/handle/unison/1935/canezaraizadaynaangelicam.pdf?sequence=1	Vega-Granillo, E.L., Cirett-Galán, S, De la Parra-Velasco, M.L., Zavala-Juárez, R. (2011). Hidrogeología de Sonora, México. in: Calmus, Thierry, ed., Panorama de la geología de Sonora, México, Universidad Nacional Autónoma de México, Instituto de Geología, Boletín 118, 267–298. Accessed May 6, 2021 from	-	Approximated from Fig. 3 by Cañez Araiza (2018) and from Fig. 5 of Vega-Granillo et al. (2011).
**Coastal Maine Crystalline Aquifers**	-	Harte, P.T., Robinson, G.R., Jr., Ayotte, J.D., Flanagan, S.F. (2008). Framework for evaluating water quality of the New England crystalline rock aquifers. U.S. Geological Survey Open-File Report 2008–1282, 47 pp. Accessed April 15, 2021 from https://pubs.usgs.gov/of/2008/1282/pdf/ofr2008-1282.pdf	-	-	Approximated from Fig. 4 and "Coastal Maine" geologic province by Harte et al. (2008).
**Coastal Rayong Basin**	Rayong Basin	Boonkaewwan, S., Sonthiphand, P., Chotpantarat, S. (2021). Mechanisms of arsenic contamination associated with hydrochemical characteristics in coastal alluvial aquifers using multivariate statistical technique and hydrogeochemical	Piyapong, J., Thidarat, B., Jaruwan, C., Siriphan, N., Passanan, A. (2019). Enhancing citizens' sense of personal responsibility and risk perception for promoting public participation in sustainable groundwater resource management in Rayong Groundwater Basin,	-	Approximated from Fig. 1 by Boonkaewwan et al. (2021), Fig. 2 by Piyapong et al. (2019). Subareas delineated on the basis of physiography, drainage and groundwater well prevelance

Aquifer system	Broader aquifer system	Reference A	Reference B	Reference C	Steps to delineate
		modeling: a case study in Rayong province, eastern Thailand. Environmental Geochemistry and Health, 43, 537-566.	Thailand. Groundwater for Sustainable Development, 9, 100252.		
**Coastal Saurashtra Miliolite Limestone Aquifer**	-	Rushton, K. R., Rao, S. R. (1988). Groundwater flow through a Miliolite limestone aquifer. Hydrological sciences journal, 33(5), 449-464.	Saha, D., Ray, R. K. (2019). Groundwater resources of India: potential, challenges and management. In Groundwater Development and Management. Springer, Cham (ed. Sikdar, P.K.), pp. 19-42.	-	Approximated from Fig. 1 by Rushton and Rao (1988) and Fig. 2.2 by Saha and Ray (2019)
**Coatzacoalcos Coastal Aquifer**	Coastal Plain of Veracruz	Camacho, E. A. S. (2010). Estimación del volumen promedio recibido por el humedal de la subcuenca del Río Blanco (bajo Papaloapan; Veracruz), a través del cálculo de un balance de aguas. Aqua-LAC, 2, 78-87.	Campos C, A., Hernández, M. E., Moreno-Casasola, P., Cejudo Espinosa, E., Robledo R, A., Infante Mata, D. (2011). Soil water retention and carbon pools in tropical forested wetlands and marshes of the Gulf of Mexico. Hydrological Sciences Journal, 56, 1388-1406.	-	Eastern margin approximated following the southeastern margin of the Veracruz Coastal Plain (from Campos et al., 2011), and western boundary defined by the Papaloapan Basin.
**Codegua and Graneros-Rancagua Aquifers**	-	Direccion General de Aguas (2017). Inventario Nacional de acuiferos. Ministerio De Obras Públicas (Gobierno De Chile) report number 403, 60 pp. Accessed May 17, 2021 from https://snia.mop.gob.cl/sad/SUB5748.pdf	-	-	Approximated from map on page 42 by Direccion General de Aguas (2017).
**Cold Lake-Beaver River Basin**	-	Alberta Environment (2006). State of the Basin: Cold Lake Beaver River Basin. Report, 85 pp. Accessed April 26, 2021 from https://open.alberta.ca/dat	-	-	Approximated from Fig. 2-2 of Albreta Environment (2006).

Aquifer system	Broader aquifer system	Reference A	Reference B	Reference C	Steps to delineate
		aset/1566ed51-e765-468d-99d5-cfb9f08be4d5/resource/e1317376-a2d4-4f93-8834-b95963c3daf7/download/2006-coldlake-beavergroundwaterreport-2006.pdf			
**Columbus Lowland and Darby Plain**	Central Lowland Till Plain	Brockman, S. (1998). Physiographic regions of Ohio. Ohio Department of Natural Resources, Division of Geological Survey map. Accessed April 12, 20221 from https://www.epa.state.oh.us/portals/27/SIP/Nonattain/F2-physiographic_regions_of_Ohio.pdf	Fenneman, N.M., Johnson, D.W. (1946). Physiographic divisions of the conterminous United States. U.S. Geological Survey map, 1:7,000,000 Scale.	-	Broader Till Plain physiographic region approximated from Fenneman and Johnson (1946). Columbus Lowland and Darby Plain areas approximated from the map by Brockman (1998).
**Columbus Salt Marsh Valley**	-	Lopes, T.J., Evetts. D.M. (2005). Ground-Water pumpage and artificial recharge estimates for calendar year 2000 and average annual natural recharge and interbasin flow by hydrographic area, Nevada. Accessed April 25, 2022 via https://pubs.usgs.gov/sir/2004/5239/sir2004-5239.pdf	Harrill, J.R., Prudic, D.E. (1998). Aquifer systems in the Great Basin region of Nevada, Utah, and adjacent states; summary report. US Geological Survey Professional Paper 1409-A, 75 pp. Accessed April 26, 2022 via https://pubs.usgs.gov/pp/1409a/report.pdf	-	Approximated from Fig. 1 by Lopes and Evetts (2005), and Fig. 2 by Harrill and Prudic (1998). Well completion records, physiography and drainage were used to delinate areas.
**Comarca Laguna Aquifer**	-	Torres-Martínez, J. A., Mora, A., Mahlknecht, J., Daesslé, L. W., Cervantes-Avilés, P. A., Ledesma-Ruiz, R. (2021). Estimation of nitrate pollution sources and transformations in groundwater of an intensive livestock-agricultural area	Mahlknecht, J., Aguilar-Barajas, I., Farias, P., Knappett, P.S., Torres-Martínez, J.A., Hoogesteger, J., Lara, R.H., Ramírez-Mendoza, R.A., Mora, A. (2023). Hydrochemical controls on arsenic contamination and	-	Approximated from Fig. 1 by Torres-Martínez et al. (2021) and Fig. 1 by Mahlknecht et al. (2023)

Aquifer system	Broader aquifer system	Reference A	Reference B	Reference C	Steps to delineate
		(Comarca Lagunera), combining major ions, stable isotopes and MixSIAR model. Environmental Pollution, 269, 115445.	its health risks in the Comarca Lagunera region (Mexico): Implications of the scientific evidence for public health policy. Science of the Total Environment, 857, 159347.		
**Conejo Basin**	-	California Department of Water Resources (2015e). California's groundwater update 2013 - Chapter 6: South Coast Hydrologic Region. California Department of Water Resources Report, 84 pp. Accessed April 27, 2022 via https://cawaterlibrary.net/wp-content/uploads/2017/05/GWU2013_Ch6_SouthCoast_Final.pdf	-	-	Approximated from Fig. 6-2 by California Department of Water Resources (2015e)
**Confined Claiborne Near Jackson**	Mississippi Embayment	Stephenson, L.W. (1941). The ground-water resources of Mississippi. U.S. Geological Survey Water-Supply Paper 576, 540 pp. Accessed March 11, 2021 from https://pubs.usgs.gov/wsp/0576/report.pdf	-	-	Discussion on page 200 describing access to Claiborne aquifer (part of the Mississippi Embayment Aquifer System) by wells near Jackson
**Connecticut Valley**	-	Olcott, P.G. (1995). Ground Water Atlas of the United States: Segment 12, Connecticut, Maine, Massachusetts, New Hampshire, New York, Rhode Island, Vermont. U.S. Geological Survey Hydrologic Atlas 730-M, 30 pp. Accessed April 14, 2021 from	Connecticut Department of Energy and Environmental Protection (2021). Webpage entitled "Overview of the Ground Water Flow System in Connecticut". Accessed April 14, 2021 from https://portal.ct.gov/DEEP/Aquifer-Protection-and-Groundwater/Ground-	-	Approximated from map displayed at top of webpage by Connecticut Department of Energy and Environmental Protection (2021) and also from Fig. 102 of Olcott (1995).

Aquifer system	Broader aquifer system	Reference A	Reference B	Reference C	Steps to delineate
		https://pubs.usgs.gov/ha/730m/report.pdf	Water/Ground-Water-Flow-System-in-Connecticut		
**Consuegra-Villacanas Aquifer**	-	Yustres, Á., Navarro, V., Asensio, L., Candel, M., García, B. (2013). Groundwater resources in the Upper Guadiana Basin (Spain): a regional modelling analysis. Hydrogeology Journal, 21, 1129.	-	-	Approximated from Fig. 1 by Yustres et al. (2013)
**Coonamble Embayment**	Great Artesian Basin	Ransley, T.R., Smerdon, B.D. (2012). Hydrostratigraphy, hydrogeology and system conceptualisation of the Great Artesian Basin. A technical report to the Australian Government from the CSIRO Great Artesian Basin Water Resource Assessment, 324 pp. Accessed July 20, 2021 from https://publications.csiro.au/rpr/download?pid=csiro:EP132693&dsid=DS5	Wolfgang, C. (2000). Hydrogeology of the Pilliga sandstone aquifer in the Western Coonamble embayment and its implications for water resource management. PhD Thesis, Australia National University, 537 pp. Accessed July 20, 2021 from https://openresearch-repository.anu.edu.au/handle/1885/9902	Smerdon, B.D., Ramsley, T.R. (2012). Water Resource Assessment for the Surat Region. A technical report to the Australian Government from the CSIRO Great Artesian Basin Water Resource Assessment, 142 pp. Accessed July 20, 2021 from https://publications.csiro.au/rpr/download?pid=csiro:EP132644&dsid=DS4	Approximated from Fig. 2.3 by Ransley and Smerdon (2012), Fig. 5.2 by Smerdon and Ransley (2012), and Fig. 2 by Wolfgang (2000)
**Cordoba Basin**	Orizaba-Cordoba Basins	CONAGUA (2015a). Actuualizacion de la disponibilidad media anual de agua en el acuifero Orizaba-Cordoba (3007), Estado de Veracruz. 23 pp. Accessed May 11, 2021 from https://www.gob.mx/cms/uploads/attachment/file/104452/DR_3007.pdf	-	-	Approximated from map on page 13 by CONAGUA (2015a)
**Corning Basin**	Sacramento Valley of	California Department of Water Resources (2015i). California's groundwater	-	-	Approximated from Fig. 7-2 by California Department of Water Resources (2015i)

Aquifer system	Broader aquifer system	Reference A	Reference B	Reference C	Steps to delineate
	the Central Valley **	update 2013 - Chapter 7: Sacramento River Hydrologic Region. California Department of Water Resources Report, 126 pp. Accessed April 27, 2022 via https://water.ca.gov/-/media/DWR-Website/Web-Pages/Programs/Groundwater-Management/Bulletin-118/Files/Statewide-Reports/GWU2013_Ch7_SacramentoRiver_Final.pdf			
**Costa de Hermosillo aquifer**	-	Steinich, B., Escolero, O., Marín, L. E. (1998). Salt-water intrusion and nitrate contamination in the Valley of Hermosillo and El Sahuaral coastal aquifers, Sonora, Mexico. Hydrogeology Journal, 6, 518-526.	Vega-Granillo, E.L., Cirett-Galán, S, De la Parra-Velasco, M.L., Zavala-Juárez, R. (2011). Hidrogeología de Sonora, México. in: Calmus, Thierry, ed., Panorama de la geología de Sonora, México, Universidad Nacional Autónoma de México, Instituto de Geología, Boletín 118, 267–298. Accessed May 6, 2021 from	Szynkiewicz, A., Medina, M. R., Modelska, M., Monreal, R., Pratt, L. M. (2008). Sulfur isotopic study of sulfate in the aquifer of Costa de Hermosillo (Sonora, Mexico) in relation to upward intrusion of saline groundwater, irrigation pumping and land cultivation. Applied Geochemistry, 23, 2539-2558. Accessed April 17, 2023 via https://www.sciencedirect.com/science/article/pii/S0883292708002291#fig2	Approximated from Fig. 5 of Vega-Granillo et al. (2011), Fig. 1 of Steinich et al. (1998) and Fig. 1 by Szynkiewica et al. (2008).
**Cosumnes Basin**	San Joaquin Basin of the Central Valley **	California Department of Water Resources (2015h). California's groundwater update 2013 - Chapter 8: San Joaquin River Hydrologic Region. California Department of Water Resources Report, 122 pp. Accessed April 28, 2022 via	-	-	Approximated from Fig. 8-2 by California Department of Water Resources (2015h)

Aquifer system	Broader aquifer system	Reference A	Reference B	Reference C	Steps to delineate
		https://water.ca.gov/-/media/DWR-Website/Web-Pages/Programs/Groundwater-Management/Data-and-Tools/Files/Statewide-Reports/California-Groundwater-Update-2013/California-Groundwater-Update-2013---Chapter-8---San-Joaquin-River.pdf			
**Covelo Round Valley**	-	California Department of Water Resources (2015j). California's groundwater update 2013 - Chapter 3: North Coast Hydrologic Region. California Department of Water Resources Report, 80 pp. Accessed April 27, 2022 via https://water.ca.gov/-/media/DWR-Website/Web-Pages/Programs/Groundwater-Management/Bulletin-118/Files/Statewide-Reports/GWU2013_Ch3_NorthCoast_Final.pdf	-	-	Approximated from Fig. 3-2 by California Department of Water Resources (2015j)
**Coyote Springs Valley**	-	Burbey, T. J. (1997). Hydrogeology and potential for ground-water development, carbonate-rock aquifers in southern Nevada and southeastern California. US Geological Survey Water-Resources Investigations Report 95-4168, 70 pp. Accessed March 10, 2021 from https://pubs.usgs.gov/wri/1995/4168/report.pdf	-	-	Approximated from Fig. 7 of Burbey (1997)

Aquifer system	Broader aquifer system	Reference A	Reference B	Reference C	Steps to delineate
**Coyote Wells Valley**	-	California Department of Water Resources (2015a). California's groundwater update 2013 - Chapter 12: Colorado River Hydrologic Region. California Department of Water Resources Report, 76 pp. Accessed April 27, 2022 via https://water.ca.gov/-/media/DWR-Website/Web-Pages/Programs/Groundwater-Management/Bulletin-118/Files/Statewide-Reports/GWU2013_Ch12_ColoradoRiver_Final.pdf	-	-	Approximated from Fig. 12-2 by California Department of Water Resources (2015a)
**Crau Plain**	-	Seraphin, P., Gonçalvès, J., Vallet-Coulomb, C., Champollion, C. (2018). Multi-approach assessment of the spatial distribution of the specific yield: application to the Crau plain aquifer, France. Hydrogeology Journal, 26, 1221-1238.	Mayer, A., Nguyen, B. T., Banton, O. (2016). Using radon-222 to study coastal groundwater/surface-water interaction in the Crau coastal aquifer (southeastern France). Hydrogeology Journal, 24, 1775-1789.	-	Approximated from Fig. 1 by Seraphin et al. (2018) and Fig. 1 by Mayer et al. (2016).
**Crawford Upland**	Interior Low Plateaus	Miller, J.A. (1995). Ground Water Atlas of the United States: Segment 10, Illinois, Indiana, Kentucky, Ohio, Tennessee. U.S. Geological Survey Hydrologic Atlas 730-K, 30 pp. Accessed April 11, 2021 from https://pubs.usgs.gov/ha/730k/report.pdf	Taylor, C.J., Nelson Jr., H.L. (2008). A compilation of provisional karst geospatial data for the Interior Low Plateaus physiographic region, central United States: U.S. Geological Survey Data Series 339, 26 pp. Accessed April 11, 2021 from https://pubs.usgs.gov/ds/339/pdf/ds339_web.pdf	Fenelon, J.M., Bobay, K.E., Greeman, T.K., Hoover, M.E., Cohen, D.A., Fowler, K.K., Woodfield, M.C., Durbin, J.M. (1994). Hydrogeologic atlas of aquifers in Indiana: U.S. Geological Survey Water-Resources Investigations Report 1992–4142, 197 pp. Accessed April 11, 2021 from https://pubs.er.usgs.gov/publication/wri924142	Broader Interior Low Plateaus outline approximated for Tennessee, Illinois, Kentucky, Indiana from Fig. 78 of Miller (1990) and Fig. 1 of Taylor and Nelson (2008). Crawford Upland approximated from Fig. 2 of Fenelon et al. (1994).

Aquifer system	Broader aquifer system	Reference A	Reference B	Reference C	Steps to delineate
**Crescent Valley in northern Nevada**	-	Bredehoeft, J.D., Farvolden, R.N. (1963). International Association of Scientific Hydrology, Commission of Subterranean Waters, Publication no. 64, p. 197–212. Accessed March 23, 2021 from http://hydrologie.org/redbooks/a064/064017.pdf	-	-	Approximated from Fig. 1 of Bredehoft and Farvolden (1963)
**Cretaceous Limestone and Guadalajara Detrital Aquifers**	-	Arauzo, M., Martínez-Bastida, J. J. (2015). Environmental factors affecting diffuse nitrate pollution in the major aquifers of central Spain: groundwater vulnerability vs. groundwater pollution. Environmental Earth Sciences, 73, 8271-8286.	Iepure, S., Martinez-Hernandez, V., Herrera, S., Rasines-Ladero, R., de Bustamante, I. (2013). Response of microcrustacean communities from the surface—groundwater interface to water contamination in urban river system of the Jarama basin (central Spain). Environmental Science and Pollution Research, 20, 5813-5826.	-	Approximated from Fig. 1 by Arauzo and Martínez-Bastida (2015), and Fig. 1 by Iepure et al. (2013)
**Crow Flats**	-	Bjorklund, L.J. (1957). Reconnaissance of Ground Water Conditions in the Crow Flats Area. New Mexico State Engineer Office Technical Report No. 8, 31 pp. Accessed April 27, 2022 via http://www.oteroswcd.org/PDF/NM%20OSE%20Reconnaissance%20of%20Ground%20Water%20Conditions%20in%20the%20Crow%20Flats%20Area%201957.pdf	-	-	Approximated from Fig. 1 by Bjorklund (1957)

Aquifer system	Broader aquifer system	Reference A	Reference B	Reference C	Steps to delineate
**Cuauhtemoc Basin**	-	Alatorre, L. C., Díaz, R. E., Miramontes, S., Bravo, L. C., Sánchez, E. (2014). Spatial and temporal evolution of the static water level of the Cuauhtemoc Aquifer during the years 1973, 1991 and 2000: A geographical approach. Journal of Geographic Information System, 6, 572-584.	Peña, L. C. B., López, D. A. S., Cejudo, L. C. A., Santander, Á. P., Olavee, M. E. T., Olivasf, A. G. (2016). Identificación de áreas potenciales de recarga hídrica en el acuífero Cuauhtémoc (Chihuahua), mediante una evaluación espacial multi criterio. Estudios territoriales en México: Percepción remota y sistemas de información espacial, 339-362.	-	Approximated from Fig. 1 by Alatorre et al. (2014) and Fig. 1 by Peña et al. (2016)
**Cuautla-Yautepec Valley**	-	Vazquez Sanchez, E., Cortes, A., Jaimes Palomera, R., Fritz, P., Aravena, R. (1989). Hidrogeologia isotopica de los valles de Cuautla y Yautepec, Mexico. Geofísica Internacional, 28, 245-264.	-	-	Approximated from Fig. 2 by Vazquez Sanchez et al. (1989).
**Cuberta de el Saltador**	-	García-Meléndez, E., Ferrer Julià, M., Goy, J.L., Zazo, C. (2002). Reconstrucción morfoestructural mediante modelos de elevación digital en un SIG del fondo de la cuenca sedimentaria de la Cubeta del Saltador (Cordilleras Béticas Orientales). Accessed April 25, 2023 via https://digital.csic.es/handle/10261/247828	-	-	Approximated from García-Meléndez et al. (2002)
**Cubeta de Oliete and Aliaga-Calanda Aquifers**	-	Carceller-Layel, T., Costa-Alandí, C., Coloma-López, P., García-Vera, M. Á., & San Román-Saldaña, J. (2007). Groundwater in the	-	-	Approximated from Fig. 3 by Carceller-Layel et al. (2007); northern boundary extended to the southern bank of the Rio Ebro.

Aquifer system	Broader aquifer system	Reference A	Reference B	Reference C	Steps to delineate
		central sector of the Ebro Basin. Water Resources Development, 23, 165-187.			
**Cuchillos-Cabras and El Molar Aquifers**	-	Rupérez-Moreno, C., Senent-Aparicio, J., Martinez-Vicente, D., García-Aróstegui, J. L., Calvo-Rubio, F. C., Pérez-Sánchez, J. (2017). Sustainability of irrigated agriculture with overexploited aquifers: The case of Segura basin (SE, Spain). Agricultural Water Management, 182, 67-76.	-	-	Approximated from Fig. 1 by Rupérez-Moreno et al. (2017)
**Cuernavaca Valley**	-	Morales, P., Casar, I., Cortes, A., Arizabalo, R.D., Aravena, R. (1989). Environmental isotopes and geochemical investigation of groundwater in the north-western part of the State of Morelos, Mexico (IAEA-TECDOC--502). International Atomic Energy Agency (IAEA), pp. 379-398. Accessed May 10, 2021 from https://inis.iaea.org/collection/NCLCollectionStore/_Public/21/031/21031083.pdf?r=1	Jaimes-Palomera, L. R., Cortes-Silva, A., Vázquez-Sánchez, E., Aravena, R., Fritz, P., Drimmie, R. (1989). Geoquimica isotopica del sistema hidrogeologico del valle de Cuerna Vaca, estado de Morelos, Mexico. Geofísica Internacional, 28, 219-244.	-	Approximated from Plano II by Morales (1989) and Fig. 2 by Jaimes-Palomera et al. (1989).
**Culiacan Coastal Plain**	-	Cardoso, P.R. (1993). Saline water intrusion in Mexico. Transactions on Ecology and the Environment, 2, 37-43. Accessed May 22, 2021 from https://www.witpress.com/elibrary/wit-transactions-	-	-	Approximated from Fig. 1 (see "12.1") by Cardos (1993).

Aquifer system	Broader aquifer system	Reference A	Reference B	Reference C	Steps to delineate
		on-ecology-and-the-environment/2/13690			
**Cumberland Basin**	Maritimes Basin	Stapinsky, M. Michaud, Y., Morin, R.H., Butler, K.E., Deblonde, C., Chi, G, Thériault, T., Castonguay, S., Boisvert, V., Julien, H.P., Conohan, D., Hulsman, B., Carrier, C. Marion, J., Boisvert, É. (2002). Groundwater resources assessment in the Carboniferous Maritimes Basin: preliminary results of the hydrogeological characterization, New Brunswick, Nova Scotia, and Prince Edward Island. Geological Survey of Canada Current Research Report 2002-D8, 14 pp. Accessed May 5, 2021 from http://www.gov.pe.ca/photos/original/cle_WA10.pdf	Rivard, C., Michaud, Y., Lefebvre, R., Deblonde, C., Rivera, A. (2008). Characterization of a regional aquifer system in the Maritimes Basin, Eastern Canada. Water Resources Management, 22, 1649-1675.	-	Approximated from Fig. 2 of Rivard et al. (2008) and Figs. 1 and 3 of Stapinsky et al. (2002). Western portion of this area delineated along divide between the "Sackville" and "Cumberland" subbasins defined in Fig. 3 by Stapinsky et al. (2002).
**Curlew Valley**	-	Graham, W.G., Campbell, L.J. (1981). Groundwater resources of Idaho. Idaho Department of Water Resources Report, 61 pp. Accessed March 23, 2021 from https://idwr.idaho.gov/files/publications/198108-MISC-GW-Resources-ID.pdf	-	-	Approximated from Plate 1 of Graham and Campbell (1981).
**Cuyama Valley**	-	Everett, R.R., Gibbs, D.R., Hanson, R.T., Sweetkind, D.S., Brandt, J.T., Falk, S.E., Harich, C.R. (2013). Geology, water-quality, hydrology, and	Sweetkind, D. S., Faunt, C. C., Hanson, R. T. (2013). Construction of 3-D geologic framework and textural models for Cuyama Valley groundwater basin,	-	Approximated from Fig. 1 of Everett et al. (2013) and Fig. 2 of Sweetkind et al. (2013)

Aquifer system	Broader aquifer system	Reference A	Reference B	Reference C	Steps to delineate
		geomechanics of the Cuyama Valley groundwater basin, California, 2008–12: U.S. Geological Survey Scientific Investigations Report 2013–5108, 62 pp. Accessed March 7, 2021 from https://pubs.usgs.gov/sir/2013/5108/pdf/sir2013-5108.pdf	California. US Geological Survey Scientific Investigations Report 2013-5127. 58 pp. Accessed March 7, 2021 from https://pubs.usgs.gov/sir/2013/5127/pdf/sir2013-5127.pdf		
**Cypress-Eastend Aquifer**	Bearpaw-Judith River Aquifer System	Davies, H., Hanley, P.T. (2010). State of the Watershed Report - 2010. Water Security Agency, Saskatchewan. Appendix A, 82 pp. Accessed April 26, 2021 from https://www.wsask.ca/Global/About%20WSA/Publications/State%20of%20the%20Watershed/d_AppendixA-ConditionIndicators.PDF	Ferris, D., Lypka, M., Ferguson, G. (2017). Hydrogeology of the Judith River formation in southwestern Saskatchewan, Canada. Hydrogeology Journal, 25(7), 1985-1995.	-	Approximated from Fig. 28 of Davies and Hanley (2010). Included in part of the Judith River Aquifer System (broader area), as wells within the area are known to tap the Judith River formation (see Fig. 3 of Ferris et al. (2017) and southern portion of map in Fig. 24 by Maathuis and Simpson (2007).
**Dagh -e Talleh Hamid Basin**	-	Hosseini, S. M., Parizi, E., Ataie-Ashtiani, B., Simmons, C. T. (2019). Assessment of sustainable groundwater resources management using integrated environmental index: Case studies across Iran. Science of the Total Environment, 676, 792-810.	-	-	Approximated from Fig. 6 by Hosseini et al. (2019)
**Dagh-e-Sorkh Basin**	-	Rezaei, A., Hassani, H. (2018). Hydrogeochemistry study and groundwater quality assessment in the north of Isfahan, Iran. Environmental	-	-	Approximated from Figs. 1 and 2 by Rezaei and Hassani (2018)

Aquifer system	Broader aquifer system	Reference A	Reference B	Reference C	Steps to delineate
		Geochemistry and Health, 40, 583-608.			
**Dakota Aquifer in southeast Nebraska**	-	Divine, D., Sibray, S.S. (2017). An overview of secondary aquifers in Nebraska. Conservation and Survey Division, Nebraska Educational Circular No. 26, 57 pp. Accessed April 27, 2022 via https://core.ac.uk/download/pdf/127441451.pdf	-	-	Approximated from Fig. 3 of Divine and Sibray (2017)
**Dakota Aquifer System**	-	Leonard, R. B., Signor, D. C., Jorgensen, D. G., Helgesen, J. O. (1983). Geohydrology and hydrochemistry of the Dakota Aquifer, central United States. Journal of the American Water Resources Association, 19(6), 903-912.	Bredehoeft, J. D., Neuzil, C. E., Milly, P. C. D. (1983). Regional flow in the Dakota aquifer: A study of the role of confining layers. US Geological Survey Water-Supply Paper 2237, 50 pp. Accessed on February 9, 2021 from https://pubs.er.usgs.gov/publication/wsp2237	Macfarlane, P. A., Doveton, J. H., Whittemore, D. O. (1998). User's Guide to the Dakota Aquifer in Kansas. Kansas Geological Survey. Accessed February 9, 2021 from http://www.kgs.ku.edu/Publications/Bulletins/TS2/index.html	Approximated the complete boundary of the Dakota Formation from Fig. 6 of Leonard et al. (1983). Other portions of the Dakota Aquifer were digitized from page 30 of Prior et al. (2003; Iowa portion) and Macfarlane et al. (1998; for eastern margin of Kansas' Dakota Aquifer (Fig. 2 of Macfarlane et al., 1998) and general boundaries extending into Canada (Fig. 1 of Macfarlane et al., 1998)). This specific portion of the Dakota was delineated by exploring well completion depths, mainly drawing from well completion records for South Dakota (specifically, records of deep wells).
**Dale Valley**	-	California Department of Water Resources (2015a). California's groundwater update 2013 - Chapter 12: Colorado River Hydrologic Region. California Department of Water Resources Report, 76 pp. Accessed April 27, 2022 via https://water.ca.gov/-	-	-	Approximated from Fig. 12-2 by California Department of Water Resources (2015a)

Aquifer system	Broader aquifer system	Reference A	Reference B	Reference C	Steps to delineate
		/media/DWR-Website/Web-Pages/Programs/Groundwater-Management/Bulletin-118/Files/Statewide-Reports/GWU2013_Ch12_ColoradoRiver_Final.pdf			
**Dalgan Basin**	Jazmurian Depression	Rezaei, A., Hassani, H., Tziritis, E., Mousavi, S. B. F., Jabbari, N. (2020). Hydrochemical characterization and evaluation of groundwater quality in Dalgan basin, SE Iran. Groundwater for Sustainable Development, 10, 100353.	Rezaei, A., Hassani, H., Hassani, S., Jabbari, N., Mousavi, S. B. F., Rezaei, S. (2019). Evaluation of groundwater quality and heavy metal pollution indices in Bazman basin, southeastern Iran. Groundwater for Sustainable Development, 9, 100245.	-	Approximated from Fig. 1 by Rezaei et al. (2020). Broader Jazmurian Depression from Fig. 2 by Rezaei et al. (2019)
**Dalmeny Aquifer**	-	Keller, C. K., Kamp, G. V. D., Cherry, J. A. (1986). Fracture permeability and groundwater flow in clayey till near Saskatoon, Saskatchewan. Canadian Geotechnical Journal, 23, 229-240.	Fortin, G., Van Der Kamp, G., Cherry, J. A. (1991). Hydrogeology and hydrochemistry of an aquifer-aquitard system within glacial deposits, Saskatchewan, Canada. Journal of Hydrology, 126, 265-292.	-	Approximated from Fig. 1 of Keller et al. (1996) and Fig. 1 of Fortin et al. (1991)
**Dalon Basin**	-	Parizi, E., Hosseini, S. M., Ataie-Ashtiani, B., Simmons, C. T. (2020). Normalized difference vegetation index as the dominant predicting factor of groundwater recharge in phreatic aquifers: case studies across Iran. Scientific Reports, 10, 17473.	-	-	Approximated from tabulated coordinates in the Supplementary Information within Parizi et al. (2020) and physiographic and drainage patterns
**Daly Basin**	Daly-Wiso-Georgina Basins	Schult, J. (2016). Herbicides, pesticides and nutrients in the Tindall aquifer, Katherine Region.	Tickell, S.J. (2002).Groundwater resources of the Oolloo Dolostone. Department of	-	Approximated from Fig. 1 by Schult (2016), Fig. 5 by Tickell (2002)

Aquifer system	Broader aquifer system	Reference A	Reference B	Reference C	Steps to delineate
		Northern Territory Government Department of Land Resource Management Report, 37 pp. Accessed October 5, 2021 from https://landresources.nt.gov.au/__data/assets/pdf_file/0019/282160/GWQ-report.pdf	Infrastructure Planning and Environment, Natural Resources Division Report, 43 pp. Accessed October 6, 2021 from https://citeseerx.ist.psu.edu/viewdoc/download?doi=10.1.1.932.9762&rep=rep1&type=pdf		
**Damanehdaran Aquifer**	-	Yonesi, H., Arshia, A., Torabipoudeh, H., Shahinejad, B., Sayedipour, M., Vahdatpour, N. (2020). Evaluating Groundwater Quality in Zayandehrood Southern Sub-Basin Aquifers. Desert Ecosystem Engineering Journal, 9, 103-115.	-	-	Approximated from Fig. 6 by Hosseini et al. (2019)
**Damghan Sedimentary Plain**	-	Ebrahimi, M., Kazemi, H., Ehtashemi, M., Rockaway, T. D. (2016). Assessment of groundwater quantity and quality and saltwater intrusion in the Damghan basin, Iran. Geochemistry, 76, 227-241.	Arabameri, A., Roy, J., Saha, S., Blaschke, T., Ghorbanzadeh, O., Tien Bui, D. (2019). Application of probabilistic and machine learning models for groundwater potentiality mapping in Damghan Sedimentary Plain, Iran. Remote Sensing, 11, 3015.	-	Approximated from Fig. 1 by Ebrahimi et al. (2016) and Fig. 1 by Arabameri et al. (2019)
**Darab Basin**	-	Hosseini, S. M., Parizi, E., Ataie-Ashtiani, B., Simmons, C. T. (2019). Assessment of sustainable groundwater resources management using integrated environmental index: Case studies across Iran. Science of the Total Environment, 676, 792-810.	Parizi, E., Hosseini, S. M., Ataie-Ashtiani, B., Simmons, C. T. (2020). Normalized difference vegetation index as the dominant predicting factor of groundwater recharge in phreatic aquifers: case studies across Iran. Scientific Reports, 10, 17473.	-	Approximated from Fig. 6 by Hosseini et al. (2019) and Fig. 2 by Parizi et al. (2020)

Aquifer system	Broader aquifer system	Reference A	Reference B	Reference C	Steps to delineate
**Darenjan Basin**	-	Hosseini, S. M., Parizi, E., Ataie-Ashtiani, B., Simmons, C. T. (2019). Assessment of sustainable groundwater resources management using integrated environmental index: Case studies across Iran. Science of the Total Environment, 676, 792-810.	-	-	Approximated from Fig. 6 by Hosseini et al. (2019)
**Dariyan Basin**	-	Hosseini, S. M., Parizi, E., Ataie-Ashtiani, B., Simmons, C. T. (2019). Assessment of sustainable groundwater resources management using integrated environmental index: Case studies across Iran. Science of the Total Environment, 676, 792-810.	-	-	Approximated from Fig. 6 by Hosseini et al. (2019)
**Darkoye and Ghareblagh Basins**	-	Hosseini, S. M., Parizi, E., Ataie-Ashtiani, B., Simmons, C. T. (2019). Assessment of sustainable groundwater resources management using integrated environmental index: Case studies across Iran. Science of the Total Environment, 676, 792-810.	-	-	Approximated from Fig. 6 by Hosseini et al. (2019)
**Darolmizan Basin**	-	Hosseini, S. M., Parizi, E., Ataie-Ashtiani, B., Simmons, C. T. (2019). Assessment of sustainable groundwater resources management using integrated environmental index: Case studies across	-	-	Approximated from Fig. 6 by Hosseini et al. (2019)

Aquifer system	Broader aquifer system	Reference A	Reference B	Reference C	Steps to delineate
		Iran. Science of the Total Environment, 676, 792-810.			
**Darulaman-Pagh Basin**	Kabul Basin	Jawadi, H. A., Sagin, J., Snow, D. D. (2020). A detailed assessment of groundwater quality in the Kabul Basin, Afghanistan, and suitability for future development. Water, 12, 2890.	-	-	Approximated from Fig. 1 and descriptions within section 2.1 by Jawadi et al. (2020).
**Daryache Namak Basin**	-	Parizi, E., Hosseini, S. M., Ataie-Ashtiani, B., Simmons, C. T. (2020). Normalized difference vegetation index as the dominant predicting factor of groundwater recharge in phreatic aquifers: case studies across Iran. Scientific Reports, 10, 17473.	-	-	Approximated from tabulated coordinates in the Supplementary Information within Parizi et al. (2020) and physiographic and drainage patterns
**Daryache Parishan Basin**	-	Hosseini, S. M., Parizi, E., Ataie-Ashtiani, B., Simmons, C. T. (2019). Assessment of sustainable groundwater resources management using integrated environmental index: Case studies across Iran. Science of the Total Environment, 676, 792-810.	-	-	Approximated from Fig. 6 by Hosseini et al. (2019); eastern boundary extended to Bikhak
**Dasht e-Afzar Basin**	-	Hosseini, S. M., Parizi, E., Ataie-Ashtiani, B., Simmons, C. T. (2019). Assessment of sustainable groundwater resources management using integrated environmental index: Case studies across	-	-	Approximated from Fig. 6 by Hosseini et al. (2019)

Aquifer system	Broader aquifer system	Reference A	Reference B	Reference C	Steps to delineate
		Iran. Science of the Total Environment, 676, 792-810.			
**Dasht e-Nemdan Basin**	-	Hosseini, S. M., Parizi, E., Ataie-Ashtiani, B., Simmons, C. T. (2019). Assessment of sustainable groundwater resources management using integrated environmental index: Case studies across Iran. Science of the Total Environment, 676, 792-810.	-	-	Approximated from Fig. 6 by Hosseini et al. (2019)
**Dashtab Basin**	-	Hosseini, S. M., Parizi, E., Ataie-Ashtiani, B., Simmons, C. T. (2019). Assessment of sustainable groundwater resources management using integrated environmental index: Case studies across Iran. Science of the Total Environment, 676, 792-810.	-	-	Approximated from Fig. 6 by Hosseini et al. (2019)
**Dasht-e Rostam Basin**	-	Hosseini, S. M., Parizi, E., Ataie-Ashtiani, B., Simmons, C. T. (2019). Assessment of sustainable groundwater resources management using integrated environmental index: Case studies across Iran. Science of the Total Environment, 676, 792-810.	-	-	Approximated from Fig. 6 by Hosseini et al. (2019)
**Dastgerdan Basin**	-	Hosseini, S. M., Parizi, E., Ataie-Ashtiani, B., Simmons, C. T. (2019). Assessment of sustainable groundwater resources	-	-	Approximated from Fig. 6 by Hosseini et al. (2019)

Aquifer system	Broader aquifer system	Reference A	Reference B	Reference C	Steps to delineate
		management using integrated environmental index: Case studies across Iran. Science of the Total Environment, 676, 792-810.			
**Datong Basin**	-	Guo, H., Wang, Y. (2005). Geochemical characteristics of shallow groundwater in Datong basin, northwestern China. Journal of Geochemical Exploration, 87, 109-120.	Li, J., Wang, Y., Xie, X., Su, C. (2012). Hierarchical cluster analysis of arsenic and fluoride enrichments in groundwater from the Datong basin, Northern China. Journal Of Geochemical Exploration, 118, 77-89. http://dx.doi.org/10.1016/j.gexplo.2012.05.002	Qian, K., Li, J., Xie, X., Wang, Y. (2017). Organic and inorganic colloids impacting total iodine behavior in groundwater from the Datong Basin, China. Science Of The Total Environment, 601, 380-390. http://dx.doi.org/10.1016/j.scitotenv.2017.05.127	Approximated from Fig. 1 by Guo and Wang (2005), Fig. 1 by Li et al. (2012) and Fig. 2 by Qian et al. (2017)
**Dauphin Lake Area**	Manitoba Carbonate Aquifer System	Betcher, R.N. (1986). Groundwater Availability Map Series - Dauphin Lake Area (62-O). Manitoba Department of Natural Resoureces map. Accessed May 5, 2021 from https://www.gov.mb.ca/water/pubs/maps/water/1987_betcher_groundwater_availability_map_series_dauphin_lake.zip	Betcher, R.N. (1988). Groundwater Availability Map Series - Neepawa Area (62-J). Manitoba Department of Natural Resoureces map. Accessed May 5, 2021 from https://www.gov.mb.ca/water/pubs/maps/water/1988_betcher_groundwater_availability_map_series_neepawa.zip	Rutulis, M. (1986). Aquifer maps of southern Manitoba. Manitoba Water Resources Branch map. Accessed May 5, 2021 from https://www.gov.mb.ca/water/pubs/maps/water/1986_rutulis_bedrock_aquifers.pdf	Approximated from Fig. 6 by Betcher (1986), Fig. 6 by Betcher (1988), and map by Rutulis (1986). Groundwater can be saline in northern and southern portions of this area (see map by Rutulis, 1986)
**Dauphine Miocene Molasses Aquifer System**	-	Clauzon, G., Mandier, P., Aguilar, J. P., Delannoy, J. J., Guendon, J. L., Klein, C., Mandier, P., Michaux, J., Rubino, J.-L., Vaudour, J. (1990). Genèse et évolution du piémont néogène subalpin du Bas-Dauphiné. Université d'Aix-Marseille II, 83 pp. Accessed May 1, 2023 via https://hal-insu.archives-	-	-	Approximated from Fig. 2 by Clauzon et al. (1990)

Aquifer system	Broader aquifer system	Reference A	Reference B	Reference C	Steps to delineate
		ouvertes.fr/file/index/docid/459143/filename/Clauzon1990.pdf			
**Dayton Valley**	Carson River Basin	Maurer, D. K. (2011). Geologic framework and hydrogeology of the middle Carson River Basin, Eagle, Dayton, and Churchill Valleys, West-Central Nevada. U. S. Geological Survey Scientific Investigations Report 2011–5055. 74 pp. Accessed March 10, 2021 from https://pubs.usgs.gov/sir/2011/5055/pdf/sir20115055.pdf	-	-	Approximated from Fig. 1 of Maurer et al. (2011).
**Deep Creek Valley**	-	Gardner, P. M., Masbruch, M. D. (2015). Hydrogeologic and geochemical characterization of groundwater resources in Deep Creek Valley and adjacent areas, Juab and Tooele Counties, Utah, and Elko and White Pine Counties, Nevada. US Geological Survey Scientific Investigations Report 2015–5097, 66 pp. Accessed March 10, 2021 from https://pubs.usgs.gov/sir/2015/5097/sir20155097.pdf	-	-	Approximated from Fig. 6 of Gardner and Masbruch (2015)
**Deh Bid Basin**	-	Parizi, E., Hosseini, S. M., Ataie-Ashtiani, B., Simmons, C. T. (2020). Normalized difference vegetation index as the	-	-	Approximated from tabulated coordinates in the Supplementary Information within Parizi et al. (2020) and physiographic and drainage patterns

Aquifer system	Broader aquifer system	Reference A	Reference B	Reference C	Steps to delineate
		dominant predicting factor of groundwater recharge in phreatic aquifers: case studies across Iran. Scientific Reports, 10, 17473.			
**Deh Kahan Basin**	-	Parizi, E., Hosseini, S. M., Ataie-Ashtiani, B., Simmons, C. T. (2020). Normalized difference vegetation index as the dominant predicting factor of groundwater recharge in phreatic aquifers: case studies across Iran. Scientific Reports, 10, 17473.	-	-	Approximated from tabulated coordinates in the Supplementary Information within Parizi et al. (2020) and physiographic and drainage patterns
**Deh Sabz Basin**	Kabul Basin	Mack, T. J., Chornack, M. P., Taher, M. R. (2013). Groundwater-level trends and implications for sustainable water use in the Kabul Basin, Afghanistan. Environment Systems and Decisions, 33, 457-467.	-	-	Approximated from Fig. 1 by Mack et al. (2013)
**Dehkyueh Basin**	-	Hosseini, S. M., Parizi, E., Ataie-Ashtiani, B., Simmons, C. T. (2019). Assessment of sustainable groundwater resources management using integrated environmental index: Case studies across Iran. Science of the Total Environment, 676, 792-810.	-	-	Approximated from Fig. 6 by Hosseini et al. (2019)
**Dehrom Basin**	-	Hosseini, S. M., Parizi, E., Ataie-Ashtiani, B., Simmons, C. T. (2019). Assessment of sustainable groundwater resources	-	-	Approximated from Fig. 6 by Hosseini et al. (2019)

Aquifer system	Broader aquifer system	Reference A	Reference B	Reference C	Steps to delineate
		management using integrated environmental index: Case studies across Iran. Science of the Total Environment, 676, 792-810.			
**Dehrood -e Tangh -e Eram Basin**	-	Hosseini, S. M., Parizi, E., Ataie-Ashtiani, B., Simmons, C. T. (2019). Assessment of sustainable groundwater resources management using integrated environmental index: Case studies across Iran. Science of the Total Environment, 676, 792-810.	-	-	Approximated from Fig. 6 by Hosseini et al. (2019)
**Delmarva Peninsula**	North Atlantic Coastal Plain	Bachman, L.J., Shedlock, R.J., Phillips, P.J. (1987). Ground-water-quality assessment of the Delmarva Peninsula, Delaware, Maryland, and Virginia. U.S. Geological Survey Open-File Report 87-112, Accessed April 1, 2021 from https://pubs.usgs.gov/of/1987/0112/report.pdf	Sanford, W. E., Pope, J. P., Selnick, D. L., Stumvoll, R. F. (2012). Simulation of groundwater flow in the shallow aquifer system of the Delmarva Peninsula, Maryland and Delaware. US Geological Survey Open-File Report 2012–1140, 68 pp. Accessed November 29, 2021 from https://pubs.usgs.gov/of/2012/1140/pdf/OFR_2012-1140.pdf	-	Approximated from Fig. 1 of Bachman et al. (1987) and Fig. 1 by Sanford et al. (2012)
**Delta-Mendota Basin**	San Joaquin Basin of the Central Valley **	California Department of Water Resources (2015h). California's groundwater update 2013 - Chapter 8: San Joaquin River Hydrologic Region. California Department of Water Resources Report, 122 pp. Accessed April 28, 2022 via	-	-	Approximated from Fig. 8-2 by California Department of Water Resources (2015h)

Aquifer system	Broader aquifer system	Reference A	Reference B	Reference C	Steps to delineate
		https://water.ca.gov/-/media/DWR-Website/Web-Pages/Programs/Groundwater-Management/Data-and-Tools/Files/Statewide-Reports/California-Groundwater-Update-2013/California-Groundwater-Update-2013---Chapter-8---San-Joaquin-River.pdf			
**Denver Basin**	-	Ruybal, C.J., Hogue, T.S., McCray, J.E. (2019). Assessment of groundwater depletion and implications for management in the Denver Basin Aquifer System. JAWRA Journal of the American Water Resources Association, 55, 1130-1148.	Malenda, H.F., Penn, C.A. (2020). Groundwater levels in the Denver Basin bedrock aquifers of Douglas County, Colorado, 2011–19. U.S. Geological Survey Scientific Investigations Report 2020–5076, 44 pp., Accessed November 29, 2021 from https://pubs.usgs.gov/sir/2020/5076/sir20205076.pdf	Paschke, S.S. (2011). Groundwater availability of the Denver Basin aquifer system, Colorado. U.S. Geological Survey Professional Paper 1770, 274 pp. Accessed January 21, 2022 from https://pubs.usgs.gov/pp/1770/contents/pp1770.pdf	Approximated from Fig. A4 of Paschke et al. (2011), Fig. 1 by Malenda and Penn (2020), and Fig. 1 by Ruybal et al. (2019)
**Desert Valley in northern Nevada**	-	Lopes, T. J. (2010). Hydrologic Evaluation of the Jungo Area, Southern Desert Valley, Nevada. U. S. Geological Survey Open-File Report 2010-1009, 18 pp. Accessed March 23, 2021 from https://pubs.usgs.gov/of/2010/1009/pdf/ofr20101009.pdf	-	-	Approximated from Fig. 2 of Lopes (2010)
**Detrital Valley Basin**	-	Tillman, F.D, Garner, B.D., Truini, M. (2013). Preliminary groundwater flow model of the basin-fill aquifers in Detrital, Hualapai, and Sacramento Valleys, Mohave County,	-	-	Approximated from Fig. 2 by Tillman et al. (2013).

Aquifer system	Broader aquifer system	Reference A	Reference B	Reference C	Steps to delineate
		northwestern Arizona. U.S. Geological Survey Scientific Investigations Report 2013–5122, 52 pp. http://pubs.usgs.gov/sir/2013/5122/			
**Devonian and Overlying Unconsolidated Aquifers**	Southern Ontario Paleozoic Aquifer System	Sharpe, D.R., Piggott, A., Carter, T., Gerber, R.E., MacRitchie, S.M., de Loë, R.C., Strynatka, S., Zwiers, G., Rivera, A. (2013). Southern Ontario hydrogeological region. in: Canada's Groundwater Resources, pp. 444-499. Fitzhenry and Whiteside Limited, Markham, Ontario, Canada. Accessed May 5, 2021 from https://www.researchgate.net/profile/David-Sharpe-2/publication/284034021_Southern_Ontario_hydrogeological_region/links/5f8f462192851c14bcd85341/Southern-Ontario-hydrogeological-region.pdf	-	-	Approximated from Figs. 12.1 and 12.5 of Sharpe et al. (2013)
**Dezghan Basin**	-	Hosseini, S. M., Parizi, E., Ataie-Ashtiani, B., Simmons, C. T. (2019). Assessment of sustainable groundwater resources management using integrated environmental index: Case studies across Iran. Science of the Total Environment, 676, 792-810.	-	-	Approximated from Fig. 6 by Hosseini et al. (2019)
**Dezhgah-Kordeh Basin**	-	Parizi, E., Hosseini, S. M., Ataie-Ashtiani, B., Simmons, C. T. (2020).	-	-	Approximated from tabulated coordinates in the Supplementary Information within Parizi et al.

Aquifer system	Broader aquifer system	Reference A	Reference B	Reference C	Steps to delineate
		Normalized difference vegetation index as the dominant predicting factor of groundwater recharge in phreatic aquifers: case studies across Iran. Scientific Reports, 10, 17473.			(2020) and physiographic and drainage patterns
**Diamond Valley in northern Nevada**	-	Bredehoeft, J.D., Farvolden, R.N. (1963). International Association of Scientific Hydrology, Commission of Subterranean Waters, Publication no. 64, p. 197–212. Accessed March 23, 2021 from http://hydrologie.org/redbooks/a064/064017.pdf	-	-	Approximated from Fig. 1 of Bredehoft and Farvolden (1963)
**Dindigul Fractured Charnockite and Gneiss Aquifers**	-	Sreenivas, A., Gowtham, B., Vinodh, K., Kumaresan, K. (2020). Aquifer mapping of hard rock terrain in parts of Dindigul district, Tamil Nadu. The International Journal of Analytical and Experimental Modal Analysis, 200-211. Accessed June 25, 2021 from http://www.ijaema.com/gallery/23-ijaema-november-4824.pdf	Saha, D., Shekhar, S., Ali, S., Vittala, S.S., Raju, N.J. (2016). Recent hydrogeological research in India. Proceedings of the Indian National Science Academy, 82, 787-803.	-	Approximated from Figs. 2-7 by Sreenivas et al. (2020) and Fig. 1 by Saha et al. (2016)
**Dixie Valley**	-	Lopes, T.J., Evetts. D.M. (2005). Ground-Water pumpage and artificial recharge estimates for calendar year 2000 and average annual natural recharge and interbasin flow by hydrographic area, Nevada. Accessed April 25,	Harrill, J.R., Prudic, D.E. (1998). Aquifer systems in the Great Basin region of Nevada, Utah, and adjacent states; summary report. US Geological Survey Professional Paper 1409-A, 75 pp. Accessed April 26, 2022 via	-	Approximated from Fig. 1 by Lopes and Evetts (2005), and Fig. 2 by Harrill and Prudic (1998). Well completion records, physiography and drainage were used to delinate areas.

Aquifer system	Broader aquifer system	Reference A	Reference B	Reference C	Steps to delineate
		2022 via https://pubs.usgs.gov/sir/2004/5239/sir2004-5239.pdf	https://pubs.usgs.gov/pp/1409a/report.pdf		
**Djursland Limestone and Sand Aquifer System**	-	Frei, R., Frei, K.M., Kristiansen, S.M., Jessen, S., Schullehner, J., Hansen, B. (2020). The link between surface water and groundwater-based drinking water–strontium isotope spatial distribution patterns and their relationships to Danish sediments. Applied Geochemistry, 121, 104698.	-	-	Approximated from Figs. 1 and 5 by Frei et al. (2020) and drainage patterns
**Don Bonito Region in the Middle Guadiana Basin**	-	Aldaya, M. M., Llamas, M. R. (2008). Water footprint analysis for the Guadiana river basin (Vol. 3). Delft, The Netherlands: UNESCO-IHE, 86 pp. Accessed May 28, 2021 from https://waterfootprint.org/media/downloads/Report35-WaterFootprint-Guadiana.pdf	Esteve, P., Varela-Ortega, C., Blanco-Gutiérrez, I., & Downing, T. E. (2015). A hydro-economic model for the assessment of climate change impacts and adaptation in irrigated agriculture. Ecological Economics, 120, 49-58.	-	Approximated from Fig. 3 by Aldaya and Llamas (2008) and Fig. 1 by Esteve et al. (2015)
**Don River Delta Aquifer**	-	Welsh, W.D. (2005). Spatial and temporal water balance estimates using a GIS. Engineers Australia. 29th Hydrology and Water Resources Symposium, February 21-23, Canberra, Australia, 8 pp.	-	-	Approximated from Fig. 1 by Welsh (2005)
**Donihue-Coinco-Coltauco Aquifer**	-	Direccion General de Aguas (2017). Inventario Nacional de acuiferos. Ministerio De Obras Públicas (Gobierno De Chile) report number 403, 60 pp. Accessed May	-	-	Approximated from map on page 42 by Direccion General de Aguas (2017).

Aquifer system	Broader aquifer system	Reference A	Reference B	Reference C	Steps to delineate
		17, 2021 from https://snia.mop.gob.cl/sad/SUB5748.pdf			
**Dougherty Plain and Marianna Lowlands**	Floridan Aquifer System	Williams, L.J., Kuniansky, E.L. (2016). Revised hydrogeologic framework of the Floridan aquifer system in Florida and parts of Georgia, Alabama, and South Carolina. U.S. Geological Survey Professional Paper 1807, 140 pp. Accessed March 31, 2021 from https://pubs.usgs.gov/pp/1807/pdf/pp1807.pdf	Clark, W. Z., Zisa, A. C. (1976). Physiographic map of Georgia: Georgia Department of Natural Resources, 1 sheet. Accessed March 31, 2021 from https://epd.georgia.gov/document/publication/sm-4-physiographic-map-georgia-12000000-1988/download	-	Approximated from Fig. 21 of Williams and Kuniansky (2016). Georgia portion of area also provided by Clark and Zisa (1976).
**Dry Lake Valley and Dreamer Valley**	-	Hurlow, H. A. (2014). Hydrogeologic studies and groundwater monitoring in Snake Valley and adjacent hydrographic areas, west-central Utah and east-central Nevada (Vol. 135). Utah Geological Survey Bulletin 135, 272 pp.	-	-	Approximated from map on page 6 of Hurlow (2014)
**Dublin Basin**	-	Blake, S., Henry, T., Murray, J., Flood, R., Muller, M. R., Jones, A. G., Rath, V. (2016). Compositional multivariate statistical analysis of thermal groundwater provenance: a hydrogeochemical case study from Ireland. Applied Geochemistry, 75, 171-188.	-	-	Approximated from Fig. 1 by Blake et al. (2016)
**Duck Lake Valley**	-	Lopes, T.J., Evetts. D.M. (2005). Ground-Water pumpage and artificial recharge estimates for	Harrill, J.R., Prudic, D.E. (1998). Aquifer systems in the Great Basin region of Nevada, Utah, and adjacent	-	Approximated from Fig. 1 by Lopes and Evetts (2005), and Fig. 2 by Harrill and Prudic (1998). Well completion records, physiography

Aquifer system	Broader aquifer system	Reference A	Reference B	Reference C	Steps to delineate
		calendar year 2000 and average annual natural recharge and interbasin flow by hydrographic area, Nevada. Accessed April 25, 2022 via https://pubs.usgs.gov/sir/2004/5239/sir2004-5239.pdf	states; summary report. US Geological Survey Professional Paper 1409-A, 75 pp. Accessed April 26, 2022 via https://pubs.usgs.gov/pp/1409a/report.pdf		and drainage were used to delinate areas.
**Dugway-Government Creek Valley**	-	Lopes, T.J., Evetts. D.M. (2005). Ground-Water pumpage and artificial recharge estimates for calendar year 2000 and average annual natural recharge and interbasin flow by hydrographic area, Nevada. Accessed April 25, 2022 via https://pubs.usgs.gov/sir/2004/5239/sir2004-5239.pdf	Harrill, J.R., Prudic, D.E. (1998). Aquifer systems in the Great Basin region of Nevada, Utah, and adjacent states; summary report. US Geological Survey Professional Paper 1409-A, 75 pp. Accessed April 26, 2022 via https://pubs.usgs.gov/pp/1409a/report.pdf	-	Approximated from Fig. 1 by Lopes and Evetts (2005), and Fig. 2 by Harrill and Prudic (1998). Well completion records, physiography and drainage were used to delinate areas.
**Dumak Basin**	-	Hosseini, S. M., Parizi, E., Ataie-Ashtiani, B., Simmons, C. T. (2019). Assessment of sustainable groundwater resources management using integrated environmental index: Case studies across Iran. Science of the Total Environment, 676, 792-810.	-	-	Approximated from Fig. 6 by Hosseini et al. (2019)
**Dumfries Basin Aquifer**	-	Robins, N. S., Ball, D. F. (2006). The Dumfries Basin aquifer. British Geological Survey Research Report RR/06/02, 68 pp. Accessed October 27, 2021 from http://nora.nerc.ac.uk/id/eprint/3685/1/RR06002.pdf	-	-	Approximated from Fig. 1.1 by Robins and Ball (2006).

Aquifer system	Broader aquifer system	Reference A	Reference B	Reference C	Steps to delineate
**Eagle Valley**	Carson River Basin	Maurer, D. K. (2011). Geologic framework and hydrogeology of the middle Carson River Basin, Eagle, Dayton, and Churchill Valleys, West-Central Nevada. U. S. Geological Survey Scientific Investigations Report 2011–5055. 74 pp. Accessed March 10, 2021 from https://pubs.usgs.gov/sir/2011/5055/pdf/sir20115055.pdf	Maurer, D.K., Thodal, C.E. (2000). Quantity and chemical quality of recharge, and updated water budgets, for the basin-fill aquifer in Eagle Valley, western Nevada: U.S. Geological Survey Water-Resources Investigations Report 99-4289, 52 pp. Accessed November 29, 2021 from https://pubs.usgs.gov/wri/1999/4289/report.pdf	-	Approximated from Fig. 1 of Maurer et al. (2011) and Fig. 1 by Maurer and Thodal (2000)
**East Bay Plain**	-	California Department of Water Resources (2015g). California's groundwater update 2013 - Chapter 4: San Francisco Bay Hydrologic Region. California Department of Water Resources Report, 78 pp. Accessed April 27, 2022 via https://cawaterlibrary.net/wp-content/uploads/2017/05/GWU2013_Ch4_SanFranciscoBay_Final.pdf	-	-	Approximated from Fig. 4-2 by California Department of Water Resources (2015g)
**East Contra Basin**	San Joaquin Basin of the Central Valley **	California Department of Water Resources (2015h). California's groundwater update 2013 - Chapter 8: San Joaquin River Hydrologic Region. California Department of Water Resources Report, 122 pp. Accessed April 28, 2022 via https://water.ca.gov/-	-	-	Approximated from Fig. 8-2 by California Department of Water Resources (2015h)

Aquifer system	Broader aquifer system	Reference A	Reference B	Reference C	Steps to delineate
		/media/DWR-Website/Web-Pages/Programs/Groundwater-Management/Data-and-Tools/Files/Statewide-Reports/California-Groundwater-Update-2013/California-Groundwater-Update-2013---Chapter-8---San-Joaquin-River.pdf			
**East Salt River Basin**	Salt Basin	Flora, S., Davis, T. (2009). Hydrologic Map Series (HMS), Water Level Change Map Series (WLCMS), and Basin Sweep Assessment Report ADWR Basins and Sub-Basins. Arizona Department of Water Resources Hydrology Division Field Services Section https://new.azwater.gov/sites/default/files/HMSWLCMSBasinSweepAssessmentReport2009.pdf	Laney, R.L., Hahn, M.E. (1986). Hydrogeology of the eastern part of the Salt River Valley area, Maricopa and Pinal Counties, Arizona. U.S. Geological Survey Water-Resources Investigations Report Water-Resources Investigations Report, 8 maps on 4 sheets. Accessed March 31, 2021 from https://pubs.er.usgs.gov/publication/wri864147	-	Approximated from Fig. 1 of Flora and Davis (2009). Hydrologic Map Series (HMS), Water Level Change Map Series (WLCMS), and Basin Sweep Assessment Report ADWR Basins and Sub-Basins. Arizona Department of Water Resources Hydrology Division Field Services Section https://new.azwater.gov/sites/default/files/HMSWLCMSBasinSweepAssessmentReport2009.pdf. See also: Laney and Hahn (1986).
**East Shore Area**	-	Price, D. (1985). Ground Water in Utah's Densely Populated Wasatch Front Area the Challenge and the Choices. U.S. Geological Survey Water-Supply Paper 2232, 78 pp. Accessed March 27, 2021 from https://pubs.usgs.gov/wsp/2232/report.pdf	-	-	Approximated from Fig. 1 of Price (1985)
**Eastern Amarkantak Plateau**	Amarkantak Plateau	Deolankar, S. B. (1980). The Deccan basalts of Maharashtra, India—their potential as aquifers. Groundwater, 18(5), 434-437.	Kale, V. S., Bodas, M., Chatterjee, P., Pande, K. (2020). Emplacement history and evolution of the Deccan Volcanic Province,	Duraiswami, R. A., Das, S., Shaikh, T. (2012). Hydrogeological framework of aquifers from the Deccan Traps, India: some insights.	Approximated from Fig. 1 by Deolankar (1980), Fig. 1 by Duraiswami et al. (2012), and Fig. 1 by Kale et al. (2016). Subarea based on topography and drainage patterns.

Aquifer system	Broader aquifer system	Reference A	Reference B	Reference C	Steps to delineate
			India. Episodes, 43, 278-299.	Memoir Geological Society of India, 1-15.	
**Eastern Armorican Massif**	Armorican Massif	Rouillard, J., Maréchal, J.-C. (2020). Groundwater in France: Resources, Use and Management Issues. In: Sustainable Groundwater Management A Comparative Analysis of French and Australian Policies and Implications to Other Countries, pp. 17-45. Accessed May 22, 2021 from https://hal.archives-ouvertes.fr/hal-02512154/document	Roques, C., Bour, O., Aquilina, L., Dewandel, B. (2016). High-yielding aquifers in crystalline basement: insights about the role of fault zones, exemplified by Armorican Massif, France. Hydrogeology Journal, 24, 2157-2170.	Vergnes, J.P., Roux, N., Habets, F., Ackerer, P., Amraoui, N., Besson, F., Caballero, Y., Courtois, Q., Dreuzy, J.R.D., Etchevers, P., Gallois, N. (2020). The AquiFR hydrometeorological modelling platform as a tool for improving groundwater resource monitoring over France: evaluation over a 60-year period. Hydrology and Earth System Sciences, 24, 633-654.	Approximated from Fig. 1 by Rouillard and Maréchal (2020), Fig. 3 by Vergnes et al. (2020), and Fig. 1 by Roques et al. (2016).
**Eastern Arroyo Seco Valley**	-	California Department of Water Resources (2015a). California's groundwater update 2013 - Chapter 12: Colorado River Hydrologic Region. California Department of Water Resources Report, 76 pp. Accessed April 27, 2022 via https://water.ca.gov/-/media/DWR-Website/Web-Pages/Programs/Groundwater-Management/Bulletin-118/Files/Statewide-Reports/GWU2013_Ch12_ColoradoRiver_Final.pdf	-	-	Approximated from Fig. 12-2 by California Department of Water Resources (2015a)
**Eastern Bohemian Cretaceous Basin**	Bohemian Cretaceous Basin	Alvarado, J. C., Pačes, T., Purtschert, R. (2013). Dating groundwater in the Bohemian Cretaceous Basin: Understanding tracer variations in the subsurface. Applied Geochemistry, 29, 189-198.	Jiráková, H., Huneau, F., Hrkal, Z., Celle-Jeanton, H., Le Coustumer, P. (2010). Carbon isotopes to constrain the origin and circulation pattern of groundwater in the north-western part of the Bohemian Cretaceous Basin	Jiráková, H., Procházka, M., Dědeček, P., Kobr, M., Hrkal, Z., Huneau, F., Le Coustumer, P. (2011). Geothermal assessment of the deep aquifers of the northwestern part of the Bohemian Cretaceous	Approximated from Fig. 1 by Jiráková et al. (2010), Fig. 1 by Jiráková et al. (2011), and Fig. 1 by Alvarado et al. (2013)

Aquifer system	Broader aquifer system	Reference A	Reference B	Reference C	Steps to delineate
			(Czech Republic). Applied Geochemistry, 25, 1265-1279.	basin, Czech Republic. Geothermics, 40, 112-124.	
**Eastern C Aquifer**	The C Aquifer	Brown, C.R., Macy, J.P. (2012). Groundwater, Surface-Water, and Water-Chemistry Data from the C-Aquifer Monitoring Program, Northeastern Arizona, 2005–2011. U.S. Geological Survey Open-File Report 2012–1196, 46 pp. Accessed April 15, 2021 from https://pubs.usgs.gov/of/2012/1196/of2012-1196.pdf	-	-	Approximated from Fig. 1 of Brown and Macy (2012). East and west portions divided near Joseph City.
**Eastern Cambay Basin**	Cambay Basin	Kumar, M. D., Ghosh, S., Patel, A., Singh, O. P., Ravindranath, R. (2006). Rainwater harvesting in India: some critical issues for basin planning and research. Land Use and Water Resources Research, 6, 1-17.	Deshpande, R.D. (2006). Groundwater in and Around Cambay Basin, Gujarat: Some Geochemical andIsotopic Investigations, Physical Research Laboratory, Ahmedabad, PhD Thesis. Accessed July 12, 2021 from https://shodhganga.inflibnet.ac.in/handle/10603/58203	Gupta, S. K., Deshpande, R. D. (2003). Origin of groundwater helium and temperature anomalies in the Cambay region of Gujarat, India. Chemical Geology, 198, 33-46.	Approximated from Fig. 6 by Kumar et al. (2006), Fig. 4.1 by Deshpande (2006), and Fig. 1 by Gupta and Deshpande (2003). West, Central and East subareas defined by two identified faults (see Fig. 4.1 by Deshpande (2006))
**Eastern Cambrian-Ordovician Aquifers**	Northern Midwest Aquifer System	Young, H.L. (1992). Hydrogeology of the Cambrian-Ordovician Aquifer System in the Northern Midwest, United States. U.S. Geological Survey Professional Paper 1405-B, 108 pp. Accessed April 12, 2021 from https://pubs.usgs.gov/pp/1405b/report.pdf	Wilson, J.T. (2012). Water-quality assessment of the Cambrian-Ordovician aquifer system in the northern Midwest, United States. U.S. Geological Survey Scientific Investigations Report 2011–5229, 174 pp. Accessed April 12, 2021 from https://pubs.usgs.gov/sir/2	-	Broader aquifer system title and its location approximated from Fig. 1 of Young (1992). Subcrop geology approximated from Fig. 9 of Wilson (2012). Southern margin extended south of the subcrop margin on the basis of the presence of deep wells.

Aquifer system	Broader aquifer system	Reference A	Reference B	Reference C	Steps to delineate
			011/5229/pdf/SIR2011522 9_web.pdf		
**Eastern Carrizo-Wilcox**	Carrizo-Wilcox	Huang, Y., Scanlon, B.R., Nicot, J.P., Reedy, R.C., Dutton, A.R., Kelley, V.A., Deeds, N E. (2012). Sources of groundwater pumpage in a layered aquifer system in the Upper Gulf Coastal Plain, USA. Hydrogeology Journal, 20(4), 783-796.	Kelley, V.A., Deeds, N.E., Fryar, D.G., Nicot, J.P. (2004). Groundwater availability models for the Queen City and Sparta aquifers. Contract report to the Texas Water Development Board, Austin, TX, 867 pp. Accessed February 24, 2022 from https://www.twdb.texas.gov/groundwater/models/gam/qcsp/QCSP_Model_Report.pdf?d=29484	Louisiana Department of Environmental Quality (2007). Carrizo-Wilcox Aquifer Summary Report 2007 Aquifer Sampling and Assessment Program (ASSET) Program. Accessed February 11, 2021 from https://deq.louisiana.gov/assets/docs/Water/Triennial_reports/AquiferSummaries_2007-2009/02Carrizo-WilcoxAquiferSummary09.pdf	Approximated from Fig. 2.2 by Kelley et al. (2004). Texas portion approximated from Fig. 1 of Huang et al. (2012). Louisiana portion approximated from Fig. 2-1 of Louisiana Department of Environmental Quality (2007). Western boundary is the western margin of the Texas Groundwater Management Area (GMA) #11 (accessed February 11, 2021 from https://www.twdb.texas.gov/groundwater/management_areas/gma12.asp)
**Eastern Celje Basin**	Celje Basin	Martínez, R., Suárez, I., Zapatero, M.A., Saftic, B., Kolenkovic, I., Car, M., Persoglia, S., Donda, F. (2009). The EU GeoCapacity project—saline aquifers storage capacity in group south countries. Energy Procedia, 1, 2733-2740.	-	-	Approximated from Fig. 4 by Martínez et al. (2009)
**Eastern Champlain Valley Lowlands**	-	Nystrom, E.A. (2006). Ground-water quality in the Lake Champlain Basin, New York, 2004. U.S. Geological Survey Open-File Report 2006-1088, 22 pp. Accessed April 15, 2021 from https://pubs.usgs.gov/of/2006/1088/pdf/Nystrom.OFR2006-1088.pdf	Scott, T.-M., Nystrom, E.A., Reddy, J.E. (2016). Groundwater quality in the Lake Champlain and Susquehanna River basins, New York, 2014. U.S. Geological Survey Open-File Report 2016–1153, 33 pp. Accessed April 15, 2021 from https://pubs.usgs.gov/of/2016/1153/ofr20161153.pdf	-	Approximated from Fig. 2 of Nystrom (2006) and Fig. 1 by Scott et al. (2016).
**Eastern Dakota Aquifer**	-	Leonard, R. B., Signor, D. C., Jorgensen, D. G., Helgesen, J. O. (1983).	Prior, J.C., Boekhoff, J.L., Howes, M.R., Libra, R.D., VanDorpe, P.E. (2003).	Leopold, R. (2008). Groundwater Resource Evaluation of the Lower	Approximated from Fig. 9 of Leonard et al. (1983), page 30 of

Aquifer system	Broader aquifer system	Reference A	Reference B	Reference C	Steps to delineate
		Geohydrology and hydrochemistry of the Dakota Aquifer, central United States. Journal of the American Water Resources Association, 19(6), 903-912.	Iowa's Groundwater Basics. Iowa Department of Natural Resources Report, 92 pp. Accessed November 29, 2021 from https://s-iihr34.iihr.uiowa.edu/publications/uploads/2014-08-24_08-08-21_es-06.pdf	Dakota Aquifer in Northwest Iowa. Iowa Geological and Water Survey Water Resources Investigation Report No. 1B, 37 pp. Accessed April 11, 2022 via http://s-iihr34.iihr.uiowa.edu/publications/uploads/WRI-1b.pdf	Prior et al. (2003), and Fig. 2 by Leopold (2008)
**Eastern Deccan Basalt Plateau**	Deccan Plateau	Deolankar, S. B. (1980). The Deccan basalts of Maharashtra, India—their potential as aquifers. Groundwater, 18(5), 434-437.	Kale, V. S., Bodas, M., Chatterjee, P., Pande, K. (2020). Emplacement history and evolution of the Deccan Volcanic Province, India. Episodes, 43, 278-299.	Duraiswami, R. A., Das, S., Shaikh, T. (2012). Hydrogeological framework of aquifers from the Deccan Traps, India: some insights. Memoir Geological Society of India, 1-15.	Approximated from Fig. 1 by Deolankar (1980), Fig. 1 by Duraiswami et al. (2012), and Fig. 1 by Kale et al. (2016). Subarea based on topography and drainage patterns.
**Eastern Dehshir and Arnan Basins**	-	Hosseini, S. M., Parizi, E., Ataie-Ashtiani, B., Simmons, C. T. (2019). Assessment of sustainable groundwater resources management using integrated environmental index: Case studies across Iran. Science of the Total Environment, 676, 792-810.	-	-	Approximated from Fig. 6 by Hosseini et al. (2019); divide between western and eastern sub basins approximated near (southwest of) کمال آباد
**Eastern Duero Basin**	Duero Basin	Esteban, E., Albiac, J. (2012). The problem of sustainable groundwater management: the case of La Mancha aquifers, Spain. Hydrogeology Journal, 20, 851-863.	-	-	Broader outline of Duero Basin approximated from Fig. 1 by Esteban and Albiac (2012), guided by the presence of recorded well completion events and delineating contributing areas upstream of Zamora.
**Eastern El Palmar Aquifer**	-	CONAGUA (2021a). Acuíferos (nacional). Webpage accessed March 8, 2022 from http://sina.conagua.gob.mx/sina/tema.php?tema=acuif	-	-	Approximated from aquifer boundaries defined by CONAGUA (2021a) web portal, physiography and drainage patterns, and well completion data. Western and Eastern areas of El Palmar divided

Aquifer system	Broader aquifer system	Reference A	Reference B	Reference C	Steps to delineate
		eros&ver=mapa&o=1&n=nacional			on the basis of physiography and drainage.
**Eastern Elsinore Valley**	-	California Department of Water Resources (2015e). California's groundwater update 2013 - Chapter 6: South Coast Hydrologic Region. California Department of Water Resources Report, 84 pp. Accessed April 27, 2022 via https://cawaterlibrary.net/wp-content/uploads/2017/05/GWU2013_Ch6_SouthCoast_Final.pdf	-	-	Approximated from Fig. 6-2 by California Department of Water Resources (2015e)
**Eastern Eromanga Basin**	Great Artesian Basin	Ransley, T.R., Radke, B.M., Feitz, A.J., Kellett, J.R., Owens, R., Bell, J. and Stewart, G. (2015). Hydrogeological Atlas of the Great Artesian Basin, Geoscience Australia. Canberra, 141 pp. Accessed July 19, 2021 from http://www.ga.gov.au/scientific-topics/water/groundwater/gab	-	-	Approximated from Map 1 on page 9 of report by Ransley et al. (2015)
**Eastern Fakhrabad Basin**	-	Parizi, E., Hosseini, S. M., Ataie-Ashtiani, B., Simmons, C. T. (2020). Normalized difference vegetation index as the dominant predicting factor of groundwater recharge in phreatic aquifers: case studies across Iran. Scientific Reports, 10, 17473.	-	-	Approximated from tabulated coordinates in the Supplementary Information within Parizi et al. (2020) and physiographic and drainage patterns

Aquifer system	Broader aquifer system	Reference A	Reference B	Reference C	Steps to delineate
**Eastern Flatwoods Northshores**	Floridan Aquifer System	Williams, L.J., Kuniansky, E.L. (2016). Revised hydrogeologic framework of the Floridan aquifer system in Florida and parts of Georgia, Alabama, and South Carolina. U.S. Geological Survey Professional Paper 1807, 140 pp. Accessed March 31, 2021 from https://pubs.usgs.gov/pp/1807/pdf/pp1807.pdf	Brooks, H. K. (1981). Physiographic divisions of Florida. Report for the Florida Cooperative Extension Service Institute of Food and Agricultural Sciences, University of Florida, 12 pp.	-	Approximated from physiographic provinces of Florida outlined by Brooks (1981). Broader Florida Aquifer System from Williams and Kuniansky (2016). Eastern Flatwoods as defined by Brooks (1981) was split at Lake Harney into two areas north and south of Lake Harney ('northshores' and 'southshores').
**Eastern Flatwoods Southshores**	Floridan Aquifer System	Williams, L.J., Kuniansky, E.L. (2016). Revised hydrogeologic framework of the Floridan aquifer system in Florida and parts of Georgia, Alabama, and South Carolina. U.S. Geological Survey Professional Paper 1807, 140 pp. Accessed March 31, 2021 from https://pubs.usgs.gov/pp/1807/pdf/pp1807.pdf	Brooks, H. K. (1981). Physiographic divisions of Florida. Report for the Florida Cooperative Extension Service Institute of Food and Agricultural Sciences, University of Florida, 12 pp.	-	Approximated from physiographic provinces of Florida outlined by Brooks (1981). Broader Florida Aquifer System from Williams and Kuniansky (2016). Eastern Flatwoods as defined by Brooks (1981) was split at Lake Harney into two areas north and south of Lake Harney ('northshores' and 'southshores').
**Eastern Ghavbandi Basin**	-	Hosseini, S. M., Parizi, E., Ataie-Ashtiani, B., Simmons, C. T. (2019). Assessment of sustainable groundwater resources management using integrated environmental index: Case studies across Iran. Science of the Total Environment, 676, 792-810.	-	-	Approximated from Fig. 6 by Hosseini et al. (2019)
**Eastern Gippsland Basin**	Gippsland Basin	Currell, M., Banfield, D., Cartwright, I., Cendón, D. I. (2017). Geochemical indicators of the origins and	Varma, S., Michael, K. (2012). Impact of multi-purpose aquifer utilisation on a variable-density	Beverly, C., Hocking, M., Cheng, X., O'Neil, C., Schroers, R., Baker, S. (2015).The Gippsland	Approximated from Fig. 4 by Beverly et al. (2015), Fig. 1 by Varma and Michael (2012), and Fig. 1 by Currell et al. (2017)

Aquifer system	Broader aquifer system	Reference A	Reference B	Reference C	Steps to delineate
		evolution of methane in groundwater: Gippsland Basin, Australia. Environmental Science and Pollution Research, 24, 13168-13183.	groundwater flow system in the Gippsland Basin, Australia. Hydrogeology Journal, 20, 119-134.	groundwater model. Technical Report, 409 pp. Accessed October 8, 2021 from https://www.parliament.vic.gov.au/images/stories/committees/EPC/Other_documents/G3_-_Gippsland_groundwater_model_report_June_2015_2.pdf	
**Eastern Goltapeh Zarin Abad Basin**	-	Hosseini, S. M., Parizi, E., Ataie-Ashtiani, B., Simmons, C. T. (2019). Assessment of sustainable groundwater resources management using integrated environmental index: Case studies across Iran. Science of the Total Environment, 676, 792-810.	-	-	Approximated from Fig. 6 by Hosseini et al. (2019)
**Eastern Guadiana Menor Basin**	-	Fernández-Chacón, F., Benavente, J., Rubio-Campos, J.C., Kohfahl, C., Jiménez, J., Meyer, H., Hubberten, H., Pekdeger, A. (2010). Isotopic composition (δ18O and δD) of precipitation and groundwater in a semi-arid, mountainous area (Guadiana Menor basin, Southeast Spain). Hydrological Processes: An International Journal, 24, 1343-1356.	-	-	Approximated from Figure 1. by Fernández-Chacón, F., Benavente, J., Rubio-Campos, J. C., Kohfahl, C., Jiménez, J., Meyer, H., ... & Pekdeger, A. (2010). Isotopic composition (δ18O and δD) of precipitation and groundwater in a semi-arid, mountainous area (Guadiana Menor basin, Southeast Spain). Hydrological Processes: An International Journal, 24(10), 1343-1356.
**Eastern Highland Rim**	Interior Low Plateaus	Miller, J.A. (1995). Ground Water Atlas of the United States: Segment 10, Illinois, Indiana, Kentucky,	Brahana, J.V., Bradley, M.W. (1986b). Preliminary delineation and description of the regional aquifers of	Taylor, C.J., Nelson Jr., H.L. (2008). A compilation of provisional karst geospatial data for the Interior Low	Broader Interior Low Plateaus outline approximated for Tennessee, Illinois, Kentucky, Indiana from Fig. 78 of Miller

Aquifer system	Broader aquifer system	Reference A	Reference B	Reference C	Steps to delineate
		Ohio, Tennessee. U.S. Geological Survey Hydrologic Atlas 730-K, 30 pp. Accessed April 11, 2021 from https://pubs.usgs.gov/ha/730k/report.pdf	Tennessee--The Highland Rim Aquifer System. Water-Resources Investigations Report 82-4054, 35 pp. Accessed April 11, 2021 from https://pubs.usgs.gov/wri/wri824054/pdf/wrir_82-4054_a.pdf	Plateaus physiographic region, central United States: U.S. Geological Survey Data Series 339, 26 pp. Accessed April 11, 2021 from https://pubs.usgs.gov/ds/339/pdf/ds339_web.pdf	(1990) and Fig. 1 of Taylor and Nelson (2008). Highland Rim subarea approximated from Fig. 1 of Taylor and Nelson (2008) and Fig. 1 of Brahana and Bradley (1986b)
**Eastern Huaihe Plain**	Huaihe River Basin	Sun, X., Lin, J., Gu, W., Min, X., Han, J., Dai, Y., Liu, P., Wang, S (2021). Analysis and evaluation of the renewability of the deep groundwater in the Huaihe River Basin, China. Environmental Earth Sciences, 80, 1-13.	Liu J., Zheng C. (2016) Towards Integrated Groundwater Management in China. In: Integrated Groundwater Management (eds. Jakeman A.J., Barreteau O., Hunt R.J., Rinaudo J.D., Ross A.). Springer, Cham. pp. 455-475	Li, Y., Wang, D., Liu, Y., Zheng, Q., Sun, G. (2017). A predictive risk model of groundwater arsenic contamination in China applied to the Huai River Basin, with a focus on the region's cluster of elevated cancer mortalities. Applied Geochemistry, 77, 178-183.	Approximated from Fig. 1 by Sun et al. (2021), Fig. 1 by Li et al. (2017), and Fig. 18.5 by Liu and Zheng (2016)
**Eastern Imperial and Amos and Oligby Valleys**	-	California Department of Water Resources (2015a). California's groundwater update 2013 - Chapter 12: Colorado River Hydrologic Region. California Department of Water Resources Report, 76 pp. Accessed April 27, 2022 via https://water.ca.gov/-/media/DWR-Website/Web-Pages/Programs/Groundwater-Management/Bulletin-118/Files/Statewide-Reports/GWU2013_Ch12_ColoradoRiver_Final.pdf	-	-	Approximated from Fig. 12-2 by California Department of Water Resources (2015a)
**Eastern Isonzo-Soca High Plain**	Isonzo-Soca High Plain	Treu, F., Zini, L., Zavagno, E., Biolchi, S., Boccali, C., Gregorič, A., Napolitano, R., Urbanc, J., Zuecco, G., Cucchi, F. (2017). Intrinsic	-	-	Approximated from Fig. 1 by Treu et al. (2017); western boundary approximated along the Isonzo River

Aquifer system	Broader aquifer system	Reference A	Reference B	Reference C	Steps to delineate
		vulnerability of the Isonzo/Soča high plain aquifer (NE Italy–W Slovenia). Journal of Maps, 13, 799-810.			
**Eastern Kachchh Coastal Plain**	Kachchh Coastal Plain	Saha, D., Gor, N. (2020). A prolific aquifer system is in peril in arid Kachchh region of India. Groundwater for Sustainable Development, 11, 100394.	Bhimani, S.A. (2013). Study on Groundwater Salinization and Formulation of Management Strategies for the Coastal Aquifers of Mundra Region, Kutch District, Gujarat State. Ph.D. Thesis, The Maharaja Sayajirao University of Baroda. 380 pp. Accessed July 5, 2021 from http://okgw.hmlibrary.ac.in:8080/jspui/handle/1/457	University of Greenwich and Gujarat Institute of Desert Ecology (2016). Ecosystem Assessment of the Coastal Plain Natural Area of Kachchh District: Planning for Biodiversity and Livelihoods into the Future. Project Presentation, 70 pp. Accessed July 5, 2021 from https://gala.gre.ac.uk/id/eprint/16221/1/16221%20BARTLETT_Coastal_Plain_of_Kachchh_2016.pdf	Approximated from Fig. 1 by Saha and Gor (2020) and Fig. 3.2 and descriptions of hydrogeologic setting in Chapter III by Bhimani (2013). Extent of broader aquifer system (region) approximated following the "Natural Character Area map" (page 4) by University of Greenwich and Gujarat Institute of Desert Ecology (2016)
**Eastern Kankakee Plains**	-	Fenneman, N.M., Johnson, D.W. (1946). Physiographic divisions of the conterminous United States. U.S. Geological Survey map, 1:7,000,000 Scale.	Leighton, M. M., Ekblaw, G. E., Horberg, L. (1948). Physiographic divisions of Illinois. The Journal of Geology, 56, 16-33.	Gray, H.H. (2001). Map of Indiana showing physiographic divisions. Indiana Geological Survey Miscellaneous Map 69, 15 pp. Accessed April 12, 2021 from https://igws.indiana.edu/ReferenceDocs/Maps/PhysiographicRegions.pdf	Broader Till Plain physiographic region approximated from Fenneman and Johnson (1946). Kankakee Plain in Illinois approximated from Figs. 1 and 2 of Leighton et al. (1948); Indiana portion of subarea approximated from Gray (2001). Western margin of subarea approximated at northwest margin of the Iroquois Till Plains (located to the southwest of this subarea)
**Eastern Karoo Basin**	Main Karoo Basin	Nyambe, I. A. (1999). Tectonic and climatic controls on sedimentation during deposition of the Sinakumbe Group and Karoo Supergroup, in the mid-Zambezi Valley Basin, southern Zambia. Journal of African Earth Sciences, 28, 443-463.	-	-	Broader aquifer system boundary (i.e., Main Karoo Basin) approximated from Fig. 1 by Nyambe (1999). Physiography (drainage patterns, geology) and well construction data were considered while digitizing subarea

Aquifer system	Broader aquifer system	Reference A	Reference B	Reference C	Steps to delineate
**Eastern Khash Basin**	-	Hosseini, S. M., Parizi, E., Ataie-Ashtiani, B., Simmons, C. T. (2019). Assessment of sustainable groundwater resources management using integrated environmental index: Case studies across Iran. Science of the Total Environment, 676, 792-810.	-	-	Approximated from Fig. 6 by Hosseini et al. (2019)
**Eastern Kome Basin**	Kome Basin	Parizi, E., Hosseini, S. M., Ataie-Ashtiani, B., Simmons, C. T. (2020). Normalized difference vegetation index as the dominant predicting factor of groundwater recharge in phreatic aquifers: case studies across Iran. Scientific Reports, 10, 17473.	-	-	Approximated from tabulated coordinates in the Supplementary Information within Parizi et al. (2020) and physiographic and drainage patterns
**Eastern La Sabana Aquifer**	-	CONAGUA (2021a). Acuíferos (nacional). Webpage accessed March 8, 2022 from http://sina.conagua.gob.mx/sina/tema.php?tema=acuiferos&ver=mapa&o=1&n=nacional	-	-	Approximated from aquifer boundaries defined by CONAGUA (2021a) web portal, physiography and drainage patterns, and well completion data. Western and eastern areas divided on the basis of drainage (i.e., divided at Laguna Tres Palos)
**Eastern Libres-Oriental Aquifer**	Libres-Oriental Aquifer	CONAGUA (2020hf). Actualización de la disponibilidad media anual de agua en el acuífero Libres-Oriental (2102), estado de Puebla. 14 pp. Accessed April 25, 2022 via https://sigagis.conagua.gob.mx/gas1/Edos_Acuiferos_18/puebla/DR_2102.pdf	-	-	Approximated from physiography, well completion data, and Fig. 1 by CONAGUA (2020hf)

Aquifer system	Broader aquifer system	Reference A	Reference B	Reference C	Steps to delineate
**Eastern Lordegan Basin**	Lordegan Basin	Parizi, E., Hosseini, S. M., Ataie-Ashtiani, B., Simmons, C. T. (2020). Normalized difference vegetation index as the dominant predicting factor of groundwater recharge in phreatic aquifers: case studies across Iran. Scientific Reports, 10, 17473.	-	-	Approximated from tabulated coordinates in the Supplementary Information within Parizi et al. (2020) and physiographic and drainage patterns
**Eastern Malwa Plateau**	Malwa Plateau	Deolankar, S. B. (1980). The Deccan basalts of Maharashtra, India—their potential as aquifers. Groundwater, 18(5), 434-437.	Kale, V. S., Bodas, M., Chatterjee, P., Pande, K. (2020). Emplacement history and evolution of the Deccan Volcanic Province, India. Episodes, 43, 278-299.	Duraiswami, R. A., Das, S., Shaikh, T. (2012). Hydrogeological framework of aquifers from the Deccan Traps, India: some insights. Memoir Geological Society of India, 1-15.	Approximated from Fig. 1 by Deolankar (1980), Fig. 1 by Duraiswami et al. (2012), and Fig. 1 by Kale et al. (2016). Subarea based on topography and drainage patterns.
**Eastern Malwa Plateau Alluvial Aquifer**	Malwa Plateau	Bhuiyan, C., Singh, R. P., Flügel, W. A. (2009). Modelling of ground water recharge-potential in the hard-rock Aravalli terrain, India: a GIS approach. Environmental Earth Sciences, 59(4), 929-938.	Central Ground Water Board (2012d). Aquifer Systems of India. Central Ground Water Board Report, 103 pp. Accessed July 12, 2021 from http://cgwb.gov.in/AQM/India.pdf	-	Approximated from Plate III by Central Ground Water Board (2012d) and Fig. 1 by Bhuiyan et al. (2009)
**Eastern Mekong Delta**	Mekong Delta	Gunnink, J. L., Pham, H. V., Oude Essink, G. H., Bierkens, M. F. (2021). The three-dimensional groundwater salinity distribution and fresh groundwater volumes in the Mekong Delta, Vietnam, inferred from geostatistical analyses. Earth System Science Data, 13, 3297-3319.	Ha, Q. K., Ngoc, T. D. T., Le Vo, P., Nguyen, H. Q., Dang, D. H. (2022). Groundwater in Southern Vietnam: Understanding geochemical processes to better preserve the critical water resource. Science of The Total Environment, 807, 151345.	Tran, D.A., Tsujimura, M., Loc, H.H., Dang, D.H., Le Vo, P., Ha, D.T., Trang, N.T.T., Thuc, P.T.B., Dang, T.D., Batdelger, O., Nguyen, T.V. (2021). Groundwater quality evaluation and health risk assessment in coastal lowland areas of the Mekong Delta, Vietnam. Groundwater for Sustainable Development, 15, 100679.	Approximated from Fig. 1 by Ha et al. (2022), Fig. 1 by Gunnink et al. (2021), and Fig. 1 by Tran et al. (2021). Western Boundary approximated by the Mekong River.

Aquifer system	Broader aquifer system	Reference A	Reference B	Reference C	Steps to delineate
**Eastern Mississippi Embayment**	Mississippi Embayment	Arthur, J.K., Taylor, R.E. (1998). Ground-water flow analysis of the Mississippi embayment aquifer system, South-Central United States. US Geological Survey Professional Paper 1416-1, 59 pp. Accessed February 10, 2021 from https://pubs.usgs.gov/pp/1416i/report.pdf	Renken, R.A. (1998). Groundwater Atlas of the United States: Arkansas, Louisiana, Mississippi (HA-730-F). US Geological Survey Hydrologic Investigations Atlas 730-F. 30 pp. Accessed November 28, 2021 fromhttps://pubs.usgs.gov/ha/730f/report.pdf	-	Approximated from Fig. 2 of Arthur and Taylor (1998) and Figs. 8-10 and Fig. 65 by Renken (1998)
**Eastern Neyshabur Plain**	Neyshabur Plain	Bhunia, G. S., Keshavarzi, A., Shit, P. K., Omran, E. S. E., Bagherzadeh, A. (2018). Evaluation of groundwater quality and its suitability for drinking and irrigation using GIS and geostatistics techniques in semiarid region of Neyshabur, Iran. Applied Water Science, 8, 1-16.	Khalili Naft Chali, A., Shahidi, A. (2017). Comparison of Lazy Algorithms and M5 Model to Estimate Groundwater Level (Case Study: Plain Neyshabur). JWSS-Isfahan University of Technology, 21, 15-26. Accessed June 2, 2021 from https://jstnar.iut.ac.ir/article-1-3296-en.html	Izady, A., Davary, K., Alizadeh, A., Ghahraman, B., Sadeghi, M., Moghaddamnia, A. (2012). Application of "panel-data" modeling to predict groundwater levels in the Neishaboor Plain, Iran. Hydrogeology Journal, 20, 435-447.	Approximated from Fig. 1 by Bhunia et al. (2018), Fig. 1 by Izady et al. (2012), and Fig. 1 by Khalili Naft Chali and Shahidi (2017)
**Eastern Otway Basin**	Otway Basin	Barnett, S., Simmons, C. T., Nelson, R. (2021). Groundwater resources in Australia—their occurrence, management, and future challenges. In: Global Groundwater Source, Scarcity, Sustainability, Security, and Solutions (eds. Mukherjee, A., Scanlon, B.R., Aureli, A., Langan, S., Guo, H., McKenzie, A.), Elsevier, pp. 35-46.	-	-	Approximated from Fig. 1 by Barnett et al. (2021). Divide between Central and Eastern Otway Basins approximated along the Hopkins River and Salt Creek
**Eastern Parecis Basin**	Parecis Basin	Fontes, S. L., Meju, M. A., Maurya, V. P., La Terra, E. F., Miquelutti, L. G. (2019). Deep structure of Parecis	-	-	Approximated from Fig. 1 by Fontes et al. (2019).

Aquifer system	Broader aquifer system	Reference A	Reference B	Reference C	Steps to delineate
		Basin, Brazil from 3D magnetotelluric imaging. Journal of South American Earth Sciences, 96, 102381.			
**Eastern Paris Basin**	Paris Basin	Beaudoin, N., Gasparrini, M., David, M. E., Lacombe, O., Koehn, D. (2019). Bedding-parallel stylolites as a tool to unravel maximum burial depth in sedimentary basins: Application to Middle Jurassic carbonate reservoirs in the Paris basin, France. GSA Bulletin, 131, 1239-1254.	Contoux, C., Violette, S., Vivona, R., Goblet, P., Patriarche, D. (2013). How basin model results enable the study of multi-layer aquifer response to pumping: the Paris Basin, France. Hydrogeology Journal, 21, 545-557.	Beccaletto, L., Hanot, F., Serrano, O., Marc, S. (2011). Overview of the subsurface structural pattern of the Paris Basin (France): Insights from the reprocessing and interpretation of regional seismic lines. Marine and Petroleum Geology, 28, 861-879.	Approximated from Fig. 1 by Beaudoin et al. (2019), Fig. 1 by Contoux et al. (2013), and Fig. 1 by Beccaletto et al. (2011)
**Eastern Pennyroyal Plateau**	Interior Low Plateaus	Miller, J.A. (1995). Ground Water Atlas of the United States: Segment 10, Illinois, Indiana, Kentucky, Ohio, Tennessee. U.S. Geological Survey Hydrologic Atlas 730-K, 30 pp. Accessed April 11, 2021 from https://pubs.usgs.gov/ha/730k/report.pdf	Taylor, C.J., Nelson Jr., H.L. (2008). A compilation of provisional karst geospatial data for the Interior Low Plateaus physiographic region, central United States: U.S. Geological Survey Data Series 339, 26 pp. Accessed April 11, 2021 from https://pubs.usgs.gov/ds/339/pdf/ds339_web.pdf	Davidson, B. (2018). Kentucky Interagency Groundwater Monitoring Network: Annual Report July 2017–June 2018. 28 pp. Accessed April 11, 2021 from http://www.uky.edu/KGS/water/gnet/itac17-18.pdf	Broader Interior Low Plateaus outline approximated for Tennessee, Illinois, Kentucky, Indiana from Fig. 78 of Miller (1990) and Fig. 1 of Taylor and Nelson (2008). Pennyroyal Plain approximated from Fig. 1 of Davidson (2018); northern limit approximated along the Salt River and Ohio River.
**Eastern Pilbara Granite-Greenstone Terrane**	Pilbara Craton	Rojas, R., Commander, P., McFarlane, D., Ali, R., Dawes, W., Barron, O., Hodgson, G., Charles, S.(2018). Groundwater resource assessment and conceptualization in the Pilbara Region, Western Australia. Earth Systems and Environment, 2(2), 345-365.	-	-	Approximated from Fig. 3 by Rojas et al. (2018). Subareas delineated on the basis of the Shaw and Yule Rivers.

Aquifer system	Broader aquifer system	Reference A	Reference B	Reference C	Steps to delineate
**Eastern Plain**	Eastern Snake River Plain	Graham, W.G., Campbell, L.J. (1981). Groundwater resources of Idaho. Idaho Department of Water Resources Report, 61 pp. Accessed March 23, 2021 from https://idwr.idaho.gov/files/publications/198108-MISC-GW-Resources-ID.pdf	-	-	Approximated from Plate 1 of Graham and Campbell (1981) (see #39 "Snake Plain"; Volcanic Rift Zone" area (see preceding aquifer in this list) excluded from delineation)
**Eastern Poti and Piaui and Cabecas and Serra Grande Aquifers**	Parnaiba Basin	Feitosa, F. A., Diniz, J. A. O., Kirchheim, R. E., Kiang, C. H., Feitosa, E. C. (2016). Assessment of Groundwater Resources in Brazil: Current Status of Knowledge. in: Groundwater Assessment, Modeling, and Management (eds. Thangarajan, M., Singh, V.P.), pp. 33-57.	Ferreira, A.L. (2018). Parnaiba Basin. Presentation at "Round 15 - Brazil: Oil and Gas Concessions". Accessed May 22, 2021 from http://www.anp.gov.br/images/Palestras/Seminario_tecnico_R15_P4/Ingles/06_Bacia_do_Parnaiba_R15_INGLES.pdf	Marques, R.M. (2011). Bacia do Parnaíba: estado atual do conhecimento e possibilidades para a produção de gás natural. Thesis Faculdade de Geologia da Universidade Federal do Pará, Belem. 91 pp. Accessed May 22, 2021 from https://bdm.ufpa.br:8443/jspui/bitstream/prefix/1809/1/TCC_BaciaParnaibaEstado.pdf	Approximated from Fig. 3.5 by Feitosa et al. (2016), Slide 13 by Ferreira (2018) and Fig. 1 by Marques (2011).
**Eastern Prince Edward Island**	Prince Edward Island	Stapinsky, M. Michaud, Y., Morin, R.H., Butler, K.E., Deblonde, C., Chi, G, Thériault, T., Castonguay, S., Boisvert, V., Julien, H.P., Conohan, D., Hulsman, B., Carrier, C. Marion, J., Boisvert, É. (2002). Groundwater resources assessment in the Carboniferous Maritimes Basin: preliminary results of the hydrogeological characterization, New Brunswick, Nova Scotia, and Prince Edward Island.	-	-	Approximated from Figs. 1 and 3 of Stapinsky et al. (2002).

Aquifer system	Broader aquifer system	Reference A	Reference B	Reference C	Steps to delineate
		Geological Survey of Canada Current Research Report 2002-D8, 14 pp. Accessed May 5, 2021 from http://www.gov.pe.ca/photos/original/cle_WA10.pdf			
**Eastern Qaderabad Basin**	-	Hosseini, S. M., Parizi, E., Ataie-Ashtiani, B., Simmons, C. T. (2019). Assessment of sustainable groundwater resources management using integrated environmental index: Case studies across Iran. Science of the Total Environment, 676, 792-810.	-	-	Approximated from Fig. 6 by Hosseini et al. (2019)
**Eastern Quibas Aquifer**	-	Lambán, L. J., Aragón, R. (2004). Influence of intensive exploitation on groundwater salinity: the Quibas hydrogeological unit (Murcia, SE Spain). In Groundwater and saline intrusion. Selected papers from the 18th Salt Water Intrusion Meeting, Cartagena, Spain (Araguás, L (pp. 551-563).	-	-	Approximated from Fig. 1 by Lambán and Aragón (2004).
**Eastern Rezvan Basin**	-	Hosseini, S. M., Parizi, E., Ataie-Ashtiani, B., Simmons, C. T. (2019). Assessment of sustainable groundwater resources management using integrated environmental index: Case studies across Iran. Science of the Total Environment, 676, 792-810.	-	-	Approximated from Fig. 6 by Hosseini et al. (2019)

Aquifer system	Broader aquifer system	Reference A	Reference B	Reference C	Steps to delineate
**Eastern Riverine Plains**	Murray Basin	Herczeg, A. L., Dogramaci, S. S., Leaney, F. W. J. (2001). Origin of dissolved salts in a large, semi-arid groundwater system: Murray Basin, Australia. Marine and Freshwater Research, 52, 41-52.	Cartwright, I., Weaver, T. R., Cendón, D. I., Fifield, L. K., Tweed, S. O., Petrides, B., Swane, I. (2012). Constraining groundwater flow, residence times, inter-aquifer mixing, and aquifer properties using environmental isotopes in the southeast Murray Basin, Australia. Applied Geochemistry, 27, 1698-1709.	Murray-Darling Basin Commission (1999). Murray-Darling Basin Groundwater - a resource for the future, Murray-Darling Basin Commission, Canberra. Accessed July 21, 2021 from https://www.mdba.gov.au/sites/default/files/archived/mdbc-GW-reports/2173_GW_a_resource_for_the_future.pdf	Approximated from Fig. 1 by Herczeg et al. (2001), Fig. 1 by Cartwright et al. (2012), and Fig. 6 by Murray-Darling Basin Commission (1999)
**Eastern Salton Sea**	-	California Department of Water Resources (2015a). California's groundwater update 2013 - Chapter 12: Colorado River Hydrologic Region. California Department of Water Resources Report, 76 pp. Accessed April 27, 2022 via https://water.ca.gov/-/media/DWR-Website/Web-Pages/Programs/Groundwater-Management/Bulletin-118/Files/Statewide-Reports/GWU2013_Ch12_ColoradoRiver_Final.pdf	-	-	Approximated from Fig. 12-2 by California Department of Water Resources (2015a)
**Eastern Salvadoran Coastal Plain**	Salvadoran Coastal Plain	Marshall, J.S. (2007). The geomorphology and physiographic provinces of Central America. Central America: geology, resources and hazards, 1, 75-121.	US Army Corps of Engineers (1998). Water resources assessment of El Salvador. 71 pp. Accessed February 7, 2023 via https://www.sam.usace.army.mil/Portals/46/docs/military/engineering/docs/WRA/ElSalvador/El%20Salvador%20WRA%20English.pdf	Ministerio de Medio Ambiente y Recursos Natural (2023). Mapa Hidrogeológico de El Salvador. Atlas accessed February 7, 2023 via http://srt.snet.gob.sv/sihi/public/atlas	Approximated from Fig. 3.5 by Marshall (2007) and Fig. A-2 within US Army Corps of Engineers (1998) and atlas by Ministerio de Medio Ambiente y Recursos Natural (2023)

Aquifer system	Broader aquifer system	Reference A	Reference B	Reference C	Steps to delineate
**Eastern San Gabriel Valley**	San Gabriel Valley	California Department of Water Resources (2015e). California's groundwater update 2013 - Chapter 6: South Coast Hydrologic Region. California Department of Water Resources Report, 84 pp. Accessed April 27, 2022 via https://cawaterlibrary.net/wp-content/uploads/2017/05/GWU2013_Ch6_SouthCoast_Final.pdf	-	-	Approximated from Fig. 6-2 by California Department of Water Resources (2015e). Western and eastern subareas delineated by the San Gabriel River
**Eastern San Joaquin Basin**	San Joaquin Basin of the Central Valley **	California Department of Water Resources (2015h). California's groundwater update 2013 - Chapter 8: San Joaquin River Hydrologic Region. California Department of Water Resources Report, 122 pp. Accessed April 28, 2022 via https://water.ca.gov/-/media/DWR-Website/Web-Pages/Programs/Groundwater-Management/Data-and-Tools/Files/Statewide-Reports/California-Groundwater-Update-2013/California-Groundwater-Update-2013---Chapter-8---San-Joaquin-River.pdf	-	-	Approximated from Fig. 8-2 by California Department of Water Resources (2015h)
**Eastern Santa Clara River Valley**	-	California Department of Water Resources (2015e). California's groundwater update 2013 - Chapter 6: South Coast Hydrologic Region. California	-	-	Approximated from Fig. 6-2 by California Department of Water Resources (2015e)

Aquifer system	Broader aquifer system	Reference A	Reference B	Reference C	Steps to delineate
		Department of Water Resources Report, 84 pp. Accessed April 27, 2022 via https://cawaterlibrary.net/wp-content/uploads/2017/05/GWU2013_Ch6_SouthCoast_Final.pdf			
**Eastern Saq Aquifer**	Ram-Saq Aquifer System	Sharaf, M. A., Hussein, M. T. (1996). Groundwater quality in the Saq aquifer, Saudi Arabia. Hydrological Sciences Journal, 41, 683-696.	Othman, A., Abotalib, A. Z. (2019). Land subsidence triggered by groundwater withdrawal under hyper-arid conditions: case study from Central Saudi Arabia. Environmental Earth Sciences, 78, 243.	Ghazaw, Y. M., Ghumman, A. R., Al-Salamah, I., Khan, Q. U. (2014). Investigations of impact of recharge wells on groundwater in Buraydah by numerical modeling. Arabian Journal for Science and Engineering, 39, 713-724.	Approximated from Fig. 1 by Sharaf and Hussein (1996), Fig. 1 by Othman and Abotalib (2019) and Fig. 1 by Ghazaw et al. (2014)
**Eastern Satpura Basalt Aquifers**	Satpura Basalts	Deolankar, S. B. (1980). The Deccan basalts of Maharashtra, India—their potential as aquifers. Groundwater, 18(5), 434-437.	Kale, V. S., Bodas, M., Chatterjee, P., Pande, K. (2020). Emplacement history and evolution of the Deccan Volcanic Province, India. Episodes, 43, 278-299.	Duraiswami, R. A., Das, S., Shaikh, T. (2012). Hydrogeological framework of aquifers from the Deccan Traps, India: some insights. Memoir Geological Society of India, 1-15.	Approximated from Fig. 1 by Deolankar (1980), Fig. 1 by Duraiswami et al. (2012), and Fig. 1 by Kale et al. (2016).
**Eastern Second Songhuajiang System**	Songnen Plain	Chen, Z., Wei, W., Liu, J., Wang, Y., Chen, J. (2011). Identifying the recharge sources and age of groundwater in the Songnen Plain (Northeast China) using environmental isotopes. Hydrogeology Journal, 19, 163-176.	Zhang, G., Deng, W., Yang, Y. S., Salama, R. B. (2007). Evolution study of a regional groundwater system using hydrochemistry and stable isotopes in Songnen Plain, northeast China. Hydrological Processes: An International Journal, 21, 1055-1065.	Zhang, B., Song, X.-F., Zhang, Y.-H., Han, D.-M., Tand, C.Y., Yang, L.-H., Wang, Z. L. (2017). The renewability and quality of shallow groundwater in Sanjiang and Songnen Plain, Northeast China. Journal ofIntegrative Agriculture, 16, 229-238.	Approximated from Fig. 1 by Chen et al. (2011), Fig. 1 by Zhang et al. (2007) and Fig. 1 by Zhang et al. (2017)
**Eastern Silurian-Devonian Aquifers**	Northern Midwest Aquifer System	Young, H.L. (1992). Hydrogeology of the Cambrian-Ordovician Aquifer System in the Northern Midwest, United States. U.S. Geological	Wilson, J.T. (2012). Water-quality assessment of the Cambrian-Ordovician aquifer system in the northern Midwest, United States. U.S. Geological	Kay, R. T., Kraske, K. A. (1996). Ground-Water Levels in Aquifers Used for Residential Supply, Campton Township, Kane County, Illinois. U.S.	Broader aquifer system title and its location approximated from Fig. 1 of Young (1992). Silurian- and Devonian-aged rock subcrop approximated from Fig. 9 of Wilson (2012). These Silurian-

Aquifer system	Broader aquifer system	Reference A	Reference B	Reference C	Steps to delineate
		Survey Professional Paper 1405-B, 108 pp. Accessed April 12, 2021 from https://pubs.usgs.gov/pp/1405b/report.pdf	Survey Scientific Investigations Report 2011–5229, 174 pp. Accessed April 12, 2021 from https://pubs.usgs.gov/sir/2011/5229/pdf/SIR20115229_web.pdf	Geological Survey Water-Resources Investigations Report 96-4009, Accessed April 12, 2021 from https://pubs.usgs.gov/wri/1996/4009/report.pdf	Devonian aquifers subcrop along the western side of Lake Michigan; however, they are underlain by the Cambrian-Ordovician aquifers that subcrop to the west (see Fig. 11 of Young (1992)) and thus this "Silurian-Devonian" area is also included as part of the broader "Cambrian-Ordovician Aquifer System". Further, wells in this area (i.e., the "Silurian-Devonian Area") are known to penetrate the deep "Mount Simon Sandstone" aquifer that is Cambrian in age (for another example, a 711 foot well penetrated the Galena-Platteville Formation in 1860 see page B67 of Young (1992)).
**Eastern Sojas Basin**	Sojas Basin	Hosseini, S. M., Parizi, E., Ataie-Ashtiani, B., Simmons, C. T. (2019). Assessment of sustainable groundwater resources management using integrated environmental index: Case studies across Iran. Science of the Total Environment, 676, 792-810.	-	-	Approximated from Fig. 6 by Hosseini et al. (2019)
**Eastern Sutter Basin**	Sacramento Valley of the Central Valley **	California Department of Water Resources (2015i). California's groundwater update 2013 - Chapter 7: Sacramento River Hydrologic Region. California Department of Water Resources Report, 126 pp. Accessed April 27, 2022 via https://water.ca.gov/-/media/DWR-Website/Web-	-	-	Approximated from Fig. 7-2 by California Department of Water Resources (2015i). Eastern and Western sub areas delineated along the Sutter By-pass

Aquifer system	Broader aquifer system	Reference A	Reference B	Reference C	Steps to delineate
		Pages/Programs/Groundwater-Management/Bulletin-118/Files/Statewide-Reports/GWU2013_Ch7_SacramentoRiver_Final.pdf			
**Eastern Tapi Valley Alluvial Aquifer**	Tapi Valley Alluvial Aquifer	Foster, S., Garduño, H., Tuinhof, A. (2007). Confronting the groundwater management. Challenge in the Deccan Traps Country of Maharashtra–India. World Bank Case Profile Collection Number 18, Washington D.C. 19 pp.	-	-	Approximated from Fig. 1 by Foster et al. (2007)
**Eastern Torbat-Zaveh Plain**	Torbat-Zaveh Plain	Nematollahi, M. J., Ebrahimi, P., Razmara, M., Ghasemi, A. (2016). Hydrogeochemical investigations and groundwater quality assessment of Torbat-Zaveh plain, Khorasan Razavi, Iran. Environmental Monitoring and Assessment, 188, 1-21.	-	-	Approximated from Fig. 1 by Nematollahi et al. (2015)
**Eastern Villa Garcia Aquifer**	-	CONAGUA (2020j). Actualización de la disponibilidad media anual de agua en el acuífero Villa Garcia (3213), Estado de Zacatecas. 29 pp. Accessed January 24, 2022 via https://sigagis.conagua.gob.mx/gas1/Edos_Acuiferos_18/zacatecas/DR_3213.pdf	-	-	Approximated from Fig. 2 by CONAGUA (2020j); western and eastern areas delineated o the basis of physicography and drainage
**Eastern Vindhyan Basin**	-	Central Ground Water Board (2013b). Aquifer Systems of Madhya Pradesh. Central Ground Water Board Report, 78 pp.	Singh, Y., Dubey, D. P. (2014). Hydrogeology of the Rewa Region, Central India. Watershed Management for	Bengtson, S., Sallstedt, T., Belivanova, V., Whitehouse, M. (2017). Three-dimensional preservation of cellular and subcellular	Approximated from Plate XXIX by Central Ground Water Board (2013b), descriptions by Singh and Dubey (2014) and Fig. 1 by Bengtson et al. (2017)

Aquifer system	Broader aquifer system	Reference A	Reference B	Reference C	Steps to delineate
		Accessed July 17, 2021 from http://cgwb.gov.in/AQM/Madhya%20Pradesh.pdf	Sustainable Development, 122-134.	structures suggests 1.6 billion-year-old crown-group red algae. PLoS Biology, 15, e2000735.	
**Eastern Washoe Valley**	-	Lopes, T.J., Evetts. D.M. (2005). Ground-Water pumpage and artificial recharge estimates for calendar year 2000 and average annual natural recharge and interbasin flow by hydrographic area, Nevada. Accessed April 25, 2022 via https://pubs.usgs.gov/sir/2004/5239/sir2004-5239.pdf	Harrill, J.R., Prudic, D.E. (1998). Aquifer systems in the Great Basin region of Nevada, Utah, and adjacent states; summary report. US Geological Survey Professional Paper 1409-A, 75 pp. Accessed April 26, 2022 via https://pubs.usgs.gov/pp/1409a/report.pdf	-	Approximated from Fig. 1 by Lopes and Evetts (2005), and Fig. 2 by Harrill and Prudic (1998). Well completion records, physiography and drainage were used to delinate areas.
**Eastside Lower Indus Valley**	Indus Basin	Bonsor, H. C., MacDonald, A. M., Ahmed, K. M., Burgess, W. G., Basharat, M., Calow, R. C., Dixit, A., Foster, S.S.D., Gopal, K., Lapworth, D.J., Moench, M., Mukherjee, A., Rao, M.S., Shamsudduha, M., Smith, L., Taylor, R.G., Tucker, J., van Steenbergen, F., Yadav, S.K., Zahid, A. (2017). Hydrogeological typologies of the Indo-Gangetic basin alluvial aquifer, South Asia. Hydrogeology Journal, 25, 1377-1406.	-	-	Approximated from Fig. 19 by Bonsor et al. (2017)
**Eastside Middle Indus Plain**	Indus Basin	Bonsor, H. C., MacDonald, A. M., Ahmed, K. M., Burgess, W. G., Basharat, M., Calow, R. C., Dixit, A., Foster, S.S.D., Gopal, K., Lapworth, D.J., Moench, M., Mukherjee, A., Rao, M.S., Shamsudduha, M., Smith,	Srivastava, M., Poonia, O.P. (2010). Transboundary Aquifers In Rajasthan, Issues & Management. Bhujal News, 25, 28-36. Accessed July 12, 2021 from http://cgwb.gov.in/Docume	-	Middle Indus area approximated from southwestern portion of highlighted aquifer boundary in Fig. 18 by Bonsor et al. (2017). Subarea approximated from Fig. 2a by Srivastava and Poonia (2010)

Aquifer system	Broader aquifer system	Reference A	Reference B	Reference C	Steps to delineate
		L., Taylor, R.G., Tucker, J., van Steenbergen, F., Yadav, S.K., Zahid, A. (2017). Hydrogeological typologies of the Indo-Gangetic basin alluvial aquifer, South Asia. Hydrogeology Journal, 25, 1377-1406.	nts/Bhujalnews-25-01-02.pdf#page=33		
**Ebro Alluvial Aquifers**	-	Carceller-Layel, T., Costa-Alandí, C., Coloma-López, P., García-Vera, M. Á., & San Román-Saldaña, J. (2007). Groundwater in the central sector of the Ebro Basin. Water Resources Development, 23, 165-187.	-	-	Approximated from Fig. 3 by Carceller-Layel et al. (2007).
**Ebro Delta**	-	Dalmau, A.B., Gimena, E.C., Vierbücher, C.L. (1997). las aguas subterráneas en el delta del ebro. Revista de Obras Públicas, 3.36847. Accessed May 23, 2021 from http://ropdigital.ciccp.es/pdf/publico/1997/1997_septiembre_3368_06.pdf	-	-	Approximated from Fig. 1 by Dalmau et al. (1997)
**Edwards Creek Valley**	-	Lopes, T.J., Evetts. D.M. (2005). Ground-Water pumpage and artificial recharge estimates for calendar year 2000 and average annual natural recharge and interbasin flow by hydrographic area, Nevada. Accessed April 25, 2022 via https://pubs.usgs.gov/sir/2004/5239/sir2004-5239.pdf	Harrill, J.R., Prudic, D.E. (1998). Aquifer systems in the Great Basin region of Nevada, Utah, and adjacent states; summary report. US Geological Survey Professional Paper 1409-A, 75 pp. Accessed April 26, 2022 via https://pubs.usgs.gov/pp/1409a/report.pdf	-	Approximated from Fig. 1 by Lopes and Evetts (2005), and Fig. 2 by Harrill and Prudic (1998). Well completion records, physiography and drainage were used to delinate areas.

Aquifer system	Broader aquifer system	Reference A	Reference B	Reference C	Steps to delineate
**Edwards Plateau**	Edwards-Trinity Aquifer System	Bruun, B., Jackson, K., Lake, P., Walker, J. (2016). Texas Aquifers Study. Texas Water Development Board Report. 336 pp. Accessed April 1, 2021 from https://www.twdb.texas.gov/groundwater/docs/studies/TexasAquifersStudy_2016.pdf#page=89	Barker, R. A., Ardis, A. F. (1996). Hydrogeological framework of the Edwards-Trinity aquifer system, west-central Texas. U.S. Geological Survey Professional Paper 1421-B, 76 pp. Accessed April 1, 2021 from https://pubs.usgs.gov/pp/1421b/report.pdf	-	Approximated from Fig. 6-11 by Bruun et al. (2016) and Fig. 3 by Barker and Ardis (1996).
**Eij Basin**	-	Hosseini, S. M., Parizi, E., Ataie-Ashtiani, B., Simmons, C. T. (2019). Assessment of sustainable groundwater resources management using integrated environmental index: Case studies across Iran. Science of the Total Environment, 676, 792-810.	-	-	Approximated from Fig. 6 by Hosseini et al. (2019)
**El Cajon Valley**	-	California Department of Water Resources (2015e). California's groundwater update 2013 - Chapter 6: South Coast Hydrologic Region. California Department of Water Resources Report, 84 pp. Accessed April 27, 2022 via https://cawaterlibrary.net/wp-content/uploads/2017/05/GWU2013_Ch6_SouthCoast_Final.pdf	-	-	Approximated from Fig. 6-2 by California Department of Water Resources (2015e)
**El Cardito Aquifer**	-	CONAGUA (2021a). Acuíferos (nacional). Webpage accessed March 8, 2022 from http://sina.conagua.gob.mx	-	-	Approximated from aquifer boundaries defined by CONAGUA (2021a) web portal, physiography and drainage patterns, and well completion data.

Aquifer system	Broader aquifer system	Reference A	Reference B	Reference C	Steps to delineate
		/sina/tema.php?tema=acuiferos&ver=mapa&o=1&n=nacional			
**El Llano Basin**	-	Municipio de El Llano (2012). Atlas de Riesgos Naturales del Municipio de El Llano 2012. Report number 201010PP047745, 174 pp. Accessed May 10, 2021 from http://rmgir.proyectomesoamerica.org/PDFMunicipales/2012/01010_El_Llano.pdf	-	-	Approximated from Map. 2 by Municipio de El Llano (2012)
**El Sahuaral Aquifer**	-	Rangel-Medina, M., Monreal, R., Minjarez, I., de la Cruz, L., Oroz, L. (2004). The saline intrusion in the Costa de Hermosillo aquifer in Sonora, México; a challenge to restore. IGME Conference SWIM, Cartagena,, Spain. Accessed May 6, 2021 from http://www.swim-site.nl/pdf/swim18/swim18_059.pdf	-	-	Approximated from Fig. 6 of Rangel-Medina et al. (2014).
**El Salvador Aquifer**	-	CONAGUA (2020m). Actualización de la disponibilidad media anual de agua en el acuífero El Salvador (3219), Estado de Zacatecas. 35 pp. Accessed March 7, 2022 via https://sigagis.conagua.gob.mx/gas1/Edos_Acuiferos_18/zacatecas/DR_3219.pdf	-	-	Approximated from Fig. 1 by CONAGUA (2020m)
**Elandsbaai and Strandfontein Coastal Aquifers**	-	Campbell, E. E., Parker-Nance, T., Bate, G. C. (1992). A compilation of information on the magnitude, nature and	-	-	Approximated from Fig. 3 on pp. 36 of Campbell et al. (1992)

Aquifer system	Broader aquifer system	Reference A	Reference B	Reference C	Steps to delineate
		importance of coastal aquifers in Southern Africa. Water Research Commission Report No. 370/1/92, 193 pp. Accessed October 14, 2021 from http://www.wrc.org.za/wp-content/uploads/mdocs/370-1-92.pdf			
**Elk City Aquifer**	-	Becker, C.J., Runkle, D., Rea, A. (1997b). Digital data sets that describe aquifer characteristics of the Elk City Aquifer in western Oklahoma. U.S. Geological Survey Open-File Report 96-449. Accessed April 5, 2021 from https://pubs.usgs.gov/of/1996/ofr96-449/	-	-	Approximated from map by Becker et al. (1997b) displayed at: https://pubs.usgs.gov/of/1996/ofr96-449/ (accessed April 5, 2021)
**Engle Basin**	-	Anderholm, S.K. (1984). Hydrogeology of the Socorro and La Jencia Basins, Socorro County, New Mexico. US Geological Survey Water-Resources Investigations Report 84-4342, 72 pp. Accessed April 26, 2022 via https://pubs.usgs.gov/wri/1984/4342/report.pdf	-	-	Approximated from Fig. 1 by Anderholm (1984)
**Enid Isolated Terrace Aquifer**	-	Becker, C. J., Runkle, D., Rea, A. (1997a). Digital Data Sets that Describe Aquifer Characteristics of the Enid Isolated Terrace Aquifer in Northwestern Oklahoma. U.S. Geological Survey Open-File Report, 96-450. Accessed April 5,	-	-	Approximated from map by Becker et al. (1997a) available at https://pubs.usgs.gov/of/1996/ofr96-450/

Aquifer system	Broader aquifer system	Reference A	Reference B	Reference C	Steps to delineate
		2021 from https://pubs.usgs.gov/of/1996/ofr96-450/			
**Equus Beds**	High Plains	Gutentag, E.D., Heimes, F.J., Krothe, N.C., Luckey, R.R., Weeks, J.B. (1984). Geohydrology of the High Plains aquifer in parts of Colorado, Kansas, Nebraska, New Mexico, Oklahoma, South Dakota, Texas, and Wyoming (No. 1400-B). Accessed February 10, 2021 from https://pubs.usgs.gov/pp/1400b/report.pdf	Kansas Geological Survey (2021). Webpage entitled "High Plains Aquifer Regions in Kansas" (Kansas High Plains Aquifer Atlas). Accessed April 15, 2021 from https://geokansas.ku.edu/kansas-high-plains-aquifer-atlas)	-	Approximated from Fig. 1 of Gutentag et al. (1984). Great Bend Prairie area approximated from the "High Plains Aquifer Regions in Kansas" (Kansas High Plains Aquifer Atlas) accessed April 15, 2021 from the Kansas Geological Survey (2021; https://geokansas.ku.edu/kansas-high-plains-aquifer-atlas)
**Escondido Valley**	-	California Department of Water Resources (2015e). California's groundwater update 2013 - Chapter 6: South Coast Hydrologic Region. California Department of Water Resources Report, 84 pp. Accessed April 27, 2022 via https://cawaterlibrary.net/wp-content/uploads/2017/05/GWU2013_Ch6_SouthCoast_Final.pdf	-	-	Approximated from Fig. 6-2 by California Department of Water Resources (2015e)
**Esfahan Basin**	Lower Zayandeh-Rud Basin	Hosseini, S. M., Parizi, E., Ataie-Ashtiani, B., Simmons, C. T. (2019). Assessment of sustainable groundwater resources management using integrated environmental index: Case studies across Iran. Science of the Total Environment, 676, 792-810.	Madani, K., Mariño, M. A. (2009). System dynamics analysis for managing Iran's Zayandeh-Rud river basin. Water Resources Management, 23, 2163-2187.	Salemi, H.R., Mamanpoush, A.R., Miranzadeh, M., Akbari, M., Torabi, M., Toomanian, N., Murray-Rust, H., Droogers, P., Sally, H., Gieske, A. (2000). Water management for sustainable irrigated agriculture in the Zayandeh Rud Basin, Esfahan Province, Iran Report by	Approximated from Fig. 6 by Hosseini et al. (2019), Fig. 1 by Madani and Mariño (2009), and Fig. 1 by Salemi et al. (2000)

Aquifer system	Broader aquifer system	Reference A	Reference B	Reference C	Steps to delineate
				Iranian Agricultural Engineering Research Institute, Esfahan Agricultural Research Center, and the International Water Management Institute, Research Report Number 1	
**Esfandagheh Basin**	-	Hosseini, S. M., Parizi, E., Ataie-Ashtiani, B., Simmons, C. T. (2019). Assessment of sustainable groundwater resources management using integrated environmental index: Case studies across Iran. Science of the Total Environment, 676, 792-810.	-	-	Approximated from Fig. 6 by Hosseini et al. (2019)
**Esfandaran Basin**	Lower Zayandeh-Rud Basin	Hosseini, S. M., Parizi, E., Ataie-Ashtiani, B., Simmons, C. T. (2019). Assessment of sustainable groundwater resources management using integrated environmental index: Case studies across Iran. Science of the Total Environment, 676, 792-810.	Madani, K., Mariño, M. A. (2009). System dynamics analysis for managing Iran's Zayandeh-Rud river basin. Water Resources Management, 23, 2163-2187.	Salemi, H.R., Mamanpoush, A.R., Miranzadeh, M., Akbari, M., Torabi, M., Toomanian, N., Murray-Rust, H., Droogers, P., Sally, H., Gieske, A. (2000). Water management for sustainable irrigated agriculture in the Zayandeh Rud Basin, Esfahan Province, Iran Report by Iranian Agricultural Engineering Research Institute, Esfahan Agricultural Research Center, and the International Water Management Institute, Research Report Number 1	Approximated from Fig. 6 by Hosseini et al. (2019), Fig. 1 by Madani and Mariño (2009), and Fig. 1 by Salemi et al. (2000)
**Esfarayen Plain**	-	Hosseini, S. M., Parizi, E., Ataie-Ashtiani, B., Simmons, C. T. (2019).	-	-	Approximated from Fig. 6 by Hosseini et al. (2019)

Aquifer system	Broader aquifer system	Reference A	Reference B	Reference C	Steps to delineate
		Assessment of sustainable groundwater resources management using integrated environmental index: Case studies across Iran. Science of the Total Environment, 676, 792-810.			
**Eshtehard Basin**	-	Ebadati, N., & Sepavandi, S. (2015). Role of geological structures and lithology in the quantitative and qualitative changes of Eshtehard aquifers. Iranian Journal of Ecohydrology, 2, 117-128.	-	-	Approximated from Fig. 1 by Ebadati and Sepavandi (2015)
**Espanola Basin**	Middle Rio Grande	Land, L. (2016a). Overview of Fresh and Brackish Water Quality in New Mexico. Open file Report 583. 4 pp. Accessed February 17, 2021 from https://geoinfo.nmt.edu/resources/water/amp/brochures/BWA/Estancia_Basin_FBWQNM.pdf	Manning, A.H. (2009). Ground-water temperature, noble gas, and carbon isotope data from the Española Basin, New Mexico: U.S. Geological Survey Scientific Investigations Report 2008–5200, 78 pp. Accessed November 28, 2021 from https://pubs.usgs.gov/sir/2008/5200/pdf/SIR08-5200.pdf	-	Approximated from map by Land (2016a), Fig. 1 by Manning (2009)
**Essex Clay Plain**	Southern Ontario Paleozoic Aquifer System	Sharpe, D.R., Piggott, A., Carter, T., Gerber, R.E., MacRitchie, S.M., de Loë, R.C., Strynatka, S., Zwiers, G., Rivera, A. (2013). Southern Ontario hydrogeological region. in: Canada's Groundwater Resources, pp. 444-499. Fitzhenry and Whiteside Limited, Markham, Ontario,	-	-	Approximated from Fig. 12.31 and Box 12-4 by Sharpe et al. (2013)

Aquifer system	Broader aquifer system	Reference A	Reference B	Reference C	Steps to delineate
		Canada. Accessed May 5, 2021 from https://www.researchgate.net/profile/David-Sharpe-2/publication/284034021_Southern_Ontario_hydrogeological_region/links/5f8f462192851c14bcd85341/Southern-Ontario-hydrogeological-region.pdf			
**Estahban Basin**	-	Hosseini, S. M., Parizi, E., Ataie-Ashtiani, B., Simmons, C. T. (2019). Assessment of sustainable groundwater resources management using integrated environmental index: Case studies across Iran. Science of the Total Environment, 676, 792-810.	-	-	Approximated from Fig. 6 by Hosseini et al. (2019)
**Estancia Basin**	-	Land, L. (2016a). Overview of Fresh and Brackish Water Quality in New Mexico. Open file Report 583. 4 pp. Accessed February 17, 2021 from https://geoinfo.nmt.edu/resources/water/amp/brochures/BWA/Estancia_Basin_FBWQNM.pdf	-	-	Approximated from Map by Land (2016a)
**Etla Valley**	Central Valleys of Oaxaca	Ojeda Olivares, E. A., Sandoval Torres, S., Belmonte Jiménez, S. I., Campos Enríquez, J. O., Zignol, F., Reygadas, Y., & Tiefenbacher, J. P. (2019). Climate change, land use/land cover change, and population growth as drivers of groundwater	Flores-Márquez, E. L., Martínez-Serrano, R. G., Chávez, R. E., Crusillo, Y., Jiménez, G., Campos-Enríquez, O. (2008). Numerical modeling of Etla Valley aquifer, Oax., Mexico: Evolution and remediation scenarios.	-	Approximated from Fig. 1 by Ojeda Olivares et al. (2019) and Fig. 1 by Flores-Márquez et al. (2008).

Aquifer system	Broader aquifer system	Reference A	Reference B	Reference C	Steps to delineate
		depletion in the central valleys, Oaxaca, Mexico. Remote Sensing, 11, 1290.	Geofísica Internacional, 47, 27-40.		
**Eureka and Eel River and Mad River Plains**	-	Johnson, M. J. (1975). Ground-water conditions in the Eureka Area, Humboldt County, California. U.S. Geological Survey Water-Resources Investigations 78-127. 51 pp. Accessed March 20, 2021 from https://pubs.usgs.gov/wri/1978/0127/report.pdf	-	-	Approximated from Fig. 1 of Johnson (1975)
**Evan Plain**	Dezful-Andimeshk Plain	Sohrabi, N., Chitsazan, M., Amiri, V., Nezhad, T. M. (2013). Evaluation of groundwater resources in alluvial aquifer based on MODFLOW program, case study: Evan plain (Iran). Int J Agri Crop Sci, 5, 1164-1170.	-	-	Approximated from Fig. 1 by Sohrabi et al. (2013)
**Eyvanekey Basin**	Tehran Plain	Hosseini, S. M., Parizi, E., Ataie-Ashtiani, B., Simmons, C. T. (2019). Assessment of sustainable groundwater resources management using integrated environmental index: Case studies across Iran. Science of the Total Environment, 676, 792-810.	Motagh, M., Walter, T. R., Sharifi, M. A., Fielding, E., Schenk, A., Anderssohn, J., Zschau, J. (2008). Land subsidence in Iran caused by widespread water reservoir overexploitation. Geophysical Research Letters, 35, L16403.	-	Approximated from Fig. 6 by Hosseini et al. (2019). Broader Tehran Plain approximated from Fig. 1a by Motagh et al. (2008)
**Fahliyan Basin**	-	Hosseini, S. M., Parizi, E., Ataie-Ashtiani, B., Simmons, C. T. (2019). Assessment of sustainable groundwater resources management using integrated environmental index: Case studies across	-	-	Approximated from Fig. 6 by Hosseini et al. (2019)

Aquifer system	Broader aquifer system	Reference A	Reference B	Reference C	Steps to delineate
		Iran. Science of the Total Environment, 676, 792-810.			
**Fall River Valley**	-	California Department of Water Resources (2015i). California's groundwater update 2013 - Chapter 7: Sacramento River Hydrologic Region. California Department of Water Resources Report, 126 pp. Accessed April 27, 2022 via https://water.ca.gov/-/media/DWR-Website/Web-Pages/Programs/Groundwater-Management/Bulletin-118/Files/Statewide-Reports/GWU2013_Ch7_SacramentoRiver_Final.pdf	-	-	Approximated from Fig. 7-2 by California Department of Water Resources (2015i)
**Farashband Plain**	-	Aref, F., Roosta, R. (2016). Assessment of groundwater quality and hydrochemical characteristics in Farashband plain, Iran. Arabian Journal of Geosciences, 9(20), 1-14.	-	-	Approximated from Fig. 1 by Aref and Roosta (2016)
**Faryab Basin**	-	Hosseini, S. M., Parizi, E., Ataie-Ashtiani, B., Simmons, C. T. (2019). Assessment of sustainable groundwater resources management using integrated environmental index: Case studies across Iran. Science of the Total Environment, 676, 792-810.	-	-	Approximated from Fig. 6 by Hosseini et al. (2019)
**Fasa Basin**	-	Bahrami, M., Khaksar, E., Khaksar, E. (2020). Spatial variation assessment of	Bagheri, R., Bagheri, F., Eggenkamp, H. G. M. (2017). Origin of	-	Approximated from Fig. 1 by Bahrami et al. (2020) and Fig. 1 by Bagheri et al. (2017)

Aquifer system	Broader aquifer system	Reference A	Reference B	Reference C	Steps to delineate
		groundwater quality using multivariate statistical analysis (Case Study: Fasa Plain, Iran). Journal of Groundwater Science and Engineering, 8, 230-243.	groundwater salinity in the Fasa Plain, southern Iran, hydrogeochemical and isotopic approaches. Environmental Earth Sciences, 76, 1-11.		
**Fedami Basin**	-	Hosseini, S. M., Parizi, E., Ataie-Ashtiani, B., Simmons, C. T. (2019). Assessment of sustainable groundwater resources management using integrated environmental index: Case studies across Iran. Science of the Total Environment, 676, 792-810.	-	-	Approximated from Fig. 6 by Hosseini et al. (2019)
**Ferdows Plains**	-	Hosseini, S. M., Parizi, E., Ataie-Ashtiani, B., Simmons, C. T. (2019). Assessment of sustainable groundwater resources management using integrated environmental index: Case studies across Iran. Science of the Total Environment, 676, 792-810.	-	-	Approximated from Fig. 6 by Hosseini et al. (2019)
**Feyzabad Plain**	-	Zandi, R., Ghahraman, K., Asadi, Z. (2019). Monitoring the Land Subsidence and its Associated Landforms Using Remote Sensing Techniques in Feyzabad Plain (north-east Iran). Journal of Hydrosciences and Environment, 3, 43-51.	-	-	Approximated from Zandi et al. (2019)
**Fezouata Oasis**	Middle Draa Basin	Szczucińska, A., Dłużewski, M., Kozłowski, R., Niedzielski, P. (2019).	-	-	Approximated from Fig. 1 by Szczucińska et al. (2019)

Aquifer system	Broader aquifer system	Reference A	Reference B	Reference C	Steps to delineate
		Hydrochemical diversity of a large alluvial aquifer in an arid zone (Draa river, S Morocco). Ecological Chemistry and Engineering S, 26, 81-100.			
**Firuzabad Plain**	-	Mokhtar, A., Aram, S. (2017). Systemic insights into agricultural groundwater management: case of Firuzabad Plain, Iran. Water Policy, 19, 867-885.	-	-	Approximated from Fig. 1 by Mokhtar and Aram (2017)
**Fish Springs Flat**	-	Lopes, T.J., Evetts. D.M. (2005). Ground-Water pumpage and artificial recharge estimates for calendar year 2000 and average annual natural recharge and interbasin flow by hydrographic area, Nevada. Accessed April 25, 2022 via https://pubs.usgs.gov/sir/2004/5239/sir2004-5239.pdf	Harrill, J.R., Prudic, D.E. (1998). Aquifer systems in the Great Basin region of Nevada, Utah, and adjacent states; summary report. US Geological Survey Professional Paper 1409-A, 75 pp. Accessed April 26, 2022 via https://pubs.usgs.gov/pp/1409a/report.pdf	-	Approximated from Fig. 1 by Lopes and Evetts (2005), and Fig. 2 by Harrill and Prudic (1998). Well completion records, physiography and drainage were used to delinate areas.
**Flint Hills Upland**	Osage Cuestas Aquifer System	Macfarlane, P.A. (2000). Revisions to the Nomenclature for Kansas Aquifers. Kansas Geological Survey Report, 14 pp. Accessed April 27, 2022 via https://journals.ku.edu/cres/article/download/11815/11159	Schoewe, W. H. (1949). The geography of Kansas: Part II. Physical geography. Transactions of the Kansas Academy of Science, 52, 261-333. Accessed April 27, 2022 via https://www.jstor.org/stable/pdf/3625794.pdf	-	Approximated from Fig. 8 by Macfarlane (2000) and Fig. 22 by Schoewe (1949)
**Forez Plain**	-	Larque, P. (1981). La sédimentation et les paléoaltérations tertiaires de la plaine du Forez: nouvelles données. Essai de corrélations stratigraphiques. Sciences	-	-	Approximated from Fig. 1 by Larque (1981)

Aquifer system	Broader aquifer system	Reference A	Reference B	Reference C	Steps to delineate
		Géologiques, Bulletins et Mémoires, 34, 21-35.			
**Fort Bragg Terrace**	-	California Department of Water Resources (2015j). California's groundwater update 2013 - Chapter 3: North Coast Hydrologic Region. California Department of Water Resources Report, 80 pp. Accessed April 27, 2022 via https://water.ca.gov/-/media/DWR-Website/Web-Pages/Programs/Groundwater-Management/Bulletin-118/Files/Statewide-Reports/GWU2013_Ch3_NorthCoast_Final.pdf	-	-	Approximated from Fig. 3-2 by California Department of Water Resources (2015j)
**Fountain Creek Alluvial Aquifer**	-	Radell, MJ., Lewis, M.E., Watts, K.R. (1994). Hydrogeologic characteristics of the alluvial aquifer and adjacent deposits of the Fountain Creek valley, El Paso County, Colorado. U.S. Geological Survey Water-Resources Investigations Report 94-4129, 3 maps and 11 cross sections, Accessed March 29, 2021 from https://pubs.er.usgs.gov/publication/wri944129	-	-	Approximated from Plate 1 of Radell et al. (1994).
**Frenchman Flat**	-	Lopes, T.J., Evetts. D.M. (2005). Ground-Water pumpage and artificial recharge estimates for calendar year 2000 and average annual natural recharge and interbasin	Harrill, J.R., Prudic, D.E. (1998). Aquifer systems in the Great Basin region of Nevada, Utah, and adjacent states; summary report. US Geological Survey Professional Paper 1409-A,	-	Approximated from Fig. 1 by Lopes and Evetts (2005), and Fig. 2 by Harrill and Prudic (1998). Well completion records, physiography and drainage were used to delinate areas.

Aquifer system	Broader aquifer system	Reference A	Reference B	Reference C	Steps to delineate
		flow by hydrographic area, Nevada. Accessed April 25, 2022 via https://pubs.usgs.gov/sir/2004/5239/sir2004-5239.pdf	75 pp. Accessed April 26, 2022 via https://pubs.usgs.gov/pp/1409a/report.pdf		
**Fuente-de-Piedra Aquifer**	-	Rodriguez-Rodriguez, M., Martos-Rosillo, S., Pedrera, A. (2016). Hydrogeological behaviour of the Fuente-de-Piedra playa lake and tectonic origin of its basin (Malaga, southern Spain). Journal of Hydrology, 543, 462-476.	-	-	Approximated from Fig. 1 by Rodriguez-Rodriguez et al. (2016)
**Galehdar Plain**	-	Adinehvand, R., Mozaffarizadeh, J., Sajadi, Z., Ansari, A. (2019). Identifying major factors affecting groundwater quality of the Galehdar plain, south of Fars province. Researches in Earth Sciences, 10, 1-14.	-	-	Approximated from Fig. 1 by Adinehvand et al. (2019)
**Galion Glaciated Low Plateau**	Central Lowland Till Plain	Brockman, S. (1998). Physiographic regions of Ohio. Ohio Department of Natural Resources, Division of Geological Survey map. Accessed April 12, 20221 from https://www.epa.state.oh.us/portals/27/SIP/Nonattain/F2-physiographic_regions_of_Ohio.pdf	Fenneman, N.M., Johnson, D.W. (1946). Physiographic divisions of the conterminous United States. U.S. Geological Survey map, 1:7,000,000 Scale.	-	Broader Till Plain physiographic region approximated from Fenneman and Johnson (1946). Galion Glaciated Low Plateau approximated from the map by Brockman (1998).
**Galisteo Aquifer**	-	Camuñas Palencia, C., Mejías Moreno, M., Hornero Díaz, J. E., Ruíz Bermudo, F., García Menéndez, O. (2022). Deep aquifers as strategic groundwater	-	-	Approximated from Fig. 3 by Camuñas Palencia et al. (2022)

Aquifer system	Broader aquifer system	Reference A	Reference B	Reference C	Steps to delineate
		reservoir in Spain. Boletín Geológico y Miner, 133, 7-26. Accessed April 25, 2023 via https://www.igme.es/boletin/2022/133_3/BGM_133-3_Art-1.pdf			
**Gallatin Valley**	-	Kendy, E. (2001). Ground-water resources of the Gallatin Local Water Quality District, southwestern Montana. US Geological Survey Fact Sheet 007-01, 4 pp. Accessed March 29, 2021 from https://pubs.usgs.gov/fs/2001/0007/report.pdf	-	-	Approximated from Fig. 1 of Kendy (2001).
**Gambier Embayment**	Otway Basin	Love, A. J., Herczeg, A. L., Leaney, F. W., Stadter, M. F., Dighton, J. C., Armstrong, D. (1994). Groundwater residence time and palaeohydrology in the Otway Basin, South Australia: 2H, 18O and 14C data. Journal of Hydrology, 153, 157-187.	Barnett, S., Lawson, J., Li, C., Morgan, L., Wright, S., Skewes, M., Harrington, N., Woods, J., Werner, A., Plush, B. (2015). A Hydrostratigraphic Model for the Shallow Aquifer Systems of the Gambier Basin and South Western Murray Basin, Goyder Institute for Water Research Technical Report Series No. 15/15, 45 pp. Accessed July 27, 2021 from http://www.goyderinstitute.org/_r344/media/system/attrib/attachment/325/15-15_Hydrostrat_model_RAC_v4.pdf	Harrington, G. A., Walker, G. R., Love, A. J., Narayan, K. A. (1999). A compartmental mixing-cell approach for the quantitative assessment of groundwater dynamics in the Otway Basin, South Australia. Journal of Hydrology, 214, 49-63.	Approximated from Fig. 1 by Love et al. (1994), Fig. 1 by Harrington et al. (1999), and Fig. 1 by Barnett et al. 2015)
**Gandia-Denia Coastal Plain**	-	Navarro, B.J.B. (2003). Estado y evolución de los procesos de intrusión marina en la unidad	-	-	Approximated from Fig. 1 by Navarro (2003).

Aquifer system	Broader aquifer system	Reference A	Reference B	Reference C	Steps to delineate
		hidrogeológica 08.38 plana de Gandía-Denia (Valencia-Alicante, España). Tecnología De La Intrusión de Agua De Mar en Acuíferos Costeros: Países Mediterráneos,11 pp. Accessed May 23, 2021 from http://aguas.igme.es/igme/publica/tiac-01/Area%20V-17.pdf			
**Gandoman-Boldaji Aquifer**	-	Malekmohammadi, B., Jahanishakib, F. (2017). Vulnerability assessment of wetland landscape ecosystem services using driver-pressure-state-impact-response (DPSIR) model. Ecological Indicators, 82, 293-303.	-	-	Approximated from Fig. 1 by Malekmohammadi and Jahanishakib (2017)
**Ganuelas-Mazarron Basin**	-	Rodrigo-Naharro, J., Aracil, E., del Villar, L. P. (2018). Geophysical investigations in the Gañuelas-Mazarrón Tertiary basin (SE Spain): A natural analogue of a geological CO2 storage affected by anthropogenic leakages. Journal of Applied Geophysics, 155, 187-198.	-	-	Approximated from Fig. 1 by Rodrigo-Naharro et al. (2018)
**Garber-Wellington Aquifer**	-	Mashburn, S.L., Ryter, D.W., Neel, C.R., Smith, S.J., Correll, J.S. (2014). Hydrogeology and simulation of ground-water flow in the Central Oklahoma (Garber-Wellington) Aquifer, Oklahoma, 1987 to 2009, and simulation of avail-able	-	-	Approximated from Fig. 4 of Mashburn et al. (2014)

Aquifer system	Broader aquifer system	Reference A	Reference B	Reference C	Steps to delineate
		water in storage, 2010–2059. U.S. Geological Survey Scientific Investigations Report 2013–5219, 92 pp. Accessed April 5, 2021 from https://pubs.usgs.gov/sir/2013/5219/pdf/sir20135219_v2.0.pdf			
**Garden Basin**	-	Lopes, T.J., Evetts. D.M. (2005). Ground-Water pumpage and artificial recharge estimates for calendar year 2000 and average annual natural recharge and interbasin flow by hydrographic area, Nevada. Accessed April 25, 2022 via https://pubs.usgs.gov/sir/2004/5239/sir2004-5239.pdf	Harrill, J.R., Prudic, D.E. (1998). Aquifer systems in the Great Basin region of Nevada, Utah, and adjacent states; summary report. US Geological Survey Professional Paper 1409-A, 75 pp. Accessed April 26, 2022 via https://pubs.usgs.gov/pp/1409a/report.pdf	-	Approximated from Fig. 1 by Lopes and Evetts (2005), and Fig. 2 by Harrill and Prudic (1998). Well completion records, physiography and drainage were used to delinate areas.
**Garden Valley**	-	Graham, W.G., Campbell, L.J. (1981). Groundwater resources of Idaho. Idaho Department of Water Resources Report, 61 pp. Accessed March 23, 2021 from https://idwr.idaho.gov/files/publications/198108-MISC-GW-Resources-ID.pdf	-	-	Approximated from Plate 1 of Graham and Campbell (1981).
**Gareh-Bygone Plain**	-	Hashemi, H., Berndtsson, R., Kompani-Zare, M. (2012). Steady-state unconfined aquifer simulation of the Gareh-Bygone Plain, Iran. The Open Hydrology Journal, 6, 58-67.	Rahimi, S., Roodposhti, M. S., Abbaspour, R. A. (2014). Using combined AHP–genetic algorithm in artificial groundwater recharge site selection of Gareh Bygone Plain, Iran. Environmental earth sciences, 72, 1979-1992.	Ahmadvand, M., Karami, E. (2009). A social impact assessment of the floodwater spreading project on the Gareh-Bygone plain in Iran: a causal comparative approach. Environmental Impact Assessment Review, 29, 126-136.	Approximated from Fig. 4 by Hashemi et al. (2012), Fig. 2 by Rahimi et al. (2014) and Fig. 1 by Ahmadvand and Karami (2009)

Aquifer system	Broader aquifer system	Reference A	Reference B	Reference C	Steps to delineate
**Garmsar Basin**	-	Nasiri, A., Shirocova, V. A., Zareie, S. (2019). Zoning of Groundwater Quality for Plain Garmsar in Iran. Water Resources, 46, 624-629.	-	-	Approximated from Fig. 1 by Nasiri et al. (2019)
**Geer Basin and Hesbaye Chalk Aquifer**	-	Orban, P., Brouyère, S., Batlle-Aguilar, J., Couturier, J., Goderniaux, P., Leroy, M., Maloszewski, P., Dassargues, A. (2010). Regional transport modelling for nitrate trend assessment and forecasting in a chalk aquifer. Journal of Contaminant Hydrology, 118, 79-93.	Goderniaux, P., Orban, P., Rorive, A., Brouyère, S., Dassargues, A. (2023). Study of historical groundwater level changes in two Belgian chalk aquifers in the context of climate change impacts. Geological Society, London, Special Publications, 517, SP517-2020.	-	Approximated from Fig. 1 by Orban et al. (2011) and Fig. 1 by Goderniaux et al. (2021)
**Gem Valley**	-	Graham, W.G., Campbell, L.J. (1981). Groundwater resources of Idaho. Idaho Department of Water Resources Report, 61 pp. Accessed March 23, 2021 from https://idwr.idaho.gov/files/publications/198108-MISC-GW-Resources-ID.pdf	-	-	Approximated from Plate 1 of Graham and Campbell (1981).
**Gerash Basin**	-	Hosseini, S. M., Parizi, E., Ataie-Ashtiani, B., Simmons, C. T. (2019). Assessment of sustainable groundwater resources management using integrated environmental index: Case studies across Iran. Science of the Total Environment, 676, 792-810.	-	-	Approximated from Fig. 6 by Hosseini et al. (2019)
**Ghaap Plateau Dolomite**	-	Department of Water Affairs and Forestry, South Africa (2006). Vaal River	Smit, P. J. (1978). Groundwater recharge in the dolomite of the Ghaap	-	Approximated from map on page 11 by Department of Water Affairs

Aquifer system	Broader aquifer system	Reference A	Reference B	Reference C	Steps to delineate
**Aquifer System**		system: large bulk water supply reconciliation strategy: groundwater assessment: dolomite aquifers. DWAF Report Number: P RSA C000/00/4406/06, 71 pp. Accessed October 19, 2021 from https://www.dws.gov.za/iwrp/Vaal/documents/LargeBulkWater/06_Dolomitic%20Groundwater%20Assessment_Final.pdf	Plateau near Kuruman in the Northern Cape, Republic of South Africa. Water SA, 4, 81-92.		and Forestry, South Africa (2006), Fig. 2 by Smit (1978)
**Gharebagh Basin**	-	Hosseini, S. M., Parizi, E., Ataie-Ashtiani, B., Simmons, C. T. (2019). Assessment of sustainable groundwater resources management using integrated environmental index: Case studies across Iran. Science of the Total Environment, 676, 792-810.	-	-	Approximated from Fig. 6 by Hosseini et al. (2019)
**Gharesoo-Gorgan Rood Coastal Aquifer**	-	Khosravi, K., Bordbar, M., Paryani, S., Saco, P. M., Kazakis, N. (2021). New hybrid-based approach for improving the accuracy of coastal aquifer vulnerability assessment maps. Science of The Total Environment, 767, 145416.	Bordbar, M., Neshat, A., Javadi, S. (2019). A new hybrid framework for optimization and modification of groundwater vulnerability in coastal aquifer. Environmental Science and Pollution Research, 26, 21808-21827.	-	Approximated from Fig. 1 by Khosravi et al. (2021) and Fig. 1 by Bordbar et al. (2019)
**Ghir Karzin Basin**	-	Hosseini, S. M., Parizi, E., Ataie-Ashtiani, B., Simmons, C. T. (2019). Assessment of sustainable groundwater resources management using	-	-	Approximated from Fig. 6 by Hosseini et al. (2019)

Aquifer system	Broader aquifer system	Reference A	Reference B	Reference C	Steps to delineate
		integrated environmental index: Case studies across Iran. Science of the Total Environment, 676, 792-810.			
**Ghoharkoh Basin**	-	Hosseini, S. M., Parizi, E., Ataie-Ashtiani, B., Simmons, C. T. (2019). Assessment of sustainable groundwater resources management using integrated environmental index: Case studies across Iran. Science of the Total Environment, 676, 792-810.	-	-	Approximated from Fig. 6 by Hosseini et al. (2019)
**Ghorghi Ziyarat Plain**	-	Hosseini, S. M., Parizi, E., Ataie-Ashtiani, B., Simmons, C. T. (2019). Assessment of sustainable groundwater resources management using integrated environmental index: Case studies across Iran. Science of the Total Environment, 676, 792-810.	-	-	Approximated from Fig. 6 by Hosseini et al. (2019)
**Ghorveh-Dehgelan Aquifer**	-	Rahmati, O., Samani, A. N., Mahmoodi, N., Mahdavi, M. (2015). Assessment of the contribution of N-fertilizers to nitrate pollution of groundwater in western Iran (Case Study: Ghorveh–Dehgelan Aquifer). Water Quality, Exposure and Health, 7, 143-151.	-	-	Approximated from Fig. 1 by Rahmati et al. (2015)
**Gila Bend Basin**	-	Tillman, F.D, Cordova, J.T., Leake, S.A., Thomas, B.E., Callegary, J.B. (2011).	-	-	Approximated from Fig. 2 of Tillman et al. (2011); boundaries also informed by the density of

Aquifer system	Broader aquifer system	Reference A	Reference B	Reference C	Steps to delineate
		Water availability and use pilot: methods development for a regional assessment of groundwater availability, southwest alluvial basins, Arizona. U.S. Geological Survey Scientific Investigations Report 2011-5071, 132 pp. Accessed April 15, 2021 from https://pubs.usgs.gov/sir/2011/5071/sir2011-5071_text.pdf			wells recorded in Arizona's well completion dataset.
**Gila Valley**	-	Knechtel, M. M., Lohr, E. W. (1938). Geology and ground-water resources of the Valley of Gila River and San Simon Creek, Graham County, Arizona; with a section on the Chemical character of the ground water. U.S. Geological Survey Water-Supply Paper 796-F, 67 pp. Accessed March 29, 2021 from https://pubs.usgs.gov/wsp/0796f/report.pdf	-	-	Approximated from map on page 10 of Knechtel and Lohr (1938).
**Gnangara Mound**	Perth Basin	Meredith, K., Cendón, D. I., Pigois, J. P., Hollins, S., Jacobsen, G. (2012). Using 14C and 3H to delineate a recharge 'window' into the Perth Basin aquifers, North Gnangara groundwater system, Western Australia. Science of the Total Environment, 414, 456-469.	Davidson, W.A., Yu, X. (2006). Perth region aquifer modelling system — PRAMS, Hydrogeology and groundwater modelling. Western Australia Department of Water Hydrogeological Record Series HG 20, 254 pp. Accessed July 26, 2021 from https://www.water.wa.gov.au/__data/assets/pdf_file/0015/5280/71802.pdf	Bekesi, G., McGuire, M., Moiler, D. (2009). Groundwater allocation using a groundwater level response management method—Gnangara groundwater system, Western Australia. Water Resources Management, 23, 1665-1683.	Approximated from Fig. 6 by Davidson and Yu (2006), Fig. 1 by Bekesi et al. (2009), and Fig. 1 by Meredith et al. (2012)

Aquifer system	Broader aquifer system	Reference A	Reference B	Reference C	Steps to delineate
**Gold Flat**	-	Lopes, T.J., Evetts. D.M. (2005). Ground-Water pumpage and artificial recharge estimates for calendar year 2000 and average annual natural recharge and interbasin flow by hydrographic area, Nevada. Accessed April 25, 2022 via https://pubs.usgs.gov/sir/2004/5239/sir2004-5239.pdf	Harrill, J.R., Prudic, D.E. (1998). Aquifer systems in the Great Basin region of Nevada, Utah, and adjacent states; summary report. US Geological Survey Professional Paper 1409-A, 75 pp. Accessed April 26, 2022 via https://pubs.usgs.gov/pp/1409a/report.pdf	-	Approximated from Fig. 1 by Lopes and Evetts (2005), and Fig. 2 by Harrill and Prudic (1998). Well completion records, physiography and drainage were used to delinate areas.
**Goleta Basin**	-	Bachman, S. (2010). Goleta Groundwater Basin Groundwater Management Plan. 91 pp. Accessed March 20,2021 from https://www.goletawater.com/assets/uploads/documents/groundwater-management/Groundwater_Management_Plan_Final_05-11-10.pdf	-	-	Approximated from Fig. 1-1 of Bachman (2010)
**Golmud Alluvial Fan**	-	Xiao, Y., Liu, K., Yan, H., Zhou, B., Huang, X., Hao, Q., Zhang, Y., Zhang, Y., Liao, X. Yin, S. (2021). Hydrogeochemical constraints on groundwater resource sustainable development in the arid Golmud alluvial fan plain on Tibetan plateau. Environmental Earth Sciences, 80, 1-17.	-	-	Approximated from Fig. 1 by Xiao et al. (2021)
**Golpayegan Aquifer**	-	Mohebbi Tafreshi, G., Mohebbi Tafreshi, A. (2020). Statistical approaches and hydrochemical modeling of groundwater in the	-	-	Approximated from Fig. 1 by Mohebbi Tafreshi and Mohebbi Tafreshi (2020)

Aquifer system	Broader aquifer system	Reference A	Reference B	Reference C	Steps to delineate
		Golpayegan Plain aquifer, Iran. Modeling Earth Systems and Environment, 6, 2391-2404.			
**Gonabad Plain**	-	Shams, M., Dobaradaran, S., Mazloomi, S., Afsharnia, M., Ghasemi, M., Bahreinie, M. (2012). Drinking water in Gonabad, Iran: fluoride levels in bottled, distribution network, point of use desalinator, and decentralized municipal desalination plant water. Fluoride, 45, 138.	Qasemi, M., Afsharnia, M., Zarei, A., Farhang, M., Allahdadi, M. (2018). Non-carcinogenic risk assessment to human health due to intake of fluoride in the groundwater in rural areas of Gonabad and Bajestan, Iran: a case study. Human and Ecological Risk Assessment: An International Journal, 1222-1233.	-	Approximated from Fig. 1 by Shams et al. (2012) and from Fig. 1 by Qasemi et al. (2018b)
**Gonzales-New Orleans Aquifer**	Gulf Coast Regional Aquifer System	Weiss, J. S. (1990). Geohydrologic units of the coastal lowlands aquifer system, south-central United States. U.S. Geological Survey Regional aquifer-systems analysis. 42 pp. Accessed March 30, 2021 from https://pubs.usgs.gov/pp/1416c/report.pdf	Louisiana Department of Transportation and Development (2014a). Water Resources of St. John the Baptist Parish, Louisiana. U.S. Geological Survey Fact Sheet 2014–3102, 6 pp. Accessed March 30, 2021 from https://pubs.usgs.gov/fs/2014/3102/pdf/fs2014-3102.pdf	Louisiana Department of Transportation and Development (2014b). Water Resources of Orleans Parish, Louisiana. U.S. Geological Survey Fact Sheet 2014–3017, 6 pp. Accessed March 30, 2021 from https://pubs.usgs.gov/fs/2014/3017/pdf/fs2014-3017.pdf	Outer margin of Gulf Coast Regional Aquifer System approximated from Fig. 2 of Weiss (1990). Detailed study site information for a portion of the subarea available from Louisiana Department of Transportation and Development (2014a, b)
**Goose Creek and Golden Valley**	Central Snake River Plain	Graham, W.G., Campbell, L.J. (1981). Groundwater resources of Idaho. Idaho Department of Water Resources Report, 61 pp. Accessed March 23, 2021 from https://idwr.idaho.gov/files/publications/198108-MISC-GW-Resources-ID.pdf	-	-	Approximated from Plate 1 of Graham and Campbell (1981).
**Goshen Valley**	-	Brooks, L.E. (2013). Evaluation of the	-	-	Approximated from Fig. 1 of Brooks (2013)

Aquifer system	Broader aquifer system	Reference A	Reference B	Reference C	Steps to delineate
		groundwater flow model for southern Utah and Goshen Valleys, Utah, updated to conditions through 2011, with new projections and groundwater management simulations: U.S. Geological Survey Open-File Report 2013–1171, 35 pp. Accessed March 7, 2021 from https://pubs.usgs.gov/of/2013/1171/pdf/ofr2013-1171.pdf			
**Goshnekan Basin**	-	Hosseini, S. M., Parizi, E., Ataie-Ashtiani, B., Simmons, C. T. (2019). Assessment of sustainable groundwater resources management using integrated environmental index: Case studies across Iran. Science of the Total Environment, 676, 792-810.	-	-	Approximated from Fig. 6 by Hosseini et al. (2019)
**Goshute Valley**	-	Lopes, T.J., Evetts. D.M. (2005). Ground-Water pumpage and artificial recharge estimates for calendar year 2000 and average annual natural recharge and interbasin flow by hydrographic area, Nevada. Accessed April 25, 2022 via https://pubs.usgs.gov/sir/2004/5239/sir2004-5239.pdf	Harrill, J.R., Prudic, D.E. (1998). Aquifer systems in the Great Basin region of Nevada, Utah, and adjacent states; summary report. US Geological Survey Professional Paper 1409-A, 75 pp. Accessed April 26, 2022 via https://pubs.usgs.gov/pp/1409a/report.pdf	-	Approximated from Fig. 1 by Lopes and Evetts (2005), and Fig. 2 by Harrill and Prudic (1998). Well completion records, physiography and drainage were used to delinate areas.
**Gotvand Basin**	Dezful-Andimeshk Plain	Hosseini, S. M., Parizi, E., Ataie-Ashtiani, B., Simmons, C. T. (2019). Assessment of sustainable	-	-	Approximated from Fig. 6 by Hosseini et al. (2019)

Aquifer system	Broader aquifer system	Reference A	Reference B	Reference C	Steps to delineate
		groundwater resources management using integrated environmental index: Case studies across Iran. Science of the Total Environment, 676, 792-810.			
**Grand Forks Aquifer**	-	Wei, M., Allen, D. M., Carmichael, V., Ronneseth, K. (2010). State of understanding of the hydrogeology of the Grand Forks aquifer. Water Stewardship Division, BC Ministry of Environment Report, 99 pp. Accessed March 15, 2021 from https://www.grandforks.ca/wp-content/uploads/reports/2010-Hydrogeology-Study-of-Grand-Forks-area.pdf	-	-	Approximated from Fig. 2 of Wei et al. (2010)
**Grande Terre Limestone Aquifer**	-	Ministere de l'Ecologie, du Developpement Durable et de l'Energie (2012). Hydrologie souterraine synthèse. BRGM Report, 184 pp. Accessed May 22, 2023 via https://professionnels.ofb.fr/sites/default/files/pdf/RE_Explore2070_Eaux_Sout_Synthese.pdf	-	-	Approximated from descriptions within section 6.4.6
**Grass Valley in northern Nevada**	-	Welch, A. H., Sorey, M. L., Olmsted, F. H. (1981). Hydrothermal system in Southern Grass Valley, Pershing County, Nevada. U.S. Geological Survey Open-File Report 81-915, 200 pp. Accessed March 22,	-	-	Approximated from Fig. 2 of Welch et al. (1981)

Aquifer system	Broader aquifer system	Reference A	Reference B	Reference C	Steps to delineate
		2021 from https://www.osti.gov/servlets/purl/5119283-5mJ8YB/			
**Grazer Feld**	-	Kralik, M., Humer, F., Fank, J., Harum, T., Klammler, G., Gooddy, D., Sültenfuß, J., Gerber, C., Purtschert, R. (2014). Using 18O/2H, 3H/3He, 85Kr and CFCs to determine mean residence times and water origin in the Grazer and Leibnitzer Feld groundwater bodies (Austria). Applied Geochemistry, 50, 150-163.	Retter, A., Griebler, C., Haas, J., Birk, S., Stumpp, C., Brielmann, H., Fillinger, L. (2021). Application of the DA-(C) index as a simple tool for microbial-ecological characterization and assessment of groundwater ecosystems—a case study of the Mur River Valley, Austria. Österr Wasser- und Abfallw, https://doi.org/10.1007/s00506-021-00799-5	-	Approximated from Fig. 2 by Kralik et al. (2014) and Fig. 1 by Retter et al. (2021)
**Great Bend Prairie**	High Plains	Gutentag, E.D., Heimes, F.J., Krothe, N.C., Luckey, R.R., Weeks, J.B. (1984). Geohydrology of the High Plains aquifer in parts of Colorado, Kansas, Nebraska, New Mexico, Oklahoma, South Dakota, Texas, and Wyoming (No. 1400-B). Accessed February 10, 2021 from https://pubs.usgs.gov/pp/1400b/report.pdf	Kansas Geological Survey (2021). Webpage entitled "High Plains Aquifer Regions in Kansas" (Kansas High Plains Aquifer Atlas). Accessed April 15, 2021 from https://geokansas.ku.edu/kansas-high-plains-aquifer-atlas)	-	Approximated from Fig. 1 of Gutentag et al. (1984). Great Bend Prairie area approximated from the "High Plains Aquifer Regions in Kansas" (Kansas High Plains Aquifer Atlas) accessed April 15, 2021 from the Kansas Geological Survey (2021; https://geokansas.ku.edu/kansas-high-plains-aquifer-atlas)
**Great Plains Dakota Aquifer**	-	Whittlemore, D.O., Macfarlane, P.A., Wilson, B.B. (2014). Water Resources of the Dakota Aquifer in Kansas. Kansas Geological Survey Bulletin 260, 68 pp. Accessed April 15, 2021 from http://www.kgs.ku.edu/Publications/Bulletins/260/Bulletin_260_Dakota.pdf	-	-	Approximated from Fig. 3 of Whittlemore et al. (2014).

Aquifer system	Broader aquifer system	Reference A	Reference B	Reference C	Steps to delineate
**Great Sand Hills North Aquifer**	Bearpaw-Judith River Aquifer System	Maathuis, H., Simpson, M. (2007b). Groundwater Resources of the Prelate (72K) area, Saskatchewan. Saskatchewan Research Council Publication No. 11975-1E07, 121 pp. Accessed April 26, 2021 from https://www.wsask.ca/Global/Water%20Info/Ground%20Water/Mapping/Prelate%20Mapsheet%2072K/Reports/11975-1E07-Prelate_Final_public.pdf	-	-	Approximated from Fig. 45 of Maathuis and Simpson (2007b). Included in part of the Judith River Aquifer System (broader area), as wells within the area are known to tap the Judith River formation (see map in Fig. 24 by Maathuis and Simpson, 2007b)
**Grouse Creek Valley**	-	Lopes, T.J., Evetts. D.M. (2005). Ground-Water pumpage and artificial recharge estimates for calendar year 2000 and average annual natural recharge and interbasin flow by hydrographic area, Nevada. Accessed April 25, 2022 via https://pubs.usgs.gov/sir/2004/5239/sir2004-5239.pdf	Harrill, J.R., Prudic, D.E. (1998). Aquifer systems in the Great Basin region of Nevada, Utah, and adjacent states; summary report. US Geological Survey Professional Paper 1409-A, 75 pp. Accessed April 26, 2022 via https://pubs.usgs.gov/pp/1409a/report.pdf	-	Approximated from Fig. 1 by Lopes and Evetts (2005), and Fig. 2 by Harrill and Prudic (1998). Well completion records, physiography and drainage were used to delinate areas.
**Guadalentin Valley**	-	Cerón, J. C., Pulido-Bosch, A. (1996). Groundwater problems resulting from CO 2 pollution and overexploitation in Alto Guadalentín aquifer (Murcia, Spain). Environmental Geology, 28, 223-228.	Ezquerro, P., Guardiola-Albert, C., Herrera, G., Fernández-Merodo, J. A., Béjar-Pizarro, M., Bonì, R. (2017). Groundwater and subsidence modeling combining geological and multi-satellite SAR data over the alto Guadalentín Aquifer (SE Spain). Geofluids, 1359325, doi.org/10.1155/2017/1359325	Calatrava, J., Guillem, A., Martínez-Granados, D. (2011). Analysis of alternatives to eliminate aquifer overdraft in the Guadalentín Valley, SE Spain. Economía Agraria y Recursos Naturales-Agricultural and Resource Economics, 11, 33-62.	Approximated from Fig. 1 by Cerón and Pulido-Bosch (1996), Fig. 6a by Ezquerro et al. (2017), and Map 2 by Calatrava et al. (2011)

Aquifer system	Broader aquifer system	Reference A	Reference B	Reference C	Steps to delineate
**Guadalupe de Las Corrientes Aquifer**	-	CONAGUA (2021a). Acuíferos (nacional). Webpage accessed March 8, 2022 from http://sina.conagua.gob.mx/sina/tema.php?tema=acuiferos&ver=mapa&o=1&n=nacional	-	-	Approximated from aquifer boundaries defined by CONAGUA (2021a) web portal, physiography and drainage patterns, and well completion data.
**Guadiana Valley**	-	Herrera, M. T. A., Montenegro, I. F., Navar, P. R., Domínguez, I. R. M., Vázquez, R. T. (2001). Contenido de arsénico en el agua potable del valle del Guadiana, México. Tecnología y ciencias del agua, 16, 63-70.	-	-	Approximated from Illustracion 1 by Herrera et al. (2001)
**Guaymas Valley**	-	Martínez-Retama, S., Flores, C., Castillo-Gurrola, J. (2007). Saline intrusion in Guaymas Valley, Mexico from time-domain electromagnetic soundings. Geofísica Internacional, 46, 175-198.	Vega-Granillo, E.L., Cirett-Galán, S, De la Parra-Velasco, M.L., Zavala-Juárez, R. (2011). Hidrogeología de Sonora, México. in: Calmus, Thierry, ed., Panorama de la geología de Sonora, México, Universidad Nacional Autónoma de México, Instituto de Geología, Boletín 118, 267–298. Accessed May 6, 2021 from	-	Approximated from Fig. 1 by Martínez-Retama et al. (2007) and from Fig. 5 of Vega-Granillo et al. (2011).
**Guelmim Basin**	-	El Mahdad, E., Ouhajou, L., El Fasskaoui, M., Aslikh, A., Nghira, A., Fdil, F., Baroud, A., Barceló, D. (2017). Experiences, success stories, and lessons learnt from the implementation of the Water Law Framework Directive in the Souss-Massa River Basin. In: The Souss-Massa River Basin,	-	-	Approximated from Fig. 1 by El Mahdad et al. (2017)

Aquifer system	Broader aquifer system	Reference A	Reference B	Reference C	Steps to delineate
		Morocco (eds. Choukr-Allah, R., Ragab, R., Bouchaou , L., Barceló, D.), pp. 303-333.			
**Guerrero-Yepomera Aquifer**	-	CONAGUA (2015i). Actualización de la disponibilidad media anual de agua en el acuífero Flores Magon-Villa Ahumada (0821), Estado de Chihuahua. 31 pp. Accessed January 23, 2022 via https://www.gob.mx/cms/uploads/attachment/file/103582/DR_0821.pdf	-	-	Approximated from descriptions within CONAGUA (2015i) and well completion data
**Gulgir Plain**	-	Hamid Reza, N., Ferdows, S. N. (2012). Comparing Vulnerability Delineative Of Aquifer Using Drastic And Fuzzy Logic Methods (Case Study: Gulgir Plain Of Masjed Solieman, Iran). Proceedings of conference entitled "GIS Ostrava 2012 -Surface models for geosciences", held January 23-25, Ostrava, Czech Republic, 14 pp. Accessed June 7, 2021 from http://gisak.vsb.cz/GIS_Ostrava/GIS_Ova_2012/sbornik/papers/nassery.pdf	-	-	Approximated from Fig. 1 by Hamid Reza and Ferdows (2012)
**Gunnedah Basin and Liverpool Plains**	-	Land and Water Commissioner (2019). Groundwater: Gunnedah Basin NSW, What water information can tell us. Presentation (19 slides) accessed October 8, 2021 from	Zhang, L., Stauffacher, M., Walker, G.R., Dyce, P. (1997). Recharge estimation in the Liverpool Plains (NSW) for input Groundwater Models. CSIRO Technical Report 10/97, 20 pp.	-	Approximated from slide 2 by Land and Water Commissioner (2019) and Fig. 1 by Zhang et al. (1997)

Aquifer system	Broader aquifer system	Reference A	Reference B	Reference C	Steps to delineate
		https://www.industry.nsw.gov.au/__data/assets/pdf_file/0020/104852/gunnedah-groundwater-presentation.pdf			
**Gushi Depression**	Weihe Basin	Zhang, W., Li, Y., Zhao, F., Han, W., Li, Y., Wang, Y., Holland, G. Zhou, Z. (2019). Using noble gases to trace groundwater evolution and assess helium accumulation in Weihe Basin, central China. Geochimica et Cosmochimica Acta, 251, 229-246.	-	-	Approximated from Fig. 1 by Zhang et al. (2019)
**Gypsum Hills**	-	Schoewe, W. H. (1949). The geography of Kansas: Part II. Physical geography. Transactions of the Kansas Academy of Science, 52, 261-333. Accessed April 27, 2022 via https://www.jstor.org/stable/pdf/3625794.pdf	-	-	Approximated from Fig. 22 by Schoewe (1949)
**Ha Tinh Coastal Quaternary Aquifers**	-	Truong, P.V. (2015). Hydrogeochemistry characteristics and salinity of groundwater in Quaternary sediments in the coastal zone of Ha Tinh province. Vietnam Journal of Earth Sciences, 37, 70-78. Accessed April 11, 2023 via https://vjs.ac.vn/index.php/jse/article/view/6557/pdf_1	-	-	Approximated from Fig. 1 by Truong (2015). Western boundary approximated by drainage divide
**Half Moon Bay Coastal Terraces**	-	California Department of Water Resources (2015g). California's groundwater	-	-	Approximated from Fig. 4-2 by California Department of Water Resources (2015g)

Aquifer system	Broader aquifer system	Reference A	Reference B	Reference C	Steps to delineate
		update 2013 - Chapter 4: San Francisco Bay Hydrologic Region. California Department of Water Resources Report, 78 pp. Accessed April 27, 2022 via https://cawaterlibrary.net/wp-content/uploads/2017/05/GWU2013_Ch4_SanFranciscoBay_Final.pdf			
**Halilan Basin**	-	Hosseini, S. M., Parizi, E., Ataie-Ashtiani, B., Simmons, C. T. (2019). Assessment of sustainable groundwater resources management using integrated environmental index: Case studies across Iran. Science of the Total Environment, 676, 792-810.	-	-	Approximated from Fig. 6 by Hosseini et al. (2019)
**Halvan Basin**	-	Hosseini, S. M., Parizi, E., Ataie-Ashtiani, B., Simmons, C. T. (2019). Assessment of sustainable groundwater resources management using integrated environmental index: Case studies across Iran. Science of the Total Environment, 676, 792-810.	-	-	Approximated from Fig. 6 by Hosseini et al. (2019)
**Hamedan-Bahar Plain**	-	Ghobadi, A., Cheraghi, M., Sobhanardakani, S., Lorestani, B., Merrikhpour, H. (2020). Hydrogeochemical characteristics, temporal, and spatial variations for	Oroji, B. (2019). Groundwater vulnerability assessment with using GIS in Hamadan–Bahar plain, Iran. Applied Water Science, 9, 1-13.	Akhavan, S., Abedi-Koupai, J., Mousavi, S. F., Afyuni, M., Eslamian, S. S., Abbaspour, K. C. (2010). Application of SWAT model to investigate nitrate leaching in Hamadan–Bahar	Approximated from Fig. 1 by Ghobadi et al. (2020), Fig. 1 by Akhavan et al. (2010), and Fig. 1 by Oroji (2019)

Aquifer system	Broader aquifer system	Reference A	Reference B	Reference C	Steps to delineate
		evaluation of groundwater quality of Hamedan–Bahar Plain as a major agricultural region, West of Iran. Environmental Earth Sciences, 79, 1-16.		Watershed, Iran. Agriculture, Ecosystems & Environment, 139, 675-688.	
**Hamlin Valley**	-	Hurlow, H. A. (2014). Hydrogeologic studies and groundwater monitoring in Snake Valley and adjacent hydrographic areas, west-central Utah and east-central Nevada (Vol. 135). Utah Geological Survey Bulletin 135, 272 pp.	-	-	Approximated from map on page 6 of Hurlow (2014)
**Hansel and North Rozel Flat**	-	Lopes, T.J., Evetts. D.M. (2005). Ground-Water pumpage and artificial recharge estimates for calendar year 2000 and average annual natural recharge and interbasin flow by hydrographic area, Nevada. Accessed April 25, 2022 via https://pubs.usgs.gov/sir/2004/5239/sir2004-5239.pdf	Harrill, J.R., Prudic, D.E. (1998). Aquifer systems in the Great Basin region of Nevada, Utah, and adjacent states; summary report. US Geological Survey Professional Paper 1409-A, 75 pp. Accessed April 26, 2022 via https://pubs.usgs.gov/pp/1409a/report.pdf	-	Approximated from Fig. 1 by Lopes and Evetts (2005), and Fig. 2 by Harrill and Prudic (1998). Well completion records, physiography and drainage were used to delinate areas.
**Harasm Basin**	-	Hosseini, S. M., Parizi, E., Ataie-Ashtiani, B., Simmons, C. T. (2019). Assessment of sustainable groundwater resources management using integrated environmental index: Case studies across Iran. Science of the Total Environment, 676, 792-810.	-	-	Approximated from Fig. 6 by Hosseini et al. (2019)
**Harat Basin**	-	Hosseini, S. M., Parizi, E., Ataie-Ashtiani, B., Simmons, C. T. (2019).	-	-	Approximated from Fig. 6 by Hosseini et al. (2019)

Aquifer system	Broader aquifer system	Reference A	Reference B	Reference C	Steps to delineate
		Assessment of sustainable groundwater resources management using integrated environmental index: Case studies across Iran. Science of the Total Environment, 676, 792-810.			
**Hasdo-Arand Basin**	Son-Mahandai Valley Basins	Chowdari, S., Singh, B., Rao, B. N., Kumar, N., Singh, A. P., Chandrasekhar, D. V. (2017). Structural mapping based on potential field and remote sensing data, South Rewa Gondwana Basin, India. Journal of Earth System Science, 126, 1-27.	Dutta, P.K., Acharyya, S.K., Chakraborty, T., Jha, N., Khangar, R., Khasdeo, L., Misra, K.G. (2015). Resolving Kamthi-related problems in Gondwana stratigraphy of peninsular India. Indian Journal of Geosciences, 69, 85-102.	Central Ground Water Board (2012e). Aquifer Systems of Chhattisgarh. Central Ground Water Board Report, 73 pp. Accessed July 13, 2021 from http://cgwb.gov.in/AQM/Chhattisgarh.pdf	Approximated from Fig. 2 by Chowdari et al. (2017), Plates III and IV by Central Ground Water Board (2012e), and Fig. 1 by Dutta et al. (2015)
**Hashtgerd Plain**	-	Nosrati, K., Van Den Eeckhaut, M. (2012). Assessment of groundwater quality using multivariate statistical techniques in Hashtgerd Plain, Iran. Environmental Earth Sciences, 65, 331-344.	-	-	Approximated from Fig. 1 by Nosrati and Van Den Eeckhaut (2012)
**Hassanabad-Dehchah Plain**	-	Hosseni, M. S., Jahanshahi, R., Asadi, N., Nasiri, M. A. (2020). Qualitative study of groundwater resources in the Hassanabad-Dehchah, Northeast of Neyriz, Fars province. Hydrogeology, 5, 150-165.	-	-	Approximated from Fig. 1 by Hosseini et al. (2020)
**Hatfield Valley**	-	Maathuis, H. (2008). The quality of natural groundwaters in Saskatchewan. Saskatchewan Research Council Publication No. 12012-1E08, 86 pp.	-	-	Approximated from Fig. 6 of Maathuis (2008).

Aquifer system	Broader aquifer system	Reference A	Reference B	Reference C	Steps to delineate
		Accessed May 4, 2021 from https://www.wsask.ca/PageFiles/2978/The%20Quality%20of%20Natural%20Groundwaters%20in%20Saskatchewan,%20January%202008,%20Maathuis,%20H.,%20SRC%20pub.%20No.%2012012-1E08.pdf			
**Hawthorne Whiskey Flat**	-	Lopes, T.J., Evetts. D.M. (2005). Ground-Water pumpage and artificial recharge estimates for calendar year 2000 and average annual natural recharge and interbasin flow by hydrographic area, Nevada. Accessed April 25, 2022 via https://pubs.usgs.gov/sir/2004/5239/sir2004-5239.pdf	Harrill, J.R., Prudic, D.E. (1998). Aquifer systems in the Great Basin region of Nevada, Utah, and adjacent states; summary report. US Geological Survey Professional Paper 1409-A, 75 pp. Accessed April 26, 2022 via https://pubs.usgs.gov/pp/1409a/report.pdf	-	Approximated from Fig. 1 by Lopes and Evetts (2005), and Fig. 2 by Harrill and Prudic (1998). Well completion records, physiography and drainage were used to delinate areas.
**Heber Valley**	-	Roark, D.M., Holmes, W.F., Shlosar, H.K. (1991). Hydrology of Heber and Round Valleys, Wasatch County, Utah, with emphasis on simulation of ground-water flow in Heber Valley. U.S. Geological Survey Technical Publication 101, 101 pp. Accessed March 27, 2021 from https://waterrights.utah.gov/docSys/v920/y920/y9200009.pdf	-	-	Approximated from Fig. 1 of Roark et al. (1991)
**Helena Valley FIll**	-	Briar, D.W., Madison, J.P. (1992). Hydrogeology of the Helena Valley-fill aquifer system, west-central Montana. U.S.	-	-	Approximated from Fig. 1 of Briar and Madison (1992)

Aquifer system	Broader aquifer system	Reference A	Reference B	Reference C	Steps to delineate
		Geological Survey Water-Resources Investigations Report 92-4023, 97 pp. Accessed March 29, 2021 from https://pubs.usgs.gov/wri/1992/4023/report.pdf			
**Hengam Basin**	-	Hosseini, S. M., Parizi, E., Ataie-Ashtiani, B., Simmons, C. T. (2019). Assessment of sustainable groundwater resources management using integrated environmental index: Case studies across Iran. Science of the Total Environment, 676, 792-810.	-	-	Approximated from Fig. 6 by Hosseini et al. (2019)
**Hengchun Plain Karst Aquifer System**	-	Hsu, S. K. (1998). Plan for a groundwater monitoring network in Taiwan. Hydrogeology Journal, 6, 405-415.	Liu, C. W., Chen, J. F. (1996). The simulation of geochemical reactions in the Heng-Chun limestone formation, Taiwan. Applied Mathematical Modelling, 20, 549-558.	-	Approximated from Fig. 1 by Liu and Chen (1996), and Fig. 2 by Hsu (1998)
**Hermanus Coastal Aquifer**	-	Campbell, E. E., Parker-Nance, T., Bate, G. C. (1992). A compilation of information on the magnitude, nature and importance of coastal aquifers in Southern Africa. Water Research Commission Report No. 370/1/92, 193 pp. Accessed October 14, 2021 from http://www.wrc.org.za/wp-content/uploads/mdocs/370-1-92.pdf	-	-	Approximated from Fig. 3 on pp. 36 of Campbell et al. (1992)

Aquifer system	Broader aquifer system	Reference A	Reference B	Reference C	Steps to delineate
**Hertfordshire and Cambridgeshire Chalk Aquifer**	Chalk Aquifer System	MacDonald, A.M., Allen, D.J. (2001). Aquifer properties of the Chalk of England. Quarterly Journal of Engineering Geology and Hydrogeology, 34, 371-384.	-	-	Approximated from Fig. 8 by MacDonald and Allen (2001)
**Hesaruiyeh Basin**	-	Hosseini, S. M., Parizi, E., Ataie-Ashtiani, B., Simmons, C. T. (2019). Assessment of sustainable groundwater resources management using integrated environmental index: Case studies across Iran. Science of the Total Environment, 676, 792-810.	-	-	Approximated from Fig. 6 by Hosseini et al. (2019)
**Hetao Basin**	-	Guo, H., Zhang, B., Li, Y., Berner, Z., Tang, X., Norra, S., Stüben, D. (2011). Hydrogeological and biogeochemical constrains of arsenic mobilization in shallow aquifers from the Hetao basin, Inner Mongolia. Environmental Pollution, 159, 876-883.	-	-	Approximated from Fig. 1 by Guo et al. (2011)
**Hidalgo Aquifer**	-	CONAGUA (2015p). Actualización de la disponibilidad media anual de agua en el acuífero Hidalgo (3202), Estado de Zacatecas. 27 pp. Accessed March 7, 2022 via https://www.gob.mx/cms/uploads/attachment/file/104507/DR_3202.pdf	-	-	Approximated from Fig. 1 by CONAGUA (2015p)
**Hill Country**	Edwards-Trinity	Bruun, B., Jackson, K., Lake, P., Walker, J. (2016). Texas Aquifers Study.	Barker, R. A., Ardis, A. F. (1996). Hydrogeological framework of the Edwards-	-	Approximated from Fig. 6-11 by Bruun et al. (2016) and Fig. 3 by Barker and Ardis (1996).

Aquifer system	Broader aquifer system	Reference A	Reference B	Reference C	Steps to delineate
	Aquifer System	Texas Water Development Board Report. 336 pp. Accessed April 1, 2021 from https://www.twdb.texas.gov/groundwater/docs/studies/TexasAquifersStudy_2016.pdf#page=89	Trinity aquifer system, west-central Texas. U.S. Geological Survey Professional Paper 1421-B, 76 pp. Accessed April 1, 2021 from https://pubs.usgs.gov/pp/1421b/report.pdf		
**Hirakud Command Area Endogenous Rock Aquifers**	-	Dhar, A., Sahoo, S., Mandal, U., Dey, S., Bishi, N., Kar, A. (2015). Hydro-environmental assessment of a regional ground water aquifer: Hirakud command area (India). Environmental Earth Sciences, 73, 4165-4178.	Sahoo, S., Dhar, A., Kar, A., Chakraborty, D. (2016). Index-based groundwater vulnerability mapping using quantitative parameters. Environmental Earth Sciences, 75, 522.	Samantaray, S., Rath, A., Swain, P. C. (2017). Conjunctive Use of Groundwater and Surface Water in a Part of Hirakud Command Area. International Journal of Engineering and Technology (IJET), 9, 3002-3010.	Approximated from Fig. 1 by Dhar et al. (2015), Fig. 1 by Sahoo et al. (2016), and Fig. 1 by Samantaray et al. (2017)
**Hiroshima Plain**	-	Tosaki, Y., Morikawa, N., Kazahaya, K., Tsukamoto, H., Togo, Y.S., Sato, T., Takahashi, H.A., Takahashi, M., Inamura, A. (2017). Deep incursion of seawater into the Hiroshima Granites during the Holocene transgression: Evidence from 36Cl age of saline groundwater in the Hiroshima area, Japan. Geochemical Journal, 51, 263-275.	-	-	Approximated from Fig. 1b by Tosaki et al. (2017)
**Hohhot Basin**	-	Smedley, P. L., Zhang, M., Zhang, G., Luo, Z. (2003). Mobilisation of arsenic and other trace elements in fluviolacustrine aquifers of the Huhhot Basin, Inner Mongolia. Applied Geochemistry, 18, 1453-1477.	-	-	Approximated from Fig. 1 by Smedley et al. (2003)

Aquifer system	Broader aquifer system	Reference A	Reference B	Reference C	Steps to delineate
**Holbox Fracture Zone**	Yucatan Peninsula	Bauer-Gottwein, P., Gondwe, B. R., Charvet, G., Marín, L. E., Rebolledo-Vieyra, M., Merediz-Alonso, G. (2011). the Yucatán Peninsula karst aquifer, Mexico. Hydrogeology Journal, 19, 507-524.	Munro-Stasiuk, M.J., Manahan, T. K. (2010). Investigating ancient Maya agricultural adaptation through ground penetrating radar (GPR) analysis ofkarst terrain, Northern Yucatán, Mexico. Acta Carsologica, 39, 123-36.	-	Approximated from Fig. 2 by Bauer-Gottwein et al. (2011), and from Fig. 2 by Munro-Stasiuk and Manahan (2010).
**Hollywood Basin**	Los Angeles Basin**	Land, M., Reichard, E.G., Crawford, S.M., Everett, R.R., Newhouse, M.W., Williams, C.F. (2004). Ground-water Quality of Coastal Aquifer Systems in the West Coast Basin, Los Angeles County, California, 1999–2002. U.S. Geological Survey Scientific Investigations Report 2004-5067, 88 pp. Accessed February 15, 2021 from https://pubs.usgs.gov/sir/2004/5067/sir2004-5067.pdf	Fram, M. S., Belitz, K. (2008). Groundwater quality in the Coastal Los Angeles Basin, California; United States Geological Survey Fact Sheet 2012-3096. Accessed February 14, 2021 from https://pubs.er.usgs.gov/publication/70039952.	Reichard, E. G., Land, M., Crawford, S.M., Johnson, T.D., Everett, R.R., Kulshan, T.V., Ponti, D.J., Halford, K.L., Johnson, T.A., Paybins, K.S., Nishikawa, T. (2003). Geohydrology, Geochemistry, and Ground-Water Simulation Optimization of the Central and West Coast Basins, Los Angeles County, California. USGS Numbered Series, 2003–4065. Accessed November 28, 2021 from https://pubs.usgs.gov/wri/wrir034065/wrir034065.pdf	Approximated from Fig. 1 by Land et al. (2004), Fig. 6 by Reichard et al. (2003) and map on page 1 of Fram and Belitz (2008)
**Homand Basin**	-	Hosseini, S. M., Parizi, E., Ataie-Ashtiani, B., Simmons, C. T. (2019). Assessment of sustainable groundwater resources management using integrated environmental index: Case studies across Iran. Science of the Total Environment, 676, 792-810.	-	-	Approximated from Fig. 6 by Hosseini et al. (2019)
**Honey Lake Valley**	-	Handman, E. H., Londquist, C. J., Maurer, D.K. (1990). Ground-water resources of	Rose, T.P., Davisson, M.L., Hudson, G.B., Varian, A.R. (1997). Environmental	Mayo, A. L., Henderson, R. M., Tingey, D., Webber, W. (2010). Chemical evolution	Approximated from Fig. 1 of Handman et al. (1990), Fig. 1 by

Aquifer system	Broader aquifer system	Reference A	Reference B	Reference C	Steps to delineate
		Honey Lake valley, Lassen County, California, and Washoe County, Nevada. Water-Resources Investigations Report 90-4050, 119 pp. Accessed March 21, 2021 from https://pubs.usgs.gov/wri/1990/4050/report.pdf	isotope investigation of groundwater flow in the Honey Lake Basin, California and Nevada. Department of Energy Report UCRL-ID-127978 ON: DE98051049. 72 pp. Accessed April 18, 2022 via https://www.osti.gov/servlets/purl/620597	of shallow playa groundwater in response to post-pluvial isostatic rebound, Honey Lake Basin, California–Nevada, USA. Hydrogeology Journal, 18, 725-747.	Mayo et al. (2010), and Fig. 1 by Rose et al. (1997)
**Hot Creek Valley**	-	Lopes, T.J., Evetts. D.M. (2005). Ground-Water pumpage and artificial recharge estimates for calendar year 2000 and average annual natural recharge and interbasin flow by hydrographic area, Nevada. Accessed April 25, 2022 via https://pubs.usgs.gov/sir/2004/5239/sir2004-5239.pdf	Harrill, J.R., Prudic, D.E. (1998). Aquifer systems in the Great Basin region of Nevada, Utah, and adjacent states; summary report. US Geological Survey Professional Paper 1409-A, 75 pp. Accessed April 26, 2022 via https://pubs.usgs.gov/pp/1409a/report.pdf	-	Approximated from Fig. 1 by Lopes and Evetts (2005), and Fig. 2 by Harrill and Prudic (1998). Well completion records, physiography and drainage were used to delinate areas.
**Houlong Alluvial Plain Aquifer**	Hsinchu-Miaoli Coastal Aquifer System	Hsu, S. K. (1998). Plan for a groundwater monitoring network in Taiwan. Hydrogeology Journal, 6, 405-415.	Han, Y. L., Kuo, M. T., Fan, K. C., Chiang, C. J., Lee, Y. P. (2006). Radon distribution in groundwater of Taiwan. Hydrogeology Journal, 14, 173-179.	Lu, H. Y., Peng, T. R., Liu, T. K., Wang, C. H., Huang, C. C. (2006). Study of stable isotopes for highly deformed aquifers in the Hsinchu-Miaoli area, Taiwan. Environmental Geology, 50, 885-898.	Approximated from Fig. 2 by Hsu (1998), Fig. 1 by Han et al. (2006), and descriptions in the final paragraph of the subsection entitled "Study area" and Fig. 1 by Lu et al. (2006)
**Houston-Galveston Area**	Gulf Coast Regional Aquifer System	Weiss, J. S. (1990). Geohydrologic units of the coastal lowlands aquifer system, south-central United States. U.S. Geological Survey Regional aquifer-systems analysis. 42 pp. Accessed March 30, 2021 from https://pubs.usgs.gov/pp/1416c/report.pdf	Braun, C.L., Ramage, J.K., Shah, S.D. (2019). Status of groundwater-level altitudes and long-term groundwater-level changes in the Chicot, Evangeline, and Jasper aquifers, Houston-Galveston region, Texas, 2019: U.S. Geological Survey Scientific Investigations Report	-	Outer margin of Gulf Coast Regional Aquifer System approximated from Fig. 2 of Weiss (1990).

Aquifer system	Broader aquifer system	Reference A	Reference B	Reference C	Steps to delineate
			2019–5089, 18 pp. https://pubs.usgs.gov/sir/2019/5089/sir20195089.pdf		
**Howard East Aquifer**	-	Nolan, S., Tan, P.-L., Cox, M. (2010). Collaborative Water Planning: Groundwater Visualisation Tool Guide. Charles Darwin University, Darwin, 38 pp. Accessed October 7, 2021 from http://www.nespnorthern.edu.au/wp-content/uploads/2016/02/GVT_Griffith-Uni_13-May-2010-with-corrections1.pdf	-	-	Approximated from Fig. 1 by Nolan et al. (2010)
**Hualapai Flat**	-	Lopes, T.J., Evetts. D.M. (2005). Ground-Water pumpage and artificial recharge estimates for calendar year 2000 and average annual natural recharge and interbasin flow by hydrographic area, Nevada. Accessed April 25, 2022 via https://pubs.usgs.gov/sir/2004/5239/sir2004-5239.pdf	Harrill, J.R., Prudic, D.E. (1998). Aquifer systems in the Great Basin region of Nevada, Utah, and adjacent states; summary report. US Geological Survey Professional Paper 1409-A, 75 pp. Accessed April 26, 2022 via https://pubs.usgs.gov/pp/1409a/report.pdf	-	Approximated from Fig. 1 by Lopes and Evetts (2005), and Fig. 2 by Harrill and Prudic (1998). Well completion records, physiography and drainage were used to delinate areas.
**Hualapai Valley Basin**	-	Tillman, F.D, Garner, B.D., Truini, M. (2013). Preliminary groundwater flow model of the basin-fill aquifers in Detrital, Hualapai, and Sacramento Valleys, Mohave County, northwestern Arizona. U.S. Geological Survey Scientific Investigations Report 2013–5122, 52 pp. http://pubs.usgs.gov/sir/2013/5122/	Knight, J.E., Gungle, B., Kennedy, J.R. (2021). Assessing potential groundwater-level declines from future withdrawals in the Hualapai Valley, northwestern Arizona: US Geological Survey Scientific Investigations Report, 74 pp. Accessed May 5, 2022 via https://pubs.usgs.gov/sir/2021/5077/sir20215077.pdf	-	Approximated from Fig. 2 by Tillman et al. (2013).

Aquifer system	Broader aquifer system	Reference A	Reference B	Reference C	Steps to delineate
**Huatulco Aquifer**	-	CONAGUA (2020hi). Actualización de la disponibilidad media anual de agua en el acuífero Huatulco (2011), estado de Oaxaca. 29 pp. Accessed March 29, 2022 via https://sigagis.conagua.gob.mx/gas1/Edos_Acuiferos_18/oaxaca/DR_2011.pdf	-	-	Approximated from physiography, well completion data, and Fig. 1 by CONAGUA (2020hi)
**Huerva-Perejiles and Monreal-Calamocha Aquifers**	-	Carceller-Layel, T., Costa-Alandí, C., Coloma-López, P., García-Vera, M. Á., & San Román-Saldaña, J. (2007). Groundwater in the central sector of the Ebro Basin. Water Resources Development, 23, 165-187.	-	-	Approximated from Fig. 3 by Carceller-Layel et al. (2007); portions of aquifers numbered (in Fig. 3 by Carceller-Layel et al., 2007) 81-90 included
**Illinoian Till Plain**	Central Lowland Till Plain	Brockman, S. (1998). Physiographic regions of Ohio. Ohio Department of Natural Resources, Division of Geological Survey map. Accessed April 12, 20221 from https://www.epa.state.oh.us/portals/27/SIP/Nonattain/F2-physiographic_regions_of_Ohio.pdf	Fenneman, N.M., Johnson, D.W. (1946). Physiographic divisions of the conterminous United States. U.S. Geological Survey map, 1:7,000,000 Scale.	-	Broader Till Plain physiographic region approximated from Fenneman and Johnson (1946). Illinoian Till Plain approximated from the map by Brockman (1998).
**Imamzadeh Jafar Gachsaran Plain**	-	Avand, M., Ekhtesasi, M. R. (2020). The effect of geological formations on the quality and quantity of groundwater (case study: Imamzadeh Jafar Gachsaran plain). Sustainable Earth Review, 1, 1-6.	-	-	Approximated from Fig. 1 by Avand and Ekhtesasi (2020).
**Imperial Valley**	-	California Department of Water Resources (2003a).	-	-	Approximated from map on page 2 of California Department of Water

Aquifer system	Broader aquifer system	Reference A	Reference B	Reference C	Steps to delineate
		Basin Boundaries Description - Imperial Valley. Bulletin 118, 4 pp. Accessed April 27, 2022 via https://water.ca.gov/-/media/DWR-Website/Web-Pages/Programs/Groundwater-Management/Bulletin-118/Files/2003-Basin-Descriptions/7_030_ImperialValley.pdf			Resources (2003a), well completion data and physiography
**Indian Springs Valley**	-	Burbey, T. J. (1997). Hydrogeology and potential for ground-water development, carbonate-rock aquifers in southern Nevada and southeastern California. US Geological Survey Water-Resources Investigations Report 95-4168, 70 pp. Accessed March 10, 2021 from https://pubs.usgs.gov/wri/1995/4168/report.pdf	-	-	Approximated from Fig. 13 of Burbey (1997)
**Indian Wells Valley**	-	California Department of Water Resources (2015b). California's groundwater update 2013 - Chapter 11: South Lahontan Hydrologic Region. California Department of Water Resources Report, 80 pp. Accessed April 27, 2022 via https://water.ca.gov/-/media/DWR-Website/Web-Pages/Programs/Groundwater-Management/Data-and-Tools/Files/Statewide-Reports/California-Groundwater-Update-2013/California-	Chapman, J. B., Thomas, J. M., Garner, C. (2022). Groundwater recharge timing based on 14C and 2H within Indian Wells Valley, California, USA. Applied Geochemistry, 105268.	-	Approximated from Fig. 11-2 by California Department of Water Resources (2015b) and Fig. 1 by Chapman et al. (2022)

Aquifer system	Broader aquifer system	Reference A	Reference B	Reference C	Steps to delineate
		Groundwater-Update-2013---Chapter-11---South-Lahontan.pdf			
**Interlake Carbonate Rock and Winnipeg Aquifers**	Manitoba Carbonate Aquifer System	Ferguson, G.A., Betcher, R.N., Grasby, S.E. (2007). Hydrogeology of the Winnipeg formation in Manitoba, Canada. Hydrogeology Journal, 15, 573.	Betcher, R.N. (1985). Groundwater Availability Map Series - Selkirk Area (62-I). Manitoba Department of Natural Resoureces map. Accessed May 5, 2021 from https://www.gov.mb.ca/water/pubs/maps/water/1986_betcher_groundwater_availability_map_series_selkirk.zip	Thorleifson, L.H., Betcher, R., Birks, J., Boyle, D., Cherry, A., Clark, I., Desbarats, A.J., Edwards, T., Farrell, D., Grasby, S. and Hinton, M., (1998). Hydrogeology and hydrogeochemistry of the Red River Valley/Interlake region of Manitoba. Manitoba Energy and Mines, Minerals Division Report of Activities, 172-185.	Interlake Region approximated from Fig. 1 by Ferguson et al. (2007). Boundary defining where the Winnipeg clastic formations become a key aquifer derives from Betcher (1985) in the southern portion of the delineated area. Groundwater in much of the Interlake Region is fresh (see Fig. 1 by Thorleifson et al., 1998).
**Interlake Carbonate Rock Aquifers**	Manitoba Carbonate Aquifer System	Grasby, S. E., Betcher, R. N. (2002). Regional hydrogeochemistry of the carbonate rock aquifer, southern Manitoba. Canadian Journal of Earth Sciences, 39, 1053-1063.	Betcher, R.N. (1985). Groundwater Availability Map Series - Selkirk Area (62-I). Manitoba Department of Natural Resoureces map. Accessed May 5, 2021 from https://www.gov.mb.ca/water/pubs/maps/water/1986_betcher_groundwater_availability_map_series_selkirk.zip	Thorleifson, L.H., Betcher, R., Birks, J., Boyle, D., Cherry, A., Clark, I., Desbarats, A.J., Edwards, T., Farrell, D., Grasby, S. and Hinton, M., (1998). Hydrogeology and hydrogeochemistry of the Red River Valley/Interlake region of Manitoba. Manitoba Energy and Mines, Minerals Division Report of Activities, 172-185.	Interlake Region approximated from Fig. 1 by Grasby and Betcher (2002). Boundary defining where the Winnipeg clastic formations become a key aquifer derives from Betcher (1985) in the southern portion of the delineated area. Groundwater in much of the Interlake Region is fresh (see Fig. 1 by Thorleifson et al., 1998).
**Intermediate Aquifer**	Floridan Aquifer System	Miller, J.A. (1990). Ground Water Atlas of the United States: Segment 6, Alabama, Florida, Georgia, South Carolina. U.S. Geological Survey Hydrologic Atlas 730-G, 30 pp. Accessed April 5, 2021 from	Knochenmus, L.A. (2006). Regional Evaluation of the Hydrogeologic Framework, Hydraulic Properties, and Chemical Characteristics of the Intermediate Aquifer System Underlying Southern West-Central Florida. U.S. Geological Survey Scientific	-	Approximated Fig. 1 of Knochenmus (2006); aquifer outline also displayed in Figs. 9 and 42 of Miller (1990)

Aquifer system	Broader aquifer system	Reference A	Reference B	Reference C	Steps to delineate
		https://www.nrc.gov/docs/ML1706/ML17060B027.pdf	Investigations Report 2006-5013, 52 pp. Accessed April 5, 2021 from https://pubs.usgs.gov/sir/2006/5013/pdf/2006-5013.pdf		
**Iranshahr Plain**	Jazmurian Depression	Golchin, I., Moghaddam, M. A. (2016). Hydro-geochemical characteristics and groundwater quality assessment in Iranshahr plain aquifer, Iran. Environmental Earth Sciences, 75, 317.	Shalyari, N., Alinejad, A., Hashemi, A. H. G., RadFard, M., Dehghani, M. (2019). Health risk assessment of nitrate in groundwater resources of Iranshahr using Monte Carlo simulation and geographic information system (GIS). MethodsX, 6, 1812-1821.	Rezaei, A., Hassani, H., Hassani, S., Jabbari, N., Mousavi, S. B. F., Rezaei, S. (2019). Evaluation of groundwater quality and heavy metal pollution indices in Bazman basin, southeastern Iran. Groundwater for Sustainable Development, 9, 100245.	Approximated from Fig. 1 by Golchin and Moghaddam (2016) and Fig. 1 by Shalyari et al. (2019). Broader Jazmurian Depression from Fig. 2 by Rezaei et al. (2019)
**Iroquois Till Plains**	Central Lowland Till Plain	Fenneman, N.M., Johnson, D.W. (1946). Physiographic divisions of the conterminous United States. U.S. Geological Survey map, 1:7,000,000 Scale.	Gray, H.H. (2001). Map of Indiana showing physiographic divisions. Indiana Geological Survey Miscellaneous Map 69, 15 pp. Accessed April 12, 2021 from https://igws.indiana.edu/ReferenceDocs/Maps/PhysiographicRegions.pdf	-	Broader Till Plain physiographic region approximated from Fenneman and Johnson (1946). "Iroquois Till Plain" delineated from Gray (2001).
**Irwin Basin**	-	Mendez, G.O., Christensen, A.H. (1997). Regional water table (1996) and water-level changes in the Mojave River, and Morongo, and the Fort Irwin ground-water basins, San Bernardino County, California. US Geological Survey Water-Resources Investigations Report 97-4160, 38 pp. Accessed April 27, 2022 via https://pubs.usgs.gov/wri/1997/4160/report.pdf	-	-	Approximated from Fig. 1 and Plate 1 by Mendez and Christensen (1997)

Aquifer system	Broader aquifer system	Reference A	Reference B	Reference C	Steps to delineate
**Isin Basin**	-	Bazrafshan, O., Parandin, F., Farokhzadeh, B. (2016). Assessment of hydro-meteorological drought effects on groundwater resources in Hormozgan region-South of Iran. Ecopersia, 4, 1569-1584.	-	-	Approximated from map on page 1571 of Bazrafshan et al. (2016)
**Islam Abad Plain**	-	Taheri, K., Missimer, T.M., Amini, V., Bahrami, J., Omidipour, R. (2020). A GIS-expert-based approach for groundwater quality monitoring network design in an alluvial aquifer: a case study and a practical guide. Environmental Monitoring and Assessment, 192, 1-20.	-	-	Approximated from Fig. 1c by Taheri et al. (2020)
**Ivanhoe and Packsaddle Plains**	-	Paul, B., Raper, P., Simons, J., Stainer, G., George, R. (2011). Weaber Plain aquifer test results. Government of Western Australia, Department of Agriculture and Food Resource Management Technical Report 367, 48 pp. Accessed May 8, 2023 via https://library.dpird.wa.gov.au/cgi/viewcontent.cgi?article=1362&context=rmtr	-	-	Approximated from Fig. 1 by Paul et al. (2011)
**Ivanhoe Plains**	Murray Basin	Herczeg, A. L., Dogramaci, S. S., Leaney, F. W. J. (2001). Origin of dissolved salts in a large, semi-arid groundwater system: Murray Basin, Australia. Marine and Freshwater Research, 52, 41-52.	Cook, P. G., Jolly, I. D., Leaney, F. W. J. (2001). Groundwater recharge in the Mallee region, and salinity implications for the Murray River: A review. CSIRO Land and Water Report, 133 pp. Accessed	Murray-Darling Basin Commission (1999). Murray-Darling Basin Groundwater - a resource for the future, Murray-Darling Basin Commission, Canberra. Accessed July 21, 2021 from	Approximated from Fig. 1 by Herczeg et al. (2001), Fig. 1.1 by Cook et al. (2001), and Fig. 6 by Murray-Darling Basin Commission (1999)

Aquifer system	Broader aquifer system	Reference A	Reference B	Reference C	Steps to delineate
			July 21, 2021 from https://publications.csiro.au/rpr/download?pid=procite:ef08494d-43a2-4dae-bda4-3d72a62e673f&dsid=DS1	https://www.mdba.gov.au/sites/default/files/archived/mdbc-GW-reports/2173_GW_a_resource_for_the_future.pdf	
**Ivanpah Valley**	-	Lopes, T.J., Evetts. D.M. (2005). Ground-Water pumpage and artificial recharge estimates for calendar year 2000 and average annual natural recharge and interbasin flow by hydrographic area, Nevada. Accessed April 25, 2022 via https://pubs.usgs.gov/sir/2004/5239/sir2004-5239.pdf	Harrill, J.R., Prudic, D.E. (1998). Aquifer systems in the Great Basin region of Nevada, Utah, and adjacent states; summary report. US Geological Survey Professional Paper 1409-A, 75 pp. Accessed April 26, 2022 via https://pubs.usgs.gov/pp/1409a/report.pdf	-	Approximated from Fig. 1 by Lopes and Evetts (2005), and Fig. 2 by Harrill and Prudic (1998). Well completion records, physiography and drainage were used to delinate areas.
**Izad Khast Basin**	-	Hosseini, S. M., Parizi, E., Ataie-Ashtiani, B., Simmons, C. T. (2019). Assessment of sustainable groundwater resources management using integrated environmental index: Case studies across Iran. Science of the Total Environment, 676, 792-810.	-	-	Approximated from Fig. 6 by Hosseini et al. (2019)
**Izeh Plain**	-	Kalantari, N., Pawar, N. J., Keshavarzi, M. R. (2009). Water resource management in the intermountain Izeh Plain, Southwest of Iran. Journal of Mountain Science, 6, 25-41.	-	-	Approximated from Fig. 1 by Kalantari et al. (2009)
**Jackson Hole Aquifer**	-	Wright, P.R. (2013). Hydrogeology and water quality in the Snake River alluvial aquifer at Jackson	-	-	Approximated from Fig. 3 of Wright (2013).

Aquifer system	Broader aquifer system	Reference A	Reference B	Reference C	Steps to delineate
		Hole Airport, Jackson, Wyoming, water years 2011 and 2012: U.S. Geological Survey Scientific Investigations Report 2013–5184, 56 pp. Accessed March 29, 2021 from https://pubs.usgs.gov/sir/2013/5184/pdf/sir2013-5184.pdf			
**Jacumba Valley**	-	California Department of Water Resources (2015a). California's groundwater update 2013 - Chapter 12: Colorado River Hydrologic Region. California Department of Water Resources Report, 76 pp. Accessed April 27, 2022 via https://water.ca.gov/-/media/DWR-Website/Web-Pages/Programs/Groundwater-Management/Bulletin-118/Files/Statewide-Reports/GWU2013_Ch12_ColoradoRiver_Final.pdf	-	-	Approximated from Fig. 12-2 by California Department of Water Resources (2015a)
**Jaghin-Tokahor Plain**	-	Moslemi, H. (2019). Assessment of Groundwater Crisis in Arid and Semiarid Areas (Case Study: Jaghin and Tokahor Plain). Irrigation Sciences and Engineering, 42, 31-46.	-	-	Approximated from Moslemi (2019)
**Jahrom Basin**	-	Hosseini, S. M., Parizi, E., Ataie-Ashtiani, B., Simmons, C. T. (2019). Assessment of sustainable groundwater resources management using integrated environmental	Parizi, E., Hosseini, S. M., Ataie-Ashtiani, B., Simmons, C. T. (2020). Normalized difference vegetation index as the dominant predicting factor of groundwater recharge in	-	Approximated from Fig. 6 by Hosseini et al. (2019) and Fig. 2 by Parizi et al. (2020)

Aquifer system	Broader aquifer system	Reference A	Reference B	Reference C	Steps to delineate
		index: Case studies across Iran. Science of the Total Environment, 676, 792-810.	phreatic aquifers: case studies across Iran. Scientific Reports, 10, 17473.		
**Jajarm Plain**	-	Javanbakht, M., Asadi, V., Dabiri, R. (2020). Evaluation of Hydrogeochemical Characteristics and Evolutionary Process of Groundwater in Jajarm Plain, Northeastern Iran. Environment and Water Engineering, 6, 206-218.	-	-	Approximated from Fig. 1 by Javanbakht et al. (2020)
**Jam Basin**	-	Hosseini, S. M., Parizi, E., Ataie-Ashtiani, B., Simmons, C. T. (2019). Assessment of sustainable groundwater resources management using integrated environmental index: Case studies across Iran. Science of the Total Environment, 676, 792-810.	-	-	Approximated from Fig. 6 by Hosseini et al. (2019)
**Jamiltepec Aquifer**	-	Mejía-González, M. Á., González-Hita, L., Espinoza-Ayala, J., González-Verdugo, J. A. (2012). Determinación de las aportaciones de agua dulce a las lagunas costeras Chacahua y Salina Grande, Oaxaca, México, por medio de isótopos ambientales. Tecnología y ciencias del agua, 3(3), 53-64.	-	-	Approximated from Fig. 1 by Mejía-González et al. (2012)
**Jangal Plain**	-	Nematollahi, M. J., Ebrahimi, P., Ebrahimi, M. (2016). Evaluating hydrogeochemical	-	-	Approximated from Fig. 1 by Nematollahi et al. (2016)

Aquifer system	Broader aquifer system	Reference A	Reference B	Reference C	Steps to delineate
		processes regulating groundwater quality in an unconfined aquifer. Environmental Processes, 3(4), 1021-1043.			
**Javim Banaruyeh Basin**	-	Hosseini, S. M., Parizi, E., Ataie-Ashtiani, B., Simmons, C. T. (2019). Assessment of sustainable groundwater resources management using integrated environmental index: Case studies across Iran. Science of the Total Environment, 676, 792-810.	-	-	Approximated from Fig. 6 by Hosseini et al. (2019)
**Jayezan Basin**	-	Hosseini, S. M., Parizi, E., Ataie-Ashtiani, B., Simmons, C. T. (2019). Assessment of sustainable groundwater resources management using integrated environmental index: Case studies across Iran. Science of the Total Environment, 676, 792-810.	-	-	Approximated from Fig. 6 by Hosseini et al. (2019)
**Jerez Aquifer**	-	CONAGUA (2021a). Acuíferos (nacional). Webpage accessed March 8, 2022 from http://sina.conagua.gob.mx/sina/tema.php?tema=acuiferos&ver=mapa&o=1&n=nacional	-	-	Approximated from aquifer boundaries defined by CONAGUA (2021a) web portal, physiography and drainage patterns, and well completion data.
**Jhonggang Alluvial Plain**	Hsinchu-Miaoli Coastal Aquifer System	Hsu, S. K. (1998). Plan for a groundwater monitoring network in Taiwan. Hydrogeology Journal, 6, 405-415.	Han, Y. L., Kuo, M. T., Fan, K. C., Chiang, C. J., Lee, Y. P. (2006). Radon distribution in groundwater of Taiwan. Hydrogeology Journal, 14, 173-179.	Lu, H. Y., Peng, T. R., Liu, T. K., Wang, C. H., Huang, C. C. (2006). Study of stable isotopes for highly deformed aquifers in the Hsinchu-Miaoli area,	Approximated from Fig. 2 by Hsu (1998), Fig. 1 by Han et al. (2006), and descriptions in the final paragraph of the subsection entitled "Study area" and Fig. 1 by Lu et al. (2006)

Aquifer system	Broader aquifer system	Reference A	Reference B	Reference C	Steps to delineate
				Taiwan. Environmental Geology, 50, 885-898.	
**Jianghan Plain**	-	Gan, Y., Wang, Y., Duan, Y., Deng, Y., Guo, X., & Ding, X. (2014). Hydrogeochemistry and arsenic contamination of groundwater in the Jianghan Plain, central China. Journal of Geochemical Exploration, 138, 81-93.	Zhou, Y., Wang, Y., Li, Y., Zwahlen, F., Boillat, J. (2013). Hydrogeochemical characteristics of central Jianghan Plain, China. Environmental Earth Sciences, 68, 765-778.	-	Approximated from Fig. 1 by Gan et al. (2014), Fig. 1 by Zhou et al. (2014),
**Jiangsu Coastal Plain**	Huaihe River Basin	Sun, X., Lin, J., Gu, W., Min, X., Han, J., Dai, Y., Liu, P., Wang, S (2021). Analysis and evaluation of the renewability of the deep groundwater in the Huaihe River Basin, China. Environmental Earth Sciences, 80, 1-13.	Xu, N., Gong, J., Yang, G. (2018). Using environmental isotopes along with major hydro-geochemical compositions to assess deep groundwater formation and evolution in eastern coastal China. Journal of Contaminant Hydrology, 208, 1-9.	-	Approximated from Fig. 1 by Sun et al. (2021) and Fig. 1 by Xu et al. (2018)
**Jining Area**	-	Ong'or, B. T., Long-Cang, S. (2009). Groundwater overdraft and the impact of artificial recharge on groundwater quality in a cone of depression, Jining, China. Water International, 34, 468-483.	Shu, L. C., Liu, P. G., Ong'or, B. T. I. (2008). Environmental impact assessment using FORM and groundwater system reliability concept: case study Jining, China. Environmental geology, 55, 661-667.	-	Approximated from Fig. 1 by Ong'or and Long (2009), Fig. 1 by Shu et al. (2008)
**Jiroft Plain**	-	Hosseini, S. M., Parizi, E., Ataie-Ashtiani, B., Simmons, C. T. (2019). Assessment of sustainable groundwater resources management using integrated environmental index: Case studies across Iran. Science of the Total	Parizi, E., Hosseini, S. M., Ataie-Ashtiani, B., Simmons, C. T. (2020). Normalized difference vegetation index as the dominant predicting factor of groundwater recharge in phreatic aquifers: case studies across Iran.	-	Approximated from Fig. 6 by Hosseini et al. (2019) and Fig. 2 by Parizi et al. (2020)

Aquifer system	Broader aquifer system	Reference A	Reference B	Reference C	Steps to delineate
		Environment, 676, 792-810.	Scientific Reports, 10, 17473.		
**Jovin Plain**	-	Salehabadi, G. (2013). The effect of groundwater in plain Settlement in Jovin. Scientific-Research Quarterly of Geographical Data (SEPEHR), 22(SEPEHR), 30-34. Accessed June 2, 2021 from http://www.sepehr.org/article_25939.html?lang=en	-	-	Approximated from Fig. 1 by Salehabadi (2013)
**Juab Valley**	-	Thiros, S. A., Stolp, B. J., Hadley, H. K., Steiger, J. I. (1996). Hydrology and simulation of ground-water flow in Juab Valley, Juab County, Utah. Utah Department of Natural Resources, Division of Water Rights Technical Report. 100 pp. Accessed March 7, 2021 from https://waterrights.utah.gov/docSys/v920/y920/y920000j.pdf	-	-	Approximated from Fig. 1 of Thiros et al. (1996)
**Jumilla-Villena Aquifer**	-	Rupérez-Moreno, C., Senent-Aparicio, J., Martinez-Vicente, D., García-Aróstegui, J. L., Calvo-Rubio, F. C., Pérez-Sánchez, J. (2017). Sustainability of irrigated agriculture with overexploited aquifers: The case of Segura basin (SE, Spain). Agricultural Water Management, 182, 67-76.	-	-	Approximated from Fig. 1 by Rupérez-Moreno et al. (2017)
**Kadkan Basin**	-	Esmaeili-Vardanjani, M., Rasa, I., Yazdi, M., Pazand, K. (2016). The	-	-	Approximated from Fig. 1 by Esmaeili-Vardanjani et al. (2016)

Aquifer system	Broader aquifer system	Reference A	Reference B	Reference C	Steps to delineate
		hydrochemical assessment of groundwater resources in the Kadkan basin, Northeast of Iran. Carbonates and Evaporites, 31, 129-138.			
**Kafehtaghestan Basin**	-	Hosseini, S. M., Parizi, E., Ataie-Ashtiani, B., Simmons, C. T. (2019). Assessment of sustainable groundwater resources management using integrated environmental index: Case studies across Iran. Science of the Total Environment, 676, 792-810.	-	-	Approximated from Fig. 6 by Hosseini et al. (2019)
**Kahorestan Basin**	-	Bazrafshan, O., Parandin, F., Farokhzadeh, B. (2016). Assessment of hydro-meteorological drought effects on groundwater resources in Hormozgan region-South of Iran. Ecopersia, 4, 1569-1584.	-	-	Approximated from map on page 1571 of Bazrafshan et al. (2016)
**Kahria Aquifer**	-	Javadzadeh, H., Ataie-Ashtiani, B., Hosseini, S. M., Simmons, C. T. (2020). Interaction of lake-groundwater levels using cross-correlation analysis: A case study of Lake Urmia Basin, Iran. Science of The Total Environment, 729, 138822.	-	-	Approximated from Fig. 1 by Javadzadeh et al. (2020)
**Kaikoura Plain**	-	Sheppard (1995). The hydrogeology of the Kaikoura Plains, North Canterbury, New Zealand. PhD Dissertation, University of Canterbury. Accessed	White, P.A., Reeves, R.R. (2002). The volume of groundwater in New Zealand 1994 to 2001. Statistics New Zealand, Client Report 2002/79, 39	-	Approximated from Fig. 4.1 by Sheppard (1995) and Fig. 2 in White and Reeves (2002)

Aquifer system	Broader aquifer system	Reference A	Reference B	Reference C	Steps to delineate
		October 20, 2021 from https://ir.canterbury.ac.nz/handle/10092/17545	pp. Accessed October 20, 2021 from https://docs.niwa.co.nz/library/public/volume-of-groundwater-in-nz-2001%5B1%5D.pdf		
**Kalacho Plain**	-	Afshin, A. A., Motlagh, K. S. (2018). The study of sharp decline in groundwater in Kohgiluyeh and Boyer province with special attention to the Calacho plain-Dehdasht-iran. International Journal of Research Publications, 3, 7 pp. Accessed June 17, 2021 from https://ijrp.org/paper-detail/67To	-	-	Approximated from Fig. 2 by Afshin and Motlagh (2018).
**Kamfruz Alluvial Aquifer**	-	Naderi, M. (2020). Assessment of water security under climate change for the large watershed of Dorudzan Dam in southern Iran. Hydrogeology Journal, 28, 1553-1574.	-	-	Approximated from Fig. 13 by Naderi (2020).
**Kamyaran Plain**	-	Saadatmand, A., Noorollahi, Y., Yousefi, H., Mohammadi, A. (2021). Investigation, modeling and analysis of qualitative parameters of groundwater resources in Kurdistan's Kamyaran plain. Iranian journal of Ecohydrology, 8, 357-367.	-	-	Approximated from description by Mohammadi et al. (2021)
**Kangavar Aquifer**	-	Tizro, T. A., Voudouris, K. S., Kamali, M. (2014). Comparative study of step drawdown and constant discharge tests to	-	-	Approximated from Fig. 1 by Tizro et al. (2014)

Aquifer system	Broader aquifer system	Reference A	Reference B	Reference C	Steps to delineate
		determine the aquifer transmissivity: the Kangavar aquifer case study, Iran. Journal of Water Resource and Hydraulic Engineering, 3, 12-21.			
**Kashan Plain**	-	Jamshidzadeh, Z., Mirbagheri, S. A. (2011). Evaluation of groundwater quantity and quality in the Kashan Basin, Central Iran. Desalination, 270, 23-30.	Ghazifard, A., Moslehi, A., Safaei, H., Roostaei, M. (2016). Effects of groundwater withdrawal on land subsidence in Kashan Plain, Iran. Bulletin of Engineering Geology and the Environment, 75, 1157-1168.	Mirzavand, M., Ghasemieh, H., Sadatinejad, S. J., Bagheri, R. (2020). Delineating the source and mechanism of groundwater salinization in crucial declining aquifer using multi-chemo-isotopes approaches. Journal of Hydrology, 586, 124877.	Approximated from Fig. 3 by Jamshidzadeh and Mirbagheri (2011) and Fig. 2 by Ghazifard et al. (2016) and Fig. 2 by Mirzavand et al. (2020)
**Kashmar-Bardaskan Plain**	-	Saeidi, H., Lashkaripour, G., Ghafoori, M. (2020). Evaluation of land subsidence in Kashmar-Bardaskan plain, NE Iran. Iranian Journal of Earth Sciences, 12, 280-291.	-	-	Approximated from Fig. 1 by Saeidi et al. (2020)
**Kavar and Kavar-Maharloo Aquifers**	-	Hosseini, S. M., Parizi, E., Ataie-Ashtiani, B., Simmons, C. T. (2019). Assessment of sustainable groundwater resources management using integrated environmental index: Case studies across Iran. Science of the Total Environment, 676, 792-810.	Alizadeh, M. R., Nikoo, M. R., Rakhshandehroo, G. R. (2017). Hydro-environmental management of groundwater resources: a fuzzy-based multi-objective compromise approach. Journal of Hydrology, 551, 540-554.	-	Approximated from Fig. 6 by Hosseini et al. (2019) and Fig. 1 by Alizadeh et al. (2017)
**Kavir Allahabad Basin**	-	Hosseini, S. M., Parizi, E., Ataie-Ashtiani, B., Simmons, C. T. (2019). Assessment of sustainable groundwater resources management using	-	-	Approximated from Fig. 6 by Hosseini et al. (2019)

Aquifer system	Broader aquifer system	Reference A	Reference B	Reference C	Steps to delineate
		integrated environmental index: Case studies across Iran. Science of the Total Environment, 676, 792-810.			
**Kavir Daranjir Basin**	-	Hosseini, S. M., Parizi, E., Ataie-Ashtiani, B., Simmons, C. T. (2019). Assessment of sustainable groundwater resources management using integrated environmental index: Case studies across Iran. Science of the Total Environment, 676, 792-810.	-	-	Approximated from Fig. 6 by Hosseini et al. (2019)
**Kavir -e Abarkooh Basin**	-	Hosseini, S. M., Parizi, E., Ataie-Ashtiani, B., Simmons, C. T. (2019). Assessment of sustainable groundwater resources management using integrated environmental index: Case studies across Iran. Science of the Total Environment, 676, 792-810.	-	-	Approximated from Fig. 6 by Hosseini et al. (2019)
**Kavir -e Siyahkoh Basin**	-	Hosseini, S. M., Parizi, E., Ataie-Ashtiani, B., Simmons, C. T. (2019). Assessment of sustainable groundwater resources management using integrated environmental index: Case studies across Iran. Science of the Total Environment, 676, 792-810.	-	-	Approximated from Fig. 6 by Hosseini et al. (2019)
**Kavir Haj Aligholi Aquifer**	-	Hosseini, S. M., Parizi, E., Ataie-Ashtiani, B., Simmons, C. T. (2019).	-	-	Approximated from Fig. 6 by Hosseini et al. (2019)

Aquifer system	Broader aquifer system	Reference A	Reference B	Reference C	Steps to delineate
		Assessment of sustainable groundwater resources management using integrated environmental index: Case studies across Iran. Science of the Total Environment, 676, 792-810.			
**Kavir Namak Plain**	-	Hosseini, S. M., Parizi, E., Ataie-Ashtiani, B., Simmons, C. T. (2019). Assessment of sustainable groundwater resources management using integrated environmental index: Case studies across Iran. Science of the Total Environment, 676, 792-810.	-	-	Approximated from Fig. 6 by Hosseini et al. (2019)
**Kavir Qatruiyeh Basin**	-	Hosseini, S. M., Parizi, E., Ataie-Ashtiani, B., Simmons, C. T. (2019). Assessment of sustainable groundwater resources management using integrated environmental index: Case studies across Iran. Science of the Total Environment, 676, 792-810.	-	-	Approximated from Fig. 6 by Hosseini et al. (2019)
**Kaweah Basin**	Tulare Basin of the Central Valley **	California Department of Water Resources (2015c). California's groundwater update 2013 - Chapter 9: Tulare Lake Hydrologic Region. California Department of Water Resources Report, 80 pp. Accessed April 27, 2022 via https://data.cnra.ca.gov/dataset/california-water-plan-	-	-	Approximated from Fig. 9-2 by California Department of Water Resources (2015c)

Aquifer system	Broader aquifer system	Reference A	Reference B	Reference C	Steps to delineate
		groundwater-update-2013/resource/8a4ae915-b786-42e1-9abe-99a8fcc23349			
**Kazanlak Kettle**	-	Orehova, T.V. (2006). Groundwater in the watershed of Tundja River, Bulgaria. In: Proceedings international conference on Water Observation and Information System for Decision Support (BALWOIS 2006), 9pp. Accessed May 22, 2023 via https://d1wqtxts1xzle7.cloudfront.net/68557217/2006_Groundwater_Tundja-libre.pdf?1628007322=&response-content-disposition=inline%3B+filename%3DGroundwater_In_The_Watershed_Of_Tundja_R.pdf&Expires=1684811457&Signature=Git77QKNAp412G6uverKoSAjMhUcaCEnB7IAWAquWpJrca~vfU-RC3wC4UvC3FREwQsbU2FgyaLFguaPDbrIS~a83nUsNL53LxZWHWmY5VZCREQeR5ixKFAD~kYR2GdaFZBtoMsxNnvatZRupMKI4ftjIvpfvaA0bz6ekbE5JqRk81I7yH9N4Q9iRU5oeQqlBMyuv2c5FEUlgIBOt1hpbpZ~~CCaRi20h4YSPQXNYDatrVSjMJP32OsdzeZo1POslCpWjHrLKxLQl-ps0oNt-i08KsevTDURbrscWn2~Mj6zD0d~bw1YLKhwet5UJDgrhFVNyHQCC~QND5o3cCdZRA__&Key-Pair-	-	-	Approximated from Fig. 1 by Orehova (2006)

Aquifer system	Broader aquifer system	Reference A	Reference B	Reference C	Steps to delineate
		Id=APKAJLOHF5GGSLRBV4ZA			
**Kazeroun Plain**	-	Asgharinia, S., Petroselli, A. (2020). A comparison of statistical methods for evaluating missing data of monitoring wells in the Kazeroun Plain, Fars Province, Iran. Groundwater for Sustainable Development, 10, 100294.	-	-	Approximated from Fig. 1 by Asgharinia and Petroselli (2020)
**Keep River Plain**	-	Paul, B., Raper, P., Simons, J., Stainer, G., George, R. (2011). Weaber Plain aquifer test results. Government of Western Australia, Department of Agriculture and Food Resource Management Technical Report 367, 48 pp. Accessed May 8, 2023 via https://library.dpird.wa.gov.au/cgi/viewcontent.cgi?article=1362&context=rmtr	-	-	Approximated from Fig. 1 by Paul et al. (2011)
**Kenwood Valley**	-	California Department of Water Resources (2015g). California's groundwater update 2013 - Chapter 4: San Francisco Bay Hydrologic Region. California Department of Water Resources Report, 78 pp. Accessed April 27, 2022 via https://cawaterlibrary.net/wp-content/uploads/2017/05/GWU2013_Ch4_SanFranciscoBay_Final.pdf	-	-	Approximated from Fig. 4-2 by California Department of Water Resources (2015g)

Aquifer system	Broader aquifer system	Reference A	Reference B	Reference C	Steps to delineate
**Kerala Tertiary Coastal Aquifer**	Malabar Coastal Plain	Manjusree, T. M., Joseph, S., Thomas, J. (2009). Hydrogeochemistry and groundwater quality in the coastal sandy clay aquifers of Alappuzha district, Kerala. Journal of the Geological Society of India, 74, 459-468.	Taylor, G. C., Ghosh, P. K. (1964). Artesian water in the Malabar coastal plain of southern Kerala, India. U.S. Geological Survey Water Supply Paper 1608-D, 19 pp. and 2 plates. Accessed June 23, 2021 from https://pubs.usgs.gov/wsp/1608d/report.pdf	Central Ground Water Board (2012a). Aquifer Systems of Kerala. Central Ground Water Board Report, 72 pp. Accessed June 23, 2021 from http://cgwb.gov.in/AQM/Kerala.pdf	Approximated from Fig. 1 by Manjusree et al. (2009) and Fig. 1 by Taylor and Ghosh (1964), and Plate III by Central Ground Water Board (2012a)
**Kerman-Baghin Aquifer**	-	Malakootian, M., Nozari, M. (2020). GIS-based DRASTIC and composite DRASTIC indices for assessing groundwater vulnerability in the Baghin aquifer, Kerman, Iran. Natural Hazards and Earth System Sciences, 20, 2351-2363.	-	-	Approximated from Fig. 1 by Malakootian and Nozari (2020)
**Kermanshah Plain**	-	Goumehei, E., Geravandi, Y., Wanglin, Y. A. N. (2016). A GIS-Based Study to Investigate Effect of Water Table Changes on DRASTIC Model: A Case Study of Kermanshah, Iran. International Journal of Environment and Geoinformatics, 3, 1-10.	Barati, K., Koopaei, J. A., Azari, A., Darvishi, E., Yousefi, A. (2019). Groundwater Modeling to Determine Hydrodynamic Coefficients in Unconfined Aquifer (Case Study: Kermanshah Plain). Iranian Journal of Soil and Water Research, 50, 687-700.	-	Approximated from Fig. 1 by Goumehei et al. (2010) and Fig. 1 by Barati et al. (2019)
**Kern River Valley**	-	California Department of Water Resources (2015c). California's groundwater update 2013 - Chapter 9: Tulare Lake Hydrologic Region. California Department of Water Resources Report, 80 pp. Accessed April 27, 2022 via https://data.cnra.ca.gov/dataset/california-water-plan-	-	-	Approximated from Fig. 9-2 by California Department of Water Resources (2015c)

Aquifer system	Broader aquifer system	Reference A	Reference B	Reference C	Steps to delineate
		groundwater-update-2013/resource/8a4ae915-b786-42e1-9abe-99a8fcc23349			
**Khafr Basin**	-	Hosseini, S. M., Parizi, E., Ataie-Ashtiani, B., Simmons, C. T. (2019). Assessment of sustainable groundwater resources management using integrated environmental index: Case studies across Iran. Science of the Total Environment, 676, 792-810.	-	-	Approximated from Fig. 6 by Hosseini et al. (2019)
**Khane Ket Basin**	-	Parizi, E., Hosseini, S. M., Ataie-Ashtiani, B., Simmons, C. T. (2020). Normalized difference vegetation index as the dominant predicting factor of groundwater recharge in phreatic aquifers: case studies across Iran. Scientific Reports, 10, 17473.	-	-	Approximated from tabulated coordinates in the Supplementary Information within Parizi et al. (2020) and physiographic and drainage patterns
**Khanmirza Plain**	-	Javi, S. T., Malekmohammadi, B., Mokhtari, H. (2014). Application of geographically weighted regression model to analysis of spatiotemporal varying relationships between groundwater quantity and land use changes (case study: Khanmirza Plain, Iran). Environmental monitoring and assessment, 186, 3123-3138.	-	-	Approximated from Fig. 1 by Javi et al. (2014)

Aquifer system	Broader aquifer system	Reference A	Reference B	Reference C	Steps to delineate
**Khash Aquifer**	-	Ahmadi, A., Aberoumand, M. (2009). Vulnerability of Khash-Plain aquifer, eastern Iran, to pollution using geographic information system (GIS). Geotechnical Geology, 5, 1-11.	Khazai, E., Riggi, M. G. (1999). Impact of urbanization on the Khash aquifer, an arid region of southeast Iran. Proceedings of IUGG 99 Symposium HS5, Birmingham, IAHS Publication number 259, 211-218. Accessed June 5, 2021 from http://hydrologie.org/redbooks/a259/iahs_259_0211.pdf	-	Approximated from Fig. 1 by Ahmadi and Aberoumand (2009) and Fig. 1 by Khazai and Riggi (1999)
**Khesht Plain**	-	Mohammadzadeh-Habili, J., Khalili, D., Zand-Parsa, S., Sabouki, A., Dindarlou, A., & Mozaffarizadeh, J. (2021a). Influences of natural salinity sources and human actions on the Shapour River salinity during the recent streamflow reduction period. Environmental Monitoring and Assessment, 193, 1-18.	-	-	Approximated from Fig. 1 by Mohammadzadeh-Habili et al. (2021a)
**Khezri Plain**	-	Aryafar, A., Khosravi, V., Zarepourfard, H., Rooki, R. (2019a). Evolving genetic programming and other AI-based models for estimating groundwater quality parameters of the Khezri plain, Eastern Iran. Environmental earth sciences, 78, 69.	-	-	Approximated from Fig. 1 by Aryafar et al. (2019a)
**Khir Plain**	-	Hosseini, S. M., Parizi, E., Ataie-Ashtiani, B., Simmons, C. T. (2019). Assessment of sustainable groundwater resources	-	-	Approximated from Fig. 6 by Hosseini et al. (2019)

Aquifer system	Broader aquifer system	Reference A	Reference B	Reference C	Steps to delineate
		management using integrated environmental index: Case studies across Iran. Science of the Total Environment, 676, 792-810.			
**Khomein Basin**	-	Hosseini, S. M., Parizi, E., Ataie-Ashtiani, B., Simmons, C. T. (2019). Assessment of sustainable groundwater resources management using integrated environmental index: Case studies across Iran. Science of the Total Environment, 676, 792-810.	-	-	Approximated from Fig. 6 by Hosseini et al. (2019)
**Khor-Fakhri Basin**	-	Hosseini, S. M., Parizi, E., Ataie-Ashtiani, B., Simmons, C. T. (2019). Assessment of sustainable groundwater resources management using integrated environmental index: Case studies across Iran. Science of the Total Environment, 676, 792-810.	-	-	Approximated from Fig. 6 by Hosseini et al. (2019)
**Khormoj Basin**	-	Hosseini, S. M., Parizi, E., Ataie-Ashtiani, B., Simmons, C. T. (2019). Assessment of sustainable groundwater resources management using integrated environmental index: Case studies across Iran. Science of the Total Environment, 676, 792-810.	-	-	Approximated from Fig. 6 by Hosseini et al. (2019)

Aquifer system	Broader aquifer system	Reference A	Reference B	Reference C	Steps to delineate
**Khosuye-Hajiabad Plain**	-	Hosseini, S. M., Parizi, E., Ataie-Ashtiani, B., Simmons, C. T. (2019). Assessment of sustainable groundwater resources management using integrated environmental index: Case studies across Iran. Science of the Total Environment, 676, 792-810.	-	-	Approximated from Fig. 6 by Hosseini et al. (2019)
**Kings Basin**	Tulare Basin of the Central Valley **	California Department of Water Resources (2015c). California's groundwater update 2013 - Chapter 9: Tulare Lake Hydrologic Region. California Department of Water Resources Report, 80 pp. Accessed April 27, 2022 via https://data.cnra.ca.gov/dataset/california-water-plan-groundwater-update-2013/resource/8a4ae915-b786-42e1-9abe-99a8fcc23349	-	-	Approximated from Fig. 9-2 by California Department of Water Resources (2015c)
**Kinokawa Valley**	-	Yamamoto, S. (1951). The Groundwater Hydrology of River Valley (2) On the Groundwater of Kinokawa Valley. Geographical Review of Japan, 24(1), 8-16.	-	-	Approximated from Fig. 1 by Yamamoto (1951)
**Kladno-Rakovnik Basin**	-	Opluštil, S. (2005). The effect of paleotopography, tectonics and sediment supply on quality of coal seams in continental basins of central and western Bohemia (Westphalian), Czech Republic.	Opluštil, S., Lojka, R., Pšenika, J. (2013). Late Variscan continental basins in western Bohemia: tectono-sedimentary, climate and biotic archives. Schriftreihe Dtsch Ges Geowiss, 82, 179-201.	-	Approximated from Fig. 1c by Opluštil (2005), Fig. 1 by Opluštil et al. (2013)

Aquifer system	Broader aquifer system	Reference A	Reference B	Reference C	Steps to delineate
		International Journal of Coal Geology, 64, 173-203.			
**Klamath River**	Upper Klamath Basin	Gannett, M.W., Breen, K.H. (2015). Groundwater levels, trends, and relations to pumping in the Bureau of Reclamation Klamath Project, Oregon and California: U.S. Geological Survey Open-File Report 2015-1145, 19 pp. Accessed March 20, 2021 from https://pubs.usgs.gov/of/2015/1145/ofr20151145.pdf	-	-	Approximated from Fig. 1 of Gannett and Breen (2015).
**Klamath Valley**	Upper Klamath Basin	Gannett, M.W., Breen, K.H. (2015). Groundwater levels, trends, and relations to pumping in the Bureau of Reclamation Klamath Project, Oregon and California: U.S. Geological Survey Open-File Report 2015-1145, 19 pp. Accessed March 20, 2021 from https://pubs.usgs.gov/of/2015/1145/ofr20151145.pdf	-	-	Approximated from Fig. 1 of Gannett and Breen (2015).
**Knox Creek Plain**	-	Paul, B., Raper, P., Simons, J., Stainer, G., George, R. (2011). Weaber Plain aquifer test results. Government of Western Australia, Department of Agriculture and Food Resource Management Technical Report 367, 48 pp. Accessed May 8, 2023 via https://library.dpird.wa.gov	-	-	Approximated from Fig. 1 by Paul et al. (2011)

Aquifer system	Broader aquifer system	Reference A	Reference B	Reference C	Steps to delineate
		.au/cgi/viewcontent.cgi?article=1362&context=rmtr			
**Kobeh Valley**	-	Lopes, T.J., Evetts. D.M. (2005). Ground-Water pumpage and artificial recharge estimates for calendar year 2000 and average annual natural recharge and interbasin flow by hydrographic area, Nevada. Accessed April 25, 2022 via https://pubs.usgs.gov/sir/2004/5239/sir2004-5239.pdf	Harrill, J.R., Prudic, D.E. (1998). Aquifer systems in the Great Basin region of Nevada, Utah, and adjacent states; summary report. US Geological Survey Professional Paper 1409-A, 75 pp. Accessed April 26, 2022 via https://pubs.usgs.gov/pp/1409a/report.pdf	-	Approximated from Fig. 1 by Lopes and Evetts (2005), and Fig. 2 by Harrill and Prudic (1998). Well completion records, physiography and drainage were used to delinate areas.
**Komijan Aquifer**	Razan Basin	Bayat-Varkeshi, M., Farahani, M., Ghabaei Sough, M. (2018). Effect of Meteorological Drought on Groundwater Resource (Case Study: Komijan Aquifer in Markazi Province). Iran-Water Resources Research, 14, 114-124.	-	-	Approximated from Fig. 1 by Bayat-Varkeshi et al. (2018)
**Kongque Piedmont Plain**	-	Sun, Y., Zhou, J., Zhou, Y., Zeng, Y., Chen, Y. (2019). Influencing Factors of Groundwater Organic Pollution around the Bosten Lake Area of Xinjiang, China. In E3S Web of Conferences (Vol. 98, p. 09029). EDP Sciences.	-	-	Approximated from Fig. 1 by Sun et al. (2019)
**Koohpayeh Basin**	Lower Zayandeh-Rud Basin	Hosseini, S. M., Parizi, E., Ataie-Ashtiani, B., Simmons, C. T. (2019). Assessment of sustainable groundwater resources management using integrated environmental index: Case studies across	Madani, K., Mariño, M. A. (2009). System dynamics analysis for managing Iran's Zayandeh-Rud river basin. Water Resources Management, 23, 2163-2187.	Salemi, H.R., Mamanpoush, A.R., Miranzadeh, M., Akbari, M., Torabi, M., Toomanian, N., Murray-Rust, H., Droogers, P., Sally, H., Gieske, A. (2000). Water management for sustainable irrigated	Approximated from Fig. 6 by Hosseini et al. (2019), Fig. 1 by Madani and Mariño (2009), and Fig. 1 by Salemi et al. (2000)

Aquifer system	Broader aquifer system	Reference A	Reference B	Reference C	Steps to delineate
		Iran. Science of the Total Environment, 676, 792-810.		agriculture in the Zayandeh Rud Basin, Esfahan Province, Iran Report by Iranian Agricultural Engineering Research Institute, Esfahan Agricultural Research Center, and the International Water Management Institute, Research Report Number 1	
**Kootenai Valley**	-	Graham, W.G., Campbell, L.J. (1981). Groundwater resources of Idaho. Idaho Department of Water Resources Report, 61 pp. Accessed March 23, 2021 from https://idwr.idaho.gov/files/publications/198108-MISC-GW-Resources-ID.pdf	-	-	Approximated from Plate 1 of Graham and Campbell (1981). Margins extended into Montana and British Columbia, guided by topography and well completion report data.
**Kordi Shirazi Basin**	-	Hosseini, S. M., Parizi, E., Ataie-Ashtiani, B., Simmons, C. T. (2019). Assessment of sustainable groundwater resources management using integrated environmental index: Case studies across Iran. Science of the Total Environment, 676, 792-810.	-	-	Approximated from Fig. 6 by Hosseini et al. (2019)
**Kordkandi-Duzduzan Plain**	-	Soltani, S., Moghaddam, A. A., Barzegar, R., Kazemian, N., Tziritis, E. (2017). Hydrogeochemistry and water quality of the Kordkandi-Duzduzan plain, NW Iran: application of multivariate statistical analysis and PoS index.	Soltani, S., Asghari Moghaddam, A., Barzegar, R., Kazemian, N. (2016). Evaluation of nitrate concentration and vulnerability of the groundwater by GODS and AVI methods (case study: Kordkandi-Duzduzan Plain,	-	Approximated from Fig. 1 by Soltani et al. (2017) and Fig. 1 by Soltani et al. (2016)

Aquifer system	Broader aquifer system	Reference A	Reference B	Reference C	Steps to delineate
		Environmental monitoring and assessment, 189, 1-20.	East Azarbaijan province). Iranian journal of Ecohydrology, 3, 517-531.		
**Korin Basin**	-	Hosseini, S. M., Parizi, E., Ataie-Ashtiani, B., Simmons, C. T. (2019). Assessment of sustainable groundwater resources management using integrated environmental index: Case studies across Iran. Science of the Total Environment, 676, 792-810.	-	-	Approximated from Fig. 6 by Hosseini et al. (2019)
**Koriyan Basin**	-	Hosseini, S. M., Parizi, E., Ataie-Ashtiani, B., Simmons, C. T. (2019). Assessment of sustainable groundwater resources management using integrated environmental index: Case studies across Iran. Science of the Total Environment, 676, 792-810.	-	-	Approximated from Fig. 6 by Hosseini et al. (2019)
**Korron Basin**	-	Hosseini, S. M., Parizi, E., Ataie-Ashtiani, B., Simmons, C. T. (2019). Assessment of sustainable groundwater resources management using integrated environmental index: Case studies across Iran. Science of the Total Environment, 676, 792-810.	Madani, K., Mariño, M. A. (2009). System dynamics analysis for managing Iran's Zayandeh-Rud river basin. Water Resources Management, 23, 2163-2187.	Salemi, H.R., Mamanpoush, A.R., Miranzadeh, M., Akbari, M., Torabi, M., Toomanian, N., Murray-Rust, H., Droogers, P., Sally, H., Gieske, A. (2000). Water management for sustainable irrigated agriculture in the Zayandeh Rud Basin, Esfahan Province, Iran Report by Iranian Agricultural Engineering Research Institute, Esfahan Agricultural Research Center, and the	Approximated from Fig. 6 by Hosseini et al. (2019), Fig. 1 by Madani and Mariño (2009), and Fig. 1 by Salemi et al. (2000)

Aquifer system	Broader aquifer system	Reference A	Reference B	Reference C	Steps to delineate
				International Water Management Institute, Research Report Number 1	
**Kouhbanan Basin**	-	Parizi, E., Hosseini, S. M., Ataie-Ashtiani, B., Simmons, C. T. (2020). Normalized difference vegetation index as the dominant predicting factor of groundwater recharge in phreatic aquifers: case studies across Iran. Scientific Reports, 10, 17473.	-	-	Approximated from tabulated coordinates in the Supplementary Information within Parizi et al. (2020) and physiographic and drainage patterns
**Kozhikkode Coastal Aquifer**	Malabar Coastal Plain	Central Ground Water Board (2012a). Aquifer Systems of Kerala. Central Ground Water Board Report, 72 pp. Accessed June 23, 2021 from http://cgwb.gov.in/AQM/Kerala.pdf	George, M. E., Babu, D. S., Akhil, T., Rafeeque, M. K. (2018). Investigation on submarine groundwater discharge at Kozhikkode Coastal Aquifer, SW Western Ghats. Journal of the Geological Society of India, 92, 626-633.	-	Approximated from Plate III by Central Ground Water Board (2012a) and Fig. 1 by George et al. (2018)
**Kristianstad Plain**	-	Lund, J. R. (1988). Regional water supply development in south Sweden. Journal of Urban Planning and Development, 114, 14-33.	-	-	Approximated from Fig. 2 by Lund (1988)
**Krsko-Brezice Polje Aquifer**	-	Mali, N., Koroša, A., Urbanc, J. (2021). Prevalence of pesticides in Krško-Brežice polje aquifer. Geologija, 64, 267-288. Accessed June 4, 2023 via https://www.geologija-revija.si/index.php/geologija/article/view/1820/1885	-	-	Approximated from Fig. 1 by Mali et al. (2021)
**Ktaoua Oasis**	Middle Draa Basin	Szczucińska, A., Dłużewski, M., Kozłowski, R., Niedzielski, P. (2019). Hydrochemical diversity of	-	-	Approximated from Fig. 1 by Szczucińska et al. (2019)

Aquifer system	Broader aquifer system	Reference A	Reference B	Reference C	Steps to delineate
		a large alluvial aquifer in an arid zone (Draa river, S Morocco). Ecological Chemistry and Engineering S, 26, 81-100.			
**Kuhdasht Plain**	-	Salehi, H., Zeinivand, H. (2016). Evaluation and mapping of groundwater quality for rigation and drinking purposes in Kuhdasht region, Iran. Environmental Resources Research, 4(1), 75-89.	-	-	Approximated from Fig. 1 by Salehi and Zeinivand (2016)
**Kunming Urban Area**	-	Yang, W. Q., Shen, L., Xiao, H., Wang, Y. Z. (2013). Impact of shallow groundwater quality evolution in Kunming Urban by human activities. Advanced Materials Research, 788, 302-306.	-	-	Approximated from Fig. 1 by Yang et al. (2013)
**Kurnool Basin**	Cuddapah Sedimentary Basin	Dar, F. A., Perrin, J., Riotte, J., Gebauer, H. D., Narayana, A. C., Shakeel, A. (2011). Karstification in the Cuddapah Sedimentary Basin, southern India: implications for groundwater resources. Acta Carsologica, 40, 457-472.	Saha, D., Ray, R. K. (2019). Groundwater resources of India: potential, challenges and management. In Groundwater Development and Management. Springer, Cham (ed. Sikdar, P.K.), pp. 19-42.	Chalapathi Rao, N. V., Gibson, S. A., Pyle, D. M., Dickin, A. P. (2004). Petrogenesis of Proterozoic lamproites and kimberlites from the Cuddapah Basin and Dharwar craton, southern India. Journal of Petrology, 45, 907-948.	Approximated from Fig. 1 by Dar et al. (2011), Fig. 2.2 by Saha and Ray (2019), and Fig. 1 by Chalapathi Rao et al. (2004)
**Kuzuryu Basin**	-	Noma, Y., Kino, Y., Goto, H. (1969). Ground water in the Kuzuryu River Basin, Fukui Prefecture (in Japanese), Monthly report of the Geological Survey, 20, 767-782. Accessed April 14, 2023 via https://www.gsj.jp/data/bull-gsj/20-12_01.pdf	-	-	Approximated from Fig. 1 by Noma et al. (1969)

Aquifer system	Broader aquifer system	Reference A	Reference B	Reference C	Steps to delineate
**Kyoto Basin**	-	Yoneda, M., Morisawa, S., Takine, N., Fukuhara, S., Takeuchi, H., Hirano, T., Takahashi, H., Inoue, Y. (2001). Groundwater deterioration caused by induced recharge: Field survey and verification of the deterioration mechanism by stochastic numerical simulation. Water, Air, and Soil Pollution, 127, 125-156.	-	-	Approximated from Fig. 1 by Yoneda et al. (2001)
**La Blanca Aquifer**	-	CONAGUA (2015n). Actualización de la disponibilidad media anual de agua en el acuífero La Blanca (3228), Estado de Zacatecas. 21 pp. Accessed January 24, 2022 via https://www.gob.mx/cms/uploads/attachment/file/104536/DR_3228.pdf	-	-	Approximated from Fig. 1 by CONAGUA (2015n)
**La Paz Coastal Aquifer**	La Paz Basin	Torres-Martinez, J. A., Mora, A., Ramos-Leal, J. A., Morán-Ramírez, J., Arango-Galván, C., Mahlknecht, J. (2019). Constraining a density-dependent flow model with the transient electromagnetic method in a coastal aquifer in Mexico to assess seawater intrusion. Hydrogeology Journal, 27, 2955-2972.	Muir, M.A.K., Martinez, A.A. (2018). Preliminary Assessment of Water Resources including Climate Considerations for the Los Cabos and La Paz Municipalities in the State of Baja California Sur, Mexico. Conference: International Water Association Water, Energy and Climate Conference (May 21 to 23, 2024), Mexico City, 13 pp. Accessed May 6, 2021 from https://arctic.ucalgary.ca/sites/default/files/April18-IWA-	Mahlknecht, J., Merchán, D., Rosner, M., Meixner, A., Ledesma-Ruiz, R. (2017). Assessing seawater intrusion in an arid coastal aquifer under high anthropogenic influence using major constituents, Sr and B isotopes in groundwater. Science of the Total Environment, 587, 282-295.	Approximated from Fig. 1 by Torres-Martinez et al. (2019), Fig. 1 by Mahlknecht et al. (2017), and from map on page 2 of Muir and Martinez (2018).

Aquifer system	Broader aquifer system	Reference A	Reference B	Reference C	Steps to delineate
			FinalConferenceVersion-MAKMuir.pdf		
**La Plana de la Galera and Tortosa Alluvial Aquifers**	-	Samper, J., Li, Y., Pisani, B., Ribeiro, L., Fakir, Y., Stigter, T. (2011). Evaluació de los impactos del cambio climático e los acuíferos de la pla a de la galera y del aluvial de Tortosa. Estudios en la Zona no Saturada del Suelo. Vol X, 359-364. Accessed May 23, 2021 from http://zonanosaturada.com/zns11/publications/p359.pdf	-	-	Approxiamted from Fig. 1 by Samper et al. (2011)
**La Posa Plain**	-	Tucci, P. (1982). Use of a three-dimensional model for the analysis of the ground-water flow system in Parker Valley, Arizona and California. US Geological Survey Open-File Report 82-1006, 60 pp. Accessed April 27, 2022 via https://pubs.usgs.gov/of/1982/1006/report.pdf	-	-	Approximated from Fig. 2 by Tucci (1982)
**La Vinilla Casablanca Aquifer**	-	Direccion General de Aguas (2017). Inventario Nacional de acuiferos. Ministerio De Obras Públicas (Gobierno De Chile) report number 403, 60 pp. Accessed May 17, 2021 from https://snia.mop.gob.cl/sad/SUB5748.pdf	-	-	Approximated from map on page 40 by Direccion General de Aguas (2017).
**La Yarada Aquifer**	-	Pino, E., Ramos, L., Avalos, O., Tacora, P., Chávarri, E., Angulo, O., Ascensios, D. and Mejía, J. (2018).	Pino-Vargas, E., Guevara-Pérez, E., Avendaño-Jihuallanga, C. (2021). Historical evolution of the	Chucuya, S., Vera, A., Pino-Vargas, E., Steenken, A., Mahlknecht, J., Montalván, I. (2022).	Approximated from Fig. 1 by Pino et al. (2018) and Fig. 2 by Pino-Vargas et al. (2021) and Fig. 1 by Chucuya et al. (2021)

Aquifer system	Broader aquifer system	Reference A	Reference B	Reference C	Steps to delineate
		Factors affecting depletion and pollution by marine intrusion in the La Yarada's coastal aquifer, Tacna, Peru. Tecnología y Ciencias del Agua, 177-214 doi:10.24850/j-tyca-2019-05-07.	hydrogeological conceptualization and the use of Caplina aquifer on the northern edge of the Atacama Desert. Revista Ingeniería UC, 28, 378-391. Accessed December 21, 2022 via http://servicio.bc.uc.edu.ve/ingenieria/revista/v28n3/art04.pdf	Hydrogeochemical characterization and identification of factors influencing groundwater quality in coastal aquifers, case: La Yarada, Tacna, Peru. International Journal of Environmental Research and Public Health, 19, 2815.	
**Lachlan Alluvium**	-	NSW Department of Planning and Environment (2019). Lachlan Alluvium Groundwater Resource Description. NSW Department of Planning and Environment Report, 97 pp. Accessed May 1, 2023 via https://www.mdba.gov.au/sites/default/files/pubs/appendix-a-lachlan-alluvium-groundwater-resource-description.pdf	-	-	Approximated from Fig. 1 by NSW Department of Planning and Environment (2019)
**Ladiz Basin**	-	Hosseini, S. M., Parizi, E., Ataie-Ashtiani, B., Simmons, C. T. (2019). Assessment of sustainable groundwater resources management using integrated environmental index: Case studies across Iran. Science of the Total Environment, 676, 792-810.	-	-	Approximated from Fig. 6 by Hosseini et al. (2019)
**Lafayette Area**	Gulf Coast Regional Aquifer System	Weiss, J. S. (1990). Geohydrologic units of the coastal lowlands aquifer system, south-central United States. U.S. Geological Survey Regional	Louisiana Department of Transportation and Development (2011). Water Resources of Lafayette Parish. U.S. Geological Survey Fact Sheet 2010–	-	Outer margin of Gulf Coast Regional Aquifer System approximated from Fig. 2 of Weiss (1990). Detailed study site information for a portion of the subarea available from Louisiana

Aquifer system	Broader aquifer system	Reference A	Reference B	Reference C	Steps to delineate
		aquifer-systems analysis. 42 pp. Accessed March 30, 2021 from https://pubs.usgs.gov/pp/1416c/report.pdf	3048, 6 pp. Accessed March 30, 2021 from https://pubs.usgs.gov/fs/2010/3048/pdf/FS2010-3048.pdf		Department of Transportation and Development (2011)
**Laguna de Santiaguillo Basin**	-	de Lourdes Corral-Bermudez, M., Sánchez-Ortiz, E., Álvarez-Bernal, D., Gutiérrez-Montenegro, M. O., Cassio-Madrazo, E. (2019). Scenarios of availability of water due to overexploitation of the aquifer in the basin of Laguna de Santiaguillo, Durango, Mexico. PeerJ, 7, e6814.	-	-	Approximated from Fig. 1 by de Lourdes Corral-Bermudez et al. (2019)
**Lahontan Valley**	-	Smith, D.W., Buto, S.G., Welborn, T.L. (2016). Groundwater-level change and evaluation of simulated water levels for irrigated areas in Lahontan Valley, Churchill County, west-central Nevada, 1992–2012: U.S. Geological Survey Scientific Investigations Report 2016-5045, 23 pp. Accessed March 10, 2021 from https://pubs.usgs.gov/sir/2016/5045/sir20165045.pdf	-	-	Approximated from Fig. 4 of Smith et al. (2016)
**Laidley Sub-basin**	Great Artesian Basin	Ransley, T.R., Smerdon, B.D. (2012). Hydrostratigraphy, hydrogeology and system conceptualisation of the Great Artesian Basin. A technical report to the Australian Government from the CSIRO Great	Smerdon, B.D., Ramsley, T.R. (2012). Water Resource Assessment for the Surat Region. A technical report to the Australian Government from the CSIRO Great Artesian Basin Water Resource Assessment, 142	-	Approximated from Fig. 2.3 by Ransley and Smerdon (2012), and Fig. 5.2 by Smerdon and Ransley (2012)

Aquifer system	Broader aquifer system	Reference A	Reference B	Reference C	Steps to delineate
		Artesian Basin Water Resource Assessment, 324 pp. Accessed July 20, 2021 from https://publications.csiro.au/rpr/download?pid=csiro:EP132693&dsid=DS5	pp. Accessed July 20, 2021 from https://publications.csiro.au/rpr/download?pid=csiro:EP132644&dsid=DS4		
**Laizhou Coastal Plain**	-	Liu, S., Tang, Z., Gao, M., Hou, G. (2017). Evolutionary process of saline-water intrusion in Holocene and Late Pleistocene groundwater in southern Laizhou Bay. Science of the Total Environment, 607, 586-599.	Han, D. M., Song, X. F., Currell, M. J., Yang, J. L., Xiao, G. Q. (2014). Chemical and isotopic constraints on evolution of groundwater salinization in the coastal plain aquifer of Laizhou Bay, China. Journal of Hydrology, 508, 12-27.	Han-xue, Q., Dong-yan, L., Guan-qun, L., Pi-hai, N. (1997). Saline water intrusion and its influence in the Laizhou area. Chinese Journal of Oceanology and Limnology, 15, 342-349.	Approximated from Fig. 1 by Liu et al. (2017), Fig. 1 by Han et al. (2014) and Fig. 1 by Han-xue et al. (1997)
**Lajas Valley**	-	Graves, R. P. (1991). Ground-water resources in Lajas Valley, Puerto Rico. US Geological Survey Water-Resources Investigations Report 89-4182, 61 pp. Accessed May 22, 2021 from https://pubs.usgs.gov/wri/1989/4182/report.pdf	Renken, R. A., Black, B. A., Ward, W. C., Gill, I. P., Gómez-Gómez, F., Rodríguez-Martínez, J. (2002). Geology and hydrogeology of the Caribbean islands aquifer system of the commonwealth of Puerto Rico and the US Virgin Islands. U.S. Geological Survey Professional Paper 1419, 148 pp. Accessed May 22, 2021 from https://pubs.usgs.gov/pp/p1419/pdf/BOOK.PDF	-	Approximated from Fig. 1 by Graves (1991); see also Fig. 2 by Renken et al. (2002)
**Lake Frome Embayment**	Great Artesian Basin	Ransley, T.R., Smerdon, B.D. (2012). Hydrostratigraphy, hydrogeology and system conceptualisation of the Great Artesian Basin. A technical report to the Australian Government	Ransley, T.R., Radke, B.M., Feitz, A.J., Kellett, J.R., Owens, R., Bell, J. and Stewart, G. (2015). Hydrogeological Atlas of the Great Artesian Basin, Geoscience Australia. Canberra, 141 pp. Accessed	-	Approximated from Fig. 2.3 by Ransley and Smerdon (2012) and Map 4 by Ransley et al. (2015)

Aquifer system	Broader aquifer system	Reference A	Reference B	Reference C	Steps to delineate
		from the CSIRO Great Artesian Basin Water Resource Assessment, 324 pp. Accessed July 20, 2021 from https://publications.csiro.au/rpr/download?pid=csiro:EP132693&dsid=DS5	July 19, 2021 from http://www.ga.gov.au/scientific-topics/water/groundwater/gab		
**Lake Mohave Basin**	-	Tillman, F.D, Garner, B.D., Truini, M. (2013). Preliminary groundwater flow model of the basin-fill aquifers in Detrital, Hualapai, and Sacramento Valleys, Mohave County, northwestern Arizona. U.S. Geological Survey Scientific Investigations Report 2013–5122, 52 pp. http://pubs.usgs.gov/sir/2013/5122/	-	-	Approximated from Fig. 2 by Tillman et al. (2013).
**Lake Valley**	-	Hurlow, H. A. (2014). Hydrogeologic studies and groundwater monitoring in Snake Valley and adjacent hydrographic areas, west-central Utah and east-central Nevada (Vol. 135). Utah Geological Survey Bulletin 135, 272 pp.	-	-	Approximated from map on page 6 of Hurlow (2014)
**Lamerd Plain**	-	Hosseini, S. M., Parizi, E., Ataie-Ashtiani, B., Simmons, C. T. (2019). Assessment of sustainable groundwater resources management using integrated environmental index: Case studies across Iran. Science of the Total Environment, 676, 792-810.	-	-	Approximated from Fig. 6 by Hosseini et al. (2019)

Aquifer system	Broader aquifer system	Reference A	Reference B	Reference C	Steps to delineate
**Lampang Basin**	-	Santha, N., Sangkajan, S., Saenton, S. (2022). Arsenic contamination in groundwater and potential health risk in Western Lampang Basin, Northern Thailand. Water, 14, 465. Accessed May 1, 2023 via https://www.mdpi.com/2073-4441/14/3/465	-	-	Approximated from Fig. 1 by Santha et al. (2022)
**Lampazos Villaldama Aquifer**	-	CONAGUA (2020f). Actualización de la disponibilidad media anual de agua en el acuífero Lampazos Villaldama (1901), Estado de Nuevo Leon. 11 pp. Accessed January 24, 2022 via https://sigagis.conagua.gob.mx/gas1/Edos_Acuiferos_18/nleon/DR_1901.pdf	-	-	Approximated from Fig. 1 and Fig. 2 by CONAGUA (2020f) and well completion data
**Landes Plain**	Aquitaine Basin	Rouillard, J., Maréchal, J.-C. (2020). Groundwater in France: Resources, Use and Management Issues. In: Sustainable Groundwater Management A Comparative Analysis of French and Australian Policies and Implications to Other Countries, pp. 17-45. Accessed May 22, 2021 from https://hal.archives-ouvertes.fr/hal-02512154/document	Willmes, M., Bataille, C.P., James, H.F., Moffat, I., McMorrow, L., Kinsley, L., Armstrong, R.A., Eggins, S., Grün, R. (2018). Mapping of bioavailable strontium isotope ratios in France for archaeological provenance studies. Applied Geochemistry 90, 75-86.	-	Approximated from Fig. 1 by Rouillard and Maréchal (2020), with broader outline of Aquitaine Basin supported by delineation in Fig. 1 by Willmes et al. (2018).
**Lannemezan Plateau**	Aquitaine Basin	Rouillard, J., Maréchal, J.-C. (2020). Groundwater in France: Resources, Use and Management Issues. In: Sustainable Groundwater Management A	Willmes, M., Bataille, C.P., James, H.F., Moffat, I., McMorrow, L., Kinsley, L., Armstrong, R.A., Eggins, S., Grün, R. (2018). Mapping of bioavailable	-	Approximated from Fig. 1 by Rouillard and Maréchal (2020), with broader outline of Aquitaine Basin supported by delineation in Fig. 1 by Willmes et al. (2018).

Aquifer system	Broader aquifer system	Reference A	Reference B	Reference C	Steps to delineate
		Comparative Analysis of French and Australian Policies and Implications to Other Countries, pp. 17-45. Accessed May 22, 2021 from https://hal.archives-ouvertes.fr/hal-02512154/document	strontium isotope ratios in France for archaeological provenance studies. Applied Geochemistry 90, 75-86.		
**Lanyang Plain**	-	Liang, C. P., Sun, C. C., Suk, H., Wang, S. W., Chen, J. S. (2021). A Machine Learning Approach for Spatial Mapping of the Health Risk Associated with Arsenic-Contaminated Groundwater in Taiwan's Lanyang Plain. International Journal of Environmental Research and Public Health, 18, 11385.	Kao, Y. H., Liu, C. W., Wang, P. L., Liao, C. M. (2015). Effect of sulfidogenesis cycling on the biogeochemical process in arsenic-enriched aquifers in the Lanyang Plain of Taiwan: Evidence from a sulfur isotope study. Journal of Hydrology, 528, 523-536.	Lewis, C., Ray, D., Chiu, K. K. (2007). Primary geologic sources of arsenic in the Chianan Plain (Blackfoot disease area) and the Lanyang Plain of Taiwan. International Geology Review, 49, 947-961.	Approximated from Fig. 1 by Liang et al. (2021), Fig. 1a by Kao et al. (2015), and Fig. 1 by Lewis et al. (2007)
**Lanzhou Valley Basin**	-	Lü, X., Han, Z., Li, H., Zheng, Y., Liu, J. (2022). Influence of urbanization on groundwater chemistry at Lanzhou Valley basin in China. Minerals, 12, 385.	-	-	Approximated from Fig. 1 by Lü et al. (2022)
**Laramie Basin**	-	Bradley, E. (1955). Summary of the ground-water resources of the Laramie River drainage basin, Wyoming, and the North Platte River drainage basin from Douglas, Wyoming, to the Wyoming-Nebraska state line. U.S. Geological Survey Open-File Report 55-17, 24 pp. Accessed March 29, 2021 from https://pubs.usgs.gov/of/1955/0017/report.pdf	-	-	Approximated from Fig. 1 of Bradley (1955)

Aquifer system	Broader aquifer system	Reference A	Reference B	Reference C	Steps to delineate
**Las Vegas Basin**	-	Burbey, T. J. (1997). Hydrogeology and potential for ground-water development, carbonate-rock aquifers in southern Nevada and southeastern California. US Geological Survey Water-Resources Investigations Report 95-4168, 70 pp. Accessed March 10, 2021 from https://pubs.usgs.gov/wri/1995/4168/report.pdf	-	-	Approximated from Fig. 10 of Burbey (1997)
**Las Vegas Basin of northeast New Mexico**	-	Land, L. (2016b). Overview of fresh and brackish water quality in New Mexico. Project Summary Report, New Mexico Bureau of Geology and Mineral Resources, Open-file Report 583, 4 pp. Accessed April 27, 2022 via https://geoinfo.nmt.edu/resources/water/amp/brochures/BWA/Raton_Las_Vegas_Basin_FBWQNM.pdf	-	-	Approximated via map on page 2 of Land (2016b)
**Lash Basin**	-	Hosseini, S. M., Parizi, E., Ataie-Ashtiani, B., Simmons, C. T. (2019). Assessment of sustainable groundwater resources management using integrated environmental index: Case studies across Iran. Science of the Total Environment, 676, 792-810.	-	-	Approximated from Fig. 6 by Hosseini et al. (2019)
**Latrobe Valley**	Gippsland Basin	Currell, M., Banfield, D., Cartwright, I., Cendón, D. I. (2017). Geochemical indicators of the origins and	Varma, S., Michael, K. (2012). Impact of multi-purpose aquifer utilisation on a variable-density	Beverly, C., Hocking, M., Cheng, X., O'Neil, C., Schroers, R., Baker, S. (2015).The Gippsland	Approximated from Fig. 4 by Beverly et al. (2015), Fig. 1 by Varma and Michael (2012), and Fig. 1 by Currell et al. (2017)

Aquifer system	Broader aquifer system	Reference A	Reference B	Reference C	Steps to delineate
		evolution of methane in groundwater: Gippsland Basin, Australia. Environmental Science and Pollution Research, 24, 13168-13183.	groundwater flow system in the Gippsland Basin, Australia. Hydrogeology Journal, 20, 119-134.	groundwater model. Technical Report, 409 pp. Accessed October 8, 2021 from https://www.parliament.vic.gov.au/images/stories/committees/EPC/Other_documents/G3_-_Gippsland_groundwater_model_report_June_2015_2.pdf	
**Leadville Limestone Aquifer**	-	Hearne, G.A., Lindner-Lunsford, J., Cain, D., Watts, K.R., Robson, S.G., Tobin, R.L., Teller, R.W., Schneider Jr, P.A., Gearhart, M.J. (1987). Colorado ground water quality. United States Geological Survey Open File Report 87-716, 14 pp. Accessed July 21, 2021 from https://pubs.usgs.gov/of/1987/0716/report.pdf	-	-	Approximated from Fig. 2 by Hearne et al. (1987)
**Leibnitzer Feld**	-	Kralik, M., Humer, F., Fank, J., Harum, T., Klammler, G., Gooddy, D., Sültenfuß, J., Gerber, C., Purtschert, R. (2014). Using 18O/2H, 3H/3He, 85Kr and CFCs to determine mean residence times and water origin in the Grazer and Leibnitzer Feld groundwater bodies (Austria). Applied Geochemistry, 50, 150-163.	Retter, A., Griebler, C., Haas, J., Birk, S., Stumpp, C., Brielmann, H., Fillinger, L. (2021). Application of the DA-(C) index as a simple tool for microbial-ecological characterization and assessment of groundwater ecosystems—a case study of the Mur River Valley, Austria. Österr Wasser- und Abfallw, https://doi.org/10.1007/s00506-021-00799-5	-	Approximated from Fig. 2 by Kralik et al. (2014) and Fig. 1 by Retter et al. (2021)
**Lemhi Valley**	-	Graham, W.G., Campbell, L.J. (1981). Groundwater	-	-	Approximated from Plate 1 of Graham and Campbell (1981).

Aquifer system	Broader aquifer system	Reference A	Reference B	Reference C	Steps to delineate
		resources of Idaho. Idaho Department of Water Resources Report, 61 pp. Accessed March 23, 2021 from https://idwr.idaho.gov/files/publications/198108-MISC-GW-Resources-ID.pdf			
**Lenjanat Basin**	-	Hosseini, S. M., Parizi, E., Ataie-Ashtiani, B., Simmons, C. T. (2019). Assessment of sustainable groundwater resources management using integrated environmental index: Case studies across Iran. Science of the Total Environment, 676, 792-810.	Amiri, V., Rezaei, M., Sohrabi, N. (2014). Groundwater quality assessment using entropy weighted water quality index (EWQI) in Lenjanat, Iran. Environmental Earth Sciences, 72, 3479-3490.	Salemi, H.R., Mamanpoush, A.R., Miranzadeh, M., Akbari, M., Torabi, M., Toomanian, N., Murray-Rust, H., Droogers, P., Sally, H., Gieske, A. (2000). Water management for sustainable irrigated agriculture in the Zayandeh Rud Basin, Esfahan Province, Iran Report by Iranian Agricultural Engineering Research Institute, Esfahan Agricultural Research Center, and the International Water Management Institute, Research Report Number 1	Approximated from Fig. 6 by Hosseini et al. (2019), Fig. 1 by Amiri et al. (2014) and Fig. 1 by Salemi et al. (2000)
**Letaba Lowveld**	-	Holland, M. (2011). Hydrogeological characterisation of crystalline basement aquifers within the Limpopo Province, South Africa. PhD Thesis, University of Pretoria, 162 pp. Accessed October 28, 2021 from https://repository.up.ac.za/bitstream/handle/2263/25713/Complete.pdf?sequence=9	-	-	Approximated from Fig. 4.11 by Holland (2011) and local physiography, drainage and well construction data

Aquifer system	Broader aquifer system	Reference A	Reference B	Reference C	Steps to delineate
**Lhasa Valley**	-	Liu, J., Gao, Z., Wang, M., Li, Y., Ma, Y., Shi, M., Zhang, H. (2018). Study on the dynamic characteristics of groundwater in the valley plain of Lhasa City. Environmental Earth Sciences, 77, 1-15.	-	-	Approximated from Fig. 1 by Liu et al. (2018)
**Lida Valley**	-	Lopes, T.J., Evetts. D.M. (2005). Ground-Water pumpage and artificial recharge estimates for calendar year 2000 and average annual natural recharge and interbasin flow by hydrographic area, Nevada. Accessed April 25, 2022 via https://pubs.usgs.gov/sir/2004/5239/sir2004-5239.pdf	Harrill, J.R., Prudic, D.E. (1998). Aquifer systems in the Great Basin region of Nevada, Utah, and adjacent states; summary report. US Geological Survey Professional Paper 1409-A, 75 pp. Accessed April 26, 2022 via https://pubs.usgs.gov/pp/1409a/report.pdf	-	Approximated from Fig. 1 by Lopes and Evetts (2005), and Fig. 2 by Harrill and Prudic (1998). Well completion records, physiography and drainage were used to delinate areas.
**Lillo-Quintanar Aquifer**	-	Yustres, Á., Navarro, V., Asensio, L., Candel, M., García, B. (2013). Groundwater resources in the Upper Guadiana Basin (Spain): a regional modelling analysis. Hydrogeology Journal, 21, 1129.	-	-	Approximated from Fig. 1 by Yustres et al. (2013)
**Limache Aquifer**	-	Direccion General de Aguas (2017). Inventario Nacional de acuiferos. Ministerio De Obras Públicas (Gobierno De Chile) report number 403, 60 pp. Accessed May 17, 2021 from https://snia.mop.gob.cl/sad/SUB5748.pdf	-	-	Approximated from map on page 40 by Direccion General de Aguas (2017).
**Limari Basin**	-	Oyarzún, R., Jofré, E., Morales, P., Maturana, H., Oyarzún, J., Kretschmer,	Oyarzún, R., Barrera, F., Salazar, P., Maturana, H., Oyarzún, J., Aguirre, E.,	-	Approximated from Fig. 1 by Oyarzún et al. (2015) and Fig. 1 by Oyarzún et al. (2014).

Aquifer system	Broader aquifer system	Reference A	Reference B	Reference C	Steps to delineate
		N., Aguirre, E., Gallardo, P., Toro, L.E., Muñoz, J.F., Aravena, R (2015). A hydrogeochemistry and isotopic approach for the assessment of surface water–groundwater dynamics in an arid basin: the Limarí watershed, North-Central Chile. Environmental Earth Sciences, 73, 39-55.	Alvarez, P., Jourde, H., Kretschmer, N. (2014). Multi-method assessment of connectivity between surface water and shallow groundwater: the case of Limarí River basin, north-central Chile. Hydrogeology Journal, 22, 1857-1873.		
**Limmat Valley**	-	Huber, E., Hendricks-Franssen, H.J., Kaiser, H.P., Stauffer, F. (2011). The role of prior model calibration on predictions with ensemble Kalman filter. Groundwater, 49(6), 845-858.	-	-	Approximated from Fig. 1 by Huber et al. (2011)
**Limpopo Plateau**	-	Holland, M. (2011). Hydrogeological characterisation of crystalline basement aquifers within the Limpopo Province, South Africa. PhD Thesis, University of Pretoria, 162 pp. Accessed October 28, 2021 from https://repository.up.ac.za/bitstream/handle/2263/25713/Complete.pdf?sequence=9	Ebrahim, G. Y., Villholth, K. G., Boulos, M. (2019). Integrated hydrogeological modelling of hard-rock semi-arid terrain: supporting sustainable agricultural groundwater use in Hout catchment, Limpopo Province, South Africa. Hydrogeology Journal, 27, 965-981.	Sorensen, J.P., Davies, J., Ebrahim, G.Y., Lindle, J., Marchant, B.P., Ascott, M.J., Bloomfield, J.P., Cuthbert, M.O., Holland, M., Jensen, K.H., Shamsudduha, M. (2021). The influence of groundwater abstraction on interpreting climate controls and extreme recharge events from well hydrographs in semi-arid South Africa. Hydrogeology Journal, 1-15.	Approximated from Fig. 4.11 by Holland (2011), Fig. 1 by Ebrahim et al. (2019), Fig. 1 by Sorenson et al. (2021) and local physiography, drainage and well construction data
**Lincolnshire Chalk Aquifer**	-	MacDonald, A.M., Allen, D.J. (2001). Aquifer properties of the Chalk of England. Quarterly Journal of Engineering Geology and	Whitehead, E.J., Lawrence, A.R. (2006). The Chalk aquifer of Lincolnshire. British Geological Survey Research Report RR/06/03, 74 pp. Accessed October	Robins, N. S., Ball, D. F. (2006). The Dumfries Basin aquifer. British Geological Survey Research Report RR/06/02, 68 pp. Accessed October 27, 2021 from	Approximated from Fig. 8 by MacDonald and Allen (2001), Fig. 1 by Gale and Rutter (2006), and map on the final page of Robins and Ball (2006)

Aquifer system	Broader aquifer system	Reference A	Reference B	Reference C	Steps to delineate
		Hydrogeology, 34, 371-384.	27, 2021 from http://nora.nerc.ac.uk/id/eprint/3699/1/RR06003.pdf	http://nora.nerc.ac.uk/id/eprint/3685/1/RR06002.pdf	
**Linfen Basin**	-	Zhang, Q., Rodríguez-Lado, L., Johnson, C. A., Xue, H., Shi, J., Zheng, Q., Sun, G. (2012). Predicting the risk of arsenic contaminated groundwater in Shanxi Province, Northern China. Environmental Pollution, 165, 118-123.	-	-	Approximated from Fig. 1 by Zhang et al. (2012)
**Lipan Flats**	-	Beach, J.A., Burton, S., Kolarik, B. (2004b). Groundwater availability model for the Lipan Aquifer in Texas. Texas Water Development Board Report, 246 pp. Accessed May 1, 2023 via https://www.twdb.texas.gov/groundwater/models/gam/lipn/LIPN_Model_Report.pdf	-	-	Approximated from Fig. 2.1.3 by Beach et al. (2004b)
**Lishtar Basin**	-	Parizi, E., Hosseini, S. M., Ataie-Ashtiani, B., Simmons, C. T. (2020). Normalized difference vegetation index as the dominant predicting factor of groundwater recharge in phreatic aquifers: case studies across Iran. Scientific Reports, 10, 17473.	-	-	Approximated from tabulated coordinates in the Supplementary Information within Parizi et al. (2020) and physiographic and drainage patterns
**Little Bitterroot Valley**	Flathead Valley	LaFave, J. I., Smith, L. N., Patton, T. W. (2004). Ground-Water Resources of the Flathead Lake Area: Flathead, Lake, Missoula, and Sanders Counties,	Meinzer, O.E. (1916a). Artesian water for irrigation in Little Bitterroot Valley, Montana. Water Supply Paper 400, 34 pp. Accessed April 7, 2021 from	-	Approximated from Fig. 2 of LaFave et al. (2004) and Fig. 1 of Meinzer (1916a).

Aquifer system	Broader aquifer system	Reference A	Reference B	Reference C	Steps to delineate
		Montana. Part A – Descriptive Overview and Water-Quality Data.Montana Bureau of Mines and Geology, Butte, MT. Montana Ground-Water Assessment Atlas 2. Accessed March 2, 2021 from http://mbmg.mtech.edu/pdf/GWA_2.pdf	https://pubs.usgs.gov/wsp/0400b/report.pdf		
**Little Chino Valley**	-	Kennedy, J.R., Kahler, L.M., Read, A.L. (2019). Aquifer storage change and storage properties, 2010–2017, in the Big Chino Subbasin, Yavapai County, Arizona: U.S. Geological Survey Scientific Investigations Report 2019–5060, 39 pp. Accessed November 29, 2021 from https://pubs.usgs.gov/sir/2019/5060/sir20195060.pdf	-	-	Approximated from Fig. 1 of Kennedy et al. (2019)
**Little Lake Valley**	-	California Department of Water Resources (2015j). California's groundwater update 2013 - Chapter 3: North Coast Hydrologic Region. California Department of Water Resources Report, 80 pp. Accessed April 27, 2022 via https://water.ca.gov/-/media/DWR-Website/Web-Pages/Programs/Groundwater-Management/Bulletin-118/Files/Statewide-Reports/GWU2013_Ch3_NorthCoast_Final.pdf	-	-	Approximated from Fig. 3-2 by California Department of Water Resources (2015j)

Aquifer system	Broader aquifer system	Reference A	Reference B	Reference C	Steps to delineate
**Little Lost River Valley**	Eastern Snake River Plain	Graham, W.G., Campbell, L.J. (1981). Groundwater resources of Idaho. Idaho Department of Water Resources Report, 61 pp. Accessed March 23, 2021 from https://idwr.idaho.gov/files/publications/198108-MISC-GW-Resources-ID.pdf	-	-	Approximated from Plate 1 of Graham and Campbell (1981).
**Little Spokane Basin**	Columbia Plateau Regional Aquifer System	Kahle, S.C., Morgan, D.S., Welch, W.B., Ely, S.R., Vaccaro, J.J., Orzol, L.L. (2011). Hydrogeologic Framework and Hydrologic Budget Components of the Columbia Plateau Regional Aquifer System, Washington, Oregon, and Idaho. US Geological Survey Scientific Investigations Report 2011–5124, 80 pp. Accessed February 16, 2021 from https://pubs.usgs.gov/sir/2011/5124/pdf/sir20115124.pdf	Kahle, S.C., Olsen, T.D., Fasser, E.T. (2013). Hydrogeology of the Little Spokane River Basin, Spokane, Stevens, and Pend Oreille Counties, Washington. US Geological Survey Scientific Investigations Report 2013-5124, 64 pp. Accessed February 18, 2021 from https://pubs.usgs.gov/sir/2013/5124/pdf/sir20135124.pdf	-	Little Spokane Basin approximated from Fig. 5 of Kahle et al. (2012). Columbia River Aquifer System approximated from Figs. 1 and 10 of Kahle et al. (2011).
**Livermore Valley**	-	California Department of Water Resources (2015g). California's groundwater update 2013 - Chapter 4: San Francisco Bay Hydrologic Region. California Department of Water Resources Report, 78 pp. Accessed April 27, 2022 via https://cawaterlibrary.net/wp-content/uploads/2017/05/G	-	-	Approximated from Fig. 4-2 by California Department of Water Resources (2015g)

Aquifer system	Broader aquifer system	Reference A	Reference B	Reference C	Steps to delineate
		WU2013_Ch4_SanFrancisco Bay_Final.pdf			
**Ljubljana Barje**	Ljubljana Alluvial Aquifer	Vizintin, G., Souvent, P., Veselič, M., Curk, B. C. (2009). Determination of urban groundwater pollution in alluvial aquifer using linked process models considering urban water cycle. Journal of Hydrology, 377, 261-273.	-	-	Approximated from Fig. 3 by Vizintin, et al. (2009)
**Ljubljana Polje**	Ljubljana Alluvial Aquifer	Vizintin, G., Souvent, P., Veselič, M., Curk, B. C. (2009). Determination of urban groundwater pollution in alluvial aquifer using linked process models considering urban water cycle. Journal of Hydrology, 377, 261-273.	-	-	Approximated from Fig. 3 by Vizintin, et al. (2009)
**Llagas Area**	Gilroy-Hollister Valley	California Department of Water Resources (2015g). California's groundwater update 2013 - Chapter 4: San Francisco Bay Hydrologic Region. California Department of Water Resources Report, 78 pp. Accessed April 27, 2022 via https://cawaterlibrary.net/wp-content/uploads/2017/05/GWU2013_Ch4_SanFrancisco Bay_Final.pdf	-	-	Approximated from Fig. 4-2 by California Department of Water Resources (2015g)
**Lo Orozco Aquifer**	-	Direccion General de Aguas (2017). Inventario Nacional de acuiferos. Ministerio De Obras Públicas (Gobierno De Chile) report number 403, 60 pp. Accessed May	-	-	Approximated from map on page 40 by Direccion General de Aguas (2017).

Aquifer system	Broader aquifer system	Reference A	Reference B	Reference C	Steps to delineate
		17, 2021 from https://snia.mop.gob.cl/sad/SUB5748.pdf			
**Lo Ovalle Aquifer**	-	Direccion General de Aguas (2017). Inventario Nacional de acuiferos. Ministerio De Obras Públicas (Gobierno De Chile) report number 403, 60 pp. Accessed May 17, 2021 from https://snia.mop.gob.cl/sad/SUB5748.pdf	-	-	Approximated from map on page 40 by Direccion General de Aguas (2017).
**Lobo Flat**	Salt Basin Bolson	Beach, J.A., Ashworth, J.B., Finch, S.T., Chaistain-Howley, A., Calhoun, K., Urbanczyk, K.M., Sharp, J.M., Olsonm J. (2004a). Groundwater availability model for the igneous and parts of the West Texas Bolsons (Wild Horse Flat, Michigan Flat, Ryan Flat and Lobo Flat) aquifers. Texas Water Development Board Report, 407 pp. Accessed May 1, 2023 via https://www.twdb.texas.gov/groundwater/models/gam/igbl/IGBL_Model_Report.pdf	-	-	Approximated from Fig. 2.1.3 by Beach et al. (2004a)
**Lochiel Lobe**	St Vincent Basin	Smith, M. L., Fontaine, K., Lewis, S. J. (2015). Regional Hydrogeological Characterisation of the St Vincent Basin, South Australia: Technical Report for the National Collaboration Framework Regional Hydrogeology Project. Geoscience Australia Record 2015/16,	-	-	Approximated from Fig. 2.2 by Smith et al. (2015)

Aquifer system	Broader aquifer system	Reference A	Reference B	Reference C	Steps to delineate
		150 pp. Accessed July 27, 2021 from https://d28rz98at9flks.cloudfront.net/78884/Rec2015_016.pdf			
**Logar Aquifer**	Kabul Basin	Zaryab, A., Nassery, H. R., Alijani, F. (2021). Identifying sources of groundwater salinity and major hydrogeochemical processes in the Lower Kabul Basin aquifer, Afghanistan. Environmental Science: Processes & Impacts, 23, 1589-1599.	Mack, T. J., Chornack, M. P., Taher, M. R. (2013). Groundwater-level trends and implications for sustainable water use in the Kabul Basin, Afghanistan. Environment Systems and Decisions, 33, 457-467.	Saffi, M.H. (2019). National alarming on groundwater natural storage depletion and water quality deterioration of Kabul City and immediate response to the drinking water crises. Scientific INvestigation Report in Afghanistan, DACAAR Report, 88 pp.	Approximated from Fig. 1 by Zaryab et al. (2021), Fig. 12 by Saffi (2019), and Fig. 1 by Mack et al. (2013). Northeastern boundary defined along the Chakari River and Lower Kabul River
**Lolo-Bitterroot Valley**	Lolo-Bitterroot -Missoula Valleys	Briar, D. W., Dutton, D. M. (1999). Hydrogeology and Aquifer Sensitivity of the Bitterroot Valley, Ravalli County, Montana. Water-Resources Investigations Report, 99-4219. Accessed March 2, 2021 from https://pubs.usgs.gov/wri/1999/4219/report.pdf	Smith, L. N., LaFave, J., I., Patton, T. W. (2013). Groundwater resources of the Lolo-Bitterroot area: Mineral, Missoula, and Ravalli Counties, Montana. Montana Bureau of Mines and Geology. Montana Groundwater Assessment Atlas No. 4. Accessed March 2, 2021 from http://www.mbmg.mtech.edu/pdf-publications/gwaa4a.pdf	Smith, L. N. (2006). Hydrologic framework of the Lolo-Bitterroot Area ground-water characterization study : Montana Bureau of Mines and Geology Montana Ground-Water Assessment Atlas 4-B-02, 1 sheet, scale 1:250,000. Accessed March 2, 2021 from http://mbmg.mtech.edu/pdf-publications/GWAA04B-02.pdf	Approximated from Fig. 1 of Birar and Dutton (1999), Fig. 2 of Smith et al. (2003) and map by Smith (2006).
**Lom- Pleven Depression**	-	Mihaylova, B., Stevanović, Z., Toteva, A., Milanović, S., Kostov, K., Vasić, L., & Benderev, A. (2022). Hydrogeological conditions on the border between Serbia and Bulgaria to assess the transboundary groundwater transfer. Transboundary Aquifers, Topic 3 Paper 11, pp. 110-107. Accessed May 22,	-	-	Approximated from Fig. 1 and Table 1 by Mihaylova et al. (2022)

Aquifer system	Broader aquifer system	Reference A	Reference B	Reference C	Steps to delineate
		2023 via http://www.indiaenvironmentportal.org.in/files/file/Transboundary%20aquifers.pdf#page=101			
**Lonesome and Prescott Valleys**	-	Matlock, W. G., Davis, P. R., Roth, R. L. (1973). Groundwater in Little Chino Valley, Arizona: Tucson, University of Arizona, College of Agriculture, Agricultural Experiment Station. Technical Bulletin, 201, 19. Accessed March 12, 2021 from https://repository.arizona.edu/bitstream/handle/10150/602177/TB178.pdf?sequence=1	-	-	Approximated from Plate 1 of Matlock et al. (1973)
**Long Island**	-	Smolensky, D.A., Buxton, H.T., Shernoff, P.K. (1990). Hydrologic framework of Long Island, New York. U.S. Geological Survey Hydrologic Atlas 709, 3 plates, Accessed April 1, 2021 from https://pubs.usgs.gov/ha/709/plate-1.pdf	Olcott, P.G. (1995). Ground Water Atlas of the United States: Segment 12, Connecticut, Maine, Massachusetts, New Hampshire, New York, Rhode Island, Vermont. U.S. Geological Survey Hydrologic Atlas 730-M, 30 pp. Accessed April 14, 2021 from https://pubs.usgs.gov/ha/730m/report.pdf	Masterson, J. P., Pope, J. P., Fienen, M. N., Monti Jr, J., Nardi, M. R., Finkelstein, J. S. (2016). Assessment of groundwater availability in the Northern Atlantic Coastal Plain aquifer system from Long Island, New York, to North Carolina (No. 1829). US Geological Survey Professional Paper 1829, 90 pp. Accessed November 29, 2021 from https://pubs.usgs.gov/pp/1829/pp1829.pdf	Approximated from Plate 1 of Smolensky et al. (1990), Fig. 6 by Masterson et al. (2016), and Fig. 62 of Olcott (1995).
**Long Valley**	-	Lopes, T.J., Evetts. D.M. (2005). Ground-Water pumpage and artificial recharge estimates for calendar year 2000 and average annual natural recharge and interbasin	Harrill, J.R., Prudic, D.E. (1998). Aquifer systems in the Great Basin region of Nevada, Utah, and adjacent states; summary report. US Geological Survey Professional Paper 1409-A,	-	Approximated from Fig. 1 by Lopes and Evetts (2005), and Fig. 2 by Harrill and Prudic (1998). Well completion records, physiography and drainage were used to delinate areas.

Aquifer system	Broader aquifer system	Reference A	Reference B	Reference C	Steps to delineate
		flow by hydrographic area, Nevada. Accessed April 25, 2022 via https://pubs.usgs.gov/sir/2004/5239/sir2004-5239.pdf	75 pp. Accessed April 26, 2022 via https://pubs.usgs.gov/pp/1409a/report.pdf		
**Long Valley - Round Valley**	-	Graham, W.G., Campbell, L.J. (1981). Groundwater resources of Idaho. Idaho Department of Water Resources Report, 61 pp. Accessed March 23, 2021 from https://idwr.idaho.gov/files/publications/198108-MISC-GW-Resources-ID.pdf	-	-	Approximated from Plate 1 of Graham and Campbell (1981).
**Long Valley in central Nevada**	-	Lopes, T.J., Evetts. D.M. (2005). Ground-Water pumpage and artificial recharge estimates for calendar year 2000 and average annual natural recharge and interbasin flow by hydrographic area, Nevada. Accessed April 25, 2022 via https://pubs.usgs.gov/sir/2004/5239/sir2004-5239.pdf	Harrill, J.R., Prudic, D.E. (1998). Aquifer systems in the Great Basin region of Nevada, Utah, and adjacent states; summary report. US Geological Survey Professional Paper 1409-A, 75 pp. Accessed April 26, 2022 via https://pubs.usgs.gov/pp/1409a/report.pdf	-	Approximated from Fig. 1 by Lopes and Evetts (2005), and Fig. 2 by Harrill and Prudic (1998). Well completion records, physiography and drainage were used to delinate areas.
**Long Valley in eastern California**	-	California Department of Water Resources (2015d). California's groundwater update 2013 - Chapter 10: North Lahontan Hydrologic Region. California Department of Water Resources Report, 72 pp. Accessed April 27, 2022 via https://water.ca.gov/-/media/DWR-Website/Web-Pages/Programs/Groundwater-Management/Bulletin-118/Files/Statewide-	-	-	Approximated from Fig. 10-2 by California Department of Water Resources (2015d)

Aquifer system	Broader aquifer system	Reference A	Reference B	Reference C	Steps to delineate
		Reports/GWU2013_Ch10_NorthLahontan_Final.pdf			
**Long Valley near Crowley Lake**	-	California Department of Water Resources (2015d). California's groundwater update 2013 - Chapter 10: North Lahontan Hydrologic Region. California Department of Water Resources Report, 72 pp. Accessed April 27, 2022 via https://water.ca.gov/-/media/DWR-Website/Web-Pages/Programs/Groundwater-Management/Bulletin-118/Files/Statewide-Reports/GWU2013_Ch10_NorthLahontan_Final.pdf	-	-	Approximated from Fig. 10-2 by California Department of Water Resources (2015d)
**Longford Tertiary Basin**	-	Water and Marine Resources Division (2012). Tasmanian Aquifer Framework. Groundwater Management Report Series. Report No. GW 2012/02 39 pp. Accessed October 8, 2021 from https://stors.tas.gov.au/au-7-0054-00588$stream	-	-	Approximated from Appendix C by Water and Marine Resources Division (2012)
**Lordsburg Valley**	-	Schwennesen, A.T., Hare, R.F. (1918). Ground water in the Animas, Playas, Hachita, and San Luis Basins, New Mexico, with analyses of water and soil. U.S. Geological Survey Water Supply Paper 422, 157 pp. Accessed March 29, 2021 from https://pubs.usgs.gov/wsp/0422/report.pdf	-	-	Approximated from Plate II of Schwennesen and Hare (1918).

Aquifer system	Broader aquifer system	Reference A	Reference B	Reference C	Steps to delineate
**Loreto Aquifer**	-	CONAGUA (2020h). Actualización de la disponibilidad media anual de agua en el acuífero Loreta (3229), Estado de Zacatecas. 36 pp. Accessed January 24, 2022 via https://sigagis.conagua.gob.mx/gas1/Edos_Acuiferos_18/zacatecas/DR_3229.pdf	-	-	Approximated from Fig. 1 by CONAGUA (2020h)
**Los Molinos Basin**	Sacramento Valley of the Central Valley **	California Department of Water Resources (2015i). California's groundwater update 2013 - Chapter 7: Sacramento River Hydrologic Region. California Department of Water Resources Report, 126 pp. Accessed April 27, 2022 via https://water.ca.gov/-/media/DWR-Website/Web-Pages/Programs/Groundwater-Management/Bulletin-118/Files/Statewide-Reports/GWU2013_Ch7_SacramentoRiver_Final.pdf	-	-	Approximated from Fig. 7-2 by California Department of Water Resources (2015i)
**Los Naranjos Aquifer**	Coastal Plain of Veracruz	Camacho, E. A. S. (2010). Estimación del volumen promedio recibido por el humedal de la subcuenca del Río Blanco (bajo Papaloapan; Veracruz), a través del cálculo de un balance de aguas. Aqua-LAC, 2, 78-87.	Campos C, A., Hernández, M. E., Moreno-Casasola, P., Cejudo Espinosa, E., Robledo R, A., Infante Mata, D. (2011). Soil water retention and carbon pools in tropical forested wetlands and marshes of the Gulf of Mexico. Hydrological Sciences Journal, 56, 1388-1406.	-	Approximated from Fig. 6 by Camacho (2010). Broader Coastal Plain of Veracruz approximated from Fig. 1 by Campos et al. (2011).
**Lower Aconcagua Basin**	Aconcagua Basin	Direccion General de Aguas (2009). Diagnóstico y Clasificación de Sectores	-	-	Approximated from Table 1.8 on page 40 by Direccion General de Aguas (2009). Divide between

Aquifer system	Broader aquifer system	Reference A	Reference B	Reference C	Steps to delineate
		Acuíferos, Volumen No. 2. Gobierno de Chile Ministerio de Obras Publicas Report, 384 pp. Accessed May 16, 2021 from https://snia.mop.gob.cl/sad/CQA5168v2.pdf			lower and middle portions of aquifer system approximated near Calera.
**Lower Amargosa Valley**	-	Lopes, T.J., Evetts. D.M. (2005). Ground-Water pumpage and artificial recharge estimates for calendar year 2000 and average annual natural recharge and interbasin flow by hydrographic area, Nevada. Accessed April 25, 2022 via https://pubs.usgs.gov/sir/2004/5239/sir2004-5239.pdf	Harrill, J.R., Prudic, D.E. (1998). Aquifer systems in the Great Basin region of Nevada, Utah, and adjacent states; summary report. US Geological Survey Professional Paper 1409-A, 75 pp. Accessed April 26, 2022 via https://pubs.usgs.gov/pp/1409a/report.pdf	-	Approximated from Fig. 1 by Lopes and Evetts (2005), and Fig. 2 by Harrill and Prudic (1998). Well completion records, physiography and drainage were used to delinate areas.
**Lower Arkansas River Eastern Reach**	-	Holmberg, M.J. (2017). Hydrogeologic characteristics and geospatial analysis of water-table changes in the alluvium of the lower Arkansas River Valley, southeastern Colorado, 2002, 2008, and 2015: U.S. Geological Survey Scientific Investigations Map 3378, 20 pp. Accessed March 29, 2021 from https://pubs.usgs.gov/sim/3378/sim3378.pdf	-	-	Approximated from Fig. 1 of Holmgren (2017); eastern and western reaches separated by John Martin Reservoir.
**Lower Arkansas River Western Reach**	-	Holmberg, M.J. (2017). Hydrogeologic characteristics and geospatial analysis of water-table changes in the alluvium of the lower Arkansas River Valley,	-	-	Approximated from Fig. 1 of Holmgren (2017); eastern and western reaches separated by John Martin Reservoir.

Aquifer system	Broader aquifer system	Reference A	Reference B	Reference C	Steps to delineate
		southeastern Colorado, 2002, 2008, and 2015: U.S. Geological Survey Scientific Investigations Map 3378, 20 pp. Accessed March 29, 2021 from https://pubs.usgs.gov/sim/3378/sim3378.pdf			
**Lower Athabasca**	Lower Athabasca Basin	Miall, A.D. (2013). Geoscience of Climate and Energy 13. The Environmental Hydro-geology of the Oil Sands, Lower Athabasca Area, Alberta. http://dx.doi.org/10.12789/geocanj.2013.40.016	-	-	Approximated from Fig. 1 of Miall (2013) and also from well completion reports and topography.
**Lower Borrego and Ocotillo-Clark Valleys**	-	Faunt, C.C., Stamos, C.L., Flint, L.E., Wright, M.T., Burgess, M.K., Sneed, M., Brandt, J., Martin, P., Coes, A.L. (2015). Hydrogeology, hydrologic effects of development, and simulation of groundwater flow in the Borrego Valley, San Diego County, California. US Geological Survey Scientific Investigations Report 2015–5150, 154 pp. Accessed April 27, 2022 via https://pubs.usgs.gov/sir/2015/5150/sir20155150.pdf	California Department of Water Resources (2016). Borrego Valley - Ocotillo Wells Basin Boundaries. California Department of Water Resources, 5 pp. Accessed April 27, 2022 via https://water.ca.gov/-/media/DWR-Website/Web-Pages/Programs/Groundwater-Management/Bulletin-118/Files/2016-Basin-Boundary-Descriptions/7_024_02_OcotilloWells.pdf	-	Approximated from Fig. 6 by Faunt et al. (2015) and map by California Department of Water Resources (2016).
**Lower Coastal Plain**	Floridan Aquifer System	Williams, L.J., Kuniansky, E.L. (2016). Revised hydrogeologic framework of the Floridan aquifer system in Florida and parts of Georgia, Alabama, and South Carolina. U.S.	Aucott, W.R. (1996). Hydrology of the Southeastern Coastal Plain Aquifer System in South Carolina and Parts of Georgia and North Carolina. U.S Geological Survey	-	Broader Florida Aquifer System from Williams and Kuniansky (2016). Approximate divide between the Upper and Lower Coastal Plain from Fig. 1 of Aucott (1996).

Aquifer system	Broader aquifer system	Reference A	Reference B	Reference C	Steps to delineate
		Geological Survey Professional Paper 1807, 140 pp. Accessed March 31, 2021 from https://pubs.usgs.gov/pp/1807/pdf/pp1807.pdf	Professional Paper 1410-E, 95 pp. Accessed March 31, 2021 from https://pubs.usgs.gov/pp/1410e/report.pdf		
**Lower Copiapo Valley**	Copiapo Valley	Donoso, G., Lictevout, E., Rinaudo, J. D. (2020). Groundwater management lessons from Chile. In: Sustainable Groundwater Management. Springer, Cham., pp. 481-509. Accessed May 12, 2021 from https://link.springer.com/chapter/10.1007/978-3-030-32766-8_25	Direccion General de Aguas (2015). Plan Nacional de Estudios Acuíferos. Report number 381, Santiago, Chile, 44 pp. Accessed May 16, 2021 from http://bibliotecadigital.ciren.cl/bitstream/handle/123456789/32415/DGA_2015_actualizacion_plan_nacional_acuiferos.pdf?sequence=1&isAllowed=y	-	Approximated from Fig. 25.2 by Donoso et al. (2020) and Fig. 2a by Direccion General de Aguas (2015)
**Lower Fortescue Valley**	Hamersley Basin	Simonson, B. M., Schubel, K. A., Hassler, S. W. (1993). Carbonate sedimentology of the early Precambrian Hamersley Group of western Australia. Precambrian Research, 60, 287-335.	-	-	Approximated from Hamersley Basin outline in Fig. 1 by Simonson et al. (1993) and local physiography
**Lower Fraser Valley**	Puget Sound Lowland	Vaccaro, J. J., Hansen, A. J., Jones, M. A. (1998). Hydrogeologic framework of the Puget Sound aquifer system, Washington and British Columbia. U.S. Geological Survey Professional Paper 1424-D, 87 pp. Accessed March 11, 2021 from https://pubs.usgs.gov/pp/1424d/report.pdf	Wilson, J. E., Brown, S., Schreier, H., Scovill, D., Zubel, M. (2008). Arsenic in groundwater wells in Quaternary deposits in the Lower Fraser Valley of British Columbia. Canadian Water Resources Journal, 33(4), 397-412.	-	Broader Puget Sound Lowland approximated from Vaccaro et al. (1998). Fraser River Delta outline approximated from Fig. 1 of Bridger and Allen (2006).
**Lower Gascoyne**	-	Leonhard, L., Burton, K., Milligan, N. (2013). Gascoyne River, Western	-	-	Approximated from Figs. 17.1 and 17.2 by Leonhard et al. (2013)

Aquifer system	Broader aquifer system	Reference A	Reference B	Reference C	Steps to delineate
**Alluvial Aquifer**		Australia; Alluvial Aquifer, Groundwater Management and Tools. In: Groundwater in the Coastal Zones of Asia-Pacific, pp. 359-378. Springer, Dordrecht.			
**Lower Gila Basin**	-	Tillman, F.D, Cordova, J.T., Leake, S.A., Thomas, B.E., Callegary, J.B. (2011). Water availability and use pilot: methods development for a regional assessment of groundwater availability, southwest alluvial basins, Arizona. U.S. Geological Survey Scientific Investigations Report 2011-5071, 132 pp. Accessed April 15, 2021 from https://pubs.usgs.gov/sir/2011/5071/sir2011-5071_text.pdf	-	-	Approximated from Fig. 2 of Tillman et al. (2011); boundaries also informed by the density of wells recorded in Arizona's well completion dataset.
**Lower Herbert Aquifer**	-	Hunter, H. (2012). Nutrients and herbicides in groundwater flows to the Great Barrier Reef lagoon. Report by individual associated with the Australian Rivers Institute and Griffith University, 86 pp. Accessed May 7, 2023 via https://www.qld.gov.au/__data/assets/pdf_file/0027/69066/rp51c-grounderwater-synthesis-great-barrier-reef.pdf	-	-	Approximated from Fig. 1.1 and descriptions within Hunter (2012)
**Lower Kabootar Ahang Basin**	Razan Basin	Hosseini, S. M., Parizi, E., Ataie-Ashtiani, B., Simmons, C. T. (2019). Assessment of sustainable	-	-	Approximated from Fig. 6 by Hosseini et al. (2019)

Aquifer system	Broader aquifer system	Reference A	Reference B	Reference C	Steps to delineate
		groundwater resources management using integrated environmental index: Case studies across Iran. Science of the Total Environment, 676, 792-810.			
**Lower Klamath Lake**	Upper Klamath Basin	Gannett, M.W., Breen, K.H. (2015). Groundwater levels, trends, and relations to pumping in the Bureau of Reclamation Klamath Project, Oregon and California: U.S. Geological Survey Open-File Report 2015-1145, 19 pp. Accessed March 20, 2021 from https://pubs.usgs.gov/of/2015/1145/ofr20151145.pdf	-	-	Approximated from Fig. 1 of Gannett and Breen (2015).
**Lower La Ligua Aquifer**	La Ligua Aquifer	Direccion General de Aguas (2009). Diagnóstico y Clasificación de Sectores Acuíferos, Volumen No. 2. Gobierno de Chile Ministerio de Obras Publicas Report, 384 pp. Accessed May 16, 2021 from https://snia.mop.gob.cl/sad/CQA5168v2.pdf	-	-	Approximated from Table 1.7 on page 37 by Direccion General de Aguas (2009). Lower and upper portions of the aquifer system delineated near Cabildo.
**Lower Liaohe River Plain**	Liaohe River Basin	Teng, Y., Zuo, R., Xiong, Y., Wu, J., Zhai, Y., Su, J. (2019). Risk assessment framework for nitrate contamination in groundwater for regional management. Science of The Total Environment, 697, 134102.	-	-	Approximated from Fig. 1 by Teng et al. (2019)
**Lower Limestone**	Murray Basin	Herczeg, A. L., Dogramaci, S. S., Leaney, F. W. J.	Department of Environment and Water of the	-	Approximated from Fig. 1 by Herczeg et al. (2001), and map

Aquifer system	Broader aquifer system	Reference A	Reference B	Reference C	Steps to delineate
**Coast Aquifer System**		(2001). Origin of dissolved salts in a large, semi-arid groundwater system: Murray Basin, Australia. Marine and Freshwater Research, 52, 41-52.	Government of South Australia (2017). Lower Limestone Coast PWA Unconfined Aquifer 2017 Groundwater level and salinity status report, 13 pp. Accessed July 21, 2021 from https://www.waterconnect.sa.gov.au/Content/Publications/DEW/Lower_Limestone_Coast_PWA_Unconfined_GSR_2017.pdf		and descriptions on page 4 of Department of Environment and Water of the Government of South Australia (2017)
**Lower Meadow Valley Wash**	-	Lopes, T.J., Evetts. D.M. (2005). Ground-Water pumpage and artificial recharge estimates for calendar year 2000 and average annual natural recharge and interbasin flow by hydrographic area, Nevada. Accessed April 25, 2022 via https://pubs.usgs.gov/sir/2004/5239/sir2004-5239.pdf	Harrill, J.R., Prudic, D.E. (1998). Aquifer systems in the Great Basin region of Nevada, Utah, and adjacent states; summary report. US Geological Survey Professional Paper 1409-A, 75 pp. Accessed April 26, 2022 via https://pubs.usgs.gov/pp/1409a/report.pdf	-	Approximated from Fig. 1 by Lopes and Evetts (2005), and Fig. 2 by Harrill and Prudic (1998). Well completion records, physiography and drainage were used to delinate areas.
**Lower Morovian Basin**	-	Kadlecová, R., Olmer, M. (2011). Review of Groundwater Resources. Geologické výzkumy na Moravě a ve Slezsku. Proceedings of The Open Congress ČGS and SGS, September 22–25, 2011, Monínec 18, pp. 31-34. Accessed October 21, 2021 from https://journals.muni.cz/gvms/article/view/1679	Labus, K., Bujok, P., Klempa, M., Porzer, M., Matýsek, D. (2016). Preliminary geochemical modeling of water–rock–gas interactions controlling CO 2 storage in the Badenian Aquifer within Czech Part of Vienna Basin. Environmental Earth Sciences, 75, 1-13.	-	Approximated from Fig. 2 by Kadlecová and Olmer (2011) and Fig. 1 by Labus et al. (2016)
**Lower Mur Valley**	-	Retter, A., Griebler, C., Haas, J., Birk, S., Stumpp, C., Brielmann, H., Fillinger,	-	-	Approximated from Fig. 1 by Retter et al. (2021)

Aquifer system	Broader aquifer system	Reference A	Reference B	Reference C	Steps to delineate
		L. (2021). Application of the DA-(C) index as a simple tool for microbial-ecological characterization and assessment of groundwater ecosystems—a case study of the Mur River Valley, Austria. Österr Wasser- und Abfallw, https://doi.org/10.1007/s00506-021-00799-5			
**Lower Nowbaran Basin**	-	Hosseini, S. M., Parizi, E., Ataie-Ashtiani, B., Simmons, C. T. (2019). Assessment of sustainable groundwater resources management using integrated environmental index: Case studies across Iran. Science of the Total Environment, 676, 792-810.	-	-	Approximated from Fig. 6 by Hosseini et al. (2019)
**Lower Qeydar Basin**	Qeydar Basin	Hosseini, S. M., Parizi, E., Ataie-Ashtiani, B., Simmons, C. T. (2019). Assessment of sustainable groundwater resources management using integrated environmental index: Case studies across Iran. Science of the Total Environment, 676, 792-810.	-	-	Approximated from Fig. 6 by Hosseini et al. (2019)
**Lower Rio Elqui Basin**	Elqui River Basin	Ribeiro, L., Kretschmer, N., Nascimento, J., Buxo, A., Rötting, T., Soto, G., Señoret, M., Oyarzún, J., Maturana, H., Oyarzún, R. (2015). Evaluating piezometric trends using the Mann-Kendall test on	Squeo, F. A., Aravena, R., Aguirre, E., Pollastri, A., Jorquera, C. B., Ehleringer, J. R. (2006). Groundwater dynamics in a coastal aquifer in north-central Chile: implications for groundwater recharge in an	-	Approximated from Fig. 1 by Ribeiro et al. (2015); aquifer extended along the coastal plain north and south of the Rio Elqui watershed (see southwestern portion of study area by Squeo et al. (2006)).

Aquifer system	Broader aquifer system	Reference A	Reference B	Reference C	Steps to delineate
		the alluvial aquifers of the Elqui River basin, Chile. Hydrological Sciences Journal, 60, 1840-1852.	arid ecosystem. Journal of Arid Environments, 67, 240-254.		
**Lower Rio Huasco Basin**	Rio Huasco Basin	Direccion General de Aguas (2021). Analisis de disponibilidad de recursos hidricos subterraneous en el sector hidrogeologico de aprovechamiento comun Huasco Desembocadura, Cuencas Rio Huasco. Report No 14593214, 19 pp. Accessed May 17, 2021 from https://dga.mop.gob.cl/Decretos_Escacez/0303-2.pdf	-	-	Approximated from Fig. 1 by Direccion General de Aguas (2021); eastern boundary extended up-valley
**Lower Rio Paz Aquifer**	Rio Paz Aquifer	Arreguín, F., López-Pérez, M., Galván, R. (2018). Acuíferos transfronterizos en México: análisis normativo hacia una estrategia de manejo, Tecnologia y Ciencias del Agua 9, 1-38.	-	-	Approximated from Fig. 3 by Arreguín et al. (2018)
**Lower Rio Petorca Basin**	Rio Petorca Basin	Bujes Moreno, N.J.I. (2015). Estudio de la propiedad del agua subterránea del acuífero del Río Petorca en la Región de Valparaíso, Chile. Universidad de Chile Thesis, 72 pp. Accessed May 17, 2021 from http://repositorio.uchile.cl/handle/2250/150902	-	-	Approximated from Fig. 12 by Bujes Moreno (2015)
**Lower Santa Ynez Valley**	Santa Ynez Valley	Upson, J. E., Thomasson, H. G. (1951). Geology and water resources of the Santa Ynez river basin, Santa Barbara County,	Hamlin, S. N. (1985). Ground-water quality in the Santa Rita, Buellton, and Los Olivos hydrologic subareas of the Santa Ynez	Bright, D.J., Stamos, C.L., Martin, P.M., Nash, D.B. (1992). Ground-water hydrology and quality in the Lompoc area, Santa	Approximated from Fig. 1 by Upson and Thomasson (1951), pp. 22 map in Hamlin (1985), and Plate 1 by Bright et al. (1997). Lower portion of Valley based on

Aquifer system	Broader aquifer system	Reference A	Reference B	Reference C	Steps to delineate
		California (Vol. 2). US Geological Survey Water Supply Report 1102, Accessed March 7, 2021 from https://pubs.usgs.gov/wsp/1107/report.pdf	River basin, Santa Barbara County, California. Water Resources Investigations Report 84-4131, 84 pp. Accessed March 7, 2021 from https://pubs.usgs.gov/wri/1984/4131/report.pdf	Barbara County, California, 1987-88. US Geological Survey Water-Resources Investigations Report 91-4172, 82 pp. Accessed April 17, 2022 via https://pubs.usgs.gov/wri/1991/4172/report.pdf	the delineated subareas: "Lompoc" and "Santa Rita" and "Buellton" subarea
**Lower Souss Basin**	Souss-Chtouka Basin	Garduño, H., Foster, S. (2010). Sustainable Groundwater Irrigation. Approaches to Reconciling Demand with Resources. GW•MATE Strategic Overview Series No. 4 World Bank (Washington DC, USA). Accessed 05/01/2023 via https://openknowledge.worldbank.org/server/api/core/bitstreams/a6957092-3680-52cd-9707-91143c386175/content	Tagma, T., Hsissou, Y., Bouchaou, L., Bouragba, L., Boutaleb, S. (2009). Groundwater nitrate pollution in Souss-Massa basin (south-west Morocco). African Journal of Environmental Science and Technology, 3, 301-309.	Bouchaou, L., Michelot, J.L., Vengosh, A., Hsissou, Y., Qurtobi, M., Gaye, C.B., Bullen, T.D., Zuppi, G. M. (2008). Application of multiple isotopic and geochemical tracers for investigation of recharge, salinization, and residence time of water in the Souss–Massa aquifer, southwest of Morocco. Journal of Hydrology, 352, 267-287.	Broader aquifer system boundaries approximated from Box 5 by Garduño and Foster (2010), Fig. 1 by Bouchaou et al. (2008), and Fig. 1 by Tagma et al. (2009). Southern limit approximated along the Massa River.
**Lower Waitaki South Coastal Canterbury Aquifer**	-	Scott, L., Hanson, C., Cressy, C. (2012). Groundwater quality investigation of the mid-Waitaki valley. Environment Canterbury Regional Council Kaunihera Taiao ki Waitaha Report, 77 pp. Accessed January 25, 2022 from http://citeseerx.ist.psu.edu/viewdoc/download?doi=10.1.1.799.6506&rep=rep1&type=pdf	-	-	Approximated from Fig. 1 by Scott et al. (2012)
**Lower Walker Basin**	-	Allander, K. K., Niswonger, R. G., Jeton, A. E. (2014). Simulation of the Lower Walker River Basin	-	-	Approximated from Fig. 1 of Allander et al. (2014) with guidance from completed well locations

Aquifer system	Broader aquifer system	Reference A	Reference B	Reference C	Steps to delineate
		hydrologic system, west central Nevada, using PRMS and MODFLOW models. U.S. Geological Survey Scientific Investigations Report 2014–5190, 108 pp. Accessed March 21, 2021 from https://pubs.usgs.gov/sir/2014/5190/pdf/sir2014-5190.pdf			
**Lund Subarea**	Escalante Valley	Sandberg, G. W. (1964). Ground-water resources of selected basins in southwestern Utah. US Geological Survey Open File Report. 66 pp. Accessed March 7, 2021 from https://cicwcd.org/wp-content/uploads/2018/09/1966-tech-pub-13-ground-water-resources-of-selected-basins-in-southwestern-utah-by-g-w-sandburg-usgs.pdf	Fix, P. F., Nelson, W. B., Lofgren, B. E., Butler, R. G. (1950). Ground water in the Escalante Valley, Beaver, Iron, and Washington Counties, Utah Technical Publication 6. 102 pp. Accessed March 7, 2021 from https://waterrights.utah.gov/docSys/v920/w920/w9200085.pdf	-	Approximated from Fig. 1 of Fix et al. (1950) with subareas ("Districts") approximated from the map on page 49 of Sandberg (1966).
**Lyonnais and South Dombes Miocene Aquifer System**	-	Commission locale de l'eau Basse Vallee de l'Ain (2013). Plan d'Aménagement et de Gestion Durable de la ressource en eau et des milieux aquatiques [PAGD]. Commission Locale de l'Eau le 17 Report, 179 pp. Accessed May 1, 2023 via https://www.gesteau.fr/sites/default/files/2-sage_pagd-adopte.pdf	-	-	Approximated from Fig. D by Commission locale de l'eau Basse Vallee de l'Ain (2013)
**Madeline Plains**	-	California Department of Water Resources (2015i). California's groundwater	-	-	Approximated from Fig. 7-2 by California Department of Water Resources (2015i)

Aquifer system	Broader aquifer system	Reference A	Reference B	Reference C	Steps to delineate
		update 2013 - Chapter 7: Sacramento River Hydrologic Region. California Department of Water Resources Report, 126 pp. Accessed April 27, 2022 via https://water.ca.gov/-/media/DWR-Website/Web-Pages/Programs/Groundwater-Management/Bulletin-118/Files/Statewide-Reports/GWU2013_Ch7_SacramentoRiver_Final.pdf			
**Madera Basin**	San Joaquin Basin of the Central Valley **	California Department of Water Resources (2015h). California's groundwater update 2013 - Chapter 8: San Joaquin River Hydrologic Region. California Department of Water Resources Report, 122 pp. Accessed April 28, 2022 via https://water.ca.gov/-/media/DWR-Website/Web-Pages/Programs/Groundwater-Management/Data-and-Tools/Files/Statewide-Reports/California-Groundwater-Update-2013/California-Groundwater-Update-2013---Chapter-8---San-Joaquin-River.pdf	-	-	Approximated from Fig. 8-2 by California Department of Water Resources (2015h)
**Madhupur Tract and Eastern Floodplains**	Bengal Basin	Shamsudduha, M. (2007). Spatial Variability and Prediction Modeling of Groundwater Arsenic Distributions in the Shallowest Alluvial Aquifers	Ravenscroft, P., McArthur, J. M., Rahman, M. S. (2018). Identifying multiple deep aquifers in the Bengal Basin: Implications for resource management.	Michael, H. A., Voss, C. I. (2009). Controls on groundwater flow in the Bengal Basin of India and Bangladesh: regional modeling analysis.	Approximated from Fig. 1 by Shamsudduha (2007), Fig. 1 by Ravenscroft et al. (2018), and Fig. 1 by Michael and Voss (2009)

Aquifer system	Broader aquifer system	Reference A	Reference B	Reference C	Steps to delineate
		in Bangladesh. Journal of Spatial Hydrology, 7, 33-46.	Hydrological Processes, 32, 3615-3632.	Hydrogeology Journal, 17, 1561-1577.	
**Madison Aquifer**	-	LaFave, J. (2011). Quality and Age of Water in the Madison Aquifer, Cascade County, Montana. Montana American Water Resources Association Conference, Session 2. 27 pp. Accessed March 29, 2021 from https://www.montanaawra.org/wp/ppts/2011/session2/5_LaFave_John_i.pdf	Downey, J. S. (1982). Geohydrology of the Madison and associated aquifers in parts of Montana, North Dakota, South Dakota, and Wyoming. U.S. Geological Survey Professional Paper 1273-G, 54 pp. Accessed March 29, 2021 from https://pubs.usgs.gov/pp/1273g/report.pdf	-	While the Madison Aquifer is extensive (spanning multiple states; see Downey (1982)), the relatively small region in central Montana is delineated here as it is "...(m)ost utilized in Cascade Co." (quote LaFave (2011)).
**Madrid-Talavera Detrital Aquifer**	-	Arauzo, M., Martínez-Bastida, J. J. (2015). Environmental factors affecting diffuse nitrate pollution in the major aquifers of central Spain: groundwater vulnerability vs. groundwater pollution. Environmental Earth Sciences, 73, 8271-8286.	Llamas, M. R., Simpson, E. S., Alfaro, P. E. M. (1982). Ground-water age distribution in Madrid Basin, Spain. Groundwater, 20, 688-695.	-	Approximated from Fig. 1 by Arauzo and Martínez-Bastida (2015) and Fig. 1 by Llamas et al. (1982)
**Mahabad Plain**	-	Javadzadeh, H., Ataie-Ashtiani, B., Hosseini, S. M., Simmons, C. T. (2020). Interaction of lake-groundwater levels using cross-correlation analysis: A case study of Lake Urmia Basin, Iran. Science of The Total Environment, 729, 138822.	-	-	Approximated from Fig. 1 by Javadzadeh et al. (2020)
**Mahanadi Basin**	Son-Mahandai Valley Basins	Chowdari, S., Singh, B., Rao, B. N., Kumar, N., Singh, A. P., Chandrasekhar, D. V. (2017). Structural mapping based on potential field and	Dutta, P.K., Acharyya, S.K., Chakraborty, T., Jha, N., Khangar, R., Khasdeo, L., Misra, K.G. (2015). Resolving Kamthi-related problems in Gondwana	Central Ground Water Board (2012e). Aquifer Systems of Chhattisgarh. Central Ground Water Board Report, 73 pp. Accessed July 13, 2021	Approximated from Fig. 2 by Chowdari et al. (2017), Plates III and IV by Central Ground Water Board (2012e), and Fig. 1 by Dutta et al. (2015)

Aquifer system	Broader aquifer system	Reference A	Reference B	Reference C	Steps to delineate
		remote sensing data, South Rewa Gondwana Basin, India. Journal of Earth System Science, 126, 1-27.	stratigraphy of peninsular India. Indian Journal of Geosciences, 69, 85-102.	from http://cgwb.gov.in/AQM/Chhattisgarh.pdf	
**Mahanadi Delta**	Utkal Coastal Plain	Kar, G., Sahoo, N., Kumar, A., Das, M., Roychoudhury, S., Chandra, D. (2007). Intergrated Technologies to enhance productivity of seasonal deep waterlogged areas. Water Technology Centre for Eastern Region Research Bulletin No. 40, 56 pp., Accessed July 14, 2021 from http://www.iiwm.res.in/pdf/Bulletin_40.pdf	Das, P. P. (2020, September). Saline Contamination Mahanadi Deltaic Aquifers: A Review. Proceedings of the Indian National Academy, 86, 1169-1176. Accessed July 17, 2021 from https://web.archive.org/web/20201213175659id_/https://insa.nic.in/writereaddata/UpLoadedFiles/PINSA/PINSA_2020_Art74.pdf	Sahu, J. K., Das, P. P., Sahoo, H. K., Mohapatra, P. P., Sahoo, S. (2018). Geospatial analysis and hydrogeochemical investigation of a part of southern Mahanadi delta, Odisha, India. Himal. Geol., 39, 92-100.	Approximated from Fig. 1 by Das (2020), and Fig. 1 by Sahu et al. (2018). Location of broader system detailed in section 5.0 and Fig. 4 by Kar et al. (2007).
**Mahidasht Plain**	-	Asadi, F., Soltanian, M., Mohmmadi, A., Setareh, P., Khezri, S. M. (2015). Geographical Zoning Physicochemical Quality Change in Groundwater Catchment Gharehsou Ten-Year Period 2003-2012. Biosciences Biotechnology Research Asia, 12, 507-515.	-	-	Approximated from Fig. 1 and study area descriptions (in section entitled "Findings") by Asadi et al. (2015)
**Main Basin****	Puget Sound Lowland	Vaccaro, J. J., Hansen, A. J., Jones, M. A. (1998). Hydrogeologic framework of the Puget Sound aquifer system, Washington and British Columbia. U.S. Geological Survey Professional Paper 1424-D, 87 pp. Accessed March 11, 2021 from https://pubs.usgs.gov/pp/1424d/report.pdf	Washington Department of Ecology (2010). Puget Sound Groundwater Toxics Loading Analysis: Direct Discharge Pathway. Publication No. 10-03-122, 26 pp. Accessed March 12, 2021 from https://apps.ecology.wa.gov/publications/documents/1003122.pdf	-	Broader Puget Sound Lowland approximated from Vaccaro et al. (1998). Sub-basin outline approximated from Fig. 2 of Washington Department of Ecology (2010).

Aquifer system	Broader aquifer system	Reference A	Reference B	Reference C	Steps to delineate
**Malad Valley**	-	Graham, W.G., Campbell, L.J. (1981). Groundwater resources of Idaho. Idaho Department of Water Resources Report, 61 pp. Accessed March 23, 2021 from https://idwr.idaho.gov/files/publications/198108-MISC-GW-Resources-ID.pdf	-	-	Approximated from Plate 1 of Graham and Campbell (1981).
**Malahat Basin**	-	Hosseini, S. M., Parizi, E., Ataie-Ashtiani, B., Simmons, C. T. (2019). Assessment of sustainable groundwater resources management using integrated environmental index: Case studies across Iran. Science of the Total Environment, 676, 792-810.	-	-	Approximated from Fig. 6 by Hosseini et al. (2019)
**Malayer Aquifer**	-	Hosseini, M., Saremi, A. (2018). Assessment and estimating groundwater vulnerability to pollution using a modified DRASTIC and GODS models (case study: Malayer Plain of Iran). Civil Engineering Journal, 4, 433-442.	-	-	Approximated from Fig. 1 by Hosseini and Saremi (2018)
**Malekan Aquifer**	Miandoab Plain	Azizi, F., Moghaddam, A. A., Nazemi, A., Gorgij, A. D. (2019). Introducing a novel method in evaluation of groundwater hydrochemical characteristics, GWQI SI index: case study—Malekan Aquifer, Northwest of Iran. Arabian Journal of Geosciences, 12, 1-15.	Norouzi, H., Moghaddam, A. A. (2020). Groundwater quality assessment using random forest method based on groundwater quality indices (case study: Miandoab plain aquifer, NW of Iran). Arabian Journal of Geosciences, 13, 1-13.	-	Approximated from Fig. 1 by Azizi et al. (2019) and Fig. 1 by Norouzi and Moghaddam (2020)

Aquifer system	Broader aquifer system	Reference A	Reference B	Reference C	Steps to delineate
**Mal-Khalife Basin**	-	Hosseini, S. M., Parizi, E., Ataie-Ashtiani, B., Simmons, C. T. (2019). Assessment of sustainable groundwater resources management using integrated environmental index: Case studies across Iran. Science of the Total Environment, 676, 792-810.	-	-	Approximated from Fig. 6 by Hosseini et al. (2019)
**Malm Valangnian Aquifer**	-	Nitcheva, O. (2018). Hydrology models approach to estimation of the groundwater recharge: case study in the Bulgarian Danube watershed. Environmental Earth Sciences, 77, 1-12.	Pulido-Bosch, A., Lopez-Chicano, M., Calaforra, J.M., Cal Vache, M.L., Machkova, M., Dimitrov, D., Velikov, B., Pentchev, P. (1999). Groundwater problems in the karstic aquifers of the Dobrich region, northeastern Bulgaria. Hydrological Sciences Journal, 44, 913-927.	-	Approximated from Fig. 3 by Nitcheva (2018) and Fig. 3 by Pulido-Bosch et al. (1999) and local drainage and physiographic patterns
**Malwa Plateau Gneiss Complex**	Malwa Plateau	Bhuiyan, C., Singh, R. P., Flügel, W. A. (2009). Modelling of ground water recharge-potential in the hard-rock Aravalli terrain, India: a GIS approach. Environmental Earth Sciences, 59(4), 929-938.	Central Ground Water Board (2012d). Aquifer Systems of India. Central Ground Water Board Report, 103 pp. Accessed July 12, 2021 from http://cgwb.gov.in/AQM/India.pdf	-	Approximated from Plate III by Central Ground Water Board (2012d) and Fig. 1 by Bhuiyan et al. (2009)
**Mancha Occidental Aquifer**	Upper Guadiana Basin	Martínez-Santos, P., Castaño-Castaño, S., Hernández-Espriú, A. (2018). Revisiting groundwater overdraft based on the experience of the Mancha Occidental Aquifer, Spain. Hydrogeology Journal, 26, 1083-1097.	-	-	Approximated from Fig. 1 by Martínez-Santos et al. (2018)

Aquifer system	Broader aquifer system	Reference A	Reference B	Reference C	Steps to delineate
**Mancha Oriental Aquifer System**	-	Sanz, D., Castaño, S., Cassiraga, E., Sahuquillo, A., Gómez-Alday, J. J., Peña, S., Calera, A. (2011). Modeling aquifer–river interactions under the influence of groundwater abstraction in the Mancha Oriental System (SE Spain). Hydrogeology Journal, 19, 475-487.	-	-	Approximated from Fig. 1 by Sanz et al. (2011)
**Manetin Basin**	-	Opluštil, S. (2005). The effect of paleotopography, tectonics and sediment supply on quality of coal seams in continental basins of central and western Bohemia (Westphalian), Czech Republic. International Journal of Coal Geology, 64, 173-203.	Opluštil, S., Lojka, R., Pšenika, J. (2013). Late Variscan continental basins in western Bohemia: tectono-sedimentary, climate and biotic archives. Schriftreihe Dtsch Ges Geowiss, 82, 179-201.	-	Approximated from Fig. 1c by Opluštil (2005), Fig. 1 by Opluštil et al. (2013)
**Mangaluru Coastal Aquifer System**	-	Kiran, D.A., Ramaraju, H.K. (2020). The study of sea water intrusion using chemical Indicators in the Coastal Region of Mangaluru. 52nd Annual Convention of Indian Water Works Association (IWWA), 10-12th Jan 2020, NIT Patna, Bihar, 10 pp.	-	-	Approximated from Fig. 1 and descriptions within Kiran and Ramaraju (2020)
**Mantinea Flats and Carlton Plain**	-	Raper, P., George, R., Schoknecht, N. (2015). Preliminary soil and groundwater assessment of the Mantinea Development area, East Kimberley, Western Australia. Western Australian Agriculture Authority resource management technical	-	-	Approximated from Fig. 1.1 by Raper et al. (2015)

Aquifer system	Broader aquifer system	Reference A	Reference B	Reference C	Steps to delineate
		report 389, 48 pp. Accessed May 8, 2023 via https://www.agric.wa.gov.au/sites/gateway/files/Preliminary%20soil%20and%20groundwater%20assessment%20of%20the%20Mantinea%20Development%20area%2C%20East%20Kimberley%20-%20RMTR%20389%20%28PDF%204.2MB%29.pdf			
**Manujan Basin**	-	Hosseini, S. M., Parizi, E., Ataie-Ashtiani, B., Simmons, C. T. (2019). Assessment of sustainable groundwater resources management using integrated environmental index: Case studies across Iran. Science of the Total Environment, 676, 792-810.	-	-	Approximated from Fig. 6 by Hosseini et al. (2019)
**Maragheh-Bonab Plain**	-	Sadeghfam, S., Hassanzadeh, Y., Nadiri, A. A., Khatibi, R. (2016). Mapping groundwater potential field using catastrophe fuzzy membership functions and Jenks optimization method: a case study of Maragheh-Bonab plain, Iran. Environmental Earth Sciences, 75, 545.	Fijani, E., Nadiri, A. A., Moghaddam, A. A., Tsai, F. T. C., Dixon, B. (2013). Optimization of DRASTIC method by supervised committee machine artificial intelligence to assess groundwater vulnerability for Maragheh–Bonab plain aquifer, Iran. Journal of hydrology, 503, 89-100.	Fijani, E., Moghaddam, A. A., Tsai, F. T. C., tayfur, G. (2017). Analysis and assessment of hydrochemical characteristics of Maragheh-Bonab plain aquifer, northwest of Iran. Water Resources Management, 31, 765-780.	Approximated from Sadeghfam et al. (2016), from Fig. 1 by Fijani et al. (2016) and Fig. 1 by Fijani et al. (2017)
**Marana-Casinca Plain**	-	Erostate, M., Huneau, F., Garel, E., Lehmann, M.F., Kuhn, T., Aquilina, L., Vergnaud-Ayraud, V., Labasque, T., Santoni, S., Robert, S. Provitolo, D.	-	-	Approximated from Fig. 1 by Erostate et al. (2018)

Aquifer system	Broader aquifer system	Reference A	Reference B	Reference C	Steps to delineate
		(2018). Delayed nitrate dispersion within a coastal aquifer provides constraints on land-use evolution and nitrate contamination in the past. Science of the Total Environment, 644, 928-940.			
**Marand Plain**	-	Rostami, A. A., Isazadeh, M., Shahabi, M., Nozari, H. (2019). Evaluation of geostatistical techniques and their hybrid in modelling of groundwater quality index in the Marand Plain in Iran. Environmental Science and Pollution Research, 26, 34993-35009.	-	-	Approximated from Fig. 1 by Rostami et al. (2019)
**Marbella-Estepona Coastal Aquifer**	-	Argamasilla Ruiz, M., Andreo-Navarro, B. (2015). Resultados preliminares de la investigación hidrogeológica del acuífero aluvial del río Guadaiza (Marbella, España).	-	-	Approximated from Fig. 1 by Argamasilla Ruiz and Andreo-Navarro (2015)
**Maricopa-Stanfield Basin**	-	Flora, S., Davis, T. (2009). Hydrologic Map Series (HMS), Water Level Change Map Series (WLCMS), and Basin Sweep Assessment Report ADWR Basins and Sub-Basins. Arizona Department of Water Resources Hydrology Division Field Services Section https://new.azwater.gov/sites/default/files/HMSWLCMSBasinSweepAssessmentReport2009.pdf	-	-	Approximated from Fig. 1 of Flora and Davis (2009). Hydrologic Map Series (HMS), Water Level Change Map Series (WLCMS), and Basin Sweep Assessment Report ADWR Basins and Sub-Basins. Arizona Department of Water Resources Hydrology Division Field Services Section https://new.azwater.gov/sites/default/files/HMSWLCMSBasinSweepAssessmentReport2009.pdf

Aquifer system	Broader aquifer system	Reference A	Reference B	Reference C	Steps to delineate
**Marsh Creek**	Eastern Snake River Plain	Graham, W.G., Campbell, L.J. (1981). Groundwater resources of Idaho. Idaho Department of Water Resources Report, 61 pp. Accessed March 23, 2021 from https://idwr.idaho.gov/files/publications/198108-MISC-GW-Resources-ID.pdf	-	-	Approximated from Plate 1 of Graham and Campbell (1981).
**Marsh Valley**	Central Snake River Plain	Graham, W.G., Campbell, L.J. (1981). Groundwater resources of Idaho. Idaho Department of Water Resources Report, 61 pp. Accessed March 23, 2021 from https://idwr.idaho.gov/files/publications/198108-MISC-GW-Resources-ID.pdf	-	-	Approximated from Plate 1 of Graham and Campbell (1981).
**Marvast Basin**	-	Hosseini, S. M., Parizi, E., Ataie-Ashtiani, B., Simmons, C. T. (2019). Assessment of sustainable groundwater resources management using integrated environmental index: Case studies across Iran. Science of the Total Environment, 676, 792-810.	-	-	Approximated from Fig. 6 by Hosseini et al. (2019)
**Marvdasht and Kharameh Plains**	-	Azma, A., Narreie, E., Shojaaddini, A., Kianfar, N., Kiyanfar, R., Seyed Alizadeh, S. M., Davarpanah, A. (2021). Statistical modeling for spatial groundwater potential map based on gis technique. Sustainability, 13, 3788.	Honarbakhsh, A., Tahmoures, M., Tashayo, B., Mousazadeh, M., Ingram, B., Ostovari, Y. (2019). GIS-based assessment of groundwater quality for drinking purpose in northern part of Fars province, Marvdasht. Journal of Water Supply:	-	Approximated from Fig. 1 by Azma et al. (2021) and Fig. 1 by Honarbakhsh et al. (2019)

Aquifer system	Broader aquifer system	Reference A	Reference B	Reference C	Steps to delineate
			Research and Technology-Aqua, 68, 187-196.		
**Marveshkan Basin**	-	Hosseini, S. M., Parizi, E., Ataie-Ashtiani, B., Simmons, C. T. (2019). Assessment of sustainable groundwater resources management using integrated environmental index: Case studies across Iran. Science of the Total Environment, 676, 792-810.	-	-	Approximated from Fig. 6 by Hosseini et al. (2019)
**Maryland Western Shores**	North Atlantic Coastal Plain	Vroblesky, D.A., Fleck, W.B. (1991). Hydrogeologic Framework of the Coastal Plain of Maryland, Delaware, and the District of Columbia. U.S. Geological Survey Professional Paper 1404-E, 52 pp. Accessed April 1, 2021 from https://pubs.usgs.gov/pp/1404e/report.pdf	-	-	Approximated from Fig. 1 of Vroblesky and Fleck (1991).
**Mason Valley**	-	Carroll, R.W., Pohll, G., McGraw, D., Garner, C., Knust, A., Boyle, D., Minor, T., Bassett, S. Pohlmann, K. (2010). Mason Valley Groundwater Model: Linking Surface Water and Groundwater in the Walker River Basin, Nevada 1. JAWRA Journal of the American Water Resources Association, 46(3), 554-573.	-	-	Approximated from Fig. 2 of Carroll et al. (2010); southern margins extended to include recorded drilled wells.
**Matanza-Riachuelo**	-	Armengol, S., Manzano, M., Ayora, C., Martínez, S. (2023). The origin of	-	-	Approximated from Fig. 1 by Armengol et al. (2023)

Aquifer system	Broader aquifer system	Reference A	Reference B	Reference C	Steps to delineate
**Aquifer System**		groundwater salinity in the Matanza-Riachuelo aquifer system, Argentina. Groundwater for Sustainable Development, 20, 100879.			
**Mazapil and Cedros Basin**	-	Herrera, E., Garfias, J. (2013). Characterizing a fractured aquifer in Mexico using geological attributes related to open-pit groundwater. Hydrogeology Journal, 21, 1323-1338.	CONAGUA (2020gz). Actualización de la disponibilidad media anual de agua en el acuífero Cedros (3218), estado de Zacatecas. 39 pp. Accessed April 25, 2022 via https://sigagis.conagua.gob.mx/gas1/Edos_Acuiferos_18/zacatecas/DR_3218.pdf	-	Approximatef from Fig. 1 by Herrera and Garfias (2013); western margin extended past the city of Cedros on the basis of groundwater well construction data. Broader boundary based on the outline by CONAGUA (2020gz)
**Meacham Aquifer**	-	Maathuis, H. (2008). The quality of natural groundwaters in Saskatchewan. Saskatchewan Research Council Publication No. 12012-1E08, 86 pp. Accessed May 4, 2021 from https://www.wsask.ca/PageFiles/2978/The%20Quality%20of%20Natural%20Groundwaters%20in%20Saskatchewan,%20January%202008,%20Maathuis,%20H.,%20SRC%20pub.%20No.%2012012-1E08.pdf	-	-	Approximated from Fig. 6 of Maathuis (2008).
**Mehraban Plain**	-	Javanmard, Z., Asghari Moghaddam, A. (2016). Using Statistical and Hydrochemical Models for Qualitative Analysis of Groundwater Resources (Case Study: Mehraban plain, In East Azerbaijan).	-	-	Approximated from Fig. 1 by Javanmard and Asghari Moghaddam (2016)

Aquifer system	Broader aquifer system	Reference A	Reference B	Reference C	Steps to delineate
		Water and Soil Science, 26, 31-50.			
**Mehrgerd Semirom**	-	Mojiri, H., Halabian, A. (2019). Evaluation of the Effects of Temporal variables of Temperature, Precipitation and Water Harvesting on Groundwater Resources in Mehrgerd basin of Semirom. Journal of Watershed Management Research, 10, 238-249.	-	-	Approximated from Fig. 1 by Mojiri and Halabian (2019).
**Mendez - San Fernando Aquifer**	-	CONAGUA (2020cx). Actualización de la disponibilidad media anual de agua en el acuífero Mendez - San Fernando (2802), estado de Tamaulipas. 40 pp. Accessed March 8, 2022 via https://sigagis.conagua.gob.mx/gas1/Edos_Acuiferos_18/tamaulipas/DR_2802.pdf	-	-	Approximated from physiography, well completion data, and Fig. 1 by CONAGUA (2020cx)
**Merced Basin**	San Joaquin Basin of the Central Valley **	California Department of Water Resources (2015h). California's groundwater update 2013 - Chapter 8: San Joaquin River Hydrologic Region. California Department of Water Resources Report, 122 pp. Accessed April 28, 2022 via https://water.ca.gov/-/media/DWR-Website/Web-Pages/Programs/Groundwater-Management/Data-and-Tools/Files/Statewide-Reports/California-Groundwater-Update-2013/California-	-	-	Approximated from Fig. 8-2 by California Department of Water Resources (2015h)

Aquifer system	Broader aquifer system	Reference A	Reference B	Reference C	Steps to delineate
		Groundwater-Update-2013---Chapter-8---San-Joaquin-River.pdf			
**Mercury Valley**	-	Lopes, T.J., Evetts. D.M. (2005). Ground-Water pumpage and artificial recharge estimates for calendar year 2000 and average annual natural recharge and interbasin flow by hydrographic area, Nevada. Accessed April 25, 2022 via https://pubs.usgs.gov/sir/2004/5239/sir2004-5239.pdf	Harrill, J.R., Prudic, D.E. (1998). Aquifer systems in the Great Basin region of Nevada, Utah, and adjacent states; summary report. US Geological Survey Professional Paper 1409-A, 75 pp. Accessed April 26, 2022 via https://pubs.usgs.gov/pp/1409a/report.pdf	-	Approximated from Fig. 1 by Lopes and Evetts (2005), and Fig. 2 by Harrill and Prudic (1998). Well completion records, physiography and drainage were used to delinate areas.
**Mesilla Valley**	Rincon-Mesilla Valleys	Nickerson, E. L., Myers, R. G. (1993). Geohydrology of the Mesilla ground-water basin, Dona Ana County, New Mexico, and El Paso County, Texas (No. 92-4156). US Geological Survey, Water Resources Division; US Geological Survey Water-Resources Investigations Report 92-4156. Accessed February 17, 2021 from https://pubs.usgs.gov/wri/1992/4156/report.pdf	Hawley, J. W., Lozinsky, R. P. (1992). Hydrogeologic framework of the Mesjlla Basin in New Mexico and western Texas. New Mexico Bureau of Mines and Mineral Resources Open File Report 323, 95 pp. Accessed November 29, 2021 from https://geoinfo.nmt.edu/publications/openfile/downloads/300-399/323/ofr_323.pdfhttps://geoinfo.nmt.edu/publications/openfile/downloads/300-399/323/ofr_323.pdf	Robertson, A.J., Matherne, A.M., Pepin, J.D., Ritchie, A.B., Sweetkind, D.S., Teeple, A.P., Granados-Olivas, A., García-Vásquez, A.C., Carroll, K.C., Fuchs, E.H., Galanter, A.E. (2022). Mesilla/Conejos-Médanos Basin: US-Mexico Transboundary Water Resources. Water, 14, 134.	Approximated from map on page 10 of Nickerson and Myers (1993), Fig. 1 by Hawley and Lozinsky (1993), and Fig. 1 by Robertson et al. (2022)
**Mesilla-Conejos-Medanos Basin**	-	Robertson, A.J., Matherne, A.M., Pepin, J.D., Ritchie, A.B., Sweetkind, D.S., Teeple, A.P., Granados-Olivas, A., García-Vásquez, A.C., Carroll, K.C., Fuchs, E.H., Galanter, A.E. (2022). Mesilla/Conejos-Médanos Basin: US-Mexico	International Boundary and Water Commission (2011). Hydrogeological activities in the Conejos-Medanos/Mesilla Basin Aquifer, Chihuahua Phase I. International Boundary and Water Commissio Report, 111 pp. Accessed January	-	Approximated from Fig. 1 by Robertson et al. (2022) and Fig. 1.1 via International Boundary and Water Commission (2011)

Aquifer system	Broader aquifer system	Reference A	Reference B	Reference C	Steps to delineate
		Transboundary Water Resources. Water, 14, 134.	23, 2022 via https://www.ibwc.gov/Files/Web/Final_report_English_Mesilla_ConejosMedanos_Study%202011.pdf		
**Mexicali Valley**	-	Payne, B. R., Quijano, L., Latorre, D. C. (1979). Environmental isotopes in a study of the origin of salinity of groundwater in the Mexicali Valley. Journal of Hydrology, 41(3-4), 201-215.	Fuentes-Arreazola, M. A., Ramírez-Hernández, J., Vázquez-González, R. (2018). Hydrogeological properties estimation from groundwater level natural fluctuations analysis as a low-cost tool for the Mexicali Valley aquifer. Water, 10, 586.	-	Approximated from Fig. 1 of Payne et al. (1979). and Fig. 1 by Fuentes-Arreazola et al. (2018).
**Meymand Aquifer**	-	Mohammadzadeh-Habili, J., Soltani, M., Khalili, D. (2021b). Effect of reservoir geometry on functionality of recharge dams influenced by sedimentation: case study of the Meymand recharge dam. Arabian Journal of Geosciences, 14, 1-9.	-	-	Approximated from Fig. 1 by Mohammadzadeh-Habili et al. (2021b)
**Meymeh Plain**	-	Mahmoudzadeh, E., Rezaian, S., Ahmadi, A. (2013). Assessment of Meymeh Plain Aquifer Vulnerability in Esfahan Using Comparative Method AVI, GODS, DRASTIC. Journal of Environmental Studies, 39, 45-60.	-	-	Approximated from Fig. 1 by Mahmoudzadeh et al. (2013)
**Mezguita Oasis**	Middle Draa Basin	Szczucińska, A., Dłużewski, M., Kozłowski, R., Niedzielski, P. (2019). Hydrochemical diversity of a large alluvial aquifer in an arid zone (Draa river, S Morocco). Ecological	-	-	Approximated from Fig. 1 by Szczucińska et al. (2019)

Aquifer system	Broader aquifer system	Reference A	Reference B	Reference C	Steps to delineate
		Chemistry and Engineering S, 26, 81-100.			
**Mhamid Oasis**	Middle Draa Basin	Szczucińska, A., Dłużewski, M., Kozłowski, R., Niedzielski, P. (2019). Hydrochemical diversity of a large alluvial aquifer in an arid zone (Draa river, S Morocco). Ecological Chemistry and Engineering S, 26, 81-100.	-	-	Approximated from Fig. 1 by Szczucińska et al. (2019)
**Miahuatlan Aquifer**	-	CONAGUA (2021a). Acuíferos (nacional). Webpage accessed March 8, 2022 from http://sina.conagua.gob.mx/sina/tema.php?tema=acuiferos&ver=mapa&o=1&n=nacional	-	-	Approximated from aquifer boundaries defined by CONAGUA (2021a) web portal, physiography and drainage patterns, and well completion data.
**Miami Basin**	-	Hosseini, S. M., Parizi, E., Ataie-Ashtiani, B., Simmons, C. T. (2019). Assessment of sustainable groundwater resources management using integrated environmental index: Case studies across Iran. Science of the Total Environment, 676, 792-810.	-	-	Approximated from Fig. 6 by Hosseini et al. (2019)
**Miandarband Plain**	-	Zare, M., Koch, M. (2017). Computation of the irrigation water demand in the Miandarband plain, Iran, using FAO-56-and satellite-estimated crop coefficients. Interdisciplinary Research Review, 12, 15-25.	-	-	Approximated from Fig. 1 by Zare and Koch (2017)
**Mianrahan Aquifer**	-	Hafezparast, M. (2021). Monitoring groundwater	-	-	Approximated from Fig. 1 by Hafezparast et al. (2021)

Aquifer system	Broader aquifer system	Reference A	Reference B	Reference C	Steps to delineate
		level changes of Mianrahan aquifer with GRACE satellite data. Iranian Journal of Irrigation & Drainage, 2, 428-443.			
**Michigan Basin**	-	Olcott, P.G. (1992). Groundwater Atlas of the United States: Segment 9 Iowa, Michigan, Minnesota, Wisconsin. U.S. Geological Survey Hydrologic Atlas 730-J, 33 pp. Accessed April 12, 2021 from https://pubs.usgs.gov/ha/730j/report.pdf	Westjohn, D.B., Weaver, T.L. (1998). Hydrogeologic framework of the Michigan Basin regional aquifer system. US Geological Survey Professional Paper 1418, 55 pp. Accessed November 29, 2021 from https://pubs.usgs.gov/pp/1418/report.pdf	-	Approximated from Figs. 50-64 by Olcott (1992) and Fig. 1 by Westjohn and Weaver (1998)
**Middle Aconcagua Basin**	Aconcagua Basin	Direccion General de Aguas (2009). Diagnóstico y Clasificación de Sectores Acuíferos, Volumen No. 2. Gobierno de Chile Ministerio de Obras Publicas Report, 384 pp. Accessed May 16, 2021 from https://snia.mop.gob.cl/sad/CQA5168v2.pdf	-	-	Approximated from Table 1.8 on page 40 by Direccion General de Aguas (2009). Middle and upper portions of the aquifer system delineated near Panquehue; divide between lower and middle portions of aquifer system approximated near Calera.
**Middle Chao Phraya Basin**	-	Putthividhya, A., Laonamsai, J. (2017). Hydrological assessment using stable isotope fingerprinting technique in the Upper Chao Phraya river basin. Lowland Technology International, 19, 27-40.	Foster, S. (2002). Thailand: strengthening capacity in groundwater resources management. World Bank Case Profile Collection, 4 pp. Accessed November 3, 2021 from https://documents1.worldbank.org/curated/en/521371468308952444/pdf/388010PAPER0TH1WMATE1CP10101PUBLIC1.pdf	-	Approximated from Fig. 1 by Putthividhya and Laonamsai (2017), and descriptions of area by Foster (2002)
**Middle Copiapo Valley**	Copiapo Valley	Donoso, G., Lictevout, E., Rinaudo, J. D. (2020). Groundwater management lessons from Chile. In:	Direccion General de Aguas (2015). Plan Nacional de Estudios Acuíferos. Report number 381, Santiago,	-	Approximated from Fig. 25.2 by Donoso et al. (2020) and Fig. 2a by Direccion General de Aguas (2015).

Aquifer system	Broader aquifer system	Reference A	Reference B	Reference C	Steps to delineate
		Sustainable Groundwater Management. Springer, Cham., pp. 481-509. Accessed May 12, 2021 from https://link.springer.com/chapter/10.1007/978-3-030-32766-8_25	Chile, 44 pp. Accessed May 16, 2021 from http://bibliotecadigital.ciren.cl/bitstream/handle/123456789/32415/DGA_2015_actualizacion_plan_nacional_acuiferos.pdf?sequence=1&isAllowed=y		
**Middle Gangetic Plain**	Indo-Gangetic Plain	Bonsor, H. C., MacDonald, A. M., Ahmed, K. M., Burgess, W. G., Basharat, M., Calow, R. C., Dixit, A., Foster, S.S.D., Gopal, K., Lapworth, D.J., Moench, M., Mukherjee, A., Rao, M.S., Shamsudduha, M., Smith, L., Taylor, R.G., Tucker, J., van Steenbergen, F., Yadav, S.K., Zahid, A. (2017). Hydrogeological typologies of the Indo-Gangetic basin alluvial aquifer, South Asia. Hydrogeology Journal, 25, 1377-1406.	Singh, J., Erenstein, O., Thorpe, W. R., Varma, A. (2007). Crop-livestock interactions and livelihoods in the Gangetic Plains of Uttar Pradesh, India. International Livestock Research Institute.	Mukherjee, A., Saha, D., Harvey, C. F., Taylor, R. G., Ahmed, K. M., Bhanja, S. N. (2015). Groundwater systems of the Indian sub-continent. Journal of Hydrology: Regional Studies, 4, 1-14.	Approximated from Fig. 3 by Mukherjee et al. (2015), Fig. 3 by Bonsor et al. (2020), and Fig. 1 by Singh et al. (2007)
**Middle Tata Alluvial Basin**	-	Echogdali, F.Z., Boutaleb, S., El Ayady, H., Aadraoui, M., Abdelrahman, K., Bendarma, A., Ikirri, M., Abu-Alam, T., Id-Belqas, M., Abioui, M. (2023). Characterization and productivity of alluvial aquifers in sustainability oasis areas: a case study of the Tata Watershed (Southeast Morocco). Applied Sciences, 13, 5473.	-	-	Approximated from Fig. 3 in Echogdali et al. (2023)
**Middle Yellowstone Area**	-	Madison, J.P., LaFave, J.I., Patton, T.W., Smith, L.N., Olson, J.N. (2014).	-	-	Approximated from Fig. 10 of Madison et al. (2014).

Aquifer system	Broader aquifer system	Reference A	Reference B	Reference C	Steps to delineate
		Groundwater resources of the Middle Yellowstone River area: Treasure and Yellowstone counties, Montana Part A* - Descriptive Overview and Water-Quality Data: Montana Bureau of Mines and Geology Montana Ground-Water Assessment Atlas 3-A, 82 pp. Accessed March 29, 2021 from http://mbmg.mtech.edu/pdf-publications/gwaa_3.pdf			
**Middle-Upper Devonian Aquifer System**	Baltic Artesian Basin	Virbulis, J., Bethers, U., Saks, T., Sennikovs, J., Timuhins, A. (2013). Hydrogeological model of the Baltic Artesian Basin. Hydrogeology Journal, 21, 845-862.	Mokrik, R., Mazeika, J., Baublyt, A., Martma, T. (2009). The groundwater age in the Middle-Upper Devonian aquifer system, Lithuania. Hydrogeology Journal, 17, 871-889.	Lithuanian Geological Survey and Latvian Environment, Geology and Meteorology Centre (2019). Cross-border groundwater body characterization and status assessment. B-Solutions initiative report, 32 pp.	Broader Baltic Artesian Basin approximated from Fig. 1 by Virbulis et al. (2013). Sub-area approximated from drainage patterns and regional geology
**Mid-Waitaki Valley**	-	Scott, L., Hanson, C., Cressy, C. (2012). Groundwater quality investigation of the mid-Waitaki valley. Environment Canterbury Regional Council Kaunihera Taiao ki Waitaha Report, 77 pp. Accessed January 25, 2022 from http://citeseerx.ist.psu.edu/viewdoc/download?doi=10.1.1.799.6506&rep=rep1&type=pdf	-	-	Approximated from Fig. 1 by Scott et al. (2012)
**Milford-Blackrock Subarea**	Escalante Valley	Sandberg, G. W. (1966). Ground-water resources of selected basins in southwestern Utah. US	Fix, P. F., Nelson, W. B., Lofgren, B. E., Butler, R. G. (1950). Ground water in the Escalante Valley,	-	Approximated from Fig. 1 of Fix et al. (1950) with subareas ("Districts") approximated from

Aquifer system	Broader aquifer system	Reference A	Reference B	Reference C	Steps to delineate
		Geological Survey Open File Report. 66 pp. Accessed March 7, 2021 from https://cicwcd.org/wp-content/uploads/2018/09/1966-tech-pub-13-ground-water-resources-of-selected-basins-in-southwestern-utah-by-g-w-sandburg-usgs.pdf	Beaver, Iron, and Washington Counties, Utah Technical Publication 6. 102 pp. Accessed March 7, 2021 from https://waterrights.utah.gov/docSys/v920/w920/w9200085.pdf		the map on page 49 of Sandberg (1966).
**Milk River**	-	Pétré, M.A., Rivera, A., Lefebvre, R., Hendry, M. J., Folnagy, A.J. (2016). A unified hydrogeological conceptual model of the Milk River transboundary aquifer, traversing Alberta (Canada) and Montana (USA). Hydrogeology Journal, 24, 1847-1871.	Phillips, F.M., Bentley, H.W., Davis, S.N., Elmore, D., Swanick, G.B. (1986). Chlorine 36 dating of very old groundwater: 2. Milk River aquifer, Alberta, Canada. Water Resources Research, 22, 2003-2016.	-	Approximated from Fig. 2 of Pétré et al. (2016) and Figs. 1 and 2 by Phillips et al. (1986)
**Mill Creek Aquifer**	-	Graham, W.G., Campbell, L.J. (1981). Groundwater resources of Idaho. Idaho Department of Water Resources Report, 61 pp. Accessed March 23, 2021 from https://idwr.idaho.gov/files/publications/198108-MISC-GW-Resources-ID.pdf	-	-	Approximated from Plate 1 of Graham and Campbell (1981).
**Mimbres Basin**	-	Hanson, R.T., McLean, J.S., Miller, R.S. (1994). Hydrogeologic framework and preliminary simulation of ground-water flow in the Mimbres Basin, Southwestern New Mexico: U.S. Geological Survey, Water-Resources Investigations Report 94-4011, 118 pp. Accessed	Finch, S. T., Mccoy, A., Melis, E. (2008). Geologic Controls on Groundwater Flow in the Mimbres Basin, South Western New Mexico. In New Mexico Geological Society Guide Book, 59th Field Conference, pp. 189-198. Accessed March 27, 2021 from https://nmgs.nmt.edu/publi	White, W. N. (1931). Preliminary report on the ground-water supply of Mimbres Valley, New Mexico. US Geological Survey Water Supply Paper 637, 23 pp. Accessed November 7, 2021 from https://pubs.usgs.gov/wsp/0637B/report.pdf	Approximated from Fig. 1 of Hansen et al. (1994), Fig. 1 of Finch et al. (2008) and Plate 1 by White (1931)

Aquifer system	Broader aquifer system	Reference A	Reference B	Reference C	Steps to delineate
		March 27, 2021 from https://pubs.usgs.gov/wri/1994/4011/report.pdf	cations/guidebooks/downloads/59/59_p0189_p0198.pdf		
**Mimosa Syncline and Eastern Surat Basin**	Great Artesian Basin	Ransley, T.R., Radke, B.M., Feitz, A.J., Kellett, J.R., Owens, R., Bell, J. and Stewart, G. (2015). Hydrogeological Atlas of the Great Artesian Basin, Geoscience Australia. Canberra, 141 pp. Accessed July 19, 2021 from http://www.ga.gov.au/scientific-topics/water/groundwater/gab	Ransley, T.R., Smerdon, B.D. (2012). Hydrostratigraphy, hydrogeology and system conceptualisation of the Great Artesian Basin. A technical report to the Australian Government from the CSIRO Great Artesian Basin Water Resource Assessment, 324 pp. Accessed July 20, 2021 from https://publications.csiro.au/rpr/download?pid=csiro:EP132693&dsid=DS5	Smerdon, B.D., Ramsley, T.R. (2012). Water Resource Assessment for the Surat Region. A technical report to the Australian Government from the CSIRO Great Artesian Basin Water Resource Assessment, 142 pp. Accessed July 20, 2021 from https://publications.csiro.au/rpr/download?pid=csiro:EP132644&dsid=DS4	Approximated from Map 1 on page 9 of report by Ransley et al. (2015), and Fig. 2.3 by Ransley and Smerdon (2012). Subarea within Surat Basin delineated on the basis of physiographic features (e.g., major rivers), elevations of the base of the Great Artesian Basin (Fig. 5.2 by Smerdon and Ransley (2012b)) and topography.
**Minab Plain**	-	Torkamanitombeki, H., Rahnamarad, J., Saadatkhah, N. (2018). Groundwater chemical indices changed due to water-level decline, Minab Plain, Iran. Environmental Earth Sciences, 77, 1-10.	-	-	Approximated from Fig. 1 by Torkamanitombeki et al. (2018)
**Minqin Basin**	Shiyang Basin	Zhu, G. F., Li, Z. Z., Su, Y. H., Ma, J. Z., Zhang, Y. Y. (2007). Hydrogeochemical and isotope evidence of groundwater evolution and recharge in Minqin Basin, Northwest China. Journal of Hydrology, 333, 239-251.	-	-	Approximated from Fig. 1 by Zhu et al. (2007)
**Mioplioquatenario de Elvas-Campo Maior and Vegas Baja Aquifers**	-	Story, J., Lopez-Gunn, E. (2010). Comparing conflict in transboundary aquifer management: some insights from a comparative study between Spain and	-	-	Approximated from Fig. 3 by Story and Lopez-Gunn (2010) and local well completion data.

Aquifer system	Broader aquifer system	Reference A	Reference B	Reference C	Steps to delineate
		Australia. UNESCO-IAH-UNEP Conference, Paris, 7 pp. Accessed April 25, 2023 via http://www.siagua.org/sites/default/files/documentos/documentos/espaa_australia.pdf			
**Mission Valley and Irvine Flats**	Flathead Valley	LaFave, J. I. (2004). Potentiometric Surface Map of the Southern Part of the Flathead Lake Area, Lake, Missoula, Sanders Counties, Montana. Montana Ground-Water Assessment Atlas No. 2, Part B, Map 4. Montana Bureau of Mines and Geology, A Department of Montana Tech of The University of Montana, Butte, MT.	Smith, L.N. (2004). Hydrogeologic framework of the southern part of the Flathead Lake Area, Flathead, Lake, Missoula, and Sanders counties, Montana: Montana Bureau of Mines and Geology Montana Ground-Water Assessment Atlas 2-B-10, 1 sheet, scale 1:300,000. Accessed March 2, 2021 from http://mbmggwic.mtech.edu/gwcpmaps/gwaa02map10untiled.pdf	-	Approximated from map by Smith (2004) and LaFave (2004).
**Missionary Plains Syncline and Mereenie Anticline**	Amadeus Basin	Lloyd, J. W., Jacobson, G. (1987). The hydrogeology of the Amadeus Basin, central Australia. Journal of Hydrology, 93, 1-24.	Brown, D. M., Lloyd, J. W., Jacobson, G. (1990). Hydrogeological model for Amadeus Basin aquifers, central Australia. Australian Journal of Earth Sciences, 37, 215-226.	Cresswell, R. G., Jacobson, G., Wischusen, J., Fifield, L. K. (1999). Ancient groundwaters in the Amadeus Basin, Central Australia: evidence from the radio-isotope 36Cl. Journal of Hydrology, 223, 212-220.	Approximated from Fig. 1 by Lloyd and Jacobson (1987), Fig. 1 by Brown et al. (1990), and Fig. 1 by Cresswell et al. (1999)
**Mississippi Delta**	Gulf Coast Regional Aquifer System	Weiss, J. S. (1990). Geohydrologic units of the coastal lowlands aquifer system, south-central United States. U.S. Geological Survey Regional aquifer-systems analysis. 42 pp. Accessed March 30,	Torak, L.J., Painter, J.A. (2019). Geostatistical estimation of the bottom altitude and thickness of the Mississippi River Valley alluvial aquifer: U.S. Geological Survey Scientific Investigations Map 3426, 2	-	Outer margin of Gulf Coast Regional Aquifer System approximated from Fig. 2 of Weiss (1990). Delta subarea delineated southeast of Lafayette Area, south of deep wells (from completion reports) near Baton Rouge, and south of Gonzales-New Orleans

Aquifer system	Broader aquifer system	Reference A	Reference B	Reference C	Steps to delineate
		2021 from https://pubs.usgs.gov/pp/1416c/report.pdf	sheets, Accessed March 31, 2021 from https://pubs.er.usgs.gov/publication/sim3426.		aquifer area. Torak and Painter (2019) provide helpful alluvial thickness data for the delta region and also the Mississippi River Valley Alluvial Aquifer extending north of this delineated subregion (connecting to the Central Mississippi Embayment); alluvium in this delineated area can be up to 120 m thick (Torak and Painter, 2019).
**Mississippian-Silurian-Devonian Carbonates**	Northern Midwest Aquifer System	Olcott, P.G. (1992). Groundwater Atlas of the United States: Segment 9 Iowa, Michigan, Minnesota, Wisconsin. U.S. Geological Survey Hydrologic Atlas 730-J, 33 pp. Accessed April 12, 2021 from https://pubs.usgs.gov/ha/730j/report.pdf	Young, H.L. (1992). Hydrogeology of the Cambrian-Ordovician Aquifer System in the Northern Midwest, United States. U.S. Geological Survey Professional Paper 1405-B, 108 pp. Accessed April 12, 2021 from https://pubs.usgs.gov/pp/1405b/report.pdf	-	Broader aquifer system title and its location approximated from Fig. 1 of Young (1992). Subarea location approximated from Figs. 10 and 11 of Olcott (1992).
**Mitchell Plateau**	Interior Low Plateaus	Miller, J.A. (1995). Ground Water Atlas of the United States: Segment 10, Illinois, Indiana, Kentucky, Ohio, Tennessee. U.S. Geological Survey Hydrologic Atlas 730-K, 30 pp. Accessed April 11, 2021 from https://pubs.usgs.gov/ha/730k/report.pdf	Taylor, C.J., Nelson Jr., H.L. (2008). A compilation of provisional karst geospatial data for the Interior Low Plateaus physiographic region, central United States: U.S. Geological Survey Data Series 339, 26 pp. Accessed April 11, 2021 from https://pubs.usgs.gov/ds/339/pdf/ds339_web.pdf	Florea, L. J., Hasenmueller, N. R., Branam, T. D., Frushour, S. S., Powell, R. L. (2018). Karst geology and hydrogeology of the Mitchell Plateau of south-central Indiana. In: Field Guide 51, Published by The Geological Society of America, pp. 95-112.	Broader Interior Low Plateaus outline approximated for Tennessee, Illinois, Kentucky, Indiana from Fig. 78 of Miller (1990) and Fig. 1 of Taylor and Nelson (2008). Mitchell Plain subarea approximated from Fig. 1 of Florea et al. (2008).
**MiyanAb Shushtar Basin**	Dezful-Andimeshk Plain	Hosseini, S. M., Parizi, E., Ataie-Ashtiani, B., Simmons, C. T. (2019). Assessment of sustainable groundwater resources management using integrated environmental	-	-	Approximated from Fig. 6 by Hosseini et al. (2019)

Aquifer system	Broader aquifer system	Reference A	Reference B	Reference C	Steps to delineate
		index: Case studies across Iran. Science of the Total Environment, 676, 792-810.			
**Mobarak Abad-Baros Basin**	-	Hosseini, S. M., Parizi, E., Ataie-Ashtiani, B., Simmons, C. T. (2019). Assessment of sustainable groundwater resources management using integrated environmental index: Case studies across Iran. Science of the Total Environment, 676, 792-810.	-	-	Approximated from Fig. 6 by Hosseini et al. (2019)
**Mobarakiyeh Basin**	Tehran Plain	Hosseini, S. M., Parizi, E., Ataie-Ashtiani, B., Simmons, C. T. (2019). Assessment of sustainable groundwater resources management using integrated environmental index: Case studies across Iran. Science of the Total Environment, 676, 792-810.	Motagh, M., Walter, T. R., Sharifi, M. A., Fielding, E., Schenk, A., Anderssohn, J., Zschau, J. (2008). Land subsidence in Iran caused by widespread water reservoir overexploitation. Geophysical Research Letters, 35, L16403.	-	Approximated from Fig. 6 by Hosseini et al. (2019). Broader Tehran Plain approximated from Fig. 1a by Motagh et al. (2008)
**Modesto Basin**	San Joaquin Basin of the Central Valley **	California Department of Water Resources (2015h). California's groundwater update 2013 - Chapter 8: San Joaquin River Hydrologic Region. California Department of Water Resources Report, 122 pp. Accessed April 28, 2022 via https://water.ca.gov/-/media/DWR-Website/Web-Pages/Programs/Groundwater-Management/Data-and-Tools/Files/Statewide-	-	-	Approximated from Fig. 8-2 by California Department of Water Resources (2015h)

Aquifer system	Broader aquifer system	Reference A	Reference B	Reference C	Steps to delineate
		Reports/California-Groundwater-Update-2013/California-Groundwater-Update-2013---Chapter-8---San-Joaquin-River.pdf			
**Mohammad Abad Area**	Lower Zayandeh-Rud Basin	Hosseini, S. M., Parizi, E., Ataie-Ashtiani, B., Simmons, C. T. (2019). Assessment of sustainable groundwater resources management using integrated environmental index: Case studies across Iran. Science of the Total Environment, 676, 792-810.	Pazand, K., Javanshir, A. R. (2014). Geochemistry and water quality assessment of groundwater around Mohammad Abad Area, Bam District, SE Iran. Water Quality, Exposure and Health, 6, 225-231.	Salemi, H.R., Mamanpoush, A.R., Miranzadeh, M., Akbari, M., Torabi, M., Toomanian, N., Murray-Rust, H., Droogers, P., Sally, H., Gieske, A. (2000). Water management for sustainable irrigated agriculture in the Zayandeh Rud Basin, Esfahan Province, Iran Report by Iranian Agricultural Engineering Research Institute, Esfahan Agricultural Research Center, and the International Water Management Institute, Research Report Number 1	Approximated from Fig. 6 by Hosseini et al. (2019), Fig. 1 by Madani and Mariño (2009), and Fig. 1 by Salemi et al. (2000)
**Mojave Basin**	-	Stamos, C. L., Christensen, A. H., Langenheim, V. (2017). Preliminary hydrogeologic assessment near the boundary of the Antelope Valley and El Mirage Valley groundwater basins, California. U.S. Geological Survey Scientific Investigations Report 2017–5065, 56 pp. Accessed March 20, 2021 from https://pubs.usgs.gov/sir/2017/5065/sir20175065.pdf	-	-	Approximated from Fig. 7 of Stamos et al. (2017)

Aquifer system	Broader aquifer system	Reference A	Reference B	Reference C	Steps to delineate
**Mokhtaran Basin**	-	Hosseini, S. M., Parizi, E., Ataie-Ashtiani, B., Simmons, C. T. (2019). Assessment of sustainable groundwater resources management using integrated environmental index: Case studies across Iran. Science of the Total Environment, 676, 792-810.	-	-	Approximated from Fig. 6 by Hosseini et al. (2019)
**Mond Basin**	-	Hosseini, S. M., Parizi, E., Ataie-Ashtiani, B., Simmons, C. T. (2019). Assessment of sustainable groundwater resources management using integrated environmental index: Case studies across Iran. Science of the Total Environment, 676, 792-810.	-	-	Approximated from Fig. 6 by Hosseini et al. (2019)
**Mons Sedimentary Basin**	-	Goderniaux, P., Orban, P., Rorive, A., Brouyère, S., Dassargues, A. (2023). Study of historical groundwater level changes in two Belgian chalk aquifers in the context of climate change impacts. Geological Society, London, Special Publications, 517, SP517-2020.	-	-	Approximated from Fig. 1 by Goderniaux et al. (2021)
**Montecito Basin**	-	Muir, K.S. (1968). Ground-water reconnaissance of the Santa Barbara-Montecito area, Santa Barbara County, California: U.S. Geological Survey Water Supply Paper 1859–A, 28 pp. Accessed March 20,	-	-	Approximated from Plate 1 of Muir (1968)

Aquifer system	Broader aquifer system	Reference A	Reference B	Reference C	Steps to delineate
		2021 from https://pubs.usgs.gov/wsp/1859a/report.pdf			
**Monterrey Valley**	-	Torres-Martínez, J. A., Mora, A., Knappett, P. S., Ornelas-Soto, N., Mahlknecht, J. (2020). Tracking nitrate and sulfate sources in groundwater of an urbanized valley using a multi-tracer approach combined with a Bayesian isotope mixing model. Water Research, 182, 115962.	Mora, A., Mahlknecht, J., Rosales-Lagarde, L., Hernández-Antonio, A. (2017). Assessment of major ions and trace elements in groundwater supplied to the Monterrey metropolitan area, Nuevo León, Mexico. Environmental monitoring and assessment, 189, 1-15.	-	Approximated from Fig. 1c by Torres-Martínez et al. (2020) and Fig. 1 by Mora et al. (2017). Northern margin approximated by the Rio Salinas.
**Moor Limestone Aquifer**	-	Arauzo, M., Martínez-Bastida, J. J. (2015). Environmental factors affecting diffuse nitrate pollution in the major aquifers of central Spain: groundwater vulnerability vs. groundwater pollution. Environmental Earth Sciences, 73, 8271-8286.	-	-	Approximated from Fig. 1 by Arauzo and Martínez-Bastida (2015)
**Moraleja Aquifer**	-	Story, J., Lopez-Gunn, E. (2010). Comparing conflict in transboundary aquifer management: some insights from a comparative study between Spain and Australia. UNESCO-IAH-UNEP Conference, Paris, 7 pp. Accessed April 25, 2023 via http://www.siagua.org/sites/default/files/documentos/documentos/espaa_australia.pdf	-	-	Approximated from Fig. 3 by Story and Lopez-Gunn (2010)

Aquifer system	Broader aquifer system	Reference A	Reference B	Reference C	Steps to delineate
**Motril-Salobrena Aquifer**	-	Duque, C., Calvache, M. L., Engesgaard, P. (2010). Investigating river–aquifer relations using water temperature in an anthropized environment (Motril-Salobreña aquifer). Journal of Hydrology, 381, 121-133.	-	-	Approximated from
**Moulton Valley**	Interior Low Plateaus	Geological Survey of Alabama (2018). Assessment of groundwater resources in Alabama, 2010-16. Geological Survey of Alabama Bulletin 186, 462 pp. Accessed April 8, 2021 from https://www.gsa.state.al.us/img/Groundwater/docs/assessment/00_B186_StatewideAssessment_Print_Document.pdf	Miller, J.A. (1990). Ground Water Atlas of the United States: Segment 6, Alabama, Florida, Georgia, South Carolina. U.S. Geological Survey Hydrologic Atlas 730-G, 30 pp. Accessed April 5, 2021 from https://www.nrc.gov/docs/ML1706/ML17060B027.pdf	-	Moulton Valley subarea approximated from Fig. 4 of Geological Survey of Alabama (2008) and Fig. 104 of Miller (1990). Broader Interior Low Plateaus outline approximated for Alabama from Fig. 4 of Geological Survey of Alabama (2008) and Fig. 104 of Miller (1990).
**Mount Vernon Hill County**	Central Lowland Till Plain	Leighton, M. M., Ekblaw, G. E., Horberg, L. (1948). Physiographic divisions of Illinois. The Journal of Geology, 56, 16-33.	Fenneman, N.M., Johnson, D.W. (1946). Physiographic divisions of the conterminous United States. U.S. Geological Survey map, 1:7,000,000 Scale.	-	Broader Till Plain physiographic region approximated from Fenneman and Johnson (1946). Mount Vernon Hill County approximated from Figs. 1 and 2 of Leighton et al. (1948)
**Mountain Home Plateau**	Western Snake River Plain	Graham, W.G., Campbell, L.J. (1981). Groundwater resources of Idaho. Idaho Department of Water Resources Report, 61 pp. Accessed March 23, 2021 from https://idwr.idaho.gov/files/publications/198108-MISC-GW-Resources-ID.pdf	Young, H.W. (1977). Reconnaissance of ground-water resources in the Mountain Home plateau area, southwest Idaho. U.S. Geological Survey Water--Resources Investigations Report 77-108, 48 pp. Accessed November 29, 2021 from https://pubs.usgs.gov/wri/1977/0108/report.pdf	Lindholm, G. F. (1996). Summary of the Snake River Plain regional aquifer-system analysis in Idaho and eastern Oregon. U.S Geological Survey Professional Paper 1408-A. 59 pp. Accessed March 1, 2021 via https://pubs.usgs.gov/pp/1408a/report.pdf	Approximated from Plate 1 of Graham and Campbell (1981) and Fig. 1 by Young (1977). Broader portions of the Snake River Plain (i.e., Central, Western and Eastern) were approximated from Fig. 1 of Lindholm (1996).

Aquifer system	Broader aquifer system	Reference A	Reference B	Reference C	Steps to delineate
**Mud Lake**	Eastern Snake River Plain	Rattray, G. (2015). Geochemical evolution of groundwater in the Mud Lake area, Eastern Idaho, USA. Environmental Earth Sciences, 73(12), 8251-8269.	-	-	Approximated from Fig. 2 of Rattray (2015)
**Mud Meadows**	-	Lopes, T.J., Evetts. D.M. (2005). Ground-Water pumpage and artificial recharge estimates for calendar year 2000 and average annual natural recharge and interbasin flow by hydrographic area, Nevada. Accessed April 25, 2022 via https://pubs.usgs.gov/sir/2004/5239/sir2004-5239.pdf	Harrill, J.R., Prudic, D.E. (1998). Aquifer systems in the Great Basin region of Nevada, Utah, and adjacent states; summary report. US Geological Survey Professional Paper 1409-A, 75 pp. Accessed April 26, 2022 via https://pubs.usgs.gov/pp/1409a/report.pdf	-	Approximated from Fig. 1 by Lopes and Evetts (2005), and Fig. 2 by Harrill and Prudic (1998). Well completion records, physiography and drainage were used to delinate areas.
**Muddy River Springs Area**	-	Lopes, T.J., Evetts. D.M. (2005). Ground-Water pumpage and artificial recharge estimates for calendar year 2000 and average annual natural recharge and interbasin flow by hydrographic area, Nevada. Accessed April 25, 2022 via https://pubs.usgs.gov/sir/2004/5239/sir2004-5239.pdf	Harrill, J.R., Prudic, D.E. (1998). Aquifer systems in the Great Basin region of Nevada, Utah, and adjacent states; summary report. US Geological Survey Professional Paper 1409-A, 75 pp. Accessed April 26, 2022 via https://pubs.usgs.gov/pp/1409a/report.pdf	-	Approximated from Fig. 1 by Lopes and Evetts (2005), and Fig. 2 by Harrill and Prudic (1998). Well completion records, physiography and drainage were used to delinate areas.
**Mulege Aquifer**	-	CONAGUA (2021). Aguas subterráneas/ Acuíferos. Webpage accessed May 6, 2021 from https://sigagis.conagua.gob.mx/aprovechamientos/	-	-	Approximated from digital dataset available for viewing at CONAGUA (2021).
**Mulgildie Outlier**	Great Artesian Basin	Ransley, T.R., Smerdon, B.D. (2012). Hydrostratigraphy, hydrogeology and system	Ransley, T.R., Radke, B.M., Feitz, A.J., Kellett, J.R., Owens, R., Bell, J. and Stewart, G. (2015).	-	Approximated from Fig. 2.3 by Ransley and Smerdon (2012) and Map 4 by Ransley et al. (2015)

Aquifer system	Broader aquifer system	Reference A	Reference B	Reference C	Steps to delineate
		conceptualisation of the Great Artesian Basin. A technical report to the Australian Government from the CSIRO Great Artesian Basin Water Resource Assessment, 324 pp. Accessed July 20, 2021 from https://publications.csiro.au/rpr/download?pid=csiro:EP132693&dsid=DS5	Hydrogeological Atlas of the Great Artesian Basin, Geoscience Australia. Canberra, 141 pp. Accessed July 19, 2021 from http://www.ga.gov.au/scientific-topics/water/groundwater/gab		
**Mulgrave Aquifer**	-	Department of Science, IT, Innovation and the Arts (2013). Mulgrave River Basin Hydrology - Development of groundwater flow model for the Mulgrave River Basin. Report prepared for the Department of Natural Resources and Mines for the Wet Tropics Draft Water Resource Plan. 81 pp. Accessed May 8, 2023 via https://nla.gov.au/nla.obj-2742766628/view	-	-	Approximated from Fig. 1.1 and descriptions in background section (i.e., section 1.1) within Department of Science, IT, Innovation and the Arts (2013)
**Murchehkhort Basin**	Lower Zayandeh-Rud Basin	Hosseini, S. M., Parizi, E., Ataie-Ashtiani, B., Simmons, C. T. (2019). Assessment of sustainable groundwater resources management using integrated environmental index: Case studies across Iran. Science of the Total Environment, 676, 792-810.	Madani, K., Mariño, M. A. (2009). System dynamics analysis for managing Iran's Zayandeh-Rud river basin. Water Resources Management, 23, 2163-2187.	Salemi, H.R., Mamanpoush, A.R., Miranzadeh, M., Akbari, M., Torabi, M., Toomanian, N., Murray-Rust, H., Droogers, P., Sally, H., Gieske, A. (2000). Water management for sustainable irrigated agriculture in the Zayandeh Rud Basin, Esfahan Province, Iran Report by Iranian Agricultural Engineering Research Institute, Esfahan	Approximated from Fig. 6 by Hosseini et al. (2019), Fig. 1 by Madani and Mariño (2009), and Fig. 1 by Salemi et al. (2000)

Aquifer system	Broader aquifer system	Reference A	Reference B	Reference C	Steps to delineate
				Agricultural Research Center, and the International Water Management Institute, Research Report Number 1	
**Muscatatuck Plateau and Dearborn Upland**	-	Gray, H.H. (2001). Map of Indiana showing physiographic divisions. Indiana Geological Survey Miscellaneous Map 69, 15 pp. Accessed April 12, 2021 from https://igws.indiana.edu/ReferenceDocs/Maps/PhysiographicRegions.pdf	Schrader, G.P. (2004). Unconsolidated Aquifer Systems of Ripley County, Indiana. Indiana Department of Natural Resources, Division of Water Report, 3 pp. Accessed April 12, 2021 from https://www.in.gov/dnr/water/files/ripley_unconsolidated_text.pdf	-	Approximated from map by Gray (2001). Muscatatuck Plateau and Dearborn Upland combined as local hydrogeologic report refers to these two regions as a single unit (e.g., quote Schrader (2004): "Muscatatuck Plateau / Dearborn Upland Till Aquifer Subsystem."
**Muteh Basin**	-	Hosseini, S. M., Parizi, E., Ataie-Ashtiani, B., Simmons, C. T. (2019). Assessment of sustainable groundwater resources management using integrated environmental index: Case studies across Iran. Science of the Total Environment, 676, 792-810.	-	-	Approximated from Fig. 6 by Hosseini et al. (2019)
**Myalup and Yalgorup Plain**	Perth Basin	Ali, R., McFarlane, D., Varma, S., Dawes, W., Emelyanova, I., Hodgson, G., Charles, S. (2012). Potential climate change impacts on groundwater resources of south-western Australia. Journal of Hydrology, 475, 456-472.	Semeniuk, V., Semeniuk, C. A. (2006). Sedimentary fill of basin wetlands, central Swan Coastal Plain, southwestern Australia. Part 2: distribution of sediment types and their stratigraphy. Journal of the Royal Society of Western Australia, 89, 185.	-	Approximated from Fig. 1b by Ali et al. (2012) and Fig. 2 by Semeniuk and Semeniuk (2006)
**Myghan Basin**	-	Hosseini, S. M., Parizi, E., Ataie-Ashtiani, B., Simmons, C. T. (2019).	-	-	Approximated from Fig. 6 by Hosseini et al. (2019)

Aquifer system	Broader aquifer system	Reference A	Reference B	Reference C	Steps to delineate
		Assessment of sustainable groundwater resources management using integrated environmental index: Case studies across Iran. Science of the Total Environment, 676, 792-810.			
**Naein Basin**	-	Hosseini, S. M., Parizi, E., Ataie-Ashtiani, B., Simmons, C. T. (2019). Assessment of sustainable groundwater resources management using integrated environmental index: Case studies across Iran. Science of the Total Environment, 676, 792-810.	-	-	Approximated from Fig. 6 by Hosseini et al. (2019)
**Nahavand Plain**	-	Banejad, H., Mohebzadeh, H., Ghobadi, M. H., Heydari, M. (2014). Numerical simulation of groundwater flow and contamination transport in Nahavand Plain aquifer, west of Iran. Journal of the Geological Society of India, 83, 83-92.	-	-	Approximated from Fig. 1 by Banejad et al. (2014)
**Namakkal Fractured Charnockite and Gneiss Aquifers**	Coimbato re Upland	Ballukraya, P. N., Kalimuthu, R. (2010). Quantitative hydrogeological and geomorphological analyses for groundwater potential assessment in hard rock terrains. Current Science, 253-259.	Saha, D., Shekhar, S., Ali, S., Vittala, S.S., Raju, N.J. (2016). Recent hydrogeological research in India. Proceedings of the Indian National Science Academy, 82, 787-803.	-	Approximated from Fig. 1 by Ballukraya and Kalimuthu (2010) and Fig. 1 by Saha et al. (2016)
**Nanaimo Lowlands**	-	Pisani, J. (2020). Regional Groundwater Level Analysis Pre-Summer 2020. Staff	-	-	Approximated from Fig. 1 of Pisani (2020).

Aquifer system	Broader aquifer system	Reference A	Reference B	Reference C	Steps to delineate
		Report to the Regional District of Nanaimo. 6 pp. Accessed April 26, 2021 from https://rdn-pub.escribemeetings.com/filestream.ashx?DocumentId=13450			
**Nanchuan Area**	Huangshui Basin	Li, H., Zhan, R., Lu, Y., Zhou, B., Wu, J. (2021). Spatiotemporal variation and periodic evolution characteristics of groundwater in the Xining area of China, eastern Qinghai–Tibet Plateau. Environmental Earth Sciences, 80, 1-19.	-	-	Approximated from Fig. 1b and Fig. 1d by Li et al. (2021)
**Nanyang Basin**	-	Cao, S., Fei, Y., Tian, X., Cui, X., Zhang, X., Yuan, R., Li, Y. (2021). Determining the origin and fate of nitrate in the Nanyang Basin, Central China, using environmental isotopes and the Bayesian mixing model. Environmental Science and Pollution Research, 1-19.	-	-	Approximated from Fig. 1 by Cao et al. (2021)
**Napa Valley**	-	Kunkle, F., Upson, J. E. (1960). Geology and ground water in Napa and Sonoma Valleys, Napa and Sonoma Counties, California. U.S. Geological Survey Water-Supply Paper 1495, 260 pp. Accessed March 27, 2021 from https://pubs.usgs.gov/wsp/1495/report.pdf	-	-	Approximated from Plate 4 of Kunkle and Upson (1960).

Aquifer system	Broader aquifer system	Reference A	Reference B	Reference C	Steps to delineate
**Napanee Limestone Plain**	-	Sharpe, D.R., Piggott, A., Carter, T., Gerber, R.E., MacRitchie, S.M., de Loë, R.C., Strynatka, S., Zwiers, G., Rivera, A. (2013). Southern Ontario hydrogeological region. in: Canada's Groundwater Resources, pp. 444-499. Fitzhenry and Whiteside Limited, Markham, Ontario, Canada. Accessed May 5, 2021 from https://www.researchgate.net/profile/David-Sharpe-2/publication/284034021_Southern_Ontario_hydrogeological_region/links/5f8f462192851c14bcd85341/Southern-Ontario-hydrogeological-region.pdf	-	-	Approximated from Box 12-5 by Sharpe et al. (2013).
**Naqadeh Plain**	-	Shahmohammadi-Kalalagh, S., Taran, F., Nasiri, H. (2020). Investigating groundwater level fluctuations via analyzing groundwater hydrograph: a case study of Naqadeh plain in north-west of Iran. Sustainable Water Resources Management, 6, 1-11.	-	-	Approximated from Fig. 1 by Shahmohammadi-Kalalagh et al. (2020)
**Nara Basin**	-	Taniguchi, M. (1994). Estimated recharge rates from groundwater temperatures in the Nara Basin, Japan. Applied hydrogeology, 2, 7-14.	Pathak, D. (2011). Hydrogeology of shallow and deep aquifers in Nara Basin, West Japan. Journal of Nepal Geological Society, 43, 267-275.	-	Approximated from Fig. 1 by Taniguchi (1994) and Fig. 1 by Pathak (2011)
**Narbonne Basin**	-	Khaska, M., La Salle, C. L. G., Lancelot, J., Mohamad, A., Verdoux, P., Noret, A.,	-	-	Approximated from Fig. 1 by Khaska et al. (2013)

Aquifer system	Broader aquifer system	Reference A	Reference B	Reference C	Steps to delineate
		Simler, R. (2013). Origin of groundwater salinity (current seawater vs. saline deep water) in a coastal karst aquifer based on Sr and Cl isotopes. Case study of the La Clape massif (southern France). Applied Geochemistry, 37, 212-227.			
**Narimani Basin**	Mashad-Narimani Basin	Khashei-Siuki, A., Sarbazi, M. (2015). Evaluation of ANFIS, ANN, and geostatistical models to spatial distribution of groundwater quality (case study: Mashhad plain in Iran). Arabian Journal of Geosciences, 8, 903-912.	Naghibi, S. A., Vafakhah, M., Hashemi, H., Pradhan, B., & Alavi, S. J. (2018). Groundwater augmentation through the site selection of floodwater spreading using a data mining approach (case study: Mashhad Plain, Iran). Water, 10, 1405.	Masoumi, M., Gharaie, M. H. M., Ahmadzadeh, H. (2019). Assessment of groundwater quality for the irrigation of melon farms: a comparison between two arable plains in northeastern Iran. Environmental Earth Sciences, 78, 1-17.	Approximated from Fig. 2 by Khashei-Siuki and Sarbazi (2015), Fig. 2 by Naghibi et al. (2018), and Fig. 2 by Masoumi et al. (2019)
**Navidad-Potosi-Raices Aquifer**	-	CONAGUA (2015o). Actualización de la disponibilidad media anual de agua en el acuífero Navidad-Potosi-Raices (1916), Estado de Nuevo Leon. 30 pp. Accessed March 7, 2022 via https://www.gob.mx/cms/uploads/attachment/file/103175/DR_1916.pdf	-	-	Approximated from Fig. 1 by CONAGUA (2015o)
**Nayband Basin**	-	Hosseini, S. M., Parizi, E., Ataie-Ashtiani, B., Simmons, C. T. (2019). Assessment of sustainable groundwater resources management using integrated environmental index: Case studies across Iran. Science of the Total	-	-	Approximated from Fig. 6 by Hosseini et al. (2019)

Aquifer system	Broader aquifer system	Reference A	Reference B	Reference C	Steps to delineate
		Environment, 676, 792-810.			
**Nehbandan Aquifer**	-	Chamanehpour, E., hossein Sayadi, M., Yousefi, E. (2020). The potential evaluation of groundwater pollution based on the intrinsic and the specific vulnerability index. Groundwater for Sustainable Development, 10, 100313.	-	-	Approximated from Fig. 1 by Chamanehpour et al. (2020)
**New Brunswick Carboniferous Basin**	Maritimes Basin	Stapinsky, M. Michaud, Y., Morin, R.H., Butler, K.E., Deblonde, C., Chi, G, Thériault, T., Castonguay, S., Boisvert, V., Julien, H.P., Conohan, D., Hulsman, B., Carrier, C. Marion, J., Boisvert, É. (2002). Groundwater resources assessment in the Carboniferous Maritimes Basin: preliminary results of the hydrogeological characterization, New Brunswick, Nova Scotia, and Prince Edward Island. Geological Survey of Canada Current Research Report 2002-D8, 14 pp. Accessed May 5, 2021 from http://www.gov.pe.ca/photos/original/cle_WA10.pdf	Rivard, C., Michaud, Y., Lefebvre, R., Deblonde, C., Rivera, A. (2008). Characterization of a regional aquifer system in the Maritimes Basin, Eastern Canada. Water Resources Management, 22, 1649-1675.	-	Approximated from Fig. 2 of Rivard et al. (2008) and Figs. 1 and 3 of Stapinsky et al. (2002). Eastern portion of this area delineated along divide between the "Sackville" and "Cumberland" subbasins defined in Fig. 3 by Stapinsky et al. (2002).
**New Hampshire Seacoast**	-	Mack, T.J. (2008). Assessment of Ground-Water Resources in the Seacoast Region of New Hampshire. Scientific Investigations Report	-	-	Approximated from Fig. 7 of Mack (2008)

Aquifer system	Broader aquifer system	Reference A	Reference B	Reference C	Steps to delineate
		2008–5222, 66 pp. Accessed April 15, 2021 from https://pubs.usgs.gov/sir/2008/5222/pdf/sir2008-5222.pdf			
**New Jersey Coastal Plain**	North Atlantic Coastal Plain	Gill, H.E., Farlekas, G.M. (1976). Geohydrologic maps of the Potomac-Raritan-Magothy aquifer system in the New Jersey Coastal Plain. U.S. Geological Survey Hydrologic Atlas 557, 2 plates. Accessed April 1, 2021 from https://pubs.er.usgs.gov/publication/ha557	Gordon, A.D., Carleton, G.B., Rosman, R. (2021). Water-level conditions in the confined aquifers of the New Jersey Coastal Plain, 2013: U.S. Geological Survey Scientific Investigations Report 2019–5146, 104 p., 9 pl., Accessed April 1, 2021 from https://pubs.usgs.gov/sir/2019/5146/sir20195146.pdf	Masterson, J.P., Pope, J.P., Monti, Jack, Jr., Nardi, M.R., Finkelstein, J.S., McCoy, K.J. (2013). Hydrogeology and hydrologic conditions of the Northern Atlantic Coastal Plain aquifer system from Long Island, New York, to North Carolina. US Geological Survey Scientific Investigations Report 2013–5133, 88 pp. Accessed June 13, 2022 via http://dx.doi.org/10.3133/sir20135133	Approximated from Plate 1 of Gill et al. (1976) and Fig. 1 of Gordon et al. (2021), with the broader aquifer system approximated from Fig. 1 by Masterton et al. (2013)
**Newcastle Till Plain**	Central Lowland Till Plain	Gray, H.H. (2001). Map of Indiana showing physiographic divisions. Indiana Geological Survey Miscellaneous Map 69, 15 pp. Accessed April 12, 2021 from https://igws.indiana.edu/ReferenceDocs/Maps/PhysiographicRegions.pdf	Brockman, S. (1998). Physiographic regions of Ohio. Ohio Department of Natural Resources, Division of Geological Survey map. Accessed April 12, 20221 from https://www.epa.state.oh.us/portals/27/SIP/Nonattain/F2-physiographic_regions_of_Ohio.pdf	Fenneman, N.M., Johnson, D.W. (1946). Physiographic divisions of the conterminous United States. U.S. Geological Survey map, 1:7,000,000 Scale.	Broader Till Plain physiographic region approximated from Fenneman and Johnson (1946). Newcastle Till Plain approximated for Indiana from map by Gray (2001) and for Ohio following the approximate border of the "Southern Ohio Loamy Till Plain" defined in map by Brockman (1998).
**Neyriz Plain**	-	Shabani, M. (2012). Determining the most suitable interpolation method for groundwater chemical characteristics mapping. Journal of Watershed Engineering and	-	-	Approximated from Fig. 2 by Shabani (2012)

Aquifer system	Broader aquifer system	Reference A	Reference B	Reference C	Steps to delineate
		Management, 10 pp. Accessed June 22, 2021 from https://jwem.areeo.ac.ir/article_101859_45e8c0d01160de2bc58f84df869baf01.pdf?lang=en			
**Niagara Peninsula**	Southern Ontario Paleozoic Aquifer System	Sharpe, D.R., Piggott, A., Carter, T., Gerber, R.E., MacRitchie, S.M., de Loë, R.C., Strynatka, S., Zwiers, G., Rivera, A. (2013). Southern Ontario hydrogeological region. in: Canada's Groundwater Resources, pp. 444-499. Fitzhenry and Whiteside Limited, Markham, Ontario, Canada. Accessed May 5, 2021 from https://www.researchgate.net/profile/David-Sharpe-2/publication/284034021_Southern_Ontario_hydrogeological_region/links/5f8f462192851c14bcd85341/Southern-Ontario-hydrogeological-region.pdf	-	-	Approximated from Figs. 12.1 and 12.5 of Sharpe et al. (2013)
**Niles Cone**	-	California Department of Water Resources (2015g). California's groundwater update 2013 - Chapter 4: San Francisco Bay Hydrologic Region. California Department of Water Resources Report, 78 pp. Accessed April 27, 2022 via https://cawaterlibrary.net/wp-content/uploads/2017/05/G	-	-	Approximated from Fig. 4-2 by California Department of Water Resources (2015g)

Aquifer system	Broader aquifer system	Reference A	Reference B	Reference C	Steps to delineate
		WU2013_Ch4_SanFranciscoBay_Final.pdf			
**Nochixtlan Aquifer**	-	CONAGUA (2021a). Acuíferos (nacional). Webpage accessed March 8, 2022 from http://sina.conagua.gob.mx/sina/tema.php?tema=acuiferos&ver=mapa&o=1&n=nacional	-	-	Approximated from aquifer boundaries defined by CONAGUA (2021a) web portal, physiography and drainage patterns, and well completion data.
**Nodej Basin**	-	Hosseini, S. M., Parizi, E., Ataie-Ashtiani, B., Simmons, C. T. (2019). Assessment of sustainable groundwater resources management using integrated environmental index: Case studies across Iran. Science of the Total Environment, 676, 792-810.	-	-	Approximated from Fig. 6 by Hosseini et al. (2019)
**Norabad Basin**	-	Hosseini, S. M., Parizi, E., Ataie-Ashtiani, B., Simmons, C. T. (2019). Assessment of sustainable groundwater resources management using integrated environmental index: Case studies across Iran. Science of the Total Environment, 676, 792-810.	-	-	Approximated from Fig. 6 by Hosseini et al. (2019)
**Norfolk Chalk Aquifer**	Chalk Aquifer System	MacDonald, A.M., Allen, D.J. (2001). Aquifer properties of the Chalk of England. Quarterly Journal of Engineering Geology and Hydrogeology, 34, 371-384.	-	-	Approximated from Fig. 8 by MacDonald and Allen (2001)

Aquifer system	Broader aquifer system	Reference A	Reference B	Reference C	Steps to delineate
**Norman Upland**	Interior Low Plateaus	Miller, J.A. (1995). Ground Water Atlas of the United States: Segment 10, Illinois, Indiana, Kentucky, Ohio, Tennessee. U.S. Geological Survey Hydrologic Atlas 730-K, 30 pp. Accessed April 11, 2021 from https://pubs.usgs.gov/ha/730k/report.pdf	Taylor, C.J., Nelson Jr., H.L. (2008). A compilation of provisional karst geospatial data for the Interior Low Plateaus physiographic region, central United States: U.S. Geological Survey Data Series 339, 26 pp. Accessed April 11, 2021 from https://pubs.usgs.gov/ds/339/pdf/ds339_web.pdf	Fenelon, J.M., Bobay, K.E., Greeman, T.K., Hoover, M.E., Cohen, D.A., Fowler, K.K., Woodfield, M.C., Durbin, J.M. (1994). Hydrogeologic atlas of aquifers in Indiana: U.S. Geological Survey Water-Resources Investigations Report 1992–4142, 197 pp. Accessed April 11, 2021 from https://pubs.er.usgs.gov/publication/wri924142	Broader Interior Low Plateaus outline approximated for Tennessee, Illinois, Kentucky, Indiana from Fig. 78 of Miller (1990) and Fig. 1 of Taylor and Nelson (2008). Norman Upland approximated from Fig. 2 of Fenelon et al. (1994).
**North American Basin**	Sacramento Valley of the Central Valley **	California Department of Water Resources (2015i). California's groundwater update 2013 - Chapter 7: Sacramento River Hydrologic Region. California Department of Water Resources Report, 126 pp. Accessed April 27, 2022 via https://water.ca.gov/-/media/DWR-Website/Web-Pages/Programs/Groundwater-Management/Bulletin-118/Files/Statewide-Reports/GWU2013_Ch7_SacramentoRiver_Final.pdf	-	-	Approximated from Fig. 7-2 by California Department of Water Resources (2015i)
**North Carolina and Virginia Coastal Plain**	North Atlantic Coastal Plain	Meng, A., Harsh, J.F. (1988). Hydrogeologic framework of the Virginia coastal plain. U.S. Geological Survey Professional Paper 1404-C, 85 pp. Accessed April 1, 2021 from https://pubs.usgs.gov/pp/pp1404-C/pdf/pp_1404-c.pdf	Winner Jr., M.D., Coble, R.W. (1989). Hydrogeologic framework of the North Carolina Coastal Plain aquifer system. U.S. Geological Survey Report 87-690, 167 pp. Accessed April 1, 2021 from https://pubs.usgs.gov/of/1987/0690/report.pdf	-	Approximated from Fig. 1 of Meng and Harsh (1988) and Fig. 1 of Winner Jr. and Coble (1989).

Aquifer system	Broader aquifer system	Reference A	Reference B	Reference C	Steps to delineate
**North Chianan Plain**	Chianan Plain	Wang, S. J., Lee, C. H., Yeh, C. F., Choo, Y. F., Tseng, H. W. (2021). Evaluation of Climate Change Impact on Groundwater Recharge in Groundwater Regions in Taiwan. Water, 13, 1153.	Reza, A.S., Jean, J.S., Yang, H.J., Lee, M.K., Hsu, H.F., Liu, C.C., Lee, Y.C., Bundschuh, J., Lin, K.H., Lee, C. Y. (2011). A comparative study on arsenic and humic substances in alluvial aquifers of Bengal delta plain (NW Bangladesh), Chianan plain (SW Taiwan) and Lanyang plain (NE Taiwan): implication of arsenic mobilization mechanisms. Environmental Geochemistry and Health, 33, 235-258.	Hsu, S. K. (1998). Plan for a groundwater monitoring network in Taiwan. Hydrogeology Journal, 6, 405-415.	Approximated from Fig. 2 by Wang et al. (2021), Fig. 1b by Reza et al. (2011) and Fig. 2 by Hsu (1998)
**North Okanagan**	Okanagan Basin	Golder Associates and Summit Environmental Consultants Ltd. (2009). Phase 2 Okanagan Water Supply and Demand Project: Groundwater Objectives 2 and 3 Basin Study.Report to Okanagan Basin Water Board, 114 pp. Accessed March 16, 2021 from https://www.obwb.ca/wsd/about/project-reports	-	-	Approximated from description on page 37 ("7.3.3. North Okanagan") of report by Golder Associates and Summit Environmental Consultants Ltd. (2009) and Fig. 5
**North Rewa Basin**	Son-Mahandai Valley Basins	Chowdari, S., Singh, B., Rao, B. N., Kumar, N., Singh, A. P., Chandrasekhar, D. V. (2017). Structural mapping based on potential field and remote sensing data, South Rewa Gondwana Basin, India. Journal of Earth System Science, 126, 1-27.	Central Ground Water Board (2013b). Aquifer Systems of Madhya Pradesh. Central Ground Water Board Report, 78 pp. Accessed July 17, 2021 from http://cgwb.gov.in/AQM/Madhya%20Pradesh.pdf	Dutta, P.K., Acharyya, S.K., Chakraborty, T., Jha, N., Khangar, R., Khasdeo, L., Misra, K.G. (2015). Resolving Kamthi-related problems in Gondwana stratigraphy of peninsular India. Indian Journal of Geosciences, 69, 85-102.	Approximated from Fig. 2 by Chowdari et al. (2017), Plate XXIX by Central Ground Water Board (2013b), and Fig. 1 by Dutta et al. (2015)

Aquifer system	Broader aquifer system	Reference A	Reference B	Reference C	Steps to delineate
**North San Benito**	Gilroy-Hollister Valley	California Department of Water Resources (2015g). California's groundwater update 2013 - Chapter 4: San Francisco Bay Hydrologic Region. California Department of Water Resources Report, 78 pp. Accessed April 27, 2022 via https://cawaterlibrary.net/wp-content/uploads/2017/05/GWU2013_Ch4_SanFranciscoBay_Final.pdf	-	-	Approximated from Fig. 4-2 by California Department of Water Resources (2015g)
**North Seeland Aquifer**	-	Musy, S., Meyzonnat, G., Barbecot, F., Hunkeler, D., Sültenfuss, J., Solomon, D. K., Purtschert, R. (2021). In-situ sampling for krypton-85 groundwater dating. Journal of Hydrology, 11, 100075.	-	-	Approximated from Fig. 7 by Musy et al. (2021)
**North West Dolomite Aquifer System**	-	Department of Water Affairs and Forestry, South Africa (2006). Vaal River system: large bulk water supply reconciliation strategy: groundwater assessment: dolomite aquifers. DWAF Report Number: P RSA C000/00/4406/06, 71 pp. Accessed October 19, 2021 from https://www.dws.gov.za/iwrp/Vaal/documents/LargeBulkWater/06_Dolomitic%20Groundwater%20Assessment_Final.pdf	-	-	Approximated from map on page 11 by Department of Water Affairs and Forestry, South Africa (2006)

Aquifer system	Broader aquifer system	Reference A	Reference B	Reference C	Steps to delineate
**North Yuba Basin**	Sacramento Valley of the Central Valley **	California Department of Water Resources (2015i). California's groundwater update 2013 - Chapter 7: Sacramento River Hydrologic Region. California Department of Water Resources Report, 126 pp. Accessed April 27, 2022 via https://water.ca.gov/-/media/DWR-Website/Web-Pages/Programs/Groundwater-Management/Bulletin-118/Files/Statewide-Reports/GWU2013_Ch7_SacramentoRiver_Final.pdf	-	-	Approximated from Fig. 7-2 by California Department of Water Resources (2015i)
**Northcentral Coromandel Coastal Alluvial Aquifer**	Coromandel Coastal Plain	Saha, D., Shekhar, S., Ali, S., Vittala, S.S., Raju, N.J. (2016). Recent hydrogeological research in India. Proceedings of the Indian National Science Academy, 82, 787-803.	Saha, D., Ray, R. K. (2019). Groundwater resources of India: potential, challenges and management. In Groundwater Development and Management. Springer, Cham (ed. Sikdar, P.K.), pp. 19-42.	-	Approximated from Fig. 2.2 by Saha and Ray (2019) and Fig. 1 by Saha et al. (2016)
**Northcentral Coromandel Schist Aquifers**	Coromandel Coastal Plain	Saha, D., Shekhar, S., Ali, S., Vittala, S.S., Raju, N.J. (2016). Recent hydrogeological research in India. Proceedings of the Indian National Science Academy, 82, 787-803.	Anand, A.V.S.S. (2009). Groundwater Brochure Nellore District, Andhra Pradesh. Central Ground Water Board, Ministry of Water Resources Report, 25 pp. Accessed June 24, 2021 from http://cgwb.gov.in/district_profile/ap/nellore.pdf	-	Approximated from Fig. 1 by Saha et al. (2016) and Fig. 5 by Anand (2013)
**Northcentral Deccan Basalt Plateau**	Deccan Plateau	Deolankar, S. B. (1980). The Deccan basalts of Maharashtra, India—their potential as aquifers.	Kale, V. S., Bodas, M., Chatterjee, P., Pande, K. (2020). Emplacement history and evolution of the Deccan Volcanic Province,	Duraiswami, R. A., Das, S., Shaikh, T. (2012). Hydrogeological framework of aquifers from the Deccan Traps, India: some insights.	Approximated from Fig. 1 by Deolankar (1980), Fig. 1 by Duraiswami et al. (2012), and Fig. 1 by Kale et al. (2016). Subarea

Aquifer system	Broader aquifer system	Reference A	Reference B	Reference C	Steps to delineate
		Groundwater, 18(5), 434-437.	India. Episodes, 43, 278-299.	Memoir Geological Society of India, 1-15.	based on topography and drainage patterns.
**North-central High Plains****	High Plains	Gutentag, E.D., Heimes, F.J., Krothe, N.C., Luckey, R.R., Weeks, J.B. (1984). Geohydrology of the High Plains aquifer in parts of Colorado, Kansas, Nebraska, New Mexico, Oklahoma, South Dakota, Texas, and Wyoming (No. 1400-B). Accessed February 10, 2021 from https://pubs.usgs.gov/pp/1400b/report.pdf	Luckey, R.L., Becker, M.F. (2003). Hydrogeology, water use, and simulation of flow in the High Plains aquifer in northwestern Oklahoma, southeastern Colorado, southwestern Kansas, northeastern New Mexico, and northwestern Texas. Water-Resources Investigations Report 99-4104, 73 pp. Accessed February 15, 2022 from https://pubs.usgs.gov/wri/wri994104/pdf/wri994104.pdf	McGuire, V.L., Johnson, M.R., Schieffer, J.S., Stanton, J.S., Sebree, S.K., Varstraeten (2003). Water in storage and approaches to groundwater management, High Plains Aquifer, 2000. US Geological Survey Circular 1243, 56 pp. Accessed February 15, 2022 from https://pubs.usgs.gov/circ/2003/circ1243/pdf/C1243.pdf	Approximated from Fig. 1 of Gutentag et al. (1984), Fig. 18 by McGuire et al. (2003), and Fig. 1 by Luckey and Becker (2003)
**Northcentral Hualien-Taitung Valley**	Hualien-Taitung Valley	Hsu, S. K. (1998). Plan for a groundwater monitoring network in Taiwan. Hydrogeology Journal, 6, 405-415.	-	-	Approximated from Fig. 1 by Fig. 2 by Hsu et al. (1998); subarea boundary based on drainage
**Northcentral Mega Aquifer System**	Mega Aquifer System	Awadh, S. M., Al-Mimar, H., Yaseen, Z. M. (2020). Groundwater availability and water demand sustainability over the upper mega aquifers of Arabian Peninsula and west region of Iraq. Environment, Development and Sustainability, 1-21.	Abotalib, A. Z., Heggy, E., Scabbia, G., Mazzoni, A. (2019). Groundwater dynamics in fossil fractured carbonate aquifers in Eastern Arabian Peninsula: a preliminary investigation. Journal of Hydrology, 571, 460-470.	-	Approximated from Fig. 1 by Awadh et al. (2020) and Fig. 1 by Abotalib et al. (2019)
**Northcentral Piedmont Plain**	North China Plain	Meng, S., Liu, J., Zhang, Z., Lei, T., Qian, Y., Li, Y., Fei, Y. (2015). Spatiotemporal evolution characteristics study on the precipitation infiltration recharge over the past 50 years in the North China Plain. Journal	Cao, G., Zheng, C., Scanlon, B. R., Liu, J., & Li, W. (2013). Use of flow modeling to assess sustainability of groundwater resources in the North China Plain.	Wang, S., Song, X., Wang, Q., Xiao, G., Liu, C., Liu, J. (2009). Shallow groundwater dynamics in North China plain. Journal of Geographical Sciences, 19, 175-188.	Approximated from Fig. 1 by Meng et al. (2015), Fig. 1 by Cao et al. (2013) and Fig. 6 by Wang et al. (2009).

Aquifer system	Broader aquifer system	Reference A	Reference B	Reference C	Steps to delineate
		of Earth Science, 26, 416-424.	Water Resources Research, 49, 159-175.		
**Northcentral Piedmont Upland**	Piedmont Plateau	Miller, J.A. (1990). Ground Water Atlas of the United States: Segment 6, Alabama, Florida, Georgia, South Carolina. U.S. Geological Survey Hydrologic Atlas 730-G, 30 pp. Accessed April 5, 2021 from https://www.nrc.gov/docs/ML1706/ML17060B027.pdf	-	-	Approximated from Fig. 3 of Miller (1990). Northeastern margin of area approximated along the Susquehanna River.
**Northcentral Swan Coastal Plain**	Perth Basin	Barron, O., Silberstein, R., Ali, R., Donohue, R., McFarlane, D.J., Davies, P., Hodgson, G., Smart, N. Donn, M., (2012). Climate change effects on water-dependent ecosystems in south-western Australia. Journal of Hydrology, 434, 95-109.	-	-	Approximated from Fig. 1 by Barron et al. (2012)
**Northcentral Valley and Ridge**	Valley and Ridge Aquifer System	Johnson, G.C., Zimmerman, T.M., Lindsey, B.D., Gross, E.L. (2011). Factors affecting groundwater quality in the Valley and Ridge aquifers, eastern United States, 1993–2002: U.S. Geological Survey Scientific Investigations Report 2011–5115, 84 pp. Accessed April 13, 2021 from https://pubs.usgs.gov/sir/2011/5115/support/sir2011-5115.pdf	-	-	Broader Valley and Ridge Aquifer System outline approximated from Fig. 1 of Johnson et al. (2011). Sorthern margin approximated at Deep Creek Lake and North Fork Shenandoah River.

Aquifer system	Broader aquifer system	Reference A	Reference B	Reference C	Steps to delineate
**Northeast Missouri Carbonates**	Northern Midwest Aquifer System	Miller, J.A. (1995). Ground Water Atlas of the United States: Segment 10, Illinois, Indiana, Kentucky, Ohio, Tennessee. U.S. Geological Survey Hydrologic Atlas 730-K, 30 pp. Accessed April 11, 2021 from https://pubs.usgs.gov/ha/730k/report.pdf	Miller, J.A., Appel, C.L. (1997). Ground Water Atlas of the United States: Segment 3, Kansas, Missouri, Nebraska. U.S. Geological Survey Hydrologic Atlas 730-D, 26 pp. Accessed April 13, 2021 from https://pubs.usgs.gov/ha/730d/report.pdf	-	Approximated from Fig. 9 of Miller and Appel (1997) and Fig. 12 of Miller (1995).
**Northeast Zealand Limestone and Sand Aquifer System**	-	Frei, R., Frei, K.M., Kristiansen, S.M., Jessen, S., Schullehner, J., Hansen, B. (2020). The link between surface water and groundwater-based drinking water–strontium isotope spatial distribution patterns and their relationships to Danish sediments. Applied Geochemistry, 121, 104698.	-	-	Approximated from Figs. 1 and 5 by Frei et al. (2020) and drainage patterns
**Northeastern Deccan Basalt Plateau**	Deccan Plateau	Deolankar, S. B. (1980). The Deccan basalts of Maharashtra, India—their potential as aquifers. Groundwater, 18(5), 434-437.	Kale, V. S., Bodas, M., Chatterjee, P., Pande, K. (2020). Emplacement history and evolution of the Deccan Volcanic Province, India. Episodes, 43, 278-299.	Duraiswami, R. A., Das, S., Shaikh, T. (2012). Hydrogeological framework of aquifers from the Deccan Traps, India: some insights. Memoir Geological Society of India, 1-15.	Approximated from Fig. 1 by Deolankar (1980), Fig. 1 by Duraiswami et al. (2012), and Fig. 1 by Kale et al. (2016). Subarea based on topography and drainage patterns.
**Northeastern Judith River**	Bearpaw-Judith River Aquifer System	Simpson, M.A. (1998). Geology and Hydrostratigraphy of the Rosetown Area (72O), Saskatchewan. Saskatchewan Research Council Publication No. 10416-2C98. Accessed May 4, 2021 from https://www.wsask.ca/Glob	Davies, H., Hanley, P.T. (2010). State of the Watershed Report - 2010. Water Security Agency, Saskatchewan. Appendix A, 82 pp. Accessed April 26, 2021 from https://www.wsask.ca/Global/About%20WSA/Publications/State%20of%20the%2	-	Approximated from Map 3 by Simpson (1998). Broad outline approximated following Fig. 28 of Davies and Hanley (2010).

Aquifer system	Broader aquifer system	Reference A	Reference B	Reference C	Steps to delineate
		al/Water%20Info/Ground%20Water/Mapping/Rosetown%20Mapsheet%2072O/Reports/10416-2C98.pdf	0Watershed/d_AppendixA-ConditionIndicators.PDF		
**Northeastern Karoo Basin**	Main Karoo Basin	Nyambe, I. A. (1999). Tectonic and climatic controls on sedimentation during deposition of the Sinakumbe Group and Karoo Supergroup, in the mid-Zambezi Valley Basin, southern Zambia. Journal of African Earth Sciences, 28, 443-463.	-	-	Broader aquifer system boundary (i.e., Main Karoo Basin) approximated from Fig. 1 by Nyambe (1999). Physiography (drainage patterns, geology) and well construction data were considered while digitizing subarea
**Northeastern Libres-Oriental Aquifer**	Libres-Oriental Aquifer	CONAGUA (2020hf). Actualización de la disponibilidad media anual de agua en el acuífero Libres-Oriental (2102), estado de Puebla. 14 pp. Accessed April 25, 2022 via https://sigagis.conagua.gob.mx/gas1/Edos_Acuiferos_18/puebla/DR_2102.pdf	-	-	Approximated from physiography, well completion data, and Fig. 1 by CONAGUA (2020hf)
**Northeastern Piedmont Plain**	North China Plain	Meng, S., Liu, J., Zhang, Z., Lei, T., Qian, Y., Li, Y., Fei, Y. (2015). Spatiotemporal evolution characteristics study on the precipitation infiltration recharge over the past 50 years in the North China Plain. Journal of Earth Science, 26, 416-424.	Cao, G., Zheng, C., Scanlon, B. R., Liu, J., & Li, W. (2013). Use of flow modeling to assess sustainability of groundwater resources in the North China Plain. Water Resources Research, 49, 159-175.	Wang, S., Song, X., Wang, Q., Xiao, G., Liu, C., Liu, J. (2009). Shallow groundwater dynamics in North China plain. Journal of Geographical Sciences, 19, 175-188.	Approximated from Fig. 1 by Meng et al. (2015), Fig. 1 by Cao et al. (2013) and Fig. 6 by Wang et al. (2009).
**Northern Aconcagua Basin**	Aconcagua Basin	Direccion General de Aguas (2009). Diagnóstico y Clasificación de Sectores Acuíferos, Volumen No. 2. Gobierno de Chile Ministerio de Obras Publicas Report, 384 pp. Accessed May 16,	-	-	Approximated from Table 1.8 on page 40 by Direccion General de Aguas (2009). Divide between northern, lower and middle portions of aquifer system approximated near Calera

Aquifer system	Broader aquifer system	Reference A	Reference B	Reference C	Steps to delineate
		2021 from https://snia.mop.gob.cl/sad/CQA5168v2.pdf			(northern portion extends into basin where Nogales is situated).
**Northern Adelaide Plains**	St Vincent Basin	Smith, M. L., Fontaine, K., Lewis, S. J. (2015). Regional Hydrogeological Characterisation of the St Vincent Basin, South Australia: Technical Report for the National Collaboration Framework Regional Hydrogeology Project. Geoscience Australia Record 2015/16, 150 pp. Accessed July 27, 2021 from https://d28rz98at9flks.cloudfront.net/78884/Rec2015_016.pdf	Bestland, E., George, A., Green, G., Olifent, V., Mackay, D., Whalen, M. (2017). Groundwater dependent pools in seasonal and permanent streams in the Clare Valley of South Australia. Journal of Hydrology: Regional Studies, 9, 216-235.	Bresciani, E., Cranswick, R. H., Banks, E. W., Batlle-Aguilar, J., Cook, P. G., Batelaan, O. (2018). Using hydraulic head, chloride and electrical conductivity data to distinguish between mountain-front and mountain-block recharge to basin aquifers. Hydrology and Earth System Sciences, 22, 1629-1648.	Approximated from Fig. 2.2 by Smith et al. (2015), Fig. 1 by Bestland et al. (2017), and Fig. 2 by Bresciani et al. (2018)
**Northern Allegheny Plateau**	Appalachian Plateaus	Trapp Jr., H., Horn, M.A. (1997). Ground water atlas of the United States: Segment 11. U.S. Geological Survey Hydrologic Investigations Atlas 730-L, 26 pp. Accessed April 13, 2021 from https://pubs.usgs.gov/ha/730l/report.pdf	-	-	Broader Appalachian Plateau Aquifer System approximated from Fig. 3 of Trapp Jr. and Horn (1997)
**Northern Aquitaine Basin**	Aquitaine Basin	Saltel, M., Rebeix, R., Thomas, B., Franceschi, M., Lavielle, B., Bertran, P. (2019). Paleoclimate variations and impact on groundwater recharge in multi-layer aquifer systems using a multi-tracer approach (northern Aquitaine basin, France).	La Gal La Salle, C., Marlin, C., Savoye, S., Fontes, J. C. (1996). Geochemistry and14C dating of groundwaters from Jurassic aquifers of North Aquitaine Basin (France). Applied Geochemistry, 11, 433-445.	Willmes, M., Bataille, C.P., James, H.F., Moffat, I., McMorrow, L., Kinsley, L., Armstrong, R.A., Eggins, S., Grün, R. (2018). Mapping of bioavailable strontium isotope ratios in France for archaeological provenance studies. Applied Geochemistry 90, 75-86.	Approximated from Fig. 1 by Saltel et al. (2019) and Fig. 1 by La Gal La Salle et al. (1996), with broader outline of Aquitaine Basin supported by delineation in Fig. 1 by Willmes et al. (2018).

Aquifer system	Broader aquifer system	Reference A	Reference B	Reference C	Steps to delineate
		Hydrogeology Journal, 27, 1439-1457.			
**Northern Cambrian-Ordovician Aquifers**	Northern Midwest Aquifer System	Young, H.L. (1992). Hydrogeology of the Cambrian-Ordovician Aquifer System in the Northern Midwest, United States. U.S. Geological Survey Professional Paper 1405-B, 108 pp. Accessed April 12, 2021 from https://pubs.usgs.gov/pp/1405b/report.pdf	Wilson, J.T. (2012). Water-quality assessment of the Cambrian-Ordovician aquifer system in the northern Midwest, United States. U.S. Geological Survey Scientific Investigations Report 2011–5229, 174 pp. Accessed April 12, 2021 from https://pubs.usgs.gov/sir/2011/5229/pdf/SIR20115229_web.pdf	-	Broader aquifer system title and its location approximated from Fig. 1 of Young (1992). Subcrop geology approximated from Fig. 9 of Wilson (2012).
**Northern Canterbury Plains**	-	Taylor, C. B., Wilson, D. D., Brown, L. J., Stewart, M. K., Burden, R. J., Brailsford, G. W. (1989). Sources and flow of north Canterbury plains groundwater, New Zealand. Journal of hydrology, 106, 311-340.	Bal, A. A. (1996). Valley fills and coastal cliffs buried beneath an alluvial plain: evidence from variation of permeabilities in gravel aquifers, Canterbury Plains, New Zealand. Journal of Hydrology (New Zealand), 1-27.	Wilson, D. D. (1973). The significance of geology in some current water resource problems, Canterbury Plains, New Zealand. Journal of Hydrology (New Zealand), 103-118.	Approximated from Fig. 1 by Taylor et al. (1989), Figs. 1 and 2 by Bal (1996) and Fig. 1 by Wilson (1973). Physiography and drinage features were applied to approximate subareas
**Northern Cauvery Delta and Cuddalore Sandstone Aquifer System**	Coromandel Coastal Plain	Vetrimurugan, E., Elango, L., Rajmohan, N. (2013). Sources of contaminants and groundwater quality in the coastal part of a river delta. International Journal of Environmental Science and Technology, 10, 473-486.	Gopinath, S., Srinivasamoorthy, K., Vasanthavigar, M., Saravanan, K., Prakash, R., Suma, C. S., Senthilnathan, D. (2018). Hydrochemical characteristics and salinity of groundwater in parts of Nagapattinam district of Tamil Nadu and the Union Territory of Puducherry, India. Carbonates and Evaporites, 33, 1-13.	Central Ground Water Board (2015). Pilot Project Report on Aquifer mapping in Lower Vellar watershed, Cuddalore District, Tamilnadu. Central Ground Water Board Report, 358 pp. Accessed June 24, 2021 from http://cgwb.gov.in/AQM/Pilot/Cuddalore%20District,%20Tami%20Nadu-Final.pdf	Approximated from Fig. 1 by Vetrimurugan et al. (2013), Fig. 1 by Gopinath et al. (2018) and Fig. 6.1 by Central Ground Water Board (2015)
**Northern Columbia River Basin**	Columbia Plateau Regional	Kahle, S.C., Morgan, D.S., Welch, W.B., Ely, S.R., Vaccaro, J.J., Orzol, L.L.	-	-	Columbia River Aquifer System approximated from Figs. 1 and 10 of Kahle et al. (2011).

Aquifer system	Broader aquifer system	Reference A	Reference B	Reference C	Steps to delineate
	Aquifer System	(2011). Hydrogeologic Framework and Hydrologic Budget Components of the Columbia Plateau Regional Aquifer System, Washington, Oregon, and Idaho. US Geological Survey Scientific Investigations Report 2011–5124, 80 pp. Accessed February 16, 2021 from https://pubs.usgs.gov/sir/2011/5124/pdf/sir20115124.pdf			
**Northern Colusa Basin**	Sacramento Valley of the Central Valley **	California Department of Water Resources (2015i). California's groundwater update 2013 - Chapter 7: Sacramento River Hydrologic Region. California Department of Water Resources Report, 126 pp. Accessed April 27, 2022 via https://water.ca.gov/-/media/DWR-Website/Web-Pages/Programs/Groundwater-Management/Bulletin-118/Files/Statewide-Reports/GWU2013_Ch7_SacramentoRiver_Final.pdf	-	-	Approximated from Fig. 7-2 by California Department of Water Resources (2015i)
**Northern Culverden Basin**	Culverden Basin	Poulsen, D. (2012). Culverden Basin hydrogeology. Environment Canterbury Regional Council Report No. R12/96, 104 pp. Accessed October 20, 2021 from https://www.ecan.govt.nz/	-	-	Approximated from Fig. 2-1 by Environment Canterbury Regional Council (2012). Subareas based on drainage and physiography.

Aquifer system	Broader aquifer system	Reference A	Reference B	Reference C	Steps to delineate
		document/download?uri=1723844			
**Northern Cumberland Plateau and Mountains**	Appalachian Plateaus	Brahana, J.V., Macy, J.A., Mulderink, D., Zemo, D. (1986). Preliminary delineation and description of the regional aquifers of Tennessee-Cumberland plateau aquifer system. U.S. Geological Survey, Water-Resources Investigations Report 82-338, 29 pp. Accessed April 13, 2021 from https://pubs.usgs.gov/wri/wrir82-338/pdf/wrir_82-338_a.pdf	Trapp Jr., H., Horn, M.A. (1997). Ground water atlas of the United States: Segment 11. U.S. Geological Survey Hydrologic Investigations Atlas 730-L, 26 pp. Accessed April 13, 2021 from https://pubs.usgs.gov/ha/730l/report.pdf	-	Approximated from Fig. 2 by Brahana et al. (1986). Broader Appalachian Plateau Aquifer System approximated from Fig. 3 of Trapp Jr. and Horn (1997)
**Northern Darz Sayebn Basin**	-	Hosseini, S. M., Parizi, E., Ataie-Ashtiani, B., Simmons, C. T. (2019). Assessment of sustainable groundwater resources management using integrated environmental index: Case studies across Iran. Science of the Total Environment, 676, 792-810.	-	-	Approximated from Fig. 6 by Hosseini et al. (2019)
**Northern Dezful-Andimeshk Plain**	Dezful-Andimeshk Plain	Hosseini, S. M., Parizi, E., Ataie-Ashtiani, B., Simmons, C. T. (2019). Assessment of sustainable groundwater resources management using integrated environmental index: Case studies across Iran. Science of the Total Environment, 676, 792-810.	-	-	Approximated from Fig. 6 by Hosseini et al. (2019)

Aquifer system	Broader aquifer system	Reference A	Reference B	Reference C	Steps to delineate
**Northern Dharwar Craton Banded Gneiss Complex**	Dharwar Craton Banded Gneiss Complex	Lancaster, P. J., Dey, S., Storey, C. D., Mitra, A., Bhunia, R. K. (2015). Contrasting crustal evolution processes in the Dharwar craton: insights from detrital zircon U–Pb and Hf isotopes. Gondwana Research, 28, 1361-1372.	-	-	Dharwar Craton geology approximated from Fig. 1 by Lancaster et al. (2015)
**Northern Duero Basin**	Duero Basin	Esteban, E., Albiac, J. (2012). The problem of sustainable groundwater management: the case of La Mancha aquifers, Spain. Hydrogeology Journal, 20, 851-863.	-	-	Broader outline of Duero Basin approximated from Fig. 1 by Esteban and Albiac (2012), guided by the presence of recorded well completion events and delineating contributing areas upstream of Zamora.
**Northern Eromanga Basin**	Great Artesian Basin	Ransley, T.R., Radke, B.M., Feitz, A.J., Kellett, J.R., Owens, R., Bell, J. and Stewart, G. (2015). Hydrogeological Atlas of the Great Artesian Basin, Geoscience Australia. Canberra, 141 pp. Accessed July 19, 2021 from http://www.ga.gov.au/scientific-topics/water/groundwater/gab	-	-	Approximated from Map 1 on page 9 of report by Ransley et al. (2015)
**Northern Georgina Basin**	Daly-Wiso-Georgina Basins	Crosbie, R. S., Rachakonda, P. K. (2021). Constraining probabilistic chloride mass-balance recharge estimates using baseflow and remotely sensed evapotranspiration: the Cambrian Limestone Aquifer in northern Australia. Hydrogeology Journal, 29, 1399-1419.	Wilkes, P. (2018). Baseline assessment of groundwater characteristics in the Beetaloo Sub-basin, NT. GISERA Project Order, 27 pp. Accessed October 6, 2021 from https://gisera.csiro.au/wp-content/uploads/2018/10/Water-16-Project-Order-1.pdf	de Caritat, P., Bastrakov, E.N., Jaireth, S., English, P.M., Clarke, J.D.A., Mernagh, T.P., Wygralak, A.S., Dulfer, H.E., Trafford, J. (2019). Groundwater geochemistry, hydrogeology and potash mineral potential of the Lake Woods region, Northern Territory, Australia. Australian Journal	Approximated from Fig. 1 by Crosbie and Rachakonda (2021), Fig. 1 by Wilkes (2018), and Fig. 2 by de Caritat et al. (2019)

Aquifer system	Broader aquifer system	Reference A	Reference B	Reference C	Steps to delineate
				of Earth Sciences, 66, 411-430.	
**Northern Guadalupe Garzaron Aquifer**	-	CONAGUA (2020l). Actualización de la disponibilidad media anual de agua en el acuífero Guadalupe Garzaron (3212), Estado de Zacatecas. 38 pp. Accessed March 7, 2022 via https://sigagis.conagua.gob.mx/gas1/Edos_Acuiferos_18/zacatecas/DR_3220.pdf	-	-	Approximated from Fig. 1 by CONAGUA (2020l). Southern and northern areas delineated on the basis of physiography and drainage
**Northern Guam Lens Aquifer *****	-	Gingerich, S.B. (2013). The effects of withdrawals and drought on groundwater availability in the Northern Guam Lens Aquifer, Guam. US Geological Survey Scientific Investigations Report 2013-5216, 92 pp. Accessed May 3, 2023 via https://pubs.usgs.gov/sir/2013/5216/pdf/sir2013-5216.pdf	Jocson, J. M. U., Jenson, J. W., Contractor, D. N. (2002). Recharge and aquifer response: northern Guam lens aquifer, Guam, Mariana Islands. Journal of Hydrology, 260(1-4), 231-254.	Rotzoll, K., Gingerich, S. B., Jenson, J. W., El-Kadi, A. I. (2013). Estimating hydraulic properties from tidal attenuation in the Northern Guam Lens Aquifer, territory of Guam, USA. Hydrogeology Journal, 21, 643-654.	Approximated from Gingerich (2013), Fig. 1 by Jocson et al. (2002), and Fig. 1 by Rotzoll et al. (2013)
**Northern Guarani Aquifer**	Guarani Aquifer System	Hirata, R., Foster, S. (2021). The Guarani Aquifer System–from regional reserves to local use. Quarterly Journal of Engineering Geology and Hydrogeology, 54, qjegh2020-091.	Gastmans, D., Chang, H. K., Hutcheon, I. (2010). Stable isotopes (2H, 18O and 13C) in groundwaters from the northwestern portion of the Guarani Aquifer System (Brazil). Hydrogeology Journal, 18, 1497-1513.	Rodríguez, L., Vives, L., Gomez, A. (2013). Conceptual and numerical modeling approach of the Guarani Aquifer System. Hydrology and Earth System Sciences, 17, 295-314.	Approximated from Fig. 1 By Gastmans et al. (2010) and Fig. 1 by Hirata and Foster (2021), who describe the three regions of the Guarani Aquifer System as delimited by two major arches: (i) the Ponta Grossa Arch (quote Hirata and Foster (2021): "which forces groundwater to flow from east to west in São Paulo State"), and (ii) the Asunción–Rio Grande (quote Hirata and Foster (2021): "..which divides the portion south of the Ponta Grossa Arch into two semi-independent sedimentary basins"). The locations of these

Aquifer system	Broader aquifer system	Reference A	Reference B	Reference C	Steps to delineate
					aforementioned arches were approximated from Fig. 2 by Rodríguez et al. (2013).
**Northern Hickory Aquifer**	-	Standen, A.R., Kane, J.A. (2005). The spatial distribution of radiological contaminants in the Hickory aquifer and other aquifers overlying the Llano Uplift, Central Texas. In Austin Geological Society Bulletin, 1, 87-101. Accessed May 1, 2023 via https://static1.squarespace.com/static/56e481e827d4bdfdac7fbe0f/t/56f7df34f699bbc538403d1f/1459085110397/Standen+and+Kane%2C+2005%2C+Radiological+Contaminants+in+Hickory+Aquifer.pdf	Hudak, P. F. (1999). Chloride and nitrate distributions in the Hickory aquifer, Central Texas, USA. Environment International, 25, 393-401.	-	Approximated from Fig. 3 by Standen and Kane (2005) and Fig. 1 by Hudak (1999)
**Northern High Plains**	High Plains	Peterson, S.M., Traylor, J.P., Guira, M. (2020). Groundwater Availability of the Northern High Plains Aquifer in Colorado, Kansas, Nebraska, South Dakota, and Wyoming. U.S. Geological Survey Professional Paper 1864, 57 p., Accessed November 28, 2021 from https://pubs.usgs.gov/pp/1864/pp1864.pdf	Miller, J.A., Appel, C.L. (1997). Ground Water Atlas of the United States: Segment 3, Kansas, Missouri, Nebraska. U.S. Geological Survey Hydrologic Atlas 730-D, 26 pp. Accessed April 13, 2021 from https://pubs.usgs.gov/ha/730d/report.pdf	Gutentag, E.D., Heimes, F.J., Krothe, N.C., Luckey, R.R., Weeks, J.B. (1984). Geohydrology of the High Plains aquifer in parts of Colorado, Kansas, Nebraska, New Mexico, Oklahoma, South Dakota, Texas, and Wyoming (No. 1400-B). Accessed February 10, 2021 from https://pubs.usgs.gov/pp/1400b/report.pdf	Approximated from Fig. 7 of Peterson et al. (2020), Fig. 46 by Miller and Appel (1997), and Fig. 1 by Gutentag et al. (1984)
**Northern Hirri Basin**	Chhattisgarh Basin	Central Ground Water Board (2012e). Aquifer Systems of Chhattisgarh. Central Ground Water Board Report, 73 pp. Accessed July 13, 2021 from	George, B.G., Ray, J.S., Kumar, S. (2019). Geochemistry of carbonate formations of the Chhattisgarh Supergroup, central India: implications for Mesoproterozoic global	Mukherjee, A., Ray, R. K., Tewari, D., Ingle, V. K., Sahoo, B. K., Khan, M. W. Y. (2014). Revisiting the stratigraphy of the Mesoproterozoic Chhattisgarh Supergroup,	Approximated from Plate IV by Central Ground Water Board (2012e), Fig. 1 by George et al. (2019), and Figs. 1 and 2 by Mukherjee et al. (2014)

Aquifer system	Broader aquifer system	Reference A	Reference B	Reference C	Steps to delineate
		http://cgwb.gov.in/AQM/Chhattisgarh.pdf	events. Canadian Journal of Earth Sciences, 56, 335-346.	Bastar craton, India based on subsurface lithoinformation. Journal of Earth System Science, 123, 617-632.	
**Northern Hosein Abad Basin**	-	Hosseini, S. M., Parizi, E., Ataie-Ashtiani, B., Simmons, C. T. (2019). Assessment of sustainable groundwater resources management using integrated environmental index: Case studies across Iran. Science of the Total Environment, 676, 792-810.	-	-	Approximated from Fig. 6 by Hosseini et al. (2019)
**Northern Hualien-Taitung Valley**	Hualien-Taitung Valley	Yeh, H. F., Lin, H. I., Lee, C. H., Hsu, K. C., Wu, C. S. (2014). Identifying seasonal groundwater recharge using environmental stable isotopes. Water, 6, 2849-2861.	Hsu, S. K. (1998). Plan for a groundwater monitoring network in Taiwan. Hydrogeology Journal, 6, 405-415.	-	Approximated from Fig. 1 by Yeh et al. (2014), Fig. 2 by Hsu et al. (1998); subarea boundary based on drainage
**Northern Jalpa-Juchipila Aquifer**	-	CONAGUA (2021a). Acuíferos (nacional). Webpage accessed March 8, 2022 from http://sina.conagua.gob.mx/sina/tema.php?tema=acuiferos&ver=mapa&o=1&n=nacional	-	-	Approximated from aquifer boundaries defined by CONAGUA (2021a) web portal, physiography and drainage patterns, and well completion data.
**Northern Karoo Basin**	Main Karoo Basin	Nyambe, I. A. (1999). Tectonic and climatic controls on sedimentation during deposition of the Sinakumbe Group and Karoo Supergroup, in the mid-Zambezi Valley Basin, southern Zambia. Journal of	-	-	Broader aquifer system boundary (i.e., Main Karoo Basin) approximated from Fig. 1 by Nyambe (1999). Physiography (drainage patterns, geology) and well construction data were considered while digitizing subarea

Aquifer system	Broader aquifer system	Reference A	Reference B	Reference C	Steps to delineate
		African Earth Sciences, 28, 443-463.			
**Northern Kern Basin**	Tulare Basin of the Central Valley **	California Department of Water Resources (2015c). California's groundwater update 2013 - Chapter 9: Tulare Lake Hydrologic Region. California Department of Water Resources Report, 80 pp. Accessed April 27, 2022 via https://data.cnra.ca.gov/dataset/california-water-plan-groundwater-update-2013/resource/8a4ae915-b786-42e1-9abe-99a8fcc23349	-	-	Approximated from Fig. 9-2 by California Department of Water Resources (2015c). Subdivided from Southern Kern Basin subarea on the basis of the Kern River
**Northern Kettleman Plain**	-	California Department of Water Resources (2015c). California's groundwater update 2013 - Chapter 9: Tulare Lake Hydrologic Region. California Department of Water Resources Report, 80 pp. Accessed April 27, 2022 via https://data.cnra.ca.gov/dataset/california-water-plan-groundwater-update-2013/resource/8a4ae915-b786-42e1-9abe-99a8fcc23349	-	-	Approximated from Fig. 9-2 by California Department of Water Resources (2015c)
**Northern Khorat Plateau**	Khorat Plateau Aquifer System	LaMoreaux, P. E., Charaljavanaphet, J., Jalichan, N., Chiengmai, P. P. N., Bunnag, D., Thavisri, A., Rakprathum, C. (1958). Reconnaissance of the geology and ground water of the Khorat Plateau, Thailand. US Geological	Phiancharoen, C. (1962). Interpretation of the chemical analyses of the ground water of the Khorat Plateau, Thailand. MSc Thesis, University of Arizona, 177 pp. Accessed November 4, 2021 from	-	Approximated from Plate 4 by LaMoreaux et al. (1958) and Plate 1 by Phiancharoen (1962). Subarea boundaries defined on the basis of physigraphy (e.g., watershed divides), drainage and well drilling data

Aquifer system	Broader aquifer system	Reference A	Reference B	Reference C	Steps to delineate
		Survey Water Supply Paper 1429, 76 pp. Accessed November 4, 2021 from https://pubs.usgs.gov/wsp/1429/report.pdf	https://repository.arizona.edu/handle/10150/191432		
**Northern Laoha River Basin**	Laoha River Basin	Hao, L., Sun, G., Liu, Y., Qian, H. (2015). Integrated modeling of water supply and demand under management options and climate change scenarios in Chifeng City, China. JAWRA Journal of the American Water Resources Association, 51, 655-671.	-	-	Approximated from Fig. 4 by Hao et al. (2015)
**Northern Littoral Plain**	North China Plain	Meng, S., Liu, J., Zhang, Z., Lei, T., Qian, Y., Li, Y., Fei, Y. (2015). Spatiotemporal evolution characteristics study on the precipitation infiltration recharge over the past 50 years in the North China Plain. Journal of Earth Science, 26, 416-424.	Cao, G., Zheng, C., Scanlon, B. R., Liu, J., & Li, W. (2013). Use of flow modeling to assess sustainability of groundwater resources in the North China Plain. Water Resources Research, 49, 159-175.	Wang, S., Song, X., Wang, Q., Xiao, G., Liu, C., Liu, J. (2009). Shallow groundwater dynamics in North China plain. Journal of Geographical Sciences, 19, 175-188.	Approximated from Fig. 1 by Meng et al. (2015), Fig. 1 by Cao et al. (2013) and Fig. 6 by Wang et al. (2009)
**Northern Malabar Khondalite and Charnockite Aquifers**	Malabar Coastal Plain	Central Ground Water Board (2012a). Aquifer Systems of Kerala. Central Ground Water Board Report, 72 pp. Accessed June 23, 2021 from http://cgwb.gov.in/AQM/Kerala.pdf	Varma, A. (2017). Groundwater Resource and Governance in Kerala, Status, Issues and Prospects. Forum for Policy Dialogue on Water Conflicts in India, Pune, Maharashtra, India. Kerala Resource Centre Report, 118 pp. Accessed June 23, 2021 from https://www.soppecom.org/pdf/Groundwater-Resource-and-Governance-in-Kerala.pdf	-	Approximated from Plate III by Central Ground Water Board (2012a). Easternmost margin of Malabar Coastal Plain approximated along the ~76m topographic contour as detailed for the "lowland" and "midland" physiographic zones in Fig. 1.2 by Varma (2017)

Aquifer system	Broader aquifer system	Reference A	Reference B	Reference C	Steps to delineate
**Northern Mallee**	Murray Basin	Herczeg, A. L., Dogramaci, S. S., Leaney, F. W. J. (2001). Origin of dissolved salts in a large, semi-arid groundwater system: Murray Basin, Australia. Marine and Freshwater Research, 52, 41-52.	Cook, P. G., Jolly, I. D., Leaney, F. W. J. (2001). Groundwater recharge in the Mallee region, and salinity implications for the Murray River: A review. CSIRO Land and Water Report, 133 pp. Accessed July 21, 2021 from https://publications.csiro.au/rpr/download?pid=procite:ef08494d-43a2-4dae-bda4-3d72a62e673f&dsid=DS1	Murray-Darling Basin Commission (1999). Murray-Darling Basin Groundwater - a resource for the future, Murray-Darling Basin Commission, Canberra. Accessed July 21, 2021 from https://www.mdba.gov.au/sites/default/files/archived/mdbc-GW-reports/2173_GW_a_resource_for_the_future.pdf	Approximated from Fig. 1 by Herczeg et al. (2001), Fig. 1.1 by Cook et al. (2001), and Fig. 6 by Murray-Darling Basin Commission (1999)
**Northern Maputaland Coastal Plain**	Maputaland Coastal Plain	Mthembu, P. P., Elumalai, V., Brindha, K., Li, P. (2020). Hydrogeochemical processes and trace metal contamination in groundwater: impact on human health in the Maputaland coastal aquifer, South Africa. Exposure and Health, 12(3), 403-426.	-	-	Approximated from Fig. 1 by Mthembu et al. (2020)
**Northern Massa Basin**	Souss-Chtouka Basin	Garduño, H., Foster, S. (2010). Sustainable Groundwater Irrigation. Approaches to Reconciling Demand with Resources. GW•MATE Strategic Overview Series No. 4 World Bank (Washington DC, USA). Accessed 05/01/2023 via https://openknowledge.worldbank.org/server/api/core/bitstreams/a6957092-3680-52cd-9707-91143c386175/content	Tagma, T., Hsissou, Y., Bouchaou, L., Bouragba, L., Boutaleb, S. (2009). Groundwater nitrate pollution in Souss-Massa basin (south-west Morocco). African Journal of Environmental Science and Technology, 3, 301-309.	Bouchaou, L., Michelot, J.L., Vengosh, A., Hsissou, Y., Qurtobi, M., Gaye, C.B., Bullen, T.D., Zuppi, G. M. (2008). Application of multiple isotopic and geochemical tracers for investigation of recharge, salinization, and residence time of water in the Souss–Massa aquifer, southwest of Morocco. Journal of Hydrology, 352, 267-287.	Broader aquifer system boundaries approximated from Box 5 by Garduño and Foster (2010), Fig. 1 by Bouchaou et al. (2008), and Fig. 1 by Tagma et al. (2009). Southern limit approximated along the Massa River.

Aquifer system	Broader aquifer system	Reference A	Reference B	Reference C	Steps to delineate
**Northern Mega Aquifer System**	Mega Aquifer System	Awadh, S. M., Al-Mimar, H., Yaseen, Z. M. (2020). Groundwater availability and water demand sustainability over the upper mega aquifers of Arabian Peninsula and west region of Iraq. Environment, Development and Sustainability, 1-21.	Abotalib, A. Z., Heggy, E., Scabbia, G., Mazzoni, A. (2019). Groundwater dynamics in fossil fractured carbonate aquifers in Eastern Arabian Peninsula: a preliminary investigation. Journal of Hydrology, 571, 460-470.	-	Approximated from Fig. 1 by Awadh et al. (2020) and Fig. 1 by Abotalib et al. (2019)
**Northern Nallamalai Basin**	Cuddapah Sedimentary Basin	Dar, F. A., Perrin, J., Riotte, J., Gebauer, H. D., Narayana, A. C., Shakeel, A. (2011). Karstification in the Cuddapah Sedimentary Basin, southern India: implications for groundwater resources. Acta Carsologica, 40, 457-472.	Saha, D., Ray, R. K. (2019). Groundwater resources of India: potential, challenges and management. In Groundwater Development and Management. Springer, Cham (ed. Sikdar, P.K.), pp. 19-42.	Chalapathi Rao, N. V., Gibson, S. A., Pyle, D. M., Dickin, A. P. (2004). Petrogenesis of Proterozoic lamproites and kimberlites from the Cuddapah Basin and Dharwar craton, southern India. Journal of Petrology, 45, 907-948.	Approximated from Fig. 1 by Dar et al. (2011), Fig. 2.2 by Saha and Ray (2019), and Fig. 1 by Chalapathi Rao et al. (2004)
**Northern Nuomin-Taoer System**	Songnen Plain	Chen, Z., Wei, W., Liu, J., Wang, Y., Chen, J. (2011). Identifying the recharge sources and age of groundwater in the Songnen Plain (Northeast China) using environmental isotopes. Hydrogeology Journal, 19, 163-176.	Zhang, G., Deng, W., Yang, Y. S., Salama, R. B. (2007). Evolution study of a regional groundwater system using hydrochemistry and stable isotopes in Songnen Plain, northeast China. Hydrological Processes: An International Journal, 21, 1055-1065.	Zhang, B., Song, X.-F., Zhang, Y.-H., Han, D.-M., Tand, C.Y., Yang, L.-H., Wang, Z. L. (2017). The renewability and quality of shallow groundwater in Sanjiang and Songnen Plain, Northeast China. Journal ofIntegrative Agriculture, 16, 229-238.	Approximated from Fig. 1 by Chen et al. (2011), Fig. 1 by Zhang et al. (2007) and Fig. 1 by Zhang et al. (2017)
**Northern Paris Basin**	Paris Basin	Beaudoin, N., Gasparrini, M., David, M. E., Lacombe, O., Koehn, D. (2019). Bedding-parallel stylolites as a tool to unravel maximum burial depth in sedimentary basins: Application to Middle Jurassic carbonate reservoirs in the Paris	Contoux, C., Violette, S., Vivona, R., Goblet, P., Patriarche, D. (2013). How basin model results enable the study of multi-layer aquifer response to pumping: the Paris Basin, France. Hydrogeology Journal, 21, 545-557.	Beccaletto, L., Hanot, F., Serrano, O., Marc, S. (2011). Overview of the subsurface structural pattern of the Paris Basin (France): Insights from the reprocessing and interpretation of regional seismic lines. Marine and	Approximated from Fig. 1 by Beaudoin et al. (2019), Fig. 1 by Contoux et al. (2013), and Fig. 1 by Beccaletto et al. (2011)

Aquifer system	Broader aquifer system	Reference A	Reference B	Reference C	Steps to delineate
		basin, France. GSA Bulletin, 131, 1239-1254.		Petroleum Geology, 28, 861-879.	
**Northern Piedmont and Tista Fan**	Bengal Basin	Woodman, N. D., Burgess, W. G., Ahmed, K. M., Zahid, A. (2019). A partially coupled hydro-mechanical analysis of the Bengal Aquifer System under hydrological loading. Hydrology and Earth System Sciences, 23, 2461-2479.	Ravenscroft, P., McArthur, J. M., Rahman, M. S. (2018). Identifying multiple deep aquifers in the Bengal Basin: Implications for resource management. Hydrological Processes, 32, 3615-3632.	Michael, H. A., Voss, C. I. (2009). Controls on groundwater flow in the Bengal Basin of India and Bangladesh: regional modeling analysis. Hydrogeology Journal, 17, 1561-1577.	Approximated from Fig. 1 by Woodman et al. (2019), Fig. 1 by Ravenscroft et al. (2018), and Fig. 1 by Michael and Voss (2009)
**Northern Piedmont Plain**	North China Plain	Meng, S., Liu, J., Zhang, Z., Lei, T., Qian, Y., Li, Y., Fei, Y. (2015). Spatiotemporal evolution characteristics study on the precipitation infiltration recharge over the past 50 years in the North China Plain. Journal of Earth Science, 26, 416-424.	Cao, G., Zheng, C., Scanlon, B. R., Liu, J., & Li, W. (2013). Use of flow modeling to assess sustainability of groundwater resources in the North China Plain. Water Resources Research, 49, 159-175.	Wang, S., Song, X., Wang, Q., Xiao, G., Liu, C., Liu, J. (2009). Shallow groundwater dynamics in North China plain. Journal of Geographical Sciences, 19, 175-188.	Approximated from Fig. 1 by Meng et al. (2015), Fig. 1 by Cao et al. (2013) and Fig. 6 by Wang et al. (2009).
**Northern Piedmont Upland**	Piedmont Plateau	Miller, J.A. (1990). Ground Water Atlas of the United States: Segment 6, Alabama, Florida, Georgia, South Carolina. U.S. Geological Survey Hydrologic Atlas 730-G, 30 pp. Accessed April 5, 2021 from https://www.nrc.gov/docs/ML1706/ML17060B027.pdf	-	-	Approximated from Fig. 3 of Miller (1990).
**Northern Pitted Karst Plain**	Yucatan Peninsula	Bauer-Gottwein, P., Gondwe, B. R., Charvet, G., Marín, L. E., Rebolledo-Vieyra, M., Merediz-Alonso, G. (2011). the Yucatán Peninsula karst aquifer, Mexico. Hydrogeology Journal, 19, 507-524.	Munro-Stasiuk, M.J., Manahan, T. K. (2010). Investigating ancient Maya agricultural adaptation through ground penetrating radar (GPR) analysis ofkarst terrain, Northern	-	Approximated from Fig. 2 by Bauer-Gottwein et al. (2011), and from Fig. 2 by Munro-Stasiuk and Manahan (2010).

Aquifer system	Broader aquifer system	Reference A	Reference B	Reference C	Steps to delineate
			Yucatán, Mexico. Acta Carsologica, 39, 123-36.		
**Northern Plane Basin**	-	Great Barrier Reef Marine Park Authority (2013). Plane Basin Assessment. Mackay Whitsunday Natural Resource Management Region Report, 121 pp. Accessed May 1, 2023 via https://elibrary.gbrmpa.gov.au/jspui/bitstream/11017/2902/2/Plane-Basin-assessment-2013.pdf	-	-	Approximated from Fig. 2 by Great Barrier Reef Marine Park Authority (2013)
**Northern Qazvin Plain**	Qazvin Plain	Yazdi, Z., Niroumand, H. (2020). Assessing land subsidence in Qazvin plain caused by groundwater level drop, using finite elements and finite difference methods. GeoTerrace-2020-043, 5 pp. Accessed June 2, 2021 from https://eage.in.ua/wp-content/uploads/2020/12/GeoTerrace-2020-043.pdf	Faghihi, N., Kave, F., Babazadeh, H. (2010). Prediction of aquifer reaction to different hydrological and management scenarios using visual MODFLOW model-Case study of Qazvin plain. Journal of Water Sciences Research 2, 39-45.	Babaee, S., Mousavi, Z., Masoumi, Z., Malekshah, A. H., Roostaei, M., Aflaki, M. (2020). Land subsidence from interferometric SAR and groundwater patterns in the Qazvin plain, Iran. International Journal of Remote Sensing, 41, 4780-4798.	Approximated from Fig. 1 by Yazdi and Niroumand (2020), with zones approximated from Fig. 7 by Faghihi et al. (2010)
**Northern Rayong Basin**	Rayong Basin	Boonkaewwan, S., Sonthiphand, P., Chotpantarat, S. (2021). Mechanisms of arsenic contamination associated with hydrochemical characteristics in coastal alluvial aquifers using multivariate statistical technique and hydrogeochemical modeling: a case study in Rayong province, eastern Thailand. Environmental	Piyapong, J., Thidarat, B., Jaruwan, C., Siriphan, N., Passanan, A. (2019). Enhancing citizens' sense of personal responsibility and risk perception for promoting public participation in sustainable groundwater resource management in Rayong Groundwater Basin, Thailand. Groundwater for Sustainable Development, 9, 100252.	-	Approximated from Fig. 1 by Boonkaewwan et al. (2021), Fig. 2 by Piyapong et al. (2019). Subareas delineated on the basis of physiography, drainage and groundwater well prevelance

Aquifer system	Broader aquifer system	Reference A	Reference B	Reference C	Steps to delineate
		Geochemistry and Health, 43, 537-566.			
**Northern Razan Plain**	Razan Basin	Pazand, K. (2016). Geochemical and statistical evaluation of groundwater in Razan basin, Western Iran. Carbonates and Evaporites, 31, 179-185.	Fallahi, M. M., Shabanlou, S., Rajabi, A., Yosefvand, F., IzadBakhsh, M. A. (2023). Effects of climate change on groundwater level variations affected by uncertainty (case study: Razan aquifer). Applied Water Science, 13, 1-16.	-	Approximated from Fig. 1 by Pazand (2016) and Fig. 1 by Fallahi et al. (2023)
**Northern Riverine Plains**	Murray Basin	Herczeg, A. L., Dogramaci, S. S., Leaney, F. W. J. (2001). Origin of dissolved salts in a large, semi-arid groundwater system: Murray Basin, Australia. Marine and Freshwater Research, 52, 41-52.	Barnett, S., Simmons, C. T., Nelson, R. (2021). Groundwater resources in Australia—their occurrence, management, and future challenges. In: Global Groundwater Source, Scarcity, Sustainability, Security, and Solutions (eds. Mukherjee, A., Scanlon, B.R., Aureli, A., Langan, S., Guo, H., McKenzie, A.), Elsevier, pp. 35-46.	Murray-Darling Basin Commission (1999). Murray-Darling Basin Groundwater - a resource for the future, Murray-Darling Basin Commission, Canberra. Accessed July 21, 2021 from https://www.mdba.gov.au/sites/default/files/archived/mdbc-GW-reports/2173_GW_a_resource_for_the_future.pdf	Approximated from Fig. 1 by Herczeg et al. (2001), Fig. 3.2 by Barnett et al. (2021), and Fig. 6 by Murray-Darling Basin Commission (1999)
**Northern San Juan Basin**	-	Kernodle, J. M. (1996). Hydrogeology and steady-state simulation of ground-water flow in the San Juan Basin, New Mexico, Colorado, Arizona, and Utah. US Geological Survey Water-Resources Investigations Report 95-4187, 126 pp. Accessed March 12, 2021 from https://pubs.usgs.gov/wri/1995/4187/report.pdf	Welder, G. E. (1986). Plan of study for the regional aquifer system analysis of the San Juan structural basin, New Mexico, Colorado, Arizona, and Utah. U.S. Geological Survey Water Resources Investigations Report 85-4294, 27 pp. Accessed April 14, 2021 from https://pubs.usgs.gov/wri/1985/4294/report.pdf	-	Approximated from Fig. 3 of Kernodle (1996) and Fig. 4 of Welder (1986). Southern portion approximated on the basis of groundwater well density (derived from New Mexico's well completion dataset).
**Northern Santiago Basin**	Santiago Basin	Araneda, M., Avendaño, M. S., Del Río, G. D. (2010). Modelo estructural de la	Steinbrügge, G., Muñoz Pardo, J. F., Fernández, B. (2005). Análisis	Briceño Aguirre, A.D. (2020). Funcionamiento hidrogeológico y geometría	Approximated from Figs. 2 and 7 by Araneda et al. (2010). Southern boundary boundary

Aquifer system	Broader aquifer system	Reference A	Reference B	Reference C	Steps to delineate
		cuenca de Santiago, Chile y su relación con la hidrogeología. Revista Geofísica, 62, 29-48.	probabilístico y optimización de los recursos de agua subterránea: el caso del acuífero Maipo-Mapocho, Chile. Ingenieria hidraulica en Mexico, XX, 85-97. Accessed May 15, 2021 from https://repositorio.uc.cl/xmlui/bitstream/handle/11534/28539/An%C3%A1lisis%20probabil%C3%ADstico%20y%20optimizaci%C3%B3n%20de%20los%20recursos%20de%20agua%20subterr%C3%A1nea%20el%20caso%20del%20acu%C3%ADfero%20Maipo-Mapocho,%20Chile.pdf?sequence=1	del acuífero del sector Norte y Centro de Santiago. Thesis Universidad de Chile, 454 pp. Accessed May 15, 2021 from http://repositorio.uchile.cl/handle/2250/176921	approximated along the Rio Mapocho. See also Illustration 3 by Steinbrügge et al. (2005) and Fig. 1 by Briceño Aguirre (2020).
**Northern Sarkhoon Aquifer**	Sarkhoon Aquifer	Bazrafshan, O., Parandin, F., Farokhzadeh, B. (2016). Assessment of hydro-meteorological drought effects on groundwater resources in Hormozgan region-South of Iran. Ecopersia, 4, 1569-1584.	-	-	Approximated from map on page 1571 of Bazrafshan et al. (2016)
**Northern Saurashtra Deccan Trap Basalts**	Saurashtra Deccan Trap Basalts	Das, S., Prakash, I. (2008). Assessment of groundwater hazards in a coastal district of Gujarat, India. Proceedings of the 6th conference on Case Histories in Geotechnical Engineering, 12 pp. Accessed June 22, 2021 from https://core.ac.uk/download/pdf/229070665.pdf	Shah, T. (2014). Towards a Managed Aquifer Recharge strategy for Gujarat, India: An economist's dialogue with hydro-geologists. Journal of Hydrology, 518, 94-107.	Gupte, P. R. (2012). Review of aquifer system of Deccan trap area, Gujarat state. Proceedings of the International Ground Water Congress, 19 pp.	Approximated from Figs. 7 and 8 by Das and Prakash (2008), Figs. 1, 2 and 4 by Shah (2014) and Fig. 1 by Gupte (2012)

Aquifer system	Broader aquifer system	Reference A	Reference B	Reference C	Steps to delineate
**Northern Shamil-Meymand Basin**	-	Hosseini, S. M., Parizi, E., Ataie-Ashtiani, B., Simmons, C. T. (2019). Assessment of sustainable groundwater resources management using integrated environmental index: Case studies across Iran. Science of the Total Environment, 676, 792-810.	-	-	Approximated from Fig. 6 by Hosseini et al. (2019)
**Northern Shuicheng Basin**	-	Li, X. D., Liu, C. Q., Harue, M., Li, S. L., Liu, X. L. (2010). The use of environmental isotopic (C, Sr, S) and hydrochemical tracers to characterize anthropogenic effects on karst groundwater quality: a case study of the Shuicheng Basin, SW China. Applied Geochemistry, 25, 1924-1936.	-	-	Approximated from Fig. 1 by Li et al. (2010) and local topographic features and drainage patterns
**Northern St Vincent Basin**	St Vincent Basin	Smith, M. L., Fontaine, K., Lewis, S. J. (2015). Regional Hydrogeological Characterisation of the St Vincent Basin, South Australia: Technical Report for the National Collaboration Framework Regional Hydrogeology Project. Geoscience Australia Record 2015/16, 150 pp. Accessed July 27, 2021 from https://d28rz98at9flks.cloudfront.net/78884/Rec2015_016.pdf	-	-	Approximated from Fig. 2.2 by Smith et al. (2015)

Aquifer system	Broader aquifer system	Reference A	Reference B	Reference C	Steps to delineate
**Northern St. Lawrence Lowlands**	St. Lawrence Lowlands	Parent, M., Rivard, C., Lefebvre, R., Carrier, M.-A., Séjourné, S. (2014). Hydrogeological systems of the Montérégie Est region, southern Québec: Fieldtrip Guidebook, GeoMontreal 2013 Conference, Geological Survey of Canada Open File 7605, 43 pp.	Olcott, P.G. (1995). Ground Water Atlas of the United States: Segment 12, Connecticut, Maine, Massachusetts, New Hampshire, New York, Rhode Island, Vermont. U.S. Geological Survey Hydrologic Atlas 730-M, 30 pp. Accessed April 14, 2021 from https://pubs.usgs.gov/ha/730m/report.pdf	Patenaude, M., Baudron, P., Labelle, L., Masse-Dufresne, J. (2020). Evaluating bank-filtration occurrence in the Province of Quebec (Canada) with a GIS approach. Water 12, 662.	St. Lawrence Lowlands hydrogeological context approximated from Fig. 2 of Parent et al. (2014; including the divide between northern and southern portions of the broader aquifer system) and Fig. 4 by Patenaude et al. (2020); see also Fig. 102 by Olcutt (1995).
**Northern Swan Coastal Plain**	Perth Basin	Government of Western Australia's Department of Water (2017). Northern Perth Basin: Geology, hydrogeology and groundwater resources. Department of Water Hydrological Bulletin Series Report No. HB1, 395 pp. Accessed July 25, 2021 from https://www.water.wa.gov.au/__data/assets/pdf_file/0011/8993/111005.pdf	Barron, O., Silberstein, R., Ali, R., Donohue, R., McFarlane, D.J., Davies, P., Hodgson, G., Smart, N. Donn, M., (2012). Climate change effects on water-dependent ecosystems in south-western Australia. Journal of Hydrology, 434, 95-109.	Ali, R., McFarlane, D., Varma, S., Dawes, W., Emelyanova, I., Hodgson, G., Charles, S. (2012). Potential climate change impacts on groundwater resources of south-western Australia. Journal of Hydrology, 475, 456-472.	Approximated from Fig. 1 by Barron et al. (2012), Fig. 1 by Ali et al. (2012), and Fig. 5 by Government of Western Australia's Department of Water (2017)
**Northern Taichung Basin**	Taichung Basin	Wang, S. J., Lee, C. H., Yeh, C. F., Choo, Y. F., Tseng, H. W. (2021). Evaluation of Climate Change Impact on Groundwater Recharge in Groundwater Regions in Taiwan. Water, 13, 1153.	Yeh, H. F. (2021). Spatiotemporal Variation of the Meteorological and Groundwater Droughts in Central Taiwan. Frontiers in Water, 3, 14.	-	Approximated from Fig. 2 by Wang et al. (2021), and Fig. 1 by Yeh (2021)
**Northern Tikapoo Valley**	-	Lopes, T.J., Evetts. D.M. (2005). Ground-Water pumpage and artificial recharge estimates for calendar year 2000 and average annual natural	Harrill, J.R., Prudic, D.E. (1998). Aquifer systems in the Great Basin region of Nevada, Utah, and adjacent states; summary report. US Geological Survey	-	Approximated from Fig. 1 by Lopes and Evetts (2005), and Fig. 2 by Harrill and Prudic (1998). Well completion records, physiography and drainage were used to delinate areas.

Aquifer system	Broader aquifer system	Reference A	Reference B	Reference C	Steps to delineate
		recharge and interbasin flow by hydrographic area, Nevada. Accessed April 25, 2022 via https://pubs.usgs.gov/sir/2004/5239/sir2004-5239.pdf	Professional Paper 1409-A, 75 pp. Accessed April 26, 2022 via https://pubs.usgs.gov/pp/1409a/report.pdf		
**Northern Tule Lake**	Upper Klamath Basin	Gannett, M.W., Breen, K.H. (2015). Groundwater levels, trends, and relations to pumping in the Bureau of Reclamation Klamath Project, Oregon and California: U.S. Geological Survey Open-File Report 2015-1145, 19 pp. Accessed March 20, 2021 from https://pubs.usgs.gov/of/2015/1145/ofr20151145.pdf	-	-	Approximated from Fig. 1 of Gannett and Breen (2015).
**Northern Valley and Ridge**	Valley and Ridge Aquifer System	Johnson, G.C., Zimmerman, T.M., Lindsey, B.D., Gross, E.L. (2011). Factors affecting groundwater quality in the Valley and Ridge aquifers, eastern United States, 1993–2002: U.S. Geological Survey Scientific Investigations Report 2011–5115, 84 pp. Accessed April 13, 2021 from https://pubs.usgs.gov/sir/2011/5115/support/sir2011-5115.pdf	-	-	Southern boundary approximated at Deep Creek Lake and North Fork Shenandoah River. Broader Valley and Ridge Aquifer System outline approximated from Fig. 1 of Johnson et al. (2011).
**Northern Vienna Basin**	Vienna Basin	Kováč, M. I. C. H. A. L., Sliva, L., Sopkova, B., Hlavata, J., & Škulová, A. D. R. I. A. N. A. (2008). Serravallian sequence stratigraphy of the northern	-	-	Approximated from Fig. 1 by Kováč et al. (2008)

Aquifer system	Broader aquifer system	Reference A	Reference B	Reference C	Steps to delineate
		Vienna Basin: high frequency cycles in the Sarmatian sedimentary record. Geologica Carpathica, 59, 545-561.			
**Northwest Coimbatore Fractured Charnockite and Gneiss Aquifers**	Coimbatore Upland	Sajil Kumar, P. J., James, E. J. (2016). Identification of hydrogeochemical processes in the Coimbatore district, Tamil Nadu, India. Hydrological Sciences Journal, 61(4), 719-731.	Saha, D., Shekhar, S., Ali, S., Vittala, S.S., Raju, N.J. (2016). Recent hydrogeological research in India. Proceedings of the Indian National Science Academy, 82, 787-803.	-	Approximated from Fig. 1 by Sajil Kumar and James (2016), and Fig. 1 by Saha et al. (2016)
**Northwest Jaipur Alluvium and Mendha Basin**	Malwa Plateau	Machiwal, D., Islam, A., Kamble, T. (2019). Trends and probabilistic stability index for evaluating groundwater quality: The case of quaternary alluvial and quartzite aquifer system of India. Journal of Environmental Management, 237, 457-475.	Central Ground Water Board (2013a). Ground water information Jaipur District, Rajasthan. Central Ground Water Board Report, 19 pp. Accessed July 12, 2021 from http://cgwb.gov.in/District_Profile/Rajasthan/Jaipur.pdf	Rathore, V. S., Nathawat, M. S., Ray, P. C. (2008). Influence of neotectonic activity on groundwater salinity and playa development in the Mendha river catchment, western India. International Journal of Remote Sensing, 29, 3975-3986.	Approximated from Machiwal et al. (2019), Fig. 2 by Central Ground Water Board (2013a) and Fig. 1 by Rathore et al. (2008)
**Northwestern Basal Cretaceous Aquifer**	Bohemian Cretaceous Basin	Alvarado, J. C., Pačes, T., Purtschert, R. (2013). Dating groundwater in the Bohemian Cretaceous Basin: Understanding tracer variations in the subsurface. Applied Geochemistry, 29, 189-198.	Jiráková, H., Huneau, F., Hrkal, Z., Celle-Jeanton, H., Le Coustumer, P. (2010). Carbon isotopes to constrain the origin and circulation pattern of groundwater in the north-western part of the Bohemian Cretaceous Basin (Czech Republic). Applied Geochemistry, 25, 1265-1279.	Kadlecová, R., Olmer, M. (2011). Review of Groundwater Resources. Geologické výzkumy na Moravě a ve Slezsku. Proceedings of The Open Congress ČGS and SGS, September 22–25, 2011, Monínec 18, pp. 31-34. Accessed October 21, 2021 from https://journals.muni.cz/gvms/article/view/1679	Approximated from Fig. 1 by Jiráková et al. (2010), Fig. 1 by Alvarado et al. (2013), and Fig. 1 by Kadlecová and Olmer (2011)
**Northwestern Deccan Basalt Plateau**	Deccan Plateau	Deolankar, S. B. (1980). The Deccan basalts of Maharashtra, India—their potential as aquifers.	Kale, V. S., Bodas, M., Chatterjee, P., Pande, K. (2020). Emplacement history and evolution of the	Duraiswami, R. A., Das, S., Shaikh, T. (2012). Hydrogeological framework of aquifers from the Deccan	Approximated from Fig. 1 by Deolankar (1980), Fig. 1 by Duraiswami et al. (2012), and Fig. 1 by Kale et al. (2016). Subarea

Aquifer system	Broader aquifer system	Reference A	Reference B	Reference C	Steps to delineate
		Groundwater, 18(5), 434-437.	Deccan Volcanic Province, India. Episodes, 43, 278-299.	Traps, India: some insights. Memoir Geological Society of India, 1-15.	based on topography and drainage patterns.
**Northwestern Kanto Plain**	Kanto Plain	Furuno, K., Kagawa, A., Kazaoka, O., Kusuda, T., Nirei, H. (2015). Groundwater management based on monitoring of land subsidence and groundwater levels in the Kanto Groundwater Basin, Central Japan. Proceedings of the International Association of Hydrological Sciences, 372, 53-57.	Tanaka, T. (2004). Groundwater resources, development and management in the Kanto Plain, Japan. In Satellite Symposium, 5 pp. Accessed April 14, 2023 via https://core.ac.uk/download/pdf/76125416.pdf	Miyakoshi, A., Uchida, Y., Sakura, Y., Hayashi, T. (2003). Distribution of subsurface temperature in the Kanto Plain, Japan; estimation of regional groundwater flow system and surface warming. Physics and Chemistry of the Earth, Parts A/B/C, 28, 467-475.	Approximated from Fig. 2 by Furuno et al. (2015), Fig. 1 by Tanaka (2004), and Fig. 1 by Miyakoshi et al. (2003) and physiography and drainage patterns
**Northwestern Karnataka Schist Aquifers**	-	Central Ground Water Board (2012c). Aquifer Systems of Karnataka. Central Ground Water Board Report, 75 pp. Accessed June 30, 2021 from http://cgwb.gov.in/AQM/Karnataka.pdf	Central Ground Water Board (2008a). Ground water information booklet Haveri District, Karnataka. Central Ground Water Board Report, 21 pp. Accessed July 3, 2021 from http://cgwb.gov.in/District_Profile/karnataka/Haveri_brochure.pdf	Central Ground Water Board (2008b). Ground water information booklet Dharwad District, Karnataka. Central Ground Water Board Report, 18 pp. Accessed July 3, 2021 from http://cgwb.gov.in/District_Profile/karnataka/DHARWAD_BROCHURE.pdf	Approximated from Fig. 3 by Central Ground Water Board (2008a), Fig. 3 by Central Ground Water Board (2008b), and Plate III by Central Ground Water Board (2012c). Division between Northwestern and Western Karnataka Schist Aquifers approximated on the basis of drainage and topography data
**Northwestern Malwa Plateau**	Malwa Plateau	Deolankar, S. B. (1980). The Deccan basalts of Maharashtra, India—their potential as aquifers. Groundwater, 18(5), 434-437.	Kale, V. S., Bodas, M., Chatterjee, P., Pande, K. (2020). Emplacement history and evolution of the Deccan Volcanic Province, India. Episodes, 43, 278-299.	Duraiswami, R. A., Das, S., Shaikh, T. (2012). Hydrogeological framework of aquifers from the Deccan Traps, India: some insights. Memoir Geological Society of India, 1-15.	Approximated from Fig. 1 by Deolankar (1980), Fig. 1 by Duraiswami et al. (2012), and Fig. 1 by Kale et al. (2016). Subarea based on topography and drainage patterns.
**Nosrat Abad Basin**	-	Hosseini, S. M., Parizi, E., Ataie-Ashtiani, B., Simmons, C. T. (2019). Assessment of sustainable groundwater resources management using integrated environmental index: Case studies across Iran. Science of the Total	-	-	Approximated from Fig. 6 by Hosseini et al. (2019)

Aquifer system	Broader aquifer system	Reference A	Reference B	Reference C	Steps to delineate
		Environment, 676, 792-810.			
**Nurabad Mamasani Plain Aquifer**	-	Negarash, H., Shafiei, N., Doraninejad, M. S. (2016). Hydro-geomorphology Effect of Nurabad Mamasani Plain Aquifer on the Region's Water Resources Using GIS. Hydrogeomorphology, 2, 55-73.	-	-	Approximated from Fig. 1 by Negarash et al. (2016)
**Oak Ridges Moraine**	-	Gerber, R. E., Howard, K. (2002). Hydrogeology of the Oak Ridges Moraine aquifer system: implications for protection and management from the Duffins Creek watershed. Canadian Journal of Earth Sciences, 39(9), 1333-1348.	Marchildon, M., Kassenaar, D. (2013). Analyzing low impact development strategies using continuous fully distributed coupled groundwater and surface water models. Journal of Water Management Modeling. R246-17, doi:10.14796/JWMM.R246-17.	-	Approximated from Figs. 1 and 2 by Gerber and Howard (2002), and Fig. 17.4 by Marchildon and Kassenaar (2013)
**Ocala Uplift**	Floridan Aquifer System	Davis, H. (1996). Hydrogeologic Investigation and Simulation of Ground-Water Flow in the Upper Floridan Aquifer of North-Central Florida and Southwestern Georgia and Delineation of Contributing Areas for Selected City of Tallahassee, Florida, Water-Supply Wells. U.S. Geological Survey Water-Resources investigations Report 95-4296, 61 pp. Accessed March 31, 2021 from https://fl.water.usgs.gov/PDF_files/wri95_4296_davis.pdf	Clark, W. Z., Zisa, A. C. (1976) Physiographic map of Georgia: Georgia Department of Natural Resources, 1 sheet. Accessed March 31, 2021 from https://epd.georgia.gov/document/publication/sm-4-physiographic-map-georgia-12000000-1988/download	Brooks, H. K. (1981). Physiographic divisions of Florida. Report for the Florida Cooperative Extension Service Institute of Food and Agricultural Sciences, University of Florida, 12 pp.	Approximated from Fig. 2 of Davis (1996) and the physiographic provinces for Georgia and Florida by Clark and Zisa (1976) and Brooks (1981)

Aquifer system	Broader aquifer system	Reference A	Reference B	Reference C	Steps to delineate
**Odessa Subregion**	Columbia Plateau Regional Aquifer System	United States Bureau of Reclamation (2012). Final Feasibility-Level Special Study Report Odessa Subarea Special Study. 244 pp. Accessed February 18, 2021 from https://www.usbr.gov/pn/programs/eis/odessa/finaleis/final.pdf	Kahle, S.C., Morgan, D.S., Welch, W.B., Ely, S.R., Vaccaro, J.J., Orzol, L.L. (2011). Hydrogeologic Framework and Hydrologic Budget Components of the Columbia Plateau Regional Aquifer System, Washington, Oregon, and Idaho. US Geological Survey Scientific Investigations Report 2011–5124, 80 pp. Accessed February 16, 2021 from https://pubs.usgs.gov/sir/2011/5124/pdf/sir20115124.pdf	Vaccaro, J.J., Kahle, S.C., Ely, D.M., Burns, E.R., Snyder, D.T., Haynes, J.V., Olsen, T.D., Welch, W.B., Morgan, D.S. (2015). Groundwater availability of the Columbia Plateau Regional Aquifer System, Washington, Oregon, and Idaho: U.S. Geological Survey Professional Paper 1817, 87 pp. Accessed February 18, 2021 from http://dx.doi.org/10.3133/pp1817.	Odessa subarea approximated from location map on page 75 (Fig. 4-10) of United States Bureau of Reclamation (2012) and Fig. 38 of Vaccaro et al. (2015). Broader Columbia River Aquifer System approximated from Figs. 1 and 10 of Kahle et al. (2011).
**Odra Gate**	-	Kadlecová, R., Olmer, M. (2011). Review of Groundwater Resources. Geologické výzkumy na Moravě a ve Slezsku. Proceedings of The Open Congress ČGS and SGS, September 22–25, 2011, Monínec 18, pp. 31-34. Accessed October 21, 2021 from https://journals.muni.cz/gvms/article/view/1679	-	-	Approximated from Fig. 2 by Kadlecová and Olmer (2011)
**Ogden Valley**	-	Jordan, J. L., Smith, S.D., Inkenbrandt, P.C., Lowe, M., Hardwick, C.L., Wallace, J., Kirby, S.M., King, J.K., Payne, E.E. (2019). Characterization of the groundwater system in Ogden Valley, Weber County, Utah, with emphasis on groundwater–	-	-	Approximated from Fig. 1 of Dustin (1978)

Aquifer system	Broader aquifer system	Reference A	Reference B	Reference C	Steps to delineate
		surface-water interaction and the groundwater budget. Utah Geological Survey Report, Special Study 165. 237 pp. Accessed March 27, 2021 from https://ugspub.nr.utah.gov/publications/special_studies/ss-165/ss-165.pdf			
**Oja Alluvial Aquifers**	-	Martínez-Bastida, J. J., Araúzo, M., Valladolid, M. (2007). Caracterización hidroquímica de las aguas superficiales y subterráneas en la cuenca del Oja-Tirón. Procesos de contaminación. Limnetica, 26, 219-232. Accessed May 1, 2023 via https://ddd.uab.cat/pub/limnetica/02138409v26n2/02138409v26n2p219.pdf	-	-	Approximated from Fig. 1 by Martínez-Bastida et al. (2007)
**Ojai Basin**	-	Manz, R.P. (1988). Groundwater flow modeling of the Ojai basin using the USGS 3 dimensional MODFLOW model. MSc Thesis, California State University, Northridge, 195 pp. Accessed January 31, 2022 from https://scholarworks.csun.edu/bitstream/handle/10211.3/132443/ManzRichard1988.pdf?sequence=1	-	-	Approximated from Fig. 3-2 by Manz (1988)
**Ojocaliente Aquifer**	-	CONAGUA (2020jj). Actualización de la disponibilidad media anual de agua en el acuífero Ojocaliente (3212), Estado de Zacatecas. 38 pp.	-	-	Approximated from topography and from Fig. 1 by CONAGUA (2020jj)

Aquifer system	Broader aquifer system	Reference A	Reference B	Reference C	Steps to delineate
		Accessed March 7, 2022 via https://sigagis.conagua.gob.mx/gas1/Edos_Acuiferos_18/zacatecas/DR_3212.pdf			
**Ojos Negros Basin**	-	Morín, P.M. (2013). Aplicación de un modelo numérico para simular el flujo hidráulico del acuífero de Ojos Negros. M.Sc. Thesis, Ensenada Center for Scientific Research and Higher Education, 93 pp. Accessed May 6, 2021 from https://cicese.repositorioinstitucional.mx/jspui/bitstream/1007/909/1/192051.pdf	Ponce, V. M., Pandey, R. P., Kumar, S. (1999). Groundwater recharge by channel infiltration in El Barbon basin, Baja California, Mexico. Journal of Hydrology, 214, 1-7.	-	Approximated from Fig. 1 of Morín (2013) and Fig. 1 by Ponce et al. (1999)
**Okanagan Lake Perimeter (west)**	Okanagan Basin	Golder Associates and Summit Environmental Consultants Ltd. (2009). Phase 2 Okanagan Water Supply and Demand Project: Groundwater Objectives 2 and 3 Basin Study.Report to Okanagan Basin Water Board, 114 pp. Accessed March 16, 2021 from https://www.obwb.ca/wsd/about/project-reports	-	-	Approximated from Fig. 7.1 of report by Golder Associates and Summit Environmental Consultants Ltd. (2009). Multiple lake perimeter aquifers shown in figure were stitched together and entitled 'Lake Perimeter' aquifers
**Okefenokee Basin**	Floridan Aquifer System	Williams, L.J., Kuniansky, E.L. (2016). Revised hydrogeologic framework of the Floridan aquifer system in Florida and parts of Georgia, Alabama, and South Carolina. U.S. Geological Survey Professional Paper 1807, 140 pp. Accessed March 31, 2021 from	Clark, W. Z., Zisa, A. C. (1976) Physiographic map of Georgia: Georgia Department of Natural Resources, 1 sheet. Accessed March 31, 2021 from https://epd.georgia.gov/document/publication/sm-4-physiographic-map-georgia-12000000-1988/download	-	Approximated from physiographic provinces of Georgia outlined by Clark and Zisa (1976). Broader Florida Aquifer System from Williams and Kuniansky (2016).

Aquifer system	Broader aquifer system	Reference A	Reference B	Reference C	Steps to delineate
		https://pubs.usgs.gov/pp/1807/pdf/pp1807.pdf			
**Oliver Sub-basin**	Okanagan Basin	Neilson-Welch, L., Allen, D. (2007). Groundwater and hydrogeological conditions in the Okanagan Basin, British Columbia: a state-of-the-basin report. Final report prepared for Objective 1 of the Phase 2 Groundwater Supply and Demand Project, 165 pp. Accessed March 15, 2021 from https://www.obwb.ca/fileadmin/docs/water_supply_demand/water_supply_demand_final_report.pdf	-	-	Approximated from Fig. 12 of Neilson-Welch and Allen (2007) and well completion records for British Columbia
**Orange County Coastal Plain**	Los Angeles Basin**	Land, M., Reichard, E.G., Crawford, S.M., Everett, R.R., Newhouse, M.W., Williams, C.F. (2004). Ground-water Quality of Coastal Aquifer Systems in the West Coast Basin, Los Angeles County, California, 1999–2002. U.S. Geological Survey Scientific Investigations Report 2004-5067, 88 pp. Accessed February 15, 2021 from https://pubs.usgs.gov/sir/2004/5067/sir2004-5067.pdf	Fram, M. S., Belitz, K. (2008). Groundwater quality in the Coastal Los Angeles Basin, California; United States Geological Survey Fact Sheet 2012-3096. Accessed February 14, 2021 from https://pubs.er.usgs.gov/publication/70039952.	Reichard, E. G., Land, M., Crawford, S.M., Johnson, T.D., Everett, R.R., Kulshan, T.V., Ponti, D.J., Halford, K.L., Johnson, T.A., Paybins, K.S., Nishikawa, T. (2003). Geohydrology, Geochemistry, and Ground-Water Simulation Optimization of the Central and West Coast Basins, Los Angeles County, California. USGS Numbered Series, 2003–4065. Accessed November 28, 2021 from https://pubs.usgs.gov/wri/wrir034065/wrir034065.pdf	Approximated from Fig. 1 by Land et al. (2004), Fig. 6 by Reichard et al. (2003) and map on page 1 of Fram and Belitz (2008)
**Orcheta Region covering the Cabeco dOr Aquifer**	-	Andreu, J. M., Alcalá, F. J., Vallejos, A., Pulido-Bosch, A. (2011). Recharge to mountainous carbonated aquifers in SE Spain:	-	-	Approximated from Fig. 1 by Andreu et al. (2011)

Aquifer system	Broader aquifer system	Reference A	Reference B	Reference C	Steps to delineate
		Different approaches and new challenges. Journal of Arid Environments, 75, 1262-1270.			
**Orizaba Basin**	Orizaba-Cordoba Basins	CONAGUA (2015a). Actuualizacion de la disponibilidad media anual de agua en el acuifero Orizaba-Cordoba (3007), Estado de Veracruz. 23 pp. Accessed May 11, 2021 from https://www.gob.mx/cms/uploads/attachment/file/104452/DR_3007.pdf	-	-	Approximated from map on page 13 by CONAGUA (2015a)
**Oropesa Plain**	-	Giménez-Forcada, E. (2019). Use of the Hydrochemical Facies Diagram (HFE-D) for the evaluation of salinization by seawater intrusion in the coastal Oropesa Plain: Comparative analysis with the coastal Vinaroz Plain, Spain. HydroResearch, 2, 76-84.	-	-	Approximated friom Fig. 2 by Giménez-Forcada (2019)
**Orzooiyeh Basin**	-	Pourkhosravani, M. (2016). Qualitative analysis of Orzooiyeh plain groundwater resources using GIS techniques. Environmental Health Engineering and Management Journal, 3, 209-215.	-	-	Approximated from Fig. 1 by Pourkhosravani (2016)
**Osage Plains**	Osage Cuestas Aquifer System	Macfarlane, P.A. (2000). Revisions to the Nomenclature for Kansas Aquifers. Kansas Geological Survey Report, 14 pp. Accessed April 27, 2022 via	Schoewe, W. H. (1949). The geography of Kansas: Part II. Physical geography. Transactions of the Kansas Academy of Science, 52, 261-333. Accessed April 27,	-	Approximated from Fig. 8 by Macfarlane (2000) and Fig. 22 by Schoewe (1949)

Aquifer system	Broader aquifer system	Reference A	Reference B	Reference C	Steps to delineate
		https://journals.ku.edu/cres/article/download/11815/11159	2022 via https://www.jstor.org/stable/pdf/3625794.pdf		
**Osaka Plain**	-	Morikawa, N., Kazahaya, K., Masuda, H., Ohwada, M., Nakama, A., Nagao, K., & Sumino, H. (2008). Relationship between geological structure and helium isotopes in deep groundwater from the Osaka Basin: application to deep groundwater hydrology. Geochemical Journal, 42, 61-74.	Shintani, T., Masuda, H., Nemoto, T., Ikawa, R., Marui, A., Tanimizu, M., & Ishikawa, T. (2022). Three-dimensional structure and sources of groundwater masses beneath the Osaka Plain, Southwest Japan. Journal of Hydrology: Regional Studies, 43, 101193.	Tomozawa, Y., Onodera, S. I., Saito, M. (2019). Estimation of groundwater recharge and salinization in a coastal alluvial plain and Osaka megacity, Japan, using δ18O, δD, and Cl−. Geomate Journal, 16, 153-158.	Approximated from Fig. 1 by Morikawa et al. (2008), Fig. 1 by Shintani et al. (2022), and Fig. 1 by Tomozawa et al. (2019)
**Osoyoos Aquifer**	Okanagan Basin	Rathfelder, K., Gregory, L. (2019). Groundwater quality assessment and proposed objectives for the Osoyoos Aquifer. Water Science Series: WSS2019-06, Province of British Columbia, Victoria, 86 pp. Accessed March 15, 2021 from https://a100.gov.bc.ca/pub/acat/documents/r57603/1_1571784531661_1784376098.pdf	-	-	Approximated from Fig. 1 of Rathfelder and Gregory (2019) and well completion records for Washington and British Columbia
**Ostuta Aquifer**	-	CONAGUA (2021). Aguas subterráneas/ Acuíferos. Webpage accessed May 6, 2021 from https://sigagis.conagua.gob.mx/aprovechamientos/	-	-	Approximated from map portal by CONAGUA (2021).
**Otava and Blanice Quaternary Aquifer System**	-	Kadlecová, R., Olmer, M. (2011). Review of Groundwater Resources. Geologické výzkumy na Moravě a ve Slezsku. Proceedings of The Open	-	-	Approximated from Fig. 2 by Kadlecová and Olmer (2011)

Aquifer system	Broader aquifer system	Reference A	Reference B	Reference C	Steps to delineate
		Congress ČGS and SGS, September 22–25, 2011, Monínec 18, pp. 31-34. Accessed October 21, 2021 from https://journals.muni.cz/gvms/article/view/1679			
**Ozark Plateaus Aquifer System**	-	Clark, B.R., Duncan, L.L., Knierim, K.J. (2019). Groundwater availability in the Ozark Plateaus aquifer system. US Geological Survey Professional Paper 1854, 95 pp. Accessed February 13, 2021 from https://pubs.er.usgs.gov/publication/pp1854	Imes, J.L., Emmett, L.F. (1994). Geohydrology of the Ozark Plateaus aquifer system in parts of Missouri, Arkansas, Oklahoma, and Kansas (No. 1414-D). Accessed February 13, 2021 from https://pubs.usgs.gov/pp/1414d/report.pdf	Hays, P.D., Knierim, K.J., Breaker, B., Westerman, D.A., Clark, B.R. (2016). Hydrogeology and hydrologic conditions of the Ozark Plateaus aquifer system: U.S. Geological Survey Scientific Investigations Report 2016–5137, 61 pp. Accessed March 24, 2023 via https://pubs.er.usgs.gov/publication/sir20165137	Approximated from Fig. 1 of Hays et al. (2016), Fig. 1 by Clark et al. (2019), and Fig. 2 by Imes and Emmett (1994)
**Pahrump Valley**	-	Burbey, T. J. (1997). Hydrogeology and potential for ground-water development, carbonate-rock aquifers in southern Nevada and southeastern California. US Geological Survey Water-Resources Investigations Report 95-4168, 70 pp. Accessed March 10, 2021 from https://pubs.usgs.gov/wri/1995/4168/report.pdf	-	-	Approximated from Fig. 15 of Burbey (1997)
**Pahsimeroi Valley**	-	Graham, W.G., Campbell, L.J. (1981). Groundwater resources of Idaho. Idaho Department of Water Resources Report, 61 pp. Accessed March 23, 2021 from	-	-	Approximated from Plate 1 of Graham and Campbell (1981).

Aquifer system	Broader aquifer system	Reference A	Reference B	Reference C	Steps to delineate
		https://idwr.idaho.gov/files/publications/198108-MISC-GW-Resources-ID.pdf			
**Pahvant Valley**	-	Holmes, W. F., Thiros, S. A. (1990). Ground-water hydrology of Pahvant Valley and adjacent areas, Utah. United State Geological Survey Technical Publication 98, 64 pp. Accessed March 7, 2021 from https://waterrights.utah.gov/docSys/v920/y920/y9200006.pdf	-	-	Approximated from Fig. 8 of Brooks et al. (2005)
**Pajaro Valley**	-	California Department of Water Resources (2015f). California's groundwater update 2013 - Chapter 5: Central Coast Hydrologic Region. California Department of Water Resources Report, 86 pp. Accessed April 27, 2022 via https://cawaterlibrary.net/wp-content/uploads/2017/05/GWU2013_Ch5_CentralCoast_Final.pdf	-	-	Approximated from Fig. 5-2 by California Department of Water Resources (2015f)
**Palakkad Gap Fractured Charnockite and Gneiss Aquifers**	-	Kumar, V. S., Amarender, B., Dhakate, R., Sankaran, S., Kumar, K. R. (2016). Assessment of groundwater quality for drinking and irrigation use in shallow hard rock aquifer of Pudunagaram, Palakkad District Kerala. Applied Water Science, 6, 149-167.	Kannan, N., Joseph, S., Sheela, A. M. (2021). Characterization of Groundwater in the Shallow and Deep Aquifers of an Agriculture-Dominated Tropical Subhumid to Semiarid Region, India: a Multivariate and GIS Approach. Journal of the Indian Society of Remote Sensing, 1-16.	Saha, D., Shekhar, S., Ali, S., Vittala, S.S., Raju, N.J. (2016). Recent hydrogeological research in India. Proceedings of the Indian National Science Academy, 82, 787-803.	Approximated from Fig. 2 by Kumar et al. (2016), Fig. 3 by Kannan et al. (2021) and Fig. 1 by Saha et al. (2016)

Aquifer system	Broader aquifer system	Reference A	Reference B	Reference C	Steps to delineate
**Palen and Chuckwalla Valleys**	-	California Department of Water Resources (2015a). California's groundwater update 2013 - Chapter 12: Colorado River Hydrologic Region. California Department of Water Resources Report, 76 pp. Accessed April 27, 2022 via https://water.ca.gov/-/media/DWR-Website/Web-Pages/Programs/Groundwater-Management/Bulletin-118/Files/Statewide-Reports/GWU2013_Ch12_ColoradoRiver_Final.pdf	-	-	Approximated from Fig. 12-2 by California Department of Water Resources (2015a)
**Palo Verde Valley**	Blythe Basin	Crow, R.S., Block, D., Felger, T.J., House, P.K., Pearthree, P.A., Gootee, B.F., Youberg, A.M., Howard, K.A., Beard, L.S., (2018). The Colorado River and its deposits downstream from Grand Canyon in Arizona, California, and Nevada. US Geological Survey Open-File Report 2018–1005, 9 pp. Accessed November 7, 2021 from https://pubs.usgs.gov/of/2018/1005/ofr20181005.pdf	-	-	Approximated from Fig. 1 by Crow et al. (2018)
**Palomas Basin**	-	Anderholm, S.K. (1984). Hydrogeology of the Socorro and La Jencia Basins, Socorro County, New Mexico. US Geological Survey Water-Resources Investigations Report 84-4342, 72 pp. Accessed April 26, 2022 via	-	-	Approximated from Fig. 1 by Anderholm (1984)

Aquifer system	Broader aquifer system	Reference A	Reference B	Reference C	Steps to delineate
		https://pubs.usgs.gov/wri/1984/4342/report.pdf			
**Palouse Basin**	Columbia Plateau Regional Aquifer System	Douglas, A.A., Osiensky, J.L., Keller, C.K. (2007). Carbon-14 dating of ground water in the Palouse Basin of the Columbia River basalts. Journal of Hydrology, 334(3-4), 502-512.	Kahle, S.C., Morgan, D.S., Welch, W.B., Ely, S.R., Vaccaro, J.J., Orzol, L.L. (2011). Hydrogeologic Framework and Hydrologic Budget Components of the Columbia Plateau Regional Aquifer System, Washington, Oregon, and Idaho. US Geological Survey Scientific Investigations Report 2011–5124, 80 pp. Accessed February 16, 2021 from https://pubs.usgs.gov/sir/2011/5124/pdf/sir20115124.pdf	Reidel, S.P., Spane, F.A., Johnson, V.G. (2002). Natural gas storage in basalt aquifers of the Columbia basin, Pacific Northwest USA: A guide to site characterization. Pacific Northwest National Lab (PNNL) Report n No. PNNL-13962. Richland, Washington, USA, 277 pp. Accessed February 16, 2021 from https://www.pnnl.gov/main/publications/external/technical_reports/PNNL-13962.pdf	Palouse Basin approximated based on Fig. 1 of Douglas et al. (2007). Broader Columbia River Aquifer System approximated from Figs. 1 and 10 of Kahle et al. (2011) and sub-province map shown in Fig. 2.1 of Reidel et al. (2002).
**Palouse Slope**	Columbia Plateau Regional Aquifer System	Kahle, S.C., Morgan, D.S., Welch, W.B., Ely, S.R., Vaccaro, J.J., Orzol, L.L. (2011). Hydrogeologic Framework and Hydrologic Budget Components of the Columbia Plateau Regional Aquifer System, Washington, Oregon, and Idaho. US Geological Survey Scientific Investigations Report 2011–5124, 80 pp. Accessed February 16, 2021 from https://pubs.usgs.gov/sir/2011/5124/pdf/sir20115124.pdf	Burns, E.R., Morgan, D.S., Peavler, R.S., Kahle, S.C. (2011). Three-dimensional model of the geologic framework for the Columbia Plateau Regional Aquifer System, Idaho, Oregon, and Washington. U.S. Geological Survey Scientific Investigations Report 2010-5246, 44 pp. Accessed April 11, 2022 via https://pubs.usgs.gov/sir/2010/5246/pdf/sir20105246.pdf	-	Physiographic Provinces (Blue Mountains, Clearwater Embayment) approximated from Fig. 1 of Kahle et al. (2011). Columbia River Aquifer System approximated from Figs. 1 and 10 of Kahle et al. (2011). See also Fig. 1a of Burns et al. (2010).
**Pampa del Tamarugal Aquifer**	-	Donoso, G., Lictevout, E., Rinaudo, J. D. (2020). Groundwater management	-	-	Approximated from Fig. 25.4 by Donoso et al. (2020).

Aquifer system	Broader aquifer system	Reference A	Reference B	Reference C	Steps to delineate
		lessons from Chile. In: Sustainable Groundwater Management. Springer, Cham., pp. 481-509. Accessed May 12, 2021 from https://link.springer.com/chapter/10.1007/978-3-030-32766-8_25			
**Panoche Valley**	-	California Department of Water Resources (2015c). California's groundwater update 2013 - Chapter 9: Tulare Lake Hydrologic Region. California Department of Water Resources Report, 80 pp. Accessed April 27, 2022 via https://data.cnra.ca.gov/dataset/california-water-plan-groundwater-update-2013/resource/8a4ae915-b786-42e1-9abe-99a8fcc23349	-	-	Approximated from Fig. 9-2 by California Department of Water Resources (2015c)
**Papaghni Basin**	Cuddapah Sedimentary Basin	Dar, F. A., Perrin, J., Riotte, J., Gebauer, H. D., Narayana, A. C., Shakeel, A. (2011). Karstification in the Cuddapah Sedimentary Basin, southern India: implications for groundwater resources. Acta Carsologica, 40, 457-472.	Saha, D., Ray, R. K. (2019). Groundwater resources of India: potential, challenges and management. In Groundwater Development and Management. Springer, Cham (ed. Sikdar, P.K.), pp. 19-42.	Goswami, S., Dey, S., Zakaulla, S., Verma, M. B. (2020). Active rifting and bimodal volcanism in Proterozoic Papaghni sub-basin, Cuddapah basin (Andhra Pradesh), India. Journal of Earth System Science, 129, 1-31.	Approximated from Fig. 1 by Dar et al. (2011), Fig. 2.2 by Saha and Ray (2019), and Fig. 3 by Goswami et al. (2020)
**Papaloapan Basin**	Coastal Plain of Veracruz	Camacho, E. A. S. (2010). Estimación del volumen promedio recibido por el humedal de la subcuenca del Río Blanco (bajo Papaloapan; Veracruz), a través del cálculo de un	Campos C, A., Hernández, M. E., Moreno-Casasola, P., Cejudo Espinosa, E., Robledo R, A., Infante Mata, D. (2011). Soil water retention and carbon pools in tropical forested	-	Approximated from Fig. 6 by Camacho (2010), combining approximate boundaries of "Costera del Papaloapan" and "Cuenca Rio Papaloapan" aquifers. Broader Coastal Plain of Veracruz

Aquifer system	Broader aquifer system	Reference A	Reference B	Reference C	Steps to delineate
		balance de aguas. Aqua-LAC, 2, 78-87.	wetlands and marshes of the Gulf of Mexico. Hydrological Sciences Journal, 56, 1388-1406.		approximated from Fig. 1 by Campos et al. (2011).
**Paradise Valley in northern Nevada**	-	Bredehoeft, J.D., Farvolden, R.N. (1963). International Association of Scientific Hydrology, Commission of Subterranean Waters, Publication no. 64, p. 197–212. Accessed March 23, 2021 from http://hydrologie.org/redbooks/a064/064017.pdf	-	-	Approximated from Fig. 1 of Bredehoft and Farvolden (1963)
**Parker Valley**	Blythe Basin	Crow, R.S., Block, D., Felger, T.J., House, P.K., Pearthree, P.A., Gootee, B.F., Youberg, A.M., Howard, K.A., Beard, L.S., (2018). The Colorado River and its deposits downstream from Grand Canyon in Arizona, California, and Nevada. US Geological Survey Open-File Report 2018–1005, 9 pp. Accessed November 7, 2021 from https://pubs.usgs.gov/of/2018/1005/ofr20181005.pdf	-	-	Approximated from Fig. 1 by Crow et al. (2018)
**Parowan Valley**	-	Marston, T.M. (2017). Water resources of Parowan Valley, Iron County, Utah: U.S. Geological Survey Scientific Investigations Report 2017–5033, 45 pp. Accessed March 7 2021 from https://doi.org/10.3133/sir20175033.	-	-	Approximated from Fig. 1 of Marston et al. (2017)

Aquifer system	Broader aquifer system	Reference A	Reference B	Reference C	Steps to delineate
**Paskapoo-Scollard**	-	Grasby, S.E., Chen, Z., Hamblin, A.P., Wozniak, P.R., Sweet, A.R. (2008). Regional characterization of the Paskapoo bedrock aquifer system, southern Alberta. Canadian Journal of Earth Sciences, 45, 1501-1516.	Parks, K., Andriashek, L. (2009). Preliminary investigation of potential, natural hydraulic pathways between the Scollard and Paskapoo formations in Alberta: implications for coalbed methane production. ERCB/AGS Open File Report 2009-16, 66 pp. Accessed February 2, 2021 from https://static.ags.aer.ca/files/document/OFR/OFR_2009_16.pdf	-	Approximated from Fig. 2 of Grasby et al. 2009, and Fig. 1 of Parks and Andriashek (2009)
**Pauma Valley**	-	Helweg, O.J., Labadie, J.W. (1976). A salinity management strategy for stream-aquifer systems. Colorado State University, Hydrology Papers, 48 pp. Accessed July 1, 2023 via https://mountainscholar.org/bitstream/handle/10217/61846/HydrologyPapers_n84.pdf?sequence=1	-	-	Approximated from Fig. II-2 by Helweg and Labadie (1976)
**Payette Valley**	-	Graham, W.G., Campbell, L.J. (1981). Groundwater resources of Idaho. Idaho Department of Water Resources Report, 61 pp. Accessed March 23, 2021 from https://idwr.idaho.gov/files/publications/198108-MISC-GW-Resources-ID.pdf	-	-	Approximated from Plate 1 of Graham and Campbell (1981). Oregon portion based on well completion data.
**Pearl and Chattahoochee Aquifer System**	-	Miller, J.A. (1990). Ground Water Atlas of the United States: Segment 6, Alabama, Florida, Georgia, South Carolina. U.S.	-	-	Approximated from Fig. 73 of Miller (1990)

Aquifer system	Broader aquifer system	Reference A	Reference B	Reference C	Steps to delineate
		Geological Survey Hydrologic Atlas 730-G, 30 pp. Accessed April 5, 2021 from https://www.nrc.gov/docs/ML1706/ML17060B027.pdf			
**Pecos Valley**	-	Bruun, B., Jackson, K., Lake, P., Walker, J. (2016). Texas Aquifers Study. Texas Water Development Board Report. 336 pp. Accessed April 1, 2021 from https://www.twdb.texas.gov/groundwater/docs/studies/TexasAquifersStudy_2016.pdf#page=89	-	-	Approximated from Fig. 6-28 by Bruun et al. (2016). New Mexico portion of aquifer approximated on the basis of well completion records.
**Peedee and Black Creek and Cape Fear Aquifers**	-	Winner Jr., M.D., Coble, R.W. (1989). Hydrogeologic framework of the North Carolina Coastal Plain aquifer system. U.S. Geological Survey Report 87-690, 167 pp. Accessed April 1, 2021 from https://pubs.usgs.gov/of/1987/0690/report.pdf	Harden, S. L., Fine, J. M., Spruill, T. B. (2003). Hydrogeology and ground-water quality of Brunswick County, North Carolina. U.S. Geological Survey, Water-Resources Investigations Report 03–4051, 98 pp. Accessed April 1, 2021 from https://pubs.usgs.gov/wri/2003/4051/wri20034051.pdf	-	Approximated from Fig. 2 of Winner Jr. and Coble (1989) and Plate 9 of Harden et al. (2003).
**Pend Orielle East**	-	Graham, W.G., Campbell, L.J. (1981). Groundwater resources of Idaho. Idaho Department of Water Resources Report, 61 pp. Accessed March 23, 2021 from https://idwr.idaho.gov/files/publications/198108-MISC-GW-Resources-ID.pdf	-	-	Approximated from Plate 1 of Graham and Campbell (1981). Eastern portion of Pend Orielle River aquifer was delineated separately from the western portion.
**Pend Orielle West**	-	Graham, W.G., Campbell, L.J. (1981). Groundwater	-	-	Approximated from Plate 1 of Graham and Campbell (1981).

Aquifer system	Broader aquifer system	Reference A	Reference B	Reference C	Steps to delineate
		resources of Idaho. Idaho Department of Water Resources Report, 61 pp. Accessed March 23, 2021 from https://idwr.idaho.gov/files/publications/198108-MISC-GW-Resources-ID.pdf			Western portion of Pend Orielle River aquifer was delineated separately from the eastern portion.
**Penoyer Valley**	-	Lopes, T.J., Evetts. D.M. (2005). Ground-Water pumpage and artificial recharge estimates for calendar year 2000 and average annual natural recharge and interbasin flow by hydrographic area, Nevada. Accessed April 25, 2022 via https://pubs.usgs.gov/sir/2004/5239/sir2004-5239.pdf	Harrill, J.R., Prudic, D.E. (1998). Aquifer systems in the Great Basin region of Nevada, Utah, and adjacent states; summary report. US Geological Survey Professional Paper 1409-A, 75 pp. Accessed April 26, 2022 via https://pubs.usgs.gov/pp/1409a/report.pdf	-	Approximated from Fig. 1 by Lopes and Evetts (2005), and Fig. 2 by Harrill and Prudic (1998). Well completion records, physiography and drainage were used to delinate areas.
**Perote-Zalayeta Aquifer**	-	CONAGUA (2020he). Actualización de la disponibilidad media anual de agua en el acuífero Perote-Zalayeta (3004), estado de Veracruz. 43 pp. Accessed March 29, 2022 via https://sigagis.conagua.gob.mx/gas1/Edos_Acuiferos_18/veracruz/DR_3004.pdf	-	-	Approximated from physiography, well completion data, and Fig. 1 by CONAGUA (2020he)
**Petaluma Valley**	-	California Department of Water Resources (2015g). California's groundwater update 2013 - Chapter 4: San Francisco Bay Hydrologic Region. California Department of Water Resources Report, 78 pp. Accessed April 27, 2022 via	-	-	Approximated from Fig. 4-2 by California Department of Water Resources (2015g)

Aquifer system	Broader aquifer system	Reference A	Reference B	Reference C	Steps to delineate
		https://cawaterlibrary.net/wp-content/uploads/2017/05/GWU2013_Ch4_SanFranciscoBay_Final.pdf			
**Peumo-Pichidega-La Cabras and Pelequen-Malloa-San Vicente De Tagua Tagua and Laguna San Vicente Aquifers**	-	Direccion General de Aguas (2017). Inventario Nacional de acuiferos. Ministerio De Obras Públicas (Gobierno De Chile) report number 403, 60 pp. Accessed May 17, 2021 from https://snia.mop.gob.cl/sad/SUB5748.pdf	-	-	Approximated from map on page 42 by Direccion General de Aguas (2017).
**Piceance Basin Aquifer System**	-	Hearne, G.A., Lindner-Lunsford, J., Cain, D., Watts, K.R., Robson, S.G., Tobin, R.L., Teller, R.W., Schneider Jr, P.A., Gearhart, M.J. (1987). Colorado ground water quality. United States Geological Survey Open File Report 87-716, 14 pp. Accessed July 21, 2021 from https://pubs.usgs.gov/of/1987/0716/report.pdf	-	-	Approximated from Fig. 2 by Hearne et al. (1987)
**Pilot Valley**	-	Lopes, T.J., Evetts. D.M. (2005). Ground-Water pumpage and artificial recharge estimates for calendar year 2000 and average annual natural recharge and interbasin flow by hydrographic area, Nevada. Accessed April 25, 2022 via https://pubs.usgs.gov/sir/2004/5239/sir2004-5239.pdf	Harrill, J.R., Prudic, D.E. (1998). Aquifer systems in the Great Basin region of Nevada, Utah, and adjacent states; summary report. US Geological Survey Professional Paper 1409-A, 75 pp. Accessed April 26, 2022 via https://pubs.usgs.gov/pp/1409a/report.pdf	-	Approximated from Fig. 1 by Lopes and Evetts (2005), and Fig. 2 by Harrill and Prudic (1998). Well completion records, physiography and drainage were used to delinate areas.

Aquifer system	Broader aquifer system	Reference A	Reference B	Reference C	Steps to delineate
**Pilsen Basin**	-	Opluštil, S. (2005). The effect of paleotopography, tectonics and sediment supply on quality of coal seams in continental basins of central and western Bohemia (Westphalian), Czech Republic. International Journal of Coal Geology, 64, 173-203.	Opluštil, S., Lojka, R., Pšenika, J. (2013). Late Variscan continental basins in western Bohemia: tectono-sedimentary, climate and biotic archives. Schriftreihe Dtsch Ges Geowiss, 82, 179-201.	-	Approximated from Fig. 1c by Opluštil (2005), Fig. 1 by Opluštil et al. (2013)
**Pine Valley**	-	Lopes, T.J., Evetts. D.M. (2005). Ground-Water pumpage and artificial recharge estimates for calendar year 2000 and average annual natural recharge and interbasin flow by hydrographic area, Nevada. Accessed April 25, 2022 via https://pubs.usgs.gov/sir/2004/5239/sir2004-5239.pdf	Harrill, J.R., Prudic, D.E. (1998). Aquifer systems in the Great Basin region of Nevada, Utah, and adjacent states; summary report. US Geological Survey Professional Paper 1409-A, 75 pp. Accessed April 26, 2022 via https://pubs.usgs.gov/pp/1409a/report.pdf	-	Approximated from Fig. 1 by Lopes and Evetts (2005), and Fig. 2 by Harrill and Prudic (1998). Well completion records, physiography and drainage were used to delinate areas.
**Pingtung Plain**	-	Hsu, K. C., Wang, C. H., Chen, K. C., Chen, C. T., Ma, K. W. (2007). Climate-induced hydrological impacts on the groundwater system of the Pingtung Plain, Taiwan. Hydrogeology Journal, 15, 903-913.	Liang, C. P., Jang, C. S., Liang, C. W., Chen, J. S. (2016). Groundwater vulnerability assessment of the Pingtung Plain in Southern Taiwan. International journal of environmental Research and Public Health, 13, 1167.	Yu, H. L., Chu, H. J. (2012). Recharge signal identification based on groundwater level observations. Environmental Monitoring and Assessment, 184, 5971-5982.	Approximated from Fig. 1 by Hsu et al. (2007), Fig. 1 by Liang et al. (2016), and Fig. 1 by Yu and Chu (2012)
**Pino and Conejeros-Albatana and Tobarra-Tedera-Pinilla Aquifers**	-	Rupérez-Moreno, C., Senent-Aparicio, J., Martinez-Vicente, D., García-Aróstegui, J. L., Calvo-Rubio, F. C., Pérez-Sánchez, J. (2017). Sustainability of irrigated agriculture with overexploited aquifers: The	-	-	Approximated from Fig. 1 by Rupérez-Moreno et al. (2017)

Aquifer system	Broader aquifer system	Reference A	Reference B	Reference C	Steps to delineate
		case of Segura basin (SE, Spain). Agricultural Water Management, 182, 67-76.			
**Pir-e-Sohrab Basin**	-	Hosseini, S. M., Parizi, E., Ataie-Ashtiani, B., Simmons, C. T. (2019). Assessment of sustainable groundwater resources management using integrated environmental index: Case studies across Iran. Science of the Total Environment, 676, 792-810.	-	-	Approximated from Fig. 6 by Hosseini et al. (2019)
**Pirie Basin**	-	Evans, S. (2004). Baroota Groundwater Resource – Monitoring Review and Augmentation. Department of Water, Land and Biodiversity Conservation Report No. 2004/56, 45 pp. Accessed May 7, 2023 via https://www.waterconnect.sa.gov.au/Content/Publications/DEW/dwlbc_report_2004_56.pdf	-	-	Approximated from Fig. 1 and descriptions within Evans (2004)
**Pittsburg Plain**	-	California Department of Water Resources (2015g). California's groundwater update 2013 - Chapter 4: San Francisco Bay Hydrologic Region. California Department of Water Resources Report, 78 pp. Accessed April 27, 2022 via https://cawaterlibrary.net/wp-content/uploads/2017/05/GWU2013_Ch4_SanFranciscoBay_Final.pdf	-	-	Approximated from Fig. 4-2 by California Department of Water Resources (2015g)

Aquifer system	Broader aquifer system	Reference A	Reference B	Reference C	Steps to delineate
**Playas Valley**	-	Schwennesen, A.T., Hare, R.F. (1918). Ground water in the Animas, Playas, Hachita, and San Luis Basins, New Mexico, with analyses of water and soil. U.S. Geological Survey Water Supply Paper 422, 157 pp. Accessed March 29, 2021 from https://pubs.usgs.gov/wsp/0422/report.pdf	-	-	Approximated from Plate II of Schwennesen and Hare (1918).
**Pleasant Valley west of the San Joaquin Basin**	-	California Department of Water Resources (2015c). California's groundwater update 2013 - Chapter 9: Tulare Lake Hydrologic Region. California Department of Water Resources Report, 80 pp. Accessed April 27, 2022 via https://data.cnra.ca.gov/dataset/california-water-plan-groundwater-update-2013/resource/8a4ae915-b786-42e1-9abe-99a8fcc23349	-	-	Approximated from Fig. 9-2 by California Department of Water Resources (2015c)
**Pliocuaternario and Mioceno de Alfamen Aquifers**	-	Carceller-Layel, T., Costa-Alandí, C., Coloma-López, P., García-Vera, M. Á., & San Román-Saldaña, J. (2007). Groundwater in the central sector of the Ebro Basin. Water Resources Development, 23, 165-187.	-	-	Approximated from Fig. 3 by Carceller-Layel et al. (2007).
**Plymouth-Carver-Kingston-Duxburya Aquifer System**	-	Masterson, J.P., Walter, D.A. (2009). Hydrogeology and groundwater resources of the coastal aquifers of southeastern Massachusetts. U.S.	-	-	Approximated from Fig. 1 of Masterson and Walter (2009).

Aquifer system	Broader aquifer system	Reference A	Reference B	Reference C	Steps to delineate
		Geological Survey Circular 1338, 16 pp. Accessed April 14, 2021 from https://pubs.usgs.gov/circ/circ1338/pdf/circular%202009-1338_508.pdf			
**Pockmarked Terrain and Northern Peninsula**	Yucatan Peninsula	Bauer-Gottwein, P., Gondwe, B. R., Charvet, G., Marín, L. E., Rebolledo-Vieyra, M., Merediz-Alonso, G. (2011). the Yucatán Peninsula karst aquifer, Mexico. Hydrogeology Journal, 19, 507-524.	Munro-Stasiuk, M.J., Manahan, T. K. (2010). Investigating ancient Maya agricultural adaptation through ground penetrating radar (GPR) analysis ofkarst terrain, Northern Yucatán, Mexico. Acta Carsologica, 39, 123-36.	Perry, E., Velaquez-Oliman, V., Socki, R.A. (2003). Hydrogeology of the Yucatán Peninsula. The Lowland Maya Area: Three Millennia at the Human-Wildland Interface. Food Products Press, New York, 115-138.	Approximated from Fig. 2 by Bauer-Gottwein et al. (2011), and from Fig. 2 by Munro-Stasiuk and Manahan (2010), and from Fig. 7.1 by Perry et al. (2003).
**Polda Basin**	Eucla Basin	Berens, V., Alcoe, D., Watt, E. (2011). Non-prescribed groundwater resources assessment — Eyre Peninsula natural resources management region. Science, Monitoring and Information Division Department for Water, 110 pp. Accessed October 6, 2021 from https://www.waterconnect.sa.gov.au/Content/Publications/DEW/EP_NRM_Non-prescribed_GW_Assessment_2011.pdf	-	-	Approximated from Fig. 4 by Berens et al. (2011)
**Pomfret-Vergelegen Dolomitic Aquifer**	-	Godfrey, L., van Dyk, G. (2002). Reserve determination for the Pomfret-Vergelegen Dolomitic Aquifer, North West province. Report No ENV-P-C 2002 -031, 26 pp. Accessed October 19, 2021 from https://scholar.ufs.ac.za/bit	-	-	Approximated from Fig. 5 by Godfrey and van Dyk (2002)

Aquifer system	Broader aquifer system	Reference A	Reference B	Reference C	Steps to delineate
		stream/handle/11660/7396/Tosca%20Reserve%20Report.pdf?sequence=6&isAllowed=y			
**Poolowanna Trough and Western Eromanga Basin**	Great Artesian Basin	Ransley, T.R., Smerdon, B.D. (2012). Hydrostratigraphy, hydrogeology and system conceptualisation of the Great Artesian Basin. A technical report to the Australian Government from the CSIRO Great Artesian Basin Water Resource Assessment, 324 pp. Accessed July 20, 2021 from https://publications.csiro.au/rpr/download?pid=csiro:EP132693&dsid=DS5	Habermehl, M. A. (2020). The evolving understanding of the Great Artesian Basin (Australia), from discovery to current hydrogeological interpretations. Hydrogeology Journal, 28, 13-36.	Sandiford, M., Lawrie, K., Brodie, R. S. (2020). Hydrogeological implications of active tectonics in the Great Artesian Basin, Australia. Hydrogeology Journal, 28, 57-73.	Approximated from Fig. 2.3 by Ransley and Smerdon (2012), Fig. 4 by Sandiford et al. (2020), and Fig. 4 by Habermehl (2020)
**Popeto Aquifer**	-	Direccion General de Aguas (2017). Inventario Nacional de acuiferos. Ministerio De Obras Públicas (Gobierno De Chile) report number 403, 60 pp. Accessed May 17, 2021 from https://snia.mop.gob.cl/sad/SUB5748.pdf	-	-	Approximated from map on page 42 by Direccion General de Aguas (2017).
**Porbander Coastal Region**	Saurashtra Deccan Trap Basalts	Jain, A.K., Nayak, K.M. (2016). Aquifer Map and Management Plan, Porbandar District, Gujarat State. Central Ground Water Board Report, 119 pp. Accessed June 22, 2021 from http://cgwb.gov.in/AQM/NAQUIM_REPORT/Gujarat/Probandar.pdf	Shah, T. (2014). Towards a Managed Aquifer Recharge strategy for Gujarat, India: An economist's dialogue with hydro-geologists. Journal of Hydrology, 518, 94-107.	Gupta, P., Sharma, A., Joshi, N. (2015). Hydrochemical Characterization of Coastal Groundwater in Porbandar Region, Gujarat, India. International JournalofEngineering Research and General Science, 3, 325-331.	Approximated from Fig. 7 by Jain and Nayak (2016), Figs. 1, 2 and 4 by Shah (2014) and Fig. 1 by Gupta et al. (2015). Region is considered as within the broader Saurashtra Deccan Trap Basalt aquifer system, as these basalts form the primary aquifer in the area despite being overlain by other rocks and sediments (Jain and Nayak, 2016)

Aquifer system	Broader aquifer system	Reference A	Reference B	Reference C	Steps to delineate
**Poway Valley**	-	California Department of Water Resources (2015e). California's groundwater update 2013 - Chapter 6: South Coast Hydrologic Region. California Department of Water Resources Report, 84 pp. Accessed April 27, 2022 via https://cawaterlibrary.net/wp-content/uploads/2017/05/GWU2013_Ch6_SouthCoast_Final.pdf	-	-	Approximated from Fig. 6-2 by California Department of Water Resources (2015e)
**Powder River Basin**	Northern Great Plains	Thamke, J.N., LeCain, G.D., Ryter, D.W., Sando, R., Long, A.J. (2014). Hydrogeologic framework of the uppermost principal aquifer systems in the Williston and Powder River structural basins, United States and Canada. US Geological Survey Scientific Investigations Report 2014-5047, 50 pp. Accessed February 15, 2021 from https://pubs.usgs.gov/sir/2014/5047/pdf/sir2014-5047.pdf	Long, A.J., Thamke, J.N., Davis, K.W., Bartos, T.T. (2018). Groundwater availability of the Williston Basin, United States and Canada: U.S. Geological Survey Professional Paper 1841, 54 pp. Accessed November 29, 2021 from https://pubs.usgs.gov/pp/1841/pp1841.pdf	-	Approximated from Fig. 1 of Long et al. (2018) and Fig. 1 by Thamke et al. (2014); "Miles City arch" boundary used to distinguish Williston and Powder River structural basins
**Poza Rica Aquifer**	-	CONAGUA (2020ib). Actualización de la disponibilidad media anual de agua en el acuífero Poza Rica (3001), estado de Veracruz. 25 pp. Accessed May 2, 2022 via https://sigagis.conagua.gob.mx/gas1/Edos_Acuiferos_18/veracruz/DR_3001.pdf	-	-	Approximated from physiography, well completion data, and Fig. 1 by CONAGUA (2020ib)

Aquifer system	Broader aquifer system	Reference A	Reference B	Reference C	Steps to delineate
**Presidio-Redford Bolson and Del Peso Valley**	-	IMTA (2018). Plan estatal hidrico 2040 de Chihuahua. Report (contract) number 060-207-E75-JCAS-PRODDER, 416 pp. Accessed February 10, 2023 via https://www.nadb.org/uploads/files/1_plan_estatal_hdrico_de_chihuahua_2040_2018.pdf	Wade, S., Jigmond, M. (2013). Groundwater availability model of west Texas Bolsons (Presidio and Redford) Aquifer. Texas Water Development Board Report, 100 pp. Accessed February 10, 2023 via https://www.twdb.texas.gov/groundwater/models/gam/prbl/PRBL_ModelFinalReport.pdf	-	Approximated from Table 1.6 and Fig. 1.15 by IMTA (2018), and Fig. 1 by Wade And Jigmond (2013)
**Promontory Mountains Area**	-	Lopes, T.J., Evetts. D.M. (2005). Ground-Water pumpage and artificial recharge estimates for calendar year 2000 and average annual natural recharge and interbasin flow by hydrographic area, Nevada. Accessed April 25, 2022 via https://pubs.usgs.gov/sir/2004/5239/sir2004-5239.pdf	Harrill, J.R., Prudic, D.E. (1998). Aquifer systems in the Great Basin region of Nevada, Utah, and adjacent states; summary report. US Geological Survey Professional Paper 1409-A, 75 pp. Accessed April 26, 2022 via https://pubs.usgs.gov/pp/1409a/report.pdf	-	Approximated from Fig. 1 by Lopes and Evetts (2005), and Fig. 2 by Harrill and Prudic (1998). Well completion records, physiography and drainage were used to delinate areas.
**Proserpine Basin**	-	Currie, D., Wiltshire, E., Irvine, E., Evans, W.R., Barron, O., Cresswell, R. (2010). Investigating the impact of climate change on groundwater resources: Aquifer characterisation. Report to the National Water Commission, 93 pp. Accessed May 7, 2023 via https://publications.csiro.au/rpr/download?pid=csiro:EP202082&dsid=DS3	-	-	Approximated from Appendix A, descriptions and figures within Currie et al. (2010), in addition to local physiography and drainage patterns.
**Puangue Aquifer**	-	Direccion General de Aguas (2017). Inventario Nacional de acuiferos. Ministerio De Obras Públicas (Gobierno	-	-	Approximated from map on page 42 by Direccion General de Aguas (2017).

Aquifer system	Broader aquifer system	Reference A	Reference B	Reference C	Steps to delineate
		De Chile) report number 403, 60 pp. Accessed May 17, 2021 from https://snia.mop.gob.cl/sad/SUB5748.pdf			
**Puerto Madero Aquifer**	-	CONAGUA (2020n). Actualización de la disponibilidad media anual de agua en el acuífero Puerto Madero (3224), Estado de Zacatecas. 37 pp. Accessed March 7, 2022 via https://sigagis.conagua.gob.mx/gas1/Edos_Acuiferos_18/zacatecas/DR_3224.pdf	-	-	Approximated from Fig. 1 by CONAGUA (2020n)
**Puerto Rico South Coast Aquifer System**	-	Veve, T.D., Taggart, B.E. (1996). Atlas of Ground-Water Resources in Puerto Rico and the U.S. Virgin Islands. U.S. Geological Survey Water Resources Investigations Report 94-4198, 151 pp. Accessed May 22, 2021 from https://pubs.usgs.gov/wri/1994/4198/report.pdf	-	-	Approximated from boundaries of Santa Isabel-Patilla and Juan Diaz-Ponce Regions in Figs. 2.2.1.C-1 and 2.2.2.E-1 by Veve and Taggart (1996)
**Qabr-e Kikha Basin**	-	Hosseini, S. M., Parizi, E., Ataie-Ashtiani, B., Simmons, C. T. (2019). Assessment of sustainable groundwater resources management using integrated environmental index: Case studies across Iran. Science of the Total Environment, 676, 792-810.	-	-	Approximated from Fig. 6 by Hosseini et al. (2019); eastern boundary extended to بابا شیخ احمد
**Qaen Aquifer**	-	Fayaji, I., Sayadi, M. H., Mousazadeh, H. (2019). Potable groundwater	-	-	Approximated from Fig. 1 by Fayaji et al. (2019)

Aquifer system	Broader aquifer system	Reference A	Reference B	Reference C	Steps to delineate
		analysis using multivariate Groundwater Quality Index technique. Global Journal of Environmental Science and Management, 5, 357-370.			
**Qatruiyeh Basin**	-	Hosseini, S. M., Parizi, E., Ataie-Ashtiani, B., Simmons, C. T. (2019). Assessment of sustainable groundwater resources management using integrated environmental index: Case studies across Iran. Science of the Total Environment, 676, 792-810.	-	-	Approximated from Fig. 6 by Hosseini et al. (2019)
**Qom Kahak Basin**	-	Hosseini, S. M., Parizi, E., Ataie-Ashtiani, B., Simmons, C. T. (2019). Assessment of sustainable groundwater resources management using integrated environmental index: Case studies across Iran. Science of the Total Environment, 676, 792-810.	-	-	Approximated from Fig. 6 by Hosseini et al. (2019)
**Quchan Plain**	Shirvan-Quchan Plain	Ghorbani, H., Sadabad, S. M. (2010). Annual Changes in Some Qualitative Parameters of Groundwater in Shirvan Plain North East of Iran. World Academy of Science, Engineering and Technology, 68, 949-952.	Parizi, E., Hosseini, S. M., Ataie-Ashtiani, B., Simmons, C. T. (2020). Normalized difference vegetation index as the dominant predicting factor of groundwater recharge in phreatic aquifers: case studies across Iran. Scientific Reports, 10, 17473.	-	Approximated from description in section II.A. by Ghorbani and Sadabad (2010) and Fig. 2 by Parizi et al. (2020)
**Quincy Subregion**	Columbia Plateau Regional	United States Bureau of Reclamation (2012). Final Feasibility-Level Special	Kahle, S.C., Morgan, D.S., Welch, W.B., Ely, S.R., Vaccaro, J.J., Orzol, L.L.	Vaccaro, J.J., Kahle, S.C., Ely, D.M., Burns, E.R., Snyder, D.T., Haynes, J.V.,	Qunicy subarea approximated from location map on page 75 (Fig. 4-10) of US Bureau of

Aquifer system	Broader aquifer system	Reference A	Reference B	Reference C	Steps to delineate
	Aquifer System	Study Report Odessa Subarea Special Study. 244 pp. Accessed February 18, 2021 from https://www.usbr.gov/pn/programs/eis/odessa/finaleis/final.pdf	(2011). Hydrogeologic Framework and Hydrologic Budget Components of the Columbia Plateau Regional Aquifer System, Washington, Oregon, and Idaho. US Geological Survey Scientific Investigations Report 2011–5124, 80 pp. Accessed February 16, 2021 from https://pubs.usgs.gov/sir/2011/5124/pdf/sir20115124.pdf	Olsen, T.D., Welch, W.B., Morgan, D.S. (2015). Groundwater availability of the Columbia Plateau Regional Aquifer System, Washington, Oregon, and Idaho: U.S. Geological Survey Professional Paper 1817, 87 pp. Accessed February 18, 2021 from http://dx.doi.org/10.3133/pp1817.	Reclamation (2012) and Fig. 38 of Vaccaro et al. (2015). Broader Columbia River Aquifer System approximated from Figs. 1 and 10 of Kahle et al. (2011).
**Quinn River Valley in northern Nevada**	-	Huntington, J.L., Minor, B., Bromley, M., Morton, C. (2018). Reconnaissance Investigation of Phreatophyte Vegetation Vigor for Selected Hydrographic Areas in Nevada. Division of Hydrologic Sciences, Desert Research Institute, Reno, NV Report, 75 pp. Accessed March 23, 2021 from http://www.conservationgateway.org/ConservationByGeography/NorthAmerica/UnitedStates/nevada/water/Documents/Final%20DRI-TNC%20spatiotemporal%20phreatophyte%20report_may31.pdf	-	-	Approximated from Fig. 6 of Huntington et al. (2018); boundary extended northward and delineated based on well completion reports.
**Rafsanjan Basin**	-	Hosseini, S. M., Parizi, E., Ataie-Ashtiani, B., Simmons, C. T. (2019). Assessment of sustainable groundwater resources management using	Parizi, E., Hosseini, S. M., Ataie-Ashtiani, B., Simmons, C. T. (2020). Normalized difference vegetation index as the dominant predicting factor	-	Approximated from Fig. 6 by Hosseini et al. (2019) and Fig. 2 by Parizi et al. (2020)

Aquifer system	Broader aquifer system	Reference A	Reference B	Reference C	Steps to delineate
		integrated environmental index: Case studies across Iran. Science of the Total Environment, 676, 792-810.	of groundwater recharge in phreatic aquifers: case studies across Iran. Scientific Reports, 10, 17473.		
**Raft River Valley**	Central Snake River Plain	Graham, W.G., Campbell, L.J. (1981). Groundwater resources of Idaho. Idaho Department of Water Resources Report, 61 pp. Accessed March 23, 2021 from https://idwr.idaho.gov/files/publications/198108-MISC-GW-Resources-ID.pdf	-	-	Approximated from Plate 1 of Graham and Campbell (1981).
**Railroad Valley**	-	Rose, T. P., Davisson, M. L., Smith, D. K., Kenneally, J. M. (1998). Isotope hydrology investigation of regional groundwater flow in central Nevada. Hydrologic Resources Management Program and Underground Test Area Operable Unit FY 1997 Progress Report. Chapter 6. Accessed March 10, 2021 from https://core.ac.uk/download/pdf/204554577.pdf#page=62	-	-	Approximated from Fig. 3 of Rose et al. (1989)
**Ralston Valley**	-	Lopes, T.J., Evetts. D.M. (2005). Ground-Water pumpage and artificial recharge estimates for calendar year 2000 and average annual natural recharge and interbasin flow by hydrographic area, Nevada. Accessed April 25, 2022 via	Harrill, J.R., Prudic, D.E. (1998). Aquifer systems in the Great Basin region of Nevada, Utah, and adjacent states; summary report. US Geological Survey Professional Paper 1409-A, 75 pp. Accessed April 26, 2022 via	-	Approximated from Fig. 1 by Lopes and Evetts (2005), and Fig. 2 by Harrill and Prudic (1998). Well completion records, physiography and drainage were used to delinate areas.

Aquifer system	Broader aquifer system	Reference A	Reference B	Reference C	Steps to delineate
		https://pubs.usgs.gov/sir/2004/5239/sir2004-5239.pdf	https://pubs.usgs.gov/pp/1409a/report.pdf		
**Ramhormoz Alluvial Plain**	-	Soltani Mohammadi, A., Sayadi Shahraki, A., Naseri, A. A. (2017). Simulation of groundwater quality parameters using ANN and ANN+ PSO models (Case study: Ramhormoz Plain). Pollution, 3, 191-200.	Ghanbari, N., Rangzan, K., Kabolizade, M., Moradi, P. (2017). Improve the results of the DRASTIC model using artificial intelligence methods to assess groundwater vulnerability in Ramhormoz alluvial aquifer plain. Journal of water and soil conservation, 24, 45-65.	-	Approximated from Fig. 1 by Soltani Mohammadi et al. (2017) and Fig. 1 by Ghanbari et al. (2017)
**Ranas Orbigo-Esla and Esla-Cea Aquifers**	-	Lopez-Geta, J.A., Del Barrio Beato, V., Vega Martin, L. (2006). Explotación de las Aguas Subterráneas En El Duero: Los Retos De La Cuenca. Conference paper, 17 pp. Accessed May 28, 2021 from https://www.researchgate.net/profile/Leticia-Vega-Martin/publication/276938341_EXPLOTACION_DE_LAS_AGUAS_SUBTERRANEAS_EN_EL_DUERO_LOS_RETOS_DE_LA_CUENCA/links/555c6b3a08ae6aea08175a6e/EXPLOTACION-DE-LAS-AGUAS-SUBTERRANEAS-EN-EL-DUERO-LOS-RETOS-DE-LA-CUENCA.pdf	-	-	Approximated from Fig. 1 by Lopez-Geta et al. (2006).
**Ranegras Plain**	-	Tillman, F.D, Cordova, J.T., Leake, S.A., Thomas, B.E., Callegary, J.B. (2011). Water availability and use pilot: methods development for a regional assessment of groundwater availability, southwest alluvial basins,	-	-	Approximated from Fig. 2 of Tillman et al. (2011); boundaries also informed by the density of wells recorded in Arizona's well completion dataset.

Aquifer system	Broader aquifer system	Reference A	Reference B	Reference C	Steps to delineate
		Arizona. U.S. Geological Survey Scientific Investigations Report 2011-5071, 132 pp. Accessed April 15, 2021 from https://pubs.usgs.gov/sir/2011/5071/sir2011-5071_text.pdf			
**Rashakan Basin**	-	Javadzadeh, H., Ataie-Ashtiani, B., Hosseini, S. M., Simmons, C. T. (2020). Interaction of lake-groundwater levels using cross-correlation analysis: A case study of Lake Urmia Basin, Iran. Science of The Total Environment, 729, 138822.	-	-	Approximated from Fig. 1 by Javadzadeh et al. (2020)
**Rashtkhar Aquifer**	-	Parizi, E., Hosseini, S. M., Ataie-Ashtiani, B., Simmons, C. T. (2020). Normalized difference vegetation index as the dominant predicting factor of groundwater recharge in phreatic aquifers: case studies across Iran. Scientific Reports, 10, 17473.	-	-	Approximated from Fig. 2 by Parizi et al. (2020)
**Ravansar Plain**	-	Asadi, F., Soltanian, M., Mohmmadi, A., Setareh, P., Khezri, S. M. (2015). Geographical Zoning Physicochemical Quality Change in Groundwater Catchment Gharehsou Ten-Year Period 2003-2012. Biosciences Biotechnology Research Asia, 12, 507-515.	-	-	Approximated from Fig. 1 and study area descriptions (in section entitled "Findings") by Asadi et al. (2015)

Aquifer system	Broader aquifer system	Reference A	Reference B	Reference C	Steps to delineate
**Ravar Basin**	-	Hosseini, S. M., Parizi, E., Ataie-Ashtiani, B., Simmons, C. T. (2019). Assessment of sustainable groundwater resources management using integrated environmental index: Case studies across Iran. Science of the Total Environment, 676, 792-810.	-	-	Approximated from Fig. 6 by Hosseini et al. (2019)
**Rayen Aquifer**	-	Pazand, K., Javanshir, A. R. (2013). Hydrogeochemistry and arsenic contamination of groundwater in the Rayen area, southeastern Iran. Environmental earth sciences, 70, 2633-2644.	-	-	Approximated from Fig. 1 by Pazand et al. (2013)
**Raymond Basin**	-	California Department of Water Resources (2015e). California's groundwater update 2013 - Chapter 6: South Coast Hydrologic Region. California Department of Water Resources Report, 84 pp. Accessed April 27, 2022 via https://cawaterlibrary.net/wp-content/uploads/2017/05/GWU2013_Ch6_SouthCoast_Final.pdf	-	-	Approximated from Fig. 6-2 by California Department of Water Resources (2015e)
**Rechna Doab**	Indus Basin	Amin, M., Khan, M. R., Jamil, A. (2018). Quantification of Groundwater Storage Variations and Stressed Areas Using Multi-temporal GRACE Data: A Case Study of Upper Indus Plains, Pakistan. In Conference of	Parvaiz, A., Khattak, J. A., Hussain, I., Masood, N., Javed, T., Farooqi, A. (2021). Salinity enrichment, sources and its contribution to elevated groundwater arsenic and fluoride levels in Rachna Doab, Punjab Pakistan:	Sikandar, P., Bakhsh, A., Arshad, M., Rana, T. (2010). The use of vertical electrical sounding resistivity method for the location of low salinity groundwater for irrigation in Chaj and Rachna Doabs.	Approximated from Fig. 1 by Amin et al. (2018), Fig. 1 by Sikandar et al. (2010) and Fig. 1 by Parvaiz et al. (2021)

Aquifer system	Broader aquifer system	Reference A	Reference B	Reference C	Steps to delineate
		the Arabian Journal of Geosciences (pp. 299-304). Springer, Cham.	Stable isotope (δ2H and δ18O) approach as an evidence. Environmental Pollution, 268, 115710.	Environmental Earth Sciences, 60, 1113-1129.	
**Red Bluff**	Sacramento Valley of the Central Valley **	California Department of Water Resources (2015i). California's groundwater update 2013 - Chapter 7: Sacramento River Hydrologic Region. California Department of Water Resources Report, 126 pp. Accessed April 27, 2022 via https://water.ca.gov/-/media/DWR-Website/Web-Pages/Programs/Groundwater-Management/Bulletin-118/Files/Statewide-Reports/GWU2013_Ch7_SacramentoRiver_Final.pdf	-	-	Approximated from Fig. 7-2 by California Department of Water Resources (2015i)
**Red River Aquifer**	-	Smith, S.J., Ellis, J.H., Paizis, N.C., Becker, C.J., Wagner, D.L., Correll, J.S., Hernandez, R.J. (2021). Hydrogeology and model-simulated groundwater availability in the Salt Fork Red River aquifer, southwestern Oklahoma, 1980–2015. U.S. Geological Survey Scientific Investigations Report 2021–5003, 85 pp. Accessed April 5, 2021 from https://pubs.usgs.gov/sir/2021/5003/sir20215003.pdf	Smith, S.J., Ellis, J.H., Wagner, D.L., Peterson, S.M. (2017). Hydrogeology and simulated groundwater flow and availability in the North Fork Red River aquifer, southwest Oklahoma, 1980–2013: U.S. Geological Survey Scientific Investigations Report 2017–5098, 107 pp. Accessed April 5, 2021 from https://pubs.usgs.gov/sir/2017/5098/sir20175098.pdf	-	Approximated from Fig. 1 of Smith et al. (2017) and Fig. 1 of Smith et al. (2021).
**Red River Delta**	-	Nguyen, T. T., Kawamura, A., Tong, T. N., Nakagawa, N., Amaguchi, H., & Gilbuena Jr, R. (2015).	Van Lam, N., Van Hoan, H., & Duc Nhan, D. (2019). Investigation into groundwater resources in	Kazmierczak, J., Postma, D., Dang, T., Van Hoang, H., Larsen, F., Hass, A.E., Hoffmann, A.H., Fensholt,	Approximated from Fig. 1 by Nguyen et al. (2015), Fig. 2 by Van Lam et al. (2019) and Fig. 2 by Kazmierczak et al. (2022)

Aquifer system	Broader aquifer system	Reference A	Reference B	Reference C	Steps to delineate
		Clustering spatio–seasonal hydrogeochemical data using self-organizing maps for groundwater quality assessment in the Red River Delta, Vietnam. Journal of Hydrology, 522, 661-673.	southern part of the Red River's Delta Plain, Vietnam by the use of isotopic techniques. Water, 11, 2120.	R., Pham, N.Q., Jakobsen, R. (2022). Groundwater arsenic content related to the sedimentology and stratigraphy of the Red River delta, Vietnam. Science of the Total Environment, 814, 152641.	
**Requena-Utiel Aquifer**	-	Llopis-González, A., Sánchez, A. L., Requena, P. M., Suárez-Varela, M. M. (2014). Assessment of the microbiological quality of groundwater in three regions of the Valencian Community (Spain). International journal of environmental research and public health, 11, 5527-5540.	-	-	Approximated from Fig. 2 by Llopis-González et al. (2014)
**Reyosh Basin**	-	Parizi, E., Hosseini, S. M., Ataie-Ashtiani, B., Simmons, C. T. (2020). Normalized difference vegetation index as the dominant predicting factor of groundwater recharge in phreatic aquifers: case studies across Iran. Scientific Reports, 10, 17473.	-	-	Approximated from tabulated coordinates in the Supplementary Information within Parizi et al. (2020) and physiographic and drainage patterns
**Rheintal Aquifer**	-	Fürst, J., Bichler, A., Konecny, F. (2015). Regional frequency analysis of extreme groundwater levels. Groundwater, 53, 414-423.	-	-	Approximated from Fig. 1 by Fürst et al. (2015)
**Ribstone Creek and Northern**	Bearpaw-Judith River	Borneuf, D.M. (2005). Hydrogeological map of the Oyen area, Alberta, NTS 72M. Alberta Energy	Maathuis, H., Simpson, M. (2002). Hydrogeology of the Ribstone Creek Aquiferin Western Canada.	Tokarsky, O. (1985). Hydrogeologic Profile Alberta-Saskatchewan Boundary, Report prepared	Approximated from map by Borneuf (2005) and Fig. 3 of Ferris et al. (2017). The Judith River Formation itself is absent in at

Aquifer system	Broader aquifer system	Reference A	Reference B	Reference C	Steps to delineate
**Judith River Aquifer**	Aquifer System	Regulator Map. Accessed May 5, 2021 from https://static.ags.aer.ca/files/document/MAP/Map_120.pdf	Saskatchewan Research Council Publication No. 11500-1E02, 86 pp. Accessed May 5, 2021 from https://www.wsask.ca/PageFiles/2978/Hydrogeology%20of%20the%20Ribstone%20Creek%20Aquifer%20in%20Western%20Canada,%20Maathuis,%20H.,%20and%20Simpson,%20M.,%202002,%20SRC%20Pub%20No%2011500-1E02.pdf	for the Prairie Provinces Water Board, 56 pp. Accessed May 5, 2021 from https://www.ppwb.ca/uploads/media/5c81764eb01c3/ppwb-report-78-no-maps-en.pdf?v1	southern boundary of this area, but it is included in the broader Judith River Aquifer System, as the Judith River Formation is expected to be hydraulically connected to the Tyner Valley Aquifer. For discussion of Alberta and Saskatchewan aquifer terminology see page 32 of Tokarsky (1985). Wells also draw water from the Ribstone Creek aquifer that underlies the Judith River Formation, hence the name of this subarea includes 'Ribstone Creek' (see Fig. 23 by Maathuis and Simpson, 2002).
**Riding Mountain Odanah Shale**	-	Rutulis, M. (1986). Aquifer maps of southern Manitoba. Manitoba Water Resources Branch map. Accessed May 5, 2021 from https://www.gov.mb.ca/water/pubs/maps/water/1986_rutulis_bedrock_aquifers.pdf	-	-	Approximated from map by Rutulis (1986).
**Rincon Valley**	Rincon-Mesilla Valleys	Fuchs, E. H., King, J. P., Carroll, K. C. (2019). Quantifying disconnection of groundwater from managed-ephemeral surface water during drought and conjunctive agricultural use. Water Resources Research, 55(7), 5871-5890.	-	-	Approximated from Fig. 2 of Fuchs et al. (2019)
**Rio Baluarte Aquifer**	-	CONAGUA (2021a). Acuíferos (nacional). Webpage accessed March 8, 2022 from http://sina.conagua.gob.mx/sina/tema.php?tema=acuif	-	-	Approximated from aquifer boundaries defined by CONAGUA (2021a) web portal, physiography and drainage patterns, and well completion data.

Aquifer system	Broader aquifer system	Reference A	Reference B	Reference C	Steps to delineate
		eros&ver=mapa&o=1&n=nacional			
**Rio Canas Aquifer**	-	CONAGUA (2020dy). Actualización de la disponibilidad media anual de agua en el acuífero Rio Canas (2513), estado de Sinaloa. 43 pp. Accessed March 8, 2022 via https://sigagis.conagua.gob.mx/gas1/Edos_Acuiferos_18/sinaloa/DR_2513.pdf	-	-	Approximated from physiography, well completion data, and Fig. 1 by CONAGUA (2020dy)
**Rio Elota Aquifer**	-	CONAGUA (2021a). Acuíferos (nacional). Webpage accessed March 8, 2022 from http://sina.conagua.gob.mx/sina/tema.php?tema=acuiferos&ver=mapa&o=1&n=nacional	-	-	Approximated from aquifer boundaries defined by CONAGUA (2021a) web portal, physiography and drainage patterns, and well completion data.
**Rio Fuerte Coastal Aquifer**	-	CONAGUA (2015c). Actualización de la disponibilidad media anual de agua en al acuifero Rio Fuerte (2501), Estado de Sinaloa. Comision Nacional del Agua Report, 29 pp. Accessed May 12, 2021 from https://www.gob.mx/cms/uploads/attachment/file/103330/DR_2501.pdf	-	-	Approximated from Fig. 1 by CONAGUA (2015c).
**Rio Grande Delta**	Gulf Coast Regional Aquifer System	Weiss, J. S. (1990). Geohydrologic units of the coastal lowlands aquifer system, south-central United States. U.S. Geological Survey Regional aquifer-systems analysis. 42 pp. Accessed March 30, 2021 from	-	-	Outer margin of Gulf Coast Regional Aquifer System approximated from Fig. 2 of Weiss (1990). Mexico portion of aquifer system delineated based on well completion data and topography.

Aquifer system	Broader aquifer system	Reference A	Reference B	Reference C	Steps to delineate
		https://pubs.usgs.gov/pp/1416c/report.pdf			
**Rio Mocorito Coastal Aquifer**	-	Rivera-Hernández, J. R., Green-Ruiz, C., Pelling-Salazar, L., Trejo-Alduenda, A. (2017). Hydrochemistry of the Mocorito river coastal aquifer, Sinaloa, Mexico: water quality assessment for human consumption and agriculture suitability. Hidrobiológica, 27, 103-113.	Campos, M. N., Muñoz-Sevilla, P., Le Bail, M. (2019). Hydrological Stress and Climate Change Impact in Arid Regions with Agricultural Valleys in Northern Mexico. in: Advances in Environmental Monitoring and Assessment, Chapter 1, pp 3-19.	-	Approximated from Fig. 1 by Rivera-Hernández et al. (2017) and Fig. 2 by Campos et al. (2019).
**Rio Piaxtla Aquifer**	-	CONAGUA (2020dt). Actualización de la disponibilidad media anual de agua en el acuífero Rio Sinaloa (2502), estado de Sinaloa. 30 pp. Accessed March 8, 2022 via https://sigagis.conagua.gob.mx/gas1/Edos_Acuiferos_18/sinaloa/DR_2502.pdf	-	-	Approximated from physiography, well completion data, and Fig. 1 by CONAGUA (2020dt)
**Rio Presido Aquifer**	-	CONAGUA (2020dv). Actualización de la disponibilidad media anual de agua en el acuífero Rio Presidio (2509), estado de Sinaloa. 32 pp. Accessed March 8, 2022 via https://sigagis.conagua.gob.mx/gas1/Edos_Acuiferos_18/sinaloa/DR_2509.pdf	-	-	Approximated from physiography, well completion data, and Fig. 1 by CONAGUA (2020dv)
**Rio Sinaloa Coastal Aquifer**	-	Herrera Barrientos, J., Peinado Guevara, H. J., Campos Gaxiola, J. D. J., Cruz Enriquez, A., Peinado Guevara, V. M., Ladrón de Guevara Torres, M. D. L. A., Belmonte Jiménez, S. I., Herrera, l. E. (2020).	Campos, M. N., de Guevara Torres, M. L., Diéguez, E. T., Sevilla, P. M., Cárdenas, O. L., Ocampo, H. G. (2013). Sectorization of environmental risk and human consumption of manganese in groundwater	-	Approximated from Fig. 1 by Herrera Barrientos et al. (2019) and Fig. 1 by Campos et al. (2013)

Aquifer system	Broader aquifer system	Reference A	Reference B	Reference C	Steps to delineate
		Determination of hydraulic transmissivity in coastal aquifer by optimal estimation of the Qe-T relationship using Kalman filter. Hidrobiológica, 30. Accessed May 12, 2021 from https://hidrobiologica.izt.uam.mx/index.php/revHidro/article/view/1412/1100	extracted from the Sinaloa River Aquifer. WIT Transactions on Ecology and the Environment, 171, 247-257.		
**Rio Verde Basin**	-	Morell, I. (2003). Acuíferos detríticos costeros. Hidrogeología y Aguas Subterráneas, 1, 31-44.	-	-	Approximated from Fig. 5 by Morell (2003)
**Rio Zanjon Basin**	-	Mendez-Estrella, R., Romo-Leon, J. R., Castellanos, A. E., Gandarilla-Aizpuro, F. J., Hartfield, K. (2016). Analyzing landscape trends on agriculture, introduced exotic grasslands and riparian ecosystems in arid regions of mexico. Remote Sensing, 8, 664.	-	-	Approximated from Fig. 1 by Mendez-Estrella et al. (2016).
**Riverside-Arlington Basin**	Upper Santa Ana Basin**	California Department of Water Resources (2015e). California's groundwater update 2013 - Chapter 6: South Coast Hydrologic Region. California Department of Water Resources Report, 84 pp. Accessed April 27, 2022 via https://cawaterlibrary.net/wp-content/uploads/2017/05/GWU2013_Ch6_SouthCoast_Final.pdf	-	-	Approximated from Fig. 6-2 by California Department of Water Resources (2015e)
**Riz Basin**	-	Hosseini, S. M., Parizi, E., Ataie-Ashtiani, B.,	-	-	Approximated from Fig. 6 by Hosseini et al. (2019)

Aquifer system	Broader aquifer system	Reference A	Reference B	Reference C	Steps to delineate
		Simmons, C. T. (2019). Assessment of sustainable groundwater resources management using integrated environmental index: Case studies across Iran. Science of the Total Environment, 676, 792-810.			
**Robat-Khorramabad Plain**	-	Hosseini, S. M., Parizi, E., Ataie-Ashtiani, B., Simmons, C. T. (2019). Assessment of sustainable groundwater resources management using integrated environmental index: Case studies across Iran. Science of the Total Environment, 676, 792-810.	-	-	Approximated from Fig. 6 by Hosseini et al. (2019)
**Rockland Valley**	Eastern Snake River Plain	Graham, W.G., Campbell, L.J. (1981). Groundwater resources of Idaho. Idaho Department of Water Resources Report, 61 pp. Accessed March 23, 2021 from https://idwr.idaho.gov/files/publications/198108-MISC-GW-Resources-ID.pdf	-	-	Approximated from Plate 1 of Graham and Campbell (1981).
**Rodan Basin**	-	Hosseini, S. M., Parizi, E., Ataie-Ashtiani, B., Simmons, C. T. (2019). Assessment of sustainable groundwater resources management using integrated environmental index: Case studies across Iran. Science of the Total Environment, 676, 792-810.	Parizi, E., Hosseini, S. M., Ataie-Ashtiani, B., Simmons, C. T. (2020). Normalized difference vegetation index as the dominant predicting factor of groundwater recharge in phreatic aquifers: case studies across Iran. Scientific Reports, 10, 17473.	-	Approximated from Fig. 6 by Hosseini et al. (2019) and Fig. 2 by Parizi et al. (2020)

Aquifer system	Broader aquifer system	Reference A	Reference B	Reference C	Steps to delineate
**Rodbar Jiroft Plain**	Jazmurian Depression	Rezaei, A., Hassani, H., Hassani, S., Jabbari, N., Mousavi, S. B. F., Rezaei, S. (2019). Evaluation of groundwater quality and heavy metal pollution indices in Bazman basin, southeastern Iran. Groundwater for Sustainable Development, 9, 100245.	Hosseini, S. M., Parizi, E., Ataie-Ashtiani, B., Simmons, C. T. (2019). Assessment of sustainable groundwater resources management using integrated environmental index: Case studies across Iran. Science of the Total Environment, 676, 792-810.	-	Jazmurian Depression approximated from Fig. 2 by Rezaei et al. (2019) and from Fig. 6 by Hosseini et al. (2019)
**Roma Shelf and Northern Surat Basin**	Great Artesian Basin	Ransley, T.R., Radke, B.M., Feitz, A.J., Kellett, J.R., Owens, R., Bell, J. and Stewart, G. (2015). Hydrogeological Atlas of the Great Artesian Basin, Geoscience Australia. Canberra, 141 pp. Accessed July 19, 2021 from http://www.ga.gov.au/scientific-topics/water/groundwater/gab	Ransley, T.R., Smerdon, B.D. (2012). Hydrostratigraphy, hydrogeology and system conceptualisation of the Great Artesian Basin. A technical report to the Australian Government from the CSIRO Great Artesian Basin Water Resource Assessment, 324 pp. Accessed July 20, 2021 from https://publications.csiro.au/rpr/download?pid=csiro:EP132693&dsid=DS5	Smerdon, B.D., Ramsley, T.R. (2012). Water Resource Assessment for the Surat Region. A technical report to the Australian Government from the CSIRO Great Artesian Basin Water Resource Assessment, 142 pp. Accessed July 20, 2021 from https://publications.csiro.au/rpr/download?pid=csiro:EP132644&dsid=DS4	Approximated from Map 1 on page 9 of report by Ransley et al. (2015), and Fig. 2.3 by Ransley and Smerdon (2012). Subarea within Surat Basin delineated on the basis of physiographic features (e.g., major rivers), elevations of the base of the Great Artesian Basin (Fig. 5.2 by Smerdon and Ransley (2012b)) and topography.
**Roniz Basin**	-	Hosseini, S. M., Parizi, E., Ataie-Ashtiani, B., Simmons, C. T. (2019). Assessment of sustainable groundwater resources management using integrated environmental index: Case studies across Iran. Science of the Total Environment, 676, 792-810.	-	-	Approximated from Fig. 6 by Hosseini et al. (2019)
**Roosevelt Valley and Uinta River**	Uinta Basin	Hood, J. W. (1976). Characteristics of aquifers in the northern Uinta Basin	Lambert, P.M., Marston, T., Kimball, B.A., Stolp, B.J. (2011). Assessment of	-	Northern Uinta Basin and Roosevelt Valley, Uinta River Basin and Pelican Lake region outlines

Aquifer system	Broader aquifer system	Reference A	Reference B	Reference C	Steps to delineate
**Basin and Pelican Lake region**		area, Utah and Colorado. U.S. Geological Survey and Utah Department of Natural Resources, Division of Water Rights Technical Publication 53, 71 pp. Accessed March 7, 2021 from https://waterrights.utah.gov/docSys/v920/w920/w920009f.pdf	groundwater/surface-water interaction and simulation of potential streamflow depletion induced by groundwater withdrawal, Uinta River near Roosevelt, Utah: U.S. Geological Survey Scientific Investigations Report 2011–5044, 47 pp. Accessed March 9, 2021 from https://pubs.usgs.gov/sir/2011/5044/pdf/sir20115044.pdf		approximated from Plates 1-3 of Hood (1976). Further information on Roosevelt Valley available via Lambert et al. (2011)
**Rose Valley**	-	California Department of Water Resources (2015b). California's groundwater update 2013 - Chapter 11: South Lahontan Hydrologic Region. California Department of Water Resources Report, 80 pp. Accessed April 27, 2022 via https://water.ca.gov/-/media/DWR-Website/Web-Pages/Programs/Groundwater-Management/Data-and-Tools/Files/Statewide-Reports/California-Groundwater-Update-2013/California-Groundwater-Update-2013---Chapter-11---South-Lahontan.pdf	-	-	Approximated from Fig. 11-2 by California Department of Water Resources (2015b) and Fig. 1 by Chapman et al. (2022)
**Roswell Basin**	-	Land, L. (2016a). Overview of Fresh and Brackish Water Quality in New Mexico. Open file Report 583. 4 pp. Accessed February 17, 2021 from	Welder, G. E. (1983). Geohydrologic framework of the Roswell ground-water basin, Chaves and Eddy Counties, New Mexico. New Mexico State Engineer	Land, L., Newton, B.T. (2007). Seasonal and long-term variations in hydraulic head in a karstic aquifer: Roswell artesian basin, New Mexico. New Mexico Bureau	Approximated from Map by Land (2016a), Fig. 1 by Land and Newton (2007), and Fig. 1 by Welder (1983)

Aquifer system	Broader aquifer system	Reference A	Reference B	Reference C	Steps to delineate
		https://geoinfo.nmt.edu/resources/water/amp/brochures/BWA/Estancia_Basin_FBWQNM.pdf	Technical Report 42, Sante Fe, New Mexico, 62 pp. Accessef April 12, 2022 via https://www.ose.state.nm.us/Library/TechnicalReports/TechReport-042.pdf	of Geology and Mineral Resources Open-File Report 503, 38 pp. Accessed April 12, 2022 via https://geoinfo.nmt.edu/publications/openfile/downloads/500-599/503/ofr_503.pdf	
**Round Valley**	-	Graham, W.G., Campbell, L.J. (1981). Groundwater resources of Idaho. Idaho Department of Water Resources Report, 61 pp. Accessed March 23, 2021 from https://idwr.idaho.gov/files/publications/198108-MISC-GW-Resources-ID.pdf	-	-	Approximated from Plate 1 of Graham and Campbell (1981).
**Round Valley and Pine Creek Basin**	-	Daskin, W.R. (1988). Preliminary evaluation of the hydrogeologic system in Owens Valley, California. US Geological Survey Water-Resources Investigations Report 88-4003, 81 pp. Accessed April 27, 2022 via https://pubs.usgs.gov/wri/1988/4003/report.pdf	-	-	Approximated from Fig. 1 by Daskin (1988)
**Roussillon Plain**	-	Serrat, P., Lenoble, J. L. (2007). La surexploitation des aquifères du Roussillon: une ressource patrimoniale en danger. La Houille Blanche, 93, 71-78.	-	-	Approximated from Fig. 1 by Serrat and Lenoble (2007)
**Ruby Valley**	-	Berger, D. L. (2006). Hydrogeology and Water Resources of Ruby Valley, Northeastern Nevada. Scientific Investigations Report 2005-5247, 48 pp.	-	-	Approximated from Fig. 3 of Berger (2006).

Aquifer system	Broader aquifer system	Reference A	Reference B	Reference C	Steps to delineate
		Accessed March 23, 2021 from https://pubs.usgs.gov/sir/2005/5247/sir2005-5247.pdf			
**Rudsar Coastal Area**	-	Alimoradi, J., Naghipour, D., Kamani, H., Asgari, G., Naimi-Joubani, M., Ashrafi, S. D. (2018). Data on corrosive water in the sources and distribution network of drinking water in north of Iran. Data in Brief, 17, 105-118.	-	-	Approximated from the study area depicted in Fig. 1 by Alimoradi et al. (2018)
**Rush Springs Aquifer**	-	Becker, M.F., Runkle, D.L. (1998). Hydrogeology, water quality, and geochemistry of the Rush Springs aquifer, western Oklahoma. U.S. Geological Survey Water-Resources Investigations Report 98-4081, 44 pp. Accessed April 5, 2021 from https://pubs.usgs.gov/wri/1998/4081/report.pdf	-	-	Approximated from Fig. 1 of Becker and Runkle (1998)
**Rush Valley**	-	Gardner, P. M., Kirby, S. (2011). Hydrogeologic and geochemical characterization of groundwater resources in Rush Valley, Tooele County, Utah. U. S. Geological Survey Scientific Investigations Report 2011–5068, 80 pp. Accessed March 7, 2021 from https://pubs.usgs.gov/sir/2011/5068/pdf/sir20115068.pdf	-	-	Approximated from Fig. 1 of Gardner and Kirby (2011).

Aquifer system	Broader aquifer system	Reference A	Reference B	Reference C	Steps to delineate
**Ryan Flat**	Salt Basin Bolson	Beach, J.A., Ashworth, J.B., Finch, S.T., Chaistain-Howley, A., Calhoun, K., Urbanczyk, K.M., Sharp, J.M., Olsonm J. (2004a). Groundwater availability model for the igneous and parts of the West Texas Bolsons (Wild Horse Flat, Michigan Flat, Ryan Flat and Lobo Flat) aquifers. Texas Water Development Board Report, 407 pp. Accessed May 1, 2023 via https://www.twdb.texas.gov/groundwater/models/gam/igbl/IGBL_Model_Report.pdf	-	-	Approximated from Fig. 2.1.3 by Beach et al. (2004a)
**Saadat Abad Basin**	-	Hosseini, S. M., Parizi, E., Ataie-Ashtiani, B., Simmons, C. T. (2019). Assessment of sustainable groundwater resources management using integrated environmental index: Case studies across Iran. Science of the Total Environment, 676, 792-810.	-	-	Approximated from Fig. 6 by Hosseini et al. (2019)
**Saanich Peninsula**	-	Kenny, S. (2004). Aquifers of the Capital Regional District. Capital Regional District Report, Victoria, B.C. 102 pp. Accessed April 26, 2021 from https://www.env.gov.bc.ca/wsd/plan_protect_sustain/groundwater/aquifers/aquifers_crd/pdfs/aquif_crd.pdf and also https://www.env.gov.bc.ca/	-	-	Approximated from Map 1 of Kenny (2004).

Aquifer system	Broader aquifer system	Reference A	Reference B	Reference C	Steps to delineate
		wsd/plan_protect_sustain/groundwater/aquifers/aquifers_crd/pdfs/append_b.pdf			
**Sabinas Aquifer**	-	CONAGUA (2020p). Actualización de la disponibilidad media anual de agua en el acuífero Sabinas (3201), Estado de Zacatecas. 36 pp. Accessed March 7, 2022 via https://sigagis.conagua.gob.mx/gas1/Edos_Acuiferos_18/zacatecas/DR_3201.pdf	-	-	Approximated from Fig. 1 by CONAGUA (2020p)
**Sabinas Paras Aquifer**	-	CONAGUA (2020ct). Actualización de la disponibilidad media anual de agua en el acuífero Sabinas Paras (1902), estado de Nuevo Leon. 16 pp. Accessed March 8, 2022 via https://sigagis.conagua.gob.mx/gas1/Edos_Acuiferos_18/nleon/DR_1902.pdf	-	-	Approximated from physiography, well completion data, and Fig. 1 by CONAGUA (2020ct)
**Sabzevar Plain**	-	Boroghani, M., Taie, M., & Mirnia, S. K. (2013). Analysis of relationship between hydrogeological and climatological droughts using SWI and SPI indices in Sabzevar Plain. Iranian Journal of Range and Desert Research, 20, 13 pp.	-	-	Approximated from Fig. 1 by Boroghani et al. (2013)
**Sadegh Abad Basin**	-	Hosseini, S. M., Parizi, E., Ataie-Ashtiani, B., Simmons, C. T. (2019). Assessment of sustainable groundwater resources management using integrated environmental index: Case studies across	-	-	Approximated from Fig. 6 by Hosseini et al. (2019)

Aquifer system	Broader aquifer system	Reference A	Reference B	Reference C	Steps to delineate
		Iran. Science of the Total Environment, 676, 792-810.			
**Sadich Basin**	-	Hosseini, S. M., Parizi, E., Ataie-Ashtiani, B., Simmons, C. T. (2019). Assessment of sustainable groundwater resources management using integrated environmental index: Case studies across Iran. Science of the Total Environment, 676, 792-810.	-	-	Approximated from Fig. 6 by Hosseini et al. (2019)
**Sahlabad Basin**	-	Hosseini, S. M., Parizi, E., Ataie-Ashtiani, B., Simmons, C. T. (2019). Assessment of sustainable groundwater resources management using integrated environmental index: Case studies across Iran. Science of the Total Environment, 676, 792-810.	-	-	Approximated from Fig. 6 by Hosseini et al. (2019)
**Sahneh-Bistoon Plain**	-	Nilzad, M., Moradi, H., Jalili, K. (2018). Estimation of Temporal and Spatial Variations of the Level of the Aquifers in Bisotun Plain of Kermanshah Province with Geostatistical Methods. Irrigation and Water Engineering, 8, 79-92.	-	-	Approximated from Fig. 1 by Nilzad et al. (2018)
**Sahre e-Bagh -e Lar Basin**	-	Hosseini, S. M., Parizi, E., Ataie-Ashtiani, B., Simmons, C. T. (2019). Assessment of sustainable groundwater resources management using integrated environmental	-	-	Approximated from Fig. 6 by Hosseini et al. (2019)

Aquifer system	Broader aquifer system	Reference A	Reference B	Reference C	Steps to delineate
		index: Case studies across Iran. Science of the Total Environment, 676, 792-810.			
**Sain Alto Aquifer**	-	CONAGUA (2020q). Actualización de la disponibilidad media anual de agua en el acuífero Sabinas (3216), Estado de Zacatecas. 38 pp. Accessed March 7, 2022 via https://sigagis.conagua.gob.mx/gas1/Edos_Acuiferos_18/zacatecas/DR_3216.pdf	-	-	Approximated from Fig. 1 by CONAGUA (2020q)
**Saint Francis Coastal Aquifer**	-	Campbell, E. E., Parker-Nance, T., Bate, G. C. (1992). A compilation of information on the magnitude, nature and importance of coastal aquifers in Southern Africa. Water Research Commission Report No. 370/1/92, 193 pp. Accessed October 14, 2021 from http://www.wrc.org.za/wp-content/uploads/mdocs/370-1-92.pdf	-	-	Approximated from Fig. 3 on pp. 36 of Campbell et al. (1992)
**Saint Marys Basin**	Maritimes Basin	Stapinsky, M. Michaud, Y., Morin, R.H., Butler, K.E., Deblonde, C., Chi, G, Thériault, T., Castonguay, S., Boisvert, V., Julien, H.P., Conohan, D., Hulsman, B., Carrier, C. Marion, J., Boisvert, É. (2002). Groundwater resources assessment in the Carboniferous Maritimes Basin:	Rivard, C., Michaud, Y., Lefebvre, R., Deblonde, C., Rivera, A. (2008). Characterization of a regional aquifer system in the Maritimes Basin, Eastern Canada. Water Resources Management, 22, 1649-1675.	Bianco, E. (2013). Seismic Interpretation of the Windsor-Kennetcook Basin. Geological Survey of Canada Open File 7452, 31 pp. Accessed May 5, 2021 from https://ftp.maps.canada.ca/pub/nrcan_rncan/publications/STPublications_PublicationsST/292/292763/of_7452.pdf	Approximated from Fig. 2 of Rivard et al. (2008) and Figs. 1 and 3 of Stapinsky et al. (2002). Area bounds approximated from Fig. 1 of Bianco (2013).

Aquifer system	Broader aquifer system	Reference A	Reference B	Reference C	Steps to delineate
		preliminary results of the hydrogeological characterization, New Brunswick, Nova Scotia, and Prince Edward Island. Geological Survey of Canada Current Research Report 2002-D8, 14 pp. Accessed May 5, 2021 from http://www.gov.pe.ca/photos/original/cle_WA10.pdf			
**Salinas Valley**	-	Salinas Valley Basin Integrated Sustainability Plan (2020). Accessed February 15, 2021 from https://svbgsa.org/wp-content/uploads/2019/03/Valley-Wide-Integrated-Sustainability-Plan-optimized.pdf	Hamlin, H. (1904). Water resources of the Salinas Valley, California. U.S. Geological Survey Water Supply Paper 89, 123 pp. Accessed April 6, 2021 from https://pubs.usgs.gov/wsp/0089/report.pdf	-	Approximated from Fig. 5-2 of Salinas Valley Basin Integrated Sustainability Plan (2020) and Plate 1 of Hamlin (1904).
**Salisbury Plain and Hampshire Chalk Aquifer**	Chalk Aquifer System	MacDonald, A.M., Allen, D.J. (2001). Aquifer properties of the Chalk of England. Quarterly Journal of Engineering Geology and Hydrogeology, 34, 371-384.	-	-	Approximated from Fig. 8 by MacDonald and Allen (2001)
**Salt Lake Valley**	-	Thiros, S.A. (2003). Hydrogeology of shallow basin-fill deposits in areas of Salt Lake Valley, Salt Lake County, Utah. U.S. Geological Survey Water-Resources Investigations Report 2003-4029, 32 pp. Accessed March 27, 2021 from https://pubs.usgs.gov/wri/wri034029/pdf/wri034029.pdf	Manning, A.H., Solomon, D.K. (2005). An integrated environmental tracer approach to characterizing groundwater circulation in a mountain block. Water Resources Research, 41, W12412, doi:10.1029/2005WR004178	Wallace, J., Lowe, M. (2009). Ground-water quality classification for the Principal Basin-fill Aquifer, Salt Lake Valley, Salt Lake County, Utah. Utah Geological Survey Open-File Report 560, 87 pp. Accessed April 18, 2022 via https://ugspub.nr.utah.gov/publications/open_file_reports/ofr-560.pdf	Approximated from Fig. 1 of Thiros (2003), Fig. 1 by Wallace and Lowe (2009), and Fig. 3 by Manning and Solomon (2005)

Aquifer system	Broader aquifer system	Reference A	Reference B	Reference C	Steps to delineate
**Salvatierra-Acambara and Eastern Cienega Prieta-Moroleon Aquifers**	-	CONAGUA (2021a). Acuíferos (nacional). Webpage accessed March 8, 2022 from http://sina.conagua.gob.mx/sina/tema.php?tema=acuiferos&ver=mapa&o=1&n=nacional	-	-	Approximated from aquifer boundaries defined by CONAGUA (2021a) web portal, physiography and drainage patterns, and well completion data. Eastern Cienega Prieta-Moroleon Aquifer divided from Cienega Prieta-Moroleon Aquifer area based on topography and drainage (specifically, the western boundary of this area was delineated along the eastern shoreline of Laguna de Yuriria)
**Salzburg Basin**	-	Dörfler, M. (2021). Analysis of aquifer-induced soil movements of heterogeneous subsoil in urban areas based on groundwater, borehole and InSAR data, a case study of Salzburg. Master Thesis, Paris-Lodron-University of Salzburg, 58 pp. Accessed June 13, 2023 via https://eplus.uni-salzburg.at/obvusbhs/content/titleinfo/7301785/full.pdf	-	-	Southern portion of region approximated from Fig. 2 by Dörfler (2021). Northern extent approximated from drainage and physiography.
**San Agustin Plains**	-	Rinehart, A., Koning, D., Timmons, S. (2017). Hydrogeology of the San Agustin Plains. Presentation at the 62nd New Mexico Water Conference, 36 slides. Accessed June 13, 2023 via https://geoinfo.nmt.edu/geoscience/research/documents/37/D2_07_Alex_Rinehart.pdf	-	-	Approximated from map on slide 3 by Rinehart et al. (2017)
**San Antonio Creek Valley**	-	Martin, P. (1984). Development and calibration of a two-	-	-	Approximated from maps on pages 8 and 9 of Martin (1984)

Aquifer system	Broader aquifer system	Reference A	Reference B	Reference C	Steps to delineate
		dimensional digital model for the analysis of the ground-water flow system in the San Antonio Creek Valley, Santa Barbara County, California. Water-Resources Investigations Report 84-4340, 73 pp. Accessed March 7, 2021 from https://pubs.usgs.gov/wri/1984/4340/report.pdf			
**San Bernardino Basin**	Upper Santa Ana Basin**	California Department of Water Resources (2015e). California's groundwater update 2013 - Chapter 6: South Coast Hydrologic Region. California Department of Water Resources Report, 84 pp. Accessed April 27, 2022 via https://cawaterlibrary.net/wp-content/uploads/2017/05/GWU2013_Ch6_SouthCoast_Final.pdf	-	-	Approximated from Fig. 6-2 by California Department of Water Resources (2015e)
**San Bruno Aquifer**	-	CONAGUA (2021a). Acuíferos (nacional). Webpage accessed March 8, 2022 from http://sina.conagua.gob.mx/sina/tema.php?tema=acuiferos&ver=mapa&o=1&n=nacional	-	-	Approximated from aquifer boundaries defined by CONAGUA (2021a) web portal, physiography and drainage patterns, and well completion data.
**San Emidio Desert**	-	Lopes, T.J., Evetts. D.M. (2005). Ground-Water pumpage and artificial recharge estimates for calendar year 2000 and average annual natural recharge and interbasin	Harrill, J.R., Prudic, D.E. (1998). Aquifer systems in the Great Basin region of Nevada, Utah, and adjacent states; summary report. US Geological Survey Professional Paper 1409-A,	-	Approximated from Fig. 1 by Lopes and Evetts (2005), and Fig. 2 by Harrill and Prudic (1998). Well completion records, physiography and drainage were used to delinate areas.

Aquifer system	Broader aquifer system	Reference A	Reference B	Reference C	Steps to delineate
		flow by hydrographic area, Nevada. Accessed April 25, 2022 via https://pubs.usgs.gov/sir/2004/5239/sir2004-5239.pdf	75 pp. Accessed April 26, 2022 via https://pubs.usgs.gov/pp/1409a/report.pdf		
**San Felipe-Punta Estrella Aquifer**	-	CONAGUA (2015l). Actualización de la disponibilidad media anual de agua en el acuífero San Felipe-Punta Estrella (0222), Estado de Baja California. 29 pp. Accessed January 24, 2022 via https://www.gob.mx/cms/uploads/attachment/file/103420/DR_0222.pdf	-	-	Approximated from Fig. 1 by CONAGUA (2015l) and well completion data
**San Fernando Valley**	-	California Department of Water Resources (2015e). California's groundwater update 2013 - Chapter 6: South Coast Hydrologic Region. California Department of Water Resources Report, 84 pp. Accessed April 27, 2022 via https://cawaterlibrary.net/wp-content/uploads/2017/05/GWU2013_Ch6_SouthCoast_Final.pdf	-	-	Approximated from Fig. 6-2 by California Department of Water Resources (2015e)
**San Jacinto Basin**	-	Kent, R., Belitz, K. (2009). Ground-water quality data in the Upper Santa Ana Watershed Study Unit, November 2006 to March 2007: Results from the California GAMA Program: U.S. Geological Survey Data Series 404, 116 pp. Accessed March 21, 2021 from	-	-	Approximated from Fig. 2 of Kent and Belitz (2009)

Aquifer system	Broader aquifer system	Reference A	Reference B	Reference C	Steps to delineate
		https://pubs.usgs.gov/ds/404/ds404.pdf			
**San Jose de Guaymas Aquifer**	-	CONAGUA (2020bf). Actualización de la disponibilidad media anual de agua en el acuífero San Jose de Guaymas (2636), estado de Sonora. 17 pp. Accessed March 8, 2022 via https://sigagis.conagua.gob.mx/gas1/Edos_Acuiferos_18/sonora/DR_2636.pdf	-	-	Approximated from physiography, well completion data, and Fig. 1 by CONAGUA (2020bf)
**San Jose del Cabo Aquifer**	-	Muir, M.A.K., Martinez, A.A. (2018). Preliminary Assessment of Water Resources including Climate Considerations for the Los Cabos and La Paz Municipalities in the State of Baja California Sur, Mexico. Conference: International Water Association Water, Energy and Climate Conference (May 21 to 23, 2024), Mexico City, 13 pp. Accessed May 6, 2021 from https://arctic.ucalgary.ca/sites/default/files/April18-IWA-FinalConferenceVersion-MAKMuir.pdf	-	-	Approximated from map on page 2 of Muir and Martinez (2018).
**San Juan Valley**	-	California Department of Water Resources (2015e). California's groundwater update 2013 - Chapter 6: South Coast Hydrologic Region. California Department of Water Resources Report, 84 pp. Accessed April 27, 2022 via	-	-	Approximated from Fig. 6-2 by California Department of Water Resources (2015e)

Aquifer system	Broader aquifer system	Reference A	Reference B	Reference C	Steps to delineate
		https://cawaterlibrary.net/wp-content/uploads/2017/05/GWU2013_Ch6_SouthCoast_Final.pdf			
**San Lucas Aquifer**	-	CONAGUA (2021a). Acuíferos (nacional). Webpage accessed March 8, 2022 from http://sina.conagua.gob.mx/sina/tema.php?tema=acuiferos&ver=mapa&o=1&n=nacional	-	-	Approximated from aquifer boundaries defined by CONAGUA (2021a) web portal, physiography and drainage patterns, and well completion data.
**San Luis Rey Valley**	-	California Department of Water Resources (2015e). California's groundwater update 2013 - Chapter 6: South Coast Hydrologic Region. California Department of Water Resources Report, 84 pp. Accessed April 27, 2022 via https://cawaterlibrary.net/wp-content/uploads/2017/05/GWU2013_Ch6_SouthCoast_Final.pdf	-	-	Approximated from Fig. 6-2 by California Department of Water Resources (2015e)
**San Luis Valley**	Middle Rio Grande	Leonard, G.J., Watts, K.R. (1989). Leonard, G. J., & Watts, K. R. (1989). Hydrogeology and simulated effects of ground-water development on an unconfined aquifer in the Closed Basin Division, San Luis Valley, Colorado. U.S. Geological Survey Water-Resources Investigations Report 87-4284, 47 pp. Accessed April 10, 2022 via	Powell, W. J. (1958). Ground-water resources of the San Luis Valley, Colorado. U.S. Geological Survey Water-Supply Paper 1379, 300 pp. Accessed April 10, 2022 via https://pubs.usgs.gov/wsp/1379/report.pdf	Siebenthal, C.E. (1910). Geology and water resources of the San Luis Valley, Colorado. U.S. Geological Survey Water-Supply Paper 240, 152 pp. Accessed April 10, 2022 via https://pubs.usgs.gov/wsp/0240/report.pdf	Approximated from Fig. 1 by Leonard and Watts (1987), Fig. 1 by Siebenthal (1910), and Fig. 6 by Powell (1958)

Aquifer system	Broader aquifer system	Reference A	Reference B	Reference C	Steps to delineate
		https://pubs.usgs.gov/wri/1987/4284/report.pdf			
**San Marcos-Palo Verde Aquifer**	-	CONAGUA (2021a). Acuíferos (nacional). Webpage accessed March 8, 2022 from http://sina.conagua.gob.mx/sina/tema.php?tema=acuiferos&ver=mapa&o=1&n=nacional	-	-	Approximated from aquifer boundaries defined by CONAGUA (2021a) web portal, physiography and drainage patterns, and well completion data.
**San Mateo Plain**	-	California Department of Water Resources (2015g). California's groundwater update 2013 - Chapter 4: San Francisco Bay Hydrologic Region. California Department of Water Resources Report, 78 pp. Accessed April 27, 2022 via https://cawaterlibrary.net/wp-content/uploads/2017/05/GWU2013_Ch4_SanFranciscoBay_Final.pdf	-	-	Approximated from Fig. 4-2 by California Department of Water Resources (2015g)
**San Pasqual Valley**	-	California Department of Water Resources (2015e). California's groundwater update 2013 - Chapter 6: South Coast Hydrologic Region. California Department of Water Resources Report, 84 pp. Accessed April 27, 2022 via https://cawaterlibrary.net/wp-content/uploads/2017/05/GWU2013_Ch6_SouthCoast_Final.pdf	-	-	Approximated from Fig. 6-2 by California Department of Water Resources (2015e)
**San Pedro Basin**	-	Callegary, J.B., Minjárez Sosa, I., Tapia Villaseñor,	Sanchez, R., Lopez, V., Eckstein, G. (2016).	Flora, S., Davis, T. (2009). Hydrologic Map Series	USA portion approximated from Fig. 1 of Flora and Davis (2009).

Aquifer system	Broader aquifer system	Reference A	Reference B	Reference C	Steps to delineate
		E.M., dos Santos, P., Monreal Saavedra, R., Grijalva Noriega, F.J., Huth, A.K., Gray, F., Scott, C.A., Megdal, S.B., Oroz Ramos, L.A., Rangel Medina, M., Leenhouts, J.M. (2016). San Pedro River Aquifer Binational Report: International Boundary and Water Commission. 173 pp. Accessed February 12, 2021 via https://www.ibwc.gov/Files/Binational_Study_Transboundary_San_Pedro_Aquifer.pdf	Identifying and characterizing transboundary aquifers along the Mexico–US border: An initial assessment. Journal of Hydrology, 535, 101-119.	(HMS), Water Level Change Map Series (WLCMS), and Basin Sweep Assessment Report ADWR Basins and Sub-Basins. Arizona Department of Water Resources Hydrology Division Field Services Section https://new.azwater.gov/sites/default/files/HMSWLCMSBasinSweepAssessmentReport2009.pdf	Hydrologic Map Series (HMS), Water Level Change Map Series (WLCMS), and Basin Sweep Assessment Report ADWR Basins and Sub-Basins. Arizona Department of Water Resources Hydrology Division Field Services Section https://new.azwater.gov/sites/default/files/HMSWLCMSBasinSweepAssessmentReport2009.pdf ("Allen Flat" basin included with "Sierra Vista" basin). Mexico Portion estimate from Fig. 3 of Sanchez et al. (2016)
**San Ramon Valley**	-	California Department of Water Resources (2015g). California's groundwater update 2013 - Chapter 4: San Francisco Bay Hydrologic Region. California Department of Water Resources Report, 78 pp. Accessed April 27, 2022 via https://cawaterlibrary.net/wp-content/uploads/2017/05/GWU2013_Ch4_SanFranciscoBay_Final.pdf	-	-	Approximated from Fig. 4-2 by California Department of Water Resources (2015g)
**San Simon Valley**	-	Schwennesen, A.T., Forbes, R.H. (1919). Ground water in San Simon Valley, Arizona and New Mexico. U.S. Geological Survey Water Supply Paper 425, 161 pp. Accessed March 29, 2021 from	-	-	Approximated from plate 1 of Schwennesen and Forbes (1919)

Aquifer system	Broader aquifer system	Reference A	Reference B	Reference C	Steps to delineate
		https://pubs.usgs.gov/wsp/0425a/report.pdf			
**San Timoteo Basin**	-	California Department of Water Resources (2015e). California's groundwater update 2013 - Chapter 6: South Coast Hydrologic Region. California Department of Water Resources Report, 84 pp. Accessed April 27, 2022 via https://cawaterlibrary.net/wp-content/uploads/2017/05/GWU2013_Ch6_SouthCoast_Final.pdf	-	-	Approximated from Fig. 6-2 by California Department of Water Resources (2015e)
**Sandlands Aquifer**	-	Cherry, A.J. (2000). A multi-tracer estimation of groundwater recharge in a glaciofluvial aquifer in southeastern Manitoba. M.Sc. Thesis, University of Ottawa, 131 pp. Accessed May 5, 2021 from https://ruor.uottawa.ca/bitstream/10393/8900/1/MQ48143.PDF	-	-	Approximated from Fig. 1 of Cherry (2000).
**Sanpete Valley**	-	Richardson, G. B. (1907). Underground water in Sanpete and Central Sevier Valleys, Utah. U.S. Geological Survey Water-Supply and Irrigation Paper No. 199, 77 pp. Accessed March 7, 2021 from https://pubs.usgs.gov/wsp/0199/report.pdf	-	-	Approximated from Fig. 1 of Richardson (1907)
**Santa Ana Aquifer**	-	Ministerio de Medio Ambiente y Recursos Natural (2016). Informe de monitoreo de los acuíferos	-	-	Approximated from map on page 28 by Ministerio de Medio Ambiente y Recursos Natural (2016)

Aquifer system	Broader aquifer system	Reference A	Reference B	Reference C	Steps to delineate
		de Zapotitán, Santa Ana y San Miguel. Ministerio de Medio Ambiente y Recursos Natural Report, 59 pp. Accessed February 8, 2023 via http://rcc.marn.gob.sv/bitstream/handle/123456789/127/Acuiferos%20%20StaAna%2c%20SnMiguel%20y%20Zapotit%c3%a1n_2016.pdf?sequence=1&isAllowed=y			
**Santa Barbara and Foothill Basin**	-	Nishikawa, T., ed. (2018). Santa Barbara and Foothill groundwater basins Geohydrology and optimal water resources management—Developed using density dependent solute transport and optimization models, U.S. Geological Survey, Scientific Investigations Report 2018-5059. Accessed March 20, 2021 from https://pubs.usgs.gov/sir/2018/5059/sir20185059_.pdf	-	-	Approximated from Fig. 1 of Nishikawa (2018)
**Santa Clara Valley**	-	Hanson, R. T., (2015). Hydrologic framework of the Santa Clara Valley, California. Geosphere 11, doi:10.1130/GES01104.1 Accessed March 27, 2021 from https://ca.water.usgs.gov/pubs/2015/Hanson2015.pdf	-	-	Approximated from Fig. 1 of Hanson (2015).

Aquifer system	Broader aquifer system	Reference A	Reference B	Reference C	Steps to delineate
**Santa Clara-Calleguas Basin**	-	Hanson, R.T., Martin, P., Koczot, K.M. (2002). Simulation of ground-water/surface-water flow in the Santa Clara-Calleguas ground-water basin, Ventura County, California. Water-Resources Investigations Report 2002-4136, 172 pp. Accessed March 20, 2021 from https://pubs.usgs.gov/wri/wri024136/wrir024136.pdf	-	-	Approximated from Fig. 7 of Hanson et al. (2002)
**Santa Cruz Coastal Terraces**	-	California Department of Water Resources (2015f). California's groundwater update 2013 - Chapter 5: Central Coast Hydrologic Region. California Department of Water Resources Report, 86 pp. Accessed April 27, 2022 via https://cawaterlibrary.net/wp-content/uploads/2017/05/GWU2013_Ch5_CentralCoast_Final.pdf	-	-	Approximated from Fig. 5-2 by California Department of Water Resources (2015f)
**Santa Margarita Valley**	-	California Department of Water Resources (2015e). California's groundwater update 2013 - Chapter 6: South Coast Hydrologic Region. California Department of Water Resources Report, 84 pp. Accessed April 27, 2022 via https://cawaterlibrary.net/wp-content/uploads/2017/05/GWU2013_Ch6_SouthCoast_Final.pdf	-	-	Approximated from Fig. 6-2 by California Department of Water Resources (2015e)

Aquifer system	Broader aquifer system	Reference A	Reference B	Reference C	Steps to delineate
**Santa Maria Basin and Nipomo Valley**	-	Hughes, J. L. (1977). Evaluation of Ground-water Quality in the Santa Maria Valley, California. US Geological Survey, Water Resources Division Report 76-128,. 77 pp. Accessed March 7, 2021 from https://pubs.usgs.gov/wri/1976/0128/report.pdf	Worts, G. F., Thomasson, H. G. (1951). Geology and ground-water resources of the Santa Maria Valley Area, California. US Geological Survey Water Supply Paper 1000, 177 pp. Accessed March 7, 2021 from https://pubs.usgs.gov/wsp/1000/report.pdf	-	Approximated from Fig. 1 of Hughes (1977) and Fig. 1 by Worts and Thomasson (1951)
**Santa Maria Valley**	-	California Department of Water Resources (2015e). California's groundwater update 2013 - Chapter 6: South Coast Hydrologic Region. California Department of Water Resources Report, 84 pp. Accessed April 27, 2022 via https://cawaterlibrary.net/wp-content/uploads/2017/05/GWU2013_Ch6_SouthCoast_Final.pdf	-	-	Approximated from Fig. 6-2 by California Department of Water Resources (2015e)
**Santa Monica Coastal Plain**	Los Angeles Basin**	Land, M., Reichard, E.G., Crawford, S.M., Everett, R.R., Newhouse, M.W., Williams, C.F. (2004). Ground-water Quality of Coastal Aquifer Systems in the West Coast Basin, Los Angeles County, California, 1999–2002. U.S. Geological Survey Scientific Investigations Report 2004-5067, 88 pp. Accessed February 15, 2021 from https://pubs.usgs.gov/sir/2004/5067/sir2004-5067.pdf	Fram, M. S., Belitz, K. (2008). Groundwater quality in the Coastal Los Angeles Basin, California; United States Geological Survey Fact Sheet 2012-3096. Accessed February 14, 2021 from https://pubs.er.usgs.gov/publication/70039952.	Reichard, E. G., Land, M., Crawford, S.M., Johnson, T.D., Everett, R.R., Kulshan, T.V., Ponti, D.J., Halford, K.L., Johnson, T.A., Paybins, K.S., Nishikawa, T. (2003). Geohydrology, Geochemistry, and Ground-Water Simulation Optimization of the Central and West Coast Basins, Los Angeles County, California. USGS Numbered Series, 2003–4065. Accessed November 28, 2021 from	Approximated from Fig. 1 by Land et al. (2004), Fig. 6 by Reichard et al. (2003) and map on page 1 of Fram and Belitz (2008)

Aquifer system	Broader aquifer system	Reference A	Reference B	Reference C	Steps to delineate
				https://pubs.usgs.gov/wri/wrir034065/wrir034065.pdf	
**Santa Rosa Valley**	-	Valin, Z.C., McLaughlin, R. J. (2005). Locations and data for water wells of the Santa Rosa Valley, Sonoma County, California. U.S. Geological Survey Open File Report 2005-1318. 16 pp. Accessed March 12, 2021 from https://pubs.usgs.gov/of/2005/1318/of2005-1318.pdf	Cardwell, G. T. (1958). Geology and ground water in the Santa Rosa and Petaluma Valley areas, Sonoma County, California. U.S. Geological Survey Water-Supply Paper 1427, 284 pp. Accessed November 29, 2021 from https://pubs.usgs.gov/wsp/1427/report.pdf	Woolfenden, L.R., Nishikawa, T. (2014). Simulation of groundwater and surface-water resources of the Santa Rosa Plain watershed, Sonoma County, California. US Geological Survey Scientific Investigations Report 2014–5052, 292 pp. Accessed May 19, 2022 via https://pubs.usgs.gov/sir/2014/5052/pdf/sir2014-5052.pdf	Approximated from map on page 2 of Valin and McLaughlin (2005), Fig. 1 by Woolfenden and Nishikawa (2014), and Fig. 1 by Cardwell (1958)
**Santiago Coastal Aquifer**	-	Muir, M.A.K., Martinez, A.A. (2018). Preliminary Assessment of Water Resources including Climate Considerations for the Los Cabos and La Paz Municipalities in the State of Baja California Sur, Mexico. Conference: International Water Association Water, Energy and Climate Conference (May 21 to 23, 2024), Mexico City, 13 pp. Accessed May 6, 2021 from https://arctic.ucalgary.ca/sites/default/files/April18-IWA-FinalConferenceVersion-MAKMuir.pdf	-	-	Approximated from maps on page 5 of Muir and Martinez (2018).
**Santo Domingo Coastal Aquifer**	-	Cardona, A., Carrillo-Rivera, J. J., Huizar-Alvarez, R., Graniel-Castro, E. (2004). Salinization in coastal aquifers of arid	Wurl, J., Imaz-Lamadrid, M. A. (2018). Coupled surface water and groundwater model to design managed aquifer recharge for the	-	Approximated from Fig. 1 by Cardona et al. (2004) and Wurl and Imaz-Lamadrid (2018).

Aquifer system	Broader aquifer system	Reference A	Reference B	Reference C	Steps to delineate
		zones: an example from Santo Domingo, Baja California Sur, Mexico. Environmental Geology, 45, 350-366.	valley of Santo Domingo, BCS, Mexico. Sustainable Water Resources Management, 4, 361-369.		
**Sarab Plain**	-	Amirkhizi, M.T., Delirhasannia, R., Haghighatjou, P., Majnooni Heris, A. (2019). Determining Water Quality of Agricultural Wells for Use in Pressurized Irrigation Systems of Sarab Plain, Iran. Journal of Water and Soil Knowledge, 29, 14 pp. Accessed June 9, 2021 from https://water-soil.tabrizu.ac.ir/mobile/article_9328_8172ff8f570310f3b6e5a7fde7846b06.pdf?lang=en	-	-	Approximated from Fig. 2 by Amirkhizi et al. (2019)
**Saravan Basin**	-	Hosseini, S. M., Parizi, E., Ataie-Ashtiani, B., Simmons, C. T. (2019). Assessment of sustainable groundwater resources management using integrated environmental index: Case studies across Iran. Science of the Total Environment, 676, 792-810.	-	-	Approximated from Fig. 6 by Hosseini et al. (2019)
**Sarayan Plain**	-	Mohammad Rezapour Tabari, M., Kabiri Samani, M. (2019). Groundwater Quality Assessment Using Entropy Weighted Osculating Value and Set Pair Analysis Methods (Case study, SARAYAN plain). Journal of Environmental	-	-	Approximated from Fig. 1 by Mohammad Rezapour Tabari and Kabiri Samani (2019)

Aquifer system	Broader aquifer system	Reference A	Reference B	Reference C	Steps to delineate
		Science and Technology, 21, 99-112.			
**Sarbisheh Basin**	-	Kardan Moghaddam, H., Dehghani, M., Rahimzadeh Kivi, Z., Kardan Moghaddam, H., Hashemi, S. R. (2017). Efficiency assessment of AHP and fuzzy logic methods in suitability mapping for artificial recharging (Case study: Sarbisheh basin, Southern Khorasan, Iran). Water Harvesting Research, 2, 57-67.	-	-	Approximated from Fig. 1 by Kardan Moghaddam et al. (2017)
**Sarchahan Aquifer**	-	Hosseini Poor, H., Ghaioomeyan, J., Ghasemi, A. R., Choopani, S. (2010). Investigating salt sources in Sarchahan aquifer in Hormozghan province using ion ratios. Journal of Watershed Engineering and Management, 16 pp. Accessed June 11, 2021 from https://jwem.areeo.ac.ir/article_101819_914d3ec834c0895469dad1a13b184903.pdf?lang=en	-	-	Approximated from Fig. 1 by Hosseini Poor et al. (2010)
**Sarcobatus Flat**	-	Lopes, T.J., Evetts. D.M. (2005). Ground-Water pumpage and artificial recharge estimates for calendar year 2000 and average annual natural recharge and interbasin flow by hydrographic area, Nevada. Accessed April 25, 2022 via	Harrill, J.R., Prudic, D.E. (1998). Aquifer systems in the Great Basin region of Nevada, Utah, and adjacent states; summary report. US Geological Survey Professional Paper 1409-A, 75 pp. Accessed April 26, 2022 via https://pubs.usgs.gov/pp/1409a/report.pdf	-	Approximated from Fig. 1 by Lopes and Evetts (2005), and Fig. 2 by Harrill and Prudic (1998). Well completion records, physiography and drainage were used to delinate areas.

Aquifer system	Broader aquifer system	Reference A	Reference B	Reference C	Steps to delineate
		https://pubs.usgs.gov/sir/2004/5239/sir2004-5239.pdf			
**Sardis-Vedder Aquifer**	-	City of Chilliwack (2021). Groundwater Protection. Webpage accessed March 12, 2021 from https://www.chilliwack.com/main/page.cfm?id=205	-	-	Map of aquifer from City of Chilliwack webpage (City of Chilliwack, 2021).
**Sari-Neka Plain**	-	Motevalli, A., Pourghasemi, H. R., Hashemi, H., Gholami, V. (2019). Assessing the vulnerability of groundwater to salinization using GIS-based data-mining techniques in a coastal aquifer. In Spatial Modeling in GIS and R for Earth and Environmental Sciences, pp. 547-571. Elsevier (eds. Pourghasemi, H.R., Gokceoglu, C.)	Nasiri, M., Hamidi, M., Kardan Moghaddam, H. (2019). Investigation of Groundwater Quantitative and Qualitative Variations Trends (Case study: Sari-Neka aquifer). Journal of Auifer and Qanat, 2, 109-122.	Khosravi, K., Nejad Roshan, M. H., Safari, A. (2017). Assessment of geostatistical methods for determining distribution patterns of groundwater resources in Sari-Neka coastal plain, northern Iran. Environmental Resources Research, 5, 124-134.	Approximated from Fig. 1 by Motevalli et al. (2019), Fig. 1 by Khosravi et al. (2017), and Fig. 1 by Nasiri et al. (2019)
**Sarvestan Plain**	-	Cheraghi, S. A. M., Nagafi, B., Shajari, S., Javan, M. (2020). The Trend of Changes in Groundwater Quantity and Quality in the Sarvestan Plain of Fars Province. Watershed Management Researches Journal, 33, 82-96.	-	-	Approximated from Fig. 1 by Cheraghi et al. (2020)
**Sawtooth Valley and Bear Valley**	-	Graham, W.G., Campbell, L.J. (1981). Groundwater resources of Idaho. Idaho Department of Water Resources Report, 61 pp. Accessed March 23, 2021 from https://idwr.idaho.gov/files/publications/198108-MISC-GW-Resources-ID.pdf	-	-	Approximated from Plate 1 of Graham and Campbell (1981).

Aquifer system	Broader aquifer system	Reference A	Reference B	Reference C	Steps to delineate
**Scott Coastal Plain**	-	Ali, R., McFarlane, D., Varma, S., Dawes, W., Emelyanova, I., Hodgson, G., Charles, S. (2012). Potential climate change impacts on groundwater resources of south-western Australia. Journal of Hydrology, 475, 456-472.	-	-	Approximated from Fig. 1b by Ali et al. (2012)
**Scott Valley**	-	Wood, P. R. (1960). Geology and ground-water features of the Butte Valley region, Siskiyou County, California. Geological Survey Water-Supply Paper 1491, 155 pp. Accessed March 20, 2021 from https://pdfs.semanticscholar.org/a448/a58a3c1ac120d400d26f75b74512f0a868a4.pdf	-	-	Approximated from Fig. 1 of Wood (1960)
**Scott Valley and Mann Creek**	-	Graham, W.G., Campbell, L.J. (1981). Groundwater resources of Idaho. Idaho Department of Water Resources Report, 61 pp. Accessed March 23, 2021 from https://idwr.idaho.gov/files/publications/198108-MISC-GW-Resources-ID.pdf	-	-	Approximated from Plate 1 of Graham and Campbell (1981).
**Scotts Valley**	-	California Department of Water Resources (2015j). California's groundwater update 2013 - Chapter 3: North Coast Hydrologic Region. California Department of Water Resources Report, 80 pp. Accessed April 27, 2022 via https://water.ca.gov/-	-	-	Approximated from Fig. 3-2 by California Department of Water Resources (2015j)

Aquifer system	Broader aquifer system	Reference A	Reference B	Reference C	Steps to delineate
		/media/DWR-Website/Web-Pages/Programs/Groundwater-Management/Bulletin-118/Files/Statewide-Reports/GWU2013_Ch3_NorthCoast_Final.pdf			
**Sea Island**	Floridan Aquifer System	Williams, L.J., Kuniansky, E.L. (2016). Revised hydrogeologic framework of the Floridan aquifer system in Florida and parts of Georgia, Alabama, and South Carolina. U.S. Geological Survey Professional Paper 1807, 140 pp. Accessed March 31, 2021 from https://pubs.usgs.gov/pp/1807/pdf/pp1807.pdf	Brooks, H. K. (1981). Physiographic divisions of Florida. Report for the Florida Cooperative Extension Service Institute of Food and Agricultural Sciences, University of Florida, 12 pp.	Clark, W. Z., Zisa, A. C. (1976) Physiographic map of Georgia: Georgia Department of Natural Resources, 1 sheet. Accessed March 31, 2021 from https://epd.georgia.gov/document/publication/sm-4-physiographic-map-georgia-12000000-1988/download	Approximated from physiographic provinces for Georgia and Florida by Clark and Zisa (1976) and Brooks (1981). Broader Florida Aquifer System from Williams and Kuniansky (2016).
**Searles Valley**	-	California Department of Water Resources (2015b). California's groundwater update 2013 - Chapter 11: South Lahontan Hydrologic Region. California Department of Water Resources Report, 80 pp. Accessed April 27, 2022 via https://water.ca.gov/-/media/DWR-Website/Web-Pages/Programs/Groundwater-Management/Data-and-Tools/Files/Statewide-Reports/California-Groundwater-Update-2013/California-Groundwater-Update-2013---Chapter-11---South-Lahontan.pdf	-	-	Approximated from Fig. 11-2 by California Department of Water Resources (2015b) and Fig. 1 by Chapman et al. (2022)
**Seaspray Region in**	Gippsland Basin	Currell, M., Banfield, D., Cartwright, I., Cendón, D.	Varma, S., Michael, K. (2012). Impact of multi-	Beverly, C., Hocking, M., Cheng, X., O’Neil, C.,	Approximated from Fig. 4 by Beverly et al. (2015), Fig. 1 by

Aquifer system	Broader aquifer system	Reference A	Reference B	Reference C	Steps to delineate
**Southwestern Gippsland Basin**		I. (2017). Geochemical indicators of the origins and evolution of methane in groundwater: Gippsland Basin, Australia. Environmental Science and Pollution Research, 24, 13168-13183.	purpose aquifer utilisation on a variable-density groundwater flow system in the Gippsland Basin, Australia. Hydrogeology Journal, 20, 119-134.	Schroers, R., Baker, S. (2015).The Gippsland groundwater model. Technical Report, 409 pp. Accessed October 8, 2021 from https://www.parliament.vic.gov.au/images/stories/committees/EPC/Other_documents/G3_-_Gippsland_groundwater_model_report_June_2015_2.pdf	Varma and Michael (2012), and Fig. 1 by Currell et al. (2017)
**Secret Valley**	-	California Department of Water Resources (2015i). California's groundwater update 2013 - Chapter 7: Sacramento River Hydrologic Region. California Department of Water Resources Report, 126 pp. Accessed April 27, 2022 via https://water.ca.gov/-/media/DWR-Website/Web-Pages/Programs/Groundwater-Management/Bulletin-118/Files/Statewide-Reports/GWU2013_Ch7_SacramentoRiver_Final.pdf	-	-	Approximated from Fig. 7-2 by California Department of Water Resources (2015i)
**Sedeh Basin**	-	Hosseini, S. M., Parizi, E., Ataie-Ashtiani, B., Simmons, C. T. (2019). Assessment of sustainable groundwater resources management using integrated environmental index: Case studies across Iran. Science of the Total Environment, 676, 792-810.	-	-	Approximated from Fig. 6 by Hosseini et al. (2019)

Aquifer system	Broader aquifer system	Reference A	Reference B	Reference C	Steps to delineate
**Sefid-Dasht Aquifer**	-	Khodabakhshi, N., Heidarzadeh, N., Asadollahfardi, G. (2017). Vulnerability Assessment of an Aquifer using Modified GIS-based Methods. Journal-American Water Works Association, 109(5), E170-E182.	-	-	Approximated from Fig. 1 by Khodabakhshi et al. (2017)
**Semelghan Plain**	-	Hosseini, S. M., Parizi, E., Ataie-Ashtiani, B., Simmons, C. T. (2019). Assessment of sustainable groundwater resources management using integrated environmental index: Case studies across Iran. Science of the Total Environment, 676, 792-810.	-	-	Approximated from Fig. 6 by Hosseini et al. (2019)
**Semirom Basin**	-	Parizi, E., Hosseini, S. M., Ataie-Ashtiani, B., Simmons, C. T. (2020). Normalized difference vegetation index as the dominant predicting factor of groundwater recharge in phreatic aquifers: case studies across Iran. Scientific Reports, 10, 17473.	-	-	Approximated from tabulated coordinates in the Supplementary Information within Parizi et al. (2020)
**Semnan and Sorkhe Aquifers**	-	Ghafari, S., Banihabib, M. E., Javadi, S. (2020). A framework to assess the impact of a hydraulic removing system of contaminate infiltration from a river into an aquifer (case study: Semnan aquifer). Groundwater for	Hosseini, S. M., Parizi, E., Ataie-Ashtiani, B., Simmons, C. T. (2019). Assessment of sustainable groundwater resources management using integrated environmental index: Case studies across Iran. Science of the Total	-	Approximated from Fig. 1 by Ghafari et al. (2020) and from Fig. 6 by Hosseini et al. (2019)

Aquifer system	Broader aquifer system	Reference A	Reference B	Reference C	Steps to delineate
		Sustainable Development, 10, 100301.	Environment, 676, 792-810.		
**Sendai River Basin**	-	Yoshioka, Y., Yoshioka, H. (2022). Spatiotemporal variability of hydrogen stable isotopes at a local scale in shallow groundwater during the warm season in Tottori Prefecture, Japan. Hydrological Research Letters, 16, 25-31.	-	-	Approximated from Fig. 1 by Yoshioka and Yoshioka (2022)
**Serranias de Cuenca Aquifer**	-	Moratilla, F. E., Pérez, C. M. O. (2012). Aplicación de la tomografía remota térmica a la investigación de la hidrogeología y dinámica de flujos de las aguas subterráneas de la cuenca del río Júcar. Crisis y medio ambiente:¿ Oportunidad o retroceso? 98, 116 pp. Accessed May 24, 2021 from https://www.miteco.gob.es/ministerio/pags/biblioteca/revistas/pdf_AM%5CAMBIENTA_101.pdf#page=100	-	-	Approximated from Fig. 3 by Moratilla and Pérez (2012)
**Sevier Desert**	-	Mower, R. W., Feltis, R. D. (1968). Ground-water hydrology of the Sevier Desert, Utah. US Geological Survey, Water Supply Paper 1854, 88 pp. Accessed March 7, 2021 from https://pubs.usgs.gov/wsp/1854/report.pdf	-	-	Approximated from Fig. 1 of Mower and Feltis (1968)
**Seville-Carmona and Aljarafe and**	Guadalquivir Basin	Vazquez, J. G., Grande, J. A., Barragán, F. J., Ocaña, J. A., De La Torre, M. L.	-	-	Approximated from Fig. 1 by Vazquez et al. (2005)

Aquifer system	Broader aquifer system	Reference A	Reference B	Reference C	Steps to delineate
**Guadalquivir Alluvial Aquifers**		(2005). Nitrate accumulation and other components of the groundwater in relation to cropping system in an aquifer in Southwestern Spain. Water Resources Management, 19, 1-22.			
**Seyedan-Farooq Aquifer**	-	Mahmoodlu, M., Heshmatpour, A., Jandaghi, N., Zare, A., Mehrabi, H. (2018). Hydrogeochemical Assessment of Groundwater Quality: Seyedan-Farooq Aquifer, Fars Province. Iranian Journal of Ecohydrology, 5, 1241-1253.	-	-	Approximated from Fig. 1 by Mahmoodlu et al. (2018)
**Seymour-Blaine Aquifer System**	-	Bruun, B., Jackson, K., Lake, P., Walker, J. (2016). Texas Aquifers Study. Texas Water Development Board Report. 336 pp. Accessed April 1, 2021 from https://www.twdb.texas.gov/groundwater/docs/studies/TexasAquifersStudy_2016.pdf#page=89	Chastain-Howley, A., Dean, K.E., Spear, A.A. (2004). Groundwater Availability Model for the Seymour Aquifer, Texas Water Development Board Report, 533 pp. Accessed April 5, 2021 from https://www.twdb.texas.gov/groundwater/models/gam/symr/symr.asp	-	Approximated from Fig. 6-33 by Bruun et al. (2016) and map by Chastain-Howley et al. (2004) available at https://www.twdb.texas.gov/groundwater/models/gam/symr/symr.asp (Accessed April 5, 2021).
**Shah Gheyb Basin**	-	Parizi, E., Hosseini, S. M., Ataie-Ashtiani, B., Simmons, C. T. (2020). Normalized difference vegetation index as the dominant predicting factor of groundwater recharge in phreatic aquifers: case studies across Iran. Scientific Reports, 10, 17473.	-	-	Approximated from tabulated coordinates in the Supplementary Information within Parizi et al. (2020) and physiographic and drainage patterns

Aquifer system	Broader aquifer system	Reference A	Reference B	Reference C	Steps to delineate
**Shahdad Basin**	-	Hosseini, S. M., Parizi, E., Ataie-Ashtiani, B., Simmons, C. T. (2019). Assessment of sustainable groundwater resources management using integrated environmental index: Case studies across Iran. Science of the Total Environment, 676, 792-810.	-	-	Approximated from Fig. 6 by Hosseini et al. (2019)
**Shahindej Basin**	-	Hosseini, S. M., Parizi, E., Ataie-Ashtiani, B., Simmons, C. T. (2019). Assessment of sustainable groundwater resources management using integrated environmental index: Case studies across Iran. Science of the Total Environment, 676, 792-810.	-	-	Approximated from Fig. 6 by Hosseini et al. (2019)
**Shahr-Babak Plain**	-	Jahanshahi, A., Moghaddamnia, A., Khosravi, H. (2015). Assessment of Desertification Density Using IMDPA Model (Case study: Shahr-Babak plain, Kerman Province). Journal of Range and Watershed Managment, 68, 247-267.	-	-	Approximated from
**Shahrekord Plain**	-	Radfar, M., Van Camp, M., Walraevens, K. (2013). Drought impacts on long-term hydrodynamic behavior of groundwater in the tertiary–quaternary aquifer system of Shahrekord Plain, Iran.	Lalehzari, R., Tabatabaei, S. H. (2015). Simulating the impact of subsurface dam construction on the change of nitrate distribution. Environmental Earth Sciences, 74(4), 3241-3249.	-	Approximated from Fig. 1 by Radfar et al. (2013) and Fig. 1 by Lalehzari and Tabatabaei (2015).

Aquifer system	Broader aquifer system	Reference A	Reference B	Reference C	Steps to delineate
		Environmental earth sciences, 70, 927-942.			
**Shahreza Plain**	-	Hosseini, S. M., Parizi, E., Ataie-Ashtiani, B., Simmons, C. T. (2019). Assessment of sustainable groundwater resources management using integrated environmental index: Case studies across Iran. Science of the Total Environment, 676, 792-810.	Madani, K., Mariño, M. A. (2009). System dynamics analysis for managing Iran's Zayandeh-Rud river basin. Water Resources Management, 23, 2163-2187.	Salemi, H.R., Mamanpoush, A.R., Miranzadeh, M., Akbari, M., Torabi, M., Toomanian, N., Murray-Rust, H., Droogers, P., Sally, H., Gieske, A. (2000). Water management for sustainable irrigated agriculture in the Zayandeh Rud Basin, Esfahan Province, Iran Report by Iranian Agricultural Engineering Research Institute, Esfahan Agricultural Research Center, and the International Water Management Institute, Research Report Number 1	Approximated from Fig. 6 by Hosseini et al. (2019), Fig. 1 by Madani and Mariño (2009), and Fig. 1 by Salemi et al. (2000)
**Shahroud Plain**	-	Arabameri, A., Rezaei, K., Cerda, A., Lombardo, L., Rodrigo-Comino, J. (2019). GIS-based groundwater potential mapping in Shahroud plain, Iran. A comparison among statistical (bivariate and multivariate), data mining and MCDM approaches. Science of the total environment, 658, 160-177.	-	-	Approximated from Fig. 1 by Arabameri et al. (2019)
**Shamil Aquifer**	-	Bazrafshan, O., Parandin, F., Farokhzadeh, B. (2016). Assessment of hydro-meteorological drought effects on groundwater resources in Hormozgan	-	-	Approximated from map on page 1571 of Bazrafshan et al. (2016)

Aquifer system	Broader aquifer system	Reference A	Reference B	Reference C	Steps to delineate
		region-South of Iran. Ecopersia, 4, 1569-1584.			
**Shandan Basin and Middle Heihe Reaches**	Heihe Basin	Zhou, J., Hu, B. X., Cheng, G., Wang, G., Li, X. (2011). Development of a three-dimensional watershed modelling system for water cycle in the middle part of the Heihe rivershed, in the west of China. Hydrological Processes, 25, 1964-1978.	Xue, Z., Du, P., Li, J., Su, H. (2017). Sparse graph regularization for robust crop mapping using hyperspectral remotely sensed imagery with very few in situ data. ISPRS Journal of Photogrammetry and Remote Sensing, 124, 1-15.	-	Approximated from Fig. 1 by Zhou et al. (2011) and Fig. 1 by Xue et al. (2017)
**Sharif Abab and Masileh Aquifers**	-	Hosseini, S. M., Parizi, E., Ataie-Ashtiani, B., Simmons, C. T. (2019). Assessment of sustainable groundwater resources management using integrated environmental index: Case studies across Iran. Science of the Total Environment, 676, 792-810.	-	-	Approximated from Fig. 6 by Hosseini et al. (2019)
**Shasta Valley**	-	Wood, P. R. (1960). Geology and ground-water features of the Butte Valley region, Siskiyou County, California. Geological Survey Water-Supply Paper 1491, 155 pp. Accessed March 20, 2021 from https://pdfs.semanticscholar.org/a448/a58a3c1ac120d400d26f75b74512f0a868a4.pdf	-	-	Approximated from Fig. 1 of Wood (1960)
**Shawnee Hills**	Interior Low Plateaus	Miller, J.A. (1995). Ground Water Atlas of the United States: Segment 10, Illinois, Indiana, Kentucky, Ohio, Tennessee. U.S. Geological Survey	Taylor, C.J., Nelson Jr., H.L. (2008). A compilation of provisional karst geospatial data for the Interior Low Plateaus physiographic region, central United	Leighton, M. M., Ekblaw, G. E., Horberg, L. (1948). Physiographic divisions of Illinois. The Journal of Geology, 56, 16-33.	Broader Interior Low Plateaus outline approximated for Tennessee, Illinois, Kentucky, Indiana from Fig. 78 of Miller (1990) and Fig. 1 of Taylor and Nelson (2008). Shawnee Hills

Aquifer system	Broader aquifer system	Reference A	Reference B	Reference C	Steps to delineate
		Hydrologic Atlas 730-K, 30 pp. Accessed April 11, 2021 from https://pubs.usgs.gov/ha/730k/report.pdf	States: U.S. Geological Survey Data Series 339, 26 pp. Accessed April 11, 2021 from https://pubs.usgs.gov/ds/339/pdf/ds339_web.pdf		approximated from Figs. 1 and 2 of Leighton et al. (1948)
**Shazand Plain**	-	Taheri Zangi, S., Vaezihir, A. (2020). Vulnerability of Shazand Plain Subsidence Caused by Groundwater Level Reduction Using Weighting Model and Its Validation Analysis Using Radar Interferometry. Iranian Journal of Ecohydrology, 7, 183-194.	-	-	Approximated from Fig. 1 by Taheri Zangi and Vaezihir (2020)
**Shigenobu-gawa Alluvial Fan**	-	Miyazaki, S., Hasegawa, S., Kayaki, T., Osamu, W. (2008). Hydrogeology of the Shigenobu-gawa Alluvial Fan, Ehime Pref. Shikoku, Japan. In: Hydro-environments of Alluvial Fans in Japan, Monograph, 36th IAH Congress, 2008, Toyama. Accessed April 12, 2023 via https://www.yachiyo-eng.co.jp/case/papers/pdf/2008_18_hasegawa.pdf	-	-	Approximated from Miyazaki et al. (2008)
**Shihezi Area**	-	Zeng, Y., Zhou, Y., Zhou, J., Jia, R., Wu, J. (2018). Distribution and enrichment factors of high-arsenic groundwater in Inland Arid area of PR China: a case study of the Shihezi area, Xinjiang. Exposure and Health, 10, 1-13.	-	-	Approximated from Fig. 2 by Zeng et al. (2018)
**Shiramin Basin**	-	Parizi, E., Hosseini, S. M., Ataie-Ashtiani, B.,	-	-	Approximated from tabulated coordinates in the Supplementary

Aquifer system	Broader aquifer system	Reference A	Reference B	Reference C	Steps to delineate
		Simmons, C. T. (2020). Normalized difference vegetation index as the dominant predicting factor of groundwater recharge in phreatic aquifers: case studies across Iran. Scientific Reports, 10, 17473.			Information within Parizi et al. (2020) and physiographic and drainage patterns
**Shiraz Plain**	-	Baghapour, M. A., Nobandegani, A. F., Talebbeydokhti, N., Bagherzadeh, S., Nadiri, A. A., Gharekhani, M., Chitsazan, N. (2016). Optimization of DRASTIC method by artificial neural network, nitrate vulnerability index, and composite DRASTIC models to assess groundwater vulnerability for unconfined aquifer of Shiraz Plain, Iran. Journal of Environmental Health Science and Engineering, 14, 1-16.	Khairy, H., Janardhana, M. R. (2013). Hydrogeochemical features of groundwater of semi-confined coastal aquifer in Amol–Ghaemshahr plain, Mazandaran Province, Northern Iran. Environmental Monitoring and Assessment, 185, 9237-9264.	-	Approximated from Fig. 1 by Baghapour et al. (2016) and Fig. 1 by Khairy and Janardhana (2013)
**Shirvan Plain**	Shirvan-Quchan Plain	Ghorbani, H., Sadabad, S. M. (2010). Annual Changes in Some Qualitative Parameters of Groundwater in Shirvan Plain North East of Iran. World Academy of Science, Engineering and Technology, 68, 949-952.	Parizi, E., Hosseini, S. M., Ataie-Ashtiani, B., Simmons, C. T. (2020). Normalized difference vegetation index as the dominant predicting factor of groundwater recharge in phreatic aquifers: case studies across Iran. Scientific Reports, 10, 17473.	-	Approximated from description in section II.A. by Ghorbani and Sadabad (2010) and Fig. 2 by Parizi et al. (2020)
**Shomali Basin**	Kabul Basin	Saffi, M.H. (2019). National alarming on groundwater natural storage depletion and water quality	Mack, T. J., Chornack, M. P., Taher, M. R. (2013). Groundwater-level trends and implications for	-	Approximated from Fig. 3.2 by Sadid (2020) and Fig. 1 by Mack et al. (2013)

Aquifer system	Broader aquifer system	Reference A	Reference B	Reference C	Steps to delineate
		deterioration of Kabul City and immediate response to the drinking water crises. Scientific INvestigation Report in Afghanistan, DACAAR Report, 88 pp.	sustainable water use in the Kabul Basin, Afghanistan. Environment Systems and Decisions, 33, 457-467.		
**Shoqan Plain**	-	Hosseini, S. M., Parizi, E., Ataie-Ashtiani, B., Simmons, C. T. (2019). Assessment of sustainable groundwater resources management using integrated environmental index: Case studies across Iran. Science of the Total Environment, 676, 792-810.	-	-	Approximated from Fig. 6 by Hosseini et al. (2019)
**Shush Plain**	Dezful-Andimeshk Plain	Mirzaei, R., Sakizadeh, M. (2016). Comparison of interpolation methods for the estimation of groundwater contamination in Andimeshk-Shush Plain, Southwest of Iran. Environmental Science and Pollution Research, 23, 2758-2769.	Ebrahimi Varzane, S., Zarei, H., TishehZan, P., Akhondali, A. M. (2019). Evaluation of Groundwater-Surface Water Interaction by Using Cluster Analysis (Case Study: Western Part of Dezful-Andimeshk Plain). Iran-Water Resources Research, 15, 246-257.	-	Approximated from Fig. 1 by Mirzaei and Sakizadeh (2016) and Fig. 2 by Ebrahimi Varzane et al. (2019)
**Sib-Souran Basin**	-	Hosseini, S. M., Parizi, E., Ataie-Ashtiani, B., Simmons, C. T. (2019). Assessment of sustainable groundwater resources management using integrated environmental index: Case studies across Iran. Science of the Total Environment, 676, 792-810.	Parizi, E., Hosseini, S. M., Ataie-Ashtiani, B., Simmons, C. T. (2020). Normalized difference vegetation index as the dominant predicting factor of groundwater recharge in phreatic aquifers: case studies across Iran. Scientific Reports, 10, 17473.	-	Approximated from Fig. 6 by Hosseini et al. (2019) and Fig. 2 by Parizi et al. (2020)
**Sidon Basin**	-	Parizi, E., Hosseini, S. M., Ataie-Ashtiani, B., Simmons, C. T. (2020).	-	-	Approximated from tabulated coordinates in the Supplementary Information within Parizi et al.

Aquifer system	Broader aquifer system	Reference A	Reference B	Reference C	Steps to delineate
		Normalized difference vegetation index as the dominant predicting factor of groundwater recharge in phreatic aquifers: case studies across Iran. Scientific Reports, 10, 17473.			(2020) and physiographic and drainage patterns
**Sief Abad e-Laghar Basin**	-	Hosseini, S. M., Parizi, E., Ataie-Ashtiani, B., Simmons, C. T. (2019). Assessment of sustainable groundwater resources management using integrated environmental index: Case studies across Iran. Science of the Total Environment, 676, 792-810.	-	-	Approximated from Fig. 6 by Hosseini et al. (2019)
**Sierra de Altomira Aquifer**	Upper Guadiana Basin	Martínez-Santos, P., Castaño-Castaño, S., Hernández-Espriú, A. (2018). Revisiting groundwater overdraft based on the experience of the Mancha Occidental Aquifer, Spain. Hydrogeology Journal, 26, 1083-1097.	-	-	Approximated from Fig. 1 by Martínez-Santos et al. (2018)
**Sierra de Crevillente Aquifer**	-	Pulido-Bosch, A., Morell, I., Andreu, J. M. (1995). Hydrogeochemical effects of groundwater mining of the Sierra de Crevillente Aquifer (Alicante, Spain). Environmental Geology, 26, 232-239.	-	-	Approximated from Fig. 1 by Pulido-Bosch et al. (1995).
**Sierra Espuna Aquifer**	-	Calatrava, J., Guillem, A., Martínez-Granados, D. (2011). Analysis of alternatives to eliminate	-	-	Approximated from Map 2 by Calatrava et al. (2011)

Aquifer system	Broader aquifer system	Reference A	Reference B	Reference C	Steps to delineate
		aquifer overdraft in the Guadalentín Valley, SE Spain. Economía Agraria y Recursos Naturales-Agricultural and Resource Economics, 11, 33-62.			
**Sierra Grossa Karst Aquifer System**	-	Pulido-Bosch, A. (2020). Principles of Karst Hydrogeology: Conceptual Models, Time Series Analysis, Hydrogeochemistry and Groundwater Exploitation. Springer Nature, 379 pp. Accessed May 23, 2021 from https://link.springer.com/content/pdf/10.1007/978-3-030-55370-8.pdf	-	-	Approximated from Fig. 6.27 by Pulido-Bosch (2020).
**Sierra Valley**	-	Bachand, P.A.M., Birt, K.S., Bachand, S.M. (2020). Groundwater relationships to pumping, precipitation and geology in high-elevation basin, Sierra Valley, CA. Technical Report to Report to Feather River Land. Sierra Valley, CA. 66 pp. Accessed March 21, 2021 from http://aquaticcommons.org/27004/1/Sierra%20Valley%20Recharge%20FINAL%202020-03-10%20SECURE.pdf	-	-	Approximated from Fig. 4 of Bachand et al. (2020)
**Sikh Darengon Basin**	-	Hosseini, S. M., Parizi, E., Ataie-Ashtiani, B., Simmons, C. T. (2019). Assessment of sustainable groundwater resources management using	-	-	Approximated from Fig. 6 by Hosseini et al. (2019)

Aquifer system	Broader aquifer system	Reference A	Reference B	Reference C	Steps to delineate
		integrated environmental index: Case studies across Iran. Science of the Total Environment, 676, 792-810.			
**Silurian and Overlying Unconsolidated Aquifers**	Southern Ontario Paleozoic Aquifer System	Sharpe, D.R., Piggott, A., Carter, T., Gerber, R.E., MacRitchie, S.M., de Loë, R.C., Strynatka, S., Zwiers, G., Rivera, A. (2013). Southern Ontario hydrogeological region. in: Canada's Groundwater Resources, pp. 444-499. Fitzhenry and Whiteside Limited, Markham, Ontario, Canada. Accessed May 5, 2021 from https://www.researchgate.net/profile/David-Sharpe-2/publication/284034021_Southern_Ontario_hydrogeological_region/links/5f8f462192851c14bcd85341/Southern-Ontario-hydrogeological-region.pdf	-	-	Approximated from Figs. 12.1 and 12.5 of Sharpe et al. (2013)
**Silurian-Devonian Aquifers in Northern Michigan**	-	Olcott, P.G. (1992). Groundwater Atlas of the United States: Segment 9 Iowa, Michigan, Minnesota, Wisconsin. U.S. Geological Survey Hydrologic Atlas 730-J, 33 pp. Accessed April 12, 2021 from https://pubs.usgs.gov/ha/730j/report.pdf	-	-	Approximated from Fig. 79 by Olcott (1992).
**Simi Valley**	-	California Department of Water Resources (2015e). California's groundwater update 2013 - Chapter 6: South Coast Hydrologic	-	-	Approximated from Fig. 6-2 by California Department of Water Resources (2015e)

Aquifer system	Broader aquifer system	Reference A	Reference B	Reference C	Steps to delineate
		Region. California Department of Water Resources Report, 84 pp. Accessed April 27, 2022 via https://cawaterlibrary.net/wp-content/uploads/2017/05/GWU2013_Ch6_SouthCoast_Final.pdf			
**Sinclinal de Calasparra Aquifer**	-	Rupérez-Moreno, C., Senent-Aparicio, J., Martinez-Vicente, D., García-Aróstegui, J. L., Calvo-Rubio, F. C., Pérez-Sánchez, J. (2017). Sustainability of irrigated agriculture with overexploited aquifers: The case of Segura basin (SE, Spain). Agricultural Water Management, 182, 67-76.	-	-	Approximated from Fig. 1 by Rupérez-Moreno et al. (2017)
**Sinclinal de la Higuera**	-	Rupérez-Moreno, C., Senent-Aparicio, J., Martinez-Vicente, D., García-Aróstegui, J. L., Calvo-Rubio, F. C., Pérez-Sánchez, J. (2017). Sustainability of irrigated agriculture with overexploited aquifers: The case of Segura basin (SE, Spain). Agricultural Water Management, 182, 67-76.	-	-	Approximated from Fig. 1 by Rupérez-Moreno et al. (2017); aquifer boundaries expanded to nothwest based on well completion records
**Sink Valley**	-	Lopes, T.J., Evetts. D.M. (2005). Ground-Water pumpage and artificial recharge estimates for calendar year 2000 and average annual natural recharge and interbasin flow by hydrographic area,	Harrill, J.R., Prudic, D.E. (1998). Aquifer systems in the Great Basin region of Nevada, Utah, and adjacent states; summary report. US Geological Survey Professional Paper 1409-A, 75 pp. Accessed April 26,	-	Approximated from Fig. 1 by Lopes and Evetts (2005), and Fig. 2 by Harrill and Prudic (1998). Well completion records, physiography and drainage were used to delinate areas.

Aquifer system	Broader aquifer system	Reference A	Reference B	Reference C	Steps to delineate
		Nevada. Accessed April 25, 2022 via https://pubs.usgs.gov/sir/2004/5239/sir2004-5239.pdf	2022 via https://pubs.usgs.gov/pp/1409a/report.pdf		
**Sirik Basin**	-	Hosseini, S. M., Parizi, E., Ataie-Ashtiani, B., Simmons, C. T. (2019). Assessment of sustainable groundwater resources management using integrated environmental index: Case studies across Iran. Science of the Total Environment, 676, 792-810.	-	-	Approximated from Fig. 6 by Hosseini et al. (2019)
**Siriz Basin**	-	Hosseini, S. M., Parizi, E., Ataie-Ashtiani, B., Simmons, C. T. (2019). Assessment of sustainable groundwater resources management using integrated environmental index: Case studies across Iran. Science of the Total Environment, 676, 792-810.	-	-	Approximated from Fig. 6 by Hosseini et al. (2019)
**Sirjan Basin**	-	Hosseini, S. M., Parizi, E., Ataie-Ashtiani, B., Simmons, C. T. (2019). Assessment of sustainable groundwater resources management using integrated environmental index: Case studies across Iran. Science of the Total Environment, 676, 792-810.	Parizi, E., Hosseini, S. M., Ataie-Ashtiani, B., Simmons, C. T. (2020). Normalized difference vegetation index as the dominant predicting factor of groundwater recharge in phreatic aquifers: case studies across Iran. Scientific Reports, 10, 17473.	-	Approximated from Fig. 6 by Hosseini et al. (2019) and Fig. 2 by Parizi et al. (2020)
**Skull Valley**	-	Lopes, T.J., Evetts. D.M. (2005). Ground-Water pumpage and artificial recharge estimates for	Harrill, J.R., Prudic, D.E. (1998). Aquifer systems in the Great Basin region of Nevada, Utah, and adjacent	-	Approximated from Fig. 1 by Lopes and Evetts (2005), and Fig. 2 by Harrill and Prudic (1998). Well completion records, physiography

Aquifer system	Broader aquifer system	Reference A	Reference B	Reference C	Steps to delineate
		calendar year 2000 and average annual natural recharge and interbasin flow by hydrographic area, Nevada. Accessed April 25, 2022 via https://pubs.usgs.gov/sir/2004/5239/sir2004-5239.pdf	states; summary report. US Geological Survey Professional Paper 1409-A, 75 pp. Accessed April 26, 2022 via https://pubs.usgs.gov/pp/1409a/report.pdf		and drainage were used to delinate areas.
**Smith Creek Valley**	-	Lopes, T.J., Evetts. D.M. (2005). Ground-Water pumpage and artificial recharge estimates for calendar year 2000 and average annual natural recharge and interbasin flow by hydrographic area, Nevada. Accessed April 25, 2022 via https://pubs.usgs.gov/sir/2004/5239/sir2004-5239.pdf	Harrill, J.R., Prudic, D.E. (1998). Aquifer systems in the Great Basin region of Nevada, Utah, and adjacent states; summary report. US Geological Survey Professional Paper 1409-A, 75 pp. Accessed April 26, 2022 via https://pubs.usgs.gov/pp/1409a/report.pdf	-	Approximated from Fig. 1 by Lopes and Evetts (2005), and Fig. 2 by Harrill and Prudic (1998). Well completion records, physiography and drainage were used to delinate areas.
**Smith River Plain**	-	Back, W. (1957). Geology and ground water features of the Smith River Plain Del Norte County California. US Geological Survey Water-Supply Paper 1254, 83 pp. Accessed May 8, 2023 via https://pubs.usgs.gov/wsp/1254/report.pdf	-	-	Approximated from Plate 1 by Back (1957)
**Smith Valley**	-	Loeltz, O. J., Eakin, T. E. (1953). Geology and water resources of Smith Valley, Lyon and Douglas Counties, Nevada. U.S. Geological Survey Water-Supply Paper 1228, 94 pp. Accessed March 21, 2021 from https://pubs.usgs.gov/wsp/1228/report.pdf	-	-	Approximated from Plate 2 of Loeltz and Eakin (1953)
**Smoke Creek Desert**	-	Lopes, T.J., Evetts. D.M. (2005). Ground-Water	Harrill, J.R., Prudic, D.E. (1998). Aquifer systems in	-	Approximated from Fig. 1 by Lopes and Evetts (2005), and Fig. 2 by

Aquifer system	Broader aquifer system	Reference A	Reference B	Reference C	Steps to delineate
		pumpage and artificial recharge estimates for calendar year 2000 and average annual natural recharge and interbasin flow by hydrographic area, Nevada. Accessed April 25, 2022 via https://pubs.usgs.gov/sir/2004/5239/sir2004-5239.pdf	the Great Basin region of Nevada, Utah, and adjacent states; summary report. US Geological Survey Professional Paper 1409-A, 75 pp. Accessed April 26, 2022 via https://pubs.usgs.gov/pp/1409a/report.pdf		Harrill and Prudic (1998). Well completion records, physiography and drainage were used to delinate areas.
**Snake Valley**	-	Gardner, P. M., Masbruch, M. D. (2015). Hydrogeologic and geochemical characterization of groundwater resources in Deep Creek Valley and adjacent areas, Juab and Tooele Counties, Utah, and Elko and White Pine Counties, Nevada. US Geological Survey Scientific Investigations Report 2015–5097, 66 pp. Accessed March 10, 2021 from https://pubs.usgs.gov/sir/2015/5097/sir20155097.pdf	-	-	Approximated from Fig. 6 of Gardner and Masbruch (2015)
**Socorro Basin**	-	Anderholm, S.K. (1984). Hydrogeology of the Socorro and La Jencia Basins, Socorro County, New Mexico. US Geological Survey Water-Resources Investigations Report 84-4342, 72 pp. Accessed April 26, 2022 via https://pubs.usgs.gov/wri/1984/4342/report.pdf	-	-	Approximated from Fig. 1 by Anderholm (1984)

Aquifer system	Broader aquifer system	Reference A	Reference B	Reference C	Steps to delineate
**Sofia Neogene Graben**	-	Shterev, K.D. (2004). The hydrogeothermal basin of Sofia graben (Bulgaria). Environmental Geology, 46, 651-660.	-	-	Approximated from Fig. 1 by Shterev (2004)
**Soghan Basin**	-	Hosseini, S. M., Parizi, E., Ataie-Ashtiani, B., Simmons, C. T. (2019). Assessment of sustainable groundwater resources management using integrated environmental index: Case studies across Iran. Science of the Total Environment, 676, 792-810.	-	-	Approximated from Fig. 6 by Hosseini et al. (2019)
**Solano Basin**	Sacramento Valley of the Central Valley **	California Department of Water Resources (2015i). California's groundwater update 2013 - Chapter 7: Sacramento River Hydrologic Region. California Department of Water Resources Report, 126 pp. Accessed April 27, 2022 via https://water.ca.gov/-/media/DWR-Website/Web-Pages/Programs/Groundwater-Management/Bulletin-118/Files/Statewide-Reports/GWU2013_Ch7_SacramentoRiver_Final.pdf	-	-	Approximated from Fig. 7-2 by California Department of Water Resources (2015i)
**Somontano del Moncayo Aquifer**	-	Carceller-Layel, T., Costa-Alandí, C., Coloma-López, P., García-Vera, M. Á., & San Román-Saldaña, J. (2007). Groundwater in the central sector of the Ebro Basin. Water Resources Development, 23, 165-187.	-	-	Approximated from Fig. 3 by Carceller-Layel et al. (2007).

Aquifer system	Broader aquifer system	Reference A	Reference B	Reference C	Steps to delineate
**Sonoma Valley**	-	California Department of Water Resources (2015g). California's groundwater update 2013 - Chapter 4: San Francisco Bay Hydrologic Region. California Department of Water Resources Report, 78 pp. Accessed April 27, 2022 via https://cawaterlibrary.net/wp-content/uploads/2017/05/GWU2013_Ch4_SanFranciscoBay_Final.pdf	-	-	Approximated from Fig. 4-2 by California Department of Water Resources (2015g)
**Sonsonate Aquifer**	-	US Army Corps of Engineers (1998). Water resources assessment of El Salvador. 71 pp. Accessed February 7, 2023 via https://www.sam.usace.army.mil/Portals/46/docs/military/engineering/docs/WRA/ElSalvador/El%20Salvador%20WRA%20English.pdf	Ministerio de Medio Ambiente y Recursos Natural (2017). Plan Nacional de Gestión Integrada del Recurso Hídrico de El Salvador, con énfasis en zonas prioritarias. Report by the Ministerio de Medio Ambiente y Recursos Naturales (MARN), 52 pp. Accessed February 7, 2023 via http://rcc.marn.gob.sv/bitstream/handle/123456789/259/Resumen%20Ejecutivo%20PNGRH%202017.compressed.pdf?sequence=1&isAllowed=y	Ministerio de Medio Ambiente y Recursos Natural (2023). Objetivos de Calidad de Agua, Ríos, Lagos y Embalses ZP1. Atlas accessed February 7, 2023 via http://srt.snet.gob.sv/sihi/public/atlas	Approximated from Mapa 3 by Ministerio de Medio Ambiente y Recursos Natural (2017) and the descriptions and Fig. A-2 within US Army Corps of Engineers (1998) and atlas by Ministerio de Medio Ambiente y Recursos Natural (2023)
**Soufian-Shabestar Aquifer**	-	Javadzadeh, H., Ataie-Ashtiani, B., Hosseini, S. M., Simmons, C. T. (2020). Interaction of lake-groundwater levels using cross-correlation analysis: A case study of Lake Urmia	-	-	Approximated from Fig. 1 by Javadzadeh et al. (2020)

Aquifer system	Broader aquifer system	Reference A	Reference B	Reference C	Steps to delineate
		Basin, Iran. Science of The Total Environment, 729, 138822.			
**South American Basin**	Sacramento Valley of the Central Valley **	California Department of Water Resources (2015i). California's groundwater update 2013 - Chapter 7: Sacramento River Hydrologic Region. California Department of Water Resources Report, 126 pp. Accessed April 27, 2022 via https://water.ca.gov/-/media/DWR-Website/Web-Pages/Programs/Groundwater-Management/Bulletin-118/Files/Statewide-Reports/GWU2013_Ch7_SacramentoRiver_Final.pdf	-	-	Approximated from Fig. 7-2 by California Department of Water Resources (2015i)
**South Chianan Plain**	Chianan Plain	Wang, S. J., Lee, C. H., Yeh, C. F., Choo, Y. F., Tseng, H. W. (2021). Evaluation of Climate Change Impact on Groundwater Recharge in Groundwater Regions in Taiwan. Water, 13, 1153.	Nath, B., Jean, J. S., Lee, M. K., Yang, H. J., Liu, C. C. (2008). Geochemistry of high arsenic groundwater in Chia-Nan plain, Southwestern Taiwan: possible sources and reactive transport of arsenic. Journal of Contaminant Hydrology, 99, 85-96.	Lu, K. L., Liu, C. W., Jang, C. S. (2012). Using multivariate statistical methods to assess the groundwater quality in an arsenic-contaminated area of Southwestern Taiwan. Environmental Monitoring and Assessment, 184, 6071-6085.	Approximated from Fig. 2 by Wang et al. (2021), Fig. 1 by Nath et al. (2008), and Fig. 1 by Lu et al. (2012)
**South Fork Pitt River Basin**	-	California Department of Water Resources (2015i). California's groundwater update 2013 - Chapter 7: Sacramento River Hydrologic Region. California Department of Water Resources Report, 126 pp. Accessed April 27, 2022 via	-	-	Approximated from Fig. 7-2 by California Department of Water Resources (2015i)

Aquifer system	Broader aquifer system	Reference A	Reference B	Reference C	Steps to delineate
		https://water.ca.gov/-/media/DWR-Website/Web-Pages/Programs/Groundwater-Management/Bulletin-118/Files/Statewide-Reports/GWU2013_Ch7_SacramentoRiver_Final.pdf			
**South Platte Basin**	-	Wellman, T.P. (2015). Evaluation of groundwater levels in the South Platte River alluvial aquifer, Colorado, 1953–2012, and design of initial well networks for monitoring groundwater levels. U.S. Geological Survey Scientific Investigations Report 2015–5015, 67 pp., Accessed March 29, 2021 from https://pubs.usgs.gov/sir/2015/5015/pdf/sir2015-5015.pdf	-	-	Approximated from Fig. 3 of Wellman (2015)
**South Rewa Basin**	Son-Mahandai Valley Basins	Chowdari, S., Singh, B., Rao, B. N., Kumar, N., Singh, A. P., Chandrasekhar, D. V. (2017). Structural mapping based on potential field and remote sensing data, South Rewa Gondwana Basin, India. Journal of Earth System Science, 126, 1-27.	Central Ground Water Board (2013b). Aquifer Systems of Madhya Pradesh. Central Ground Water Board Report, 78 pp. Accessed July 17, 2021 from http://cgwb.gov.in/AQM/Madhya%20Pradesh.pdf	Dutta, P.K., Acharyya, S.K., Chakraborty, T., Jha, N., Khangar, R., Khasdeo, L., Misra, K.G. (2015). Resolving Kamthi-related problems in Gondwana stratigraphy of peninsular India. Indian Journal of Geosciences, 69, 85-102.	Approximated from Fig. 2 by Chowdari et al. (2017), Plate XXIX by Central Ground Water Board (2013b), and Fig. 1 by Dutta et al. (2015)
**South Sound and Commencement Bay**	Puget Sound Lowland	Vaccaro, J. J., Hansen, A. J., Jones, M. A. (1998). Hydrogeologic framework of the Puget Sound aquifer system, Washington and British Columbia. U.S. Geological Survey Professional Paper 1424-D,	Washington Department of Ecology (2010). Puget Sound Groundwater Toxics Loading Analysis: Direct Discharge Pathway. Publication No. 10-03-122, 26 pp. Accessed March 12, 2021 from	-	Broader Puget Sound Lowland approximated from Vaccaro et al. (1998). Sub-basin outline approximated from Fig. 2 of Washington Department of Ecology (2010).

Aquifer system	Broader aquifer system	Reference A	Reference B	Reference C	Steps to delineate
		87 pp. Accessed March 11, 2021 from https://pubs.usgs.gov/pp/1424d/report.pdf	https://apps.ecology.wa.gov/publications/documents/1003122.pdf		
**South Yuba Basin**	Sacramento Valley of the Central Valley **	California Department of Water Resources (2015i). California's groundwater update 2013 - Chapter 7: Sacramento River Hydrologic Region. California Department of Water Resources Report, 126 pp. Accessed April 27, 2022 via https://water.ca.gov/-/media/DWR-Website/Web-Pages/Programs/Groundwater-Management/Bulletin-118/Files/Statewide-Reports/GWU2013_Ch7_SacramentoRiver_Final.pdf	-	-	Approximated from Fig. 7-2 by California Department of Water Resources (2015i)
**Southcentral Coromandel Fractured Gneiss Aquifers**	Coromandel Coastal Plain	Central Ground Water Board (2012b). Aquifer Systems of Tamilnadu and Puducherry. Central Ground Water Board Report, 78 pp. Accessed June 23, 2021 from http://cgwb.gov.in/AQM/TamilNadu.pdf	-	-	Approximated from Plate IV by Central Ground Water Board (2012b)
**Southcentral Deccan Basalt Plateau**	Deccan Plateau	Deolankar, S. B. (1980). The Deccan basalts of Maharashtra, India—their potential as aquifers. Groundwater, 18(5), 434-437.	Kale, V. S., Bodas, M., Chatterjee, P., Pande, K. (2020). Emplacement history and evolution of the Deccan Volcanic Province, India. Episodes, 43, 278-299.	Duraiswami, R. A., Das, S., Shaikh, T. (2012). Hydrogeological framework of aquifers from the Deccan Traps, India: some insights. Memoir Geological Society of India, 1-15.	Approximated from Fig. 1 by Deolankar (1980), Fig. 1 by Duraiswami et al. (2012), and Fig. 1 by Kale et al. (2016). Subarea based on topography and drainage patterns.
**Southcentral Floodplains**	Bengal Basin	Woodman, N. D., Burgess, W. G., Ahmed, K. M., Zahid, A. (2019). A partially coupled hydro-mechanical	Ravenscroft, P., McArthur, J. M., Rahman, M. S. (2018). Identifying multiple deep aquifers in the Bengal	Michael, H. A., Voss, C. I. (2009). Controls on groundwater flow in the Bengal Basin of India and	Approximated from Fig. 1 by Woodman et al. (2019), Fig. 1 by Ravenscroft et al. (2018), and Fig. 1 by Michael and Voss (2009)

Aquifer system	Broader aquifer system	Reference A	Reference B	Reference C	Steps to delineate
		analysis of the Bengal Aquifer System under hydrological loading. Hydrology and Earth System Sciences, 23, 2461-2479.	Basin: Implications for resource management. Hydrological Processes, 32, 3615-3632.	Bangladesh: regional modeling analysis. Hydrogeology Journal, 17, 1561-1577.	
**South-central High Plains****	High Plains	Gutentag, E.D., Heimes, F.J., Krothe, N.C., Luckey, R.R., Weeks, J.B. (1984). Geohydrology of the High Plains aquifer in parts of Colorado, Kansas, Nebraska, New Mexico, Oklahoma, South Dakota, Texas, and Wyoming (No. 1400-B). Accessed February 10, 2021 from https://pubs.usgs.gov/pp/1400b/report.pdf	Luckey, R.L., Becker, M.F. (2003). Hydrogeology, water use, and simulation of flow in the High Plains aquifer in northwestern Oklahoma, southeastern Colorado, southwestern Kansas, northeastern New Mexico, and northwestern Texas. Water-Resources Investigations Report 99-4104, 73 pp. Accessed February 15, 2022 from https://pubs.usgs.gov/wri/wri994104/pdf/wri994104.pdf	McGuire, V.L., Johnson, M.R., Schieffer, J.S., Stanton, J.S., Sebree, S.K., Varstraeten (2003). Water in storage and approaches to groundwater management, High Plains Aquifer, 2000. US Geological Survey Circular 1243, 56 pp. Accessed February 15, 2022 from https://pubs.usgs.gov/circ/2003/circ1243/pdf/C1243.pdf	Approximated from Fig. 1 of Gutentag et al. (1984), Fig. 18 by McGuire et al. (2003), and Fig. 1 by Luckey and Becker (2003)
**Southcentral Hualien-Taitung Valley**	Hualien-Taitung Valley	Hsu, S. K. (1998). Plan for a groundwater monitoring network in Taiwan. Hydrogeology Journal, 6, 405-415.	-	-	Approximated from Fig. 1 by Fig. 2 by Hsu et al. (1998); subarea boundary based on drainage and topography
**Southcentral Karoo Basin**	Main Karoo Basin	Nyambe, I. A. (1999). Tectonic and climatic controls on sedimentation during deposition of the Sinakumbe Group and Karoo Supergroup, in the mid-Zambezi Valley Basin, southern Zambia. Journal of African Earth Sciences, 28, 443-463.	-	-	Broader aquifer system boundary (i.e., Main Karoo Basin) approximated from Fig. 1 by Nyambe (1999). Physiography (drainage patterns, geology) and well construction data were considered while digitizing subarea
**Southcentral Mega Aquifer System**	Mega Aquifer System	Awadh, S. M., Al-Mimar, H., Yaseen, Z. M. (2020). Groundwater availability and water demand	Abotalib, A. Z., Heggy, E., Scabbia, G., Mazzoni, A. (2019). Groundwater dynamics in fossil fractured	-	Approximated from Fig. 1 by Awadh et al. (2020) and Fig. 1 by Abotalib et al. (2019)

Aquifer system	Broader aquifer system	Reference A	Reference B	Reference C	Steps to delineate
		sustainability over the upper mega aquifers of Arabian Peninsula and west region of Iraq. Environment, Development and Sustainability, 1-21.	carbonate aquifers in Eastern Arabian Peninsula: a preliminary investigation. Journal of Hydrology, 571, 460-470.		
**Southcentral Piedmont Plain**	North China Plain	Meng, S., Liu, J., Zhang, Z., Lei, T., Qian, Y., Li, Y., Fei, Y. (2015). Spatiotemporal evolution characteristics study on the precipitation infiltration recharge over the past 50 years in the North China Plain. Journal of Earth Science, 26, 416-424.	Cao, G., Zheng, C., Scanlon, B. R., Liu, J., & Li, W. (2013). Use of flow modeling to assess sustainability of groundwater resources in the North China Plain. Water Resources Research, 49, 159-175.	Wang, S., Song, X., Wang, Q., Xiao, G., Liu, C., Liu, J. (2009). Shallow groundwater dynamics in North China plain. Journal of Geographical Sciences, 19, 175-188.	Approximated from Fig. 1 by Meng et al. (2015), Fig. 1 by Cao et al. (2013) and Fig. 6 by Wang et al. (2009).
**Southcentral Piedmont Upland**	Piedmont Plateau	Miller, J.A. (1990). Ground Water Atlas of the United States: Segment 6, Alabama, Florida, Georgia, South Carolina. U.S. Geological Survey Hydrologic Atlas 730-G, 30 pp. Accessed April 5, 2021 from https://www.nrc.gov/docs/ML1706/ML17060B027.pdf	-	-	Approximated from Fig. 3 of Miller (1990). Northeastern margin of area approximated along the South Yadkin and Pee Dee Rivers.
**Southcentral Valley and Ridge**	Valley and Ridge Aquifer System	Johnson, G.C., Zimmerman, T.M., Lindsey, B.D., Gross, E.L. (2011). Factors affecting groundwater quality in the Valley and Ridge aquifers, eastern United States, 1993–2002: U.S. Geological Survey Scientific Investigations Report 2011–5115, 84 pp. Accessed April 13, 2021 from https://pubs.usgs.gov/sir/2	Brahana, J.V., Macy, J.A., Mulderink, D., Zemo, D. (1986). Preliminary delineation and description of the regional aquifers of Tennessee-Cumberland plateau aquifer system. U.S. Geological Survey, Water-Resources Investigations Report 82-338, 29 pp. Accessed April 13, 2021 from https://pubs.usgs.gov/wri/	-	Northern margin approximated along the Hiwassee River. Broader Valley and Ridge Aquifer System outline approximated from Fig. 1 of Johnson et al. (2011) and the Tennessee portion approximated from Fig. 1 of Brahana et al. (1986)

Aquifer system	Broader aquifer system	Reference A	Reference B	Reference C	Steps to delineate
		011/5115/support/sir2011-5115.pdf	wrir82-338/pdf/wrir_82-338_a.pdf		
**Southeast Lower Chao Phraya Basin**	Lower Chao Phraya Basin	Hosono, T., Lorphensriand, O., Onodera, S.I., Okawa, H., Nakano, T., Yamanaka, T., Tsujimura, M, Taniguchi, M. (2014). Different isotopic evolutionary trends of δ34S and δ18O compositions of dissolved sulfate in an anaerobic deltaic aquifer system. Applied Geochemistry, 46, 30-42.	Taweesin, K., Seeboonruang, U., Saraphirom, P. (2018). The influence of climate variability effects on groundwater time series in the lower central plains of Thailand. Water, 10, 290.	Sinsakul, S. (2000). Late quaternary geology of the lower central plain, Thailand. Journal of Asian Earth Sciences, 18, 415-426.	Approximated from Fig. 2 by Hosono et al. (2014), Fig. 1 by Taweesin et al. (2018), and Fig. 1 by Sinsakul (2000)
**Southeastern Allegheny Plateau**	Appalachian Plateaus	Trapp Jr., H., Horn, M.A. (1997). Ground water atlas of the United States: Segment 11. U.S. Geological Survey Hydrologic Investigations Atlas 730-L, 26 pp. Accessed April 13, 2021 from https://pubs.usgs.gov/ha/730l/report.pdf	-	-	Broader Appalachian Plateau Aquifer System approximated from Fig. 3 of Trapp Jr. and Horn (1997)
**Southeastern Bengal Basin**	Bengal Basin	Woodman, N. D., Burgess, W. G., Ahmed, K. M., Zahid, A. (2019). A partially coupled hydro-mechanical analysis of the Bengal Aquifer System under hydrological loading. Hydrology and Earth System Sciences, 23, 2461-2479.	Ravenscroft, P., McArthur, J. M., Rahman, M. S. (2018). Identifying multiple deep aquifers in the Bengal Basin: Implications for resource management. Hydrological Processes, 32, 3615-3632.	Michael, H. A., Voss, C. I. (2009). Controls on groundwater flow in the Bengal Basin of India and Bangladesh: regional modeling analysis. Hydrogeology Journal, 17, 1561-1577.	Approximated from Fig. 1 by Woodman et al. (2019), Fig. 1 by Ravenscroft et al. (2018), and Fig. 1 by Michael and Voss (2009)
**Southeastern Coastal Karoo Basin**	Main Karoo Basin	Nyambe, I. A. (1999). Tectonic and climatic controls on sedimentation during deposition of the Sinakumbe Group and Karoo Supergroup, in the	-	-	Broader aquifer system boundary (i.e., Main Karoo Basin) approximated from Fig. 1 by Nyambe (1999). Physiography (drainage patterns, geology) and

Aquifer system	Broader aquifer system	Reference A	Reference B	Reference C	Steps to delineate
		mid-Zambezi Valley Basin, southern Zambia. Journal of African Earth Sciences, 28, 443-463.			well construction data were considered while digitizing subarea
**Southeastern Divandareh-Bijar Basin**	-	Hosseini, S. M., Parizi, E., Ataie-Ashtiani, B., Simmons, C. T. (2019). Assessment of sustainable groundwater resources management using integrated environmental index: Case studies across Iran. Science of the Total Environment, 676, 792-810.	-	-	Approximated from Fig. 6 by Hosseini et al. (2019)
**Southeastern Eucla Basin**	Eucla Basin	Berens, V., Alcoe, D., Watt, E. (2011). Non-prescribed groundwater resources assessment — Eyre Peninsula natural resources management region. Science, Monitoring and Information Division Department for Water, 110 pp. Accessed October 6, 2021 from https://www.waterconnect.sa.gov.au/Content/Publications/DEW/EP_NRM_Non-prescribed_GW_Assessment_2011.pdf	-	-	Approximated from Fig. 4 by Berens et al. (2011)
**Southeastern Jazmurian Depression**	Jazmurian Depression	Rezaei, A., Hassani, H., Hassani, S., Jabbari, N., Mousavi, S. B. F., Rezaei, S. (2019). Evaluation of groundwater quality and heavy metal pollution indices in Bazman basin, southeastern Iran. Groundwater for	-	-	Jazmurian Depression approximated from Fig. 2 by Rezaei et al. (2019)

Aquifer system	Broader aquifer system	Reference A	Reference B	Reference C	Steps to delineate
		Sustainable Development, 9, 100245.			
**Southeastern Surat Basin**	Great Artesian Basin	Ransley, T.R., Radke, B.M., Feitz, A.J., Kellett, J.R., Owens, R., Bell, J. and Stewart, G. (2015). Hydrogeological Atlas of the Great Artesian Basin, Geoscience Australia. Canberra, 141 pp. Accessed July 19, 2021 from http://www.ga.gov.au/scientific-topics/water/groundwater/gab	Ransley, T.R., Smerdon, B.D. (2012). Hydrostratigraphy, hydrogeology and system conceptualisation of the Great Artesian Basin. A technical report to the Australian Government from the CSIRO Great Artesian Basin Water Resource Assessment, 324 pp. Accessed July 20, 2021 from https://publications.csiro.au/rpr/download?pid=csiro:EP132693&dsid=DS5	Smerdon, B.D., Ramsley, T.R. (2012). Water Resource Assessment for the Surat Region. A technical report to the Australian Government from the CSIRO Great Artesian Basin Water Resource Assessment, 142 pp. Accessed July 20, 2021 from https://publications.csiro.au/rpr/download?pid=csiro:EP132644&dsid=DS4	Approximated from Map 1 on page 9 of report by Ransley et al. (2015), and Fig. 2.3 by Ransley and Smerdon (2012). Subarea within Surat Basin delineated on the basis of physiographic features (e.g., major rivers), elevations of the base of the Great Artesian Basin (Fig. 5.2 by Smerdon and Ransley (2012b)) and topography.
**Southeastern System**	Amadeus Basin	Lloyd, J. W., Jacobson, G. (1987). The hydrogeology of the Amadeus Basin, central Australia. Journal of Hydrology, 93, 1-24.	Brown, D. M., Lloyd, J. W., Jacobson, G. (1990). Hydrogeological model for Amadeus Basin aquifers, central Australia. Australian Journal of Earth Sciences, 37, 215-226.	Cresswell, R. G., Jacobson, G., Wischusen, J., Fifield, L. K. (1999). Ancient groundwaters in the Amadeus Basin, Central Australia: evidence from the radio-isotope 36Cl. Journal of Hydrology, 223, 212-220.	Approximated from Fig. 1 by Lloyd and Jacobson (1987), Fig. 1 by Brown et al. (1990), and Fig. 1 by Cresswell et al. (1999)
**Southern Bohemian Tertiary and Cretaceous Sedimentary Aquifers**	Bohemian Cretaceous Basin	Alvarado, J. C., Pačes, T., Purtschert, R. (2013). Dating groundwater in the Bohemian Cretaceous Basin: Understanding tracer variations in the subsurface. Applied Geochemistry, 29, 189-198.	Jiráková, H., Huneau, F., Hrkal, Z., Celle-Jeanton, H., Le Coustumer, P. (2010). Carbon isotopes to constrain the origin and circulation pattern of groundwater in the north-western part of the Bohemian Cretaceous Basin (Czech Republic). Applied Geochemistry, 25, 1265-1279.	Kadlecová, R., Olmer, M. (2011). Review of Groundwater Resources. Geologické výzkumy na Moravě a ve Slezsku. Proceedings of The Open Congress ČGS and SGS, September 22–25, 2011, Monínec 18, pp. 31-34. Accessed October 21, 2021 from https://journals.muni.cz/gvms/article/view/1679	Approximated from Fig. 1 by Jiráková et al. (2010), Fig. 1 by Alvarado et al. (2013), and Fig. 1 by Kadlecová and Olmer (2011)

Aquifer system	Broader aquifer system	Reference A	Reference B	Reference C	Steps to delineate
**Southern Butte Valley**	-	Lopes, T.J., Evetts. D.M. (2005). Ground-Water pumpage and artificial recharge estimates for calendar year 2000 and average annual natural recharge and interbasin flow by hydrographic area, Nevada. Accessed April 25, 2022 via https://pubs.usgs.gov/sir/2004/5239/sir2004-5239.pdf	Harrill, J.R., Prudic, D.E. (1998). Aquifer systems in the Great Basin region of Nevada, Utah, and adjacent states; summary report. US Geological Survey Professional Paper 1409-A, 75 pp. Accessed April 26, 2022 via https://pubs.usgs.gov/pp/1409a/report.pdf	-	Approximated from Fig. 1 by Lopes and Evetts (2005), and Fig. 2 by Harrill and Prudic (1998). Well completion records, physiography and drainage were used to delinate areas.
**Southern Canterbury Plains**	-	Taylor, C. B., Wilson, D. D., Brown, L. J., Stewart, M. K., Burden, R. J., Brailsford, G. W. (1989). Sources and flow of north Canterbury plains groundwater, New Zealand. Journal of hydrology, 106, 311-340.	Bal, A. A. (1996). Valley fills and coastal cliffs buried beneath an alluvial plain: evidence from variation of permeabilities in gravel aquifers, Canterbury Plains, New Zealand. Journal of Hydrology (New Zealand), 1-27.	Wilson, D. D. (1973). The significance of geology in some current water resource problems, Canterbury Plains, New Zealand. Journal of Hydrology (New Zealand), 103-118.	Approximated from Fig. 1 by Taylor et al. (1989), Figs. 1 and 2 by Bal (1996) and Fig. 1 by Wilson (1973). Physiography and drinage features were applied to approximate subareas
**Southern Carbonate Rock Aquifers**	Manitoba Carbonate Aquifer System	Betcher, R.N., Pupp, C., Grove, G. (1995). Groundwater in Manitoba: hydrogeology, quality concerns, management. Environment Canada, National Hydrology Research Institute Report No. C2-93017, 53 pp. Accessed May5, 2021 from https://web.viu.ca/earle/geol304/hg-manitoba.pdf	-	-	Approximated from Fig. 11 by Betcher et al. (1995).
**Southern Carpentaria Basin**	Great Artesian Basin	Ransley, T.R., Smerdon, B.D. (2012). Hydrostratigraphy, hydrogeology and system conceptualisation of the Great Artesian Basin. A technical report to the Australian Government	-	-	Approximated from Fig. 2.4 by Ransley and Smerdon (2012)

Aquifer system	Broader aquifer system	Reference A	Reference B	Reference C	Steps to delineate
		from the CSIRO Great Artesian Basin Water Resource Assessment, 324 pp. Accessed July 20, 2021 from https://publications.csiro.au/rpr/download?pid=csiro:EP132693&dsid=DS5			
**Southern Cauvery Delta and Orathanadu Aquifer System**	Coromandel Coastal Plain	Dhinagaran, V. (2009). District Groundwater Brochure Thanjavur District, Tamil Nadu. Central Ground Water Board, Ministry of Water Resources Report, 22 pp. Accessed June 24, 2021 from http://cgwb.gov.in/District_Profile/TamilNadu/Thanjavur.pdf	Dunlop, G., Palanichamy, J., Kokkat, A., James, E. J., Palani, S. (2019). Simulation of saltwater intrusion into coastal aquifer of Nagapattinam in the lower cauvery basin using SEAWAT. Groundwater for Sustainable Development, 8, 294-301.	Juran, L., MacDonald, M. C., Basu, N. B., Hubbard, S., Rajagopal, R., Rajagopalan, P., Philip, L. (2017). Development and application of a multi-scalar, participant-driven water poverty index in post-tsunami India. International Journal of Water Resources Development, 33, 955-975.	Approximated from Plate II and descriptions of hydrogeologic conditions in text by Dhinagaran (2009) and descriptions of hydrogeology and Fig. 1 by Dunlop et al. (2019). The Miocene-aged (Dhinagaran, 2009) Orathanadu aquifer is known to be an important source of groundwater in Nagapattinam and Karaikal Districts (Juran et al., 2016); the northern limit of this area was delineated close to Karaikal.
**Southern Chalk Aquifer**	Chalk Aquifer System	MacDonald, A.M., Allen, D.J. (2001). Aquifer properties of the Chalk of England. Quarterly Journal of Engineering Geology and Hydrogeology, 34, 371-384.	-	-	Approximated from Fig. 8 by MacDonald and Allen (2001)
**Southern Coastal Gujarat Alluvial Plain**	-	Qasim, A., Singh, S.P., Chandrashekhar, A.K. (2022). Geochemical and isotope tracing of groundwater salinity in the coastal Gujarat alluvial plain, India. Journal of Contaminant Hydrology, 248, 104000.	-	-	Approximated from Qasim et al. (2022)
**Southern Coimbatore Fractured Charnockite**	Coimbatore Upland	Sajil Kumar, P. J., James, E. J. (2016). Identification of hydrogeochemical processes in the	Saha, D., Shekhar, S., Ali, S., Vittala, S.S., Raju, N.J. (2016). Recent hydrogeological research in	-	Approximated from Fig. 1 by Sajil Kumar and James (2016), and Fig. 1 by Saha et al. (2016)

Aquifer system	Broader aquifer system	Reference A	Reference B	Reference C	Steps to delineate
**and Gneiss Aquifers**		Coimbatore district, Tamil Nadu, India. Hydrological Sciences Journal, 61(4), 719-731.	India. Proceedings of the Indian National Science Academy, 82, 787-803.		
**Southern Colusa Basin**	Sacramento Valley of the Central Valley **	California Department of Water Resources (2015i). California's groundwater update 2013 - Chapter 7: Sacramento River Hydrologic Region. California Department of Water Resources Report, 126 pp. Accessed April 27, 2022 via https://water.ca.gov/-/media/DWR-Website/Web-Pages/Programs/Groundwater-Management/Bulletin-118/Files/Statewide-Reports/GWU2013_Ch7_SacramentoRiver_Final.pdf	-	-	Approximated from Fig. 7-2 by California Department of Water Resources (2015i)
**Southern Coromandel Coastal Alluvial Aquifer**	Coromandel Coastal Plain	Singaraja, C., Chidambaram, S., Anandhan, P., Prasanna, M. V., Thivya, C., Thilagavathi, R. (2015). A study on the status of saltwater intrusion in the coastal hard rock aquifer of South India. Environment, Development and Sustainability, 17, 443-475.	Central Ground Water Board (2012b). Aquifer Systems of Tamilnadu and Puducherry. Central Ground Water Board Report, 78 pp. Accessed June 23, 2021 from http://cgwb.gov.in/AQM/TamilNadu.pdf	Subramanian, S., Balasubramanian, A. (1994). Hydrochemical studies of Tiruchendur Coast, Tamilnadu, India. Proceedings of Regional Workshop on Environmental Aspects of Groundwater Development, Oct 17-19, 1994, 7 pp.	Approximated from Fig. 2 by Singaraja et al. (2015), Fig. 1 by Subramanian and Balasubramanian (1994), and Plate IV by Central Ground Water Board (2012b)
**Southern Coromandel Fractured Charnockite and Gneiss Aquifers**	Coromandel Coastal Plain	Singaraja, C., Chidambaram, S., Anandhan, P., Prasanna, M. V., Thivya, C., Thilagavathi, R. (2015). A study on the status of saltwater intrusion in the coastal hard rock aquifer of South India. Environment, Development	Balachandran, A. (2009). District groundwater brochure Tirunelveli district, Tamil Nadu. Central Ground Water Board Technical Report Series, 21 pp. Accessed June 23, 2021 from http://cgwb.gov.in/District_	Central Ground Water Board (2012b). Aquifer Systems of Tamilnadu and Puducherry. Central Ground Water Board Report, 78 pp. Accessed June 23, 2021 from http://cgwb.gov.in/AQM/TamilNadu.pdf	Approximated from Fig. 2 by Singaraja et al. (2015), Plate V by Balachandran (2009), and Plate IV by Central Ground Water Board (2012b); northern margin approximated along the Vaigai River

Aquifer system	Broader aquifer system	Reference A	Reference B	Reference C	Steps to delineate
		and Sustainability, 17, 443-475.	Profile/TamilNadu/Tirunelveli.pdf		
**Southern Culverden Basin**	Culverden Basin	Poulsen, D. (2012). Culverden Basin hydrogeology. Environment Canterbury Regional Council Report No. R12/96, 104 pp. Accessed October 20, 2021 from https://www.ecan.govt.nz/document/download?uri=1723844	-	-	Approximated from Fig. 2-1 by Environment Canterbury Regional Council (2012). Subareas based on drainage and physiography.
**Southern Cumberland Plateau - Pottsville Aquifer**	Appalachian Plateaus	Jennings, S.P. (2013). Hydrogeology and groundwater assessment of the water distribution area of the town of Hodges water department, Franklin and Marion Counties, Alabama. Geological Survey of Alabama Report, 15pp. Accessed April 8, 2021 from https://www.ogb.state.al.us/img/Groundwater/OFR/OFR1311.pdf	Geological Survey of Alabama (2018). Assessment of groundwater resources in Alabama, 2010-16. Geological Survey of Alabama Bulletin 186, 462 pp. Accessed April 8, 2021 from https://www.gsa.state.al.us/img/Groundwater/docs/assessment/00_B186_StatewideAssessment_Print_Document.pdf	-	Physiographic provinces from Fig. 1 of Jennings (2013) and Fig. 4 of Geological Survey of Alabama (2018). Aquifer recharge areas defined in Fig. 6 of Geological Survey of Alabama (2018). Northern limit of area was approximated at Guntersville Lake.
**Southern Darz Sayebn Basin**	-	Hosseini, S. M., Parizi, E., Ataie-Ashtiani, B., Simmons, C. T. (2019). Assessment of sustainable groundwater resources management using integrated environmental index: Case studies across Iran. Science of the Total Environment, 676, 792-810.	-	-	Approximated from Fig. 6 by Hosseini et al. (2019)
**Southern Deccan Basalt Plateau**	Deccan Plateau	Deolankar, S. B. (1980). The Deccan basalts of Maharashtra, India—their potential as aquifers.	Kale, V. S., Bodas, M., Chatterjee, P., Pande, K. (2020). Emplacement history and evolution of the Deccan Volcanic Province,	Duraiswami, R. A., Das, S., Shaikh, T. (2012). Hydrogeological framework of aquifers from the Deccan Traps, India: some insights.	Approximated from Fig. 1 by Deolankar (1980), Fig. 1 by Duraiswami et al. (2012), and Fig. 1 by Kale et al. (2016). Subarea

Aquifer system	Broader aquifer system	Reference A	Reference B	Reference C	Steps to delineate
		Groundwater, 18(5), 434-437.	India. Episodes, 43, 278-299.	Memoir Geological Society of India, 1-15.	based on topography and drainage patterns.
**Southern Dezful-Andimeshk Plain**	Dezful-Andimeshk Plain	Hosseini, S. M., Parizi, E., Ataie-Ashtiani, B., Simmons, C. T. (2019). Assessment of sustainable groundwater resources management using integrated environmental index: Case studies across Iran. Science of the Total Environment, 676, 792-810.	-	-	Approximated from Fig. 6 by Hosseini et al. (2019)
**Southern Dharwar Craton Banded Gneiss Complex**	Dharwar Craton Banded Gneiss Complex	Lancaster, P. J., Dey, S., Storey, C. D., Mitra, A., Bhunia, R. K. (2015). Contrasting crustal evolution processes in the Dharwar craton: insights from detrital zircon U–Pb and Hf isotopes. Gondwana Research, 28, 1361-1372.	Central Ground Water Board (2012c). Aquifer Systems of Karnataka. Central Ground Water Board Report, 75 pp. Accessed June 30, 2021 from http://cgwb.gov.in/AQM/Karnataka.pdf	-	Dharwar Craton geology approximated from Fig. 1 by Lancaster et al. (2015); hydrogeologic boundaries for Karnataka approximated from Plate III by Central Ground Water Board (2012c)
**Southern Downs Chalk Aquifer**	Chalk Aquifer System	MacDonald, A.M., Allen, D.J. (2001). Aquifer properties of the Chalk of England. Quarterly Journal of Engineering Geology and Hydrogeology, 34, 371-384.	Robins, N. S., Ball, D. F. (2006). The Dumfries Basin aquifer. British Geological Survey Research Report RR/06/02, 68 pp. Accessed October 27, 2021 from http://nora.nerc.ac.uk/id/eprint/3685/1/RR06002.pdf	-	Approximated from Fig. 8 by MacDonald and Allen (2001), and map on the final page of Robins and Ball (2006)
**Southern Duero Basin**	Duero Basin	Esteban, E., Albiac, J. (2012). The problem of sustainable groundwater management: the case of La Mancha aquifers, Spain. Hydrogeology Journal, 20, 851-863.	-	-	Broader outline of Duero Basin approximated from Fig. 1 by Esteban and Albiac (2012), guided by the presence of recorded well completion events and delineating contributing areas upstream of Zamora.
**Southern Goose Lake Valley**	-	California Department of Water Resources (2015i). California's groundwater update 2013 - Chapter 7:	-	-	Approximated from Fig. 7-2 by California Department of Water Resources (2015i)

Aquifer system	Broader aquifer system	Reference A	Reference B	Reference C	Steps to delineate
		Sacramento River Hydrologic Region. California Department of Water Resources Report, 126 pp. Accessed April 27, 2022 via https://water.ca.gov/-/media/DWR-Website/Web-Pages/Programs/Groundwater-Management/Bulletin-118/Files/Statewide-Reports/GWU2013_Ch7_SacramentoRiver_Final.pdf			
**Southern Guadalupe Garzaron Aquifer**	-	CONAGUA (2020l). Actualización de la disponibilidad media anual de agua en el acuífero Guadalupe Garzaron (3212), Estado de Zacatecas. 38 pp. Accessed March 7, 2022 via https://sigagis.conagua.gob.mx/gas1/Edos_Acuiferos_18/zacatecas/DR_3220.pdf	-	-	Approximated from Fig. 1 by CONAGUA (2020l). Southern and northern areas delineated on the basis of physiography and drainage
**Southern Guadiana Menor Basin**	-	Fernández-Chacón, F., Benavente, J., Rubio-Campos, J.C., Kohfahl, C., Jiménez, J., Meyer, H., Hubberten, H., Pekdeger, A. (2010). Isotopic composition (δ18O and δD) of precipitation and groundwater in a semi-arid, mountainous area (Guadiana Menor basin, Southeast Spain). Hydrological Processes: An International Journal, 24, 1343-1356.	-	-	Approximated from Figure 1. by Fernández-Chacón, F., Benavente, J., Rubio-Campos, J. C., Kohfahl, C., Jiménez, J., Meyer, H., ... & Pekdeger, A. (2010). Isotopic composition (δ18O and δD) of precipitation and groundwater in a semi-arid, mountainous area (Guadiana Menor basin, Southeast Spain). Hydrological Processes: An International Journal, 24(10), 1343-1356.

Aquifer system	Broader aquifer system	Reference A	Reference B	Reference C	Steps to delineate
**Southern High Plains**	High Plains	Gutentag, E.D., Heimes, F.J., Krothe, N.C., Luckey, R.R., Weeks, J.B. (1984). Geohydrology of the High Plains aquifer in parts of Colorado, Kansas, Nebraska, New Mexico, Oklahoma, South Dakota, Texas, and Wyoming (No. 1400-B). Accessed February 10, 2021 from https://pubs.usgs.gov/pp/1400b/report.pdf	Deeds, N.E., Harding, J.J., Jones, T.L., Singh, A., Hamlin, S., Reedy, R.C., Yan, T., Jigmond, M., Lupton, D., Scanlon, B.R., Seni, S., Dutton, A. (2015). Final Conceptual Model Report for the High Plains Aquifer System Groundwater Availability Model. Texas Water Development Board Report, 590 pp. Accessed February 15, 2022 from https://www.twdb.texas.gov/groundwater/models/gam/hpas/HPAS_GAM_Conceptual_Report.pdf	-	Approximated from Fig. 1 of Gutentag et al. (1984) and Fig. 2.0.1 by Deeds et al. (2015).
**Southern Highland Rim**	Interior Low Plateaus	Miller, J.A. (1995). Ground Water Atlas of the United States: Segment 10, Illinois, Indiana, Kentucky, Ohio, Tennessee. U.S. Geological Survey Hydrologic Atlas 730-K, 30 pp. Accessed April 11, 2021 from https://pubs.usgs.gov/ha/730k/report.pdf	Brahana, J.V., Bradley, M.W. (1986b). Preliminary delineation and description of the regional aquifers of Tennessee--The Highland Rim Aquifer System. Water-Resources Investigations Report 82-4054, 35 pp. Accessed April 11, 2021 from https://pubs.usgs.gov/wri/wri824054/pdf/wrir_82-4054_a.pdf	Taylor, C.J., Nelson Jr., H.L. (2008). A compilation of provisional karst geospatial data for the Interior Low Plateaus physiographic region, central United States: U.S. Geological Survey Data Series 339, 26 pp. Accessed April 11, 2021 from https://pubs.usgs.gov/ds/339/pdf/ds339_web.pdf	Broader Interior Low Plateaus outline approximated for Tennessee, Illinois, Kentucky, Indiana from Fig. 78 of Miller (1990) and Fig. 1 of Taylor and Nelson (2008). Highland Rim subarea approximated from Fig. 1 of Taylor and Nelson (2008) and Fig. 1 of Brahana and Bradley (1986b)
**Southern Hills**	Gulf Coast Regional Aquifer System	Weiss, J. S. (1990). Geohydrologic units of the coastal lowlands aquifer system, south-central United States. U.S. Geological Survey Regional aquifer-systems analysis. 42 pp. Accessed March 30, 2021 from	Buono, A. (1983). The Southern Hills Regional Aquifer System of Southeastern Louisiana And Southwestern Mississippi. U.S. Geological Survey Water-Resources Investigations Report 83-4189, 44 pp. Accessed March 31, 2021 from	Boswell, E.H. (1979). The Citronelle aquifers in Mississippi. U.S. Geological Survey Water-Resources Investigations Report 78-131, 1 plate. Accessed March 31, 2021 from https://pubs.usgs.gov/wri/1978/0131/plate-1.pdf	Margin of Gulf Coast Regional Aquifer System approximated from Fig. 2 of Weiss (1990); northwestern boundary is Central Mississippi Embayment. Eastern border approximated from Figs. 1 and 2 of Buono (1983) and the Pearl River. Shallow aquifer ("Citronelle aquifers") information from Boswell (1979).

Aquifer system	Broader aquifer system	Reference A	Reference B	Reference C	Steps to delineate
		https://pubs.usgs.gov/pp/1416c/report.pdf	https://pubs.usgs.gov/wri/1983/4189/report.pdf		
**Southern Hirri Basin**	Chhattisgarh Basin	Central Ground Water Board (2012e). Aquifer Systems of Chhattisgarh. Central Ground Water Board Report, 73 pp. Accessed July 13, 2021 from http://cgwb.gov.in/AQM/Chhattisgarh.pdf	George, B.G., Ray, J.S., Kumar, S. (2019). Geochemistry of carbonate formations of the Chhattisgarh Supergroup, central India: implications for Mesoproterozoic global events. Canadian Journal of Earth Sciences, 56, 335-346.	Mukherjee, A., Ray, R. K., Tewari, D., Ingle, V. K., Sahoo, B. K., Khan, M. W. Y. (2014). Revisiting the stratigraphy of the Mesoproterozoic Chhattisgarh Supergroup, Bastar craton, India based on subsurface lithoinformation. Journal of Earth System Science, 123, 617-632.	Approximated from Plate IV by Central Ground Water Board (2012e), Fig. 1 by George et al. (2019), and Figs. 1 and 2 by Mukherjee et al. (2014)
**Southern Hosein Abad Basin**	-	Hosseini, S. M., Parizi, E., Ataie-Ashtiani, B., Simmons, C. T. (2019). Assessment of sustainable groundwater resources management using integrated environmental index: Case studies across Iran. Science of the Total Environment, 676, 792-810.	-	-	Approximated from Fig. 6 by Hosseini et al. (2019)
**Southern Huaihe River Basin**	Huaihe River Basin	Sun, X., Lin, J., Gu, W., Min, X., Han, J., Dai, Y., Liu, P., Wang, S (2021). Analysis and evaluation of the renewability of the deep groundwater in the Huaihe River Basin, China. Environmental Earth Sciences, 80, 1-13.	Liu J., Zheng C. (2016) Towards Integrated Groundwater Management in China. In: Integrated Groundwater Management (eds. Jakeman A.J., Barreteau O., Hunt R.J., Rinaudo J.D., Ross A.). Springer, Cham. pp. 455-475	Li, Y., Wang, D., Liu, Y., Zheng, Q., Sun, G. (2017). A predictive risk model of groundwater arsenic contamination in China applied to the Huai River Basin, with a focus on the region's cluster of elevated cancer mortalities. Applied Geochemistry, 77, 178-183.	Approximated from Fig. 1 by Sun et al. (2021), Fig. 1 by Li et al. (2017), and Fig. 18.5 by Liu and Zheng (2016)
**Southern Hualien-Taitung Valley**	Hualien-Taitung Valley	Hsu, S. K. (1998). Plan for a groundwater monitoring network in Taiwan. Hydrogeology Journal, 6, 405-415.	-	-	Approximated from Fig. 1 by Fig. 2 by Hsu et al. (1998); subarea boundary based on drainage and topography

Aquifer system	Broader aquifer system	Reference A	Reference B	Reference C	Steps to delineate
**Southern Jalpa-Juchipila Aquifer**	-	CONAGUA (2021a). Acuíferos (nacional). Webpage accessed March 8, 2022 from http://sina.conagua.gob.mx/sina/tema.php?tema=acuiferos&ver=mapa&o=1&n=nacional	-	-	Approximated from aquifer boundaries defined by CONAGUA (2021a) web portal, physiography and drainage patterns, and well completion data. Northern and southern Jalpa-Juchipila Aquifers delineated on the basis of topography
**Southern Kern Basin**	Tulare Basin of the Central Valley **	California Department of Water Resources (2015c). California's groundwater update 2013 - Chapter 9: Tulare Lake Hydrologic Region. California Department of Water Resources Report, 80 pp. Accessed April 27, 2022 via https://data.cnra.ca.gov/dataset/california-water-plan-groundwater-update-2013/resource/8a4ae915-b786-42e1-9abe-99a8fcc23349	-	-	Approximated from Fig. 9-2 by California Department of Water Resources (2015c). Subdivided from Northern Kern Basin subarea on the basis of the Kern River
**Southern Kettleman Plain**	-	California Department of Water Resources (2015c). California's groundwater update 2013 - Chapter 9: Tulare Lake Hydrologic Region. California Department of Water Resources Report, 80 pp. Accessed April 27, 2022 via https://data.cnra.ca.gov/dataset/california-water-plan-groundwater-update-2013/resource/8a4ae915-b786-42e1-9abe-99a8fcc23349	-	-	Approximated from Fig. 9-2 by California Department of Water Resources (2015c)
**Southern Malabar Fractured**	Malabar Coastal Plain	Taylor, G. C., Ghosh, P. K. (1964). Artesian water in the Malabar coastal plain of	Central Ground Water Board (2012a). Aquifer Systems of Kerala. Central	Nandakumaran, P., Balakrishnan, K. (2020). Groundwater quality	Approximated from Fig. 1 by Manjusree et al. (2009) and Fig. 1 by Nandakumaran and

Aquifer system	Broader aquifer system	Reference A	Reference B	Reference C	Steps to delineate
**Charnockite Aquifers**		southern Kerala, India. U.S. Geological Survey Water Supply Paper 1608-D, 19 pp. and 2 plates. Accessed June 23, 2021 from https://pubs.usgs.gov/wsp/1608d/report.pdf	Ground Water Board Report, 72 pp. Accessed June 23, 2021 from http://cgwb.gov.in/AQM/Kerala.pdf	variations in Precambrian hard rock aquifers: a case study from Kerala, India. Applied Water Science, 10, 1-13.	Balakrishnan (2020), and Plate III by Central Ground Water Board (2012a)
**Southern Malabar Khondalites**	Malabar Coastal Plain	Taylor, G. C., Ghosh, P. K. (1964). Artesian water in the Malabar coastal plain of southern Kerala, India. U.S. Geological Survey Water Supply Paper 1608-D, 19 pp. and 2 plates. Accessed June 23, 2021 from https://pubs.usgs.gov/wsp/1608d/report.pdf	Central Ground Water Board (2012a). Aquifer Systems of Kerala. Central Ground Water Board Report, 72 pp. Accessed June 23, 2021 from http://cgwb.gov.in/AQM/Kerala.pdf	Varma, A. (2017). Groundwater Resource and Governance in Kerala, Status, Issues and Prospects. Forum for Policy Dialogue on Water Conflicts in India, Pune, Maharashtra, India. Kerala Resource Centre Report, 118 pp. Accessed June 23, 2021 from https://www.soppecom.org/pdf/Groundwater-Resource-and-Governance-in-Kerala.pdf	Approximated from Fig. 1 by Manjusree et al. (2009) and Plate III by Central Ground Water Board (2012a). Easternmost margin of Malabar Coastal Plain approximated along the ~76m topographic contour as detailed for the "lowland" and "midland" physiographic zones in Fig. 1.2 by Varma (2017)
**Southern Mallee**	Murray Basin	Herczeg, A. L., Dogramaci, S. S., Leaney, F. W. J. (2001). Origin of dissolved salts in a large, semi-arid groundwater system: Murray Basin, Australia. Marine and Freshwater Research, 52, 41-52.	Cook, P. G., Jolly, I. D., Leaney, F. W. J. (2001). Groundwater recharge in the Mallee region, and salinity implications for the Murray River: A review. CSIRO Land and Water Report, 133 pp. Accessed July 21, 2021 from https://publications.csiro.au/rpr/download?pid=procite:ef08494d-43a2-4dae-bda4-3d72a62e673f&dsid=DS1	Murray-Darling Basin Commission (1999). Murray-Darling Basin Groundwater - a resource for the future, Murray-Darling Basin Commission, Canberra. Accessed July 21, 2021 from https://www.mdba.gov.au/sites/default/files/archived/mdbc-GW-reports/2173_GW_a_resource_for_the_future.pdf	Approximated from Fig. 1 by Herczeg et al. (2001), Fig. 1.1 by Cook et al. (2001), and Fig. 6 by Murray-Darling Basin Commission (1999)
**Southern Malwa Plateau**	Malwa Plateau	Deolankar, S. B. (1980). The Deccan basalts of Maharashtra, India—their potential as aquifers.	Kale, V. S., Bodas, M., Chatterjee, P., Pande, K. (2020). Emplacement history and evolution of the Deccan Volcanic Province,	Duraiswami, R. A., Das, S., Shaikh, T. (2012). Hydrogeological framework of aquifers from the Deccan Traps, India: some insights.	Approximated from Fig. 1 by Deolankar (1980), Fig. 1 by Duraiswami et al. (2012), and Fig. 1 by Kale et al. (2016). Subarea

Aquifer system	Broader aquifer system	Reference A	Reference B	Reference C	Steps to delineate
		Groundwater, 18(5), 434-437.	India. Episodes, 43, 278-299.	Memoir Geological Society of India, 1-15.	based on topography and drainage patterns.
**Southern Maputaland Coastal Plain**	Maputaland Coastal Plain	Mthembu, P. P., Elumalai, V., Brindha, K., Li, P. (2020). Hydrogeochemical processes and trace metal contamination in groundwater: impact on human health in the Maputaland coastal aquifer, South Africa. Exposure and Health, 12(3), 403-426.	-	-	Approximated from Fig. 1 by Mthembu et al. (2020)
**Southern Maryborough Basin**	-	Marshall, S.K., Fontaine, K., Kilgour, P.L., Lewis, S.J. (2015). Regional Hydrogeological Characterisation of the Maryborough Basin, Queensland. Technical report for the National Collaboration Framework Regional Hydrogeology Project, 135 pp. Accessed July 2, 2023 via https://wetlandinfo.des.qld.gov.au/resources/static/pdf/ecology/catchment-stories/gss/marshall-2015.pdf	-	-	Approximated from Fig. 3.1 by Marshall et al. (2015) and regional drainage patterns.
**Southern Mekong Delta**	Mekong Delta	Minderhoud, P. S. J., Erkens, G., Pham, V. H., Bui, V. T., Erban, L., Kooi, H., & Stouthamer, E. (2017). Impacts of 25 years of groundwater extraction on subsidence in the Mekong delta, Vietnam. Environmental Research Letters, 12, 064006.	Ha, Q. K., Ngoc, T. D. T., Le Vo, P., Nguyen, H. Q., Dang, D. H. (2022). Groundwater in Southern Vietnam: Understanding geochemical processes to better preserve the critical water resource. Science of The Total Environment, 807, 151345.	Tran, D.A., Tsujimura, M., Loc, H.H., Dang, D.H., Le Vo, P., Ha, D.T., Trang, N.T.T., Thuc, P.T.B., Dang, T.D., Batdelger, O., Nguyen, T.V. (2021). Groundwater quality evaluation and health risk assessment in coastal lowland areas of the Mekong Delta, Vietnam. Groundwater for	Approximated from Fig. 1 by Ha et al. (2022), Fig. 1 by Minderhoud et al. (2017), and Fig. 1 by Tran et al. (2021)

Aquifer system	Broader aquifer system	Reference A	Reference B	Reference C	Steps to delineate
				Sustainable Development, 15, 100679.	
**Southern Miandoab Plain**	Miandoab Plain	Norouzi, H., Moghaddam, A. A. (2020). Groundwater quality assessment using random forest method based on groundwater quality indices (case study: Miandoab plain aquifer, NW of Iran). Arabian Journal of Geosciences, 13, 1-13.	-	-	Approximated from Fig. 1 by Norouzi and Moghaddam (2020)
**Southern Morongo Basin**	-	Zhen, L., Martin, P. (2011). Geohydrology, simulation of regional groundwater flow, and assessment of water-management strategies, Twentynine Palms area, California: U.S. Geological Survey Scientific Investigations Report 2010–5249, 106 pp. Accessed May 22, 2021 from https://pubs.usgs.gov/sir/2010/5249/pdf/sir20105249.pdf	-	-	Approximated from Fig. 3 by Li and Martin (2011).
**Southern Nallamalai Basin**	Cuddapah Sedimentary Basin	Dar, F. A., Perrin, J., Riotte, J., Gebauer, H. D., Narayana, A. C., Shakeel, A. (2011). Karstification in the Cuddapah Sedimentary Basin, southern India: implications for groundwater resources. Acta Carsologica, 40, 457-472.	Saha, D., Ray, R. K. (2019). Groundwater resources of India: potential, challenges and management. In Groundwater Development and Management. Springer, Cham (ed. Sikdar, P.K.), pp. 19-42.	Chalapathi Rao, N. V., Gibson, S. A., Pyle, D. M., Dickin, A. P. (2004). Petrogenesis of Proterozoic lamproites and kimberlites from the Cuddapah Basin and Dharwar craton, southern India. Journal of Petrology, 45, 907-948.	Approximated from Fig. 1 by Dar et al. (2011), Fig. 2.2 by Saha and Ray (2019), and Fig. 1 by Chalapathi Rao et al. (2004)
**Southern Namaqualand Coastal Aquifer**	Namaqualand Coastal Aquifers	Campbell, E. E., Parker-Nance, T., Bate, G. C. (1992). A compilation of information on the magnitude, nature and	Nell, J. P., Van Huyssteen, C. W. (2014). Geology and groundwater regions to quantify primary salinity, sodicity and alkalinity in	-	Approximated from Fig. 3 on pp. 36 of Campbell et al. (1992) and Fig. 2 by Nell and Van Huyssteen (2014)

Aquifer system	Broader aquifer system	Reference A	Reference B	Reference C	Steps to delineate
		importance of coastal aquifers in Southern Africa. Water Research Commission Report No. 370/1/92, 193 pp. Accessed October 14, 2021 from http://www.wrc.org.za/wp-content/uploads/mdocs/370-1-92.pdf	South African soils. South African Journal of Plant and Soil, 31, 127-135.		
**Southern Neyshabur Plain**	Neyshabur Plain	Bhunia, G. S., Keshavarzi, A., Shit, P. K., Omran, E. S. E., Bagherzadeh, A. (2018). Evaluation of groundwater quality and its suitability for drinking and irrigation using GIS and geostatistics techniques in semiarid region of Neyshabur, Iran. Applied Water Science, 8, 1-16.	Khalili Naft Chali, A., Shahidi, A. (2017). Comparison of Lazy Algorithms and M5 Model to Estimate Groundwater Level (Case Study: Plain Neyshabur). JWSS-Isfahan University of Technology, 21, 15-26. Accessed June 2, 2021 from https://jstnar.iut.ac.ir/article-1-3296-en.html	Izady, A., Davary, K., Alizadeh, A., Ghahraman, B., Sadeghi, M., Moghaddamnia, A. (2012). Application of "panel-data" modeling to predict groundwater levels in the Neishaboor Plain, Iran. Hydrogeology Journal, 20, 435-447.	Approximated from Fig. 1 by Bhunia et al. (2018), Fig. 1 by Izady et al. (2012), and Fig. 1 by Khalili Naft Chali and Shahidi (2017)
**Southern Nuomin-Taoer System**	Songnen Plain	Chen, Z., Wei, W., Liu, J., Wang, Y., Chen, J. (2011). Identifying the recharge sources and age of groundwater in the Songnen Plain (Northeast China) using environmental isotopes. Hydrogeology Journal, 19, 163-176.	Zhang, G., Deng, W., Yang, Y. S., Salama, R. B. (2007). Evolution study of a regional groundwater system using hydrochemistry and stable isotopes in Songnen Plain, northeast China. Hydrological Processes: An International Journal, 21, 1055-1065.	Zhang, B., Song, X.-F., Zhang, Y.-H., Han, D.-M., Tand, C.Y., Yang, L.-H., Wang, Z. L. (2017). The renewability and quality of shallow groundwater in Sanjiang and Songnen Plain, Northeast China. Journal ofIntegrative Agriculture, 16, 229-238.	Approximated from Fig. 1 by Chen et al. (2011), Fig. 1 by Zhang et al. (2007) and Fig. 1 by Zhang et al. (2017)
**Southern Odanah Shale**	-	Rutulis, M. (1986). Aquifer maps of southern Manitoba. Manitoba Water Resources Branch map. Accessed May 5, 2021 from https://www.gov.mb.ca/water/pubs/maps/water/1986	Tokarsky, O. (1985). Hydrogeologic Profile Saskatchewan-Manitoba Boundary. Report prepared for the Prairie Provinces Water Board, 41 pp. Accessed May 5, 2021 from https://www.ppwb.ca/uploa	Betcher, R.N. (1983). Groundwater Availability Map Series - Virden Area (62-F). Manitoba Department of Natural Resoureces map. Accessed May 5, 2021 from https://www.gov.mb.ca/wa	Approximated from maps by Rutulis (1986) and Betcher (1983). The aquifer outline was truncated close to the Saskactewan-Manitoba border because the Odanah Formation is not thought to host sufficient fractures to provide high yields in

Aquifer system	Broader aquifer system	Reference A	Reference B	Reference C	Steps to delineate
		_rutulis_bedrock_aquifers.pdf	ds/media/5c81764f23261/ppwb-report-79-no-maps-en.pdf?v1	ter/pubs/maps/water/1983_betcher_groundwater_availability_map_series_virden.zip	much of this area (read Tokarsky (1986) pages 17-19).
**Southern Owens Valley**	-	Güler, C., Thyne, G.D. (2004). Hydrologic and geologic factors controlling surface and groundwater chemistry in Indian Wells-Owens Valley area, southeastern California, USA. Journal of Hydrology, 285, 177-198.	-	-	Approximated from Fig. 2 by Güler and Thyne (2004)
**Southern Paris Basin**	Paris Basin	Beaudoin, N., Gasparrini, M., David, M. E., Lacombe, O., Koehn, D. (2019). Bedding-parallel stylolites as a tool to unravel maximum burial depth in sedimentary basins: Application to Middle Jurassic carbonate reservoirs in the Paris basin, France. GSA Bulletin, 131, 1239-1254.	Contoux, C., Violette, S., Vivona, R., Goblet, P., Patriarche, D. (2013). How basin model results enable the study of multi-layer aquifer response to pumping: the Paris Basin, France. Hydrogeology Journal, 21, 545-557.	Beccaletto, L., Hanot, F., Serrano, O., Marc, S. (2011). Overview of the subsurface structural pattern of the Paris Basin (France): Insights from the reprocessing and interpretation of regional seismic lines. Marine and Petroleum Geology, 28, 861-879.	Approximated from Fig. 1 by Beaudoin et al. (2019), Fig. 1 by Contoux et al. (2013), and Fig. 1 by Beccaletto et al. (2011)
**Southern Piedmont Plain**	North China Plain	Meng, S., Liu, J., Zhang, Z., Lei, T., Qian, Y., Li, Y., Fei, Y. (2015). Spatiotemporal evolution characteristics study on the precipitation infiltration recharge over the past 50 years in the North China Plain. Journal of Earth Science, 26, 416-424.	Cao, G., Zheng, C., Scanlon, B. R., Liu, J., & Li, W. (2013). Use of flow modeling to assess sustainability of groundwater resources in the North China Plain. Water Resources Research, 49, 159-175.	Wang, S., Song, X., Wang, Q., Xiao, G., Liu, C., Liu, J. (2009). Shallow groundwater dynamics in North China plain. Journal of Geographical Sciences, 19, 175-188.	Approximated from Fig. 1 by Meng et al. (2015), Fig. 1 by Cao et al. (2013) and Fig. 6 by Wang et al. (2009).
**Southern Piedmont Upland**	Piedmont Plateau	Geological Survey of Alabama (2018). Assessment of groundwater resources in Alabama, 2010-16. Geological Survey of Alabama Bulletin 186,	Miller, J.A. (1990). Ground Water Atlas of the United States: Segment 6, Alabama, Florida, Georgia, South Carolina. U.S. Geological Survey	-	Approximated from Fig. 4 of Geological Survey of Alabama (2018) and Fig. 3 of Miller (1990). Northeastern margin of area approximated along the Savannah River.

Aquifer system	Broader aquifer system	Reference A	Reference B	Reference C	Steps to delineate
		462 pp. Accessed April 8, 2021 from https://www.gsa.state.al.us/img/Groundwater/docs/assessment/00_B186_StatewideAssessment_Print_Document.pdf	Hydrologic Atlas 730-G, 30 pp. Accessed April 5, 2021 from https://www.nrc.gov/docs/ML1706/ML17060B027.pdf		
**Southern Qazvin Plain**	Qazvin Plain	Yazdi, Z., Niroumand, H. (2020). Assessing land subsidence in Qazvin plain caused by groundwater level drop, using finite elements and finite difference methods. GeoTerrace-2020-043, 5 pp. Accessed June 2, 2021 from https://eage.in.ua/wp-content/uploads/2020/12/GeoTerrace-2020-043.pdf	Faghihi, N., Kave, F., Babazadeh, H. (2010). Prediction of aquifer reaction to different hydrological and management scenarios using visual MODFLOW model-Case study of Qazvin plain. Journal of Water Sciences Research 2, 39-45.	Babaee, S., Mousavi, Z., Masoumi, Z., Malekshah, A. H., Roostaei, M., Aflaki, M. (2020). Land subsidence from interferometric SAR and groundwater patterns in the Qazvin plain, Iran. International Journal of Remote Sensing, 41, 4780-4798.	Approximated from Fig. 1 by Yazdi and Niroumand (2020), with zones approximated from Fig. 7 by Faghihi et al. (2010; "Zone3" and "Zone4" outlines estimated for this southern subarea)
**Southern Railroad Valley**	-	Lopes, T.J., Evetts. D.M. (2005). Ground-Water pumpage and artificial recharge estimates for calendar year 2000 and average annual natural recharge and interbasin flow by hydrographic area, Nevada. Accessed April 25, 2022 via https://pubs.usgs.gov/sir/2004/5239/sir2004-5239.pdf	Harrill, J.R., Prudic, D.E. (1998). Aquifer systems in the Great Basin region of Nevada, Utah, and adjacent states; summary report. US Geological Survey Professional Paper 1409-A, 75 pp. Accessed April 26, 2022 via https://pubs.usgs.gov/pp/1409a/report.pdf	-	Approximated from Fig. 1 by Lopes and Evetts (2005), and Fig. 2 by Harrill and Prudic (1998). Well completion records, physiography and drainage were used to delinate areas.
**Southern Razan Plain**	Razan Basin	Pazand, K. (2016). Geochemical and statistical evaluation of groundwater in Razan basin, Western Iran. Carbonates and Evaporites, 31, 179-185.	-	-	Approximated from Fig. 1 by Pazand (2016)
**Southern Riverine Plains**	Murray Basin	Herczeg, A. L., Dogramaci, S. S., Leaney, F. W. J. (2001). Origin of dissolved	Barnett, S., Harrington, N., Cook, P., & Simmons, C. T. (2020). Groundwater in	Murray-Darling Basin Commission (1999). Murray-Darling Basin	Approximated from Fig. 1 by Herczeg et al. (2001), Fig. 6.2 by Barnett et al. (2020) and Fig. 6 by

Aquifer system	Broader aquifer system	Reference A	Reference B	Reference C	Steps to delineate
		salts in a large, semi-arid groundwater system: Murray Basin, Australia. Marine and Freshwater Research, 52, 41-52.	Australia: Occurrence and Management Issues. In: Sustainable Groundwater Management: A Comparative Analysis of French and Australian Policies and Implications to Other Countries (eds. Rinaudo, J.-D., Hollet, C., Barnett, S., Montginoul, M.), 24, 109-127	Groundwater - a resource for the future, Murray-Darling Basin Commission, Canberra. Accessed July 21, 2021 from https://www.mdba.gov.au/sites/default/files/archived/mdbc-GW-reports/2173_GW_a_resource_for_the_future.pdf	Murray-Darling Basin Commission (1999)
**Southern San Gabriel Valley**	San Gabriel Valley	California Department of Water Resources (2015e). California's groundwater update 2013 - Chapter 6: South Coast Hydrologic Region. California Department of Water Resources Report, 84 pp. Accessed April 27, 2022 via https://cawaterlibrary.net/wp-content/uploads/2017/05/GWU2013_Ch6_SouthCoast_Final.pdf	-	-	Approximated from Fig. 6-2 by California Department of Water Resources (2015e)
**Southern San Juan Basin**	-	Kernodle, J. M. (1996). Hydrogeology and steady-state simulation of ground-water flow in the San Juan Basin, New Mexico, Colorado, Arizona, and Utah. US Geological Survey Water-Resources Investigations Report 95-4187, 126 pp. Accessed March 12, 2021 from https://pubs.usgs.gov/wri/1995/4187/report.pdf	-	-	Approximated from Fig. 3 of Kernodle (1996). Southern portion approximated on the basis of groundwater well density (derived from New Mexico's well completion dataset).
**Southern Santiago Basin**	Santiago Basin	Araneda, M., Avendaño, M. S., Del Río, G. D. (2010). Modelo estructural de la	Steinbrügge, G., Muñoz Pardo, J. F., Fernández, B. (2005). Análisis	-	Approximated from Figs. 2 and 7 by Araneda et al. (2010). Northwestern boundary of subarea

Aquifer system	Broader aquifer system	Reference A	Reference B	Reference C	Steps to delineate
		cuenca de Santiago, Chile y su relación con la hidrogeología. Revista Geofísica, 62, 29-48.	probabilístico y optimización de los recursos de agua subterránea: el caso del acuífero Maipo-Mapocho, Chile. Ingenieria hidraulica en Mexico, XX, 85-97. Accessed May 15, 2021 from https://repositorio.uc.cl/xmlui/bitstream/handle/11534/28539/An%C3%A1lisis%20probabil%C3%ADstico%20y%20optimizaci%C3%B3n%20de%20los%20recursos%20de%20agua%20subterr%C3%A1nea%20el%20caso%20del%20acu%C3%ADfero%20Maipo-Mapocho,%20Chile.pdf?sequence=1		approximated along the Rio Maipo. See also Illustration 3 by Steinbrügge et al. (2005).
**Southern Sarbisheh Basin**	-	Hosseini, S. M., Parizi, E., Ataie-Ashtiani, B., Simmons, C. T. (2019). Assessment of sustainable groundwater resources management using integrated environmental index: Case studies across Iran. Science of the Total Environment, 676, 792-810.	-	-	Approximated from Fig. 6 by Hosseini et al. (2019)
**Southern Sarkhoon Aquifer**	Sarkhoon Aquifer	Bazrafshan, O., Parandin, F., Farokhzadeh, B. (2016). Assessment of hydro-meteorological drought effects on groundwater resources in Hormozgan region-South of Iran. Ecopersia, 4, 1569-1584.	-	-	Approximated from map on page 1571 of Bazrafshan et al. (2016)

Aquifer system	Broader aquifer system	Reference A	Reference B	Reference C	Steps to delineate
**Southern Saurashtra Deccan Trap Basalts**	Saurashtra Deccan Trap Basalts	Saha, D., Ray, R. K. (2019). Groundwater resources of India: potential, challenges and management. In Groundwater Development and Management. Springer, Cham (ed. Sikdar, P.K.), pp. 19-42.	Shah, T. (2014). Towards a Managed Aquifer Recharge strategy for Gujarat, India: An economist's dialogue with hydro-geologists. Journal of Hydrology, 518, 94-107.	Kulkarni, H., Deolankar, S. B., Lalwani, A., Joseph, B., Pawar, S. (2000). Hydrogeological framework of the Deccan basalt groundwater systems, west-central India. Hydrogeology Journal, 8, 368-378.	Approximated from Fig. 2.2 by Saha and Ray (2019), Figs. 1, 2 and 4 by Shah (2014) and Fig. 1 by Kulkarni et al. (2000)
**Southern Shamil-Meymand Basin**	-	Hosseini, S. M., Parizi, E., Ataie-Ashtiani, B., Simmons, C. T. (2019). Assessment of sustainable groundwater resources management using integrated environmental index: Case studies across Iran. Science of the Total Environment, 676, 792-810.	-	-	Approximated from Fig. 6 by Hosseini et al. (2019)
**Southern St. Lawrence Lowlands**	St. Lawrence Lowlands	Parent, M., Rivard, C., Lefebvre, R., Carrier, M.-A., Séjourné, S. (2014). Hydrogeological systems of the Montérégie Est region, southern Québec: Fieldtrip Guidebook, GeoMontreal 2013 Conference, Geological Survey of Canada Open File 7605, 43 pp.	Olcott, P.G. (1995). Ground Water Atlas of the United States: Segment 12, Connecticut, Maine, Massachusetts, New Hampshire, New York, Rhode Island, Vermont. U.S. Geological Survey Hydrologic Atlas 730-M, 30 pp. Accessed April 14, 2021 from https://pubs.usgs.gov/ha/730m/report.pdf	Patenaude, M., Baudron, P., Labelle, L., Masse-Dufresne, J. (2020). Evaluating bank-filtration occurrence in the Province of Quebec (Canada) with a GIS approach. Water 12, 662.	St. Lawrence Lowlands hydrogeological context approximated from Fig. 2 of Parent et al. (2014; including the divide between northern and southern portions of the broader aquifer system) and Fig. 4 by Patenaude et al. (2020); see also Fig. 102 by Olcutt (1995).
**Southern Swan Coastal Plain**	Perth Basin	Raper, G.P., Speed, R., Simons, J.A., Killen, A.L., Blake, A., Ryder, A.T., Smith, R.H., Stainer, G., Bourke, L. (2014). Groundwater trend analysis and salinity risk assessment for the south-west	Ali, R., McFarlane, D., Varma, S., Dawes, W., Emelyanova, I., Hodgson, G., Charles, S. (2012). Potential climate change impacts on groundwater resources of south-western	-	Approximated from Fig. 1 by Raper et al. (2014) and from Fig. 1b by Ali et al. (2012)

Aquifer system	Broader aquifer system	Reference A	Reference B	Reference C	Steps to delineate
		agricultural region of Western Australia, 2007–12. Government of Western Australia Department of Agriculture and Food Resource Management Technical Report 388, 148 pp. Accessed July 26, 2021 from https://researchlibrary.agric.wa.gov.au/cgi/viewcontent.cgi?article=1372&context=rmtr	Australia. Journal of Hydrology, 475, 456-472.		
**Southern Taichung Basin**	Taichung Basin	Wang, S. J., Lee, C. H., Yeh, C. F., Choo, Y. F., Tseng, H. W. (2021). Evaluation of Climate Change Impact on Groundwater Recharge in Groundwater Regions in Taiwan. Water, 13, 1153.	-	-	Approximated from Fig. 2 by Wang et al. (2021)
**Southern Tikapoo Valley**	-	Lopes, T.J., Evetts. D.M. (2005). Ground-Water pumpage and artificial recharge estimates for calendar year 2000 and average annual natural recharge and interbasin flow by hydrographic area, Nevada. Accessed April 25, 2022 via https://pubs.usgs.gov/sir/2004/5239/sir2004-5239.pdf	Harrill, J.R., Prudic, D.E. (1998). Aquifer systems in the Great Basin region of Nevada, Utah, and adjacent states; summary report. US Geological Survey Professional Paper 1409-A, 75 pp. Accessed April 26, 2022 via https://pubs.usgs.gov/pp/1409a/report.pdf	-	Approximated from Fig. 1 by Lopes and Evetts (2005), and Fig. 2 by Harrill and Prudic (1998). Well completion records, physiography and drainage were used to delinate areas.
**Southern Tule Lake**	Upper Klamath Basin	Gannett, M.W., Breen, K.H. (2015). Groundwater levels, trends, and relations to pumping in the Bureau of Reclamation Klamath Project, Oregon and California: U.S. Geological Survey Open-File Report	-	-	Approximated from Fig. 1 of Gannett and Breen (2015).

Aquifer system	Broader aquifer system	Reference A	Reference B	Reference C	Steps to delineate
		2015-1145, 19 pp. Accessed March 20, 2021 from https://pubs.usgs.gov/of/2015/1145/ofr20151145.pdf			
**Southern Utah Valley**	-	Brooks, L.E. (2013). Evaluation of the groundwater flow model for southern Utah and Goshen Valleys, Utah, updated to conditions through 2011, with new projections and groundwater management simulations: U.S. Geological Survey Open-File Report 2013–1171, 35 pp. Accessed March 7, 2021 from https://pubs.usgs.gov/of/2013/1171/pdf/ofr2013-1171.pdf	Selck, B. J., Carling, G. T., Kirby, S. M., Hansen, N. C., Bickmore, B. R., Tingey, D. G., Rey, K., Wallace, J. Jordan, J. L. (2018). Investigating anthropogenic and geogenic sources of groundwater contamination in a semi-arid alluvial basin, Goshen Valley, UT, USA. Water, Air, Soil Pollution, 229(6), 1-17.	-	Approximated from Fig. 1 of Brooks (2013)
**Southern Valley and Ridge**	Valley and Ridge Aquifer System	Johnson, G.C., Zimmerman, T.M., Lindsey, B.D., Gross, E.L. (2011). Factors affecting groundwater quality in the Valley and Ridge aquifers, eastern United States, 1993–2002: U.S. Geological Survey Scientific Investigations Report 2011–5115, 84 pp. Accessed April 13, 2021 from https://pubs.usgs.gov/sir/2011/5115/support/sir2011-5115.pdf	Geological Survey of Alabama (2018). Assessment of groundwater resources in Alabama, 2010-16. Geological Survey of Alabama Bulletin 186, 462 pp. Accessed April 8, 2021 from https://www.gsa.state.al.us/img/Groundwater/docs/assessment/00_B186_StatewideAssessment_Print_Document.pdf	-	Northern margin approximated along the Watauga River. Broader Valley and Ridge Aquifer System outline approximated from Fig. 1 of Johnson et al. (2011), with southernmost extent approximated from Fig. 4 of Geological Survey of Alabama (2018)
**Southern Vienna Basin**	Vienna Basin	Stolp, B. J., Solomon, D. K., Suckow, A., Vitvar, T., Rank, D., Aggarwal, P. K., Han, L. F. (2010). Age	Kováč, M. I. C. H. A. L., Sliva, L., Sopkova, B., Hlavata, J., & Škulová, A. D. R. I. A. N. A. (2008).	-	Approximated from Fig. 1 by Stolp et al. (2010) and Fig. 1 by Kováč et al. (2008)

Aquifer system	Broader aquifer system	Reference A	Reference B	Reference C	Steps to delineate
		dating base flow at springs and gaining streams using helium-3 and tritium: Fischa-Dagnitz system, southern Vienna Basin, Austria. Water Resources Research, 46, W07503.	Serravallian sequence stratigraphy of the northern Vienna Basin: high frequency cycles in the Sarmatian sedimentary record. Geologica Carpathica, 59, 545-561.		
**Southern Willamette Valley**	Willamette Valley	Woodward, D.G., Gannett, M.W., Vaccaro, J.J. (1998). Hydrogeologic framework of the Willamette Lowland aquifer system, Oregon and Washington. US Geological Survey Professional Paper 1424-B, 92 pp. Accessed February 16, 2021 from https://pubs.usgs.gov/pp/1424b/report.pdf	Herrera, N.B., Burns, E.R., Conlon, T.D. (2014). Simulation of groundwater flow and the interaction of groundwater and surface water in the Willamette Basin and Central Willamette Subbasin, Oregon. US Geological Survey Scientific Investigations Report 2014–5136, 174 pp. Accessed February 16, 2021 from https://pubs.usgs.gov/sir/2014/5136/pdf/sir20145136.pdf	-	Approximated from Fig. 2 of Woodward et al. (1998) and Fig. 1 by Herrera et al. (2014). Basin extent was broadened to include foothills of surrounding mountains, as a considerable number of wells exist in these areas. The Waldo and Salem Hills were used to divide this southern portion of the aquifer system from the Central Willamette Valley to the north.
**Southern Zona Citricola Aquifer**	Gulf Coast Regional Aquifer System	Pastén-Zapata, E., Ledesma-Ruiz, R., Harter, T., Ramírez, A. I., Mahlknecht, J. (2014). Assessment of sources and fate of nitrate in shallow groundwater of an agricultural area by using a multi-tracer approach. Science of the Total Environment, 470, 855-864.	Ledesma-Ruiz, R., Pastén-Zapata, E., Parra, R., Harter, T., & Mahlknecht, J. (2015). Investigation of the geochemical evolution of groundwater under agricultural land: a case study in northeastern Mexico. Journal of Hydrology, 521, 410-423.	-	Approximated from Fig. 1 by Pastén-Zapata et al. (2014) and Fig. 2 by Ledesma-Ruiz et al. (2015). The area is within the "Coastal Plain of the Gulf of Mexico" (quoting Pastén-Zapata et al., 2014) and thus was included in the "Gulf Coast Regional Aquifer System.
**Southern Zululand Primary Coastal Aquifer**	-	Kelbe, B. E., Germishuyse, T. (2001). Geohydrological Studies of the Primary Coastal Aquifer in Zululand. Water Research	South African Department of Water Affairs (2012). Aquifer classification of South Africa. Map, 1 pp. Accessed October 18, 2021	-	Approximated from Fig. 47 by Kelbe and Germishuyse (2001) and South African Department of Water Affairs (2012)

Aquifer system	Broader aquifer system	Reference A	Reference B	Reference C	Steps to delineate
		Commission Report No. K5/720/1/01.	from https://www.dws.gov.za/Groundwater/documents/Aquifer%20Classification.pdf		
**Southwest Rayong Basin**	Rayong Basin	Boonkaewwan, S., Sonthiphand, P., Chotpantarat, S. (2021). Mechanisms of arsenic contamination associated with hydrochemical characteristics in coastal alluvial aquifers using multivariate statistical technique and hydrogeochemical modeling: a case study in Rayong province, eastern Thailand. Environmental Geochemistry and Health, 43, 537-566.	Piyapong, J., Thidarat, B., Jaruwan, C., Siriphan, N., Passanan, A. (2019). Enhancing citizens' sense of personal responsibility and risk perception for promoting public participation in sustainable groundwater resource management in Rayong Groundwater Basin, Thailand. Groundwater for Sustainable Development, 9, 100252.	-	Approximated from Fig. 1 by Boonkaewwan et al. (2021), Fig. 2 by Piyapong et al. (2019). Subareas delineated on the basis of physiography, drainage and groundwater well prevelance
**Southwestern Allegheny Plateau**	Appalachian Plateaus	Brockman, S. (1998). Physiographic regions of Ohio. Ohio Department of Natural Resources, Division of Geological Survey map. Accessed April 12, 20221 from https://www.epa.state.oh.us/portals/27/SIP/Nonattain/F2-physiographic_regions_of_Ohio.pdf	-	-	Approximated from map by Brockman (1998). Northern boundary approximated along the Hackman River.
**Southwestern Aquitaine Basin**	Aquitaine Basin	Rouillard, J., Maréchal, J.-C. (2020). Groundwater in France: Resources, Use and Management Issues. In: Sustainable Groundwater Management A Comparative Analysis of French and Australian Policies and Implications to	Willmes, M., Bataille, C.P., James, H.F., Moffat, I., McMorrow, L., Kinsley, L., Armstrong, R.A., Eggins, S., Grün, R. (2018). Mapping of bioavailable strontium isotope ratios in France for archaeological	Biteau, J. J., Le Marrec, A., Le Vot, M., Masset, J. M. (2006). The aquitaine basin. Petroleum Geoscience, 12, 247-273.	Approximated from Fig. 1 by Rouillard and Maréchal (2020), and Fig. 1 by Biteau et al.(2006), with broader outline of Aquitaine Basin supported by delineation in Fig. 1 by Willmes et al. (2018).

Aquifer system	Broader aquifer system	Reference A	Reference B	Reference C	Steps to delineate
		Other Countries, pp. 17-45. Accessed May 22, 2021 from https://hal.archives-ouvertes.fr/hal-02512154/document	provenance studies. Applied Geochemistry 90, 75-86.		
**Southwestern Bengal Basin**	Bengal Basin	Woodman, N. D., Burgess, W. G., Ahmed, K. M., Zahid, A. (2019). A partially coupled hydro-mechanical analysis of the Bengal Aquifer System under hydrological loading. Hydrology and Earth System Sciences, 23, 2461-2479.	Ravenscroft, P., McArthur, J. M., Rahman, M. S. (2018). Identifying multiple deep aquifers in the Bengal Basin: Implications for resource management. Hydrological Processes, 32, 3615-3632.	Michael, H. A., Voss, C. I. (2009). Controls on groundwater flow in the Bengal Basin of India and Bangladesh: regional modeling analysis. Hydrogeology Journal, 17, 1561-1577.	Approximated from Fig. 1 by Woodman et al. (2019), Fig. 1 by Ravenscroft et al. (2018), and Fig. 1 by Michael and Voss (2009)
**Southwestern Bohemian Cretaceous Basin**	Bohemian Cretaceous Basin	Alvarado, J. C., Pačes, T., Purtschert, R. (2013). Dating groundwater in the Bohemian Cretaceous Basin: Understanding tracer variations in the subsurface. Applied Geochemistry, 29, 189-198.	Jiráková, H., Huneau, F., Hrkal, Z., Celle-Jeanton, H., Le Coustumer, P. (2010). Carbon isotopes to constrain the origin and circulation pattern of groundwater in the north-western part of the Bohemian Cretaceous Basin (Czech Republic). Applied Geochemistry, 25, 1265-1279.	Kadlecová, R., Olmer, M. (2011). Review of Groundwater Resources. Geologické výzkumy na Moravě a ve Slezsku. Proceedings of The Open Congress ČGS and SGS, September 22–25, 2011, Monínec 18, pp. 31-34. Accessed October 21, 2021 from https://journals.muni.cz/gvms/article/view/1679	Approximated from Fig. 1 by Jiráková et al. (2010), Fig. 1 by Alvarado et al. (2013), and Fig. 1 by Kadlecová and Olmer (2011)
**Southwestern Chaco-Lower Parana Basin**	Guarani Aquifer System	Hirata, R., Foster, S. (2021). The Guarani Aquifer System–from regional reserves to local use. Quarterly Journal of Engineering Geology and Hydrogeology, 54, qjegh2020-091.	Gonçalves, R. D., Teramoto, E. H., Chang, H. K. (2020). Regional Groundwater Modeling of the Guarani Aquifer System. Water, 12, 2323.	Rodríguez, L., Vives, L., Gomez, A. (2013). Conceptual and numerical modeling approach of the Guarani Aquifer System. Hydrology and Earth System Sciences, 17, 295-314.	Approximated from Fig. 2 by Gonçalves et al. (2020) and Fig. 1 by Hirata and Foster (2021), who describe the three regions of the Guarani Aquifer System as delimited by two major arches: (i) the Ponta Grossa Arch (quote Hirata and Foster (2021): "which forces groundwater to flow from east to west in São Paulo State"), and (ii) the Asunción–Rio Grande (quote Hirata and Foster (2021): "..which divides the portion south

Aquifer system	Broader aquifer system	Reference A	Reference B	Reference C	Steps to delineate
					of the Ponta Grossa Arch into two semi-independent sedimentary basins"). The locations of these aforementioned arches were approximated from Fig. 2 by Rodríguez et al. (2013). Subarea name derives from discussion in section 2 of publication by Rodríguez et al. (2013).
**Southwestern Coastal Sandveld and Elandsfontein Aquifer**	-	Nell, J. P., Van Huyssteen, C. W. (2014). Geology and groundwater regions to quantify primary salinity, sodicity and alkalinity in South African soils. South African Journal of Plant and Soil, 31, 127-135.	-	-	Approximated from Fig. 2 by Nell and Van Huyssteen (2014)
**Southwestern Flatwoods**	Floridan Aquifer System	Williams, L.J., Kuniansky, E.L. (2016). Revised hydrogeologic framework of the Floridan aquifer system in Florida and parts of Georgia, Alabama, and South Carolina. U.S. Geological Survey Professional Paper 1807, 140 pp. Accessed March 31, 2021 from https://pubs.usgs.gov/pp/1807/pdf/pp1807.pdf	Brooks, H. K. (1981). Physiographic divisions of Florida. Report for the Florida Cooperative Extension Service Institute of Food and Agricultural Sciences, University of Florida, 12 pp.	-	Approximated from physiographic provinces of Florida outlined by Brooks (1981). Broader Florida Aquifer System from Williams and Kuniansky (2016).
**Southwestern Jazmurian Depression**	Jazmurian Depression	Rezaei, A., Hassani, H., Hassani, S., Jabbari, N., Mousavi, S. B. F., Rezaei, S. (2019). Evaluation of groundwater quality and heavy metal pollution indices in Bazman basin, southeastern Iran. Groundwater for Sustainable Development, 9, 100245.	-	-	Jazmurian Depression approximated from Fig. 2 by Rezaei et al. (2019)

Aquifer system	Broader aquifer system	Reference A	Reference B	Reference C	Steps to delineate
**Southwestern Khorat Plateau**	Khorat Plateau Aquifer System	LaMoreaux, P. E., Charaljavanaphet, J., Jalichan, N., Chiengmai, P. P. N., Bunnag, D., Thavisri, A., Rakprathum, C. (1958). Reconnaissance of the geology and ground water of the Khorat Plateau, Thailand. US Geological Survey Water Supply Paper 1429, 76 pp. Accessed November 4, 2021 from https://pubs.usgs.gov/wsp/1429/report.pdf	Phiancharoen, C. (1962). Interpretation of the chemical analyses of the ground water of the Khorat Plateau, Thailand. MSc Thesis, University of Arizona, 177 pp. Accessed November 4, 2021 from https://repository.arizona.edu/handle/10150/191432	-	Approximated from Plate 4 by LaMoreaux et al. (1958) and Plate 1 by Phiancharoen (1962). Subarea boundaries defined on the basis of physigraphy (e.g., watershed divides), drainage and well drilling data
**Southwestern Qaderabad Basin**	-	Hosseini, S. M., Parizi, E., Ataie-Ashtiani, B., Simmons, C. T. (2019). Assessment of sustainable groundwater resources management using integrated environmental index: Case studies across Iran. Science of the Total Environment, 676, 792-810.	-	-	Approximated from Fig. 6 by Hosseini et al. (2019)
**Spanish Springs Valley**	-	Berger, D. L., Ross, W. C., Thodal, C. E., Robledo, A. R. (1997). Hydrogeology and simulated effects of urban development on water resources of Spanish Springs Valley, Washoe County, West-Central Nevada. U.S. Geological Survey Water-Resources Investigations Report 96-4297, 87 pp. Accessed March 22, 2021 from https://pubs.usgs.gov/wri/1996/4297/report.pdf	-	-	Approximated from Fig. 4 of Berger et al. (1997)

Aquifer system	Broader aquifer system	Reference A	Reference B	Reference C	Steps to delineate
**Spokane Valley-Rathdrum Prairie Aquifer**	Columbia Plateau Regional Aquifer System	Kahle, S.C., Morgan, D.S., Welch, W.B., Ely, S.R., Vaccaro, J.J., Orzol, L.L. (2011). Hydrogeologic Framework and Hydrologic Budget Components of the Columbia Plateau Regional Aquifer System, Washington, Oregon, and Idaho. US Geological Survey Scientific Investigations Report 2011–5124, 80 pp. Accessed February 16, 2021 from https://pubs.usgs.gov/sir/2011/5124/pdf/sir20115124.pdf	Hsieh, P. A., Barber, M. E., Contor, B. A., Hossain, M., Johnson, G. S., Jones, J. L., Wylie, A. H. (2007). Ground-water flow model for the Spokane valley-Rathdrum prairie aquifer, Spokane County, Washington, and Bonner and Kootenai Counties, Idaho. U. S. Geological Survey Scientific Investigations Report 2007-5044, 90 pp. Accessed February 18, 2021 from https://pubs.usgs.gov/sir/2007/5044/pdf/sir20075044.pdf	Graham, W.G., Campbell, L.J. (1981). Groundwater resources of Idaho. Idaho Department of Water Resources Report, 61 pp. Accessed March 23, 2021 from https://idwr.idaho.gov/files/publications/198108-MISC-GW-Resources-ID.pdf	Spokane Valley-Rathdrum Prairie Aquifer approximated from Fig. 1 of Hseih et al. (2007). Columbia River Aquifer System approximated from Figs. 1 and 10 of Kahle et al. (2011). Idaho portion delineated from Graham and Campbell (1981).
**Sprague Basin**	Upper Klamath Basin	Gannett, M. W., Lite, K. E., La Marche, J. L., Fisher, B. J., Polette, D. J. (2007). Ground-water hydrology of the upper Klamath Basin, Oregon and California. U. S. Geological Survey Scientific Investigations Report 2007–5050. 98 pp. Accessed March 20, 2021 from https://pubs.usgs.gov/sir/2007/5050/pdf/sir20075050.pdf	-	-	Approximated from Fig. 18 of Gannett et al. (2007)
**Spring Valley**	-	Gardner, P. M., Masbruch, M. D. (2015). Hydrogeologic and geochemical characterization of groundwater resources in Deep Creek Valley and adjacent areas, Juab and Tooele Counties, Utah, and	-	-	Approximated from Fig. 6 of Gardner and Masbruch (2015)

Aquifer system	Broader aquifer system	Reference A	Reference B	Reference C	Steps to delineate
		Elko and White Pine Counties, Nevada. US Geological Survey Scientific Investigations Report 2015–5097, 66 pp. Accessed March 10, 2021 from https://pubs.usgs.gov/sir/2015/5097/sir20155097.pdf			
**Springbok Flats Karoo Basin**	-	Nel, L. (2012). The geology of the Springbok Flats. PhD Dissertation, University of the Free State, 258 pp. Accessed October 18, 2021 from https://scholar.ufs.ac.za/handle/11660/5485	Heaton, T. H. E. (1985). Isotopic and chemical aspects of nitrate in the groundwater of the Springbok Flats. Water SA, 11, 199-208.	-	Approximated from Fig. 1 by Heaton (1985) and Fig. 1.1 by Nel (2012)
**Springfield Plain**	Central Lowland Till Plain	Leighton, M. M., Ekblaw, G. E., Horberg, L. (1948). Physiographic divisions of Illinois. The Journal of Geology, 56, 16-33.	Fenneman, N.M., Johnson, D.W. (1946). Physiographic divisions of the conterminous United States. U.S. Geological Survey map, 1:7,000,000 Scale.	Gray, H.H. (2001). Map of Indiana showing physiographic divisions. Indiana Geological Survey Miscellaneous Map 69, 15 pp. Accessed April 12, 2021 from https://igws.indiana.edu/ReferenceDocs/Maps/PhysiographicRegions.pdf	Broader Till Plain physiographic region approximated from Fenneman and Johnson (1946). Springfield Plain approximated from Figs. 1 and 2 of Leighton et al. (1948); northern portion of "Wabash Lowland" delineated by Gray (2001) was included for the Indiana portion of the subarea
**Srisailam Basin**	Cuddapah Sedimentary Basin	Dar, F. A., Perrin, J., Riotte, J., Gebauer, H. D., Narayana, A. C., Shakeel, A. (2011). Karstification in the Cuddapah Sedimentary Basin, southern India: implications for groundwater resources. Acta Carsologica, 40, 457-472.	Saha, D., Ray, R. K. (2019). Groundwater resources of India: potential, challenges and management. In Groundwater Development and Management. Springer, Cham (ed. Sikdar, P.K.), pp. 19-42.	Chalapathi Rao, N. V., Gibson, S. A., Pyle, D. M., Dickin, A. P. (2004). Petrogenesis of Proterozoic lamproites and kimberlites from the Cuddapah Basin and Dharwar craton, southern India. Journal of Petrology, 45, 907-948.	Approximated from Fig. 1 by Dar et al. (2011), Fig. 2.2 by Saha and Ray (2019), and Fig. 1 by Chalapathi Rao et al. (2004)
**Stampriet Kalahari Karoo**	-	Uugulu, S., Wanke, H. (2020). Estimation of groundwater recharge in savannah aquifers along a	JICA (2002). The study on the groundwater potential evaluation and management plan in the	Abiye, T.A. (2012). Groundwater need assessment ORASECOM. Africa Groundwater	Approximated from Fig. 1 by Uugulu and Wanke, H. (2020), map on page 3 of JICA (2002) and Fig. 1 by Abiye (2012)

Aquifer system	Broader aquifer system	Reference A	Reference B	Reference C	Steps to delineate
**Aquifer System**		precipitation gradient using chloride mass balance method and environmental isotopes, Namibia. Physics and Chemistry of the Earth, Parts A/B/C, 116, 102844.	southeast Kalahari (Stampriet) Artesian Basin in The Republic of Namibia. 33 pp. Accessed October 14, 2021 from https://openjicareport.jica.go.jp/pdf/11681699_01.PDF	Network (AGWNET) Report, 56 pp.	
**Staten River Embayment and Central Carpentaria Basin**	Great Artesian Basin	Ransley, T.R., Smerdon, B.D. (2012). Hydrostratigraphy, hydrogeology and system conceptualisation of the Great Artesian Basin. A technical report to the Australian Government from the CSIRO Great Artesian Basin Water Resource Assessment, 324 pp. Accessed July 20, 2021 from https://publications.csiro.au/rpr/download?pid=csiro:EP132693&dsid=DS5	-	-	Approximated from Fig. 2.4 by Ransley and Smerdon (2012)
**Stellarton Basin**	Maritimes Basin	Stapinsky, M. Michaud, Y., Morin, R.H., Butler, K.E., Deblonde, C., Chi, G, Thériault, T., Castonguay, S., Boisvert, V., Julien, H.P., Conohan, D., Hulsman, B., Carrier, C. Marion, J., Boisvert, É. (2002). Groundwater resources assessment in the Carboniferous Maritimes Basin: preliminary results of the hydrogeological characterization, New Brunswick, Nova Scotia, and Prince Edward Island.	Rivard, C., Michaud, Y., Lefebvre, R., Deblonde, C., Rivera, A. (2008). Characterization of a regional aquifer system in the Maritimes Basin, Eastern Canada. Water Resources Management, 22, 1649-1675.	Bianco, E. (2013). Seismic Interpretation of the Windsor-Kennetcook Basin. Geological Survey of Canada Open File 7452, 31 pp. Accessed May 5, 2021 from https://ftp.maps.canada.ca/pub/nrcan_rncan/publications/STPublications_PublicationsST/292/292763/of_7452.pdf	Approximated from Fig. 2 of Rivard et al. (2008) and Figs. 1 and 3 of Stapinsky et al. (2002). Area bounds approximated from Fig. 1 of Bianco (2013).

Aquifer system	Broader aquifer system	Reference A	Reference B	Reference C	Steps to delineate
		Geological Survey of Canada Current Research Report 2002-D8, 14 pp. Accessed May 5, 2021 from http://www.gov.pe.ca/photos/original/cle_WA10.pdf			
**Steptoe Valley**	-	Frick, E. (1985). Quantitative analysis of groundwater flow in valley-fill deposits in Steptoe Valley, Nevada. Doctoral dissertation, University of Nevada, Reno. 199 pp. Accessed March 10, 2021 from https://scholarworks.unr.edu/bitstream/handle/11714/1293/Mackay219_Frick.pdf?sequence=1	-	-	Approximated from Fig. 1 of Frick (1985)
**Stilbaai Coastal Aquifer**	-	Campbell, E. E., Parker-Nance, T., Bate, G. C. (1992). A compilation of information on the magnitude, nature and importance of coastal aquifers in Southern Africa. Water Research Commission Report No. 370/1/92, 193 pp. Accessed October 14, 2021 from http://www.wrc.org.za/wp-content/uploads/mdocs/370-1-92.pdf	-	-	Approximated from Fig. 3 on pp. 36 of Campbell et al. (1992)
**Stockton Plateau**	Edwards-Trinity Aquifer System	Bruun, B., Jackson, K., Lake, P., Walker, J. (2016). Texas Aquifers Study. Texas Water Development Board Report. 336 pp. Accessed April 1, 2021 from https://www.twdb.texas.go	Barker, R. A., Ardis, A. F. (1996). Hydrogeological framework of the Edwards-Trinity aquifer system, west-central Texas. U.S. Geological Survey Professional Paper 1421-B,	Ryder, P. (1996). Ground Water Atlas of the United States Segment 4 Oklahoma, Texas. Hydrologic Investigations Atlas 730-E, 32 pp. Accessed November 29,	Approximated from Fig. 6-11 by Bruun et al. (2016), Fig. 84 by Ryder (1996), and Fig. 3 by Barker and Ardis (1996).

Aquifer system	Broader aquifer system	Reference A	Reference B	Reference C	Steps to delineate
		v/groundwater/docs/studies/TexasAquifersStudy_2016.pdf#page=89	76 pp. Accessed April 1, 2021 from https://pubs.usgs.gov/pp/1421b/report.pdf	2021 from https://pubs.usgs.gov/ha/730e/report.pdf	
**Stone Cabin Valley**	-	Lopes, T.J., Evetts. D.M. (2005). Ground-Water pumpage and artificial recharge estimates for calendar year 2000 and average annual natural recharge and interbasin flow by hydrographic area, Nevada. Accessed April 25, 2022 via https://pubs.usgs.gov/sir/2004/5239/sir2004-5239.pdf	Harrill, J.R., Prudic, D.E. (1998). Aquifer systems in the Great Basin region of Nevada, Utah, and adjacent states; summary report. US Geological Survey Professional Paper 1409-A, 75 pp. Accessed April 26, 2022 via https://pubs.usgs.gov/pp/1409a/report.pdf	-	Approximated from Fig. 1 by Lopes and Evetts (2005), and Fig. 2 by Harrill and Prudic (1998). Well completion records, physiography and drainage were used to delinate areas.
**Sundarbans and Southern Delta**	Bengal Basin	Woodman, N. D., Burgess, W. G., Ahmed, K. M., Zahid, A. (2019). A partially coupled hydro-mechanical analysis of the Bengal Aquifer System under hydrological loading. Hydrology and Earth System Sciences, 23, 2461-2479.	Ravenscroft, P., McArthur, J. M., Rahman, M. S. (2018). Identifying multiple deep aquifers in the Bengal Basin: Implications for resource management. Hydrological Processes, 32, 3615-3632.	Michael, H. A., Voss, C. I. (2009). Controls on groundwater flow in the Bengal Basin of India and Bangladesh: regional modeling analysis. Hydrogeology Journal, 17, 1561-1577.	Approximated from Fig. 1 by Woodman et al. (2019), Fig. 1 by Ravenscroft et al. (2018), and Fig. 1 by Michael and Voss (2009)
**Surprise Valley**	-	Cantwell, C.A., Fawler, A.P.G. (2014). Fluid Geochemistry of the Surprise Valley Geothermal System. Proceedings of the Thirty-Ninth Workshop on Geothermal Reservoir EngineeringStanford University, Stanford, California, February 24-26, 2014SGP-TR-202. Accessed March 27, 2021 from https://pangea.stanford.edu/ERE/pdf/IGAstandard/SGW/2014/Cantwell.pdf	-	-	Approximated from Fig. 1 of Cantwell and Fowler (2014).

**Aquifer system**	**Broader aquifer system**	**Reference A**	**Reference B**	**Reference C**	**Steps to delineate**
**Swan Lake and Upper Klamath Lake Perimeter**	Upper Klamath Basin	Gannett, M. W., Lite, K. E., La Marche, J. L., Fisher, B. J., Polette, D. J. (2007). Ground-water hydrology of the upper Klamath Basin, Oregon and California. U. S. Geological Survey Scientific Investigations Report 2007–5050. 98 pp. Accessed March 20, 2021 from https://pubs.usgs.gov/sir/2007/5050/pdf/sir20075050.pdf	-	-	Approximated from Fig. 18 of Gannett et al. (2007)
**Sydney Basin**	-	Australian Government (2018). Sydney Basin bioregion assessment. Webpage accessed October 8, 2021 from https://www.bioregionalassessments.gov.au/assessments/sydney-basin-bioregion	Danis, C. (2014). Use of groundwater temperature data in geothermal exploration: the example of Sydney Basin, Australia. Hydrogeology Journal, 22, 87-106.	Mather, B., Müller, R. D., O'Neill, C., Beall, A., Vervoort, R. W., Moresi, L. (2022). Constraining the response of continental-scale groundwater flow to climate change. Scientific Reports, 12, 1-16.	Approximated from Fig. 3 by Danis (2014), Fig. 1 by Mather et al. (2022), physiography and well data, and maps in section 1.1.4.3 of bioregion assessment: https://www.bioregionalassessments.gov.au/assessments/11-context-statement-sydney-basin-bioregion/1143-groundwater-flow
**Sydney Maritimes Basin**	Maritimes Basin	Stapinsky, M. Michaud, Y., Morin, R.H., Butler, K.E., Deblonde, C., Chi, G, Thériault, T., Castonguay, S., Boisvert, V., Julien, H.P., Conohan, D., Hulsman, B., Carrier, C. Marion, J., Boisvert, É. (2002). Groundwater resources assessment in the Carboniferous Maritimes Basin: preliminary results of the hydrogeological characterization, New Brunswick, Nova Scotia, and Prince Edward Island. Geological Survey of	Rivard, C., Michaud, Y., Lefebvre, R., Deblonde, C., Rivera, A. (2008). Characterization of a regional aquifer system in the Maritimes Basin, Eastern Canada. Water Resources Management, 22, 1649-1675.	Bianco, E. (2013). Seismic Interpretation of the Windsor-Kennetcook Basin. Geological Survey of Canada Open File 7452, 31 pp. Accessed May 5, 2021 from https://ftp.maps.canada.ca/pub/nrcan_rncan/publications/STPublications_PublicationsST/292/292763/of_7452.pdf	Approximated from Fig. 2 of Rivard et al. (2008) and Figs. 1 and 3 of Stapinsky et al. (2002). Area bounds approximated from Fig. 1 of Bianco (2013).

Aquifer system	Broader aquifer system	Reference A	Reference B	Reference C	Steps to delineate
		Canada Current Research Report 2002-D8, 14 pp. Accessed May 5, 2021 from http://www.gov.pe.ca/photos/original/cle_WA10.pdf			
**Sylhet Basin**	Bengal Basin	Burgess, W. G., Hoque, M. A., Michael, H. A., Voss, C. I., Breit, G. N., Ahmed, K. M. (2010). Vulnerability of deep groundwater in the Bengal Aquifer System to contamination by arsenic. Nature Geoscience, 3, 83-87.	Shamsudduha, M. (2007). Spatial Variability and Prediction Modeling of Groundwater Arsenic Distributions in the Shallowest Alluvial Aquifers in Bangladesh. Journal of Spatial Hydrology, 7, 33-46.	Ravenscroft, P., McArthur, J. M., Rahman, M. S. (2018). Identifying multiple deep aquifers in the Bengal Basin: Implications for resource management. Hydrological Processes, 32, 3615-3632.	Approximated from Fig. 1 by Burgess et al. (2010), Fig. 1 by Fig. 1 by Shamsudduha (2007), and Fig. 1 by Ravenscroft et al. (2018)
**Tabas Basin**	-	Aryafar, A., Khosravi, V., Hooshfar, F. (2019b). GIS-based comparative characterization of groundwater quality of Tabas basin using multivariate statistical techniques and computational intelligence. International Journal of Environmental Science and Technology, 16(10), 6277-6290.	-	-	Approximated from Fig. 1 by Aryafar et al. (2019b)
**Tabriz Plain**	-	Javadzadeh, H., Ataie-Ashtiani, B., Hosseini, S. M., Simmons, C. T. (2020). Interaction of lake-groundwater levels using cross-correlation analysis: A case study of Lake Urmia Basin, Iran. Science of The Total Environment, 729, 138822.	Vaezihir, A., Tabarmayeh, M. (2015). Total vulnerability estimation for the Tabriz aquifer (Iran) by combining a new model with DRASTIC. Environmental Earth Sciences, 74, 2949-2965.	Barzegar, R., Moghaddam, A. A., Tziritis, E. (2017). Hydrogeochemical features of groundwater resources in Tabriz plain, northwest of Iran. Applied Water Science, 7, 3997-4011.	Approximated from Fig. 1 by Javadzadeh et al. (2020), Fig. 1 by Barzegar et al. (2017), and Fig. 1 by Vaezihir and Tabarmayeh (2015)
**Tafresh Basin**	-	Parizi, E., Hosseini, S. M., Ataie-Ashtiani, B., Simmons, C. T. (2020). Normalized difference	-	-	Approximated from tabulated coordinates in the Supplementary Information within Parizi et al.

Aquifer system	Broader aquifer system	Reference A	Reference B	Reference C	Steps to delineate
		vegetation index as the dominant predicting factor of groundwater recharge in phreatic aquifers: case studies across Iran. Scientific Reports, 10, 17473.			(2020) and physiographic and drainage patterns
**Tahoe Valley South Basin**	-	Carroll, R.W.H., Pohll, G., Rajagopal, S. (2016). South Lake Tahoe groundwater model. Desert Research Institute Report, 27 pp. Accessed May 8, 2023 via https://www.stpud.us/Phase%20I%20Report_revised_Feb_25_2016.pdf	-	-	Approximated from Fig. 1 by Carroll et al. (2016)
**Taichung Coastal Alluvial Aquifer**	Taichung Basin	Hsu, S. K. (1998). Plan for a groundwater monitoring network in Taiwan. Hydrogeology Journal, 6, 405-415.	-	-	Approximated from Fig. 1 by Fig. 2 by Hsu et al. (1998); subarea boundary based on topography and drainage
**Taipei Basin**	-	Chen, C. T., Hu, J. C., Lu, C. Y., Lee, J. C., Chan, Y. C. (2007). Thirty-year land elevation change from subsidence to uplift following the termination of groundwater pumping and its geological implications in the Metropolitan Taipei Basin, Northern Taiwan. Engineering Geology, 95, 30-47.	Liu, C. W., Chou, Y. L., Lin, S. T., Lin, G. J., Jang, C. S. (2010). Management of high groundwater level aquifer in the Taipei Basin. Water resources management, 24, 3513-3525.	Hosono, T., Wang, C.H., Umezawa, Y., Nakano, T., Onodera, S.I., Nagata, T., Yoshimizu, C., Tayasu, I., Taniguchi, M. (2011). Multiple isotope (H, O, N, S and Sr) approach elucidates complex pollution causes in the shallow groundwaters of the Taipei urban area. Journal of Hydrology, 397, 23-36.	Approximated from Fig. 1 by Chen et al. (2007), Fig. 2 by Liu et al. (2010), and Fig. 2b by Hosono et al. (2011)
**Taiyuan Basin**	-	Zhang, Q., Rodríguez-Lado, L., Johnson, C. A., Xue, H., Shi, J., Zheng, Q., Sun, G. (2012). Predicting the risk of arsenic contaminated groundwater in Shanxi Province, Northern China.	Guo, Q., Wang, Y., Ma, T., Ma, R. (2007). Geochemical processes controlling the elevated fluoride concentrations in groundwaters of the Taiyuan Basin, Northern China. Journal of	Guo, C., Shi, J., Zhang, Z., Zhang, F. (2019). Using tritium and radiocarbon to determine groundwater age and delineate the flow regime in the Taiyuan Basin, China. Arabian	Approximated from Fig. 1 by Zhang et al. (2012), Fig. 1 by Guo et al. (2007), and Fig. 1 by Guo et al. (2019)

Aquifer system	Broader aquifer system	Reference A	Reference B	Reference C	Steps to delineate
		Environmental Pollution, 165, 118-123.	Geochemical Exploration, 93, 1-12.	Journal of Geosciences, 12, 1-14.	
**Tajuna-Montes Universales Aquifer**	-	Casado, M. (2010). The Tagus basin: Groundwater and Transboundary Aquifers. Presentation at the workshop on transboundary water resources management in western and central Europe. Budapest, 8-10 february 2011. Accessed April 26, 2023 via https://www.researchgate.net/publication/341251820_The_Tagus_basin_Groundwater_and_Transboundary_Aquifers	-	-	Approximated from slide 21 by Casado (2010)
**Takht Deraz Basin**	-	Parizi, E., Hosseini, S. M., Ataie-Ashtiani, B., Simmons, C. T. (2020). Normalized difference vegetation index as the dominant predicting factor of groundwater recharge in phreatic aquifers: case studies across Iran. Scientific Reports, 10, 17473.	-	-	Approximated from tabulated coordinates in the Supplementary Information within Parizi et al. (2020) and physiographic and drainage patterns
**Taoyuan-Chungli Tableland**	-	Hsu, S. K. (1998). Plan for a groundwater monitoring network in Taiwan. Hydrogeology Journal, 6, 405-415.	Peng, T. R., Huang, C. C., Wang, C. H., Liu, T. K., Lu, W. C., Chen, K. Y. (2012). Using oxygen, hydrogen, and tritium isotopes to assess pond water's contribution to groundwater and local precipitation in the pediment tableland areas of northwestern Taiwan. Journal of Hydrology, 450, 105-116.	-	Approximated from Fig. 2 by Hsu (1998), Fig. 1 by Peng et al. (2012),

Aquifer system	Broader aquifer system	Reference A	Reference B	Reference C	Steps to delineate
**Tashkoyeh Basin**	-	Hosseini, S. M., Parizi, E., Ataie-Ashtiani, B., Simmons, C. T. (2019). Assessment of sustainable groundwater resources management using integrated environmental index: Case studies across Iran. Science of the Total Environment, 676, 792-810.	-	-	Approximated from Fig. 6 by Hosseini et al. (2019)
**Tavabeh Arsanjan Basin**	-	Hosseini, S. M., Parizi, E., Ataie-Ashtiani, B., Simmons, C. T. (2019). Assessment of sustainable groundwater resources management using integrated environmental index: Case studies across Iran. Science of the Total Environment, 676, 792-810.	-	-	Approximated from Fig. 6 by Hosseini et al. (2019)
**Taznakht Plain**	-	Jeddi, T. A., Hssaisoune, M., Saad, A., El Morjani, Z. E. A., Mazziz, M., Beraaouz, M., Bouchaou, L. (2021). Water resources status to global changes in the Taznakht plain, Draa basin, Morocco. Frontiers in Science and Engineering, 11, 43-58. Accessed June 19, 2023 via https://doi.org/10.34874/IMIST.PRSM/fsejournal-v11i2.29008	-	-	Approximated from Fig. 1 by Jeddi et al. (2021)
**Tecolutla Aquifer**	-	CONAGUA (2021a). Acuíferos (nacional). Webpage accessed March 8, 2022 from http://sina.conagua.gob.mx	-	-	Approximated from aquifer boundaries defined by CONAGUA (2021a) web portal, physiography and drainage patterns, and well completion data.

Aquifer system	Broader aquifer system	Reference A	Reference B	Reference C	Steps to delineate
		/sina/tema.php?tema=acuiferos&ver=mapa&o=1&n=nacional			
**Tedori Alluvial Fan**	-	Yoshioka, Y., Nakamura, K., Takimoto, H., Sakurai, S., Nakagiri, T., Horino, H., & Tsuchihara, T. (2020). Multiple-indicator study of the response of groundwater recharge sources to highly turbid river water after a landslide in the Tedori River alluvial fan, Japan. Hydrological Processes, 34, 3539-3554.	-	-	Approximated from Fig. 1 by Yoshioka et al. (2020)
**Tehran-Karaj Plain**	Tehran Plain	Motagh, M., Walter, T. R., Sharifi, M. A., Fielding, E., Schenk, A., Anderssohn, J., Zschau, J. (2008). Land subsidence in Iran caused by widespread water reservoir overexploitation. Geophysical Research Letters, 35, L16403.	Panahi, M. R., Mousavi, S. M., Rahimzadegan, M. (2017). Delineation of groundwater potential zones using remote sensing, GIS, and AHP technique in Tehran–Karaj plain, Iran. Environmental Earth Sciences, 76, 792.	-	Approximated from Fig. 1 by Panahi et al. (2017). Broader Tehran Plain approximated from Fig. 1a by Motagh et al. (2008)
**Tehuantepec Aquifer**	-	CONAGUA (2021). Aguas subterráneas/ Acuíferos. Webpage accessed May 6, 2021 from https://sigagis.conagua.gob.mx/aprovechamientos/	-	-	Approximated from map portal by CONAGUA (2021).
**Tejo Alluvium**	-	Moura, A., Velho, J.L. (2012). Aguas Subterraneas. Chapter 57 in Recursos Geologicos de Portugal, pp. 523-536. Accessed May 1, 2023 via https://dspace.uevora.pt/rdpc/bitstream/10174/7918/1/2011_Aveiro.pdf	-	-	Approximated from Fig. 57.9 by Moura and Velho (2012)

Aquifer system	Broader aquifer system	Reference A	Reference B	Reference C	Steps to delineate
**Temata Oasis**	Middle Draa Basin	Szczucińska, A., Dłużewski, M., Kozłowski, R., Niedzielski, P. (2019). Hydrochemical diversity of a large alluvial aquifer in an arid zone (Draa river, S Morocco). Ecological Chemistry and Engineering S, 26, 81-100.	-	-	Approximated from Fig. 1 by Szczucińska et al. (2019)
**Temecula Valley**	-	California Department of Water Resources (2015e). California's groundwater update 2013 - Chapter 6: South Coast Hydrologic Region. California Department of Water Resources Report, 84 pp. Accessed April 27, 2022 via https://cawaterlibrary.net/wp-content/uploads/2017/05/GWU2013_Ch6_SouthCoast_Final.pdf	-	-	Approximated from Fig. 6-2 by California Department of Water Resources (2015e)
**Temescal Basin**	Upper Santa Ana Basin**	California Department of Water Resources (2015e). California's groundwater update 2013 - Chapter 6: South Coast Hydrologic Region. California Department of Water Resources Report, 84 pp. Accessed April 27, 2022 via https://cawaterlibrary.net/wp-content/uploads/2017/05/GWU2013_Ch6_SouthCoast_Final.pdf	-	-	Approximated from Fig. 6-2 by California Department of Water Resources (2015e)
**Tepalcingo-Axochiapan Aquifer**	-	Robles, E., Ramirez, E., de Guadalupe Sáinz, M., Duran, A., González, M. E. (2013). Bacteriological and	Morales-Casique, E. (2012). Mixing of groundwaters with uncertain end-members: case study in the	-	Approximated from description of study area and Fig. 1 by Robles et al. (2013) and Fig. 4 by Morales-Casique (2012).

Aquifer system	Broader aquifer system	Reference A	Reference B	Reference C	Steps to delineate
		Physicochemical Study on the Water of an Aquifer in Mexico. Universal Journal of Environmental Research & Technology, 3, 158-172.	Tepalcingo-Axochiapan aquifer, Mexico. Hydrogeology Journal, 20, 605-613.		
**Terwilliger Valley**	-	California Department of Water Resources (2015e). California's groundwater update 2013 - Chapter 6: South Coast Hydrologic Region. California Department of Water Resources Report, 84 pp. Accessed April 27, 2022 via https://cawaterlibrary.net/wp-content/uploads/2017/05/GWU2013_Ch6_SouthCoast_Final.pdf	-	-	Approximated from Fig. 6-2 by California Department of Water Resources (2015e)
**Teton Valley**	-	Graham, W.G., Campbell, L.J. (1981). Groundwater resources of Idaho. Idaho Department of Water Resources Report, 61 pp. Accessed March 23, 2021 from https://idwr.idaho.gov/files/publications/198108-MISC-GW-Resources-ID.pdf	-	-	Approximated from Plate 1 of Graham and Campbell (1981).
**Thal Doab**	Indus Basin	Amin, M., Khan, M. R., Jamil, A. (2018). Quantification of Groundwater Storage Variations and Stressed Areas Using Multi-temporal GRACE Data: A Case Study of Upper Indus Plains, Pakistan. In Conference of the Arabian Journal of Geosciences (pp. 299-304). Springer, Cham.	Hussain, Y., Ullah, S. F., Hussain, M. B., Aslam, A. Q., Akhter, G., Martinez-Carvajal, H., Cárdenas-Soto, M. (2017). Modelling the vulnerability of groundwater to contamination in an unconfined alluvial aquifer in Pakistan. Environmental Earth Sciences, 76, 84.	Greenman, D. W., Bennett, G. D., Swarzenski, W. V. (1967). Ground-water hydrology of the Punjab, West Pakistan, with emphasis on problems caused by canal irrigation. US Geological Survey Water-Supply Paper 1608-H, 81 pp. Accessed July 11, 2021 from	Approximated from Fig. 1 by Amin et al. (2018), Plate 2 by Greenman et al. (1967), and Fig. 1 by Hussain et al. (2017)

Aquifer system	Broader aquifer system	Reference A	Reference B	Reference C	Steps to delineate
				https://pubs.usgs.gov/wsp/1608h/report.pdf	
**Thames Valley and London Basin**	Chalk Aquifer System	MacDonald, A.M., Allen, D.J. (2001). Aquifer properties of the Chalk of England. Quarterly Journal of Engineering Geology and Hydrogeology, 34, 371-384.	-	-	Approximated from Fig. 8 by MacDonald and Allen (2001)
**Thau Lagoon and Coastal Karstic System**	-	Tournoud, M. G., Payraudeau, S., Cernesson, F., Salles, C. (2006). Origins and quantification of nitrogen inputs into a coastal lagoon: Application to the Thau lagoon (France). Ecological Modelling, 193, 19-33.	Pinault, J. L., Doerfliger, N., Ladouche, B., & Bakalowicz, M. (2004). Characterizing a coastal karst aquifer using an inverse modeling approach: The saline springs of Thau, southern France. Water Resources Research, 40, W08501.	-	Approximated from Fig. 2 by Tournoud et al. (2006) and Fig. 4 by Pinault et al. (2004).
**Theni Fractured Charnockite and Gneiss Aquifers**	-	Magesh, N. S., Chandrasekar, N., Soundranayagam, J. P. (2012). Delineation of groundwater potential zones in Theni district, Tamil Nadu, using remote sensing, GIS and MIF techniques. Geoscience Frontiers, 3, 189-196.	Saha, D., Shekhar, S., Ali, S., Vittala, S.S., Raju, N.J. (2016). Recent hydrogeological research in India. Proceedings of the Indian National Science Academy, 82, 787-803.	-	Approximated from Fig. 4 by Magesh et al. (2012) and Fig. 1 by Saha et al. (2016)
**Thousand Oaks and Russell Valley**	-	California Department of Water Resources (2015e). California's groundwater update 2013 - Chapter 6: South Coast Hydrologic Region. California Department of Water Resources Report, 84 pp. Accessed April 27, 2022 via https://cawaterlibrary.net/wp-content/uploads/2017/05/G	-	-	Approximated from Fig. 6-2 by California Department of Water Resources (2015e)

Aquifer system	Broader aquifer system	Reference A	Reference B	Reference C	Steps to delineate
		WU2013_Ch6_SouthCoast_Final.pdf			
**Three Lakes Valley**	-	Burbey, T. J. (1997). Hydrogeology and potential for ground-water development, carbonate-rock aquifers in southern Nevada and southeastern California. US Geological Survey Water-Resources Investigations Report 95-4168, 70 pp. Accessed March 10, 2021 from https://pubs.usgs.gov/wri/1995/4168/report.pdf	-	-	Approximated from Fig. 12 of Burbey (1997)
**Ti Tree Basin**	-	Harrington, G.A., Herczeg, A.L., Cook, P.G. (1999). Groundwater Sustainability and Water Quality in the Ti-Tree Basin, Central Australia. CSIRO Report, 14 pp. Accessed October 6, 2021 from http://www.clw.csiro.au/publications/technical99/tr53-99.pdf	Harrington, G. A., Cook, P. G., Herczeg, A. L. (2002). Spatial and temporal variability of ground water recharge in central Australia: a tracer approach. Groundwater, 40, 518-527.	Calf, G. E., McDonald, P. S., Jacobson, G. (1991). Recharge mechanism and groundwater age in the Ti-Tree Basin, Northern Territory. Australian Journal of Earth Sciences, 38, 299-306.	Approximated from Fig. 1 by Harrington et al. (1999), Fig. 1 by Harrington et al. (2002) and Fig. 1 by Calf et al. (1991)
**Tietar Valley Aquifer**	-	Bejarano, M.D., Gonzalez del Tanago, M., de Jalón, D.G., Marchamalo, M., Sordo-Ward, Á., Solana-Gutiérrez, J. (2012). Responses of riparian guilds to flow alterations in a Mediterranean stream. Journal of Vegetation Science, 23, 443-458.	-	-	Approximated from Fig. 1 by Bejarano et al. (2012)
**Tifton Upland**	Floridan Aquifer System	Davis, H. (1996). Hydrogeologic Investigation and Simulation of Ground-Water Flow in the Upper Floridan Aquifer of North-	Clark, W. Z., Zisa, A. C. (1976) Physiographic map of Georgia: Georgia Department of Natural Resources, 1 sheet.	Brooks, H. K. (1981). Physiographic divisions of Florida. Report for the Florida Cooperative Extension Service Institute	Approximated from Fig. 2 of Davis (1996) and the physiographic provinces for Georgia and Florida by Clark and Zisa (1976) and Brooks (1981)

Aquifer system	Broader aquifer system	Reference A	Reference B	Reference C	Steps to delineate
		Central Florida and Southwestern Georgia and Delineation of Contributing Areas for Selected City of Tallahassee, Florida, Water-Supply Wells. U.S. Geological Survey Water-Resources investigations Report 95-4296, 61 pp. Accessed March 31, 2021 from https://fl.water.usgs.gov/PDF_files/wri95_4296_davis.pdf	Accessed March 31, 2021 from https://epd.georgia.gov/document/publication/sm-4-physiographic-map-georgia-12000000-1988/download	of Food and Agricultural Sciences, University of Florida, 12 pp.	
**Tijuana-San Diego Basin**	-	International Hydrological Programme, Division of Water Sciences (2009). Atlas of Transboundary Aquifers. UNESCO Report, 322 pp. Accessed March 21, 2021 from https://isarm.org/sites/default/files/resources/files/2%20Atlas%20of%20TBA.pdf	Anders, R., Mendez, G.O., Futa, K., Danskin, W.R. (2014). A geochemical approach to determine sources and movement of saline groundwater in a coastal aquifer. Groundwater, 52, 756-768.	-	Approximated from map on page 99 of International Hydrological Programme, Division of Water Sciences (2009) and Fig. 1 by Anders et al. (2014)
**Tillman Terrace**	-	Osborn, N.I. (2002). Update of the Hydrologic Survey of the Tillman Terrace Groundwater Basin, Southwestern Oklahoma. Oklahoma Water Resources Board Technical Report GW2002-1, 21 pp., Accessed April 5, 2021 from https://www.owrb.ok.gov/studies/reports/reports_pdf/tillman_update.pdf	-	-	Approximated from well completion data and Fig. 1 of Osborn (2002).
**Tinguiririca Inferior Aquifer**	Tinguiririca Aquifer	Direccion General de Aguas (2017). Inventario Nacional de acuiferos. Ministerio De Obras Públicas (Gobierno De Chile) report number	-	-	Approximated from map on page 42 by Direccion General de Aguas (2017).

Aquifer system	Broader aquifer system	Reference A	Reference B	Reference C	Steps to delineate
		403, 60 pp. Accessed May 17, 2021 from https://snia.mop.gob.cl/sad/SUB5748.pdf			
**Tinguiririca Superior Aquifer**	Tinguiririca Aquifer	Direccion General de Aguas (2017). Inventario Nacional de acuiferos. Ministerio De Obras Públicas (Gobierno De Chile) report number 403, 60 pp. Accessed May 17, 2021 from https://snia.mop.gob.cl/sad/SUB5748.pdf	-	-	Approximated from map on page 42 by Direccion General de Aguas (2017).
**Tinzouline Oasis**	Middle Draa Basin	Szczucińska, A., Dłużewski, M., Kozłowski, R., Niedzielski, P. (2019). Hydrochemical diversity of a large alluvial aquifer in an arid zone (Draa river, S Morocco). Ecological Chemistry and Engineering S, 26, 81-100.	-	-	Approximated from Fig. 1 by Szczucińska et al. (2019)
**Tipton Till Plain**	Central Lowland Till Plain	Gray, H.H. (2001). Map of Indiana showing physiographic divisions. Indiana Geological Survey Miscellaneous Map 69, 15 pp. Accessed April 12, 2021 from https://igws.indiana.edu/ReferenceDocs/Maps/PhysiographicRegions.pdf	Fenneman, N.M., Johnson, D.W. (1946). Physiographic divisions of the conterminous United States. U.S. Geological Survey map, 1:7,000,000 Scale.	-	Broader Till Plain physiographic region approximated from Fenneman and Johnson (1946). Tipton Till Plain approximated from the map by Gray (2001).
**Tiruvadanai Aquifer System**	Coromandel Coastal Plain	Central Ground Water Board (2012b). Aquifer Systems of Tamilnadu and Puducherry. Central Ground Water Board Report, 78 pp. Accessed June 23, 2021 from http://cgwb.gov.in/AQM/TamilNadu.pdf	Kumar, U. S., Sharma, S., Navada, S.V., Deodhar, A. S. (2009). Environmental isotopes investigation on recharge processes and hydrodynamics of the coastal sedimentary aquifers of Tiruvadanai, Tamilnadu State, India.	-	Approximated from Plate IV by Central Ground Water Board (2012b) and Fig. 1 by Kumar et al. (2009)

Aquifer system	Broader aquifer system	Reference A	Reference B	Reference C	Steps to delineate
			Journal of Hydrology, 364, 23-39.		
**Tlacolula Valley**	Central Valleys of Oaxaca	Ojeda Olivares, E. A., Sandoval Torres, S., Belmonte Jiménez, S. I., Campos Enríquez, J. O., Zignol, F., Reygadas, Y., & Tiefenbacher, J. P. (2019). Climate change, land use/land cover change, and population growth as drivers of groundwater depletion in the central valleys, Oaxaca, Mexico. Remote Sensing, 11, 1290.	-	-	Approximated from Fig. 1 by Ojeda Olivares et al. (2019).
**Tlaltenango-Tepechitlan Aquifer**	-	CONAGUA (2021a). Acuíferos (nacional). Webpage accessed March 8, 2022 from http://sina.conagua.gob.mx/sina/tema.php?tema=acuiferos&ver=mapa&o=1&n=nacional	-	-	Approximated from aquifer boundaries defined by CONAGUA (2021a) web portal, physiography and drainage patterns, and well completion data.
**Tokio-Woodbine Aquifer**	-	Rodgers, K. D. (2017). Water-level trends and potentiometric surfaces in the Nacatoch Aquifer in northeastern and southwestern Arkansas and in the Tokio Aquifer in southwestern Arkansas, 2014–15. US Geological Survey Scientific Investigations Report 2017-5090, 38 pp. Accessed November 11, 2021 from https://pubs.usgs.gov/sir/2017/5090/sir20175090.pdf	-	-	Approximated from Fig. 13 by Rodgers (2017)
**Toole Valley**	-	Thomas, H. E. (1946). Ground water in Tooele Valley, Tooele County, Utah	-	-	Approximated from Fig. 1 of Gardner and Kirby (2011).

Aquifer system	Broader aquifer system	Reference A	Reference B	Reference C	Steps to delineate
		(No. 4, pp. 91-238). Utah Department of Natural Resources, Division of Water Rights. 148 pp. Accessed March 7, 2021 from https://waterrights.utah.gov/docSys/v920/w920/w9200083.pdf			
**Toucian-Fongshan Alluvial Plain**	Hsinchu-Miaoli Coastal Aquifer System	Hsu, S. K. (1998). Plan for a groundwater monitoring network in Taiwan. Hydrogeology Journal, 6, 405-415.	Han, Y. L., Kuo, M. T., Fan, K. C., Chiang, C. J., Lee, Y. P. (2006). Radon distribution in groundwater of Taiwan. Hydrogeology Journal, 14, 173-179.	Lu, H. Y., Peng, T. R., Liu, T. K., Wang, C. H., Huang, C. C. (2006). Study of stable isotopes for highly deformed aquifers in the Hsinchu-Miaoli area, Taiwan. Environmental Geology, 50, 885-898.	Approximated from Fig. 2 by Hsu (1998), Fig. 1 by Han et al. (2006), and descriptions in the final paragraph of the subsection entitled "Study area" and Fig. 1 by Lu et al. (2006)
**Tournaisis Region Carbonate Aquifers**	-	BRGM (2013). l’aquifère des calcaires carbonifères. Presentation for an Interreg IVB NWE project for a better quality of surface and groundwater bodies in the Scheldt International River Basin District (IRBD). 10 pp. Accessed April 24, 2023 via https://www.isc-cie.org/wp-content/uploads/PLEN_1701_pres-Parmentier_BRGM_Carbonifere.pdf	-	-	Approximated from slide 3 within BRGM (2013)
**Toyooka Basin**	-	Nakai, I., Mori, N., Hirata, S., Masuma, H., Takino, M., Isomura, K., Teranishi, K. (1992). Quality of the groundwater in Toyooka Basin. Journal of Groundwater Hydrology, 34, 1-12. Accessed April 13, 2023 via	Tanigawa, K., Hyodo, M., Sato, H. (2013). Holocene relative sea-level change and rate of sea-level rise from coastal deposits in the Toyooka Basin, western Japan. The Holocene, 23, 1039-1051.	-	Approximated from Fig. 1 by Nakai (1992), and Fig. 1 by Tanigama et al. (2013)

Aquifer system	Broader aquifer system	Reference A	Reference B	Reference C	Steps to delineate
		https://www.jstage.jst.go.jp/article/jagh1987/34/1/34_1/_article/-char/ja/			
**Tracy Basin**	San Joaquin Basin of the Central Valley **	California Department of Water Resources (2015h). California's groundwater update 2013 - Chapter 8: San Joaquin River Hydrologic Region. California Department of Water Resources Report, 122 pp. Accessed April 28, 2022 via https://water.ca.gov/-/media/DWR-Website/Web-Pages/Programs/Groundwater-Management/Data-and-Tools/Files/Statewide-Reports/California-Groundwater-Update-2013/California-Groundwater-Update-2013---Chapter-8---San-Joaquin-River.pdf	-	-	Approximated from Fig. 8-2 by California Department of Water Resources (2015h)
**Trans Gangetic Plain**	Indo-Gangetic Plain	Bonsor, H. C., MacDonald, A. M., Ahmed, K. M., Burgess, W. G., Basharat, M., Calow, R. C., Dixit, A., Foster, S.S.D., Gopal, K., Lapworth, D.J., Moench, M., Mukherjee, A., Rao, M.S., Shamsudduha, M., Smith, L., Taylor, R.G., Tucker, J., van Steenbergen, F., Yadav, S.K., Zahid, A. (2017). Hydrogeological typologies of the Indo-Gangetic basin alluvial aquifer, South Asia. Hydrogeology Journal, 25, 1377-1406.	Joshi, S.K., Gupta, S., Sinha, R., Densmore, A.L., Rai, S.P., Shekhar, S., Mason, P.J., van Dijk, W.M. (2021). Strongly heterogeneous patterns of groundwater depletion in northwestern India. Journal of Hydrology, 598, 126492.	Mukherjee, A., Saha, D., Harvey, C. F., Taylor, R. G., Ahmed, K. M., Bhanja, S. N. (2015). Groundwater systems of the Indian sub-continent. Journal of Hydrology: Regional Studies, 4, 1-14.	Approximated from Fig. 3 by Mukherjee et al. (2015), Fig. 3 by Bonsor et al. (2020), and including much of the Sutlej- Yamuna plain delineated in Fig. 1 by Joshi et al. (2021)

Aquifer system	Broader aquifer system	Reference A	Reference B	Reference C	Steps to delineate
**Transboundary Judith River Aquifer**	Bearpaw-Judith River Aquifer System	Davies, H., Hanley, P.T. (2010). State of the Watershed Report - 2010. Water Security Agency, Saskatchewan. Appendix A, 82 pp. Accessed April 26, 2021 from https://www.wsask.ca/Global/About%20WSA/Publications/State%20of%20the%20Watershed/d_AppendixA-ConditionIndicators.PDF	Crowley, J.J., LaFave, J.I., Bergantino, R.N., Carstarphen, C.A., Patton, T.W. (2017). Principle Aquifers of Montana. Montana Bureau of Mines and Geology Hydrogeologic Map 11. Accessed April 26, 2021 from https://www.leg.mt.gov/content/Committees/Interim/2017-2018/Water-Policy/Meetings/Jan-2018/Exhibits/Jan9/Exhibit5.pdf	-	Approximated from Fig. 28 of Davies and Hanley (2010) and map by Crowley et al. (2017).
**Transboundary Odanah Shale**	-	Rutulis, M. (1986). Aquifer maps of southern Manitoba. Manitoba Water Resources Branch map. Accessed May 5, 2021 from https://www.gov.mb.ca/water/pubs/maps/water/1986_rutulis_bedrock_aquifers.pdf	Randich, P.G., Kuzniar, R.L. (1984). Ground-water resources of Towner County, North Dakota. North Dakota State Water Commission Report, 31 pp. Accessed May 5, 2021 from http://swc.state.nd.us/info_edu/reports_and_publications/county_groundwater_studies/pdfs/Towner_Part_III.pdf	Hutchinson, R.D., Klausing, R.L. (1980). Ground-water resources of Ramsey County, North Dakota. North Dakota State Water Commission Report, 41 pp. Accessed May 5, 2021 from http://www.swc.state.nd.us/info_edu/reports_and_publications/county_groundwater_studies/pdfs/Ramsey_Part_III.pdf	Approximated from map by Rutulis (1986). Southern margin of area in North Dakota includes much of Towner County, as a local hydrogeological report idenitifies the Odanah as a source of water in the county (read pages 10-11 by Randich and Kuzniar, 1984). However, the Odanah Shale is not a major aquifer in Ramsey County, as suggested by a county hydrogeological report by Hutchinson and Klausing (1980); the southern margin of th area was cut off within Ramsey County.
**Trebon Basin**	-	Kadlecová, R., Olmer, M. (2011). Review of Groundwater Resources. Geologické výzkumy na Moravě a ve Slezsku. Proceedings of The Open Congress ČGS and SGS, September 22–25, 2011, Monínec 18, pp. 31-34. Accessed October 21, 2021 from	Silar, J., Silar, J. (1995). Using environmental isotopes for groundwater flow analysis in basinal structures. IAHS Publications-Series of Proceedings and Reports-Intern Assoc Hydrological Sciences, 232, 141-150.	-	Approximated from Fig. 2 by Kadlecová and Olmer (2011) and Fig. 1 by Silar and Silar (1995)

Aquifer system	Broader aquifer system	Reference A	Reference B	Reference C	Steps to delineate
		https://journals.muni.cz/gvms/article/view/1679			
**Trinity Aquifer System**	Edwards-Trinity Aquifer System	Bruun, B., Jackson, K., Lake, P., Walker, J. (2016). Texas Aquifers Study. Texas Water Development Board Report. 336 pp. Accessed April 1, 2021 from https://www.twdb.texas.gov/groundwater/docs/studies/TexasAquifersStudy_2016.pdf#page=89	Gordon, C.H. (1913). Geology and underground waters of the Wichita region, north-central Texas. U.S. Geological Survey Water Supply Paper 317, 90 pp. Accessed April 7, 2021 from https://pubs.usgs.gov/wsp/0317/report.pdf	Ryder, P. (1996). Ground Water Atlas of the United States Segment 4 Oklahoma, Texas. Hydrologic Investigations Atlas 730-E, 32 pp. Accessed November 29, 2021 from https://pubs.usgs.gov/ha/730e/report.pdf	Approximated from Fig. 6-37 by Bruun et al. (2016) and Fig. 114 by Ryder (1996). Delineated area includes the northeast portion of the Balcones Fault Zone following the major aquifer delineation of in map on page vii of Bruun et al. (2016). Local hydrogeology in northern area of aquifer are detailed in Plate 1 of Gordon (1913).
**Truckee Meadows and Pleasant Valley**	-	Lopes, T.J., Evetts. D.M. (2005). Ground-Water pumpage and artificial recharge estimates for calendar year 2000 and average annual natural recharge and interbasin flow by hydrographic area, Nevada. Accessed April 25, 2022 via https://pubs.usgs.gov/sir/2004/5239/sir2004-5239.pdf	Harrill, J.R., Prudic, D.E. (1998). Aquifer systems in the Great Basin region of Nevada, Utah, and adjacent states; summary report. US Geological Survey Professional Paper 1409-A, 75 pp. Accessed April 26, 2022 via https://pubs.usgs.gov/pp/1409a/report.pdf	-	Approximated from Fig. 1 by Lopes and Evetts (2005), and Fig. 2 by Harrill and Prudic (1998). Well completion records, physiography and drainage were used to delinate areas.
**Tualatin and Portland Basins**	Willamette Valley	Woodward, D.G., Gannett, M.W., Vaccaro, J.J. (1998). Hydrogeologic framework of the Willamette Lowland aquifer system, Oregon and Washington. US Geological Survey Professional Paper 1424-B, 92 pp. Accessed February 16, 2021 from https://pubs.usgs.gov/pp/1424b/report.pdf	Herrera, N.B., Burns, E.R., Conlon, T.D. (2014). Simulation of groundwater flow and the interaction of groundwater and surface water in the Willamette Basin and Central Willamette Subbasin, Oregon. US Geological Survey Scientific Investigations Report 2014–5136, 174 pp. Accessed February 16, 2021 from https://pubs.usgs.gov/sir/2	Jones, M.A. (1999). Geologic framework for the Puget Sound aquifer system, Washington and British Columbia. US Geological Survey Professional Report 1424-C, 44 pp. Accessed February 25, 2021 from https://pubs.usgs.gov/pp/1424c/report.pdf	Approximated from Fig. 2 of Woodward et al. (1998) and Fig. 1 by Herrera et al. (2014). Basin extent was broadened to include foothills of surrounding mountains, as a considerable number of wells exist in these areas. The Chehalem Mountains were used as an approximate divide between this Tualatin Basin and the Central Willamette Valley to the south. The northern extent of the Basin crosses the Columbia River and is based on the approximate northern limit delineated in Fig. 1

Aquifer system	Broader aquifer system	Reference A	Reference B	Reference C	Steps to delineate
			014/5136/pdf/sir20145136.pdf		of Jones (1999) (see page 12 or "C4" of report).
**Tulare Lake Basin**	Tulare Basin of the Central Valley **	California Department of Water Resources (2015c). California's groundwater update 2013 - Chapter 9: Tulare Lake Hydrologic Region. California Department of Water Resources Report, 80 pp. Accessed April 27, 2022 via https://data.cnra.ca.gov/dataset/california-water-plan-groundwater-update-2013/resource/8a4ae915-b786-42e1-9abe-99a8fcc23349	-	-	Approximated from Fig. 9-2 by California Department of Water Resources (2015c)
**Tularosa Basin**	Tularosa-Huelco	McLean, J. S. (1970). Saline ground-water resources of the Tularosa basin, New Mexico (No. 561). US Geological Survey. Accessed February 17, 2021 from https://pubs.usgs.gov/unnumbered/70139928/report.pdf	Huff, G. F. (2005). Simulation of ground-water flow in the basin-fill aquifer of the Tularosa Basin, south-central New Mexico, predevelopment through 2040 (Vol. 4, No. 4). US Geological Survey Scientific Investigations Report 2004-5197, 108 pp. Accessed February 17, 2021 from https://pubs.usgs.gov/sir/2004/5197/pdf/sir20045197.pdf	-	Approximated from Fig. 1 of McLean (1970) and Fig. 1 by Huff (2005)
**Tule Basin**	Tulare Basin of the Central Valley **	California Department of Water Resources (2015c). California's groundwater update 2013 - Chapter 9: Tulare Lake Hydrologic Region. California Department of Water Resources Report, 80 pp. Accessed April 27, 2022 via https://data.cnra.ca.gov/da	-	-	Approximated from Fig. 9-2 by California Department of Water Resources (2015c)

Aquifer system	Broader aquifer system	Reference A	Reference B	Reference C	Steps to delineate
		taset/california-water-plan-groundwater-update-2013/resource/8a4ae915-b786-42e1-9abe-99a8fcc23349			
**Tule Valley**	-	Lopes, T.J., Evetts. D.M. (2005). Ground-Water pumpage and artificial recharge estimates for calendar year 2000 and average annual natural recharge and interbasin flow by hydrographic area, Nevada. Accessed April 25, 2022 via https://pubs.usgs.gov/sir/2004/5239/sir2004-5239.pdf	Harrill, J.R., Prudic, D.E. (1998). Aquifer systems in the Great Basin region of Nevada, Utah, and adjacent states; summary report. US Geological Survey Professional Paper 1409-A, 75 pp. Accessed April 26, 2022 via https://pubs.usgs.gov/pp/1409a/report.pdf	-	Approximated from Fig. 1 by Lopes and Evetts (2005), and Fig. 2 by Harrill and Prudic (1998). Well completion records, physiography and drainage were used to delinate areas.
**Tuli Karoo Aquifer**	-	Majola, K., Xu, Y., Kanyerere, T. (2021). Assessment of climate change impacts on groundwater-dependent ecosystems in transboundary aquifer settings with reference to the Tuli-Karoo transboundary aquifer. Ecohydrology & Hydrobiology.	Gomo, M., Vermeulen, D. (2017). A transboundary aquifer of potential concern in Southern Africa. Water Policy, 19, 1160-1171.	-	Approximated from Fig. 1 by Majola et al. (2021) and Fig. 1 by Gomo and Vermeulen (2017)
**Turlock Basin**	San Joaquin Basin of the Central Valley **	California Department of Water Resources (2015h). California's groundwater update 2013 - Chapter 8: San Joaquin River Hydrologic Region. California Department of Water Resources Report, 122 pp. Accessed April 28, 2022 via https://water.ca.gov/-/media/DWR-Website/Web-	-	-	Approximated from Fig. 8-2 by California Department of Water Resources (2015h)

Aquifer system	Broader aquifer system	Reference A	Reference B	Reference C	Steps to delineate
		Pages/Programs/Groundwater-Management/Data-and-Tools/Files/Statewide-Reports/California-Groundwater-Update-2013/California-Groundwater-Update-2013---Chapter-8---San-Joaquin-River.pdf			
**Tuyserkan Plain**	-	ParsiMehr, M., Shayesteh, K., Godini, K. (2020). The modeling and prediction of the quality of the groundwater resources in Tuyserkan plain using the optimized artificial neural network. Journal of Advances in Environmental Health Research, 8, 107-118.	-	-	Approximated from Fig. 1 by ParsiMehr et al. (2020)
**Twin Falls**	Central Snake River Plain	Graham, W.G., Campbell, L.J. (1981). Groundwater resources of Idaho. Idaho Department of Water Resources Report, 61 pp. Accessed March 23, 2021 from https://idwr.idaho.gov/files/publications/198108-MISC-GW-Resources-ID.pdf	-	-	Approximated from Plate 1 of Graham and Campbell (1981).
**Tyner Valley Aquifer**	Bearpaw-Judith River Aquifer System	Sloan, M., Gillies, J. A., Norum, D. I. (1991). Using poor quality groundwater for irrigation in Saskatchewan, Canada. Canadian Water Resources Journal, 16, 45-64.	Maathuis, H., Simpson, M. (2007b). Groundwater Resources of the Prelate (72K) area, Saskatchewan. Saskatchewan Research Council Publication No. 11975-1E07, 121 pp. Accessed April 26, 2021 from https://www.wsask.ca/Global/Water%20Info/Ground%	Ferris, D., Lypka, M., Ferguson, G. (2017). Hydrogeology of the Judith River formation in southwestern Saskatchewan, Canada. Hydrogeology Journal, 25(7), 1985-1995.	Approximated following Fig. 1 of Sloan et al. (1991). Area included in broader Judith River Aquifer System because the Tyner Valley aquifer itself is hydraulically connected to the Judith River Formation (Maathuis and Simpson, 2007), even through the Judith River Formation itself does not subcrop in much of the area (see Fig. 3 of Ferris et al. (2017))

Aquifer system	Broader aquifer system	Reference A	Reference B	Reference C	Steps to delineate
			20Water/Mapping/Prelate%20Mapsheet%2072K/Reports/11975-1E07-Prelate_Final_public.pdf		
**Uitenhage Artesian Basin**	-	Maclear, L.G.A (2001). The hydrogeology of the Uitenhage Artesian Basin with reference to the Table Mountain Group Aquifer. Water SA, 27, 499-506.	Heaton, T.H.E., Talma, A. S., Vogel, J. C. (1986). Dissolved gas paleotemperatures and 18O variations derived from groundwater near Uitenhage, South Africa. Quaternary Research, 25, 79-88.	Vogel, J. C., Talma, A. S., Heaton, T. H. E., Kronfeld, J. (1999). Evaluating the rate of migration of an uranium deposition front within the Uitenhage Aquifer. Journal of Geochemical Exploration, 66, 269-276.	Approximated from Fig. 1 by Maclear (2001), Fig. 1 by Heaton et al. (1986), Fig. 2 by Vogel et al. (1999).
**Ukiah Valley**	-	California Department of Water Resources (2015j). California's groundwater update 2013 - Chapter 3: North Coast Hydrologic Region. California Department of Water Resources Report, 80 pp. Accessed April 27, 2022 via https://water.ca.gov/-/media/DWR-Website/Web-Pages/Programs/Groundwater-Management/Bulletin-118/Files/Statewide-Reports/GWU2013_Ch3_NorthCoast_Final.pdf	-	-	Approximated from Fig. 3-2 by California Department of Water Resources (2015j)
**Uley Basin**	Eucla Basin	Zulfic, D., Harrington, N. Evans, S. (2006). Uley Basin Groundwater Modelling Project, Volume 2: Groundwater Flow Model, DWLBC Report 2007/04, Department of Water, Land and Biodiversity Conservation, Adelaide. 128 pp. Accessed October 6, 2021 from https://www.waterconnect.	Berens, V., Alcoe, D., Watt, E. (2011). Non-prescribed groundwater resources assessment — Eyre Peninsula natural resources management region. Science, Monitoring and Information Division Department for Water, 110 pp. Accessed October 6, 2021 from https://www.waterconnect.	-	Approximated from Fig. 1 by Zulfic et al. (2006), with the broader Eucla Basin approximated frolloing Fig. 4 by Berens et al. (2011)

Aquifer system	Broader aquifer system	Reference A	Reference B	Reference C	Steps to delineate
		sa.gov.au/Content/Publications/DEW/ki_dwlbc_report_2007_04.pdf	sa.gov.au/Content/Publications/DEW/EP_NRM_Non-prescribed_GW_Assessment_2011.pdf		
**Umatilla Basin and Horse Heaven Hills**	Columbia Plateau Regional Aquifer System	Kahle, S.C., Morgan, D.S., Welch, W.B., Ely, S.R., Vaccaro, J.J., Orzol, L.L. (2011). Hydrogeologic Framework and Hydrologic Budget Components of the Columbia Plateau Regional Aquifer System, Washington, Oregon, and Idaho. US Geological Survey Scientific Investigations Report 2011–5124, 80 pp. Accessed February 16, 2021 from https://pubs.usgs.gov/sir/2011/5124/pdf/sir20115124.pdf	Herrera, N. B., Ely, K., Mehta, S., Stonewall, A. J., Risley, J. C., Hinkle, S. R., Conlon, T. D. (2017). Hydrogeologic framework and selected components of the groundwater budget for the upper Umatilla River Basin, Oregon (No. 2017-5020). US Geological Survey Scientific Investigations Report 2017-5020, 68 pp. Accessed February 18, 2021 from https://pubs.usgs.gov/sir/2017/5020/sir20175020.pdf	Davies-Smith, A., Bolke, E. L., Collins, C. A. (1988). Geohydrology and digital simulation of the ground-water flow system in the Umatilla Plateau and Horse Heaven Hills area, Oregon and Washington. Water-Resources Investigations Report 87-4268, 77 pp. Accessed February 23, 2021 from https://pubs.usgs.gov/wri/1987/4268/report.pdf	Approximate Umatilla Basin boundaries from Fig. 9 of Kahle et al. (2011); see also Fig. 3 of Herrera et al. (2017). Horse Heaven Hills extent (and co-study with Umatilla Basin) from Fig. 1 of Davies-Smith et al. (1983). Columbia River Aquifer System approximated from Figs. 1 and 10 of Kahle et al. (2011).
**Uncompahgre Basin**	-	Craig, T.W. (1971). Ground water of the Uncompahgre Valley Montrose County, Colorado. MSc Thesis, University of Missouri-Rolla, 119 pp. Accessed March 28, 2021 from https://scholarsmine.mst.edu/cgi/viewcontent.cgi?article=6120&context=masters_theses	-	-	Approximated from Fig. 5 of Craig et al. (1971)
**Upper Aconcagua Basin**	Aconcagua Basin	Direccion General de Aguas (2009). Diagnóstico y Clasificación de Sectores Acuíferos, Volumen No. 2. Gobierno de Chile Ministerio de Obras Publicas Report, 384 pp. Accessed May 16, 2021 from	Taucare, M., Viguier, B., Daniele, L., Heuser, G., Arancibia, G., Leonardi, V. (2020). Connectivity of fractures and groundwater flows analyses into the Western Andean Front by means of a topological	-	Approximated from Table 1.8 on page 40 by Direccion General de Aguas (2009). Middle and upper portions of the aquifer system delineated near Panquehue. See also Fig. 1 by Taucare et al. (2020).

Aquifer system	Broader aquifer system	Reference A	Reference B	Reference C	Steps to delineate
		https://snia.mop.gob.cl/sad/CQA5168v2.pdf	approach (Aconcagua Basin, Central Chile). Hydrogeology Journal, 28, 2429-2438.		
**Upper American and Auburn Basins**	Sierra Nevada Foothills **	Shelton, J.L., Fram, M.S., Munday, C.M., Belitz, K. (2010). Groundwater-quality data for the Sierra Nevada study unit, 2008. Results from the California GAMA program. U.S. Geological Survey Data Series 534, 106 pp. Accessed April 15, 2021 from https://pubs.usgs.gov/ds/534/ds_534.pdf	-	-	Approximated on the basis of the density of completed wells (from the California Department of Water Resources); general hydrogeology of region summarized by Shelton et al. (2010).
**Upper Arkansas River Basin**	-	Watts, K.R. (2005). Hydrogeology and quality of ground water in the upper Arkansas River Basin from Buena Vista to Salida, Colorado, 2000-2003. U.S. Geological Survey Scientific Investigations Report 2005-5179, 61 pp. Accessed March 27, 2021 from https://pubs.usgs.gov/sir/2005/5179/pdf/SIR2005-5179.pdf	-	-	Approximated from Fig. 1 by Watts (2005)
**Upper Bear Basin**	Sierra Nevada Foothills **	Shelton, J.L., Fram, M.S., Munday, C.M., Belitz, K. (2010). Groundwater-quality data for the Sierra Nevada study unit, 2008. Results from the California GAMA program. U.S. Geological Survey Data Series 534, 106 pp. Accessed April 15, 2021 from	-	-	Approximated on the basis of the density of completed wells (from the California Department of Water Resources); general hydrogeology of region summarized by Shelton et al. (2010).

Aquifer system	Broader aquifer system	Reference A	Reference B	Reference C	Steps to delineate
		https://pubs.usgs.gov/ds/534/ds_534.pdf			
**Upper Beaverhead Basin**	-	Uthman, W., Beck J. (1998). Hydrogeology of the Upper Beaverhead Basin near Dillon, Montana. Montana Bureau of Mines and Geology Open-File Report 384, 605 pp. Accessed March 29, 20221 from http://dnrc.mt.gov/divisions/water/management/docs/ground-water-studies/hydrogeology_upper_beaverhead_near_dillon.pdf	-	-	Approximated from Fig. 1 of Uthman and Beck (1998); northeast boundary extended beyond Fig. 1 outline based on well completion data.
**Upper Beichuan Area**	Huangshui Basin	Li, H., Zhan, R., Lu, Y., Zhou, B., Wu, J. (2021). Spatiotemporal variation and periodic evolution characteristics of groundwater in the Xining area of China, eastern Qinghai–Tibet Plateau. Environmental Earth Sciences, 80, 1-19.	-	-	Approximated from Fig. 1b and Fig. 1d by Li et al. (2021)
**Upper Borrego Valley**	-	Mathany, T.M., Wright, M.T., Beuttel, B.S., Belitz, K. (2012). Groundwater-quality data in the Borrego Valley, Central Desert, and Low-Use Basins of the Mojave and Sonoran Deserts study unit, 2008–2010—Results from the California GAMA Program: U.S. Geological Survey Data Series 659, 100 pp. Accessed April 27, 2022 via	Faunt, C.C., Stamos, C.L., Flint, L.E., Wright, M.T., Burgess, M.K., Sneed, M., Brandt, J., Martin, P., Coes, A.L. (2015). Hydrogeology, hydrologic effects of development, and simulation of groundwater flow in the Borrego Valley, San Diego County, California. US Geological Survey Scientific Investigations Report 2015–5150, 154 pp.	-	Approximated from Fig. 2 by Mathany et al. (2012) and Fig. 6 by Faunt et al. (2015)

Aquifer system	Broader aquifer system	Reference A	Reference B	Reference C	Steps to delineate
		https://pubs.usgs.gov/ds/659/pdf/ds659.pdf	Accessed April 27, 2022 via https://pubs.usgs.gov/sir/2015/5150/sir20155150.pdf		
**Upper Butte Creek Basin**	Sierra Nevada Foothills **	Shelton, J.L., Fram, M.S., Munday, C.M., Belitz, K. (2010). Groundwater-quality data for the Sierra Nevada study unit, 2008. Results from the California GAMA program. U.S. Geological Survey Data Series 534, 106 pp. Accessed April 15, 2021 from https://pubs.usgs.gov/ds/534/ds_534.pdf	-	-	Approximated on the basis of the density of completed wells (from the California Department of Water Resources); general hydrogeology of region summarized by Shelton et al. (2010).
**Upper Calaveras Basin**	Sierra Nevada Foothills **	Shelton, J.L., Fram, M.S., Munday, C.M., Belitz, K. (2010). Groundwater-quality data for the Sierra Nevada study unit, 2008. Results from the California GAMA program. U.S. Geological Survey Data Series 534, 106 pp. Accessed April 15, 2021 from https://pubs.usgs.gov/ds/534/ds_534.pdf	-	-	Approximated on the basis of the density of completed wells (from the California Department of Water Resources); general hydrogeology of region summarized by Shelton et al. (2010).
**Upper Carbonate Aquifer**	Northern Midwest Aquifer System	Olcott, P.G. (1992). Groundwater Atlas of the United States: Segment 9 Iowa, Michigan, Minnesota, Wisconsin. U.S. Geological Survey Hydrologic Atlas 730-J, 33 pp. Accessed April 12, 2021 from https://pubs.usgs.gov/ha/730j/report.pdf	Young, H.L. (1992). Hydrogeology of the Cambrian-Ordovician Aquifer System in the Northern Midwest, United States. U.S. Geological Survey Professional Paper 1405-B, 108 pp. Accessed April 12, 2021 from https://pubs.usgs.gov/pp/1405b/report.pdf	Savoca, M.E., Sadorf, E.M., Akers, K.K. (1999). Ground-water quality in the eastern part of the Silurian-Devonian and Upper Carbonate Aquifers in the eastern Iowa basins, Iowa and Minnesota, 1996. US Geological Survey Water-Resources Investigations Report 98-4224, 35 pp. Accessed May 18, 2022 via	Broader aquifer system title and its location approximated from Fig. 1 of Young (1992). Upper Carbonate Aquifer location approximated from Fig. 17 of Olcott (1992) and Fig. 2 by Savoca et al. (1999)

Aquifer system	Broader aquifer system	Reference A	Reference B	Reference C	Steps to delineate
				https://pubs.usgs.gov/wri/1998/wri984224/pdf/wri98-4224.pdf	
**Upper Cauvery Banded Gneiss Aquifers**	Dharwar Craton Banded Gneiss Complex	Central Ground Water Board (2012c). Aquifer Systems of Karnataka. Central Ground Water Board Report, 75 pp. Accessed June 30, 2021 from http://cgwb.gov.in/AQM/Karnataka.pdf	-	-	Approximated from Plate III by Central Ground Water Board (2012c)
**Upper Chowchilla Basin**	Sierra Nevada Foothills **	Shelton, J.L., Fram, M.S., Munday, C.M., Belitz, K. (2010). Groundwater-quality data for the Sierra Nevada study unit, 2008. Results from the California GAMA program. U.S. Geological Survey Data Series 534, 106 pp. Accessed April 15, 2021 from https://pubs.usgs.gov/ds/534/ds_534.pdf	-	-	Approximated on the basis of the density of completed wells (from the California Department of Water Resources); general hydrogeology of region summarized by Shelton et al. (2010).
**Upper Coastal Plain**	Floridan Aquifer System	Williams, L.J., Kuniansky, E.L. (2016). Revised hydrogeologic framework of the Floridan aquifer system in Florida and parts of Georgia, Alabama, and South Carolina. U.S. Geological Survey Professional Paper 1807, 140 pp. Accessed March 31, 2021 from https://pubs.usgs.gov/pp/1807/pdf/pp1807.pdf	Aucott, W.R. (1996). Hydrology of the Southeastern Coastal Plain Aquifer System in South Carolina and Parts of Georgia and North Carolina. U.S Geological Survey Professional Paper 1410-E, 95 pp. Accessed March 31, 2021 from https://pubs.usgs.gov/pp/1410e/report.pdf	-	Broader Florida Aquifer System from Williams and Kuniansky (2016). Approximate divide between the Upper and Lower Coastal Plain from Fig. 1 of Aucott (1996).
**Upper Copiapo Valley**	Copiapo Valley	Donoso, G., Lictevout, E., Rinaudo, J. D. (2020). Groundwater management	Direccion General de Aguas (2015). Plan Nacional de Estudios Acuíferos. Report	-	Approximated from Fig. 25.2 by Donoso et al. (2020) and Fig. 2a

Aquifer system	Broader aquifer system	Reference A	Reference B	Reference C	Steps to delineate
		lessons from Chile. In: Sustainable Groundwater Management. Springer, Cham., pp. 481-509. Accessed May 12, 2021 from https://link.springer.com/chapter/10.1007/978-3-030-32766-8_25	number 381, Santiago, Chile, 44 pp. Accessed May 16, 2021 from http://bibliotecadigital.ciren.cl/bitstream/handle/123456789/32415/DGA_2015_actualizacion_plan_nacional_acuiferos.pdf?sequence=1&isAllowed=y		by Direccion General de Aguas (2015)
**Upper Cosumnes Basin**	Sierra Nevada Foothills **	Shelton, J.L., Fram, M.S., Munday, C.M., Belitz, K. (2010). Groundwater-quality data for the Sierra Nevada study unit, 2008. Results from the California GAMA program. U.S. Geological Survey Data Series 534, 106 pp. Accessed April 15, 2021 from https://pubs.usgs.gov/ds/534/ds_534.pdf	-	-	Approximated on the basis of the density of completed wells (from the California Department of Water Resources); general hydrogeology of region summarized by Shelton et al. (2010).
**Upper Deschutes Basin**	-	Gannett, M.W., Lite, Jr., K.E., Morgan, D.S., and Collins, C.A. (2001). Ground-water hydrology of the upper Deschutes Basin, Oregon: U.S. Geological Survey Water-Resources Investigations Report 00-4162, 74 p. Accessed February 24, 2021 from https://pubs.usgs.gov/wri/wri004162/	Oregon Water Resources Department (2021). Well Report Query. Web Page Accessed February 24, 2021 from https://apps.wrd.state.or.us/apps/gw/well_log/Default.aspx	-	Approximated from Fig.1 of Gannett et al. (2001) and influenced by the spatial distribution of groundwater wells recorded by the Oregon Water Resources Department (2021).
**Upper Dry and King Basins**	Sierra Nevada Foothills **	Shelton, J.L., Fram, M.S., Munday, C.M., Belitz, K. (2010). Groundwater-quality data for the Sierra Nevada study unit, 2008. Results from the California GAMA program. U.S.	-	-	Approximated on the basis of the density of completed wells (from the California Department of Water Resources); general hydrogeology of region summarized by Shelton et al. (2010).

Aquifer system	Broader aquifer system	Reference A	Reference B	Reference C	Steps to delineate
		Geological Survey Data Series 534, 106 pp. Accessed April 15, 2021 from https://pubs.usgs.gov/ds/534/ds_534.pdf			
**Upper Feather River Basin**	Sierra Nevada Foothills **	Shelton, J.L., Fram, M.S., Munday, C.M., Belitz, K. (2010). Groundwater-quality data for the Sierra Nevada study unit, 2008. Results from the California GAMA program. U.S. Geological Survey Data Series 534, 106 pp. Accessed April 15, 2021 from https://pubs.usgs.gov/ds/534/ds_534.pdf	-	-	Approximated on the basis of the density of completed wells (from the California Department of Water Resources); general hydrogeology of region summarized by Shelton et al. (2010).
**Upper Fresno Basin**	Sierra Nevada Foothills **	Shelton, J.L., Fram, M.S., Munday, C.M., Belitz, K. (2010). Groundwater-quality data for the Sierra Nevada study unit, 2008. Results from the California GAMA program. U.S. Geological Survey Data Series 534, 106 pp. Accessed April 15, 2021 from https://pubs.usgs.gov/ds/534/ds_534.pdf	-	-	Approximated on the basis of the density of completed wells (from the California Department of Water Resources); general hydrogeology of region summarized by Shelton et al. (2010).
**Upper Gangetic Plain**	Indo-Gangetic Plain	Bonsor, H. C., MacDonald, A. M., Ahmed, K. M., Burgess, W. G., Basharat, M., Calow, R. C., Dixit, A., Foster, S.S.D., Gopal, K., Lapworth, D.J., Moench, M., Mukherjee, A., Rao, M.S., Shamsudduha, M., Smith, L., Taylor, R.G., Tucker, J.,	Singh, J., Erenstein, O., Thorpe, W. R., Varma, A. (2007). Crop-livestock interactions and livelihoods in the Gangetic Plains of Uttar Pradesh, India. International Livestock Research Institute.	Mukherjee, A., Saha, D., Harvey, C. F., Taylor, R. G., Ahmed, K. M., Bhanja, S. N. (2015). Groundwater systems of the Indian sub-continent. Journal of Hydrology: Regional Studies, 4, 1-14.	Approximated from Fig. 3 by Mukherjee et al. (2015), Fig. 3 by Bonsor et al. (2020), and Fig. 1 by Singh et al. (2007)

Aquifer system	Broader aquifer system	Reference A	Reference B	Reference C	Steps to delineate
		van Steenbergen, F., Yadav, S.K., Zahid, A. (2017). Hydrogeological typologies of the Indo-Gangetic basin alluvial aquifer, South Asia. Hydrogeology Journal, 25, 1377-1406.			
**Upper Guadalquivir Basin**	Guadalquivir Basin	Dumont, A., Salmoral, G., Llamas, M. R. (2013). The water footprint of a river basin with a special focus on groundwater: The case of Guadalquivir basin (Spain). Water Resources and Industry, 1, 60-76.	-	-	Broader basin boundaries approximated from Fig. 1 by Dumont et al. (2013)
**Upper Humboldt Basin**	-	Plume, R. W. (2009). Hydrogeologic Framework and Occurrence and Movement of Ground Water in the Upper Humboldt River Basin, Northeastern Nevada. US Geological Survey Scientific Investigations Report 2009–5014, 30 pp. Accessed March 2021 from https://pubs.usgs.gov/sir/2009/5014/pdf/sir20095014.pdf	-	-	Approximated from Plate 1 of Plume (2009)
**Upper Kabootar Ahang Basin**	Razan Basin	Hosseini, S. M., Parizi, E., Ataie-Ashtiani, B., Simmons, C. T. (2019). Assessment of sustainable groundwater resources management using integrated environmental index: Case studies across Iran. Science of the Total Environment, 676, 792-810.	-	-	Approximated from Fig. 6 by Hosseini et al. (2019)

Aquifer system	Broader aquifer system	Reference A	Reference B	Reference C	Steps to delineate
**Upper Kaweah Basin**	Sierra Nevada Foothills **	Shelton, J.L., Fram, M.S., Munday, C.M., Belitz, K. (2010). Groundwater-quality data for the Sierra Nevada study unit, 2008. Results from the California GAMA program. U.S. Geological Survey Data Series 534, 106 pp. Accessed April 15, 2021 from https://pubs.usgs.gov/ds/534/ds_534.pdf	-	-	Approximated on the basis of the density of completed wells (from the California Department of Water Resources); general hydrogeology of region summarized by Shelton et al. (2010).
**Upper La Ligua Aquifer**	La Ligua Aquifer	Direccion General de Aguas (2009). Diagnóstico y Clasificación de Sectores Acuíferos, Volumen No. 2. Gobierno de Chile Ministerio de Obras Publicas Report, 384 pp. Accessed May 16, 2021 from https://snia.mop.gob.cl/sad/CQA5168v2.pdf	-	-	Approximated from Table 1.7 on page 37 by Direccion General de Aguas (2009). Lower and upper portions of the aquifer system delineated near Cabildo.
**Upper La Paz Basin**	La Paz Basin	Muir, M.A.K., Martinez, A.A. (2018). Preliminary Assessment of Water Resources including Climate Considerations for the Los Cabos and La Paz Municipalities in the State of Baja California Sur, Mexico. Conference: International Water Association Water, Energy and Climate Conference (May 21 to 23, 2024), Mexico City, 13 pp. Accessed May 6, 2021 from https://arctic.ucalgary.ca/sites/default/files/April18-IWA-	-	-	Approximated from map on page 2 of Muir and Martinez (2018).

Aquifer system	Broader aquifer system	Reference A	Reference B	Reference C	Steps to delineate
		FinalConferenceVersion-MAKMuir.pdf			
**Upper Lost River**	Upper Klamath Basin	Gannett, M.W., Breen, K.H. (2015). Groundwater levels, trends, and relations to pumping in the Bureau of Reclamation Klamath Project, Oregon and California: U.S. Geological Survey Open-File Report 2015-1145, 19 pp. Accessed March 20, 2021 from https://pubs.usgs.gov/of/2015/1145/ofr20151145.pdf	-	-	Approximated from Fig. 1 of Gannett and Breen (2015).
**Upper Mashad Plain**	Mashad-Narimani Basin	Khashei-Siuki, A., Sarbazi, M. (2015). Evaluation of ANFIS, ANN, and geostatistical models to spatial distribution of groundwater quality (case study: Mashhad plain in Iran). Arabian Journal of Geosciences, 8, 903-912.	Naghibi, S. A., Vafakhah, M., Hashemi, H., Pradhan, B., & Alavi, S. J. (2018). Groundwater augmentation through the site selection of floodwater spreading using a data mining approach (case study: Mashhad Plain, Iran). Water, 10, 1405.	Hosseini, S. M., Parizi, E., Ataie-Ashtiani, B., Simmons, C. T. (2019). Assessment of sustainable groundwater resources management using integrated environmental index: Case studies across Iran. Science of the Total Environment, 676, 792-810.	Approximated from Fig. 2 by Khashei-Siuki and Sarbazi (2015), Fig. 2 by Naghibi et al. (2018), and Fig. 6 by Hosseini et al. (2019)
**Upper Merced Basin**	Sierra Nevada Foothills **	Shelton, J.L., Fram, M.S., Munday, C.M., Belitz, K. (2010). Groundwater-quality data for the Sierra Nevada study unit, 2008. Results from the California GAMA program. U.S. Geological Survey Data Series 534, 106 pp. Accessed April 15, 2021 from https://pubs.usgs.gov/ds/534/ds_534.pdf	-	-	Approximated on the basis of the density of completed wells (from the California Department of Water Resources); general hydrogeology of region summarized by Shelton et al. (2010).

Aquifer system	Broader aquifer system	Reference A	Reference B	Reference C	Steps to delineate
**Upper Mokelumne Basin**	Sierra Nevada Foothills **	Shelton, J.L., Fram, M.S., Munday, C.M., Belitz, K. (2010). Groundwater-quality data for the Sierra Nevada study unit, 2008. Results from the California GAMA program. U.S. Geological Survey Data Series 534, 106 pp. Accessed April 15, 2021 from https://pubs.usgs.gov/ds/534/ds_534.pdf	-	-	Approximated on the basis of the density of completed wells (from the California Department of Water Resources); general hydrogeology of region summarized by Shelton et al. (2010).
**Upper Morghab Basin**	-	Hosseini, S. M., Parizi, E., Ataie-Ashtiani, B., Simmons, C. T. (2019). Assessment of sustainable groundwater resources management using integrated environmental index: Case studies across Iran. Science of the Total Environment, 676, 792-810.	-	-	Approximated from Fig. 6 by Hosseini et al. (2019)
**Upper Morovian Basin**	-	Kadlecová, R., Olmer, M. (2011). Review of Groundwater Resources. Geologické výzkumy na Moravě a ve Slezsku. Proceedings of The Open Congress ČGS and SGS, September 22–25, 2011, Monínec 18, pp. 31-34. Accessed October 21, 2021 from https://journals.muni.cz/gvms/article/view/1679	-	-	Approximated from Fig. 2 by Kadlecová and Olmer (2011)
**Upper Nowbaran Basin**	-	Hosseini, S. M., Parizi, E., Ataie-Ashtiani, B., Simmons, C. T. (2019). Assessment of sustainable	-	-	Approximated from Fig. 6 by Hosseini et al. (2019)

Aquifer system	Broader aquifer system	Reference A	Reference B	Reference C	Steps to delineate
		groundwater resources management using integrated environmental index: Case studies across Iran. Science of the Total Environment, 676, 792-810.			
**Upper Peninsula Jacobsville Sandstone and Cambrian-Ordovician and Silurian-Devonian Aquifers**	Northern Midwest Aquifer System	Olcott, P.G. (1992). Groundwater Atlas of the United States: Segment 9 Iowa, Michigan, Minnesota, Wisconsin. U.S. Geological Survey Hydrologic Atlas 730-J, 33 pp. Accessed April 12, 2021 from https://pubs.usgs.gov/ha/730j/report.pdf	-	-	Approximated from Figs. 77, 97 and 133 by Olcott (1992).
**Upper Poso Basin**	Sierra Nevada Foothills **	Shelton, J.L., Fram, M.S., Munday, C.M., Belitz, K. (2010). Groundwater-quality data for the Sierra Nevada study unit, 2008. Results from the California GAMA program. U.S. Geological Survey Data Series 534, 106 pp. Accessed April 15, 2021 from https://pubs.usgs.gov/ds/534/ds_534.pdf	-	-	Approximated on the basis of the density of completed wells (from the California Department of Water Resources); general hydrogeology of region summarized by Shelton et al. (2010).
**Upper Qeydar Basin**	Qeydar Basin	Hosseini, S. M., Parizi, E., Ataie-Ashtiani, B., Simmons, C. T. (2019). Assessment of sustainable groundwater resources management using integrated environmental index: Case studies across Iran. Science of the Total Environment, 676, 792-810.	-	-	Approximated from Fig. 6 by Hosseini et al. (2019)

Aquifer system	Broader aquifer system	Reference A	Reference B	Reference C	Steps to delineate
**Upper Rio Elqui Basin**	Elqui River Basin	Ribeiro, L., Kretschmer, N., Nascimento, J., Buxo, A., Rötting, T., Soto, G., Señoret, M., Oyarzún, J., Maturana, H., Oyarzún, R. (2015). Evaluating piezometric trends using the Mann-Kendall test on the alluvial aquifers of the Elqui River basin, Chile. Hydrological Sciences Journal, 60, 1840-1852.	-	-	Approximated from Fig. 1 by Ribeiro et al. (2015)
**Upper Rio Fuerte Aquifer**	-	CONAGUA (2015c). Actualización de la disponibilidad media anual de agua en al acuifero Rio Fuerte (2501), Estado de Sinaloa. Comision Nacional del Agua Report, 29 pp. Accessed May 12, 2021 from https://www.gob.mx/cms/uploads/attachment/file/103330/DR_2501.pdf	-	-	Approximated from Fig. 1 by CONAGUA (2015c).
**Upper Rio Petorca Basin**	Rio Petorca Basin	Bujes Moreno, N.J.I. (2015). Estudio de la propiedad del agua subterránea del acuífero del Río Petorca en la Región de Valparaíso, Chile. Universidad de Chile Thesis, 72 pp. Accessed May 17, 2021 from http://repositorio.uchile.cl/handle/2250/150902	-	-	Approximated from Fig. 12 by Bujes Moreno (2015)
**Upper Rustaq-Fork Basin**	-	Parizi, E., Hosseini, S. M., Ataie-Ashtiani, B., Simmons, C. T. (2020). Normalized difference vegetation index as the dominant predicting factor	-	-	Approximated from tabulated coordinates in the Supplementary Information within Parizi et al. (2020) and physiographic and drainage patterns

Aquifer system	Broader aquifer system	Reference A	Reference B	Reference C	Steps to delineate
		of groundwater recharge in phreatic aquifers: case studies across Iran. Scientific Reports, 10, 17473.			
**Upper Salmon Basin**	-	Graham, W.G., Campbell, L.J. (1981). Groundwater resources of Idaho. Idaho Department of Water Resources Report, 61 pp. Accessed March 23, 2021 from https://idwr.idaho.gov/files/publications/198108-MISC-GW-Resources-ID.pdf	-	-	Approximated from Plate 1 of Graham and Campbell (1981).
**Upper Santa Cruz Basin**	Tucson Basin and Avra Valley	Flora, S., Davis, T. (2009). Hydrologic Map Series (HMS), Water Level Change Map Series (WLCMS), and Basin Sweep Assessment Report ADWR Basins and Sub-Basins. Arizona Department of Water Resources Hydrology Division Field Services Section https://new.azwater.gov/sites/default/files/HMSWLCMSBasinSweepAssessmentReport2009.pdf	Coes, A., Gellenbeck, D.J., Towne, D.C., Freark, M.C. (2002). Ground water quality in the Upper Santa Cruz Basin. U.S. Geological Survey Water-Resources Investigations Report 00-4117, 66 pp. Accessed March 31, 2021 from https://pubs.usgs.gov/wri/2000/4117/report.pdf	Hanson, R.T. (1989). Aquifer-system compaction, Tucson Basin and Avra Valley, Arizona. Water-Resources Investigations Report 88-4172, 75 pp. Accessed April 15, 2021 from https://pubs.usgs.gov/wri/1988/4172/report.pdf	Approximated from Fig. 1 of Flora and Davis (2009). See also Coes et al. (2002). Broader Tucson Basin and Avra Valley outline from Fig. 1 of Hanson (1989).
**Upper Santa Ynez Valley**	Santa Ynez Valley	Upson, J. E., Thomasson, H. G. (1951). Geology and water resources of the Santa Ynez river basin, Santa Barbara County, California (Vol. 2). US Geological Survey Water Supply Report 1102, Accessed March 7, 2021 from	Hamlin, S. N. (1985). Ground-water quality in the Santa Rita, Buellton, and Los Olivos hydrologic subareas of the Santa Ynez River basin, Santa Barbara County, California. Water Resources Investigations Report 84-4131, 84 pp. Accessed March 7, 2021 from	Wilson, H. D. (1959). Ground-water appraisal of Santa Ynez River basin, Santa Barbara County, California, 1945-52. Water Supply Paper 1467. 128 pp. Accessed March 7, 2021 from https://pubs.usgs.gov/wsp/1467/report.pdf	Approximated from Fig. 1 by Upson and Thomasson (1951), pp. 22 map in Hamlin (1985), and Fig. 2 of Wilson (1959). Upper portion of Valley based on the delineated subareas: "Santa Ynez" and "Headwater Subarea"

Aquifer system	Broader aquifer system	Reference A	Reference B	Reference C	Steps to delineate
		https://pubs.usgs.gov/wsp/1107/report.pdf	https://pubs.usgs.gov/wri/1984/4131/report.pdf		
**Upper Souss Basin**	Souss-Chtouka Basin	Garduño, H., Foster, S. (2010). Sustainable Groundwater Irrigation. Approaches to Reconciling Demand with Resources. GW•MATE Strategic Overview Series No. 4 World Bank (Washington DC, USA). Accessed 05/01/2023 via https://openknowledge.worldbank.org/server/api/core/bitstreams/a6957092-3680-52cd-9707-91143c386175/content	Tagma, T., Hsissou, Y., Bouchaou, L., Bouragba, L., Boutaleb, S. (2009). Groundwater nitrate pollution in Souss-Massa basin (south-west Morocco). African Journal of Environmental Science and Technology, 3, 301-309.	Bouchaou, L., Michelot, J.L., Vengosh, A., Hsissou, Y., Qurtobi, M., Gaye, C.B., Bullen, T.D., Zuppi, G. M. (2008). Application of multiple isotopic and geochemical tracers for investigation of recharge, salinization, and residence time of water in the Souss–Massa aquifer, southwest of Morocco. Journal of Hydrology, 352, 267-287.	Broader aquifer system boundaries approximated from Box 5 by Garduño and Foster (2010), Fig. 1 by Bouchaou et al. (2008), and Fig. 1 by Tagma et al. (2009). Southern limit approximated along the Massa River.
**Upper Stanislaus and Rock Creek Basins**	Sierra Nevada Foothills **	Shelton, J.L., Fram, M.S., Munday, C.M., Belitz, K. (2010). Groundwater-quality data for the Sierra Nevada study unit, 2008. Results from the California GAMA program. U.S. Geological Survey Data Series 534, 106 pp. Accessed April 15, 2021 from https://pubs.usgs.gov/ds/534/ds_534.pdf	-	-	Approximated on the basis of the density of completed wells (from the California Department of Water Resources); general hydrogeology of region summarized by Shelton et al. (2010).
**Upper Thracian Plio-Quaternary Aquifer**	-	Machkova, M., Velikov, B., Machkova, M., Dimitrov, D., Neytchev, N. (2008). Quality status of the water in the Upper Thracian Plio-Quaternary Aquifer (south Bulgaria). In: Natural Groundwater Quality (eds. Edmunds, W.M., Shand, P.), pp. 391-403.	-	-	Approximated from Fig. 18.1 by Machkova et al. (2008)

Aquifer system	Broader aquifer system	Reference A	Reference B	Reference C	Steps to delineate
**Upper Tule Basin**	Sierra Nevada Foothills **	Shelton, J.L., Fram, M.S., Munday, C.M., Belitz, K. (2010). Groundwater-quality data for the Sierra Nevada study unit, 2008. Results from the California GAMA program. U.S. Geological Survey Data Series 534, 106 pp. Accessed April 15, 2021 from https://pubs.usgs.gov/ds/534/ds_534.pdf	-	-	Approximated on the basis of the density of completed wells (from the California Department of Water Resources); general hydrogeology of region summarized by Shelton et al. (2010).
**Upper Tuolumne Basin**	Sierra Nevada Foothills **	Shelton, J.L., Fram, M.S., Munday, C.M., Belitz, K. (2010). Groundwater-quality data for the Sierra Nevada study unit, 2008. Results from the California GAMA program. U.S. Geological Survey Data Series 534, 106 pp. Accessed April 15, 2021 from https://pubs.usgs.gov/ds/534/ds_534.pdf	-	-	Approximated on the basis of the density of completed wells (from the California Department of Water Resources); general hydrogeology of region summarized by Shelton et al. (2010).
**Upper Yuba Basin**	Sierra Nevada Foothills **	Shelton, J.L., Fram, M.S., Munday, C.M., Belitz, K. (2010). Groundwater-quality data for the Sierra Nevada study unit, 2008. Results from the California GAMA program. U.S. Geological Survey Data Series 534, 106 pp. Accessed April 15, 2021 from https://pubs.usgs.gov/ds/534/ds_534.pdf	-	-	Approximated on the basis of the density of completed wells (from the California Department of Water Resources); general hydrogeology of region summarized by Shelton et al. (2010).
**Uraba Aquifer**	-	Villegas, P., Paredes, V., Betancur, T., Ribeiro, L.	Ossa-Valencia, J., Betancur-Vargas, T.	Bocanegra, E., Da Silva, G. C., Custodio, E., Manzano,	Approximated from Fig. 1 by Villegas et al. (2013), Fig. 1 by

Aquifer system	Broader aquifer system	Reference A	Reference B	Reference C	Steps to delineate
		(2013). Assessing the hydrochemistry of the Urabá Aquifer, Colombia by principal component analysis. Journal of Geochemical Exploration, 134, 120-129.	(2018). Hydrogeochemical characterization and identification of a system of regional flow. Case study: the aquifer on the Gulf of Urabá, Colombia. Revista Facultad de Ingeniería Universidad de Antioquia, 86, 9-18.	M., Montenegro, S. (2010). State of knowledge of coastal aquifer management in South America. Hydrogeology Journal, 18, 261-267.	Ossa-Valencia and Betancur-Vargas (2018) and Fig. 1 by Bocanegra et al. (2010)
**Urmia Aquifer**	-	Javadzadeh, H., Ataie-Ashtiani, B., Hosseini, S. M., Simmons, C. T. (2020). Interaction of lake-groundwater levels using cross-correlation analysis: A case study of Lake Urmia Basin, Iran. Science of The Total Environment, 729, 138822.	-	-	Approximated from Fig. 1 by Javadzadeh et al. (2020)
**Urumqi Valley**	Junggar Basin	Wang, D., Yang, C., Shao, L. (2021). The spatiotemporal evolution of hydrochemical characteristics and groundwater quality assessment in Urumqi, Northwest China. Arabian Journal of Geosciences, 14, 1-14.	Zhou, Z., & Zhong, J. (2022). Role of Atmospheric Temperature and seismic activity in spring water hydrogeochemistry in Urumqi, China. International Journal of Environmental Research and Public Health, 19, 12004.	-	Approximated from Fig. 1 by Wang et al. (2021) and from Fig. 1 by Zhou and Zhong (2022)
**Utah Lake Valley**	-	Dustin, J.D. (1978). Hydrogeology of Utah Lake with Emphasis on Goshen Bay. PhD Dissertation, Brigham Young University. 184 pp. Accessed March 27, 2021 from https://apps.dtic.mil/sti/pdfs/ADA065478.pdf	-	-	Approximated from Fig. 1 of Dustin (1978)

Aquifer system	Broader aquifer system	Reference A	Reference B	Reference C	Steps to delineate
**Uvas Valley**	-	State of New Mexico Office of the State Engineer (1998). Nutt-Hockett Basin Hydrographic Survey Report. 267 pp. Accessed March 27, 2021 from https://www.ose.state.nm.us/HydroSurvey/nutt-hockett/report.pdf	-	-	Approximated from map on page vi of State of New Mexico, Office of the State Engineer (1998)
**Valencia Plain**	-	Hernández, F., Marín, J. M., Pozo, Ó. J., Sancho, J. V., López, F. J., Morell, I. (2008). Pesticide residues and transformation products in groundwater from a Spanish agricultural region on the Mediterranean Coast. International Journal of Environmental and Analytical Chemistry, 88, 409-424.	-	-	Approximated from Fig. 1 by Hernández et al. (2008)
**Valle de Chicalote**	-	Cigna, F., Tapete, D. (2021). Satellite InSAR survey of structurally-controlled land subsidence due to groundwater exploitation in the Aguascalientes Valley, Mexico. Remote Sensing of Environment, 254, 112254.	-	-	Approximated from Fig. 1b by Cigna and Tapete (2021).
**Valle de Escuinapa**	-	CONAGUA (2020dx). Actualización de la disponibilidad media anual de agua en el acuífero Valle de Escuinapa (2511), estado de Sinaloa. 29 pp. Accessed March 8, 2022 via https://sigagis.conagua.gob.mx/gas1/Edos_Acuiferos_18/sinaloa/DR_2511.pdf	-	-	Approximated from physiography, well completion data, and Fig. 1 by CONAGUA (2020dx)

Aquifer system	Broader aquifer system	Reference A	Reference B	Reference C	Steps to delineate
**Valle de Juarez and Hueco Bolson**	Tularosa-Huelco	Sanchez, R., Eckstein, G. (2020). Groundwater management in the borderlands of Mexico and Texas: The beauty of the unknown, the negligence of the present, and the way forward. Water Resources Research, 56(3), e2019WR026068.	Day, J. C. (1978). International Aquifer Management: The Hueco Bolson on the Rio Grande River. Natural Resources Journal, 163-180.	-	Approximated from Fig. 1 of Day (1978) and Fig. 2 of Sanchez and Eckstein (2020).
**Valparaiso Aquifer**	-	CONAGUA (2021a). Acuíferos (nacional). Webpage accessed March 8, 2022 from http://sina.conagua.gob.mx/sina/tema.php?tema=acuiferos&ver=mapa&o=1&n=nacional	-	-	Approximated from aquifer boundaries defined by CONAGUA (2021a) web portal, physiography and drainage patterns, and well completion data.
**Vamoosa Ada Aquifer**	-	D'Lugosz, J.J., McClaflin, R.G. (1986). Geohydrology of the Vamoosa-Ada aquifer east-central Oklahoma with a section on chemical quality of water. U.S. Geological Survey Circular 87, 48 pp. Accessed April 5, 2021 from http://www.ogs.ou.edu/pubsscanned/Circulars/circular87mm.pdf	-	-	Approximated from plate 1 of D'Lugosz and McClaflin (1986)
**Vanegas-Catorce Aquifer**	-	CONAGUA (2020gi). Actualización de la disponibilidad media anual de agua en el acuífero Vanegas-Catorce (2401), estado de San Luis Potosí. 12 pp. Accessed March 29, 2022 via https://sigagis.conagua.gob.mx/gas1/Edos_Acuiferos_1	-	-	Approximated from physiography, well completion data, and Fig. 1 by CONAGUA (2020gi)

Aquifer system	Broader aquifer system	Reference A	Reference B	Reference C	Steps to delineate
		8/sanluispotosi/DR_2401.pdf			
**Vanrhynsdorp Aquifer**	-	Department of Water and Sanitation (2015). Groundwater status report - Western Cape Region. Department of Water and Sanitation map, 1 pp. Accessed May 3, 2023 via https://www.dws.gov.za/Groundwater/GroundwaterOffices/WC/Annual%20report_%20groundwater%20status%20A0%20-%20201503.pdf	-	-	Approximated from map within map by Department of Water and Sanitation (2015)
**Varamin Aquifer**	Tehran Plain	Motagh, M., Walter, T. R., Sharifi, M. A., Fielding, E., Schenk, A., Anderssohn, J., Zschau, J. (2008). Land subsidence in Iran caused by widespread water reservoir overexploitation. Geophysical Research Letters, 35, L16403.	Tafreshi, G. M., Nakhaei, M., Lak, R. (2019). Land subsidence risk assessment using GIS fuzzy logic spatial modeling in Varamin aquifer, Iran. GeoJournal, 86, 1203-1223.	-	Approximated from Fig. 1a by Motagh et al. (2008) and Fig. 1 by Tafreshi et al. (2021).
**Vega Baja and Media of the Segura River Basin**	-	Tomás, R., Márquez, Y., Lopez-Sanchez, J.M., Delgado, J., Blanco, P., Mallorquí, J.J., Martínez, M., Herrera, G., Mulas, J. (2005). Mapping ground subsidence induced by aquifer overexploitation using advanced Differential SAR Interferometry: Vega Media of the Segura River (SE Spain) case study. Remote Sensing of Environment, 98, pp.269-283.	Tomás, R., Lopez-Sanchez, J. M., Delgado, J., Mallorquí Franquet, J. J., & Herrera García, G. (2008). DInSAR monitoring of aquifer compaction due to water withdrawal: Vegas Baja and Media of the Segura river (SE, Spain) case study. In: Droughts: Causes, Effects and Predictions (ed. Sánchez, J.M.), pp. 253-276.	-	Approximated from Fig. 1 by Tomás et al. (2005) and Fig. 1 by Tomás et al. (2008)

Aquifer system	Broader aquifer system	Reference A	Reference B	Reference C	Steps to delineate
**Vega de Granada Aquifer**	-	Chica-Olmo, M., Luque-Espinar, J.A., Rodriguez-Galiano, V., Pardo-Igúzquiza, E., Chica-Rivas, L. (2014). Categorical Indicator Kriging for assessing the risk of groundwater nitrate pollution: the case of Vega de Granada aquifer (SE Spain). Science of the Total Environment, 470, 229-239.	-	-	Approximated from Fig. 1 by Chica-Olmo et al. (2014)
**Velez Aquifer**	-	Morell, I. (2003). Acuíferos detríticos costeros. Hidrogeología y Aguas Subterráneas, 1, 31-44.	-	-	Approximated from Fig. 5 by Morell (2003)
**Vicente Guerrero-Poanas Aquifer**	-	CONAGUA (2020dk). Actualización de la disponibilidad media anual de agua en el acuífero Vicente Guerrero-Poanas (1004), estado de Durango. 38 pp. Accessed March 8, 2022 via https://sigagis.conagua.gob.mx/gas1/Edos_Acuiferos_18/durango/DR_1004.pdf	-	-	Approximated from physiography, well completion data, and Fig. 1 by CONAGUA (2020dk)
**Victoria Area**	Gulf Coast Regional Aquifer System	Weiss, J. S. (1990). Geohydrologic units of the coastal lowlands aquifer system, south-central United States. U.S. Geological Survey Regional aquifer-systems analysis. 42 pp. Accessed March 30, 2021 from https://pubs.usgs.gov/pp/1416c/report.pdf	Marvin, R.F., Shafer, G.H., Dale, O.C. (1962). Groundwater resources of Victoria and Calhoun Counties, Texas. Accessed March 30, 2021 from https://www.twdb.texas.gov/publications/reports/bulletins/doc/Bull.htm/B6202.asp	-	Outer margin of Gulf Coast Regional Aquifer System approximated from Fig. 2 of Weiss (1990). Delineation of central portion of subarea (and regional geology) aided by plate 1a of Marvin et al. (1962). Western margin of subarea approximated along the Nueses River.

Aquifer system	Broader aquifer system	Reference A	Reference B	Reference C	Steps to delineate
**Vidalia Upland**	Floridan Aquifer System	Williams, L.J., Kuniansky, E.L. (2016). Revised hydrogeologic framework of the Floridan aquifer system in Florida and parts of Georgia, Alabama, and South Carolina. U.S. Geological Survey Professional Paper 1807, 140 pp. Accessed March 31, 2021 from https://pubs.usgs.gov/pp/1807/pdf/pp1807.pdf	Clark, W. Z., Zisa, A. C. (1976) Physiographic map of Georgia: Georgia Department of Natural Resources, 1 sheet. Accessed March 31, 2021 from https://epd.georgia.gov/document/publication/sm-4-physiographic-map-georgia-12000000-1988/download	-	Approximated from physiographic provinces of Georgia outlined by Clark and Zisa (1976). Broader Florida Aquifer System from Williams and Kuniansky (2016). Northeastern boundary set along the Savannah River.
**Villa de Arriaga and Pino Suarez Aquifers**	-	CONAGUA (2020gu). Actualización de la disponibilidad media anual de agua en el acuífero Villa de Arriaga (2406), estado de San Luis Potosi. 12 pp. Accessed March 29, 2022 via https://sigagis.conagua.gob.mx/gas1/Edos_Acuiferos_18/sanluispotosi/DR_2406.pdf	CONAGUA (2020gv). Actualización de la disponibilidad media anual de agua en el acuífero Pino Suarez (3233), estado de Zacatecas. 30 pp. Accessed March 29, 2022 via https://sigagis.conagua.gob.mx/gas1/Edos_Acuiferos_18/zacatecas/DR_3233.pdf	-	Approximated from physiography, well completion data, and Fig. 1 by CONAGUA (2020gu) and CONAGUA (2020gv)
**Villanueva Aquifer**	-	CONAGUA (2021a). Acuíferos (nacional). Webpage accessed March 8, 2022 from http://sina.conagua.gob.mx/sina/tema.php?tema=acuiferos&ver=mapa&o=1&n=nacional	-	-	Approximated from aquifer boundaries defined by CONAGUA (2021a) web portal, physiography and drainage patterns, and well completion data.
**Villena-Benejama Region covering the Solana Aquifer**	-	Andreu, J. M., Alcalá, F. J., Vallejos, A., Pulido-Bosch, A. (2011). Recharge to mountainous carbonated aquifers in SE Spain: Different approaches and new challenges. Journal of	-	-	Approximated from Fig. 1 by Andreu et al. (2011)

Aquifer system	Broader aquifer system	Reference A	Reference B	Reference C	Steps to delineate
		Arid Environments, 75, 1262-1270.			
**Vina Basin**	Sacramento Valley of the Central Valley **	California Department of Water Resources (2015i). California's groundwater update 2013 - Chapter 7: Sacramento River Hydrologic Region. California Department of Water Resources Report, 126 pp. Accessed April 27, 2022 via https://water.ca.gov/-/media/DWR-Website/Web-Pages/Programs/Groundwater-Management/Bulletin-118/Files/Statewide-Reports/GWU2013_Ch7_SacramentoRiver_Final.pdf	-	-	Approximated from Fig. 7-2 by California Department of Water Resources (2015i)
**Vinaroz Plain**	-	Hernández, F., Marín, J. M., Pozo, Ó. J., Sancho, J. V., López, F. J., Morell, I. (2008). Pesticide residues and transformation products in groundwater from a Spanish agricultural region on the Mediterranean Coast. International Journal of Environmental and Analytical Chemistry, 88, 409-424.	Giménez-Forcada, E. (2014). Space/time development of seawater intrusion: a study case in Vinaroz coastal plain (Eastern Spain) using HFE-Diagram, and spatial distribution of hydrochemical facies. Journal of Hydrology, 517, 617-627.	Giménez-Forcada, E. (2019). Use of the Hydrochemical Facies Diagram (HFE-D) for the evaluation of salinization by seawater intrusion in the coastal Oropesa Plain: Comparative analysis with the coastal Vinaroz Plain, Spain. HydroResearch, 2, 76-84.	Approximated from Fig. 1 by Hernández et al. (2008), Fig. 2 by Giménez-Forcada (2019), and Fig. 1 by Giménez-Forcada (2014)
**Virgin River Valley**	-	Lopes, T.J., Evetts. D.M. (2005). Ground-Water pumpage and artificial recharge estimates for calendar year 2000 and average annual natural recharge and interbasin flow by hydrographic area, Nevada. Accessed April 25,	Harrill, J.R., Prudic, D.E. (1998). Aquifer systems in the Great Basin region of Nevada, Utah, and adjacent states; summary report. US Geological Survey Professional Paper 1409-A, 75 pp. Accessed April 26, 2022 via	-	Approximated from Fig. 1 by Lopes and Evetts (2005), and Fig. 2 by Harrill and Prudic (1998). Well completion records, physiography and drainage were used to delinate areas.

Aquifer system	Broader aquifer system	Reference A	Reference B	Reference C	Steps to delineate
		2022 via https://pubs.usgs.gov/sir/2004/5239/sir2004-5239.pdf	https://pubs.usgs.gov/pp/1409a/report.pdf		
**Viscaino Aquifer**	-	Brito-Castillo, L., Méndez Rodríguez, L. C., Chávez López, S., Acosta Vargas, B. (2010). Groundwater differentiation of the aquifer in the Vizcaino Biosphere Reserve, Baja California Peninsula, Mexico. Geofísica Internacional, 49, 167-179.	-	-	Approximated from Brito-Castillo et al. (2010).
**Vitoria-Gasteiz Quaternary Aquifer**	-	Arrate, I., Sanchez-Perez, J.M., Antiguedad, I., Vallecillo, M.A., Iribar, V., Ruiz, M. (1997). Groundwater pollution in Quaternary aquifer of Vitoria–Gasteiz (Basque Country, Spain) Influence of agricultural activities and water-resource management. Environmental Geology, 30, 257-265.	-	-	Approximated from Fig. 1 by Arrate et al. (1997)
**Volcanic Rift Zone**	Eastern Snake River Plain	Whitehead, R. L. (1992). Geohydrologic framework of the Snake River Plain regional aquifer system, Idaho and eastern Oregon. US Geological Survey Professional Paper 1408-B, 39 pp. Accessed March 1, 2021 via https://pubs.usgs.gov/pp/1408b/report.pdf	-	-	Approximated from Plate 1 of Whitehead (1992)
**Wadi As-Sirhan Basin**	-	Othman, A., Sultan, M., Becker, R., Alsefry, S., Alharbi, T., Gebremichael, E., Alharbi, H. and Abdelmohsen, K., (2018).	Souid, F., Birkle, P., Worrall, F. (2019). Water-rock interaction of the Jilh and Tawil aquifers in the Wadi Sirhan Basin, NW	-	Approximated from Fig. 1 by Othman et al. (2018) and Fig. 1 by Souid et al. (2019)

Aquifer system	Broader aquifer system	Reference A	Reference B	Reference C	Steps to delineate
		Use of geophysical and remote sensing data for assessment of aquifer depletion and related land deformation. Surveys in Geophysics, 39, 543-566.	Saudi Arabia. In E3S Web of Conferences (Vol. 98, p. 01047). EDP Sciences.		
**Wah Wah Valley**	-	Lopes, T.J., Evetts. D.M. (2005). Ground-Water pumpage and artificial recharge estimates for calendar year 2000 and average annual natural recharge and interbasin flow by hydrographic area, Nevada. Accessed April 25, 2022 via https://pubs.usgs.gov/sir/2004/5239/sir2004-5239.pdf	Harrill, J.R., Prudic, D.E. (1998). Aquifer systems in the Great Basin region of Nevada, Utah, and adjacent states; summary report. US Geological Survey Professional Paper 1409-A, 75 pp. Accessed April 26, 2022 via https://pubs.usgs.gov/pp/1409a/report.pdf	-	Approximated from Fig. 1 by Lopes and Evetts (2005), and Fig. 2 by Harrill and Prudic (1998). Well completion records, physiography and drainage were used to delinate areas.
**Waipara Alluvial Basin**	-	Loris, P. (2000). Hydrogeology of the Waipara Alluvial Basin. MSc Thesis, Univeristy of Canterbury, 223 pp. Accessed October 20, 2021 from https://core.ac.uk/download/pdf/35469119.pdf	-	-	Approximated from Fig. 1.1 by Loris (2000)
**Walker Basin Creek Valley**	-	California Department of Water Resources (2015c). California's groundwater update 2013 - Chapter 9: Tulare Lake Hydrologic Region. California Department of Water Resources Report, 80 pp. Accessed April 27, 2022 via https://data.cnra.ca.gov/dataset/california-water-plan-groundwater-update-2013/resource/8a4ae915-	-	-	Approximated from Fig. 9-2 by California Department of Water Resources (2015c)

Aquifer system	Broader aquifer system	Reference A	Reference B	Reference C	Steps to delineate
		b786-42e1-9abe-99a8fcc23349			
**Walla Walla Basin**	Columbia Plateau Regional Aquifer System	Kahle, S.C., Morgan, D.S., Welch, W.B., Ely, S.R., Vaccaro, J.J., Orzol, L.L. (2011). Hydrogeologic Framework and Hydrologic Budget Components of the Columbia Plateau Regional Aquifer System, Washington, Oregon, and Idaho. US Geological Survey Scientific Investigations Report 2011–5124, 80 pp. Accessed February 16, 2021 from https://pubs.usgs.gov/sir/2011/5124/pdf/sir20115124.pdf	Henry, R., Lindsay, K., Wolcott, B., Patten, S., Baker, T. (2013). Walla Walla Basin Aquifer Recharge Strategic Plan. Walla Walla Basin Watershed Council Report, 106 pp. Accessed February 23, 2021 from http://wwbwc.org/images/Projects/AR/Reports/RechargeStrategy_FINAL_1-29-13_sp.pdf	Newcomb, R.C. (1965). Geology and ground-water resources of the Walla Walla River Basin, Washington-Oregon: Washington Division of Water Resources Water-Supply Bulletin 21, 162 pp. Accessed November 29, 2021 from https://apps.ecology.wa.gov/publications/documents/wsb21.pdfComment created	Walla Walla Basin outline approximated from Fig. 1 of Walla Walla Basin Watershed Council Report by Henry et al. (2013) and Fig. 1 by Newcomb (1965). Columbia River Aquifer System approximated from Figs. 1 and 10 of Kahle et al. (2011).
**Warner Valley**	-	California Department of Water Resources (2015e). California's groundwater update 2013 - Chapter 6: South Coast Hydrologic Region. California Department of Water Resources Report, 84 pp. Accessed April 27, 2022 via https://cawaterlibrary.net/wp-content/uploads/2017/05/GWU2013_Ch6_SouthCoast_Final.pdf	-	-	Approximated from Fig. 6-2 by California Department of Water Resources (2015e)
**Weaber Plain**	-	Paul, B., Raper, P., Simons, J., Stainer, G., George, R. (2011). Weaber Plain aquifer test results. Government of Western Australia, Department of Agriculture and Food	-	-	Approximated from Fig. 1 by Paul et al. (2011)

Aquifer system	Broader aquifer system	Reference A	Reference B	Reference C	Steps to delineate
		Resource Management Technical Report 367, 48 pp. Accessed May 8, 2023 via https://library.dpird.wa.gov.au/cgi/viewcontent.cgi?article=1362&context=rmtr			
**Weipa Depression and Northern Carpentaria Basin**	Great Artesian Basin	Ransley, T.R., Smerdon, B.D. (2012). Hydrostratigraphy, hydrogeology and system conceptualisation of the Great Artesian Basin. A technical report to the Australian Government from the CSIRO Great Artesian Basin Water Resource Assessment, 324 pp. Accessed July 20, 2021 from https://publications.csiro.au/rpr/download?pid=csiro:EP132693&dsid=DS5	-	-	Approximated from Fig. 2.4 by Ransley and Smerdon (2012)
**Weiser River Valley**	-	Graham, W.G., Campbell, L.J. (1981). Groundwater resources of Idaho. Idaho Department of Water Resources Report, 61 pp. Accessed March 23, 2021 from https://idwr.idaho.gov/files/publications/198108-MISC-GW-Resources-ID.pdf	-	-	Approximated from Plate 1 of Graham and Campbell (1981).
**West Coast Basin**	Los Angeles Basin**	Land, M., Reichard, E.G., Crawford, S.M., Everett, R.R., Newhouse, M.W., Williams, C.F. (2004). Ground-water Quality of Coastal Aquifer Systems in the West Coast Basin, Los Angeles County, California,	Fram, M. S., Belitz, K. (2008). Groundwater quality in the Coastal Los Angeles Basin, California; United States Geological Survey Fact Sheet 2012-3096. Accessed February 14, 2021 from	Reichard, E. G., Land, M., Crawford, S.M., Johnson, T.D., Everett, R.R., Kulshan, T.V., Ponti, D.J., Halford, K.L., Johnson, T.A., Paybins, K.S., Nishikawa, T. (2003). Geohydrology,	Approximated from Fig. 1 by Land et al. (2004), Fig. 6 by Reichard et al. (2003) and map on page 1 of Fram and Belitz (2008)

Aquifer system	Broader aquifer system	Reference A	Reference B	Reference C	Steps to delineate
		1999–2002. U.S. Geological Survey Scientific Investigations Report 2004-5067, 88 pp. Accessed February 15, 2021 from https://pubs.usgs.gov/sir/2004/5067/sir2004-5067.pdf	https://pubs.er.usgs.gov/publication/70039952.	Geochemistry, and Ground-Water Simulation Optimization of the Central and West Coast Basins, Los Angeles County, California. USGS Numbered Series, 2003–4065. Accessed November 28, 2021 from https://pubs.usgs.gov/wri/wrir034065/wrir034065.pdf	
**West Jutland Quaternary and Tertiary Sand Aquifer System**	-	Frei, R., Frei, K.M., Kristiansen, S.M., Jessen, S., Schullehner, J., Hansen, B. (2020). The link between surface water and groundwater-based drinking water−strontium isotope spatial distribution patterns and their relationships to Danish sediments. Applied Geochemistry, 121, 104698.	-	-	Approximated from Figs. 1 and 5 by Frei et al. (2020) and drainage patterns
**West Salt River Basin**	Salt Basin	Flora, S., Davis, T. (2009). Hydrologic Map Series (HMS), Water Level Change Map Series (WLCMS), and Basin Sweep Assessment Report ADWR Basins and Sub-Basins. Arizona Department of Water Resources Hydrology Division Field Services Section https://new.azwater.gov/sites/default/files/HMSWLCMSBasinSweepAssessmentReport2009.pdf	Anning, D. (2014). Conceptual Understanding and Groundwater Quality of Selected Basin-Fill Aquifers in the Southwestern United States: Section 7, Conceptual Understanding and Groundwater Quality of the Basin-Fill Aquifer in the West Salt River Valley, Arizona. U.S. Geological Survey Professional Paper 1781, 24 pp. Accessed March 31, 2021 from https://pubs.usgs.gov/pp/1781/pdf/pp1781_section7.pdf	-	Approximated from Fig. 1 of Flora and Davis (2009). Hydrologic Map Series (HMS), Water Level Change Map Series (WLCMS), and Basin Sweep Assessment Report ADWR Basins and Sub-Basins. Arizona Department of Water Resources Hydrology Division Field Services Section https://new.azwater.gov/sites/default/files/HMSWLCMSBasinSweepAssessmentReport2009.pdf. See also: Anning (2014).

Aquifer system	Broader aquifer system	Reference A	Reference B	Reference C	Steps to delineate
**West Zealand Quaternary Sand Aquifer System**	-	Frei, R., Frei, K.M., Kristiansen, S.M., Jessen, S., Schullehner, J., Hansen, B. (2020). The link between surface water and groundwater-based drinking water–strontium isotope spatial distribution patterns and their relationships to Danish sediments. Applied Geochemistry, 121, 104698.	-	-	Approximated from Figs. 1 and 5 by Frei et al. (2020) and drainage patterns
**West-central Kanto Plain**	Kanto Plain	Furuno, K., Kagawa, A., Kazaoka, O., Kusuda, T., Nirei, H. (2015). Groundwater management based on monitoring of land subsidence and groundwater levels in the Kanto Groundwater Basin, Central Japan. Proceedings of the International Association of Hydrological Sciences, 372, 53-57.	Tanaka, T. (2004). Groundwater resources, development and management in the Kanto Plain, Japan. In Satellite Symposium, 5 pp. Accessed April 14, 2023 via https://core.ac.uk/download/pdf/76125416.pdf	Miyakoshi, A., Uchida, Y., Sakura, Y., Hayashi, T. (2003). Distribution of subsurface temperature in the Kanto Plain, Japan; estimation of regional groundwater flow system and surface warming. Physics and Chemistry of the Earth, Parts A/B/C, 28, 467-475.	Approximated from Fig. 2 by Furuno et al. (2015), Fig. 1 by Tanaka (2004), and Fig. 1 by Miyakoshi et al. (2003) and physiography and drainage patterns
**Western Allegheny Plateau**	Appalachian Plateaus	Brockman, S. (1998). Physiographic regions of Ohio. Ohio Department of Natural Resources, Division of Geological Survey map. Accessed April 12, 20221 from https://www.epa.state.oh.us/portals/27/SIP/Nonattain/F2-physiographic_regions_of_Ohio.pdf	Trapp Jr., H., Horn, M.A. (1997). Ground water atlas of the United States: Segment 11. U.S. Geological Survey Hydrologic Investigations Atlas 730-L, 26 pp. Accessed April 13, 2021 from https://pubs.usgs.gov/ha/730l/report.pdf	-	Approximated from map by Brockman (1998). Southern boundary approximated along the Hackman River. Broader Appalachian Plateau Aquifer System approximated from Fig. 3 of Trapp Jr. and Horn (1997)
**Western Amarkantak Plateau**	Amarkantak Plateau	Deolankar, S. B. (1980). The Deccan basalts of Maharashtra, India—their potential as aquifers.	Kale, V. S., Bodas, M., Chatterjee, P., Pande, K. (2020). Emplacement history and evolution of the	Duraiswami, R. A., Das, S., Shaikh, T. (2012). Hydrogeological framework of aquifers from the Deccan	Approximated from Fig. 1 by Deolankar (1980), Fig. 1 by Duraiswami et al. (2012), and Fig. 1 by Kale et al. (2016). Subarea

Aquifer system	Broader aquifer system	Reference A	Reference B	Reference C	Steps to delineate
		Groundwater, 18(5), 434-437.	Deccan Volcanic Province, India. Episodes, 43, 278-299.	Traps, India: some insights. Memoir Geological Society of India, 1-15.	based on topography and drainage patterns.
**Western Armorican Massif**	Armorican Massif	Rouillard, J., Maréchal, J.-C. (2020). Groundwater in France: Resources, Use and Management Issues. In: Sustainable Groundwater Management A Comparative Analysis of French and Australian Policies and Implications to Other Countries, pp. 17-45. Accessed May 22, 2021 from https://hal.archives-ouvertes.fr/hal-02512154/document	Roques, C., Bour, O., Aquilina, L., Dewandel, B. (2016). High-yielding aquifers in crystalline basement: insights about the role of fault zones, exemplified by Armorican Massif, France. Hydrogeology Journal, 24, 2157-2170.	Vergnes, J.P., Roux, N., Habets, F., Ackerer, P., Amraoui, N., Besson, F., Caballero, Y., Courtois, Q., Dreuzy, J.R.D., Etchevers, P., Gallois, N. (2020). The AquiFR hydrometeorological modelling platform as a tool for improving groundwater resource monitoring over France: evaluation over a 60-year period. Hydrology and Earth System Sciences, 24, 633-654.	Approximated from Fig. 1 by Rouillard and Maréchal (2020), Fig. 3 by Vergnes et al. (2020), and Fig. 1 by Roques et al. (2016).
**Western Bengal Basin**	Bengal Basin	Mukherjee, A., Fryar, A. E., Howell, P. D. (2007). Regional hydrostratigraphy and groundwater flow modeling in the arsenic-affected areas of the western Bengal basin, West Bengal, India. Hydrogeology Journal, 15, 1397-1418.	Ravenscroft, P., McArthur, J. M., Rahman, M. S. (2018). Identifying multiple deep aquifers in the Bengal Basin: Implications for resource management. Hydrological Processes, 32, 3615-3632.	Michael, H. A., Voss, C. I. (2009). Controls on groundwater flow in the Bengal Basin of India and Bangladesh: regional modeling analysis. Hydrogeology Journal, 17, 1561-1577.	Approximated from Fig. 2 by Mukherjee et al. (2007), Fig. 1 by Ravenscroft et al. (2018), and Fig. 1 by Michael and Voss (2009)
**Western Blackwood Plateau**	-	Ali, R., McFarlane, D., Varma, S., Dawes, W., Emelyanova, I., Hodgson, G., Charles, S. (2012). Potential climate change impacts on groundwater resources of south-western Australia. Journal of Hydrology, 475, 456-472.	-	-	Approximated from Fig. 1b by Ali et al. (2012)
**Western Brahmaputra Basin Aquifer**	Brahmaputra Basin Aquifer	Mukherjee, A., Saha, D., Harvey, C. F., Taylor, R. G., Ahmed, K. M., Bhanja, S. N. (2015). Groundwater systems of the Indian sub-	-	-	Approximated from Fig. 3 by Mukherjee et al. (2015)

Aquifer system	Broader aquifer system	Reference A	Reference B	Reference C	Steps to delineate
		continent. Journal of Hydrology: Regional Studies, 4, 1-14.			
**Western C Aquifer**	The C Aquifer	Brown, C.R., Macy, J.P. (2012). Groundwater, Surface-Water, and Water-Chemistry Data from the C-Aquifer Monitoring Program, Northeastern Arizona, 2005–2011. U.S. Geological Survey Open-File Report 2012–1196, 46 pp. Accessed April 15, 2021 from https://pubs.usgs.gov/of/2012/1196/of2012-1196.pdf	-	-	Approximated from Fig. 1 of Brown and Macy (2012). East and west portions divided near Joseph City.
**Western Cambay Basin**	Cambay Basin	Kumar, M. D., Ghosh, S., Patel, A., Singh, O. P., Ravindranath, R. (2006). Rainwater harvesting in India: some critical issues for basin planning and research. Land Use and Water Resources Research, 6, 1-17.	Deshpande, R.D. (2006). Groundwater in and Around Cambay Basin, Gujarat: Some Geochemical andIsotopic Investigations, Physical Research Laboratory, Ahmedabad, PhD Thesis. Accessed July 12, 2021 from https://shodhganga.inflibnet.ac.in/handle/10603/58203	Gupta, S. K., Deshpande, R. D. (2003). Origin of groundwater helium and temperature anomalies in the Cambay region of Gujarat, India. Chemical Geology, 198, 33-46.	Approximated from Fig. 6 by Kumar et al. (2006), Fig. 4.1 by Deshpande (2006), and Fig. 1 by Gupta and Deshpande (2003). West, Central and East subareas defined by two identified faults (see Fig. 4.1 by Deshpande (2006))
**Western Cambrian-Ordovician Aquifers**	Northern Midwest Aquifer System	Young, H.L. (1992). Hydrogeology of the Cambrian-Ordovician Aquifer System in the Northern Midwest, United States. U.S. Geological Survey Professional Paper 1405-B, 108 pp. Accessed April 12, 2021 from https://pubs.usgs.gov/pp/1405b/report.pdf	Wilson, J.T. (2012). Water-quality assessment of the Cambrian-Ordovician aquifer system in the northern Midwest, United States. U.S. Geological Survey Scientific Investigations Report 2011–5229, 174 pp. Accessed April 12, 2021 from https://pubs.usgs.gov/sir/2	-	Broader aquifer system title and its location approximated from Fig. 1 of Young (1992). Subcrop geology approximated from Fig. 9 of Wilson (2012). Southern margin extended south of the subcrop margin on the basis of the presence of deep wells.

Aquifer system	Broader aquifer system	Reference A	Reference B	Reference C	Steps to delineate
			011/5229/pdf/SIR2011522 9_web.pdf		
**Western Cape Breton**	Maritimes Basin	Stapinsky, M. Michaud, Y., Morin, R.H., Butler, K.E., Deblonde, C., Chi, G, Thériault, T., Castonguay, S., Boisvert, V., Julien, H.P., Conohan, D., Hulsman, B., Carrier, C. Marion, J., Boisvert, É. (2002). Groundwater resources assessment in the Carboniferous Maritimes Basin: preliminary results of the hydrogeological characterization, New Brunswick, Nova Scotia, and Prince Edward Island. Geological Survey of Canada Current Research Report 2002-D8, 14 pp. Accessed May 5, 2021 from http://www.gov.pe.ca/photos/original/cle_WA10.pdf	Rivard, C., Michaud, Y., Lefebvre, R., Deblonde, C., Rivera, A. (2008). Characterization of a regional aquifer system in the Maritimes Basin, Eastern Canada. Water Resources Management, 22, 1649-1675.	Bianco, E. (2013). Seismic Interpretation of the Windsor-Kennetcook Basin. Geological Survey of Canada Open File 7452, 31 pp. Accessed May 5, 2021 from https://ftp.maps.canada.ca/pub/nrcan_rncan/publications/STPublications_PublicationsST/292/292763/of_7452.pdf	Approximated from Fig. 2 of Rivard et al. (2008) and Figs. 1 and 3 of Stapinsky et al. (2002). Area bounds approximated from Fig. 1 of Bianco (2013).
**Western Carrizo-Wilcox**	Carrizo-Wilcox	Huang, Y., Scanlon, B.R., Nicot, J.P., Reedy, R.C., Dutton, A.R., Kelley, V.A., Deeds, N E. (2012). Sources of groundwater pumpage in a layered aquifer system in the Upper Gulf Coastal Plain, USA. Hydrogeology Journal, 20(4), 783-796.	Kelley, V.A., Deeds, N.E., Fryar, D.G., Nicot, J.P. (2004). Groundwater availability models for the Queen City and Sparta aquifers. Contract report to the Texas Water Development Board, Austin, TX, 867 pp. Accessed February 24, 2022 from https://www.twdb.texas.gov/groundwater/models/gam/qcsp/QCSP_Model_Report.pdf?d=29484	-	Approximated from Fig. 2.2 by Kelley et al. (2004). Texas portion approximated from Fig. 1 of Huang et al. (2012), with the eastern boundary approximated by the division between Texas' 12th and 13th Groundwater Management Area (GMA; accessed February 11, 2021 from https://www.twdb.texas.gov/groundwater/management_areas/gma12.asp). The Mexico portion of the aquifer was approximated based on well depth data.

Aquifer system	Broader aquifer system	Reference A	Reference B	Reference C	Steps to delineate
**Western Champlain Valley Lowlands**	-	Nystrom, E.A. (2006). Ground-water quality in the Lake Champlain Basin, New York, 2004. U.S. Geological Survey Open-File Report 2006-1088, 22 pp. Accessed April 15, 2021 from https://pubs.usgs.gov/of/2006/1088/pdf/Nystrom.OFR2006-1088.pdf	Scott, T.-M., Nystrom, E.A., Reddy, J.E. (2016). Groundwater quality in the Lake Champlain and Susquehanna River basins, New York, 2014. U.S. Geological Survey Open-File Report 2016–1153, 33 pp. Accessed April 15, 2021 from https://pubs.usgs.gov/of/2016/1153/ofr20161153.pdf	-	Approximated from Fig. 2 of Nystrom (2006) and Fig. 1 by Scott et al. (2016).
**Western Chikugo-Saga Plain**	Chikugo-Saga Plain	Wang, L., Iwao, Y. (2000). Groundwater characteristics of the Saga Plain, Japan. Journal of Nepal Geological Society, 22, 343-350.	Sakai, A. (2001). Land subsidence due to seasonal pumping of groundwater in Saga Plain, Japan. Lowland Technology International, 3(1, June), 25-40. Accessed April 13, 2023 via https://cot.unhas.ac.id/journals/index.php/ialt_lti/article/view/287	Cai, Y., Esaki, T., Liu, S., Mitani, Y. (2014). Effect of substitute water projects on tempo-spatial distribution of groundwater withdrawals in Chikugo-Saga plain, Japan. Water Resources Management, 28, 4645-4663.	Approximated from Fig. 1 by Wang and Iwao (2000), Fig. 1 by Sakai (2001), and Fig. 1 by Cai et al. (2014)
**Western Dehshir Basin**	-	Hosseini, S. M., Parizi, E., Ataie-Ashtiani, B., Simmons, C. T. (2019). Assessment of sustainable groundwater resources management using integrated environmental index: Case studies across Iran. Science of the Total Environment, 676, 792-810.	-	-	Approximated from Fig. 6 by Hosseini et al. (2019); divide between western and eastern sub basins approximated near (southwest of) کمال آباد
**Western Doon Valley**	-	Jasrotia, A. S., Kumar, A., Aasim, M. (2011). Morphometric analysis and hydrogeomorphology for delineating groundwater potential zones of Western Doon Valley, Uttarakhand, India. International Journal	-	-	Approximated from Fig. 1 by Jasrotia et al. (2011)

Aquifer system	Broader aquifer system	Reference A	Reference B	Reference C	Steps to delineate
		of Geomatics and Geosciences, 2, 1078-1096.			
**Western El Palmar Aquifer**	-	CONAGUA (2021a). Acuíferos (nacional). Webpage accessed March 8, 2022 from http://sina.conagua.gob.mx/sina/tema.php?tema=acuiferos&ver=mapa&o=1&n=nacional	-	-	Approximated from aquifer boundaries defined by CONAGUA (2021a) web portal, physiography and drainage patterns, and well completion data. Western and Eastern areas of El Palmar divided on the basis of physiography and drainage.
**Western Elsinore Valley**	-	California Department of Water Resources (2015e). California's groundwater update 2013 - Chapter 6: South Coast Hydrologic Region. California Department of Water Resources Report, 84 pp. Accessed April 27, 2022 via https://cawaterlibrary.net/wp-content/uploads/2017/05/GWU2013_Ch6_SouthCoast_Final.pdf	-	-	Approximated from Fig. 6-2 by California Department of Water Resources (2015e)
**Western Fakhrabad Basin**	-	Parizi, E., Hosseini, S. M., Ataie-Ashtiani, B., Simmons, C. T. (2020). Normalized difference vegetation index as the dominant predicting factor of groundwater recharge in phreatic aquifers: case studies across Iran. Scientific Reports, 10, 17473.	-	-	Approximated from tabulated coordinates in the Supplementary Information within Parizi et al. (2020) and physiographic and drainage patterns
**Western Fomanat Plain**	-	Gholami, V.C.K.W., Chau, K.W., Fadaee, F., Torkaman, J., Ghaffari, A. (2015). Modeling of groundwater level fluctuations using	-	-	Approximated from Fig. 1 by Gholami et al. (2015)

Aquifer system	Broader aquifer system	Reference A	Reference B	Reference C	Steps to delineate
		dendrochronology in alluvial aquifers. Journal of Hydrology, 529, 1060-1069.			
**Western Galilee Basin**	-	Rostkier-Edelstein, D., Liu, Y., Wu, W., Kunin, P., Givati, A., Ge, M. (2014). Towards a high-resolution climatography of seasonal precipitation over Israel. International Journal of Climatology, 34, 1964-1979.	-	-	Approximated from Fig. 1 by Rostkier-Edelstein et al. (2014) and local physiography and drainage patterns
**Western Huaihe Plain**	Huaihe River Basin	Sun, X., Lin, J., Gu, W., Min, X., Han, J., Dai, Y., Liu, P., Wang, S (2021). Analysis and evaluation of the renewability of the deep groundwater in the Huaihe River Basin, China. Environmental Earth Sciences, 80, 1-13.	Liu J., Zheng C. (2016) Towards Integrated Groundwater Management in China. In: Integrated Groundwater Management (eds. Jakeman A.J., Barreteau O., Hunt R.J., Rinaudo J.D., Ross A.). Springer, Cham. pp. 455-475	Li, Y., Wang, D., Liu, Y., Zheng, Q., Sun, G. (2017). A predictive risk model of groundwater arsenic contamination in China applied to the Huai River Basin, with a focus on the region's cluster of elevated cancer mortalities. Applied Geochemistry, 77, 178-183.	Approximated from Fig. 1 by Sun et al. (2021), Fig. 1 by Li et al. (2017), and Fig. 18.5 by Liu and Zheng (2016)
**Western Kachchh Coastal Plain**	Kachchh Coastal Plain	Saha, D., Gor, N. (2020). A prolific aquifer system is in peril in arid Kachchh region of India. Groundwater for Sustainable Development, 11, 100394.	Bhimani, S.A. (2013). Study on Groundwater Salinization and Formulation of Management Strategies for the Coastal Aquifers of Mundra Region, Kutch District, Gujarat State. Ph.D. Thesis, The Maharaja Sayajirao University of Baroda. 380 pp. Accessed July 5, 2021 from http://okgw.hmlibrary.ac.in:8080/jspui/handle/1/457	University of Greenwich and Gujarat Institute of Desert Ecology (2016). Ecosystem Assessment of the Coastal Plain Natural Area of Kachchh District: Planning for Biodiversity and Livelihoods into the Future. Project Presentation, 70 pp. Accessed July 5, 2021 from https://gala.gre.ac.uk/id/eprint/16221/1/16221%20BARTLETT_Coastal_Plain_of_Kachchh_2016.pdf	Approximated from Fig. 1 by Saha and Gor (2020) and Fig. 3.2 and descriptions of hydrogeologic setting in Chapter III by Bhimani (2013). Extent of broader aquifer system (region) approximated following the "Natural Character Area map" (page 4) by University of Greenwich and Gujarat Institute of Desert Ecology (2016)
**Western Kankakee Plains**	-	Fenneman, N.M., Johnson, D.W. (1946). Physiographic divisions of the	Leighton, M. M., Ekblaw, G. E., Horberg, L. (1948). Physiographic divisions of	-	Broader Till Plain physiographic region approximated from Fenneman and Johnson (1946).

Aquifer system	Broader aquifer system	Reference A	Reference B	Reference C	Steps to delineate
		conterminous United States. U.S. Geological Survey map, 1:7,000,000 Scale.	Illinois. The Journal of Geology, 56, 16-33.		Kankakee Plain in Illinois approximated from Figs. 1 and 2 of Leighton et al. (1948). Eastern margin of subarea approximated along the Iroquois River.
**Western Karnataka Laterites**	-	Central Ground Water Board (2012c). Aquifer Systems of Karnataka. Central Ground Water Board Report, 75 pp. Accessed June 30, 2021 from http://cgwb.gov.in/AQM/Karnataka.pdf	-	-	Approximated from Plate III by Central Ground Water Board (2012c)
**Western Karnataka Schist Aquifers**	-	Central Ground Water Board (2012c). Aquifer Systems of Karnataka. Central Ground Water Board Report, 75 pp. Accessed June 30, 2021 from http://cgwb.gov.in/AQM/Karnataka.pdf	Central Ground Water Board (2008a). Ground water information booklet Haveri District, Karnataka. Central Ground Water Board Report, 21 pp. Accessed July 3, 2021 from http://cgwb.gov.in/District_Profile/karnataka/Haveri_brochure.pdf	Central Ground Water Board (2008b). Ground water information booklet Dharwad District, Karnataka. Central Ground Water Board Report, 18 pp. Accessed July 3, 2021 from http://cgwb.gov.in/District_Profile/karnataka/DHARWAD_BROCHURE.pdf	Approximated from Fig. 3 by Central Ground Water Board (2008a), Fig. 3 by Central Ground Water Board (2008b), and Plate III by Central Ground Water Board (2012c). Division between Northwestern and Western Karnataka Schist Aquifers approximated on the basis of drainage and topography data
**Western Karoo Basin**	Main Karoo Basin	Nyambe, I. A. (1999). Tectonic and climatic controls on sedimentation during deposition of the Sinakumbe Group and Karoo Supergroup, in the mid-Zambezi Valley Basin, southern Zambia. Journal of African Earth Sciences, 28, 443-463.	-	-	Broader aquifer system boundary (i.e., Main Karoo Basin) approximated from Fig. 1 by Nyambe (1999). Physiography (drainage patterns, geology) and well construction data were considered while digitizing subarea
**Western Karstic Plain**	Yucatan Peninsula	Bauer-Gottwein, P., Gondwe, B. R., Charvet, G., Marín, L. E., Rebolledo-Vieyra, M., Merediz-Alonso, G. (2011). the Yucatán Peninsula karst aquifer,	Munro-Stasiuk, M.J., Manahan, T. K. (2010). Investigating ancient Maya agricultural adaptation through ground penetrating radar (GPR) analysis ofkarst terrain, Northern	-	Approximated from Fig. 2 by Bauer-Gottwein et al. (2011), and from Fig. 2 by Munro-Stasiuk and Manahan (2010). Approximated by the area south of the Chicxulub Sedimentary Basin but within the Yucatan Coastal Plain.

Aquifer system	Broader aquifer system	Reference A	Reference B	Reference C	Steps to delineate
		Mexico. Hydrogeology Journal, 19, 507-524.	Yucatán, Mexico. Acta Carsologica, 39, 123-36.		
**Western Kome Basin**	Kome Basin	Parizi, E., Hosseini, S. M., Ataie-Ashtiani, B., Simmons, C. T. (2020). Normalized difference vegetation index as the dominant predicting factor of groundwater recharge in phreatic aquifers: case studies across Iran. Scientific Reports, 10, 17473.	-	-	Approximated from tabulated coordinates in the Supplementary Information within Parizi et al. (2020) and physiographic and drainage patterns
**Western La Sabana Aquifer**	-	CONAGUA (2021a). Acuíferos (nacional). Webpage accessed March 8, 2022 from http://sina.conagua.gob.mx/sina/tema.php?tema=acuiferos&ver=mapa&o=1&n=nacional	-	-	Approximated from aquifer boundaries defined by CONAGUA (2021a) web portal, physiography and drainage patterns, and well completion data. Western and eastern areas divided on the basis of drainage (i.e., divided at Laguna Tres Palos)
**Western Liaohe River Basin**	Liaohe River Basin	Zhong, Y., Zhong, M., Feng, W., Zhang, Z., Shen, Y., Wu, D. (2018). Groundwater depletion in the West Liaohe River Basin, China and its implications revealed by GRACE and in situ measurements. Remote Sensing, 10, 493.	-	-	Approximated from Fig. 1 by Zhong et al. (2018)
**Western Lordegan Basin**	Lordegan Basin	Parizi, E., Hosseini, S. M., Ataie-Ashtiani, B., Simmons, C. T. (2020). Normalized difference vegetation index as the dominant predicting factor of groundwater recharge in phreatic aquifers: case studies across Iran.	-	-	Approximated from tabulated coordinates in the Supplementary Information within Parizi et al. (2020) and physiographic and drainage patterns

Aquifer system	Broader aquifer system	Reference A	Reference B	Reference C	Steps to delineate
		Scientific Reports, 10, 17473.			
**Western Mississippi Embayment**	Mississippi Embayment	Arthur, J.K., Taylor, R.E. (1998). Ground-water flow analysis of the Mississippi embayment aquifer system, South-Central United States. US Geological Survey Professional Paper 1416-1, 59 pp. Accessed February 10, 2021 from https://pubs.usgs.gov/pp/1416i/report.pdf	Renken, R.A. (1998). Groundwater Atlas of the United States: Arkansas, Louisiana, Mississippi (HA-730-F). US Geological Survey Hydrologic Investigations Atlas 730-F. 30 pp. Accessed November 28, 2021 fromhttps://pubs.usgs.gov/ha/730f/report.pdf	-	Approximated from Fig. 2 of Arthur and Taylor (1998) and Figs. 8-10 and Fig. 65 by Renken (1998)
**Western Nakambe Basin**	Baoule-Mossi Domain of the southern West African Craton	Courtois, N., Lachassagne, P., Wyns, R., Blanchin, R., Bougaïré, F. D., Somé, S., Tapsoba, A. (2010). Large-scale mapping of hard-rock aquifer properties applied to Burkina Faso. Groundwater, 48, 269-283.	Grenholm, O. H. M. (2019). The geodynamic evolution of a Paleoproterozoic orogenic system-a local to global perspective on the ca. 2.27-1.96 Ga Birimian Orogen in the Baoule Mossi domain of West Africa. University of Western Australia Thesis. Accessed May 28, 2023 via https://research-repository.uwa.edu.au/en/publications/the-geodynamic-evolution-of-a-paleoproterozoic-orogenic-system-a-	Yangouliba, G.I., Zoungrana, B.J.B., Hackman, K.O., Koch, H., Liersch, S., Sintondji, L.O., Dipama, J.M., Kwawuvi, D., Ouedraogo, V., Yabré, S., Bonkoungou, B., Sougué, M., Gadiaga, A., Koffi, B. (2022). Modelling past and future land use and land cover dynamics in the Nakambe River Basin, West Africa. Modeling Earth Systems and Environment, 9, 1651-1667.	Approximated via descriptions and Fig. 1 by Courtois et al. (2010) and Fig. 1 in Grenhold (2019). Subarea delineated via local drainage patterns and Fig. 1 by Yangouliba et al. (2009)
**Western Neyshabur Plain**	Neyshabur Plain	Bhunia, G. S., Keshavarzi, A., Shit, P. K., Omran, E. S. E., Bagherzadeh, A. (2018). Evaluation of groundwater quality and its suitability for drinking and irrigation using GIS and geostatistics techniques in semiarid region of Neyshabur, Iran. Applied Water Science, 8, 1-16.	Khalili Naft Chali, A., Shahidi, A. (2017). Comparison of Lazy Algorithms and M5 Model to Estimate Groundwater Level (Case Study: Plain Neyshabur). JWSS-Isfahan University of Technology, 21, 15-26. Accessed June 2, 2021 from	Izady, A., Davary, K., Alizadeh, A., Ghahraman, B., Sadeghi, M., Moghaddamnia, A. (2012). Application of "panel-data" modeling to predict groundwater levels in the Neishaboor Plain, Iran. Hydrogeology Journal, 20, 435-447.	Approximated from Fig. 1 by Bhunia et al. (2018), Fig. 1 by Izady et al. (2012), and Fig. 1 by Khalili Naft Chali and Shahidi (2017)

Aquifer system	Broader aquifer system	Reference A	Reference B	Reference C	Steps to delineate
			https://jstnar.iut.ac.ir/article-1-3296-en.html		
**Western Ouarzazate Basin**	-	Steuer, A., Helwig, S. L., Tezkan, B. (2008). Aquifer characterization in the Ouarzazate Basin (Morocco): A contribution by TEM and RMT data. Near Surface Geophysics, 6, 5-14.	-	-	Approximated from Fig. 1 by Steuer et al. (2008)
**Western Outer Bluegrass**	Interior Low Plateaus	Miller, J.A. (1995). Ground Water Atlas of the United States: Segment 10, Illinois, Indiana, Kentucky, Ohio, Tennessee. U.S. Geological Survey Hydrologic Atlas 730-K, 30 pp. Accessed April 11, 2021 from https://pubs.usgs.gov/ha/730k/report.pdf	Taylor, C.J., Nelson Jr., H.L. (2008). A compilation of provisional karst geospatial data for the Interior Low Plateaus physiographic region, central United States: U.S. Geological Survey Data Series 339, 26 pp. Accessed April 11, 2021 from https://pubs.usgs.gov/ds/339/pdf/ds339_web.pdf	Davidson, B. (2018). Kentucky Interagency Groundwater Monitoring Network: Annual Report July 2017–June 2018. 28 pp. Accessed April 11, 2021 from http://www.uky.edu/KGS/water/gnet/itac17-18.pdf	Broader Interior Low Plateaus outline approximated for Tennessee, Illinois, Kentucky, Indiana from Fig. 78 of Miller (1990) and Fig. 1 of Taylor and Nelson (2008). Outer Bluegrass approximated from Fig. 1 of Davidson (2018).
**Western Parecis Basin**	Parecis Basin	Fontes, S. L., Meju, M. A., Maurya, V. P., La Terra, E. F., Miquelutti, L. G. (2019). Deep structure of Parecis Basin, Brazil from 3D magnetotelluric imaging. Journal of South American Earth Sciences, 96, 102381.	-	-	Approximated from Fig. 1 by Fontes et al. (2019).
**Western Paris Basin**	Paris Basin	Beaudoin, N., Gasparrini, M., David, M. E., Lacombe, O., Koehn, D. (2019). Bedding-parallel stylolites as a tool to unravel maximum burial depth in sedimentary basins: Application to Middle Jurassic carbonate reservoirs in the Paris	Contoux, C., Violette, S., Vivona, R., Goblet, P., Patriarche, D. (2013). How basin model results enable the study of multi-layer aquifer response to pumping: the Paris Basin, France. Hydrogeology Journal, 21, 545-557.	Beccaletto, L., Hanot, F., Serrano, O., Marc, S. (2011). Overview of the subsurface structural pattern of the Paris Basin (France): Insights from the reprocessing and interpretation of regional seismic lines. Marine and	Approximated from Fig. 1 by Beaudoin et al. (2019), Fig. 1 by Contoux et al. (2013), and Fig. 1 by Beccaletto et al. (2011)

Aquifer system	Broader aquifer system	Reference A	Reference B	Reference C	Steps to delineate
		basin, France. GSA Bulletin, 131, 1239-1254.		Petroleum Geology, 28, 861-879.	
**Western Pilbara Granite-Greenstone Terrane**	Pilbara Craton	Rojas, R., Commander, P., McFarlane, D., Ali, R., Dawes, W., Barron, O., Hodgson, G., Charles, S.(2018). Groundwater resource assessment and conceptualization in the Pilbara Region, Western Australia. Earth Systems and Environment, 2(2), 345-365.	Simonson, B. M., Schubel, K. A., Hassler, S. W. (1993). Carbonate sedimentology of the early Precambrian Hamersley Group of western Australia. Precambrian Research, 60, 287-335.	-	Approximated from Fig. 3 by Rojas et al. (2018) and Fig. 1 by Simonson et al. (1993). Eastern limit delineated on the basis of the Yule River.
**Western Port Basin**	-	Lee, S. (2015). Investigating the origin and dynamics of salinity in a confined aquifer system in southeast Australia (Western Port Basin). BSc Thesis, RMIT, 314 pp. Accessed July 27, 2021 from https://ap-st01.ext.exlibrisgroup.com/61RMIT_INST/storage/alma/CD/C7/50/9F/4A/9C/21/2B/19/98/C6/81/6B/E1/E9/76/Lee.pdf?Expires=1627421927&Signature=HW4117JHX2bzhK-LggVzjnSxSYLrOqMYIhzEE~JkeUIU1QJ-VBQHWGb462M7NI8QE6b8QW31V~ieixKm1t1JlPuyb~SCNBXaxcwE3Tro3oO3UYvfpwdnIDhBNxW93Y9x1h93R5-FKTrm~B9ya8kuV1hyNQjtJ-1ZoiCcqEj1cHyBUDd9et8RSPSfxLdJKERxHPKsuFLVe98ULaWo9DCXnjUiITmTaNKY03b4TK8hCBRc5RBVq7UhTwa	Lee, S., Currell, M., Cendón, D. I. (2016). Marine water from mid-Holocene sea level highstand trapped in a coastal aquifer: evidence from groundwater isotopes, and environmental significance. Science of the Total Environment, 544, 995-1007.	Currell, M., Cendón, D. I., Cheng, X. (2013). Analysis of environmental isotopes in groundwater to understand the response of a vulnerable coastal aquifer to pumping: Western Port Basin, south-eastern Australia. Hydrogeology Journal, 21, 1413-1427.	Approximated from Fig. 9 by Lee (2015), Fig. 1 by Lee et al. (2016), and Fig. 1 by Currell et al. (2013)

Aquifer system	Broader aquifer system	Reference A	Reference B	Reference C	Steps to delineate
		K~nvFVMBhCDLYhv8S-nUt7R~KoBr4W0MGO7qKEbXigNDf3zsYmDEci5s5x5PGQHZx1NM6VmL-zzj1aUHHakZebW1uM1MSZw__&Key-Pair-Id=APKAJ72OZCZ36VGVASIA			
**Western Prince Edward Island**	Prince Edward Island	Stapinsky, M. Michaud, Y., Morin, R.H., Butler, K.E., Deblonde, C., Chi, G, Thériault, T., Castonguay, S., Boisvert, V., Julien, H.P., Conohan, D., Hulsman, B., Carrier, C. Marion, J., Boisvert, É. (2002). Groundwater resources assessment in the Carboniferous Maritimes Basin: preliminary results of the hydrogeological characterization, New Brunswick, Nova Scotia, and Prince Edward Island. Geological Survey of Canada Current Research Report 2002-D8, 14 pp. Accessed May 5, 2021 from http://www.gov.pe.ca/photos/original/cle_WA10.pdf	-	-	Approximated from Figs. 1 and 3 of Stapinsky et al. (2002).
**Western Qazvin Plain**	Qazvin Plain	Yazdi, Z., Niroumand, H. (2020). Assessing land subsidence in Qazvin plain caused by groundwater level drop, using finite elements and finite difference methods. GeoTerrace-2020-043, 5 pp. Accessed June 2, 2021 from	Faghihi, N., Kave, F., Babazadeh, H. (2010). Prediction of aquifer reaction to different hydrological and management scenarios using visual MODFLOW model-Case study of Qazvin plain. Journal of Water	Babaee, S., Mousavi, Z., Masoumi, Z., Malekshah, A. H., Roostaei, M., Aflaki, M. (2020). Land subsidence from interferometric SAR and groundwater patterns in the Qazvin plain, Iran. International Journal of Remote Sensing, 41, 4780-4798.	Approximated from Fig. 1 by Yazdi and Niroumand (2020), with zones approximated from Fig. 7 by Faghihi et al. (2010)

Aquifer system	Broader aquifer system	Reference A	Reference B	Reference C	Steps to delineate
		https://eage.in.ua/wp-content/uploads/2020/12/GeoTerrace-2020-043.pdf	Sciences Research 2, 39-45.		
**Western Quibas and Serral-Salinas Aquifers**	-	Custodio, E., Andreu-Rodes, J.M., Aragón, R., Estrela, T., Ferrer, J., García-Aróstegui, J.L., Manzano, M., Rodríguez-Hernández, L., Sahuquillo, A., Del Villar, A. (2016). Groundwater intensive use and mining in south-eastern peninsular Spain: Hydrogeological, economic and social aspects. Science of the Total Environment, 559, 302-316.	-	-	Approximated from Fig. 1 by Custodio et al. (2016)
**Western Rezvan Basin**	-	Hosseini, S. M., Parizi, E., Ataie-Ashtiani, B., Simmons, C. T. (2019). Assessment of sustainable groundwater resources management using integrated environmental index: Case studies across Iran. Science of the Total Environment, 676, 792-810.	-	-	Approximated from Fig. 6 by Hosseini et al. (2019)
**Western Riverine Plains**	Murray Basin	Herczeg, A. L., Dogramaci, S. S., Leaney, F. W. J. (2001). Origin of dissolved salts in a large, semi-arid groundwater system: Murray Basin, Australia. Marine and Freshwater Research, 52, 41-52.	Barnett, S., Simmons, C. T., Nelson, R. (2021). Groundwater resources in Australia—their occurrence, management, and future challenges. In: Global Groundwater Source, Scarcity, Sustainability, Security, and Solutions (eds. Mukherjee, A., Scanlon, B.R., Aureli, A., Langan, S., Guo, H.,	Murray-Darling Basin Commission (1999). Murray-Darling Basin Groundwater - a resource for the future, Murray-Darling Basin Commission, Canberra. Accessed July 21, 2021 from https://www.mdba.gov.au/sites/default/files/archived/mdbc-GW-reports/2173_GW_a_resource_for_the_future.pdf	Approximated from Fig. 1 by Herczeg et al. (2001), Fig. 3.2 by Barnett et al. (2021), and Fig. 6 by Murray-Darling Basin Commission (1999)

Aquifer system	Broader aquifer system	Reference A	Reference B	Reference C	Steps to delineate
			McKenzie, A.), Elsevier, pp. 35-46.		
**Western Saldanah and Langebaan Lagoon Aquifer System**	-	Campbell, E. E., Parker-Nance, T., Bate, G. C. (1992). A compilation of information on the magnitude, nature and importance of coastal aquifers in Southern Africa. Water Research Commission Report No. 370/1/92, 193 pp. Accessed October 14, 2021 from http://www.wrc.org.za/wp-content/uploads/mdocs/370-1-92.pdf	Smith, K. (2020). Assessing the hydrogeologic characteristics and sources of groundwater recharge and flow in the Elandsfontein aquifer, West Coast, Western Cape, South Africa, University of the Western Cape, MSc Thesis, 122 pp. Accessed October 14, 2021 from http://etd.uwc.ac.za/xmlui/bitstream/handle/11394/7724/smith_m_nsc_2020.pdf?sequence=1&isAllowed=y	-	Appoximated from Fig. 6.6 by Smith (2020) and Fig. 3 by Campbell et al. (1992)
**Western Salvadoran Coastal Plain**	Salvadoran Coastal Plain	Marshall, J.S. (2007). The geomorphology and physiographic provinces of Central America. Central America: geology, resources and hazards, 1, 75-121.	US Army Corps of Engineers (1998). Water resources assessment of El Salvador. 71 pp. Accessed February 7, 2023 via https://www.sam.usace.army.mil/Portals/46/docs/military/engineering/docs/WRA/ElSalvador/El%20Salvador%20WRA%20English.pdf	Ministerio de Medio Ambiente y Recursos Natural (2023). Mapa Hidrogeológico de El Salvador. Atlas accessed February 7, 2023 via http://srt.snet.gob.sv/sihi/public/atlas	Approximated from Fig. 3.5 by Marshall (2007) and Fig. A-2 within US Army Corps of Engineers (1998) and atlas by Ministerio de Medio Ambiente y Recursos Natural (2023)
**Western San Gabriel Valley**	San Gabriel Valley	California Department of Water Resources (2015e). California's groundwater update 2013 - Chapter 6: South Coast Hydrologic Region. California Department of Water Resources Report, 84 pp. Accessed April 27, 2022 via https://cawaterlibrary.net/wp-content/uploads/2017/05/GWU2013_Ch6_SouthCoast_Final.pdf	-	-	Approximated from Fig. 6-2 by California Department of Water Resources (2015e). Western and eastern subareas delineated by the San Gabriel River

Aquifer system	Broader aquifer system	Reference A	Reference B	Reference C	Steps to delineate
**Western Satpura Basalt Aquifers**	Satpura Basalts	Deolankar, S. B. (1980). The Deccan basalts of Maharashtra, India—their potential as aquifers. Groundwater, 18(5), 434-437.	Kale, V. S., Bodas, M., Chatterjee, P., Pande, K. (2020). Emplacement history and evolution of the Deccan Volcanic Province, India. Episodes, 43, 278-299.	Duraiswami, R. A., Das, S., Shaikh, T. (2012). Hydrogeological framework of aquifers from the Deccan Traps, India: some insights. Memoir Geological Society of India, 1-15.	Approximated from Fig. 1 by Deolankar (1980), Fig. 1 by Duraiswami et al. (2012), and Fig. 1 by Kale et al. (2016).
**Western Second Songhuajiang System**	Songnen Plain	Chen, Z., Wei, W., Liu, J., Wang, Y., Chen, J. (2011). Identifying the recharge sources and age of groundwater in the Songnen Plain (Northeast China) using environmental isotopes. Hydrogeology Journal, 19, 163-176.	Zhang, G., Deng, W., Yang, Y. S., Salama, R. B. (2007). Evolution study of a regional groundwater system using hydrochemistry and stable isotopes in Songnen Plain, northeast China. Hydrological Processes: An International Journal, 21, 1055-1065.	Zhang, B., Song, X.-F., Zhang, Y.-H., Han, D.-M., Tand, C.Y., Yang, L.-H., Wang, Z. L. (2017). The renewability and quality of shallow groundwater in Sanjiang and Songnen Plain, Northeast China. Journal ofIntegrative Agriculture, 16, 229-238.	Approximated from Fig. 1 by Chen et al. (2011), Fig. 1 by Zhang et al. (2007) and Fig. 1 by Zhang et al. (2017)
**Western St Vincent Basin**	St Vincent Basin	Smith, M. L., Fontaine, K., Lewis, S. J. (2015). Regional Hydrogeological Characterisation of the St Vincent Basin, South Australia: Technical Report for the National Collaboration Framework Regional Hydrogeology Project. Geoscience Australia Record 2015/16, 150 pp. Accessed July 27, 2021 from https://d28rz98at9flks.cloudfront.net/78884/Rec2015_016.pdf	-	-	Approximated from Fig. 2.2 by Smith et al. (2015)
**Western Surat Basin**	Great Artesian Basin	Ransley, T.R., Radke, B.M., Feitz, A.J., Kellett, J.R., Owens, R., Bell, J. and Stewart, G. (2015). Hydrogeological Atlas of the Great Artesian Basin, Geoscience Australia.	Ransley, T.R., Smerdon, B.D. (2012). Hydrostratigraphy, hydrogeology and system conceptualisation of the Great Artesian Basin. A technical report to the	Smerdon, B.D., Ramsley, T.R. (2012). Water Resource Assessment for the Surat Region. A technical report to the Australian Government from the CSIRO Great	Approximated from Map 1 on page 9 of report by Ransley et al. (2015), and Fig. 2.3 by Ransley and Smerdon (2012). Subarea within Surat Basin delineated on the basis of physiographic features (e.g., major rivers), elevations of

Aquifer system	Broader aquifer system	Reference A	Reference B	Reference C	Steps to delineate
		Canberra, 141 pp. Accessed July 19, 2021 from http://www.ga.gov.au/scientific-topics/water/groundwater/gab	Australian Government from the CSIRO Great Artesian Basin Water Resource Assessment, 324 pp. Accessed July 20, 2021 from https://publications.csiro.au/rpr/download?pid=csiro:EP132693&dsid=DS5	Artesian Basin Water Resource Assessment, 142 pp. Accessed July 20, 2021 from https://publications.csiro.au/rpr/download?pid=csiro:EP132644&dsid=DS4	the base of the Great Artesian Basin (Fig. 5.2 by Smerdon and Ransley (2012b)) and topography.
**Western Sutter Basin**	Sacramento Valley of the Central Valley **	California Department of Water Resources (2015i). California's groundwater update 2013 - Chapter 7: Sacramento River Hydrologic Region. California Department of Water Resources Report, 126 pp. Accessed April 27, 2022 via https://water.ca.gov/-/media/DWR-Website/Web-Pages/Programs/Groundwater-Management/Bulletin-118/Files/Statewide-Reports/GWU2013_Ch7_SacramentoRiver_Final.pdf	-	-	Approximated from Fig. 7-2 by California Department of Water Resources (2015i). Eastern and Western sub areas delineated along the Sutter By-pass
**Western Tapi Valley Alluvial Aquifer**	Tapi Valley Alluvial Aquifer	Gupta, G., Erram, V. C., Kumar, S. (2012). Temporal geoelectric behaviour of dyke aquifers in northern Deccan Volcanic Province, India. Journal of Earth System Science, 121, 723-732.	Chatterjee, S., Biswal, B. P., Sinha, U. K., Patbhaje, S. D. (2021). Isotope-geochemical assessment of thermal waters and their impact on surrounding potable water resources in the Tapi valley geothermal area, Maharashtra, India. Environmental Earth Sciences, 80, 1-15.	Foster, S., Garduño, H., Tuinhof, A. (2007). Confronting the groundwater management. Challenge in the Deccan Traps Country of Maharashtra–India. World Bank Case Profile Collection Number 18, Washington D.C. 19 pp.	Approximated from Fig. 2 by Gupta et al. (2012), descriptions in section "Hydrogeology" and Fig. 2 by Chatterjee et al. (2021) and Fig. 1 by Foster et al. (2007)
**Western Torbat-Zaveh Plain**	Torbat-Zaveh Plain	Nematollahi, M. J., Ebrahimi, P., Razmara, M., Ghasemi, A. (2016). Hydrogeochemical	-	-	Approximated from Fig. 1 by Nematollahi et al. (2015)

Aquifer system	Broader aquifer system	Reference A	Reference B	Reference C	Steps to delineate
		investigations and groundwater quality assessment of Torbat-Zaveh plain, Khorasan Razavi, Iran. Environmental Monitoring and Assessment, 188, 1-21.			
**Western Uinta Basin**	Uinta Basin	Hood, J. W. (1976). Characteristics of aquifers in the northern Uinta Basin area, Utah and Colorado. U.S. Geological Survey and Utah Department of Natural Resources, Division of Water Rights Technical Publication 53, 71 pp. Accessed March 7, 2021 from https://waterrights.utah.gov/docSys/v920/w920/w920009f.pdf	-	-	Northern Uinta Basin approximated from Plates 1-3 of Hood (1976).
**Western Villa Garcia Aquifer**	-	CONAGUA (2020j). Actualización de la disponibilidad media anual de agua en el acuífero Villa Garcia (3213), Estado de Zacatecas. 29 pp. Accessed January 24, 2022 via https://sigagis.conagua.gob.mx/gas1/Edos_Acuiferos_18/zacatecas/DR_3213.pdf	-	-	Approximated from Fig. 2 by CONAGUA (2020j); western and eastern areas delineated o the basis of physicography and drainage
**Western Washoe Valley**	-	Lopes, T.J., Evetts. D.M. (2005). Ground-Water pumpage and artificial recharge estimates for calendar year 2000 and average annual natural recharge and interbasin flow by hydrographic area, Nevada. Accessed April 25, 2022 via	Harrill, J.R., Prudic, D.E. (1998). Aquifer systems in the Great Basin region of Nevada, Utah, and adjacent states; summary report. US Geological Survey Professional Paper 1409-A, 75 pp. Accessed April 26, 2022 via	-	Approximated from Fig. 1 by Lopes and Evetts (2005), and Fig. 2 by Harrill and Prudic (1998). Well completion records, physiography and drainage were used to delinate areas.

Aquifer system	Broader aquifer system	Reference A	Reference B	Reference C	Steps to delineate
		https://pubs.usgs.gov/sir/2004/5239/sir2004-5239.pdf	https://pubs.usgs.gov/pp/1409a/report.pdf		
**Western Zayandeh-Rud Basin**	-	Hosseini, S. M., Parizi, E., Ataie-Ashtiani, B., Simmons, C. T. (2019). Assessment of sustainable groundwater resources management using integrated environmental index: Case studies across Iran. Science of the Total Environment, 676, 792-810.	Madani, K., Mariño, M. A. (2009). System dynamics analysis for managing Iran’s Zayandeh-Rud river basin. Water Resources Management, 23, 2163-2187.	Salemi, H.R., Mamanpoush, A.R., Miranzadeh, M., Akbari, M., Torabi, M., Toomanian, N., Murray-Rust, H., Droogers, P., Sally, H., Gieske, A. (2000). Water management for sustainable irrigated agriculture in the Zayandeh Rud Basin, Esfahan Province, Iran Report by Iranian Agricultural Engineering Research Institute, Esfahan Agricultural Research Center, and the International Water Management Institute, Research Report Number 1	Approximated from Fig. 6 by Hosseini et al. (2019), Fig. 1 by Madani and Mariño (2009), and Fig. 1 by Salemi et al. (2000)
**Westside Basin**	Tulare Basin of the Central Valley **	California Department of Water Resources (2015c). California's groundwater update 2013 - Chapter 9: Tulare Lake Hydrologic Region. California Department of Water Resources Report, 80 pp. Accessed April 27, 2022 via https://data.cnra.ca.gov/dataset/california-water-plan-groundwater-update-2013/resource/8a4ae915-b786-42e1-9abe-99a8fcc23349	-	-	Approximated from Fig. 9-2 by California Department of Water Resources (2015c)
**Westside Middle Indus Plain**	Indus Basin	Bonsor, H. C., MacDonald, A. M., Ahmed, K. M., Burgess, W. G., Basharat, M., Calow, R. C., Dixit, A., Foster, S.S.D., Gopal, K.,	Srivastava, M., Poonia, O.P. (2010). Transboundary Aquifers In Rajasthan, Issues & Management. Bhujal News, 25, 28-36.	-	Middle Indus area approximated from southwestern portion of highlighted aquifer boundary in Fig. 18 by Bonsor et al. (2017). Subarea approximated from Fig.

Aquifer system	Broader aquifer system	Reference A	Reference B	Reference C	Steps to delineate
		Lapworth, D.J., Moench, M., Mukherjee, A., Rao, M.S., Shamsudduha, M., Smith, L., Taylor, R.G., Tucker, J., van Steenbergen, F., Yadav, S.K., Zahid, A. (2017). Hydrogeological typologies of the Indo-Gangetic basin alluvial aquifer, South Asia. Hydrogeology Journal, 25, 1377-1406.	Accessed July 12, 2021 from http://cgwb.gov.in/Documents/Bhujalnews-25-01-02.pdf#page=33		2a by Srivastava and Poonia (2010)
**Wet Mountain Valley**	-	Londquist, C. J., Livingston, R.K. (1978). Water-resources appraisal of the Wet Mountain Valley, in parts of Custer and Fremont Counties, Colorado. Water-Resources Investigations 78-1, 62 pp. Accessed March 27, 2021 from https://pubs.usgs.gov/wri/1978/0001/report.pdf	-	-	Approximated from Fig. 3 of Londquist and Livingston (1978)
**White River Valley**	-	Maxey, G. B., Eakin, T. E. (1949). Ground water in White River Valley, White Pine, Nye, and Lincoln Counties, Nevada. U.S. Department of the Interior Water Resources Bulletin 8, 64 pp. Accessed March 10, 2021 from https://www.nrc.gov/docs/ML0331/ML033140348.pdf	-	-	Approximated from maps on page 63 and 64 of Maxey and Eakin (1949)
**White Wolf Basin**	Tulare Basin of the Central Valley **	California Department of Water Resources (2015c). California's groundwater update 2013 - Chapter 9: Tulare Lake Hydrologic Region. California	-	-	Approximated from Fig. 9-2 by California Department of Water Resources (2015c)

Aquifer system	Broader aquifer system	Reference A	Reference B	Reference C	Steps to delineate
		Department of Water Resources Report, 80 pp. Accessed April 27, 2022 via https://data.cnra.ca.gov/dataset/california-water-plan-groundwater-update-2013/resource/8a4ae915-b786-42e1-9abe-99a8fcc23349			
**Wiggertal Valley**	-	Hoffman, S., Hunkeler, D., Maurer, M. (2014). Approvisionnement en eau et assainissement des eaux usées durables en Suisse: défis et mesures possibles. Synthèse thématique 3 dans le cadre du Programme national de recherche PNR 61, Gestion durable de l'eau, Berne, Switzerland, 88 pp. Accessed April 23, 2023 via https://media.snf.ch/rWjOZoYQfS9iabW/nfp61_thematische_synthese_3_f.pdf	-	-	Approximated from map on pp. 27 by Hoffman et al. (2014)
**Wild Horse and Michigan Flats**	Salt Basin Bolson	Beach, J.A., Ashworth, J.B., Finch, S.T., Chaistain-Howley, A., Calhoun, K., Urbanczyk, K.M., Sharp, J.M., Olsonm J. (2004a). Groundwater availability model for the igneous and parts of the West Texas Bolsons (Wild Horse Flat, Michigan Flat, Ryan Flat and Lobo Flat) aquifers. Texas Water Development Board Report, 407 pp. Accessed May 1, 2023 via https://www.twdb.texas.gov/groundwater/models/gam	-	-	Approximated from Fig. 2.1.3 by Beach et al. (2004a)

Aquifer system	Broader aquifer system	Reference A	Reference B	Reference C	Steps to delineate
		/igbl/IGBL_Model_Report.pdf			
**Willchra Basin**	-	Magarey, P., Deane, D. (2005). Willochra Basin Groundwater Monitoring Status Report 2005. Department of Water, Land and Biodiversity Conservation Report No. 2005/39, 50 pp. Accessed May 7, 2023 via https://www.waterconnect.sa.gov.au/Content/Publications/DEW/ki_dwlbc_2005_39.pdf	-	-	Approximated from Magarey and Deane (2005)
**Willcox-Douglas Basin**	-	Arizona Department of Water Resources (2018). Groundwater Flow Model of the Willcox Basin, Arizona Department of Water Resources Report, 196 pp. Accessed on February 12, 2021 from https://new.azwater.gov/sites/default/files/Willcox_Report_2018.pdf	Flora, S., Davis, T. (2009). Hydrologic Map Series (HMS), Water Level Change Map Series (WLCMS), and Basin Sweep Assessment Report ADWR Basins and Sub-Basins. Arizona Department of Water Resources Hydrology Division Field Services Section https://new.azwater.gov/sites/default/files/HMSWLCMSBasinSweepAssessmentReport2009.pdf	-	Approximated from Arizona Department of Water Resources (2018). This report states (quote AZ DWR): "..the Willcox Basin is a closed basin that is in direct hydraulic connection with the Aravaipa and Douglas basins to the north and south, respectively." And thus the Aravaipa and Douglas Basins of Flora and Davis (2009) were included in the delineated boundary for the Willcox Basin.
**Williston Basin**	Northern Great Plains	Thamke, J.N., LeCain, G.D., Ryter, D.W., Sando, R., Long, A.J. (2014). Hydrogeologic framework of the uppermost principal aquifer systems in the Williston and Powder River structural basins, United States and Canada. US Geological Survey Scientific Investigations Report 2014-	Long, A.J., Thamke, J.N., Davis, K.W., Bartos, T.T. (2018). Groundwater availability of the Williston Basin, United States and Canada: U.S. Geological Survey Professional Paper 1841, 54 pp. Accessed November 29, 2021 from https://pubs.usgs.gov/pp/1841/pp1841.pdf	-	Approximated from Fig. 1 of Long et al. (2018) and Fig. 1 by Thamke et al. (2014); "Miles City arch" boundary used to distinguish Williston and Powder River structural basins

Aquifer system	Broader aquifer system	Reference A	Reference B	Reference C	Steps to delineate
		5047, 50 pp. Accessed February 15, 2021 from https://pubs.usgs.gov/sir/2014/5047/pdf/sir2014-5047.pdf			
**Willow Creek Basin**	-	California Department of Water Resources (2015i). California's groundwater update 2013 - Chapter 7: Sacramento River Hydrologic Region. California Department of Water Resources Report, 126 pp. Accessed April 27, 2022 via https://water.ca.gov/-/media/DWR-Website/Web-Pages/Programs/Groundwater-Management/Bulletin-118/Files/Statewide-Reports/GWU2013_Ch7_SacramentoRiver_Final.pdf	-	-	Approximated from Fig. 7-2 by California Department of Water Resources (2015i)
**Willunga and Noarlunga Embayments**	St Vincent Basin	Smith, M. L., Fontaine, K., Lewis, S. J. (2015). Regional Hydrogeological Characterisation of the St Vincent Basin, South Australia: Technical Report for the National Collaboration Framework Regional Hydrogeology Project. Geoscience Australia Record 2015/16, 150 pp. Accessed July 27, 2021 from https://d28rz98at9flks.cloudfront.net/78884/Rec2015_016.pdf	-	-	Approximated from Fig. 2.2 by Smith et al. (2015)
**Wimmera Plain**	Murray Basin	Herczeg, A. L., Dogramaci, S. S., Leaney, F. W. J. (2001). Origin of dissolved	Cook, P. G., Jolly, I. D., Leaney, F. W. J. (2001). Groundwater recharge in	Murray-Darling Basin Commission (1999). Murray-Darling Basin	Approximated from Fig. 1 by Herczeg et al. (2001), Fig. 1.1 by Cook et al. (2001), and Fig. 6 by

Aquifer system	Broader aquifer system	Reference A	Reference B	Reference C	Steps to delineate
		salts in a large, semi-arid groundwater system: Murray Basin, Australia. Marine and Freshwater Research, 52, 41-52.	the Mallee region, and salinity implications for the Murray River: A review. CSIRO Land and Water Report, 133 pp. Accessed July 21, 2021 from https://publications.csiro.au/rpr/download?pid=procite:ef08494d-43a2-4dae-bda4-3d72a62e673f&dsid=DS1	Groundwater - a resource for the future, Murray-Darling Basin Commission, Canberra. Accessed July 21, 2021 from https://www.mdba.gov.au/sites/default/files/archived/mdbc-GW-reports/2173_GW_a_resource_for_the_future.pdf	Murray-Darling Basin Commission (1999)
**Wind River Basin West**	Wind River Basin	Whitcomb, H.A., Lowry, M.E. (1968). Ground-water resources and geology of the Wind River Basin area, central Wyoming. U.S. Geological Survey Hydrologic Atlas 270, 14 pp. Accessed March 29, 2021 from https://pubs.usgs.gov/ha/270/report.pdf	-	-	Approximated from Plate 1 of Whitcomb and Lowry (1968). East-west basin divide approximated south of Boysen Reservoir
**Winfield-Wood Lake and Kalamalka Lake Aquifers**	Okanagan Basin	Golder Associates and Summit Environmental Consultants Ltd. (2009). Phase 2 Okanagan Water Supply and Demand Project: Groundwater Objectives 2 and 3 Basin Study.Report to Okanagan Basin Water Board, 114 pp. Accessed March 16, 2021 from https://www.obwb.ca/wsd/about/project-reports	-	-	Approximated from description on pages 37 and 38 of report by Golder Associates and Summit Environmental Consultants Ltd. (2009) and Fig. 5
**Winkler Alluvial Aquifer**	-	Render, F.W. (1987). Aquifer capacity investigations 1980-1986. Manitoba Water Resources Hydrotechnical Services Report, 162 pp. Accessed	-	-	Approximated from map on page 135 of Render (1987).

Aquifer system	Broader aquifer system	Reference A	Reference B	Reference C	Steps to delineate
		May 5, 2021 from https://www.gov.mb.ca/water/pubs/water-science-management/groundwater/publication/1987_render_aquifer_capacity_investigations_1980_1986.pdf			
**Wisconsin Precambrian Aquifer**	-	U.S. Geological Survey (1984). National water summary 1984: Hydrologic events, selected water-quality trends, and ground-water resources. U.S. Geological Survey Water Supply Paper 2275, 477 pp. Accessed April 13, 2021 from https://pubs.usgs.gov/wsp/2275/report.pdf	-	-	Approximated from Fig. 1 of the "National Water Summary-Wisconsin" (page 449) by the U.S. Geological Survey (1984).
**Wiso Basin**	Daly-Wiso-Georgina Basins	Crosbie, R. S., Rachakonda, P. K. (2021). Constraining probabilistic chloride mass-balance recharge estimates using baseflow and remotely sensed evapotranspiration: the Cambrian Limestone Aquifer in northern Australia. Hydrogeology Journal, 29, 1399-1419.	Wilkes, P. (2018). Baseline assessment of groundwater characteristics in the Beetaloo Sub-basin, NT. GISERA Project Order, 27 pp. Accessed October 6, 2021 from https://gisera.csiro.au/wp-content/uploads/2018/10/Water-16-Project-Order-1.pdf	de Caritat, P., Bastrakov, E.N., Jaireth, S., English, P.M., Clarke, J.D.A., Mernagh, T.P., Wygralak, A.S., Dulfer, H.E., Trafford, J. (2019). Groundwater geochemistry, hydrogeology and potash mineral potential of the Lake Woods region, Northern Territory, Australia. Australian Journal of Earth Sciences, 66, 411-430.	Approximated from Fig. 1 by Crosbie and Rachakonda (2021), Fig. 1 by Wilkes (2018), and Fig. 2 by de Caritat et al. (2019)
**Wood River Valley**	-	Graham, W.G., Campbell, L.J. (1981). Groundwater resources of Idaho. Idaho Department of Water Resources Report, 61 pp. Accessed March 23, 2021 from https://idwr.idaho.gov/files	-	-	Approximated from Plate 1 of Graham and Campbell (1981).

Aquifer system	Broader aquifer system	Reference A	Reference B	Reference C	Steps to delineate
		/publications/198108-MISC-GW-Resources-ID.pdf			
**Wuhai Basin**	-	Liang, K., Sun, C., Ma, B., Tian, Q., Li, D., Li, D., He, Z. (2018). Investigation of the Yellow River buried fault in the Wuhai basin, northwestern Ordos Block, China, using deep/shallow seismic reflection and drilling techniques. Journal of Asian Earth Sciences, 163, 54-69.	-	-	Approximated from Fig. 1 by Liang et al. (2018)
**Wuwei Basin**	Shiyang Basin	Zhu, G. F., Li, Z. Z., Su, Y. H., Ma, J. Z., Zhang, Y. Y. (2007). Hydrogeochemical and isotope evidence of groundwater evolution and recharge in Minqin Basin, Northwest China. Journal of Hydrology, 333, 239-251.	-	-	Approximated from Fig. 1 by Zhu et al. (2007)
**Wuyuer-Shuangyang System**	Songnen Plain	Chen, Z., Wei, W., Liu, J., Wang, Y., Chen, J. (2011). Identifying the recharge sources and age of groundwater in the Songnen Plain (Northeast China) using environmental isotopes. Hydrogeology Journal, 19, 163-176.	Zhang, G., Deng, W., Yang, Y. S., Salama, R. B. (2007). Evolution study of a regional groundwater system using hydrochemistry and stable isotopes in Songnen Plain, northeast China. Hydrological Processes: An International Journal, 21, 1055-1065.	Zhang, B., Song, X.-F., Zhang, Y.-H., Han, D.-M., Tand, C.Y., Yang, L.-H., Wang, Z. L. (2017). The renewability and quality of shallow groundwater in Sanjiang and Songnen Plain, Northeast China. Journal ofIntegrative Agriculture, 16, 229-238.	Approximated from Fig. 1 by Chen et al. (2011), Fig. 1 by Zhang et al. (2007) and Fig. 1 by Zhang et al. (2017)
**Wyandotte Creek Basin**	Sacramento Valley of the Central Valley **	California Department of Water Resources (2015i). California's groundwater update 2013 - Chapter 7: Sacramento River Hydrologic Region. California Department of Water Resources Report, 126 pp. Accessed April 27,	-	-	Approximated from Fig. 7-2 by California Department of Water Resources (2015i)

Aquifer system	Broader aquifer system	Reference A	Reference B	Reference C	Steps to delineate
		2022 via https://water.ca.gov/-/media/DWR-Website/Web-Pages/Programs/Groundwater-Management/Bulletin-118/Files/Statewide-Reports/GWU2013_Ch7_SacramentoRiver_Final.pdf			
**Xian Depression**	Weihe Basin	Zhang, W., Li, Y., Zhao, F., Han, W., Li, Y., Wang, Y., Holland, G. Zhou, Z. (2019). Using noble gases to trace groundwater evolution and assess helium accumulation in Weihe Basin, central China. Geochimica et Cosmochimica Acta, 251, 229-246.	-	-	Approximated from Fig. 1 by Zhang et al. (2019)
**Xinachuan Area**	Huangshui Basin	Li, H., Zhan, R., Lu, Y., Zhou, B., Wu, J. (2021). Spatiotemporal variation and periodic evolution characteristics of groundwater in the Xining area of China, eastern Qinghai–Tibet Plateau. Environmental Earth Sciences, 80, 1-19.	-	-	Approximated from Fig. 1b and Fig. 1d by Li et al. (2021)
**Xinzo de Limia Aquifer**	-	Garzon-Vidueira, R., Rial-Otero, R., Garcia-Nocelo, M.L., Rivas-Gonzalez, E., Moure-Gonzalez, D., Fompedriña-Roca, D., Vadillo-Santos, I., Simal-Gandara, J. (2020). Identification of nitrates origin in Limia river basin and pollution-determinant factors. Agriculture,	-	-	Approximated from Fig. 1 by Garzon-Vidueira et al. (2020)

Aquifer system	Broader aquifer system	Reference A	Reference B	Reference C	Steps to delineate
		Ecosystems & Environment, 290, 106775.			
**Yakima Basin**	Columbia Plateau Regional Aquifer System	Burns, E.R., Snyder, D.T., Haynes, J.V., Waibel, M.S. (2012). Groundwater status and trends for the Columbia Plateau Regional Aquifer System, Washington, Oregon, and Idaho. U.S. Geological Survey Scientific Investigations Report 2012–5261, 52 pp. Accessed February 18, 2021 from http://pubs.er.usgs.gov/publication/sir20125261.	Kahle, S.C., Morgan, D.S., Welch, W.B., Ely, S.R., Vaccaro, J.J., Orzol, L.L. (2011). Hydrogeologic Framework and Hydrologic Budget Components of the Columbia Plateau Regional Aquifer System, Washington, Oregon, and Idaho. US Geological Survey Scientific Investigations Report 2011–5124, 80 pp. Accessed February 16, 2021 from https://pubs.usgs.gov/sir/2011/5124/pdf/sir20115124.pdf	Reidel, S.P., Spane, F.A., Johnson, V.G. (2002). Natural gas storage in basalt aquifers of the Columbia basin, Pacific Northwest USA: A guide to site characterization. Pacific Northwest National Lab (PNNL) Report n No. PNNL-13962. Richland, Washington, USA, 277 pp. Accessed February 16, 2021 from https://www.pnnl.gov/main/publications/external/technical_reports/PNNL-13962.pdf	Yakima Basin approximated based on Fig. 7 of Burns et al. (2012). Broader Columbia River Aquifer System approximated from Figs. 1 and 10 of Kahle et al. (2011) and sub-province map shown in Fig. 2.1 of Reidel et al. (2002).
**Yangtze Delta at Shanghai**	-	Xu, Y. S., Shen, S. L., Ma, L., Sun, W. J., Yin, Z. Y. (2014). Evaluation of the blocking effect of retaining walls on groundwater seepage in aquifers with different insertion depths. Engineering Geology, 183, 254-264.	Luo, C. Y., Shen, S. L., Han, J., Ye, G. L., Horpibulsuk, S. (2015). Hydrogeochemical environment of aquifer groundwater in Shanghai and potential hazards to underground infrastructures. Natural Hazards, 78, 753-774.	Zhang, Y., Wu, J., Xue, Y., Wang, Z., Yao, Y., Yan, X., Wang, H. (2015). Land subsidence and uplift due to long-term groundwater extraction and artificial recharge in Shanghai, China. Hydrogeology Journal, 23, 1851-1866.	Approximated from Fig. 1 by Zhu et al. (2014), Fig. 2 by Luo et al. (2015) and Fig. 1 by Zhang et al. (2015).
**Yaqui Valley**	-	Vega-Granillo, E.L., Cirett-Galán, S, De la Parra-Velasco, M.L., Zavala-Juárez, R. (2011). Hidrogeología de Sonora, México. in: Calmus, Thierry, ed., Panorama de la geología de Sonora, México, Universidad Nacional Autónoma de México, Instituto de Geología,	-	-	Approximated from Fig. 5 of Vega-Granillo et al. (2011).

Aquifer system	Broader aquifer system	Reference A	Reference B	Reference C	Steps to delineate
		Boletín 118, 267–298. Accessed May 6, 2021 from			
**Yasuj Basin**	-	Hosseini, S. M., Parizi, E., Ataie-Ashtiani, B., Simmons, C. T. (2019). Assessment of sustainable groundwater resources management using integrated environmental index: Case studies across Iran. Science of the Total Environment, 676, 792-810.	-	-	Approximated from Fig. 6 by Hosseini et al. (2019)
**Yazd-Ardakan Basin**	-	Eslamizadeh, A., Samanirad, S. (2010). Land subsidence and fissuring due to ground water withdrawal in Yazd-Ardakan basin, central Iran. World Academy of Science, Engineering and Technology, 48, 489-492.	Hekmatnia, H., Barzegari Banadkooki, F., Moosavi, V., Zare Chahouki, A. (2021). Evaluation of groundwater suitability for drinking, irrigation, and industrial purposes (Case study: Yazd-Ardakan Aquifer, Yazd Province, Iran). ECOPERSIA, 9, 11-21.	-	Approximated from Fig. 8 by Eslamizadeh and Samanirad (2010) and Fig. 1 by Hekmatnia et al. (2021)
**Yellow River Alluvial Plain**	North China Plain	Meng, S., Liu, J., Zhang, Z., Lei, T., Qian, Y., Li, Y., Fei, Y. (2015). Spatiotemporal evolution characteristics study on the precipitation infiltration recharge over the past 50 years in the North China Plain. Journal of Earth Science, 26, 416-424.	Cao, G., Zheng, C., Scanlon, B. R., Liu, J., & Li, W. (2013). Use of flow modeling to assess sustainability of groundwater resources in the North China Plain. Water Resources Research, 49, 159-175.	Wang, S., Song, X., Wang, Q., Xiao, G., Liu, C., Liu, J. (2009). Shallow groundwater dynamics in North China plain. Journal of Geographical Sciences, 19, 175-188.	Approximated from Fig. 1 by Meng et al. (2015), Fig. 1 by Cao et al. (2013) and Fig. 6 by Wang et al. (2009); eastern portion extended south and includes city of Jinan
**Yengejeh Basin**	-	Parizi, E., Hosseini, S. M., Ataie-Ashtiani, B., Simmons, C. T. (2020). Normalized difference vegetation index as the dominant predicting factor of groundwater recharge in	-	-	Approximated from tabulated coordinates in the Supplementary Information within Parizi et al. (2020) and physiographic and drainage patterns

Aquifer system	Broader aquifer system	Reference A	Reference B	Reference C	Steps to delineate
		phreatic aquifers: case studies across Iran. Scientific Reports, 10, 17473.			
**Ygnacio Valley**	-	California Department of Water Resources (2015g). California's groundwater update 2013 - Chapter 4: San Francisco Bay Hydrologic Region. California Department of Water Resources Report, 78 pp. Accessed April 27, 2022 via https://cawaterlibrary.net/wp-content/uploads/2017/05/GWU2013_Ch4_SanFranciscoBay_Final.pdf	-	-	Approximated from Fig. 4-2 by California Department of Water Resources (2015g)
**Yinchuan Plain**	-	Hui, Q., Li, P. (2011). Hydrochemical characteristics of groundwater in Yinchuan plain and their control factors. Asian Journal of Chemistry, 23, 2927.	-	-	Approximated from Fig. 1 by Hui and Li (2011).
**Yolo Basin**	Sacramento Valley of the Central Valley **	California Department of Water Resources (2015i). California's groundwater update 2013 - Chapter 7: Sacramento River Hydrologic Region. California Department of Water Resources Report, 126 pp. Accessed April 27, 2022 via https://water.ca.gov/-/media/DWR-Website/Web-Pages/Programs/Groundwater-Management/Bulletin-118/Files/Statewide-	-	-	Approximated from Fig. 7-2 by California Department of Water Resources (2015i)

Aquifer system	Broader aquifer system	Reference A	Reference B	Reference C	Steps to delineate
		Reports/GWU2013_Ch7_SacramentoRiver_Final.pdf			
**Yorkshire Chalk Aquifer**	-	MacDonald, A.M., Allen, D.J. (2001). Aquifer properties of the Chalk of England. Quarterly Journal of Engineering Geology and Hydrogeology, 34, 371-384.	Gale, I.N., Rutter, H.K. (2006). The Chalk aquifer of Yorkshire. British Geological Survey Research Report RR/06/04, 72 pp. Accessed October 27, 2021 from http://nora.nerc.ac.uk/id/eprint/3700/1/RR06004.pdf	Robins, N. S., Ball, D. F. (2006). The Dumfries Basin aquifer. British Geological Survey Research Report RR/06/02, 68 pp. Accessed October 27, 2021 from http://nora.nerc.ac.uk/id/eprint/3685/1/RR06002.pdf	Approximated from Fig. 8 by MacDonald and Allen (2001), Fig. 1 by Gale and Rutter (2006), and map on the final page of Robins and Ball (2006)
**Yoshino Basin**	-	Dong, L., Guo, Y., Tang, W., Xu, W., Fan, Z. (2022). Statistical evaluation of the influences of precipitation and river level fluctuations on groundwater in Yoshino River Basin, Japan. Water, 14, 625.	-	-	Approximated from Fig. 1 by Dong et al. (2022)
**Yucaipa Basin**	-	California Department of Water Resources (2015e). California's groundwater update 2013 - Chapter 6: South Coast Hydrologic Region. California Department of Water Resources Report, 84 pp. Accessed April 27, 2022 via https://cawaterlibrary.net/wp-content/uploads/2017/05/GWU2013_Ch6_SouthCoast_Final.pdf	-	-	Approximated from Fig. 6-2 by California Department of Water Resources (2015e)
**Yucca Flat**	-	Lopes, T.J., Evetts. D.M. (2005). Ground-Water pumpage and artificial recharge estimates for calendar year 2000 and average annual natural recharge and interbasin flow by hydrographic area,	Harrill, J.R., Prudic, D.E. (1998). Aquifer systems in the Great Basin region of Nevada, Utah, and adjacent states; summary report. US Geological Survey Professional Paper 1409-A, 75 pp. Accessed April 26,	-	Approximated from Fig. 1 by Lopes and Evetts (2005), and Fig. 2 by Harrill and Prudic (1998). Well completion records, physiography and drainage were used to delinate areas.

Aquifer system	Broader aquifer system	Reference A	Reference B	Reference C	Steps to delineate
		Nevada. Accessed April 25, 2022 via https://pubs.usgs.gov/sir/2004/5239/sir2004-5239.pdf	2022 via https://pubs.usgs.gov/pp/1409a/report.pdf		
**Yuma Basin**	-	Olmsted, F.H., Loeltz, O.J., Irelan, B. (1973). Geohydrology of the Yuma area, Arizona and California. US Geological Survey Professional Paper 486-H, 237 pp. Accessed February 15, 2021 from https://pubs.usgs.gov/pp/0486h/report.pdf	-	-	Approximated from Fig. 2 of Olmsted et al. (1973); southwestern margin approximated by Colorado River
**Yuncheng Basin**	-	Li, C., Gao, X., Wang, Y. (2015). Hydrogeochemistry of high-fluoride groundwater at Yuncheng Basin, northern China. Science of the Total Environment, 508, 155-165.	Gao, X., Wang, Y., Li, Y., Guo, Q. (2007). Enrichment of fluoride in groundwater under the impact of saline water intrusion at the salt lake area of Yuncheng basin, northern China. Environmental Geology, 53, 795-803.	Khair, A.M., Li, C., Hu, Q., Gao, X., Wanga, Y. (2014). Fluoride and arsenic hydrogeochemistry of groundwater at Yuncheng Basin, Northern China. Geochemistry International, 52, 868-881. http://dx.doi.org/10.1134/S0016702914100024	Approximated from Fig. 1 by Li et al. (2015), Fig. 1 by Gao et al. (2007), and Fig. 1 by Khair et al. (2014)
**Zaachila Valley**	Central Valleys of Oaxaca	Ojeda Olivares, E. A., Sandoval Torres, S., Belmonte Jiménez, S. I., Campos Enríquez, J. O., Zignol, F., Reygadas, Y., & Tiefenbacher, J. P. (2019). Climate change, land use/land cover change, and population growth as drivers of groundwater depletion in the central valleys, Oaxaca, Mexico. Remote Sensing, 11, 1290.	-	-	Approximated from Fig. 1 by Ojeda Olivares et al. (2019).
**Zaboli Aquifer**	-	Hosseini, S. M., Parizi, E., Ataie-Ashtiani, B., Simmons, C. T. (2019). Assessment of sustainable	-	-	Approximated from Fig. 6 by Hosseini et al. (2019)

Aquifer system	Broader aquifer system	Reference A	Reference B	Reference C	Steps to delineate
		groundwater resources management using integrated environmental index: Case studies across Iran. Science of the Total Environment, 676, 792-810.			
**Zacatepec Aquifer**	-	Ramirez, E., Robles, E., Sainz, M., Ayala, R., Campoy, E. (2009). Microbiological quality of the Zacatepec aquifer, Morelos, Mexico. Revista internacional de contaminación ambiental, 25, 247-255.	-	-	Approximated from Fig. 1 by Ramirez et al. (2009)
**Zanjan Plain**	-	Arasteh, S. M., & Shoaei, S. M. (2020). An assessment of the effects of excessive groundwater abstraction on the quality of groundwater resources of the Zanjan Plain, Iran. Environmental Earth Sciences, 79(23), 1-13.	-	-	Approximated from Fig. 6 by Hosseini et al. (2019)
**Zapotitan Aquifer**	-	Ministerio de Medio Ambiente y Recursos Natural (2016). Informe de monitoreo de los acuíferos de Zapotitán, Santa Ana y San Miguel. Ministerio de Medio Ambiente y Recursos Natural Report, 59 pp. Accessed February 8, 2023 via http://rcc.marn.gob.sv/bitstream/handle/123456789/127/Acuiferos%20%20StaAna%2c%20SnMiguel%20y%20Zapotit%c3%a1n_2016.p	-	-	Approximated from map on page 14 by Ministerio de Medio Ambiente y Recursos Natural (2016)

Aquifer system	Broader aquifer system	Reference A	Reference B	Reference C	Steps to delineate
		df?sequence=1&isAllowed=y			
**Zaran -e Saveh Basin**	-	Hosseini, S. M., Parizi, E., Ataie-Ashtiani, B., Simmons, C. T. (2019). Assessment of sustainable groundwater resources management using integrated environmental index: Case studies across Iran. Science of the Total Environment, 676, 792-810.	-	-	Approximated from Fig. 6 by Hosseini et al. (2019)
**Zarand Basin**	-	Hosseini, S. M., Parizi, E., Ataie-Ashtiani, B., Simmons, C. T. (2019). Assessment of sustainable groundwater resources management using integrated environmental index: Case studies across Iran. Science of the Total Environment, 676, 792-810.	Tezangi, M. F. (2016). Studying the effects of drought on groundwater aquifers of Zarand, Kerman. International Journal Of Pharmaceutical Research And Allied Sciences 5, 437-447.	-	Approximated from Fig. 6 by Hosseini et al. (2019) and Fig. 4 by Tezangi (2016)
**Zhangye Basin**	Heihe Basin	Qin, D., Qian, Y., Han, L., Wang, Z., Li, C., Zhao, Z. (2011). Assessing impact of irrigation water on groundwater recharge and quality in arid environment using CFCs, tritium and stable isotopes, in the Zhangye Basin, Northwest China. Journal of Hydrology, 405, 194-208.	Fang, J., Ding, Y. J. (2010). Assessment of groundwater contamination by NO 3− using geographical information system in the Zhangye Basin, Northwest China. Environmental Earth Sciences, 60, 809-816.	Chang, J., Wang, G. (2010). Major ions chemistry of groundwater in the arid region of Zhangye Basin, northwestern China. Environmental Earth Sciences, 61, 539-547.	Approximated from Fig. 1 by Qin et al. (2011), Fig. 1 by Fang and Ding (2010) and Fig. 1 by Chang and Wang (2010)

* aquifer system delineation processes published by ref.[1288] (full citation: GebreEgziabher, M., Jasechko, S., Perrone, D. (2022). Widespread and increased drilling of wells into fossil aquifers in the USA. *Nature Communications* **13,** 1-12) are reproduced (i.e., quoted, directly, from GebreEgziabher et al. (2022)) in this table. We quote text from ref.[1288] for these select aquifer systems so that the table above comprises a complete account of all 1,693 aquifer systems

** updated slightly from version published by ref.[1288]: GebreEgziabher, M., Jasechko, S., Perrone, D. (2022). Widespread and increased drilling of wells into fossil aquifers in the USA. *Nature Communications* **13,** 1-12. Aquifer-specific updates (i.e., changes made to the geospatial data analysed in this study relative to the geospatial data presented by GebreEgziabher et al. (2022)) include: (i) for the Los Angeles Basin - redefined boundaries and split up the Los Angeles Basin into numerous subareas after consulting ref.[213]: California Department of Water Resources (2015). California's groundwater update 2013 - Chapter 6: South

Coast Hydrologic Region. California Department of Water Resources Report, 84 pp.; (ii) for the California Central Valley – this broader aquifer system (i.e., the entire Central Valley, as presented by GebreEgziabher et al. (2022) has now been split into three new ‘Broader Aquifer Systems’ (the Sacramento Valley, the San Joaquin Basin, and the Tulare Basin, each with numerous subareas; see Supplementary Table 2); (iii) for the Sierra Nevada Foothills – the western boundary has been updated slightly to remain adjacent to the revised boundaries of California Central Valley subareas (see “(ii)” directly above within this paragraph) and the word “Upper” has been appended to precede each basin (e.g., “Upper Kaweah Basin” instead of “Kaweah Basin” to distinguish these areas from the updated titles of subbasins within the California Central Valley (iv) for the Central High Plains – aquifer system has been further subdivided into three areas (Northcentral, Southcentral and Central High Plains); (v) for the Upper Santa Ana Basin – updated boundaries subdivide this broader aquifer system into several subareas. (vi) for the ‘Main Basin and Sinclair Inlet and Western Hood Canal’ (original title in ref.[1288] – area now contains only the main basin (i.e., area near Seattle) and does not encompass the polygon representing Bainbridge Island and the surrounding area as published by ref.[1288]

*** cropland cover for the Northern Guam Lens Aquifer System estimated from https://data.worldbank.org/indicator/AG.LND.CROP.ZS?locations=GU (accessed July 3, 2023), as the gridded cropland cover database we analysed for all other aquifer systems in our study did not cover Guam

8 21ST CENTURY GROUNDWATER STORAGE TRENDS FROM GRACE SATELLITES

We compiled four GRACE-based groundwater storage anomaly datasets (for detailed description of these datasets see ref.[1307]: Shamsudduha, M., & Taylor, R.G. Groundwater storage dynamics in the world's large aquifer systems from GRACE: uncertainty and role of extreme precipitation. *Earth System Dynamics* **11,** 755–774 (2020)):

(i) CSR land (for details see ref.[1308]: Landerer, F. W. and Swenson, S. C. Accuracy of scaled GRACE terrestrial water storage estimates, Water Resources Research **48,** W04531, https://doi.org/10.1029/2011WR011453 (2012)).

(ii) JPL Global Mascon solutions from NASA (http://grace.jpl.nasa.gov/data, accessed August 5, 2020; for details see refs.[1309,1310]: Watkins, M.M., Wiese, D.N., Yuan, D.-N., Boening, C., Landerer, F.W. Improved methods for observing Earth’s time variable mass distribution with GRACE using spherical cap mascons, J. Geophys. Res.-Sol. Ea. **120,** 2648–2671 (2015). and Wiese, D.N., Landerer, F.W., Watkins, M. M. Quantifying and reducing leakage errors in the JPL RL05M GRACE mascon solution, Water Resources Research **52,** 7490–7502 (2016)).

(iii) GRGS GRACE solution (CNES/GRGS release RL03-v1) from the French space agency: Centre National D’études Spatiales (CNES; see ref.[1311]: Biancale, R., Lemoine, J.-M., Balmino, G., Loyer, S., Bruisma, S., Perosanz, F., Marty, J.-C., and Gégout,

P.: 3 Years of Geoid Variations from GRACE and LAGEOS Data at 10-day Intervals from July 2002 to March 2005, CNES/GRGS, 2006).

(iv) an ensemble mean of these three GRACE solutions (i.e., the average of (i), (ii) and (iii) above, calculated on a monthly time step; for details see ref.[1307]: Shamsudduha, M., & Taylor, R.G. (2020). Groundwater storage dynamics in the world's large aquifer systems from GRACE: uncertainty and role of extreme precipitation. *Earth System Dynamics* **11,** 755–774 (2020)).

We calculated annual median groundwater storage anomalies for each calendar year for each GRACE grid cell. We calculated annual median groundwater storage anomalies in an effort to be as consistent as possible with the steps we applied to analyse our monitoring well data (see main text Methods). Next, we calculated trends in annual median groundwater storage anomalies over time for each grid cell over the years 2002-2016 via robust regression (Theil-Sen slopes). Last, we overlaid boundaries of aquifer systems where our monitoring well data suggest aquifer-scale groundwater levels declined faster than 0.5 m/year (n=210 polygons; see figures below).

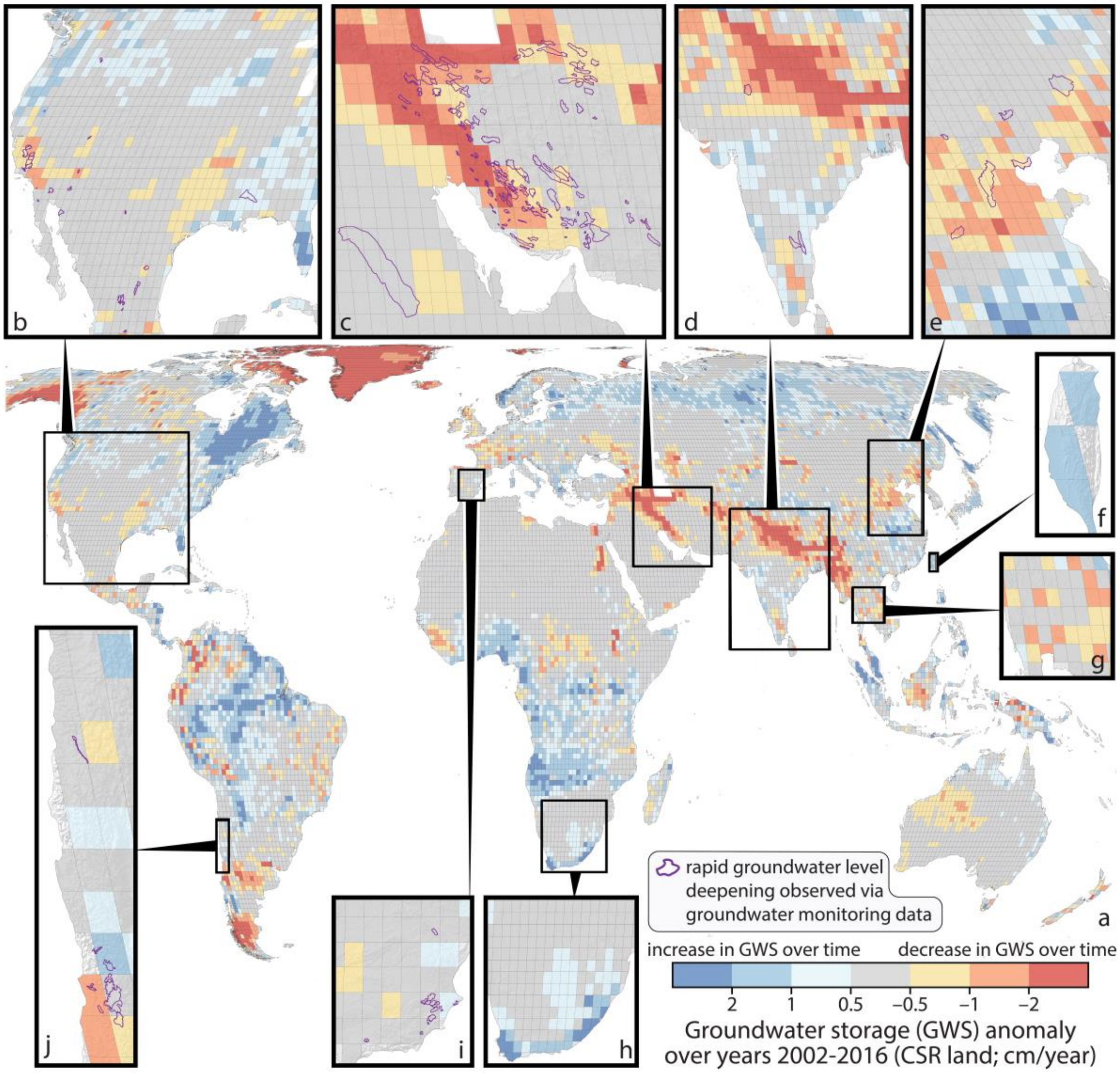

Supplementary Fig. 14. Groundwater storage anomaly trends from GRACE satellites (data presented here from CSR land). Red areas are places where GRACE-based data suggest groundwater storage declined from 2002-2016, and blue areas are places where GRACE-based data suggest groundwater storage increased. Panels a-j present regional maps for various regions around the globe. Purple polygons within these regional maps mark the boundaries of aquifer systems where the trend in depth to groundwater exceeds 0.5 m/year (groundwater levels became deeper over time rapidly; median Theil-Sen slope). The CSR GRACE dataset suggests that groundwater storage declined in many of the places where our monitoring wells demonstrate this rapid (>0.5 m/year) groundwater level deepening, as demonstrated by the red shades (which mark declining groundwater storage) overlapping many of the purple polygons. However, there are some aquifer systems where groundwater levels have become deeper over time although this GRACE product suggests an increase in groundwater storage, including central Saudi Arabia (panel c), southeastern Spain (panel i) and central Chile (panel j).

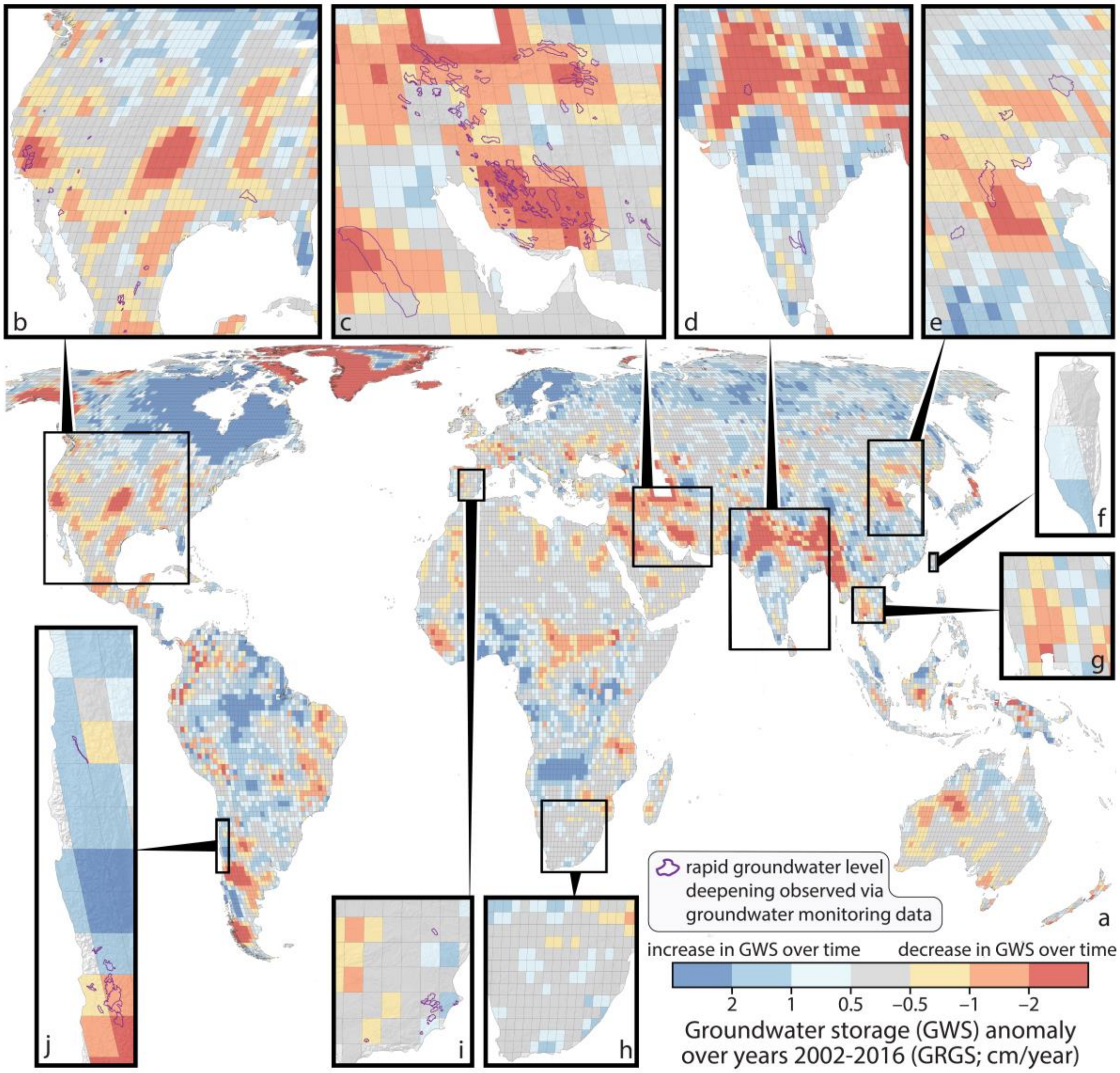

Supplementary Fig. 15. Groundwater storage anomaly trends from GRACE satellites (data presented here based on GRGS). Red areas are places where GRACE-based data suggest groundwater storage declined from 2002-2016, and blue areas are places where GRACE-based data suggest groundwater storage increased. Panels a-j present regional maps for various regions around the globe. Purple polygons within these regional maps mark the boundaries of aquifer systems where the trend in depth to groundwater exceeds 0.5 m/year (groundwater levels became deeper over time rapidly; median Theil-Sen slope). The GRGS GRACE dataset suggests groundwater storage declined in many of the places where our monitoring wells demonstrate this rapid (>0.5 m/year) groundwater level deepening, as demonstrated by the red shades (which mark declining groundwater storage) overlapping many of the purple polygons. However, there are some aquifer systems where groundwater levels have become deeper over time although this GRACE product suggests an increase in groundwater storage, including southeastern Spain (panel i) and southeastern India (panel d).

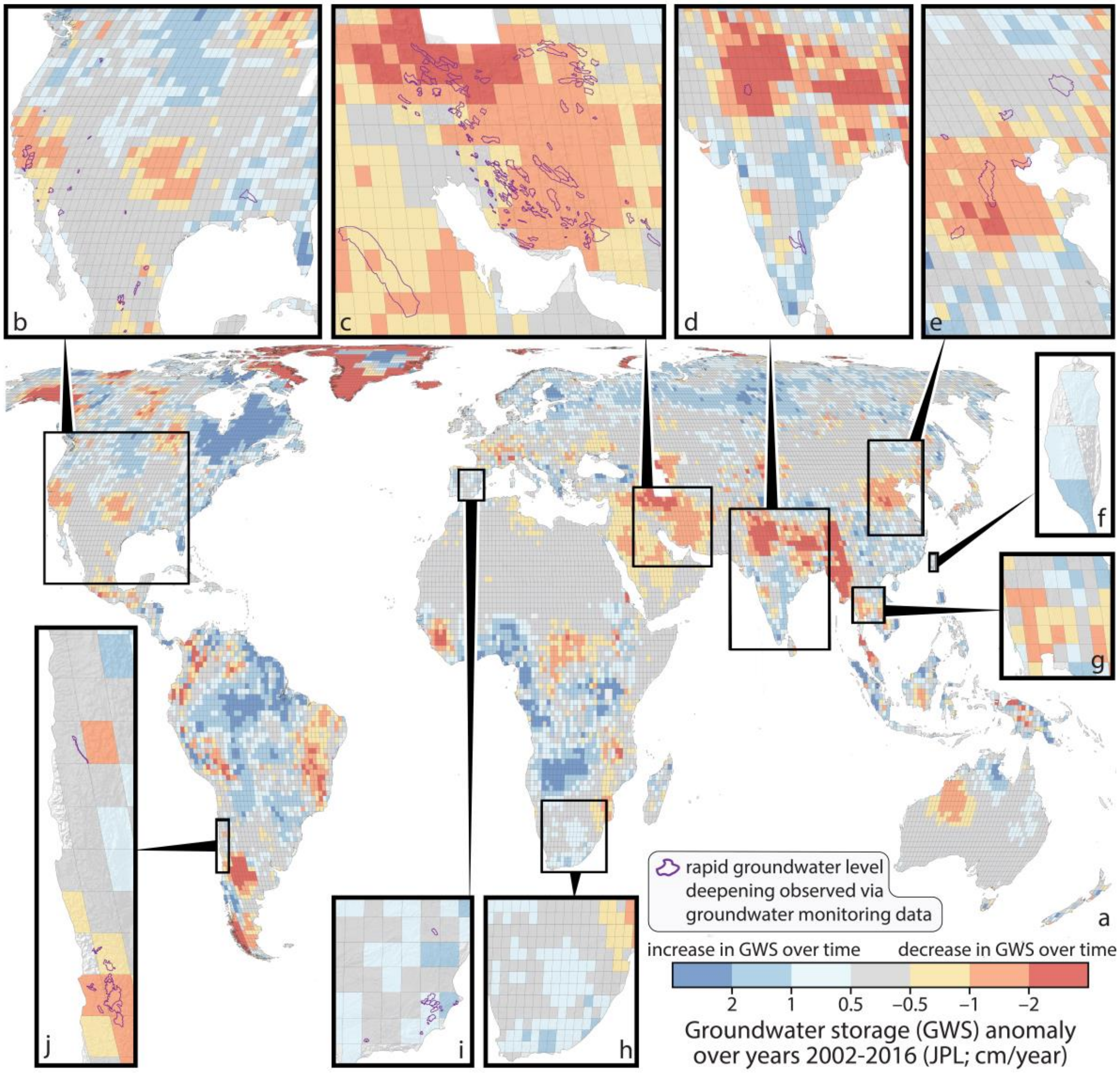

Supplementary Fig. 16. Groundwater storage anomaly trends from GRACE satellites (data presented here based on JPL). Red areas are places where GRACE-based data suggest groundwater storage declined from 2002-2016, and blue areas are places where GRACE-based data suggest groundwater storage increased. Panels a-j present regional maps for various regions around the globe. Purple polygons within these regional maps mark the boundaries of aquifer systems where the trend in depth to groundwater exceeds 0.5 m/year (groundwater levels became deeper over time rapidly; median Theil-Sen slope). The JPL GRACE dataset suggests groundwater storage declined in many of the places where our monitoring wells demonstrate this rapid (>0.5 m/year) groundwater level deepening, as demonstrated by the red shades (which mark declining groundwater storage) overlapping many of the purple polygons. However, there are some aquifer systems where groundwater levels have become deeper over time although this GRACE product suggests an increase in groundwater storage, including south-eastern Spain (panel i) and south-eastern India (panel d).

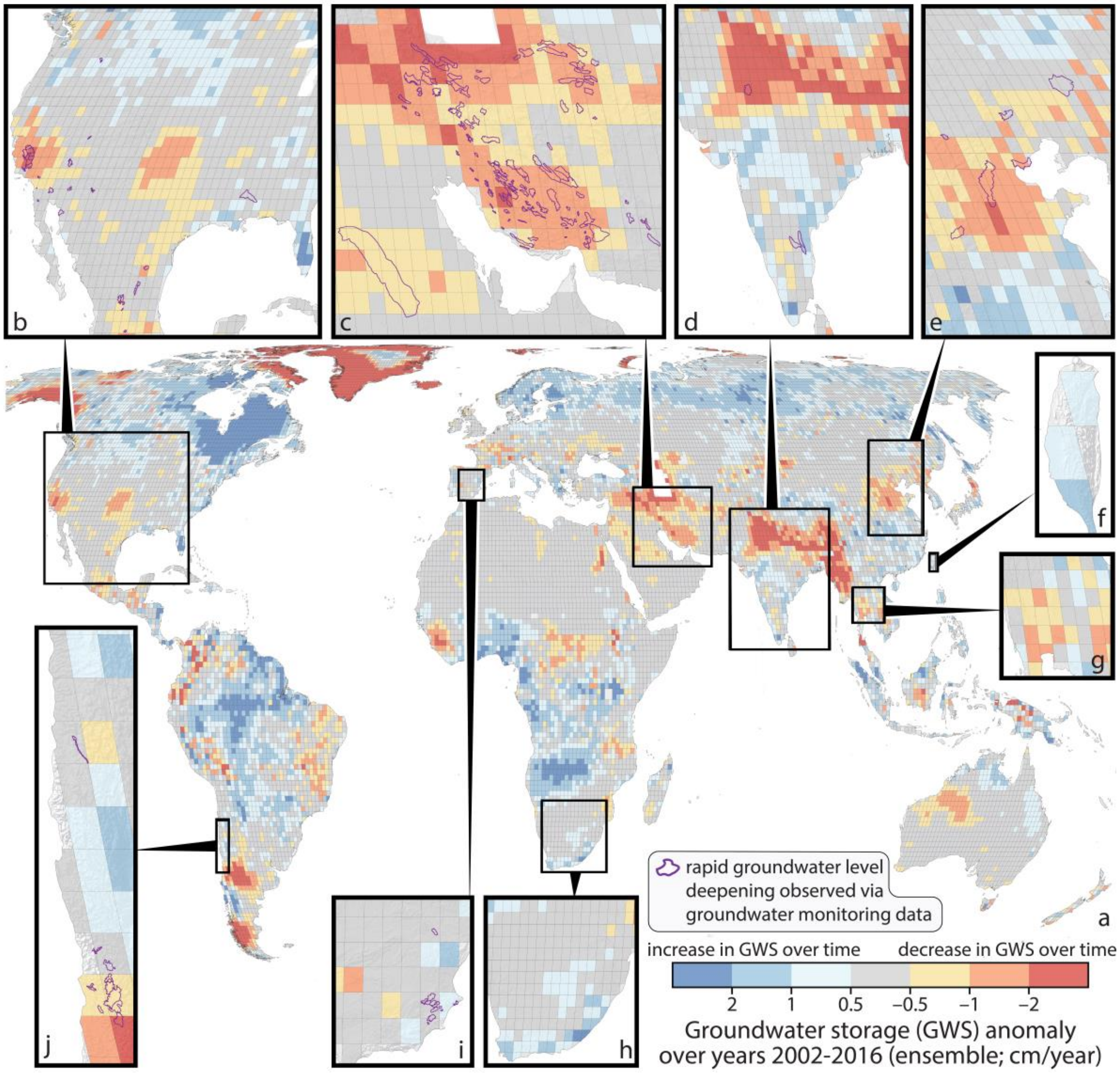

Supplementary Fig. 17. Groundwater storage anomaly trends from GRACE satellites (data presented here based on an ensemble mean of groundwater storage anomaly values from other three GRACE datasets presented above). Red areas are places where GRACE-based data suggest groundwater storage declined from 2002-2016, and blue areas are places where GRACE-based data suggest groundwater storage increased. Panels a-j present regional maps for various regions around the globe. Purple polygons within these regional maps mark the boundaries of aquifer systems where the trend in depth to groundwater exceeds 0.5 m/year (groundwater levels became deeper over time rapidly; median Theil-Sen slope). The ensemble GRACE dataset suggests groundwater storage declined in many of the places where our monitoring wells demonstrate this rapid (>0.5 m/year) groundwater level deepening, as demonstrated by the red shades (which mark declining groundwater storage) overlapping many of the purple polygons. However, there are some aquifer systems where groundwater levels have become deeper over time although this GRACE product suggests an increase in groundwater storage, including south-eastern Spain (panel i) and south-eastern India (panel d).

Groundwater storage decreased over time (Theil-Sen slopes based on GRACE data) in many of the aquifer systems where groundwater levels became deeper faster than 0.5 m/year (median Theil-Sen slope based on monitoring well data; see overlap of red areas and purple polygons in southern California, Iran, and northern China in Supplementary Figs. 14-17). These areas—where GRACE and piezometric data provide consistent results—highlight the complementary nature of GRACE and monitoring well datasets.

On the other hand, GRACE datasets suggest groundwater storage has remained stable (or even increased) in some aquifer systems where monitoring wells demonstrate that groundwater levels became deeper at rates exceeding 0.5 m/year (e.g., southeastern India, southeastern Spain, and central Chile; panels d, i and j in Supplementary Figs. 14-17). The cause of these inconsistencies remains unclear, but could be related in part to (a) the coarse spatial resolution of the GRACE products compared to the relatively small areas of many aquifer systems, and/or (b) changes in other water stores, such as glaciers or soil moisture, that influence GRACE-based groundwater storage estimates but do not influence groundwater level measurements directly, and/or (c) challenges in converting raw GRACE-satellite observations into groundwater storage anomalies, as evidenced by differences between the JPL, CSR and GRGS datasets in some areas (e.g., differences in groundwater storage anomaly trends among CSR, JPL and GRGS in central Chile; panel j in the figures above), and/or (d) differences in the time intervals captured by monitoring well data and GRACE data.

A factor that complicates efforts to compare GRACE observations and monitoring well measurements arises from aquifer storage coefficients. Specifically, monitoring well water level measurements are influenced by aquifer storage coefficients (i.e., defined as the volume of water that is expelled from an aquifer per unit change in groundwater level) but GRACE, on the other hand, provides insights into groundwater

storage and is not impacted directly by spatial variations in aquifer storage coefficients. Unconfined aquifers typically have relatively high storage coefficients (i.e., typical values range from 1% to 40%), as unconfined storage coefficients derive from the volume of water that can be drained via gravity from the aquifer (i.e., storage coefficient ≈ ‘specific yield’). Conversely, confined aquifers typically have small storage coefficients (e.g., values of <0.1 %), because storage coefficients are equivalent to ‘specific storage’, which is the volume of water expelled per unit decline in hydraulic head arising from the expansion of stored water and the compression of the mineral skeleton. Consequently, a unit change in groundwater level (recorded in piezometric datasets) cannot be straightforwardly converted into a unit change in groundwater storage (recorded in GRACE datasets) because we lack adequate hydrogeologic data to estimate storage coefficients in the great majority of the 1,693 aquifer systems that we studied here.

9 21ST CENTURY GROUNDWATER LEVEL TRENDS COMPARED TO 1980-2000 GROUNDWATER LEVEL TRENDS

We identified n=45,911 monitoring wells in our compilation that have adequate data to evaluate trends in depth to groundwater during both 1980-2000 and during 2000-2022. For each of these monitoring wells, we calculated trends in depth to groundwater for each time interval (Theil-Sen slope). Next, we calculated the median of these Theil-Sen slopes among all monitoring wells within each aquifer system. We calculated the median based solely on monitoring wells that had sufficient data to analyse trends during 1980-2000 *and* during 2000-2022. We excluded any aquifer systems with fewer than 5 unique monitoring wells. Last, we categorised aquifer systems on the basis of their 1980-2000 and 2000-2022 trends in depth to groundwater (see categories (i) to (v) below):

(i) *groundwater levels became shallower over time from 1980-2000, and continued to become shallower over time since the year 2000 (purple in display items);*
(ii) *groundwater levels became shallower over time from 1980-2000, but have since become deeper over time since the year 2000 (yellow in display items);*
(iii) *groundwater levels became deeper over time from 1980-2000, but have since become shallower over time since the year 2000 (blue in display items);*
(iv) *groundwater levels became deeper over time from 1980-2000, and continued to become deeper over time*

from 2000-2022 but at a slower rate (orange in display items); or,

(v) groundwater levels became deeper over time from 1980-2000, and continued to become deeper over time from 2000-2022 at a faster rate (red in display items).

Supplementary Table 6. n=542 aquifer systems categorised by groundwater level trends from 1980-2000 and 2000-2022 (background shades key to the colours in the legend in main text figure and the supplementary figures on the following pages)

Category (colours match those for each category shown in figures on following pages)	Number of aquifer systems in category
groundwater levels became **shallower over time from 1980-2000**, and **continued to become shallower** over time since the year 2000	**n=69** aquifer systems (**12.7%** of aquifer systems)
groundwater levels became **shallower over time from 1980-2000**, but have **since become deeper** over time since the year 2000	**n=117** aquifer systems (**21.6%** of aquifer systems)
groundwater levels became **deeper over time from 1980-2000**, but have since **become shallower** over time since the year 2000	**n=86** aquifer systems (**15.9%** of aquifer systems)
groundwater levels became **deeper over time from 1980-2000**, and **continued to become deeper** over time from 2000-2022 but at a **slower rate**	**n=107** aquifer systems (**19.7%** of aquifer systems)
groundwater levels became **deeper over time from 1980-2000**, and **continued to become deeper** over time from 2000-2022 at a **faster rate**	**n=163** aquifer systems (**30.1%** of aquifer systems)

We grouped aquifer systems based on (i) average precipitation divided by evapotranspiration, and (ii) the proportion of land under cultivation. We show that accelerated groundwater level deepening (i.e., where groundwater levels became deeper over time from 1980-2000, and continued to become deeper over time from 2000-2022 at a faster rate) is especially common in aquifer systems that are located in cultivated drylands (see table below).

Supplementary Table 7. Percentage of aquifer-scale groundwater-levels trends classified as accelerated groundwater level deepening (groundwater levels became deeper over time from 1980-2000, and continued to deepen from 2000-2022 but at a faster rate)

Climate classification	**Percent of land area under cultivation**		
	**<1% of land area under cultivation**	**1 to 20% of land area under cultivation**	**>20% of land area under cultivation**
Hyper-arid	**13%** (1 of 8 aquifer systems)	**17%** (1 of 6 aquifer systems)	**0%** (0 of 2 aquifer systems)
Arid	**27%** (13 of 48 aquifer systems)	**39%** (24 of 61 aquifer systems)	**58%** (44 of 76 aquifer systems)
Semi-arid	**17%** (2 of 12 aquifer systems)	**30%** (24 of 79 aquifer systems)	**29%** (31 of 106 aquifer systems)
Dry subhumid	**0%** (0 of 1 aquifer systems)	**12%** (2 of 17 aquifer systems)	**22%** (4 of 18 aquifer systems)
Humid	**0%** (0 of 1 aquifer systems)	**5%** (3 of 60 aquifer systems)	**30%** (14 of 47 aquifer systems)

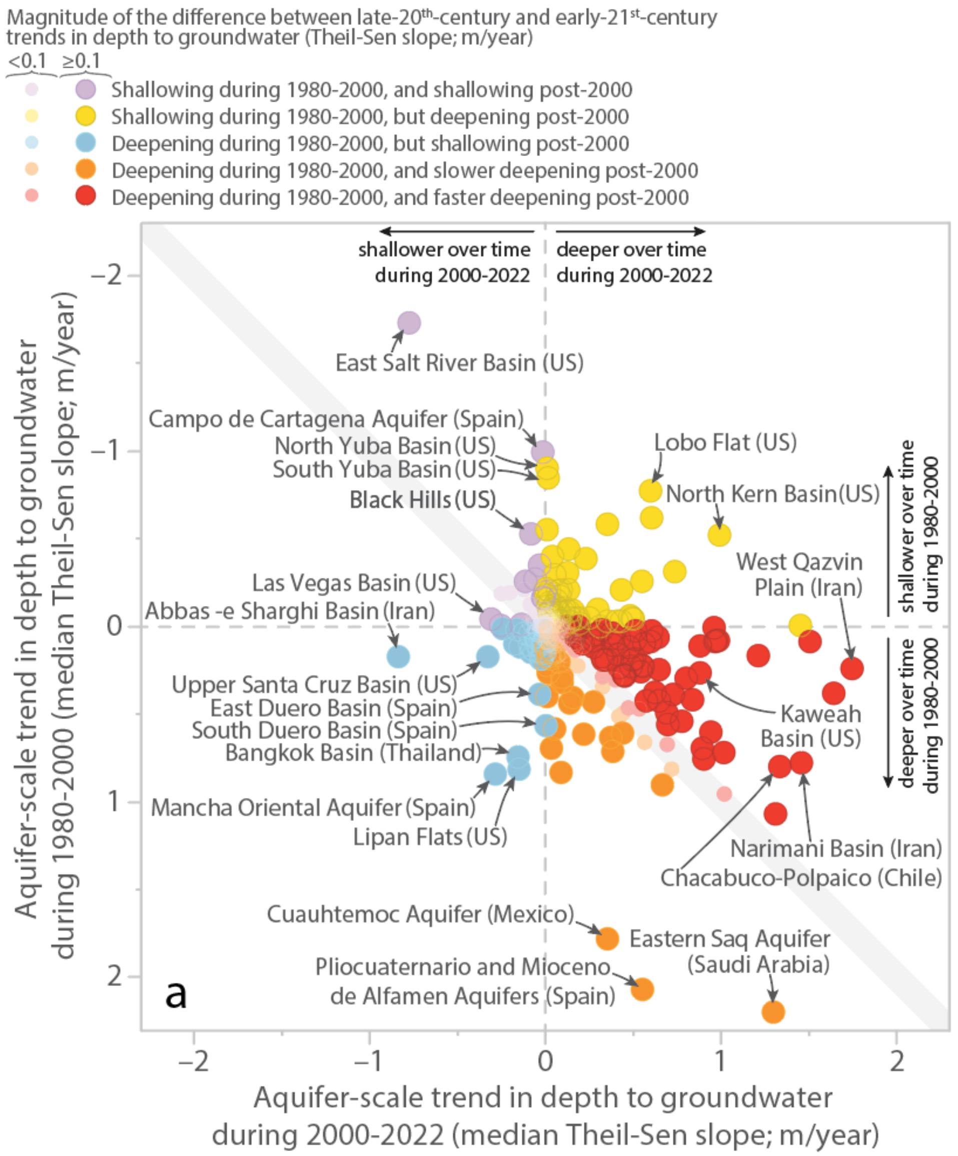

Supplementary Fig. 18. Comparison of aquifer-scale trends in depth to groundwater during 1980-2000 and during 2000-2022. Scatterplot of aquifer-scale groundwater-level trends (median Theil-Sen slopes) during two different time intervals: 2000-2022 (x-axis values), and 1980-2000 (y-axis values). Each circle represents one aquifer system and its colour categorises its groundwater-level trends as one of the following: (i) groundwater levels became shallower during 1980-2000, and continued to become shallower (purple circles); (ii) groundwater levels became shallower during 1980-2000, but have since become deeper (yellow circles); (iii) groundwater levels became deeper during 1980-2000, but have since become shallower (blue circles); (iv) groundwater levels became deeper during 1980-2000, and continued to become deeper but at a slower rate (i.e., groundwater-level deepening continued at a decelerated pace; orange circles); and (v) groundwater levels became deeper during 1980-2000, and continued to become deeper at a faster rate (i.e., groundwater-level deepening continued at an accelerated pace; red circles). A disproportionately large share of our aquifer systems fall under category "v" (i.e., where rates of groundwater-level deepening during the 21st century took place at a steeper rate than groundwater-level deepening during 1980-2000). The intensity of the colours scale to the absolute value (i.e., magnitude) of the difference between the late-20th and early-21st century groundwater-level trends. Labels provide titles of a larger number of individual aquifer systems (thus distinguishing this supplementary figure from main text figure).

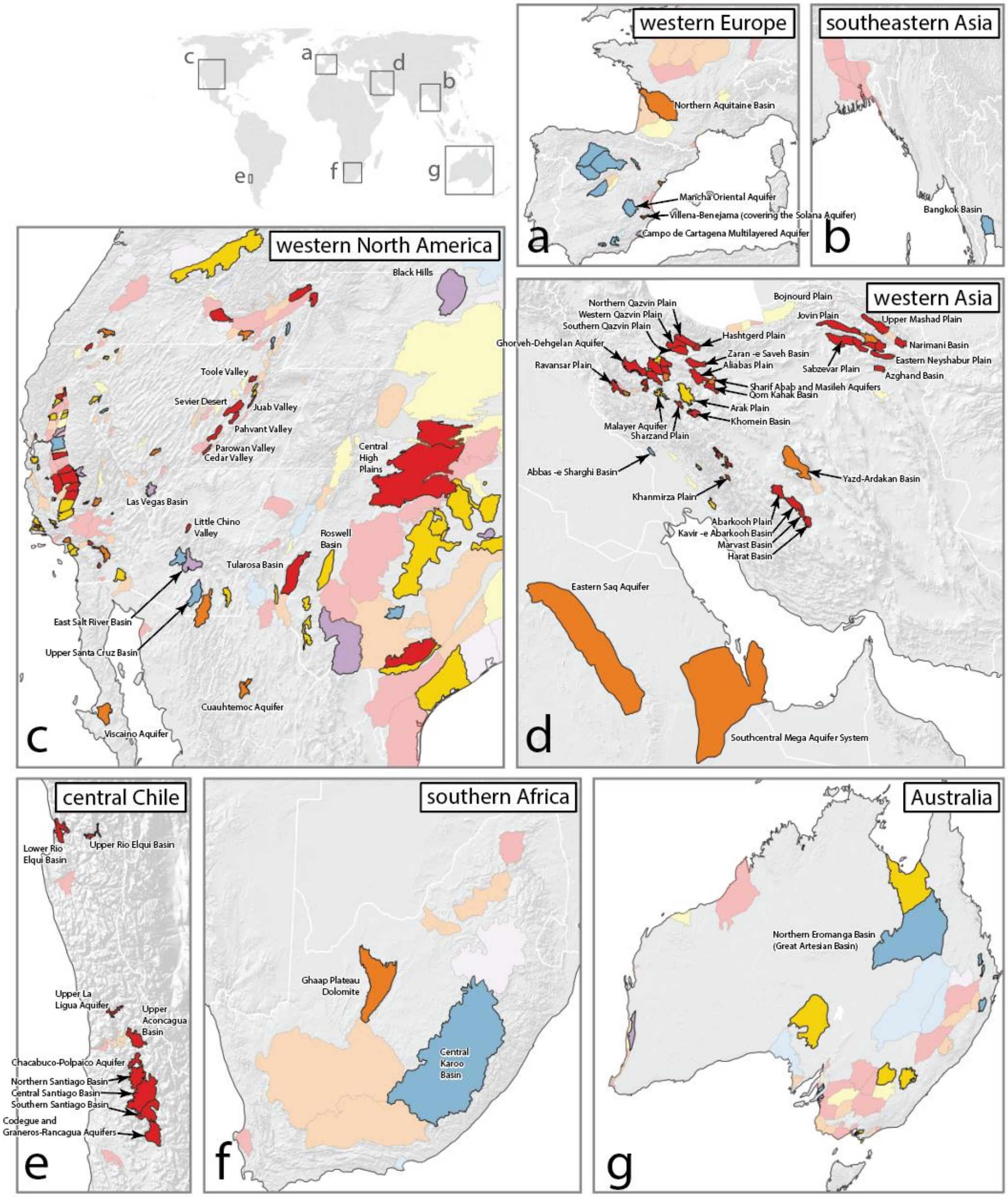

Supplementary Fig. 19. Spatial distribution of aquifer systems categorised by trends in depth to groundwater during 1980-2000 and during 2000-2022. Each polygon represents one aquifer system. The colour of each polygon classifies the aquifer system's groundwater level trends into one of our five categories (see previous figure caption). The intensity of the colours scale to the absolute value (i.e., magnitude) of the difference between the late-20th and early-21st century groundwater-level trends. 21st century groundwater levels have deepened at a faster rate than during the 1980s and 1990s (red polygons) in **c)** the southeastern California Central Valley, Central High Plains, several valleys in west-central Utah, **d)** numerous basins in central, northwestern and northeastern Iran; and, **e)** alluvial basins in central Chile (e.g., the Santiago Basin).

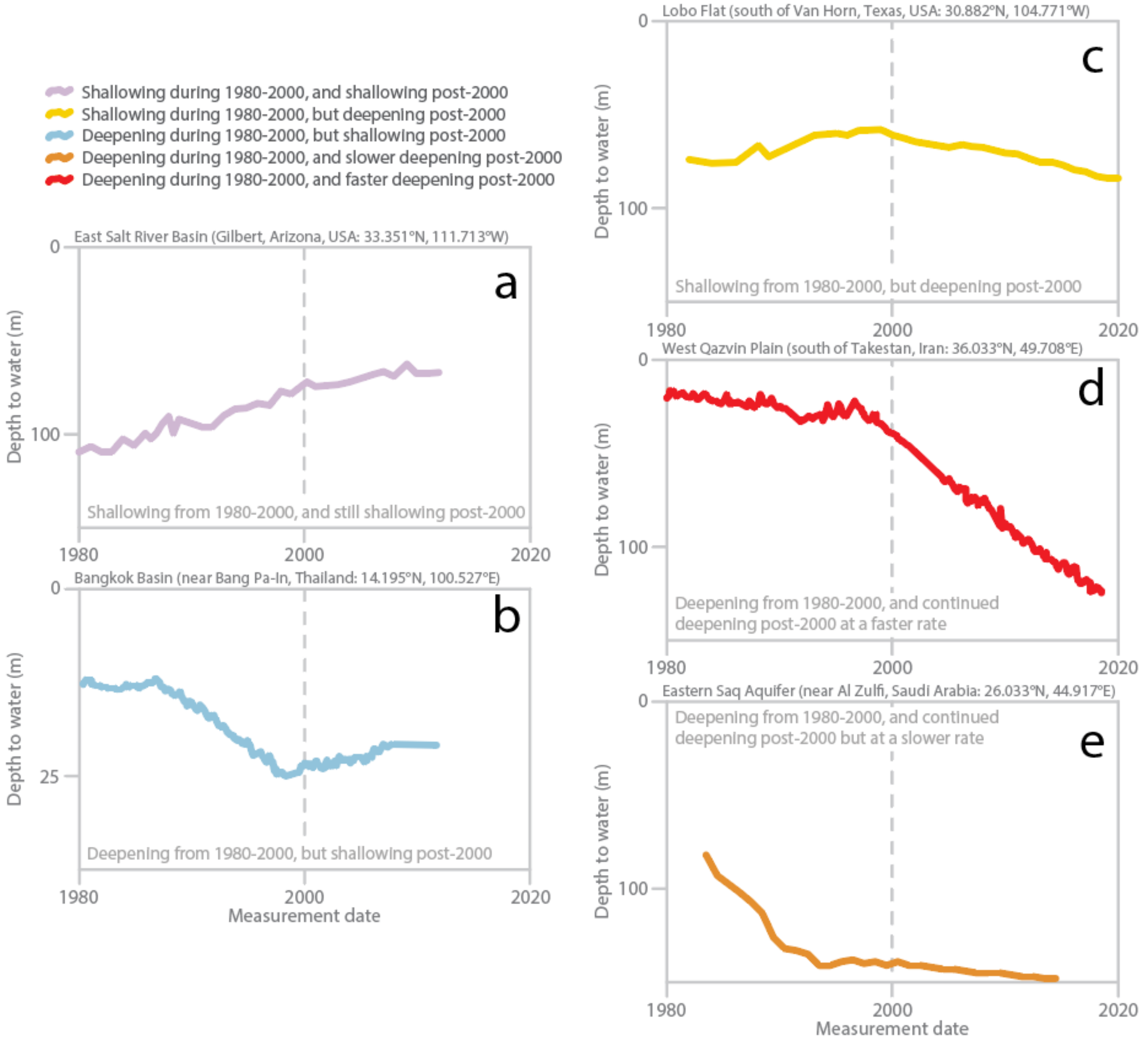

Supplementary Fig. 20. Groundwater level time series for individual monitoring wells exemplifying the five categories juxtaposing late-20th-century and early-21st-century trends in depth to groundwater. Each time series represents one of our five categories (the colour of each line corresponds to a category described in the legend). The y-axis is inverted so that the land surface is represented by the top of each plot (i.e., by a y-axis value of zero). The label overlying each plot provides information about the location of the monitoring well (including its latitude and longitude). a) Near Gilbert, Arizona (USA), groundwater levels rose in the late 20th century, and continued to rise in the early 21st century. b) Near Bang Pa-In (Thailand), groundwater levels became deeper over time during the late 20th century, but these trends reversed as groundwater levels became shallower over time during the early 21st century. c) Near Van Horn, Texas (USA), groundwater levels rose in the late 20th century, but these trends reversed as groundwater levels became deeper over time during the early 21st century. d) Near Takestan (Iran), groundwater levels became deeper over time in the late 20th century, and this deepening trend continued during the early 21st century at an even faster rate (i.e., acceleration of groundwater level declines). e) Near Al Zulfi (Saudi Arabia), groundwater levels became deeper over time in the late 20th century, and this deepening trend continued during the early 21st century but at a slower rate (i.e., deceleration of groundwater level declines). Cases of deceleration in groundwater-level declines may reflect changes in water balances or transitions from confined conditions to unconfined conditions (for examples of such transitions see (i) ref.[1364]: Rushton, K.R., Zaman, M.A., & Mehedi Hasan, M. Sustainable abstraction due to unconfined conditions in multi-layered aquifers: Examples from northwest Bangladesh. Groundwater for Sustainable Development 20, 100901 (2023), and, (ii) ref.[1363]: Shamsudduha, M., Taylor, R.G., Ahmed, K.M., & Zahid, A. The impact of intensive groundwater abstraction on recharge to a shallow regional aquifer system: evidence from Bangladesh. Hydrogeology Journal, 19, 901-916 (2011)). These time series—each representing a single monitoring well—exemplify the five categories described in the main text.

Some of the aquifer-scale trends in depth to groundwater for the 21st century (i.e., values in main text Fig. 3) were calculated using a larger number of monitoring wells than the late-20th century aquifer-scale trends in depth to groundwater. These differences can arise where an aquifer system contains, within its boundaries, one or more monitoring wells that (a) have adequate data to meet our criteria to calculate a trend in depth to groundwater for the early 21st century (2000-2022), and (b) did not have adequate data to meet our criteria to calculate a trend in depth to groundwater for the late 20th century (1980-2000). The schematic below depicts such a case, highlighting the (i) steps applied to produce the results presented in the main text Fig. 3 (and discussed within the main text, and presented in the display items above this paragraph within this Supplementary Note 9), and (ii) a separate analysis (i.e., set of steps) that we present here that test how our main results would differ if we exclusively analyse monitoring wells that met our criteria for analyses for both the early-21st and late-20th centuries when calculating aquifer-scale trends in depth to groundwater for the early-21st century.

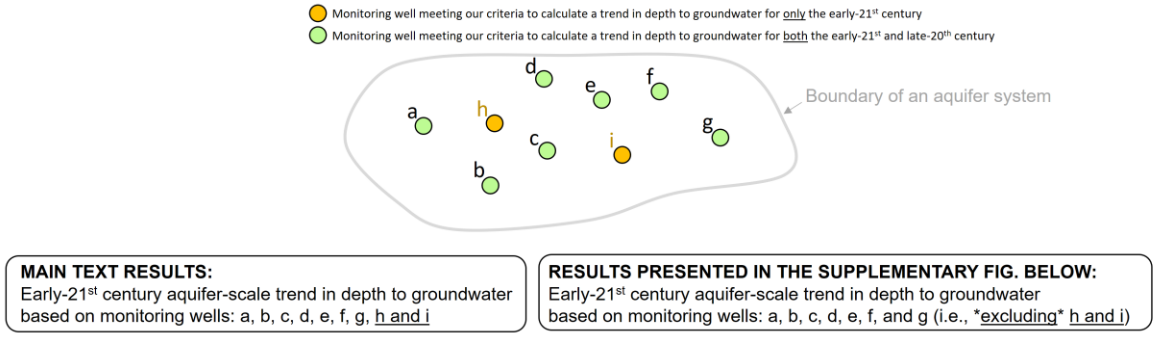

Supplementary Fig. 21. Schematic of two different data filtering processes for the calculation of aquifer-scale trends in depth to groundwater for the 21st-century. Each circle in the upper portion of the figure represents a hypothetical monitoring well. The green circles represent monitoring wells with adequate data for analyses of groundwater level trends for *both* the late-20th and the early-21st centuries. The orange circles represent monitoring wells that had adequate data to calculate a trend in depth to groundwater for the early-21st century, but not for the late-20th century. Our main text results (i.e., the x-axis values in Fig. 3) are based on the inclusion of all monitoring wells within the aquifer system meeting our minimum criteria to calculate a 21st-century trend in depth to groundwater (i.e., including the orange and the green circles to calculate a 21st-century trend in depth to groundwater, thus yielding identical trends to those presented in main text Fig 2 and 4). However, an alternate approach is to analyse only the wells with adequate time series data for both the late-20th and early-21st centuries when calculating the aquifer-scale trend in depth to groundwater for the earl-21st century (i.e., taking the median of the monitoring well trends in depth to groundwater for 2000-2022 using *only* the green circles (and excluding orange circles)). This alternate scenario is presented in the supplementary figure below.

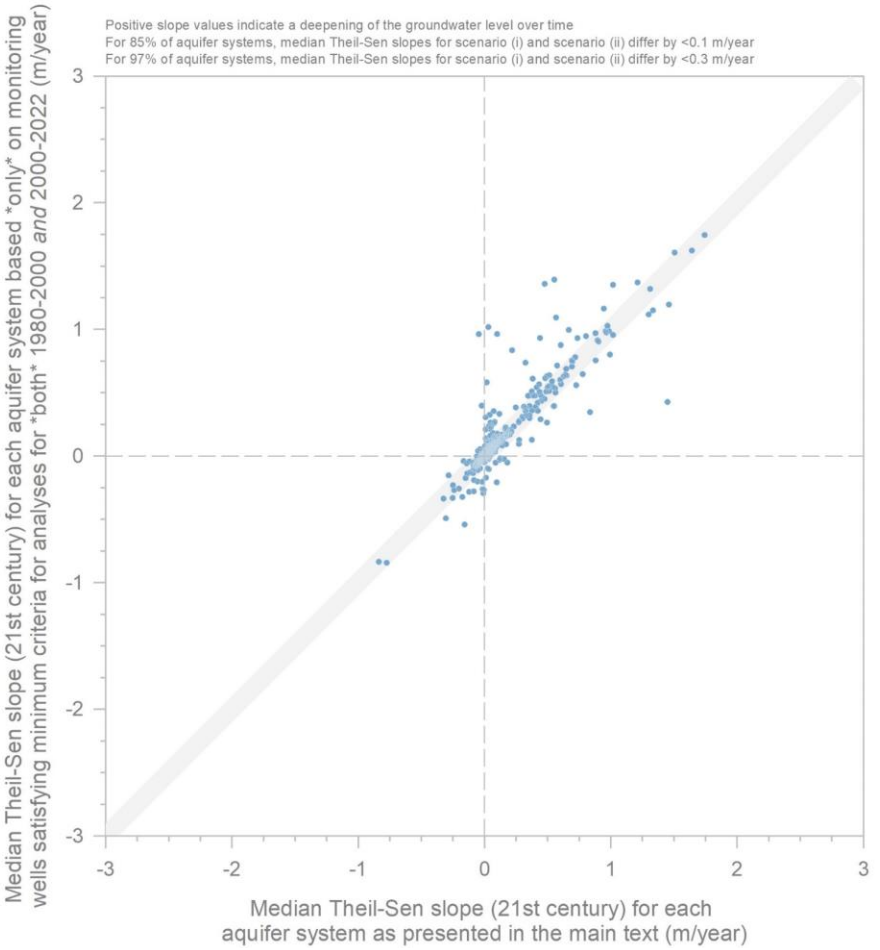

Supplementary Fig. 22. 21st century aquifer-scale trends in depth to groundwater following two different data filtering processes (for schematic of the two data filtering processes, see the supplementary figure on the preceding page). Each circle in the figure represents one of the 542 aquifer systems depicted in main text Fig. 3. The x-axis presents 21st-century trends in depth to groundwater as presented in the main text (Figs. 2, 3 and 4); these main text results (plotted on the x-axis here) are based on the inclusion of all monitoring wells within the aquifer system meeting our minimum criteria to calculate a 21st-century trend in depth to groundwater (i.e., including the orange and the green circles, as depicted in the schematic on the previous page of this Supplementary Note, to calculate a 21st-century trend in depth to groundwater). The y-axis values represent aquifer-scale trends in depth to groundwater (i.e., median of all monitoring wells within an aquifer system's boundaries with adequate data for analyses) based *only* on the wells with adequate time series data for both the late-20th and early-21st centuries (i.e., taking the median of the monitoring well trends in depth to groundwater for 2000-2022 using *only* the green circles (and excluding orange circles) as depicted in the supplementary figure on the previous page). Nearly all (97%) of the 21st century aquifer-scale trends in depth to groundwater differ by less than 0.3 m/year, and the great majority (85%) differ by less than 0.1 m/year; this figure demonstrates that the results we present in main text Fig. 3 are not highly sensitive to the decision to include (versus exclude) monitoring wells that only have adequate data for the 21st century (but lack adequate data for analyses of trends in the late-20th century). We calculated the proportions of aquifer systems that fall under each of our five categories (i.e., five colours in main text Fig. 3), if we were to use the values presented on the y-axis in this plot (instead of the values plotted on the x-axis). We find trivial differences in the proportion of aquifer systems that fall into each of the five categories if we use these y-axis values (plotted above). Specifically, the proportions (keyed to the categories in Supplementary Table 7 above) are: (i) 20.8% have rising groundwater levels followed by deepening levels (yellow), (ii) 32.3% of cases are accelerating (red), (iii) 17.0% of cases are decelerating (orange), (iv) 16.4% have recovering groundwater levels (blue), and (v) 13.5% have continually rising groundwater levels (purple; see main text Fig. 3 or Supplementary Table 7 above for detailed description of categories). These proportions are nearly identical to those presented in Supplementary Table 7, highlighting that our results are largely insensitive to the inclusion or exclusion of monitoring wells with adequate data for solely the early 21st century (i.e., the two scenarios presented in the preceding supplementary figure).

10 PRECIPITATION VARIABILITY OVER FOUR DECADES IN AQUIFER SYSTEMS

Groundwater quantity can be influenced by climate variability via direct impacts (e.g., changes in recharge rates) or via indirect impacts (e.g., changes in groundwater demands; ref.[27]: Taylor, R.G. et al. (2013). Ground water and climate change. *Nature Climate Change* **3,** 322-329). We analyse precipitation time series for our 1,693 aquifer systems. Specifically, we analysed gridded precipitation data from:

> (A) 'TerraClimate' (ref.[1372]: Abatzoglou, J.T., Dobrowski, S.Z., Parks, S.A., & Hegewisch, K.C. TerraClimate, a high-resolution global dataset of monthly climate and climatic water balance from 1958–2015. *Scientific Data* 5 (2018) data downloaded July 21, 2023 via https://www.climatologylab.org/wget-terraclimate.html); and,
>
> (B) 'CHELSA' (ref.[1373]: Karger, D.N., Wilson, A.M., Mahony, C., Zimmermann, N.E., Jetz, W. Global daily 1km land surface precipitation based on cloud cover-informed downscaling. *Scientific Data* doi.org/10.1038/s41597-021-01084-6 (2021) see: https://chelsa-climate.org)

We calculated annual precipitation rates for each of our aquifers by aggregating all grid cells within the boundaries of each aquifer system, and summing up values on an annual time step (to calculate an aquifer-scale annual precipitation rate for each calendar year for each aquifer system; units of mm/year). Next, we calculated a four-decade mean precipitation rate for each aquifer system (see maps to follow; 'CHELSA' four-decade average precipitation rates are based on calendar years 1980-2018, whereas 'TerraClimate' data are based on calendar years 1980-2022).

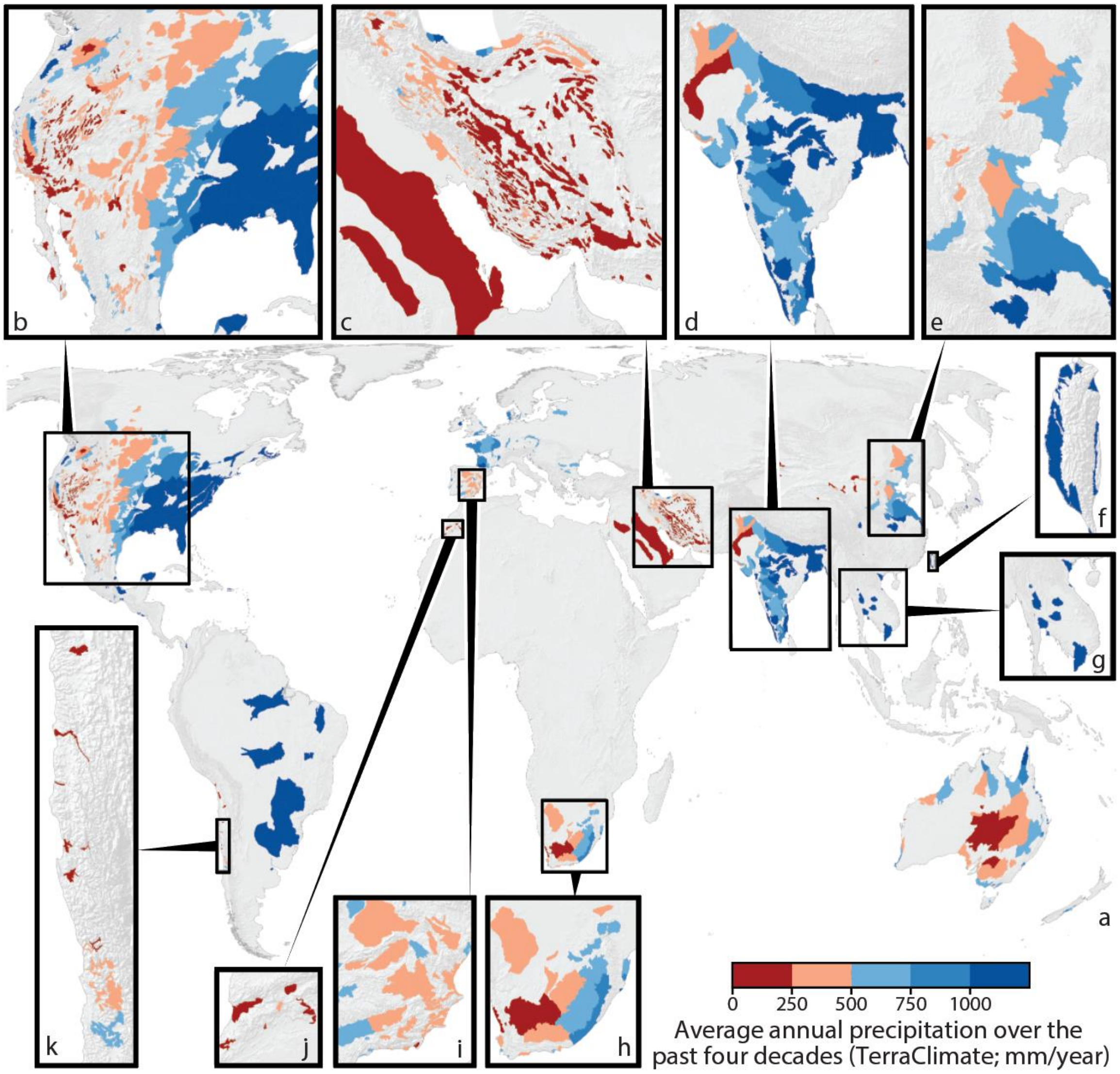

Supplementary Fig. 23. Annual precipitation (mm/year) for each of our study aquifers over the years 1980-2022 (data from 'TerraClimate'; ref.[1372]: Abatzoglou, J.T., Dobrowski, S.Z., Parks, S.A., & Hegewisch, K.C. TerraClimate, a high-resolution global dataset of monthly climate and climatic water balance from 1958–2015. *Scientific Data* **5** (2018). data downloaded July 21, 2023 via https://www.climatologylab.org/wget-terraclimate.html). Annual average precipitation rates vary substantially among the 1,693 aquifer systems that we study.

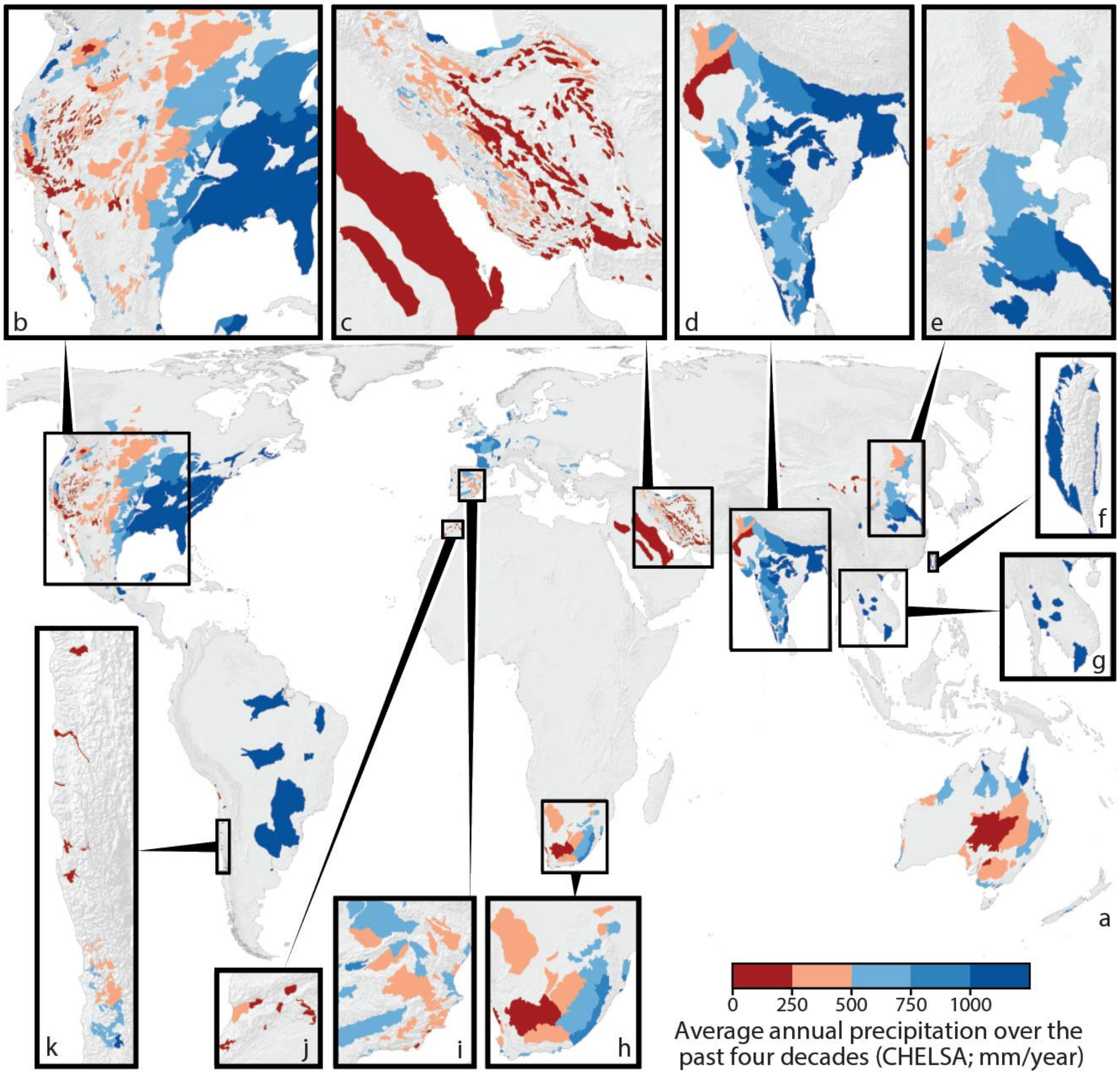

Supplementary Fig. 24. Annual precipitation (mm/year) for each of our study aquifers over the years 1980-2022 (data from 'CHELSA'; ref.[1373]: Karger, D.N., Wilson, A.M., Mahony, C., Zimmermann, N.E., Jetz, W. Global daily 1km land surface precipitation based on cloud cover-informed downscaling. Scientific Data, doi.org/10.1038/s41597-021-01084-6 (2021). see: https://chelsa-climate.org). Annual average precipitation rates vary substantially among the 1,693 aquifer systems that we study.

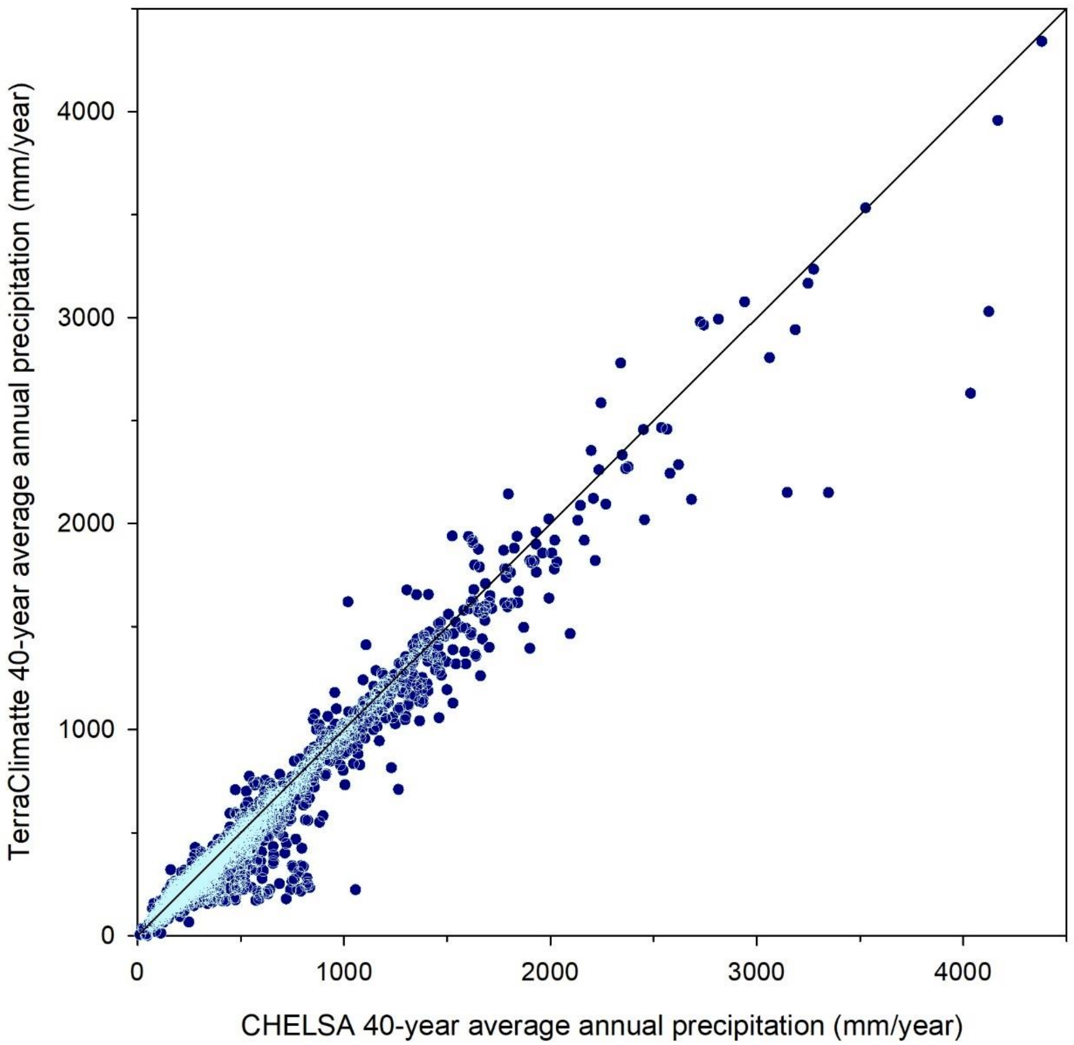

Supplementary Fig. 25. Comparison of aquifer-scale average annual precipitation (mm/year over the past 4 decades) as reported by (i) the 'TerraClimate' dataset, versus (ii) the 'CHELSA' dataset. ('CHELSA' dataset: ref.[1373]: Karger, D.N., Wilson, A.M., Mahony, C., Zimmermann, N.E., Jetz, W. (2021): Global daily 1km land surface precipitation based on cloud cover-informed downscaling. Scientific Data. doi.org/10.1038/s41597-021-01084-6. see: https://chelsa-climate.org; 'TerraClimate' dataset: ref.[1372]: Abatzoglou, J.T., Dobrowski, S.Z., Parks, S.A., & Hegewisch, K.C. TerraClimate, a high-resolution global dataset of monthly climate and climatic water balance from 1958–2015. *Scientific Data* **5** (2018). data downloaded July 21, 2023 via https://www.climatologylab.org/wget-terraclimate.html). Precipitation rates from the two data products are similar for most aquifer systems.

We also calculated average annual precipitation rates over the late 20th century and over the early 21st century. We then compared these two ~20-year precipitation records by dividing the early-21st-century mean annual precipitation rate by the late-20th-century mean annual precipitation rate. The results are presented in the maps to follow (the early-21st-century time interval, over which we calculated an average annual precipitation rate, is 2000-2018 for the 'CHELSA' dataset and 2000-2022 for the 'TerraClimate' dataset; the late-20th-century time interval, over which we calculated an average annual precipitation rate, is 1980-2000 for both 'TerraClimate' and 'CHELSA').

Our comparison of late-20th-century and early-21st-century average annual precipitation rates demonstrates that both the TerraClimate and CHELSA datasets suggest:

- Early-21st-century precipitation rates were lower than late-20th-century precipitation rates for many aquifer systems in (a) the western US, (b) eastern Iran, (c) central Saudi Arabia, (d) central Chile, and (e) southeastern Australia;

- Early-21st-century precipitation rates were higher than late-20th-century precipitation rates in (a) the northeastern US), (b) western Taiwan, (c) southeastern India, and (d) some parts of northern Australia.

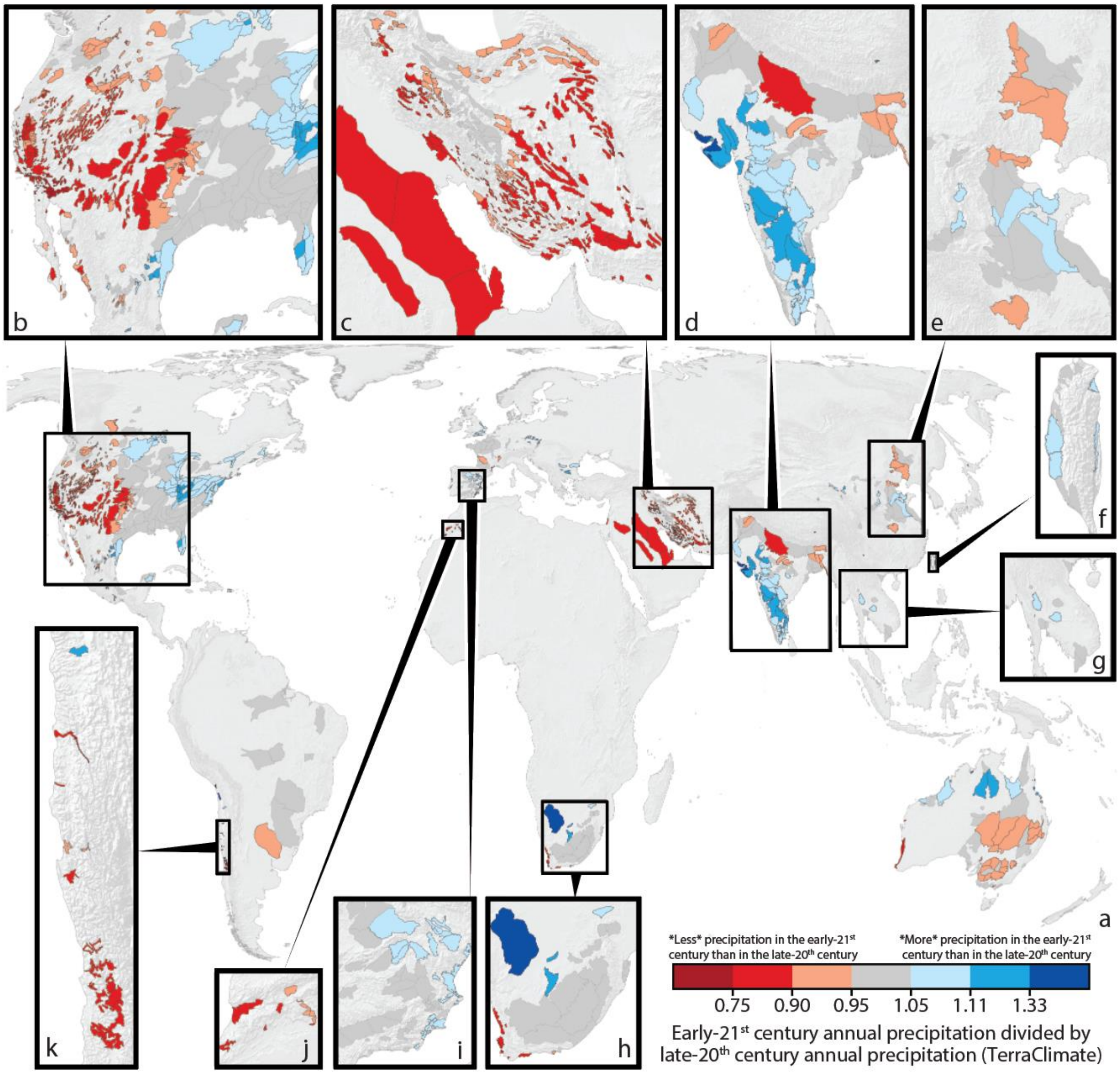

Supplementary Fig. 26. Comparison of aquifer-scale average annual precipitation during the early 21st century (2000-2022) versus the late 20th century (1980-2000) as reported by the 'TerraClimate' dataset ('TerraClimate' dataset: ref.[1372]: Abatzoglou, J.T., Dobrowski, S.Z., Parks, S.A., & Hegewisch, K.C. TerraClimate, a high-resolution global dataset of monthly climate and climatic water balance from 1958–2015. *Scientific Data* **5** (2018). data downloaded July 21, 2023 via https://www.climatologylab.org/wget-terraclimate.html). Aquifer systems where average annual precipitation during the early 21st century was lower than average annual precipitation during the late 20th century are shaded orange or red. Aquifer systems where average annual precipitation during the early 21st century was higher than average annual precipitation during the late 20th century are shaded blue. 'TerraClimate' data suggests that early 21st century annual precipitation was lower than late-20th-century annual precipitation in much of the western US, Iran, central Chile and east-central Australia.

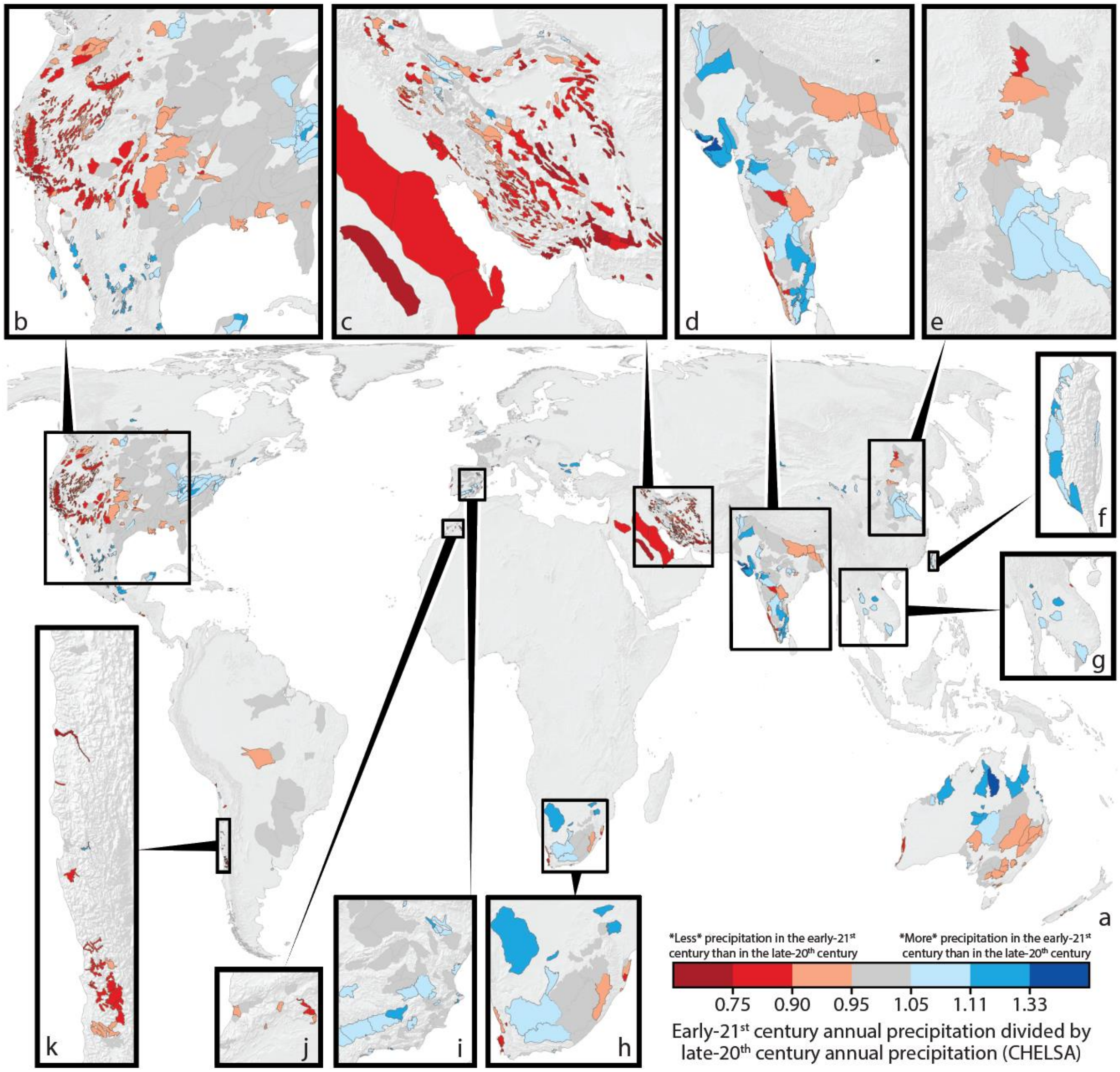

Supplementary Fig. 27. Comparison of aquifer-scale average annual precipitation during the early-21st-century (2000-2022) versus the late-20th-century (1980-2000) as reported by the 'CHELSA' dataset ('CHELSA' dataset: ref.[1373]: Karger, D.N., Wilson, A.M., Mahony, C., Zimmermann, N.E., Jetz, W. (2021): Global daily 1km land surface precipitation based on cloud cover-informed downscaling. Scientific Data. doi.org/10.1038/s41597-021-01084-6. see: https://chelsa-climate.org). Aquifer systems where average annual precipitation during the early 21st century was <u>lower</u> than average annual precipitation during the late 20th century are shaded orange or red. Aquifer systems where average annual precipitation during the early 21st century was <u>higher</u> than average annual precipitation during the late 20th century are shaded blue. 'CHELSA' data suggests that early 21st century annual precipitation was lower than late 20th century annual precipitation in much of the western US, Iran, central Chile and east-central Australia.

To better understand potential spatiotemporal statistical relationships between precipitation variability, we plotted the 542 aquifer systems for which we had adequate data to meet our criteria for juxtaposition of late 20th versus early 21st century aquifer scales in depth to groundwater (i.e., the aquifer systems represented in main text Fig. 3). Specifically, we compare average annual precipitation rates for the early 21st and late 20th centuries (i.e., early-21st-century average annual precipitation divided by late-20th-century average annual precipitation; values on x-axes of supplementary figures to follow) and 21st-century groundwater level trends (aquifer-scale trends calculated as the median of all monitoring well water level trends within the boundaries of an aquifer system; y-axis values in plots to follow). Each plot represents one of the categories presented in the main text (Fig. 3); specifically, these categories are: (i) groundwater levels became shallower during 1980-2000, and continued to become shallower (purple points), (ii) groundwater levels became shallower during 1980-2000, but have since become deeper (yellow points), (iii) groundwater levels became deeper during 1980-2000, but have since become shallower (blue points), (iv) groundwater levels became deeper during 1980-2000, and continued to become deeper but at a slower rate (i.e., decelerated deepening; orange points), and, (v) groundwater levels became deeper during 1980-2000, and continued to become deeper at a faster rate (i.e., accelerated deepening; red points).

Our analysis demonstrates that: (a) the majority (83-90%) of aquifer systems categorized as exhibiting '*accelerated deepening*' (i.e., groundwater levels became deeper during 1980-2000, and continued to become deeper at a faster rate) also received *less precipitation in the early-21st century* than in the late-20th century, and (b) a slight majority (56-62%) of aquifer systems categorized as exhibiting '*recovering*' groundwater levels (i.e., groundwater levels became deeper during 1980-2000, but have since become shallower) received *more precipitation in the early 21st century* than in the late 20th century (see table below and supplementary figures to follow).

Supplementary Table 8. Average annual precipitation in the early 21st versus the late 20th centuries for aquifer systems with adequate data to assess groundwater level trends over the past 4 decades (i.e., those depicted in main text Fig. 3)

Category*	Number of aquifer systems	Percent of aquifer systems where there was *less* precipitation in the early 21st century than in the late 20th century	Percent of aquifer systems where there was *more* precipitation in the early 21st century than in the late 20th century
groundwater levels became **shallower** during 1980-2000, but **have since become deeper** (yellow points),	117	**79% - 85%**	15% - 21%
groundwater levels became deeper during 1980-2000, and continued to become deeper at a faster rate (i.e., **accelerated deepening**; red points)	163	**83% - 90%**	10% - 17%
groundwater levels became deeper during 1980-2000, and continued to become deeper but at a slower rate (i.e., **decelerated deepening**; orange points)	107	**70% - 79%**	21% - 30%
groundwater levels became **deeper** during 1980-2000, but have **since become shallower** (blue points)	86	38% - 44%	**56% - 62%**
groundwater levels became **shallower** during 1980-2000, and **continued to become shallower** (purple points)	69	**55% - 58%**	42% - 45%

* each row corresponds to one of the categories of aquifer-scale groundwater level trends depicted in main text Fig. 3 and in the supplementary figures on the pages to follow. The range of percentages correspond to range of results obtained from two different precipitation time series datasets: (a) 'CHELSA' dataset: ref.[1373]: Karger, D.N., Wilson, A.M., Mahony, C., Zimmermann, N.E., Jetz, W. (2021): Global daily 1km land surface precipitation based on cloud cover-informed downscaling. Scientific Data. doi.org/10.1038/s41597-021-01084-6. see: https://chelsa-climate.org; and (b) 'TerraClimate' dataset: ref.[1372]: Abatzoglou, J.T., Dobrowski, S.Z., Parks, S.A., & Hegewisch, K.C. TerraClimate, a high-resolution global dataset of monthly climate and climatic water balance from 1958–2015. Scientific Data 5 (2018). data downloaded July 21, 2023 via https://www.climatologylab.org/wget-terraclimate.html).

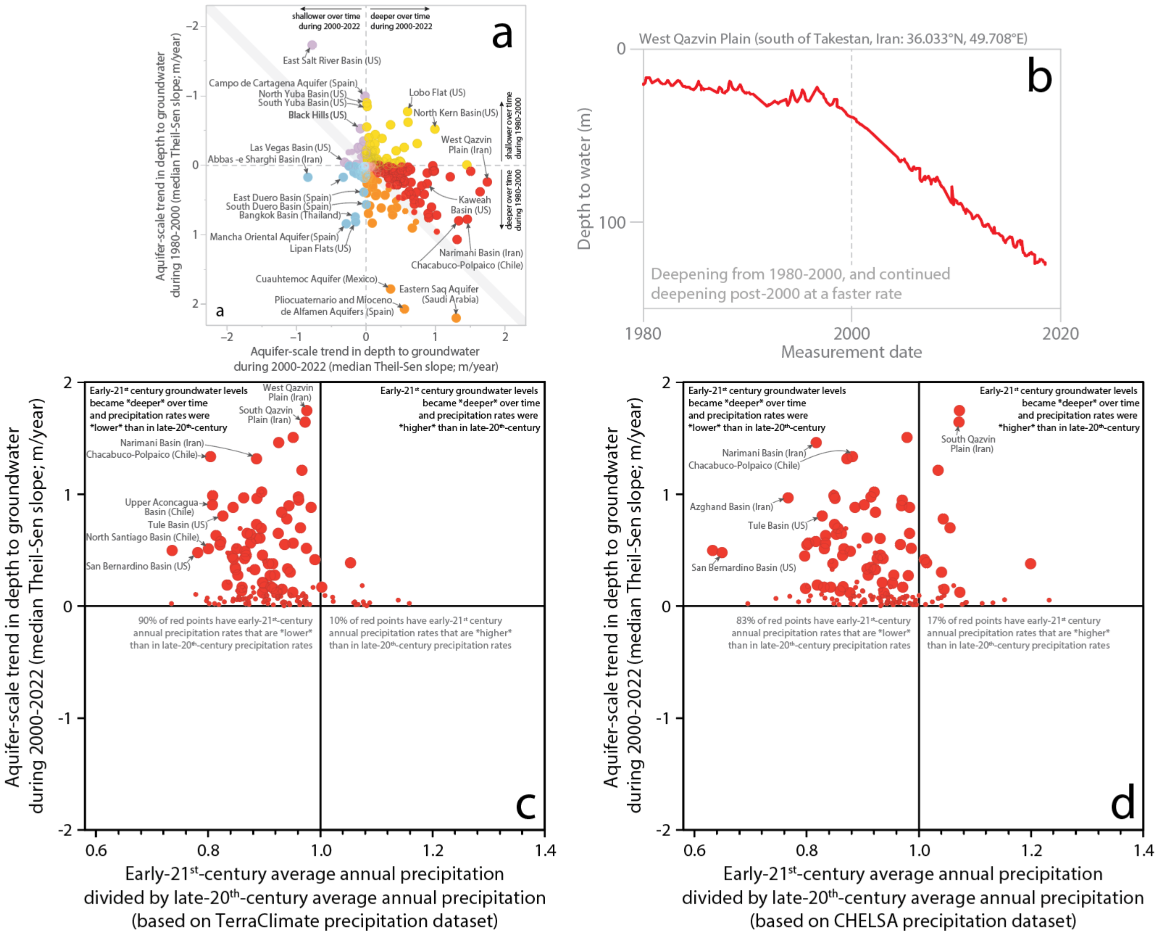

Supplementary Fig. 28. Comparison of trends in depth to groundwater and annual precipitation rates for 'red points'. (a) Comparison of trends in depth to groundwater during two different time intervals (early 21st century and late 20th century). This panel presents identical data and categories as that displayed in main text Fig. 3 (see that figure caption for details). **(b)** An example of a time series in depth to groundwater exhibiting accelerated groundwater level declines (i.e., red points in panel a). **(c-d)** Comparison of average annual precipitation rates for the early 21st and late 20th centuries (i.e., early-21st-century average annual precipitation divided by late-20th-century average annual precipitation; values on x-axes) and 21st-century groundwater level trends (aquifer-scale trends calculated as the median of all monitoring well water level trends within the boundaries of an aquifer system; y-axis values). Each point represents one of our 542 aquifer systems. X-axis values of less than 1 indicate that average annual precipitation during the early 21st century was lower than average annual precipitation during the late 20th century. Panels c and d only display aquifer systems categorized as exhibiting accelerated groundwater level declines (i.e., only the red points in panel a are displayed in panels c and d). The x-axis values in panel c are based on the 'TerraClimate' dataset, whereas the x-axis values in panel d are based on the 'CHELSA' dataset (see text above for full references to these two precipitation data sources). Our analysis of precipitation time series demonstrates that the majority (83-90%) of aquifer systems categorized as exhibiting 'accelerated deepening' (i.e., groundwater levels became deeper during 1980-2000, and continued to become deeper at a faster rate) also received less precipitation in the early 21st century than in the late 20th century.

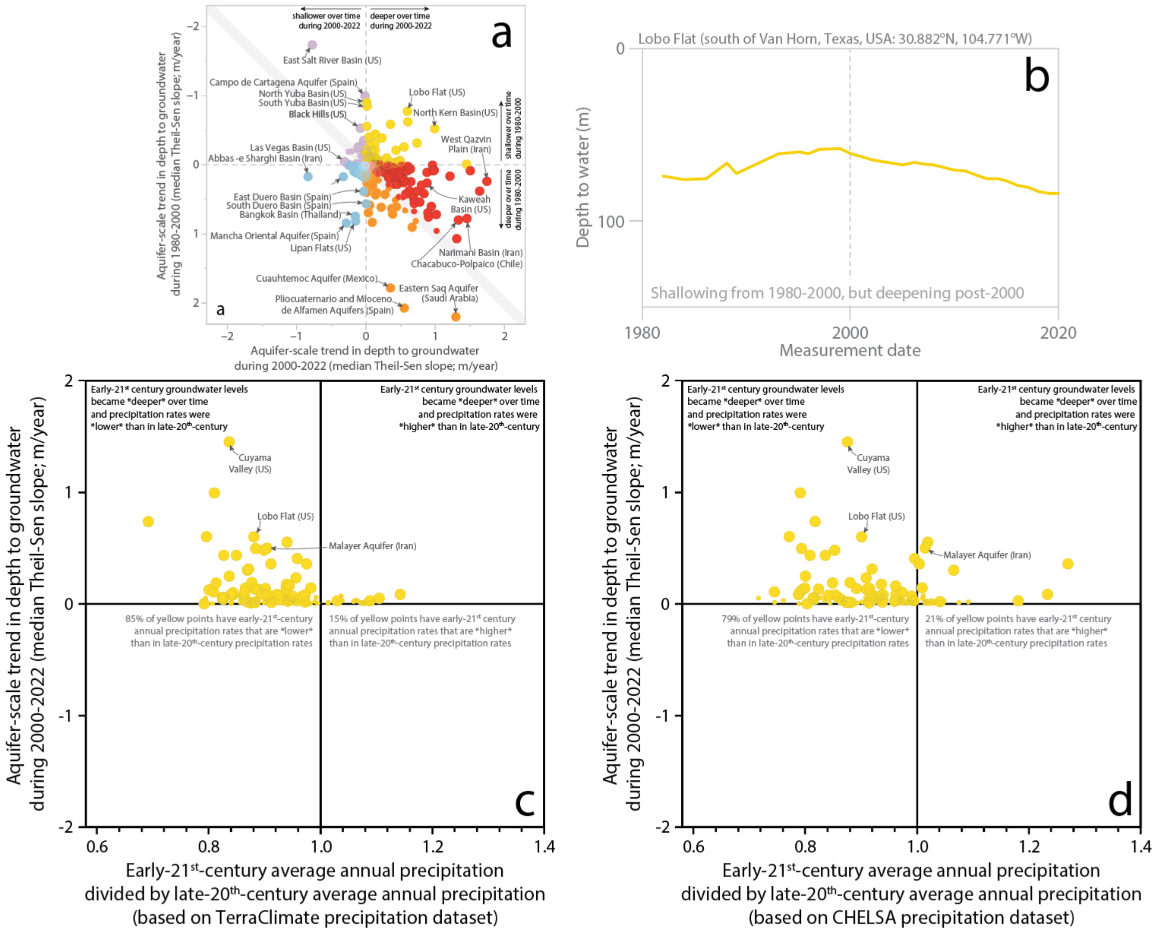

Supplementary Fig. 29. Comparison of trends in depth to groundwater and annual precipitation rates for 'yellow points'. (a) Comparison of trends in depth to groundwater during two different time intervals (early 21st century and late 20th century). This panel presents identical data and categories as that displayed in main text Fig. 3 (see that figure caption for details). **(b)** An example of a time series in depth to groundwater exhibiting shallowing groundwater levels followed by declining groundwater levels (i.e., yellow points in panel a). **(c-d)** Comparison of average annual precipitation rates for the early 21st and late 20th centuries (i.e., early-21st-century average annual precipitation divided by late-20th-century average annual precipitation; values on x-axes) and 21st-century groundwater level trends (aquifer-scale trends calculated as the median of all monitoring well water level trends within the boundaries of an aquifer system; y-axis values). Each point represents one of our 542 aquifer systems. X-axis values of less than 1 indicate that average annual precipitation during the early-21st-century was lower than average annual precipitation during the late 20th century. Panels c and d only display aquifer systems categorized as exhibiting shallowing followed by deepening groundwater level trends (i.e., only the yellow points in panel a are displayed in panels c and d). The x-axis values in panel c are based on the 'TerraClimate' dataset, whereas the x-axis values in panel d are based on the 'CHELSA' dataset (see text above for full references to these two precipitation data sources). Our analysis of precipitation time series demonstrates that the majority (79-85%) of aquifer systems shown here (i.e., groundwater levels became shallower during 1980-2000, but have since become deeper) also received less precipitation in the early 21st century than in the late 20th century.

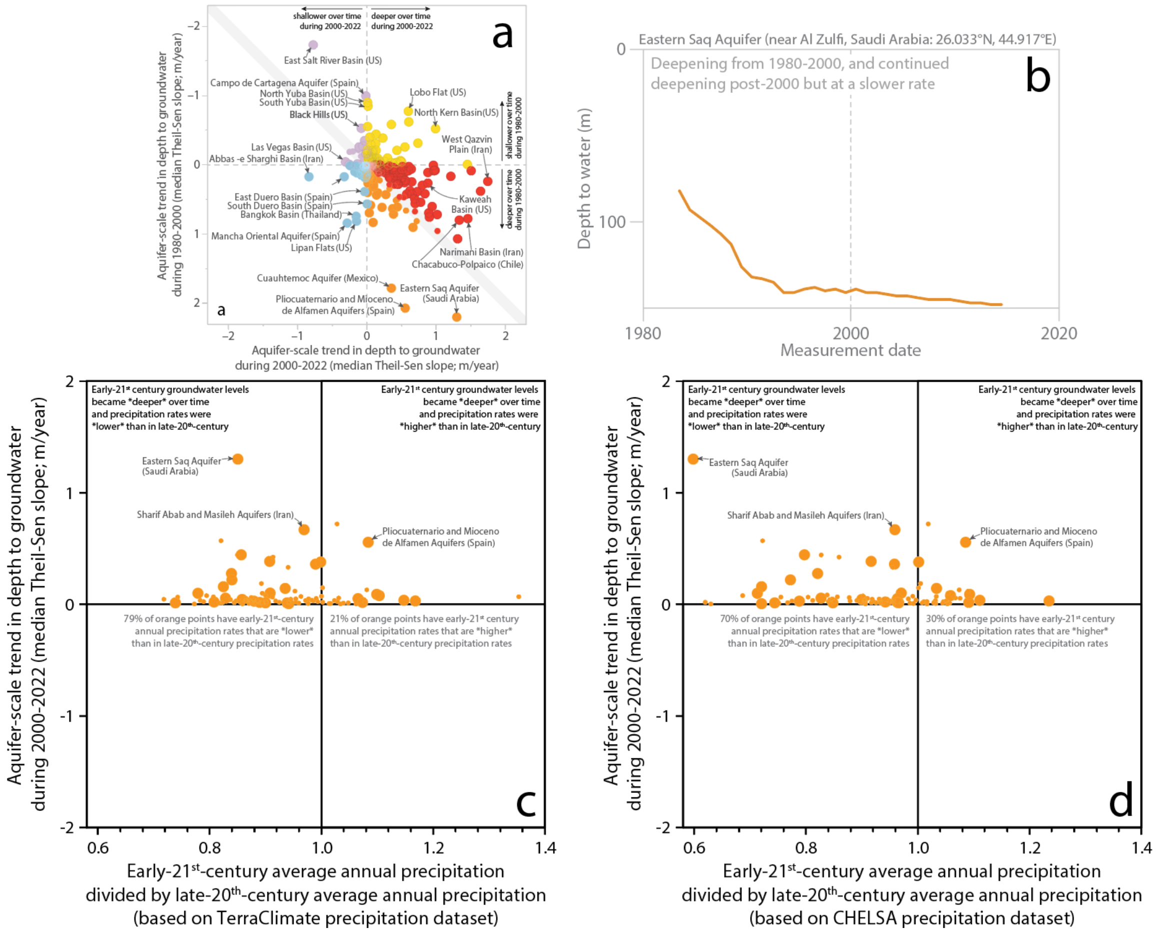

Supplementary Fig. 30. Comparison of trends in depth to groundwater and annual precipitation rates for 'orange points'. (a) Comparison of trends in depth to groundwater during two different time intervals (early 21st century and late 20th century). This panel presents identical data and categories as that displayed in main text Fig. 3 (see that figure caption for details). **(b)** An example of a time series in depth to groundwater exhibiting decelerated groundwater level declines (i.e., orange points in panel a). **(c-d)** Comparison of average annual precipitation rates for the early 21st and late 20th centuries (i.e., early-21st-century average annual precipitation divided by late-20th-century average annual precipitation; values on x-axes) and 21st-century groundwater level trends (aquifer-scale trends calculated as the median of all monitoring well water level trends within the boundaries of an aquifer system; y-axis values). Each point represents one of our 542 aquifer systems. X-axis values of less than 1 indicate that average annual precipitation during the early 21st century was <u>lower</u> than average annual precipitation during the late 20th century. Panels c and d only display aquifer systems categorized as exhibiting decelerated groundwater level declines (i.e., only the orange points in panel a are displayed in panels c and d). The x-axis values in panel c are based on the 'TerraClimate' dataset, whereas the x-axis values in panel d are based on the 'CHELSA' dataset (see text above for full references to these two precipitation data sources). Our analysis of precipitation time series demonstrates that the majority (70-79%) of aquifer systems categorized as exhibiting decelerated groundwater level deepening (i.e., groundwater levels became deeper during 1980-2000, and continued to become deeper but at a slower rate) also received <u>less</u> precipitation in the early 21st century than in the late 20th century.

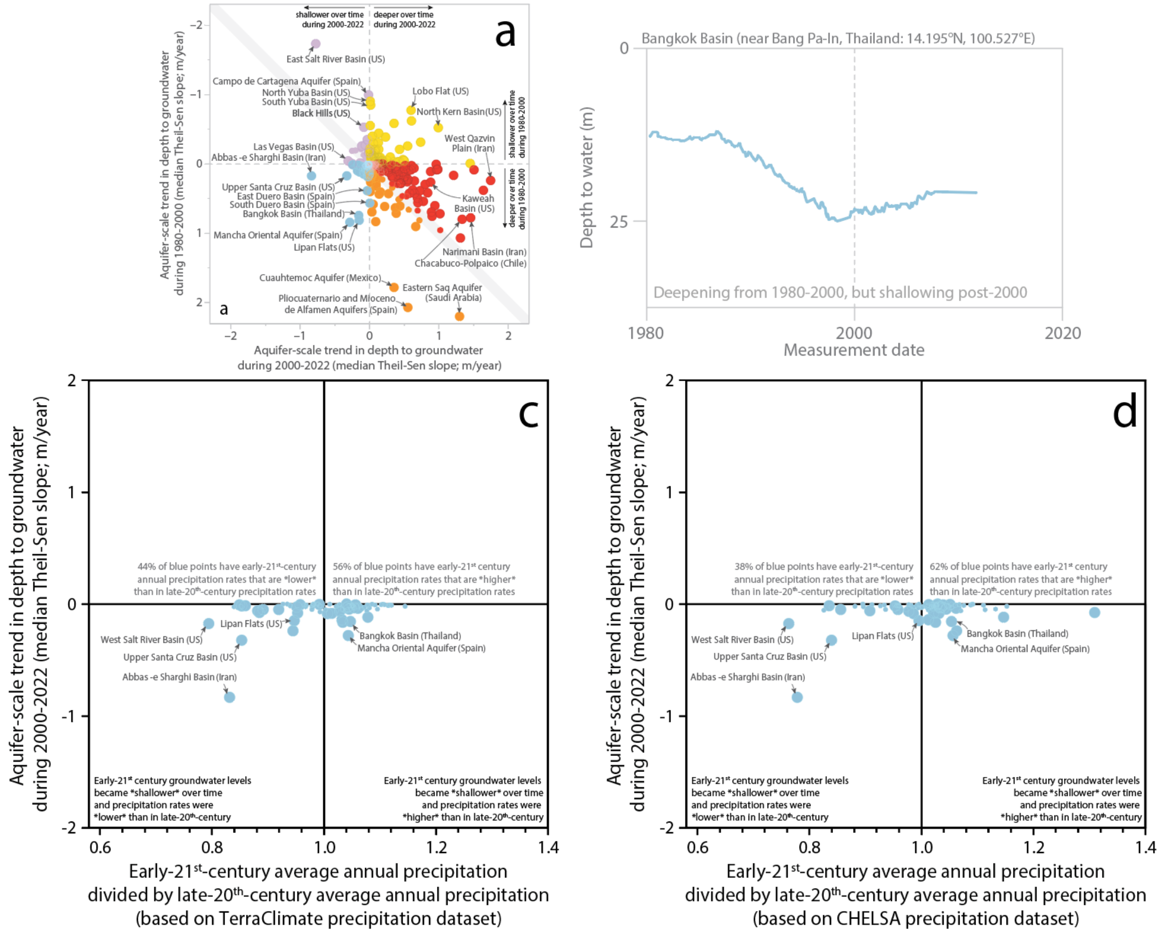

Supplementary Fig. 31. Comparison of trends in depth to groundwater and annual precipitation rates for 'blue points'. (a) Comparison of trends in depth to groundwater during two different time intervals (early 21st century and late 20th century). This panel presents identical data and categories as that displayed in main text Fig. 3 (see that figure caption for details). **(b)** An example of a time series in depth to groundwater exhibiting recovering groundwater levels (i.e., blue points in panel a). **(c-d)** Comparison of average annual precipitation rates for the early 21st and late 20th centuries (i.e., early-21st-century average annual precipitation divided by late-20th-century average annual precipitation; values on x-axes) and 21st-century groundwater level trends (aquifer-scale trends calculated as the median of all monitoring well water level trends within the boundaries of an aquifer system; y-axis values). Each point represents one of our 542 aquifer systems. X-axis values of less than 1 indicate that average annual precipitation during the early 21st century was lower than average annual precipitation during the late 20th century. Panels c and d only display aquifer systems categorized as exhibiting recovering groundwater level trends (i.e., only the blue points in panel a are displayed in panels c and d). The x-axis values in panel c are based on the 'TerraClimate' dataset, whereas the x-axis values in panel d are based on the 'CHELSA' dataset (see text above for full references to these two precipitation data sources). Our analysis of precipitation time series demonstrates that the slight majority (56-62%) of aquifer systems categorized as exhibiting recovering groundwater levels (i.e., groundwater levels became deeper during 1980-2000, but have since become shallower) also received more precipitation in the early 21st century than in the late 20th century.

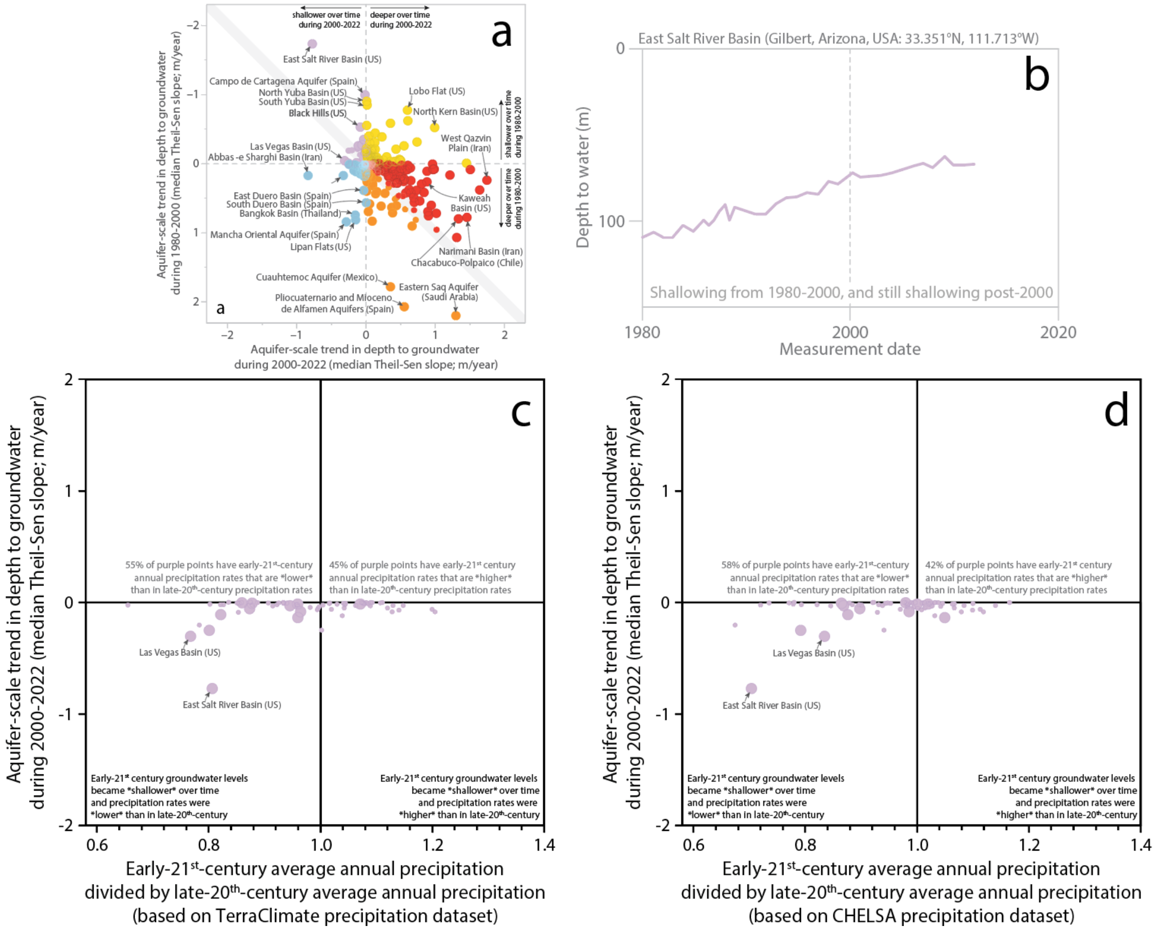

Supplementary Fig. 32. Comparison of trends in depth to groundwater and annual precipitation rates for 'purple points'. (a) Comparison of trends in depth to groundwater during two different time intervals (early 21st century and late 20th century). This panel presents identical data and categories as that displayed in main text Fig. 3 (see that figure caption for details). **(b)** An example of a time series in depth to groundwater exhibiting continually shallowing groundwater levels (i.e., exemplifying purple points in panel a). **(c-d)** Comparison of average annual precipitation rates for the early 21st and late 20th centuries (i.e., early-21st-century average annual precipitation divided by late-20th-century average annual precipitation; values on x-axes) and 21st-century groundwater level trends (aquifer-scale trends calculated as the median of all monitoring well water level trends within the boundaries of an aquifer system; y-axis values). Each point represents one of our 542 aquifer systems. X-axis values of less than 1 indicate that average annual precipitation during the early-21st-century was <u>lower</u> than average annual precipitation during the late 20th century. Panels c and d only display aquifer systems categorized as exhibiting continually shallowing groundwater levels (i.e., only the purple points in panel a are displayed in panels c and d). The x-axis values in panel c are based on the 'TerraClimate' dataset, whereas the x-axis values in panel d are based on the 'CHELSA' dataset (see text above for full references to these two precipitation data sources). Our analysis of precipitation time series demonstrates that the slight majority (55-58%) of aquifer systems categorized as exhibiting continually shallowing trends (i.e., groundwater levels became shallower during 1980-2000, and continued to become shallower) also received <u>less</u> precipitation in the early 21st century than in the late 20th century.

11 RAPID GROUNDWATER LEVEL DEEPENING MOST COMMON IN CULTIVATED AREAS AND IN DRIER CLIMATES

We compared 21st century aquifer-scale trends in depth to groundwater to (i) average annual precipitation divided by potential evapotranspiration, and (ii) the percentage of land under cultivation.

> (i) Among all aquifer-scale trends demonstrating that groundwater levels became deeper over time at a rate of faster than 0.5 m/year, 99% (208 of 210 aquifer systems) are in hyper-arid, arid or semi-arid climate zones (where annual precipitation divided by annual potential evapotranspiration values of less than 0.5; Supplementary Table 9).
>
> (ii) Among all aquifer-scale trends demonstrating that groundwater levels became deeper over time at a rate of faster than 0.5 m/year, 64.3% (135 of 210 aquifer systems) underlie areas where more than 20% of lands are under cultivation (Supplementary Table 10).

In our main text we report a statistical relationship between trends in depth to groundwater and climatic aridity. We also report a statistical relationship between trends in depth to groundwater and cultivation. Climatic aridity and the prevalence of cultivation are inter-related variables. Therefore, we used multiple regression on their rank transforms to estimate what each of their rank correlations with groundwater-level deepening would be, if the other variable could be held constant. The corresponding partial rank correlation coefficients

are 0.32 for the fraction of land area devoted to crop cultivation, and −0.48 for precipitation divided by potential evapotranspiration. Even though these statistical relationships are highly significant, we stress that substantial variance remains unexplained (coefficient of determination R^2 = 0.25), suggesting that other physiographic, environmental and land-use characteristics may also influence 21st-century groundwater-level trends.

Supplementary Table 9. Percentage of aquifer systems where groundwater levels have deepened faster than 0.5 m/year (median Theil-Sen slope) among aquifer systems classified on the basis of annual precipitation divided by potential evapotranspiration values

Climate classification based on annual precipitation divided by potential evapotranspiration	Percentage of aquifer systems where 21st-century groundwater levels deepened faster than 0.5 m/year (median Theil-Sen slope)
**Hyper-arid**	11% (10 of 87 aquifer systems)
**Arid**	24% (159 of 652 aquifer systems)
**Semi-arid**	8% (39 of 517 aquifer systems)
**Dry subhumid**	0% (0 of 100 aquifer systems)
**Humid**	1% (2 of 337 aquifer systems)

Supplementary Table 10. Percentage of aquifer systems where groundwater levels have deepened faster than 0.5 m/year (median Theil-Sen slope) among aquifer systems classified on the basis of the proportion of land under cultivation

**Classification based on the proportion of land under cultivation**	**Percentage of aquifer systems where 21st-century groundwater levels deepened faster than 0.5 m/year (median Theil-Sen slope)**
**<1% cropland**	1% (2 of 250 aquifer systems)
**1-20% cropland**	11% (73 of 652 aquifer systems)
**>20% cropland**	17% (135 of 791 aquifer systems)

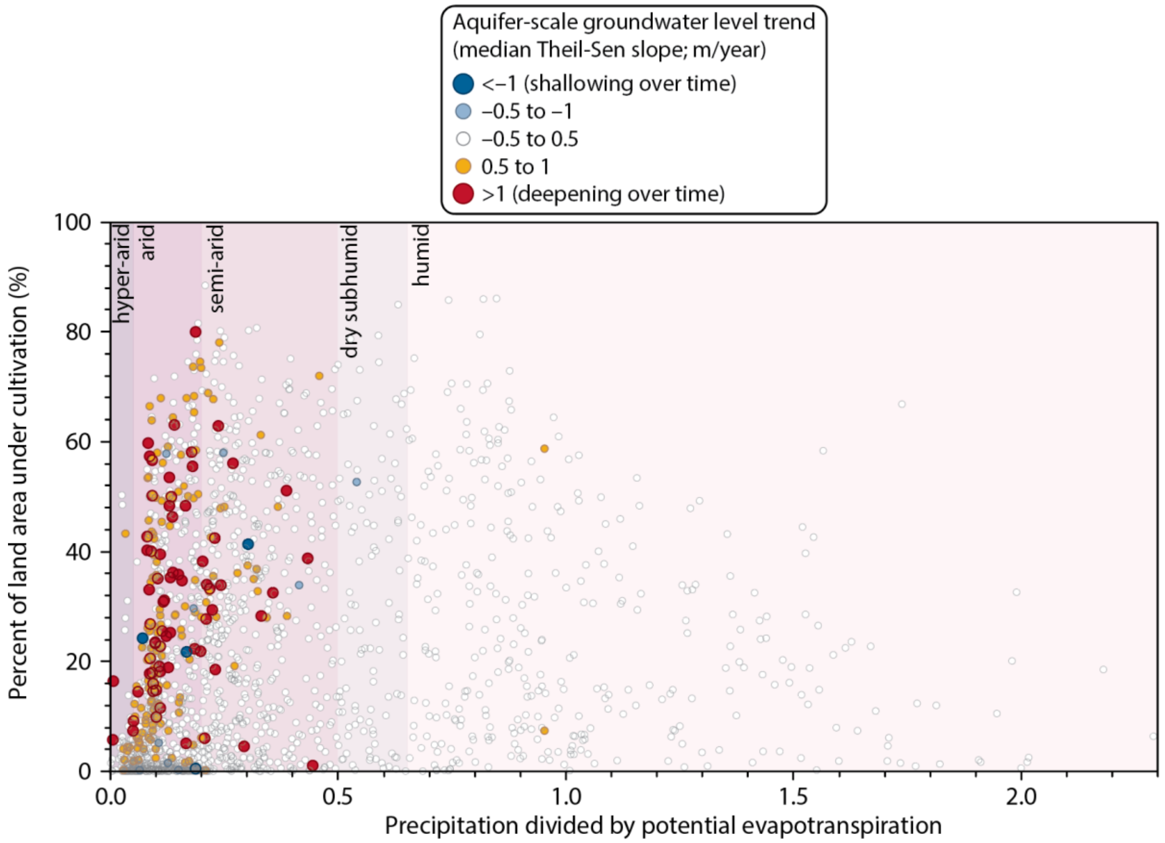

Supplementary Fig. 33. 21st-century trends in depth to groundwater in the context of land use (y-axis values are the proportion of study area represented by cultivated lands) and climate (x-axis values are average long-term annual precipitation divided by potential evapotranspiration). Each point represents one aquifer system. Values along the horizontal axis are average annual precipitation divided by potential evapotranspiration. Values along the vertical axis represent the percentage of the study area covered by cropland as of the year 2015. The colour of each point represents the 21st century aquifer-scale trend in depth to groundwater (median Theil-Sen slope). Orange and red points represent aquifer systems with rapidly deepening groundwater levels (median Theil-Sen slope of >0.5 m/year). Blue points represent aquifer systems with rapidly shallowing groundwater levels (median Theil-Sen slope of <−0.5 m/year). Background shades represent climate zones classified by annual precipitation divided by potential evapotranspiration (i.e., x-axis values), and range from hyper-arid (darker pink background) to humid (lighter pink background). A key difference between this figure and the main text figure are the linear axes (rather than log axes) presented here, which enables us to plot aquifer systems that were excluded from the main text figure.

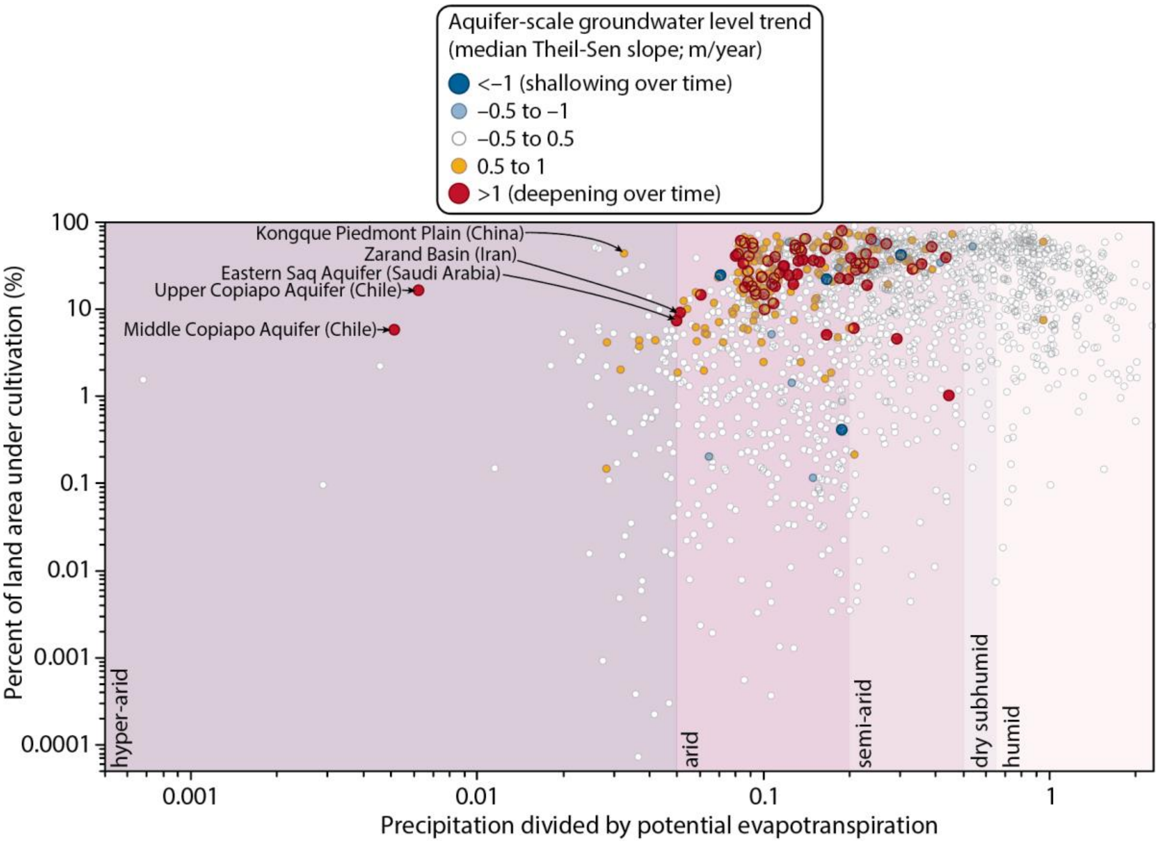

Supplementary Fig. 34. 21st-century trends in depth to groundwater in the context of land use (y-axis values are the proportion of study area represented by cultivated lands) and climate (x-axis values are average long-term annual precipitation divided by potential evapotranspiration). Each point represents one aquifer system. Values along the horizontal axis are average annual precipitation divided by potential evapotranspiration. Values along the vertical axis represent the percentage of the study area covered by cropland as of the year 2015. The colour of each point represents the 21st-century aquifer-scale trend in depth to groundwater (median Theil-Sen slope). Orange and red points represent aquifer systems with rapidly deepening groundwater levels (median Theil-Sen slope of >0.5 m/year). Blue points represent aquifer systems with rapidly shallowing groundwater levels (median Theil-Sen slope of <−0.5 m/year). Background shades represent climate zones classified by annual precipitation divided by potential evapotranspiration (i.e., x-axis values), and range from hyper-arid (darker pink background) to humid (lighter pink background). Three aquifer systems are absent from this plot because no land is under cultivation (i.e., value of 0% incompatible with the log-scale of the y-axis; three aquifer systems). A key difference between this figure and the main text figure is the more expansive range of values on the x-axis, which enables us to plot several aquifer systems that were excluded from the main text figure.

12 SPATIAL VARIATIONS IN MONITORING WELL TIME SERIES DURATIONS AND MEASUREMENT FREQUENCIES

Here we present the following information for each monitoring well that met our criteria for analysis:

- *The proportion of all monitoring wells (for a given database) – meeting our criteria for analyses – with time series that spanning a given calendar year*
- *The number of unique calendar years (postdating December 31, 1999) with at least one water level measurement;*
- *The number of years separating the earliest and most recent unique calendar years (postdating December 31, 1999) with at least one water level measurement; and,*
- *The earliest unique calendar year (postdating December 31, 1999) with at least one water level measurement among monitoring wells meeting our criteria for analyses.*

	2000	2001	2002	2003	2004	2005	2006	2007	2008	2009	2010	2011	2012	2013	2014	2015	2016	2017	2018	2019	2020	2021	2022
Afghanistan	0%	0%	0%	0%	86%	100%	100%	100%	100%	100%	100%	100%	100%	96%	0%	0%	0%	0%	0%	0%	0%	0%	0%
Argentina	0%	0%	0%	0%	0%	0%	0%	0%	0%	0%	0%	52%	78%	79%	100%	100%	100%	100%	100%	100%	99%	99%	97%
Australia	71%	75%	80%	83%	86%	88%	90%	92%	94%	93%	93%	92%	86%	79%	73%	70%	67%	63%	56%	47%	43%	35%	0%
Austria	0%	0%	0%	0%	0%	0%	0%	0%	100%	100%	100%	100%	100%	100%	100%	100%	100%	98%	98%	98%	0%	0%	0%
Bangladesh	100%	100%	100%	100%	100%	100%	100%	100%	100%	100%	100%	100%	100%	99%	99%	96%	0%	0%	0%	0%	0%	0%	0%
Belgium	3%	6%	6%	7%	8%	10%	11%	11%	11%	12%	13%	93%	97%	100%	100%	100%	100%	100%	98%	96%	87%	87%	0%
Brazil	0%	0%	0%	0%	0%	0%	0%	0%	10%	10%	45%	100%	100%	100%	100%	100%	100%	100%	99%	87%	0%	0%	0%
Bulgaria	0%	0%	0%	0%	0%	0%	0%	0%	0%	0%	71%	78%	100%	100%	100%	100%	100%	100%	100%	97%	93%	0%	0%
Burkina Faso	92%	92%	92%	100%	100%	100%	100%	100%	100%	100%	100%	100%	100%	100%	100%	83%	58%	33%	0%	0%	0%	0%	0%
Canada	50%	58%	66%	75%	77%	80%	83%	88%	91%	92%	96%	96%	95%	90%	89%	85%	79%	66%	62%	38%	0%	0%	0%
Chile	85%	89%	96%	99%	99%	99%	100%	100%	100%	100%	100%	100%	99%	97%	94%	87%	70%	55%	3%	0%	0%	0%	0%
China	0%	0%	0%	0%	0%	100%	100%	100%	100%	100%	100%	100%	100%	100%	71%	71%	71%	0%	0%	0%	0%	0%	0%
Colombia	0%	0%	17%	67%	67%	67%	67%	67%	100%	100%	100%	100%	100%	100%	100%	100%	100%	100%	100%	83%	0%	0%	0%
Czech Republic	99%	99%	99%	99%	99%	99%	99%	100%	100%	100%	100%	100%	100%	100%	100%	100%	100%	100%	100%	100%	99%	0%	0%
Denmark	63%	66%	69%	70%	81%	85%	85%	85%	85%	83%	91%	87%	85%	42%	42%	42%	42%	42%	41%	41%	40%	0%	0%
El Salvador	0%	0%	0%	0%	0%	0%	0%	0%	0%	0%	0%	0%	6%	98%	100%	100%	100%	100%	100%	100%	100%	100%	2%
France	56%	61%	65%	69%	73%	79%	81%	85%	88%	90%	94%	95%	96%	94%	87%	86%	86%	85%	84%	81%	77%	0%	0%
Germany	65%	88%	100%	100%	100%	100%	100%	100%	100%	100%	100%	92%	92%	62%	62%	62%	62%	58%	58%	0%	0%	0%	0%
Guam	100%	100%	100%	100%	100%	100%	100%	100%	100%	100%	100%	100%	100%	100%	100%	100%	100%	100%	100%	0%	0%	0%	0%
India	0%	0%	0%	0%	0%	0%	0%	91%	99%	100%	100%	100%	100%	100%	100%	100%	96%	90%	0%	0%	0%	0%	0%
Iran	27%	33%	65%	71%	75%	80%	84%	88%	91%	94%	97%	98%	97%	96%	95%	94%	92%	90%	88%	82%	0%	0%	0%
Ireland	4%	4%	4%	5%	5%	5%	5%	5%	59%	93%	95%	100%	100%	100%	100%	100%	100%	100%	100%	100%	0%	0%	0%
Israel	0%	0%	0%	0%	0%	0%	0%	0%	0%	0%	88%	100%	100%	100%	100%	100%	100%	100%	100%	100%	100%	100%	100%
Japan	65%	65%	76%	83%	84%	92%	95%	95%	95%	91%	93%	92%	91%	90%	83%	72%	72%	69%	65%	64%	57%	13%	0%
Latvia	0%	0%	0%	0%	0%	0%	0%	0%	0%	0%	0%	0%	100%	100%	100%	100%	100%	100%	100%	100%	100%	0%	0%
Mexico	39%	53%	66%	80%	88%	93%	95%	100%	100%	99%	96%	91%	76%	62%	47%	30%	10%	0%	0%	0%	0%	0%	0%
Morocco	98%	98%	99%	99%	99%	100%	100%	100%	100%	97%	96%	75%	70%	69%	68%	64%	61%	40%	11%	11%	11%	4%	0%
New Zealand	58%	64%	73%	85%	95%	96%	97%	99%	99%	98%	97%	92%	86%	80%	74%	70%	65%	59%	44%	42%	35%	0%	0%
Norway	72%	76%	83%	90%	93%	95%	97%	98%	98%	98%	97%	98%	93%	91%	90%	90%	88%	88%	86%	83%	0%	0%	0%
Pakistan	0%	0%	0%	45%	45%	76%	95%	95%	97%	99%	100%	100%	95%	92%	89%	81%	37%	30%	28%	0%	0%	0%	0%
Portugal	94%	100%	100%	100%	100%	100%	100%	100%	100%	100%	100%	100%	94%	0%	0%	0%	0%	0%	0%	0%	0%	0%	0%
Saudi Arabia	96%	96%	98%	98%	98%	98%	98%	100%	100%	100%	96%	96%	96%	93%	93%	78%	24%	0%	0%	0%	0%	0%	0%
Slovenia	0%	0%	0%	0%	0%	0%	0%	0%	0%	0%	0%	0%	100%	100%	100%	100%	100%	100%	100%	100%	100%	100%	0%
South Africa	35%	48%	56%	63%	72%	79%	86%	90%	95%	95%	95%	95%	93%	90%	87%	86%	83%	79%	76%	60%	38%	0%	0%
Spain	41%	49%	55%	56%	61%	65%	68%	88%	100%	98%	96%	95%	94%	93%	88%	87%	77%	0%	0%	0%	0%	0%	0%
Sweden	92%	93%	95%	96%	97%	97%	99%	99%	99%	99%	99%	98%	97%	97%	95%	94%	90%	84%	78%	65%	0%	0%	0%
Switzerland	0%	0%	6%	13%	13%	13%	16%	16%	84%	84%	100%	100%	100%	100%	100%	100%	100%	100%	100%	100%	97%	0%	0%
Taiwan	59%	65%	69%	71%	74%	79%	80%	85%	93%	95%	97%	98%	97%	97%	97%	94%	94%	63%	53%	49%	46%	0%	0%
Thailand	8%	8%	8%	9%	23%	25%	35%	66%	87%	89%	96%	100%	98%	98%	98%	97%	96%	95%	92%	91%	89%	0%	0%
United Kingdom	100%	100%	100%	100%	100%	100%	100%	100%	100%	100%	100%	100%	100%	100%	100%	100%	100%	100%	100%	95%	84%	0%	0%
USA	27%	38%	55%	61%	66%	79%	83%	86%	89%	90%	90%	89%	86%	82%	76%	69%	63%	59%	55%	50%	45%	41%	34%
Vietnam	0%	0%	0%	57%	63%	64%	64%	68%	70%	81%	88%	99%	100%	100%	100%	98%	98%	98%	97%	97%	95%	93%	65%

Supplementary Fig. 35. The proportion (in %) of all monitoring wells (meeting our criteria for analyses) with time series that span a given calendar year. Each row represents a dataset (specified in leftmost column). The years at the top of the figure (i.e., top row) specify a given calendar year. The percent values (with green shading marking higher values) represent the proportion of all monitoring wells (for a given database) – meeting our criteria for analyses – with time series that span a given calendar year.

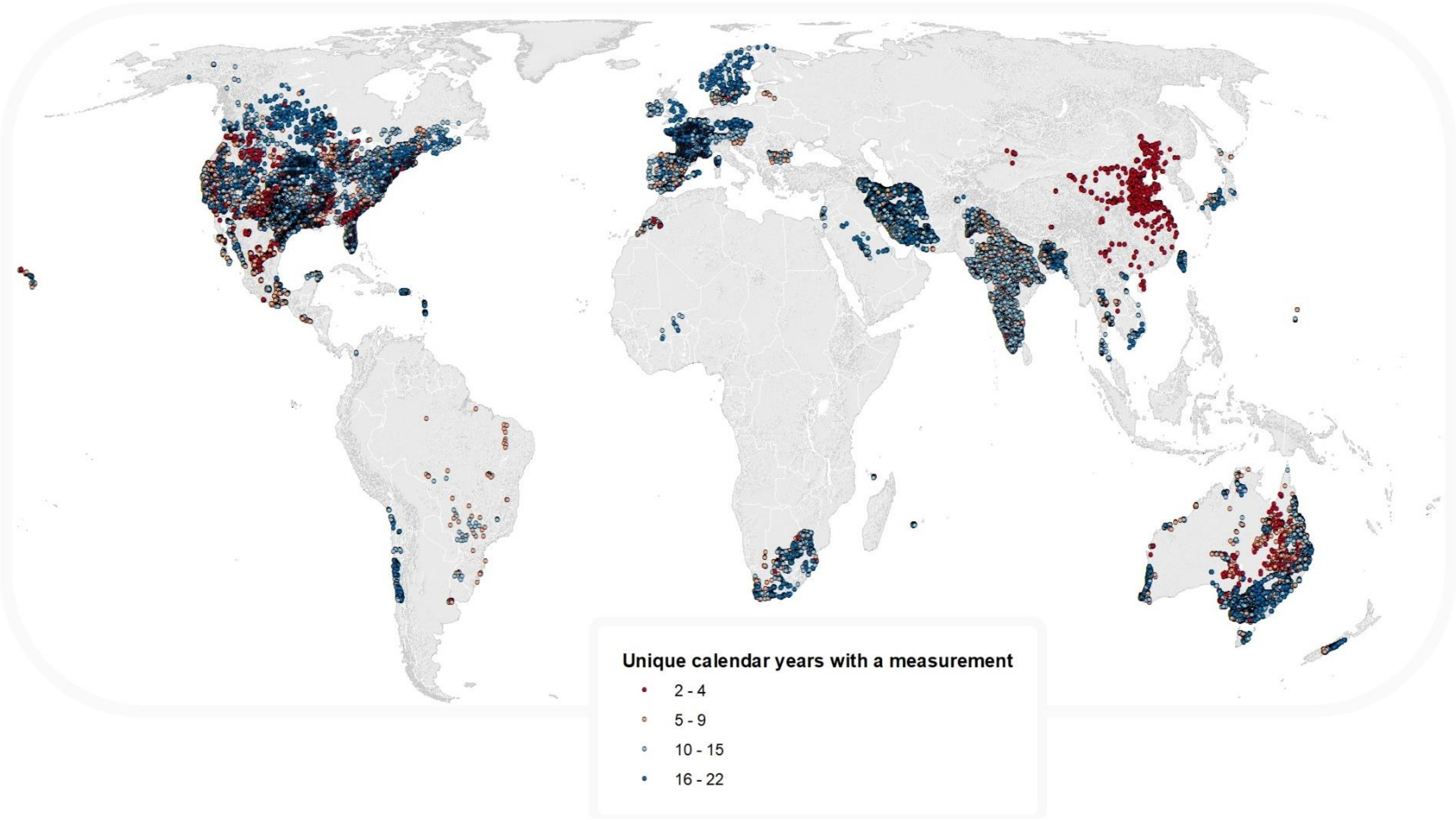

Supplementary Fig. 36. The number of unique calendar years (postdating December 31, 1999) with at least one water level measurement among monitoring wells meeting our criteria for analyses. Each circle represents one monitoring well meeting our criteria for analyses. Blue (light and dark shades) points represent wells with 10 or more calendar years with at least one water level measurement. Orange and red points represent wells with fewer than 10 unique calendar years with at least one measurement.

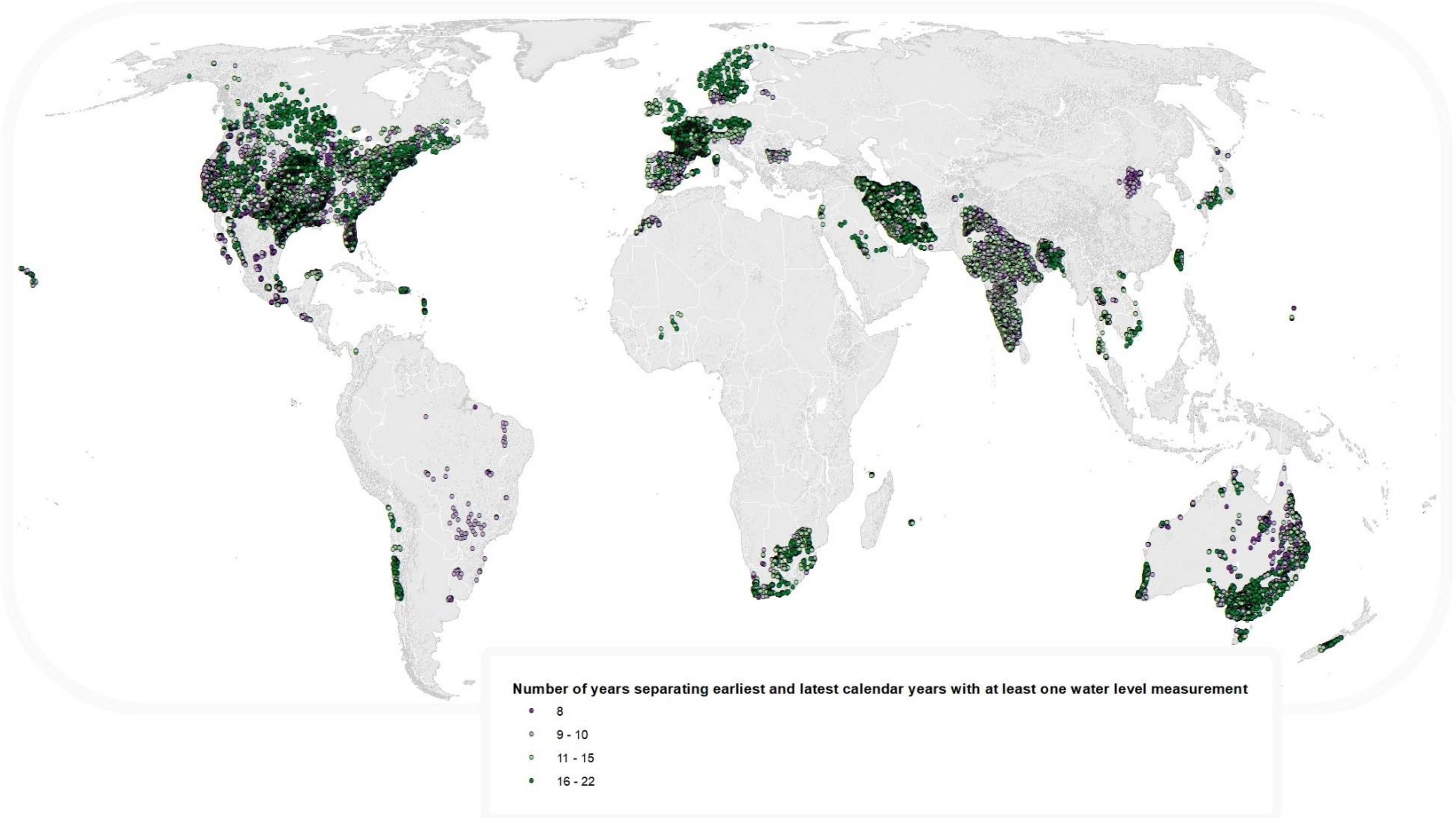

Supplementary Fig. 37. The number of years separating the earliest and most-recent unique calendar years (postdating December 31, 1999) with at least one water level measurement among monitoring wells meeting our criteria for analyses. Each circle represents one monitoring well. Green (light and dark shades) points represent monitoring wells with more than 10 years separating the earliest and most-recent unique calendar years in the time series. Purple (light and dark shades) points represent monitoring wells with 10 or fewer calendar years separating the earliest and most recent unique calendar years with at least one measurement in the time series.

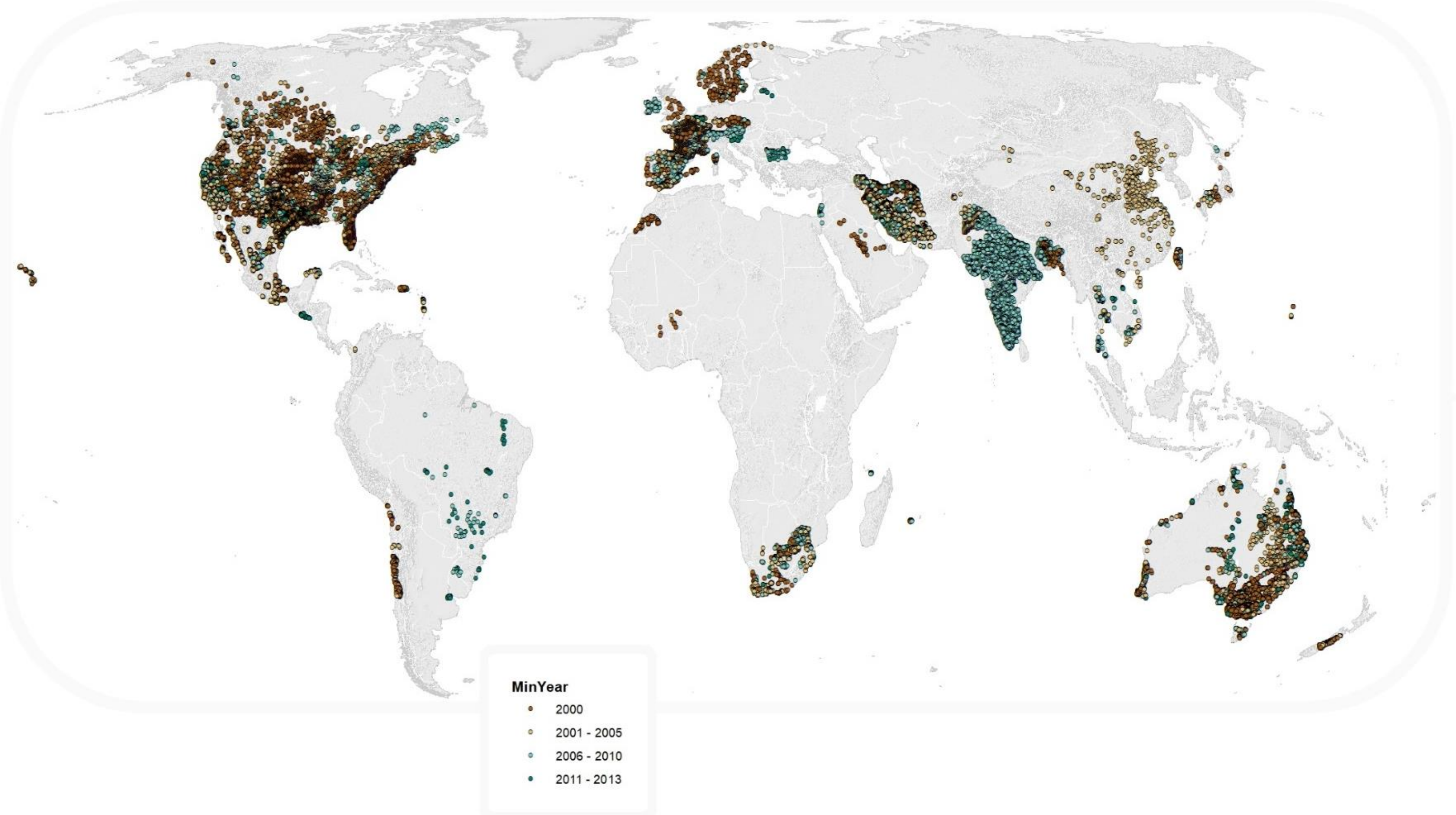

Supplementary Fig. 38. The earliest unique calendar year (postdating December 31, 1999) with at least one water level measurement among monitoring wells meeting our criteria for analyses. Each circle represents one monitoring well. Brown (light and dark shades) points represent monitoring wells with at least one water level measurement prior to the year 2006. Teal (light and dark shades) points represent monitoring wells where the earliest water level measurement in the time series falls after December 31, 2005.

13 REGRESSION ANALYSES IF WE INCLUDE OUTLIERS IDENTIFIED BY THE MACHINE LEARNING ALGORITHM

The results presented in the main text are based on the exclusion of some well water level measurements defined to be outliers because they fall outside of the range defined by the machine-learning-model-predicted groundwater level ±0.75 times its confidence interval (for schematic, see Supplementary Note 2). The figure below compares the aquifer-scale median trends in depth to groundwater presented in the main text (i.e., after excluding points defined to be outliers on the basis of the machine-learning-algorithm as described in the main text methods) versus aquifer-scale median trends in depth to groundwater as determined without applying the machine-learning-algorithm (i.e., *including* points and time series that were excluded from our main text results on the basis of the machine learning algorithm).

The plot below demonstrates a very close correspondence of aquifer-scale trends in depth to groundwater after excluding values on the basis of a machine-learning-algorithm and its predicted groundwater level time series (x-axis values below) and aquifer-scale trends in depth to groundwater if no such machine-learning-based exclusion approach is applied. Specifically, all but 3 of our 1,693 aquifer systems (i.e., >99% of aquifer systems) have aquifer-scale trends in depth to groundwater that differ by less than 0.1 m/year between the following two scenarios: (i) after excluding points defined to be outliers on the basis of the machine-learning-algorithm as described in the main text methods, versus (ii) without applying the machine-learning-

algorithm (i.e., *including* points and time series that were excluded from our main text results on the basis of the machine learning algorithm).

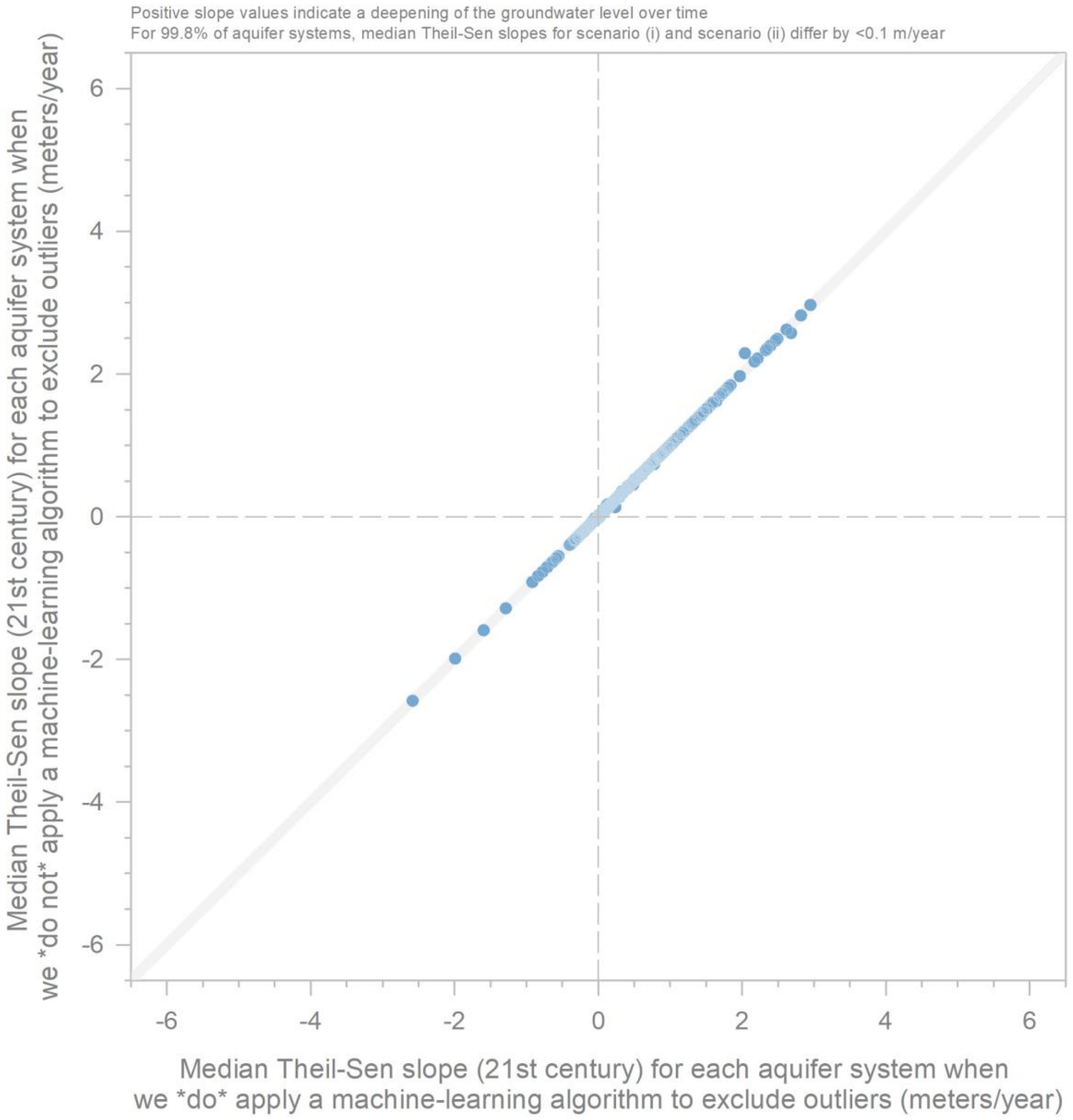

Supplementary Fig. 39. Aquifer-scale trends in depth to groundwater (median Theil-Sen slopes) when we either (i) exclude points defined to be outliers on the basis of the machine-learning-algorithm as described in the main text methods (x-axis values), versus (ii) do <u>not</u> apply a machine-learning-algorithm to exclude points (i.e., *including* points and time series that were excluded from our main text results on the basis of the machine learning algorithm). Each circle represents one aquifer system. Points within the light grey area shaded area have x-axis and the y-axis values that differ by less than 0.1 m/year. 99.8% of aquifer-scale trends in depth to groundwater differ by less than 0.1 m/year under scenario (i) versus scenario (ii).

14 SIMULATED GROUNDWATER DEPLETION FROM A HYDROLOGIC MODEL

We present spatial patterns of simulated groundwater depletion estimated for the years 1960-2010; simulated groundwater depletion data are from ref.[16] (i.e., de Graaf, I.E., Gleeson, T., Van Beek, L.P.H., Sutanudjaja, E.H., & Bierkens, M.F.P. Environmental flow limits to global groundwater pumping. *Nature* **574,** 90-94 (2019); results are available for areas described in ref.[1312]: de Graaf, I.D., Sutanudjaja, E.H., Van Beek, L.P.H., & Bierkens, M.F.P. A high-resolution global-scale groundwater model. *Hydrology and Earth System Sciences* **19,** 823-837 (2015)).

These simulations suggest that groundwater depletion may have occurred in recent decades in some places where we lack monitoring well data (e.g., Tunisia; supplementary figure on following page). These spatial patterns of simulated groundwater depletion (map below) motivated us to complete a literature review of groundwater level changes over time to complement our compiled monitoring well data (see Supplementary Note 15).

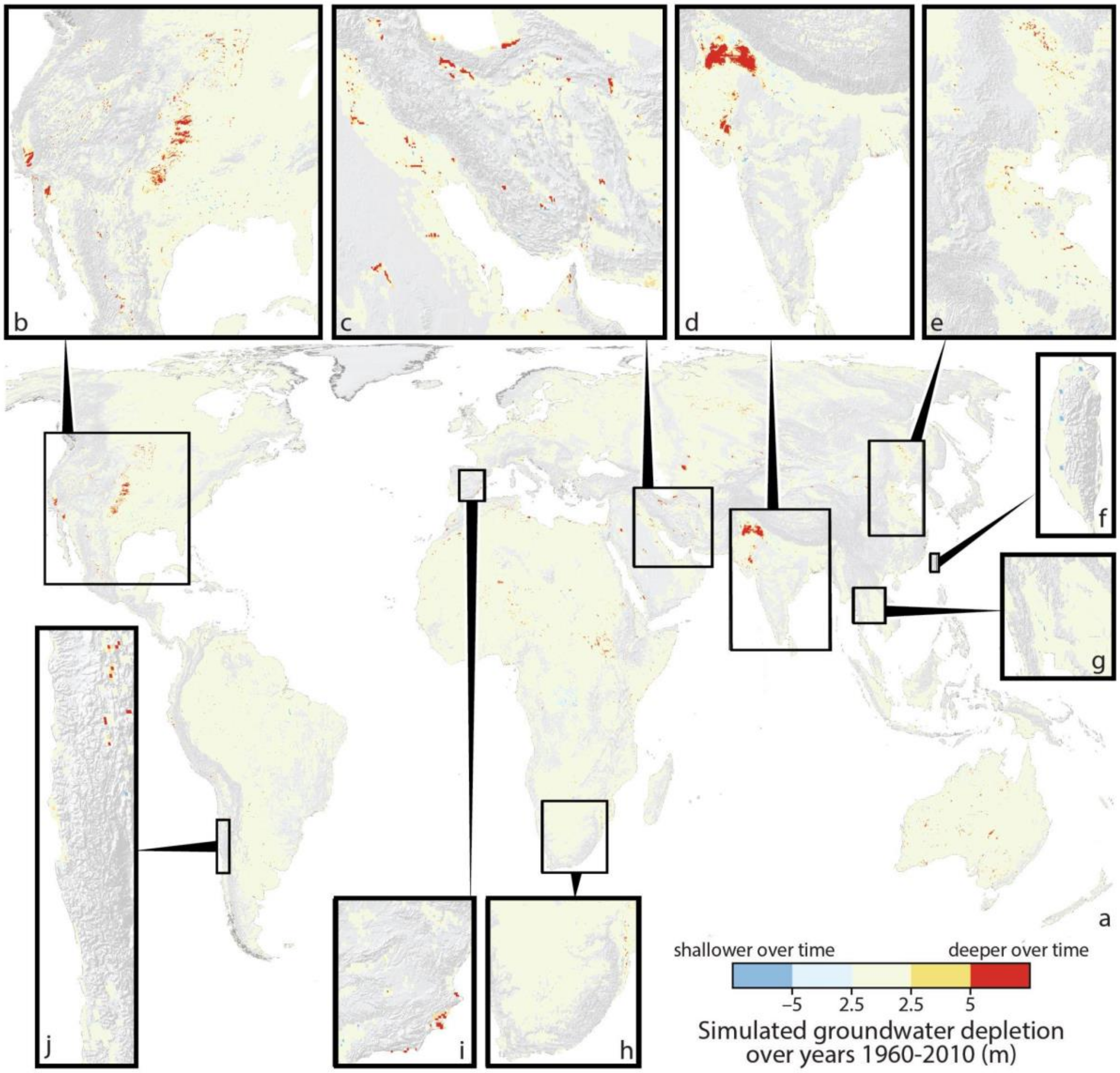

Supplementary Fig. 40. Groundwater depletion simulated with a global hydrologic model. Red areas are places where the model simulations suggest groundwater depletion over 1960-2010. Model results were provided by I.E. de Graaf via personal communication with co-author M. Shamsudduha, and the readme file states that the results are those from ref.[16]: de Graaf, I. E., Gleeson, T., Van Beek, L.P.H., Sutanudjaja, E.H., & Bierkens, M.F. Environmental flow limits to global groundwater pumping. *Nature* **574,** 90-94 (2019). The documentation associated with the spatial database states that the data represents the (the following text is quoted directly from the readme file): "estimated groundwater depletion (in meters) over the period 1960-2010 (estimated from groundwater head decline and aquifer storage/specific yields)."

15 LITERATURE REVIEW OF STUDIES CAPTURING GROUNDWATER TRENDS VIA MONITORING WELLS

15.1 LITERATURE REVIEW OF GROUNDWATER LEVEL DEEPENING

To examine evidence for groundwater level deepening in countries beyond those for which we were able to compile monitoring well time series, we completed a literature review. First, we compiled a series of articles published since January 1, 2017 that we thought should be included in a literature review of groundwater depletion. Next, we developed a search string that was able to return these same articles. Specifically, we searched Web of Science Core Collection (via "All fields") on December 29, 2022 for "groundwater deplet*" OR "aquifer deplet*" OR "deplet* groundwater*" OR "deplet* aquifer*" with a publication date between January 1, 2017 and December 29, 2022 (query link: https://www.webofscience.com/wos/woscc/summary/5284d469-a2e8-4d59-91c1-cf64cff2e6b2-67d22adf/relevance/1). The Web of Science search was completed using the University of California, Santa Barbara library; the default search via the Web of Science Core Collection for the University of California, Santa Barbara (the following is a direct quote from personal communication with a University of California, Santa Barbara librarian from August 19, 2021: *"Our default is the Web of Science Core Collection, which includes Science Citation Index Expanded, Social Sciences Citation Index, Arts & Humanities Citation Index, Conference Proceedings Citation Index, Book Citation Index, and Current Chemical Reactions and Index Chemicus."*). At the time the search was completed (December 29,

2022), the Web of Science Core Collection search returned 1,092 results. The title and abstract of each publication were examined for a statement suggesting that the manuscript provides evidence of groundwater depletion from piezometric data. 106 publications (of the original 1,092 Web of Science search results) were identified as potentially reporting evidence of groundwater depletion from piezometric data; the full text of these 106 articles was reviewed, and relevant articles were added to our database of studies reporting 21st-century groundwater level deepening (see table on the following pages).

Our Web of Science literature search is imperfect, as it may not capture abundant grey literature sources. Additional and grey literature sources were sought via a series of Google (and Google scholar) searches for "groundwater deplet*" (in an effort to reduce (though by no means eliminate) biases in the aforementioned search strategy; see ref.[1356]; Livoreil, B., Glanville, J., Haddaway, N.R., Bayliss, H., Bethel, A., de Lachapelle, F.F., Robalino, S., Savilaakso, S., Zhou, W., Petrokofsky, G. and Frampton, G. Systematic searching for environmental evidence using multiple tools and sources. Environmental Evidence, 6, https://environmentalevidencejournal.biomedcentral.com/articles/10.1186/s13750-017-0099-6 (2017)).

Our literature review is incomplete for a number of reasons (e.g., we did not search for publications in non-English languages). Consequently, the following table and map present some, but not all, places where groundwater level declines have been documented.

The compiled local-scale studies that have documented groundwater level deepening are displayed below (see figure and table on the following pages). Our literature review highlights that (i) groundwater levels are declining in some nations that are not part of the analyses presented in the main text (as we did not compile monitoring well data for these areas; e.g., Konya Basin in Turkey), and (ii) there are some

areas within some of our aquifer systems that are experiencing substantial groundwater level deepening, but the spatial distribution of our network of the monitoring well data we analysed (see main text figures) does not record this groundwater level deepening because we lack monitoring well data at specific locations where groundwater levels are deepening (e.g., substantial groundwater level deepening in Dhaka (Bangladesh) not captured by our network of monitoring wells – see “Dhaka City” in table below).

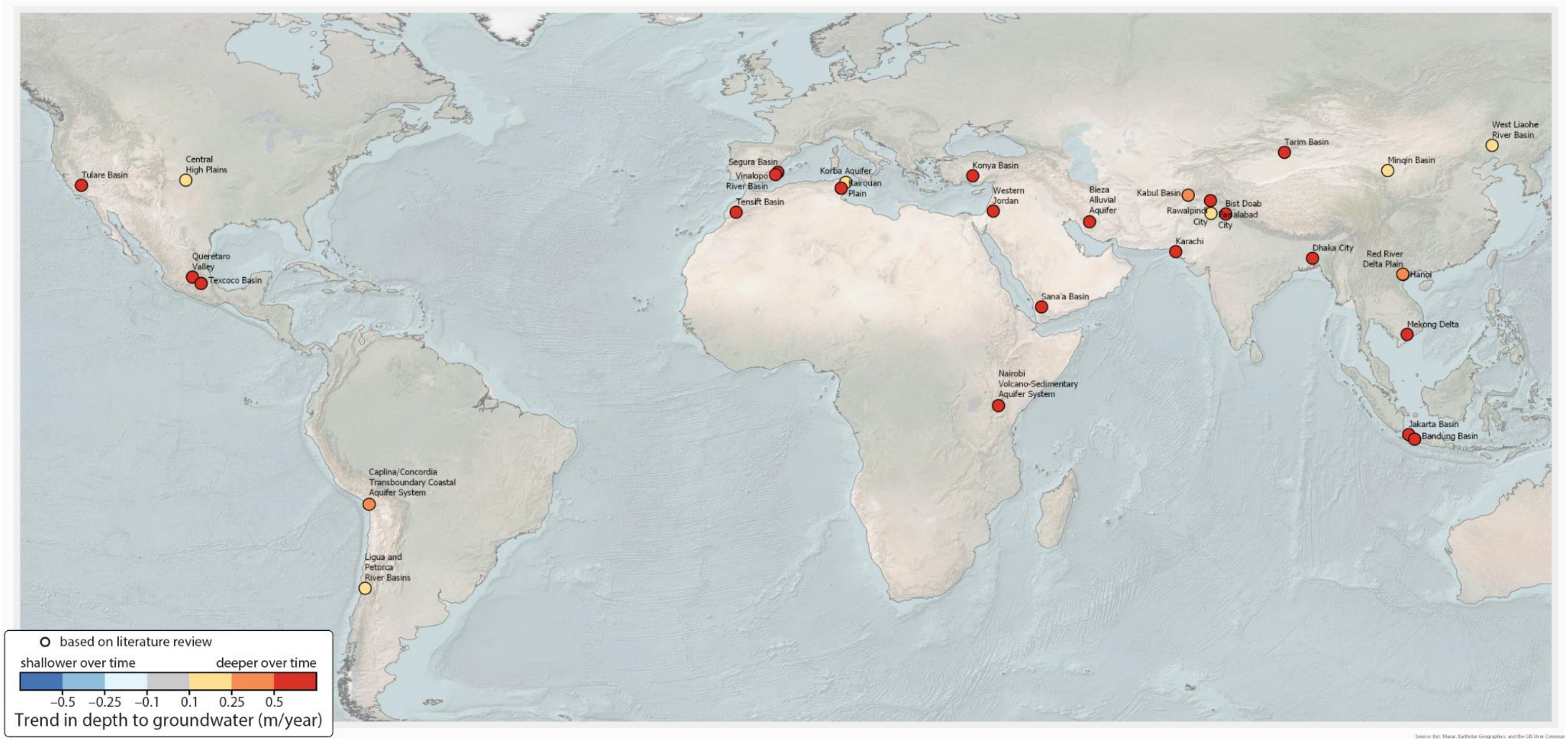

Supplementary Fig. 41. Locations where groundwater level deepening has been observed in one or more monitoring wells. Each circle represents the location of a local- to regional-scale study area where one or more published works have recorded groundwater level deepening in one or more monitoring wells (for details for each study area, see table on the following pages). The colour of each circle corresponds to the average of the minimum and maximum literature values (see table on the following pages). Groundwater level deepening has been captured in study areas beyond the countries for which we have monitoring well data (i.e., groundwater level deepening in some countries that are not part of the analyses presented in the main text figures), including areas in Kenya, Yemen, Turkey, Jordan, Tunisia and Indonesia.

Supplementary Table 11. Literature review 21st century groundwater level deepening***

Location	Country	Lat*	Lon*	Quote (or other source for estimated rate of 21st century groundwater level deepening)	Time-span	Ground-water level trend (m per year)**		Reference
**Ligua and Petorca River Basins**	Chile	-32.4	-71.0	Approximated from Fig. 3 (see also Fig. 7 by Duran-Llacer, I., Arumí, J.L., Arriagada, L., Aguayo, M., Rojas, O., González-Rodríguez, L., Rodríguez-López, L., Martínez-Retureta, R., Oyarzún, R., & Singh, S.K (2022). A new method to map groundwater-dependent ecosystem zones in semi-arid environments: A case study in Chile. Science of The Total Environment, 816, 151528).	2002-2017	0.1	0.3	Duran-Llacer, I., Munizaga, J., Arumí, J.L., Ruybal, C., Aguayo, M., Sáez-Carrillo, K., Arriagada, L., & Rojas, O. Lessons to be learned: Groundwater depletion in Chile's Ligua and Petorca watersheds through an Interdisciplinary approach. *Water* **12,** 2446 (2020). https://www.mdpi.com/2073-4441/12/9/2446
**Caplina/Concordia Transboundary Coastal Aquifer System**	Chile and Peru	-18.1	-70.3	Approximated from observed groundwater level time series presented in Fig. 7 (see also Fig. 7 by Pino, E., Ramos, L., Avalos, O., Tacora, P., Chávarri, E., Angulo, O., Ascensios, D. and Mejía, J., (2019). Factors affecting depletion and pollution by marine intrusion in the La Yarada´s coastal aquifer, Tacna, Peru. Tecnología y ciencias del agua, 10(5), 177-213)	2002-2016	0	0.9	Narvaez-Montoya, C., Torres-Martínez, J. A., Pino-Vargas, E., Cabrera-Olivera, F., Loge, F.J., & Mahlknecht, J. Predicting adverse scenarios for a transboundary coastal aquifer system in the Atacama Desert (Peru/Chile). Science of The Total Environment, 806, 150386 (2022). https://www.sciencedirect.com/science/article/pii/S0048969721054632#f0020
**Nairobi Volcano-Sedimentary Regional**	Kenya	-1.3	36.9	"Across the monitored wells, the mean decline rate is 0.43 m/year (4.3 m/decade) since 2007".	2006-2019 (some records span 2006-2015)	0.1	2.3	Oiro, S., Comte, J. C., Soulsby, C., MacDonald, A., & Mwakamba, C. (2020). Depletion of groundwater resources under rapid urbanisation in

Location	Country	Lat*	Lon*	Quote (or other source for estimated rate of 21st century groundwater level deepening)	Time-span	Ground-water level trend (m per year)**		Reference
**Aquifer System**				Range of rates of change in groundwater levels were approximated from Fig. 8				Africa: recent and future trends in the Nairobi Aquifer System, Kenya. Hydrogeology Journal 28, 2635-2656 (2020).
**Mekong Delta**	Vietnam	10.8	106.5	Approximated from time series presented in Fig. 7	2000-2015 (some records extend 2000-2011)	0.2	1.3	Minderhoud, P. S. J., Erkens, G., Pham, V. H., Bui, V. T., Erban, L., Kooi, H., & Stouthamer, E. Impacts of 25 years of groundwater extraction on subsidence in the Mekong delta, Vietnam. Environmental Research Letters 12, 064006 (2017).
**Querétaro Valley**	Mexico	20.6	-100.4	Approximated from Fig. 5b	2000-2017 (data are available for 1970-2017; only the most recent 17 years were examined to estimate the groundwater level deepening rate in this table)	0.3	2.3	Castellazzi, P., Garfias, J., & Martel, R. Assessing the efficiency of mitigation measures to reduce groundwater depletion and related land subsidence in Querétaro (Central Mexico) from decadal InSAR observations. International Journal of Applied Earth Observation and Geoinformation 105, 102632 (2021). https://www.sciencedirect.com/science/article/pii/S0303243421003391
**Red River Delta Plain**	Vietnam	21	105.8	Approximated from time series in Fig. 6 (*note time series extends from 1997-2018 in figure; rates approximated for years 2000-2018)	2000-2018	0	0.7	Nguyen, M., Lin, Y. N., Tran, Q. C., Ni, C. F., Chan, Y. C., Tseng, K. H., & Chang, C. P. Assessment of long-term ground subsidence and groundwater depletion in Hanoi, Vietnam. Engineering Geology 299, 106555 (2022). https://www.sciencedirect.com/science/article/pii/S0013795222000400
**Hanoi**	Vietnam	21.05	105.75	"During the last two decades, the annual drawdown of the groundwater level ranging from 0.3 m to 0.5 m..." (*note, this rate	2000-2015	0.3	0.5	Bui, L. K., Le, P. V., Dao, P. D., Long, N. Q., Pham, H. V., Tran, H. H., & Xie, L. (2021). Recent land deformation detected by Sentinel-1A InSAR data (2016–2020) over Hanoi, Vietnam, and the relationship with groundwater level change. GIScience

Location	Country	Lat*	Lon*	Quote (or other source for estimated rate of 21st century groundwater level deepening)	Time-span	Ground-water level trend (m per year)**		Reference
				corroborated by time series extending to the year 2015 in Fig. 6 by Vu, T. T., and N. V. T. Tran (2018). Assessment of urbanization impact on groundwater resources in Hanoi, Vietnam. Journal of Environmental Management 227, 107–116. doi:10.1016/j.jenvman.2018.08.087.				& Remote Sensing 58, 161-179 (2021). https://www.tandfonline.com/doi/full/10.1080/15481603.2020.1868198
**Dhaka City**	Bangladesh	23.8	90.4	"Approximated from Fig. 8 (*note: our groundwater monitoring dataset lacks sufficient observations near Dhaka for direct comparison with this study). Rates corroborated by Roy and Zahid (2021) who state (quote): "In densely populated city areas, the trend is extremely negative, with steep declines and annual changing rates that range from − 1.5 m/year to − 2.11 m/year; − 1.01 m/year to − 1.09 m/year in newly urbanized areas; and that range from 0.01 m/year to − 0.36 m/year in the rural areas." (quoting Roy, S. K., & Zahid, A. (2021). Assessment of declining groundwater levels due to	2000-2018	0	2.3	Moshfika, M., Biswas, S., & Mondal, M. S. Assessing groundwater level declination in Dhaka city and identifying adaptation options for sustainable water supply. Sustainability 14, 1518 (2022). https://www.mdpi.com/2071-1050/14/3/1518

Location	Country	Lat*	Lon*	Quote (or other source for estimated rate of 21st century groundwater level deepening)	Time-span	Ground-water level trend (m per year)**		Reference
				excessive pumping in the Dhaka District of Bangladesh. Environmental Earth Sciences, 80(8), 1-11.)				
**Karachi**	Pakistan	24.9	67.1	"In the study area, the groundwater depletion trend shows that the water table in 1999 was 6 m, and in 2018 it was 130 m. These values show the groundwater table going down continuously (Fig. 17). The data were collected by taking short interviews with drillers and other relative persons." quoting reference (see also their Fig. 17)	2000-2018	-	6.5	Sohail, M. T., Hussan, A., Ehsan, M., Al-Ansari, N., Akhter, M. M., Manzoor, Z., & Elbeltagi, A. Groundwater budgeting of Nari and Gaj formations and groundwater mapping of Karachi, Pakistan. Applied Water Science 12, 1-24 (2022). https://link.springer.com/article/10.1007/s13201-022-01795-0
**Bieza Alluvial Aquifer**	Iran	30	52.4	Approximated from Fig. 8	2002-2018	0.2	2.3	Dehghani, F., Mohammadi, Z., & Zare, M. (2021). Assessment of groundwater depletion in a heterogeneous aquifer: historical reconnaissance and current situation. Environmental Earth Sciences 80, 1-17 (2021). https://link.springer.com/article/10.1007/s12665-021-09839-5
**Bist Doab**	India	31.3	75.6	Approximated from Fig. 3; *note that the time interval examined is 1996-2016, which partly overlaps with the time interval focues on in this study (2000-present)	1996-2016	0	1.3	Gautam, A., Rai, S. C., & Rai, S. P. Impact of anthropogenic activities on the alluvial aquifers of north-east Punjab, India. Environmental Monitoring and Assessment 192, 1-18 (2020). https://link.springer.com/article/10.1007/s10661-020-08496-8
**Faisalabad City**	Pakistan	31.4	73.1	Approximated from Fig. 5	2000-2015	-	0.4	Sajjad, M. M., Wang, J., Abbas, H., Ullah, I., Khan, R., & Ali, F. Impact of Climate and Land-Use Change on Groundwater Resources, Study of Faisalabad District,

Location	Country	Lat*	Lon*	Quote (or other source for estimated rate of 21st century groundwater level deepening)	Time-span	Ground-water level trend (m per year)**		Reference
								Pakistan. Atmosphere 13, 1097 (2022). https://www.mdpi.com/2073-4433/13/7/1097
**Tensift Basin**	Morocco	31.6	-7.8	Approximated from Fig. 8 (see also the following quote within the reference: "In Chouiter (744/53, B), the decrease rate is around 2 m.y-1 since 2000, while in Mejjate (1133/53, A) the pace was around 1.5 m.y-1 between 2002 and 2016 and is close to 5 m.y-1 since 2016.")	2000-2021	0	2	Ouassanouan, Y., Fakir, Y., Simonneaux, V., Kharrou, M.H., Bouimouass, H., Najar, I., Benrhanem, M., Sguir, F. & Chehbouni, A. Multi-decadal analysis of water resources and agricultural change in a Mediterranean semiarid irrigated piedmont under water scarcity and human interaction. Science of The Total Environment, 834, 155328 (2022). https://www.sciencedirect.com/science/article/pii/S0048969722024214#f0020
**Western Jordan ("Azraq, Dead Sea, Hammad, Jordan Side Valleys, Yarmouk, and Zarqa Groundwater Basins" quoting reference)**	Jordan	31.8	36	Approximated from Appendicies (numerous PDFs, one per monitoring well). Rates presented here are approximates based on inspection of groundwater level time series (for years 2000 onward) for many of these appendix PDFs.	2000-2011	0	2	Goode, D.J., Senior, L.A., Subah, Ali, and Jaber, Ayman. Groundwater-level trends and forecasts, and salinity trends, in the Azraq, Dead Sea, Hammad, Jordan Side Valleys, Yarmouk, and Zarqa groundwater basins, Jordan. U.S. Geological Survey Open-File Report 2013–1061, 80 pp. (2013). Accessed December 30, 2022 via http://pubs.usgs.gov/of/2013/1061/
**Rawalpindi City**	Pakistan	33.6	73	Approximated from Fig. 7 (*note: depicting groundwater levels from 1991-2017; groundwater level rate of change estimated for years 2000-2017)	2000-2017	-	1.6	Naeem, U. A., Gabriel, H. F., Khan, N. M., Ahmad, I., Ur Rehman, H., & Zafar, M. A. Impact of urbanization on groundwater levels in Rawalpindi City, Pakistan. Pure and Applied Geophysics 178, 491-500 (2021). https://link.springer.com/article/10.1007/s00024-021-02660-y

Location	Country	Lat*	Lon*	Quote (or other source for estimated rate of 21st century groundwater level deepening)	Time-span	Ground-water level trend (m per year)**		Reference
**Mio-Plio-Quaternary Plain of Kairouan (Central Tunisia)**	Tunisia	35.7	10.1	Approximated from groundwater level time series depicted in Fig. 9	2000-2017 (data are available for 1969-2017; only the most recent 17 years were examined to estimate the groundwater level deepening rate in this table)	0.5	0.8	Snoussi, M., Jerbi, H., & Tarhouni, J. Integrated Groundwater Flow Modeling for Managing a Complex Alluvial Aquifer Case of Study Mio-Plio-Quaternary Plain of Kairouan (Central Tunisia). Water 14, 668 (2022). https://www.mdpi.com/2073-4441/14/4/668
**Korba Aquifer**	Tunisia	36.7	10.9	Approximated from Fig. 11b	2000-2013 (time series extends back to 1955; groundwater level trends (m/year) estimates in this table approximated for years 2000-2013)	0	0.3	Zghibi, A., Mirchi, A., Zouhri, L., Taupin, J. D., Chekirbane, A., & Tarhouni, J. (2019). Implications of groundwater development and seawater intrusion for sustainability of a Mediterranean coastal aquifer in Tunisia. Environmental monitoring and assessment 191, 1-23 (2019). https://link.springer.com/article/10.1007/s10661-019-7866-5/figures/11
**Central High Plains**	USA	37.1	-101.5	Approximated from the "Mean Water Level Change (m)" value depicted in Fig. 1c	2000-2014	-	0.3	Cotterman, K. A., Kendall, A. D., Basso, B., & Hyndman, D. W. (2018). Groundwater depletion and climate change: future prospects of crop production in the Central High Plains Aquifer. Climatic Change 146, 187-200 (2018). https://link.springer.com/article/10.1007/s10584-017-1947-7
**Konya Basin**	Turkey	37.8	32.5	Approximated from Fig. 8 (for years 2000-2018)	2000-2018	0.5	2	Orhan, O. Monitoring of land subsidence due to excessive groundwater extraction using small baseline subset technique in Konya, Turkey. Environmental Monitoring

Location	Country	Lat*	Lon*	Quote (or other source for estimated rate of 21st century groundwater level deepening)	Time-span	Ground-water level trend (m per year)**		Reference
								and Assessment 193, 1-17 (2021). https://link.springer.com/article/10.1007/s10661-021-08962-x
**Minqin Basin**	China	38.7	103.2	Approximated from Fig. 3	2001-2015	0	0.5	Hao, Y., Xie, Y., Ma, J., Zhang, W. The critical role of local policy effects in arid watershed groundwater resources sustainability: A case study in the Minqin oasis, China. Science of The Total Environment 601, 1084-1096 (2017). https://www.sciencedirect.com/science/article/pii/S0048969717310215
**Tarim Basin**	China	41.8	85.6	Approximated from Fig. 4	2002-2014	0.4	1	Xia, J., Wu, X., Zhan, C., Qiao, Y., Hong, S., Yang, P., & Zou, L. Evaluating the dynamics of groundwater depletion for an arid land in the Tarim Basin, China. Water 11, 186 (2019). https://www.mdpi.com/2073-4441/11/2/186
**Segura Basin**	Spain	38.4	-0.7	Approximated from Fig. 7 (time series extend to the 1960s or 1970s; rates added here are estimates for the years 2000-2015)	2000-2015	0	2.5	Custodio, E., Albiac, J., Cermerón, M., Hernández, M., Llamas, M. R., & Sahuquillo, A. Groundwater mining: benefits, problems and consequences in Spain. Sustainable Water Resources Management 3, 213-226 (2017). https://link.springer.com/article/10.1007/s40899-017-0099-2
**High and Mid Vinalopó River Basin**	Spain	38	-1.1	"The drawdown rate varies between 0.2 and 10 m/year, with a median of about 2.5 m/year." (quoting reference refering to the entire time series, which extends to the 1960s or 1970s for some records; the rates added to this table are approximations based on Figs. 6 and 7)	2000-2015	0.2	10	Custodio, E., Albiac, J., Cermerón, M., Hernández, M., Llamas, M. R., & Sahuquillo, A. Groundwater mining: benefits, problems and consequences in Spain. Sustainable Water Resources Management 3, 213-226 (2017). https://link.springer.com/article/10.1007/s40899-017-0099-2

Location	Country	Lat*	Lon*	Quote (or other source for estimated rate of 21st century groundwater level deepening)	Time-span	Ground-water level trend (m per year)**		Reference
**Sana'a Basin**	Yemen	15.5	44.22	"Groundwater abstraction is five times higher than natural recharge and the water-level decline is about 4–8 m/year." (quoting reference). Rate corroborated by another publication that states "As a result, the annual decline in groundwater levels is typically 2.5–4.5 m in many areas, with Sana'a Basin exceeding this and the decline being closer to 6 m/year" (quoting Taher, T., Bruns, B., Bamaga, O., Al-Weshali, A., & Van Steenbergen, F. (2012). Local groundwater governance in Yemen: building on traditions and enabling communities to craft new rules. Hydrogeology Journal, 20(6), 1177-1188). Further, see "Well HSA-49" on page 10 of Rybakov (2012) showing a time series of groundwater levels for Spring-2007 to Winter-2010, with a decline in the groundwater level of ~10 m: Rybakov, V. (2012). Water crisis in Yemen: speculations, realities and mitigation actions. 24 pp. Accessed December 31, 2022 via	2007-2010	3	8	Taher, T.M. Groundwater abstraction management in Sana'a Basin, Yemen: a local community approach. Hydrogeology Journal 24, 1593-1605 (2016). https://link.springer.com/article/10.1007/s10040-016-1435-6

Location	Country	Lat*	Lon*	Quote (or other source for estimated rate of 21st century groundwater level deepening)	Time-span	Ground-water level trend (m per year)**		Reference
				https://static1.squarespace.com/static/5eb18d627d53aa0e85b60c65/t/5eda46ed1c956a6bc14ae36c/1591363321836/Report-victor.pdf)				
**Jakarta Basin**	Indonesia	-6.2	106.8	Approximated from Fig. 6.5	2000-2005 (note, relatively brief time interval)	0	2	Delinom, R. M. The proposed groundwater management for the Greater Jakarta Area, Indonesia. In Groundwater and Subsurface Environments. Springer, Tokyo, 113-125 (2011). https://link.springer.com/content/pdf/10.1007/978-4-431-53904-9.pdf?pdf=button
**Bandung Basin**	Indonesia	-7	107.8	"...the deep groundwater potential for 2010 showed some groundwater depression cones in the textile industry areas such as Cimahi (CMHI), Rancaekek (RCK), and Dayeuhkolot (DHYK; Fig. 2b). The results for the deep groundwater potentials in this study (Fig. 2b) clearly show that the groundwater depressions have become deeper. In the CMHI area, which has the biggest drawdown, groundwater levels dropped from 620 m relative to sea level (sl) in 2010 to 610 m sl in 2015." (quoting reference). See also groundwater level data in Fig. 11 by Abidin (2009), showing groundwater level	2010-2015	0	3	Taufiq, A., Hosono, T., Ide, K., Kagabu, M., Iskandar, I., Effendi, A.J., Hutasoit, L.M., Shimada, J. Impact of excessive groundwater pumping on rejuvenation processes in the Bandung basin (Indonesia) as determined by hydrogeochemistry and modeling. Hydrogeology Journal 26, 1263-1279 (2018). https://link.springer.com/article/10.1007/s10040-017-1696-8

Location	Country	Lat*	Lon*	Quote (or other source for estimated rate of 21st century groundwater level deepening)	Time-span	Ground-water level trend (m per year)**		Reference
				deepening for 2000-2005 of ~0.6 m/year to ~3 m/year (Abidin, H. Z., Andreas, H., Gumilar, I., Wangsaatmaja, S., Fukuda, Y., & Deguchi, T. (2009). Land subsidence and groundwater extraction in Bandung Basin, Indonesia. IAHS publication, 20, 145).				
**Kabul Basin**	Afghanistan	34.5	69.2	"The groundwater hydrograph shows that groundwater levels declined 12 m over 15 years (from 2005 to 2020) at a rate of 80 cm/year in some parts of the Kabul aquifers."	2005-2020	-	0.8	Zaryab, A., Nassery, H. R., & Alijani, F. The effects of urbanization on the groundwater system of the Kabul shallow aquifers, Afghanistan. Hydrogeology Journal 30, 429-443 (2022). https://link.springer.com/article/10.1007/s10040-021-02445-6
**Texcoco Basin**	Mexico	19.5	-98.9	Approximated from Fig. 6.5	2000-2014	0.3	1	Carrillo, M., Gomez, Y. A., Valle, S., & Prado, J. V. (2016). Behavior of groundwater levels in Texcoco Aquifer (1507) when they are lowered by excessive pumping from 1968 through 2014. In 2016 ASABE Annual International Meeting. American Society of Agricultural and Biological Engineers. https://elibrary.asabe.org/abstract.asp?aid=47273
**West Liaohe River Basin**	China	43	121	" Because the groundwater table in the WLRB was relatively shallow during 1980–1998 (i.e., 2–3 m, shown in Figure 3a), the effect of the delayed response of groundwater is limited. From 1998 to 2015, however, the	2000-2015	0.1	0.3	Zhong, Y., Zhong, M., Feng, W., Zhang, Z., Shen, Y., & Wu, D. Groundwater depletion in the West Liaohe River Basin, China and its implications revealed by GRACE and in situ measurements. Remote Sensing 10, 493 (2018). https://www.mdpi.com/2072-4292/10/4/493

Location	Country	Lat*	Lon*	Quote (or other source for estimated rate of 21st century groundwater level deepening)	Time-span	Ground-water level trend (m per year)**		Reference
				groundwater table reaches a depth of 5–6 m."				
**Tulare Basin (southern California Central Valley)**	USA	36.2	-119.3	Approximated from Fig. 4	2011-2015	0	6	Ojha, C., Werth, S., & Shirzaei, M. Groundwater loss and aquifer system compaction in San Joaquin Valley during 2012–2015 drought. Journal of Geophysical Research: Solid Earth 124, 3127-3143 (2019).

*approximated study location

**two columns provide an approximation of the range of groundwater level trends over time. Positive values represent groundwater level deepening over time.

***full citations for compiled literature are also presented in refs.[20,427,802,1282,1313-1355]; here we focus our compilation to local- and regional-scale studies rather than those that are national in scope, but highlight that numerous national-scale studies of groundwater level changes over time have also been published recently, such as ref.[1339] (i.e., Noori, R. et al. Anthropogenic depletion of Iran's aquifers. *Proceedings of the National Academy of Sciences* **118,** e2024221118 (2021)), and ref.[1340] (i.e., Ashraf, S., Nazemi, A., & AghaKouchak, A. Anthropogenic drought dominates groundwater depletion in Iran. *Scientific Reports* **11,** 9135 (2021))

15.2 LITERATURE REVIEW OF GROUNDWATER LEVEL SHALLOWING

We also completed a literature search for evidence for groundwater level shallowing in countries beyond those for which we were able to compile monitoring well time series.

We followed a similar approach to our literature review of cases of groundwater level deepening (see supplementary subsection above entitled ‘Literature review of groundwater level deepening’). Specifically, we compiled articles returned from the following Web of Science Core Collection (via “All fields”) search completed on March 10, 2023: “rising groundwater*” OR "groundwater level* rose" OR "groundwater elevation* rose*" OR "revers* deplet*" OR "aquifer* refill*" OR "shallower groundwater" OR "groundwater became shallow*" OR "groundwater level* became shallow*" OR "increas* groundwater*" OR "groundwater elevation* increase*" OR "groundwater stor* increase*" OR "groundwater recover*" (publication date between January 1, 2017 and December 29, 2022; query link: https://www.webofscience.com/wos/woscc/summary/b0d2e4b2-4935-4e4e-9f15-fa073e04eaee-77a84c83/relevance/1). Consistent with our review of groundwater level deepening cases, this Web of Science search was completed using the University of California, Santa Barbara default search via the Web of Science Core Collection for the University of California, Santa Barbara (see details in supplementary subsection above entitled ‘Literature review of groundwater level deepening’).

At the time the search was completed (March 10, 2023), the Web of Science Core Collection search returned 619 results. The title and abstract of each publication were examined for a statement suggesting that the manuscript provides evidence of rising groundwater levels based on monitoring well water level time series. 34 publications (of the original n=619 Web of Science search results) were identified as potentially reporting evidence of groundwater level shallowing on the basis of monitoring well water level data; the full text of these 34

articles was reviewed, and relevant articles were added to our database of studies reporting 21st-century groundwater level shallowing (see the following figure and table). We then completed several supplementary searches using other relevant keywords (e.g., “groundwater recovery”) in Google Scholar, and added several more articles that report on rising 21st-century groundwater levels to our compilation (see figure below).

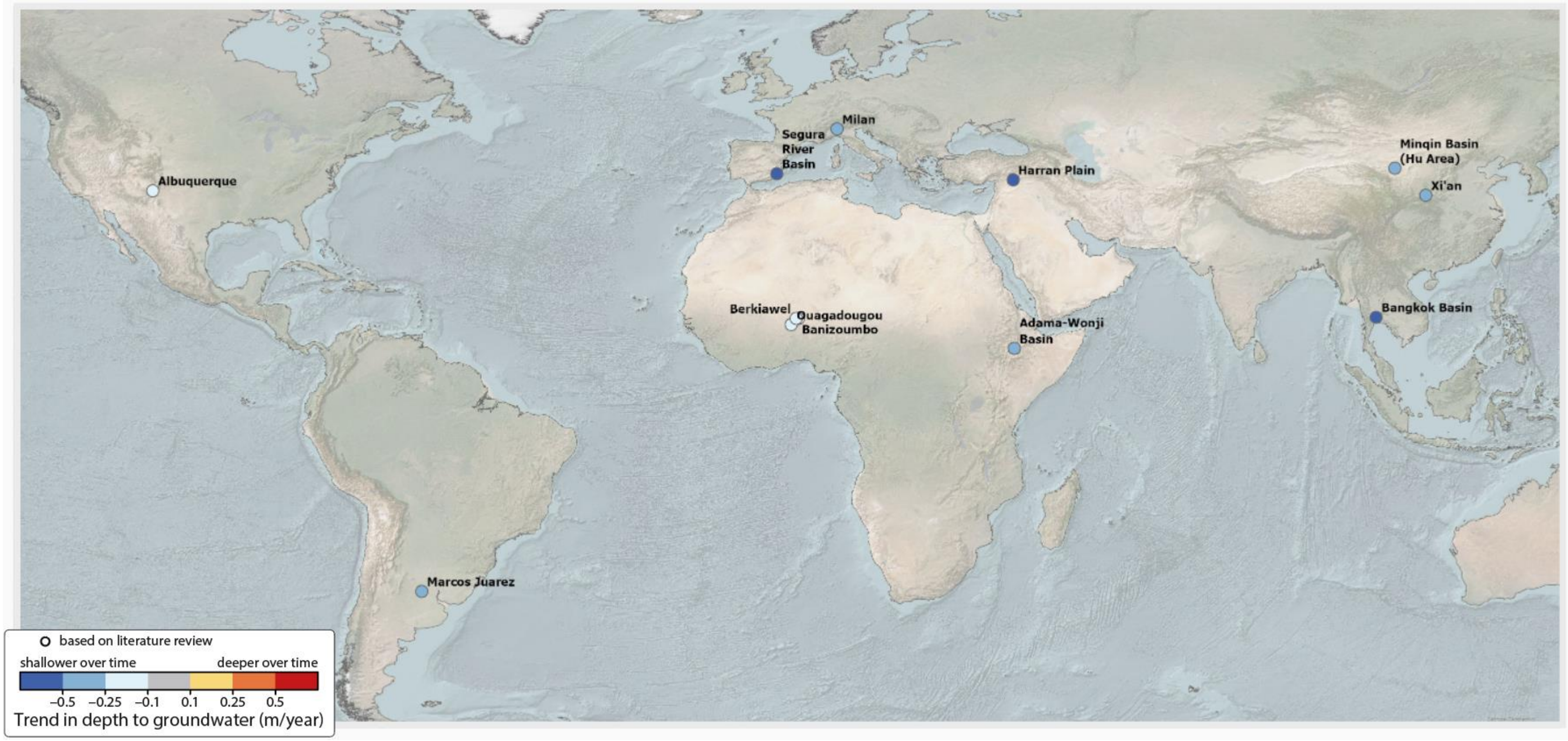

Supplementary Fig. 42. Locations where groundwater level shallowing has been observed in one or more monitoring wells. Each circle represents the location of a local- to regional-scale study area where one or more published works have recorded groundwater level shallowing in one or more monitoring wells (for details for each study area, see table on the following pages). The colour of each circle corresponds to the average of the minimum and maximum literature values (see table on the following pages). Groundwater level shallowing has been captured in study areas beyond the countries for which we have monitoring well data (i.e., groundwater level shallowing in some countries that are not part of the analyses presented in the main text figures), including areas in Burkina Faso and Niger. Imagery from https://www.arcgis.com/home/item.html?id=10df2279f9684e4a9f6a7f08febac2a9.

Supplementary Table 12. Literature review 21st-century groundwater level shallowing***

Location	Country	Lat*	Lon*	Quote (or other source for estimated rate of 21st century groundwater level trend)	Time-span	Groundwater level trend (m per year)**		Reference
**Bangkok Basin**	Thailand	13.7	100.5	"As seen in Fig. 4a–d the recovery rate varies from 0.73 to 0.99 m/year for the PD aquifer"	1997-2016	-0.73	-0.99	Saowiang, K., & Giao, P.H. Numerical analysis of subsurface deformation induced by groundwater level changes in the Bangkok aquifer system. Acta Geotechnica 16, 1265-1279 (2021).
**Xi'an**	China	34.3	108.9	"The confined water level showed a gradual upward trend over time..."; groundwater level trend estimated from Fig. 9c	2007-2017	-0.48	-	Shi, W., Chen, G., Meng, X., Jiang, W., Chong, Y., Zhang, Y., Dong, Y., Zhang, M. Spatial-temporal evolution of land subsidence and rebound over Xi'an in western China revealed by SBAS-InSAR analysis. Remote Sensing 12, 3756 (2020).
**Milan**	Italy	45.5	9.2	"86 out of 95 MWs showed an increasing trend throughout the considered period, with a medium slope ranging from 0.20 to 0.60 in the northern sector, from 0.10 to 0.20 in the central portion of the domain, and from 0.00 to 0.10 in the southern area."	2005-2019	0	-0.6	Sartirana, D., Rotiroti, M., Bonomi, T., De Amicis, M., Nava, V., Fumagalli, L., & Zanotti, C. Data-driven decision management of urban underground infrastructure through groundwater-level time-series cluster analysis: The case of Milan (Italy). Hydrogeology Journal 30, 1157-1177 (2022).
**Ouagadougou**	Burkina Faso	12.4	1.5	Extended Data Fig. 1	2000-2017	-0.16	-	Cuthbert, M.O. et al. Observed controls on resilience of groundwater to climate variability in sub-Saharan Africa. *Nature* **572,** 230-234 (2019).

Location	Country	Lat*	Lon*	Quote (or other source for estimated rate of 21st century groundwater level trend)	Time-span	Groundwater level trend (m per year)**		Reference
**Banizoumbo**	Niger	13.5	2.7	Extended Data Fig. 1	2000-2016	-0.24	-	Cuthbert, M.O. et al. Observed controls on resilience of groundwater to climate variability in sub-Saharan Africa. *Nature* **572,** 230-234 (2019).
**Berkiawel**	Niger	13.5	2.3	Extended Data Fig. 1	2000-2016	-0.15	-	Cuthbert, M.O. et al. Observed controls on resilience of groundwater to climate variability in sub-Saharan Africa. *Nature* **572,** 230-234 (2019).
**Marcos Juarez**	Argentina	-32.7	-61.1	Trend approximated from Fig. 3c (latitude and longitude derived from Supplementary Materials)	2000-2020	-0.3	-	Houspanossian, J., Giménez, R., Whitworth-Hulse, J.I., Nosetto, M.D., Tych, W., Atkinson, P.M., Rufino, M.C., & Jobbágy, E.G. Agricultural expansion raises groundwater and increases flooding in the South American plains. *Science* **380,** 1344-1348 (2023).
**Albuquerque**	USA	35.1	-106.6	Estimated from Fig. 3 (Relevant quote: "This recent groundwater-level rebound is apparent in time-series analysis of piezometers in the study area (fig. 3) and correlates with reduced groundwater withdrawals in the study area")	2000-2020	0	-0.4	Galanter, A.E., & Curry, L.T.S. Estimated 2016 groundwater level and drawdown from predevelopment to 2016 in the Santa Fe Group aquifer system in the Albuquerque area, central New Mexico US Geological Survey Scientific Investigations Map 3433, 1 sheet, 13 pp. pamphlet (2019). Accessed July 16, 2023 via https://doi.org/10.3133/sim3433
**Minqin Basin (Hu Area)**	China	38.9	103.6	"In the CTSRB I period...a sharp increase in the level occurred in Hu at a	2006-2010 | 2010-2015	0	-0.65	Hao, Y., Xie, Y., Ma, J., & Zhang, W. The critical role of local policy effects in arid watershed groundwater resources sustainability: A case study in the

Location	Country	Lat*	Lon*	Quote (or other source for estimated rate of 21st century groundwater level trend)	Time-span	Groundwater level trend (m per year)**		Reference
				rate of 0.64 m/year." | "During the CTSRB II period... the groundwater level ...continued to rise in Hu." ('CTSRB I' corresponds to years 2006 to 2010, and 'CTSRB II' corresponds to years 2010 to 2015)				Minqin oasis, China. *Science of The Total Environment* **601,** 1084-1096 (2017).
**Kuki, Saitama (Kanto Plain)**	Japan	36.1	139.6	Approximated from Fig. 3 (high-magnitude estimate of -0.5 based on record for "Kuki (Saitama)")	2000-2009	0	-0.5	Furuno, K., Kagawa, A., Kazaoka, O., Kusuda, T., & Nirei, H. Groundwater management based on monitoring of land subsidence and groundwater levels in the Kanto Groundwater Basin, Central Japan. *Proceedings of the International Association of Hydrological Sciences* **372,** 53-57 (2015).
**Segura River Basin**	Spain	38	-1	"In 8 of the most intensively exploited aquifers of the Segura river basin, the drawdown rate was between 2 and 15 m/year from the 1970s to the mid-2000s, with a median value between 4 and 6 m/year. In the period 2005–2013, a recovery between 0 and 10 m/year was observed in some of these aquifers, with a	2005-2013	-1	-2	Custodio, E., Andreu-Rodes, J.M., Aragón, R., Estrela, T., Ferrer, J., García-Aróstegui, J.L., Manzano, M., Rodríguez-Hernández, L., Sahuquillo, A., & Del Villar, A. Groundwater intensive use and mining in south-eastern peninsular Spain: Hydrogeological, economic and social aspects. *Science of the Total Environment* **559,** 302-316 (2016).

Location	Country	Lat*	Lon*	Quote (or other source for estimated rate of 21st century groundwater level trend)	Time-span	Groundwater level trend (m per year)**		Reference
				median value of 1 to 2 m/year."				
**Adama-Wonji Basin**	Ethiopia	8.38	39.25	Approximated from Fig. 4	2000-2007	-0.4	-	Furi, W., Razack, M., Haile, T., Abiye, T. A., & Legesse, D. The hydrogeology of Adama-Wonji basin and assessment of groundwater level changes in Wonji wetland, Main Ethiopian Rift: results from 2D tomography and electrical sounding methods. *Environmental Earth Sciences* **62,** 1323-1335 (2011).
**Harran Plain**	Turkey	36.9	39.0	Approximated from groundater level time series figure on the base of page 49. "Groundwater level declines from 1977 to 1995 (Fig. 6). After uncontrolled irrigation, groundwater level has been raised from 1995 up to now"	2000-2015	-0.4	-2.0	Özel, N., Bozdağ, Ş., & Baba, A. (2019). Effect of irrigation system on groundwater resources in Harran Plain (Southeastern Turkey). *Journal of Food Science and Engineering* **9,** 45-51. Accessed July 16, 2023 via https://core.ac.uk/download/pdf/324145047.pdf

*approximated study location

**two columns provide an approximation of the range of groundwater level trends over time. Negative values represent groundwater level shallowing over time.

***full citations for compiled literature are also presented in refs.[20,427,802,1282,1313-1355]

16 SPATIAL VARIABILITY IN GROUNDWATER LEVEL TRENDS FOR INDIVIDUAL SUB-CONTINENTS AND CONTINENTS

We present maps of trends in depth to groundwater (Theil-Sen slopes) for individual monitoring wells (see the following figures). These maps highlight the wide variability in groundwater level trends across space. The maps also enable us to present our entire dataset of monitoring wells, including wells that fall outside of the areas covered by our 1,693 aquifer systems.

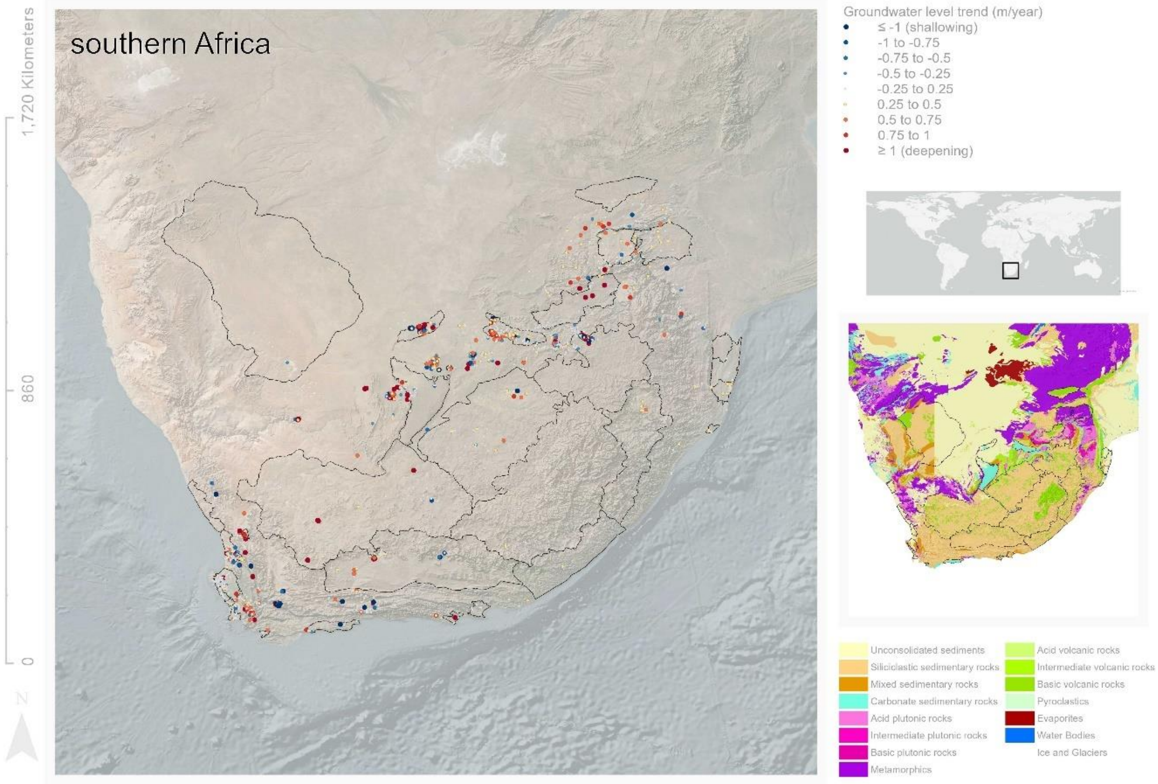

Supplementary Fig. 43. 21st-century trends in depth to groundwater in monitoring wells across southern Africa. The largest map (on the left-hand side of the figure) depicts 21st-century trends in depth to groundwater. Each point (coloured circle) represents a monitoring well; the colour of the point corresponds to the 21st-century trend in depth to groundwater for the monitoring well (Theil-Sen slope). Orange and red points represent monitoring wells where groundwater levels became deeper over time (Theil-Sen slope >0.25 m/year). Blue points represent monitoring wells where groundwater levels became shallower over time (see legend in upper-right corner of figure). The grey map (right-hand side of figure) shows the location of the area depicted in the large map on the left-hand side of the figure. The map in the lower-right corner of the figure presents spatial variations in near-surface geology (data from ref.[1360]: Hartmann, J., & Moosdorf, N. (2012). The new global lithological map database GLiM: A representation of rock properties at the Earth surface. *Geochemistry, Geophysics, Geosystems* 13, doi.org/10.1029/2012GC004370). Imagery from https://www.arcgis.com/home/item.html?id=10df2279f9684e4a9f6a7f08febac2a9

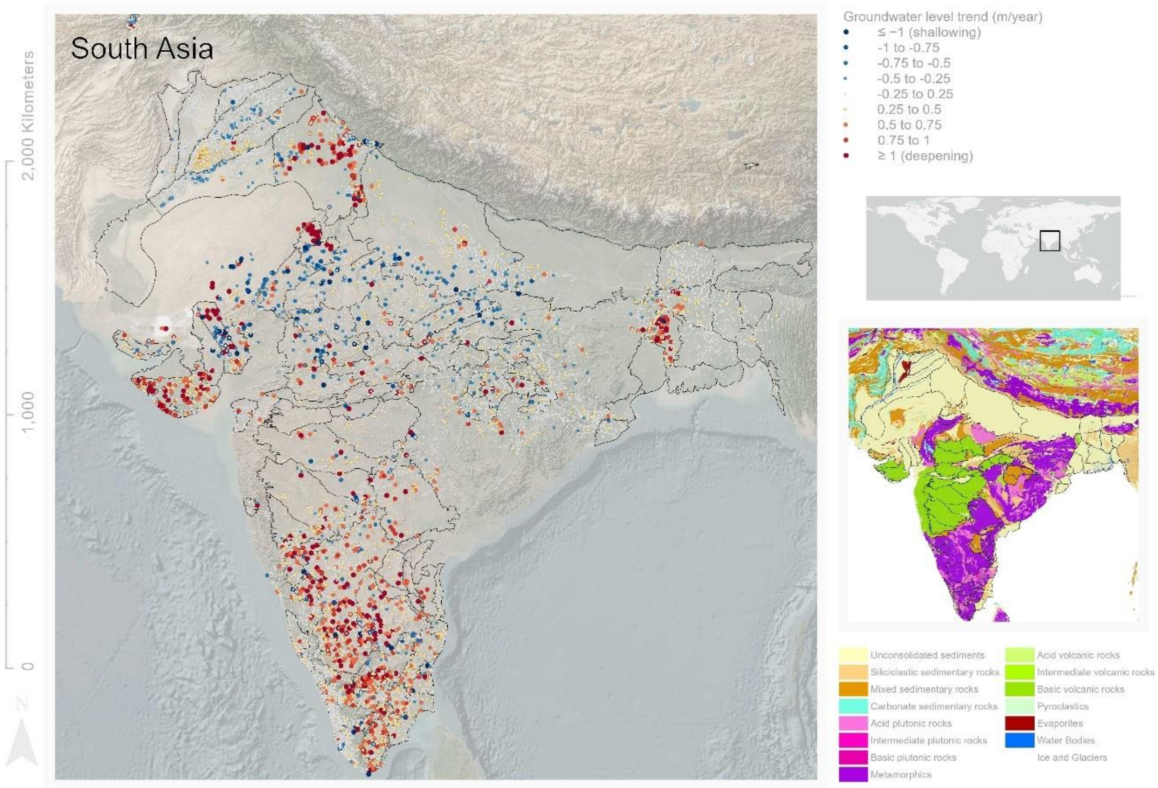

Supplementary Fig. 44. 21st-century trends in depth to groundwater in monitoring wells across South Asia. The largest map (on the left-hand side of the figure) depicts 21st-century trends in depth to groundwater. Each point (coloured circle) represents a monitoring well; the colour of the point corresponds to the 21st-century trend in depth to groundwater for the monitoring well (Theil-Sen slope). Orange and red points represent monitoring wells where groundwater levels became deeper over time (Theil-Sen slope >0.25 m/year). Blue points represent monitoring wells where groundwater levels became shallower over time (see legend in upper-right corner of figure). The grey map (right-hand side of figure) shows the location of the area depicted in the large map on the left-hand side of the figure. The map in the lower-right corner of the figure presents spatial variations in near-surface geology (data from ref.[1360]: Hartmann, J., & Moosdorf, N. (2012). The new global lithological map database GLiM: A representation of rock properties at the Earth surface. *Geochemistry, Geophysics, Geosystems* 13, doi.org/10.1029/2012GC004370). Imagery from https://www.arcgis.com/home/item.html?id=10df2279f9684e4a9f6a7f08febac2a9

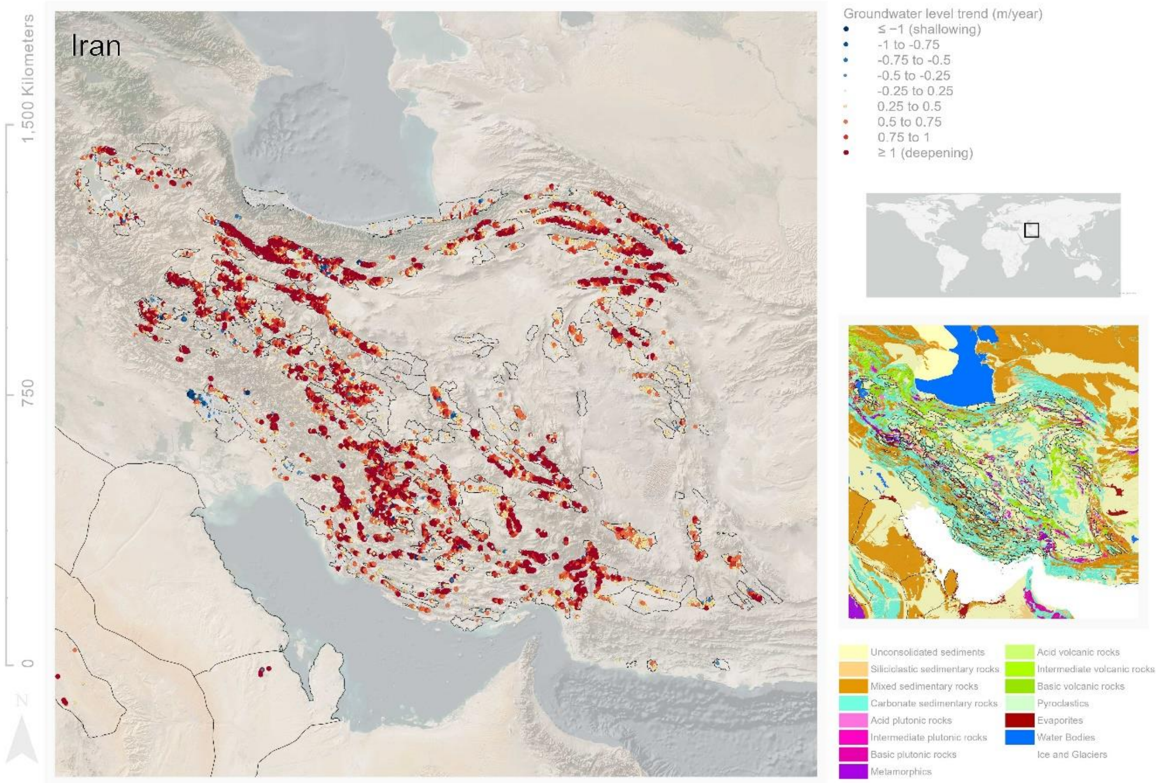

Supplementary Fig. 45. 21st-century trends in depth to groundwater in monitoring wells across Iran. The largest map (on the left-hand side of the figure) depicts 21st-century trends in depth to groundwater. Each point (coloured circle) represents a monitoring well; the colour of the point corresponds to the 21st-century trend in depth to groundwater for the monitoring well (Theil-Sen slope). Orange and red points represent monitoring wells where groundwater levels became deeper over time (Theil-Sen slope >0.25 m/year). Blue points represent monitoring wells where groundwater levels became shallower over time (see legend in upper-right corner of figure). The grey map (right-hand side of figure) shows the location of the area depicted in the large map on the left-hand side of the figure. The map in the lower-right corner of the figure presents spatial variations in near-surface geology (data from ref.[1360]: Hartmann, J., & Moosdorf, N. (2012). The new global lithological map database GLiM: A representation of rock properties at the Earth surface. *Geochemistry, Geophysics, Geosystems* 13, doi.org/10.1029/2012GC004370). Imagery from https://www.arcgis.com/home/item.html?id=10df2279f9684e4a9f6a7f08febac2a9

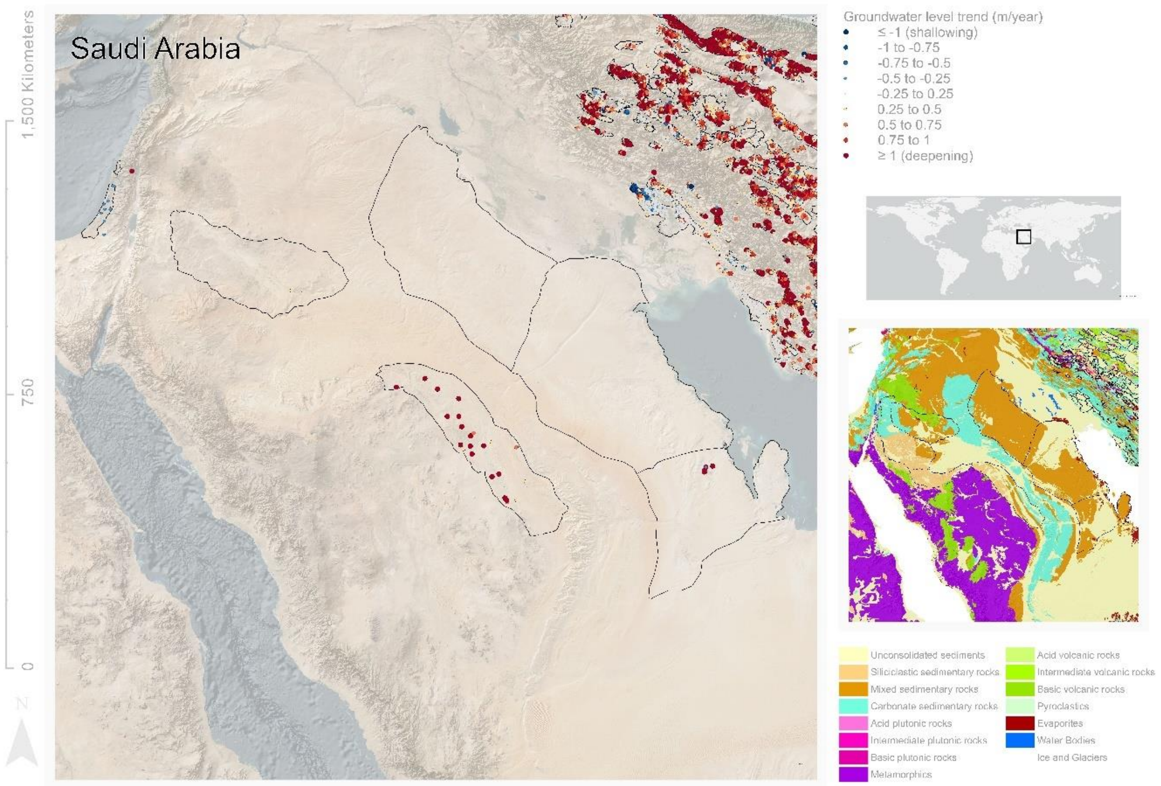

Supplementary Fig. 46. 21st-century trends in depth to groundwater in monitoring wells across Saudi Arabia. The largest map (on the left-hand side of the figure) depicts 21st-century trends in depth to groundwater. Each point (coloured circle) represents a monitoring well; the colour of the point corresponds to the 21st-century trend in depth to groundwater for the monitoring well (Theil-Sen slope). Orange and red points represent monitoring wells where groundwater levels became deeper over time (Theil-Sen slope >0.25 m/year). Blue points represent monitoring wells where groundwater levels became shallower over time (see legend in upper-right corner of figure). The grey map (right-hand side of figure) shows the location of the area depicted in the large map on the left-hand side of the figure. The map in the lower-right corner of the figure presents spatial variations in near-surface geology (data from ref.[1360]: Hartmann, J., & Moosdorf, N. (2012). The new global lithological map database GLiM: A representation of rock properties at the Earth surface. *Geochemistry, Geophysics, Geosystems* 13, doi.org/10.1029/2012GC004370). Imagery from https://www.arcgis.com/home/item.html?id=10df2279f9684e4a9f6a7f08febac2a9

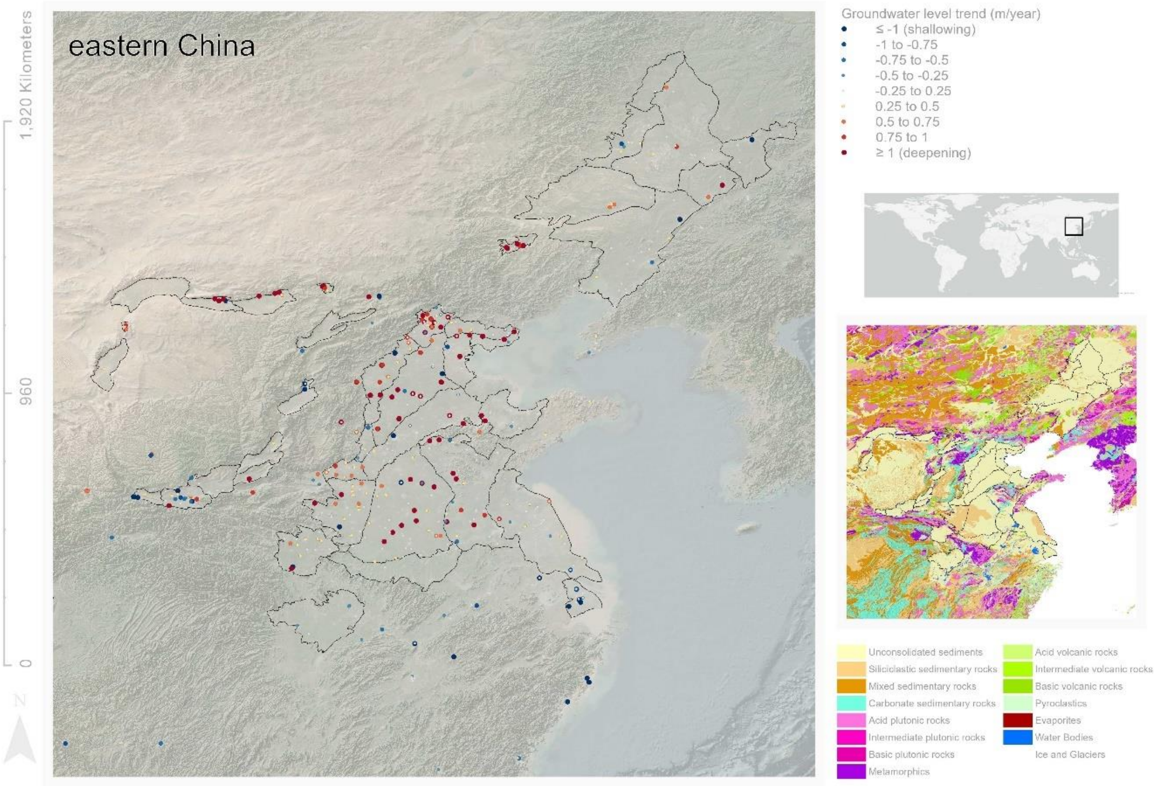

Supplementary Fig. 47. 21st-century trends in depth to groundwater in monitoring wells across northeastern China. The largest map (on the left-hand side of the figure) depicts 21st-century trends in depth to groundwater. Each point (coloured circle) represents a monitoring well; the colour of the point corresponds to the 21st-century trend in depth to groundwater for the monitoring well (Theil-Sen slope). Orange and red points represent monitoring wells where groundwater levels became deeper over time (Theil-Sen slope >0.25 m/year). Blue points represent monitoring wells where groundwater levels became shallower over time (see legend in upper-right corner of figure). The grey map (right-hand side of figure) shows the location of the area depicted in the large map on the left-hand side of the figure. The map in the lower-right corner of the figure presents spatial variations in near-surface geology (data from ref.[1360]: Hartmann, J., & Moosdorf, N. (2012). The new global lithological map database GLiM: A representation of rock properties at the Earth surface. *Geochemistry, Geophysics, Geosystems* 13, doi.org/10.1029/2012GC004370). Imagery is available via: https://www.arcgis.com/home/item.html?id=10df2279f9684e4a9f6a7f08febac2a9

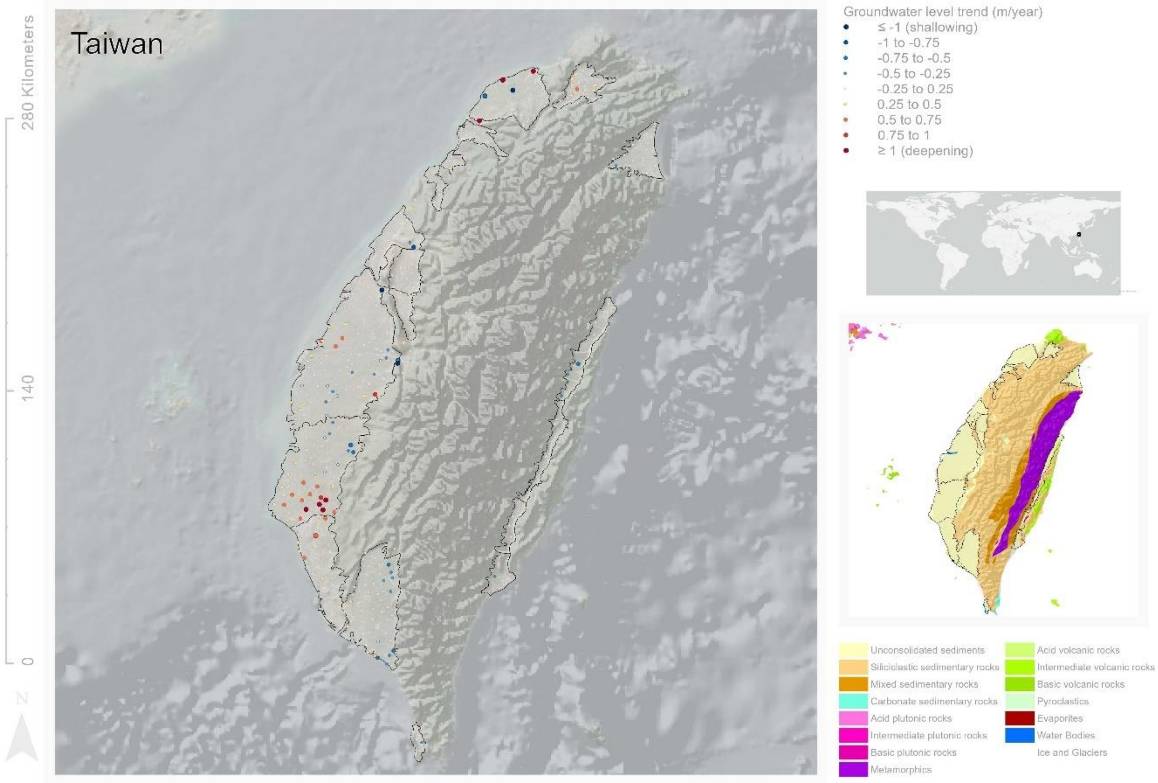

Supplementary Fig. 48. 21st-century trends in depth to groundwater in monitoring wells across Taiwan. The largest map (on the left-hand side of the figure) depicts 21st-century trends in depth to groundwater. Each point (coloured circle) represents a monitoring well; the colour of the point corresponds to the 21st-century trend in depth to groundwater for the monitoring well (Theil-Sen slope). Orange and red points represent monitoring wells where groundwater levels became deeper over time (Theil-Sen slope >0.25 m/year). Blue points represent monitoring wells where groundwater levels became shallower over time (see legend in upper-right corner of figure). The grey map (right-hand side of figure) shows the location of the area depicted in the large map on the left-hand side of the figure. The map in the lower-right corner of the figure presents spatial variations in near-surface geology (data from ref.[1360]: Hartmann, J., & Moosdorf, N. (2012). The new global lithological map database GLiM: A representation of rock properties at the Earth surface. *Geochemistry, Geophysics, Geosystems* 13, doi.org/10.1029/2012GC004370). Imagery from https://www.arcgis.com/home/item.html?id=10df2279f9684e4a9f6a7f08febac2a9

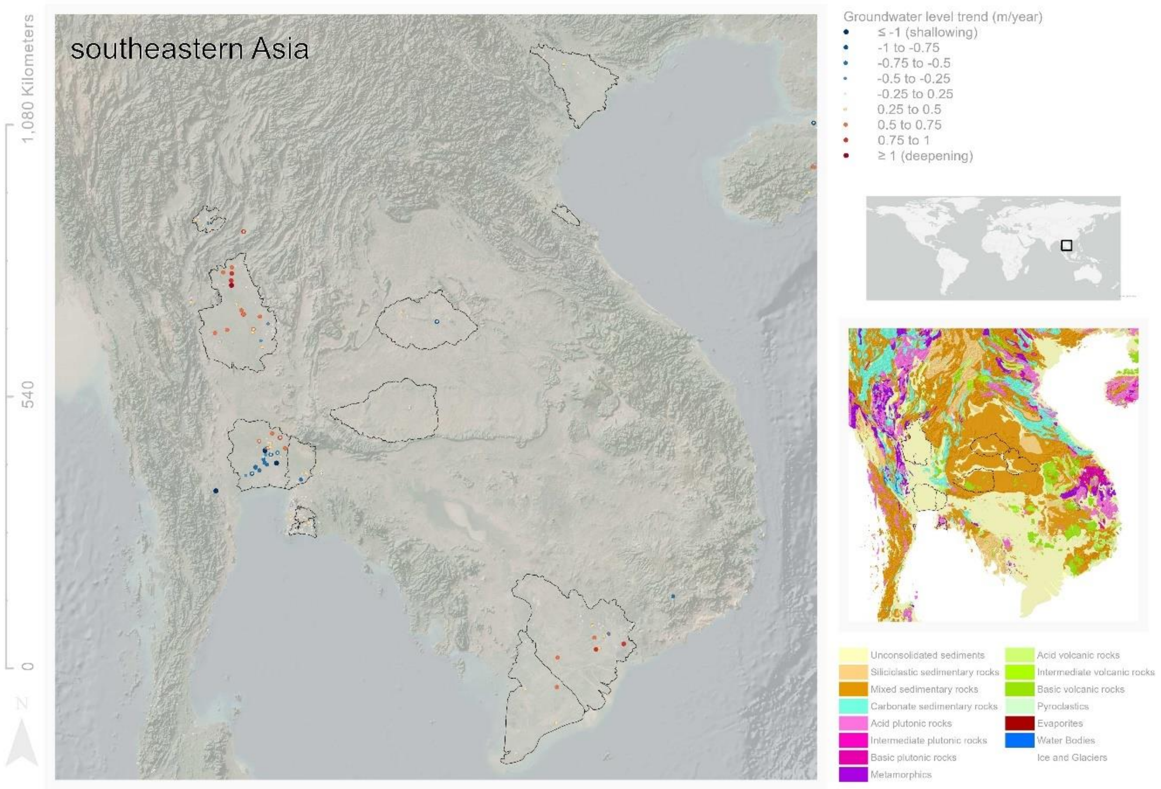

Supplementary Fig. 49. 21st-century trends in depth to groundwater in monitoring wells across southeastern Asia. The largest map (on the left-hand side of the figure) depicts 21st-century trends in depth to groundwater. Each point (coloured circle) represents a monitoring well; the colour of the point corresponds to the 21st-century trend in depth to groundwater for the monitoring well (Theil-Sen slope). Orange and red points represent monitoring wells where groundwater levels became deeper over time (Theil-Sen slope >0.25 m/year). Blue points represent monitoring wells where groundwater levels became shallower over time (see legend in upper-right corner of figure). The grey map (right-hand side of figure) shows the location of the area depicted in the large map on the left-hand side of the figure. The map in the lower-right corner of the figure presents spatial variations in near-surface geology (data from ref.[1360]: Hartmann, J., & Moosdorf, N. (2012). The new global lithological map database GLiM: A representation of rock properties at the Earth surface. *Geochemistry, Geophysics, Geosystems* 13, doi.org/10.1029/2012GC004370). Imagery from https://www.arcgis.com/home/item.html?id=10df2279f9684e4a9f6a7f08febac2a9

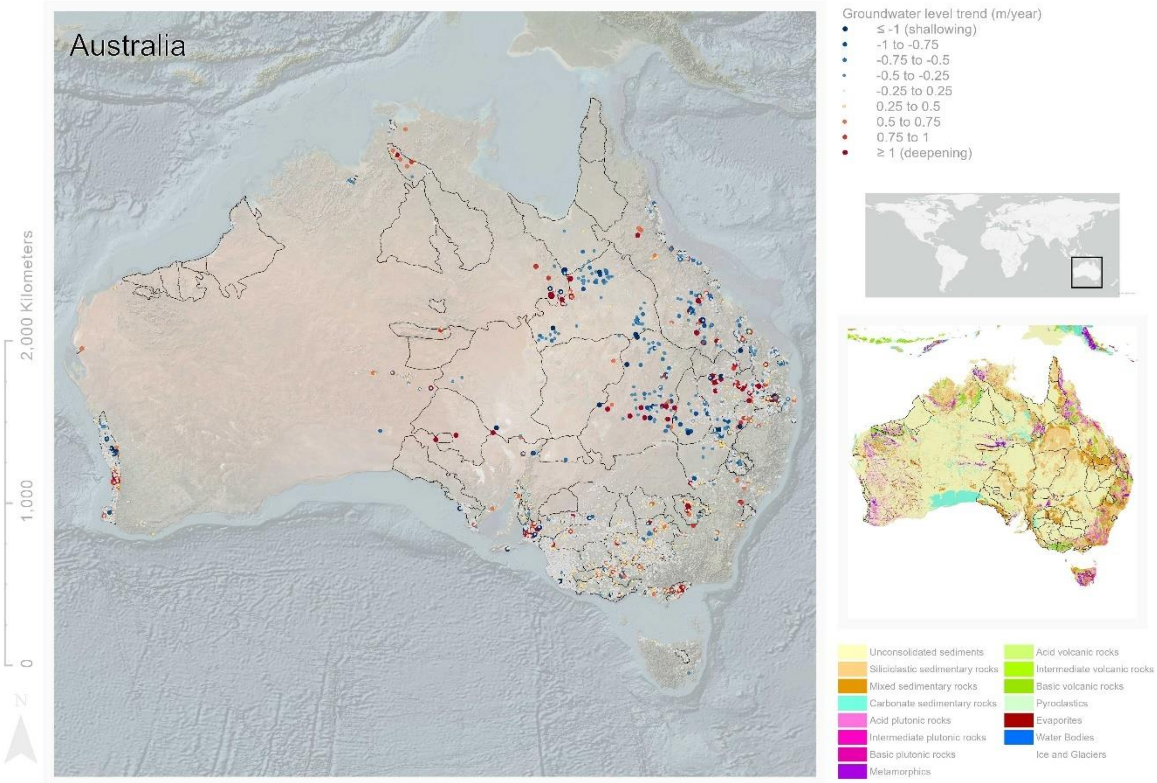

Supplementary Fig. 50. 21st-century trends in depth to groundwater in monitoring wells across Australia. The largest map (on the left-hand side of the figure) depicts 21st-century trends in depth to groundwater. Each point (coloured circle) represents a monitoring well; the colour of the point corresponds to the 21st-century trend in depth to groundwater for the monitoring well (Theil-Sen slope). Orange and red points represent monitoring wells where groundwater levels became deeper over time (Theil-Sen slope >0.25 m/year). Blue points represent monitoring wells where groundwater levels became shallower over time (see legend in upper-right corner of figure). The grey map (right-hand side of figure) shows the location of the area depicted in the large map on the left-hand side of the figure. The map in the lower-right corner of the figure presents spatial variations in near-surface geology (data from ref.[1360]: Hartmann, J., & Moosdorf, N. (2012). The new global lithological map database GLiM: A representation of rock properties at the Earth surface. *Geochemistry, Geophysics, Geosystems* 13, doi.org/10.1029/2012GC004370). Imagery from https://www.arcgis.com/home/item.html?id=10df2279f9684e4a9f6a7f08febac2a9

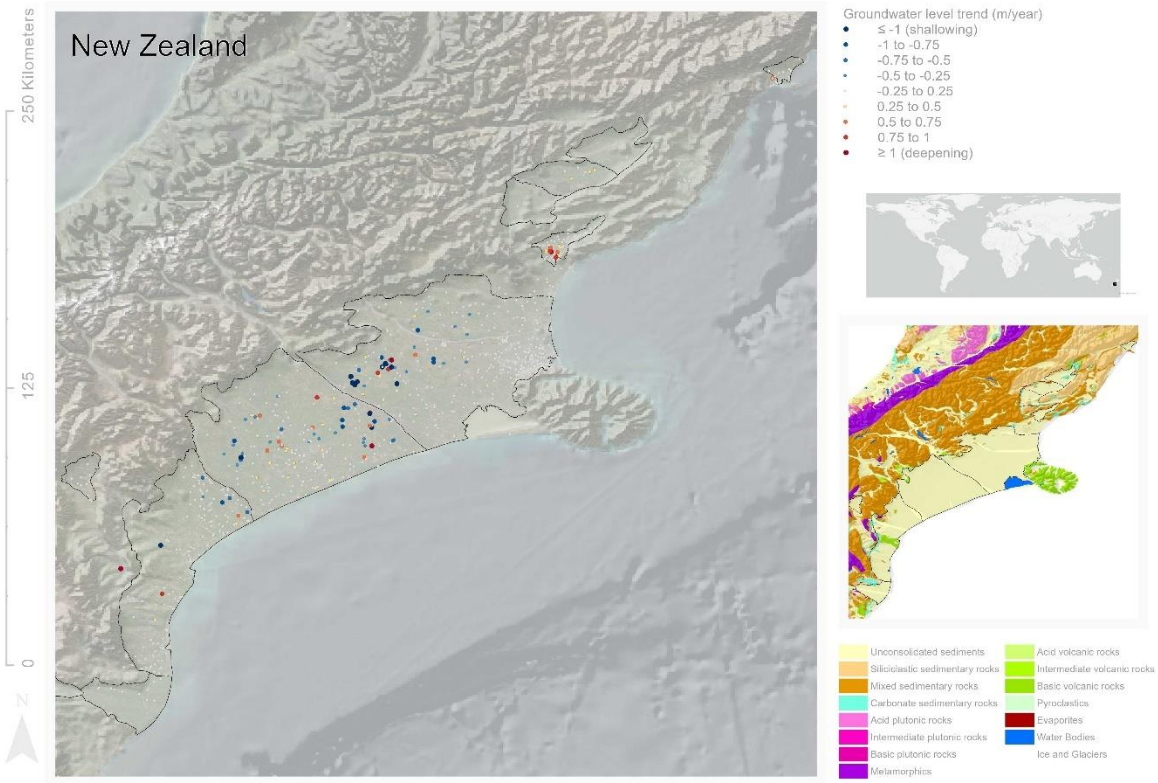

Supplementary Fig. 51. 21st-century trends in depth to groundwater in monitoring wells in New Zealand (east side of South Island). The largest map (on the left-hand side of the figure) depicts 21st-century trends in depth to groundwater. Each point (coloured circle) represents a monitoring well; the colour of the point corresponds to the 21st-century trend in depth to groundwater for the monitoring well (Theil-Sen slope). Orange and red points represent monitoring wells where groundwater levels became deeper over time (Theil-Sen slope >0.25 m/year). Blue points represent monitoring wells where groundwater levels became shallower over time (see legend in upper-right corner of figure). The grey map (right-hand side of figure) shows the location of the area depicted in the large map on the left-hand side of the figure. The map in the lower-right corner of the figure presents spatial variations in near-surface geology (data from ref.[1360]: Hartmann, J., & Moosdorf, N. (2012). The new global lithological map database GLiM: A representation of rock properties at the Earth surface. *Geochemistry, Geophysics, Geosystems* 13, doi.org/10.1029/2012GC004370). Imagery from https://www.arcgis.com/home/item.html?id=10df2279f9684e4a9f6a7f08febac2a9

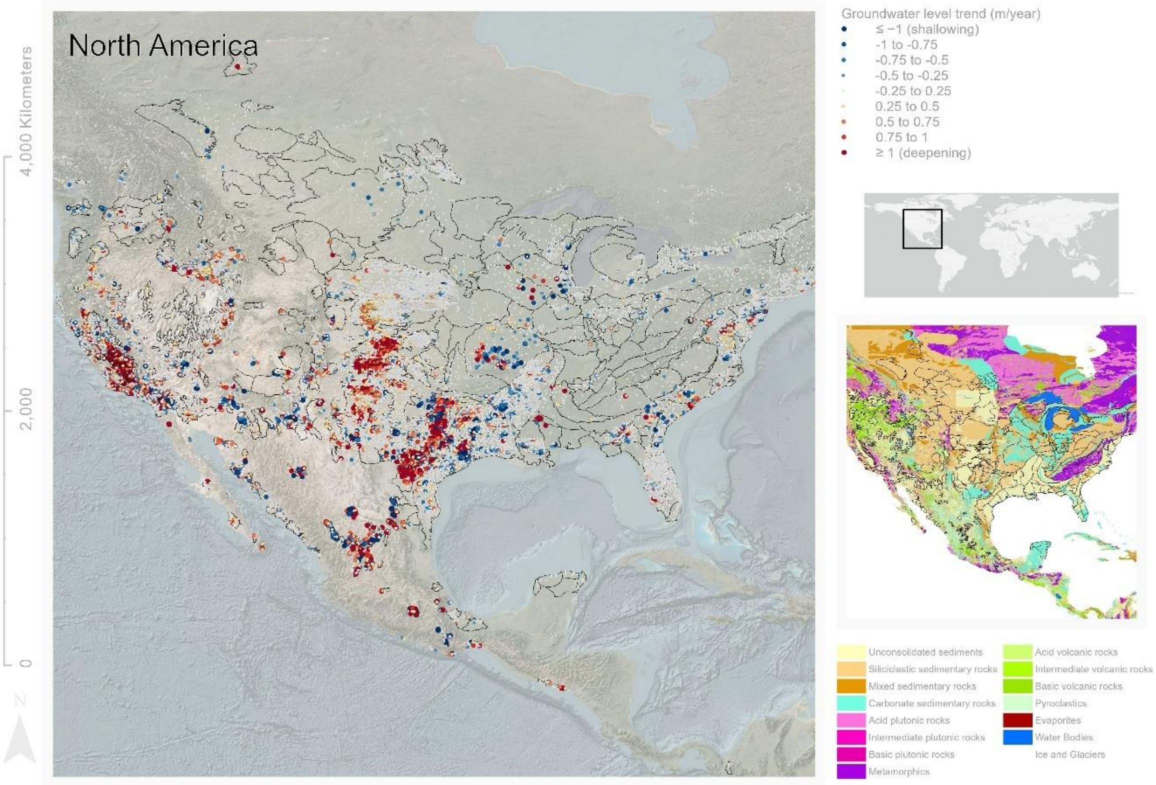

Supplementary Fig. 52. 21st-century trends in depth to groundwater in monitoring wells across North America. The largest map (on the left-hand side of the figure) depicts 21st-century trends in depth to groundwater. Each point (coloured circle) represents a monitoring well; the colour of the point corresponds to the 21st-century trend in depth to groundwater for the monitoring well (Theil-Sen slope). Orange and red points represent monitoring wells where groundwater levels became deeper over time (Theil-Sen slope >0.25 m/year). Blue points represent monitoring wells where groundwater levels became shallower over time (see legend in upper-right corner of figure). The grey map (right-hand side of figure) shows the location of the area depicted in the large map on the left-hand side of the figure. The map in the lower-right corner of the figure presents spatial variations in near-surface geology (data from ref.[1360]: Hartmann, J., & Moosdorf, N. (2012). The new global lithological map database GLiM: A representation of rock properties at the Earth surface. *Geochemistry, Geophysics, Geosystems* 13, doi.org/10.1029/2012GC004370). Imagery from https://www.arcgis.com/home/item.html?id=10df2279f9684e4a9f6a7f08febac2a9

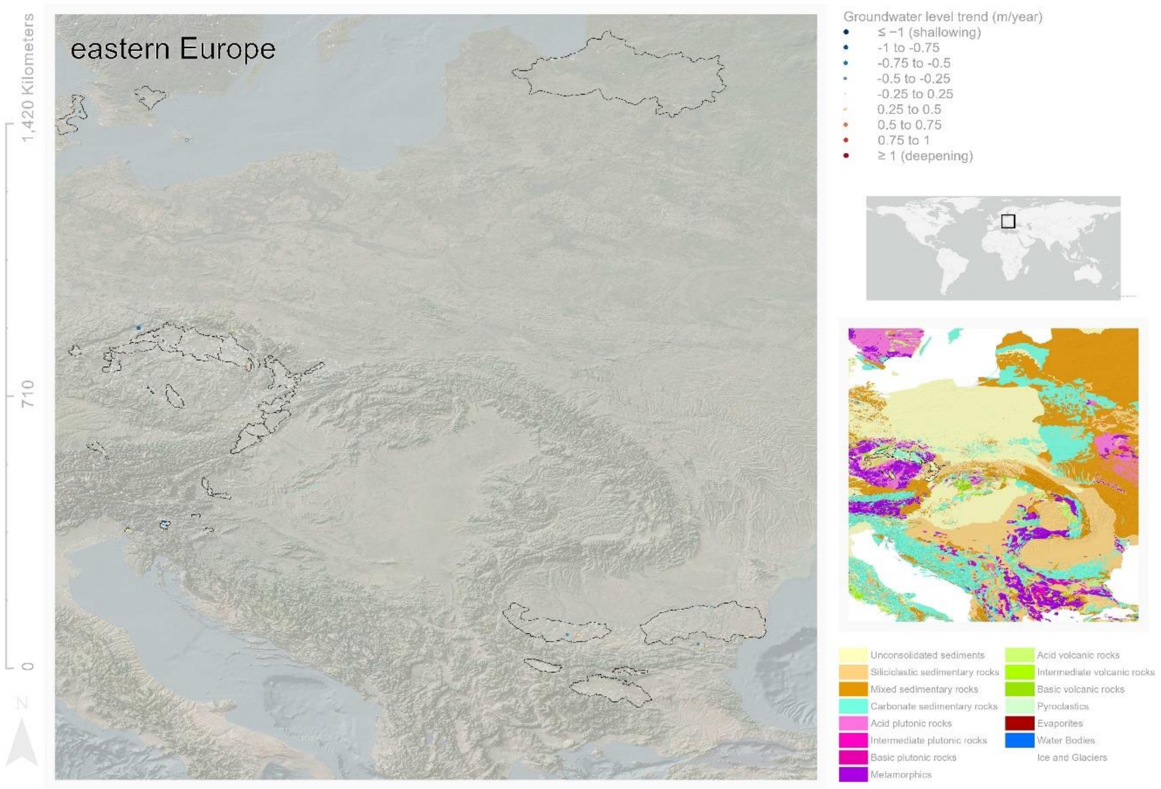

Supplementary Fig. 53. 21st-century trends in depth to groundwater in monitoring wells across eastern Europe. The largest map (on the left-hand side of the figure) depicts 21st-century trends in depth to groundwater. Each point (coloured circle) represents a monitoring well; the colour of the point corresponds to the 21st-century trend in depth to groundwater for the monitoring well (Theil-Sen slope). Orange and red points represent monitoring wells where groundwater levels became deeper over time (Theil-Sen slope >0.25 m/year). Blue points represent monitoring wells where groundwater levels became shallower over time (see legend in upper-right corner of figure). The grey map (right-hand side of figure) shows the location of the area depicted in the large map on the left-hand side of the figure. The map in the lower-right corner of the figure presents spatial variations in near-surface geology (data from ref.[1360]: Hartmann, J., & Moosdorf, N. (2012). The new global lithological map database GLiM: A representation of rock properties at the Earth surface. *Geochemistry, Geophysics, Geosystems* 13, doi.org/10.1029/2012GC004370). Imagery from https://www.arcgis.com/home/item.html?id=10df2279f9684e4a9f6a7f08febac2a9

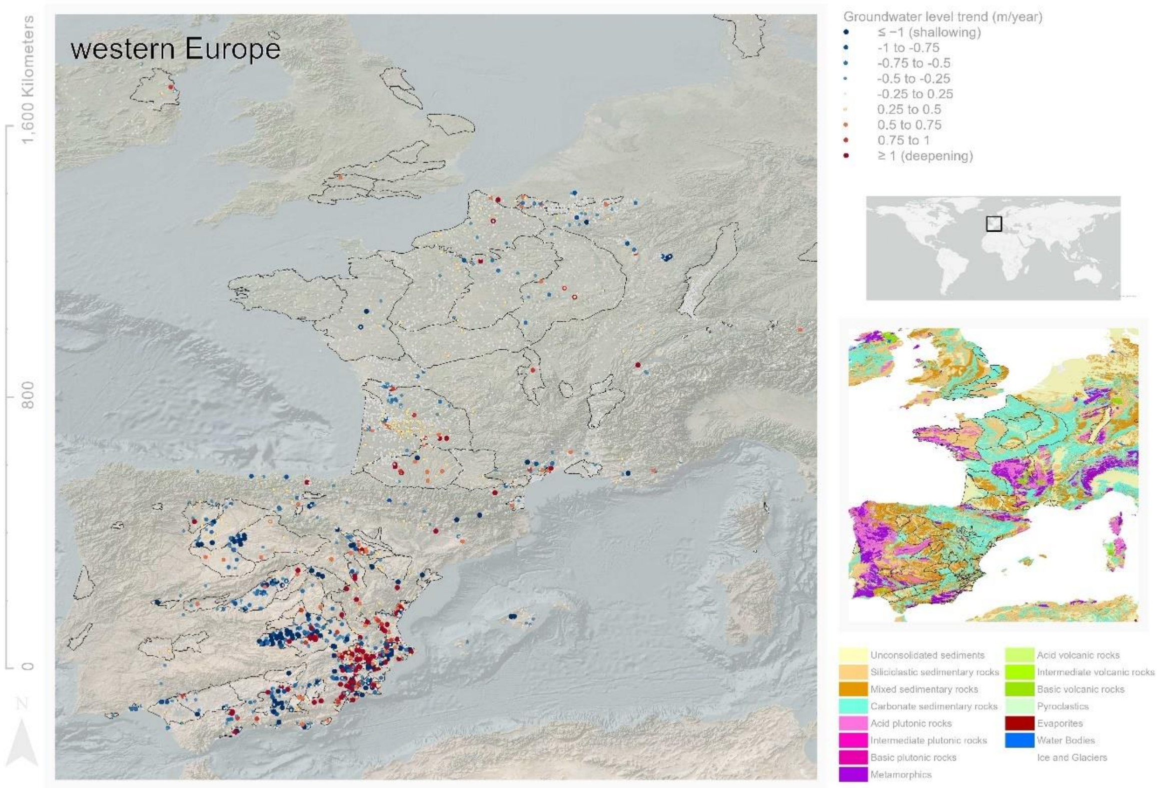

Supplementary Fig. 54. 21st-century trends in depth to groundwater in monitoring wells across western Europe. The largest map (on the left-hand side of the figure) depicts 21st-century trends in depth to groundwater. Each point (coloured circle) represents a monitoring well; the colour of the point corresponds to the 21st-century trend in depth to groundwater for the monitoring well (Theil-Sen slope). Orange and red points represent monitoring wells where groundwater levels became deeper over time (Theil-Sen slope >0.25 m/year). Blue points represent monitoring wells where groundwater levels became shallower over time (see legend in upper-right corner of figure). The grey map (right-hand side of figure) shows the location of the area depicted in the large map on the left-hand side of the figure. The map in the lower-right corner of the figure presents spatial variations in near-surface geology (data from ref.[1360]: Hartmann, J., & Moosdorf, N. (2012). The new global lithological map database GLiM: A representation of rock properties at the Earth surface. *Geochemistry, Geophysics, Geosystems* 13, doi.org/10.1029/2012GC004370). Imagery from https://www.arcgis.com/home/item.html?id=10df2279f9684e4a9f6a7f08febac2a9

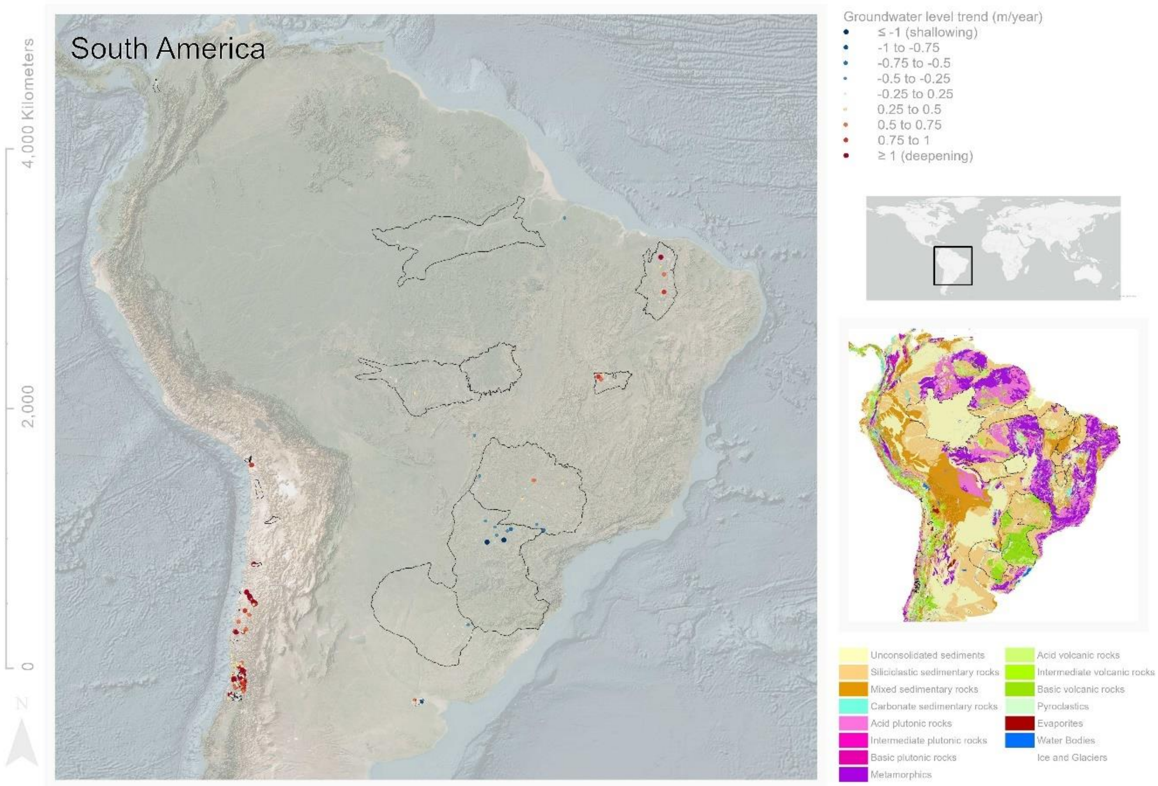

Supplementary Fig. 55. 21st-century trends in depth to groundwater in monitoring wells across South America. The largest map (on the left-hand side of the figure) depicts 21st-century trends in depth to groundwater. Each point (coloured circle) represents a monitoring well; the colour of the point corresponds to the 21st-century trend in depth to groundwater for the monitoring well (Theil-Sen slope). Orange and red points represent monitoring wells where groundwater levels became deeper over time (Theil-Sen slope >0.25 m/year). Blue points represent monitoring wells where groundwater levels became shallower over time (see legend in upper-right corner of figure). The grey map (right-hand side of figure) shows the location of the area depicted in the large map on the left-hand side of the figure. The map in the lower-right corner of the figure presents spatial variations in near-surface geology (data from ref.[1360]: Hartmann, J., & Moosdorf, N. (2012). The new global lithological map database GLiM: A representation of rock properties at the Earth surface. *Geochemistry, Geophysics, Geosystems* 13, doi.org/10.1029/2012GC004370). Imagery from https://www.arcgis.com/home/item.html?id=10df2279f9684e4a9f6a7f08febac2a9

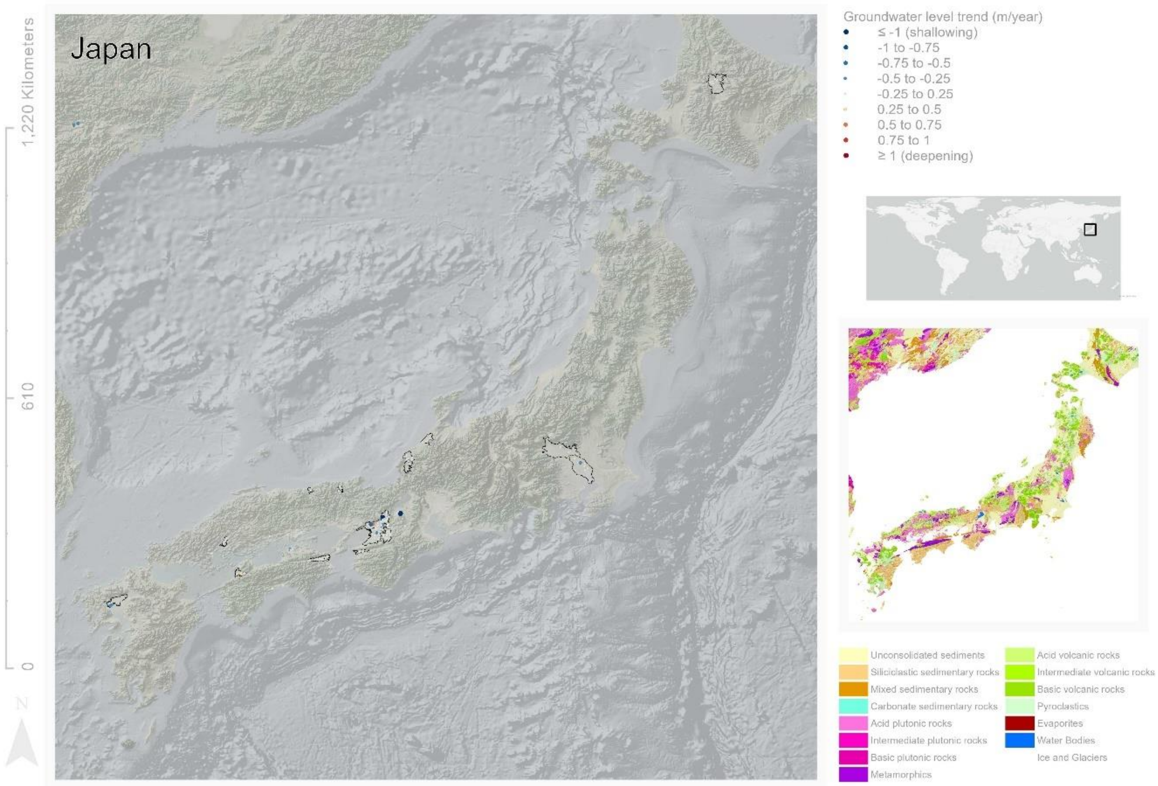

Supplementary Fig. 56. 21st-century trends in depth to groundwater in monitoring wells across Japan. The largest map (on the left-hand side of the figure) depicts 21st-century trends in depth to groundwater. Each point (coloured circle) represents a monitoring well; the colour of the point corresponds to the 21st-century trend in depth to groundwater for the monitoring well (Theil-Sen slope). Orange and red points represent monitoring wells where groundwater levels became deeper over time (Theil-Sen slope >0.25 m/year). Blue points represent monitoring wells where groundwater levels became shallower over time (see legend in upper-right corner of figure). The grey map (right-hand side of figure) shows the location of the area depicted in the large map on the left-hand side of the figure. The map in the lower-right corner of the figure presents spatial variations in near-surface geology (data from ref.[1360]: Hartmann, J., & Moosdorf, N. (2012). The new global lithological map database GLiM: A representation of rock properties at the Earth surface. *Geochemistry, Geophysics, Geosystems* 13, doi.org/10.1029/2012GC004370). Imagery from https://www.arcgis.com/home/item.html?id=10df2279f9684e4a9f6a7f08febac2a9

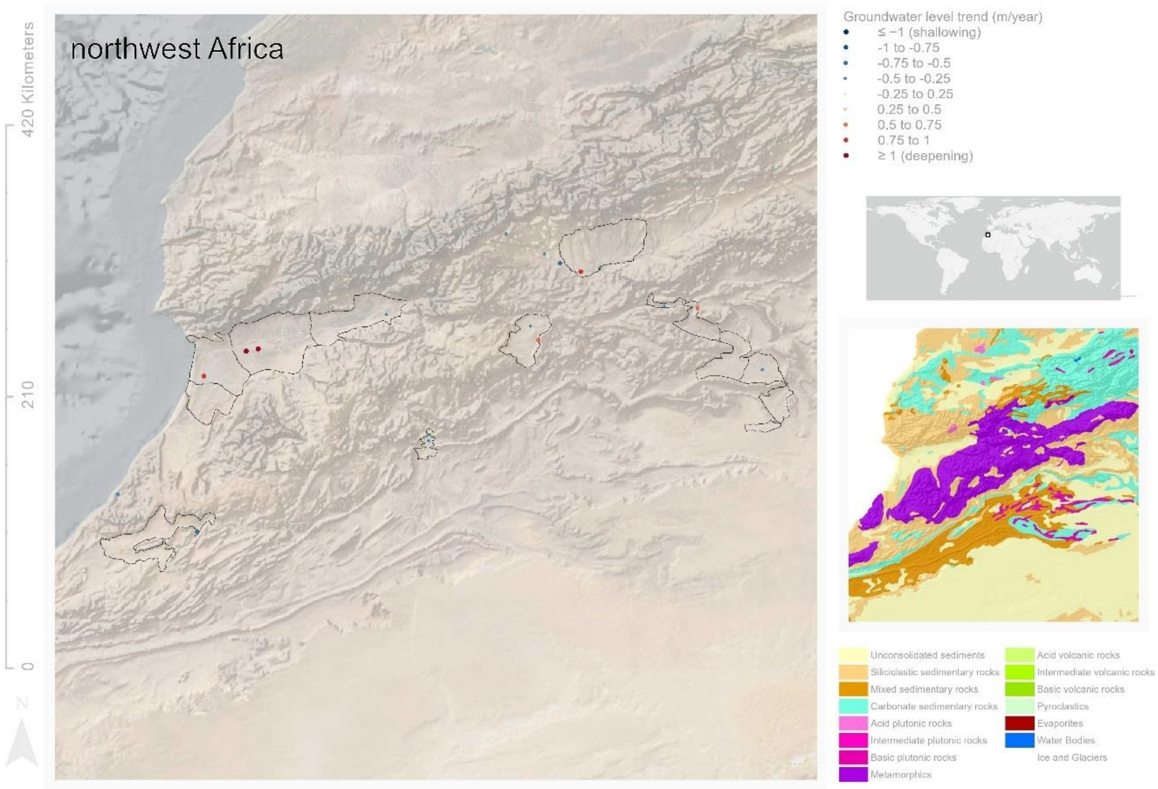

Supplementary Fig. 57. 21st-century trends in depth to groundwater in monitoring wells across northwest Africa. The largest map (on the left-hand side of the figure) depicts 21st-century trends in depth to groundwater. Each point (coloured circle) represents a monitoring well; the colour of the point corresponds to the 21st-century trend in depth to groundwater for the monitoring well (Theil-Sen slope). Orange and red points represent monitoring wells where groundwater levels became deeper over time (Theil-Sen slope >0.25 m/year). Blue points represent monitoring wells where groundwater levels became shallower over time (see legend in upper-right corner of figure). The grey map (right-hand side of figure) shows the location of the area depicted in the large map on the left-hand side of the figure. The map in the lower-right corner of the figure presents spatial variations in near-surface geology (data from ref.[1360]: Hartmann, J., & Moosdorf, N. (2012). The new global lithological map database GLiM: A representation of rock properties at the Earth surface. *Geochemistry, Geophysics, Geosystems* 13, doi.org/10.1029/2012GC004370). Imagery from https://www.arcgis.com/home/item.html?id=10df2279f9684e4a9f6a7f08febac2a9

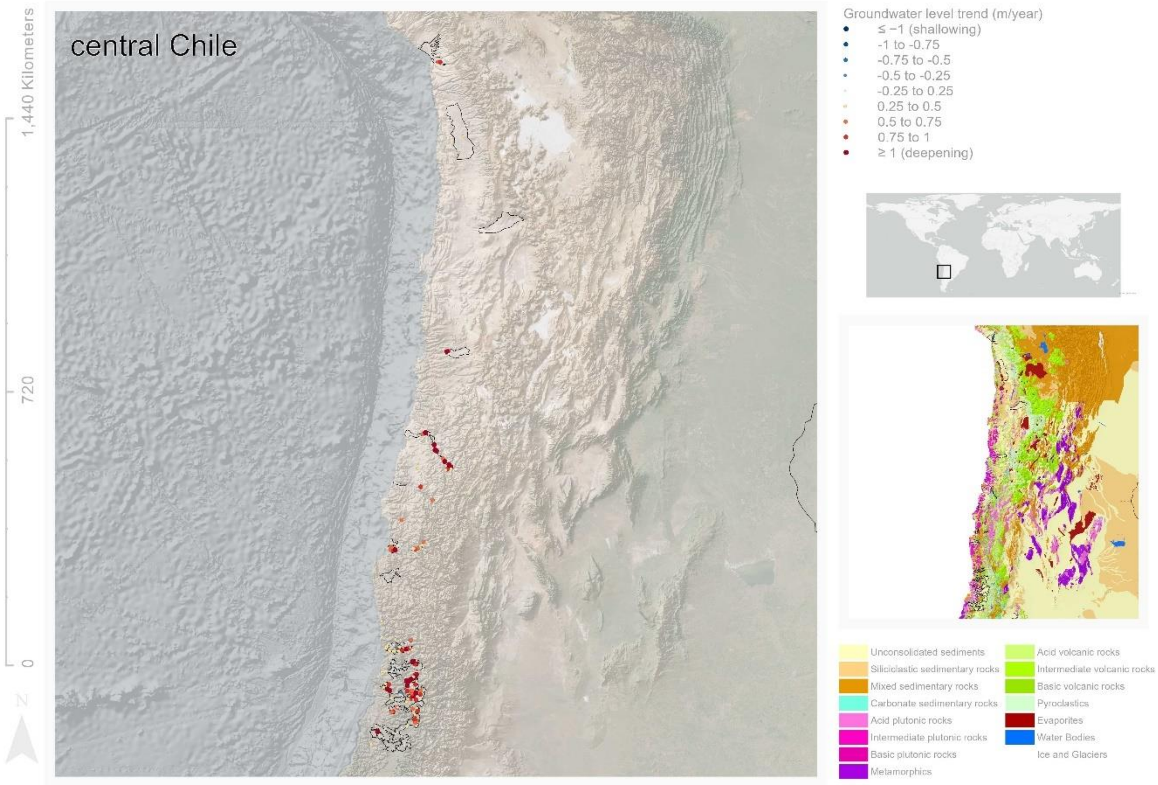

Supplementary Fig. 58. 21st-century trends in depth to groundwater in monitoring wells across central Chile. The largest map (on the left-hand side of the figure) depicts 21st-century trends in depth to groundwater. Each point (coloured circle) represents a monitoring well; the colour of the point corresponds to the 21st-century trend in depth to groundwater for the monitoring well (Theil-Sen slope). Orange and red points represent monitoring wells where groundwater levels became deeper over time (Theil-Sen slope >0.25 m/year). Blue points represent monitoring wells where groundwater levels became shallower over time (see legend in upper-right corner of figure). The grey map (right-hand side of figure) shows the location of the area depicted in the large map on the left-hand side of the figure. The map in the lower-right corner of the figure presents spatial variations in near-surface geology (data from ref.[1360]: Hartmann, J., & Moosdorf, N. (2012). The new global lithological map database GLiM: A representation of rock properties at the Earth surface. *Geochemistry, Geophysics, Geosystems* 13, doi.org/10.1029/2012GC004370). Imagery from https://www.arcgis.com/home/item.html?id=10df2279f9684e4a9f6a7f08febac2a9

17 SPATIAL VARIABILITY IN GROUNDWATER LEVEL TRENDS WITHIN INDIVIDUAL AQUIFER SYSTEMS

Here we present maps of 21st-century trends in depth to groundwater (Theil-Sen slopes) for individual monitoring wells within the boundaries of an aquifer system. We present maps for 207 of our 1,693 aquifer systems that met both of the following criteria: (i) the aquifer system contains at least 10 monitoring wells with adequate groundwater level time series data for analysis, and (ii) the density of monitoring wells (i.e., wells per unit area) within the aquifer system is greater than 1 well per 10 km^2.

The following figures contain similar content for different aquifer systems.

(panel a) The large map on the left-hand side (panel a) depicts 21st-century groundwater level trends over time. Each point (coloured circle) represents a monitoring well; the colour of the point corresponds to the 21st-century trend in depth to groundwater for the monitoring well (Theil-Sen slope). Orange and red points represent monitoring wells where the Theil-Sen slope suggests groundwater levels became deeper over time. Blue points represent monitoring wells where groundwater levels became shallower over time (see legend in upper-right corner of figure). The aquifer-scale trend in depth to groundwater stated in the title (i.e., in the text at the top of the figure) is the median Theil-Sen slope among all monitoring wells within the boundaries of the aquifer system. The grey map (right hand side of figure) shows the location of the study area (i.e., the area depicted in the large map on the left side of the figure). Imagery from: https://www.arcgis.com/home/item.html?id=10df2279f9684e4a9f6a7f08febac2a9

(panels b-d) The maps along the bottom of the figure present spatial variations in average annual precipitation divided by potential evapotranspiration (panel b), the percent of land under cultivation (panel c), and near-surface geology (data from ref.[1360]: Hartmann, J., & Moosdorf, N. (2012). The new global lithological map database GLiM: A representation of rock properties at the Earth surface. *Geochemistry, Geophysics, Geosystems* **13,** doi.org/10.1029/2012GC004370). The extent of the map matches that in panel a. The black lines outline the boundaries of aquifer systems.

Abdan-Dayer Coastal Plain (median Theil-Sen slope of 0.2 m/year)

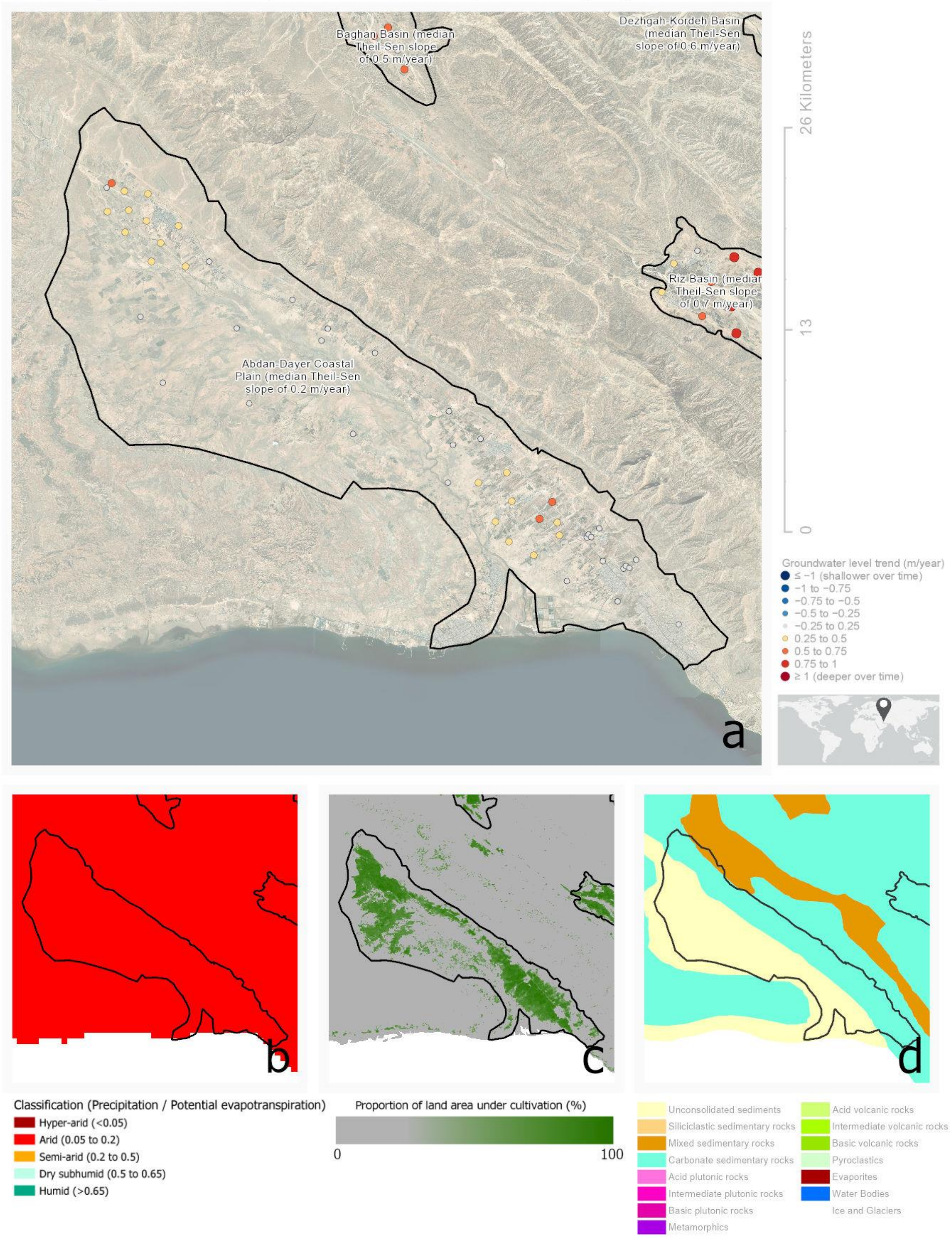

Supplementary Fig. 59. Spatial variability of trends in depth to groundwater Abdan-Dayer Coastal Plain. For detailed description of the contents of each panel see the text at the beginning of Supplementary Note 17.

Anderson Valley (median Theil-Sen slope of 0.1 m/year)

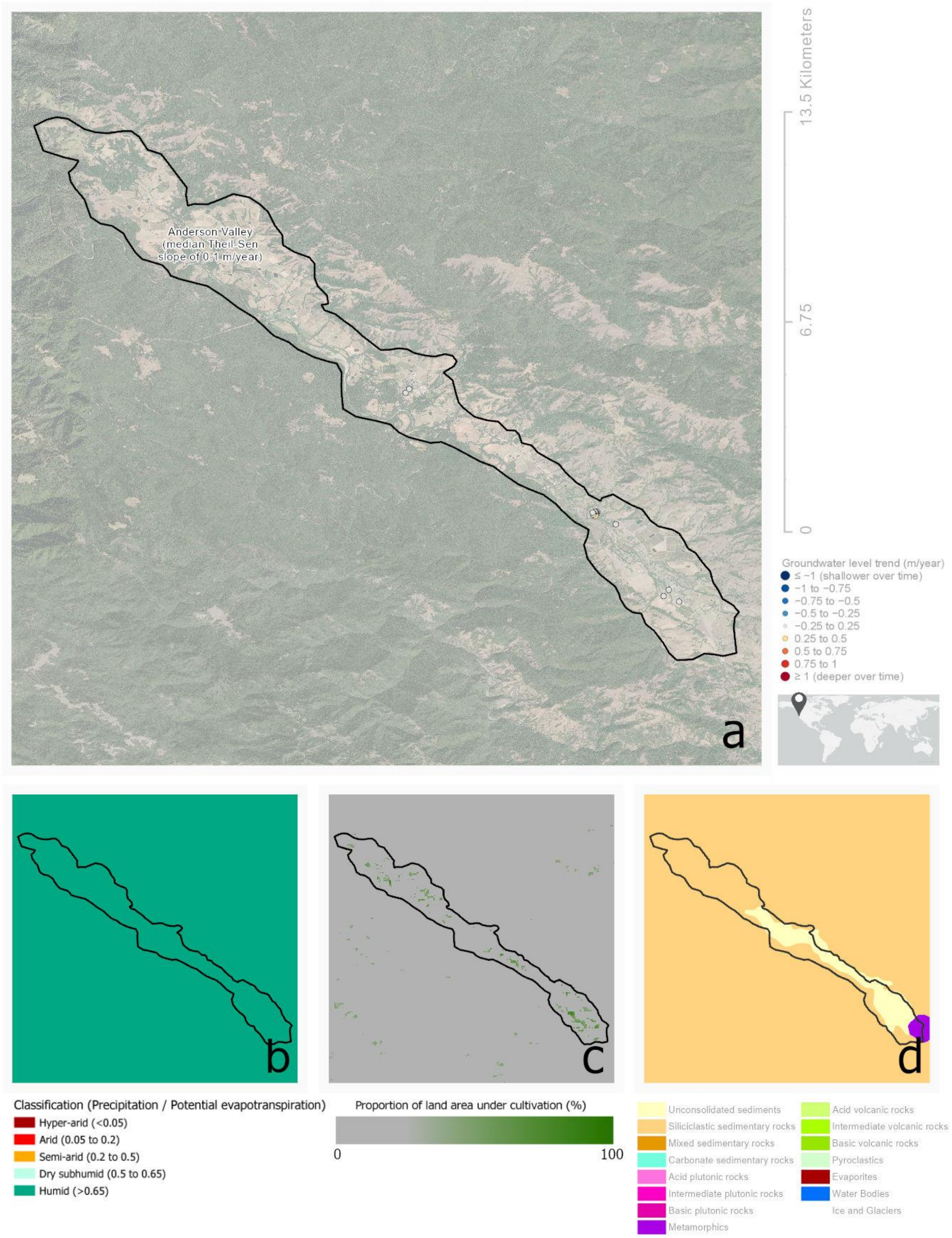

Supplementary Fig. 60. Spatial variability of trends in depth to groundwater Anderson Valley. For detailed description of the contents of each panel see the text at the beginning of Supplementary Note 17.

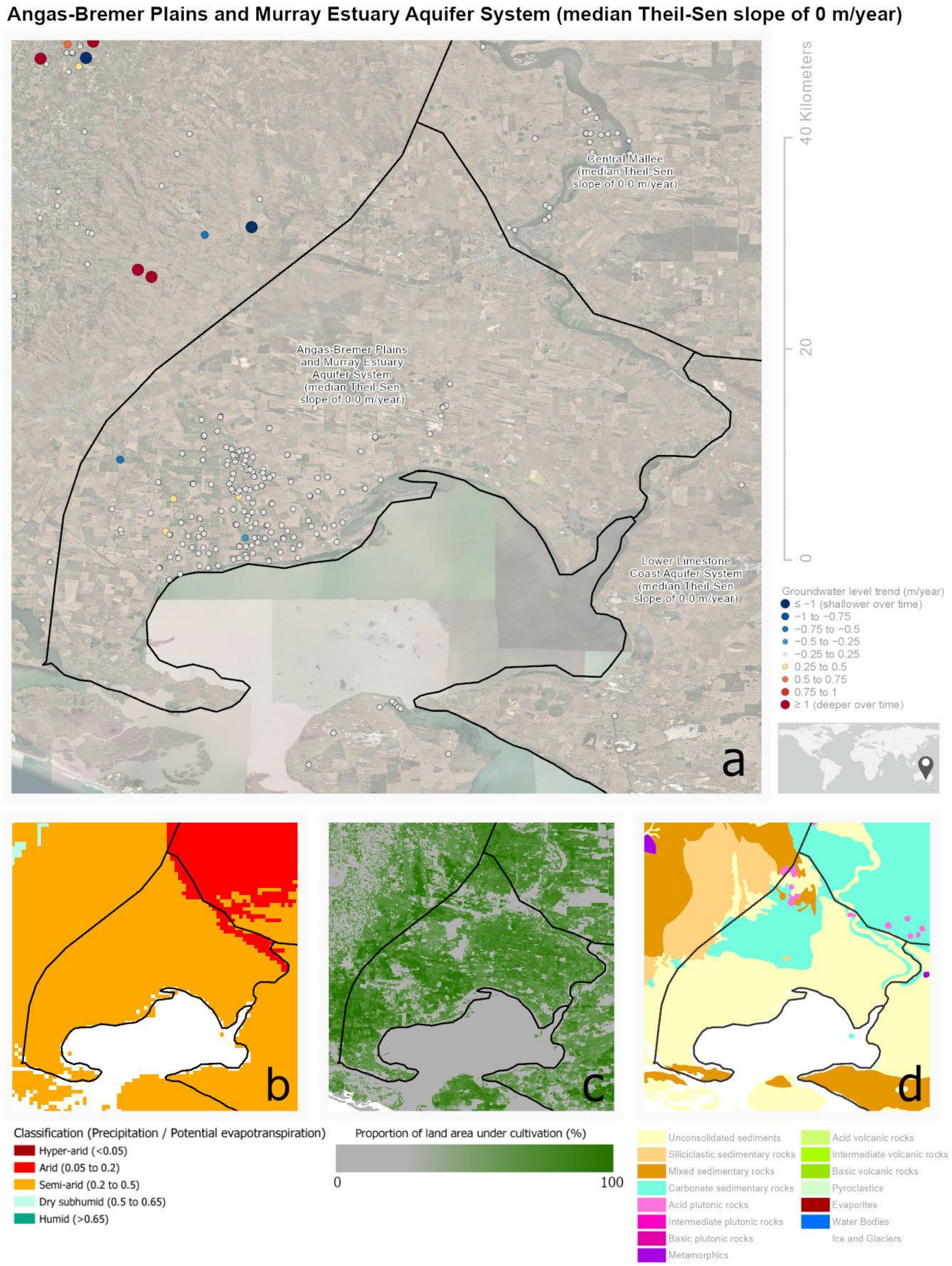

Supplementary Fig. 61. Spatial variability of trends in depth to groundwater Angas-Bremer Plains and Murray Estuary Aquifer System (within the broader Murray Basin). For detailed description of the contents of each panel see the text at the beginning of Supplementary Note 17.

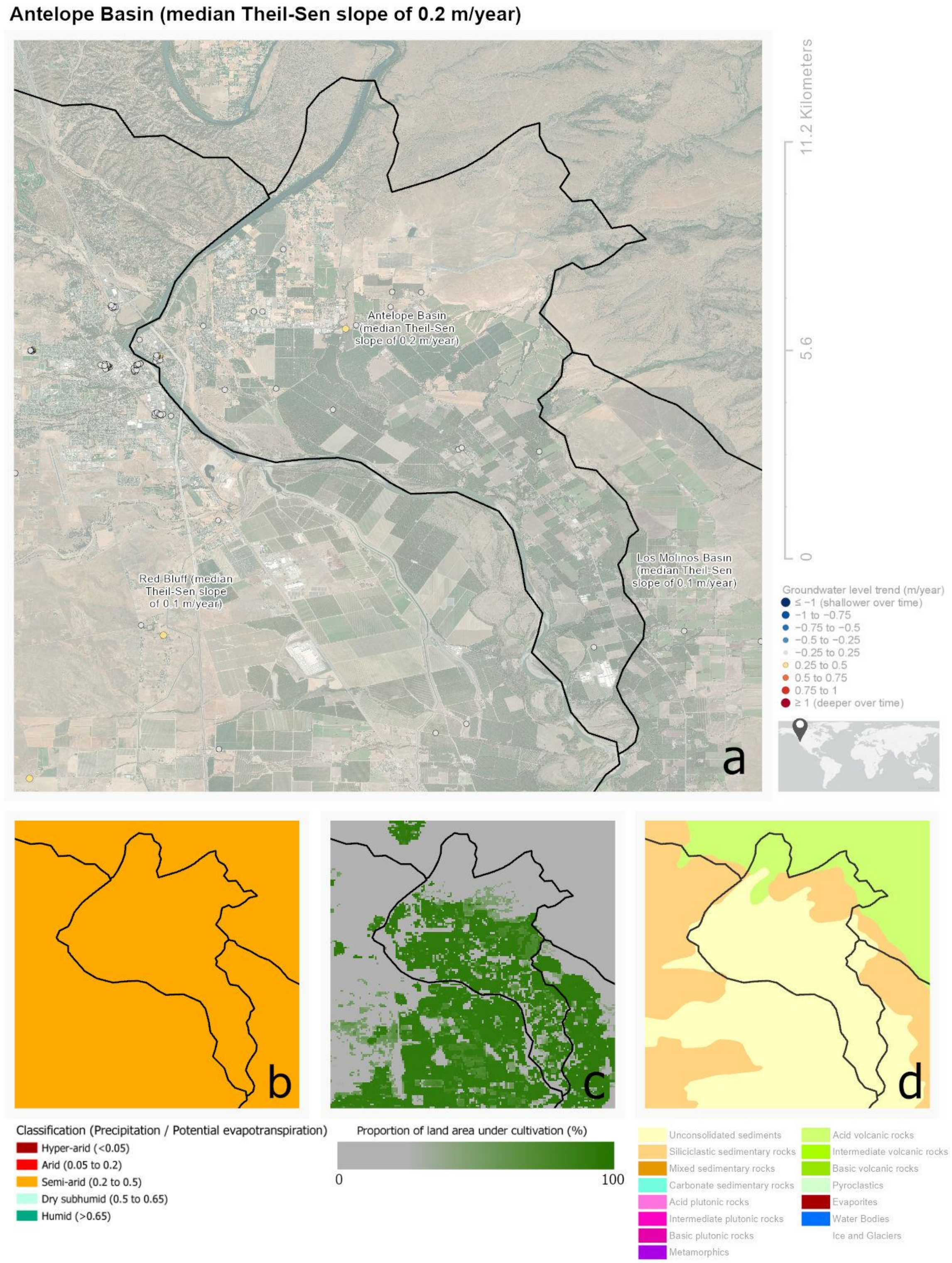

Supplementary Fig. 62. Spatial variability of trends in depth to groundwater Antelope Basin (within the broader Sacramento Valley of the Central Valley). For detailed description of the contents of each panel see the text at the beginning of Supplementary Note 17.

Arriga Flats (median Theil-Sen slope of -0.1 m/year)

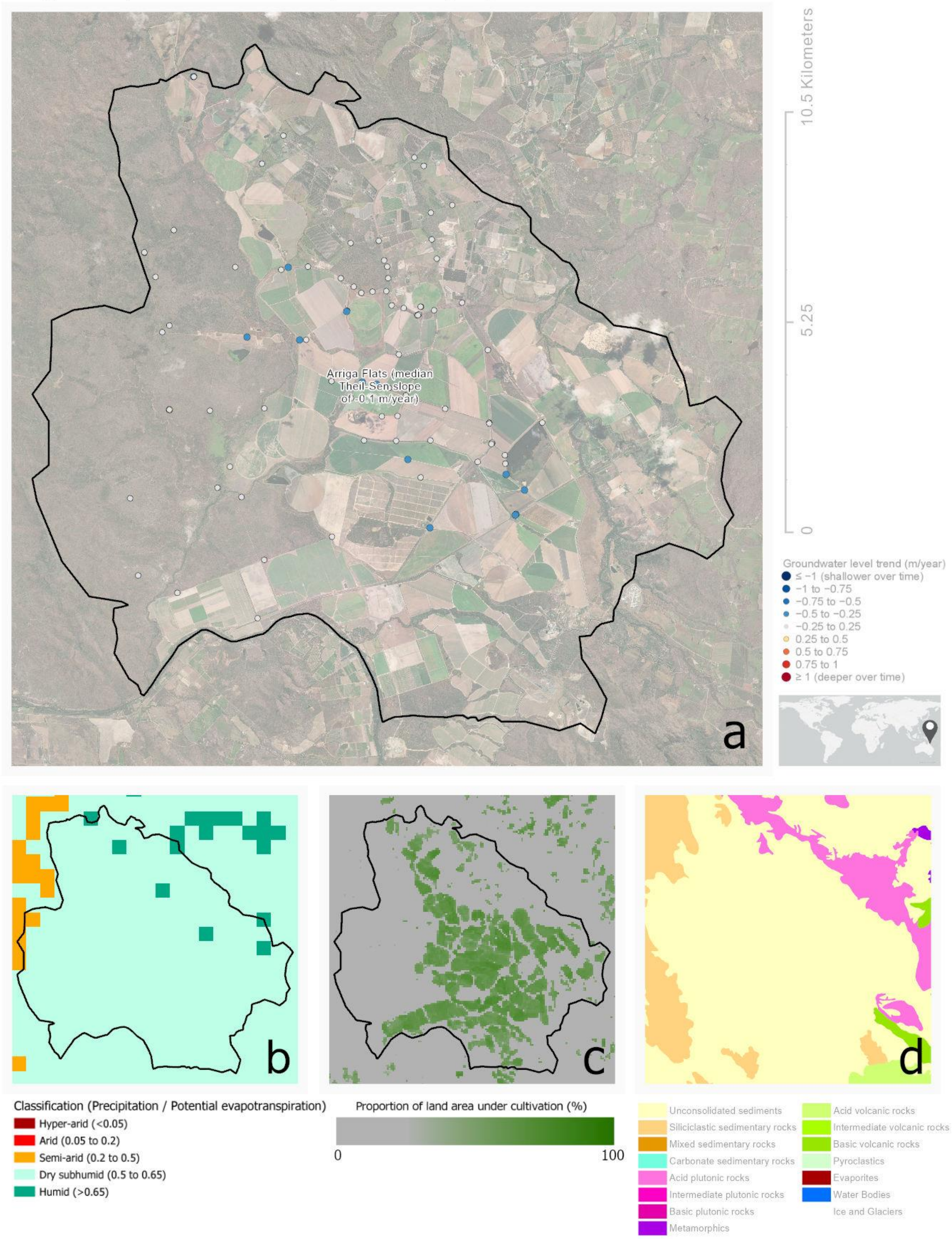

Supplementary Fig. 63. Spatial variability of trends in depth to groundwater Arriga Flats. For detailed description of the contents of each panel see the text at the beginning of Supplementary Note 17.

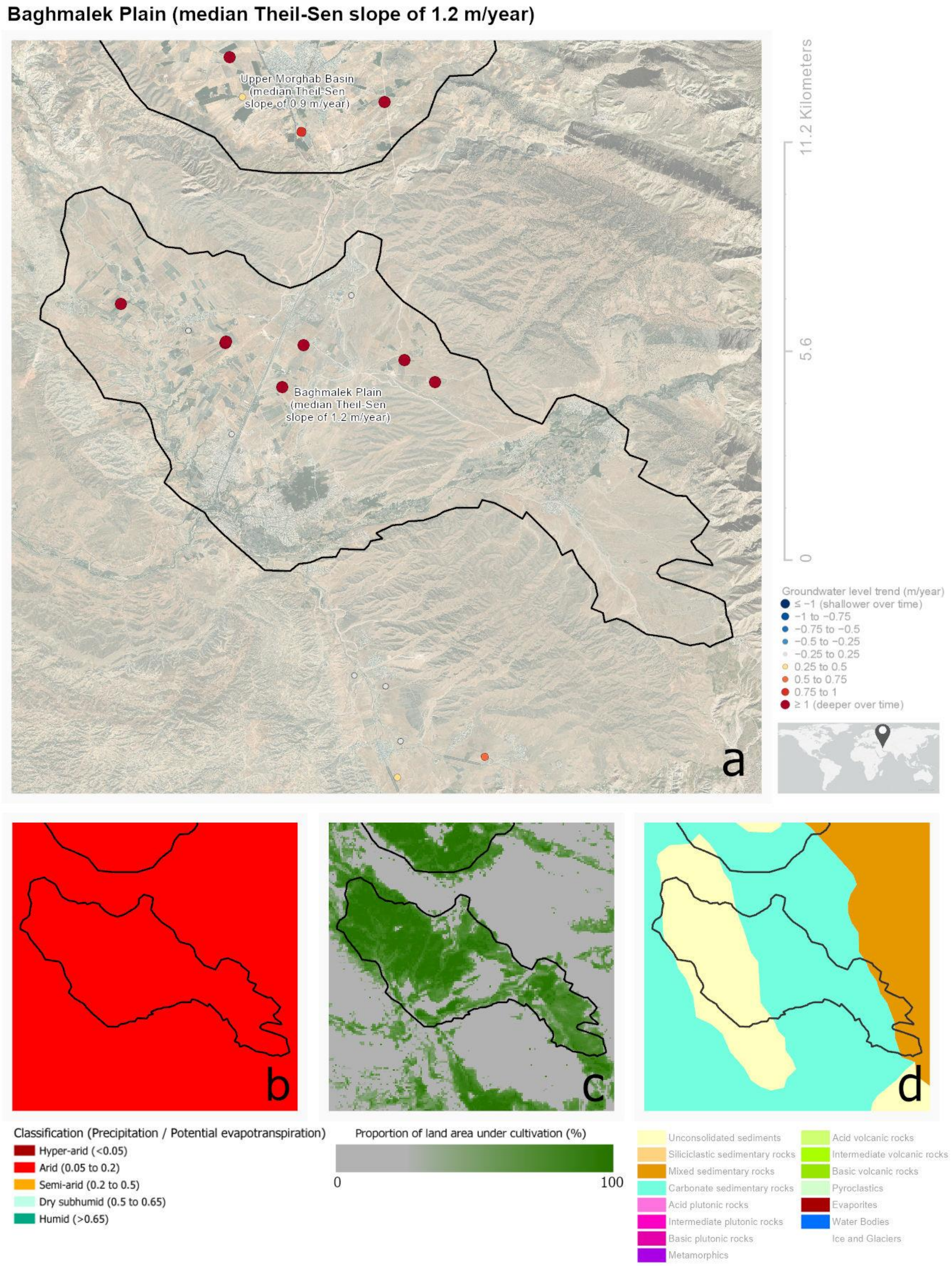

Supplementary Fig. 64. Spatial variability of trends in depth to groundwater Baghmalek Plain. For detailed description of the contents of each panel see the text at the beginning of Supplementary Note 17.

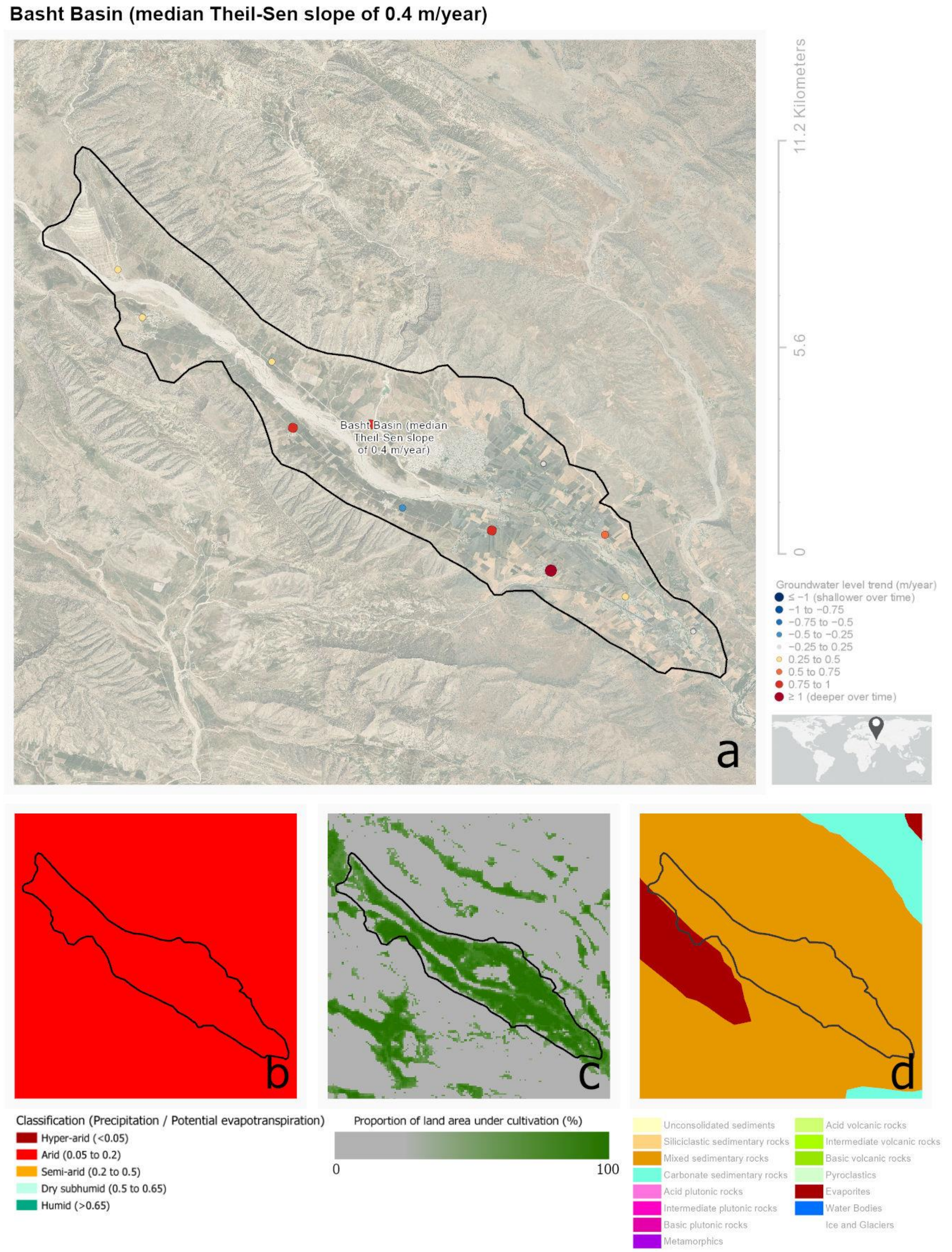

Supplementary Fig. 65. Spatial variability of trends in depth to groundwater Basht Basin. For detailed description of the contents of each panel see the text at the beginning of Supplementary Note 17.

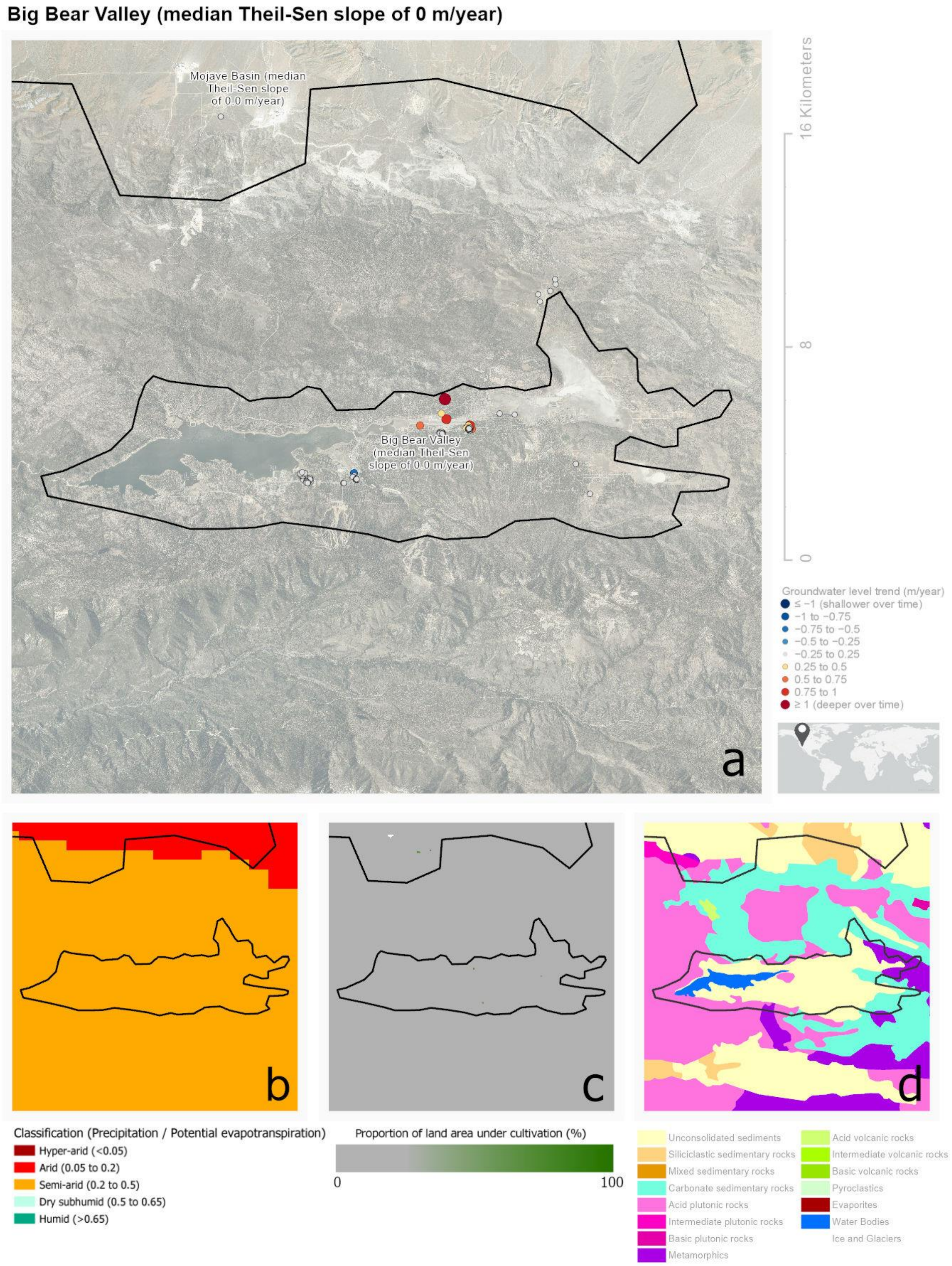

Supplementary Fig. 66. Spatial variability of trends in depth to groundwater Big Bear Valley. For detailed description of the contents of each panel see the text at the beginning of Supplementary Note 17.

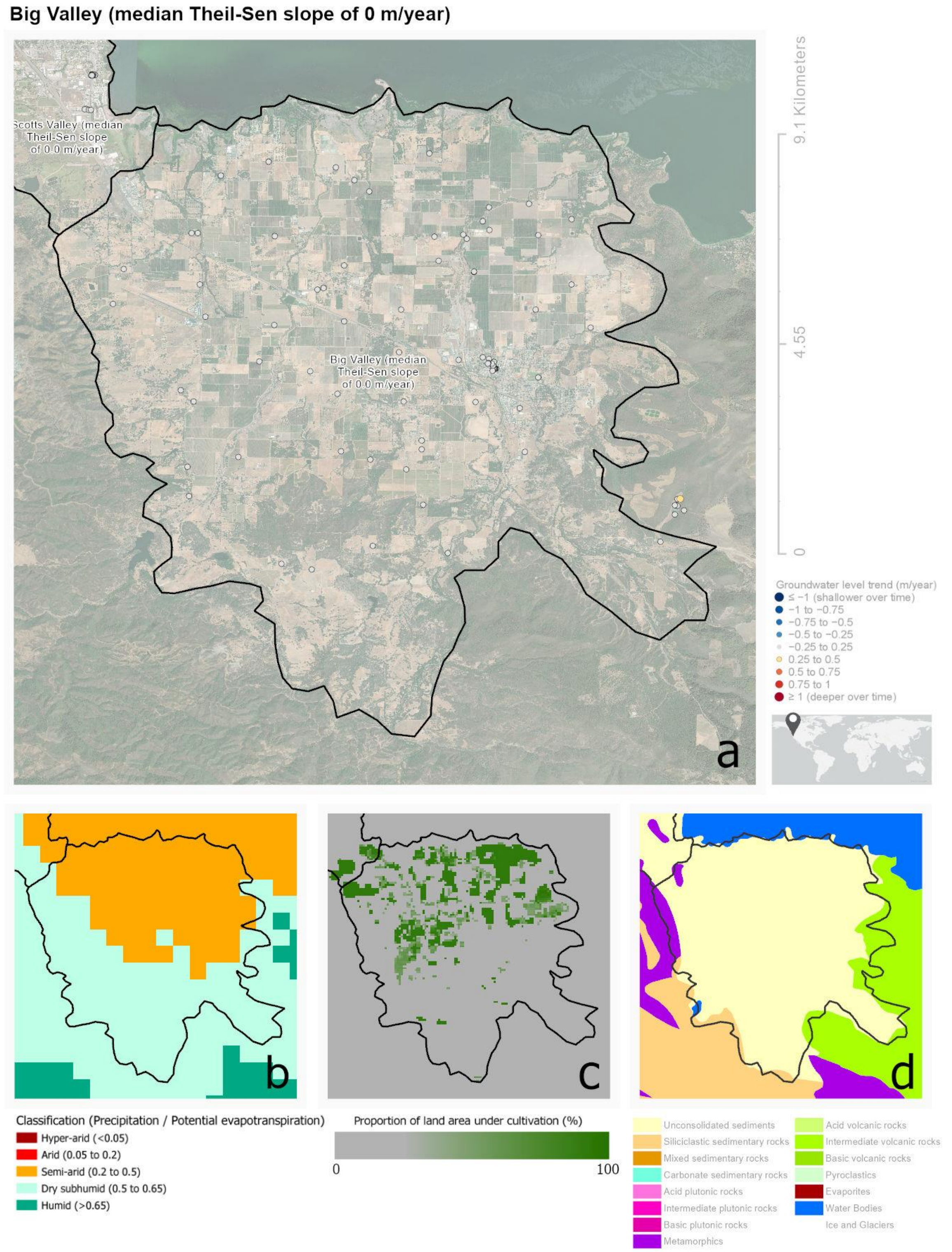

Supplementary Fig. 67. Spatial variability of trends in depth to groundwater Big Valley. For detailed description of the contents of each panel see the text at the beginning of Supplementary Note 17.

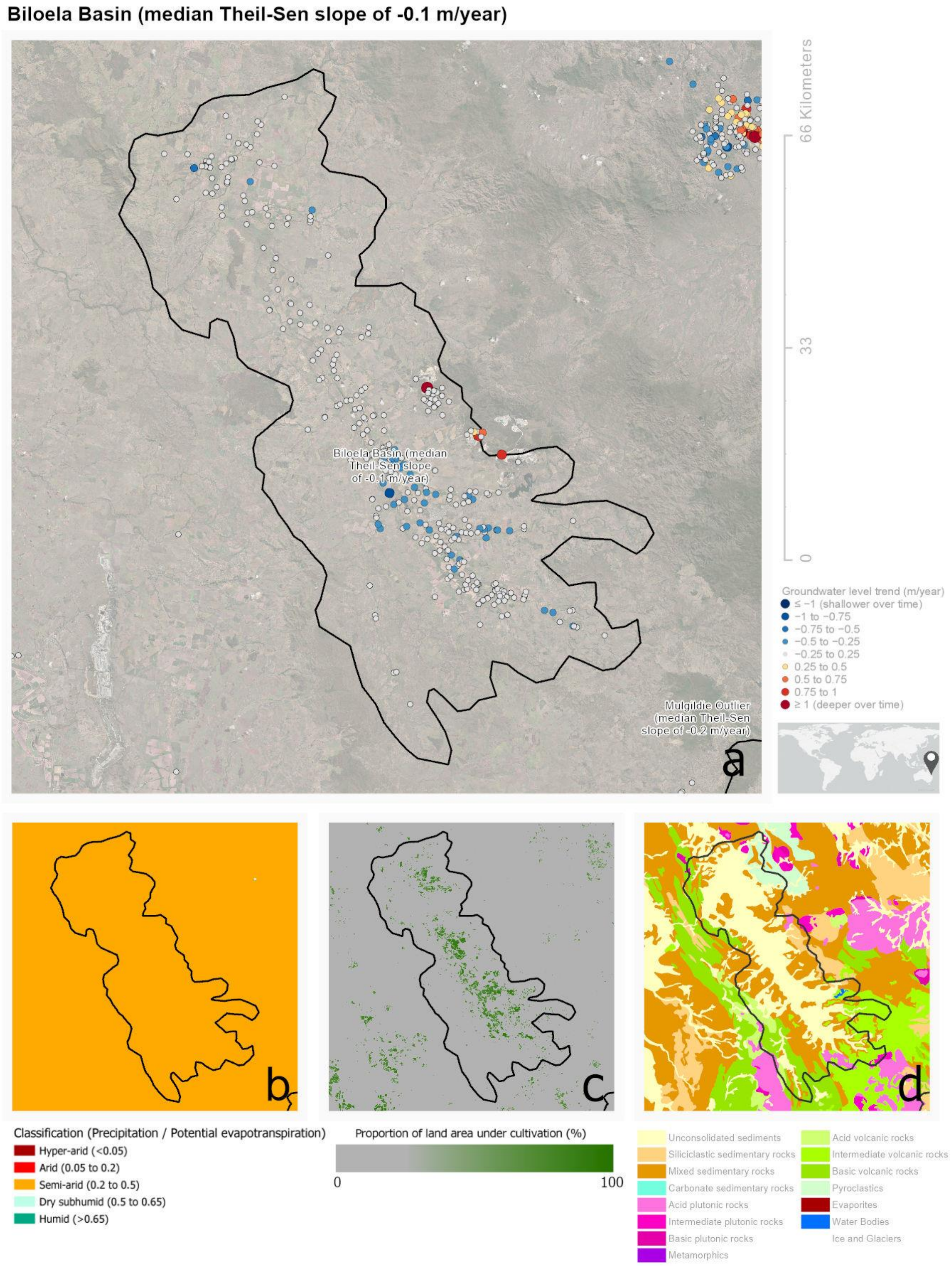

Supplementary Fig. 68. Spatial variability of trends in depth to groundwater Biloela Basin. For detailed description of the contents of each panel see the text at the beginning of Supplementary Note 17.

Bishop Basin (median Theil-Sen slope of 0 m/year)

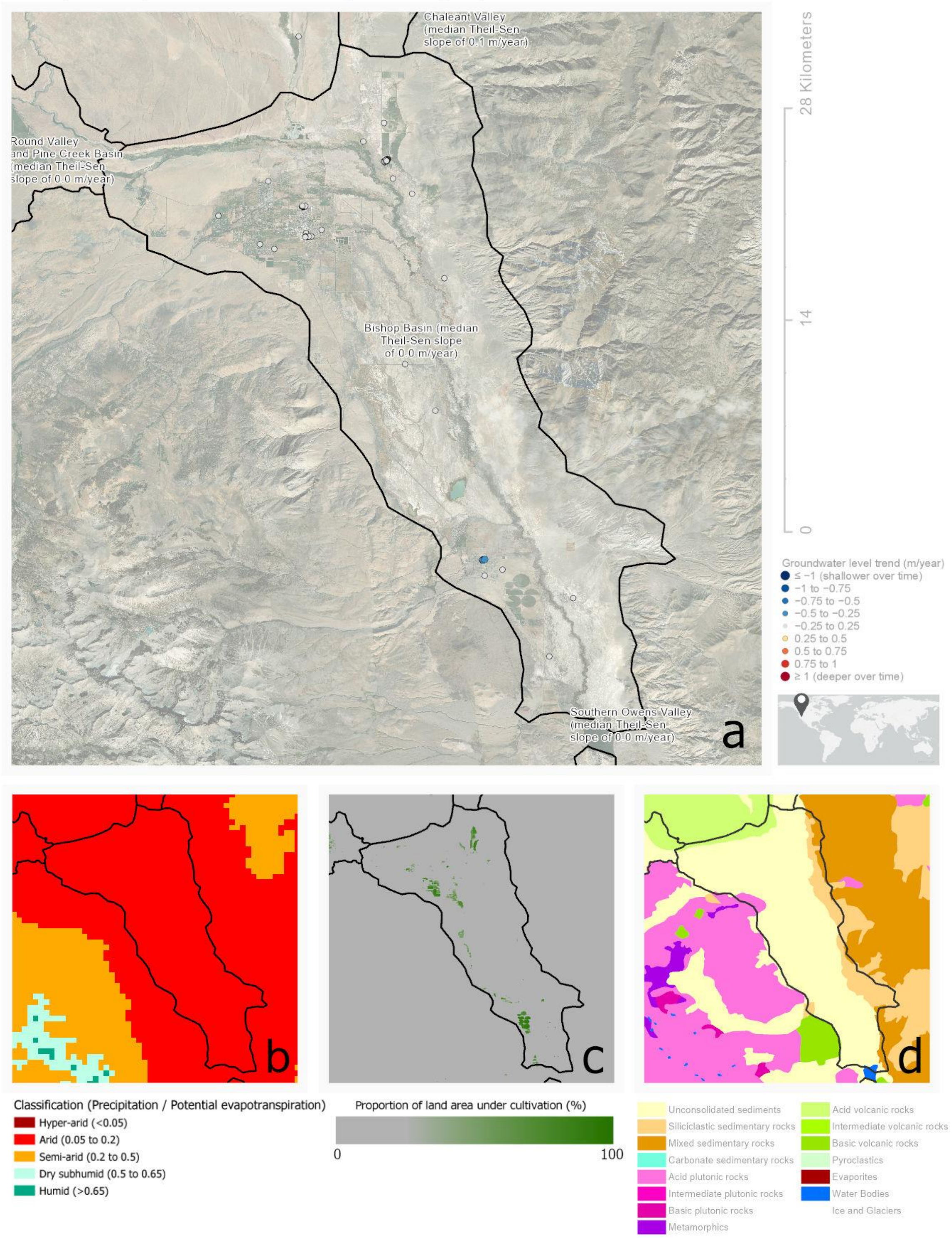

Supplementary Fig. 69. Spatial variability of trends in depth to groundwater Bishop Basin. For detailed description of the contents of each panel see the text at the beginning of Supplementary Note 17.

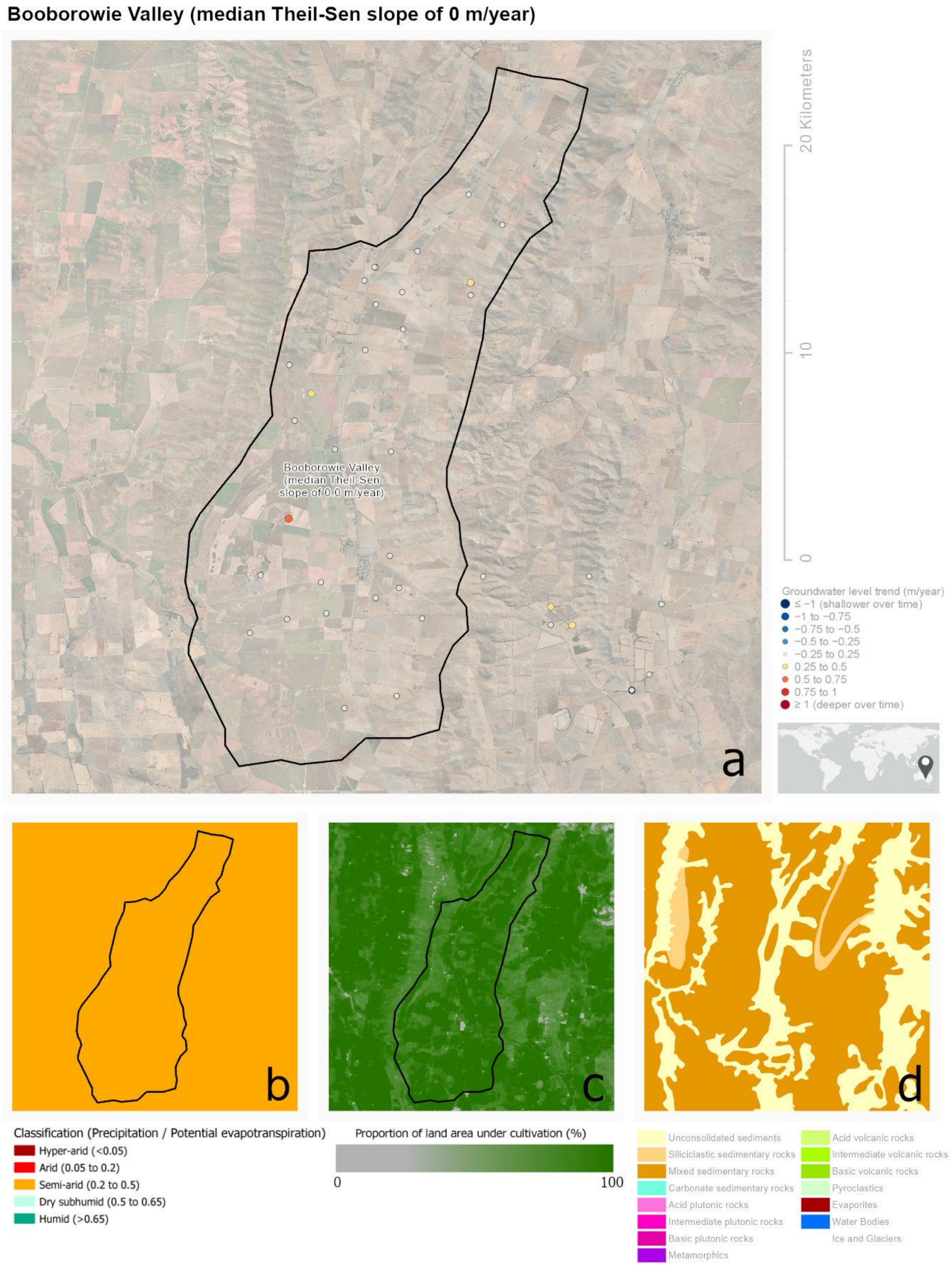

Supplementary Fig. 70. Spatial variability of trends in depth to groundwater Booborowie Valley. For detailed description of the contents of each panel see the text at the beginning of Supplementary Note 17.

Bridgeport Valley (median Theil-Sen slope of 0 m/year)

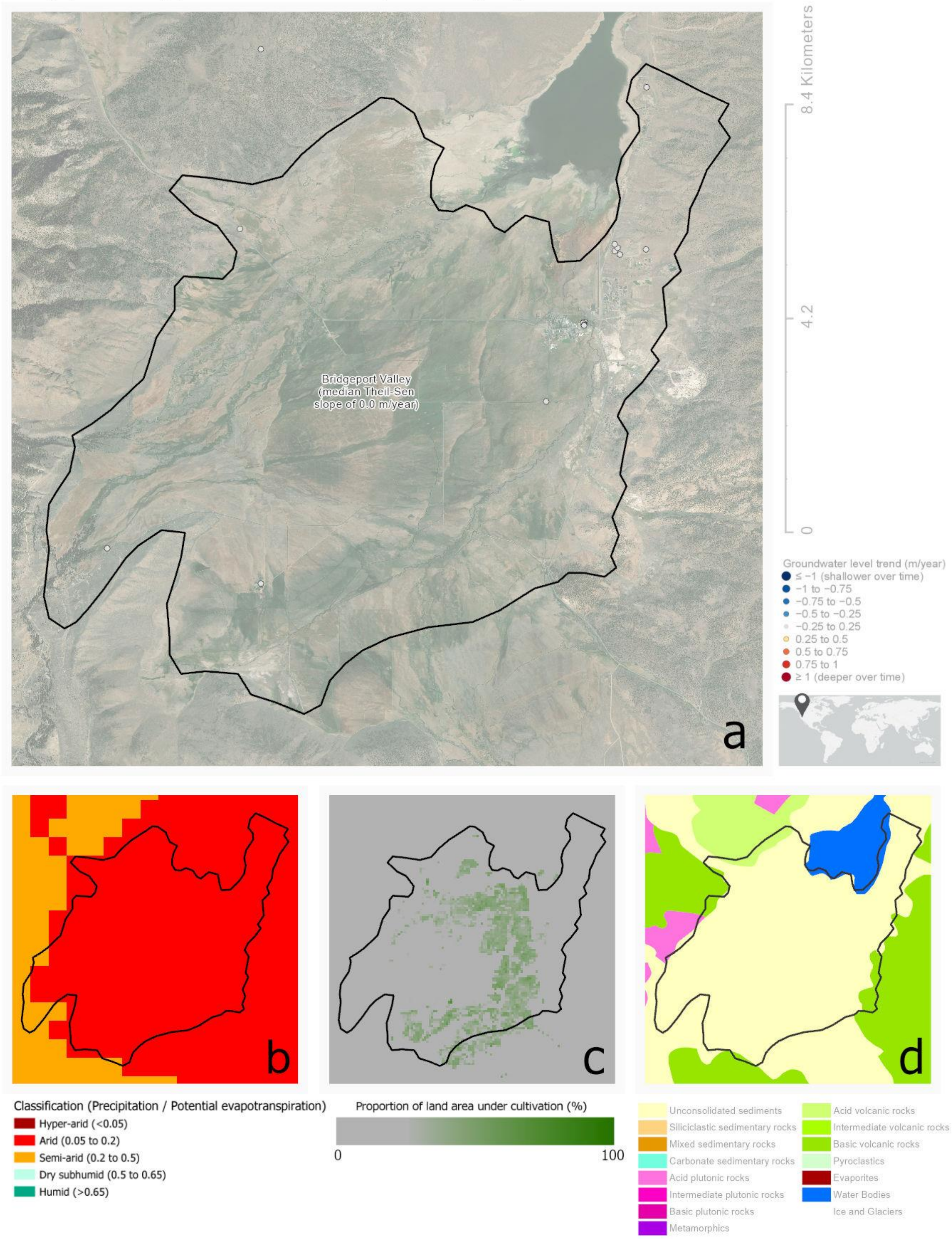

Supplementary Fig. 71. Spatial variability of trends in depth to groundwater Bridgeport Valley. For detailed description of the contents of each panel see the text at the beginning of Supplementary Note 17.

Bundaberg Aquifer (median Theil-Sen slope of 0 m/year)

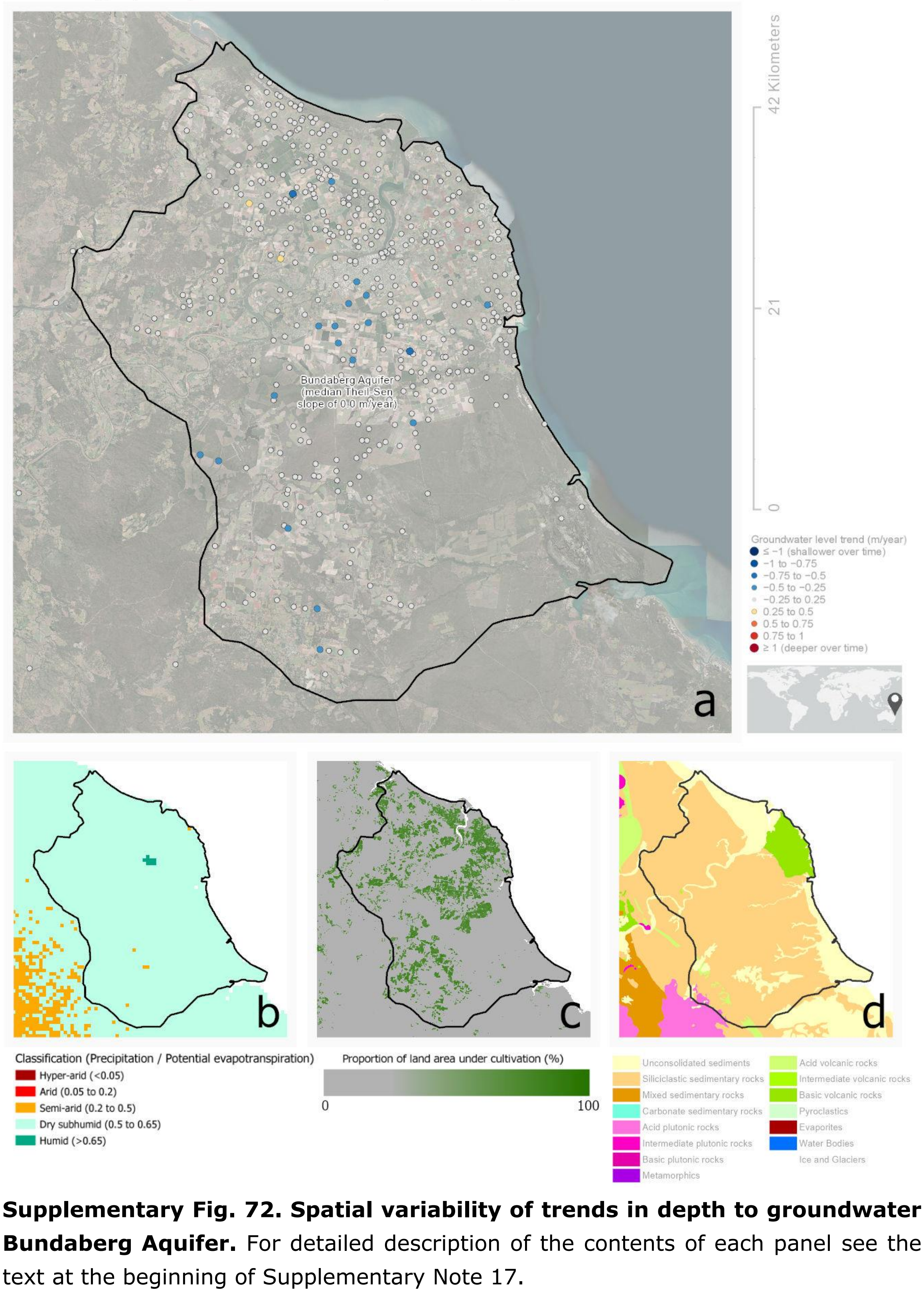

Supplementary Fig. 72. Spatial variability of trends in depth to groundwater Bundaberg Aquifer. For detailed description of the contents of each panel see the text at the beginning of Supplementary Note 17.

Burdekin Delta (median Theil-Sen slope of -0.1 m/year)

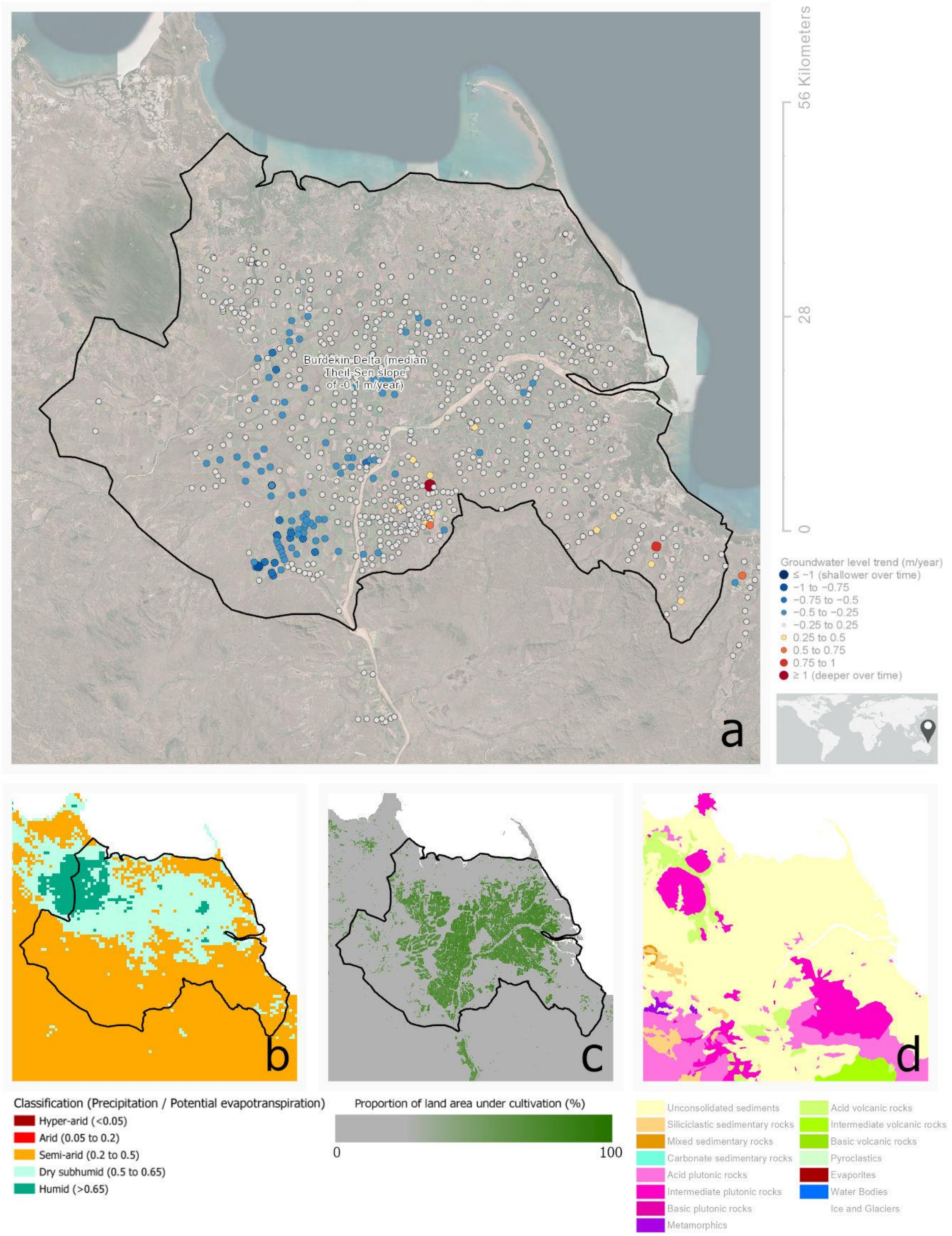

Supplementary Fig. 73. Spatial variability of trends in depth to groundwater Burdekin Delta. For detailed description of the contents of each panel see the text at the beginning of Supplementary Note 17.

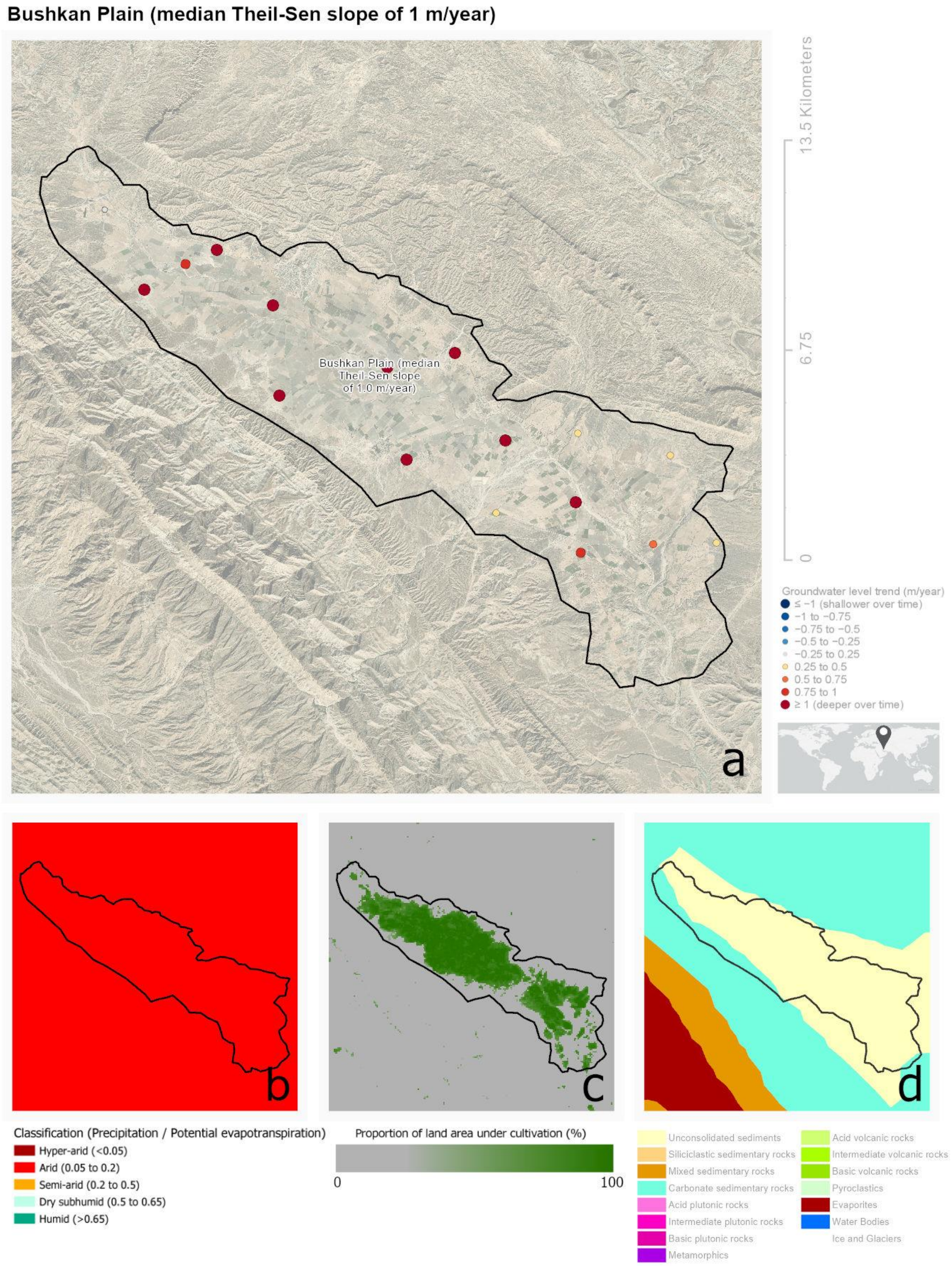

Supplementary Fig. 74. Spatial variability of trends in depth to groundwater Bushkan Plain. For detailed description of the contents of each panel see the text at the beginning of Supplementary Note 17.

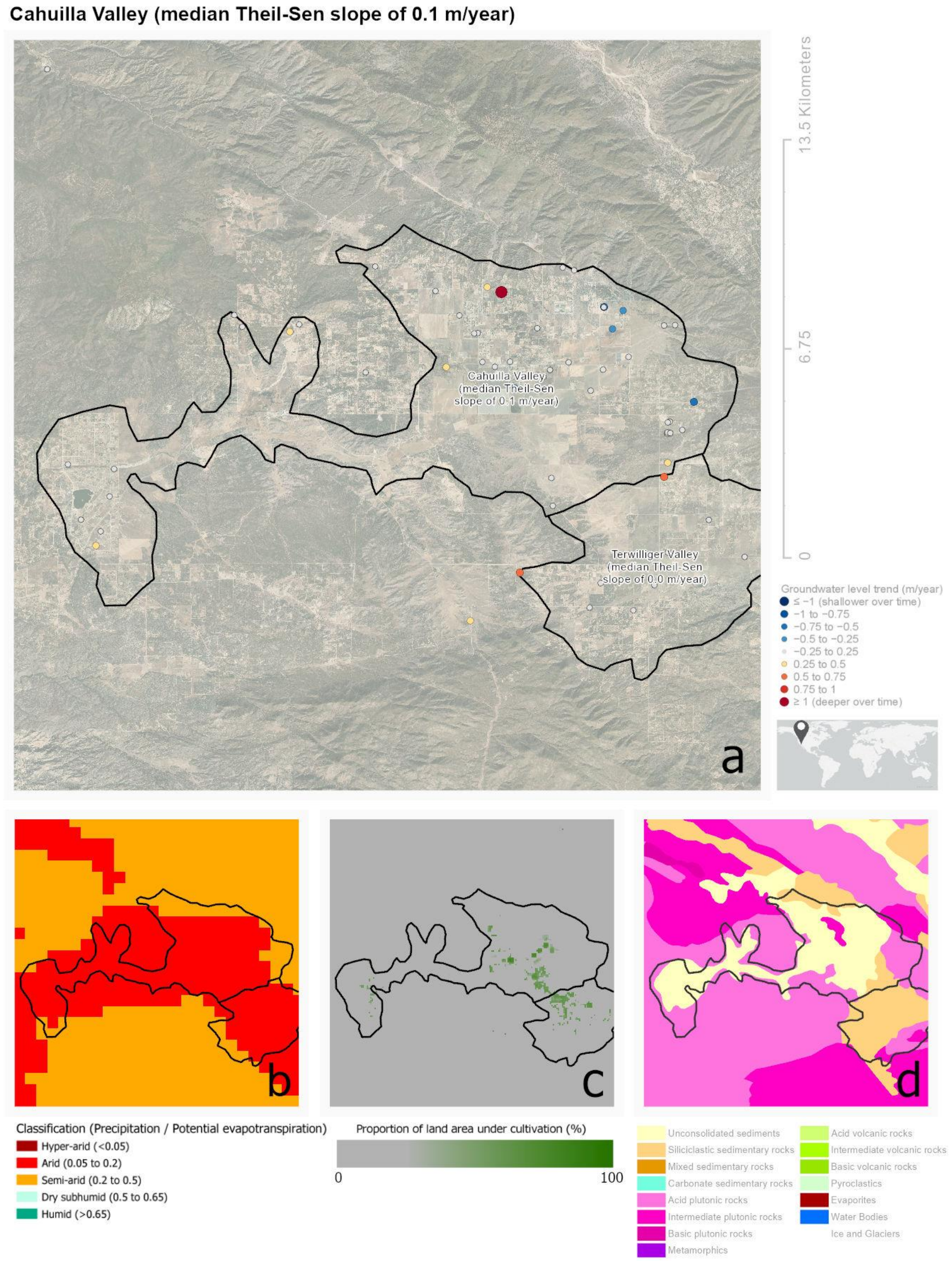

Supplementary Fig. 75. Spatial variability of trends in depth to groundwater Cahuilla Valley. For detailed description of the contents of each panel see the text at the beginning of Supplementary Note 17.

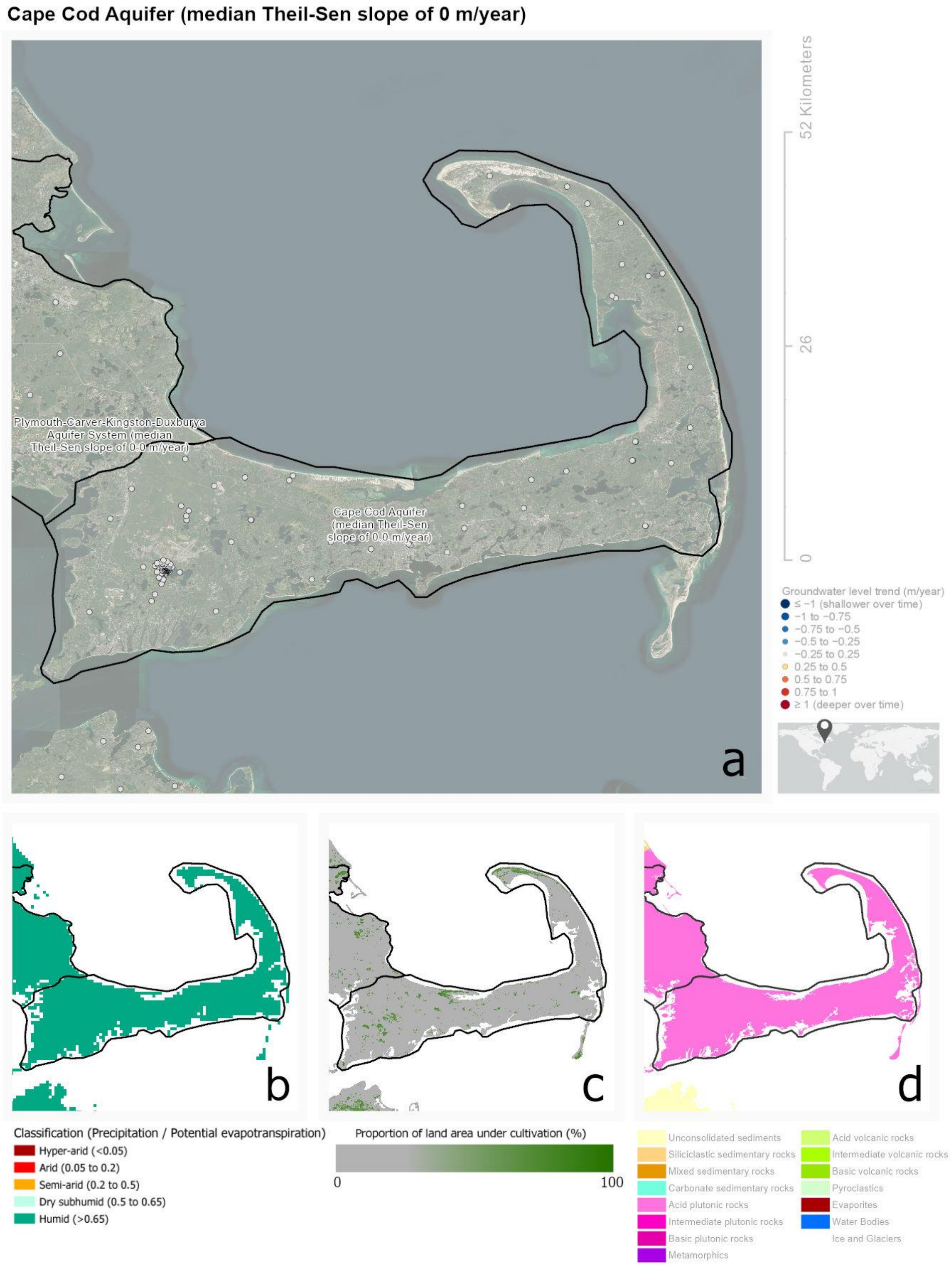

Supplementary Fig. 76. Spatial variability of trends in depth to groundwater Cape Cod Aquifer. For detailed description of the contents of each panel see the text at the beginning of Supplementary Note 17.

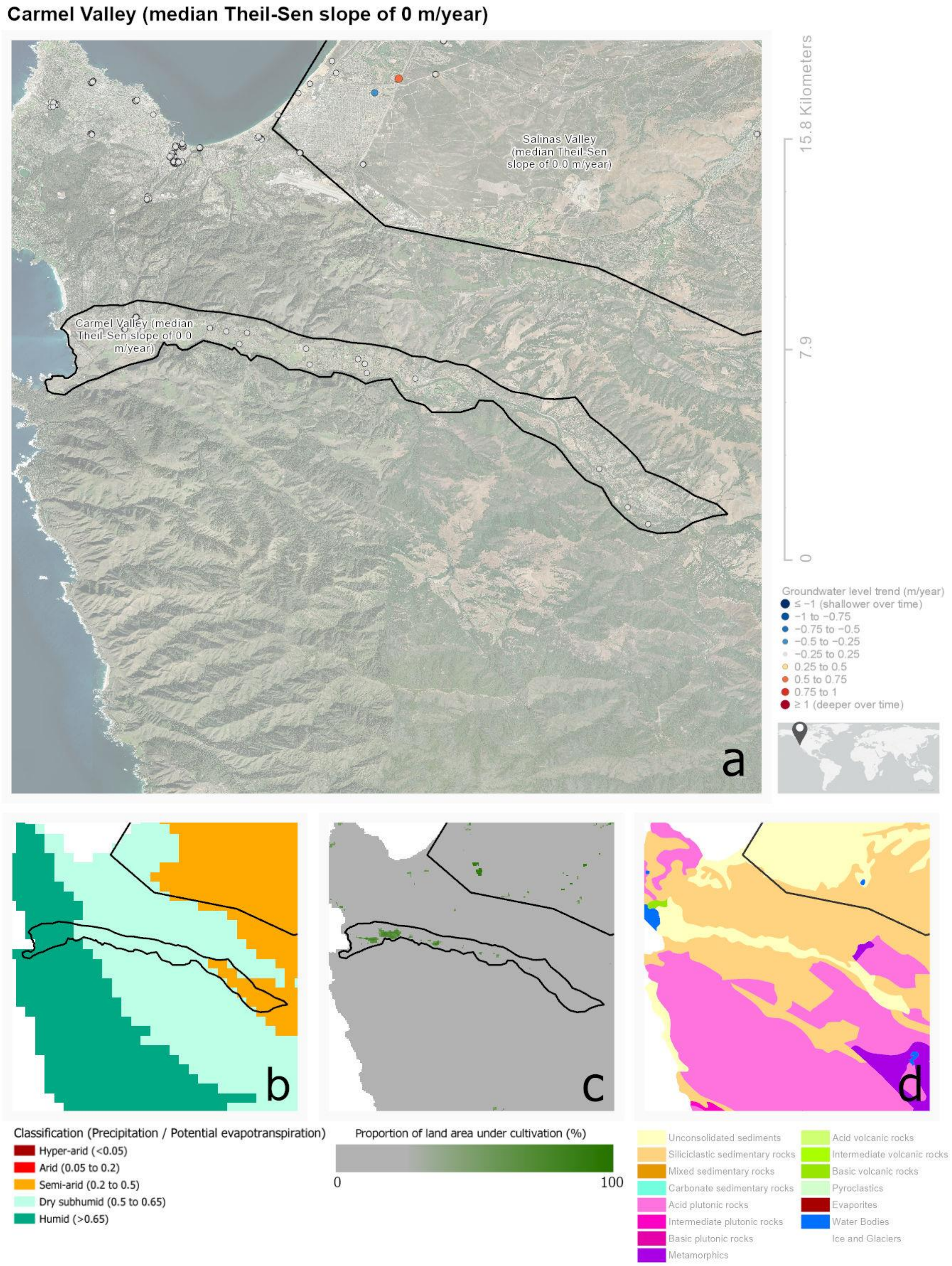

Supplementary Fig. 77. Spatial variability of trends in depth to groundwater Carmel Valley. For detailed description of the contents of each panel see the text at the beginning of Supplementary Note 17.

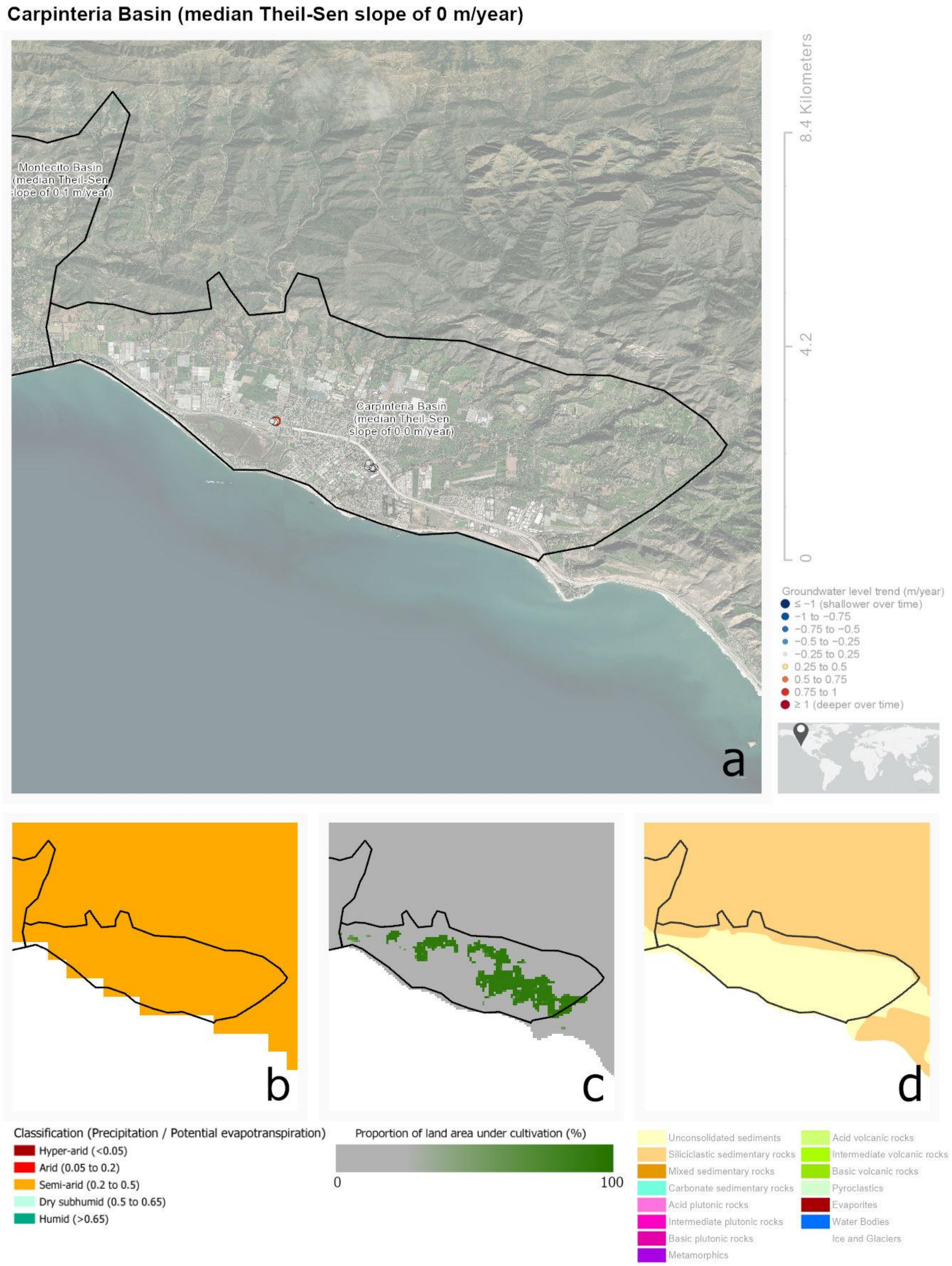

Supplementary Fig. 78. Spatial variability of trends in depth to groundwater Carpinteria Basin. For detailed description of the contents of each panel see the text at the beginning of Supplementary Note 17.

Central Adelaide Plains (median Theil-Sen slope of 0 m/year)

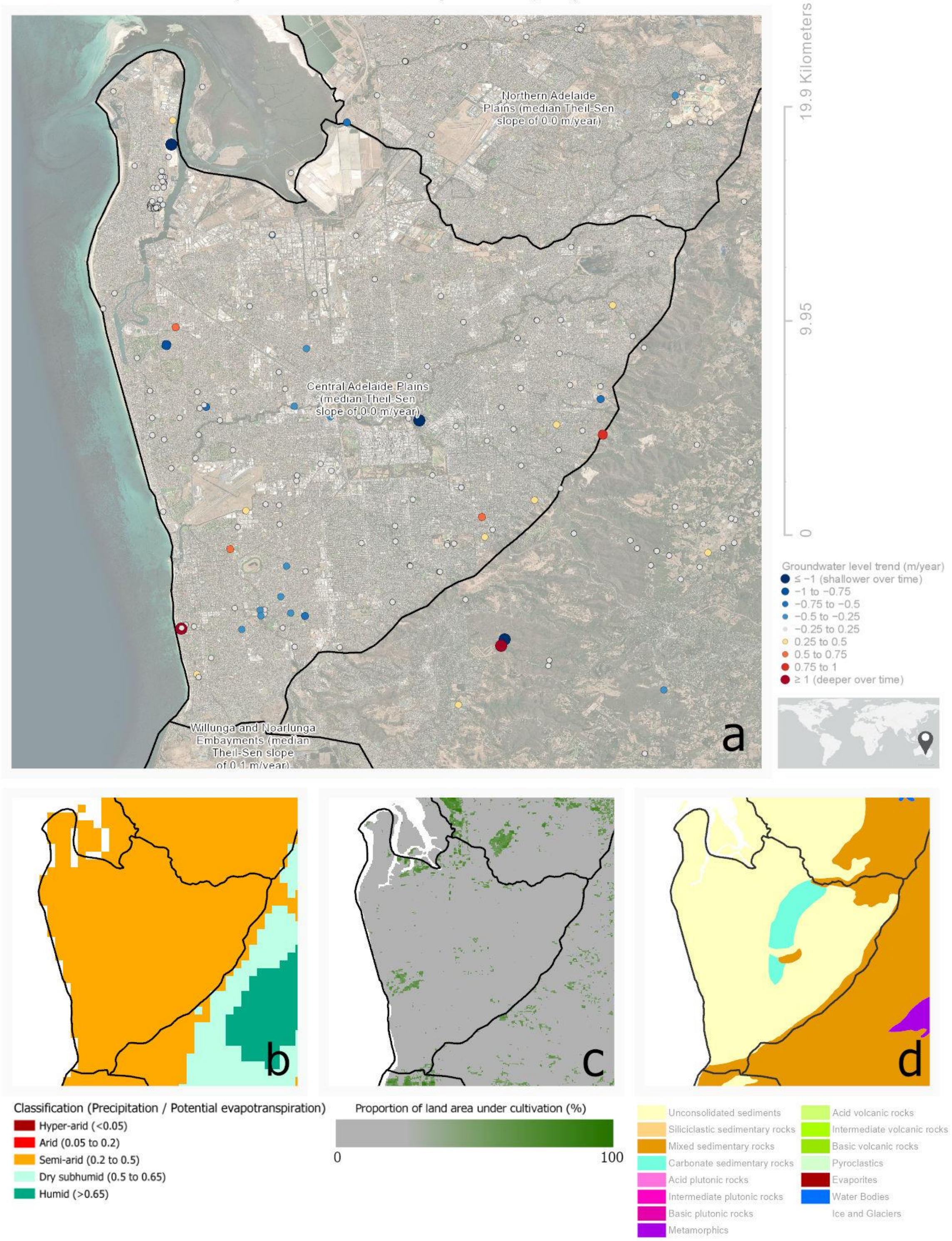

Supplementary Fig. 79. Spatial variability of trends in depth to groundwater Central Adelaide Plains (within the broader St Vincent Basin). For detailed description of the contents of each panel see the text at the beginning of Supplementary Note 17.

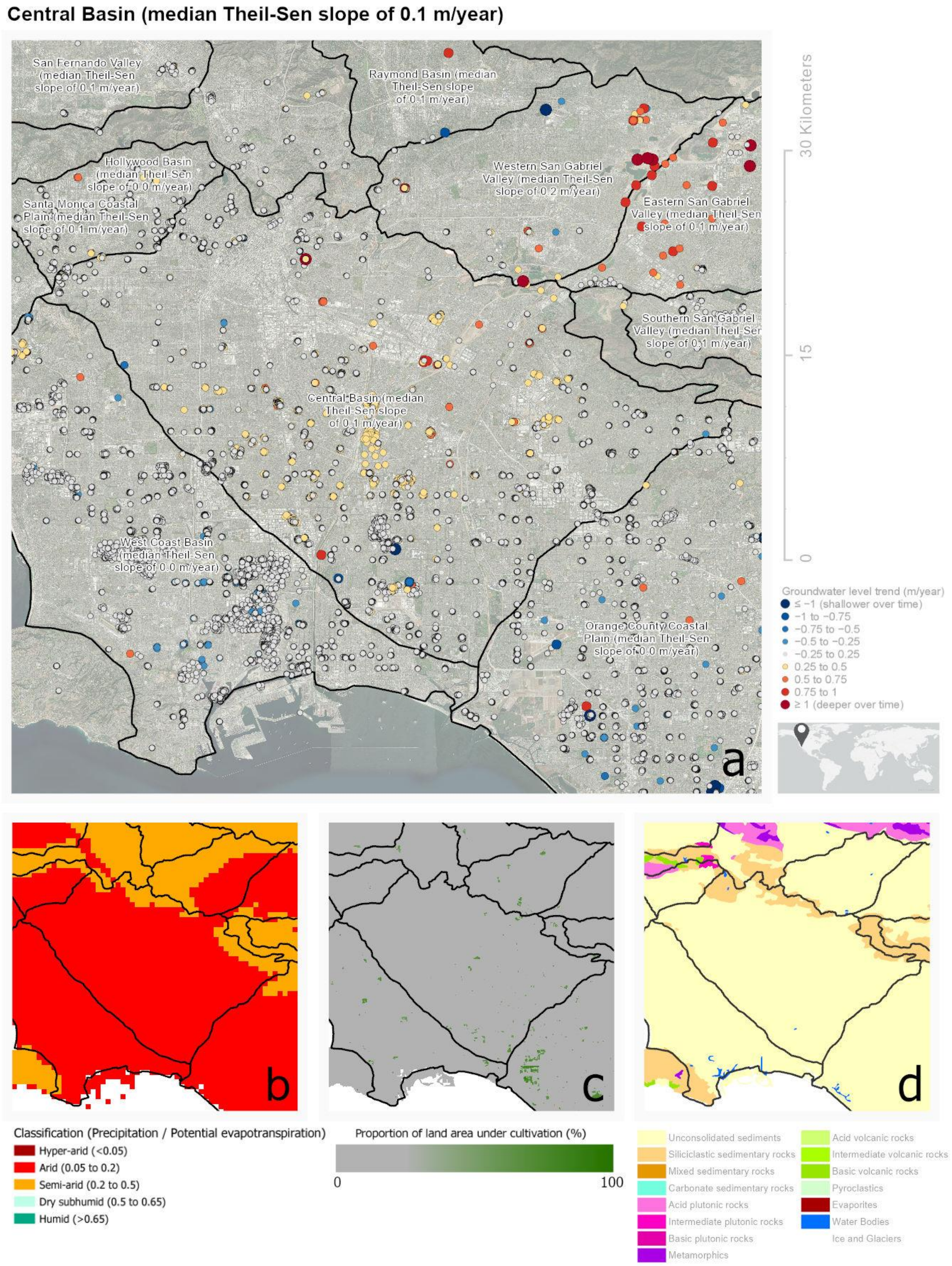

Supplementary Fig. 80. Spatial variability of trends in depth to groundwater Central Basin (within the broader Los Angeles Basin). For detailed description of the contents of each panel see the text at the beginning of Supplementary Note 17.

Central Colusa Basin (median Theil-Sen slope of 0.1 m/year)

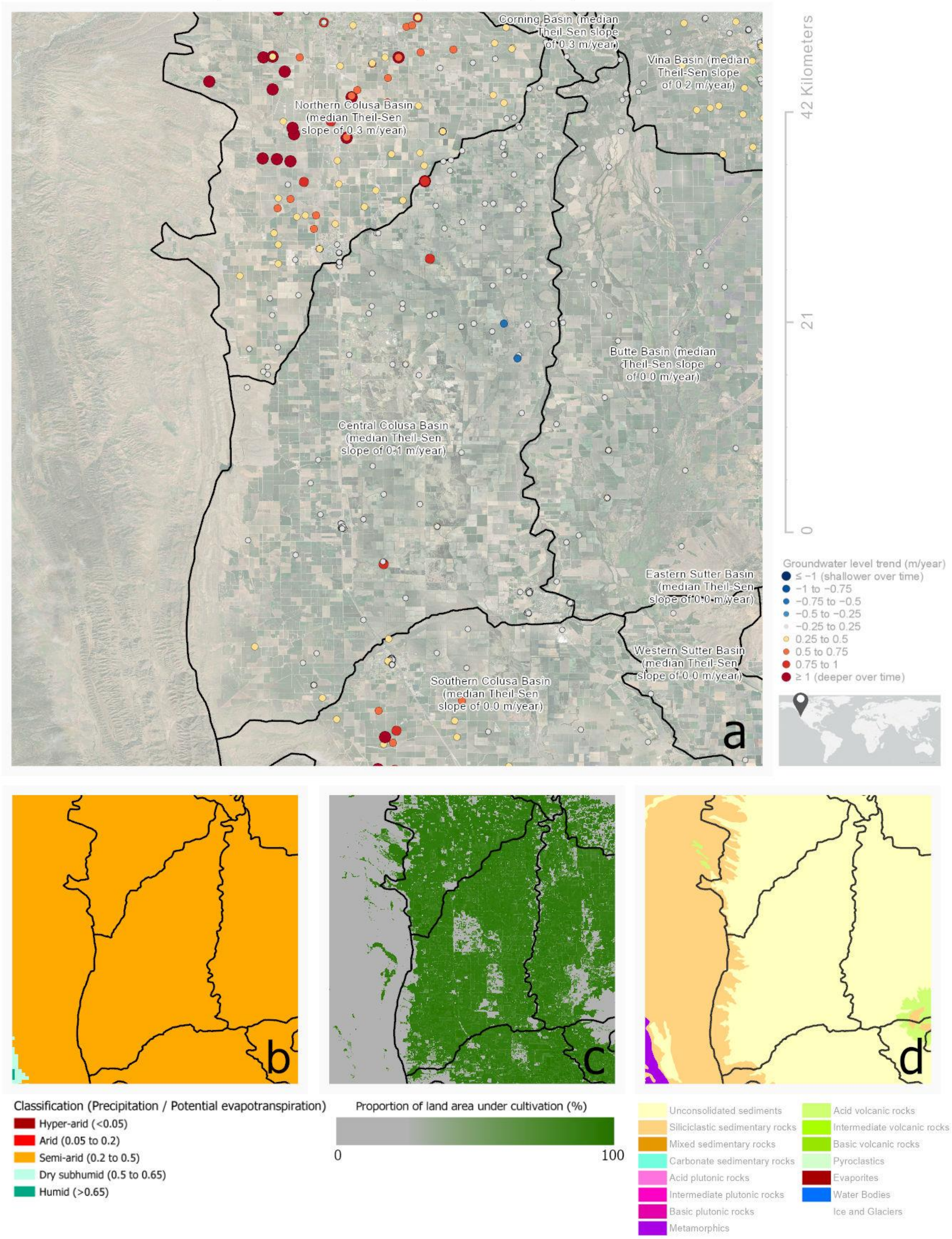

Supplementary Fig. 81. Spatial variability of trends in depth to groundwater Central Colusa Basin (within the broader Sacramento Valley of the Central Valley). For detailed description of the contents of each panel see the text at the beginning of Supplementary Note 17.

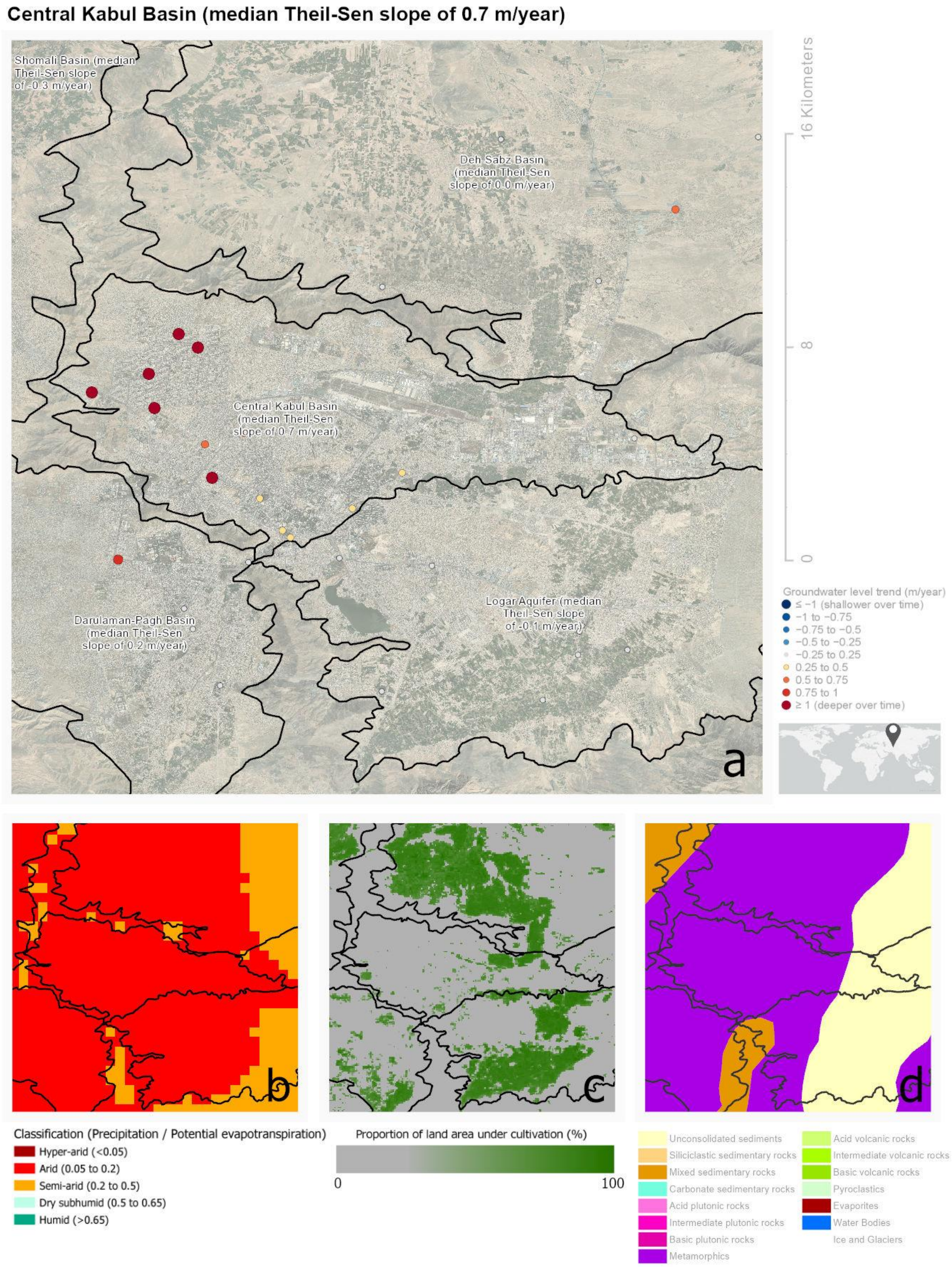

Supplementary Fig. 82. Spatial variability of trends in depth to groundwater Central Kabul Basin (within the broader Kabul Basin). For detailed description of the contents of each panel see the text at the beginning of Supplementary Note 17.

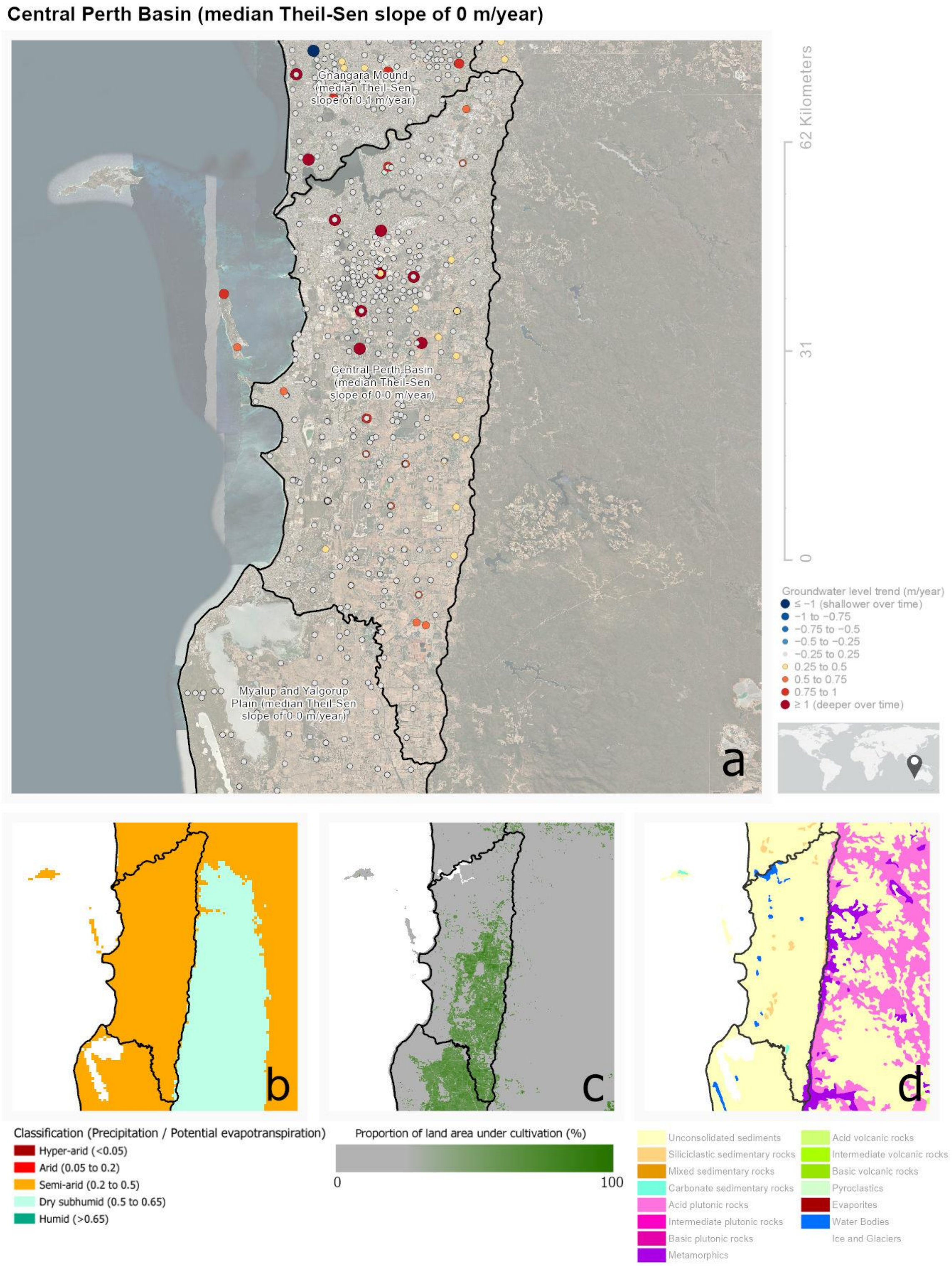

Supplementary Fig. 83. Spatial variability of trends in depth to groundwater Central Perth Basin (within the broader Perth Basin). For detailed description of the contents of each panel see the text at the beginning of Supplementary Note 17.

Chino Basin (median Theil-Sen slope of 0.1 m/year)

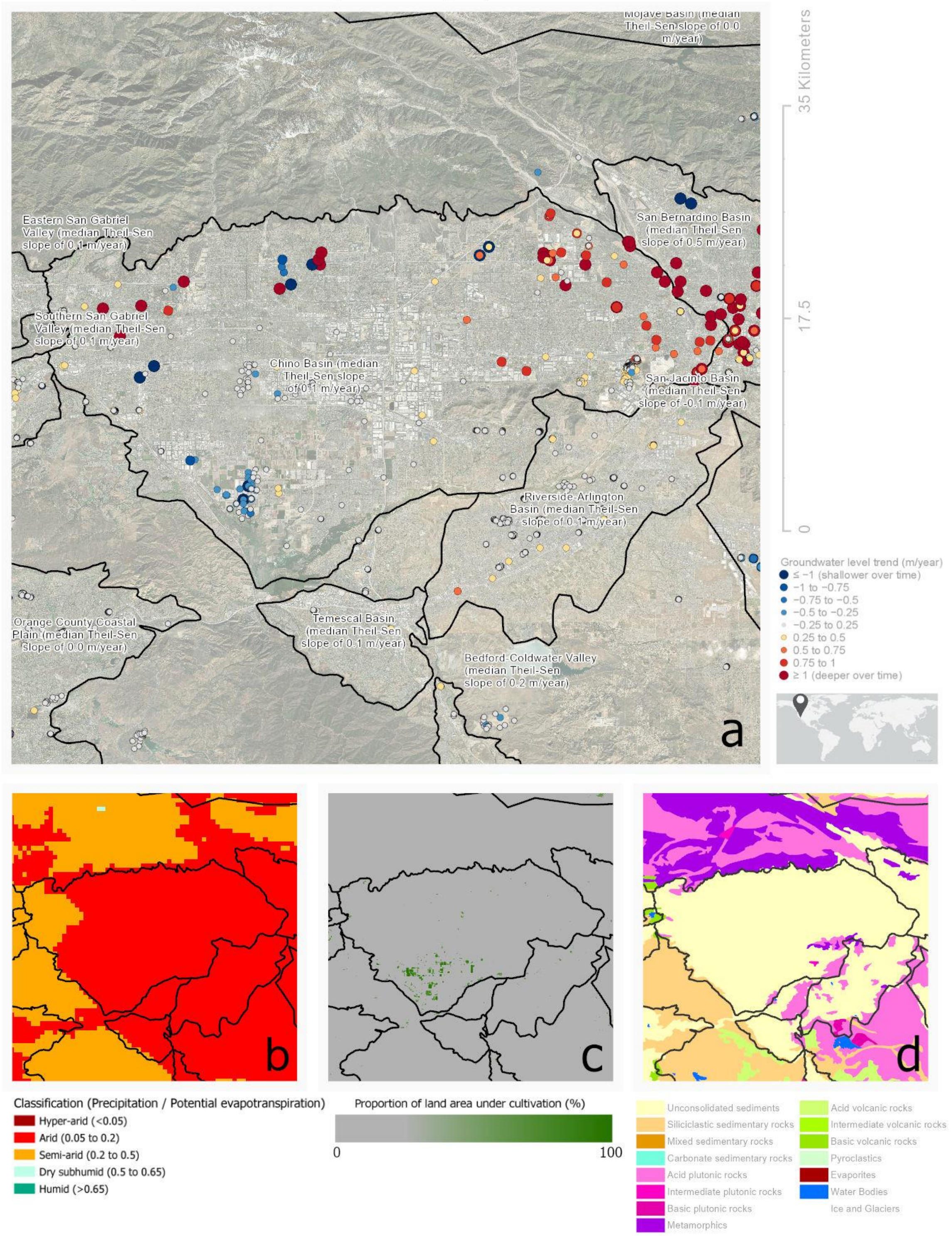

Supplementary Fig. 84. Spatial variability of trends in depth to groundwater Chino Basin (within the broader Upper Santa Ana Basin). For detailed description of the contents of each panel see the text at the beginning of Supplementary Note 17.

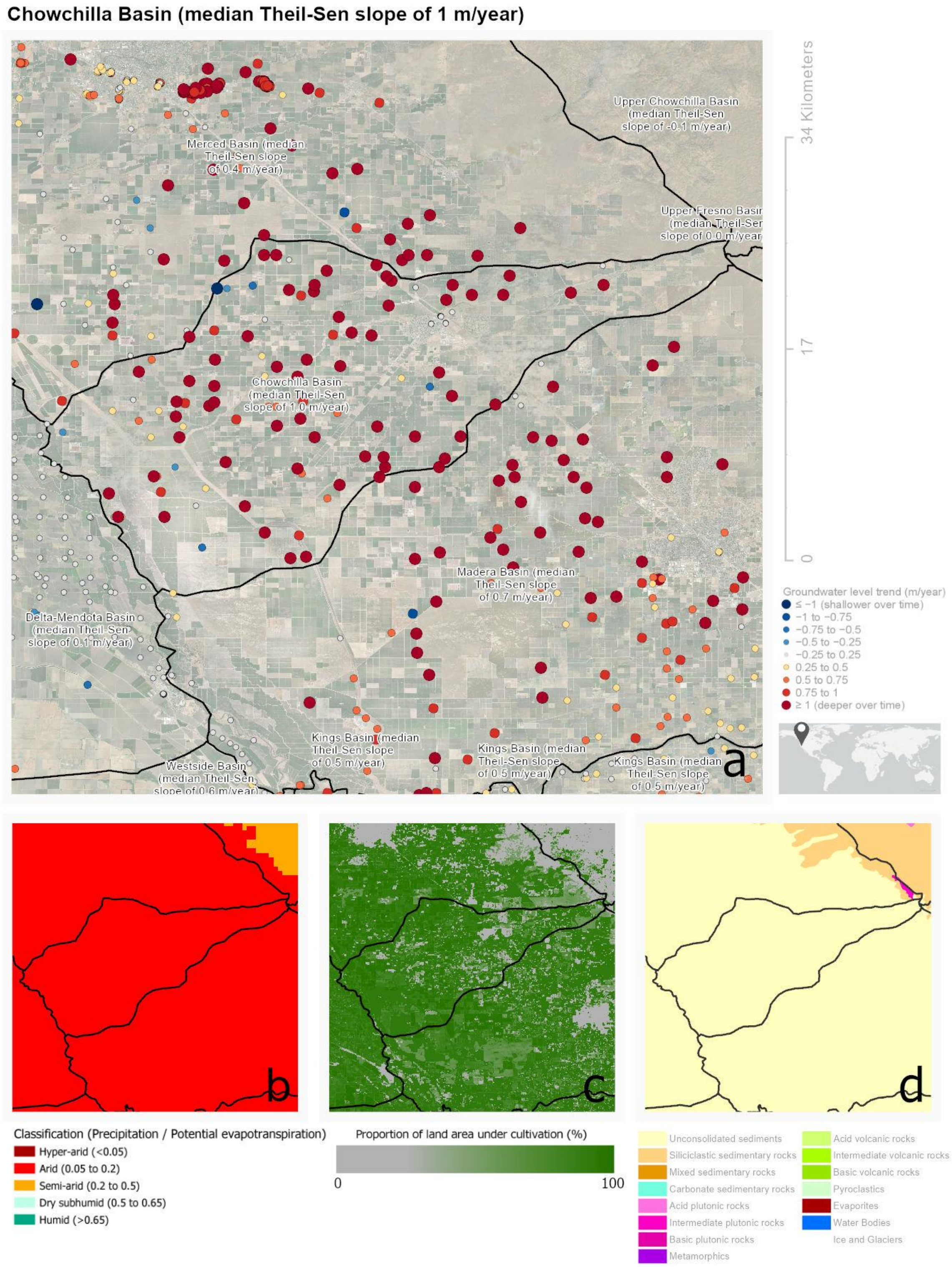

Supplementary Fig. 85. Spatial variability of trends in depth to groundwater Chowchilla Basin (within the broader San Joaquin Basin of the Central Valley). For detailed description of the contents of each panel see the text at the beginning of Supplementary Note 17.

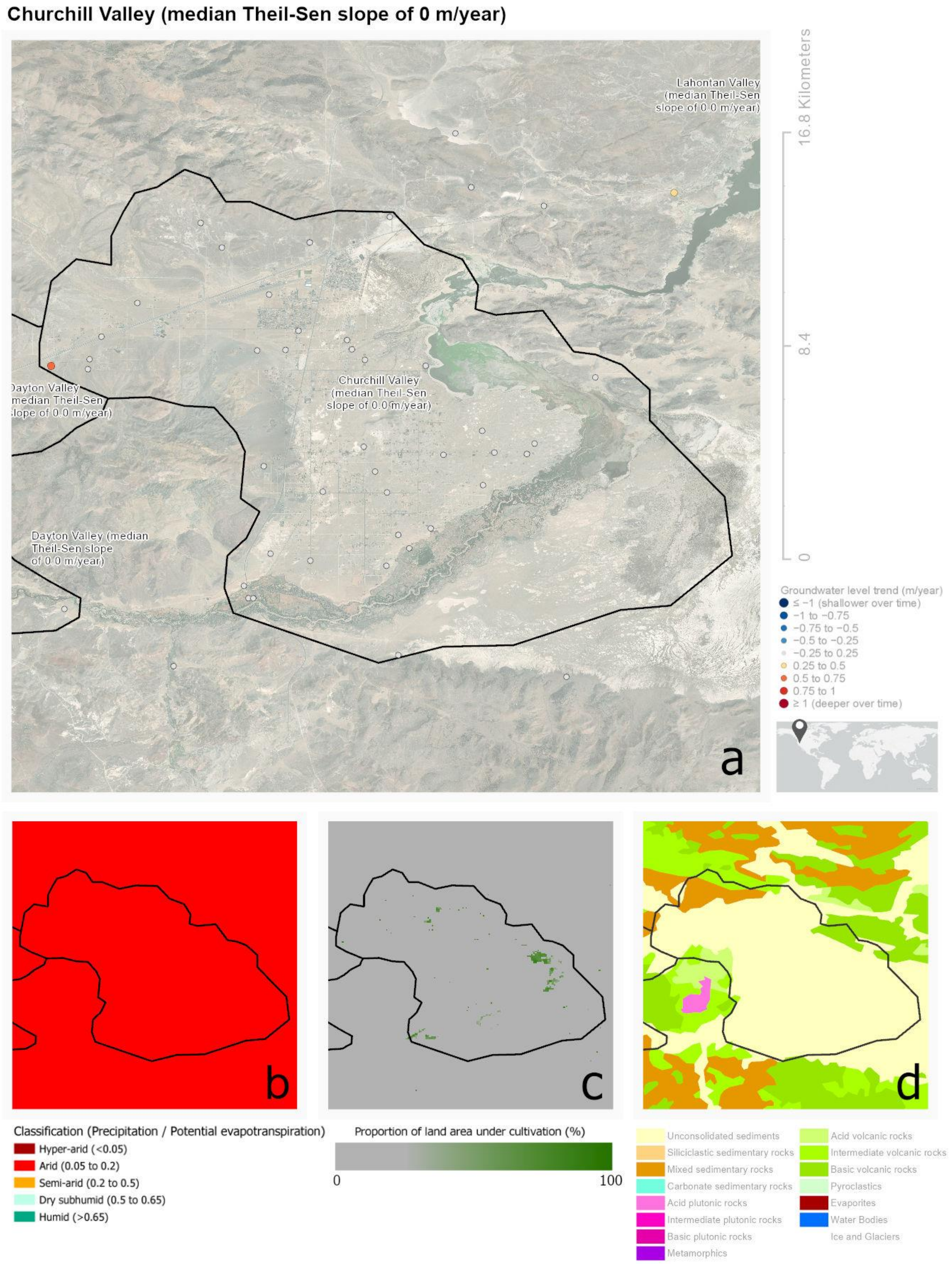

Supplementary Fig. 86. Spatial variability of trends in depth to groundwater Churchill Valley (within the broader Carson River Basin). For detailed description of the contents of each panel see the text at the beginning of Supplementary Note 17.

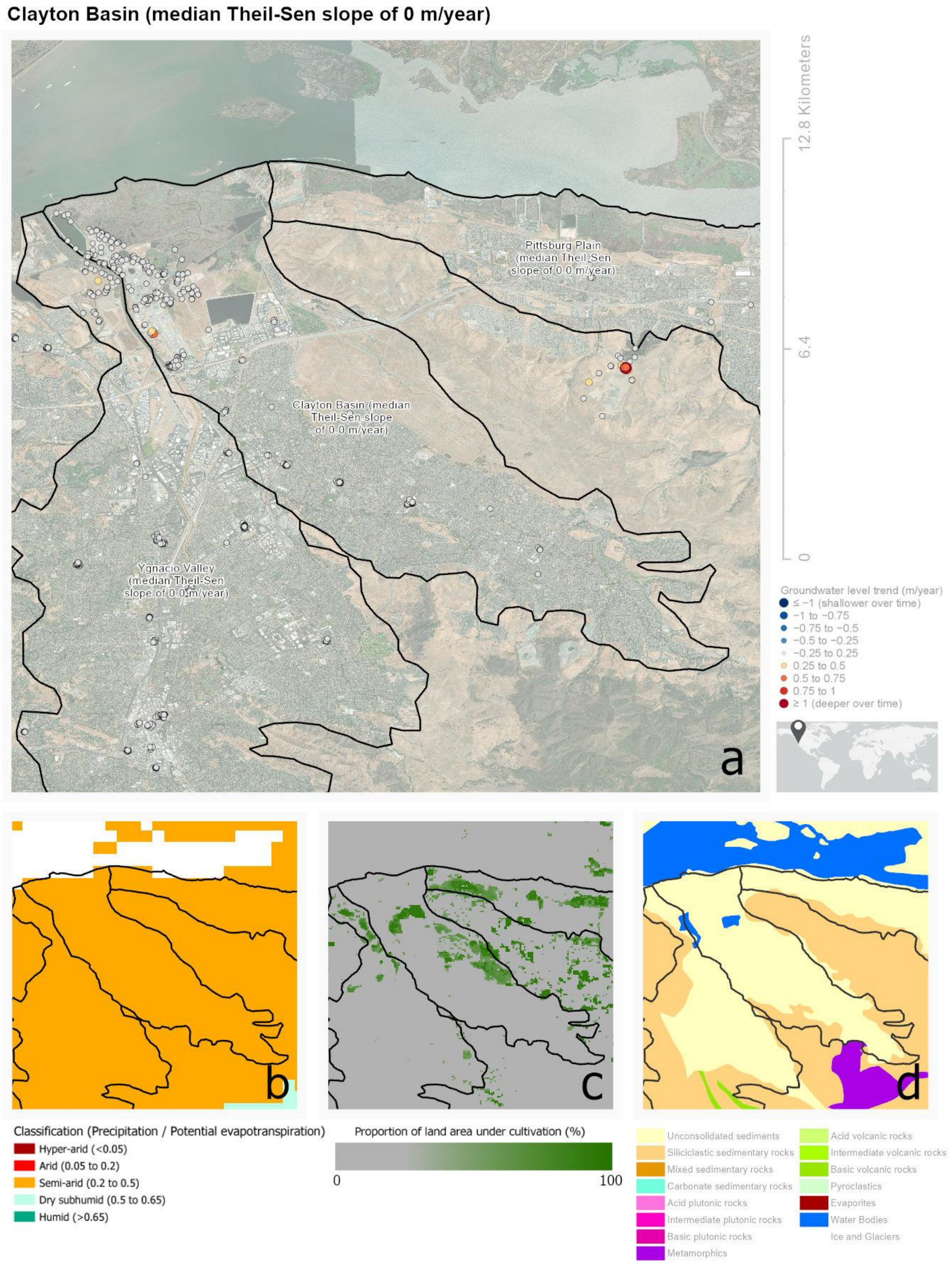

Supplementary Fig. 87. Spatial variability of trends in depth to groundwater Clayton Basin. For detailed description of the contents of each panel see the text at the beginning of Supplementary Note 17.

Coachella Valley (median Theil-Sen slope of 0 m/year)

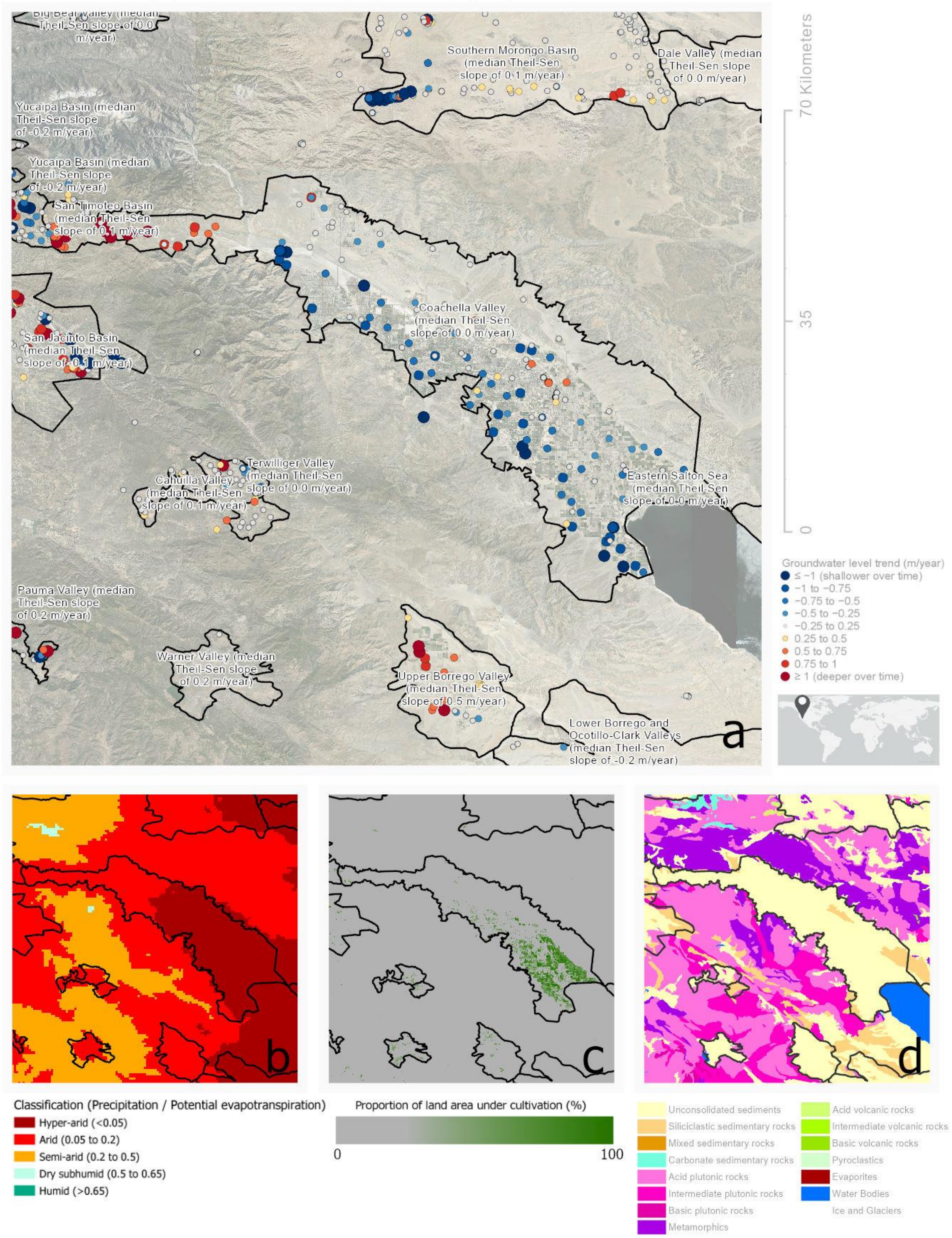

Supplementary Fig. 88. Spatial variability of trends in depth to groundwater Coachella Valley. For detailed description of the contents of each panel see the text at the beginning of Supplementary Note 17.

Conejo Basin (median Theil-Sen slope of 0 m/year)

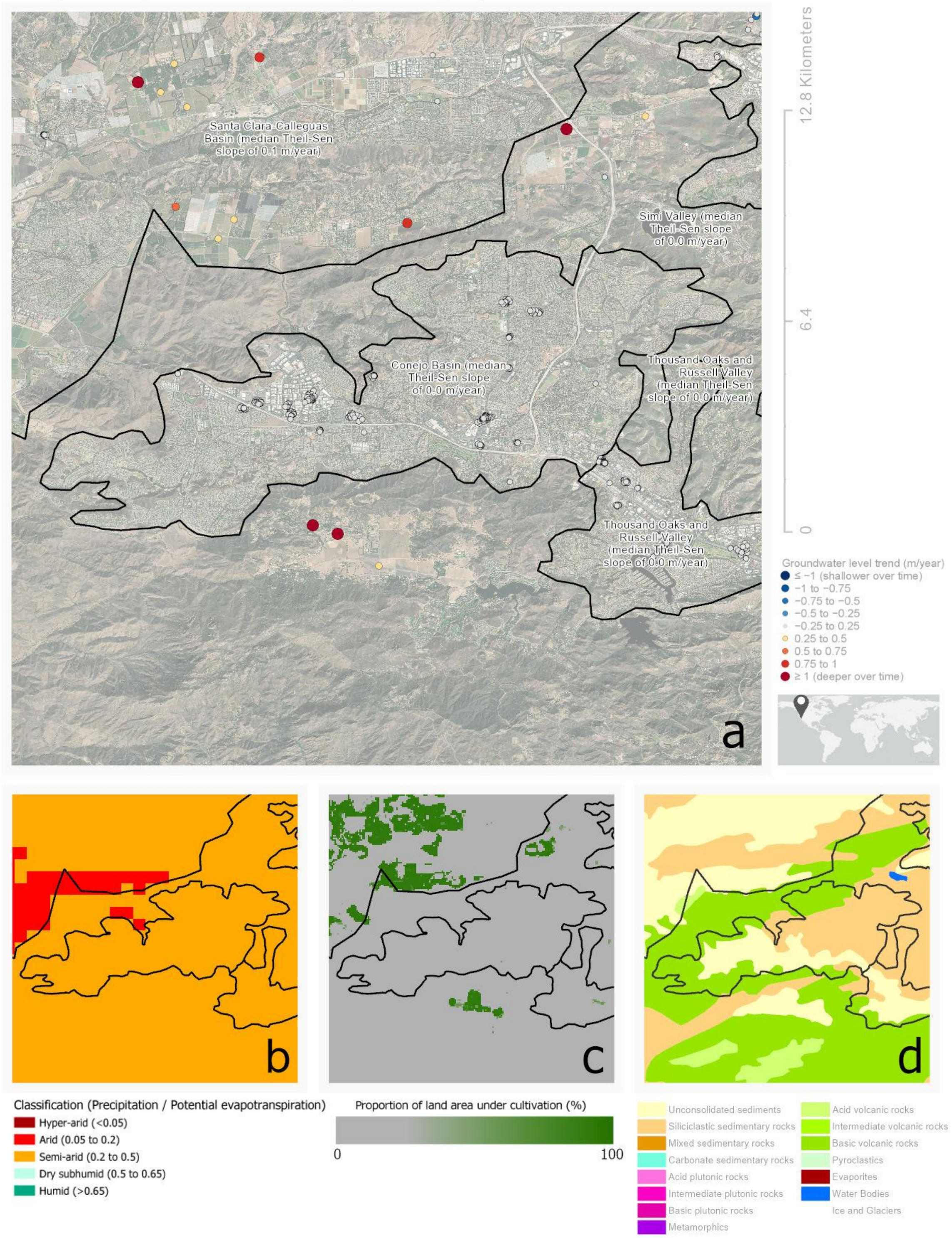

Supplementary Fig. 89. Spatial variability of trends in depth to groundwater Conejo Basin. For detailed description of the contents of each panel see the text at the beginning of Supplementary Note 17.

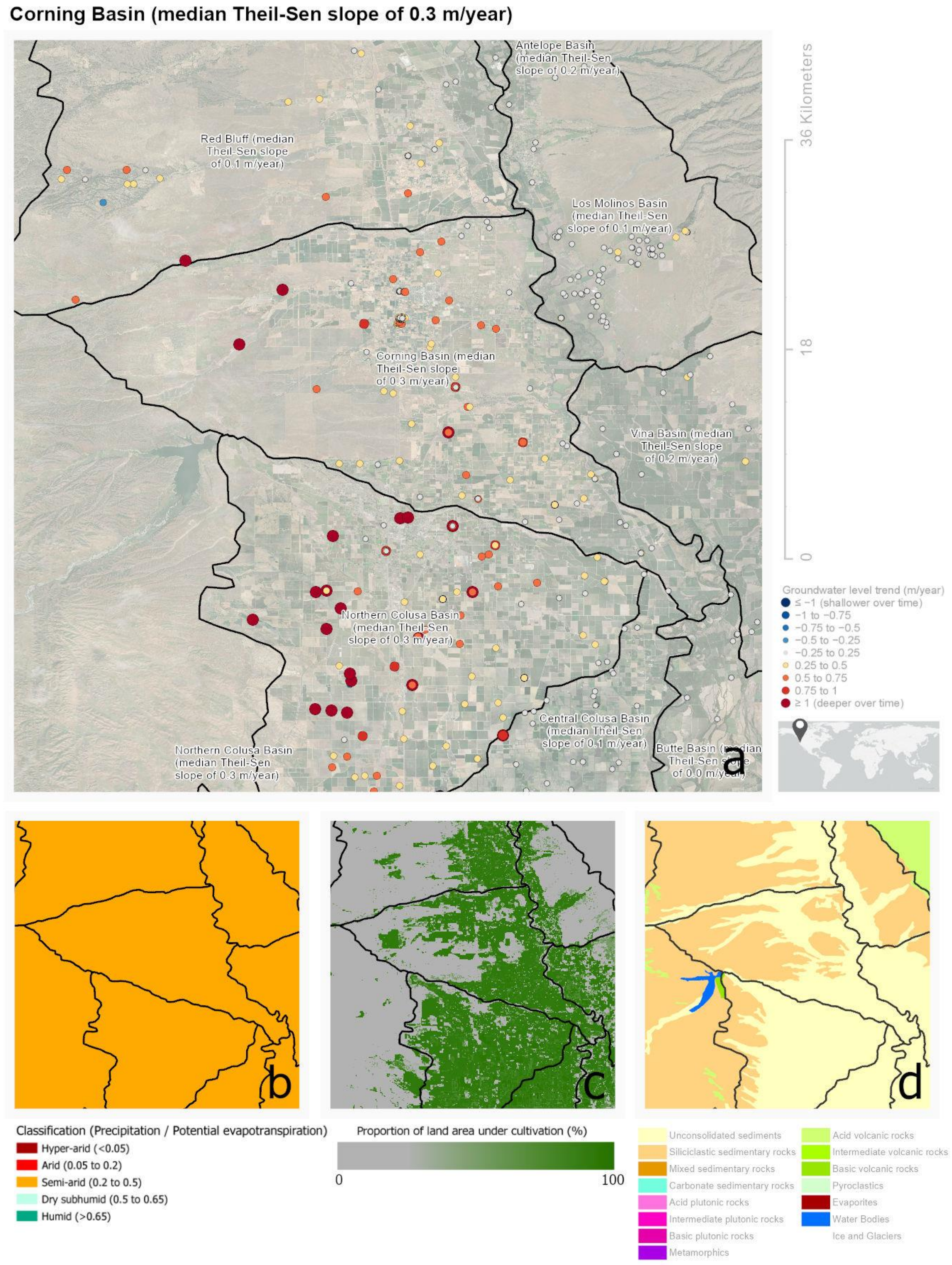

Supplementary Fig. 90. Spatial variability of trends in depth to groundwater Corning Basin (within the broader Sacramento Valley of the Central Valley). For detailed description of the contents of each panel see the text at the beginning of Supplementary Note 17.

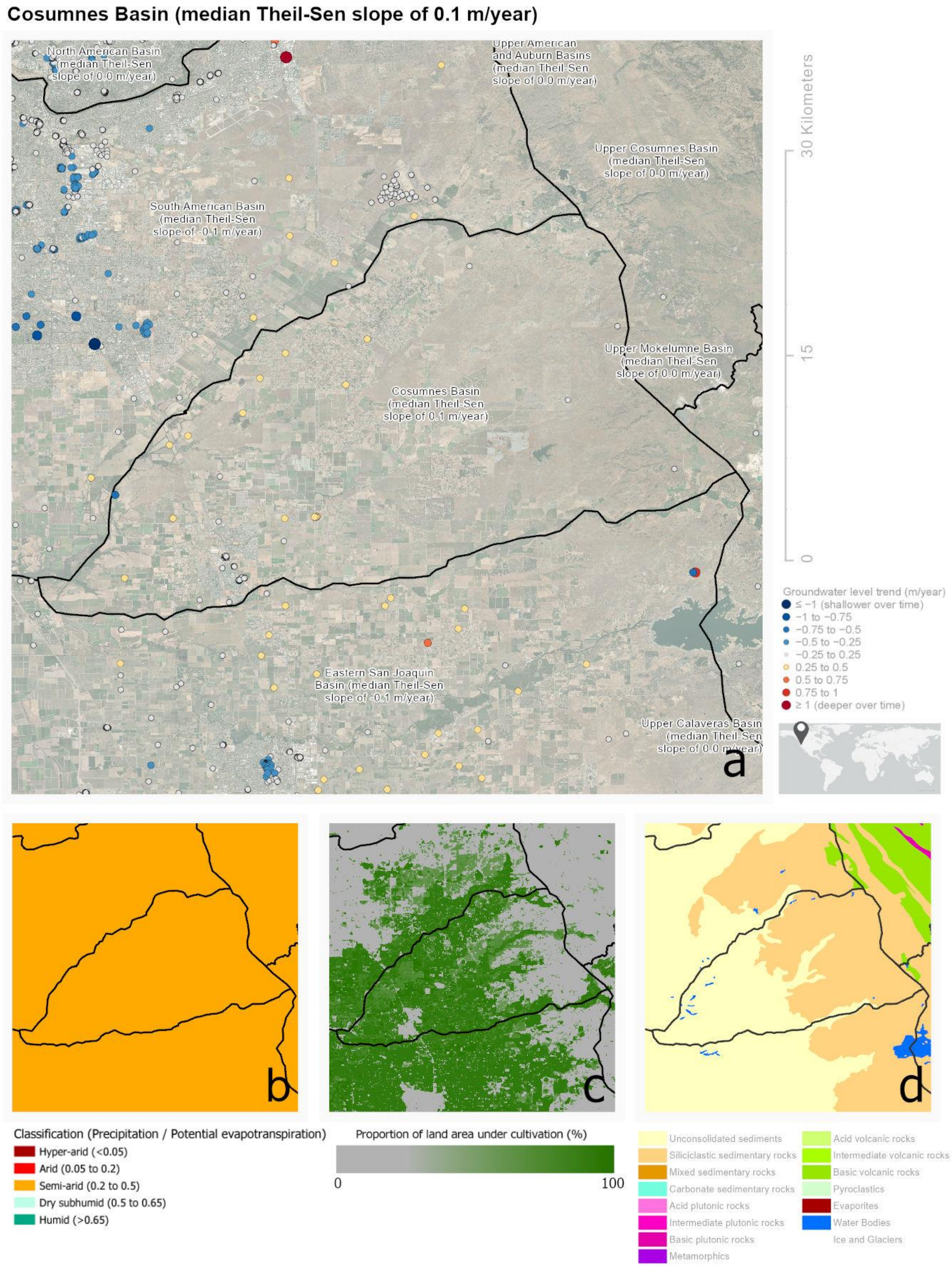

Supplementary Fig. 91. Spatial variability of trends in depth to groundwater Cosumnes Basin (within the broader San Joaquin Basin of the Central Valley). For detailed description of the contents of each panel see the text at the beginning of Supplementary Note 17.

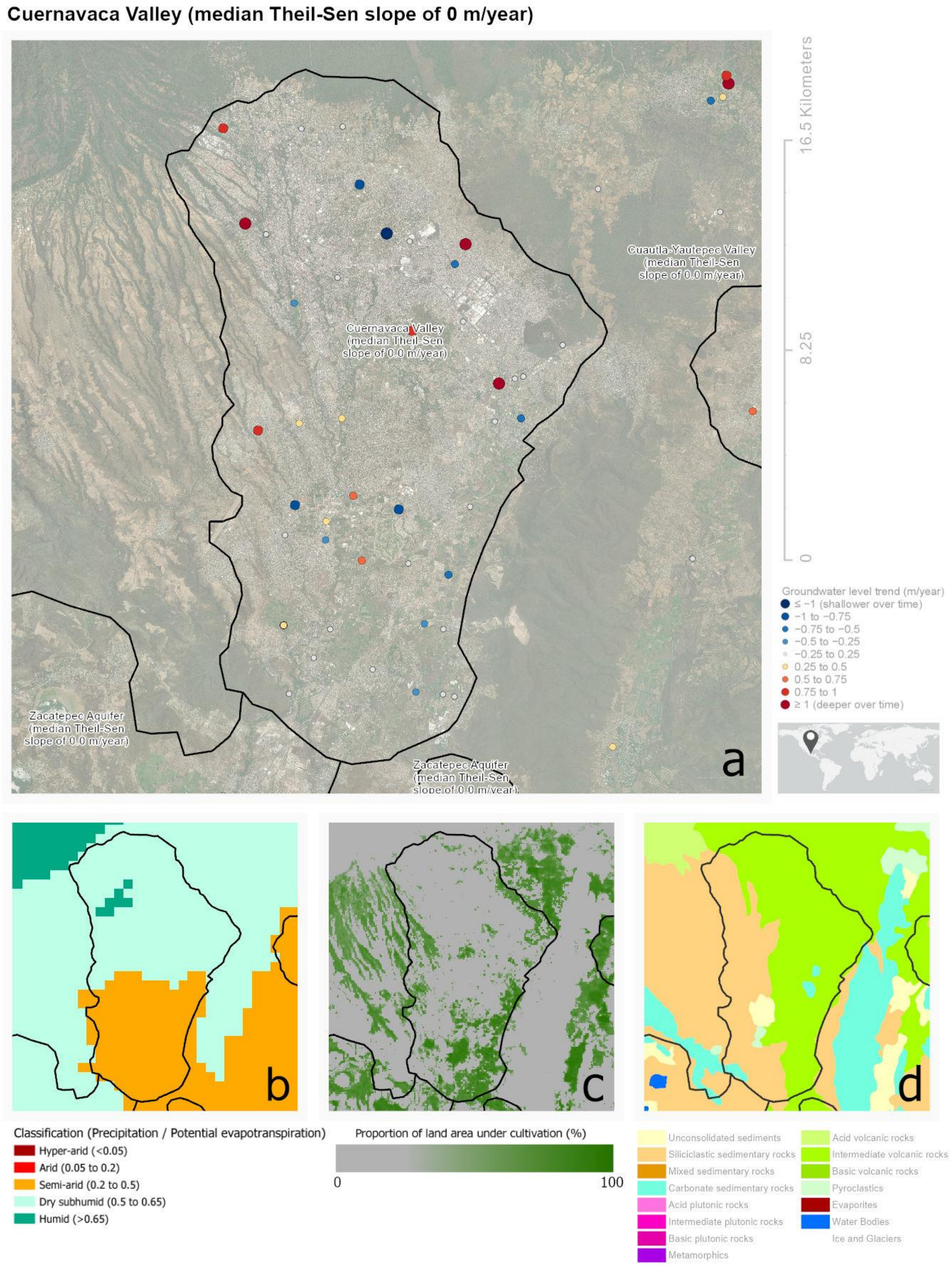

Supplementary Fig. 92. Spatial variability of trends in depth to groundwater Cuernavaca Valley. For detailed description of the contents of each panel see the text at the beginning of Supplementary Note 17.

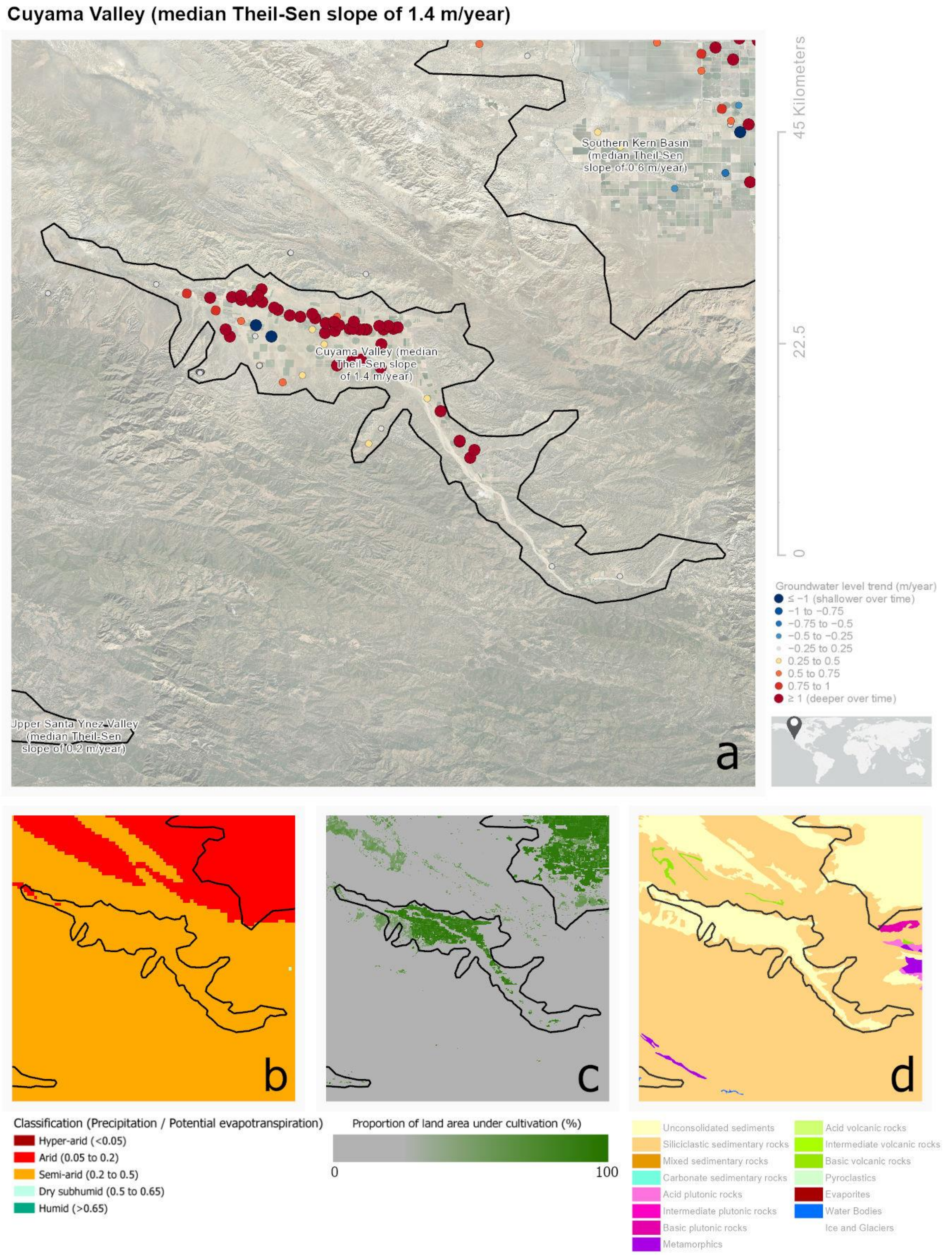

Supplementary Fig. 93. Spatial variability of trends in depth to groundwater Cuyama Valley. For detailed description of the contents of each panel see the text at the beginning of Supplementary Note 17.

Darolmizan Basin (median Theil-Sen slope of 0.1 m/year)

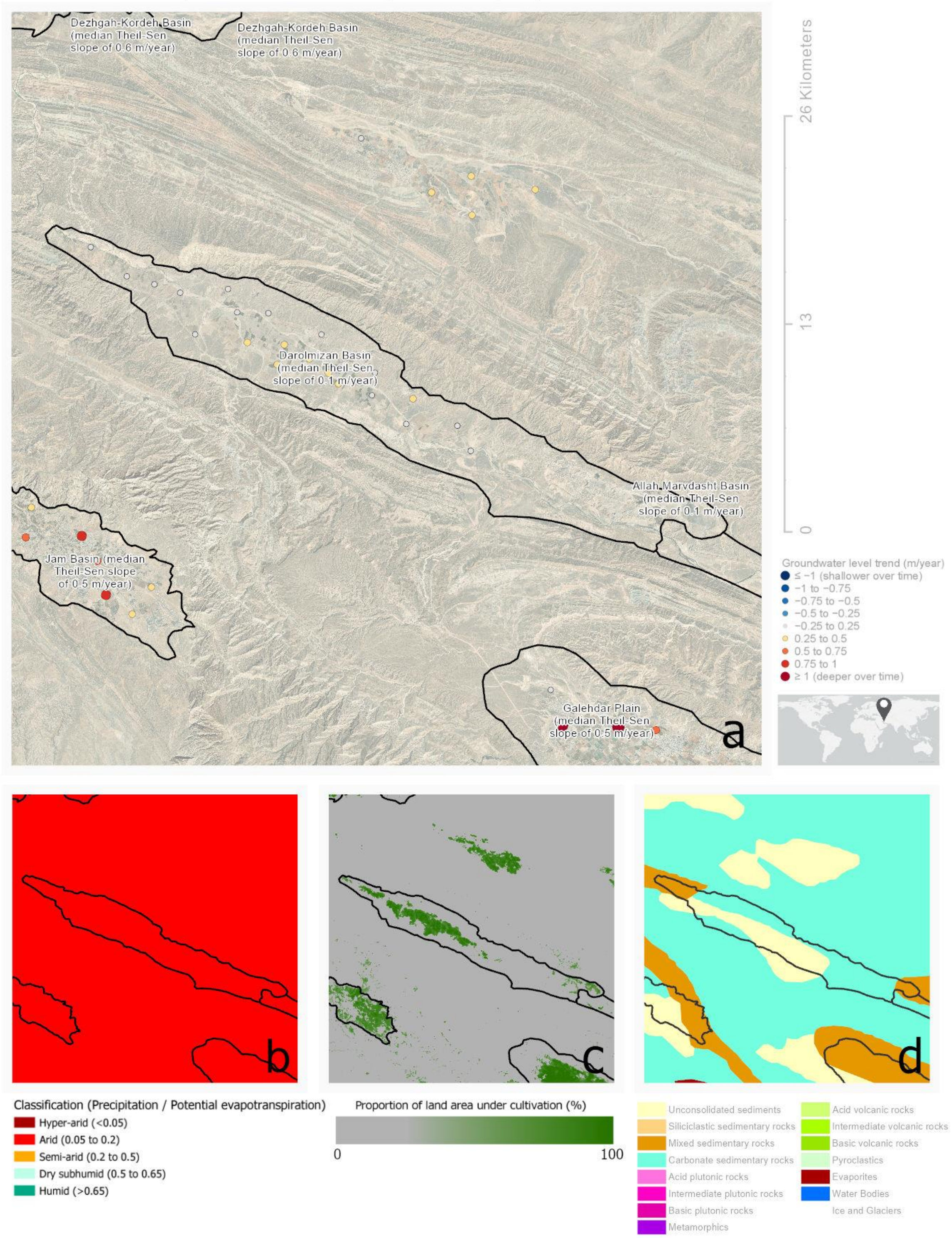

Supplementary Fig. 94. Spatial variability of trends in depth to groundwater Darolmizan Basin. For detailed description of the contents of each panel see the text at the beginning of Supplementary Note 17.

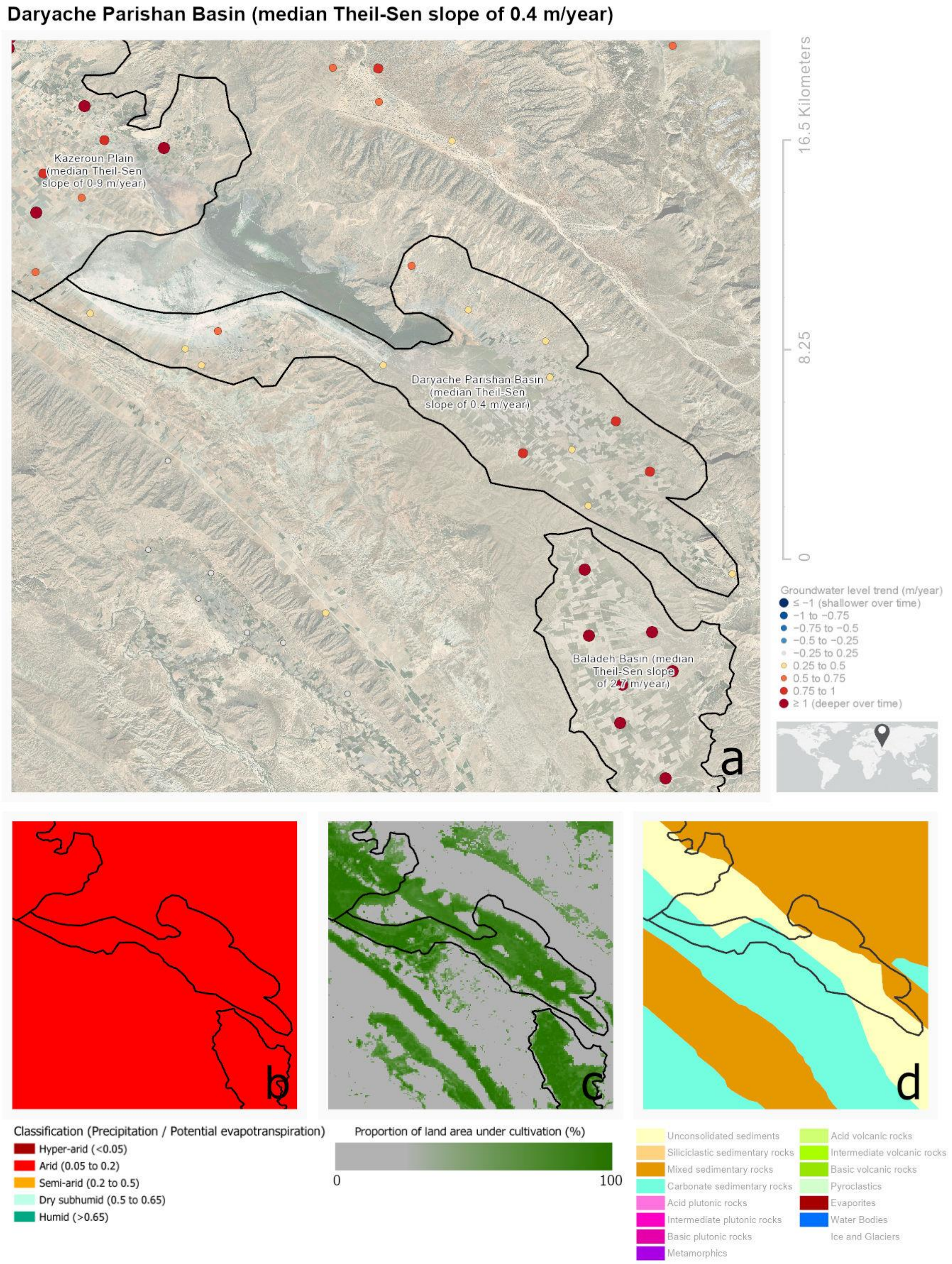

Supplementary Fig. 95. Spatial variability of trends in depth to groundwater Daryache Parishan Basin. For detailed description of the contents of each panel see the text at the beginning of Supplementary Note 17.

Dasht e- Afzar Basin (median Theil-Sen slope of 0.2 m/year)

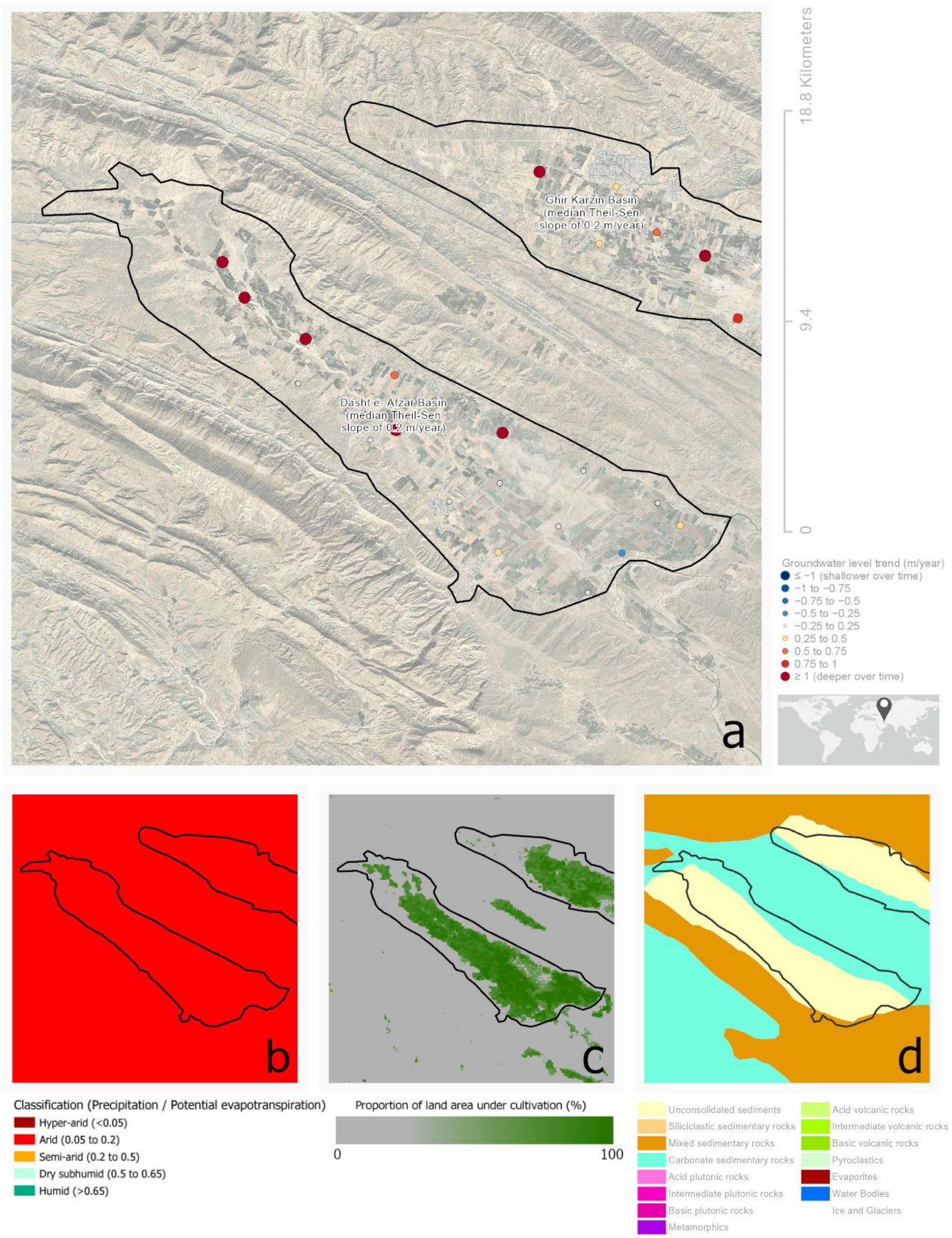

Supplementary Fig. 96. Spatial variability of trends in depth to groundwater Dasht e- Afzar Basin. For detailed description of the contents of each panel see the text at the beginning of Supplementary Note 17.

Dasht-e Rostam Basin (median Theil-Sen slope of 0.3 m/year)

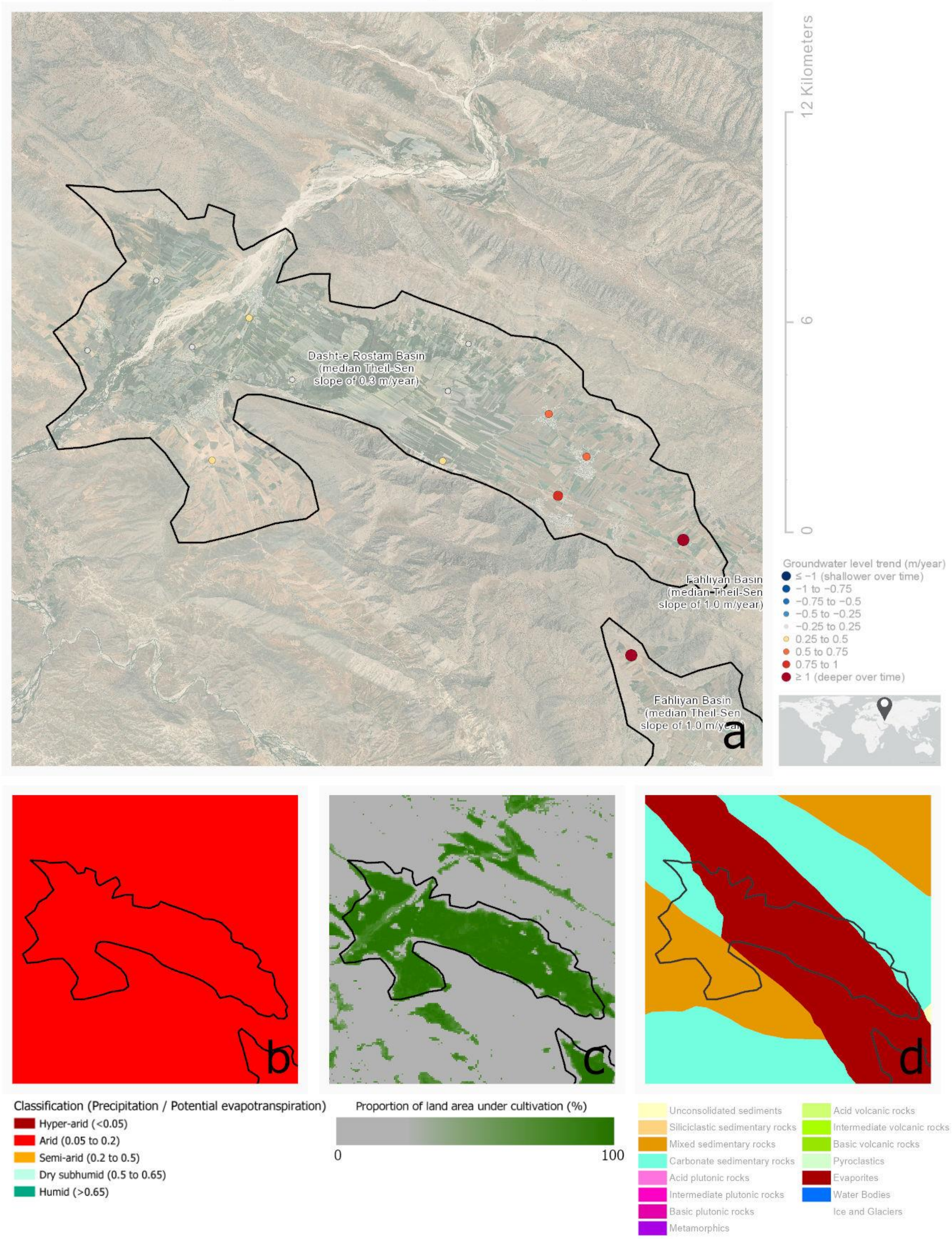

Supplementary Fig. 97. Spatial variability of trends in depth to groundwater Dasht-e Rostam Basin. For detailed description of the contents of each panel see the text at the beginning of Supplementary Note 17.

Dayton Valley (median Theil-Sen slope of 0 m/year)

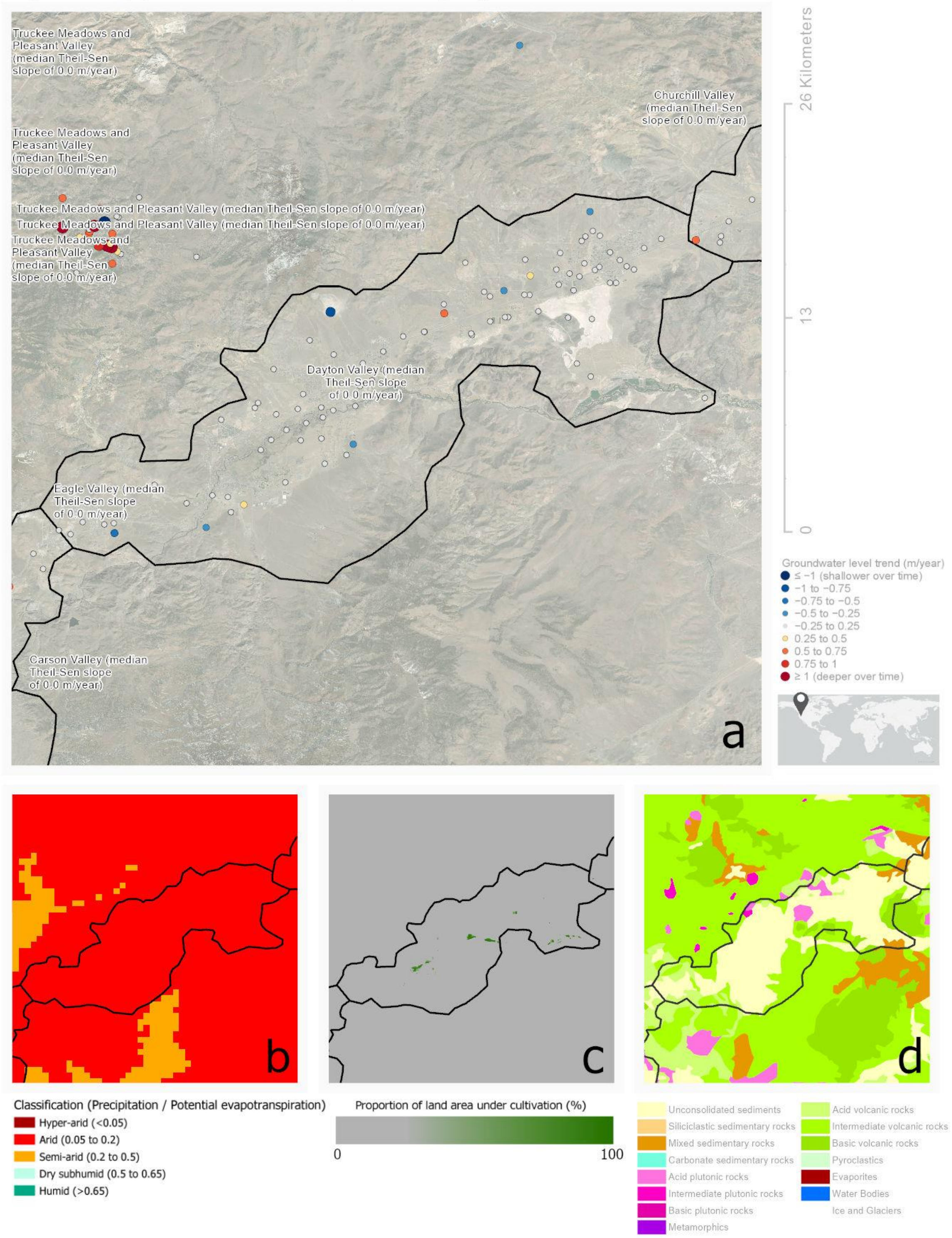

Supplementary Fig. 98. Spatial variability of trends in depth to groundwater Dayton Valley (within the broader Carson River Basin). For detailed description of the contents of each panel see the text at the beginning of Supplementary Note 17.

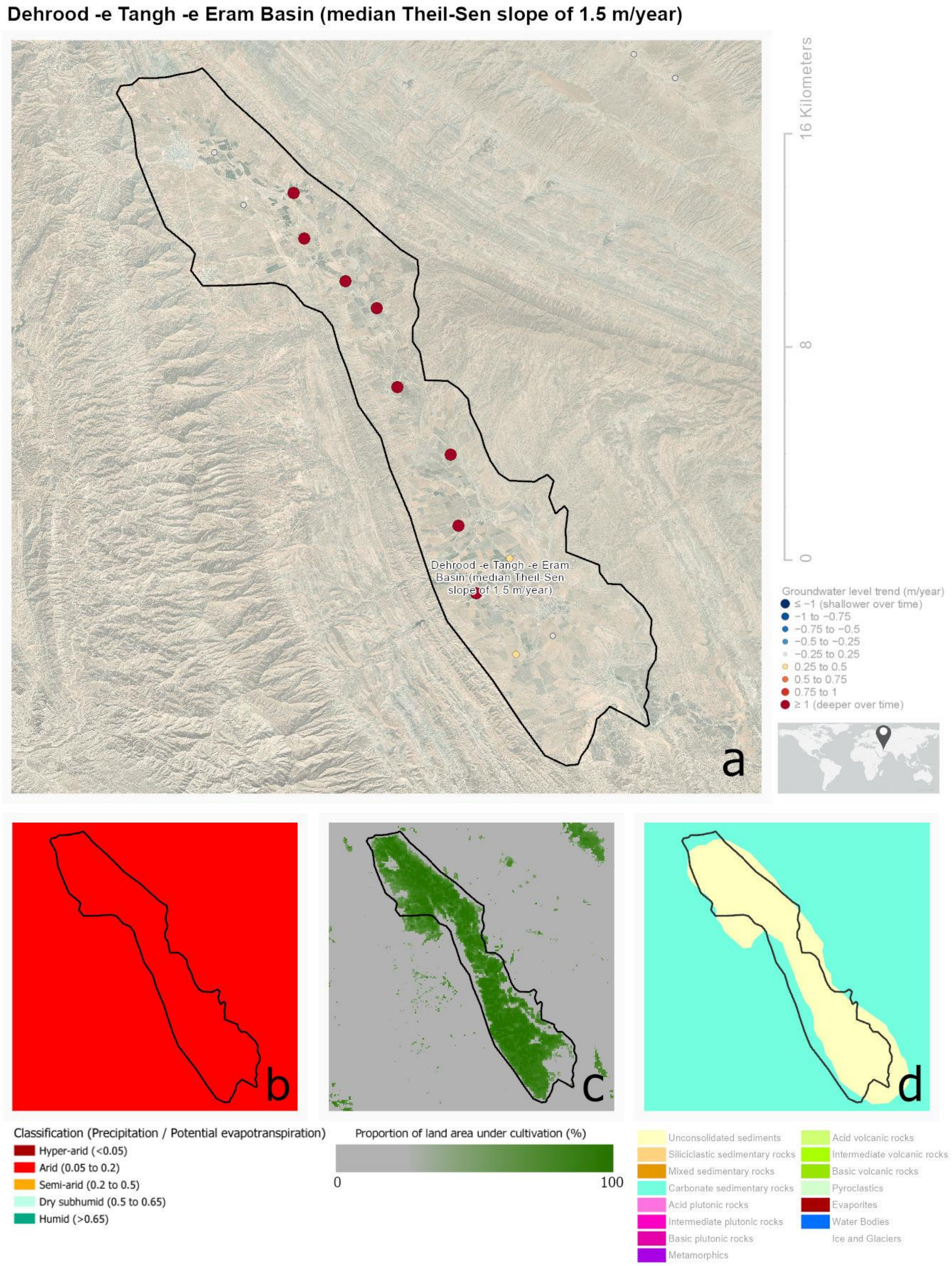

Supplementary Fig. 99. Spatial variability of trends in depth to groundwater Dehrood -e Tangh -e Eram Basin. For detailed description of the contents of each panel see the text at the beginning of Supplementary Note 17.

Delta-Mendota Basin (median Theil-Sen slope of 0.1 m/year)

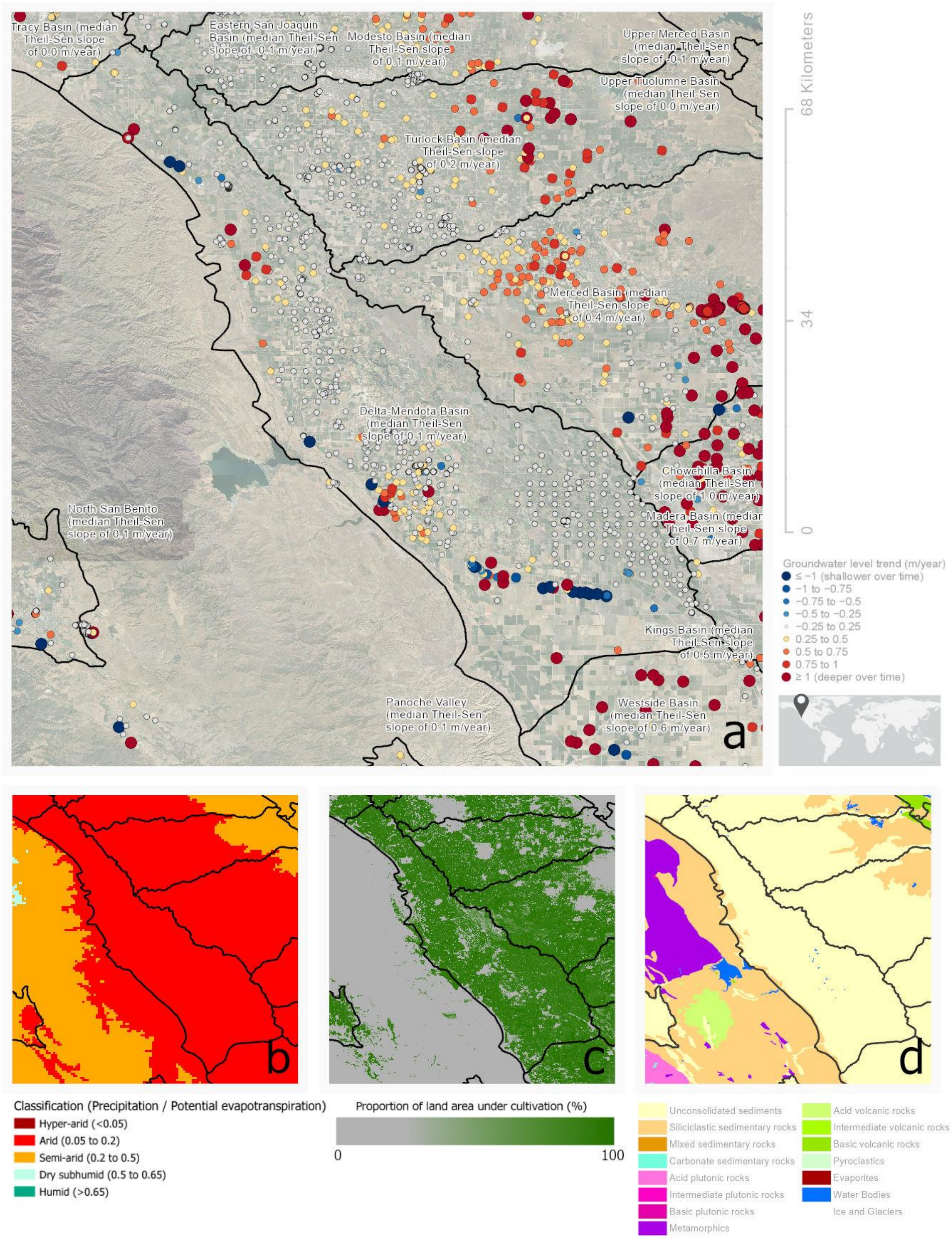

Supplementary Fig. 100. Spatial variability of trends in depth to groundwater Delta-Mendota Basin (within the broader San Joaquin Basin of the Central Valley). For detailed description of the contents of each panel see the text at the beginning of Supplementary Note 17.

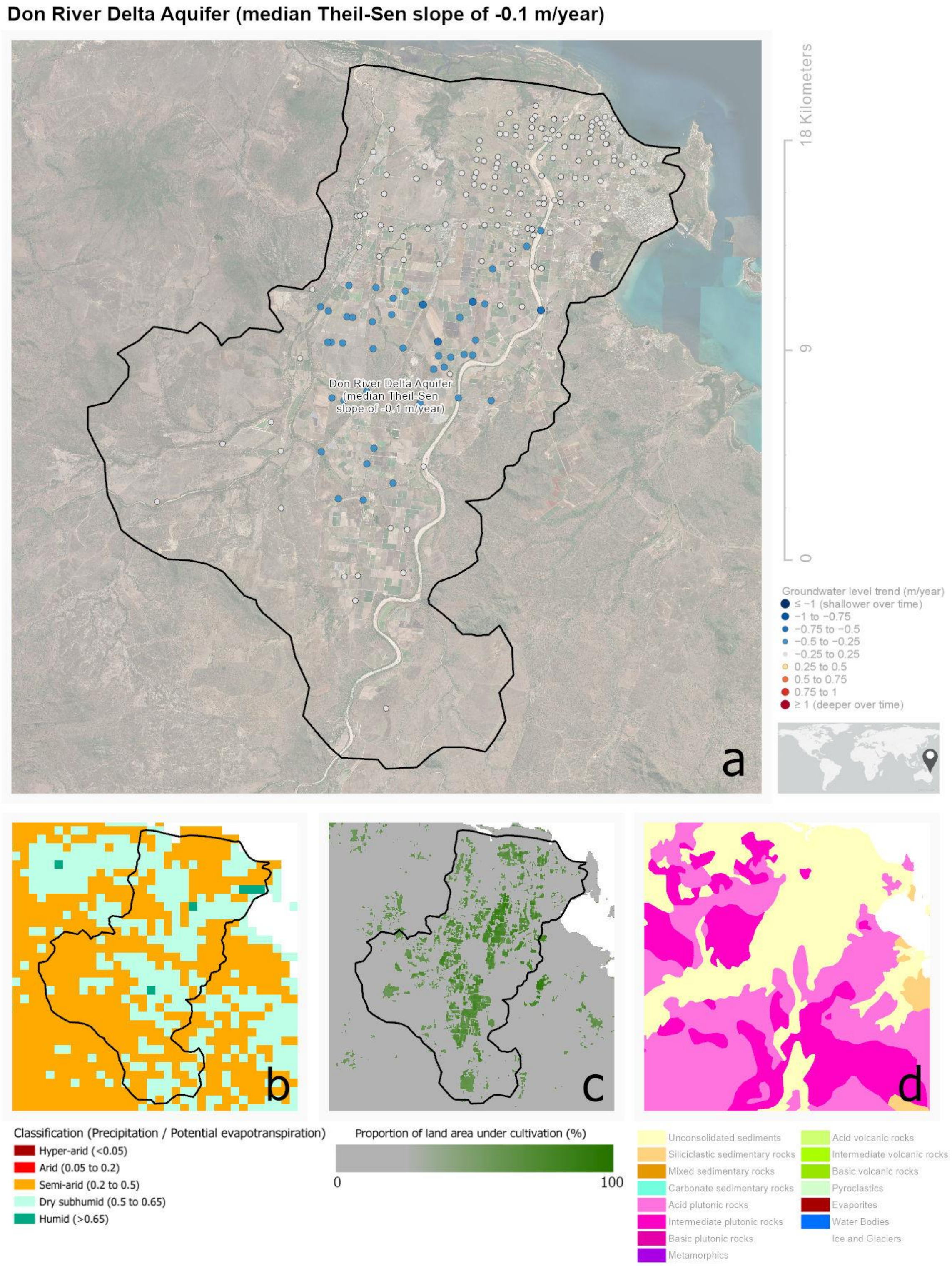

Supplementary Fig. 101. Spatial variability of trends in depth to groundwater Don River Delta Aquifer. For detailed description of the contents of each panel see the text at the beginning of Supplementary Note 17.

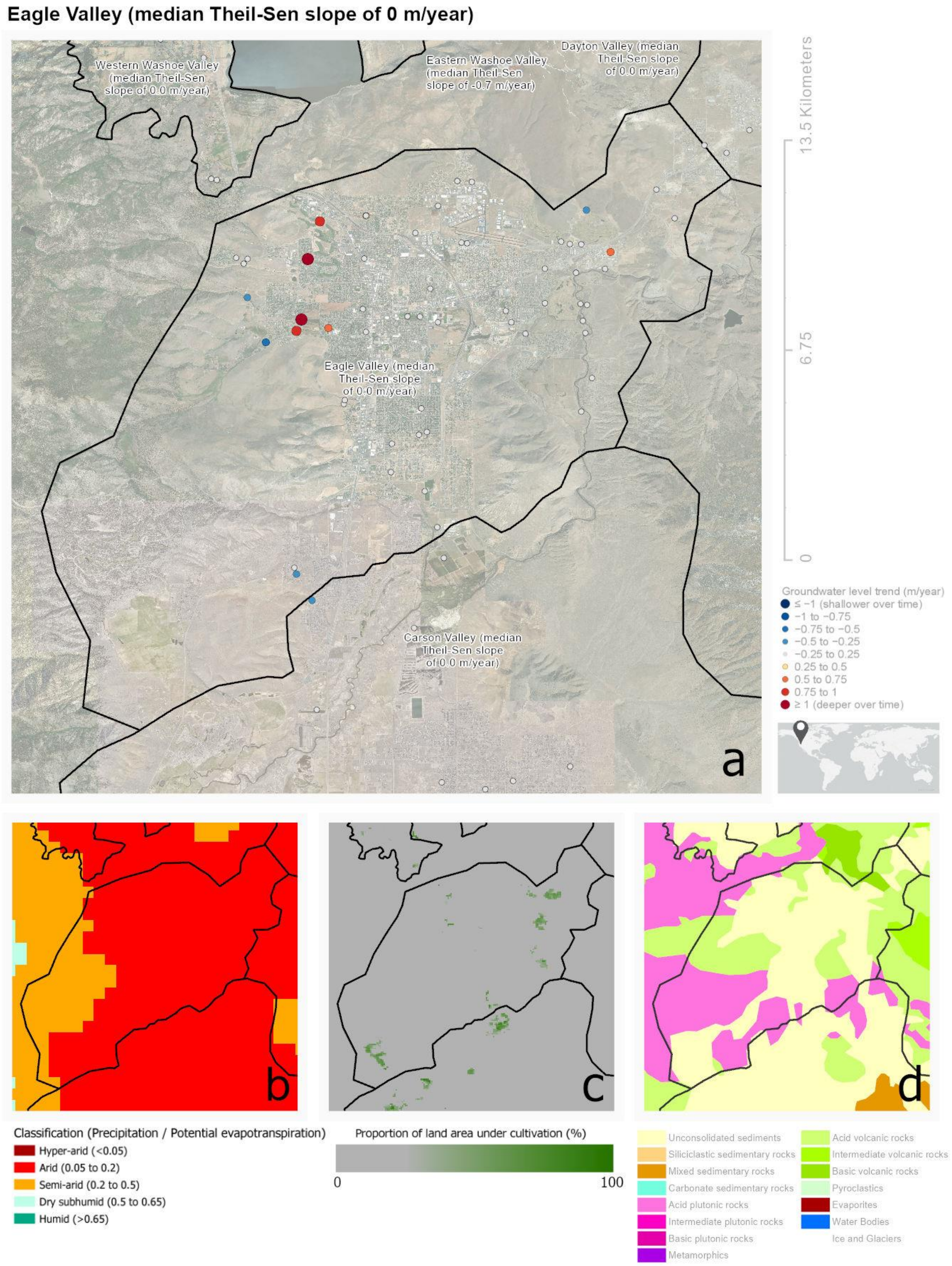

Supplementary Fig. 102. Spatial variability of trends in depth to groundwater Eagle Valley (within the broader Carson River Basin). For detailed description of the contents of each panel see the text at the beginning of Supplementary Note 17.

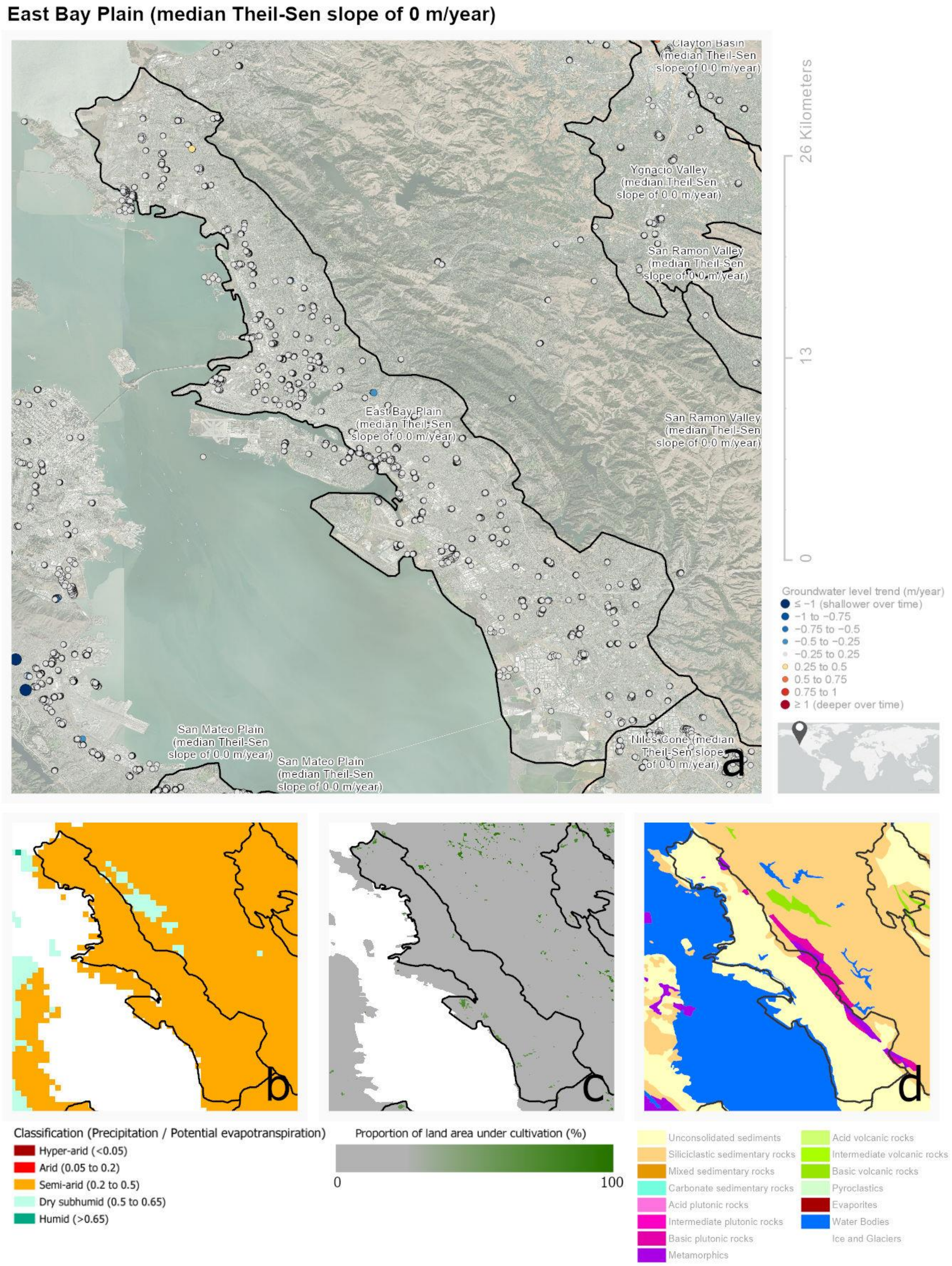

Supplementary Fig. 103. Spatial variability of trends in depth to groundwater East Bay Plain. For detailed description of the contents of each panel see the text at the beginning of Supplementary Note 17.

East Contra Basin (median Theil-Sen slope of 0 m/year)

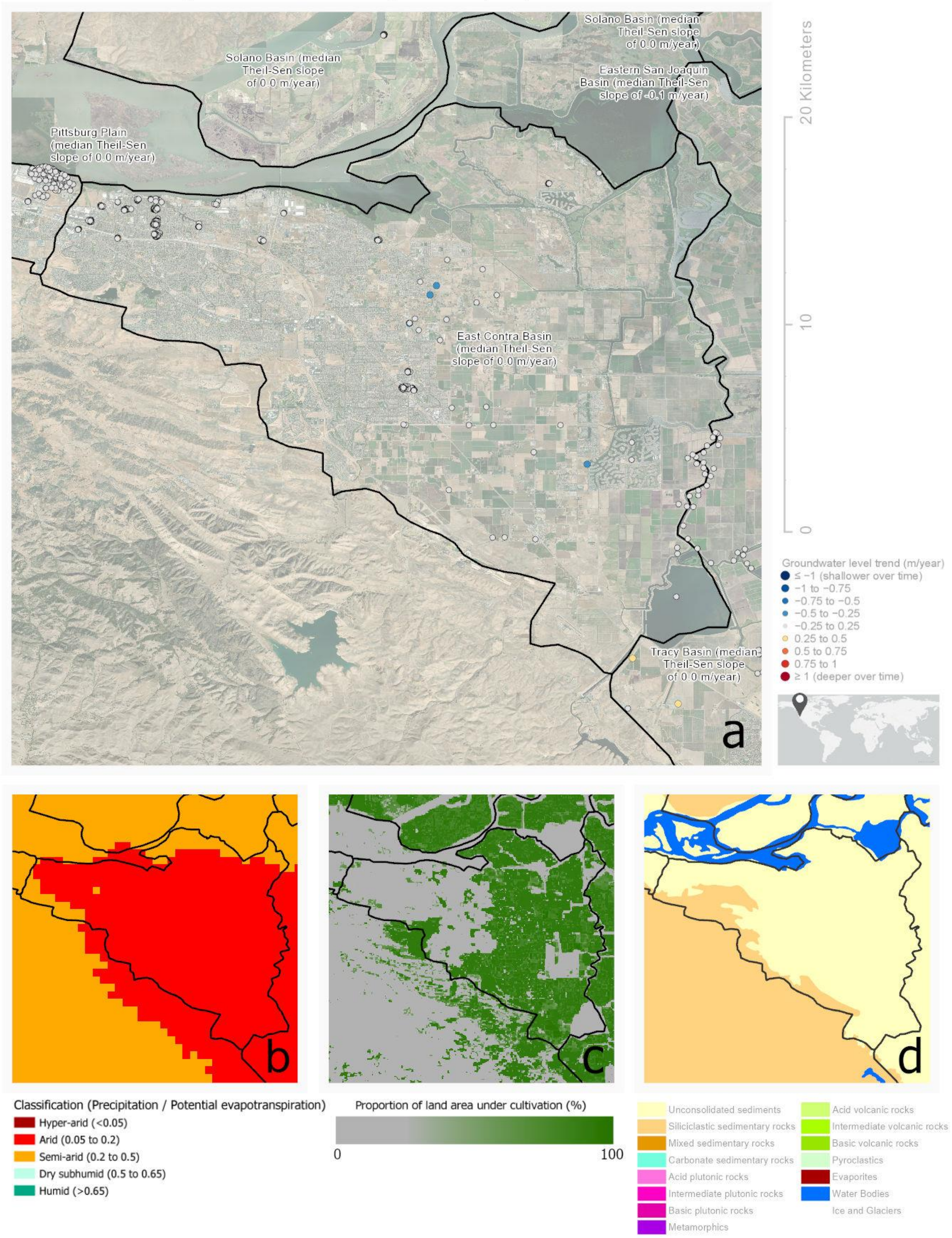

Supplementary Fig. 104. Spatial variability of trends in depth to groundwater East Contra Basin (within the broader San Joaquin Basin of the Central Valley). For detailed description of the contents of each panel see the text at the beginning of Supplementary Note 17.

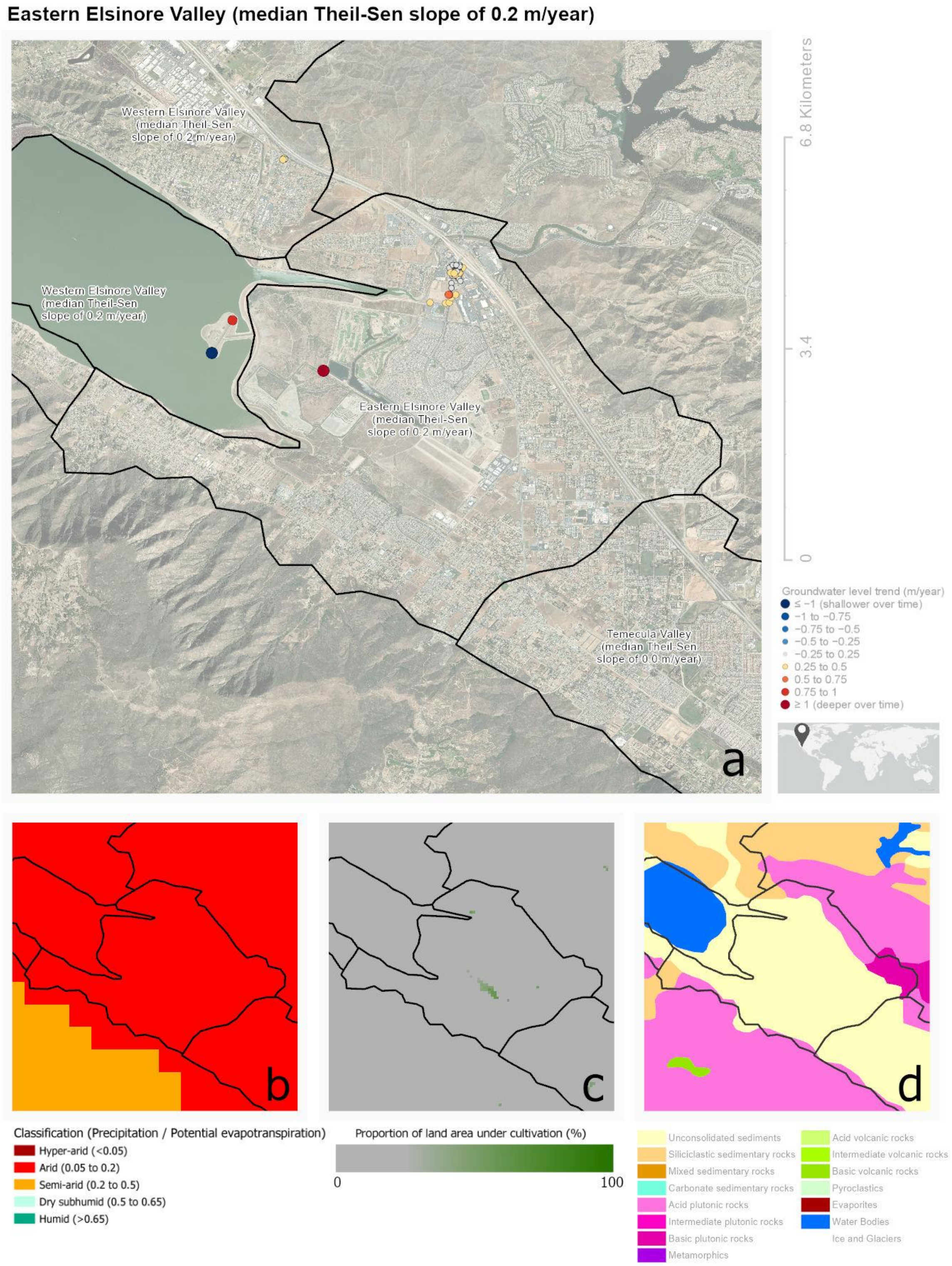

Supplementary Fig. 105. Spatial variability of trends in depth to groundwater Eastern Elsinore Valley. For detailed description of the contents of each panel see the text at the beginning of Supplementary Note 17.

Eastern Rezvan Basin (median Theil-Sen slope of 0.9 m/year)

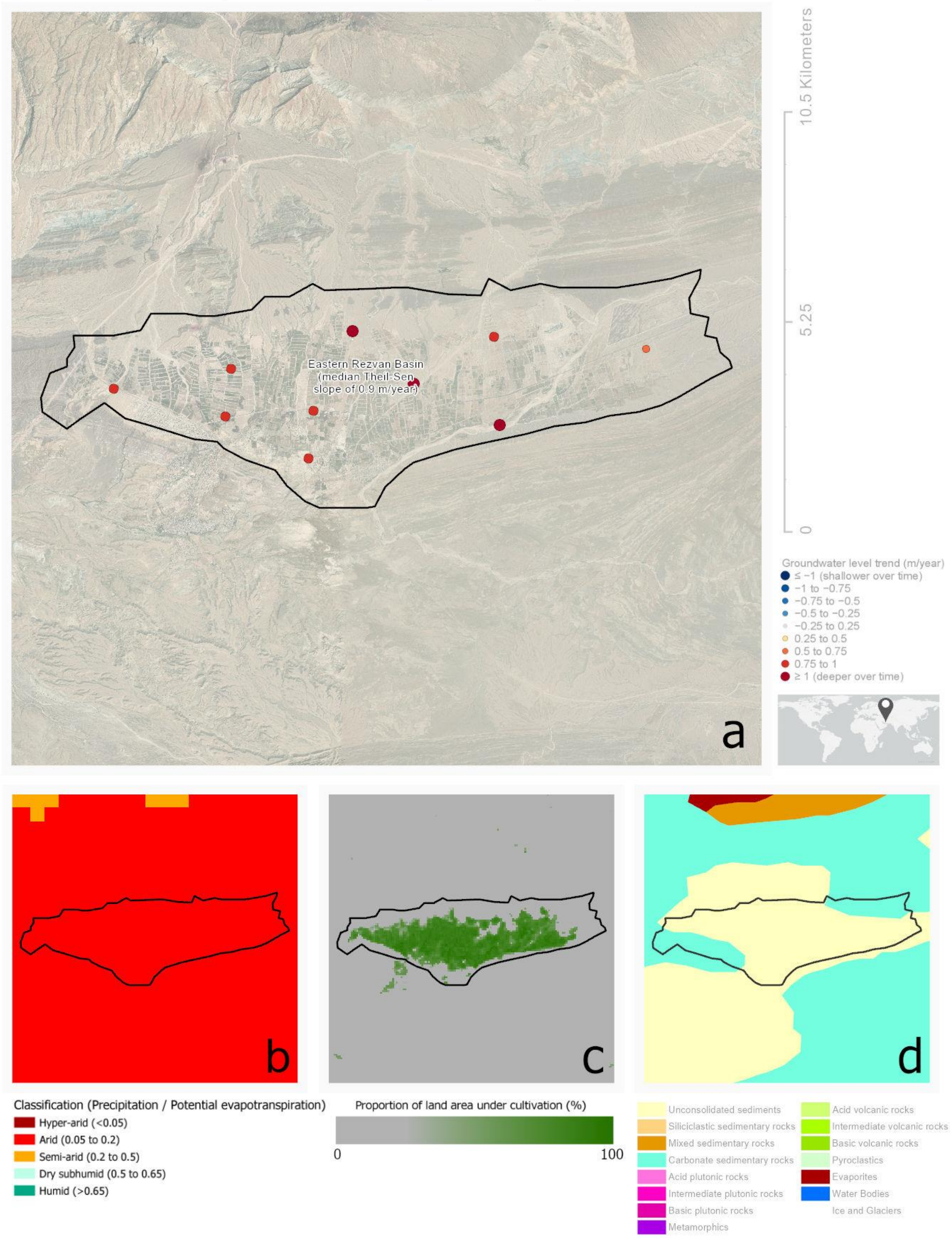

Supplementary Fig. 106. Spatial variability of trends in depth to groundwater Eastern Rezvan Basin. For detailed description of the contents of each panel see the text at the beginning of Supplementary Note 17.

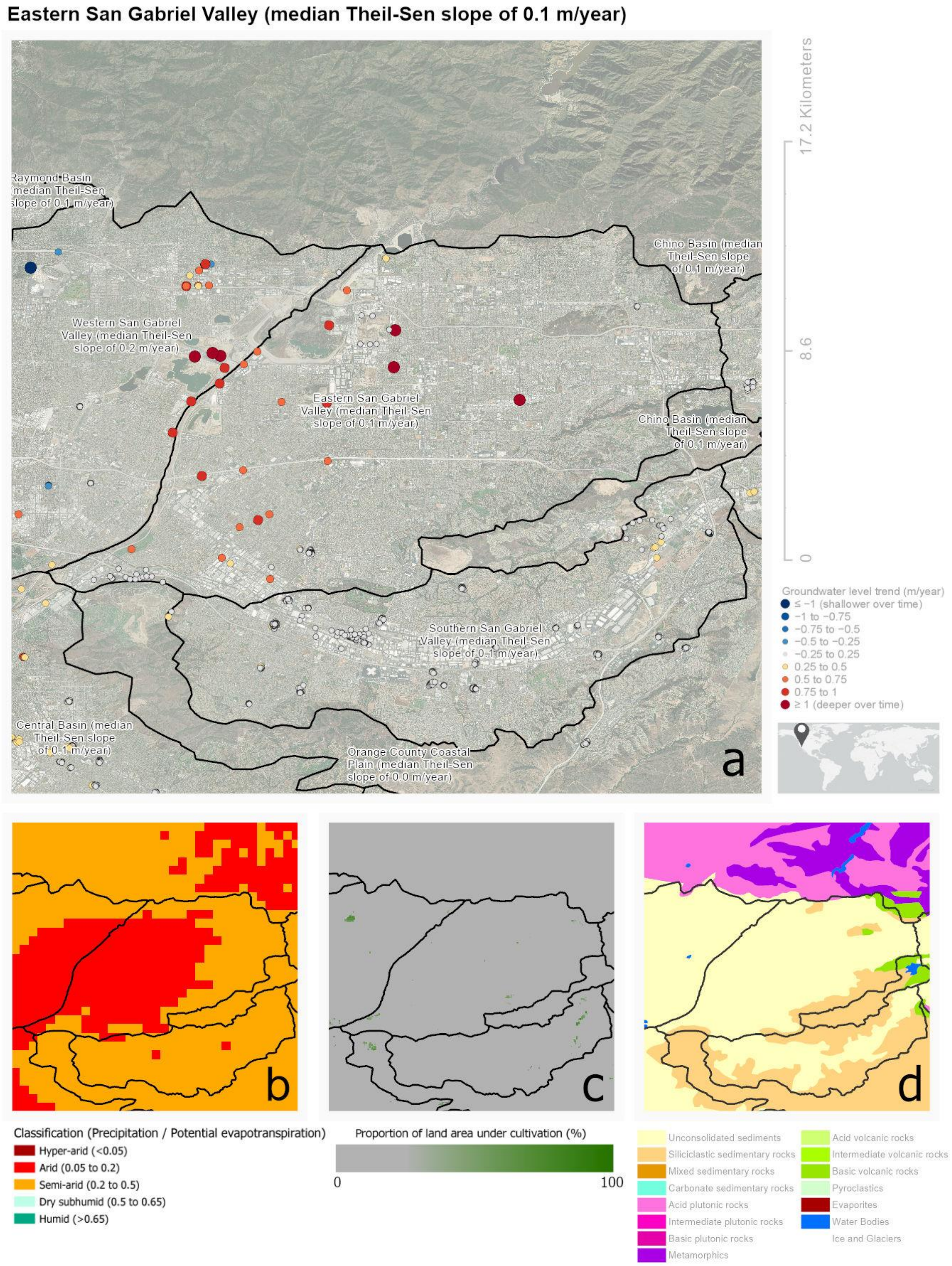

Supplementary Fig. 107. Spatial variability of trends in depth to groundwater Eastern San Gabriel Valley (within the broader San Gabriel Valley). For detailed description of the contents of each panel see the text at the beginning of Supplementary Note 17.

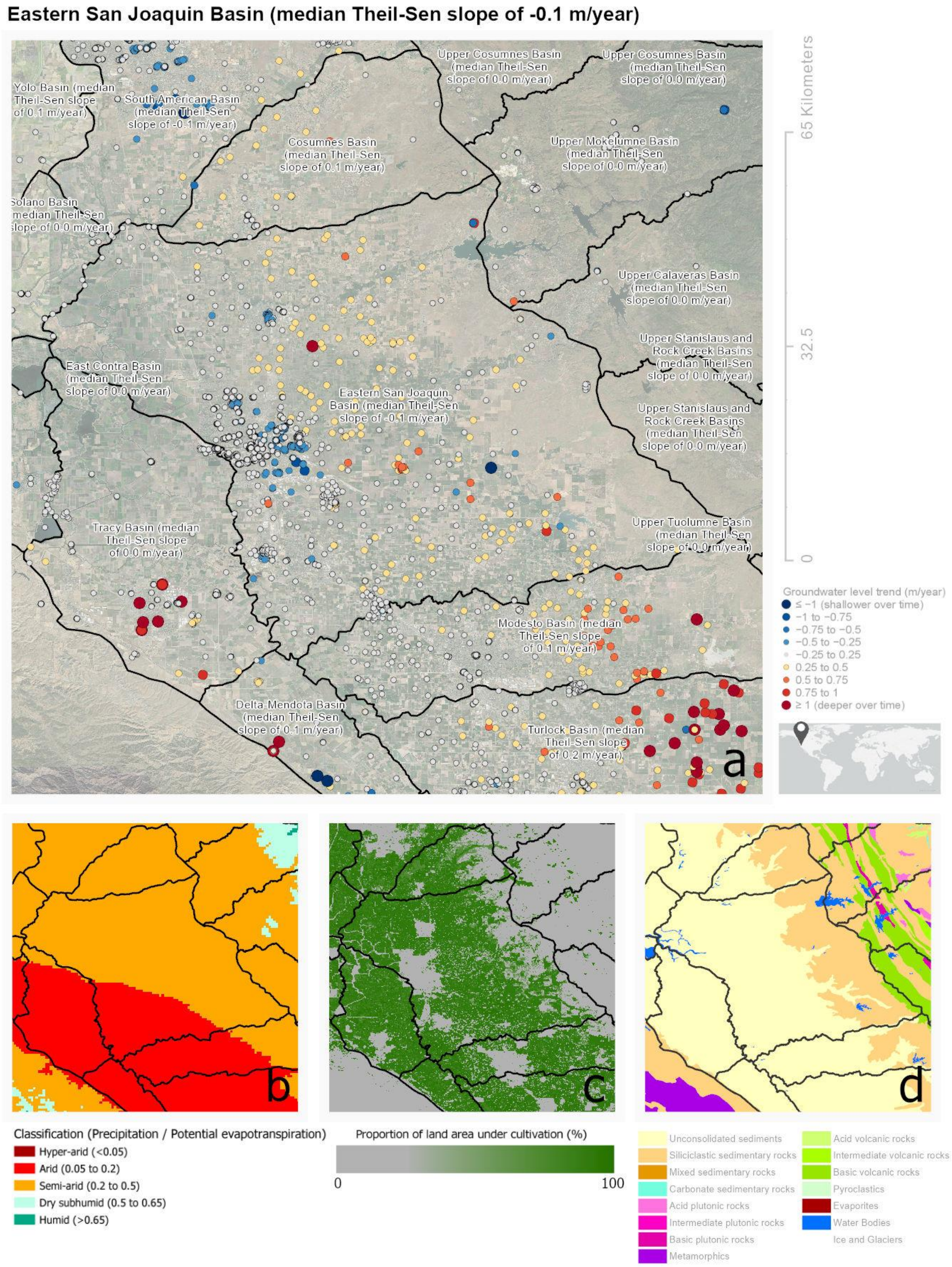

Supplementary Fig. 108. Spatial variability of trends in depth to groundwater Eastern San Joaquin Basin (within the broader San Joaquin Basin of the Central Valley). For detailed description of the contents of each panel see the text at the beginning of Supplementary Note 17.

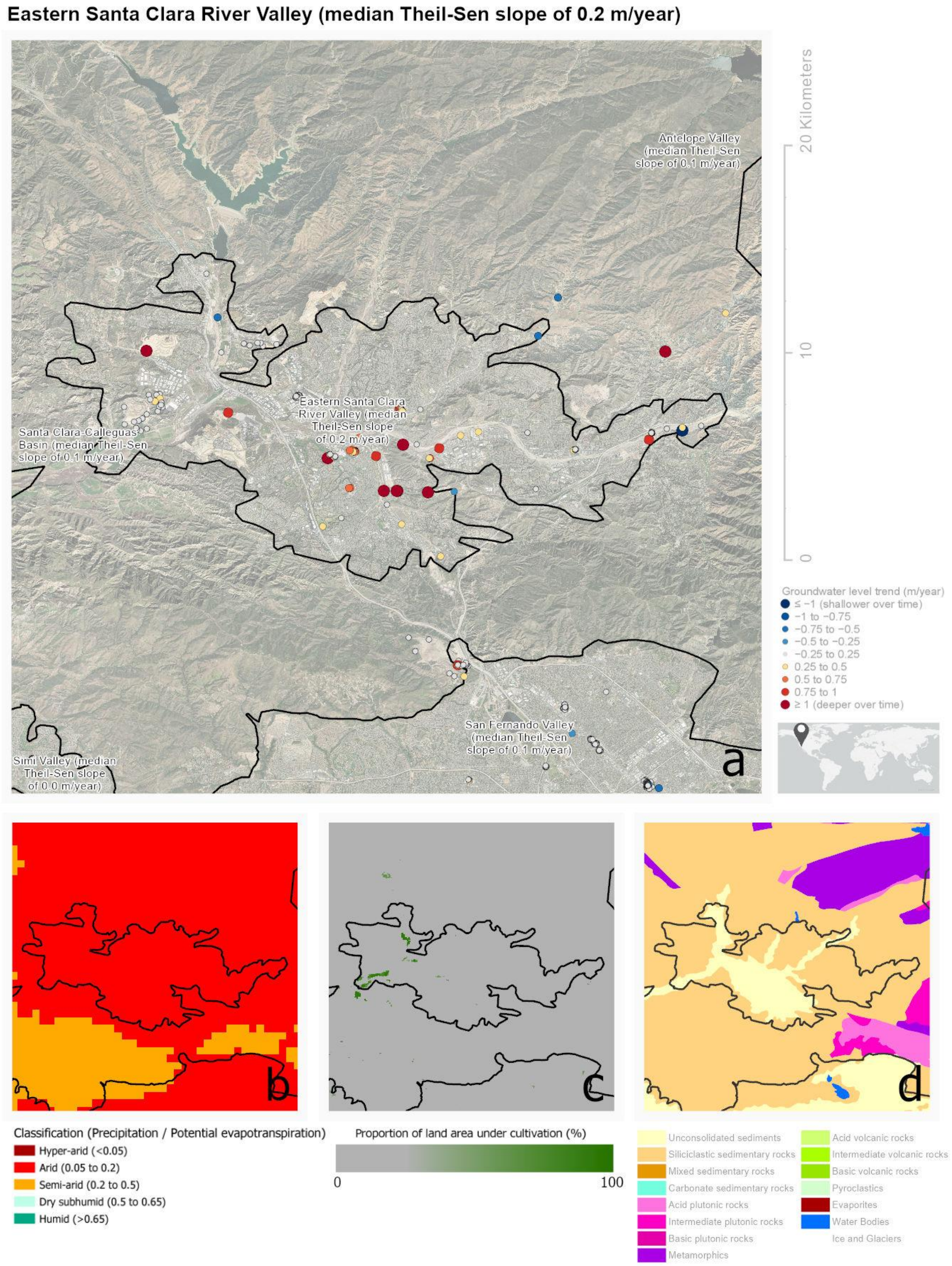

Supplementary Fig. 109. Spatial variability of trends in depth to groundwater Eastern Santa Clara River Valley. For detailed description of the contents of each panel see the text at the beginning of Supplementary Note 17.

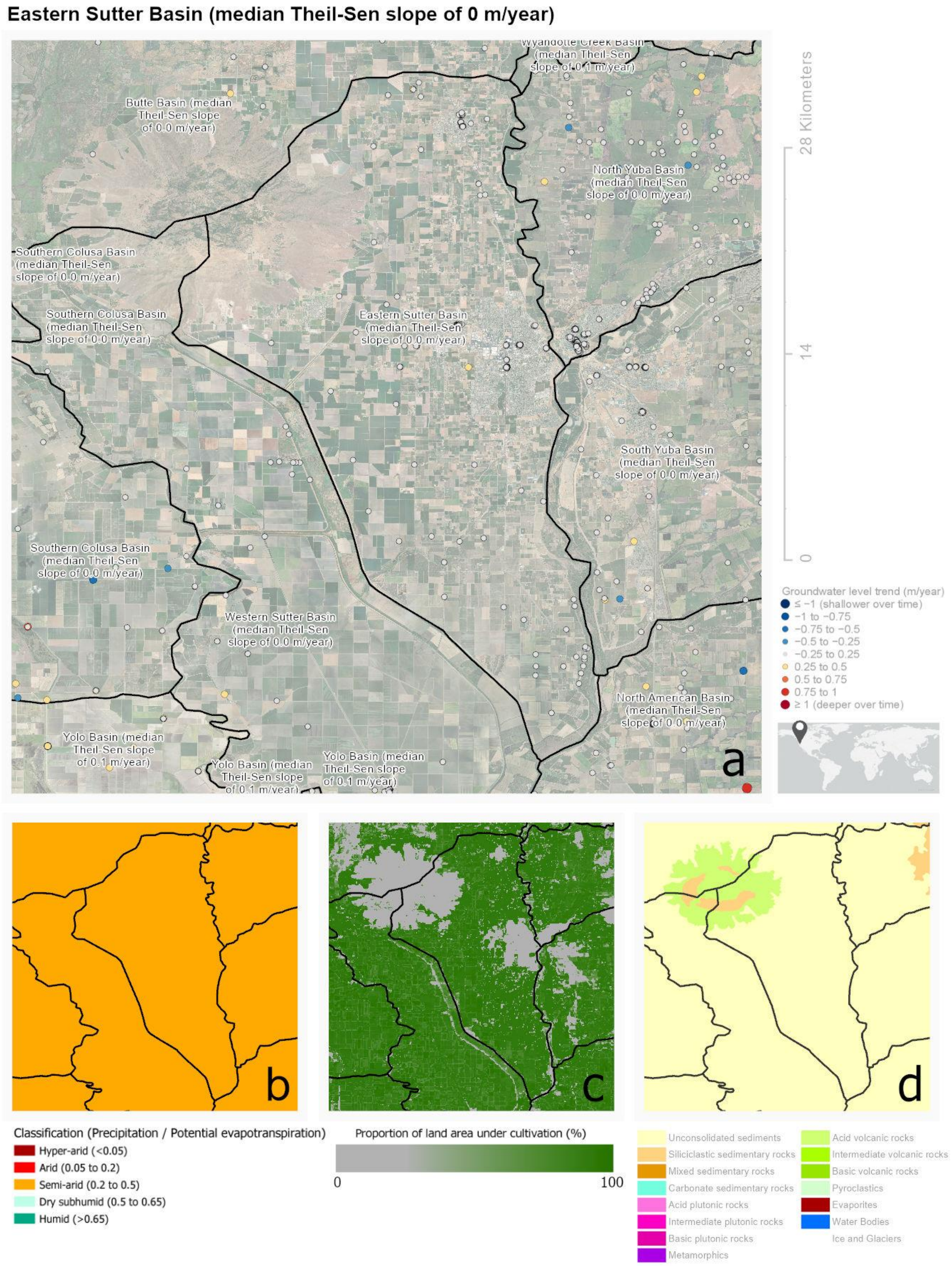

Supplementary Fig. 110. Spatial variability of trends in depth to groundwater Eastern Sutter Basin (within the broader Sacramento Valley of the Central Valley). For detailed description of the contents of each panel see the text at the beginning of Supplementary Note 17.

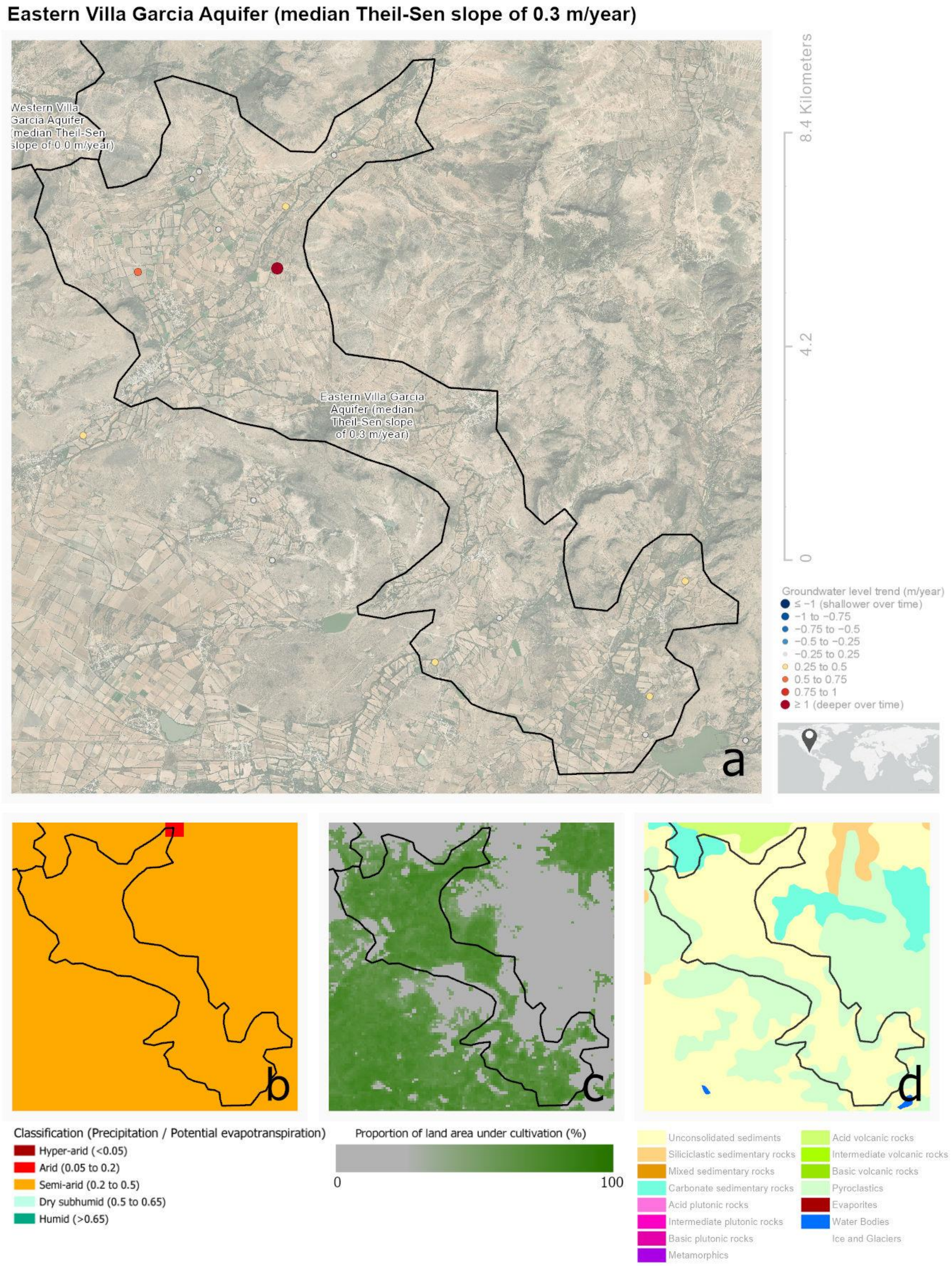

Supplementary Fig. 111. Spatial variability of trends in depth to groundwater Eastern Villa Garcia Aquifer. For detailed description of the contents of each panel see the text at the beginning of Supplementary Note 17.

El Cajon Valley (median Theil-Sen slope of 0 m/year)

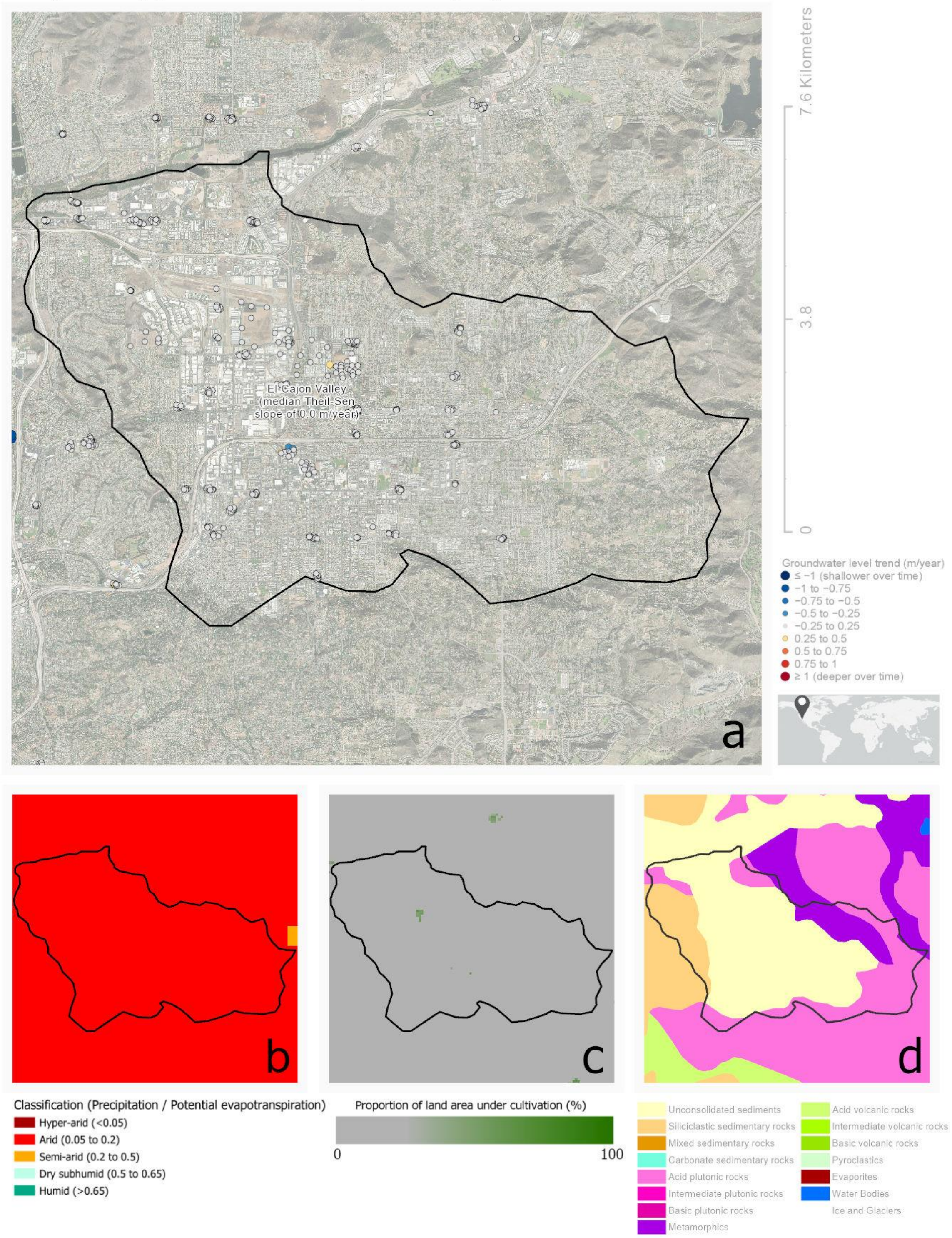

Supplementary Fig. 112. Spatial variability of trends in depth to groundwater El Cajon Valley. For detailed description of the contents of each panel see the text at the beginning of Supplementary Note 17.

Equus Beds (median Theil-Sen slope of 0 m/year)

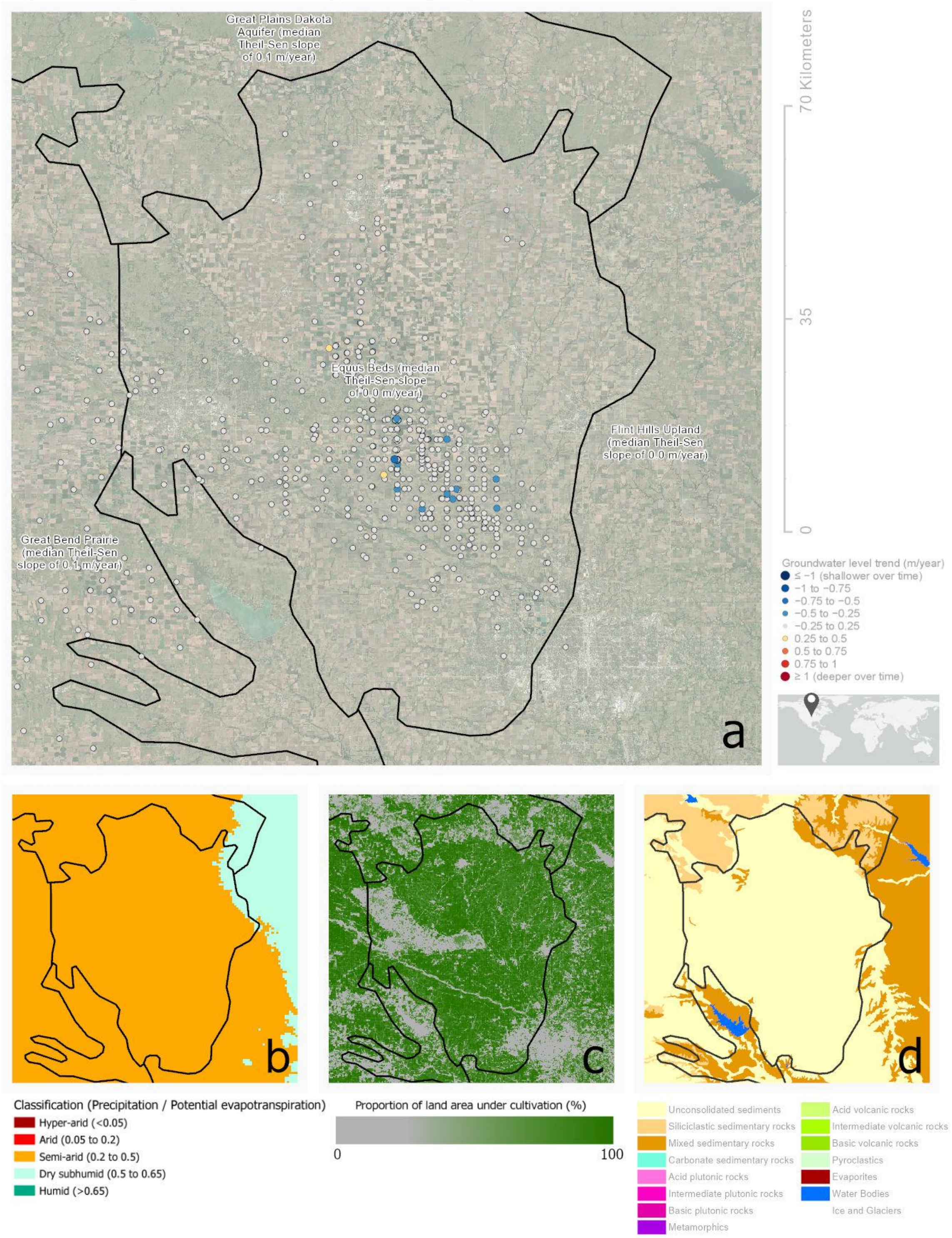

Supplementary Fig. 113. Spatial variability of trends in depth to groundwater Equus Beds (within the broader High Plains). For detailed description of the contents of each panel see the text at the beginning of Supplementary Note 17.

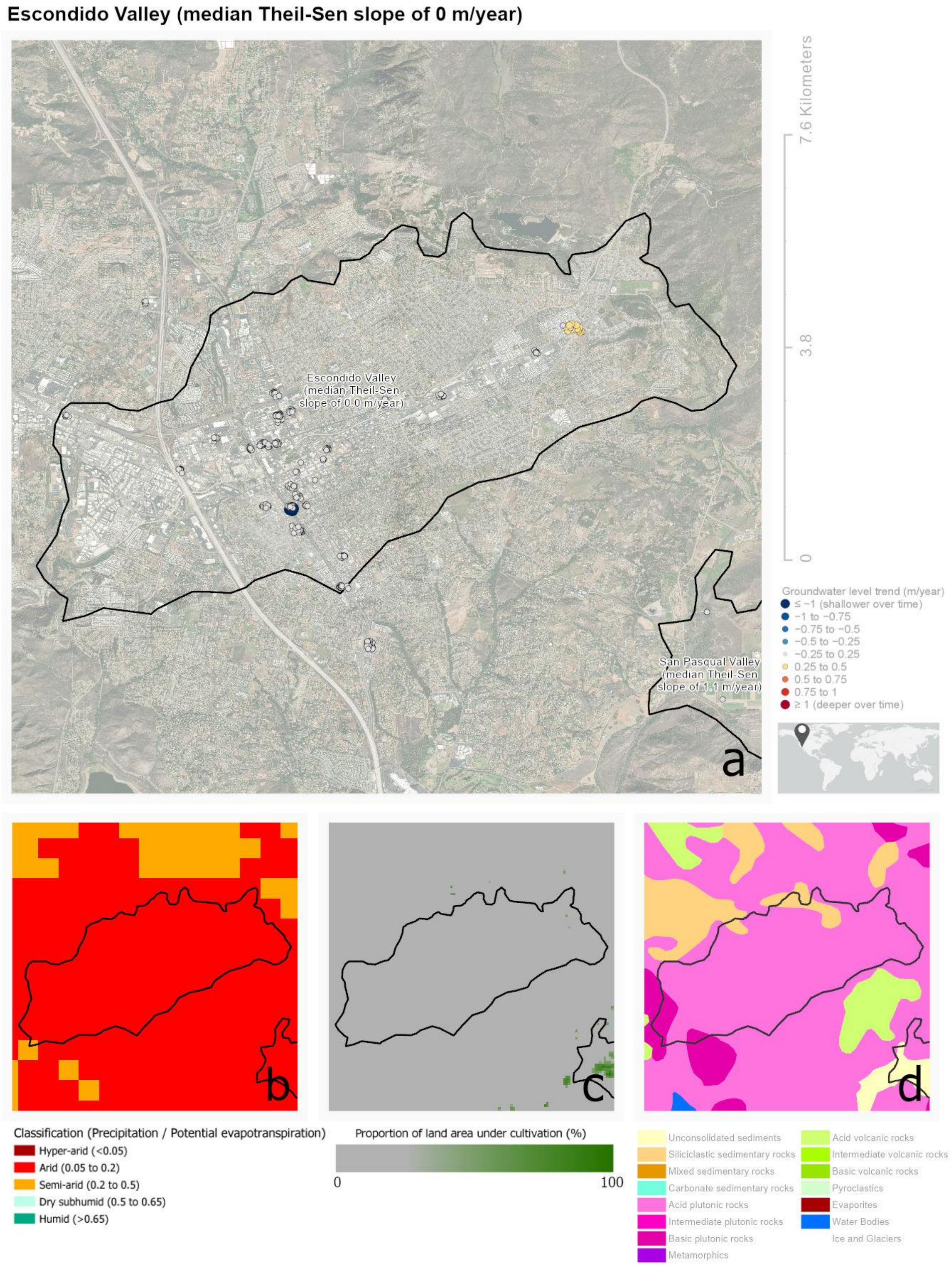

Supplementary Fig. 114. Spatial variability of trends in depth to groundwater Escondido Valley. For detailed description of the contents of each panel see the text at the beginning of Supplementary Note 17.

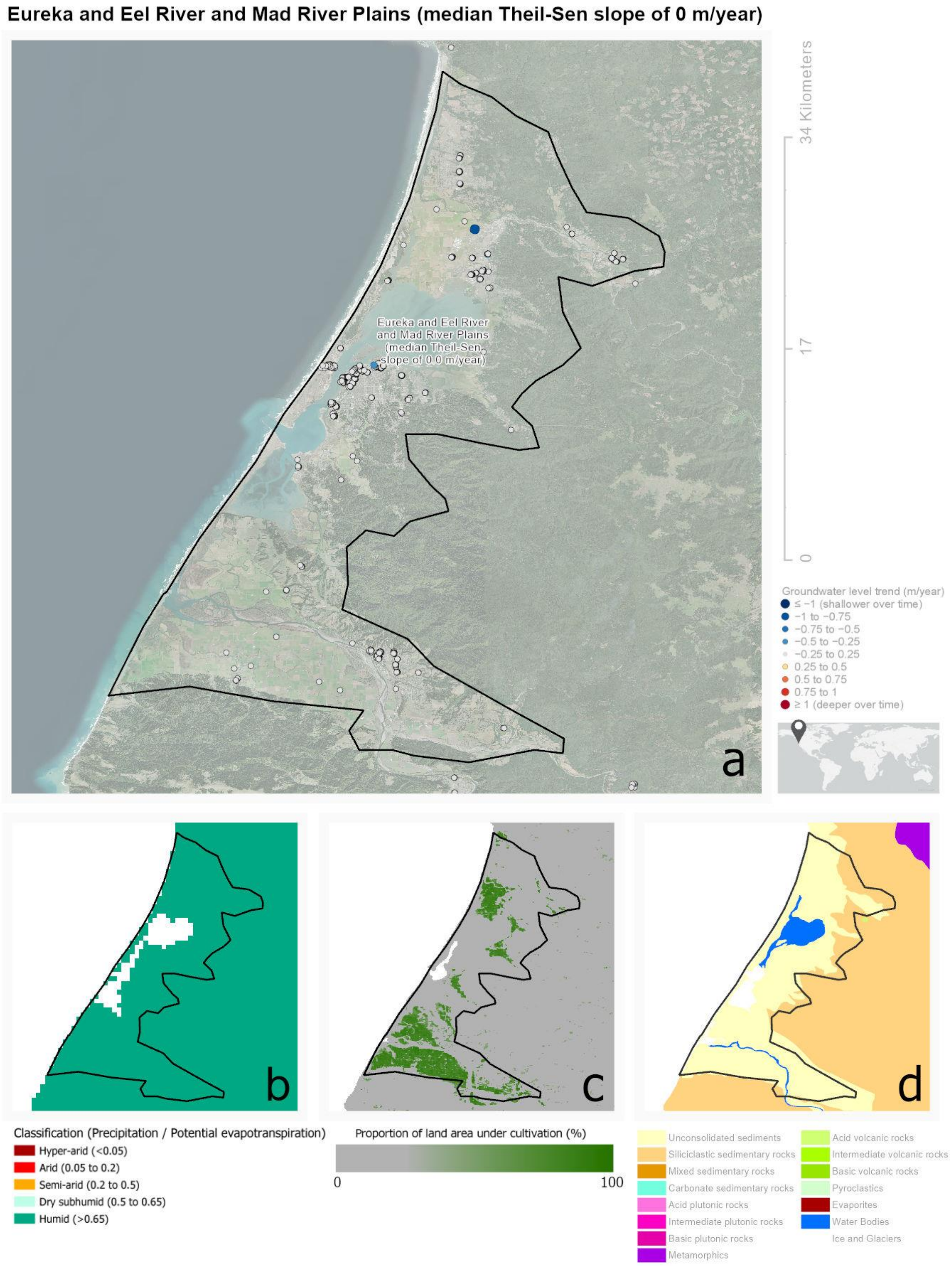

Supplementary Fig. 115. Spatial variability of trends in depth to groundwater Eureka and Eel River and Mad River Plains. For detailed description of the contents of each panel see the text at the beginning of Supplementary Note 17.

Fahliyan Basin (median Theil-Sen slope of 1 m/year)

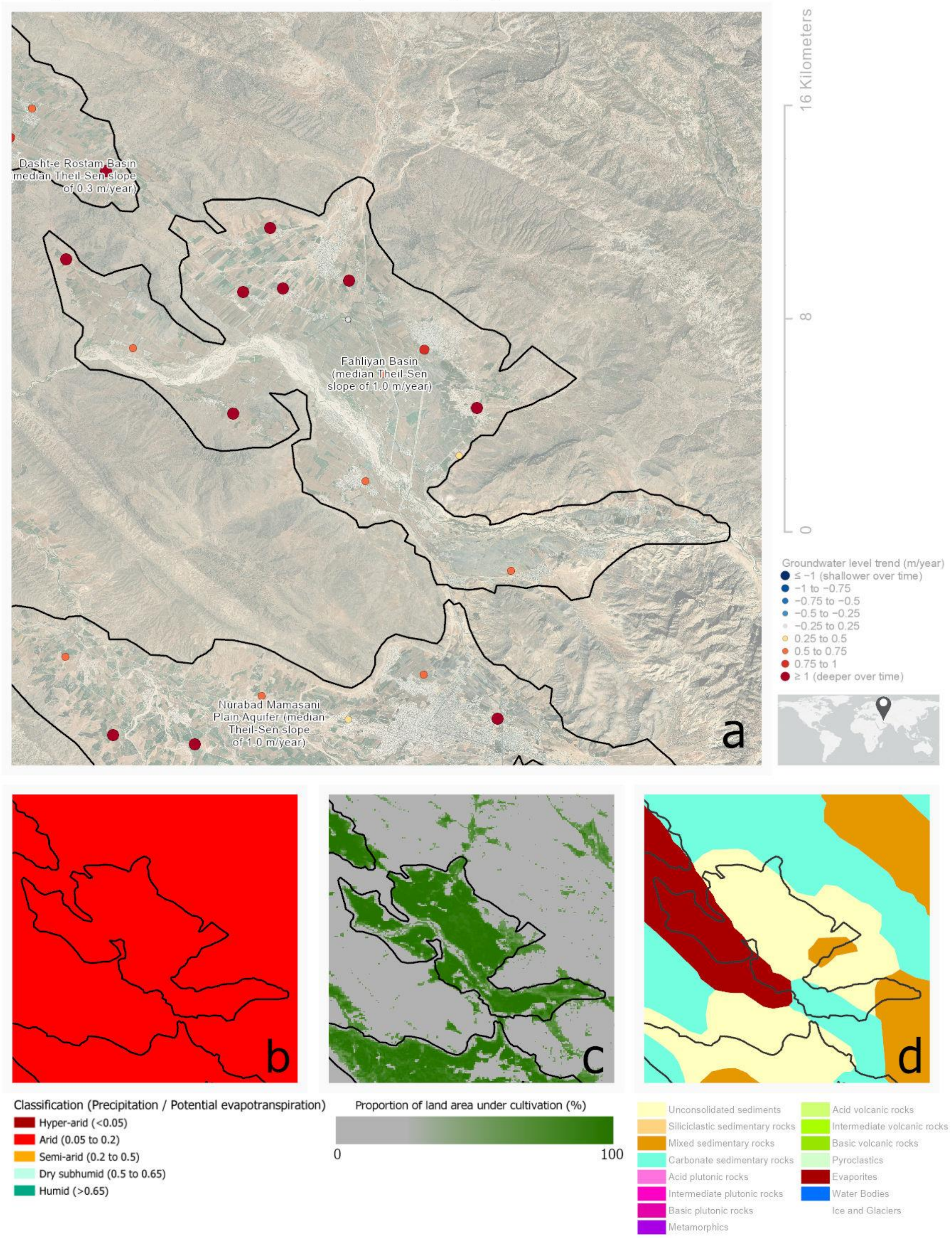

Supplementary Fig. 116. Spatial variability of trends in depth to groundwater Fahliyan Basin. For detailed description of the contents of each panel see the text at the beginning of Supplementary Note 17.

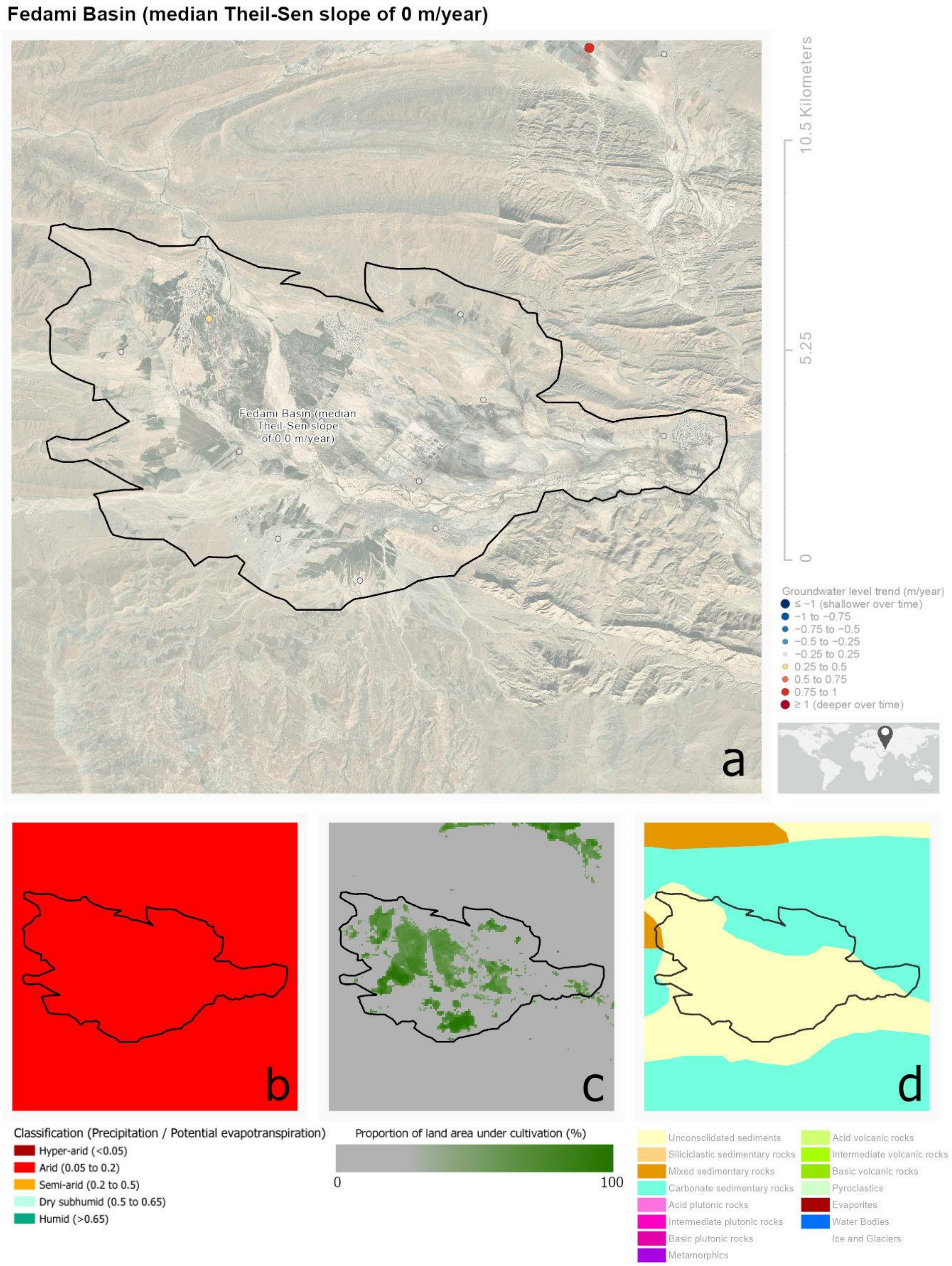

Supplementary Fig. 117. Spatial variability of trends in depth to groundwater Fedami Basin. For detailed description of the contents of each panel see the text at the beginning of Supplementary Note 17.

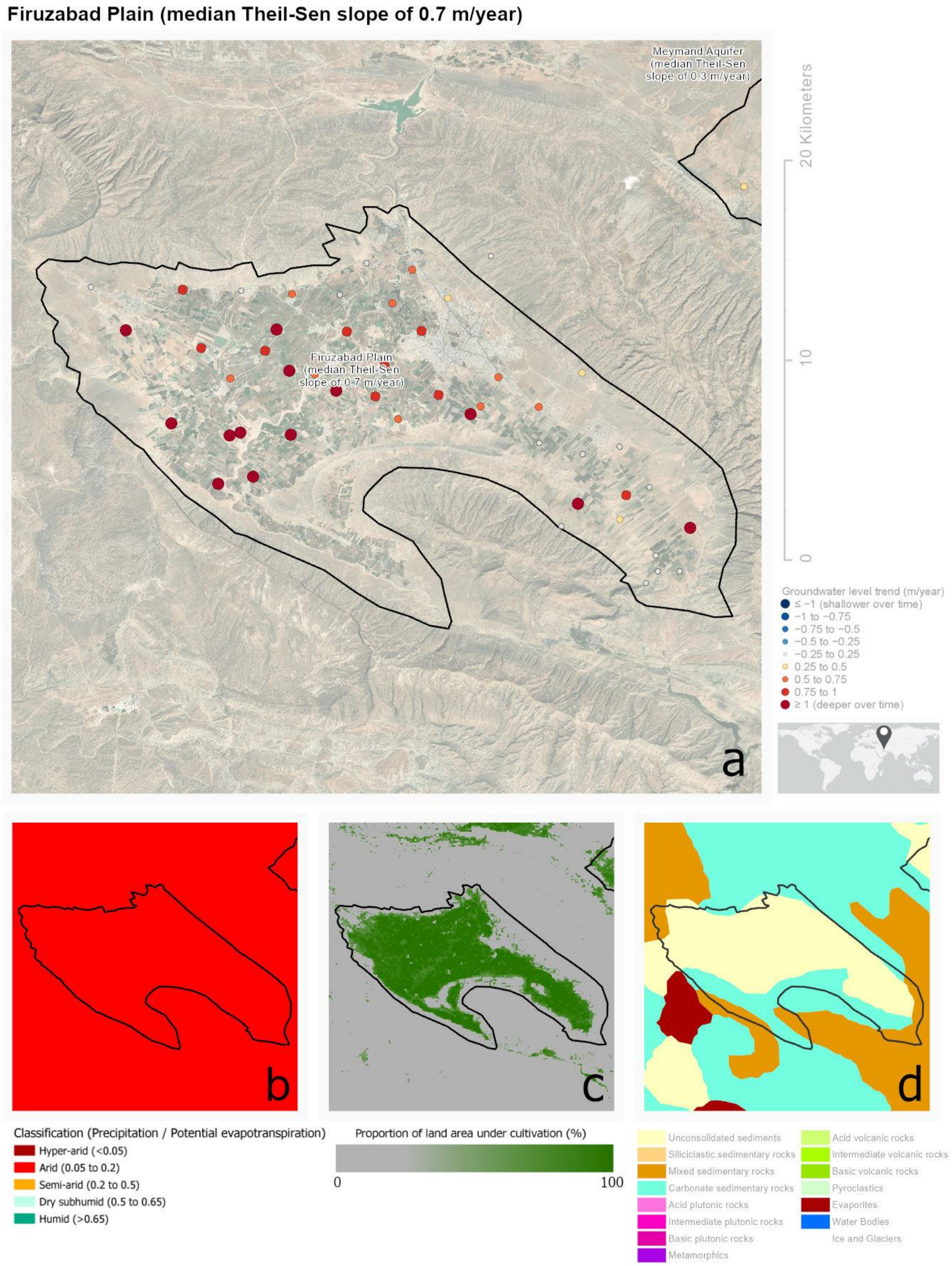

Supplementary Fig. 118. Spatial variability of trends in depth to groundwater Firuzabad Plain. For detailed description of the contents of each panel see the text at the beginning of Supplementary Note 17.

Fort Bragg Terrace (median Theil-Sen slope of 0 m/year)

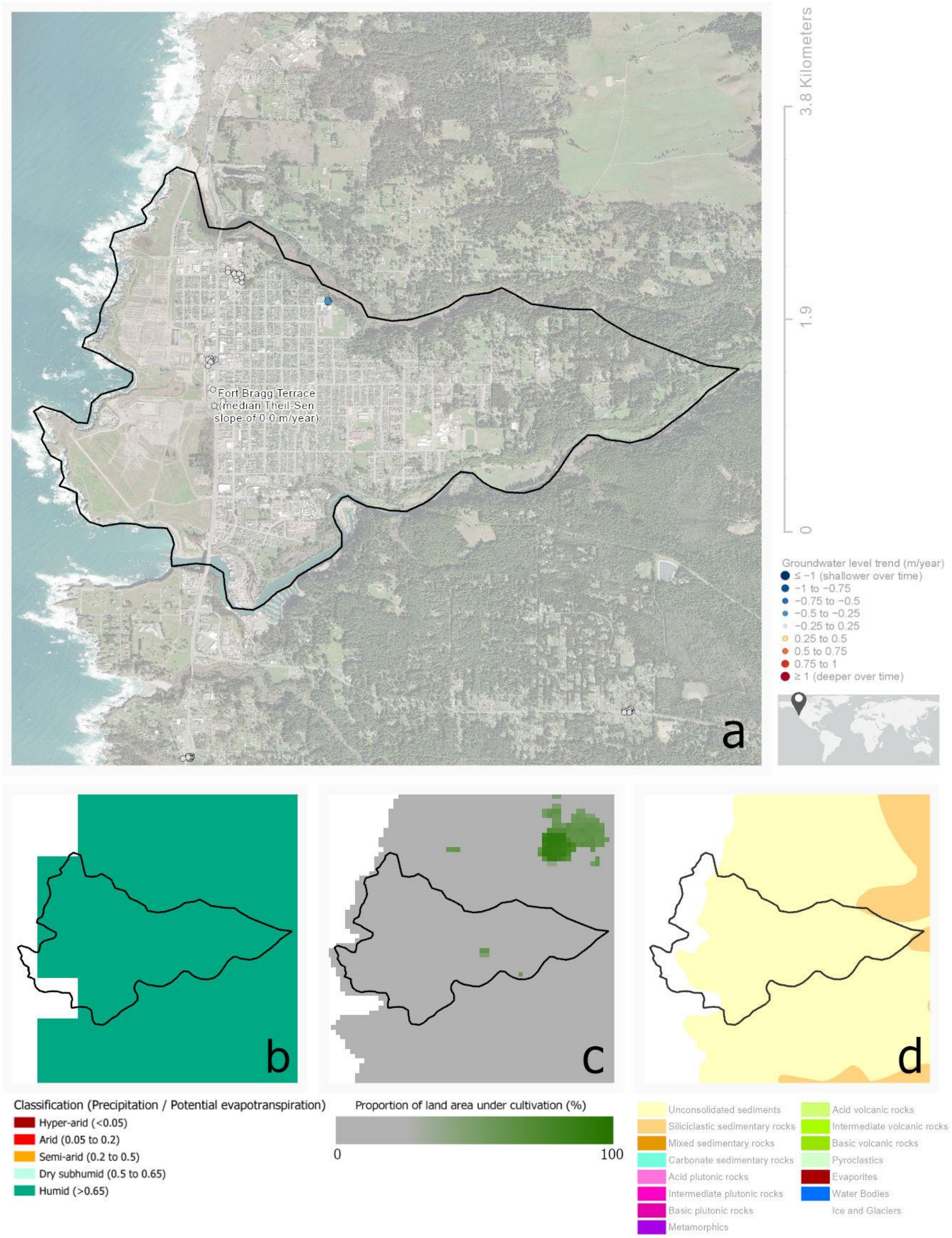

Supplementary Fig. 119. Spatial variability of trends in depth to groundwater Fort Bragg Terrace. For detailed description of the contents of each panel see the text at the beginning of Supplementary Note 17.

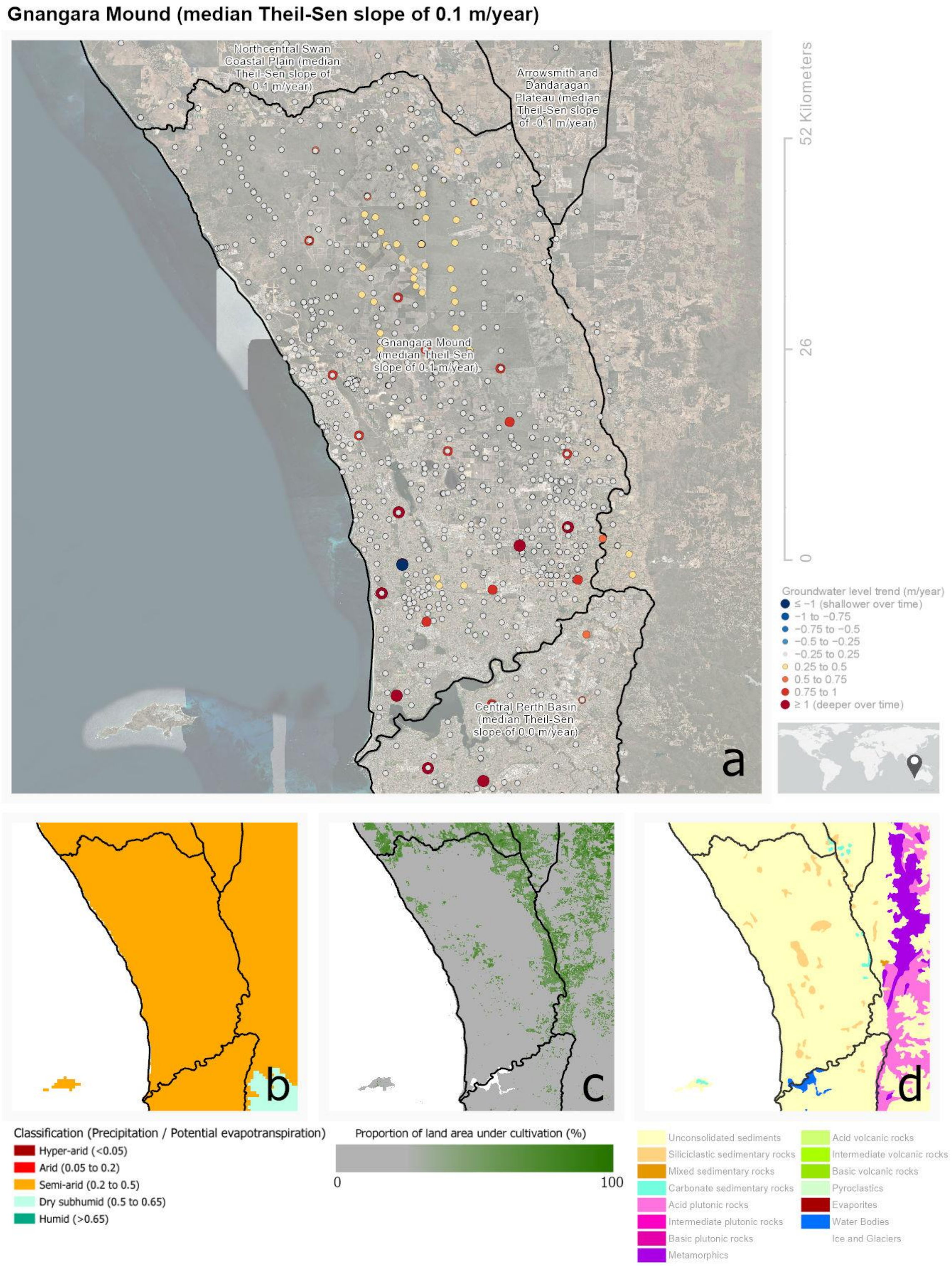

Supplementary Fig. 120. Spatial variability of trends in depth to groundwater Gnangara Mound (within the broader Perth Basin). For detailed description of the contents of each panel see the text at the beginning of Supplementary Note 17.

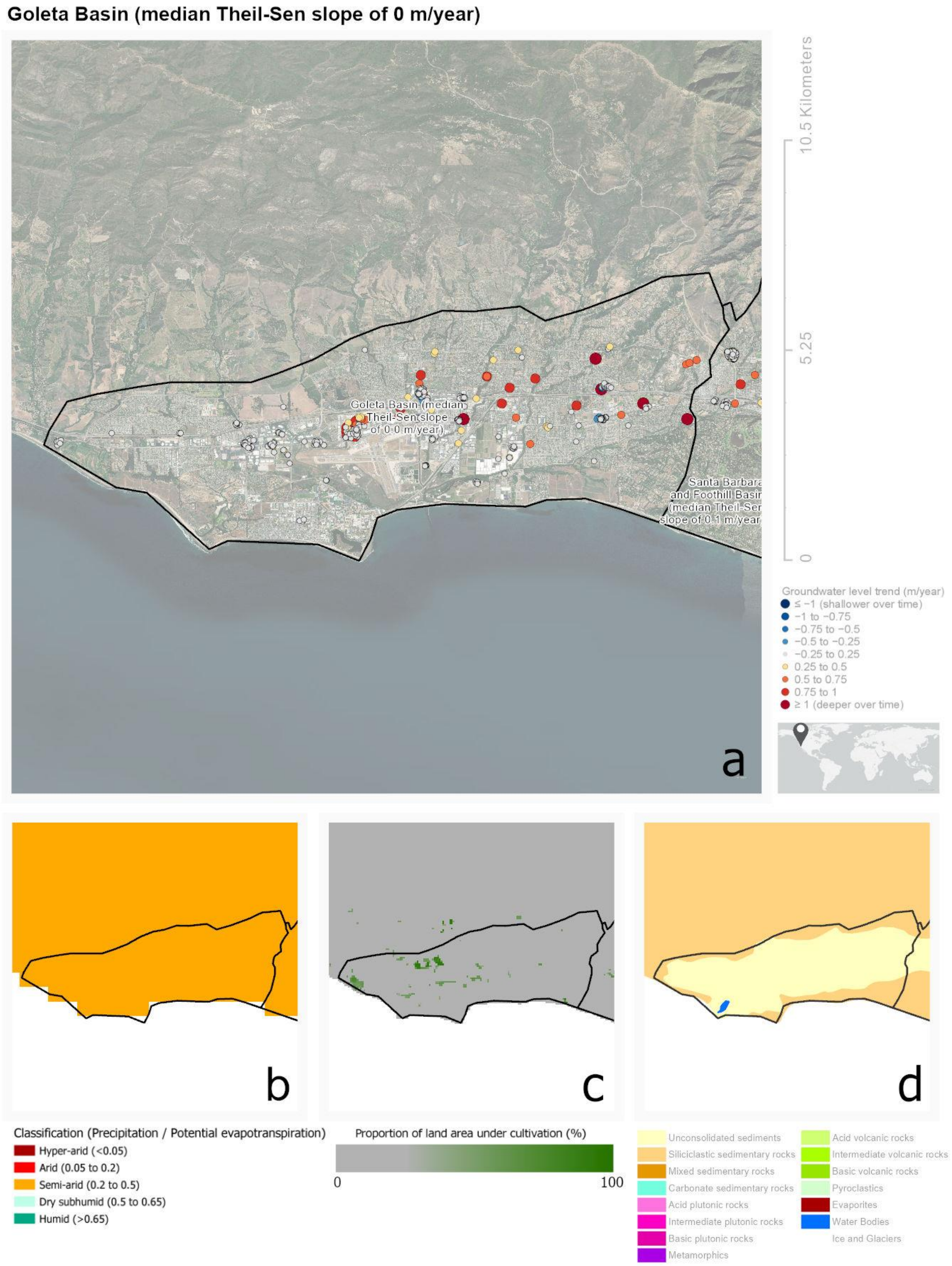

Supplementary Fig. 121. Spatial variability of trends in depth to groundwater Goleta Basin. For detailed description of the contents of each panel see the text at the beginning of Supplementary Note 17.

Gulgir Plain (median Theil-Sen slope of 0.4 m/year)

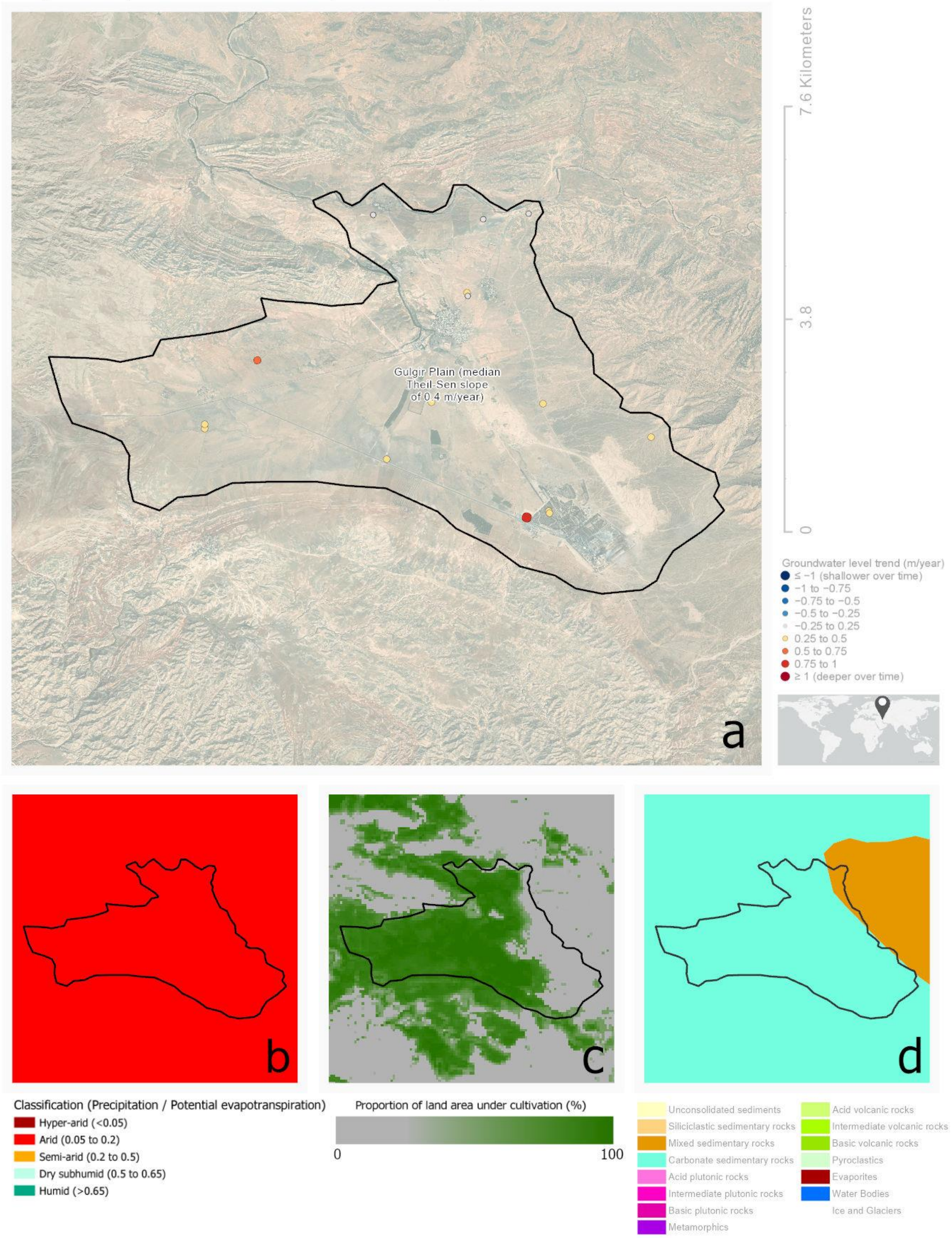

Supplementary Fig. 122. Spatial variability of trends in depth to groundwater Gulgir Plain. For detailed description of the contents of each panel see the text at the beginning of Supplementary Note 17.

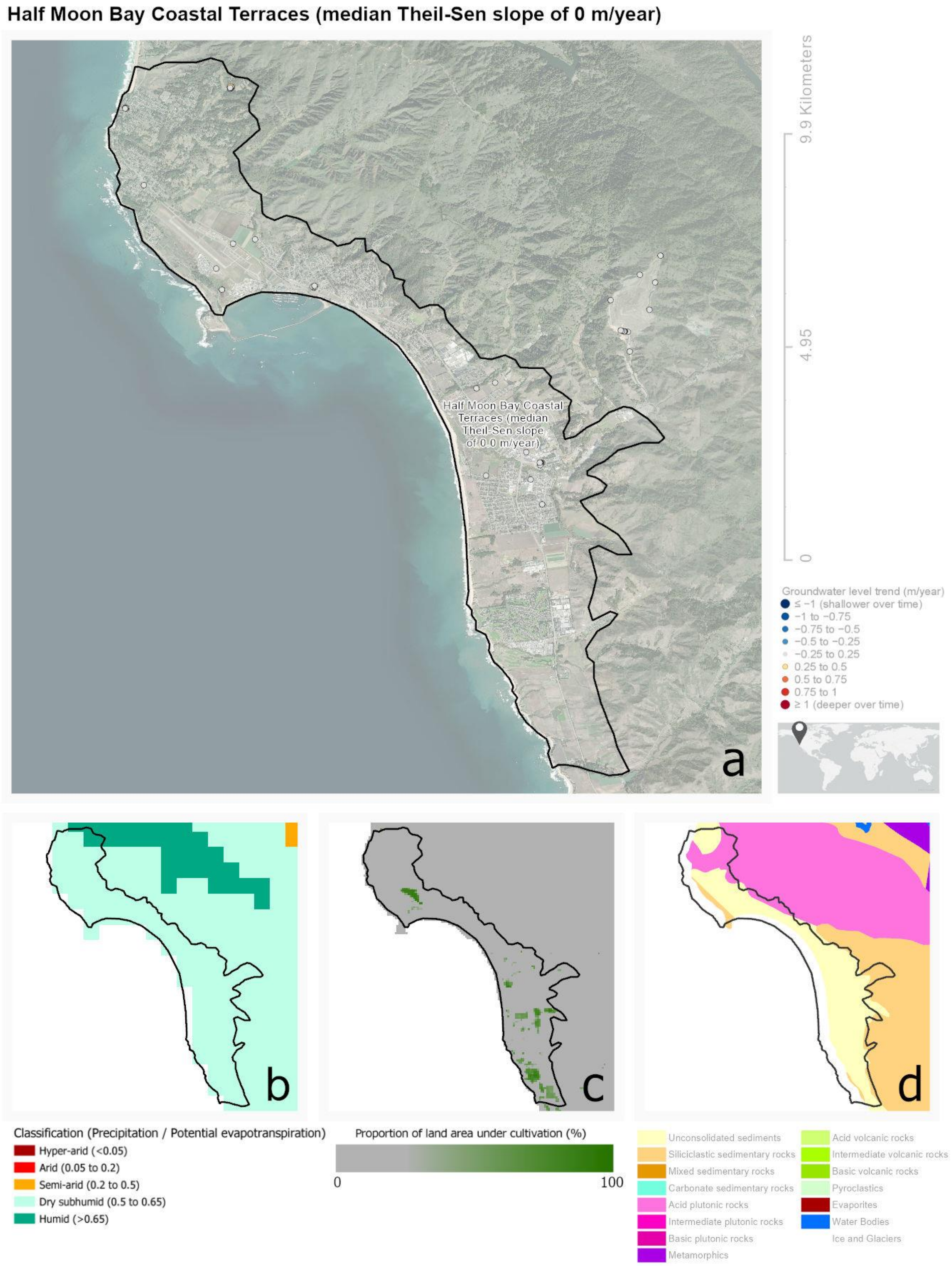

Supplementary Fig. 123. Spatial variability of trends in depth to groundwater Half Moon Bay Coastal Terraces. For detailed description of the contents of each panel see the text at the beginning of Supplementary Note 17.

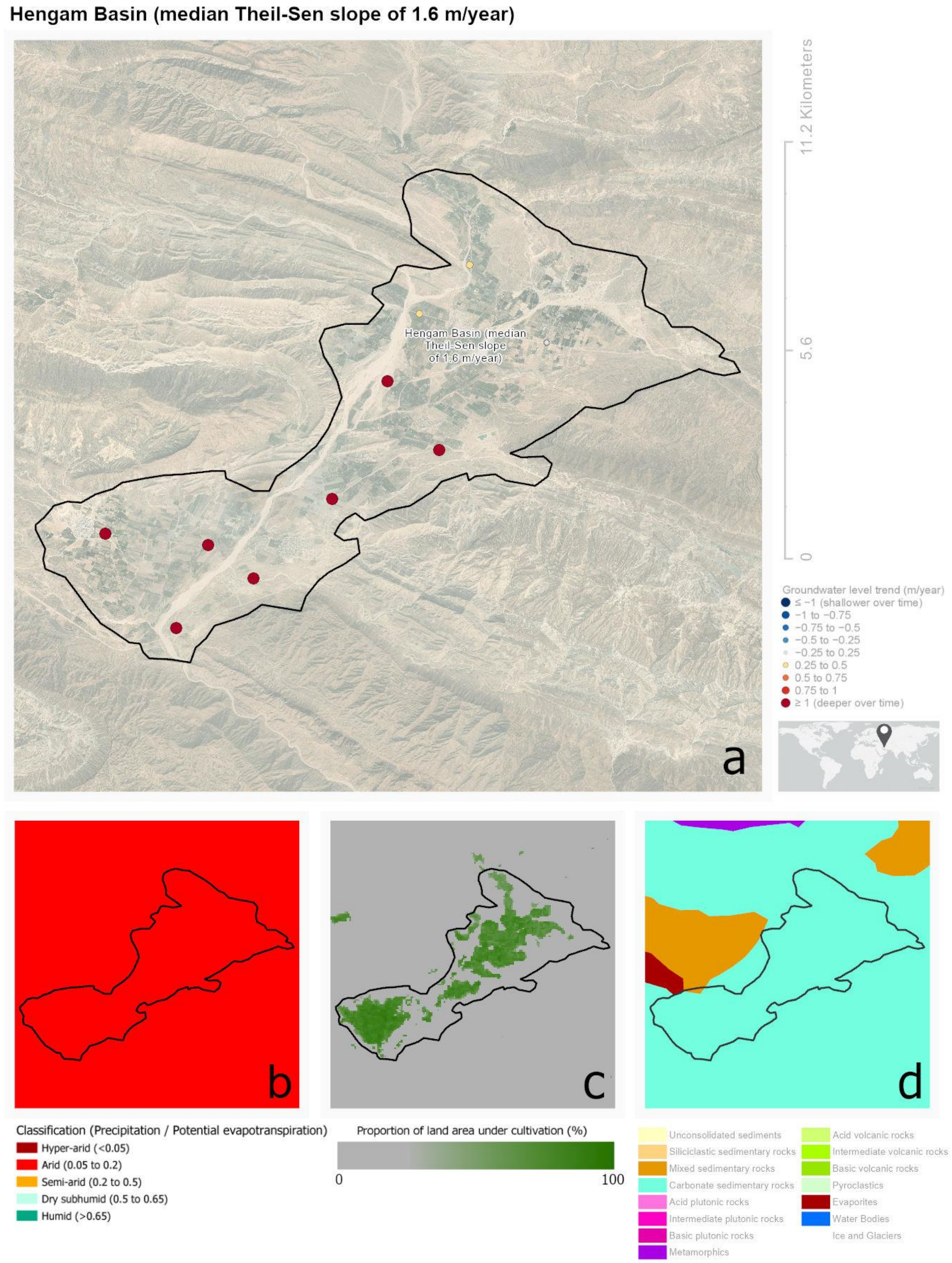

Supplementary Fig. 124. Spatial variability of trends in depth to groundwater Hengam Basin. For detailed description of the contents of each panel see the text at the beginning of Supplementary Note 17.

Hollywood Basin (median Theil-Sen slope of 0 m/year)

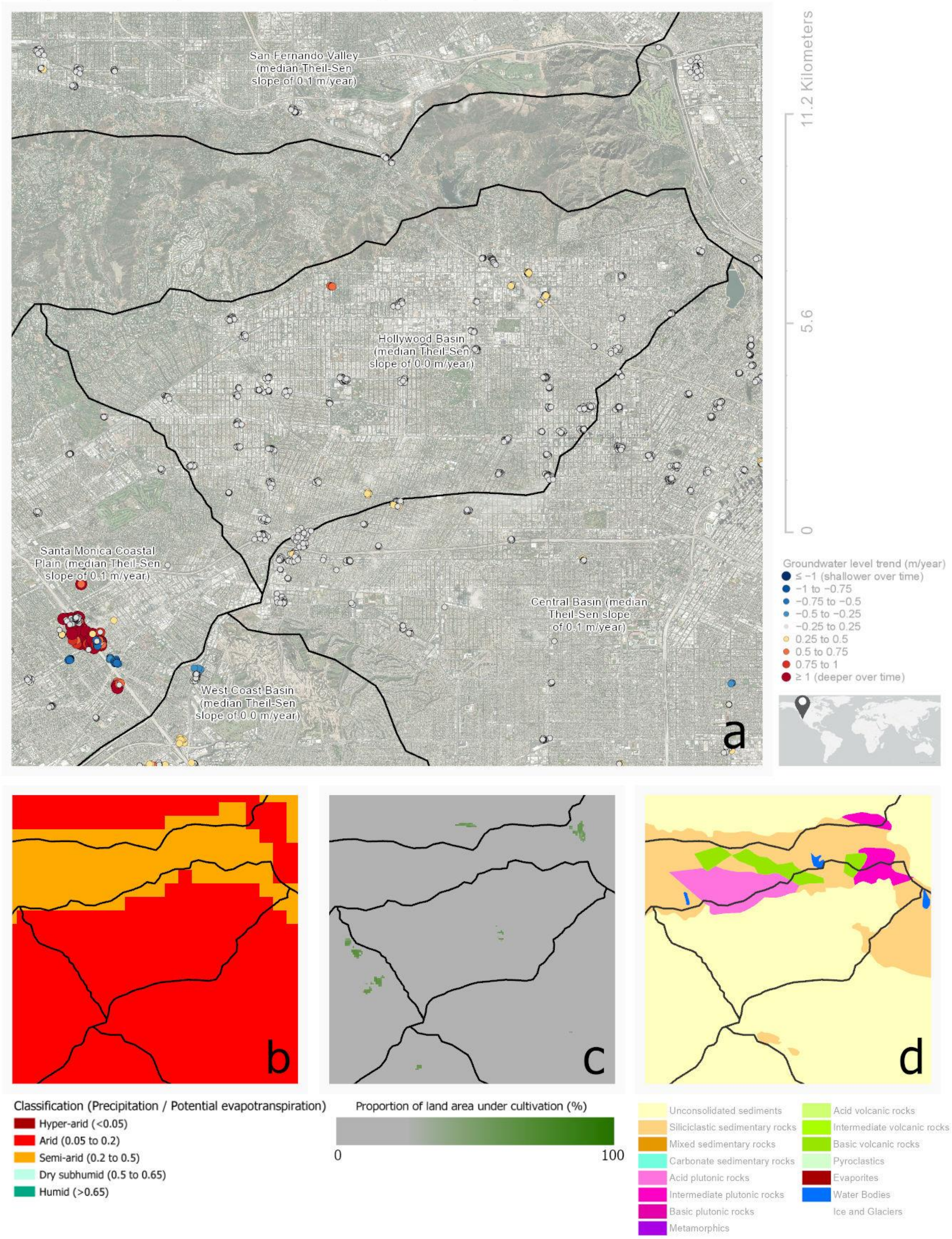

Supplementary Fig. 125. Spatial variability of trends in depth to groundwater Hollywood Basin (within the broader Los Angeles Basin). For detailed description of the contents of each panel see the text at the beginning of Supplementary Note 17.

Houlong Alluvial Plain Aquifer (median Theil-Sen slope of 0 m/year)

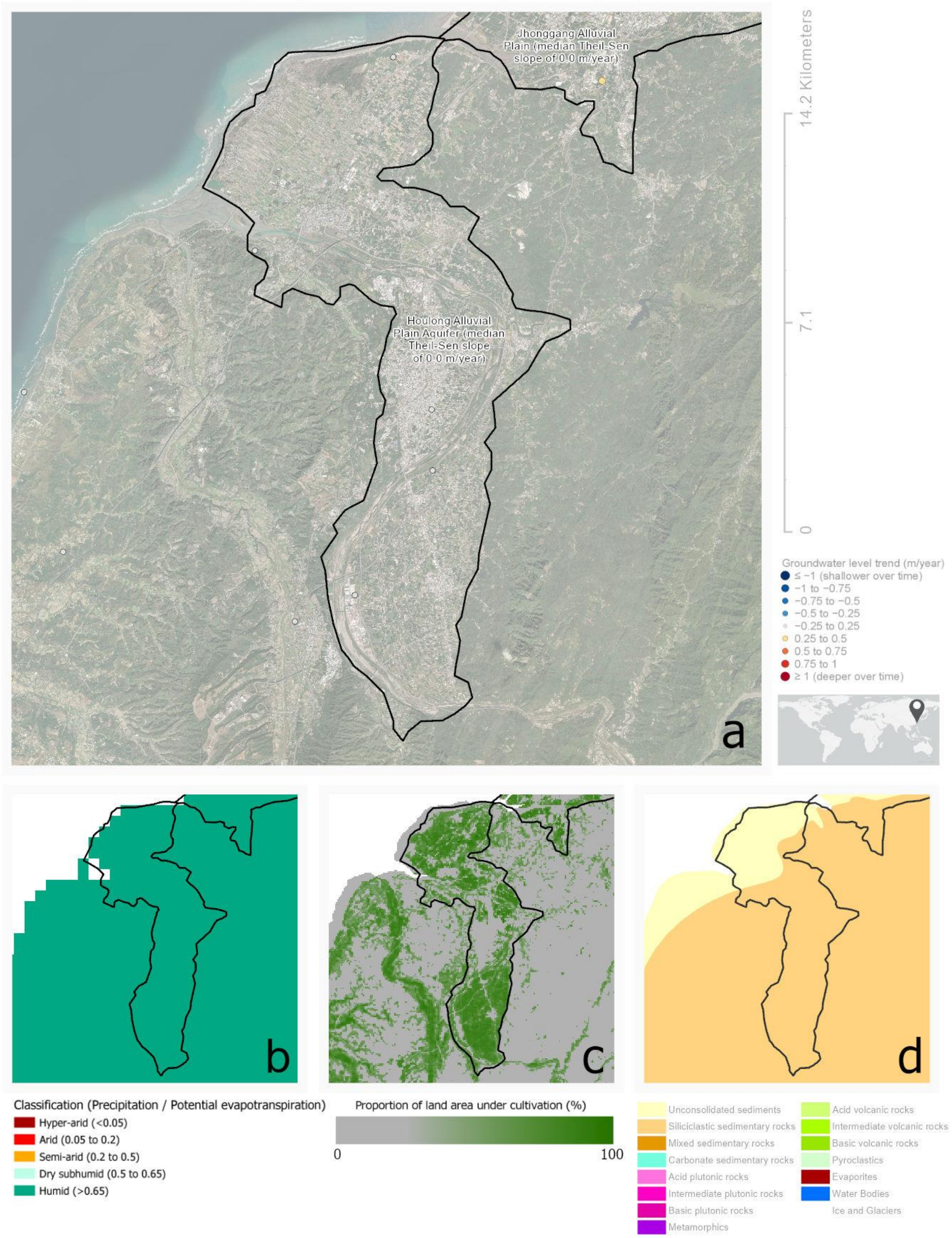

Supplementary Fig. 126. Spatial variability of trends in depth to groundwater Houlong Alluvial Plain Aquifer (within the broader Hsinchu-Miaoli Coastal Aquifer System). For detailed description of the contents of each panel see the text at the beginning of Supplementary Note 17.

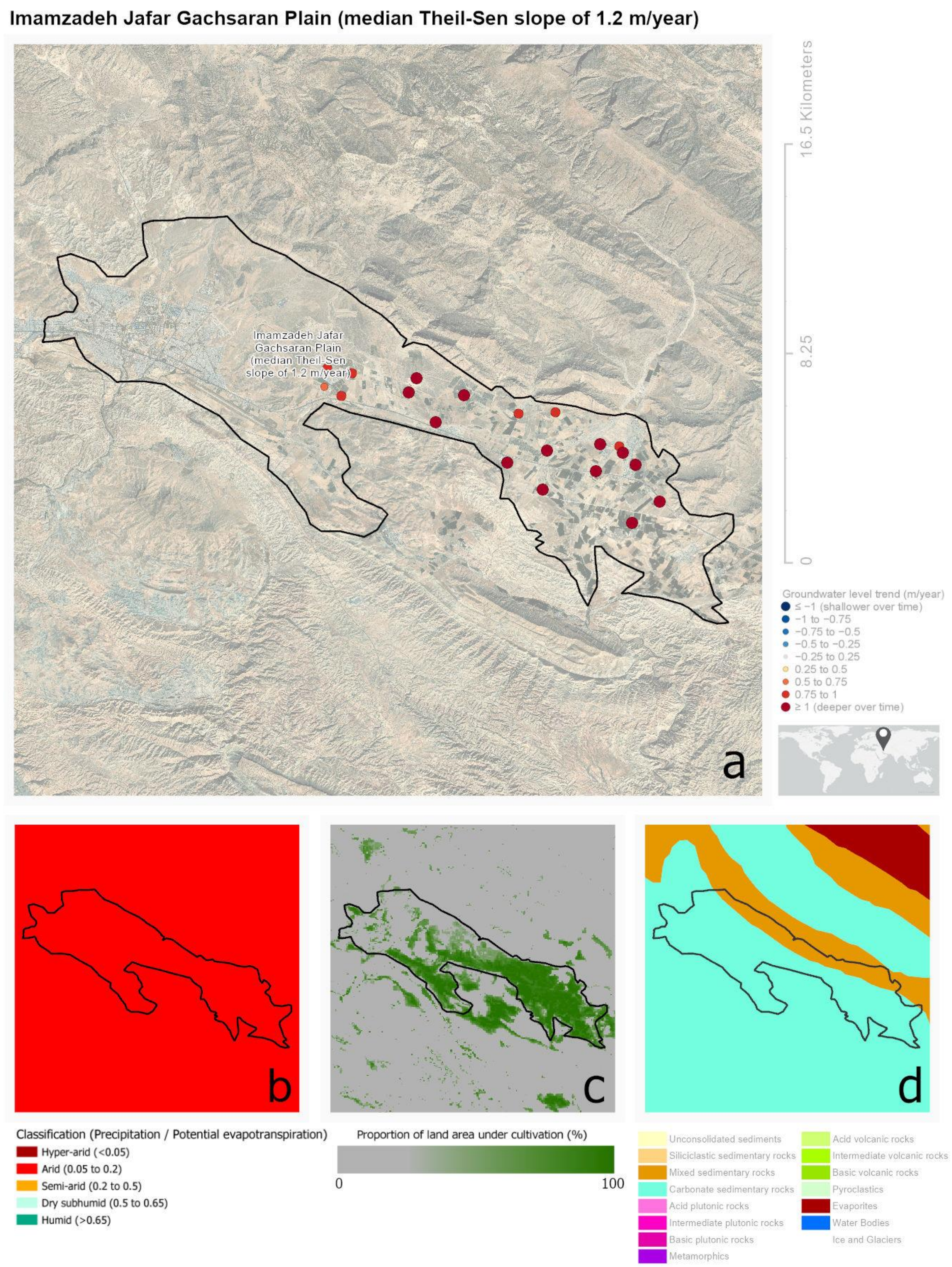

Supplementary Fig. 127. Spatial variability of trends in depth to groundwater Imamzadeh Jafar Gachsaran Plain. For detailed description of the contents of each panel see the text at the beginning of Supplementary Note 17.

Irwin Basin (median Theil-Sen slope of -0.3 m/year)

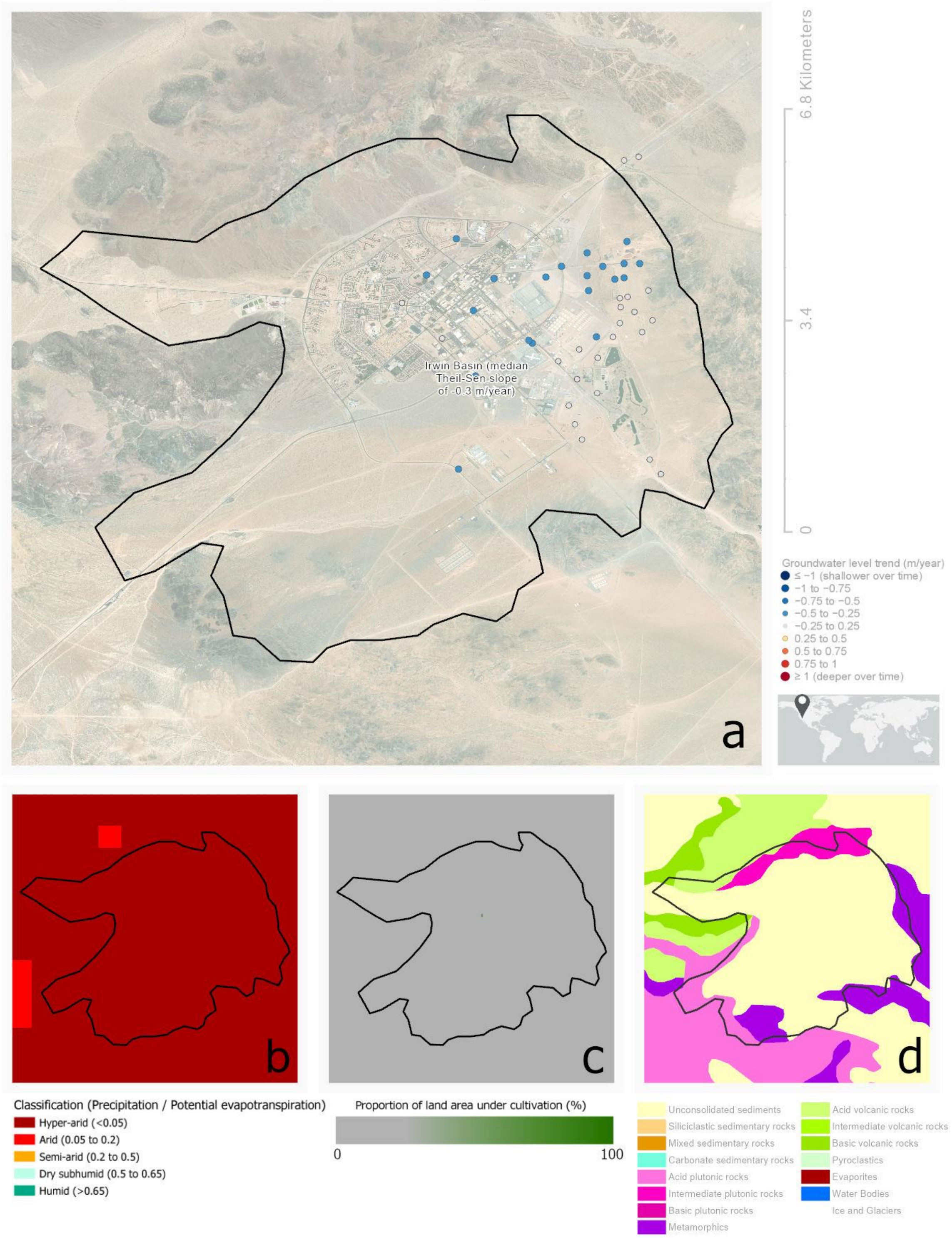

Supplementary Fig. 128. Spatial variability of trends in depth to groundwater Irwin Basin. For detailed description of the contents of each panel see the text at the beginning of Supplementary Note 17.

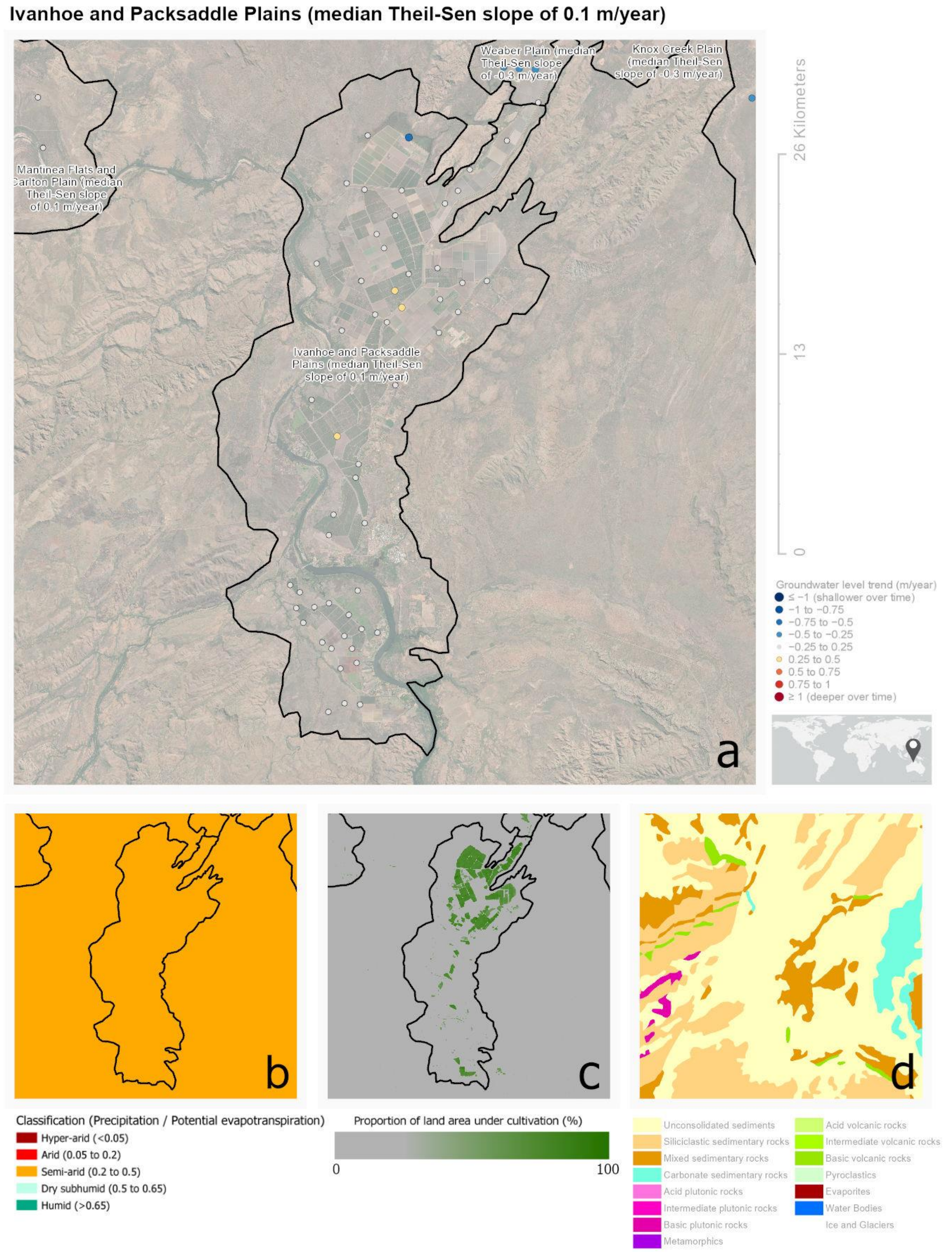

Supplementary Fig. 129. Spatial variability of trends in depth to groundwater Ivanhoe and Packsaddle Plains. For detailed description of the contents of each panel see the text at the beginning of Supplementary Note 17.

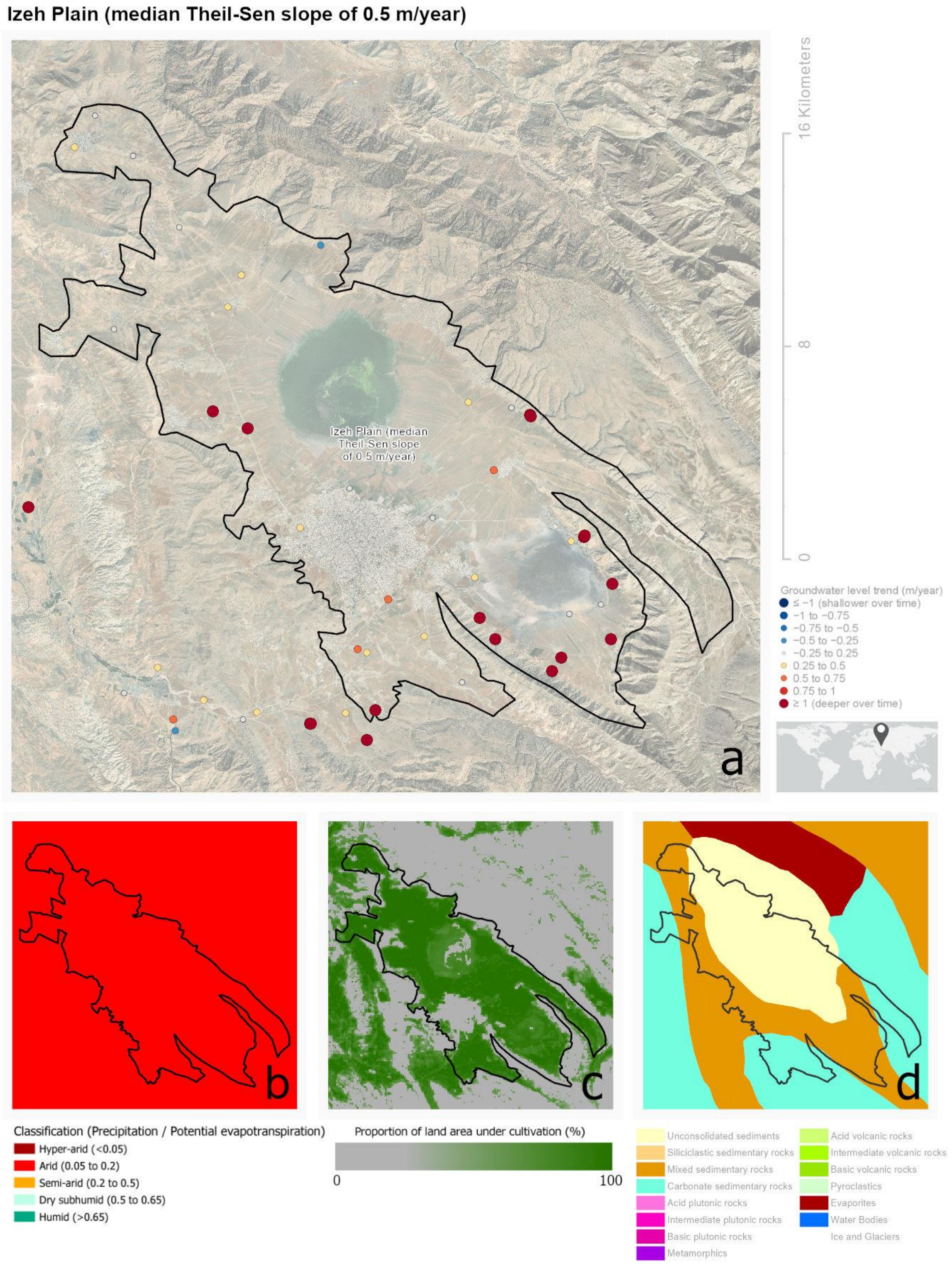

Supplementary Fig. 130. Spatial variability of trends in depth to groundwater Izeh Plain. For detailed description of the contents of each panel see the text at the beginning of Supplementary Note 17.

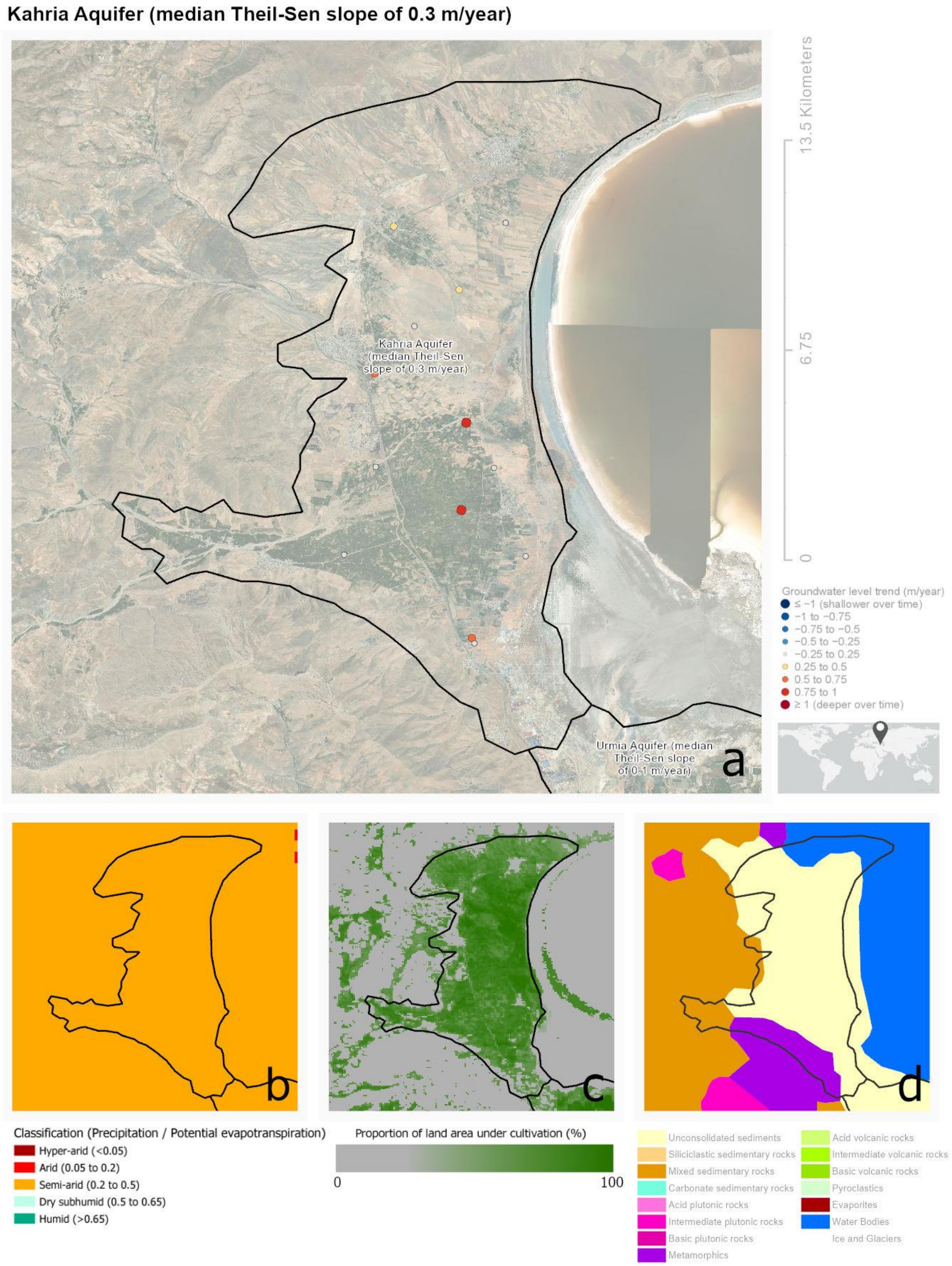

Supplementary Fig. 131. Spatial variability of trends in depth to groundwater Kahria Aquifer. For detailed description of the contents of each panel see the text at the beginning of Supplementary Note 17.

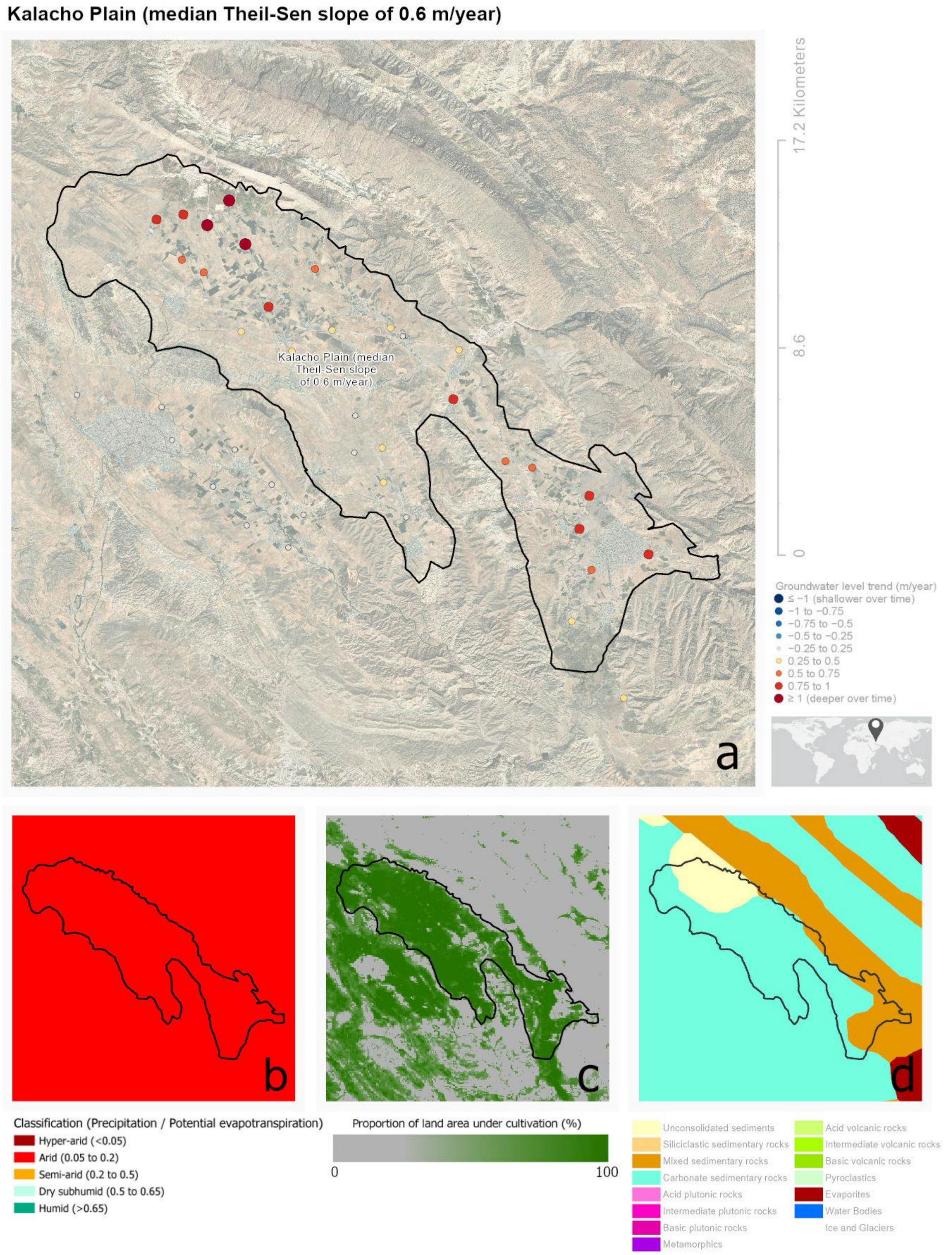

Supplementary Fig. 132. Spatial variability of trends in depth to groundwater Kalacho Plain. For detailed description of the contents of each panel see the text at the beginning of Supplementary Note 17.

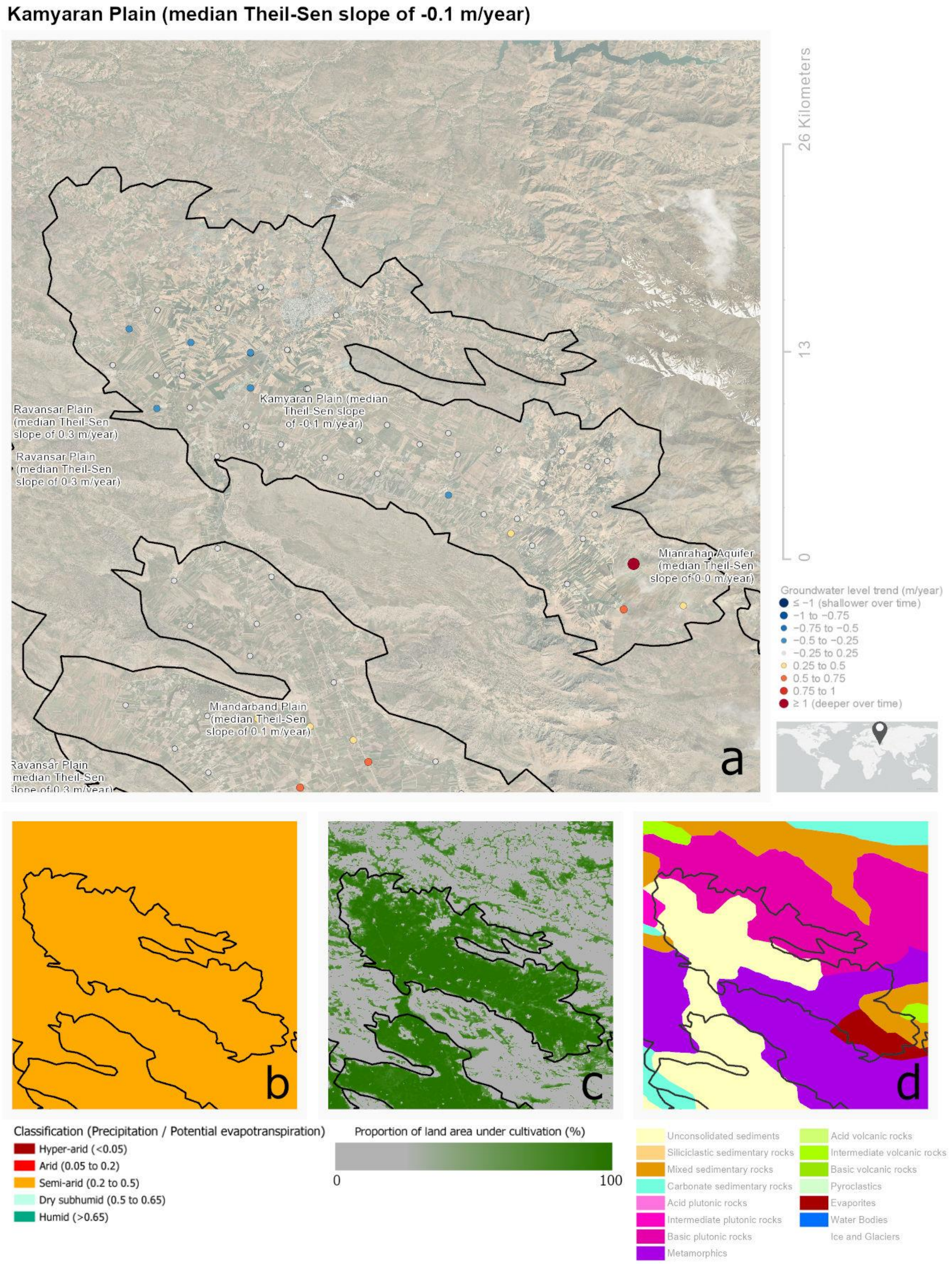

Supplementary Fig. 133. Spatial variability of trends in depth to groundwater Kamyaran Plain. For detailed description of the contents of each panel see the text at the beginning of Supplementary Note 17.

Kaweah Basin (median Theil-Sen slope of 0.9 m/year)

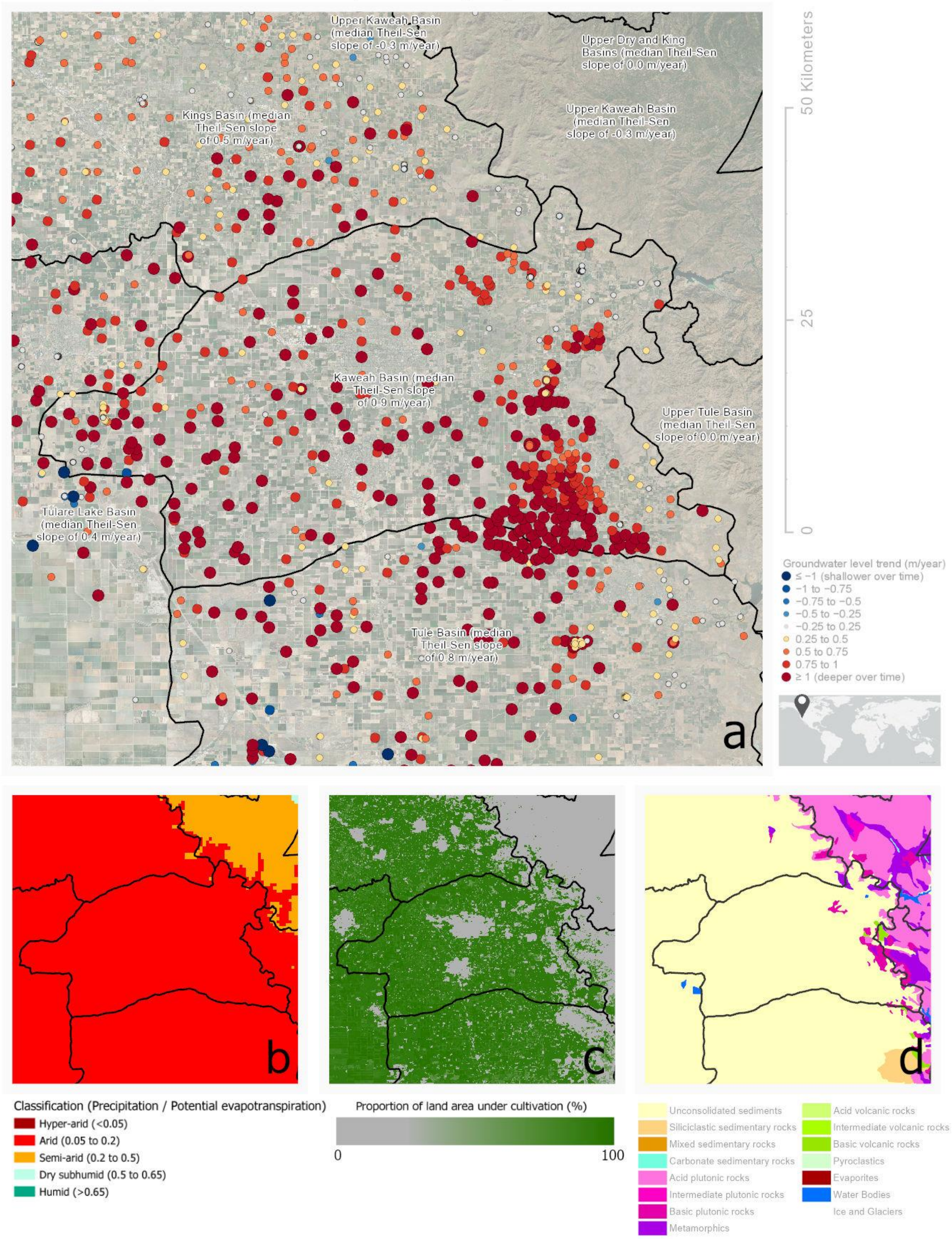

Supplementary Fig. 134. Spatial variability of trends in depth to groundwater Kaweah Basin (within the broader Tulare Basin of the Central Valley). For detailed description of the contents of each panel see the text at the beginning of Supplementary Note 17.

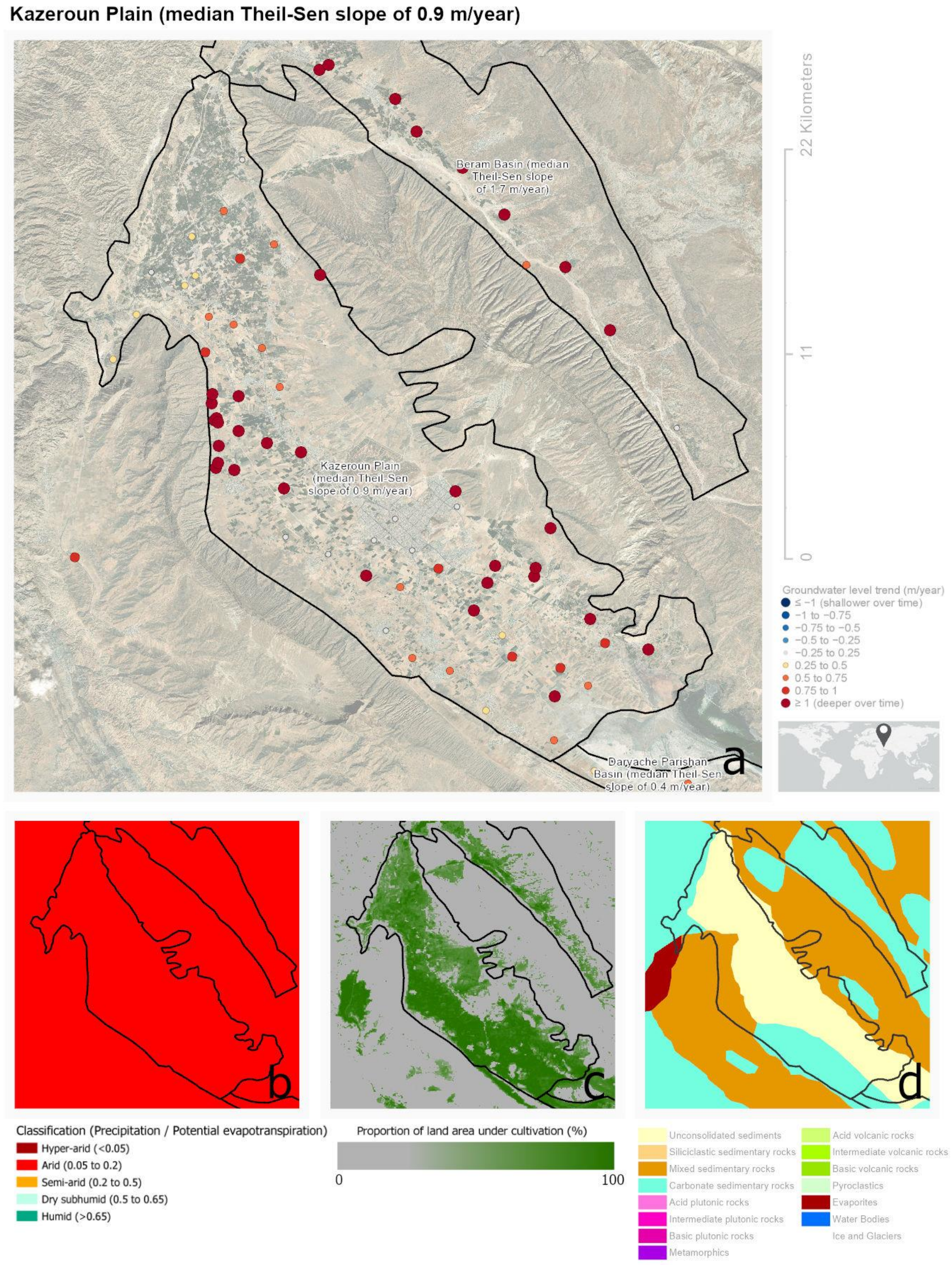

Supplementary Fig. 135. Spatial variability of trends in depth to groundwater Kazeroun Plain. For detailed description of the contents of each panel see the text at the beginning of Supplementary Note 17.

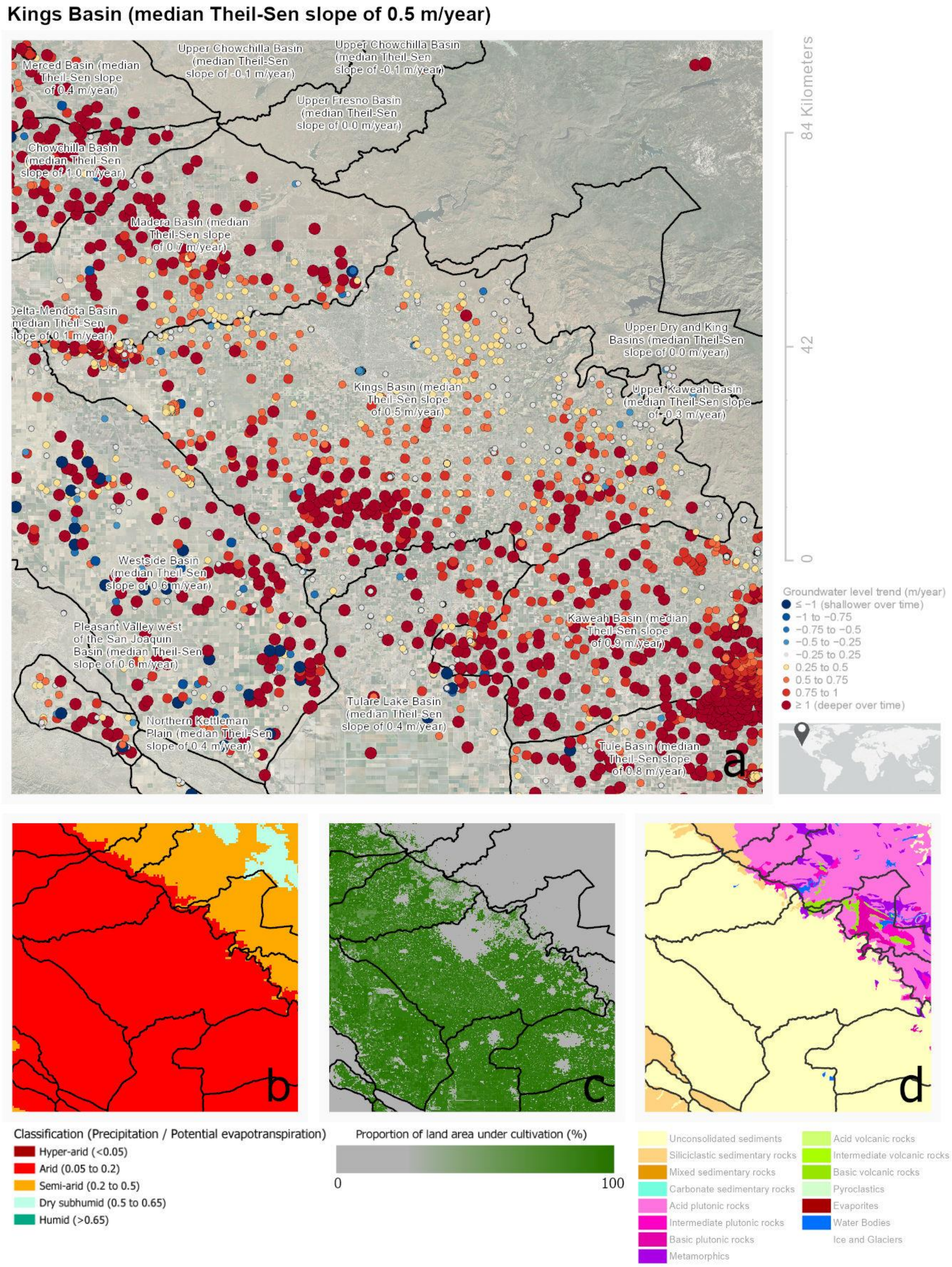

Supplementary Fig. 136. Spatial variability of trends in depth to groundwater Kings Basin (within the broader Tulare Basin of the Central Valley). For detailed description of the contents of each panel see the text at the beginning of Supplementary Note 17.

La Paz Coastal Aquifer (median Theil-Sen slope of 0.1 m/year)

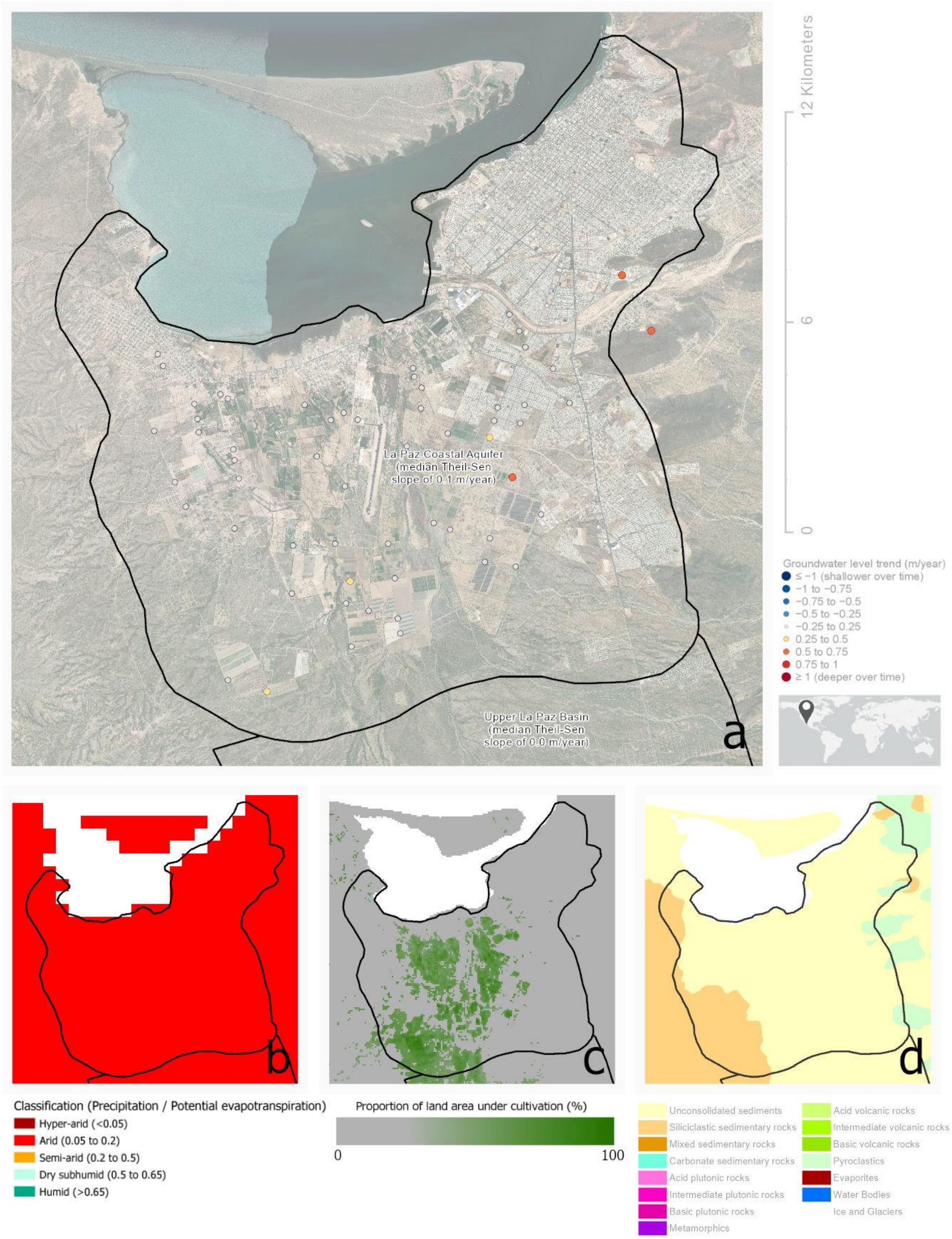

Supplementary Fig. 137. Spatial variability of trends in depth to groundwater La Paz Coastal Aquifer (within the broader La Paz Basin). For detailed description of the contents of each panel see the text at the beginning of Supplementary Note 17.

Laidley Sub-basin (median Theil-Sen slope of -0.1 m/year)

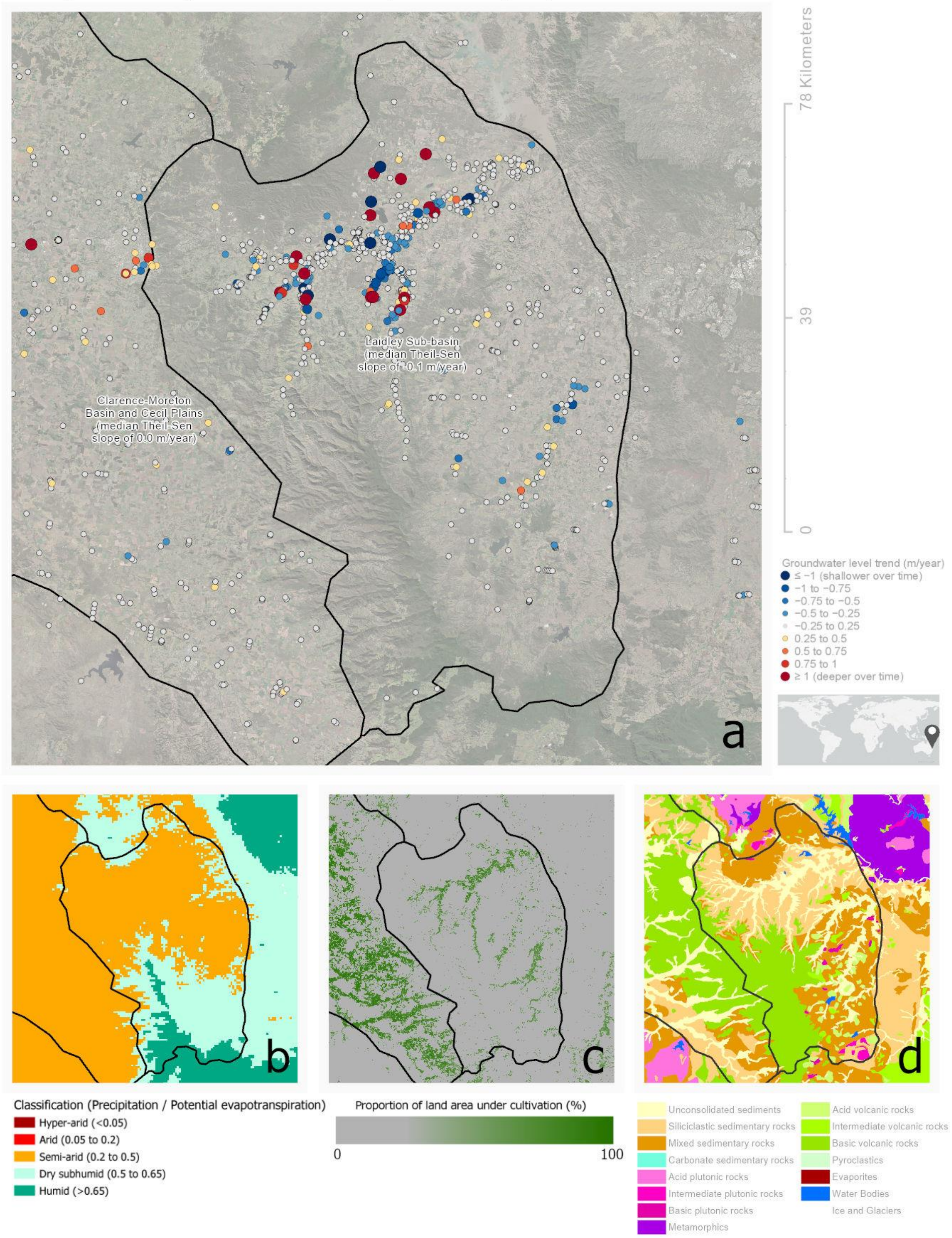

Supplementary Fig. 138. Spatial variability of trends in depth to groundwater Laidley Sub-basin (within the broader Great Artesian Basin). For detailed description of the contents of each panel see the text at the beginning of Supplementary Note 17.

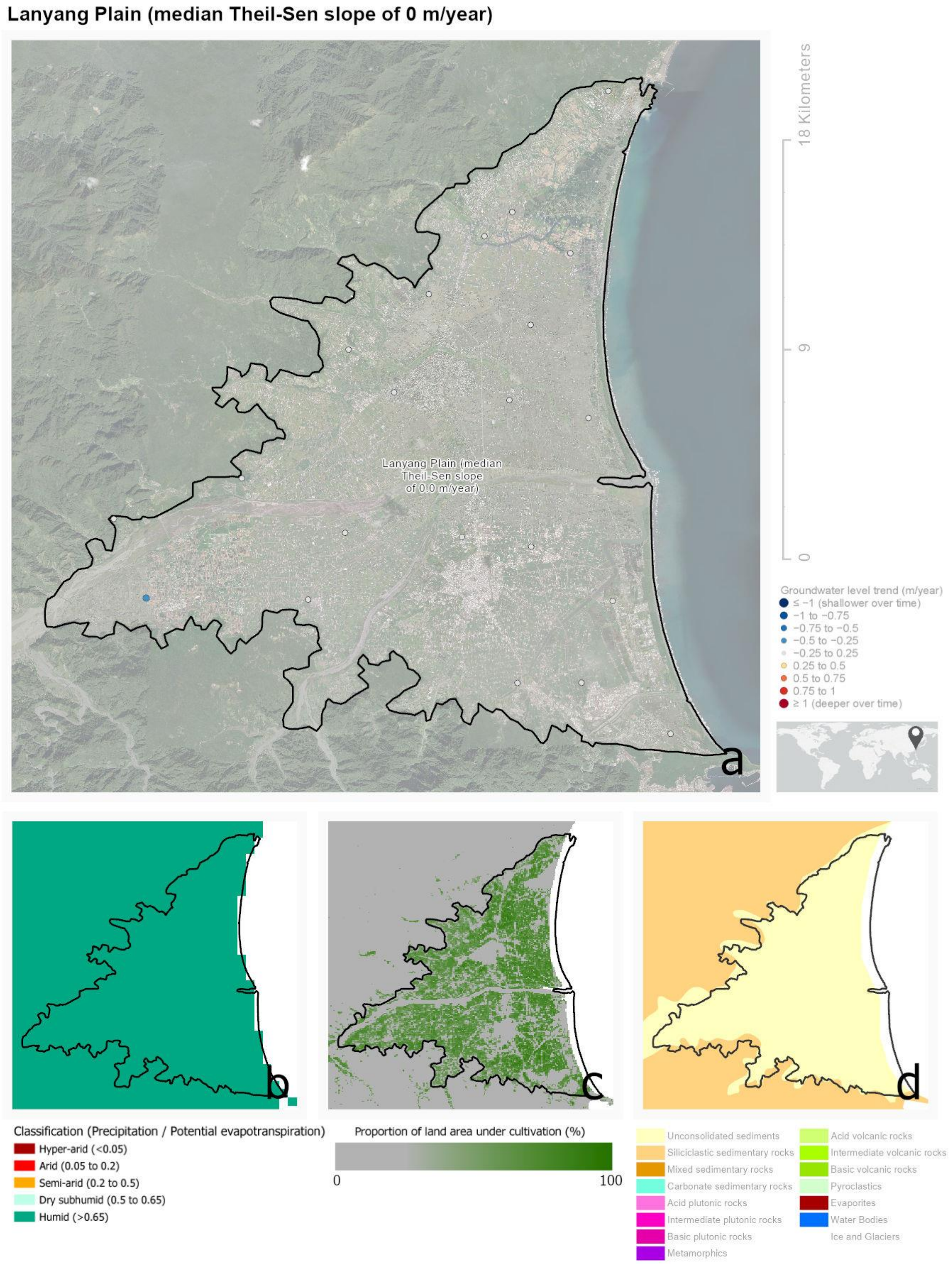

Supplementary Fig. 139. Spatial variability of trends in depth to groundwater Lanyang Plain. For detailed description of the contents of each panel see the text at the beginning of Supplementary Note 17.

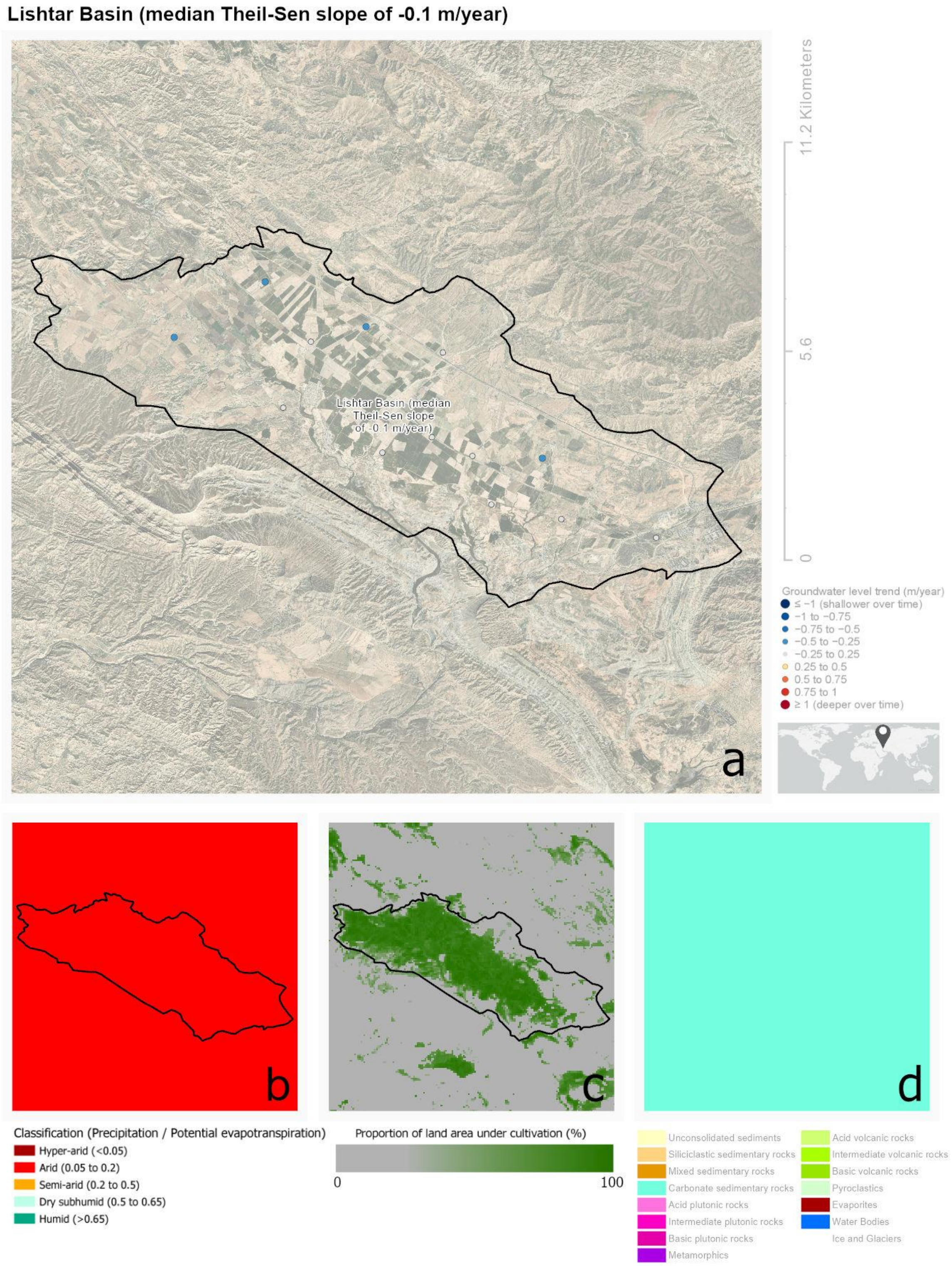

Supplementary Fig. 140. Spatial variability of trends in depth to groundwater Lishtar Basin. For detailed description of the contents of each panel see the text at the beginning of Supplementary Note 17.

Little Lake Valley (median Theil-Sen slope of 0 m/year)

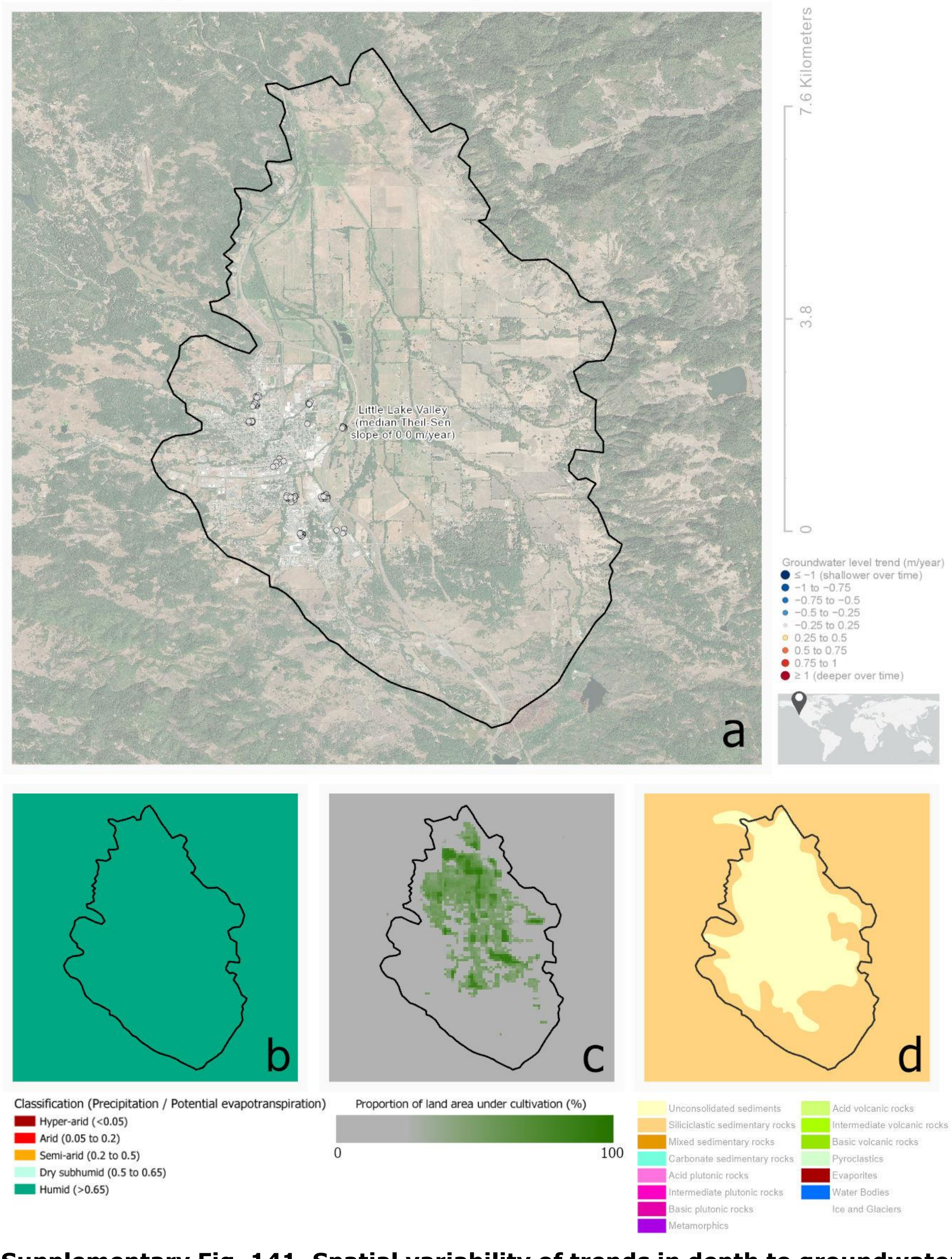

Supplementary Fig. 141. Spatial variability of trends in depth to groundwater Little Lake Valley. For detailed description of the contents of each panel see the text at the beginning of Supplementary Note 17.

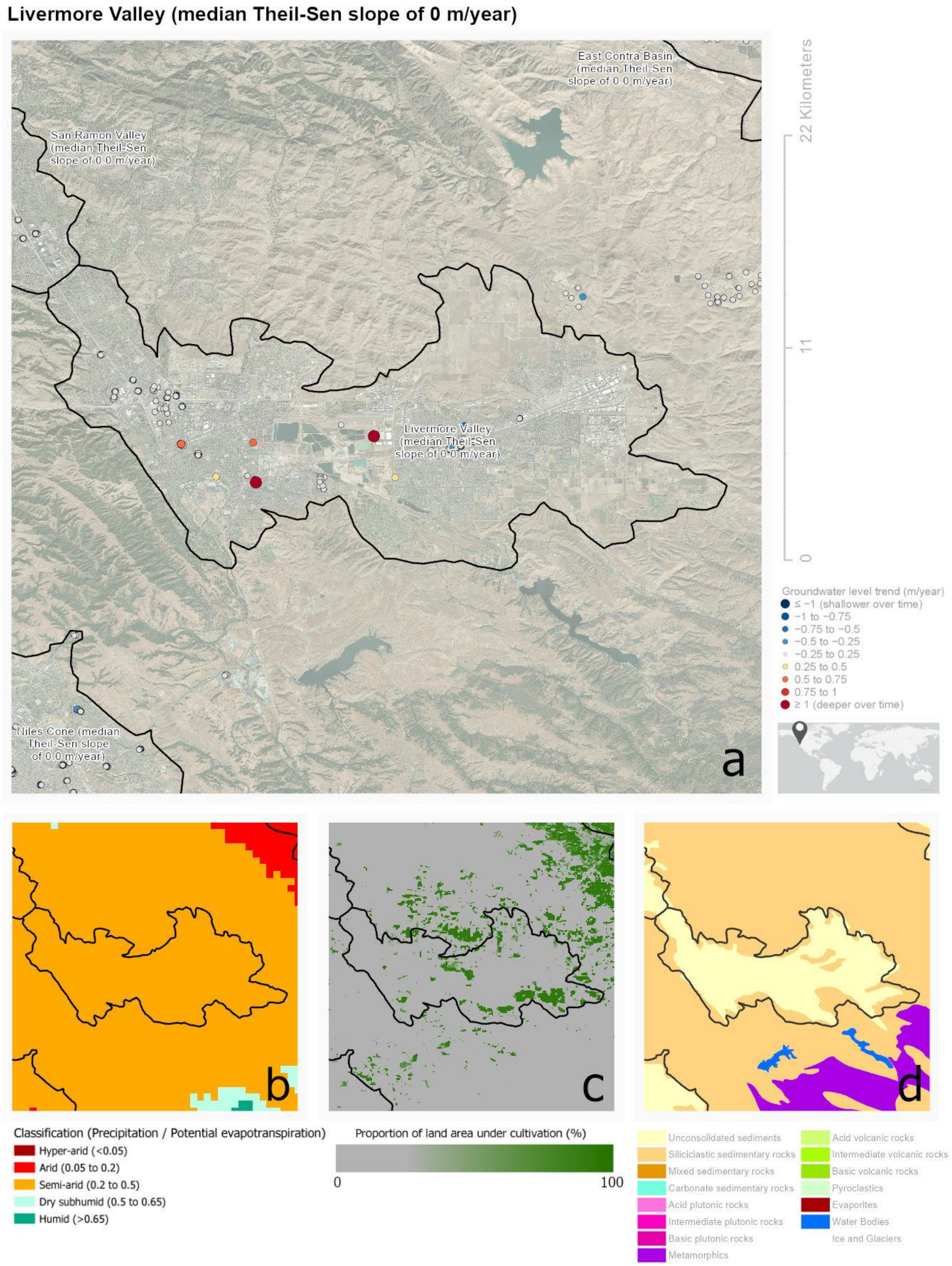

Supplementary Fig. 142. Spatial variability of trends in depth to groundwater Livermore Valley. For detailed description of the contents of each panel see the text at the beginning of Supplementary Note 17.

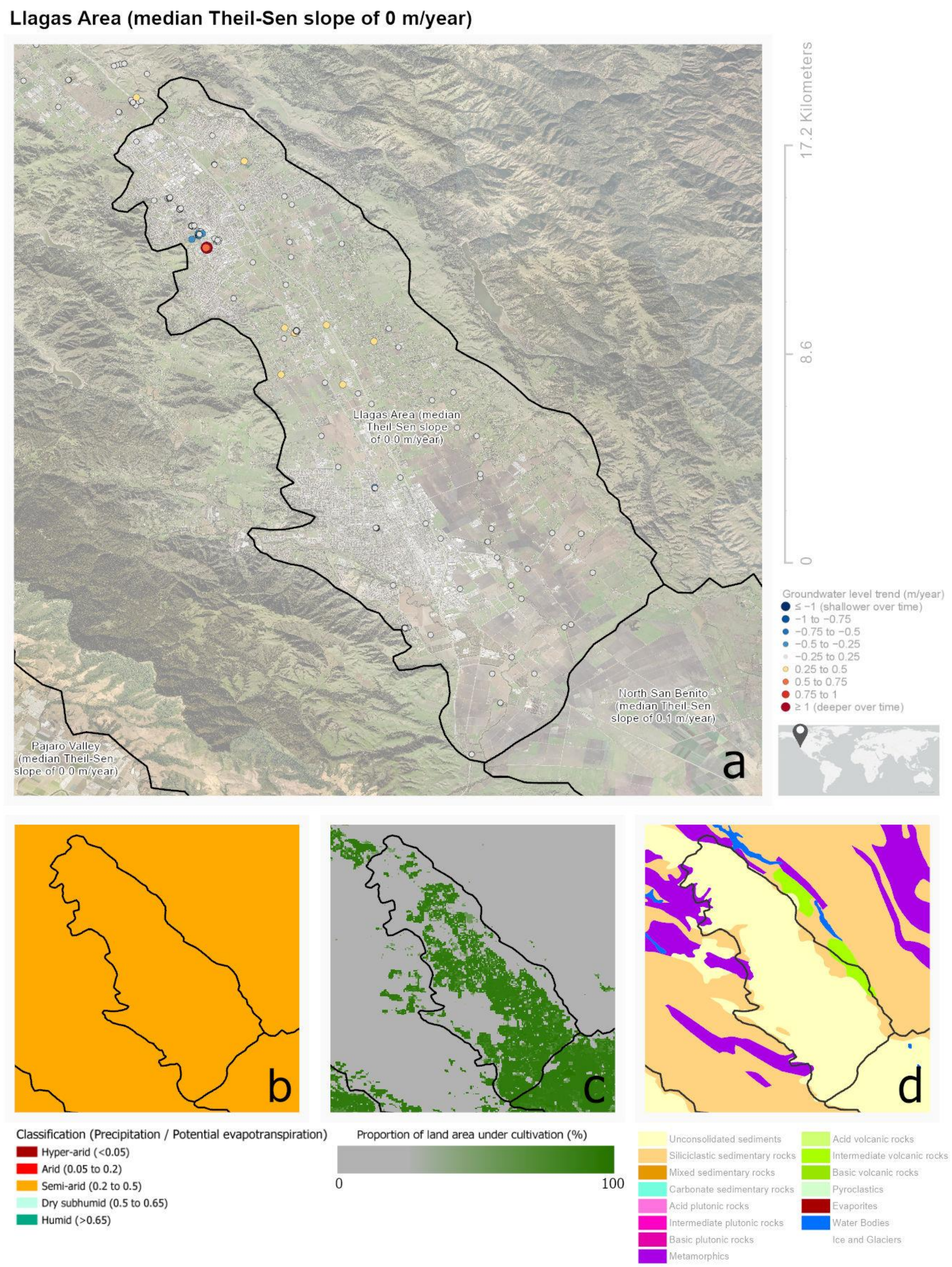

Supplementary Fig. 143. Spatial variability of trends in depth to groundwater Llagas Area (within the broader Gilroy-Hollister Valley). For detailed description of the contents of each panel see the text at the beginning of Supplementary Note 17.

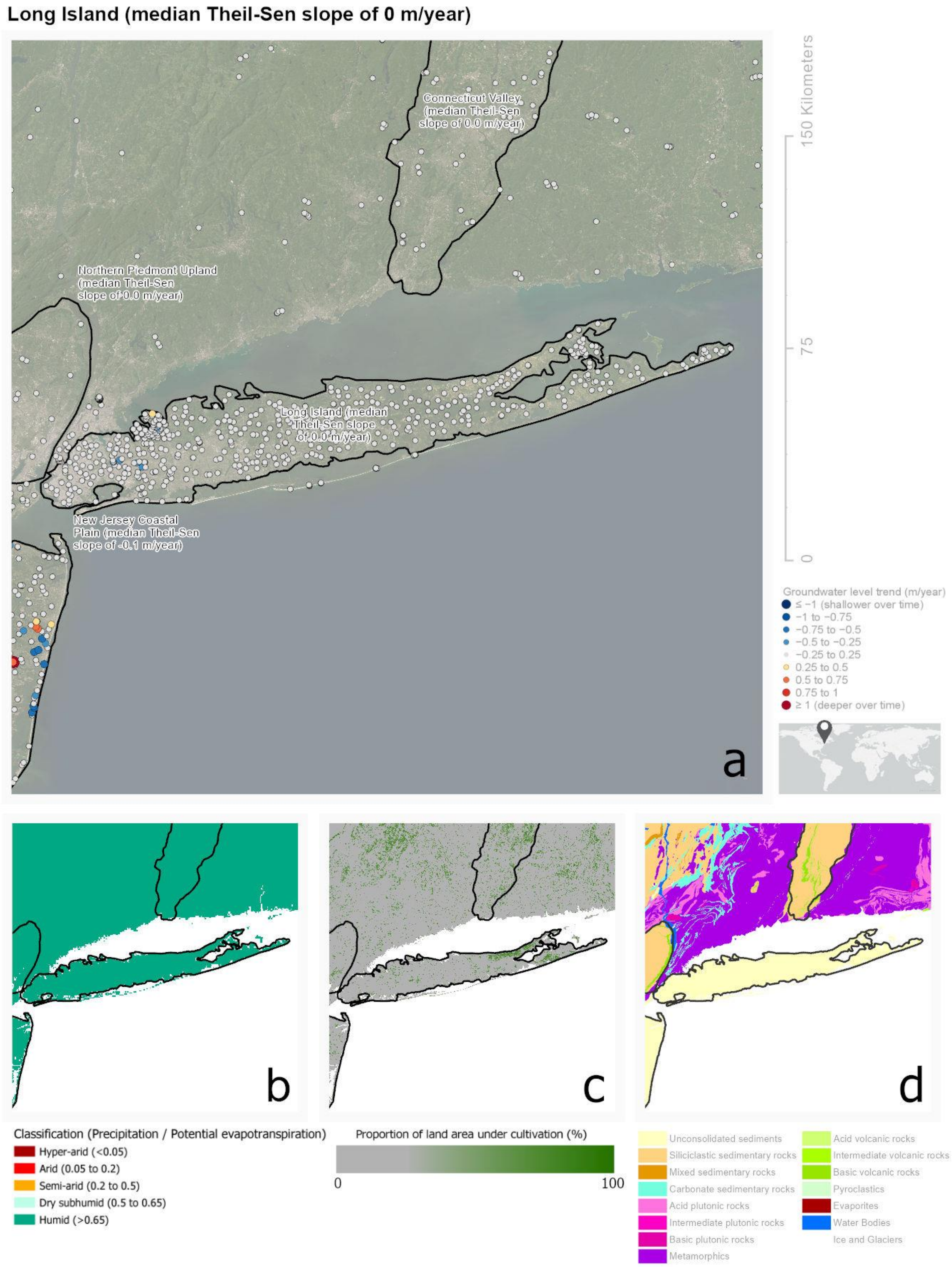

Supplementary Fig. 144. Spatial variability of trends in depth to groundwater Long Island. For detailed description of the contents of each panel see the text at the beginning of Supplementary Note 17.

Long Valley in eastern California (median Theil-Sen slope of 0.1 m/year)

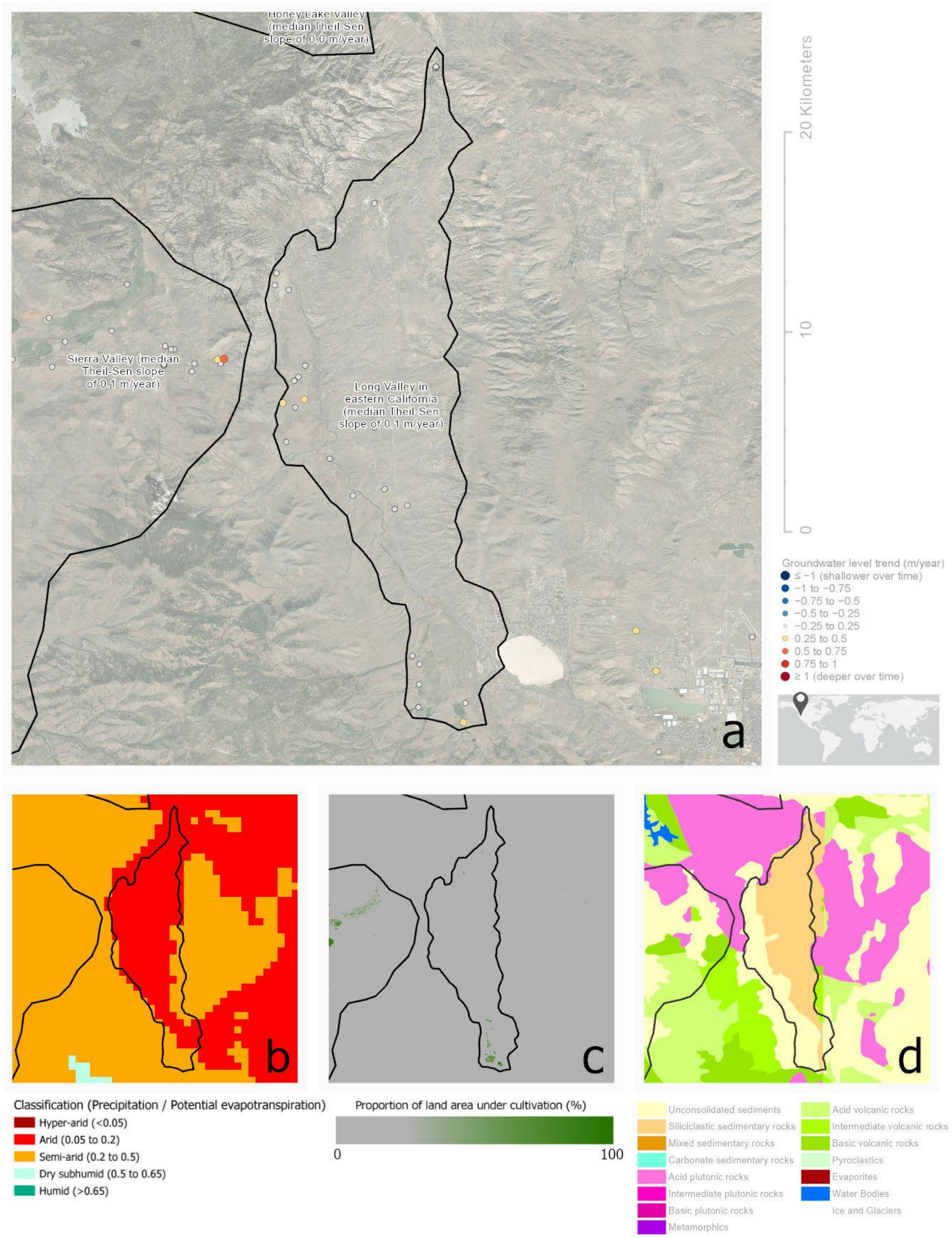

Supplementary Fig. 145. Spatial variability of trends in depth to groundwater Long Valley in eastern California. For detailed description of the contents of each panel see the text at the beginning of Supplementary Note 17.

Los Molinos Basin (median Theil-Sen slope of 0.1 m/year)

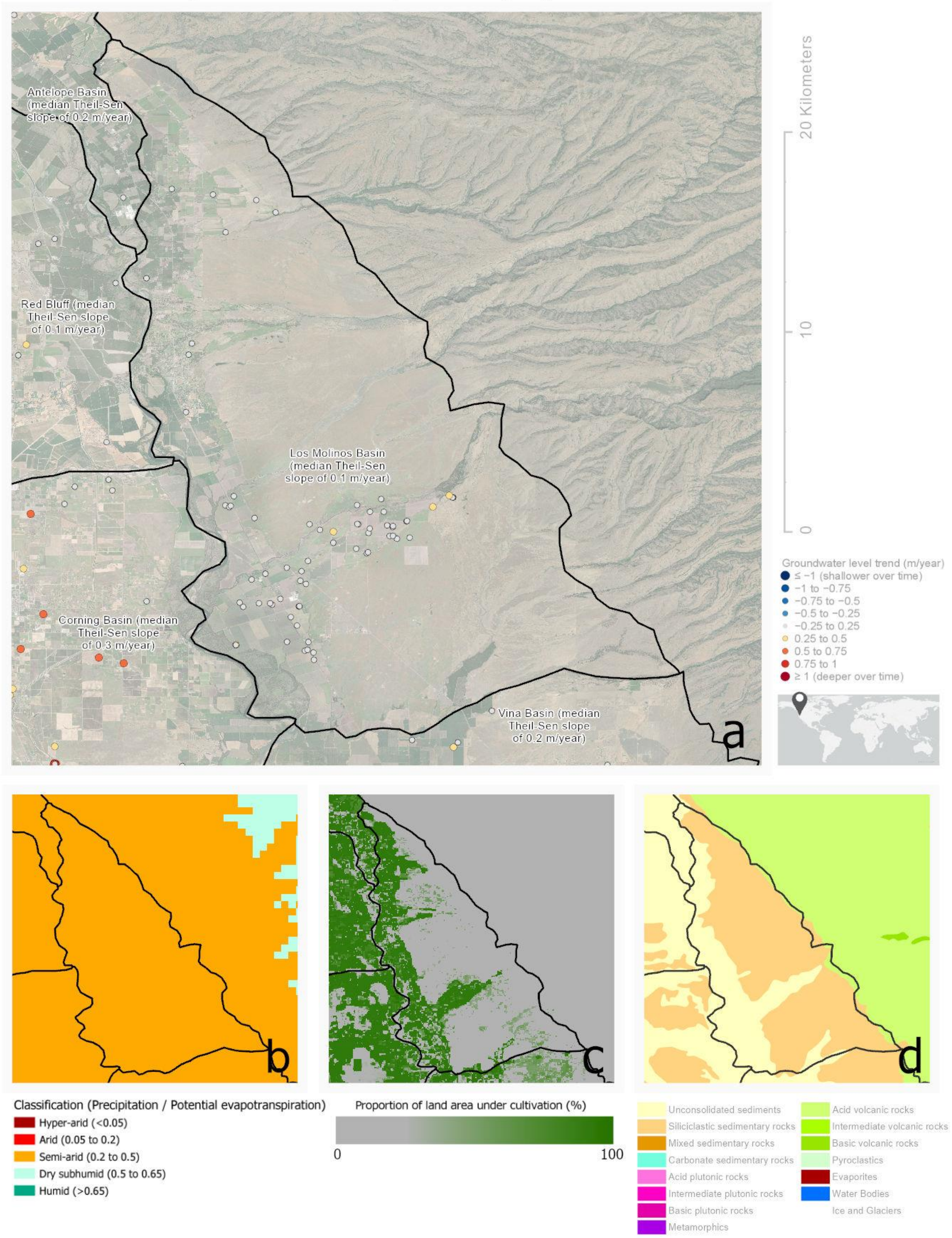

Supplementary Fig. 146. Spatial variability of trends in depth to groundwater Los Molinos Basin (within the broader Sacramento Valley of the Central Valley). For detailed description of the contents of each panel see the text at the beginning of Supplementary Note 17.

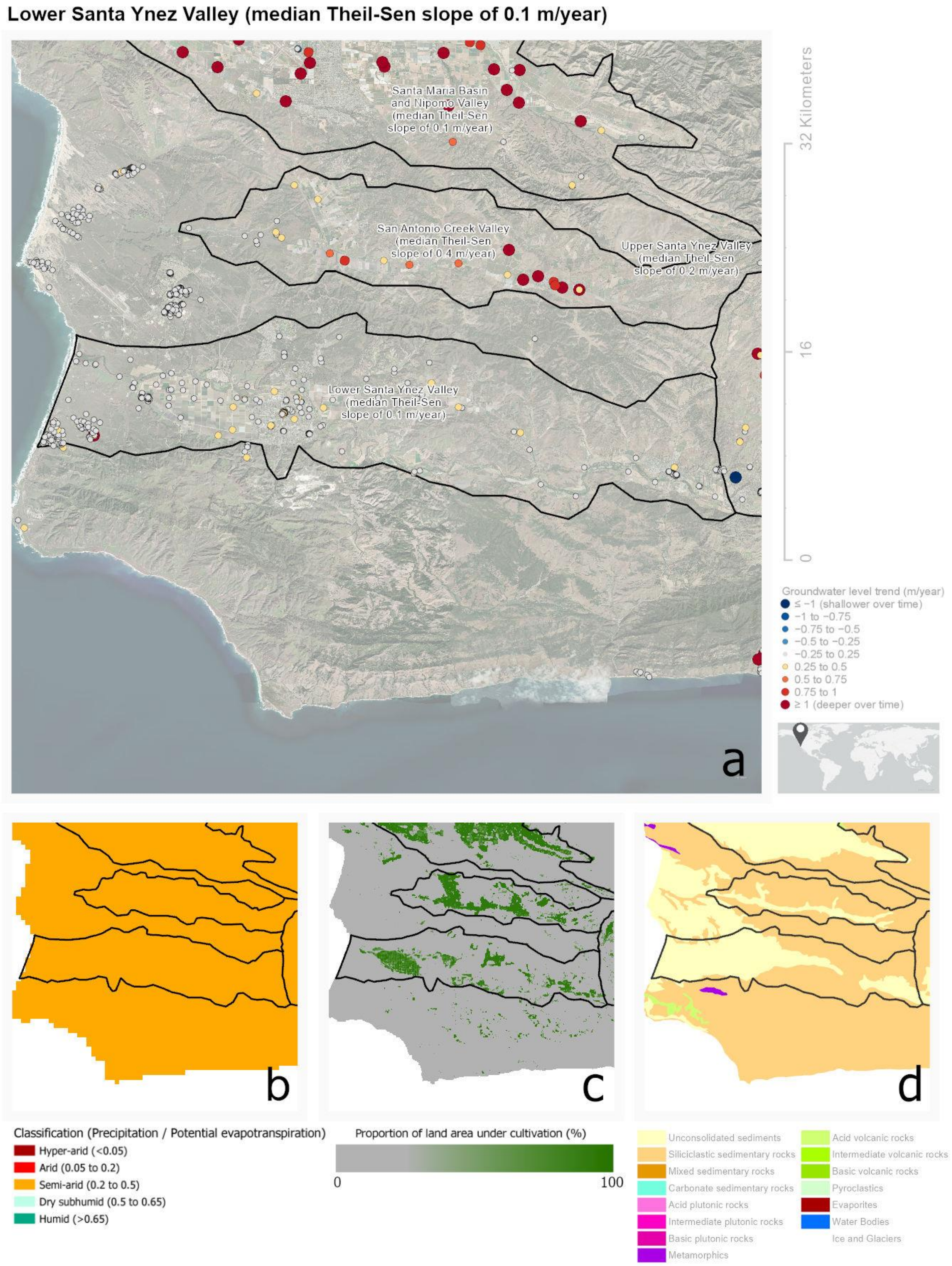

Supplementary Fig. 147. Spatial variability of trends in depth to groundwater Lower Santa Ynez Valley (within the broader Santa Ynez Valley). For detailed description of the contents of each panel see the text at the beginning of Supplementary Note 17.

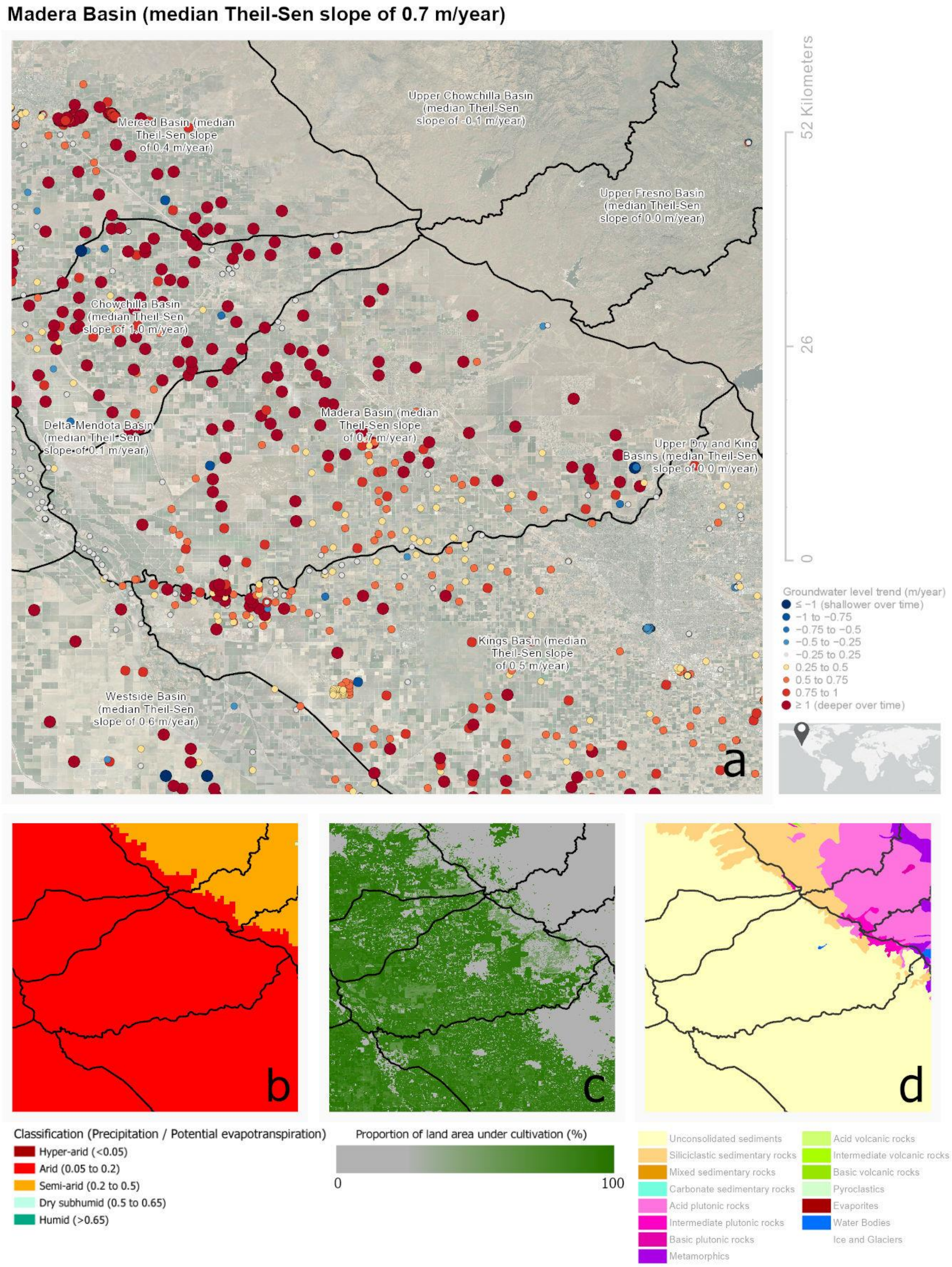

Supplementary Fig. 148. Spatial variability of trends in depth to groundwater Madera Basin (within the broader San Joaquin Basin of the Central Valley). For detailed description of the contents of each panel see the text at the beginning of Supplementary Note 17.

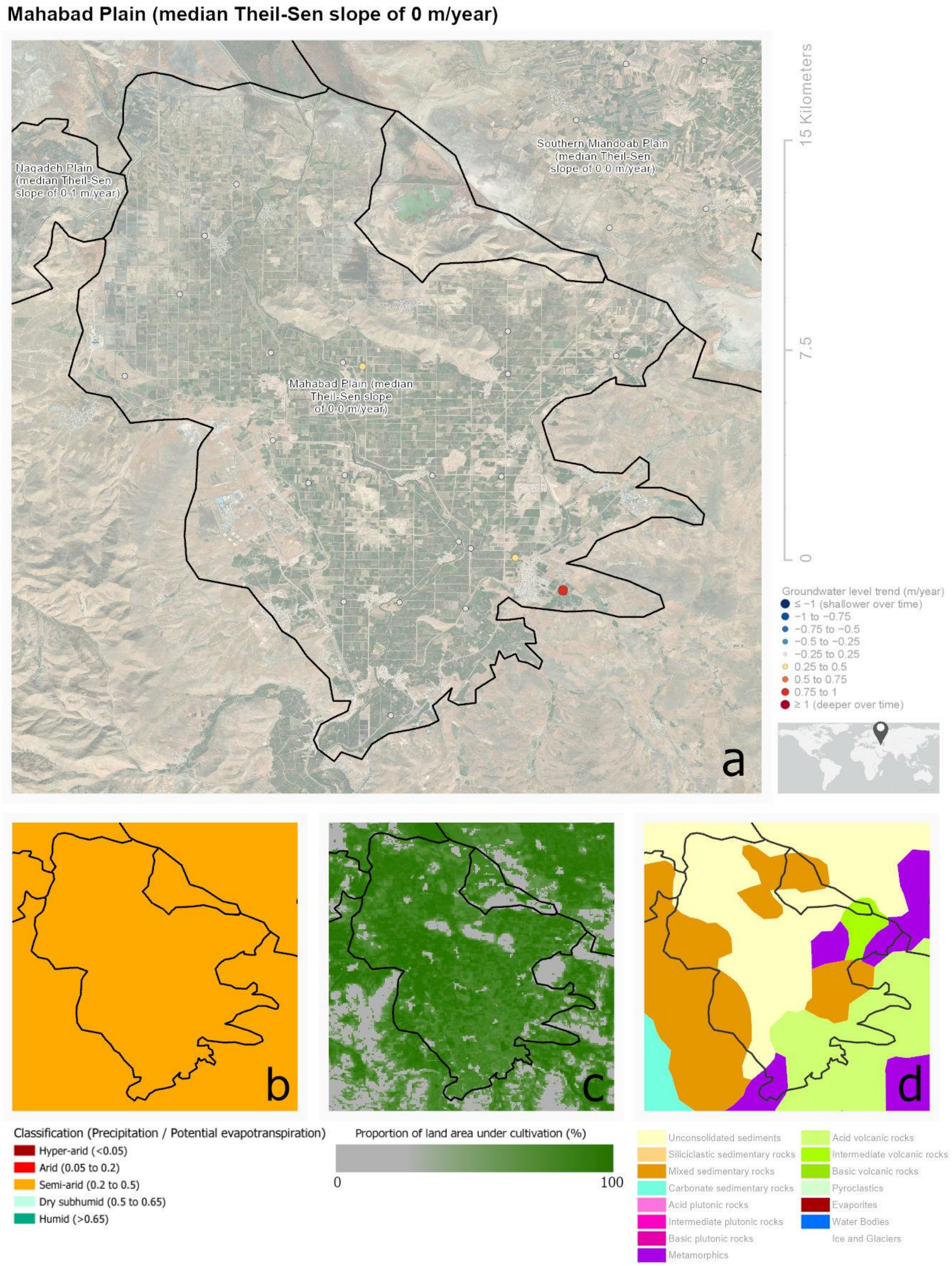

Supplementary Fig. 149. Spatial variability of trends in depth to groundwater Mahabad Plain. For detailed description of the contents of each panel see the text at the beginning of Supplementary Note 17.

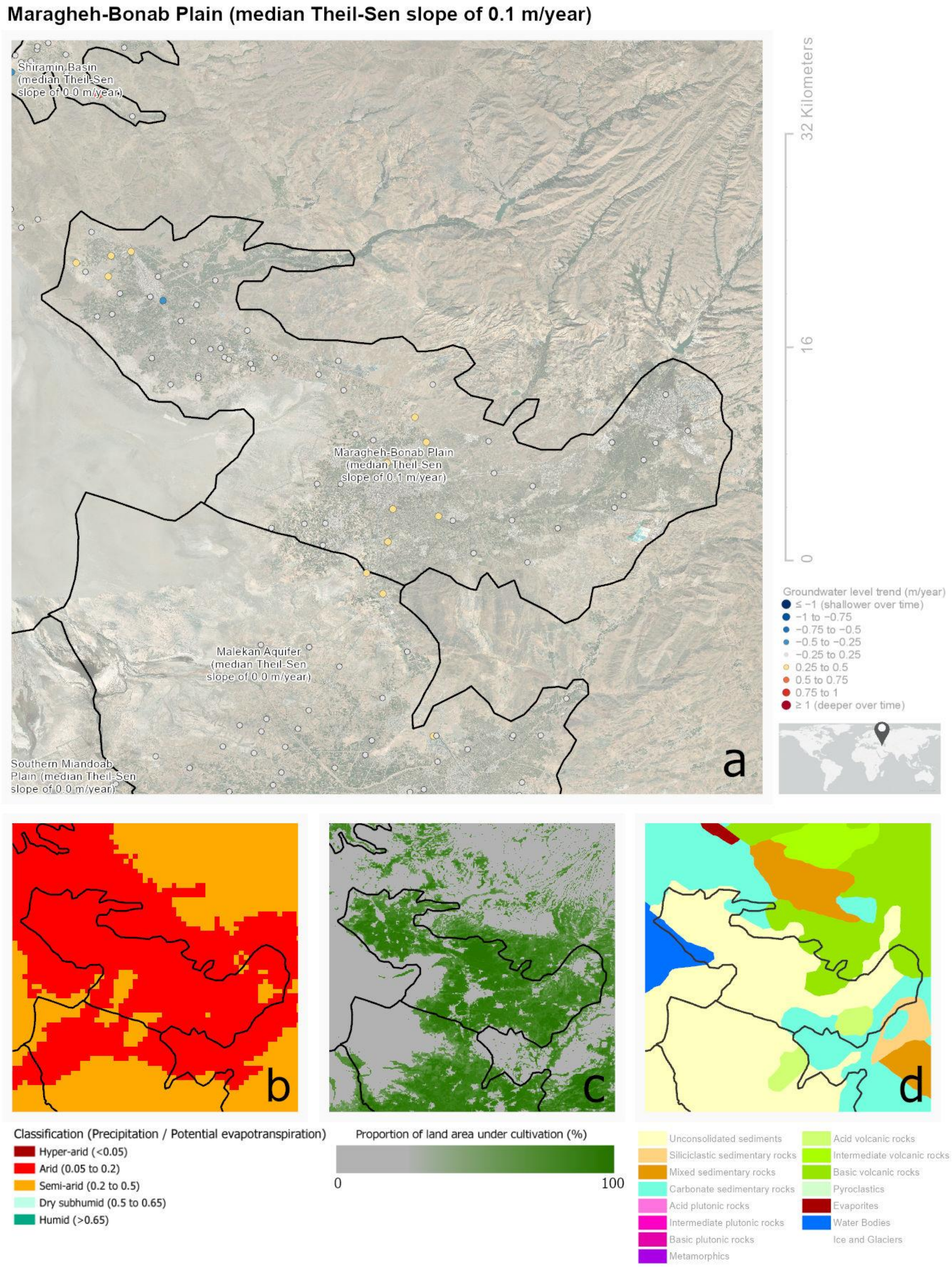

Supplementary Fig. 150. Spatial variability of trends in depth to groundwater Maragheh-Bonab Plain. For detailed description of the contents of each panel see the text at the beginning of Supplementary Note 17.

Mason Valley (median Theil-Sen slope of 0.1 m/year)

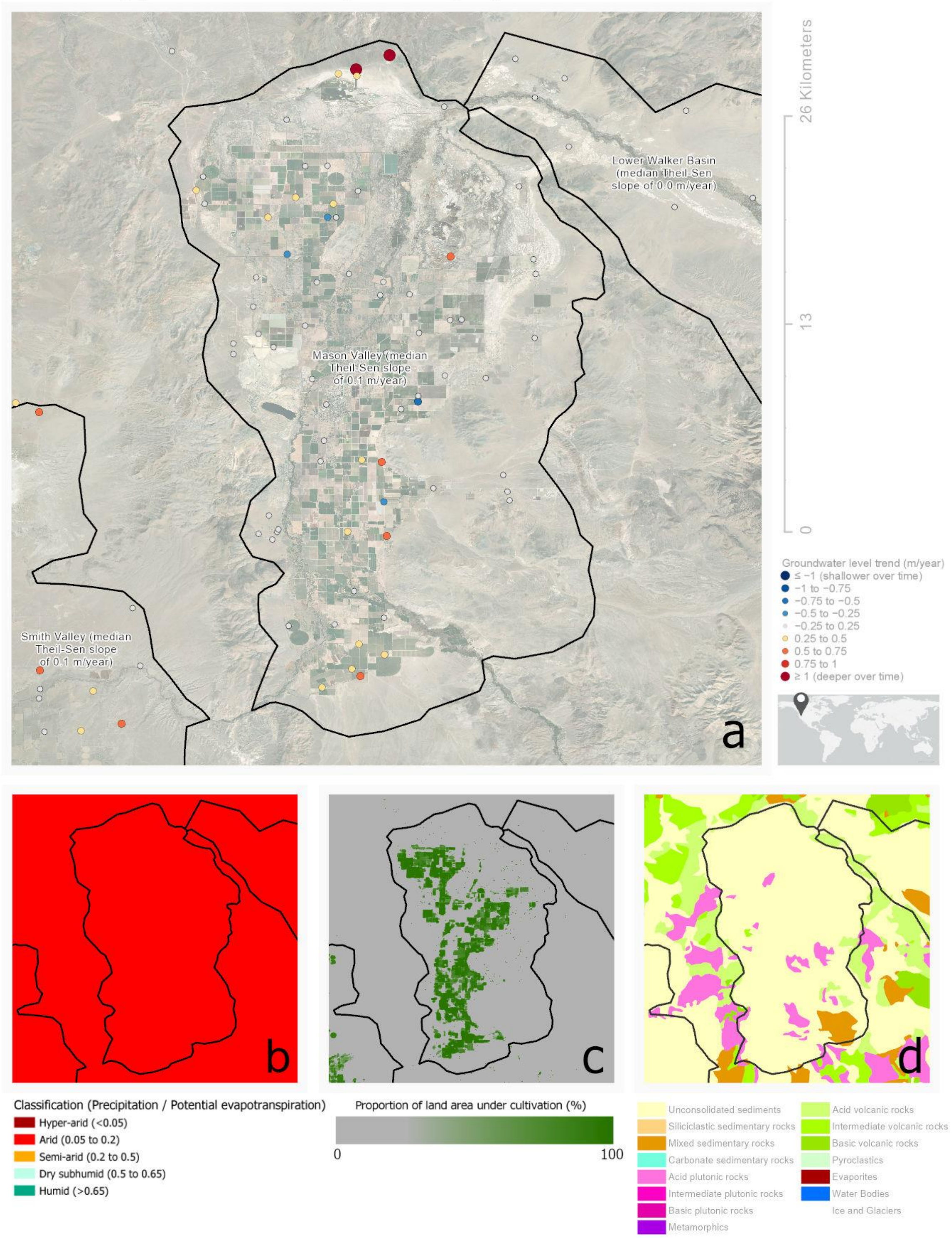

Supplementary Fig. 151. Spatial variability of trends in depth to groundwater Mason Valley. For detailed description of the contents of each panel see the text at the beginning of Supplementary Note 17.

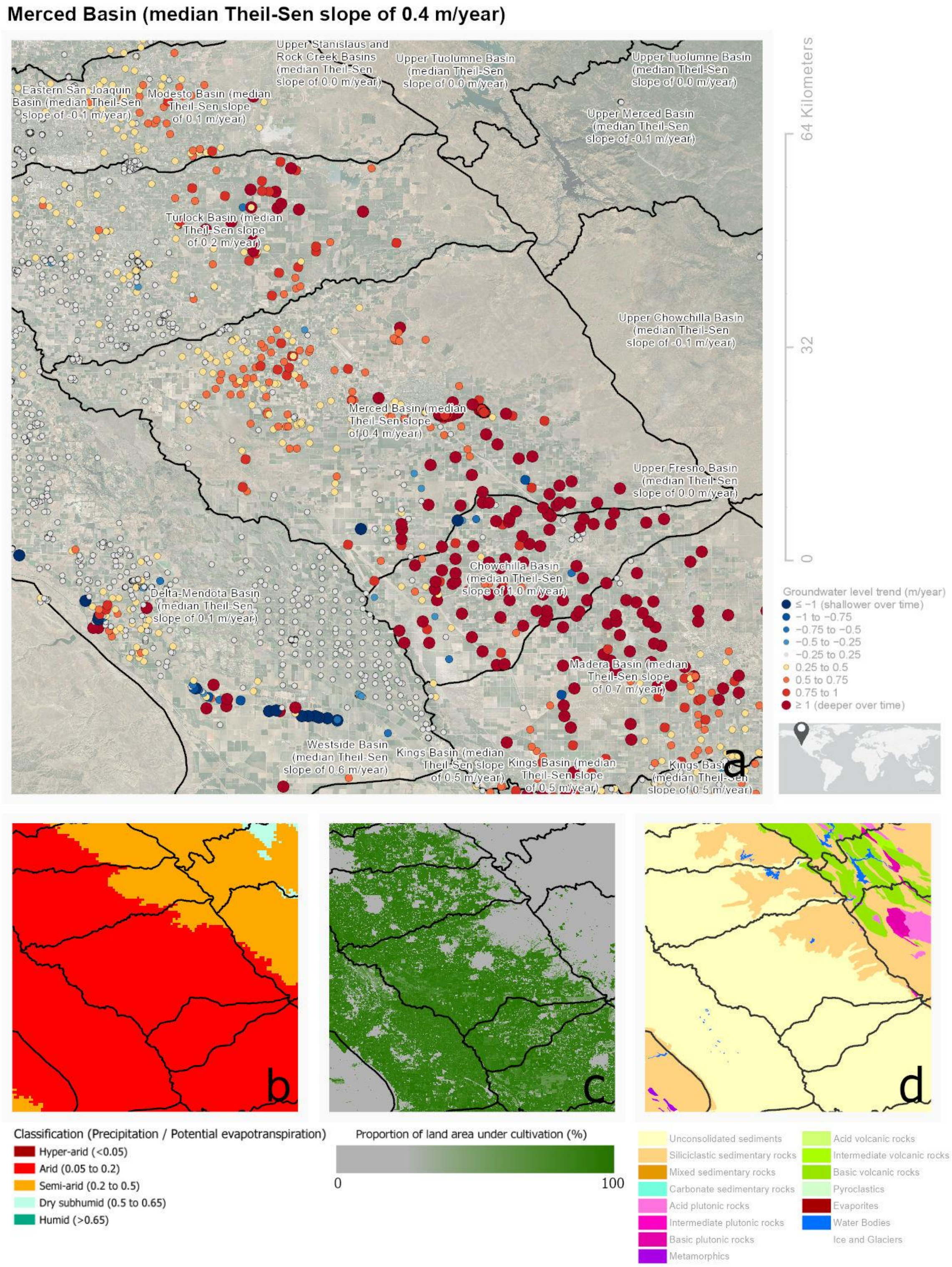

Supplementary Fig. 152. Spatial variability of trends in depth to groundwater Merced Basin (within the broader San Joaquin Basin of the Central Valley). For detailed description of the contents of each panel see the text at the beginning of Supplementary Note 17.

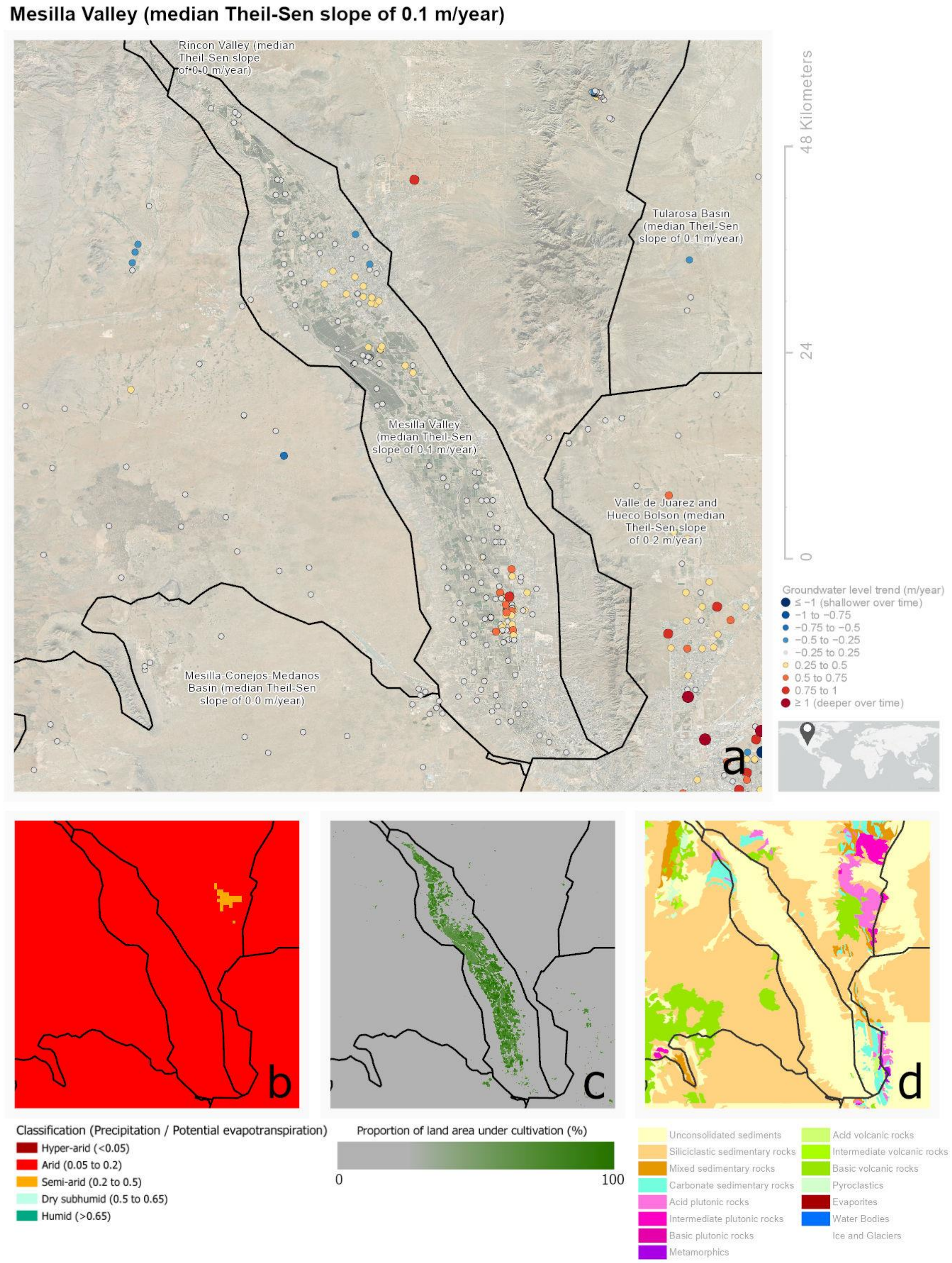

Supplementary Fig. 153. Spatial variability of trends in depth to groundwater Mesilla Valley (within the broader Rincon-Mesilla Valleys). For detailed description of the contents of each panel see the text at the beginning of Supplementary Note 17.

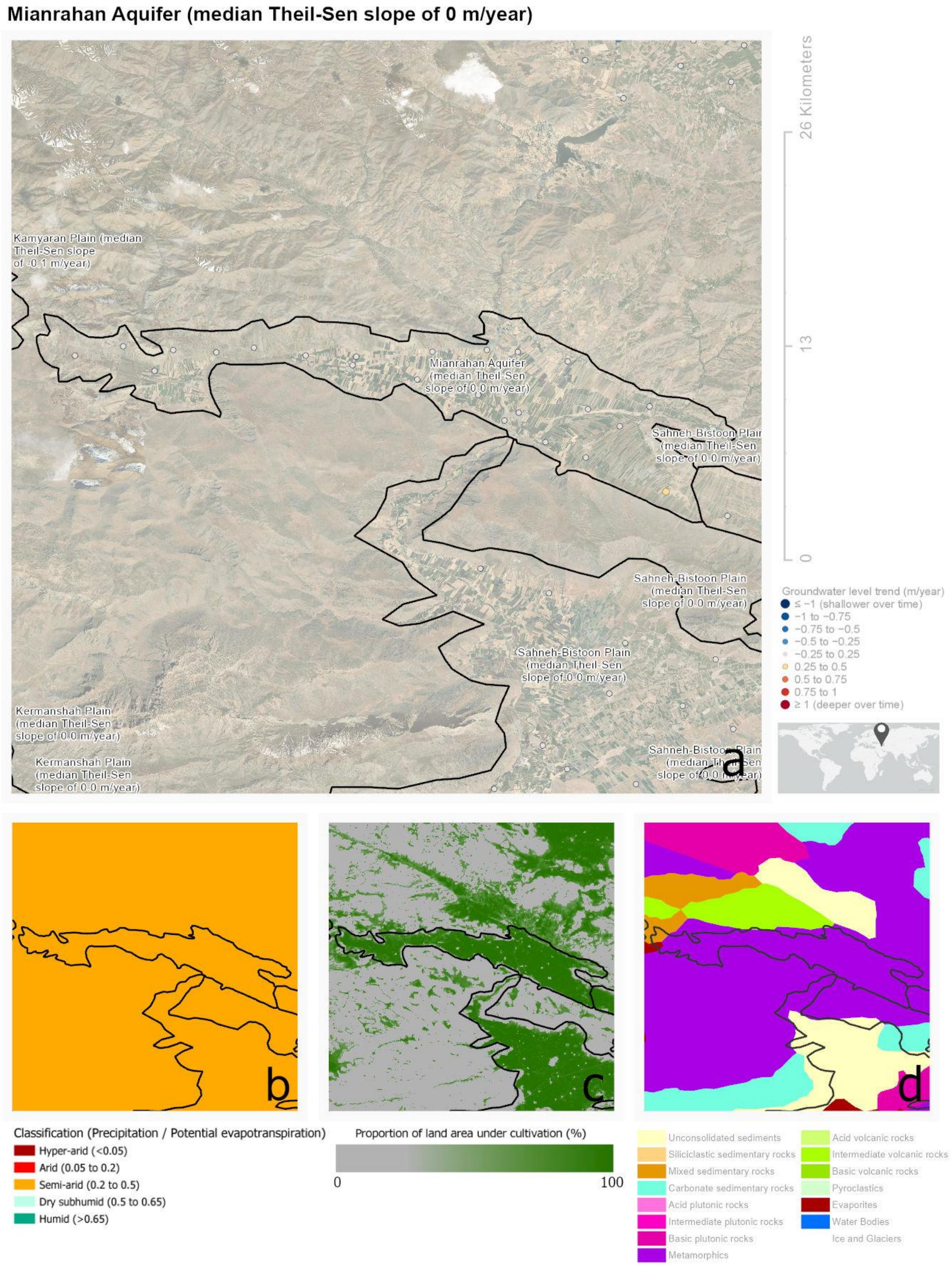

Supplementary Fig. 154. Spatial variability of trends in depth to groundwater Mianrahan Aquifer. For detailed description of the contents of each panel see the text at the beginning of Supplementary Note 17.

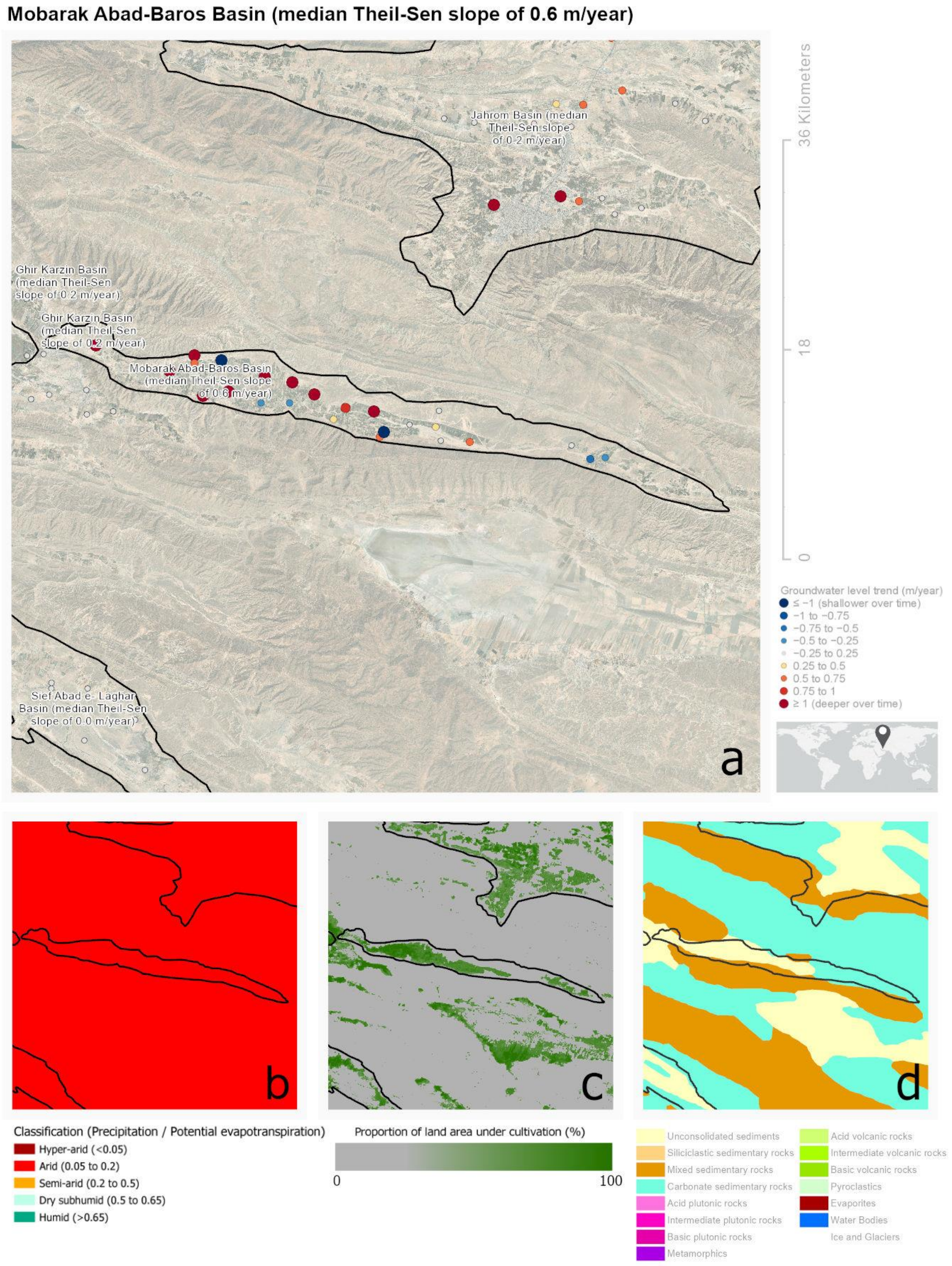

Supplementary Fig. 155. Spatial variability of trends in depth to groundwater Mobarak Abad-Baros Basin. For detailed description of the contents of each panel see the text at the beginning of Supplementary Note 17.

Modesto Basin (median Theil-Sen slope of 0.1 m/year)

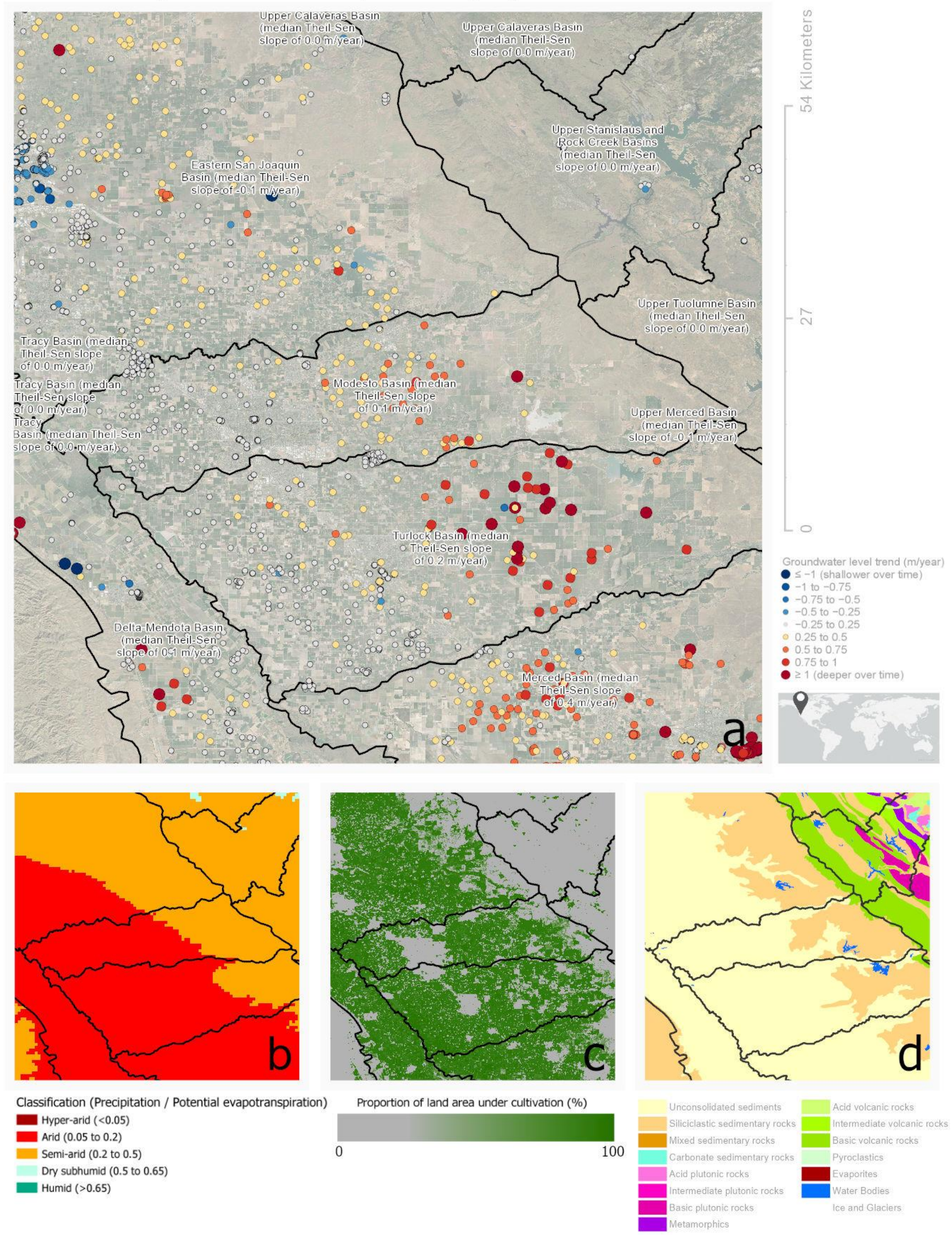

Supplementary Fig. 156. Spatial variability of trends in depth to groundwater Modesto Basin (within the broader San Joaquin Basin of the Central Valley). For detailed description of the contents of each panel see the text at the beginning of Supplementary Note 17.

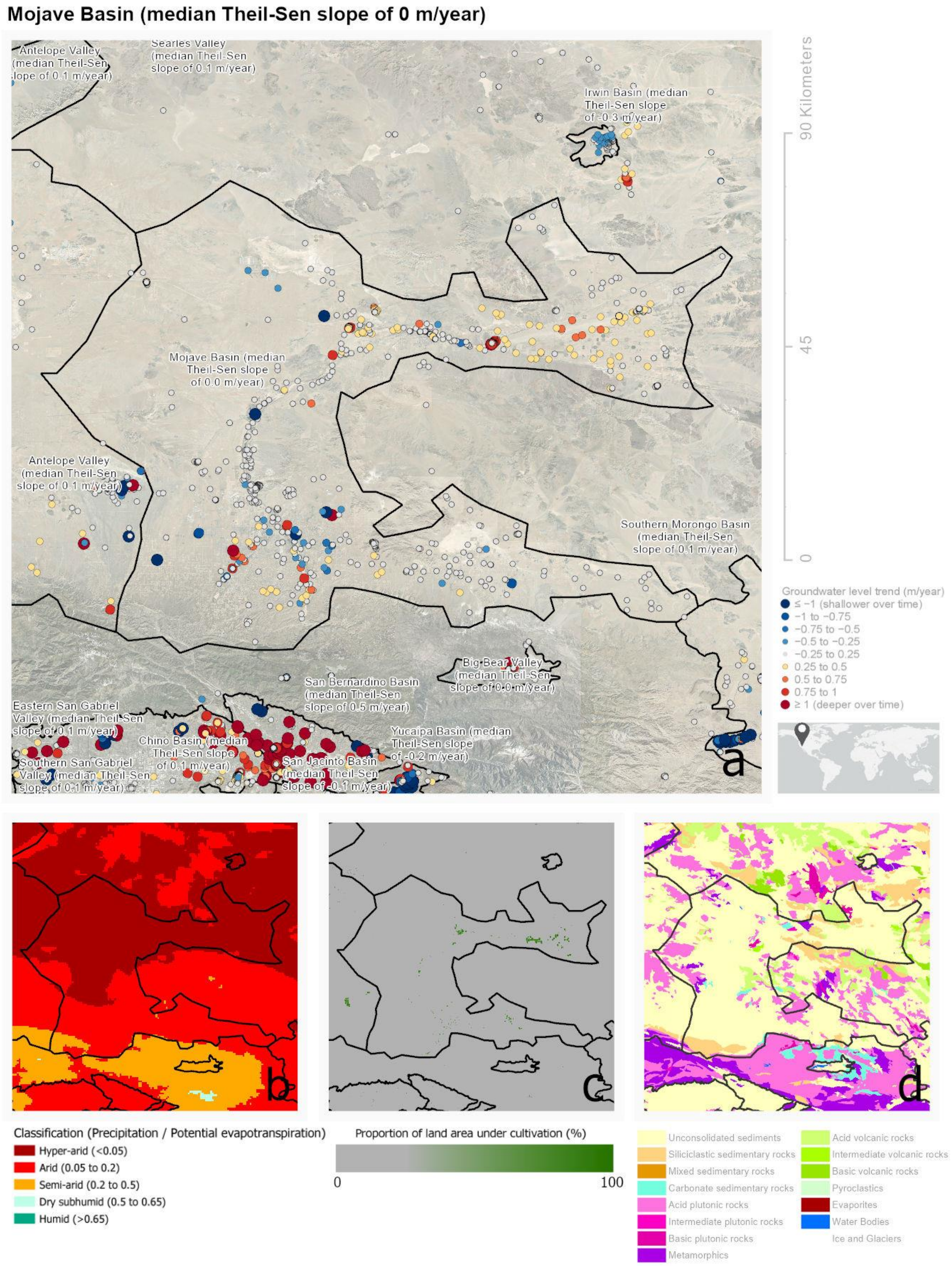

Supplementary Fig. 157. Spatial variability of trends in depth to groundwater Mojave Basin. For detailed description of the contents of each panel see the text at the beginning of Supplementary Note 17.

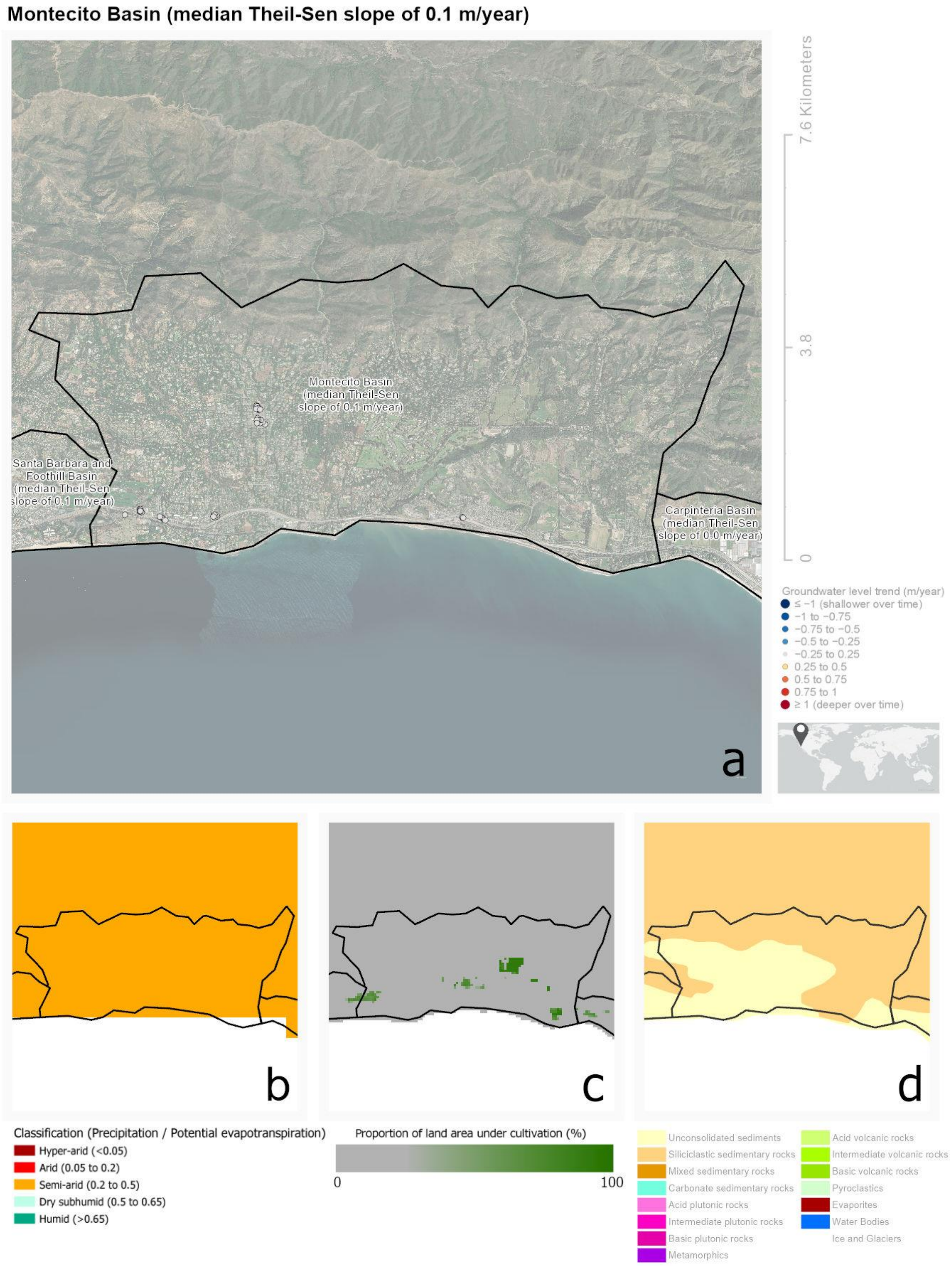

Supplementary Fig. 158. Spatial variability of trends in depth to groundwater Montecito Basin. For detailed description of the contents of each panel see the text at the beginning of Supplementary Note 17.

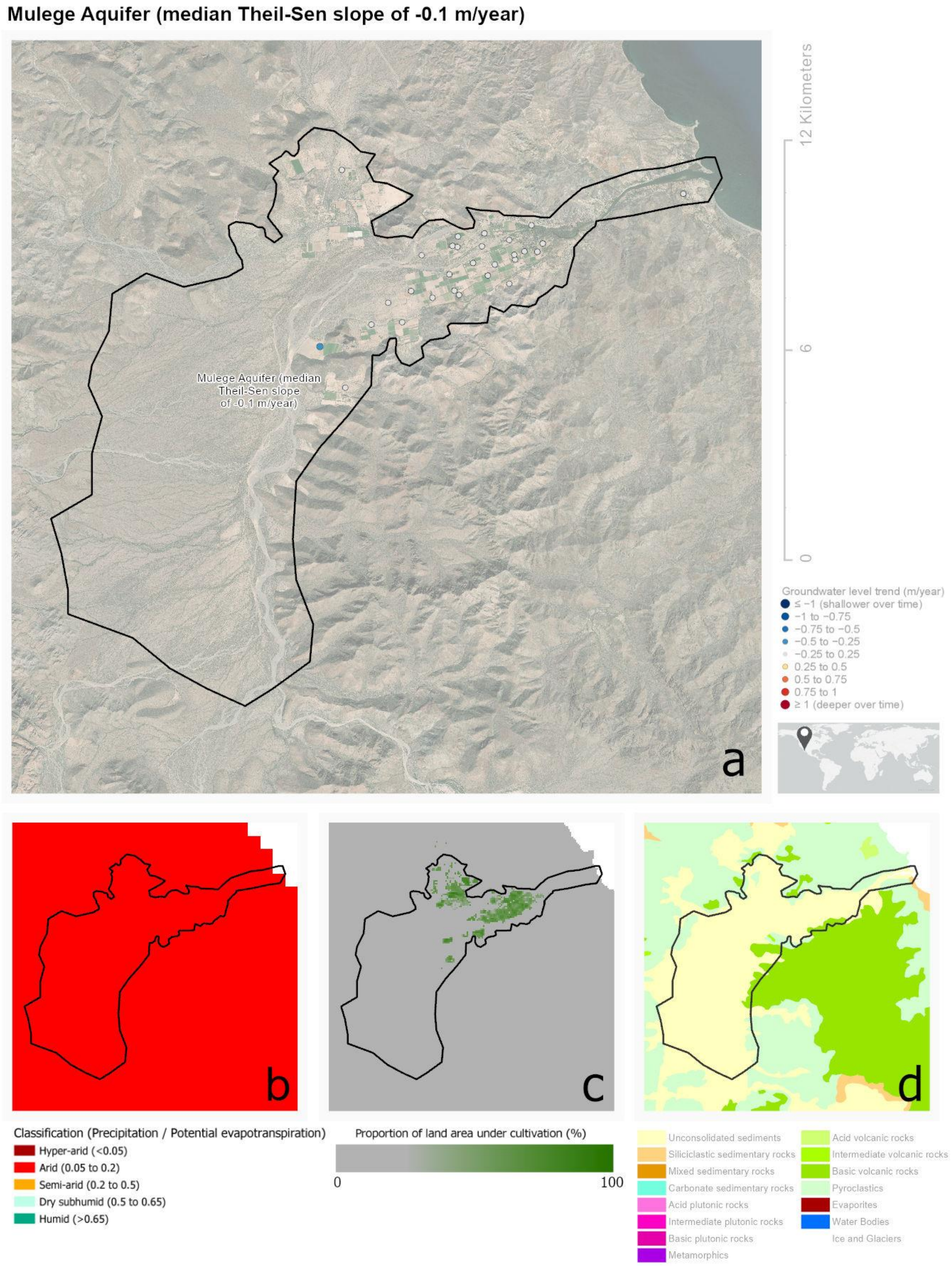

Supplementary Fig. 159. Spatial variability of trends in depth to groundwater Mulege Aquifer. For detailed description of the contents of each panel see the text at the beginning of Supplementary Note 17.

Mulgrave Aquifer (median Theil-Sen slope of 0 m/year)

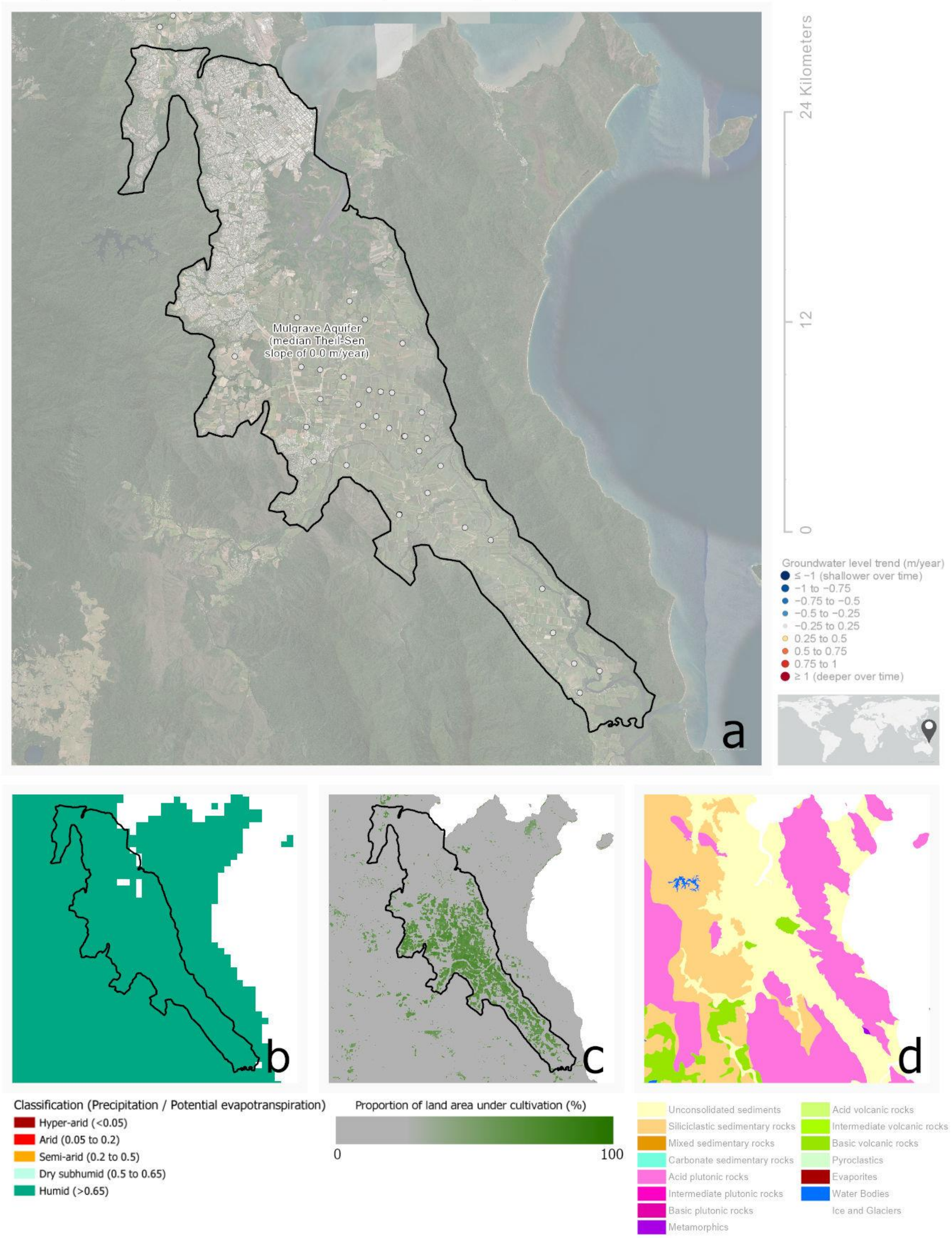

Supplementary Fig. 160. Spatial variability of trends in depth to groundwater Mulgrave Aquifer. For detailed description of the contents of each panel see the text at the beginning of Supplementary Note 17.

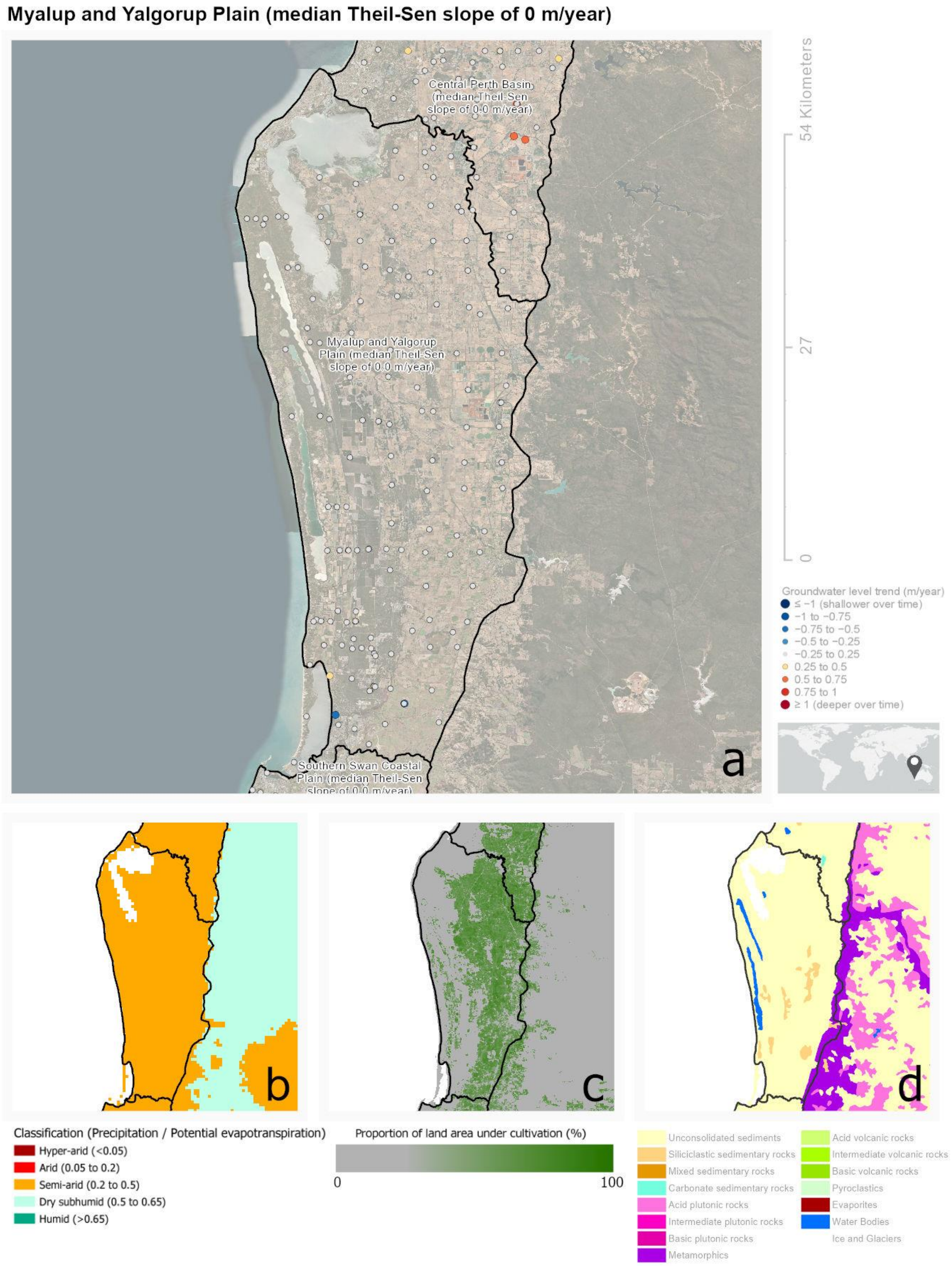

Supplementary Fig. 161. Spatial variability of trends in depth to groundwater Myalup and Yalgorup Plain (within the broader Perth Basin). For detailed description of the contents of each panel see the text at the beginning of Supplementary Note 17.

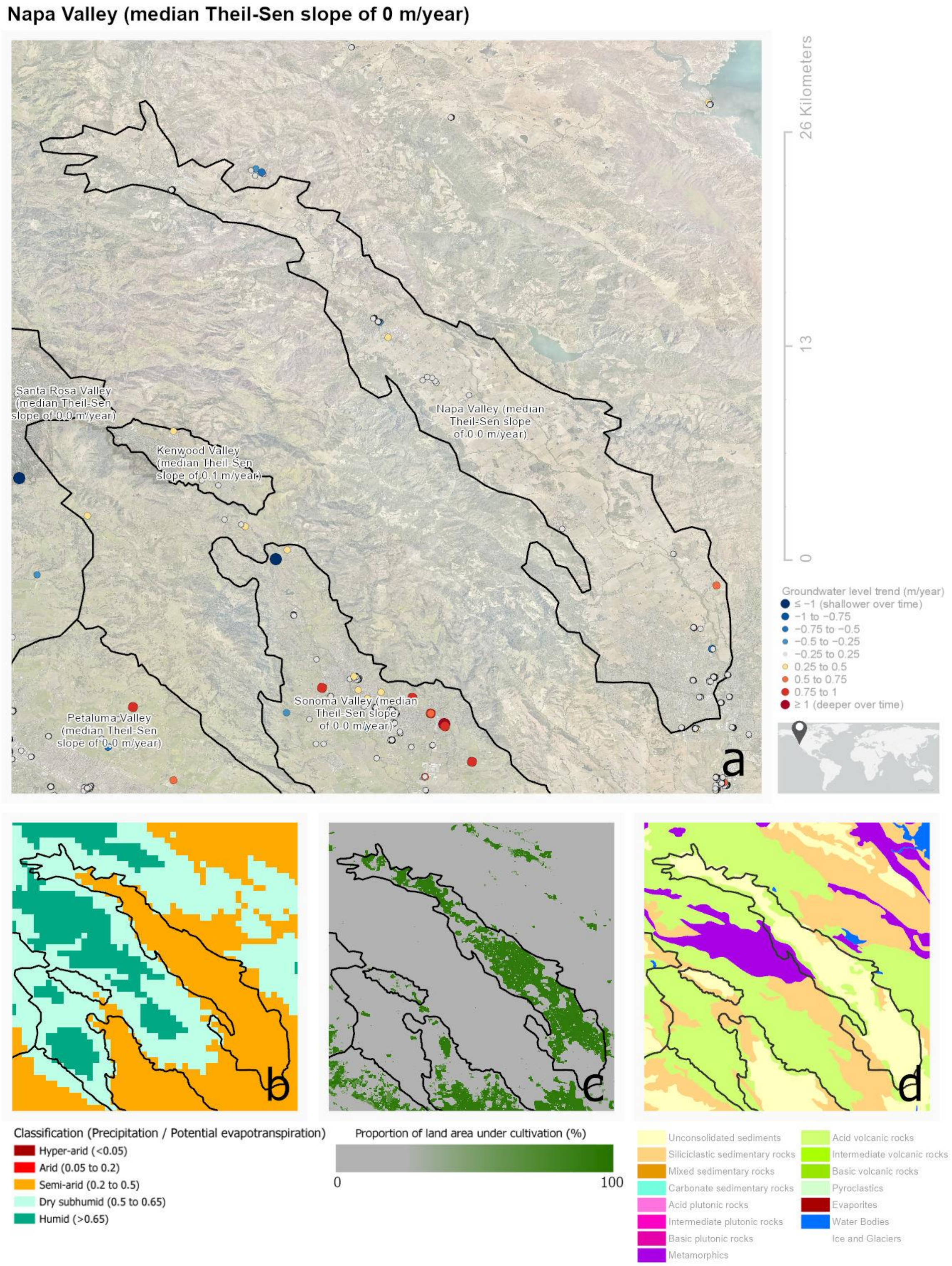

Supplementary Fig. 162. Spatial variability of trends in depth to groundwater Napa Valley. For detailed description of the contents of each panel see the text at the beginning of Supplementary Note 17.

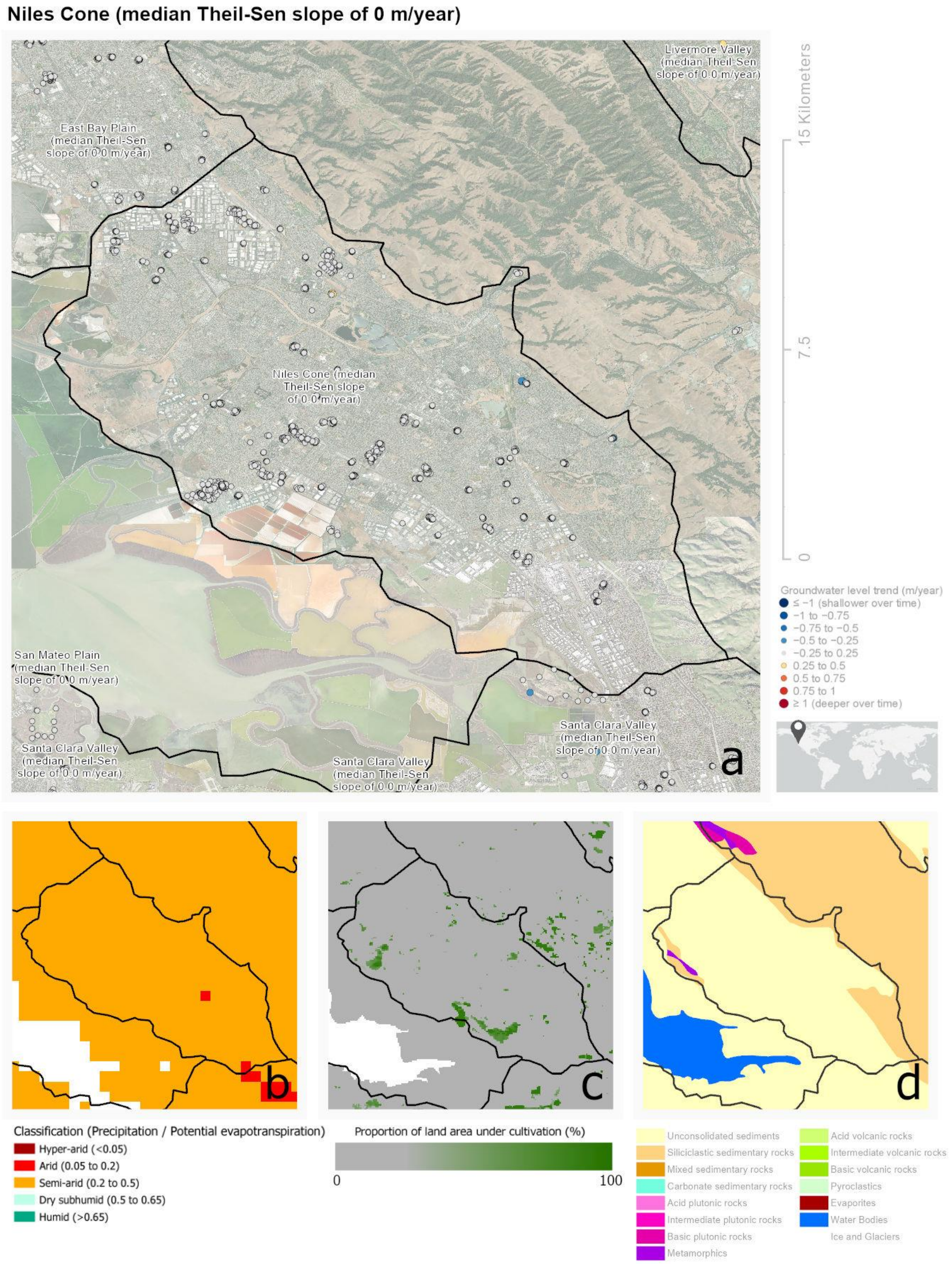

Supplementary Fig. 163. Spatial variability of trends in depth to groundwater Niles Cone. For detailed description of the contents of each panel see the text at the beginning of Supplementary Note 17.

North American Basin (median Theil-Sen slope of 0 m/year)

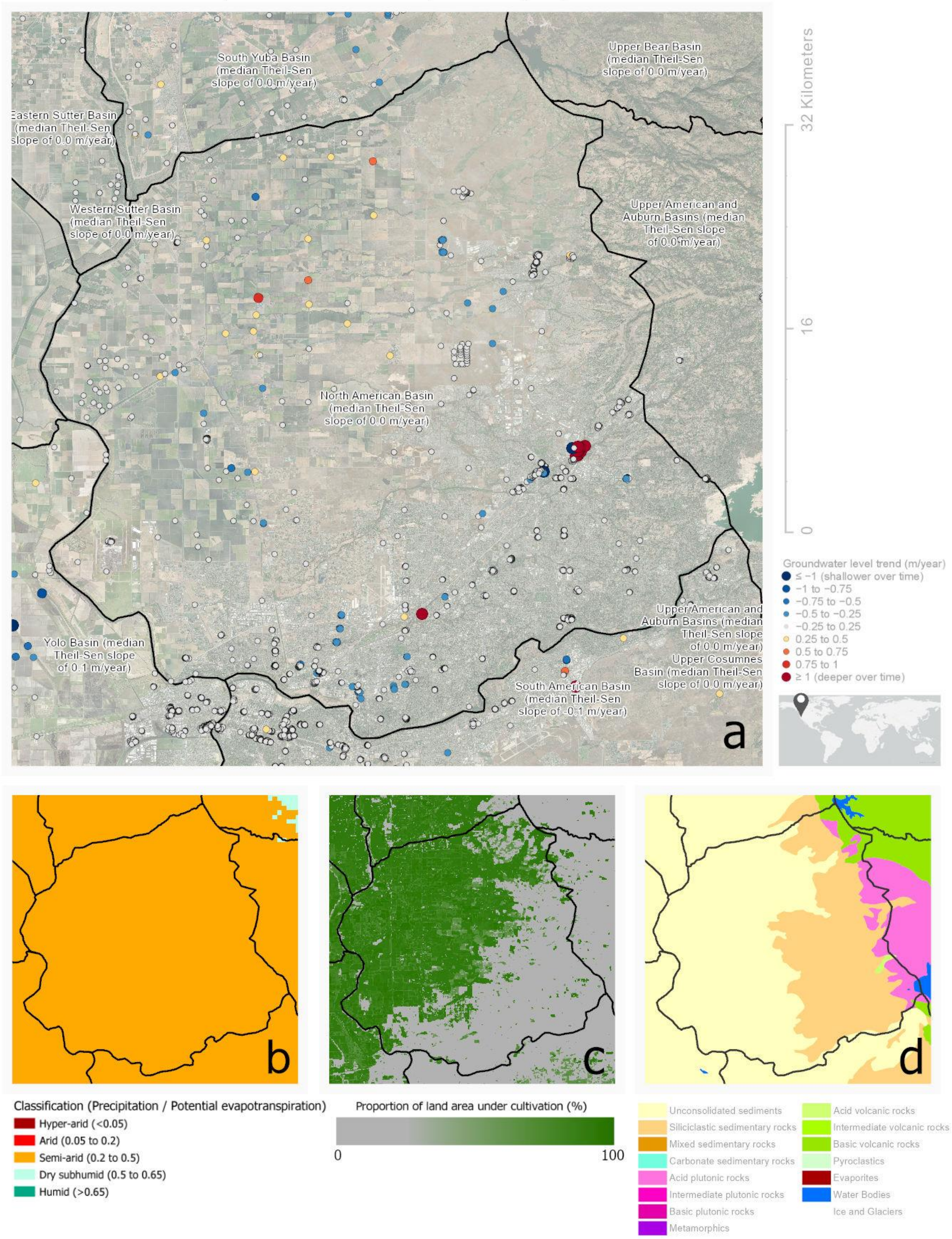

Supplementary Fig. 164. Spatial variability of trends in depth to groundwater North American Basin (within the broader Sacramento Valley of the Central Valley). For detailed description of the contents of each panel see the text at the beginning of Supplementary Note 17.

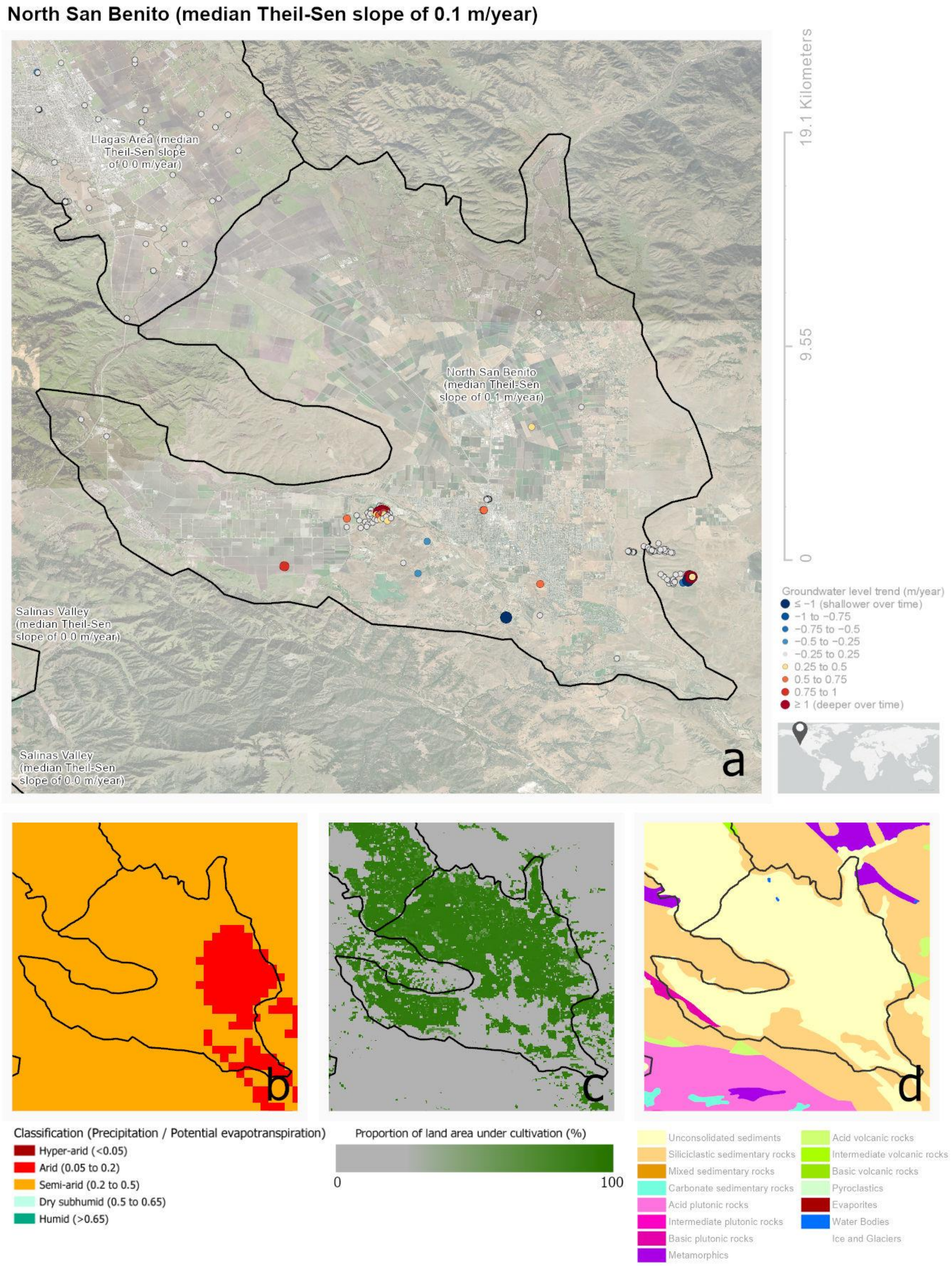

Supplementary Fig. 165. Spatial variability of trends in depth to groundwater North San Benito (within the broader Gilroy-Hollister Valley). For detailed description of the contents of each panel see the text at the beginning of Supplementary Note 17.

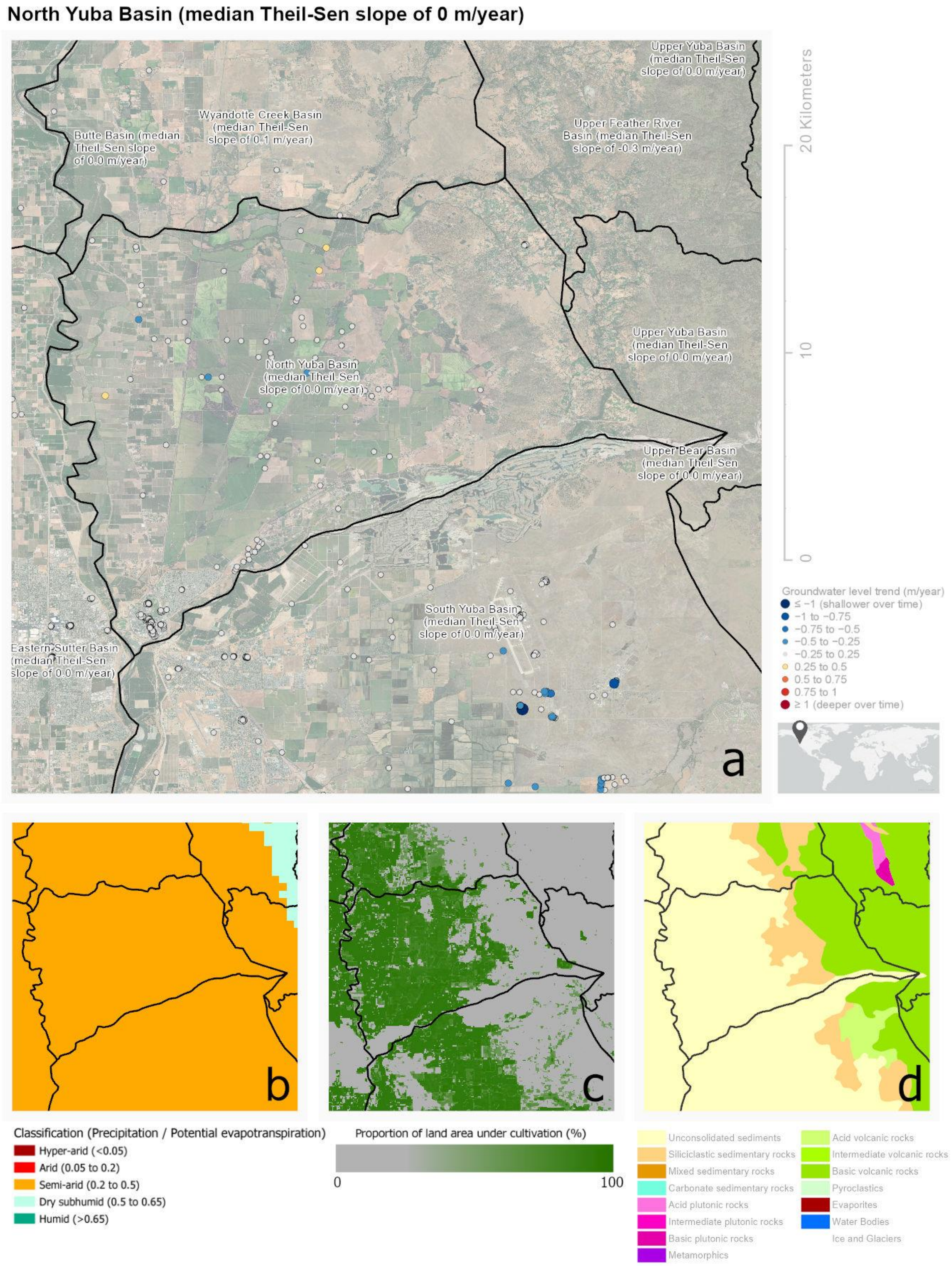

Supplementary Fig. 166. Spatial variability of trends in depth to groundwater North Yuba Basin (within the broader Sacramento Valley of the Central Valley). For detailed description of the contents of each panel see the text at the beginning of Supplementary Note 17.

Northern Adelaide Plains (median Theil-Sen slope of 0 m/year)

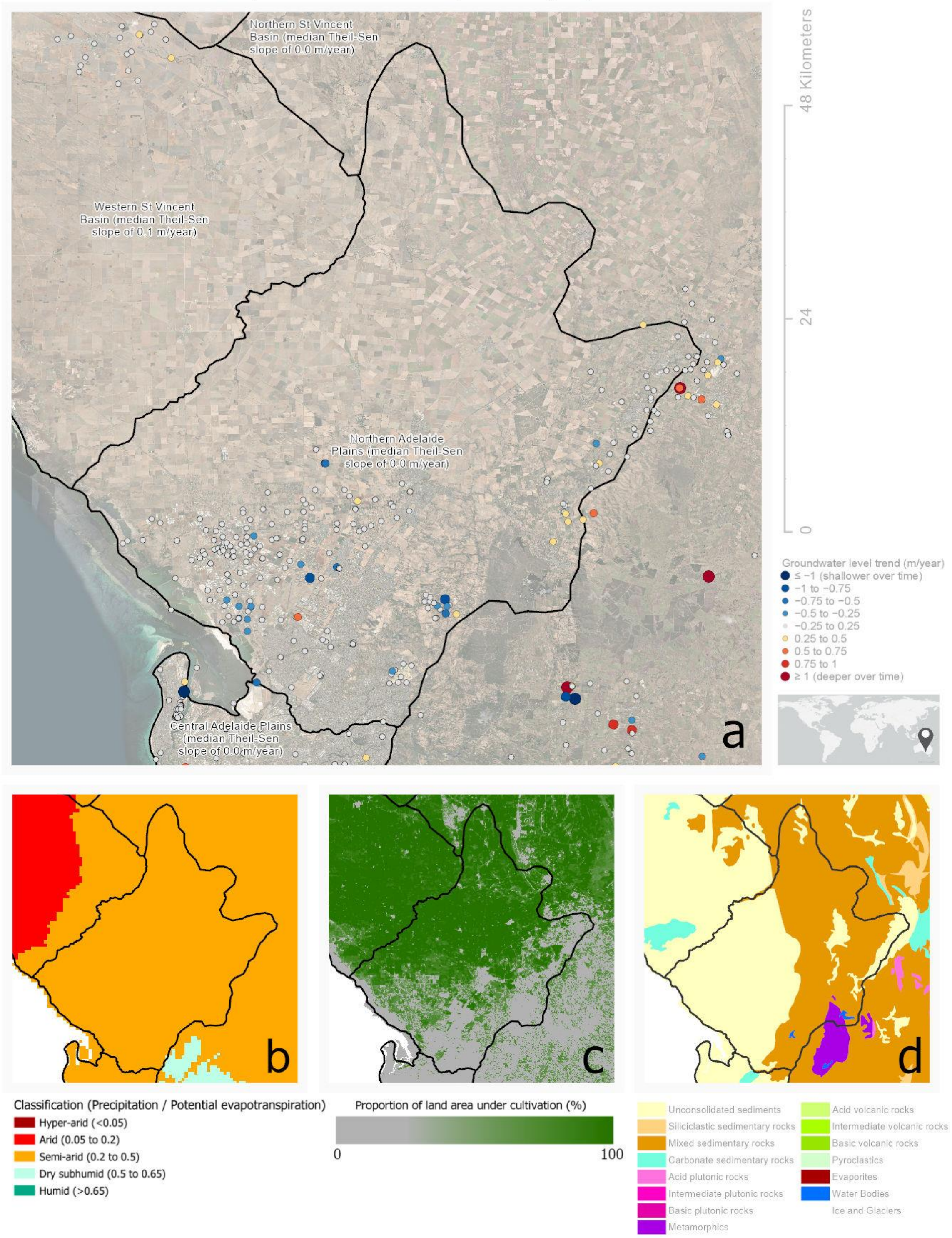

Supplementary Fig. 167. Spatial variability of trends in depth to groundwater Northern Adelaide Plains (within the broader St Vincent Basin). For detailed description of the contents of each panel see the text at the beginning of Supplementary Note 17.

Northern Colusa Basin (median Theil-Sen slope of 0.3 m/year)

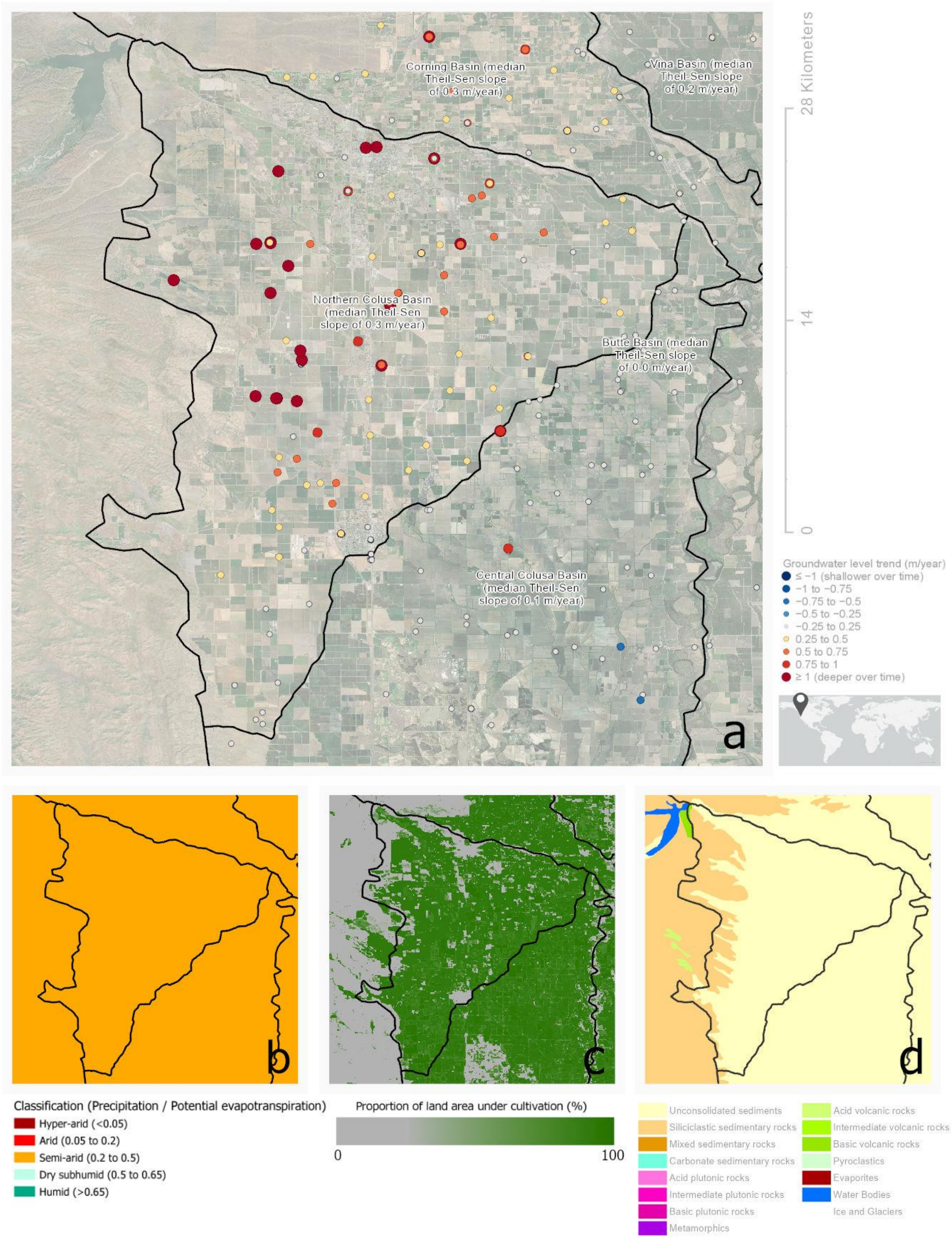

Supplementary Fig. 168. Spatial variability of trends in depth to groundwater Northern Colusa Basin (within the broader Sacramento Valley of the Central Valley). For detailed description of the contents of each panel see the text at the beginning of Supplementary Note 17.

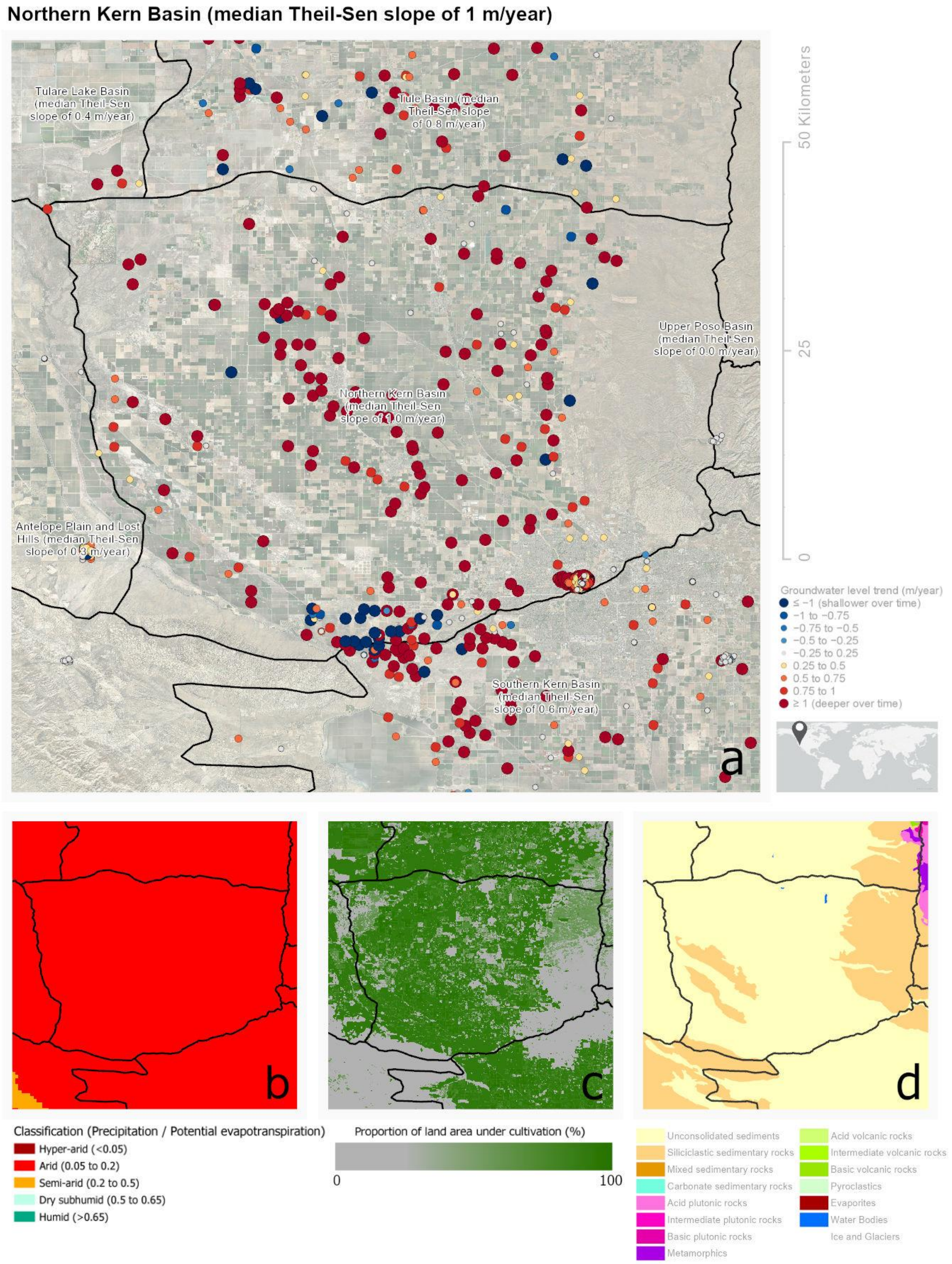

Supplementary Fig. 169. Spatial variability of trends in depth to groundwater Northern Kern Basin (within the broader Tulare Basin of the Central Valley). For detailed description of the contents of each panel see the text at the beginning of Supplementary Note 17.

Northern Plane Basin (median Theil-Sen slope of 0 m/year)

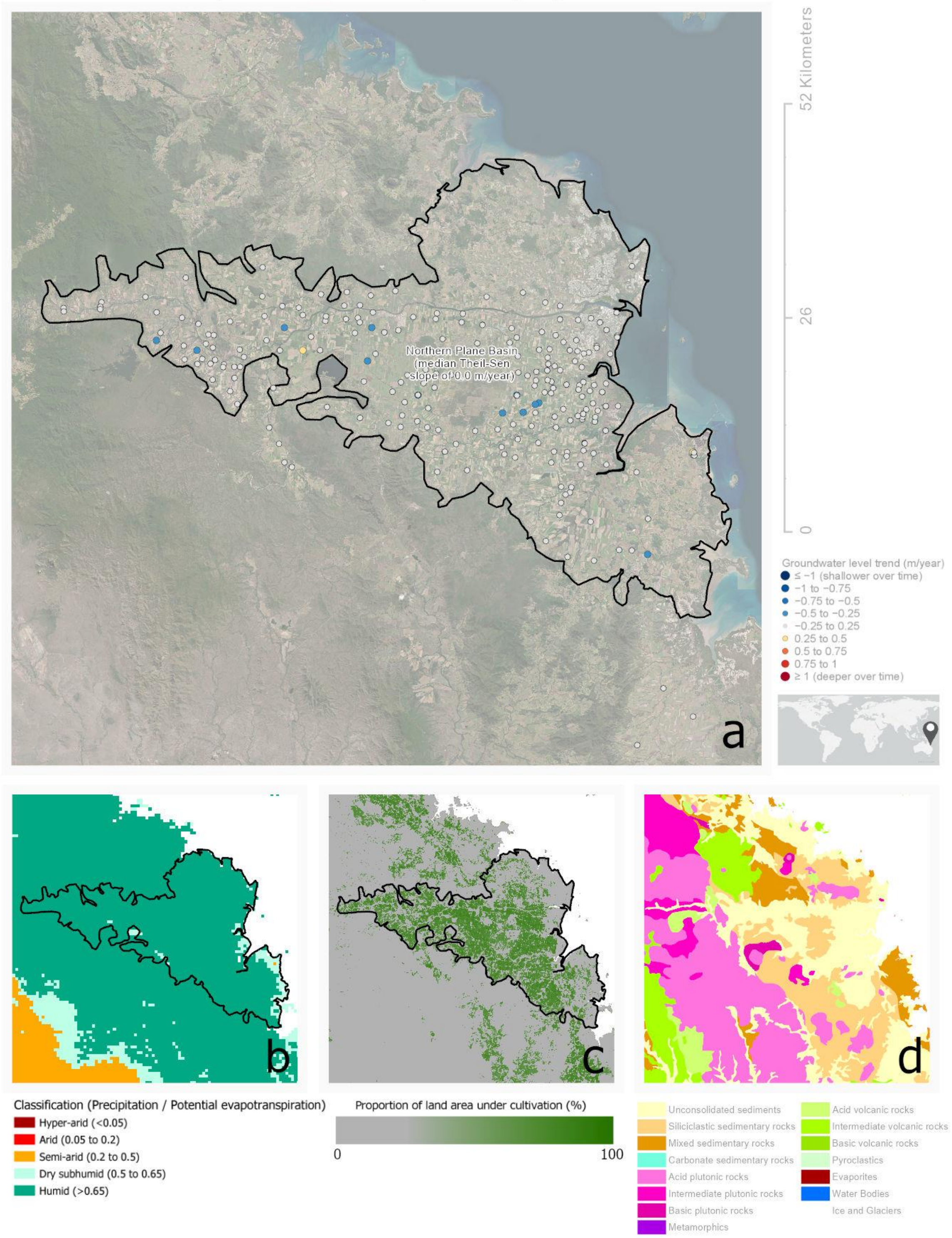

Supplementary Fig. 170. Spatial variability of trends in depth to groundwater Northern Plane Basin. For detailed description of the contents of each panel see the text at the beginning of Supplementary Note 17.

Northern Tule Lake (median Theil-Sen slope of 0.1 m/year)

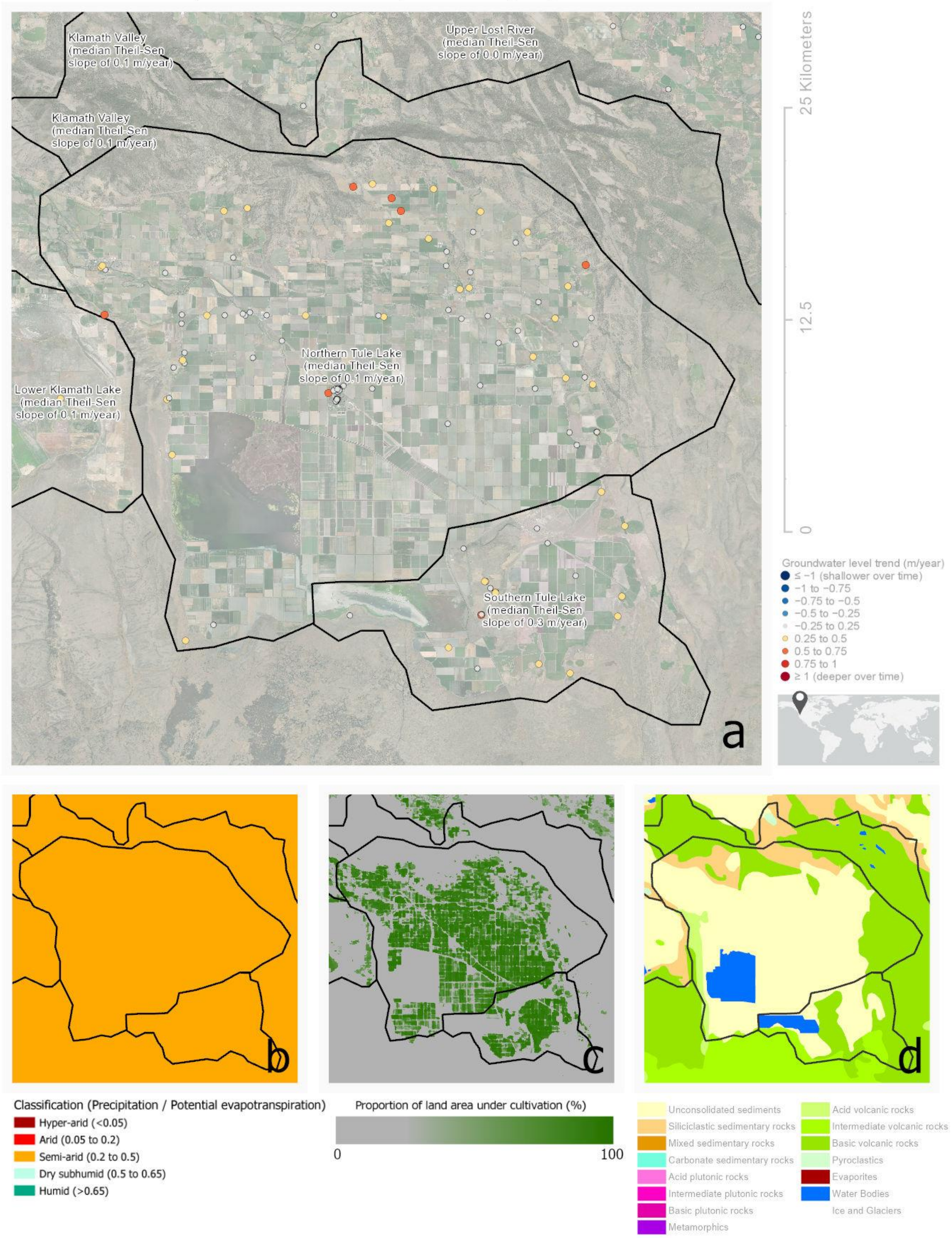

Supplementary Fig. 171. Spatial variability of trends in depth to groundwater Northern Tule Lake (within the broader Upper Klamath Basin). For detailed description of the contents of each panel see the text at the beginning of Supplementary Note 17.

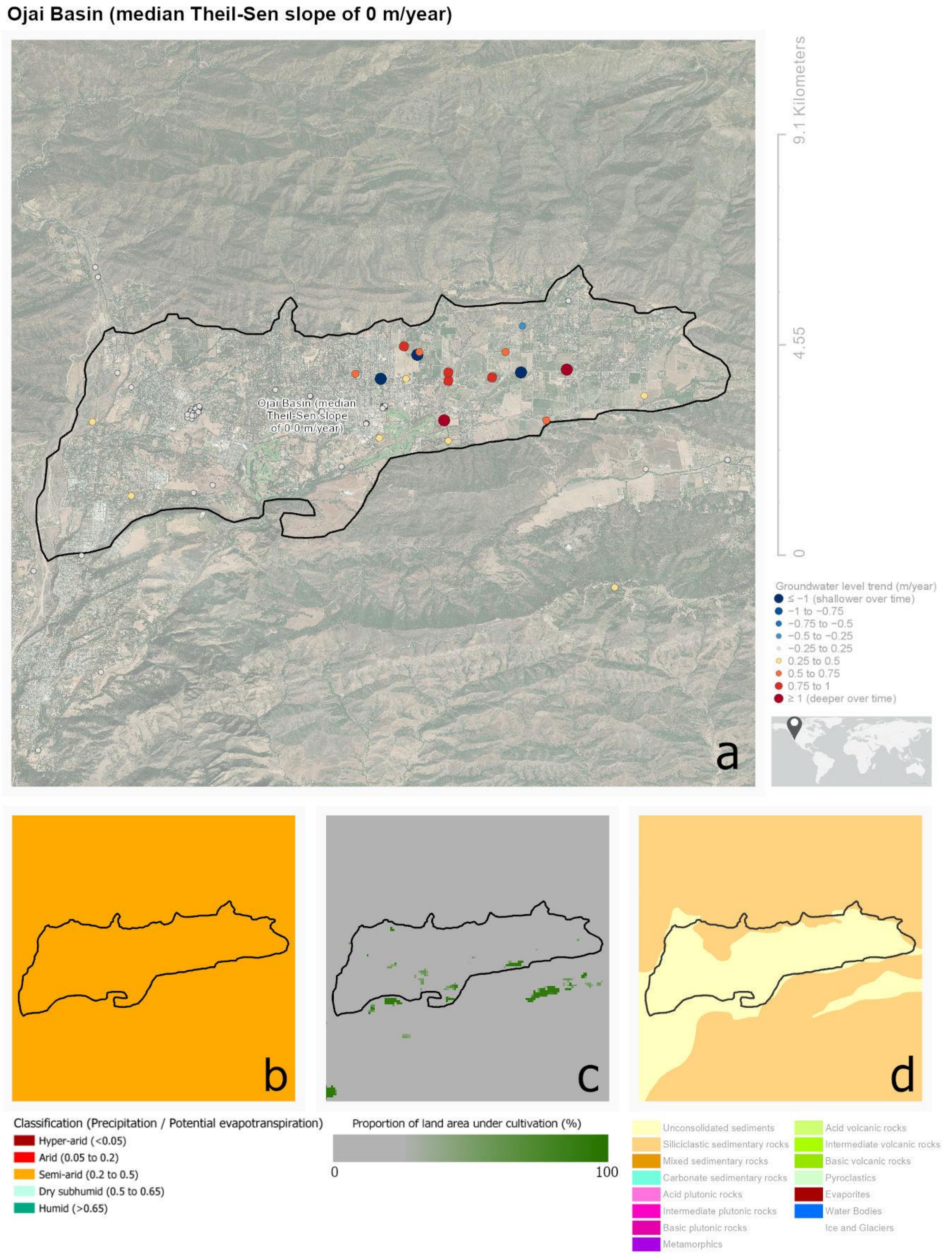

Supplementary Fig. 172. Spatial variability of trends in depth to groundwater Ojai Basin. For detailed description of the contents of each panel see the text at the beginning of Supplementary Note 17.

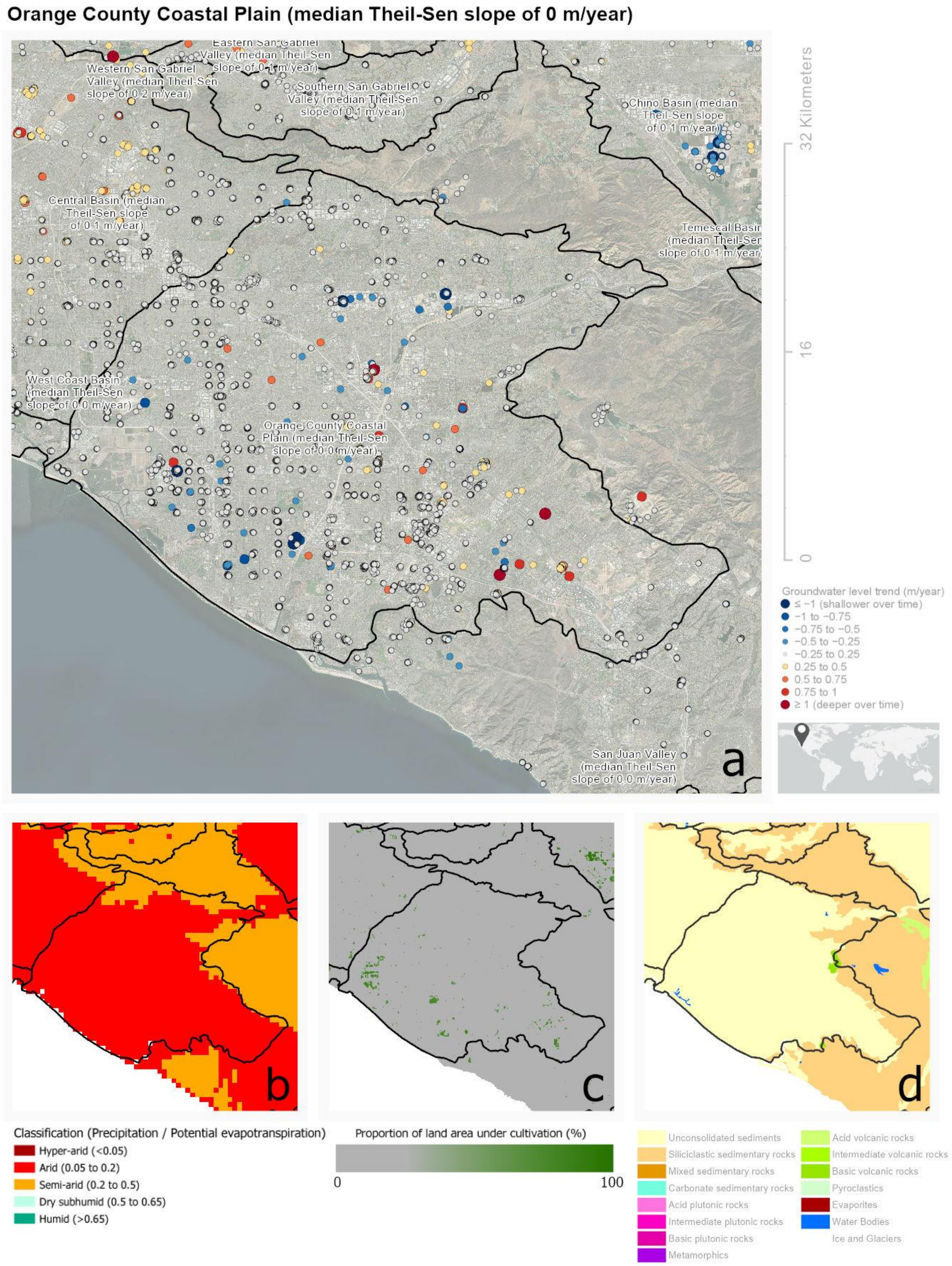

Supplementary Fig. 173. Spatial variability of trends in depth to groundwater Orange County Coastal Plain (within the broader Los Angeles Basin). For detailed description of the contents of each panel see the text at the beginning of Supplementary Note 17.

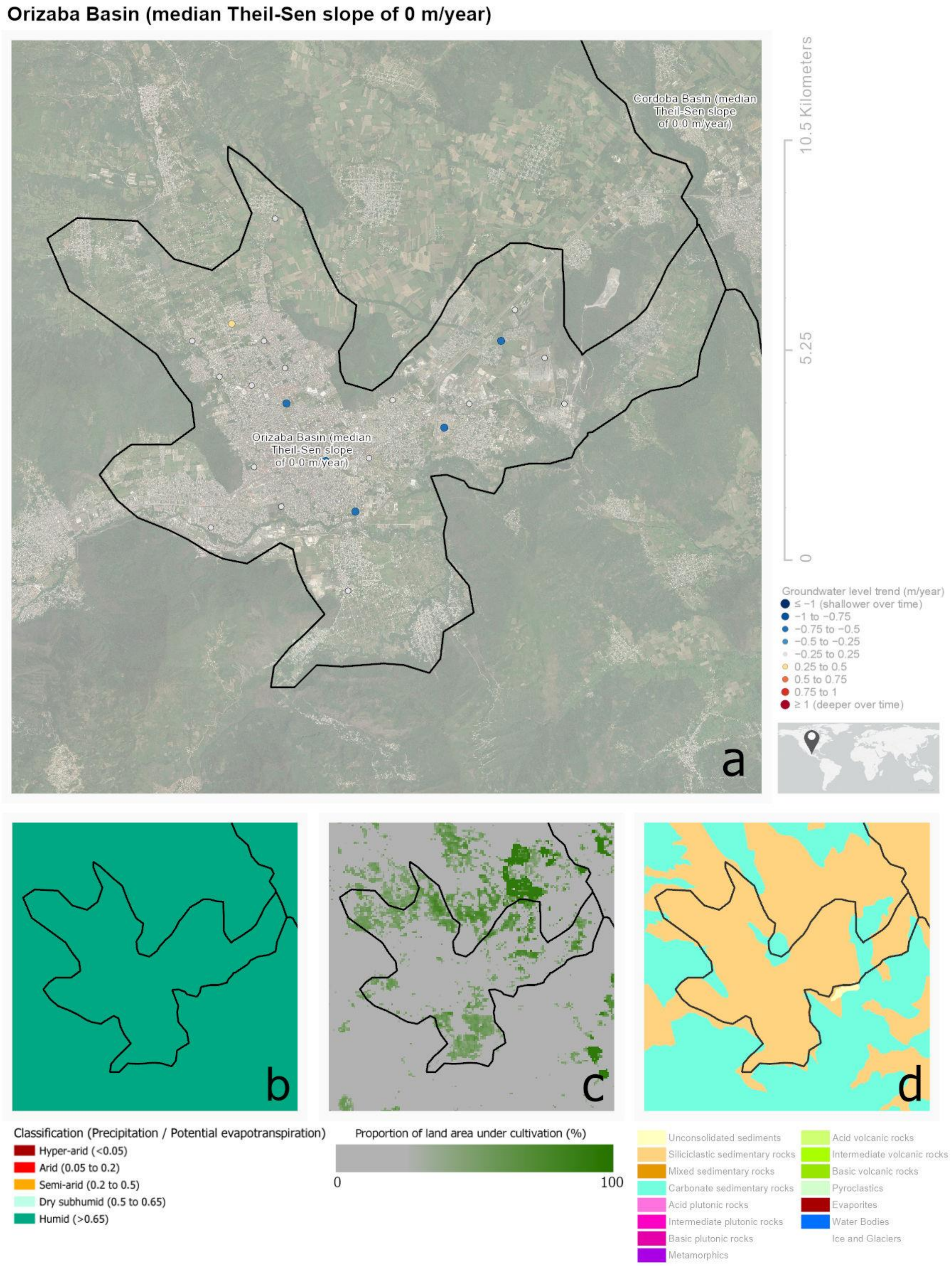

Supplementary Fig. 174. Spatial variability of trends in depth to groundwater Orizaba Basin (within the broader Orizaba-Cordoba Basins). For detailed description of the contents of each panel see the text at the beginning of Supplementary Note 17.

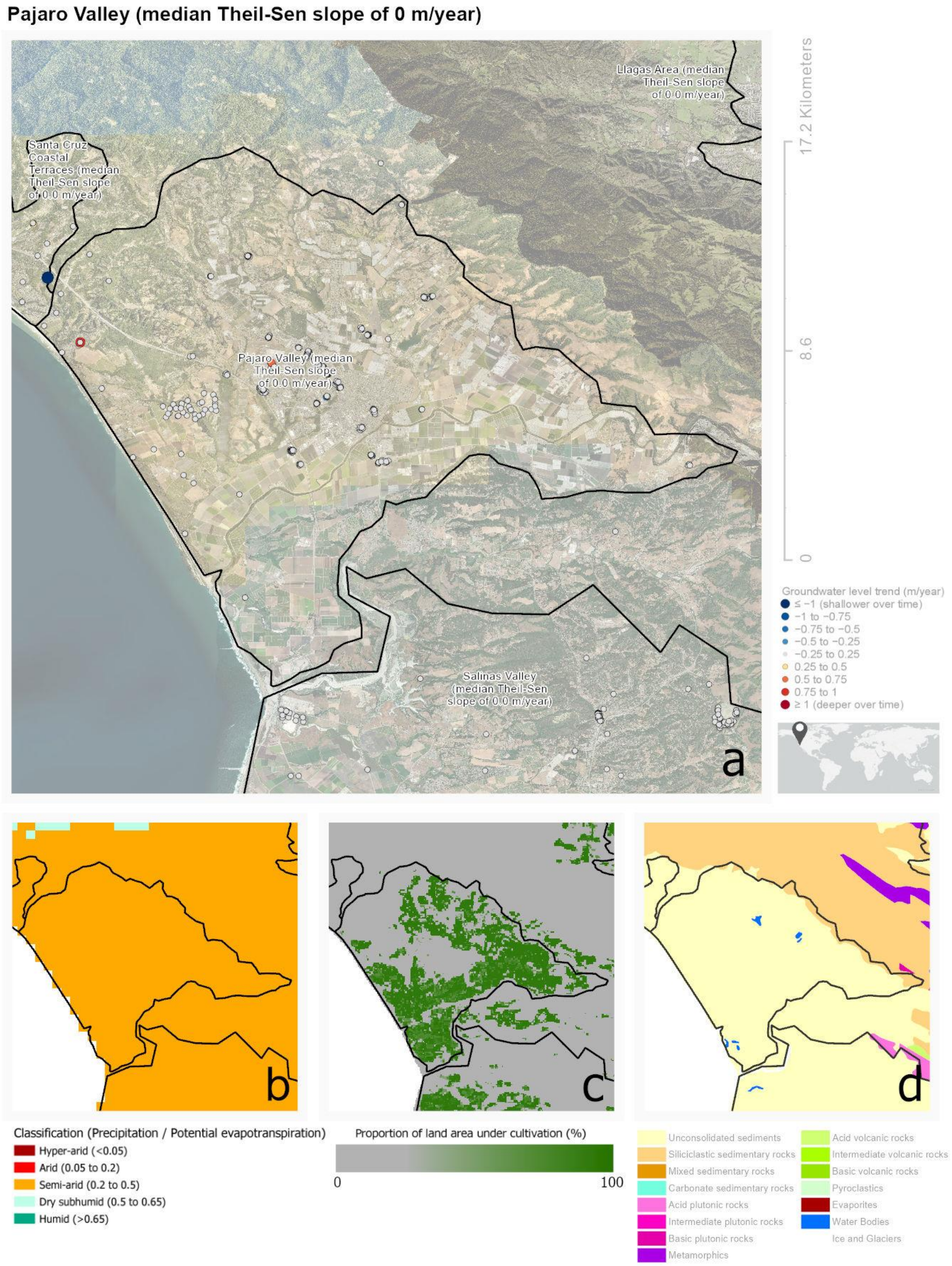

Supplementary Fig. 175. Spatial variability of trends in depth to groundwater Pajaro Valley. For detailed description of the contents of each panel see the text at the beginning of Supplementary Note 17.

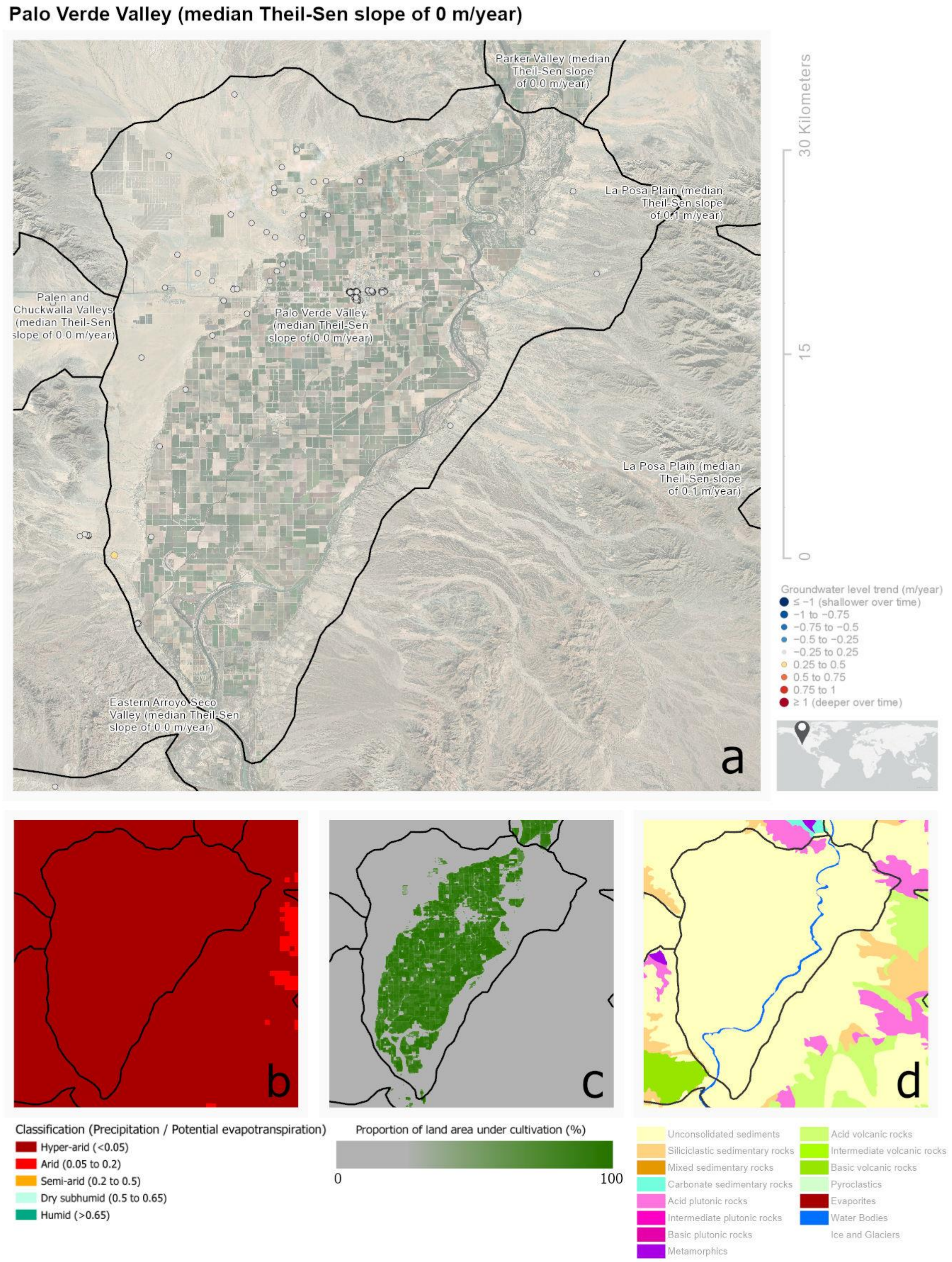

Supplementary Fig. 176. Spatial variability of trends in depth to groundwater Palo Verde Valley (within the broader Blythe Basin). For detailed description of the contents of each panel see the text at the beginning of Supplementary Note 17.

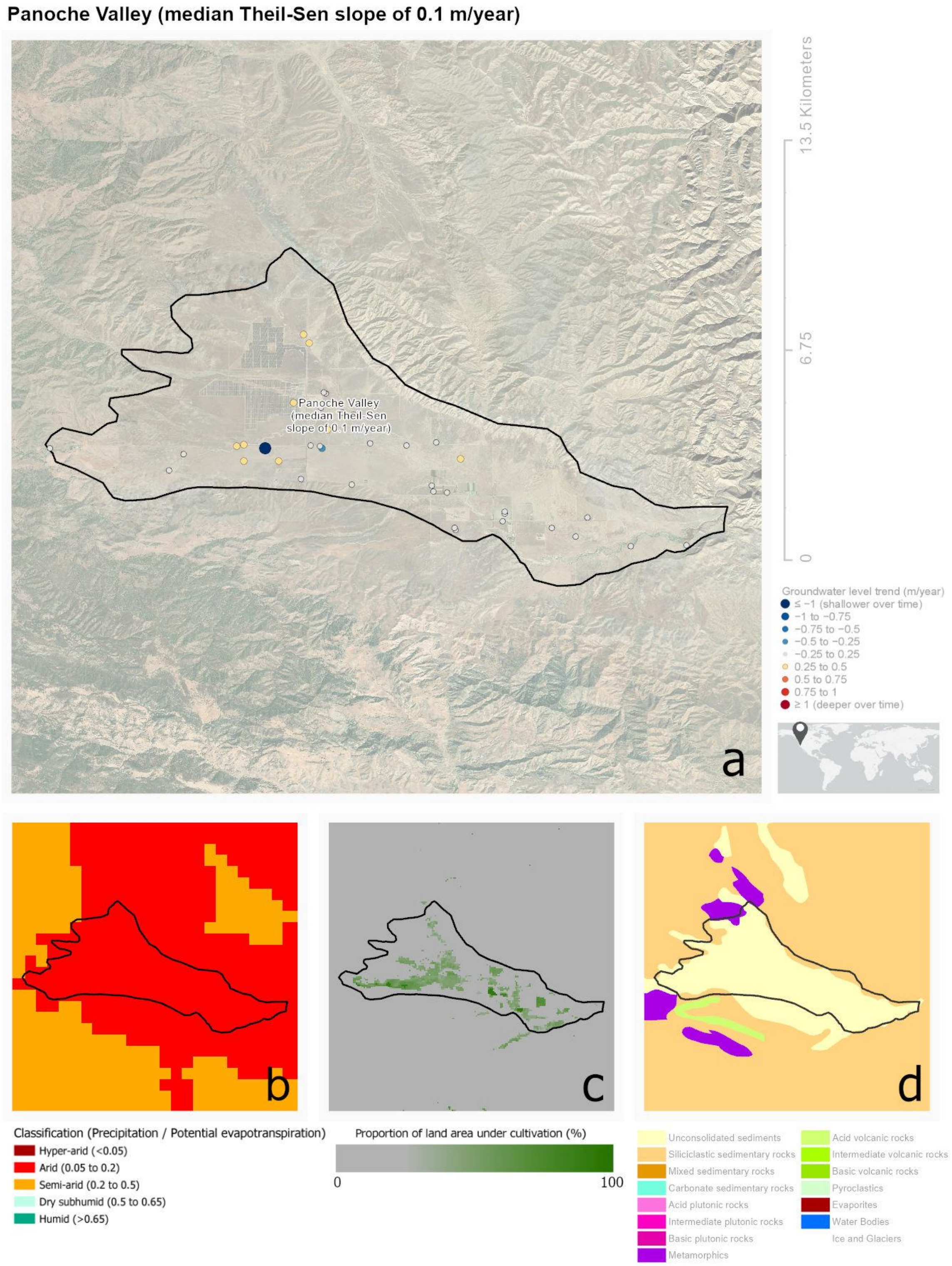

Supplementary Fig. 177. Spatial variability of trends in depth to groundwater Panoche Valley. For detailed description of the contents of each panel see the text at the beginning of Supplementary Note 17.

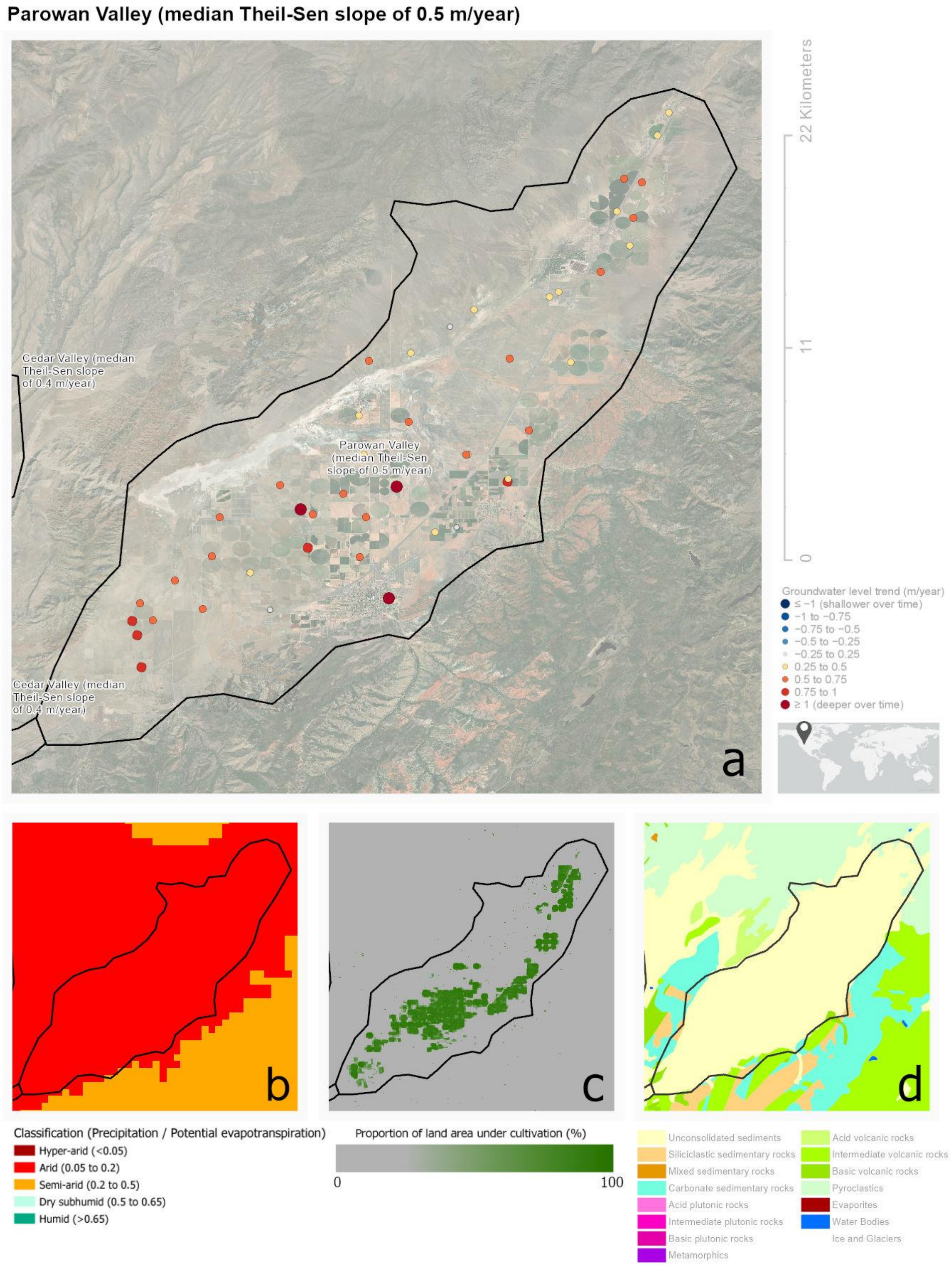

Supplementary Fig. 178. Spatial variability of trends in depth to groundwater Parowan Valley. For detailed description of the contents of each panel see the text at the beginning of Supplementary Note 17.

Pauma Valley (median Theil-Sen slope of 0.2 m/year)

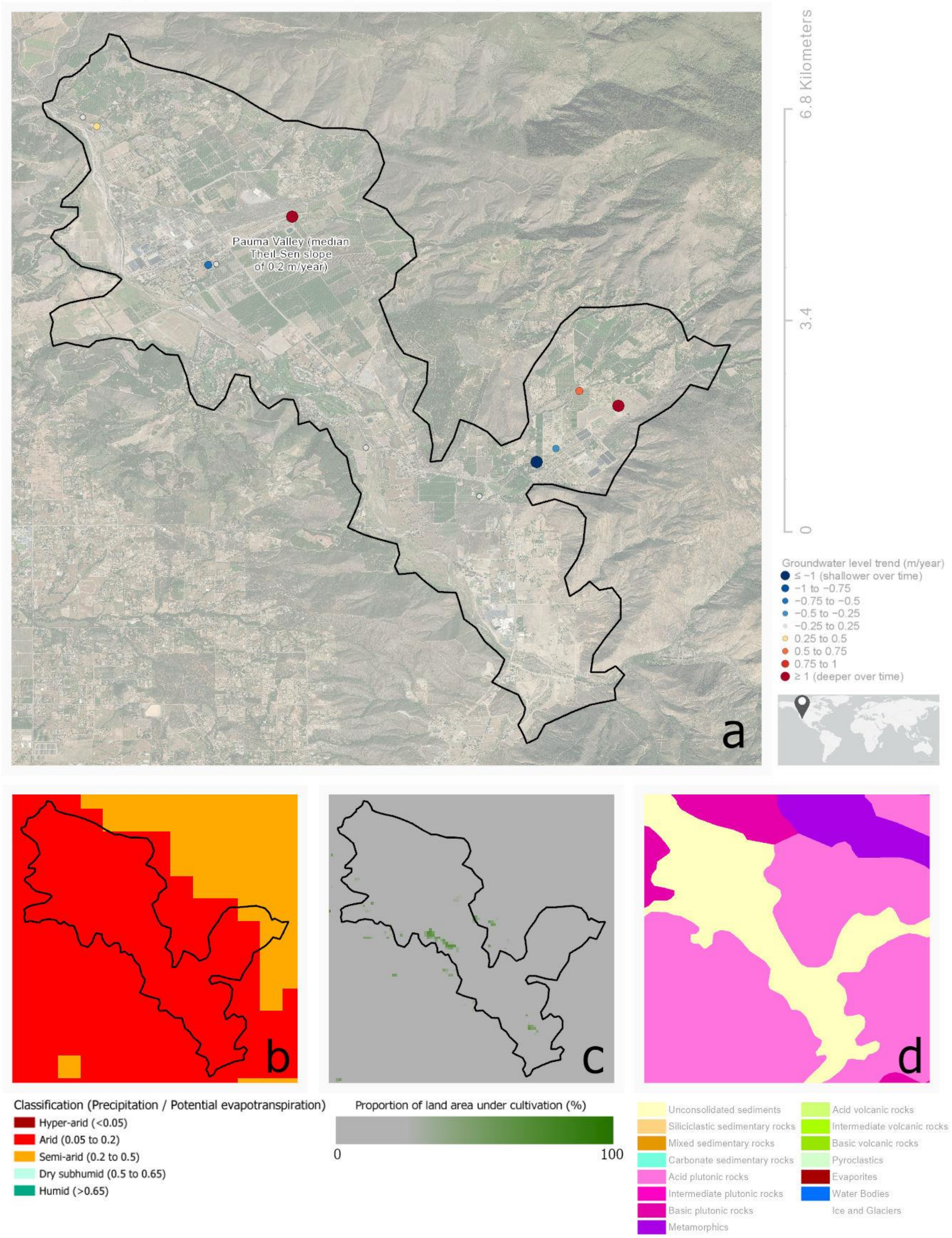

Supplementary Fig. 179. Spatial variability of trends in depth to groundwater Pauma Valley. For detailed description of the contents of each panel see the text at the beginning of Supplementary Note 17.

Petaluma Valley (median Theil-Sen slope of 0 m/year)

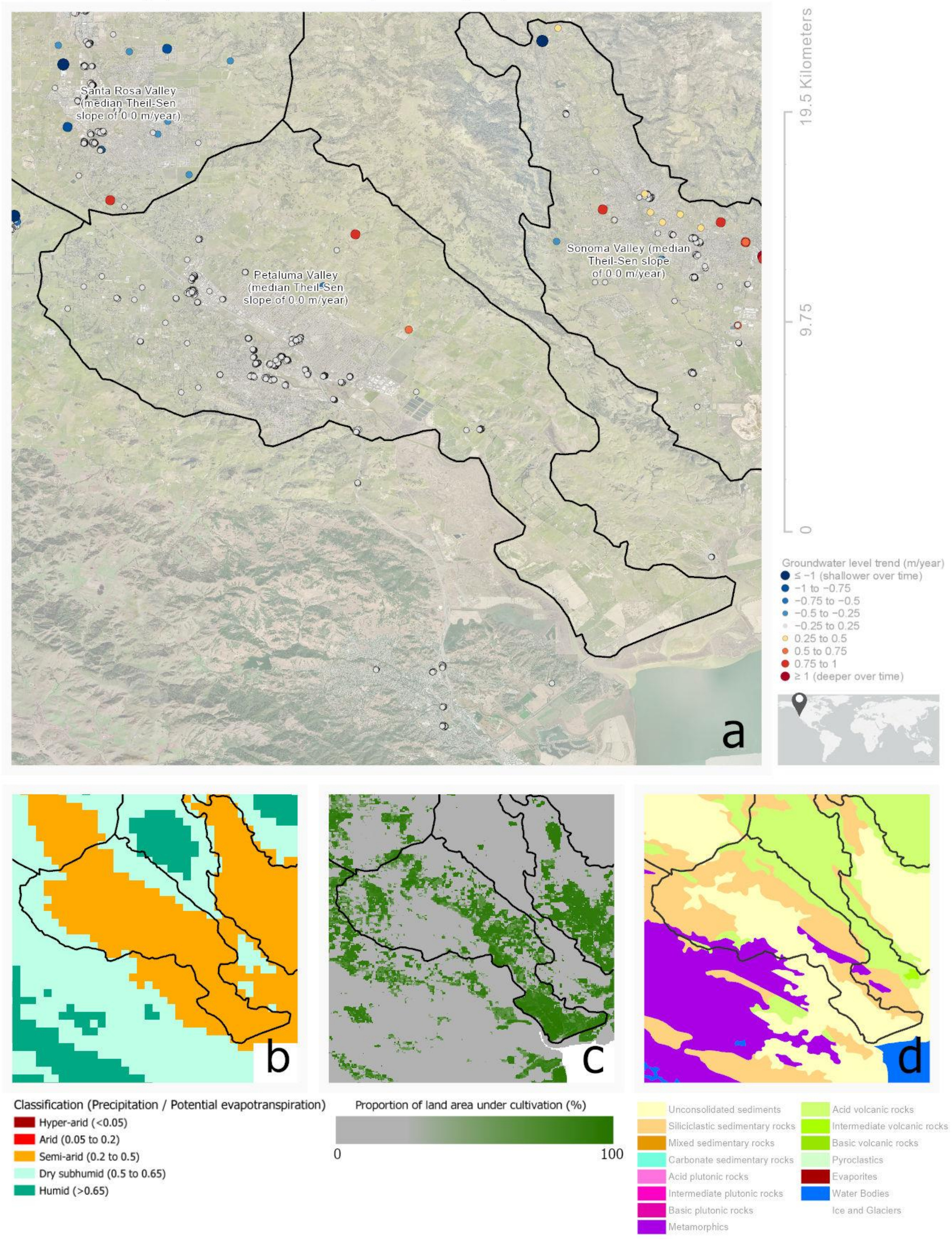

Supplementary Fig. 180. Spatial variability of trends in depth to groundwater Petaluma Valley. For detailed description of the contents of each panel see the text at the beginning of Supplementary Note 17.

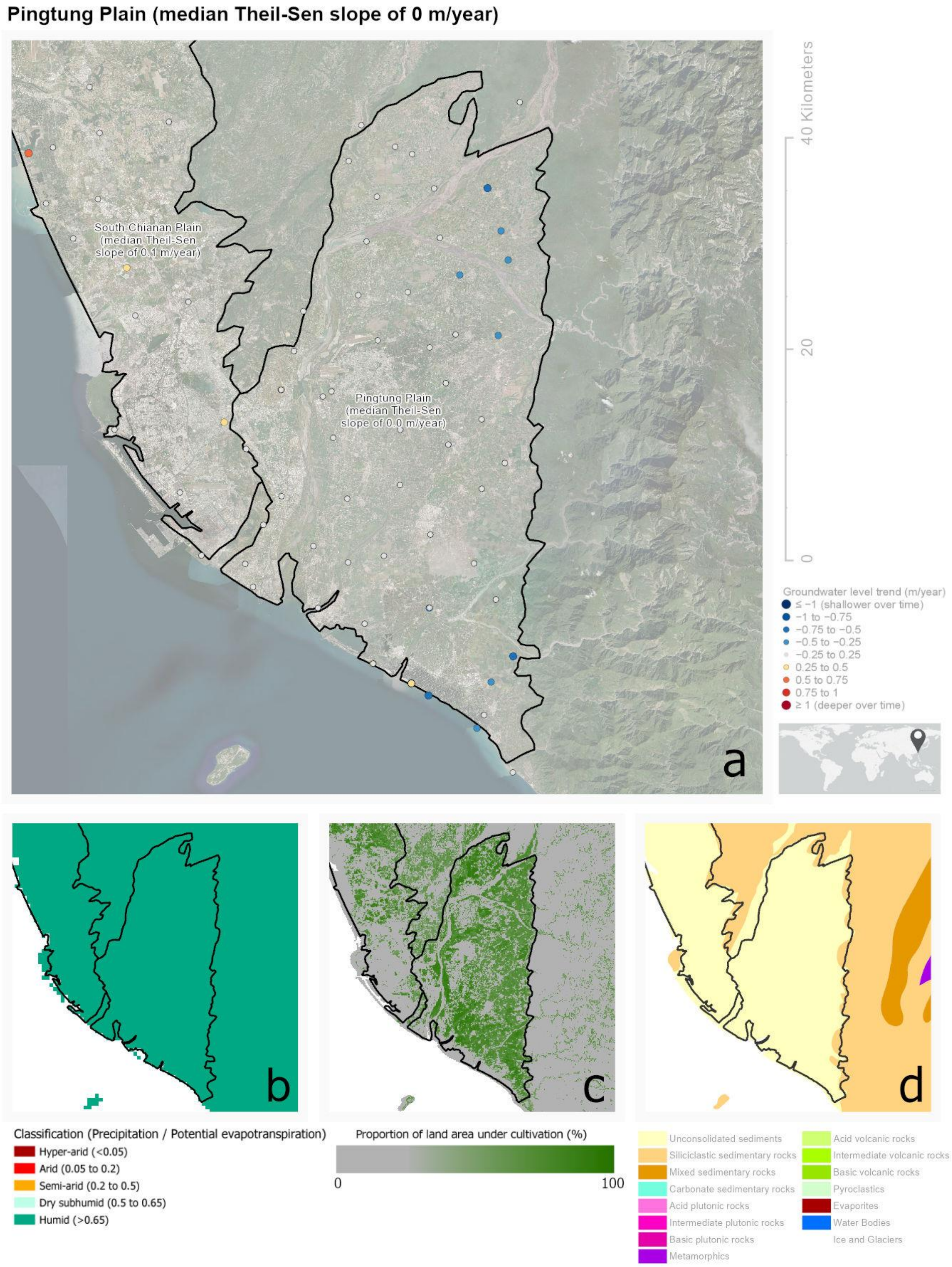

Supplementary Fig. 181. Spatial variability of trends in depth to groundwater Pingtung Plain. For detailed description of the contents of each panel see the text at the beginning of Supplementary Note 17.

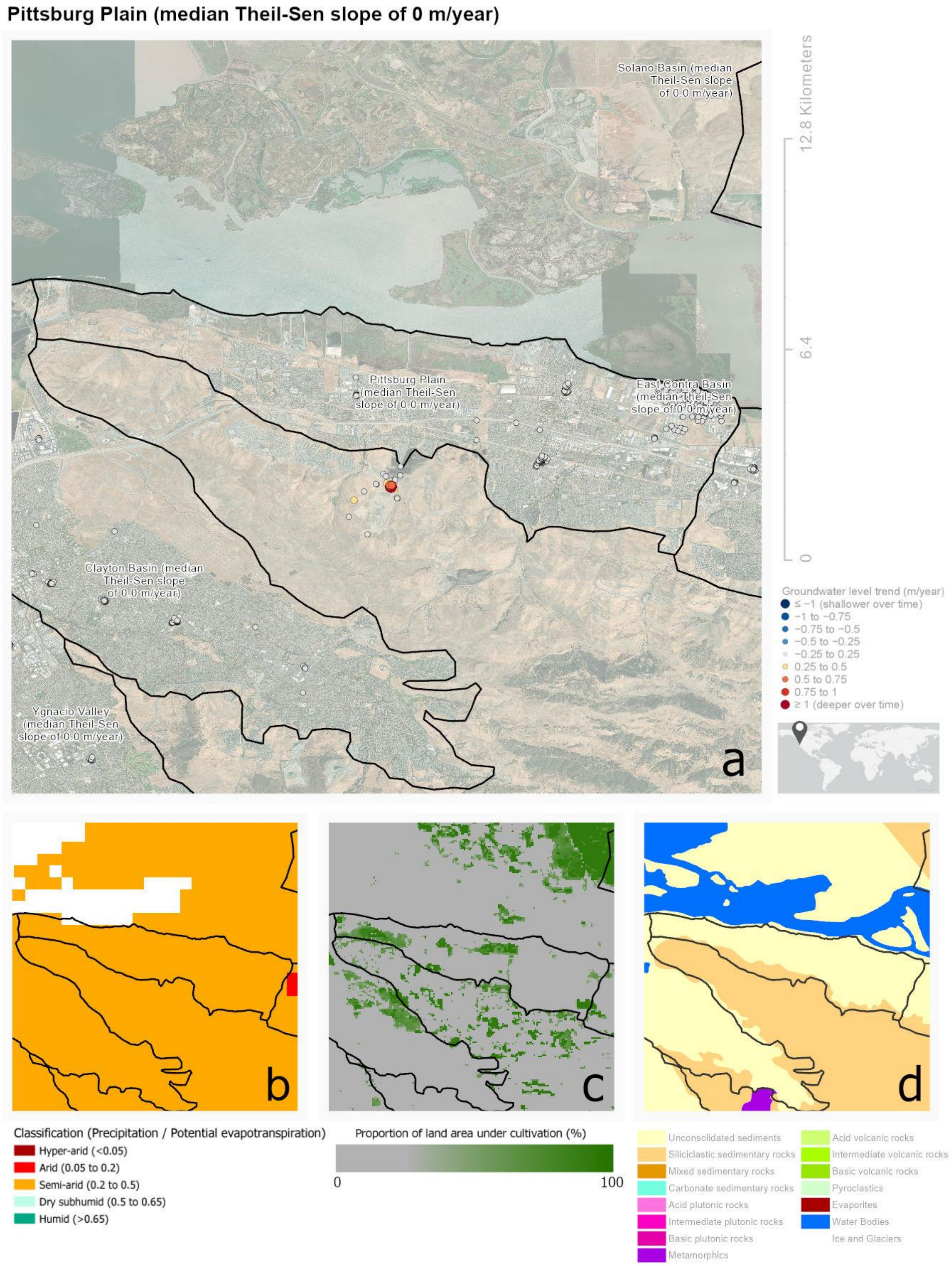

Supplementary Fig. 182. Spatial variability of trends in depth to groundwater Pittsburg Plain. For detailed description of the contents of each panel see the text at the beginning of Supplementary Note 17.

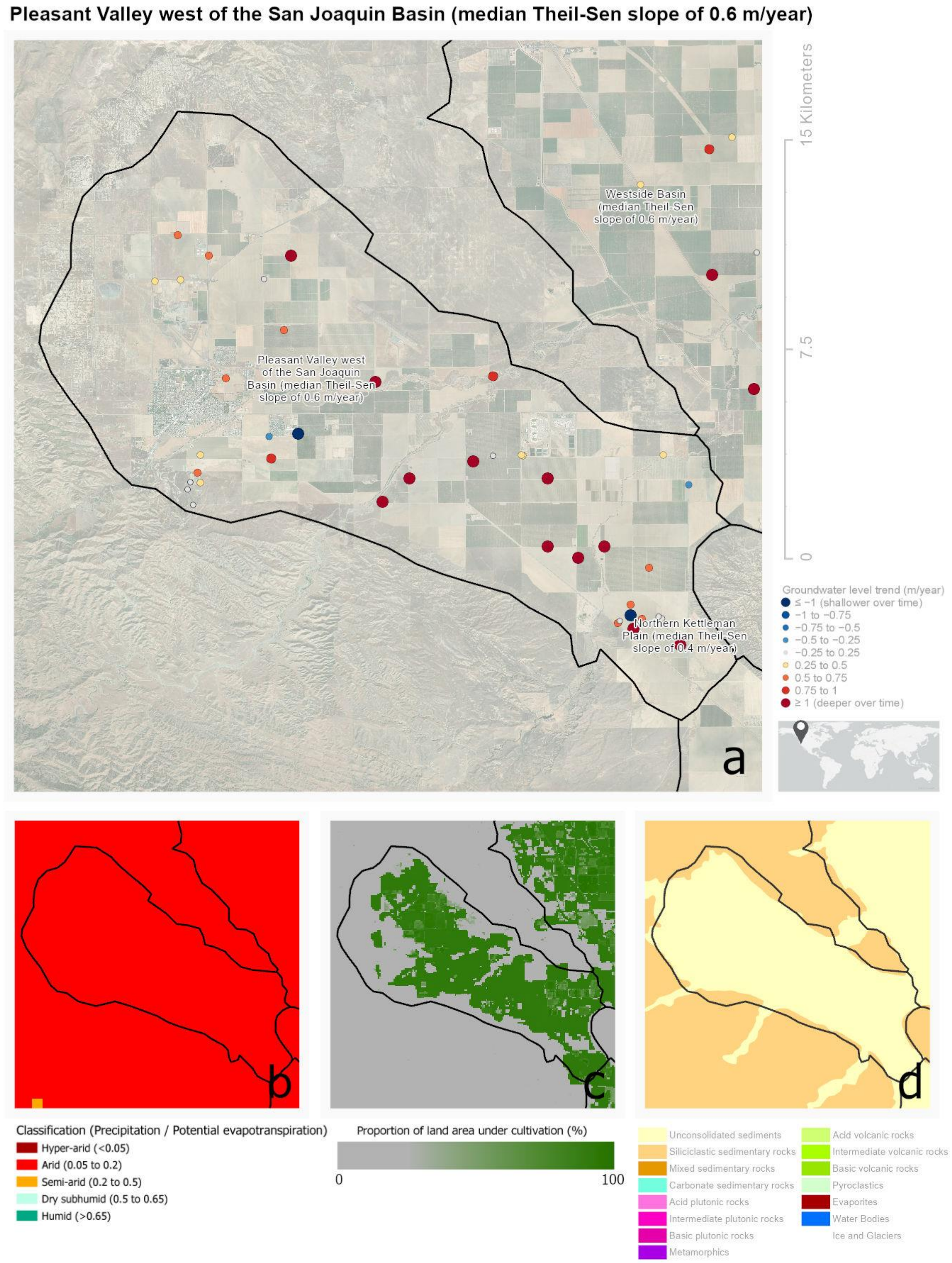

Supplementary Fig. 183. Spatial variability of trends in depth to groundwater Pleasant Valley west of the San Joaquin Basin. For detailed description of the contents of each panel see the text at the beginning of Supplementary Note 17.

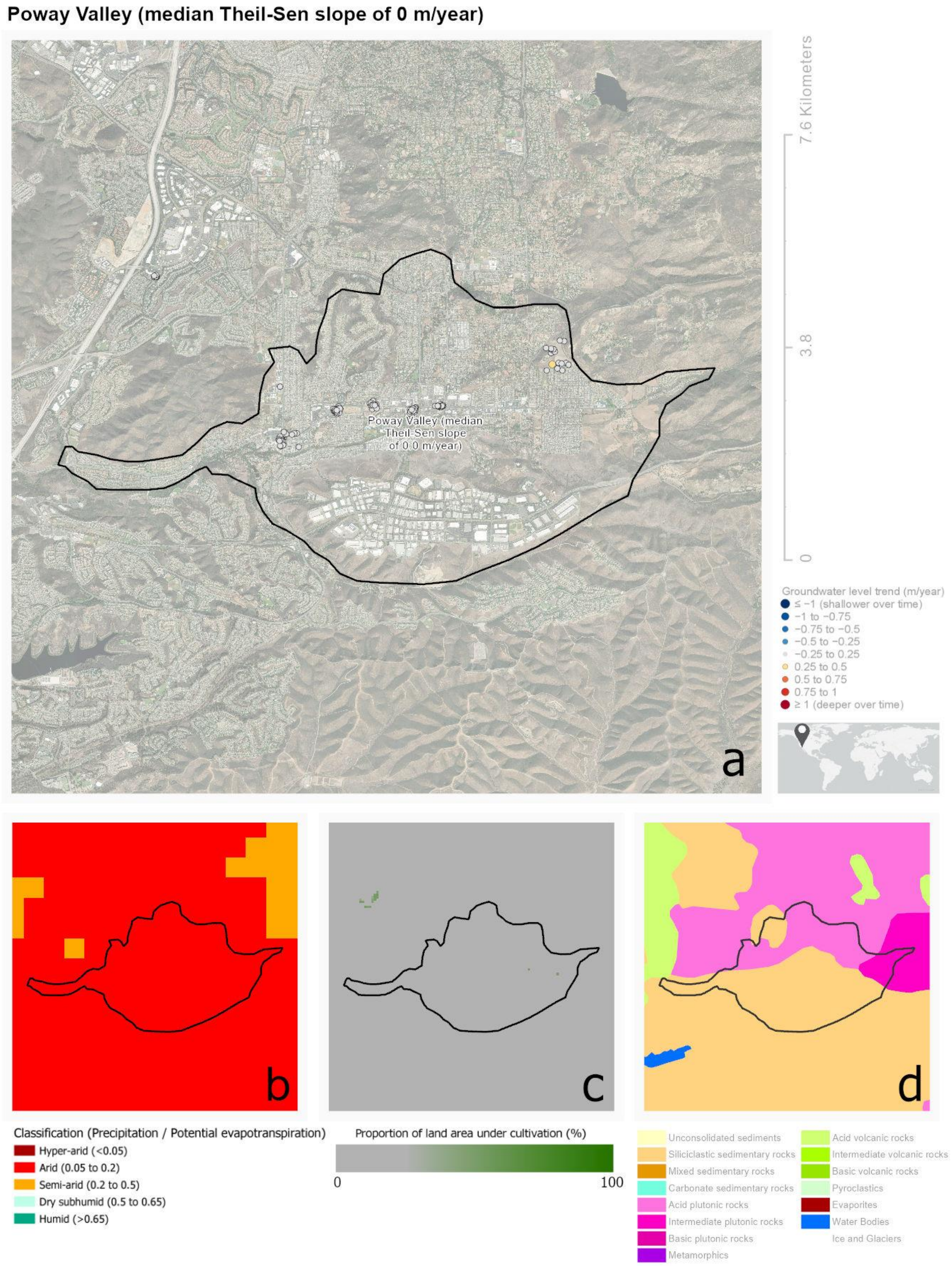

Supplementary Fig. 184. Spatial variability of trends in depth to groundwater Poway Valley. For detailed description of the contents of each panel see the text at the beginning of Supplementary Note 17.

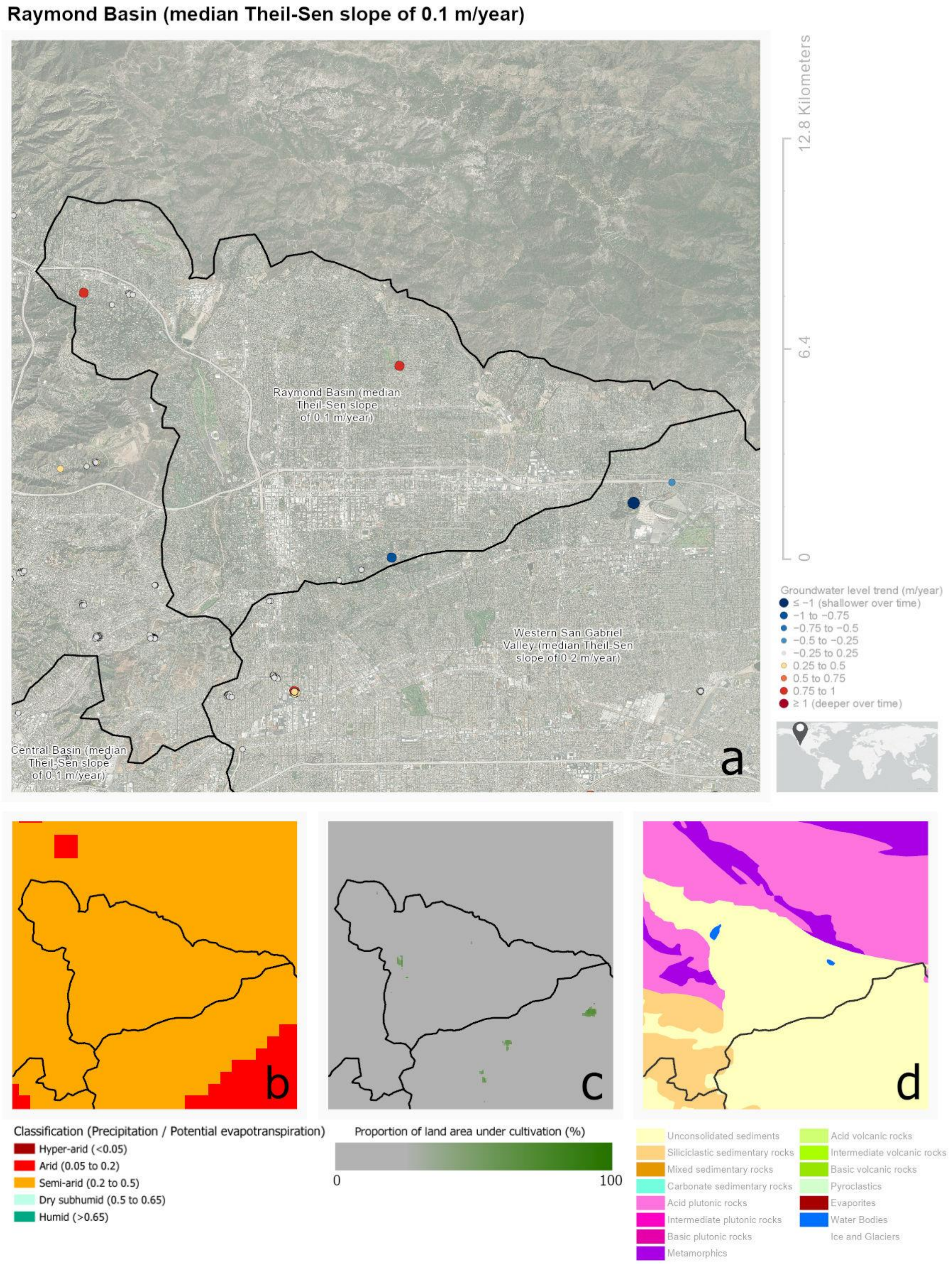

Supplementary Fig. 185. Spatial variability of trends in depth to groundwater Raymond Basin. For detailed description of the contents of each panel see the text at the beginning of Supplementary Note 17.

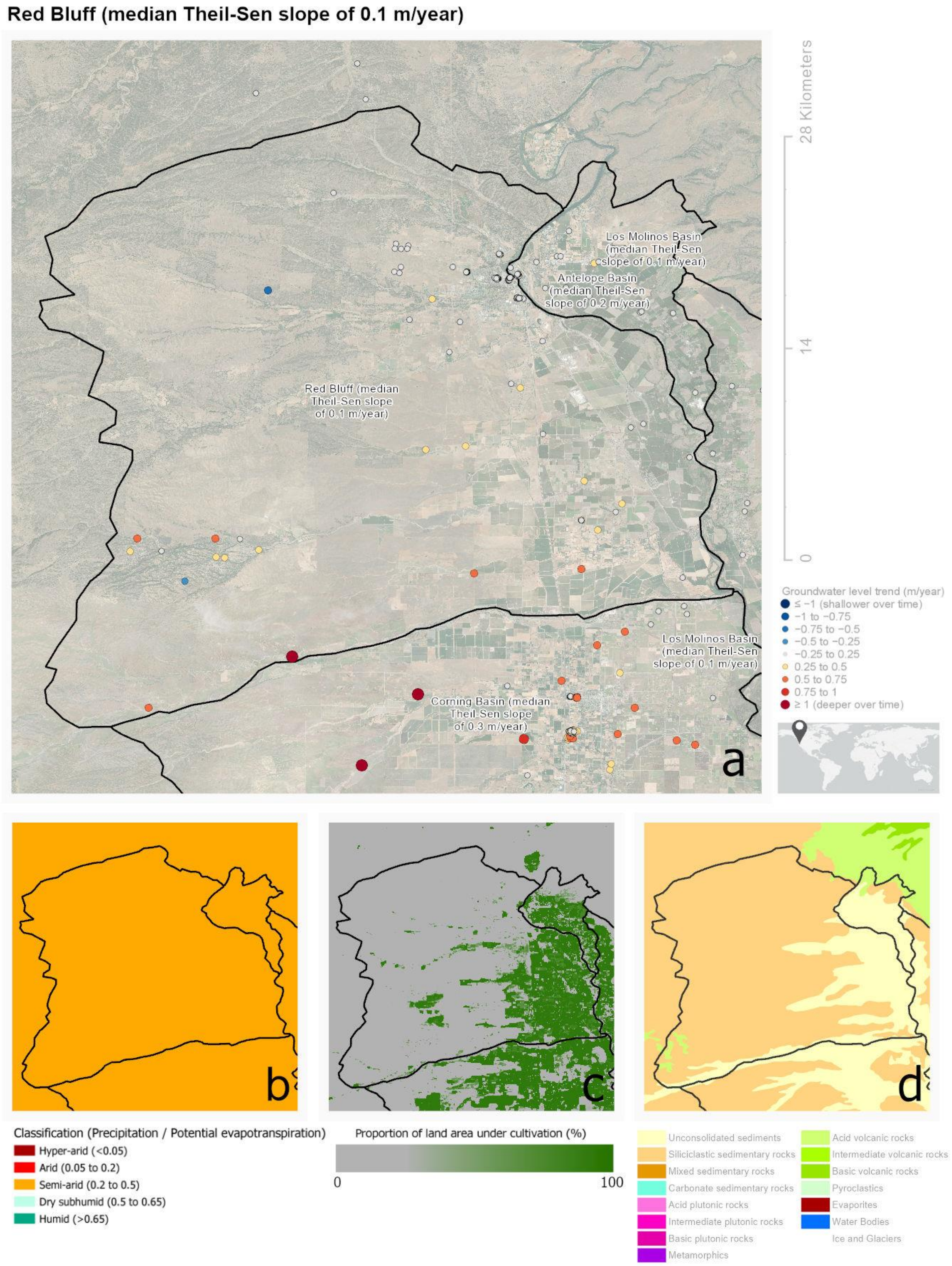

Supplementary Fig. 186. Spatial variability of trends in depth to groundwater Red Bluff (within the broader Sacramento Valley of the Central Valley). For detailed description of the contents of each panel see the text at the beginning of Supplementary Note 17.

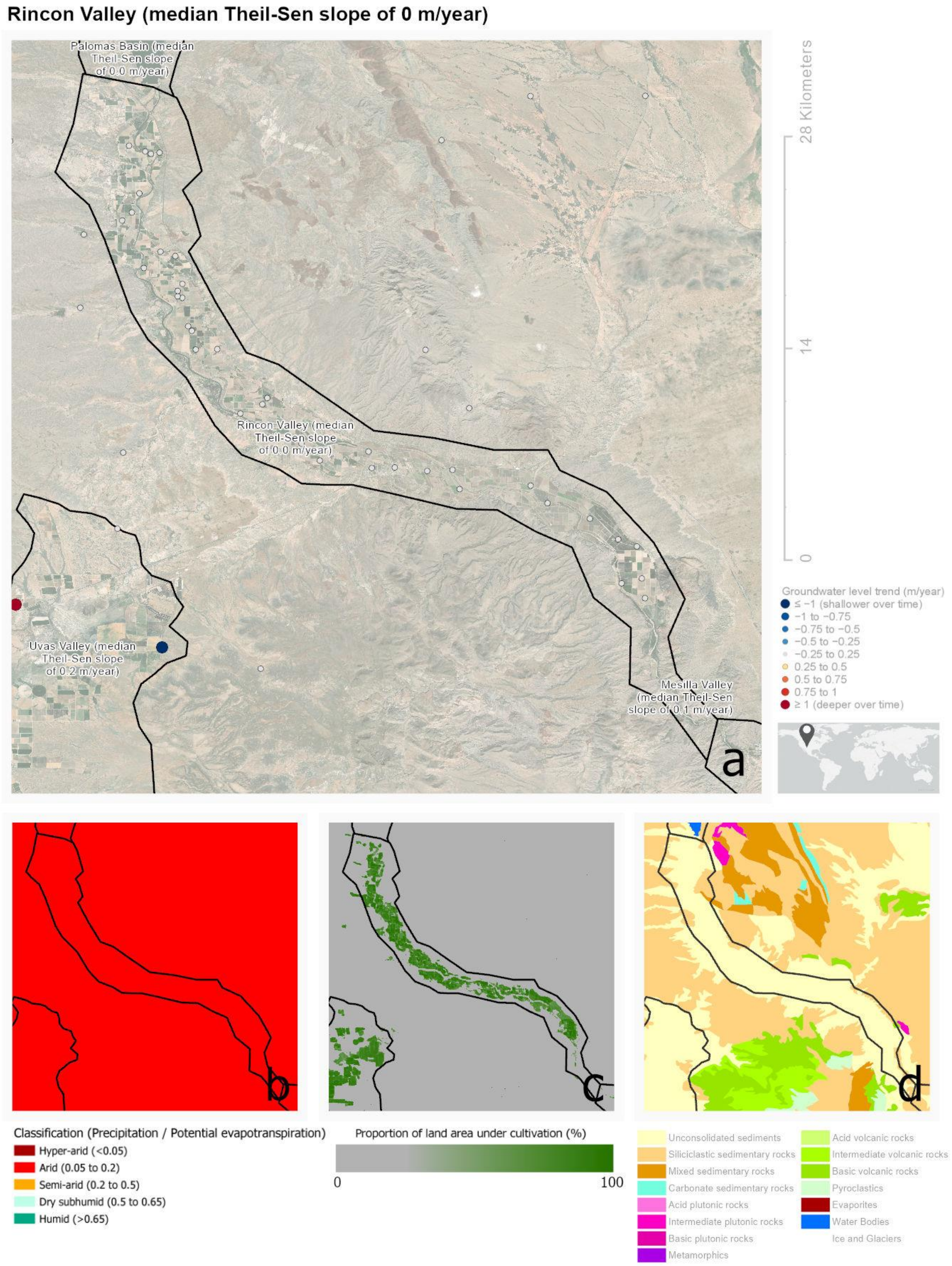

Supplementary Fig. 187. Spatial variability of trends in depth to groundwater Rincon Valley (within the broader Rincon-Mesilla Valleys). For detailed description of the contents of each panel see the text at the beginning of Supplementary Note 17.

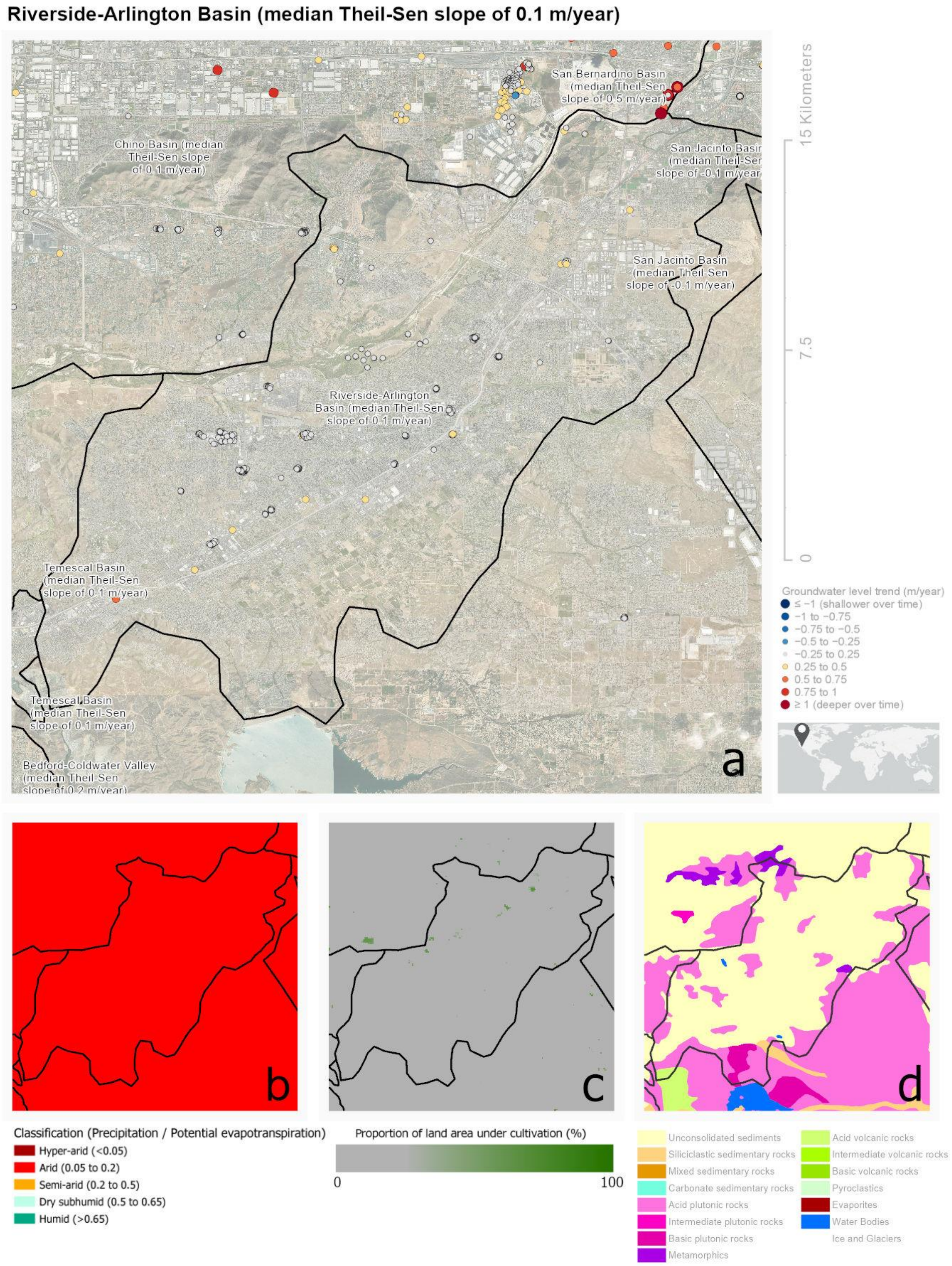

Supplementary Fig. 188. Spatial variability of trends in depth to groundwater Riverside-Arlington Basin (within the broader Upper Santa Ana Basin). For detailed description of the contents of each panel see the text at the beginning of Supplementary Note 17.

Riz Basin (median Theil-Sen slope of 0.7 m/year)

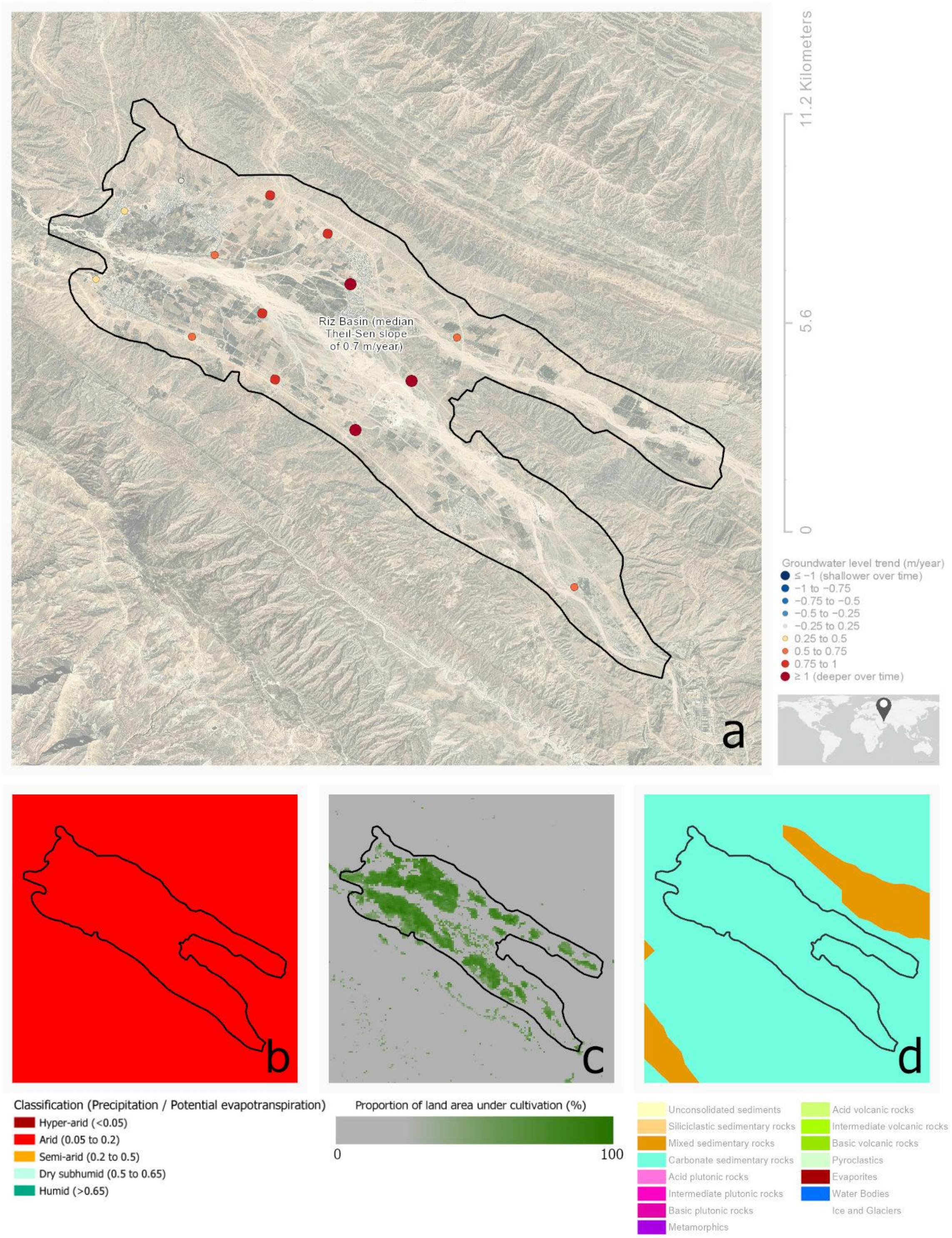

Supplementary Fig. 189. Spatial variability of trends in depth to groundwater Riz Basin. For detailed description of the contents of each panel see the text at the beginning of Supplementary Note 17.

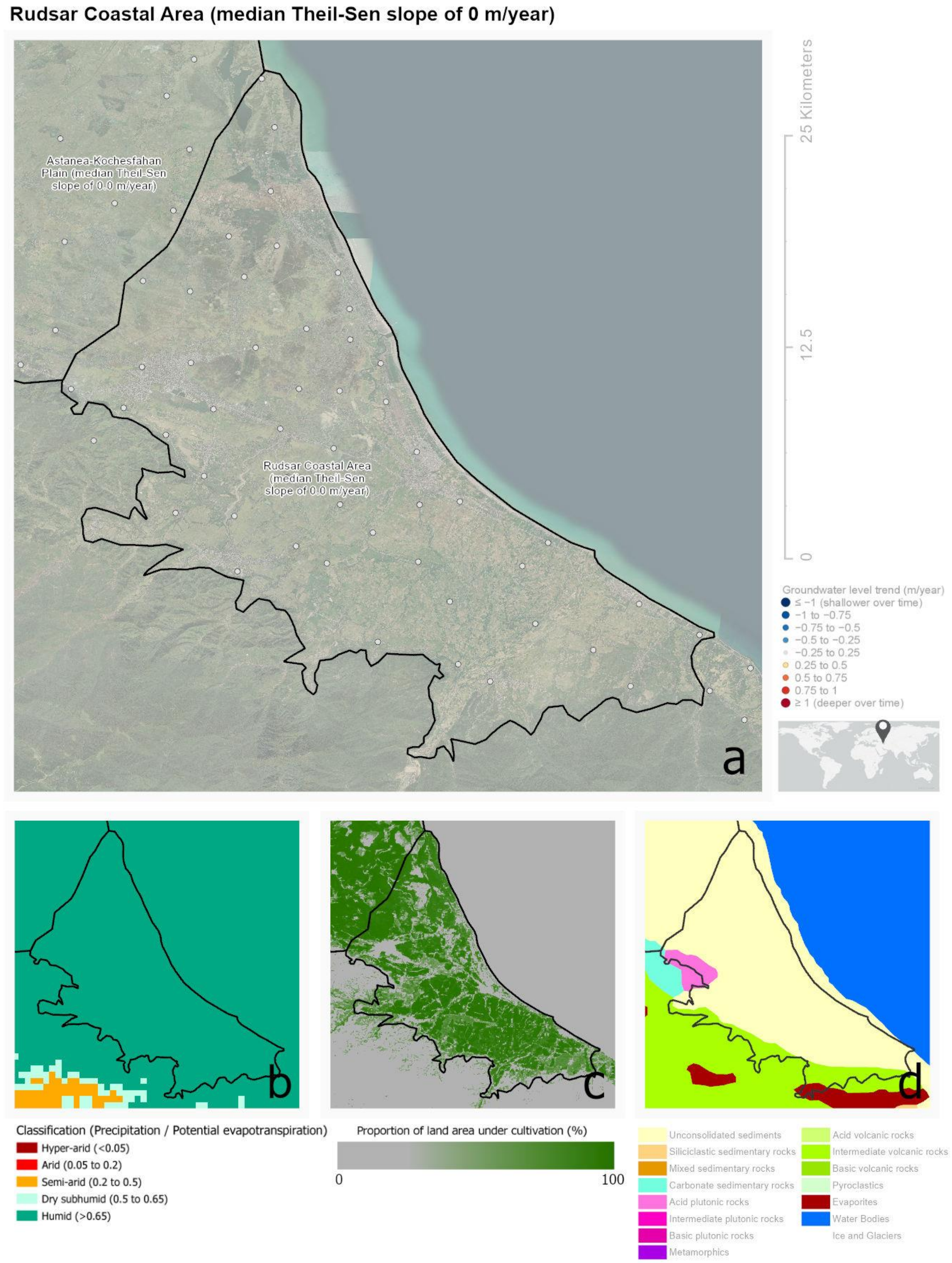

Supplementary Fig. 190. Spatial variability of trends in depth to groundwater Rudsar Coastal Area. For detailed description of the contents of each panel see the text at the beginning of Supplementary Note 17.

Salinas Valley (median Theil-Sen slope of 0 m/year)

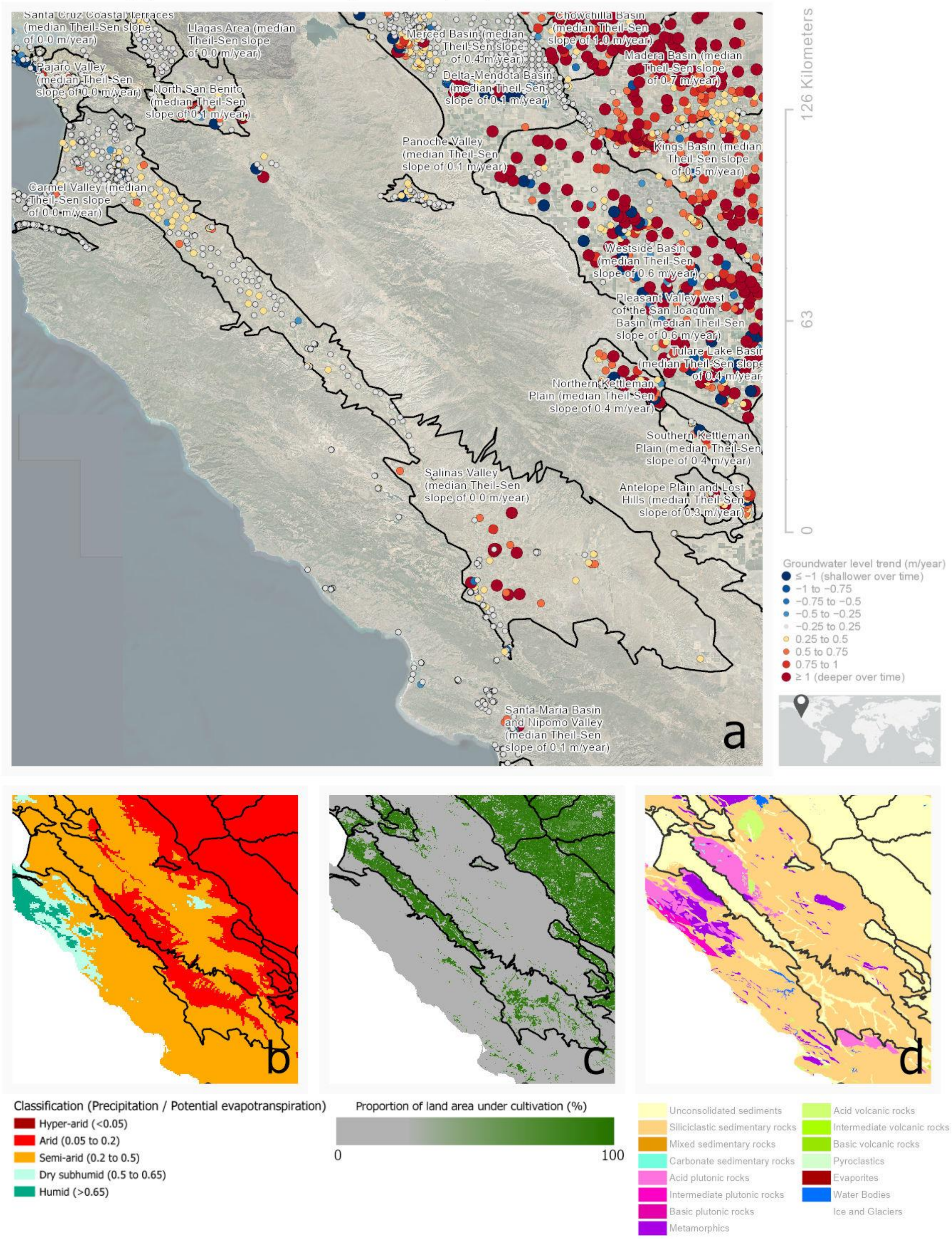

Supplementary Fig. 191. Spatial variability of trends in depth to groundwater Salinas Valley. For detailed description of the contents of each panel see the text at the beginning of Supplementary Note 17.

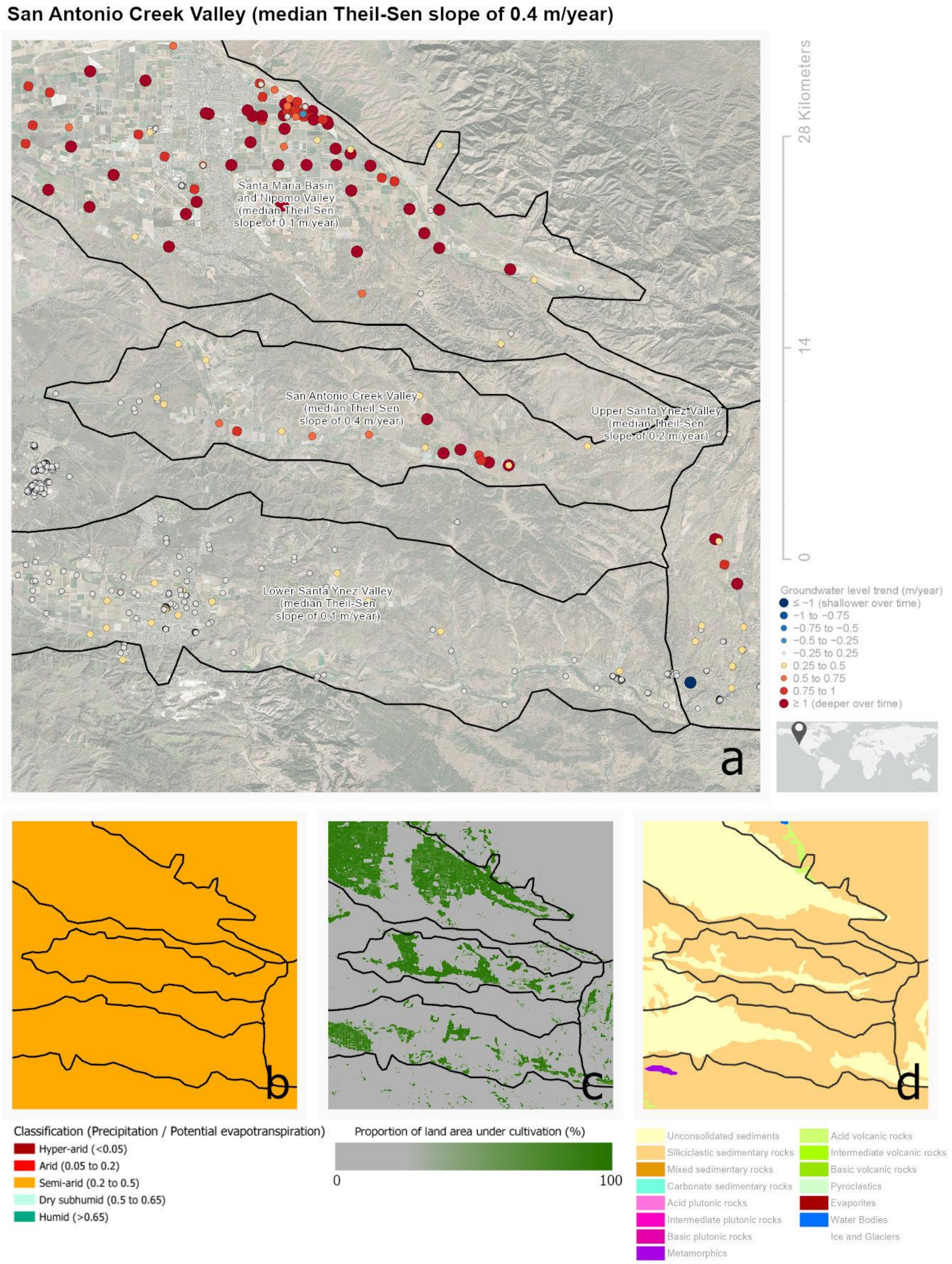

Supplementary Fig. 192. Spatial variability of trends in depth to groundwater San Antonio Creek Valley. For detailed description of the contents of each panel see the text at the beginning of Supplementary Note 17.

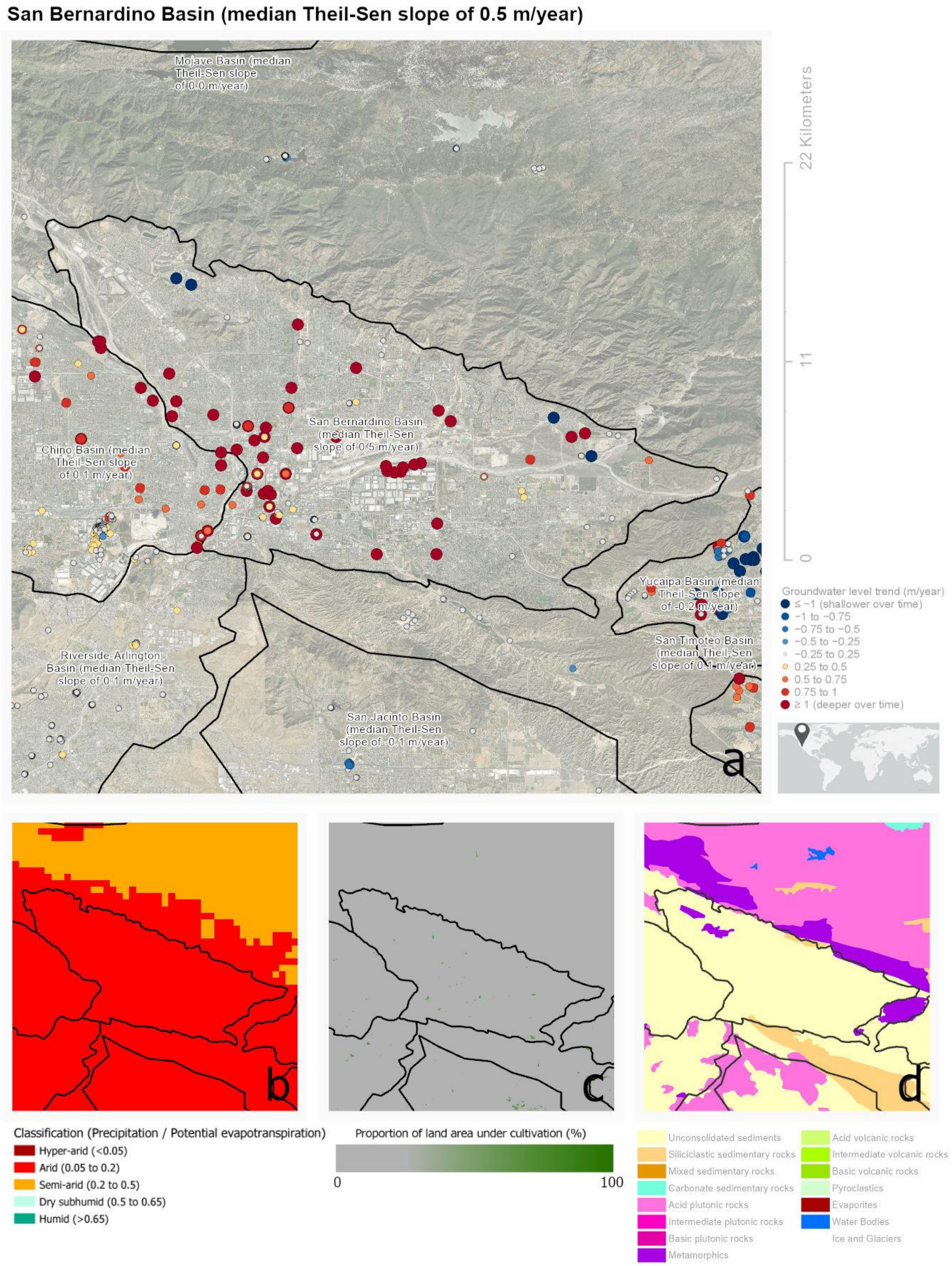

Supplementary Fig. 193. Spatial variability of trends in depth to groundwater San Bernardino Basin (within the broader Upper Santa Ana Basin). For detailed description of the contents of each panel see the text at the beginning of Supplementary Note 17.

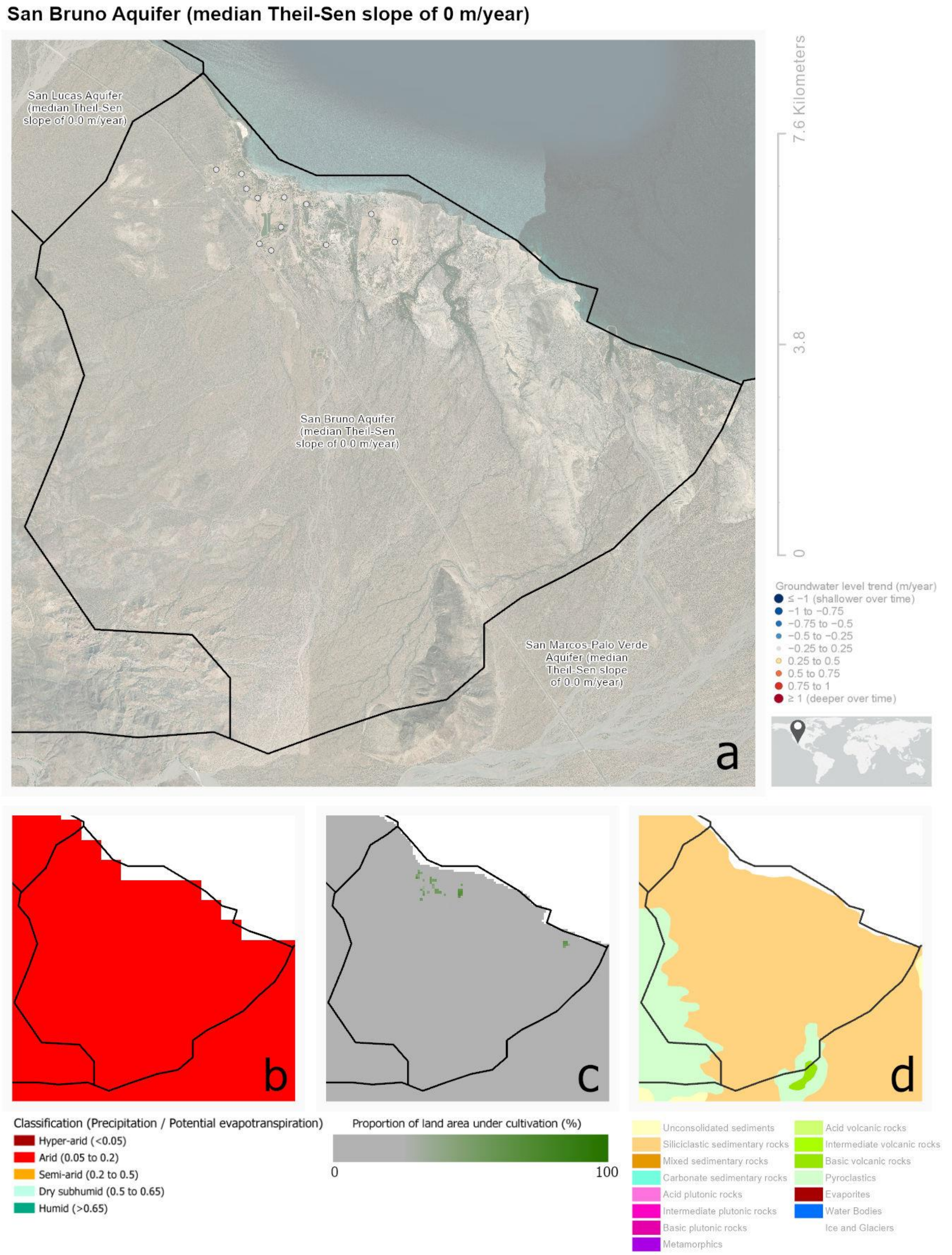

Supplementary Fig. 194. Spatial variability of trends in depth to groundwater San Bruno Aquifer. For detailed description of the contents of each panel see the text at the beginning of Supplementary Note 17.

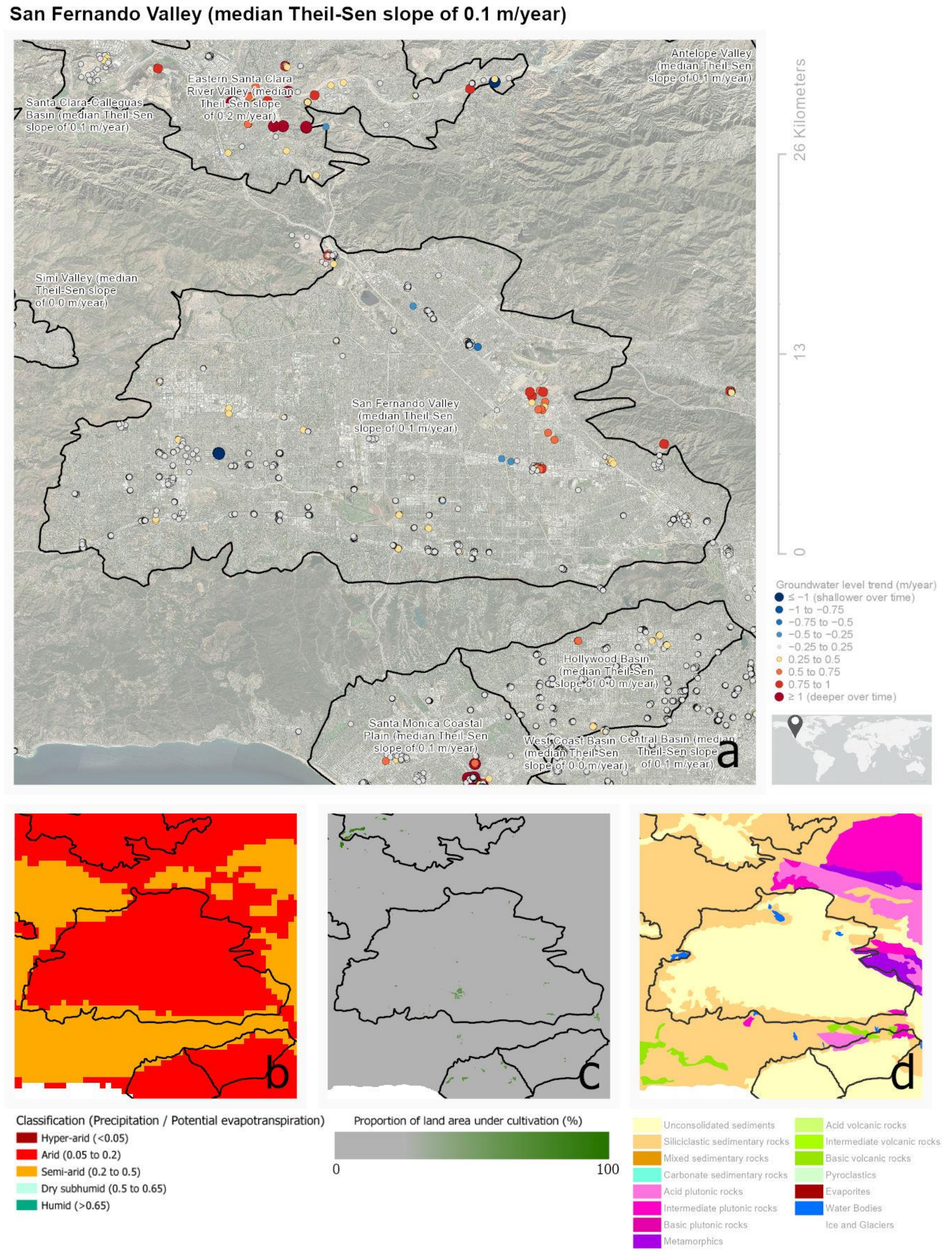

Supplementary Fig. 195. Spatial variability of trends in depth to groundwater San Fernando Valley. For detailed description of the contents of each panel see the text at the beginning of Supplementary Note 17.

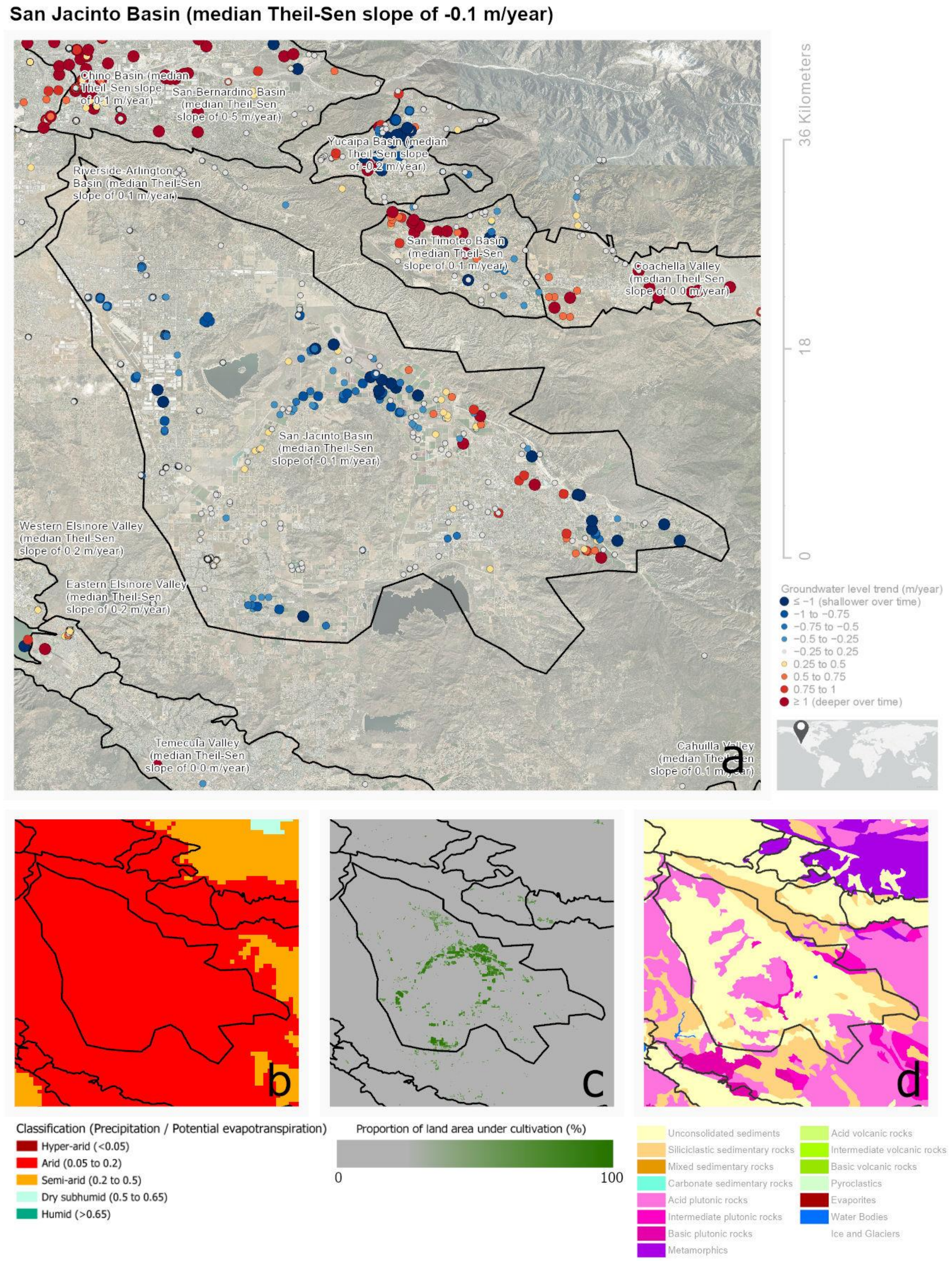

Supplementary Fig. 196. Spatial variability of trends in depth to groundwater San Jacinto Basin. For detailed description of the contents of each panel see the text at the beginning of Supplementary Note 17.

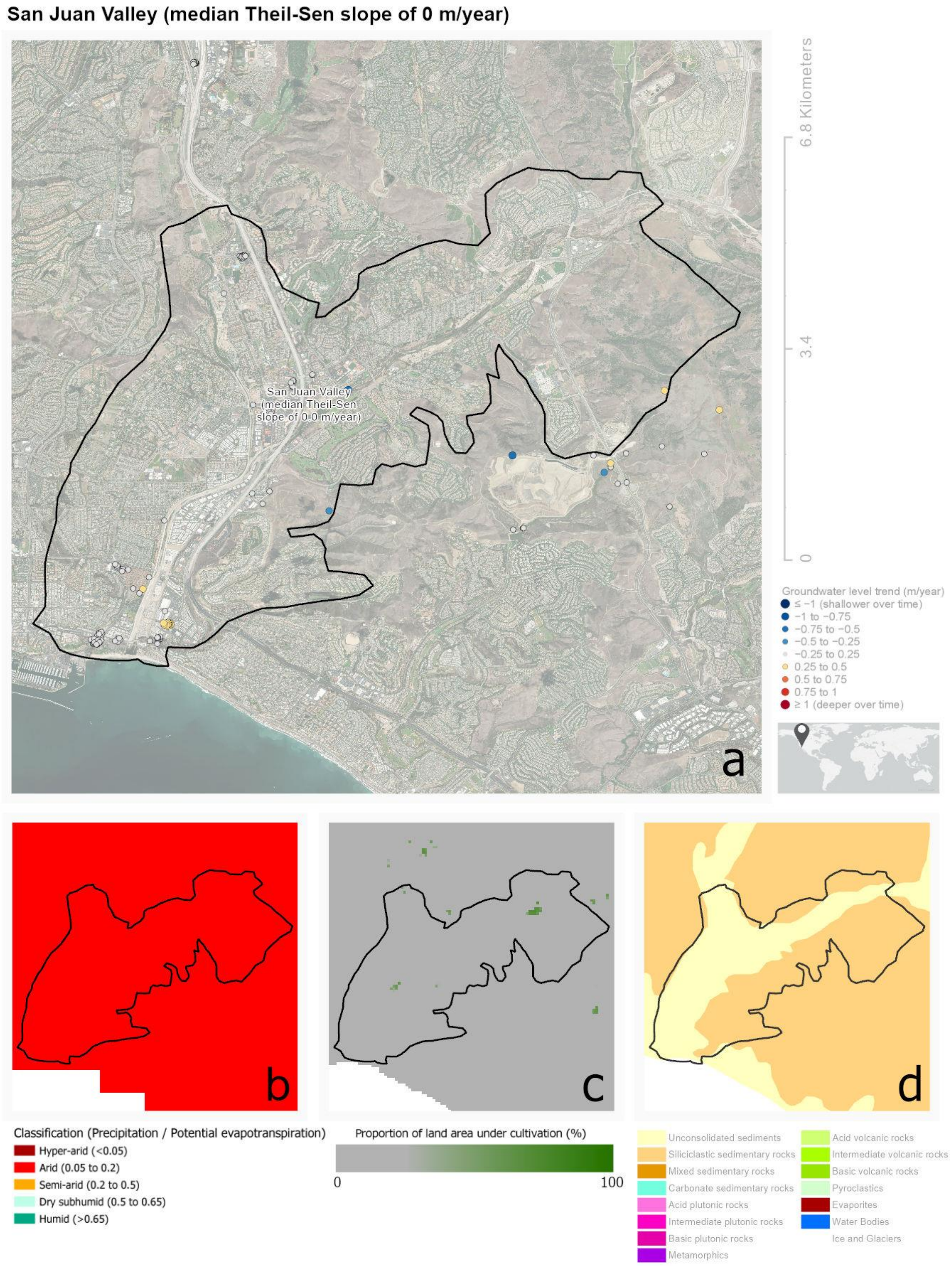

Supplementary Fig. 197. Spatial variability of trends in depth to groundwater San Juan Valley. For detailed description of the contents of each panel see the text at the beginning of Supplementary Note 17.

San Luis Rey Valley (median Theil-Sen slope of 0.1 m/year)

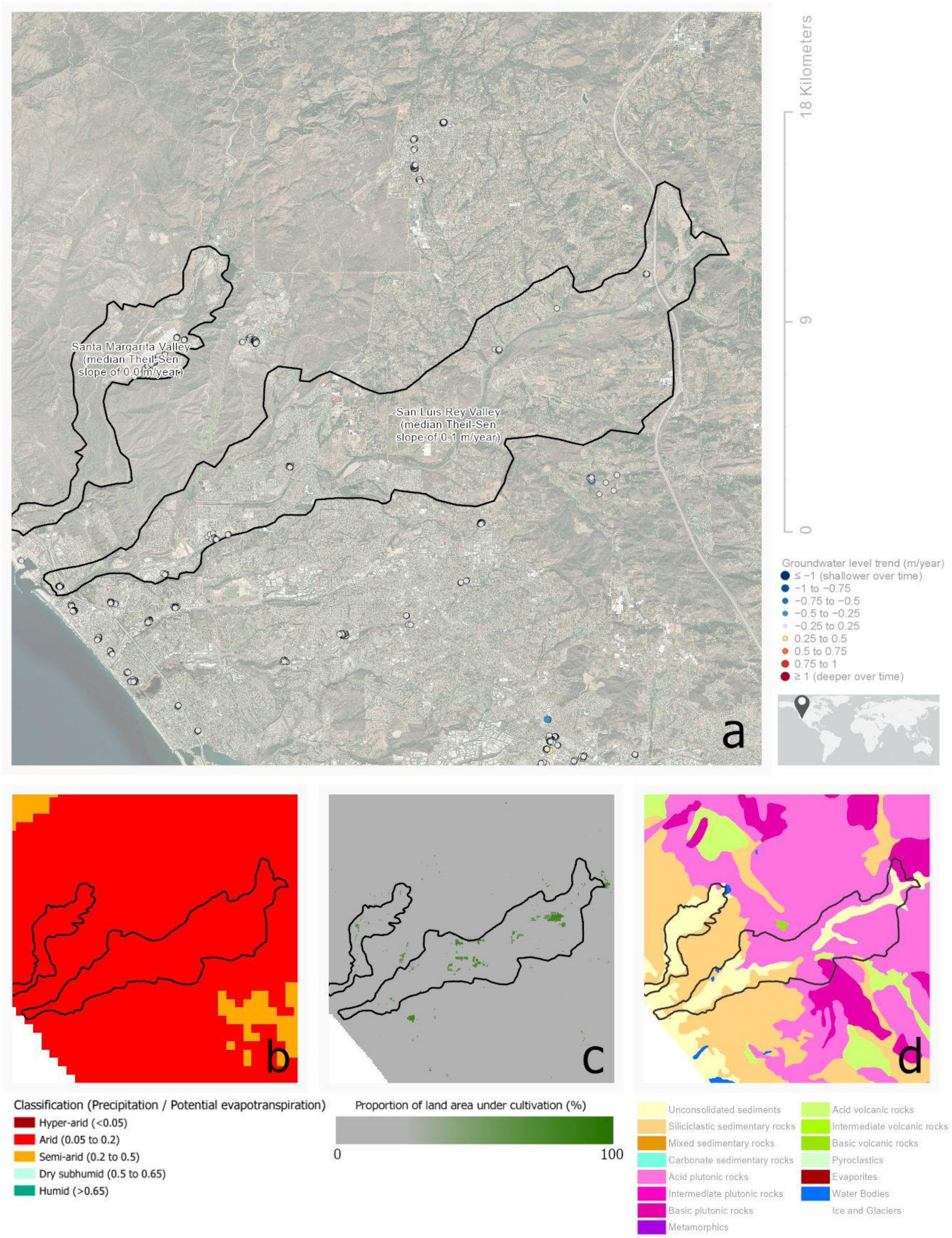

Supplementary Fig. 198. Spatial variability of trends in depth to groundwater San Luis Rey Valley. For detailed description of the contents of each panel see the text at the beginning of Supplementary Note 17.

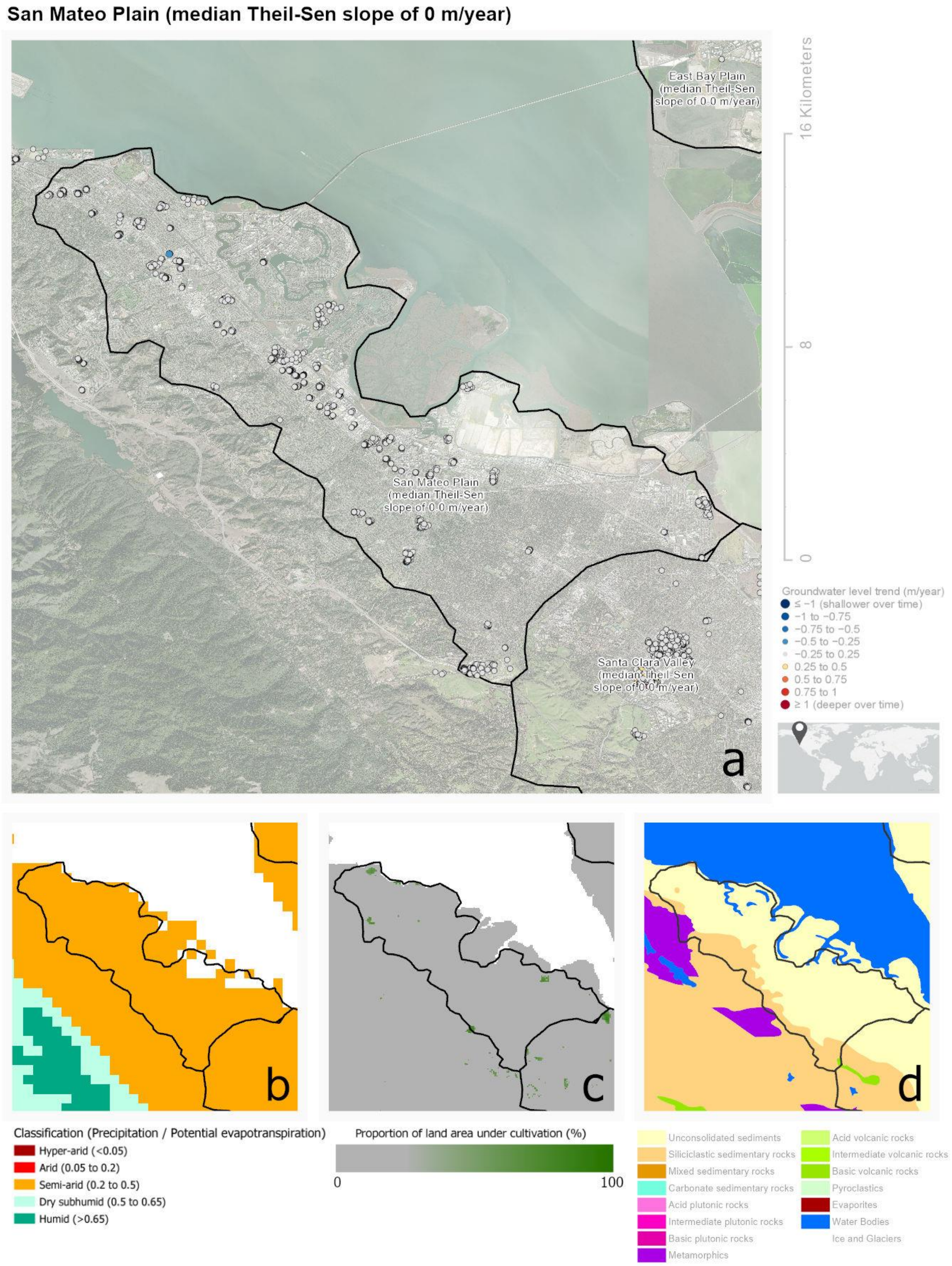

Supplementary Fig. 199. Spatial variability of trends in depth to groundwater San Mateo Plain. For detailed description of the contents of each panel see the text at the beginning of Supplementary Note 17.

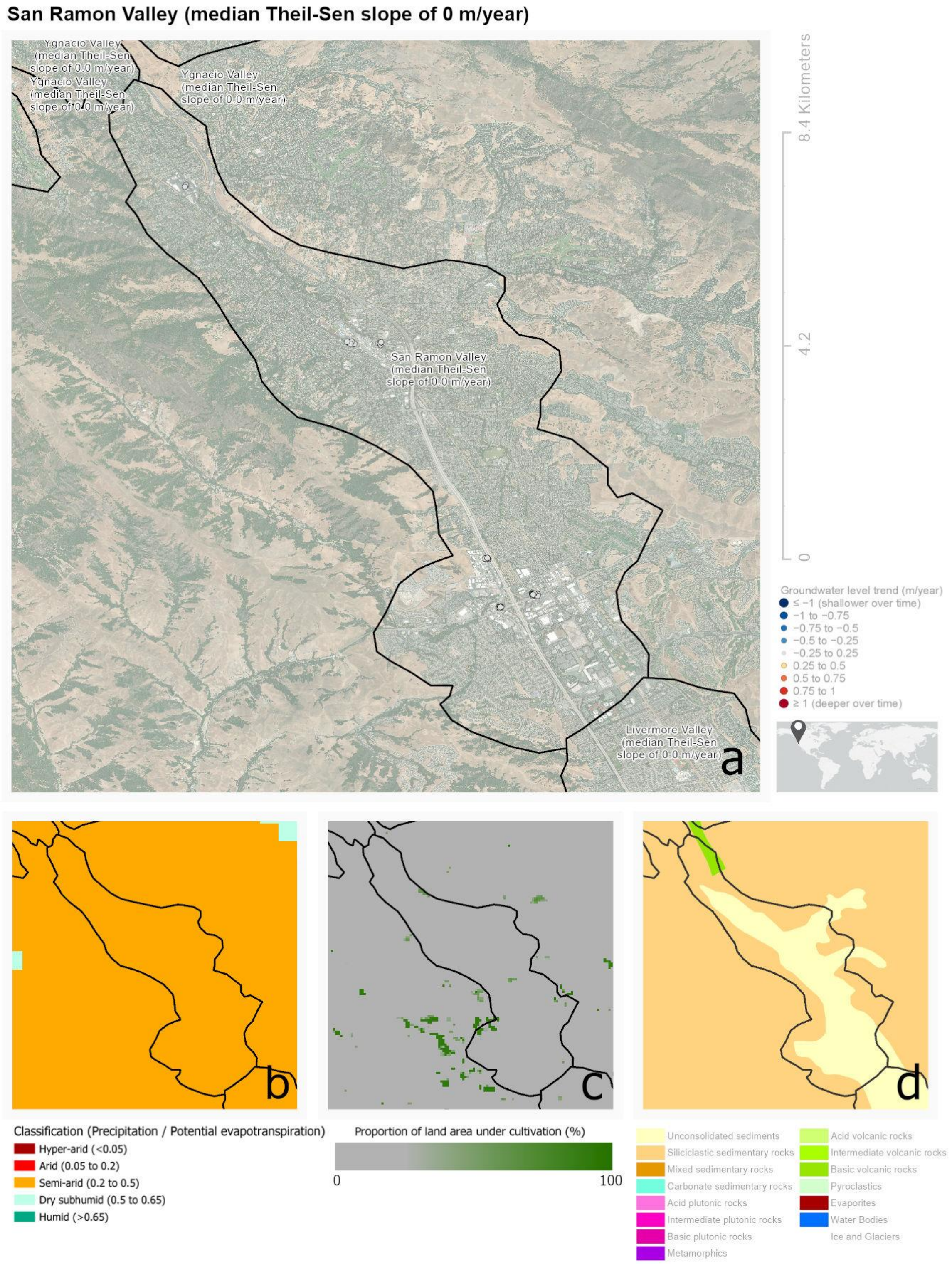

Supplementary Fig. 200. Spatial variability of trends in depth to groundwater San Ramon Valley. For detailed description of the contents of each panel see the text at the beginning of Supplementary Note 17.

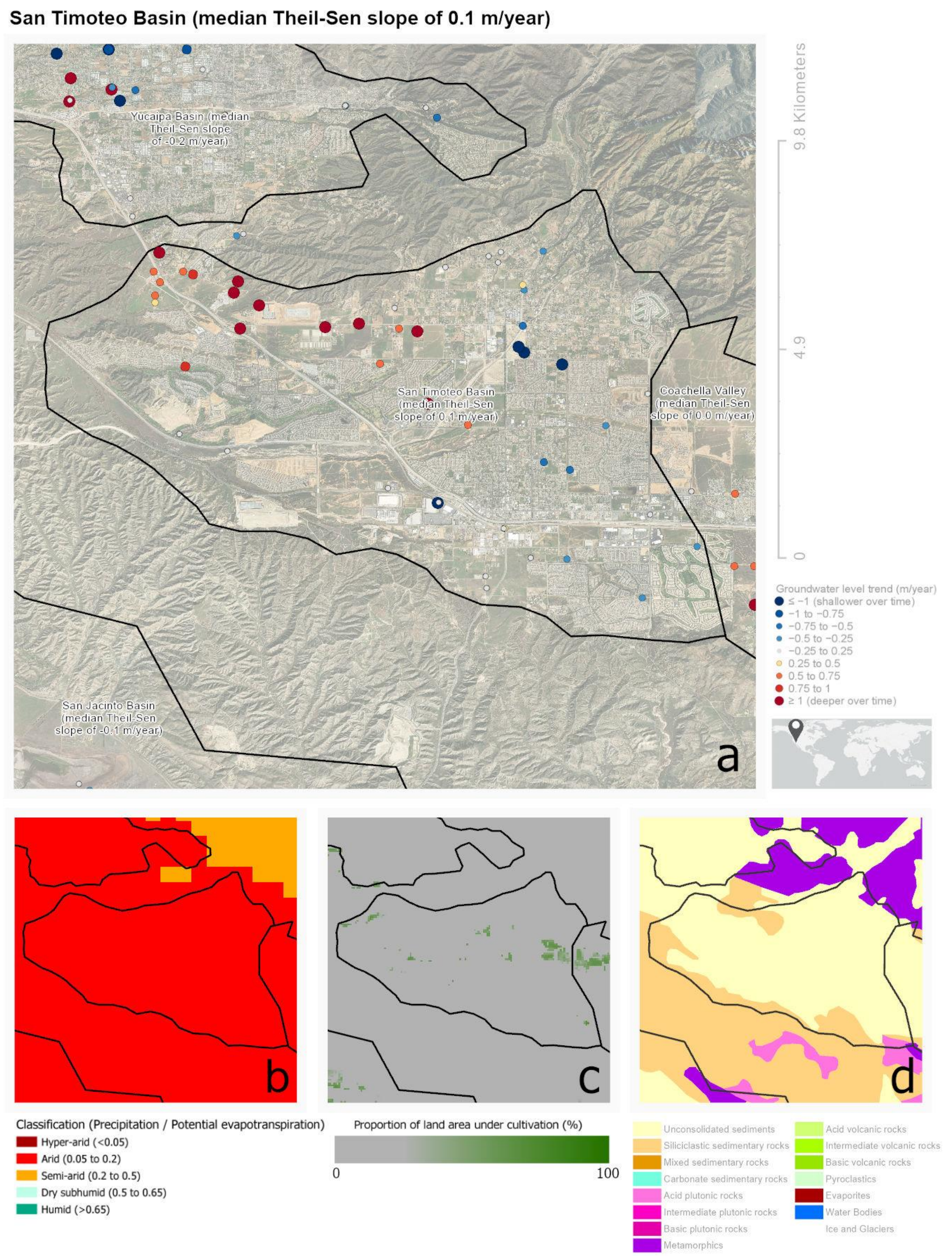

Supplementary Fig. 201. Spatial variability of trends in depth to groundwater San Timoteo Basin. For detailed description of the contents of each panel see the text at the beginning of Supplementary Note 17.

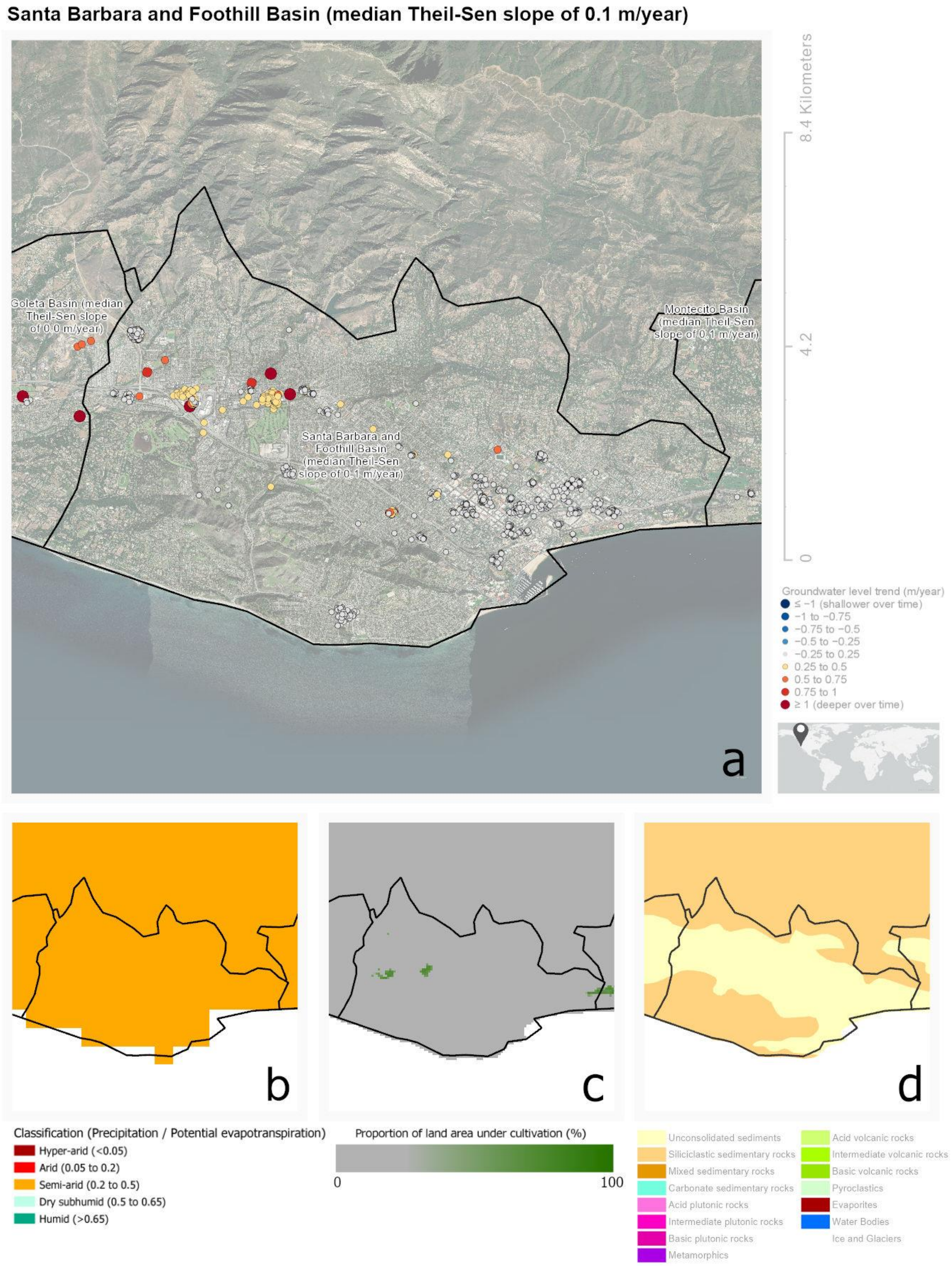

Supplementary Fig. 202. Spatial variability of trends in depth to groundwater Santa Barbara and Foothill Basin. For detailed description of the contents of each panel see the text at the beginning of Supplementary Note 17.

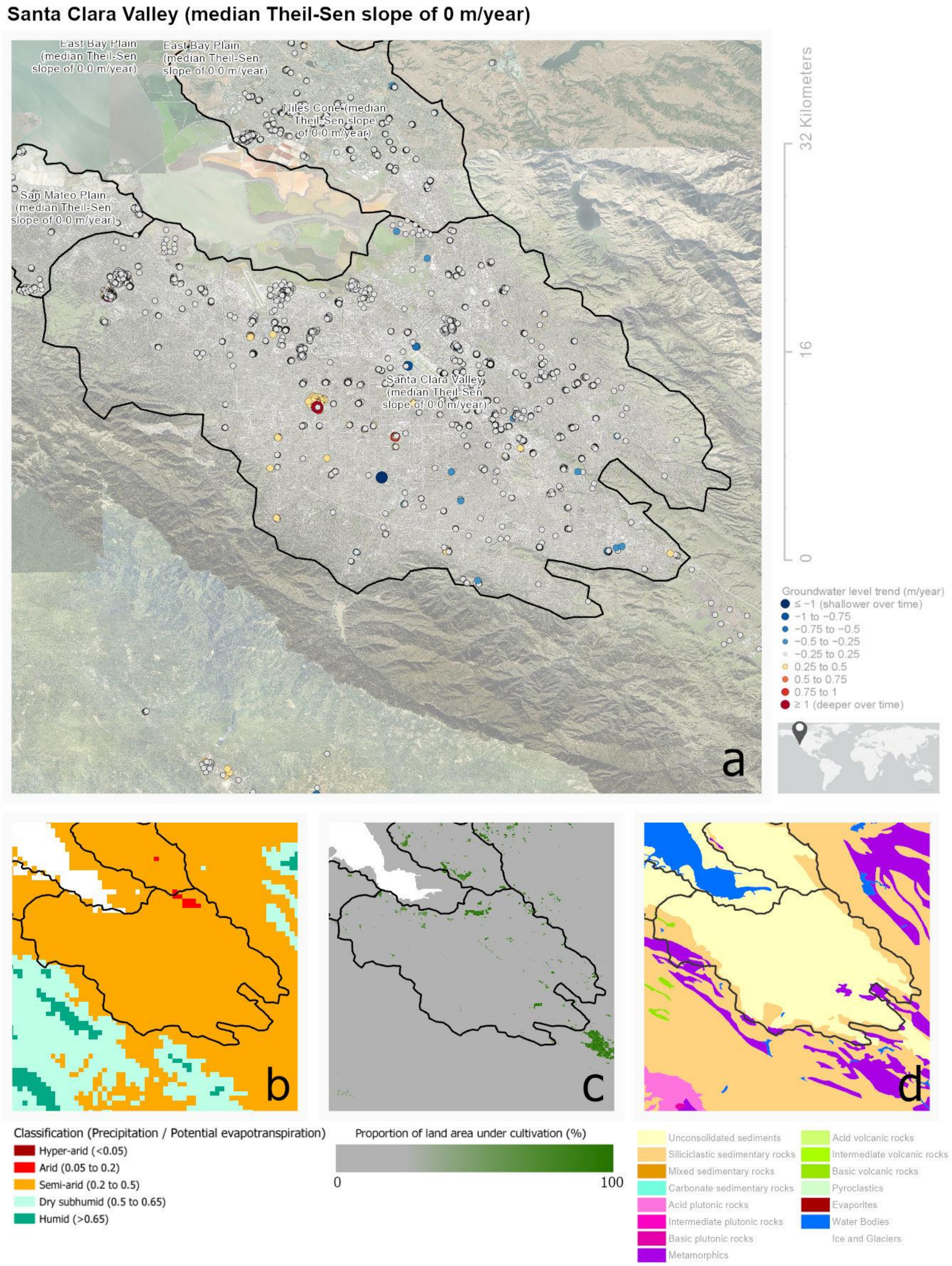

Supplementary Fig. 203. Spatial variability of trends in depth to groundwater Santa Clara Valley. For detailed description of the contents of each panel see the text at the beginning of Supplementary Note 17.

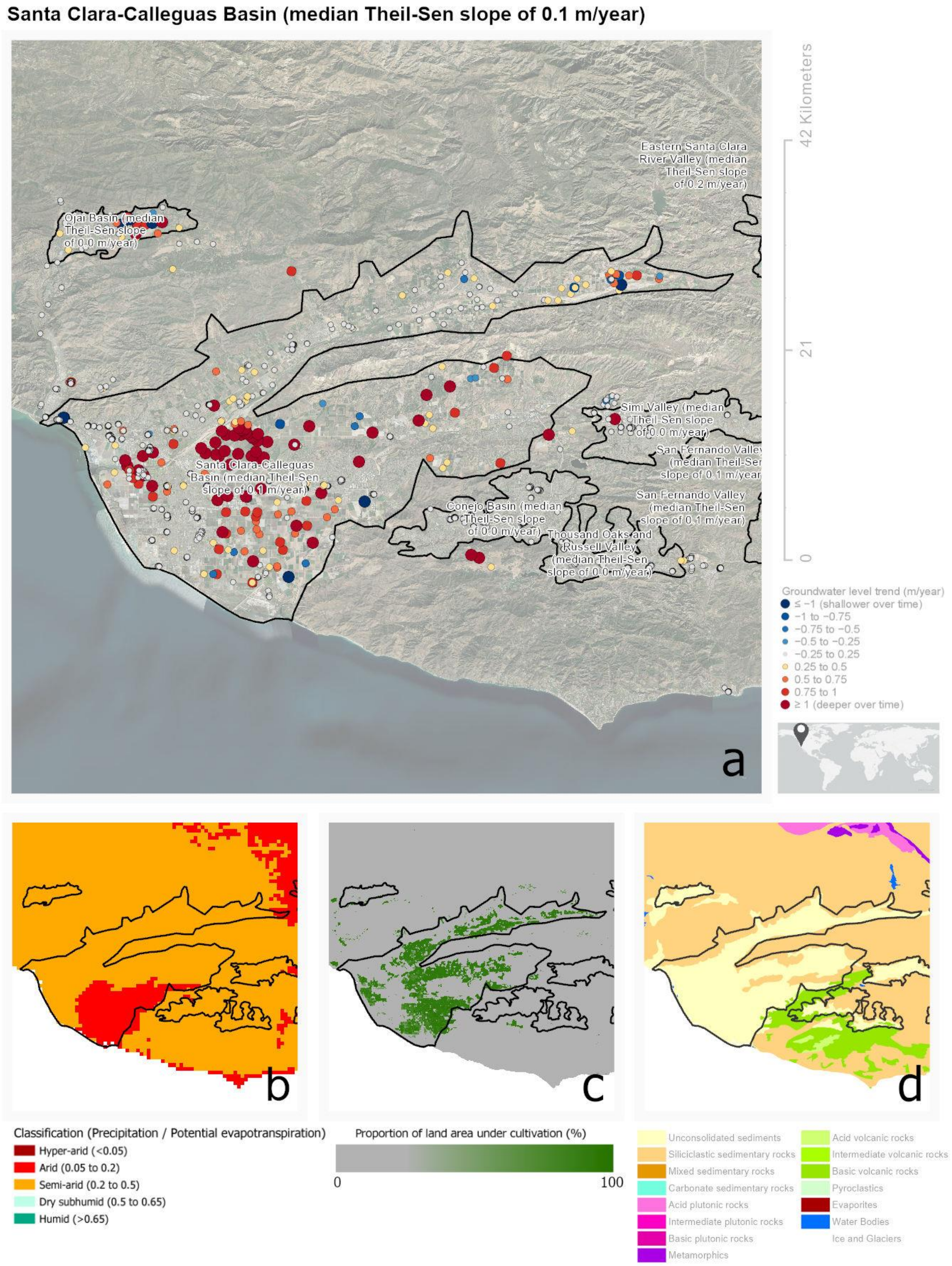

Supplementary Fig. 204. Spatial variability of trends in depth to groundwater Santa Clara-Calleguas Basin. For detailed description of the contents of each panel see the text at the beginning of Supplementary Note 17.

Santa Cruz Coastal Terraces (median Theil-Sen slope of 0 m/year)

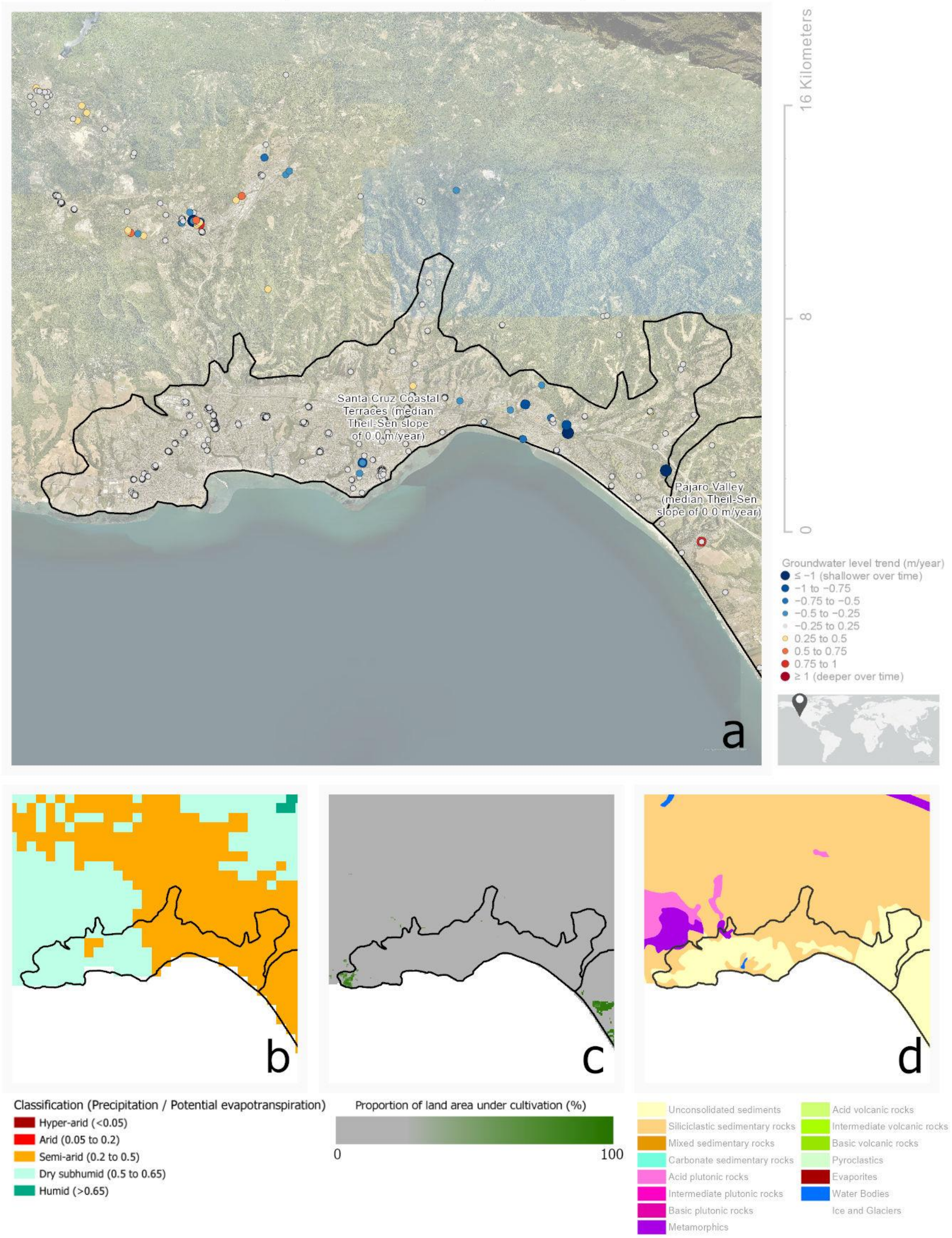

Supplementary Fig. 205. Spatial variability of trends in depth to groundwater Santa Cruz Coastal Terraces. For detailed description of the contents of each panel see the text at the beginning of Supplementary Note 17.

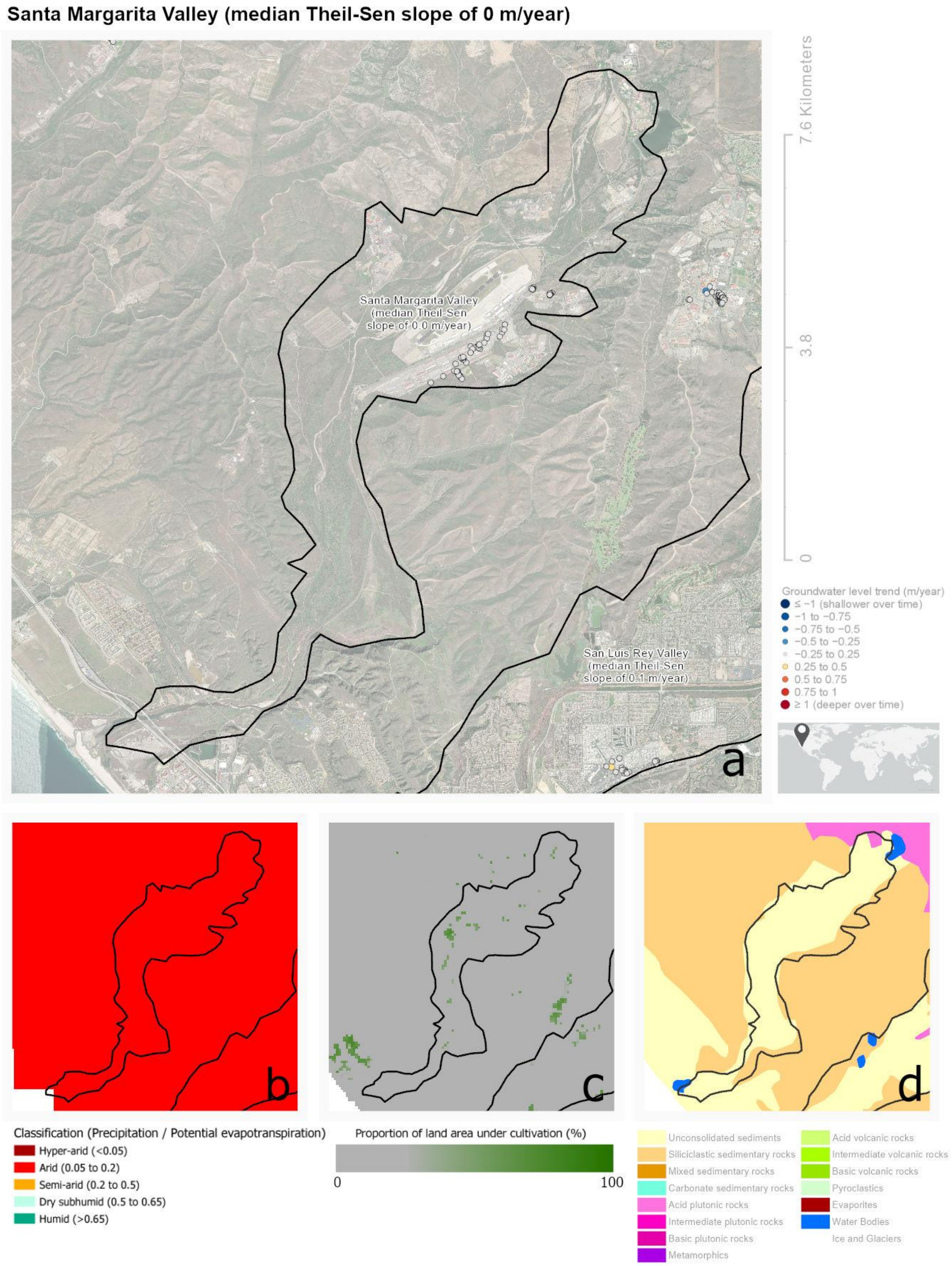

Supplementary Fig. 206. Spatial variability of trends in depth to groundwater Santa Margarita Valley. For detailed description of the contents of each panel see the text at the beginning of Supplementary Note 17.

Santa Maria Basin and Nipomo Valley (median Theil-Sen slope of 0.1 m/year)

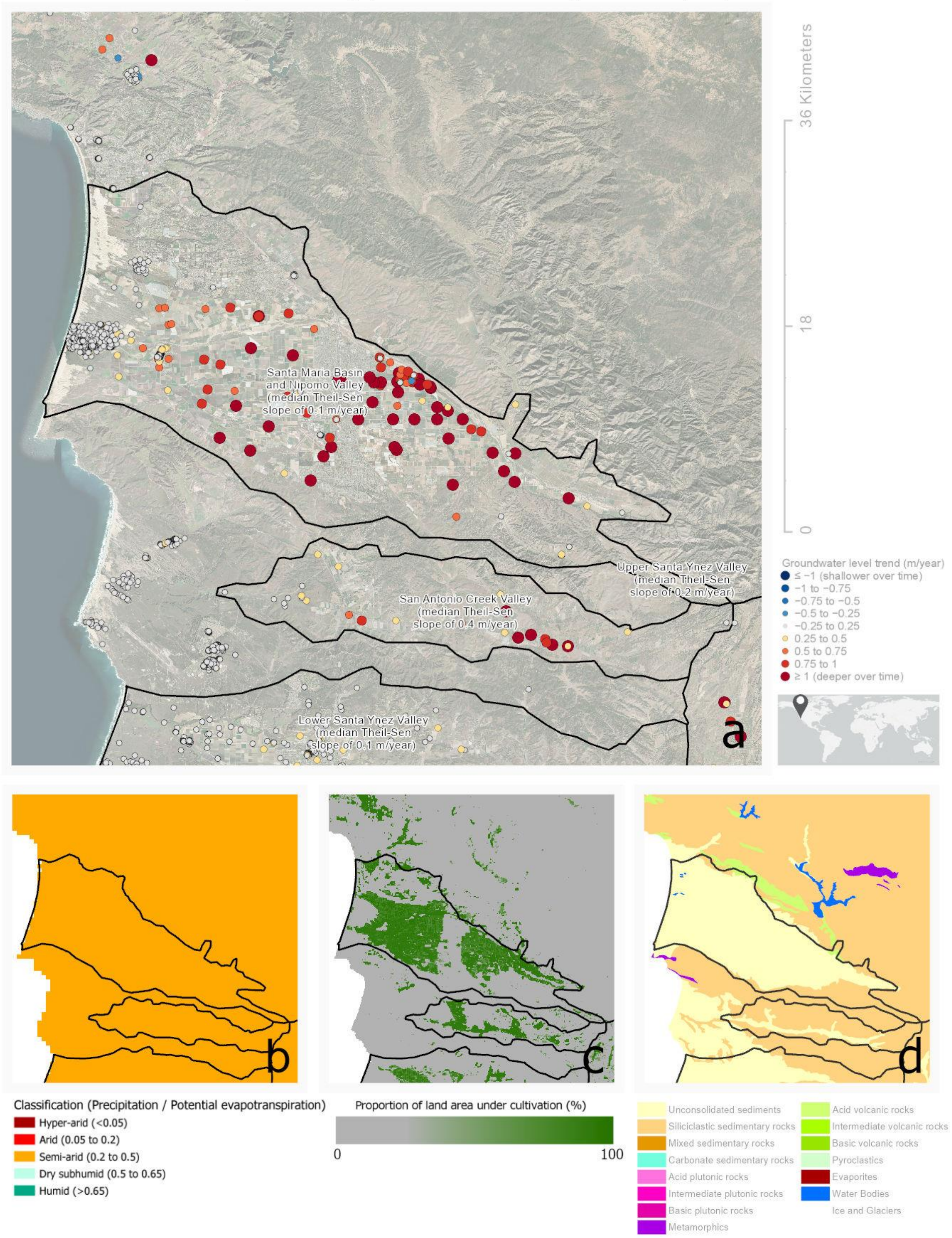

Supplementary Fig. 207. Spatial variability of trends in depth to groundwater Santa Maria Basin and Nipomo Valley. For detailed description of the contents of each panel see the text at the beginning of Supplementary Note 17.

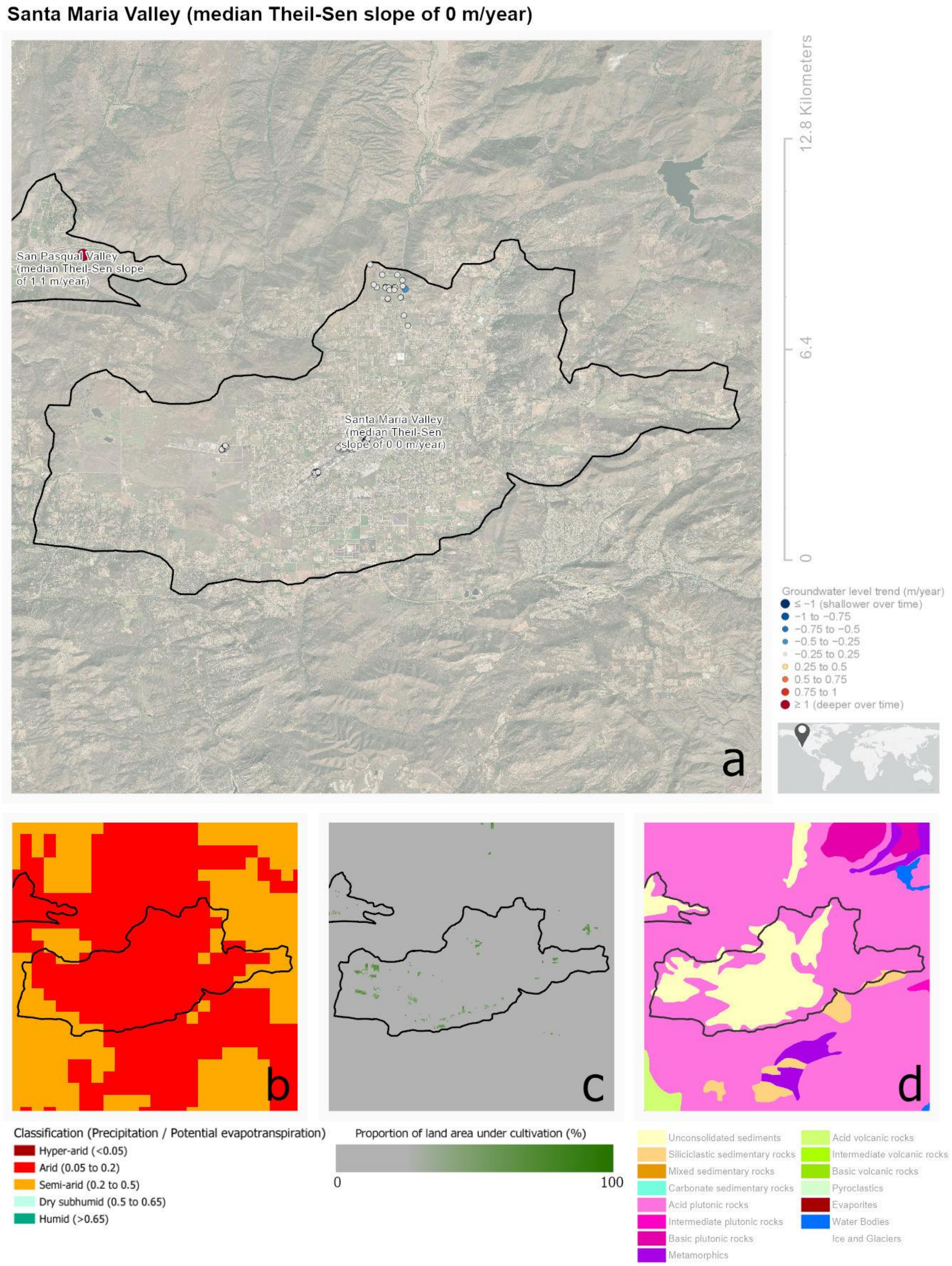

Supplementary Fig. 208. Spatial variability of trends in depth to groundwater Santa Maria Valley. For detailed description of the contents of each panel see the text at the beginning of Supplementary Note 17.

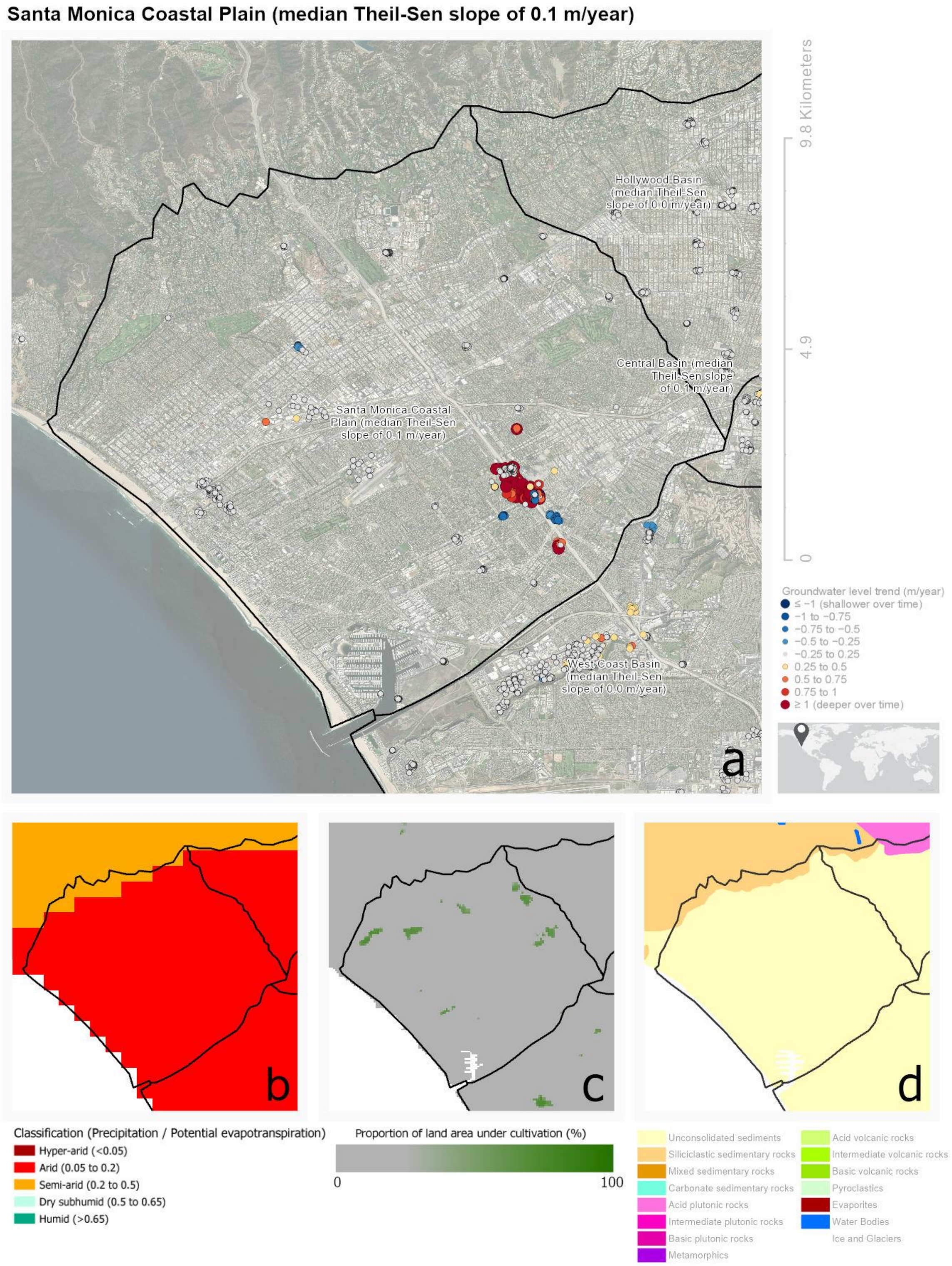

Supplementary Fig. 209. Spatial variability of trends in depth to groundwater Santa Monica Coastal Plain (within the broader Los Angeles Basin). For detailed description of the contents of each panel see the text at the beginning of Supplementary Note 17.

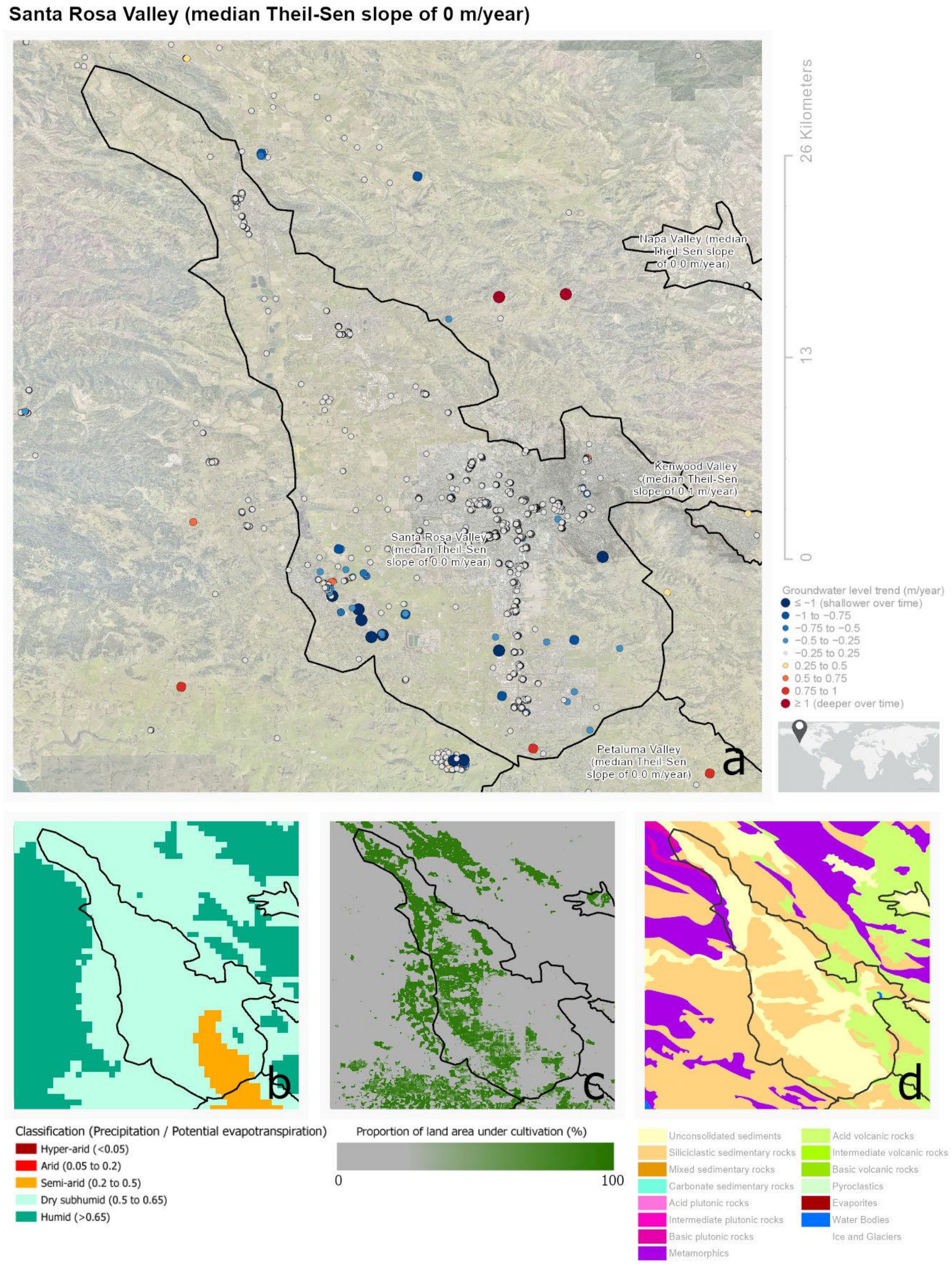

Supplementary Fig. 210. Spatial variability of trends in depth to groundwater Santa Rosa Valley. For detailed description of the contents of each panel see the text at the beginning of Supplementary Note 17.

Scotts Valley (median Theil-Sen slope of 0 m/year)

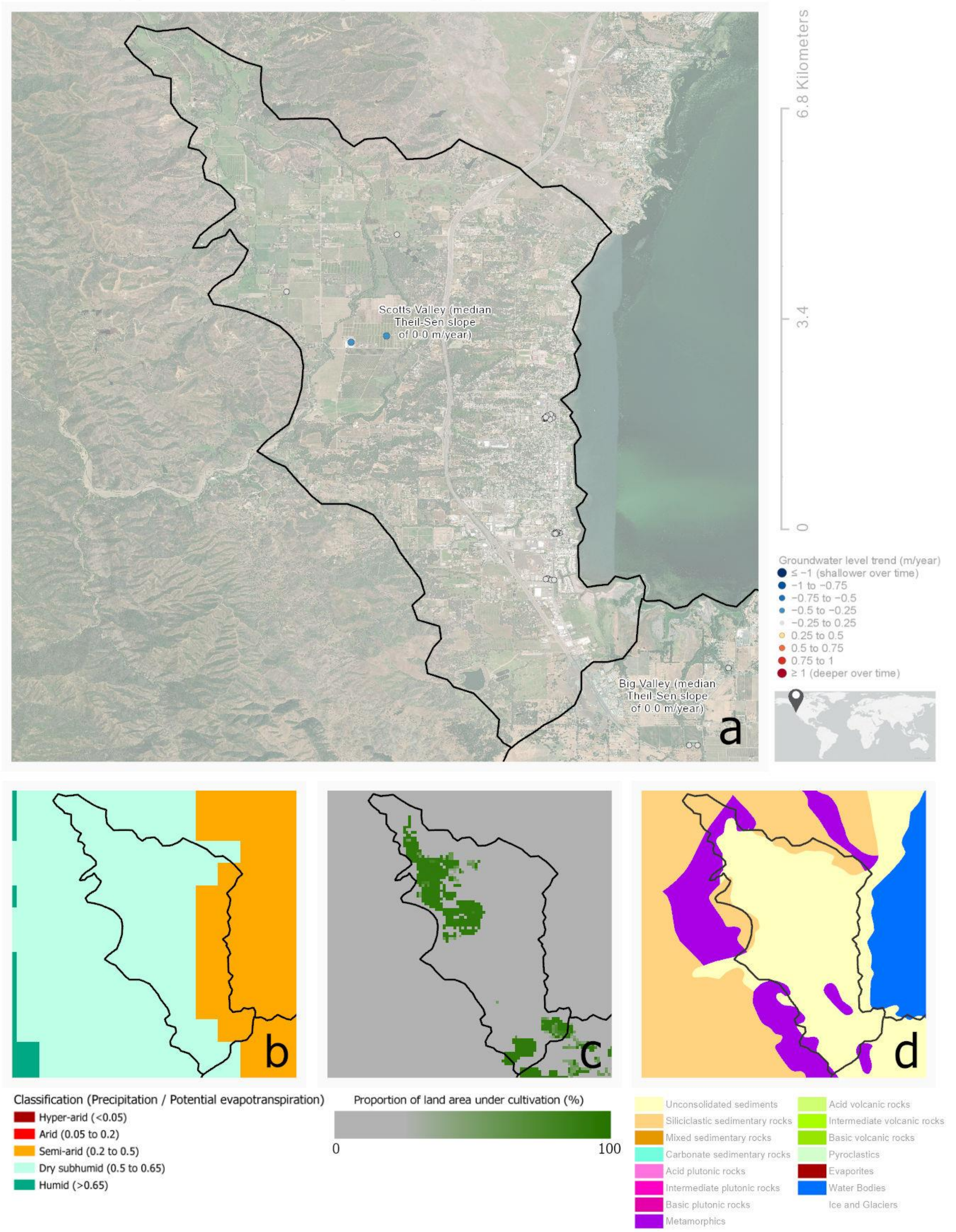

Supplementary Fig. 211. Spatial variability of trends in depth to groundwater Scotts Valley. For detailed description of the contents of each panel see the text at the beginning of Supplementary Note 17.

Sendai River Basin (median Theil-Sen slope of -0.1 m/year)

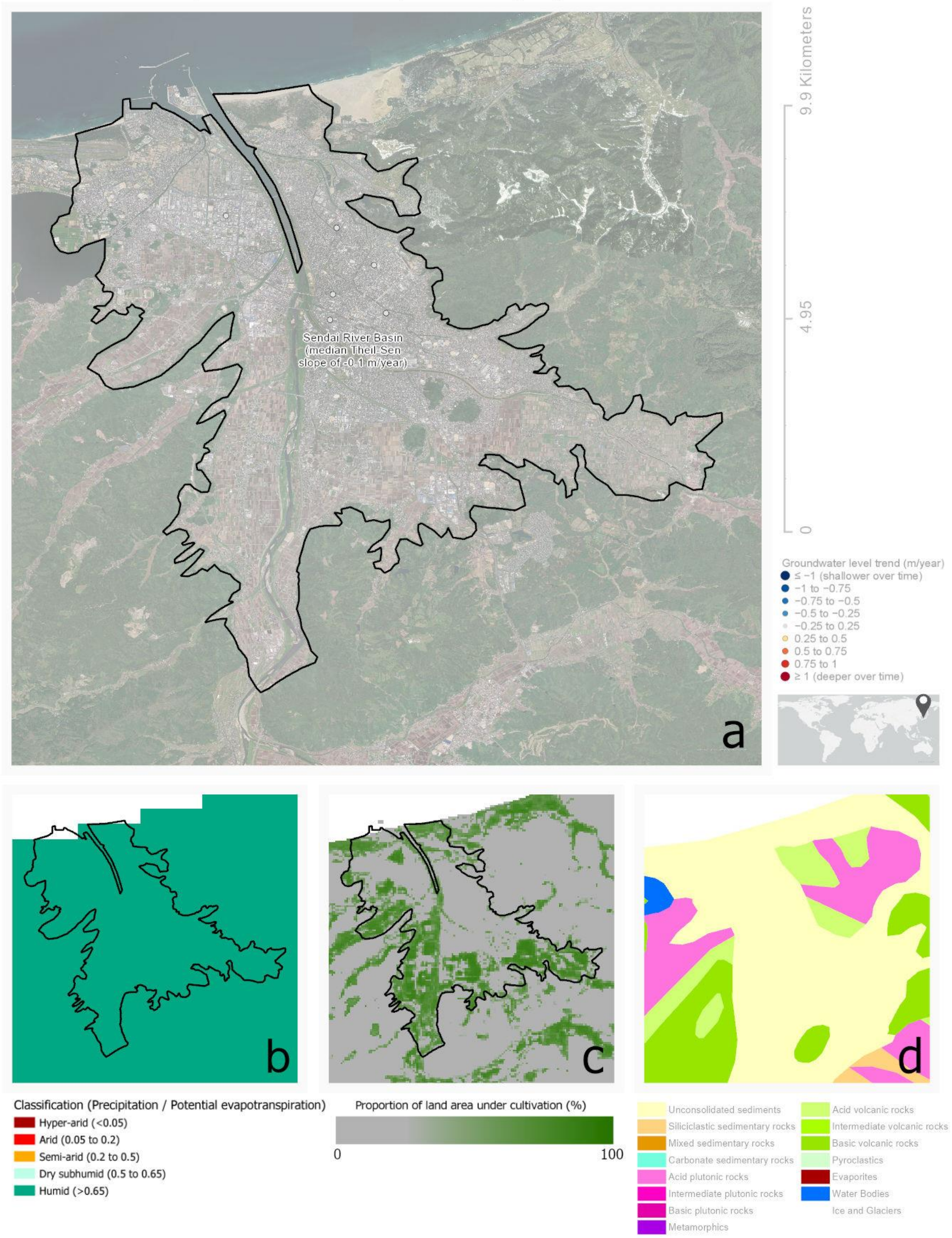

Supplementary Fig. 212. Spatial variability of trends in depth to groundwater Sendai River Basin. For detailed description of the contents of each panel see the text at the beginning of Supplementary Note 17.

Shiramin Basin (median Theil-Sen slope of 0 m/year)

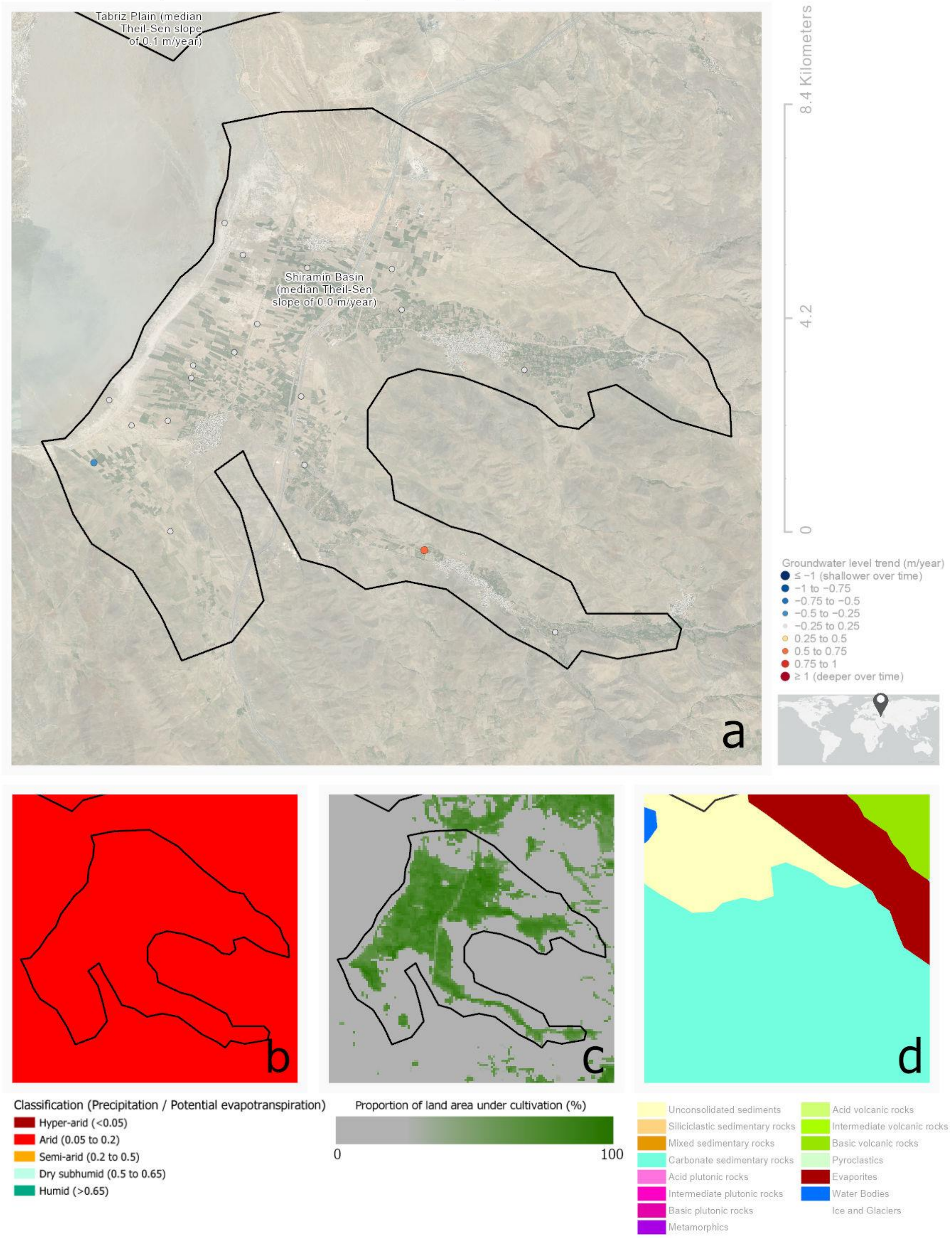

Supplementary Fig. 213. Spatial variability of trends in depth to groundwater Shiramin Basin. For detailed description of the contents of each panel see the text at the beginning of Supplementary Note 17.

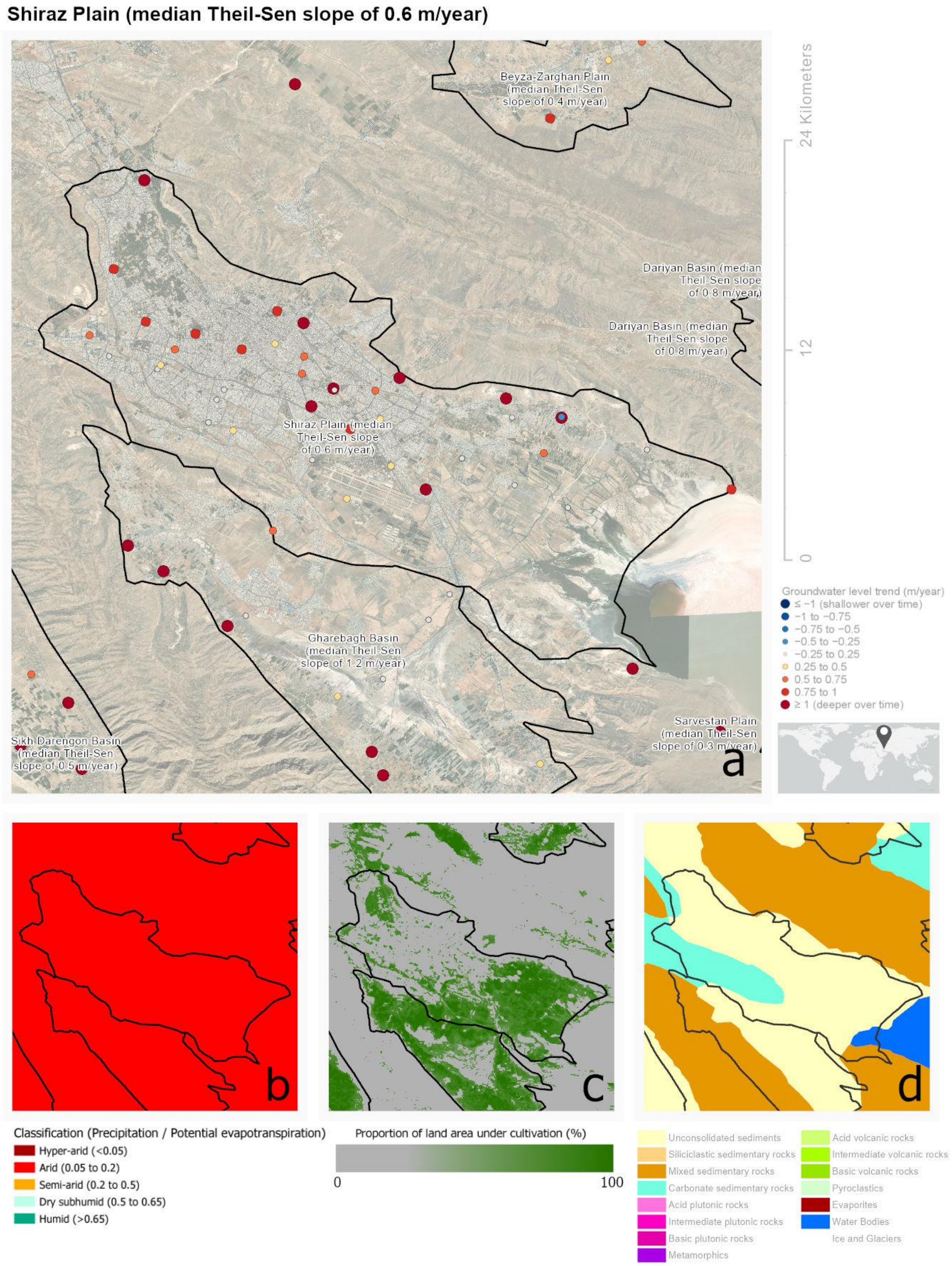

Supplementary Fig. 214. Spatial variability of trends in depth to groundwater Shiraz Plain. For detailed description of the contents of each panel see the text at the beginning of Supplementary Note 17.

Sierra Valley (median Theil-Sen slope of 0.1 m/year)

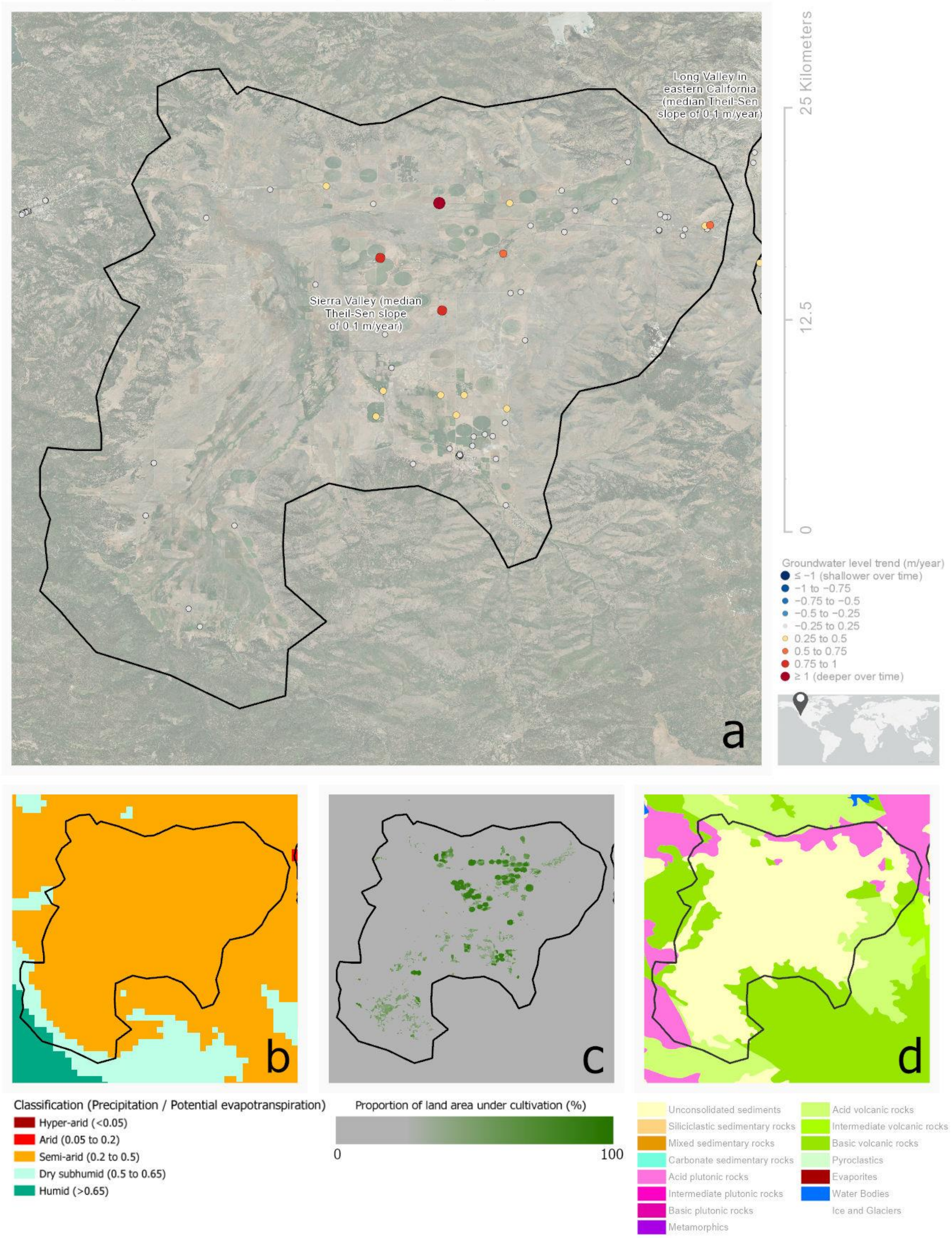

Supplementary Fig. 215. Spatial variability of trends in depth to groundwater Sierra Valley. For detailed description of the contents of each panel see the text at the beginning of Supplementary Note 17.

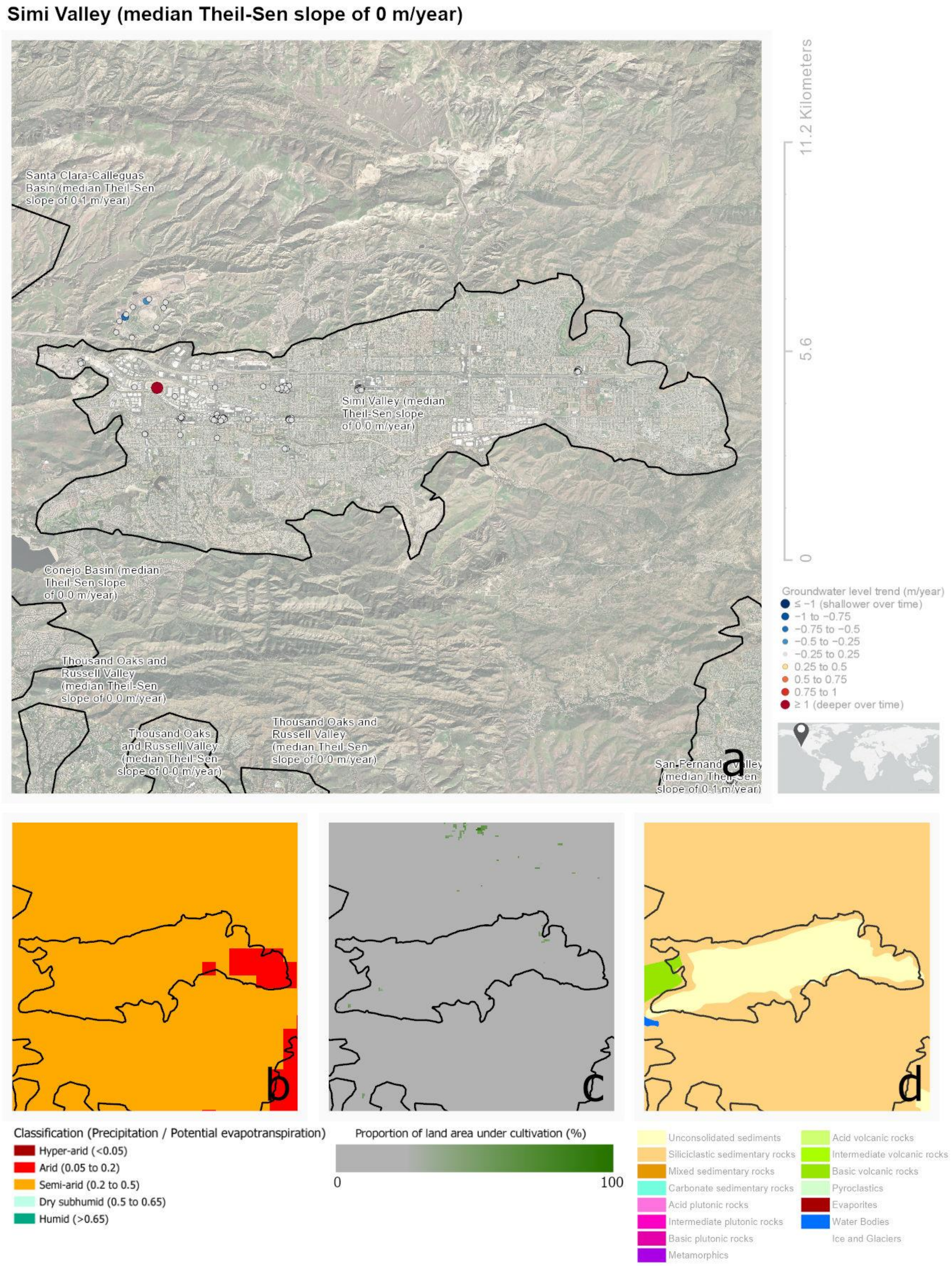

Supplementary Fig. 216. Spatial variability of trends in depth to groundwater Simi Valley. For detailed description of the contents of each panel see the text at the beginning of Supplementary Note 17.

Sirik Basin (median Theil-Sen slope of 0.1 m/year)

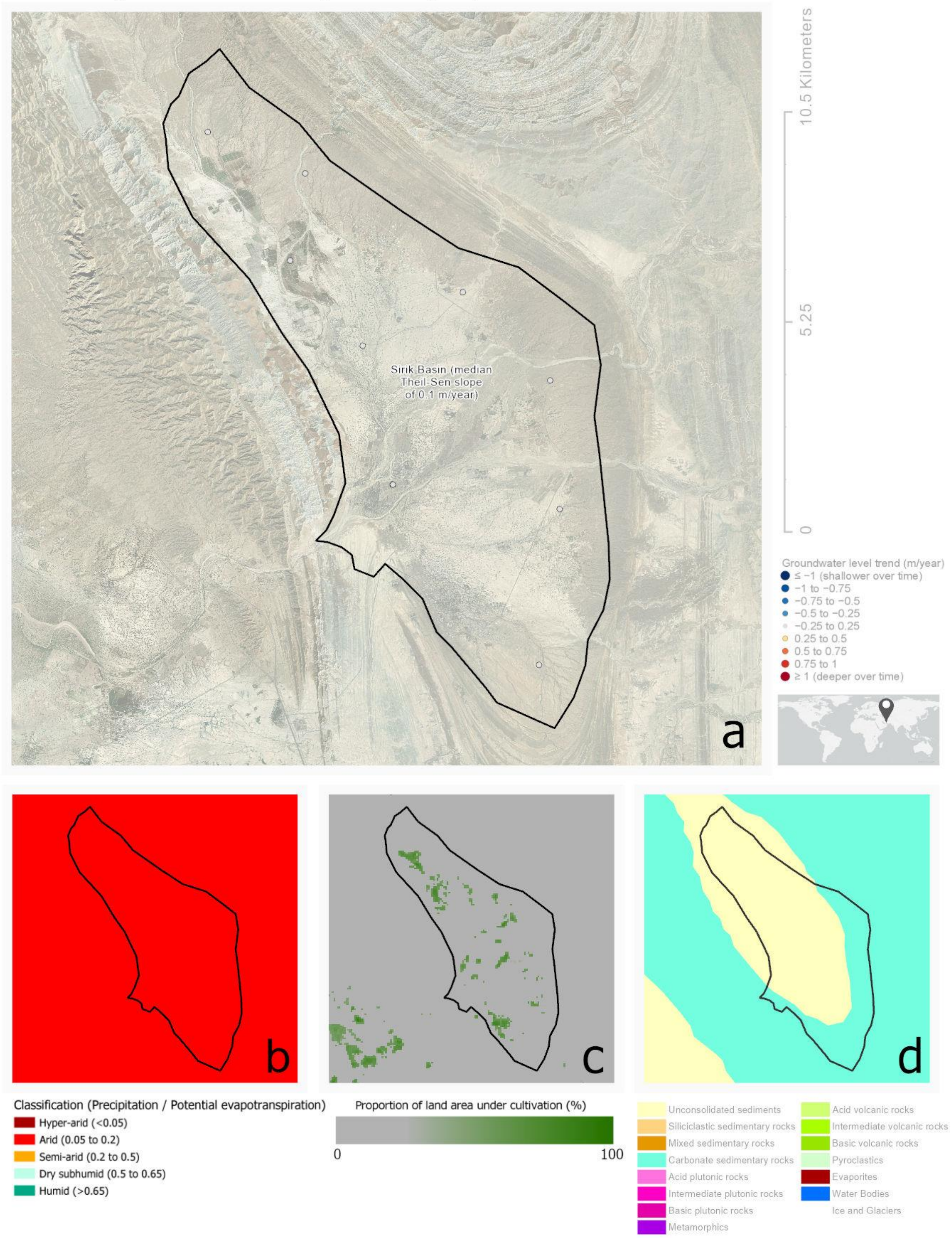

Supplementary Fig. 217. Spatial variability of trends in depth to groundwater Sirik Basin. For detailed description of the contents of each panel see the text at the beginning of Supplementary Note 17.

Smith River Plain (median Theil-Sen slope of 0 m/year)

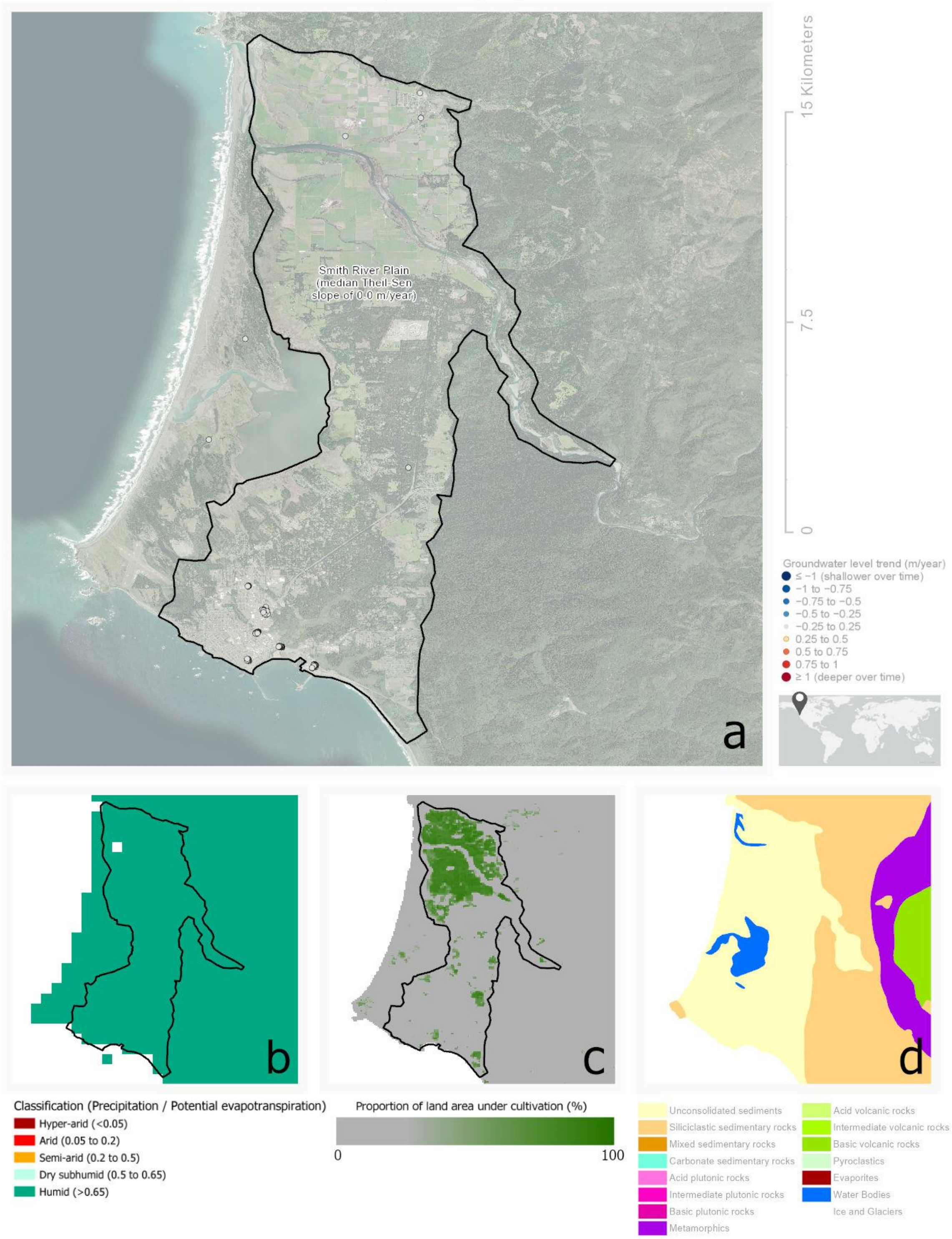

Supplementary Fig. 218. Spatial variability of trends in depth to groundwater Smith River Plain. For detailed description of the contents of each panel see the text at the beginning of Supplementary Note 17.

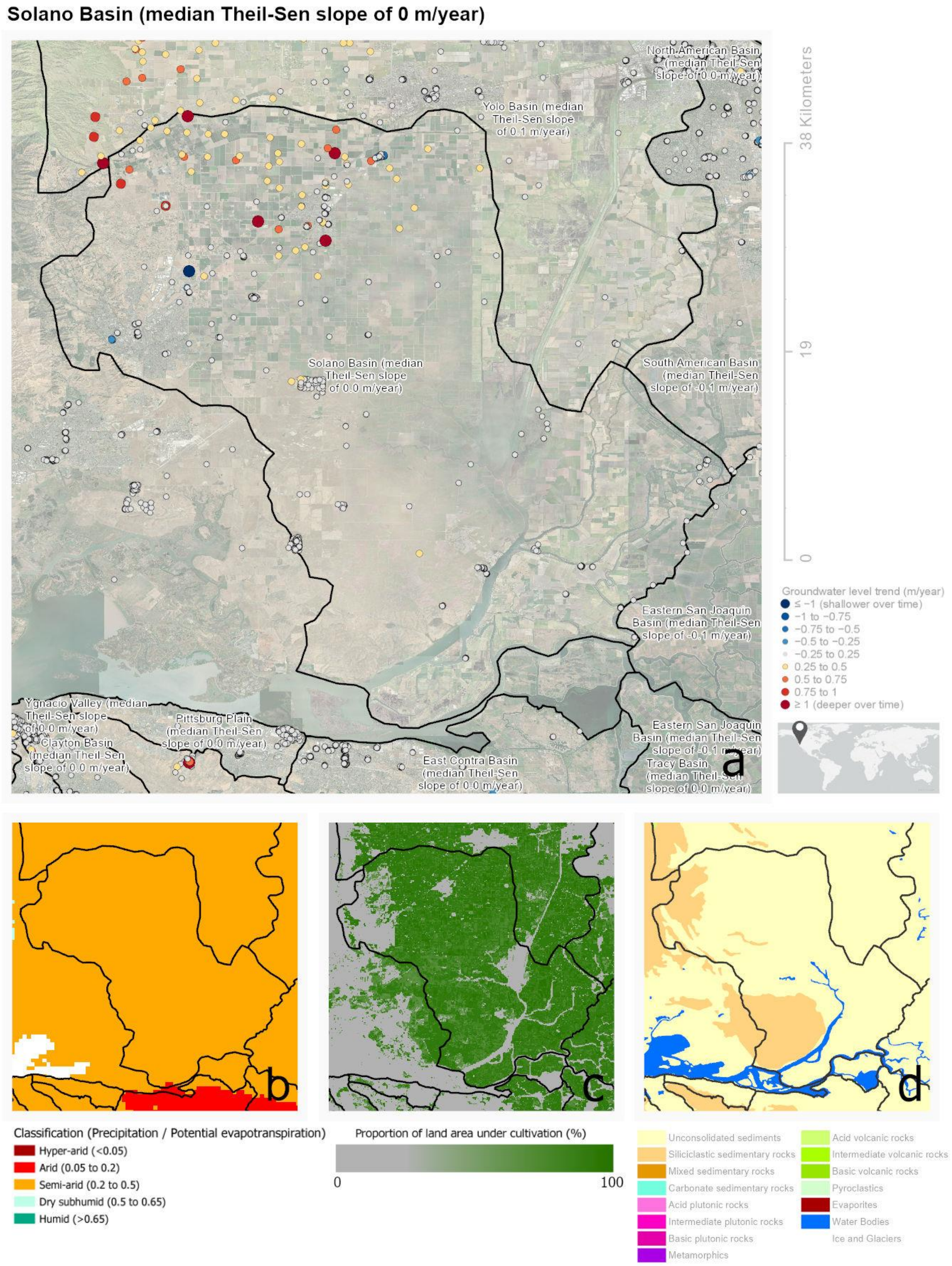

Supplementary Fig. 219. Spatial variability of trends in depth to groundwater Solano Basin (within the broader Sacramento Valley of the Central Valley). For detailed description of the contents of each panel see the text at the beginning of Supplementary Note 17.

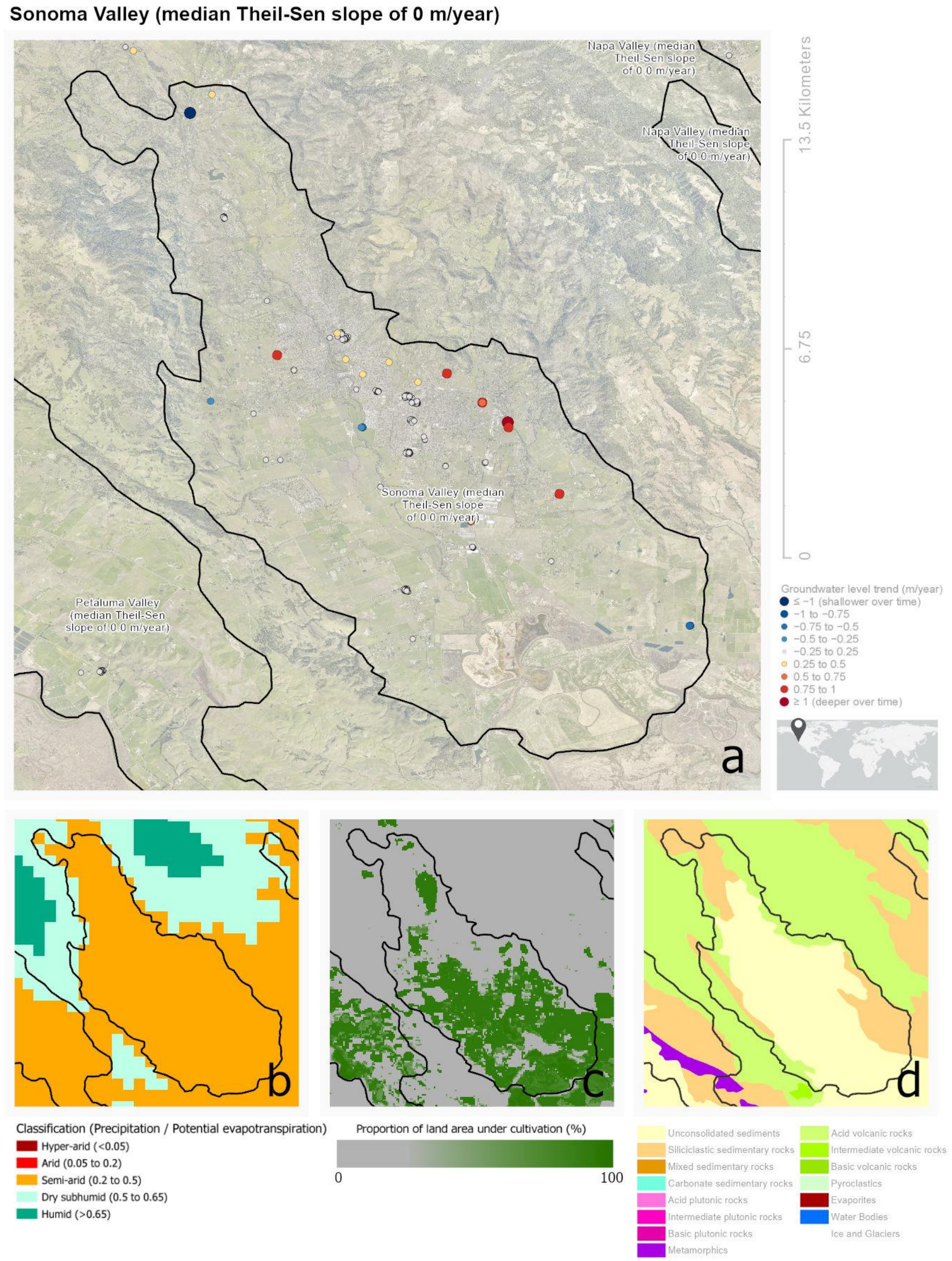

Supplementary Fig. 220. Spatial variability of trends in depth to groundwater Sonoma Valley. For detailed description of the contents of each panel see the text at the beginning of Supplementary Note 17.

South American Basin (median Theil-Sen slope of -0.1 m/year)

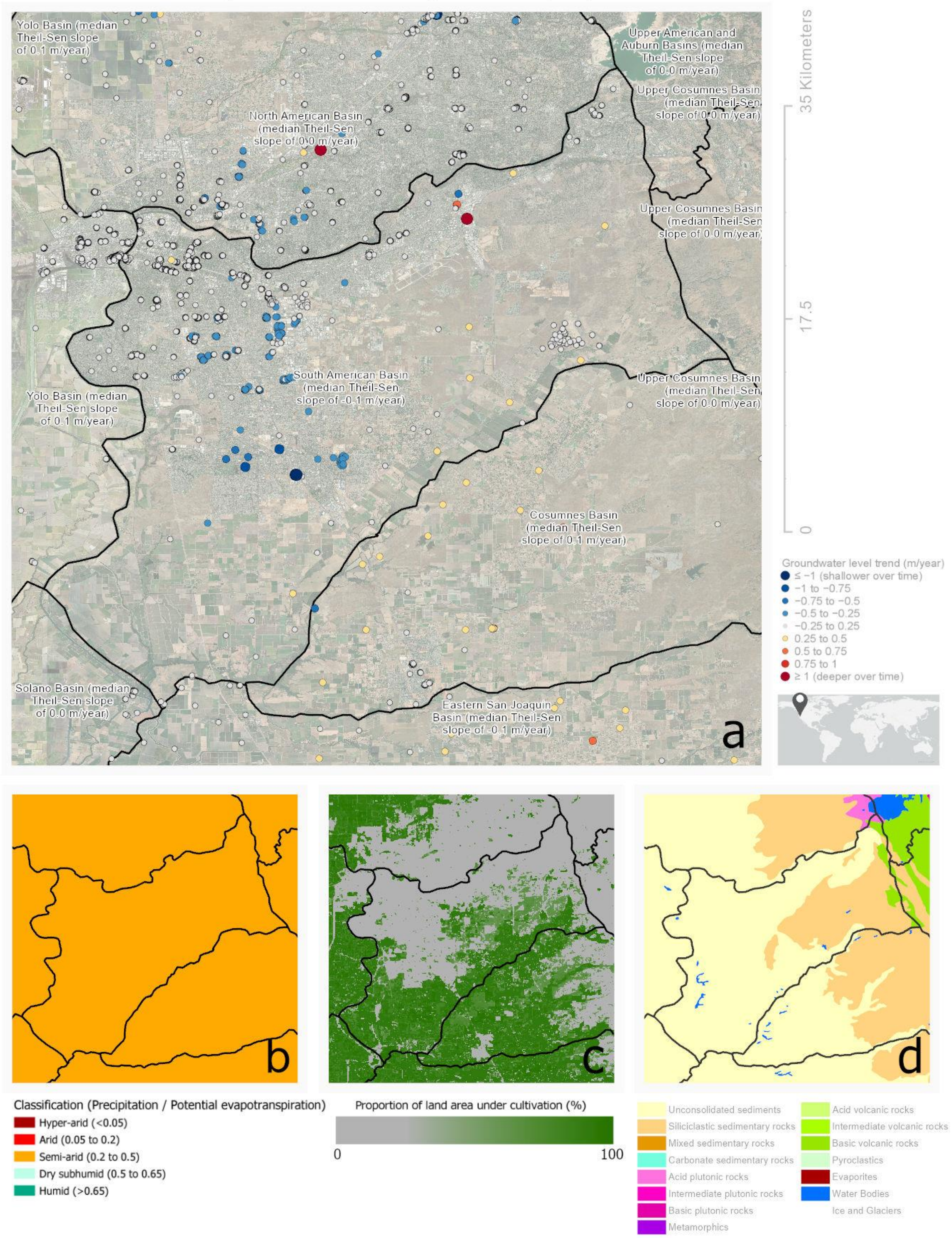

Supplementary Fig. 221. Spatial variability of trends in depth to groundwater South American Basin (within the broader Sacramento Valley of the Central Valley). For detailed description of the contents of each panel see the text at the beginning of Supplementary Note 17.

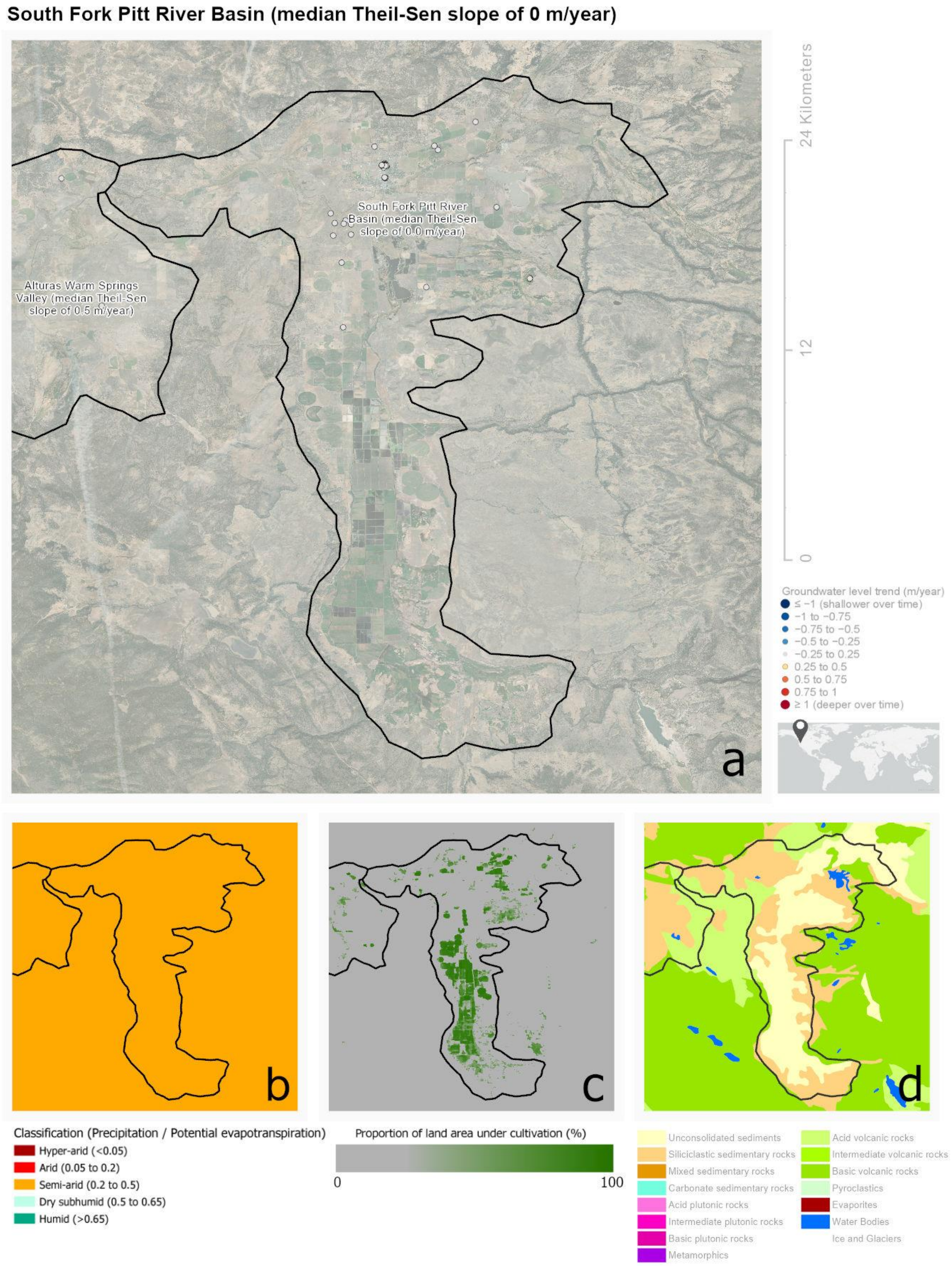

Supplementary Fig. 222. Spatial variability of trends in depth to groundwater South Fork Pitt River Basin. For detailed description of the contents of each panel see the text at the beginning of Supplementary Note 17.

South Yuba Basin (median Theil-Sen slope of 0 m/year)

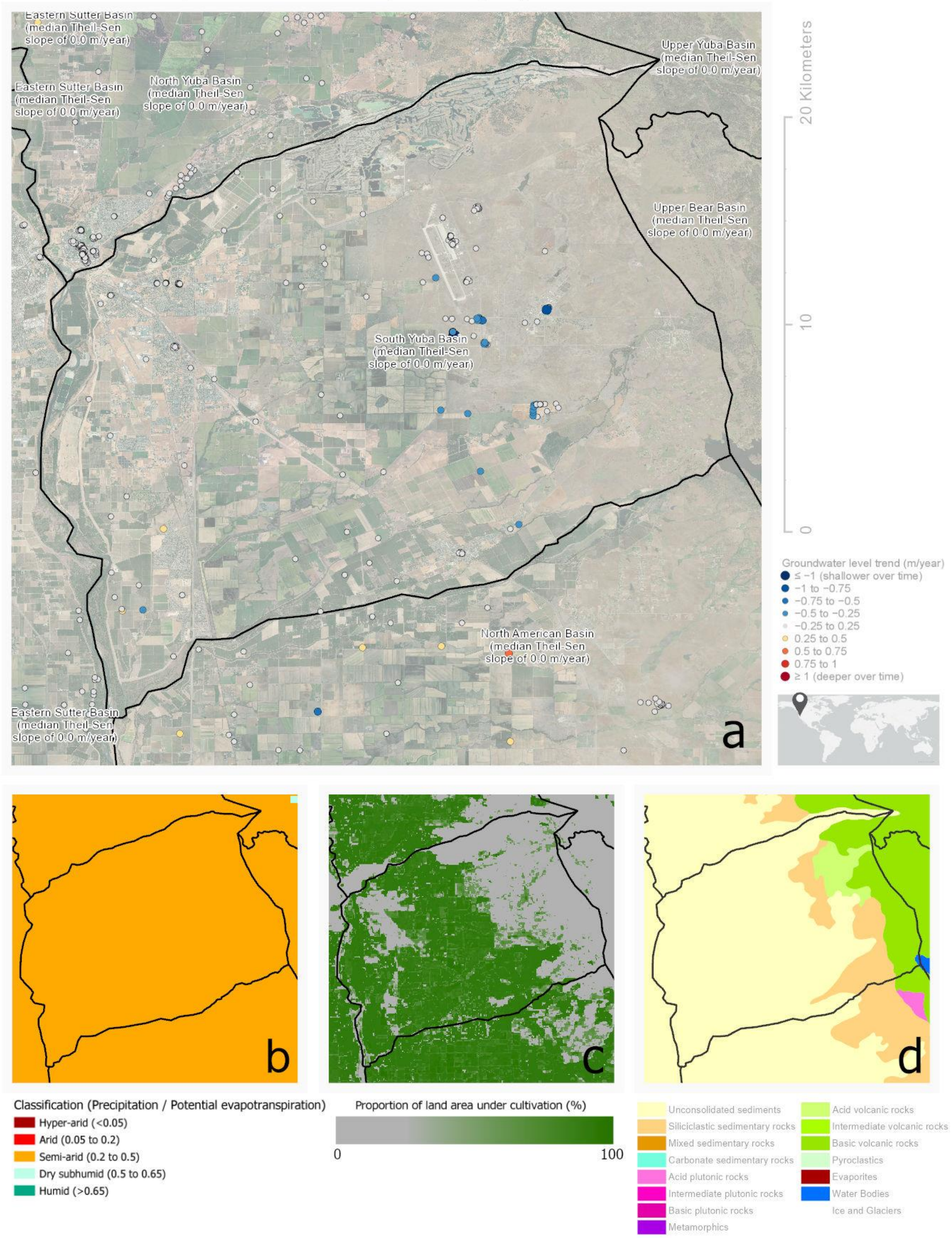

Supplementary Fig. 223. Spatial variability of trends in depth to groundwater South Yuba Basin (within the broader Sacramento Valley of the Central Valley). For detailed description of the contents of each panel see the text at the beginning of Supplementary Note 17.

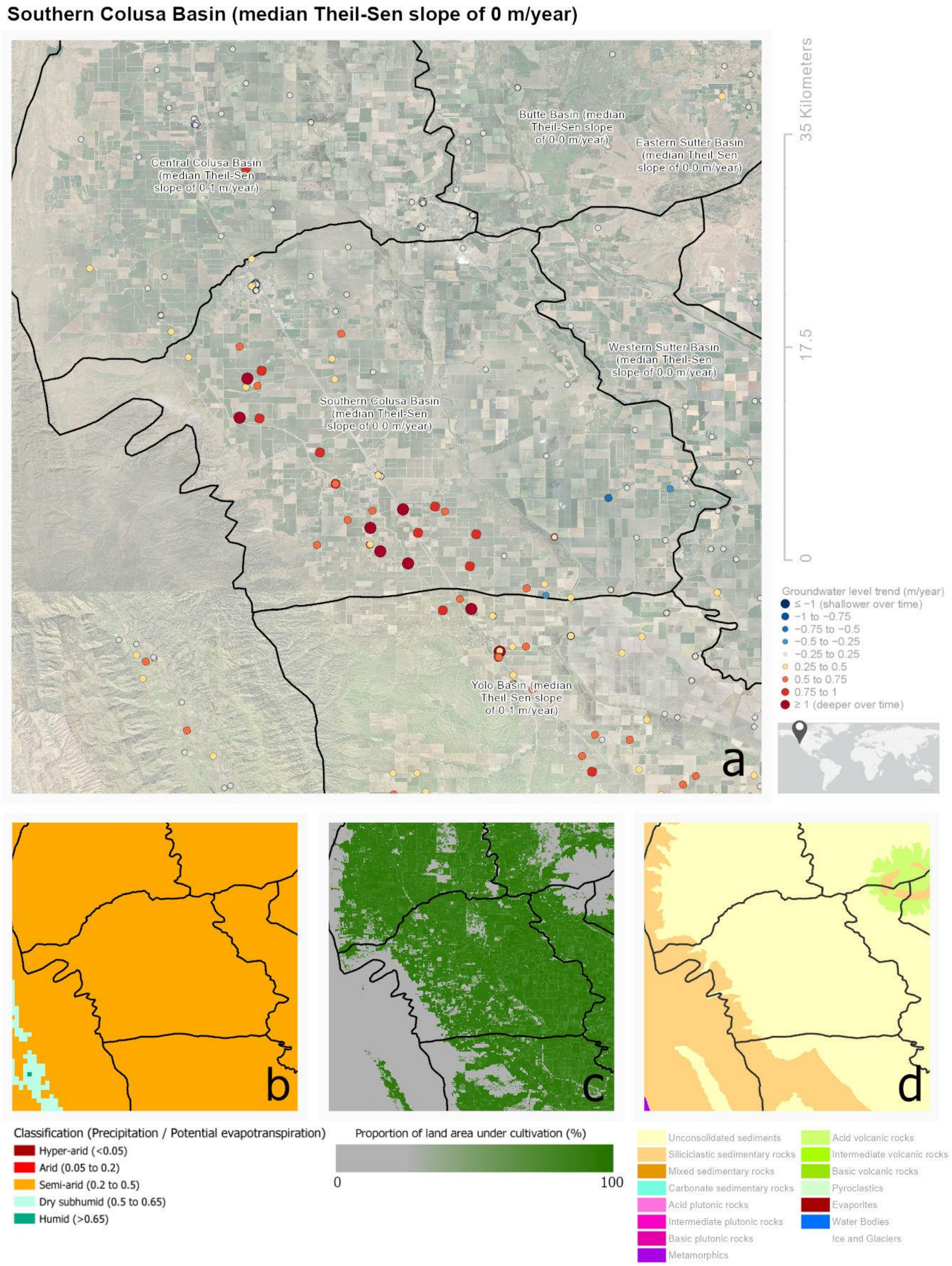

Supplementary Fig. 224. Spatial variability of trends in depth to groundwater Southern Colusa Basin (within the broader Sacramento Valley of the Central Valley). For detailed description of the contents of each panel see the text at the beginning of Supplementary Note 17.

Southern Kettleman Plain (median Theil-Sen slope of 0.4 m/year)

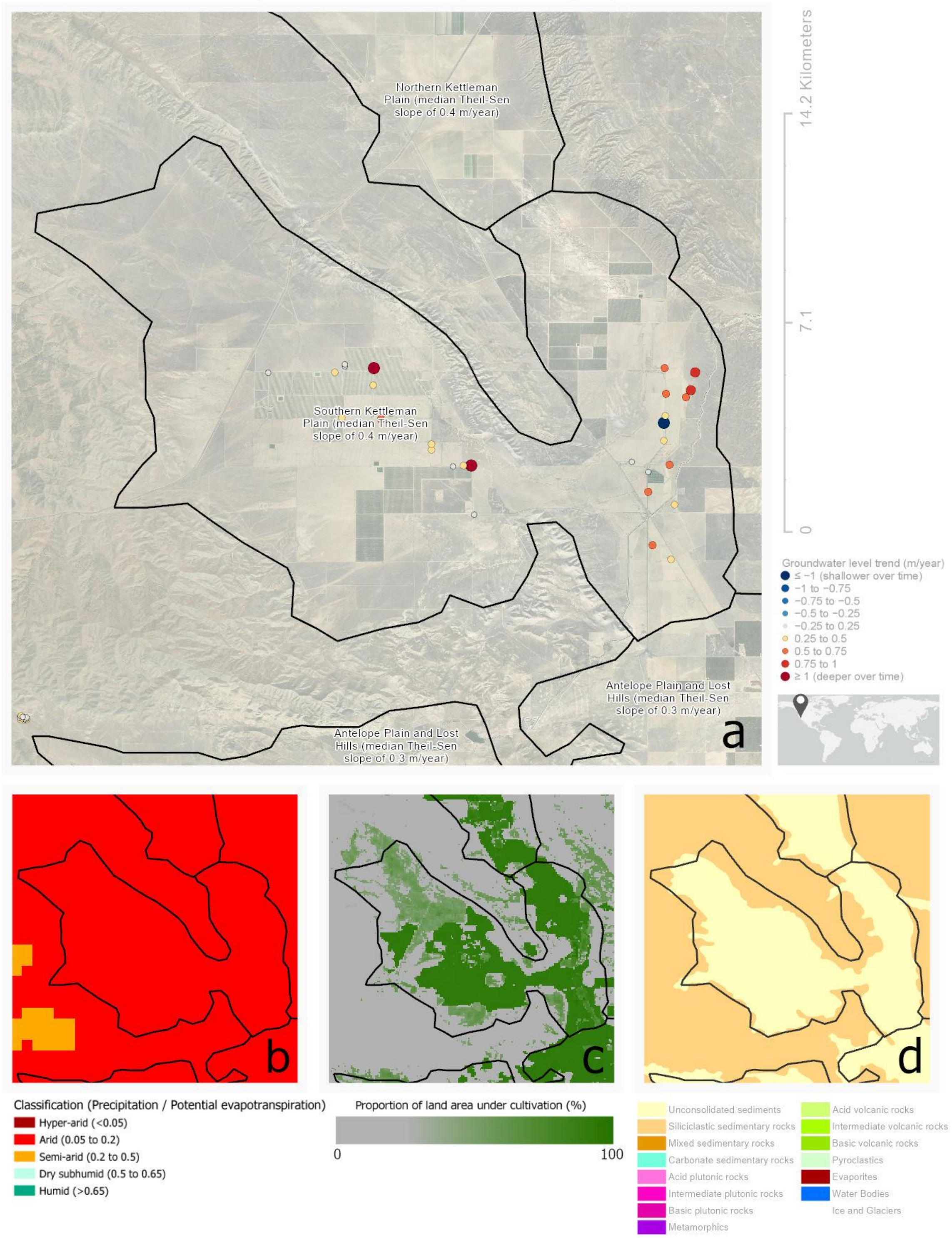

Supplementary Fig. 225. Spatial variability of trends in depth to groundwater Southern Kettleman Plain. For detailed description of the contents of each panel see the text at the beginning of Supplementary Note 17.

Southern Morongo Basin (median Theil-Sen slope of 0.1 m/year)

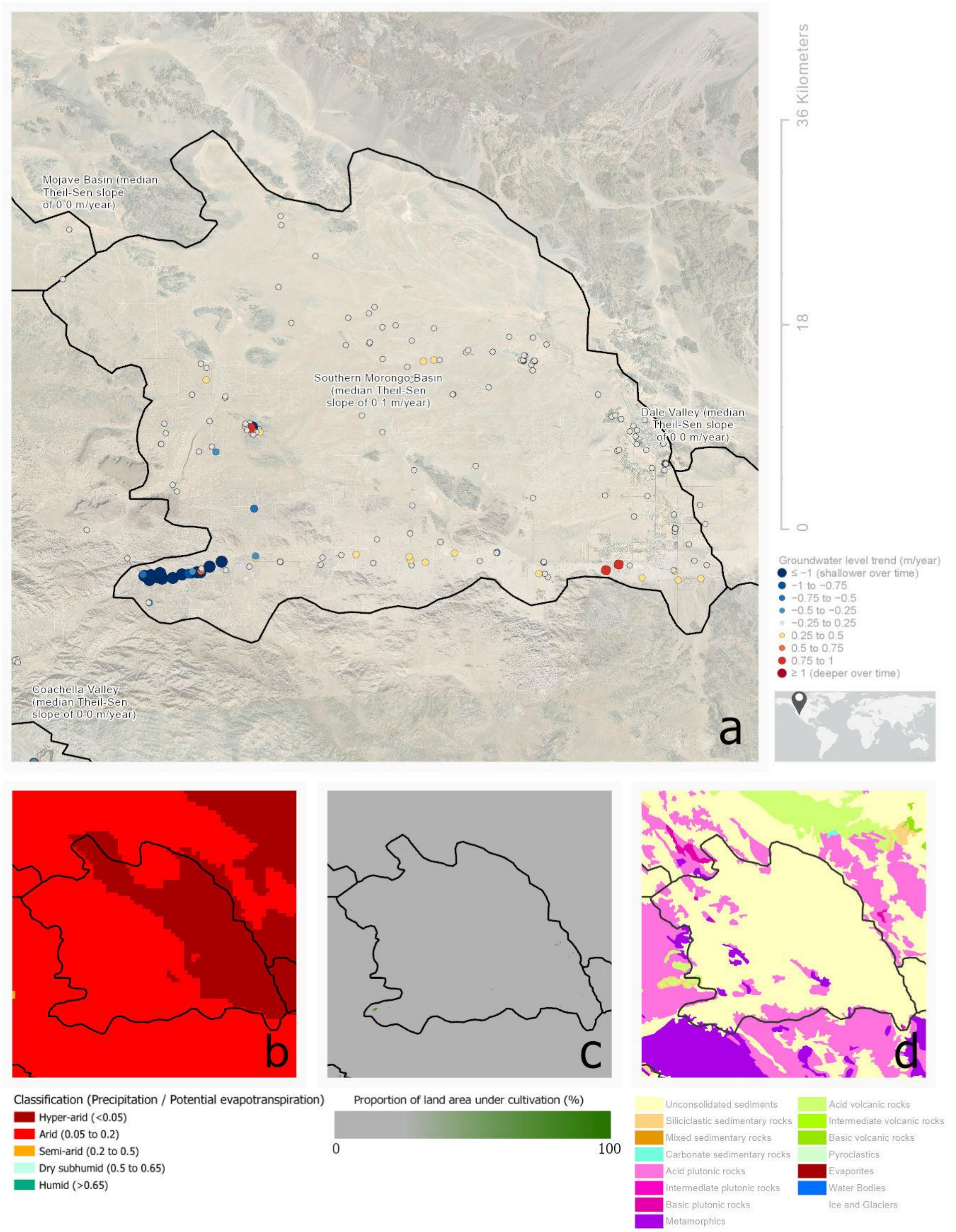

Supplementary Fig. 226. Spatial variability of trends in depth to groundwater Southern Morongo Basin. For detailed description of the contents of each panel see the text at the beginning of Supplementary Note 17.

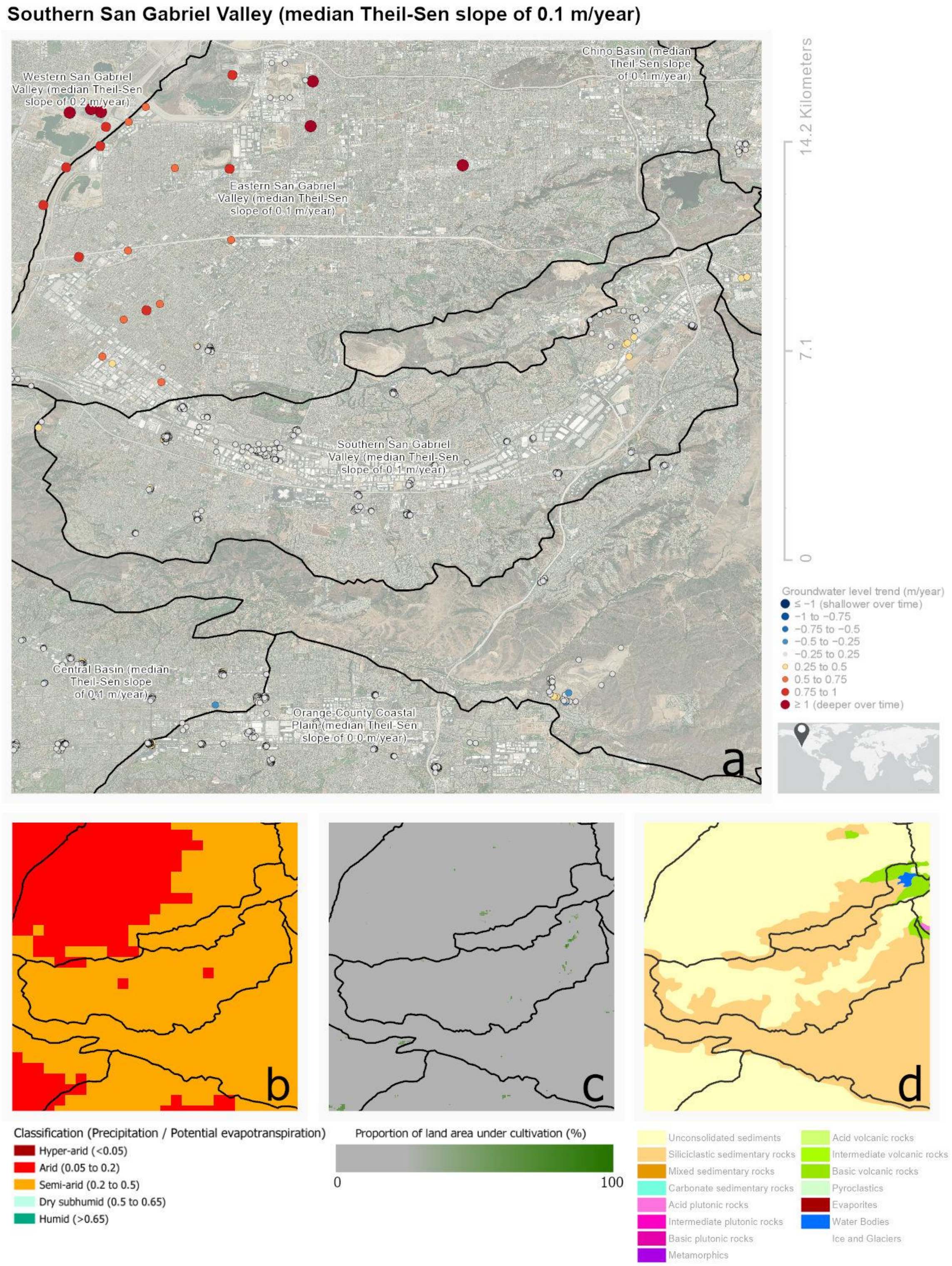

Supplementary Fig. 227. Spatial variability of trends in depth to groundwater Southern San Gabriel Valley (within the broader San Gabriel Valley). For detailed description of the contents of each panel see the text at the beginning of Supplementary Note 17.

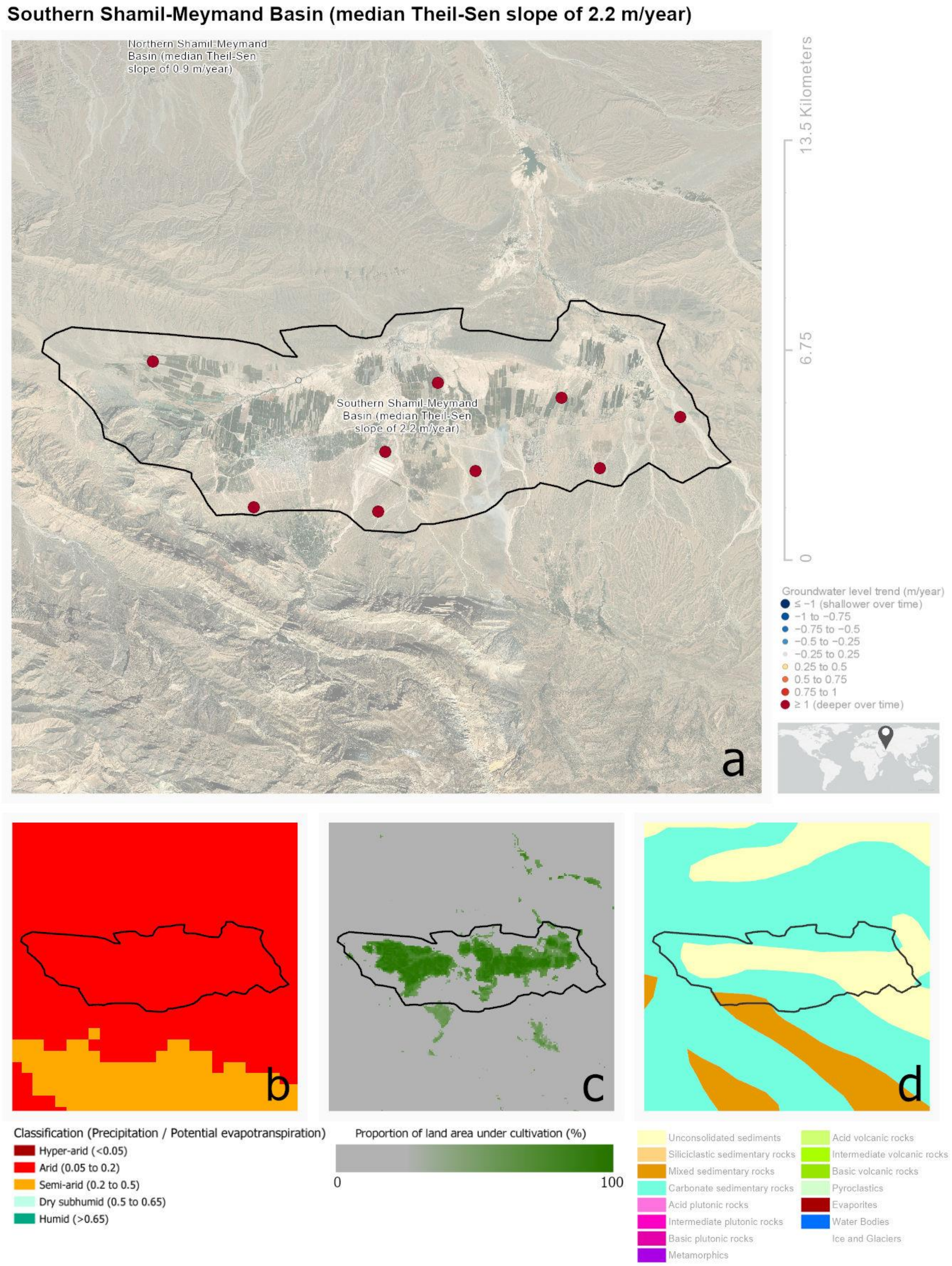

Supplementary Fig. 228. Spatial variability of trends in depth to groundwater Southern Shamil-Meymand Basin. For detailed description of the contents of each panel see the text at the beginning of Supplementary Note 17.

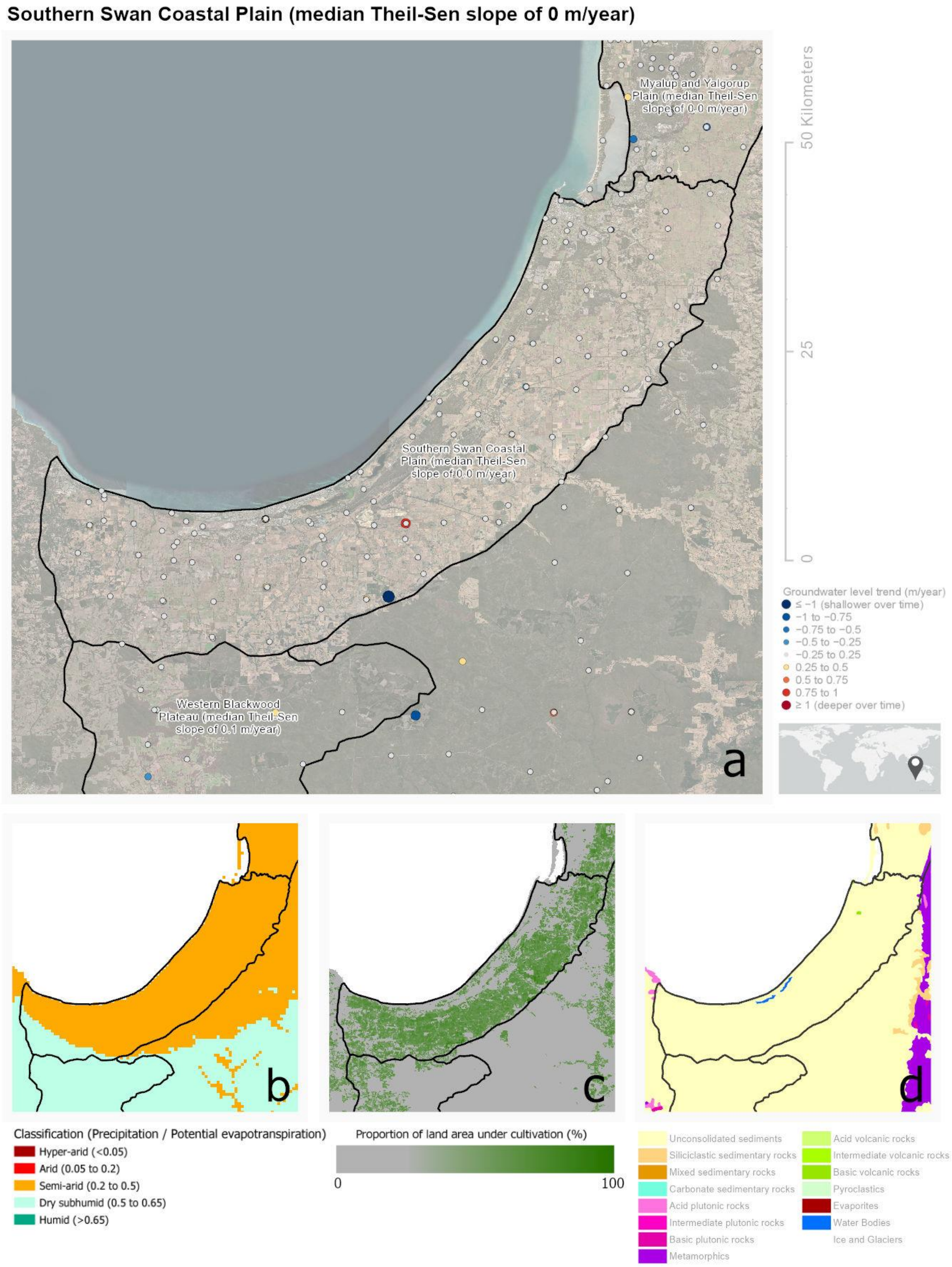

Supplementary Fig. 229. Spatial variability of trends in depth to groundwater Southern Swan Coastal Plain (within the broader Perth Basin). For detailed description of the contents of each panel see the text at the beginning of Supplementary Note 17.

Southern Tule Lake (median Theil-Sen slope of 0.3 m/year)

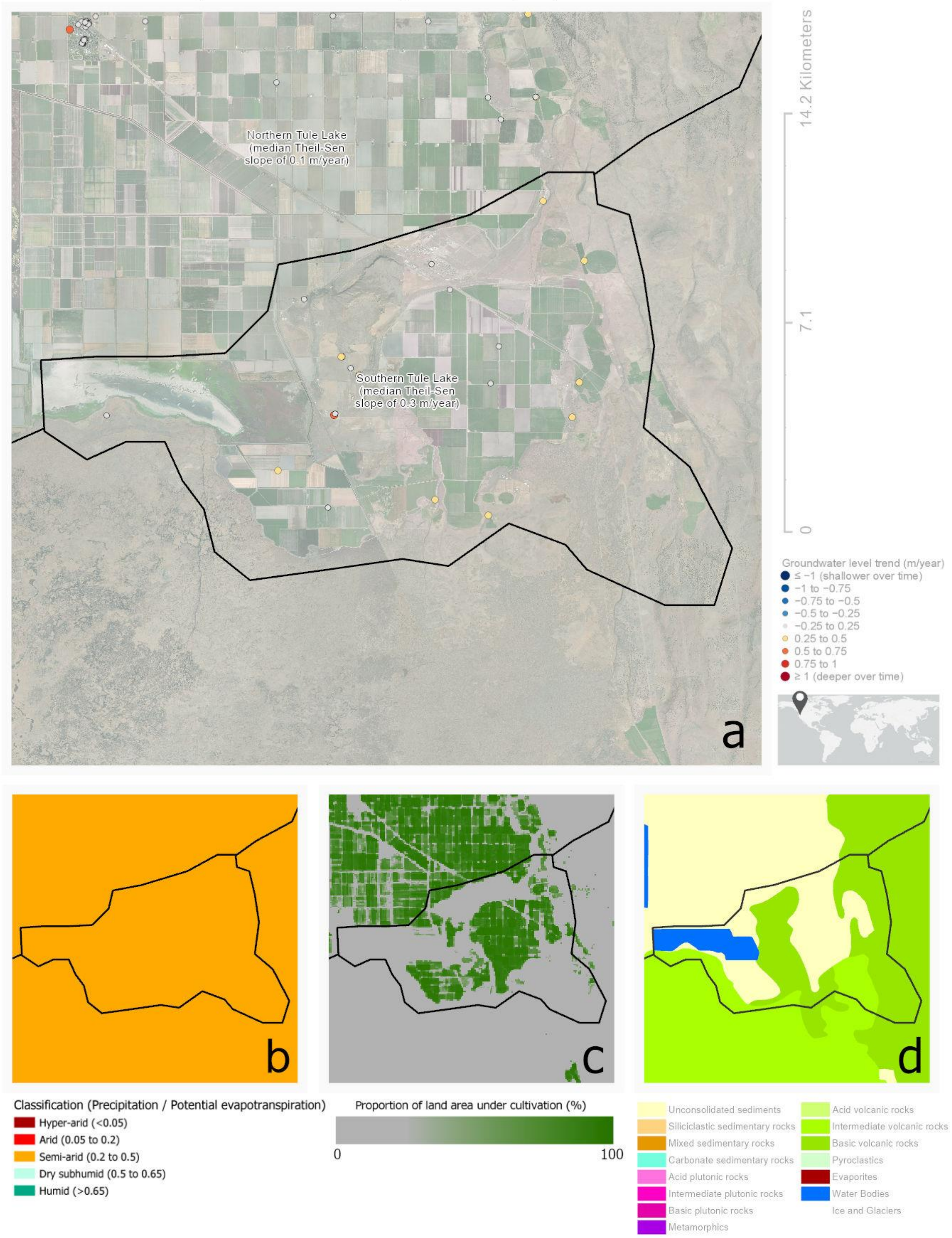

Supplementary Fig. 230. Spatial variability of trends in depth to groundwater Southern Tule Lake (within the broader Upper Klamath Basin). For detailed description of the contents of each panel see the text at the beginning of Supplementary Note 17.

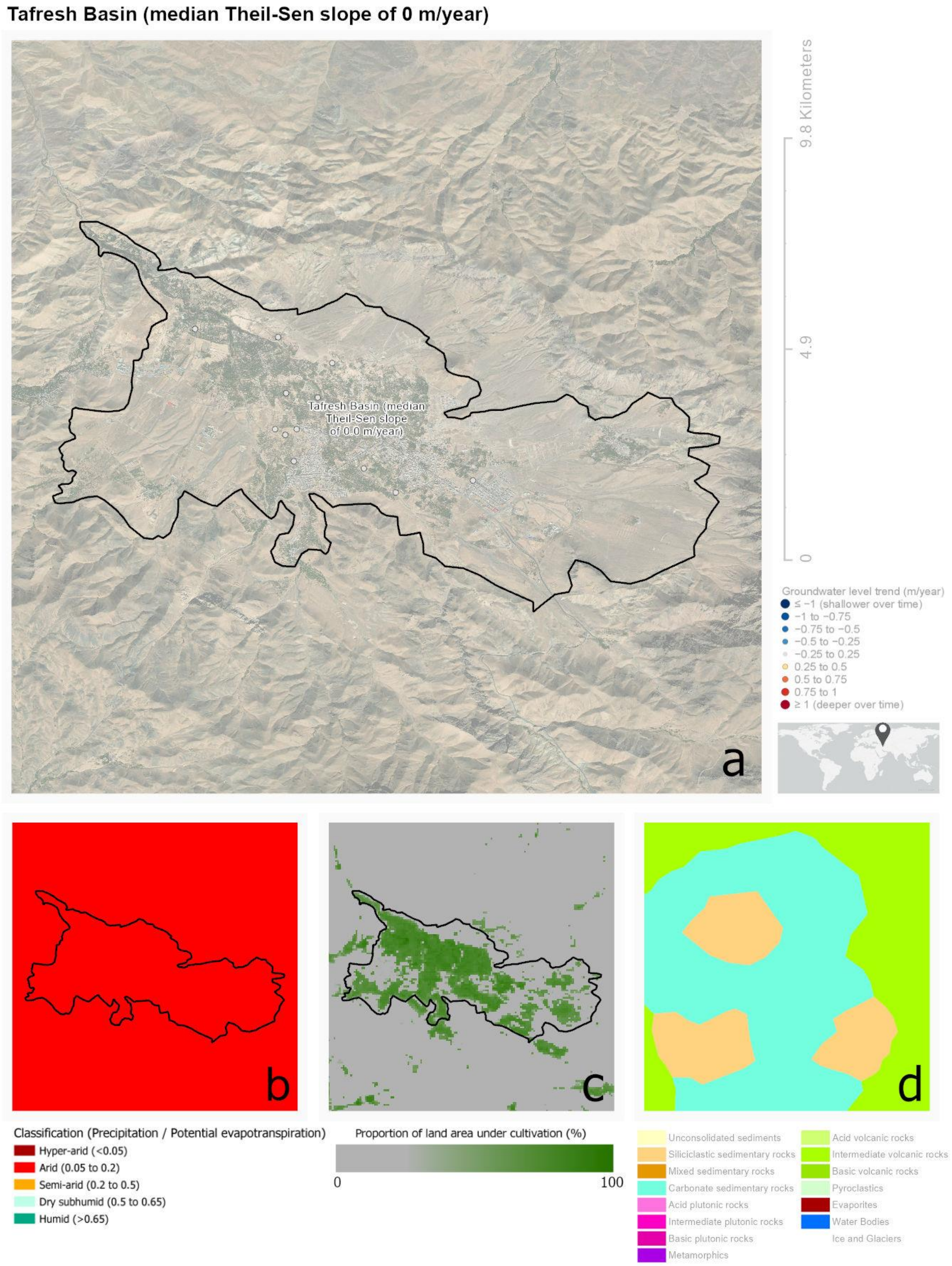

Supplementary Fig. 231. Spatial variability of trends in depth to groundwater Tafresh Basin. For detailed description of the contents of each panel see the text at the beginning of Supplementary Note 17.

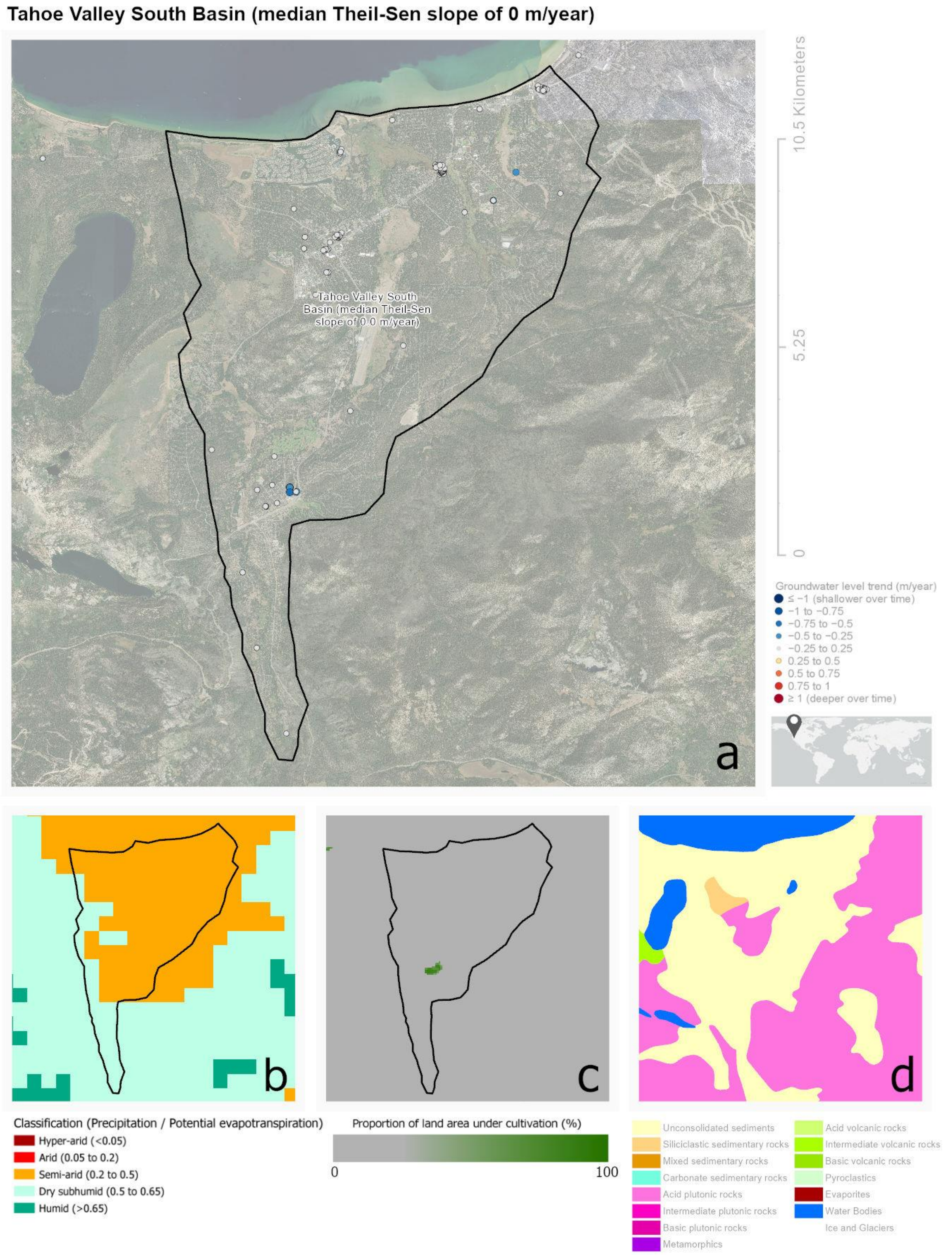

Supplementary Fig. 232. Spatial variability of trends in depth to groundwater Tahoe Valley South Basin. For detailed description of the contents of each panel see the text at the beginning of Supplementary Note 17.

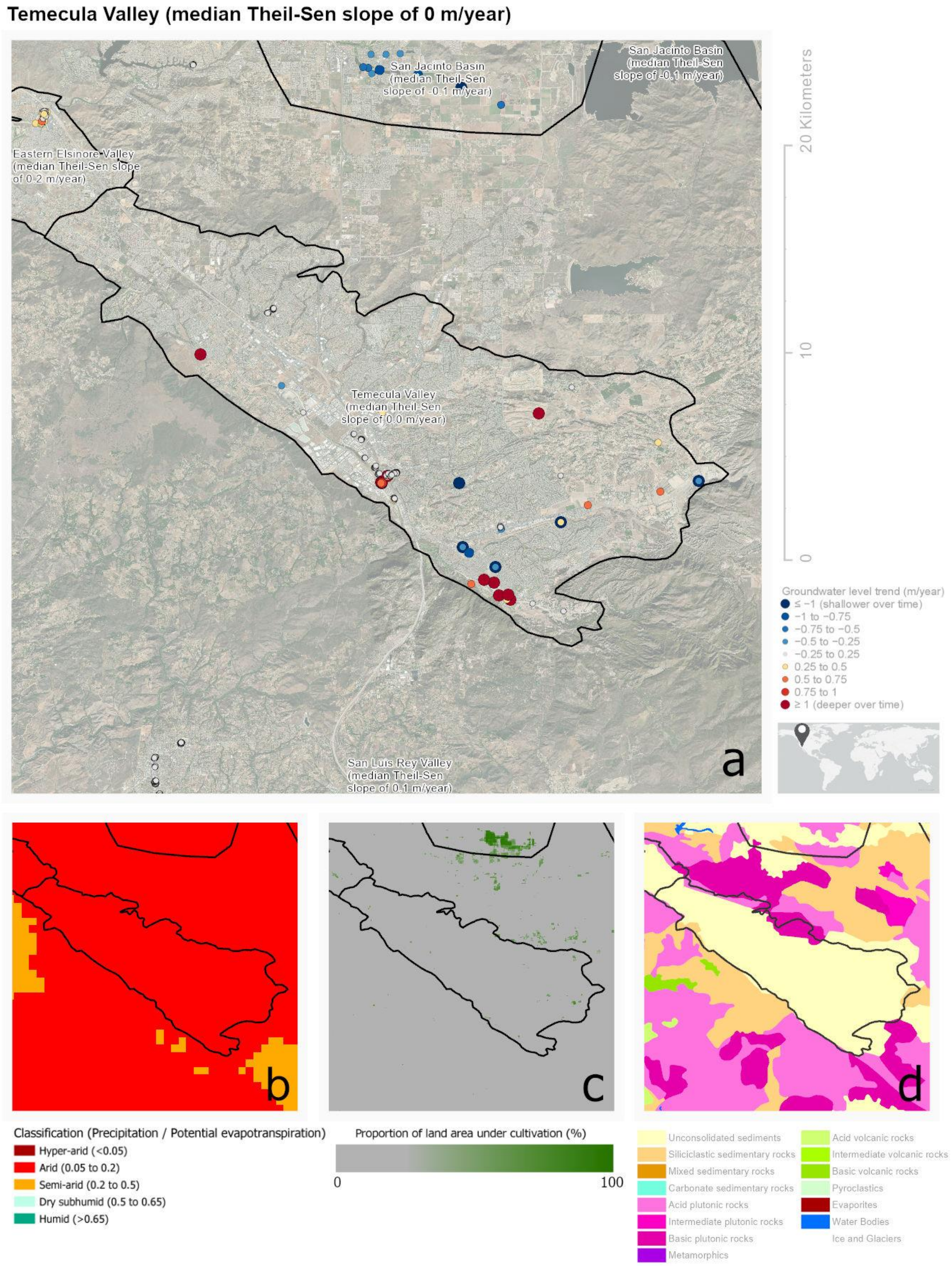

Supplementary Fig. 233. Spatial variability of trends in depth to groundwater Temecula Valley. For detailed description of the contents of each panel see the text at the beginning of Supplementary Note 17.

Temescal Basin (median Theil-Sen slope of 0.1 m/year)

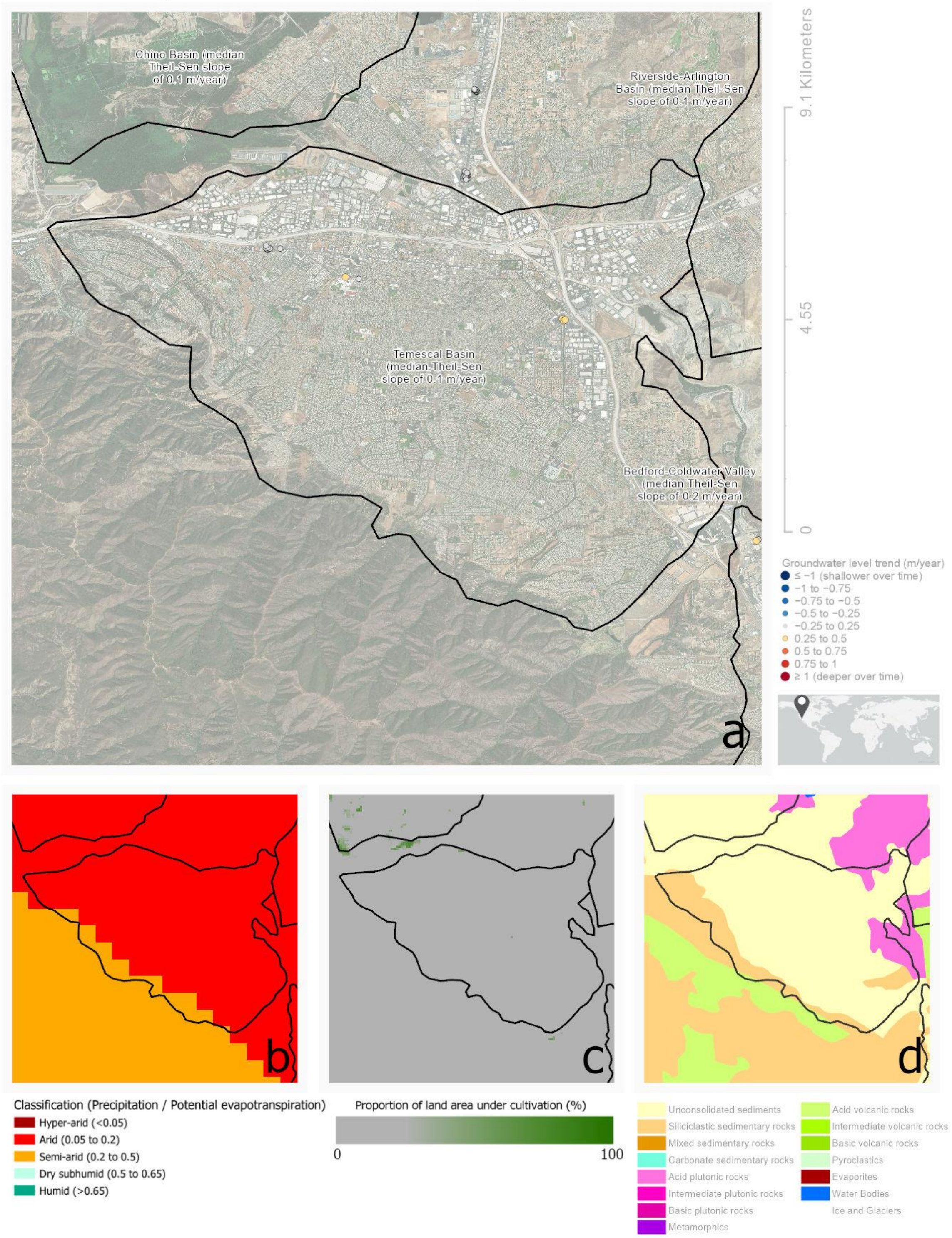

Supplementary Fig. 234. Spatial variability of trends in depth to groundwater Temescal Basin (within the broader Upper Santa Ana Basin). For detailed description of the contents of each panel see the text at the beginning of Supplementary Note 17.

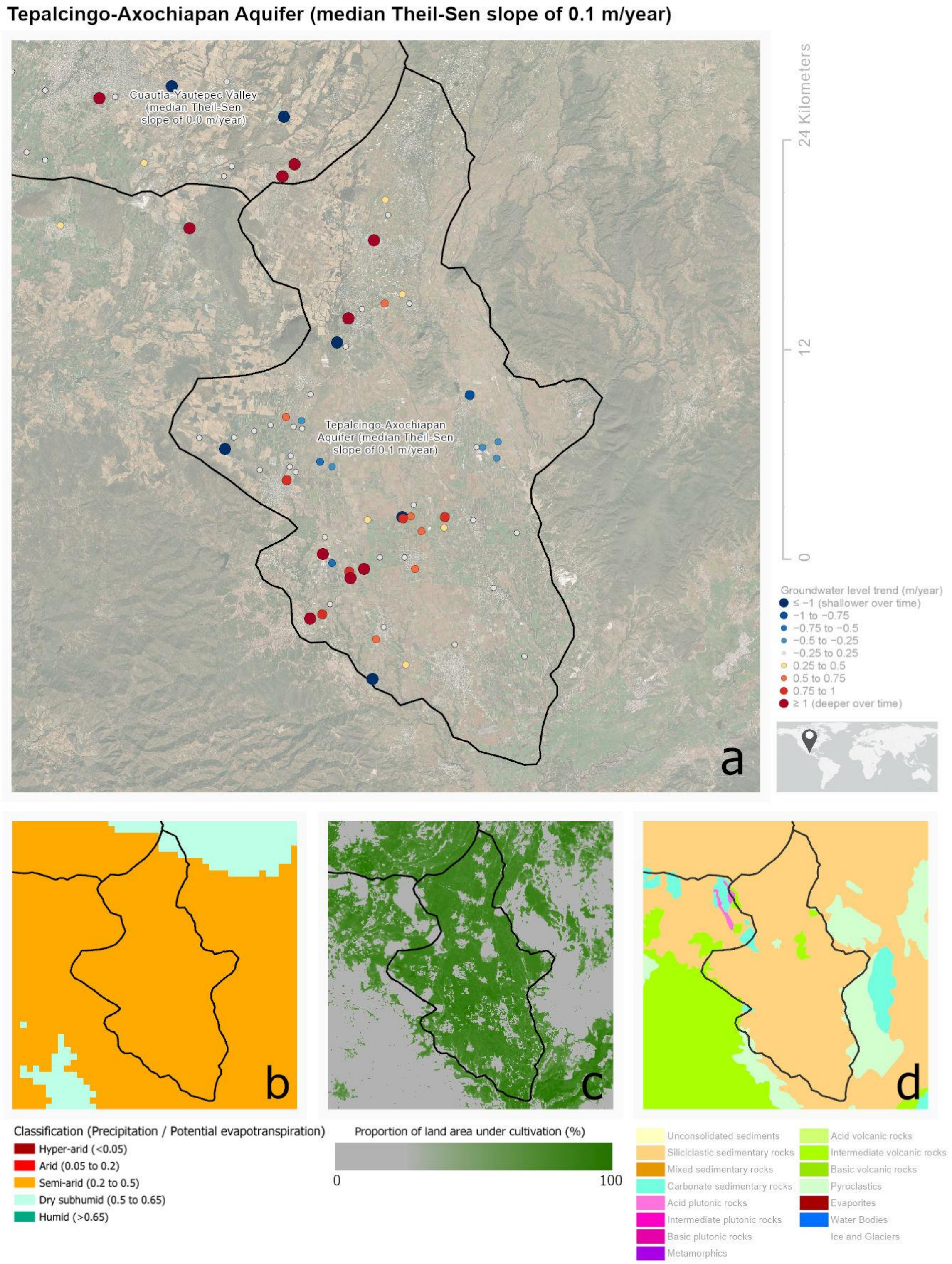

Supplementary Fig. 235. Spatial variability of trends in depth to groundwater Tepalcingo-Axochiapan Aquifer. For detailed description of the contents of each panel see the text at the beginning of Supplementary Note 17.

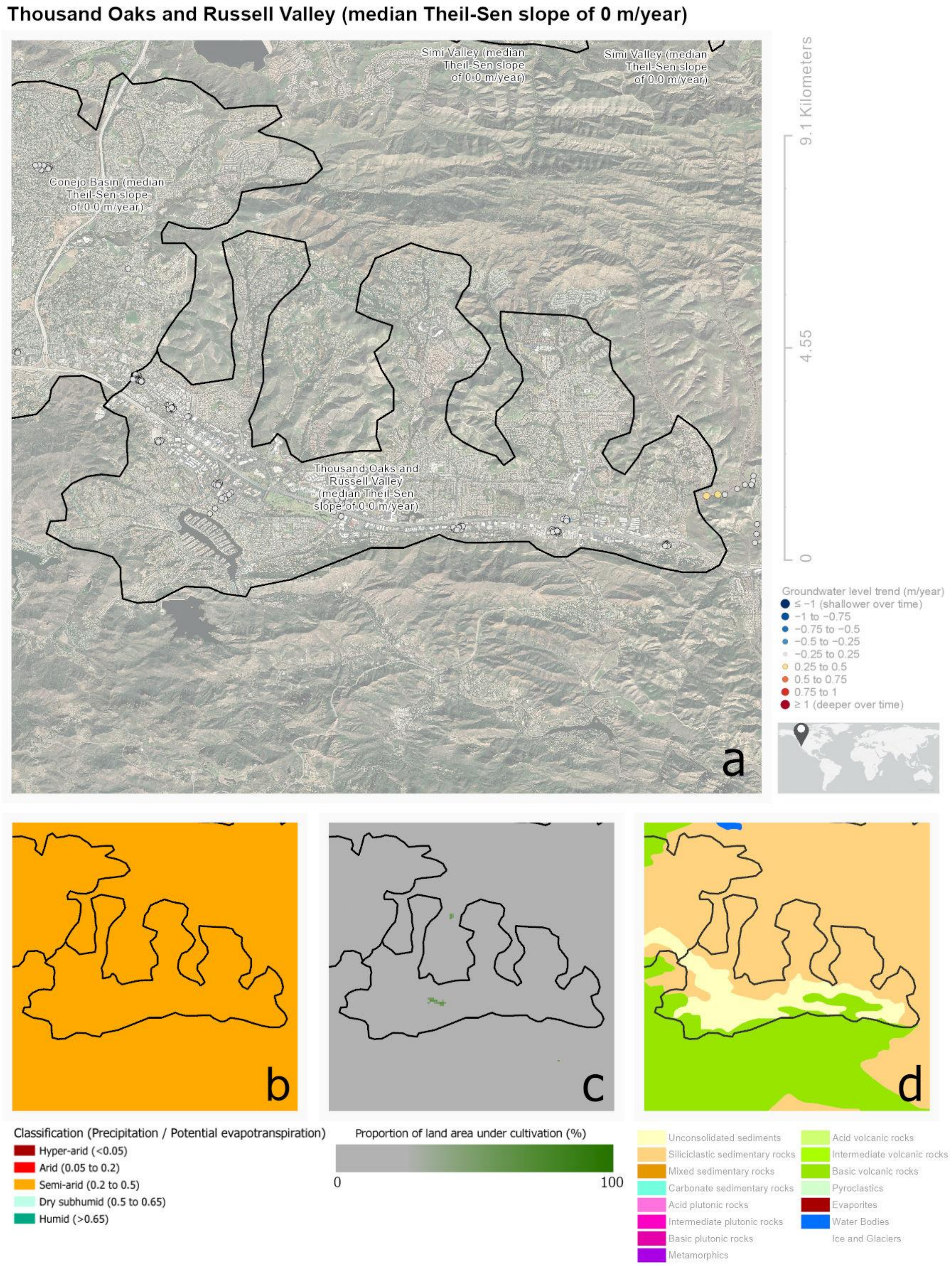

Supplementary Fig. 236. Spatial variability of trends in depth to groundwater Thousand Oaks and Russell Valley. For detailed description of the contents of each panel see the text at the beginning of Supplementary Note 17.

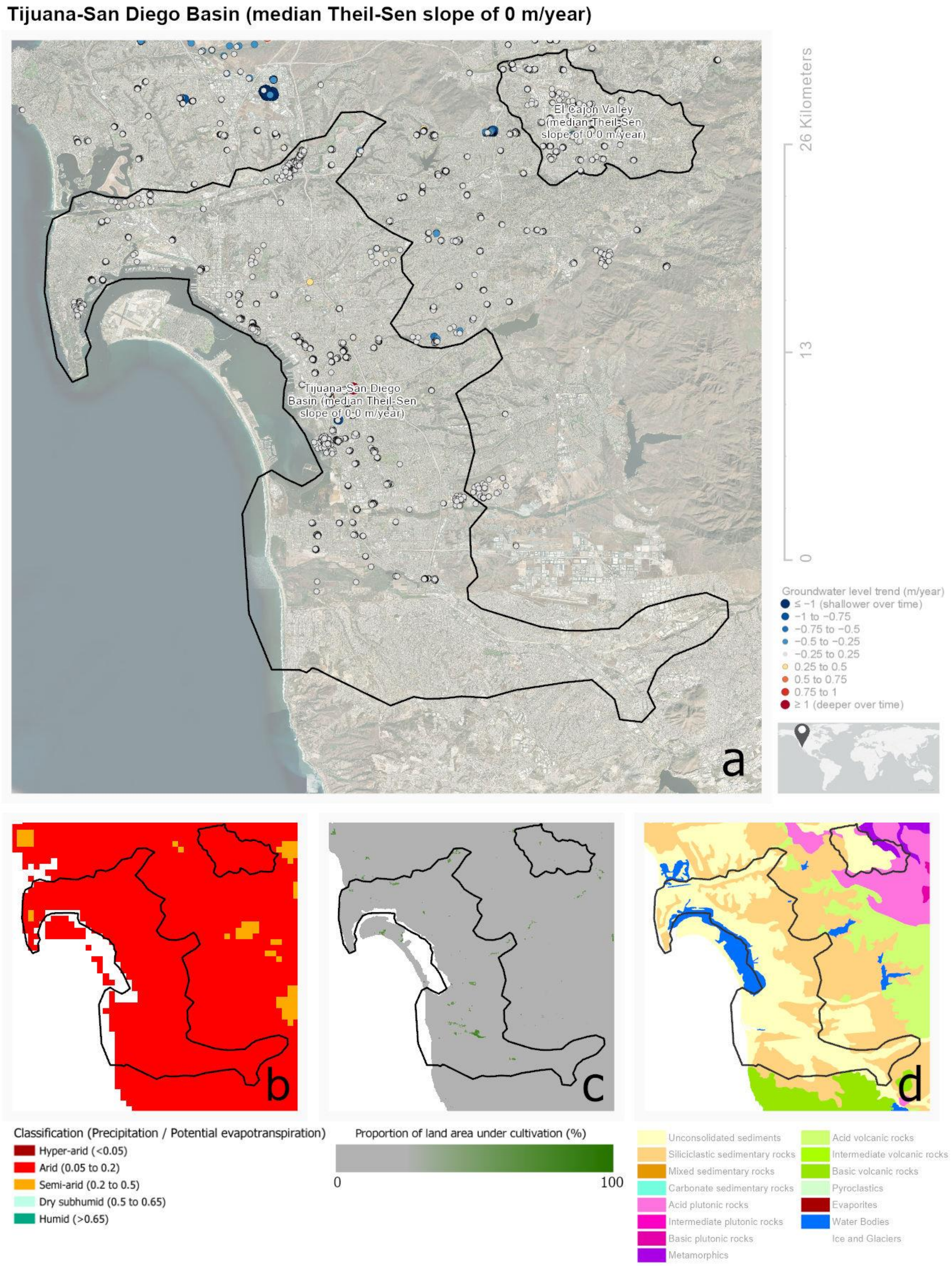

Supplementary Fig. 237. Spatial variability of trends in depth to groundwater Tijuana-San Diego Basin. For detailed description of the contents of each panel see the text at the beginning of Supplementary Note 17.

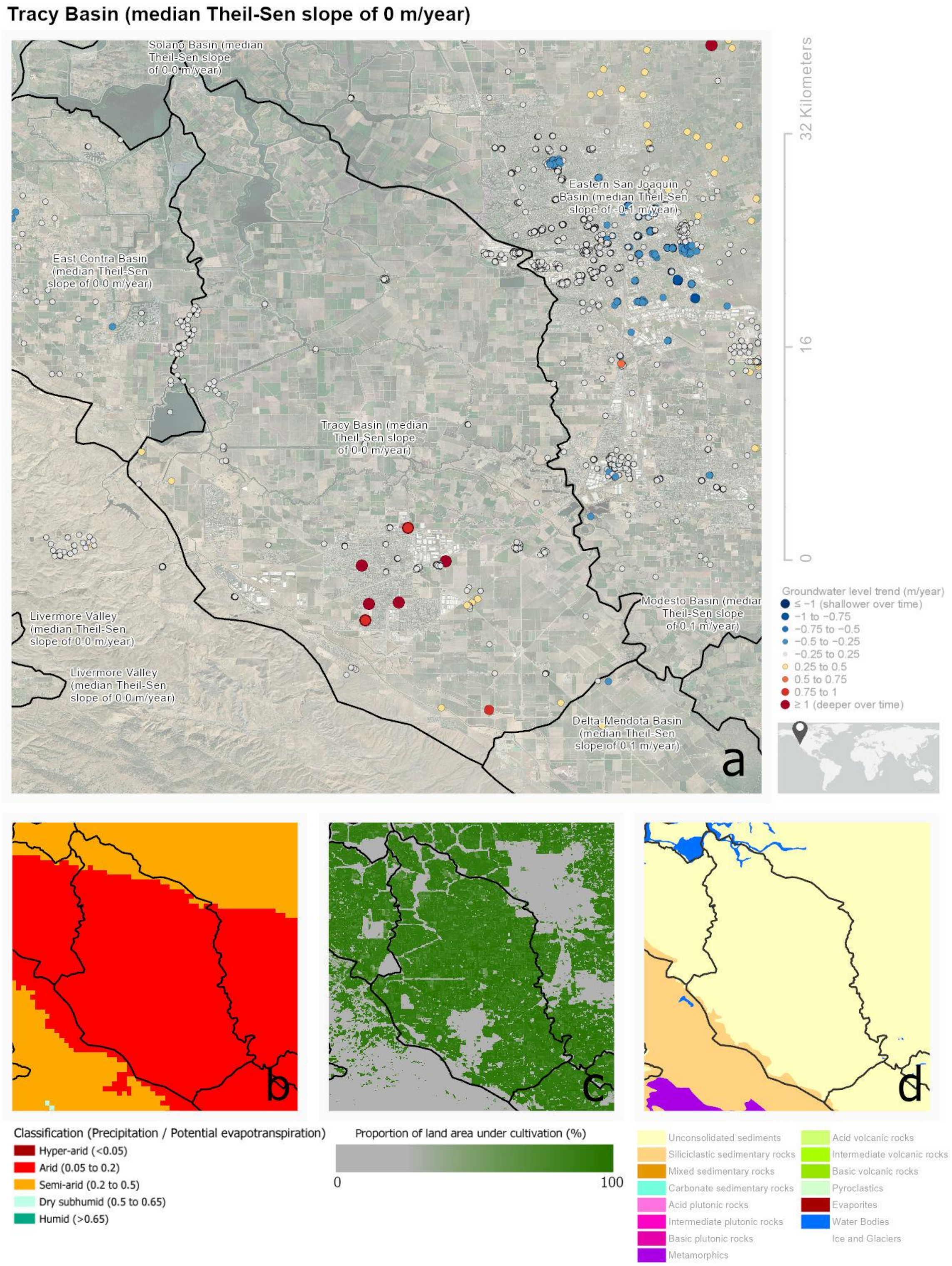

Supplementary Fig. 238. Spatial variability of trends in depth to groundwater Tracy Basin (within the broader San Joaquin Basin of the Central Valley). For detailed description of the contents of each panel see the text at the beginning of Supplementary Note 17.

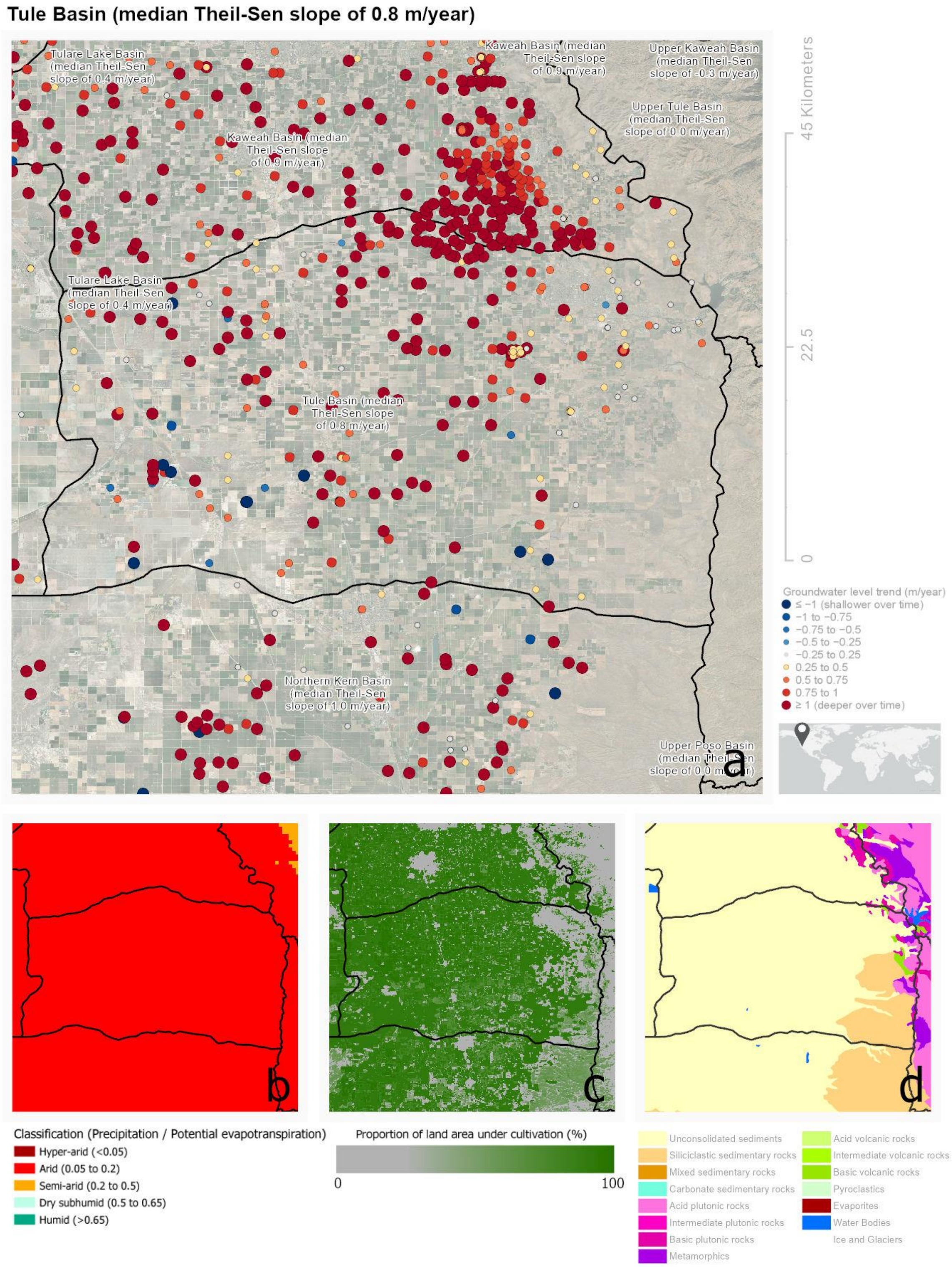

Supplementary Fig. 239. Spatial variability of trends in depth to groundwater Tule Basin (within the broader Tulare Basin of the Central Valley). For detailed description of the contents of each panel see the text at the beginning of Supplementary Note 17.

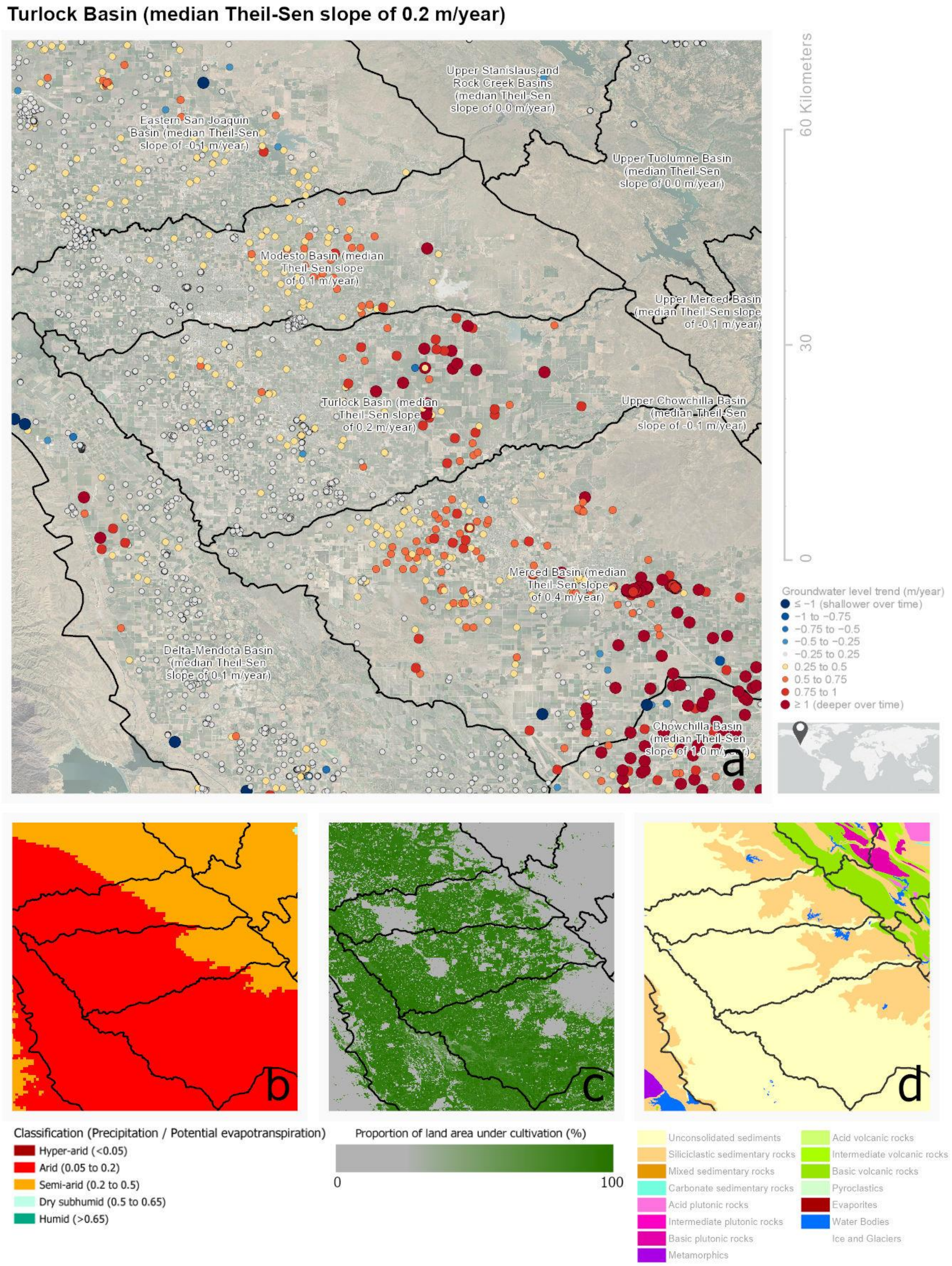

Supplementary Fig. 240. Spatial variability of trends in depth to groundwater Turlock Basin (within the broader San Joaquin Basin of the Central Valley). For detailed description of the contents of each panel see the text at the beginning of Supplementary Note 17.

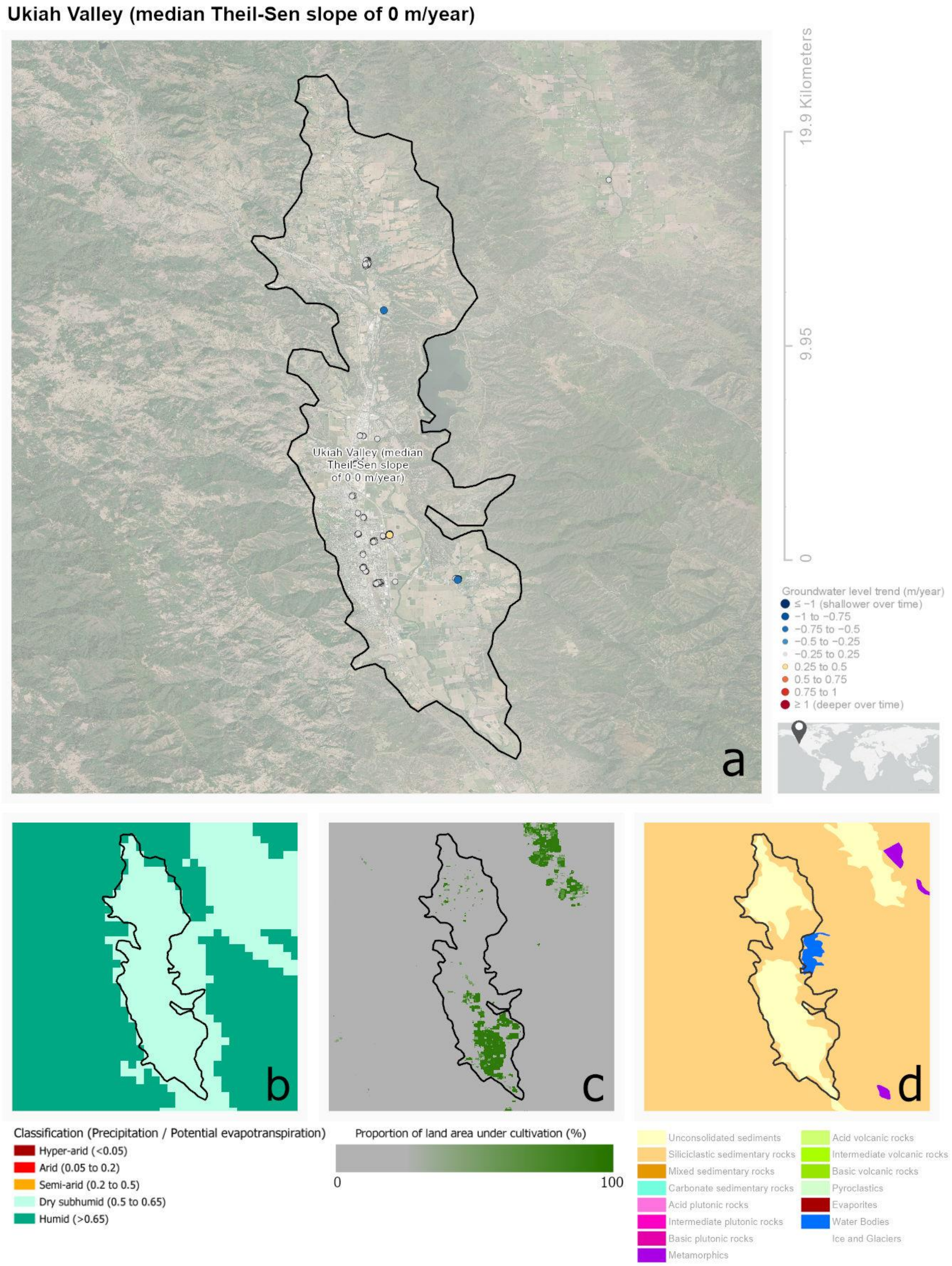

Supplementary Fig. 241. Spatial variability of trends in depth to groundwater Ukiah Valley. For detailed description of the contents of each panel see the text at the beginning of Supplementary Note 17.

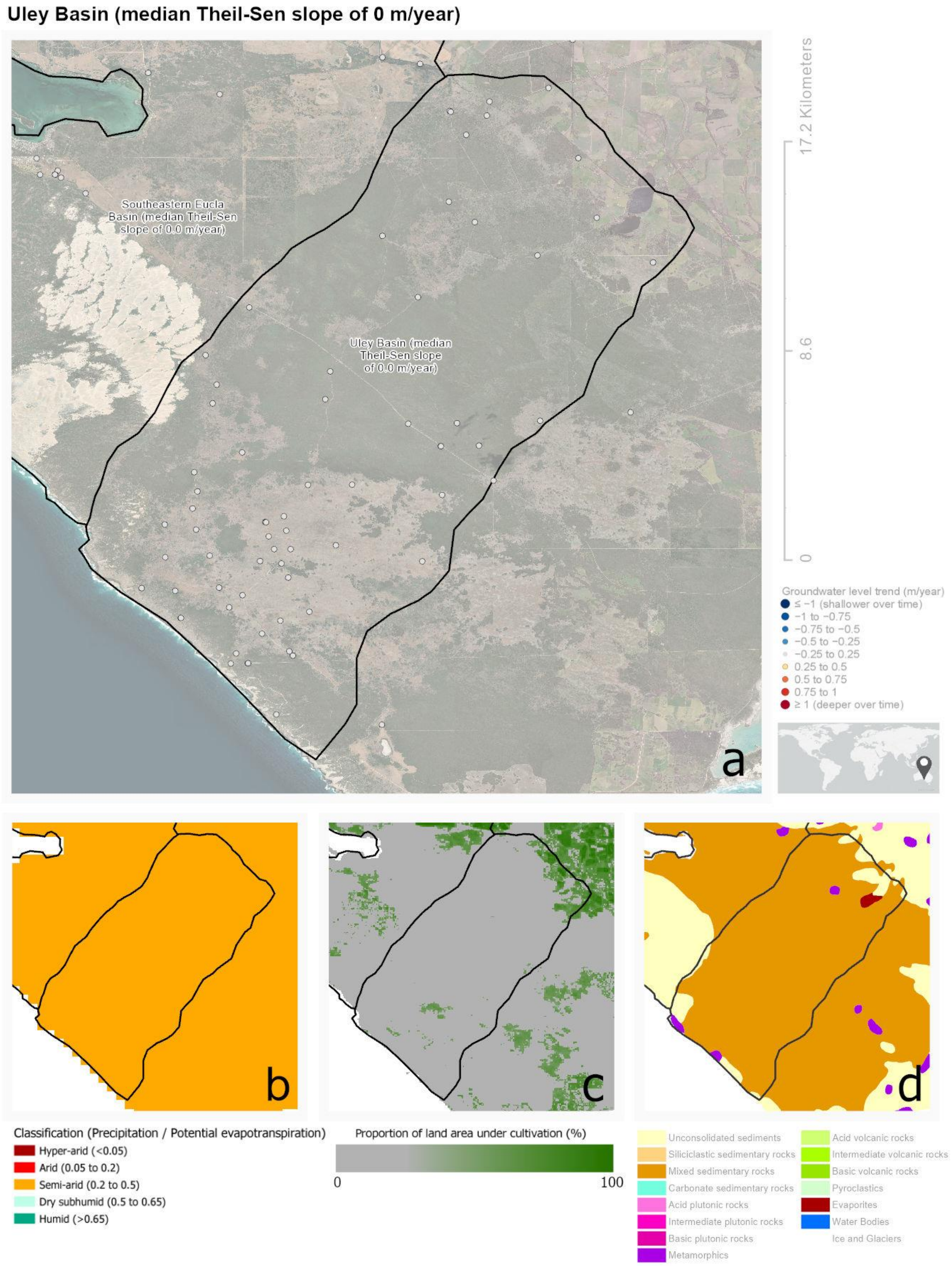

Supplementary Fig. 242. Spatial variability of trends in depth to groundwater Uley Basin (within the broader Eucla Basin). For detailed description of the contents of each panel see the text at the beginning of Supplementary Note 17.

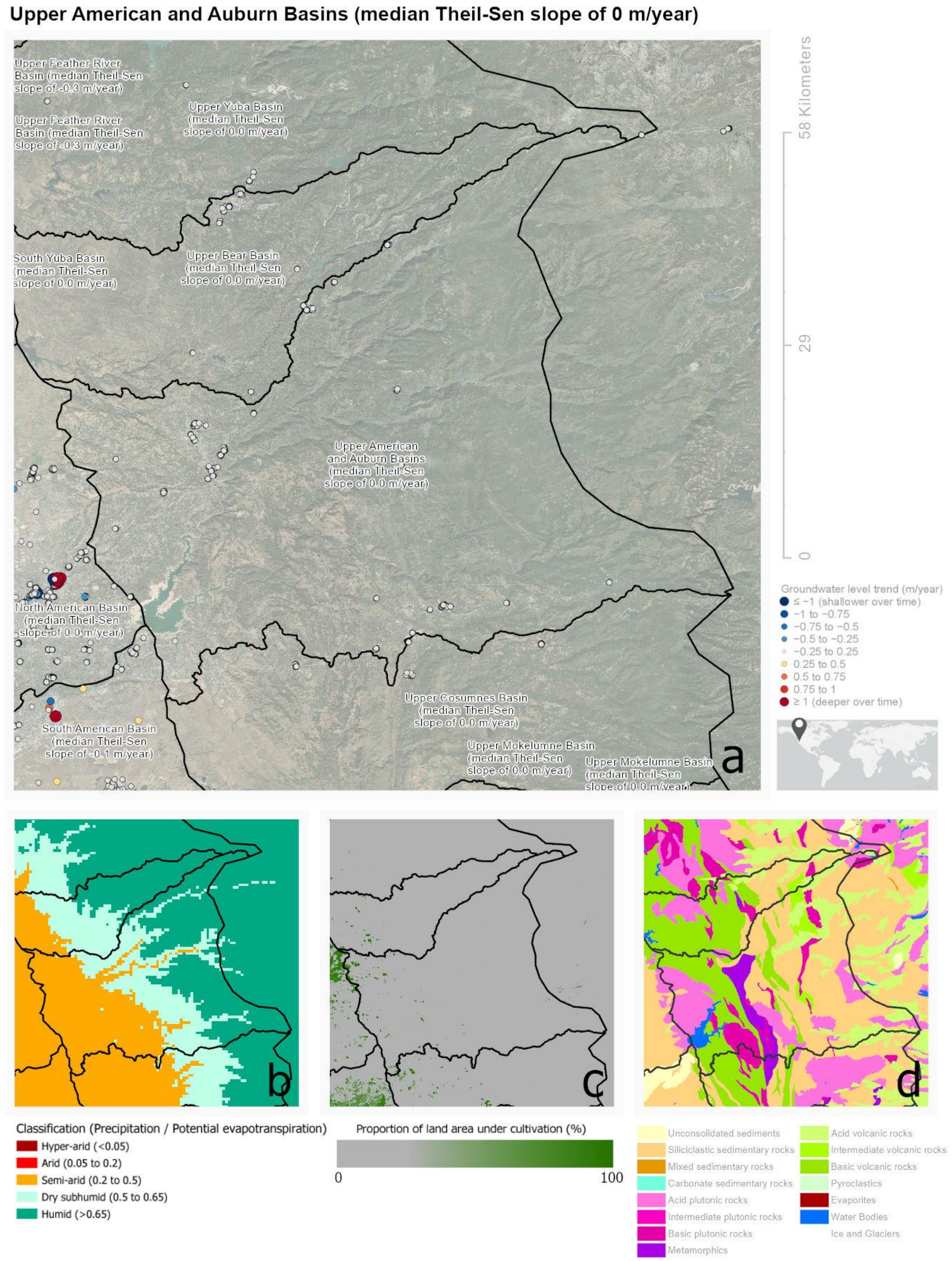

Supplementary Fig. 243. Spatial variability of trends in depth to groundwater Upper American and Auburn Basins (within the broader Sierra Nevada Foothills). For detailed description of the contents of each panel see the text at the beginning of Supplementary Note 17.

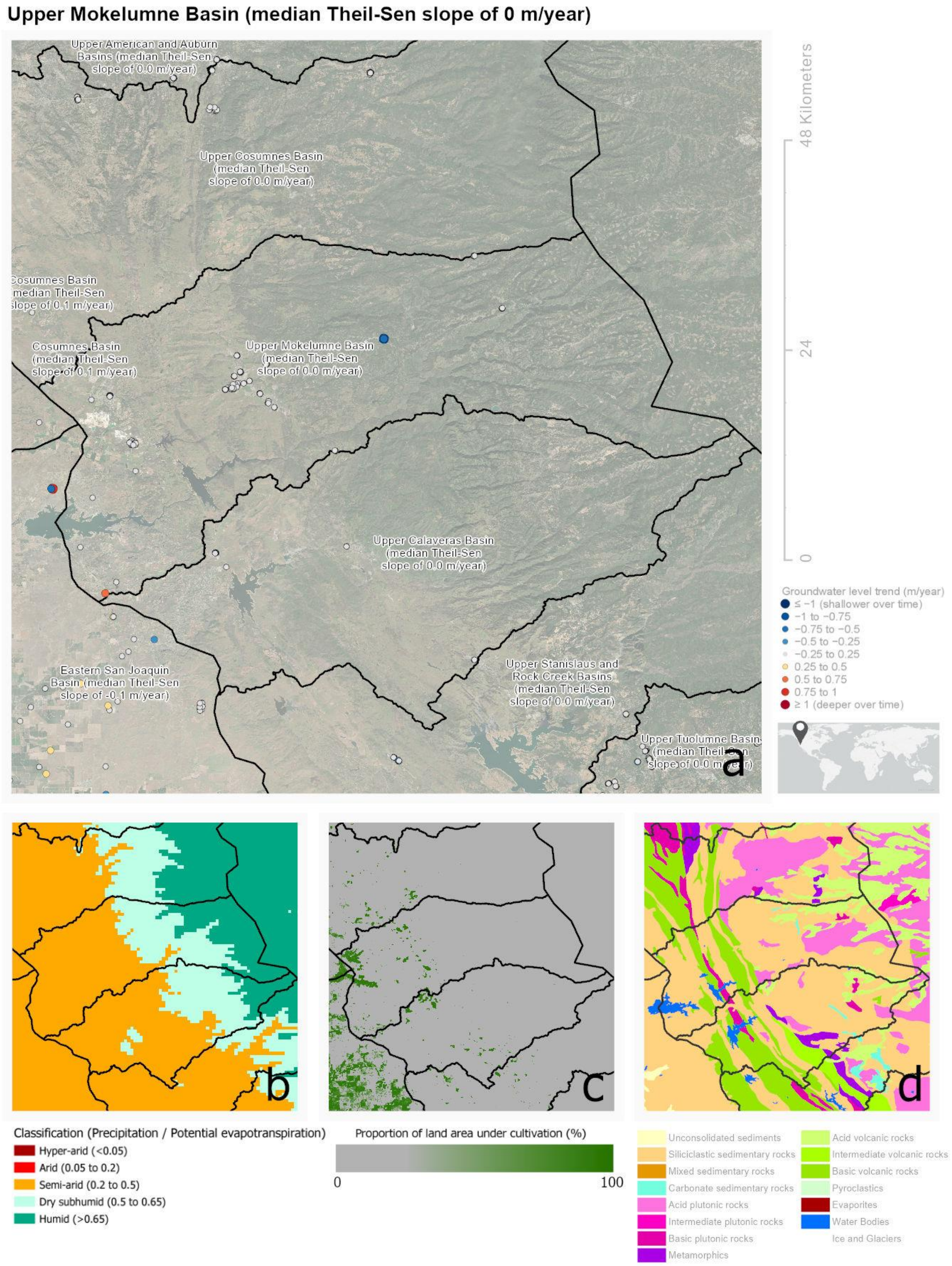

Supplementary Fig. 244. Spatial variability of trends in depth to groundwater Upper Mokelumne Basin (within the broader Sierra Nevada Foothills). For detailed description of the contents of each panel see the text at the beginning of Supplementary Note 17.

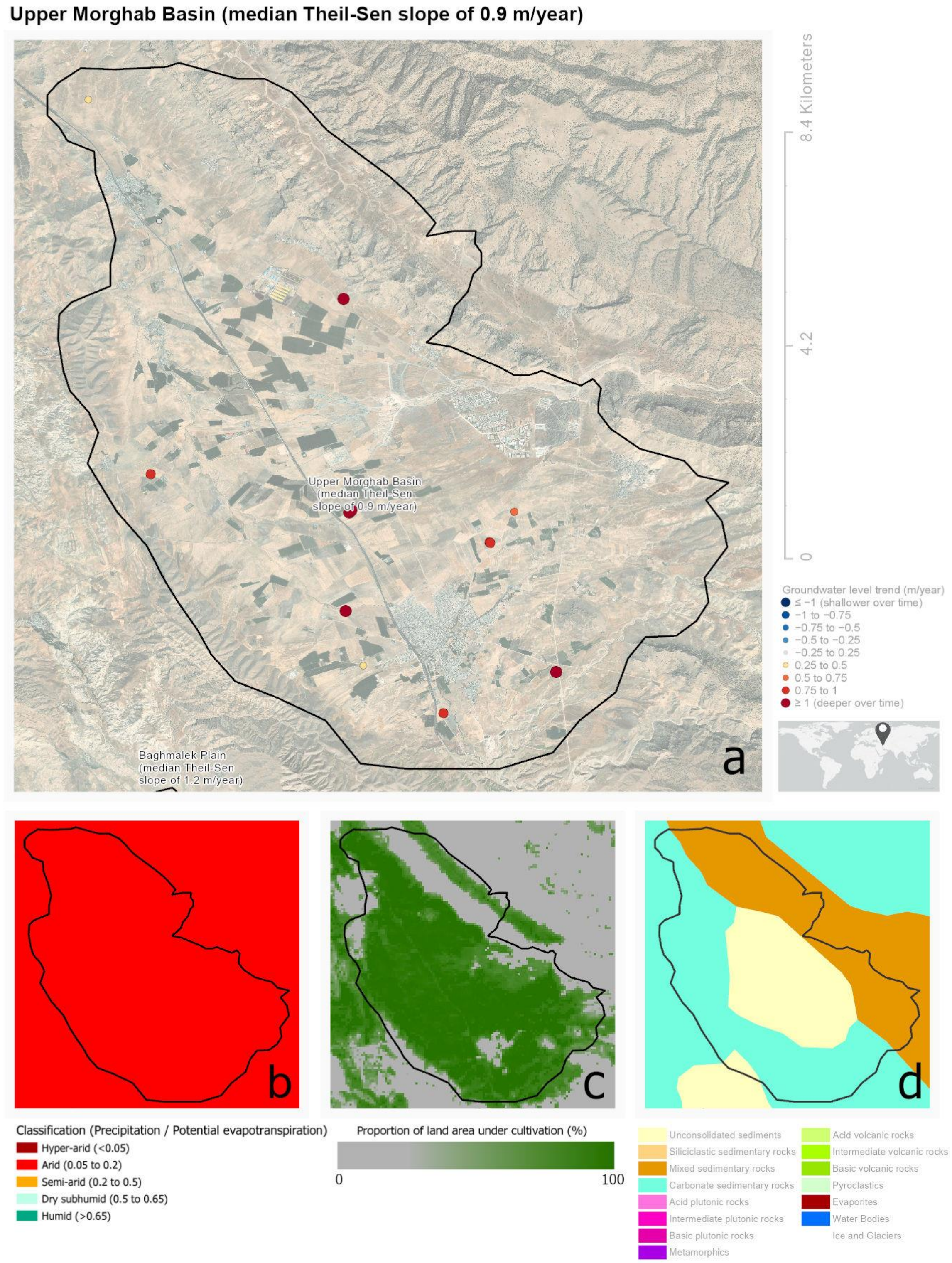

Supplementary Fig. 245. Spatial variability of trends in depth to groundwater Upper Morghab Basin. For detailed description of the contents of each panel see the text at the beginning of Supplementary Note 17.

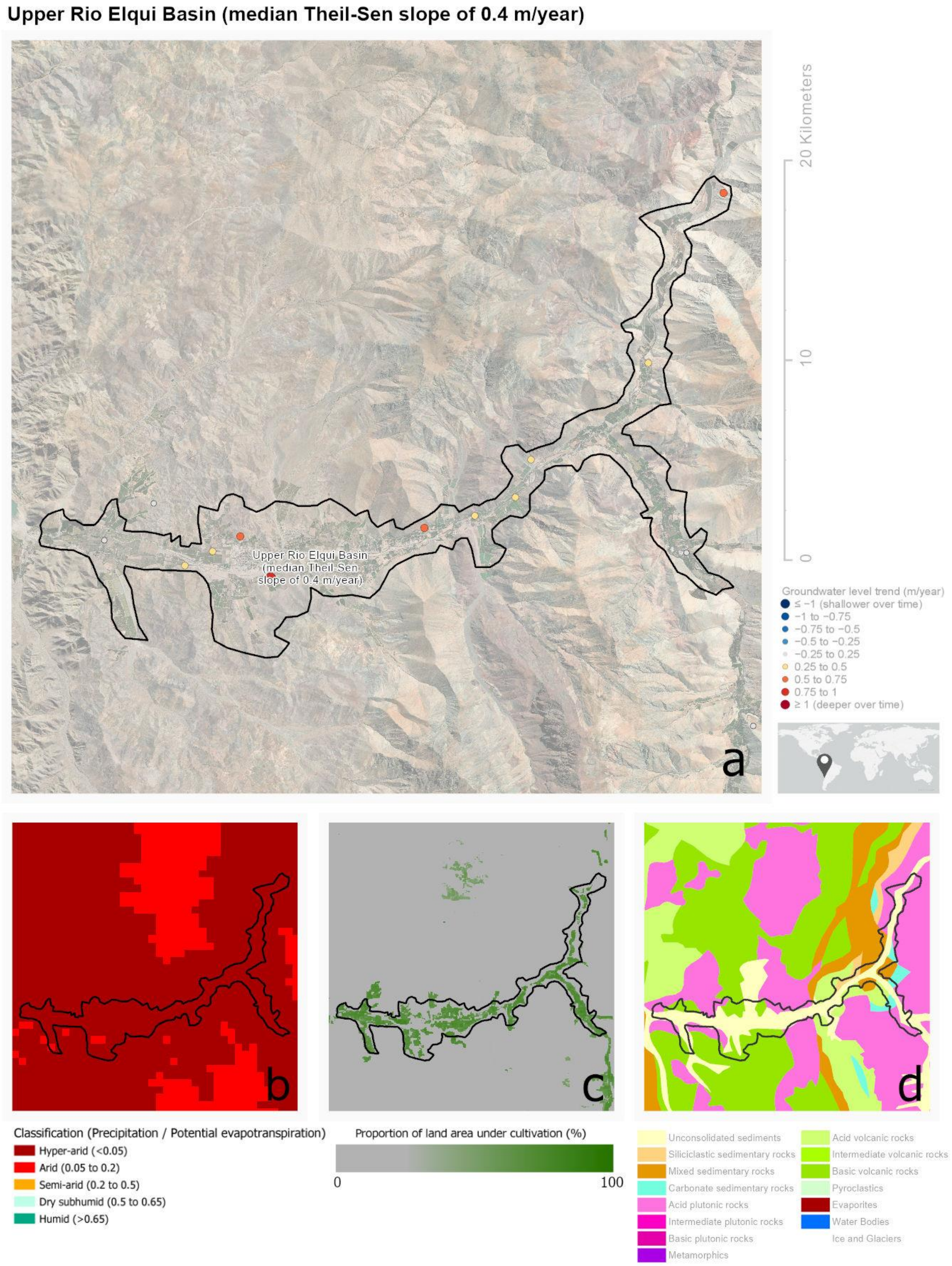

Supplementary Fig. 246. Spatial variability of trends in depth to groundwater Upper Rio Elqui Basin (within the broader Elqui River Basin). For detailed description of the contents of each panel see the text at the beginning of Supplementary Note 17.

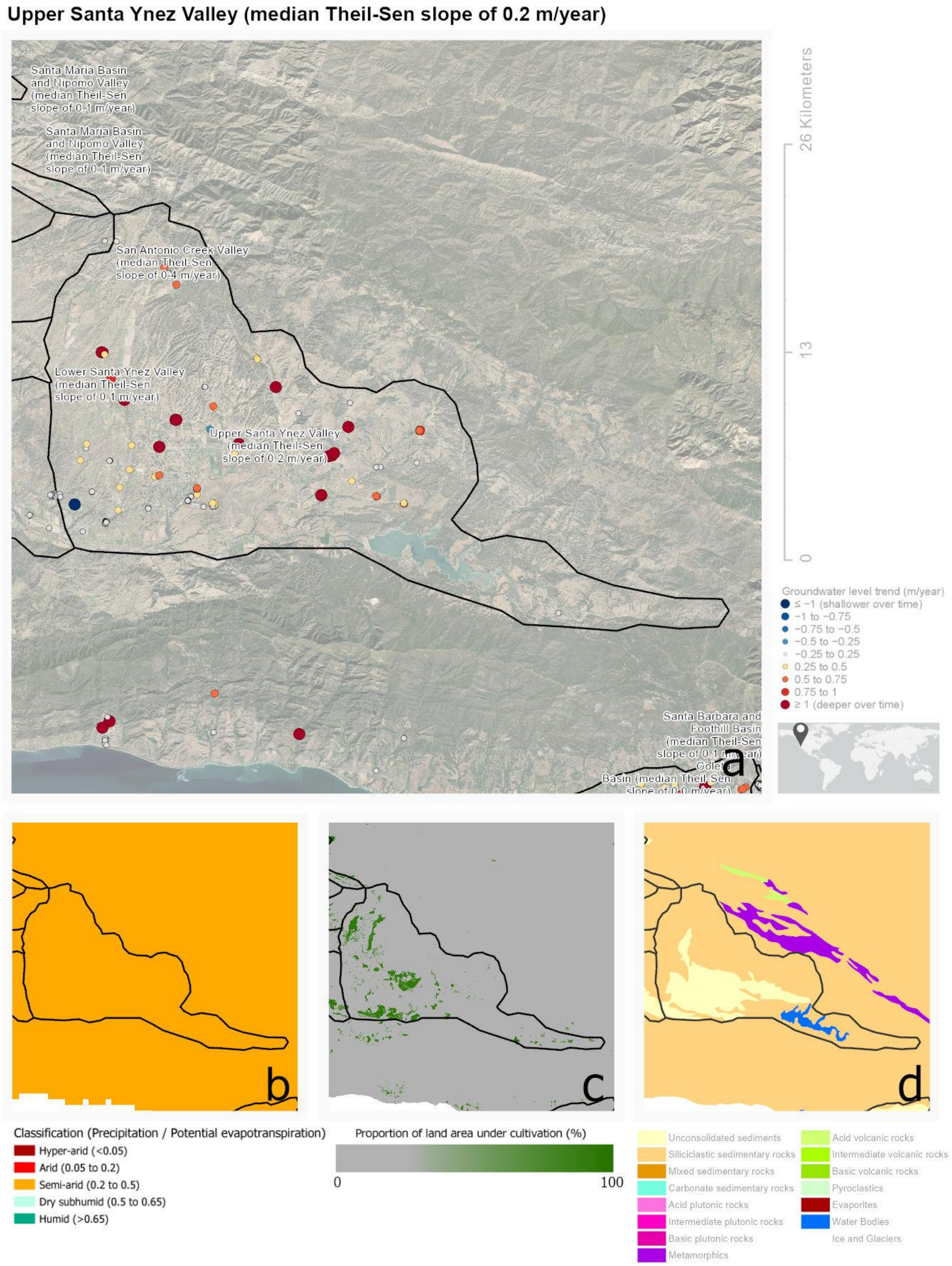

Supplementary Fig. 247. Spatial variability of trends in depth to groundwater Upper Santa Ynez Valley (within the broader Santa Ynez Valley). For detailed description of the contents of each panel see the text at the beginning of Supplementary Note 17.

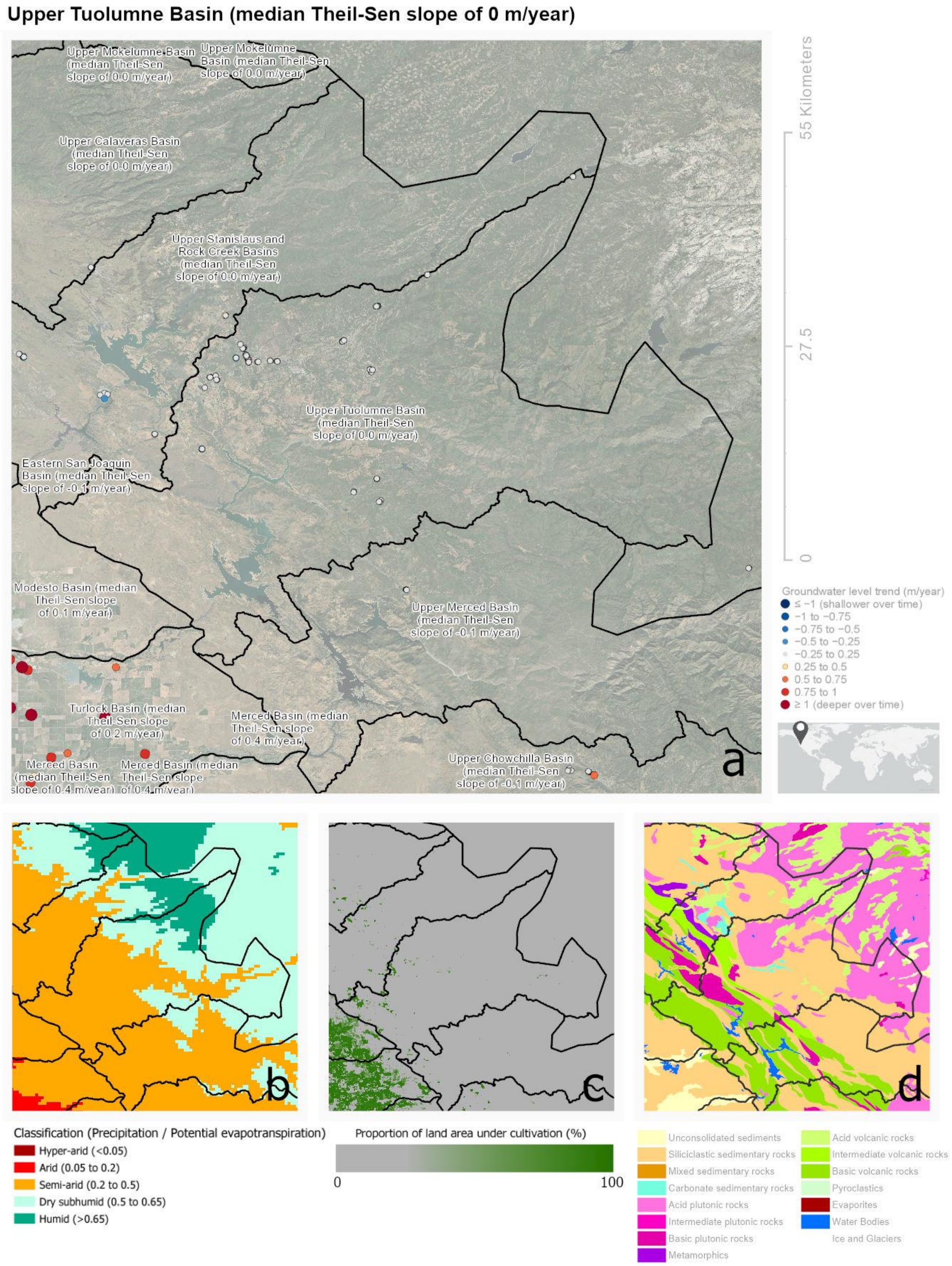

Supplementary Fig. 248. Spatial variability of trends in depth to groundwater Upper Tuolumne Basin (within the broader Sierra Nevada Foothills). For detailed description of the contents of each panel see the text at the beginning of Supplementary Note 17.

Vina Basin (median Theil-Sen slope of 0.2 m/year)

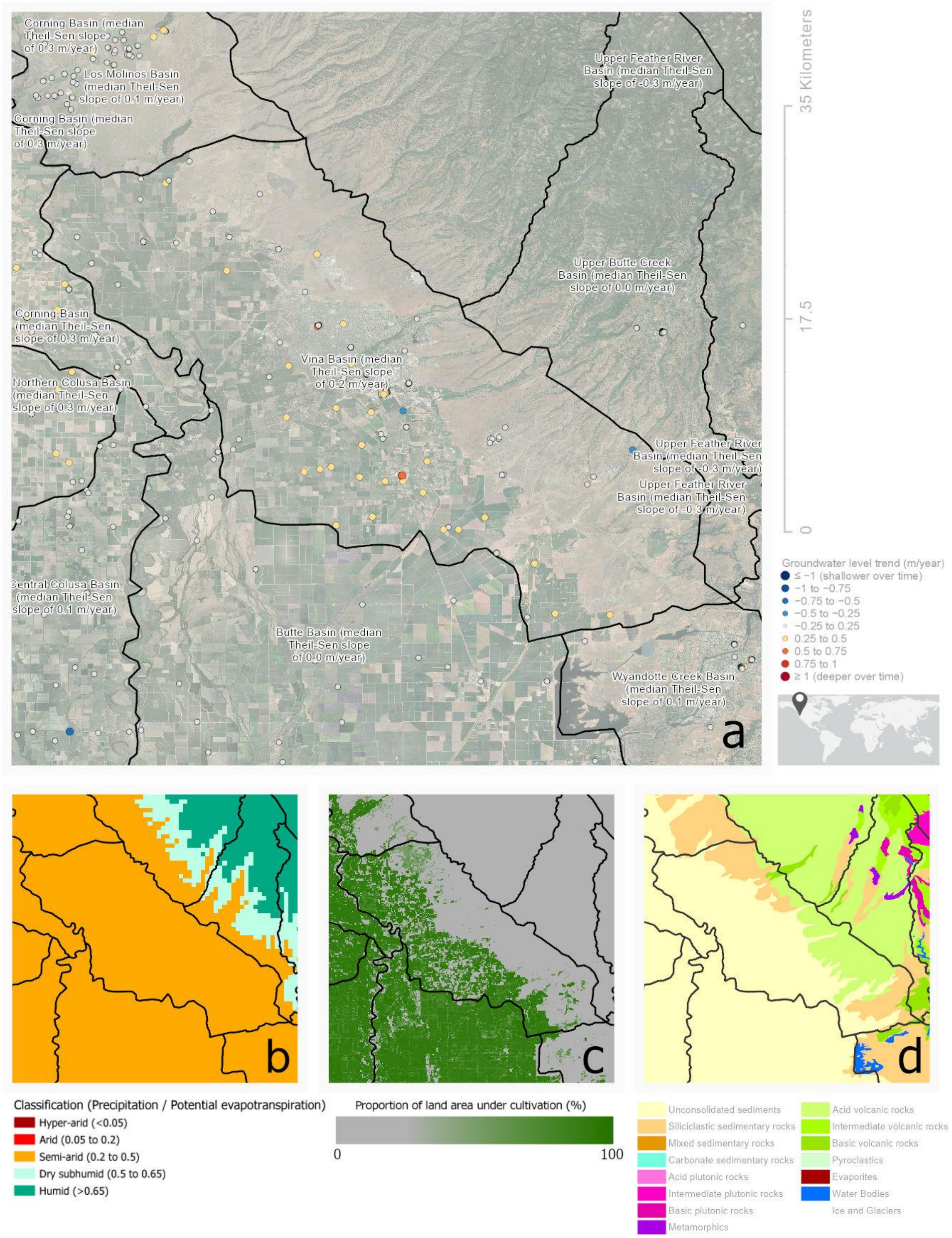

Supplementary Fig. 249. Spatial variability of trends in depth to groundwater Vina Basin (within the broader Sacramento Valley of the Central Valley). For detailed description of the contents of each panel see the text at the beginning of Supplementary Note 17.

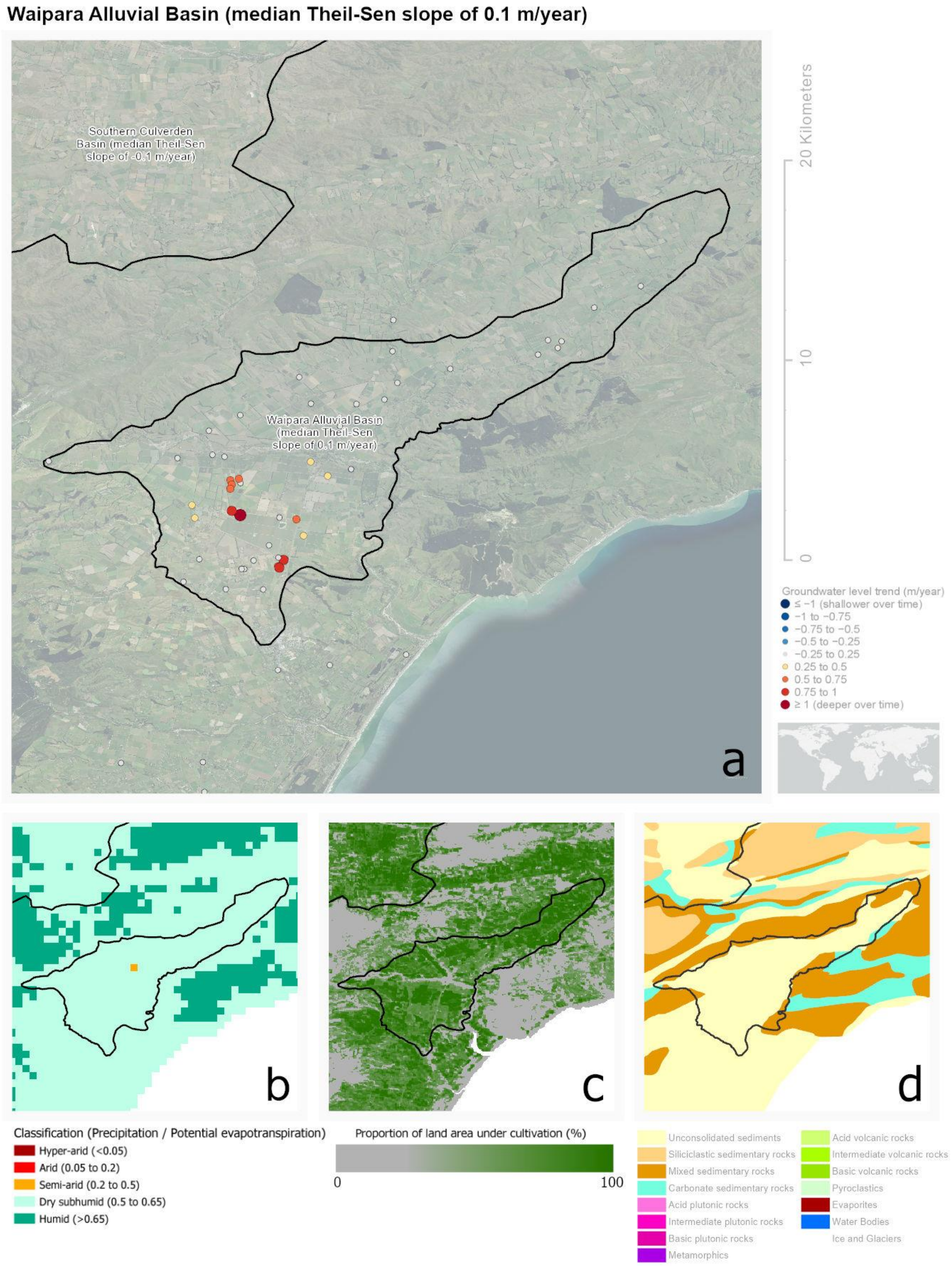

Supplementary Fig. 250. Spatial variability of trends in depth to groundwater Waipara Alluvial Basin. For detailed description of the contents of each panel see the text at the beginning of Supplementary Note 17.

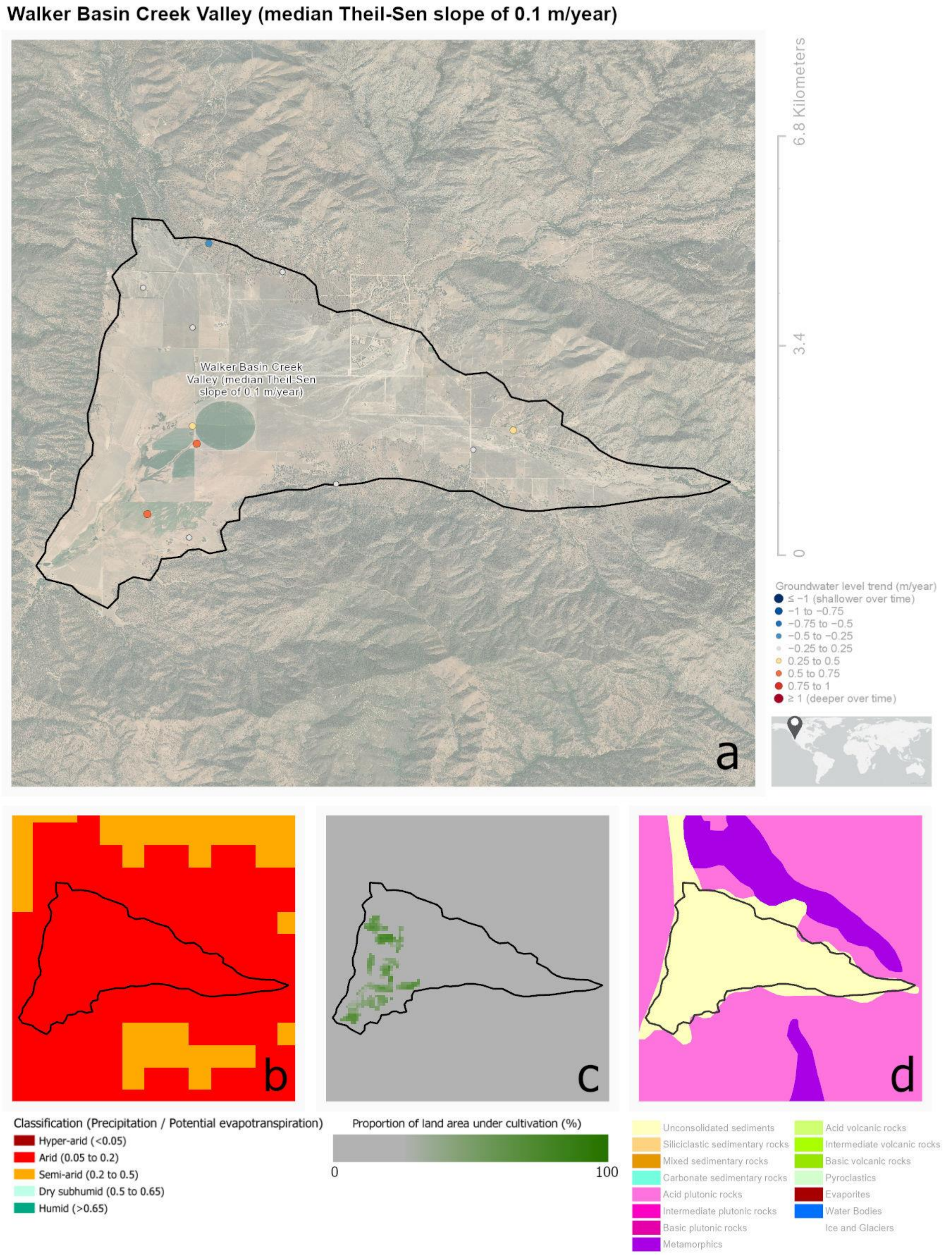

Supplementary Fig. 251. Spatial variability of trends in depth to groundwater Walker Basin Creek Valley. For detailed description of the contents of each panel see the text at the beginning of Supplementary Note 17.

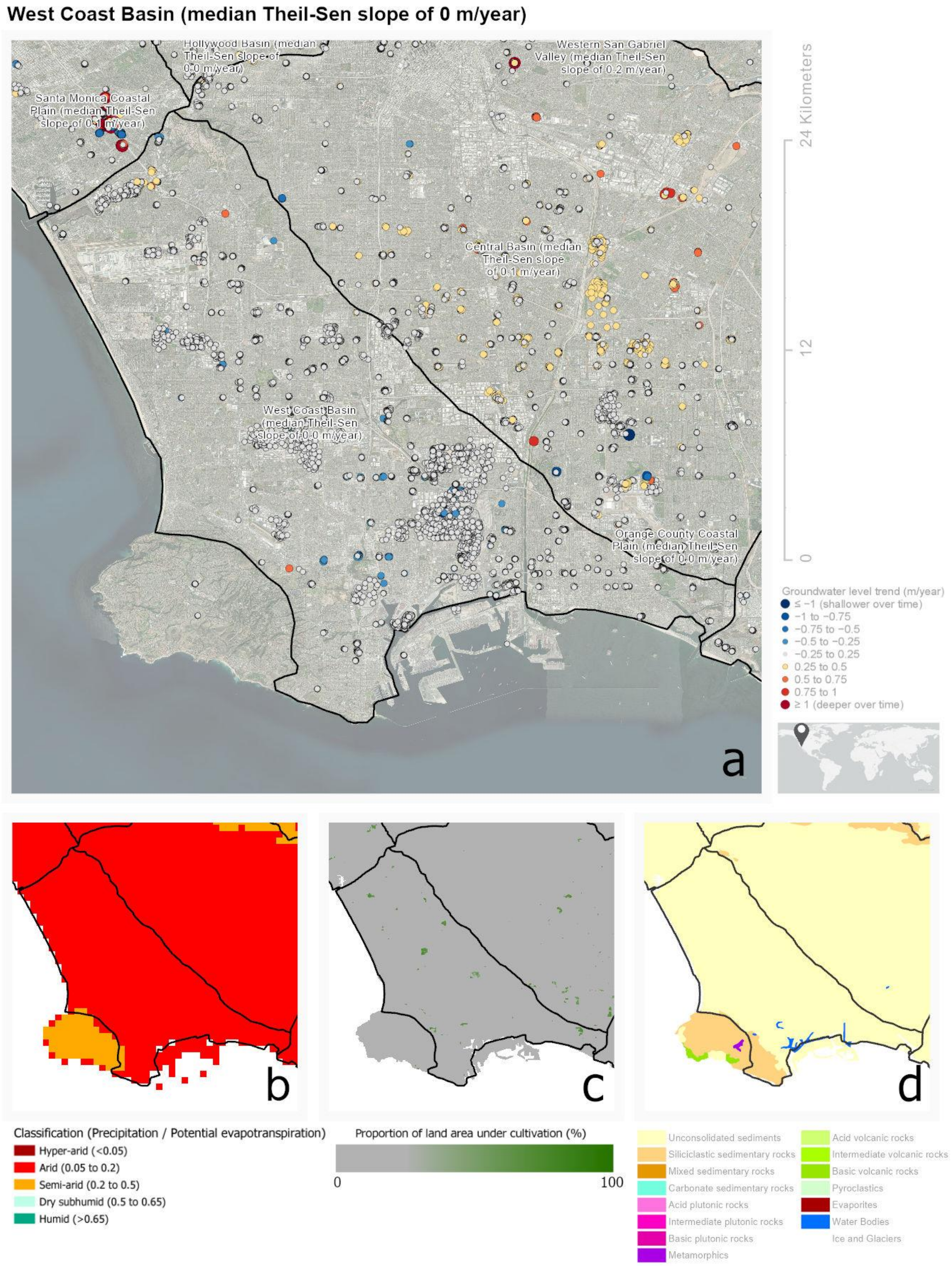

Supplementary Fig. 252. Spatial variability of trends in depth to groundwater West Coast Basin (within the broader Los Angeles Basin). For detailed description of the contents of each panel see the text at the beginning of Supplementary Note 17.

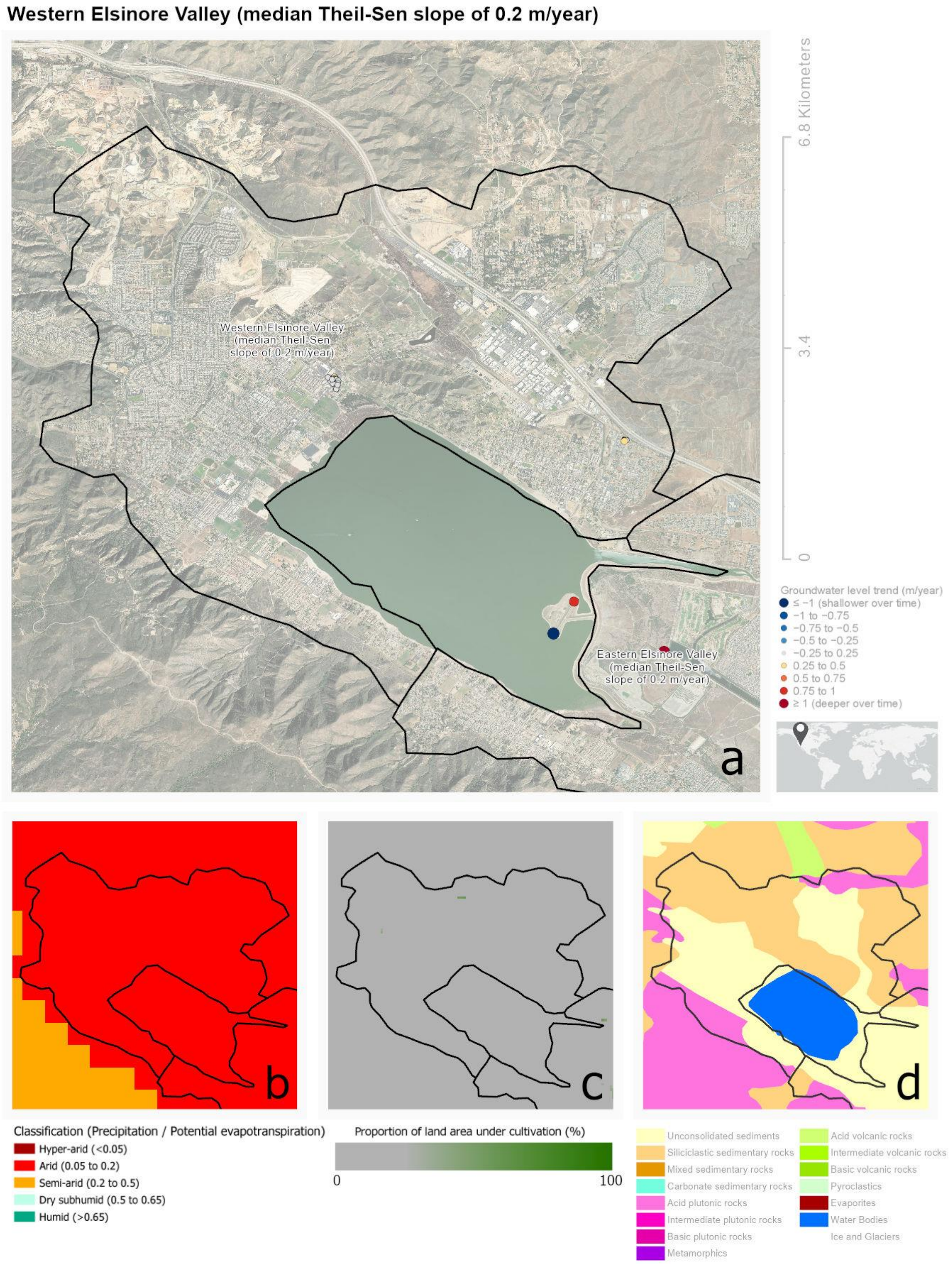

Supplementary Fig. 253. Spatial variability of trends in depth to groundwater Western Elsinore Valley. For detailed description of the contents of each panel see the text at the beginning of Supplementary Note 17.

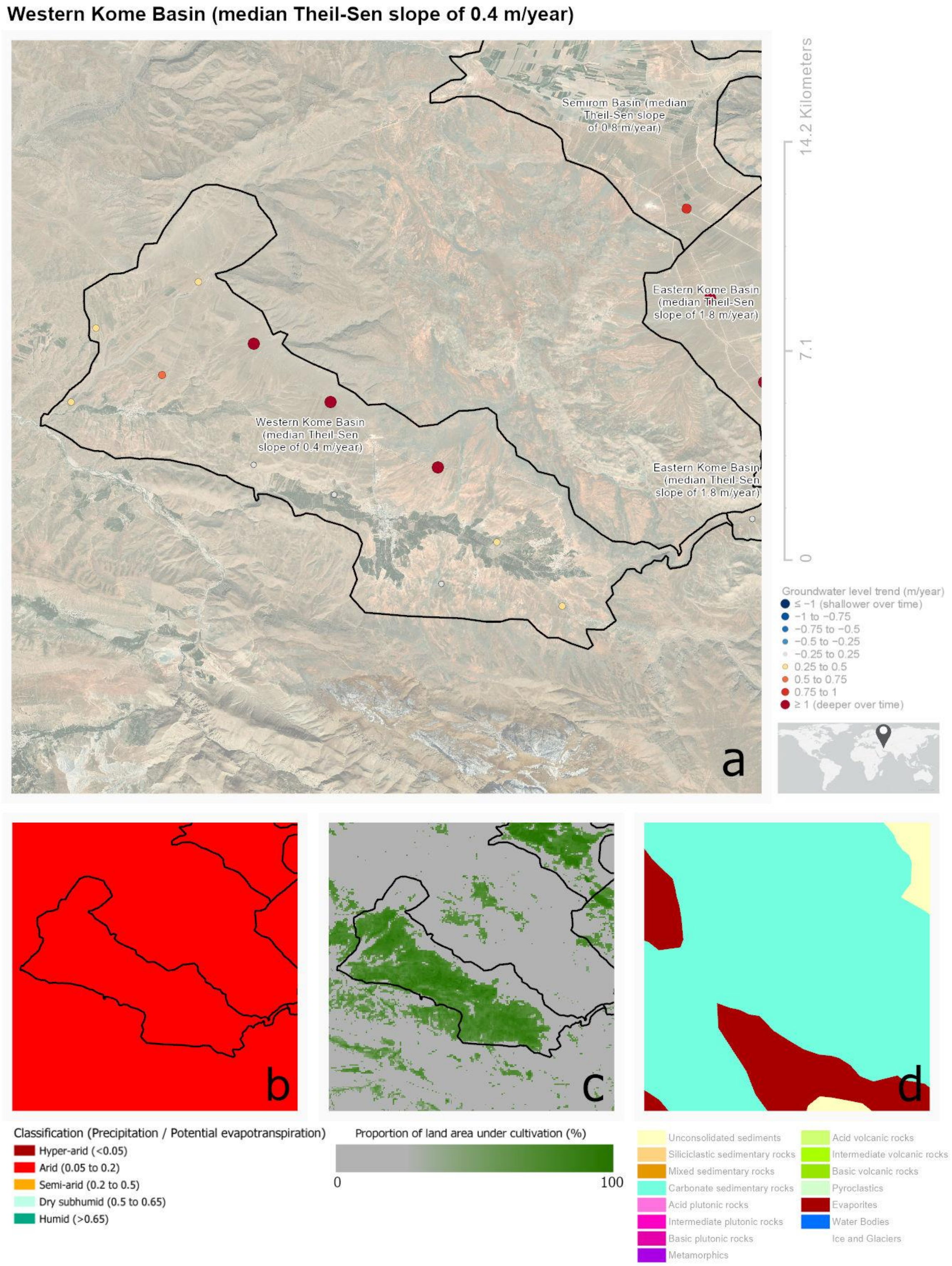

Supplementary Fig. 254. Spatial variability of trends in depth to groundwater Western Kome Basin (within the broader Kome Basin). For detailed description of the contents of each panel see the text at the beginning of Supplementary Note 17.

Western Lordegan Basin (median Theil-Sen slope of 0.6 m/year)

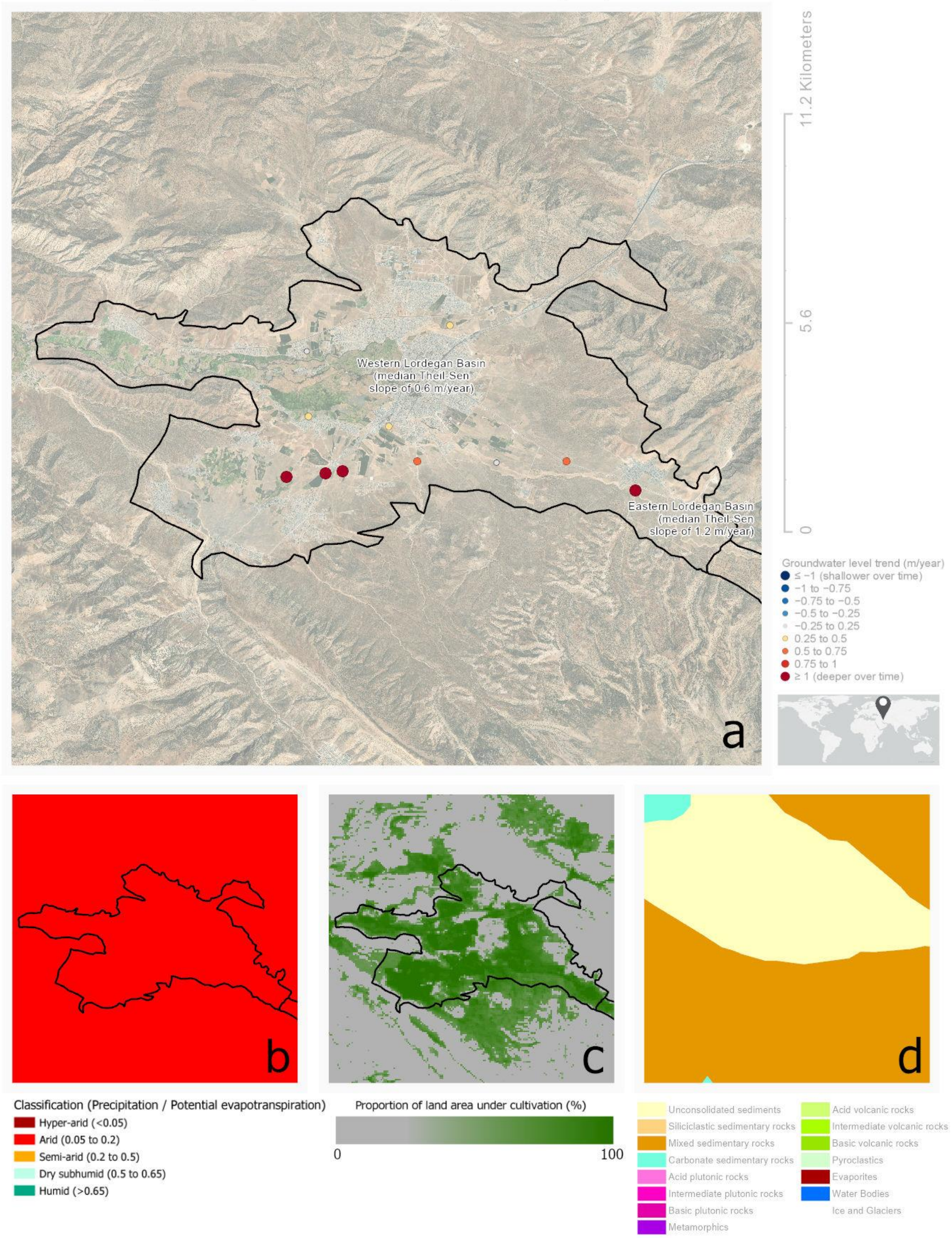

Supplementary Fig. 255. Spatial variability of trends in depth to groundwater Western Lordegan Basin (within the broader Lordegan Basin). For detailed description of the contents of each panel see the text at the beginning of Supplementary Note 17.

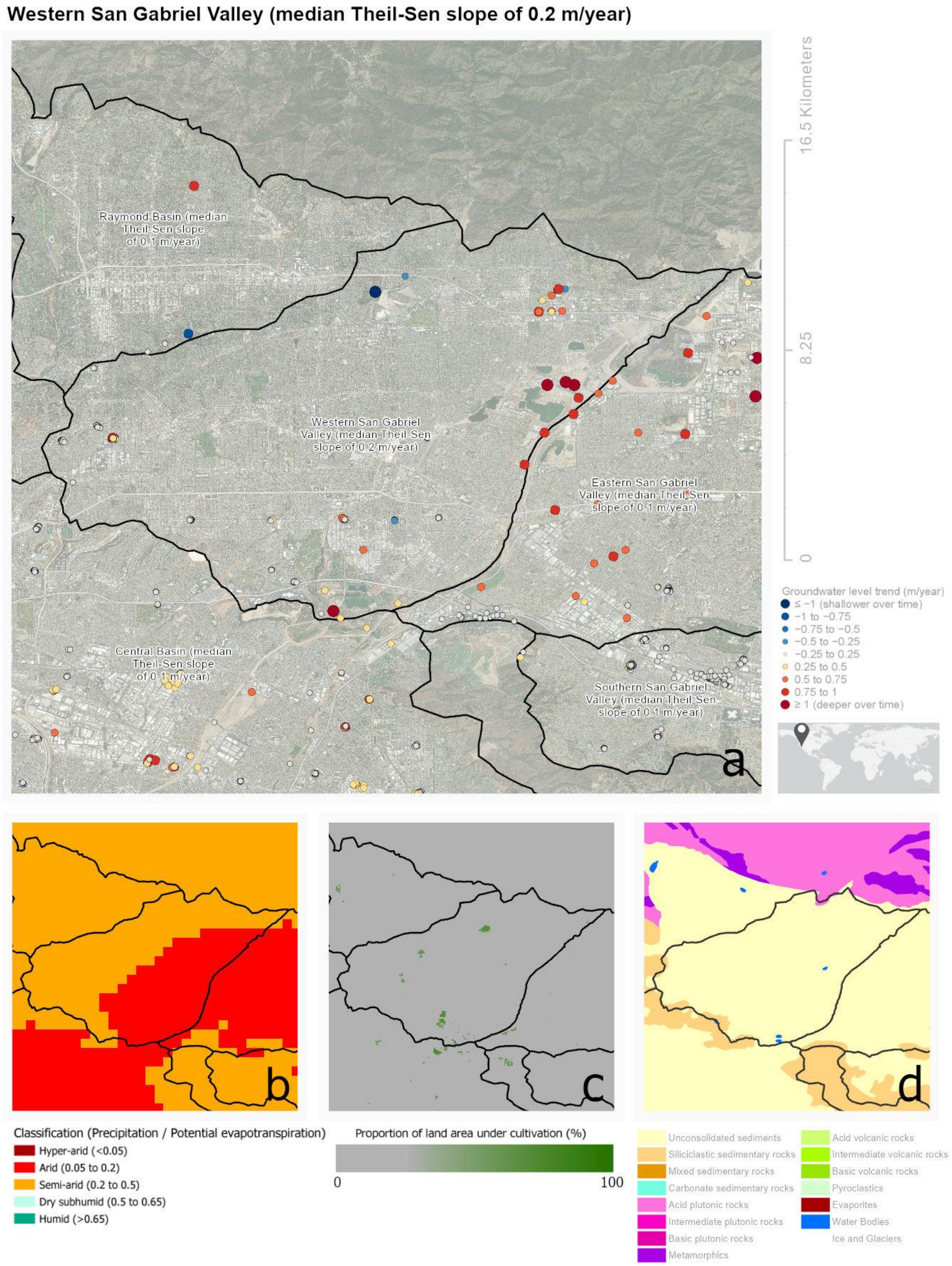

Supplementary Fig. 256. Spatial variability of trends in depth to groundwater Western San Gabriel Valley (within the broader San Gabriel Valley). For detailed description of the contents of each panel see the text at the beginning of Supplementary Note 17.

Western Sutter Basin (median Theil-Sen slope of 0 m/year)

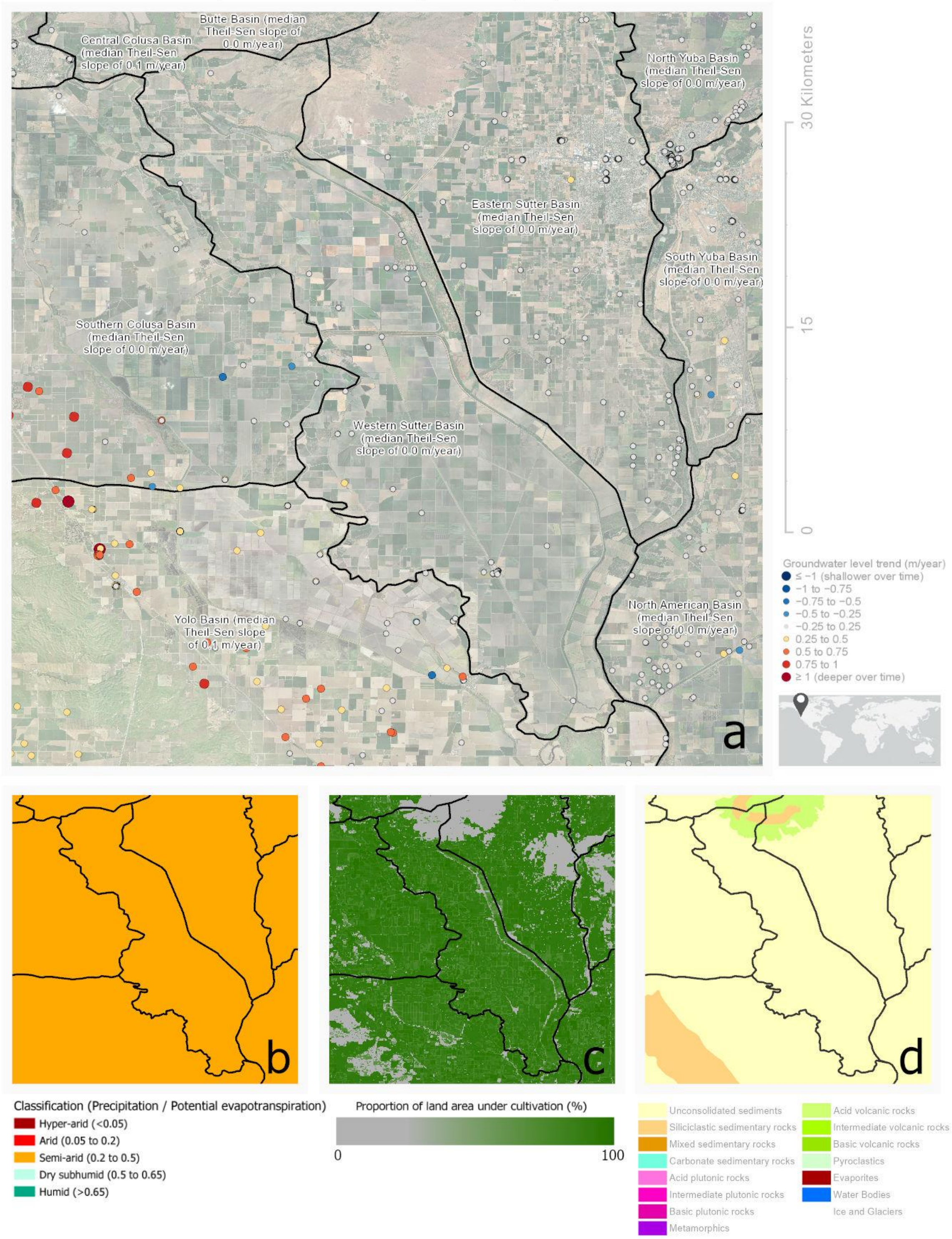

Supplementary Fig. 257. Spatial variability of trends in depth to groundwater Western Sutter Basin (within the broader Sacramento Valley of the Central Valley). For detailed description of the contents of each panel see the text at the beginning of Supplementary Note 17.

Western Villa Garcia Aquifer (median Theil-Sen slope of 0 m/year)

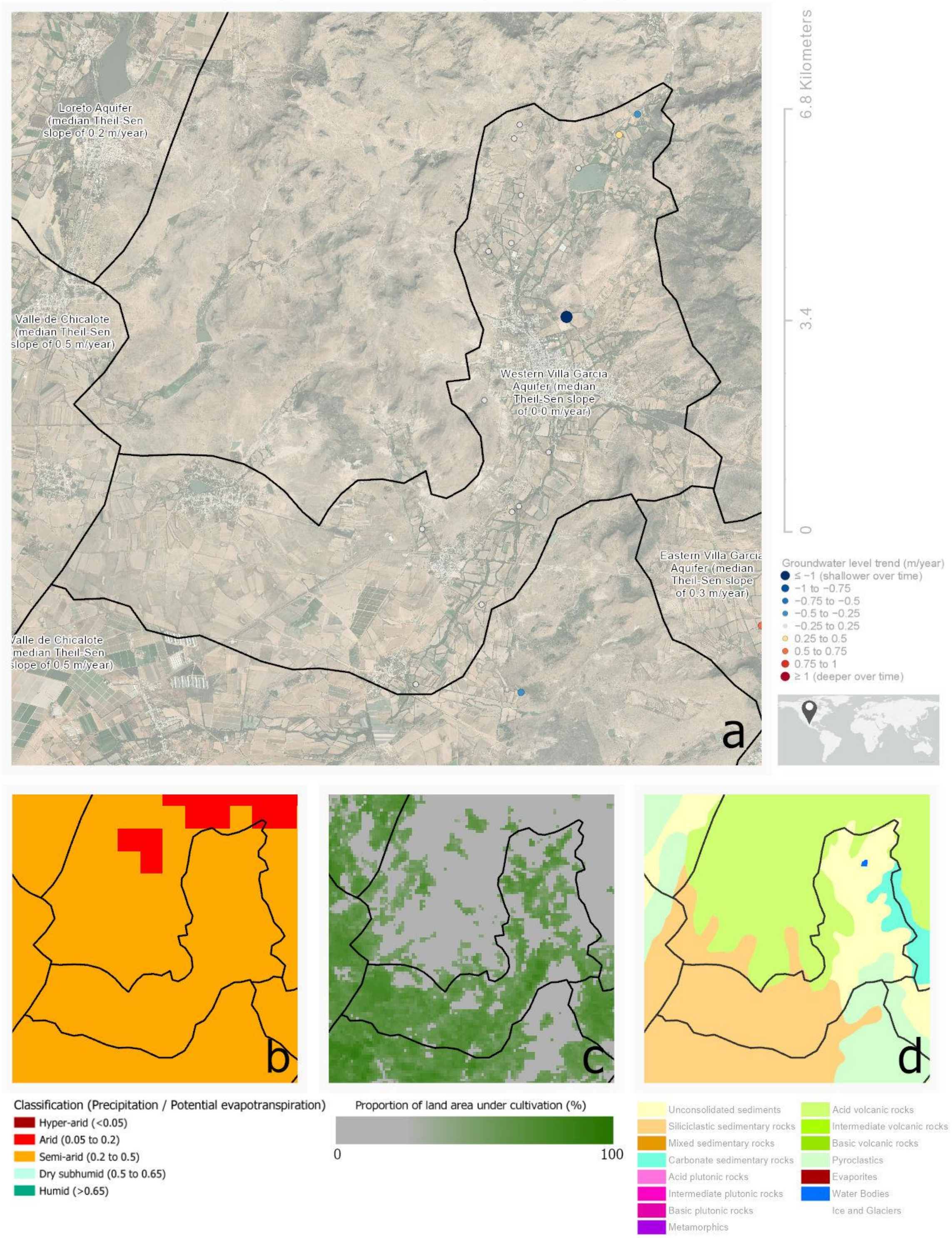

Supplementary Fig. 258. Spatial variability of trends in depth to groundwater Western Villa Garcia Aquifer. For detailed description of the contents of each panel see the text at the beginning of Supplementary Note 17.

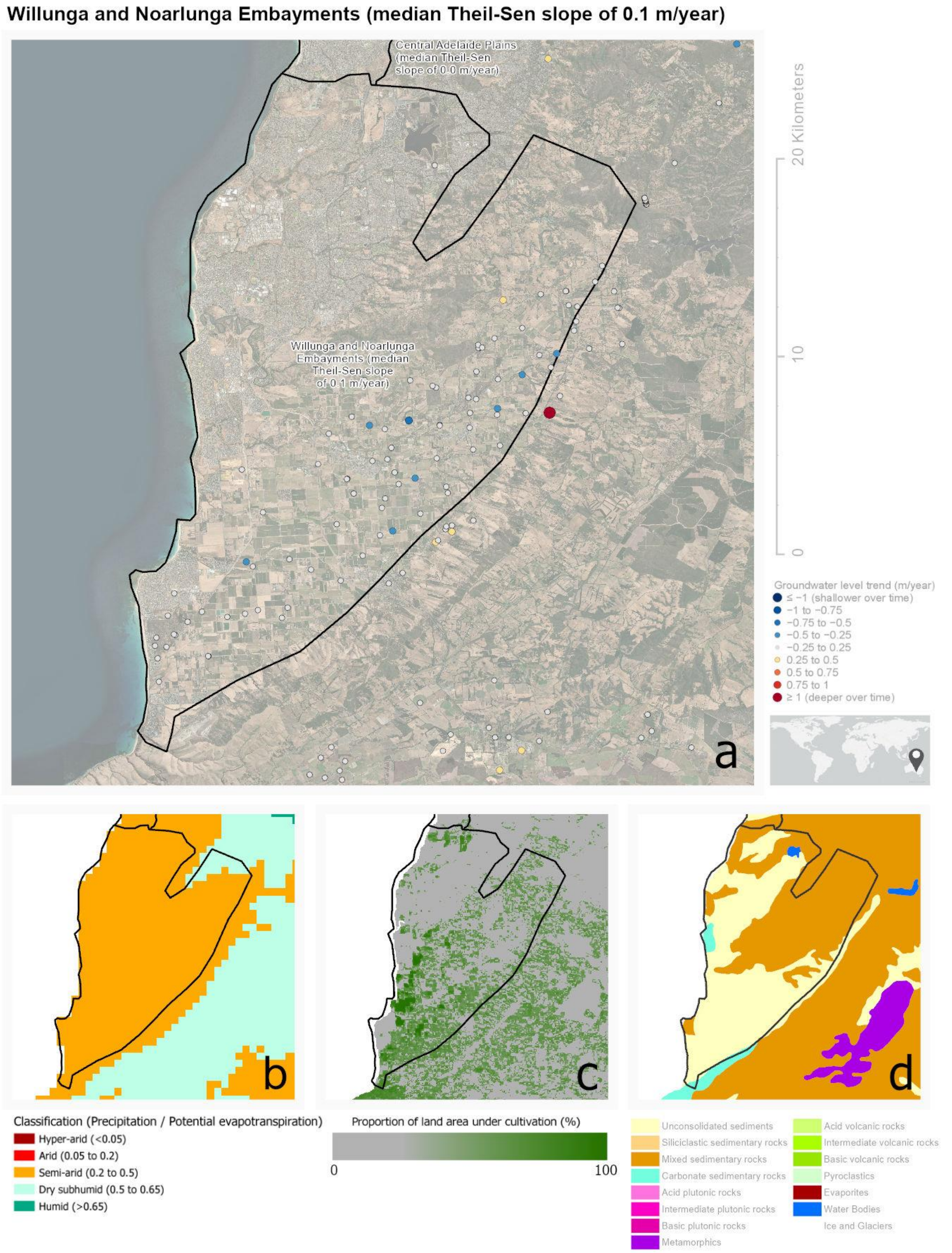

Supplementary Fig. 259. Spatial variability of trends in depth to groundwater Willunga and Noarlunga Embayments (within the broader St Vincent Basin). For detailed description of the contents of each panel see the text at the beginning of Supplementary Note 17.

Winkler Alluvial Aquifer (median Theil-Sen slope of 0 m/year)

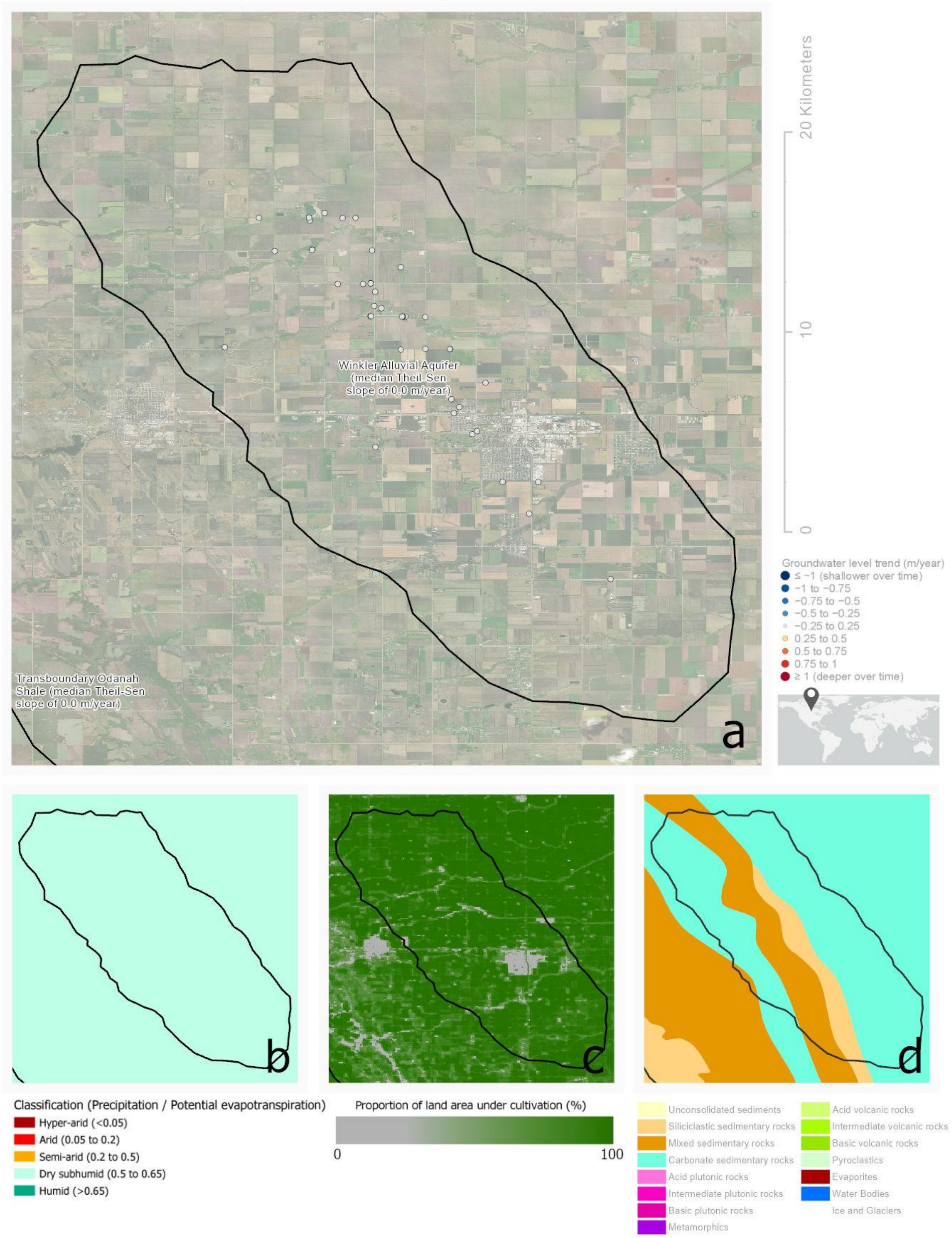

Supplementary Fig. 260. Spatial variability of trends in depth to groundwater Winkler Alluvial Aquifer. For detailed description of the contents of each panel see the text at the beginning of Supplementary Note 17.

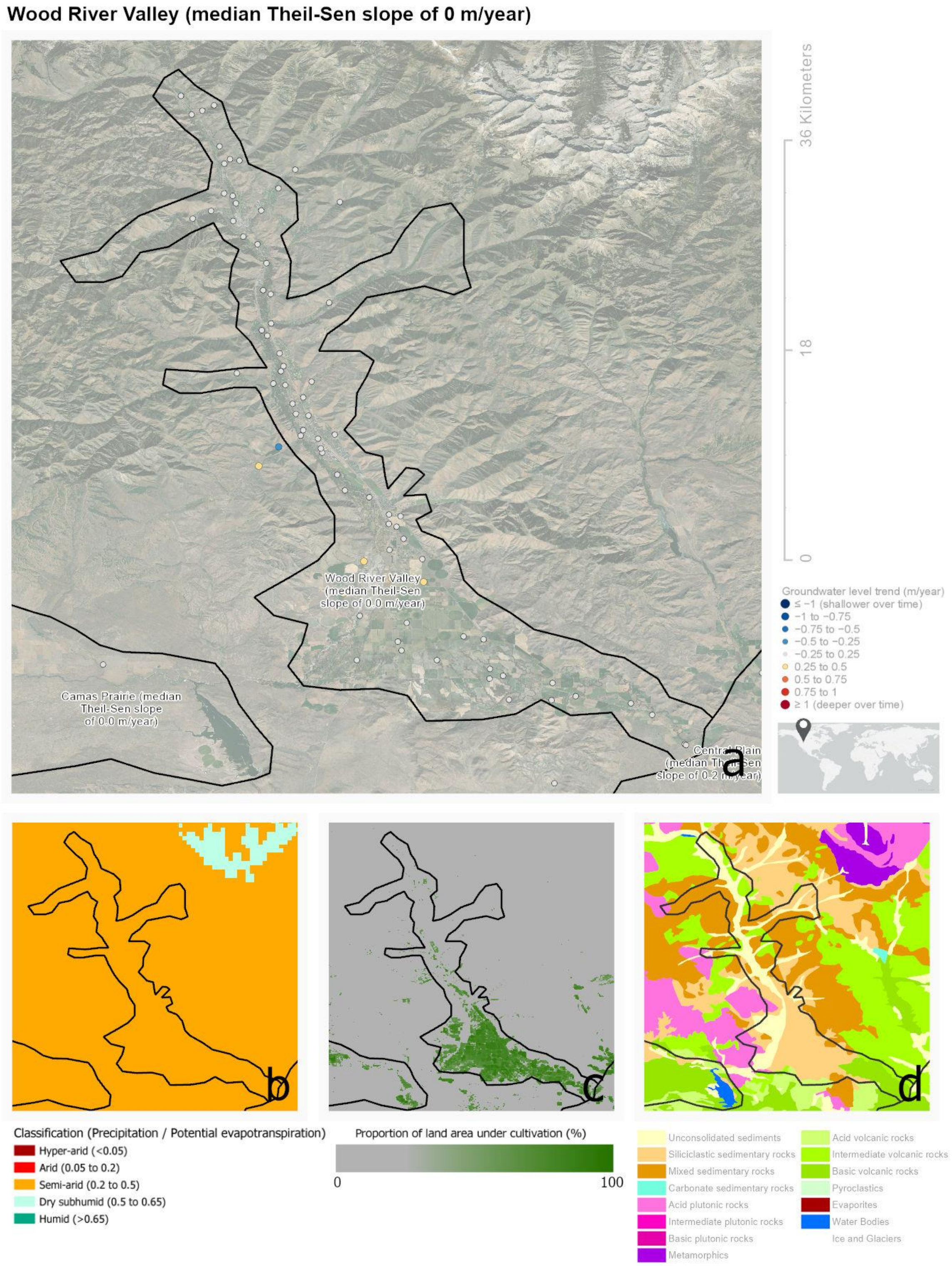

Supplementary Fig. 261. Spatial variability of trends in depth to groundwater Wood River Valley. For detailed description of the contents of each panel see the text at the beginning of Supplementary Note 17.

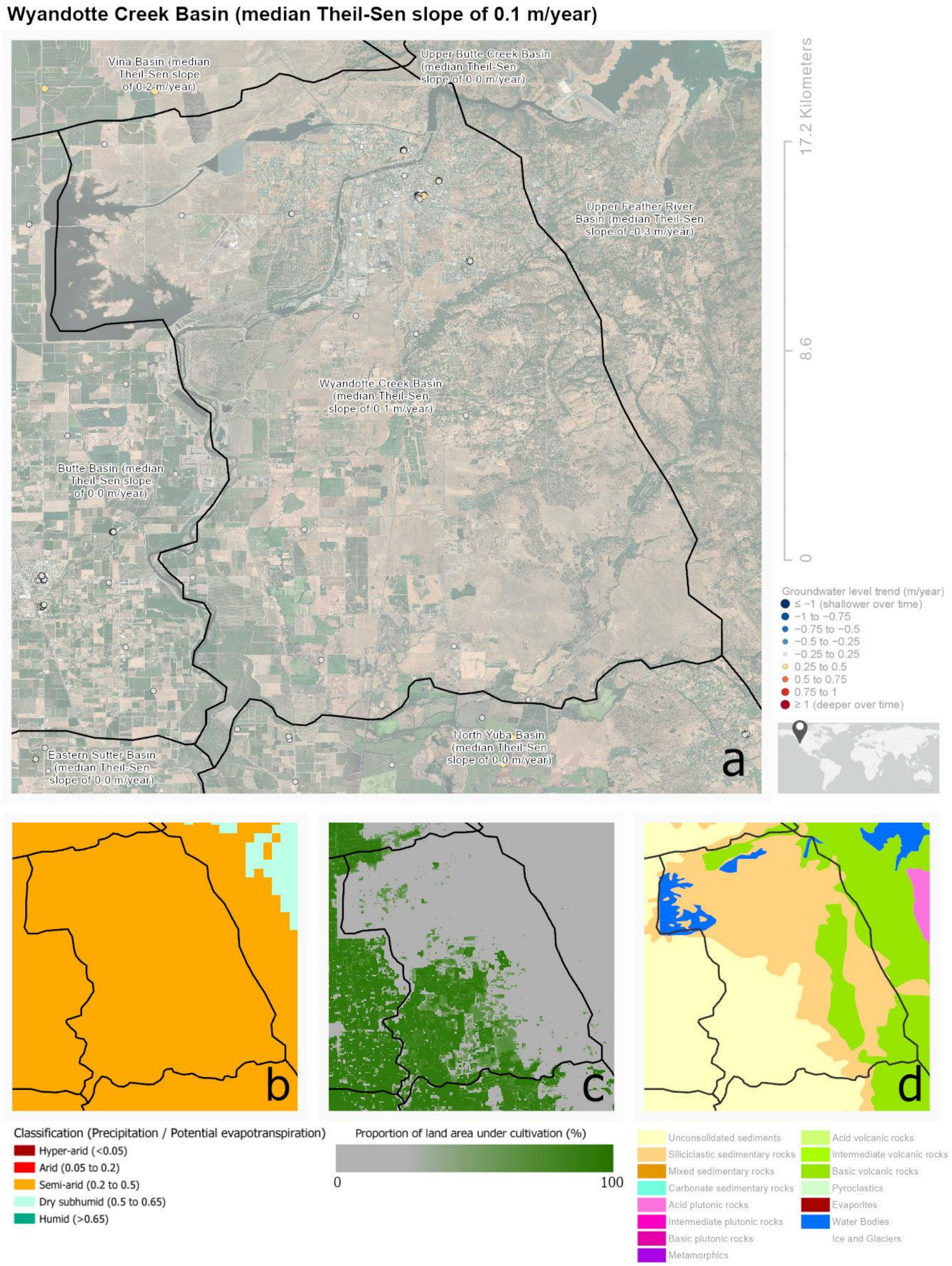

Supplementary Fig. 262. Spatial variability of trends in depth to groundwater Wyandotte Creek Basin (within the broader Sacramento Valley of the Central Valley). For detailed description of the contents of each panel see the text at the beginning of Supplementary Note 17.

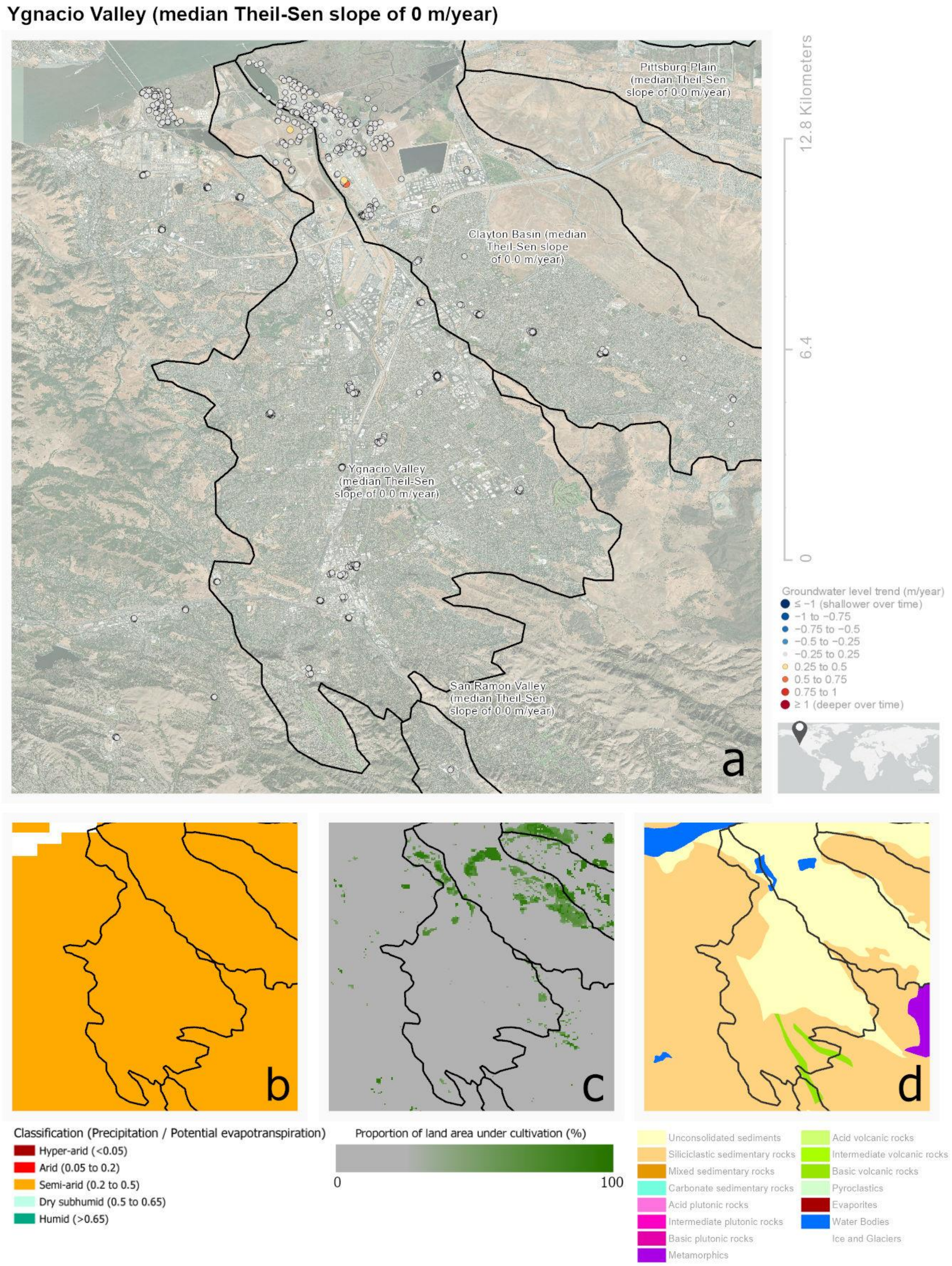

Supplementary Fig. 263. Spatial variability of trends in depth to groundwater Ygnacio Valley. For detailed description of the contents of each panel see the text at the beginning of Supplementary Note 17.

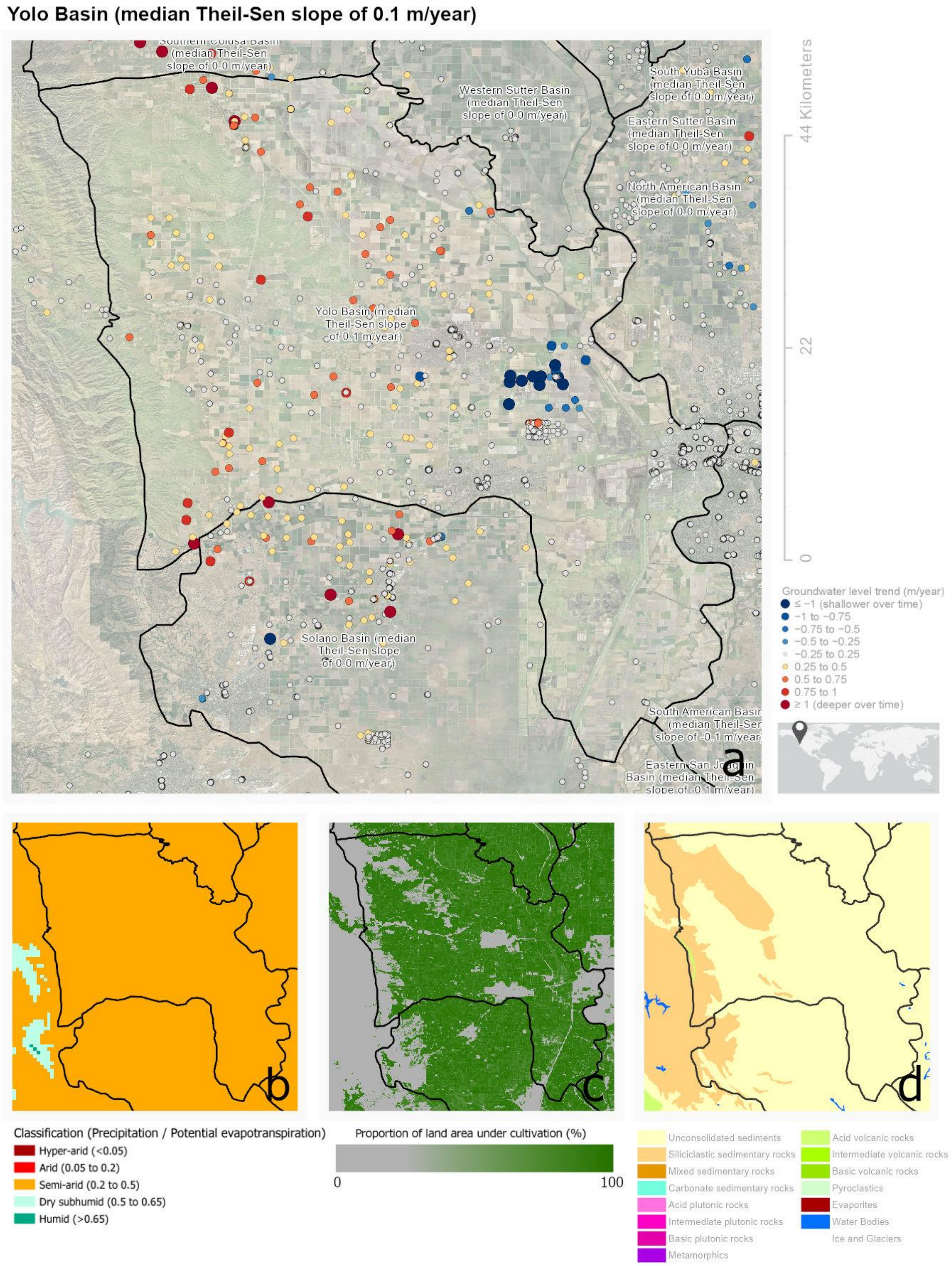

Supplementary Fig. 264. Spatial variability of trends in depth to groundwater Yolo Basin (within the broader Sacramento Valley of the Central Valley). For detailed description of the contents of each panel see the text at the beginning of Supplementary Note 17.

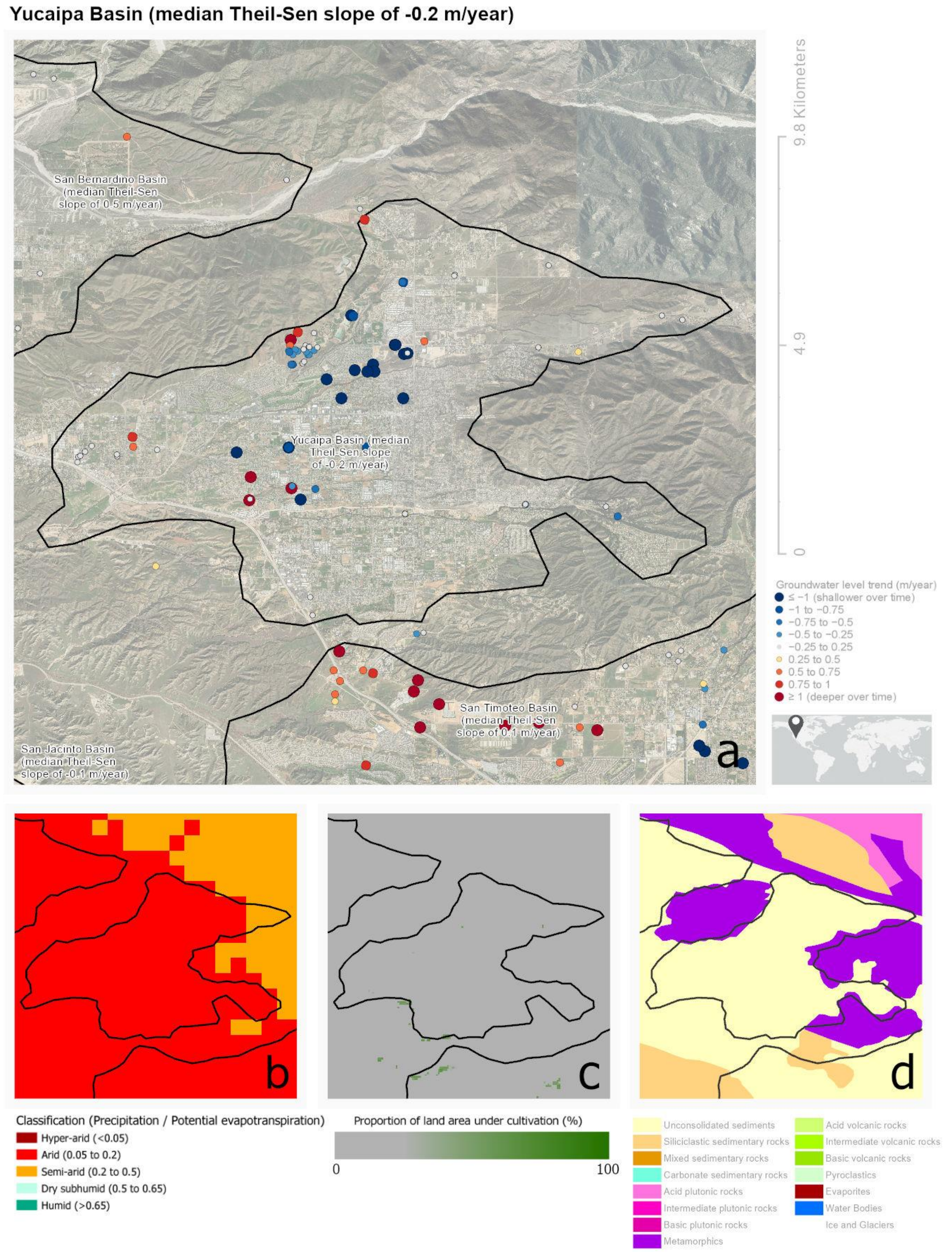

Supplementary Fig. 265. Spatial variability of trends in depth to groundwater Yucaipa Basin. For detailed description of the contents of each panel see the text at the beginning of Supplementary Note 17.

18 HOTSPOTS OF GROUNDWATER LEVEL DEEPENING WITHIN SOME AQUIFER SYSTEMS

Here we examine spatial variability in trends in depth to groundwater within the boundaries of individual aquifer systems.

Our compiled datasets enable us to examine these spatial patterns because the majority of our aquifer systems contain multiple monitoring wells with sufficient data for analysis. Specifically, the great majority (93%) of our aquifer systems have more than 1 monitoring well, most (81%) have 5 or more monitoring wells, and two-thirds (67%) have 10 or more monitoring wells (Supplementary Fig. 266).

Because some aquifer systems cover larger areas than others, a simple count of monitoring wells (i.e., Supplementary Fig. 266) is inadequate to compare monitoring well coverage across larger versus smaller aquifer systems. Therefore, we calculated the density of monitoring wells within each aquifer system (see figures on following pages). The great majority (87%) of our aquifer systems have more than 0.001 wells/km^2 (i.e., more than 1 well per 1,000 km^2). Further, more than half (59%) of our aquifer systems have more than 0.01 wells/km^2 (i.e., >1 well per 100 km^2; Supplementary Fig. 267).

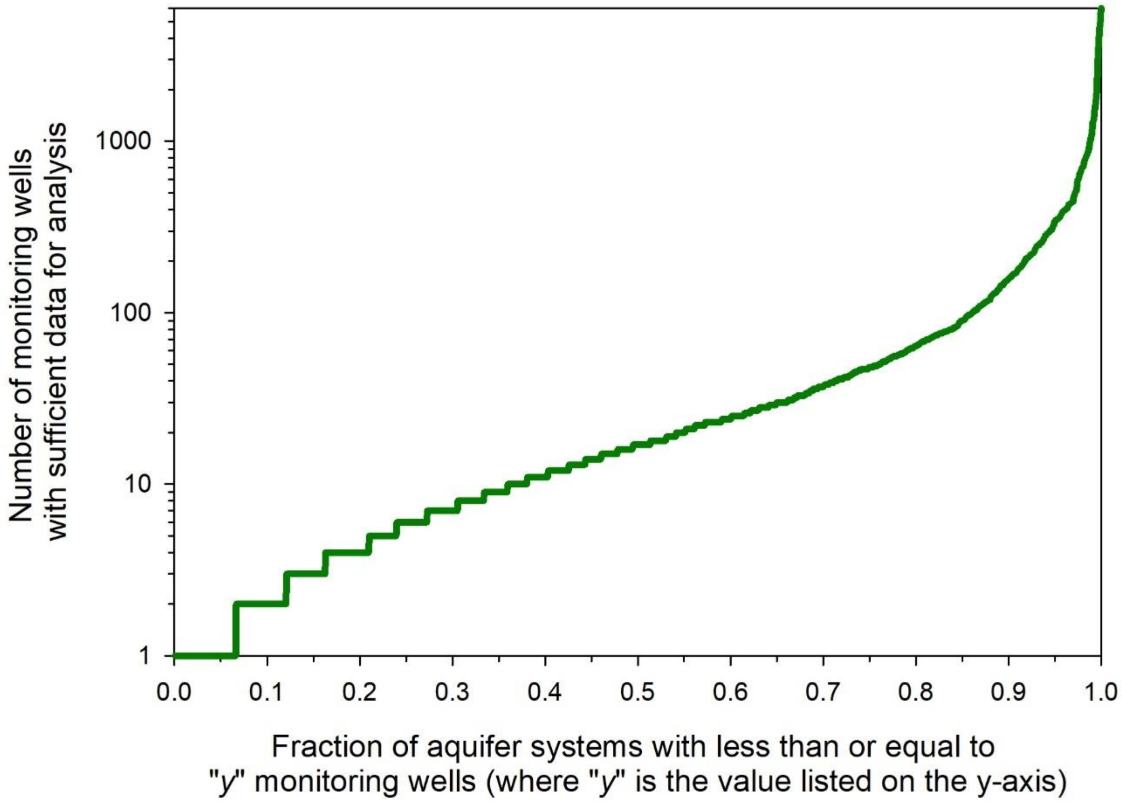

Supplementary Fig. 266. The number of unique monitoring wells within our aquifer systems that have sufficient data for analyses. The x-axis values represent the fraction of our aquifer systems (i.e., with "1.0" representing all n=1,693 aquifer systems) that have "y" monitoring wells with sufficient data for analyses (where "y" is the y-axis value). The majority of our aquifer systems have numerous unique monitoring wells with sufficient data for analysis. Specifically, 93% (i.e., 0.07 on x-axis) of our aquifer systems have more than 1 monitoring well, 79% of aquifer systems (n=1,338) have 5 or more monitoring wells, and 64% of aquifer systems (n=1,085) have 10 or more monitoring wells.

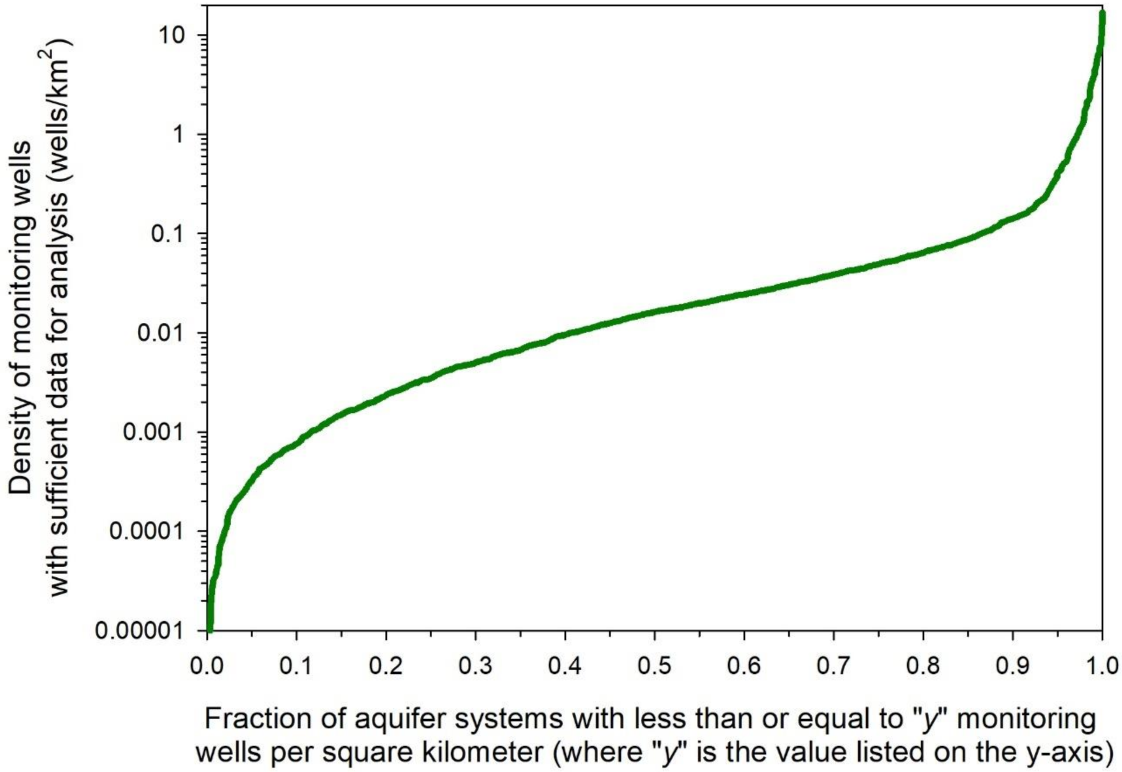

Supplementary Fig. 267. The density (wells/km^2) of unique monitoring wells within our aquifer systems that have sufficient data for analyses. The x-axis values represent the fraction of our aquifer systems (i.e., with "1.0" representing all n=1,693 aquifer systems) that have a density of "y" monitoring wells with sufficient data for analyses (where "y" is the y-axis value). The majority of our aquifer systems have a relatively high density of monitoring wells with sufficient data for analysis. Specifically, 88% (i.e., 0.12 on x-axis) of our aquifer systems have more than 0.001 wells/km^2 (i.e., more than 1 well per 1,000 km^2), and 59% of our aquifer systems (n=688) have more than 0.01 wells/km^2 (i.e., more than 1 well per 100 km^2).

18.1 PROPORTION OF MONITORING WELLS WITHIN A STUDY AREA EXHIBITING RELATIVELY RAPID RATES OF GROUNDWATER LEVEL DEEPENING

We report the proportion of wells within an aquifer system where depths to groundwater levels became deeper over time faster than 0.5 m/year (see map below). In 34% (n=579) of our 1,693 aquifer systems, groundwater levels became deeper over time faster than 0.5 m/year in at least one-in-ten monitoring wells (see following map), evidencing that at least some monitoring wells have recorded rapid groundwater level deepening in more than one-third of our studied aquifer systems.

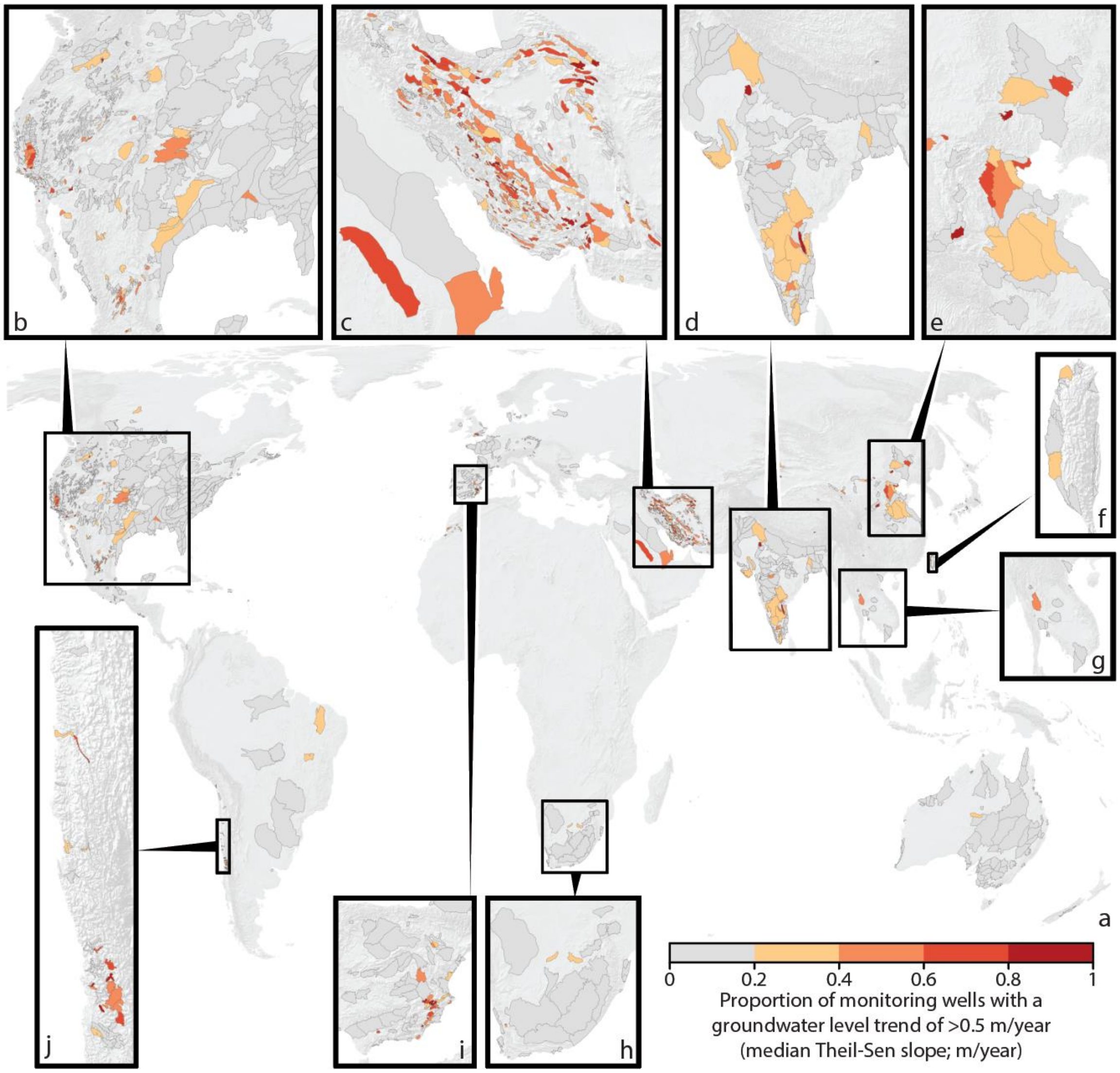

Supplementary Fig. 268. Proportion of monitoring wells within each of our 1,693 aquifer systems with a 21st-century trend in depth to groundwater (Theil-Sen slope) exceeding 0.5 m/year (i.e., well water levels deepening over time). Deeper shades of purple represent aquifer systems where the majority of monitoring wells exhibit relatively rapid rates of groundwater level deepening over time. **a)** Results presented at global scale. **b-i)** Zoomed-in maps for various regions around the globe.

18.2 HOTSPOTS OF RELATIVELY RAPID GROUNDWATER LEVEL DEEPENING

To further highlight the substantial spatial heterogeneity of typical groundwater level trends, we report median groundwater level trends (Theil-Sen slopes) for 1,000 km^2 areas (i.e., the median Theil-Sen slope for all monitoring wells within each 1,000 km^2 area; see figures below).

This analysis demonstrates that depths to groundwater deepened faster than 0.5 m/year in small areas covering only portions of some of our 1,693 aquifer systems, including some where the aquifer-wide median trend in depth to groundwater is nearly zero. For example, median depth to groundwater trends exceeds 1 m/year (deeper over time) in the southeast and northern parts of the Trinity Aquifer System (east-central Texas), despite the median groundwater level trend among all monitoring wells within this aquifer system being just 0.1 m/year (see Supplementary Fig. 269).

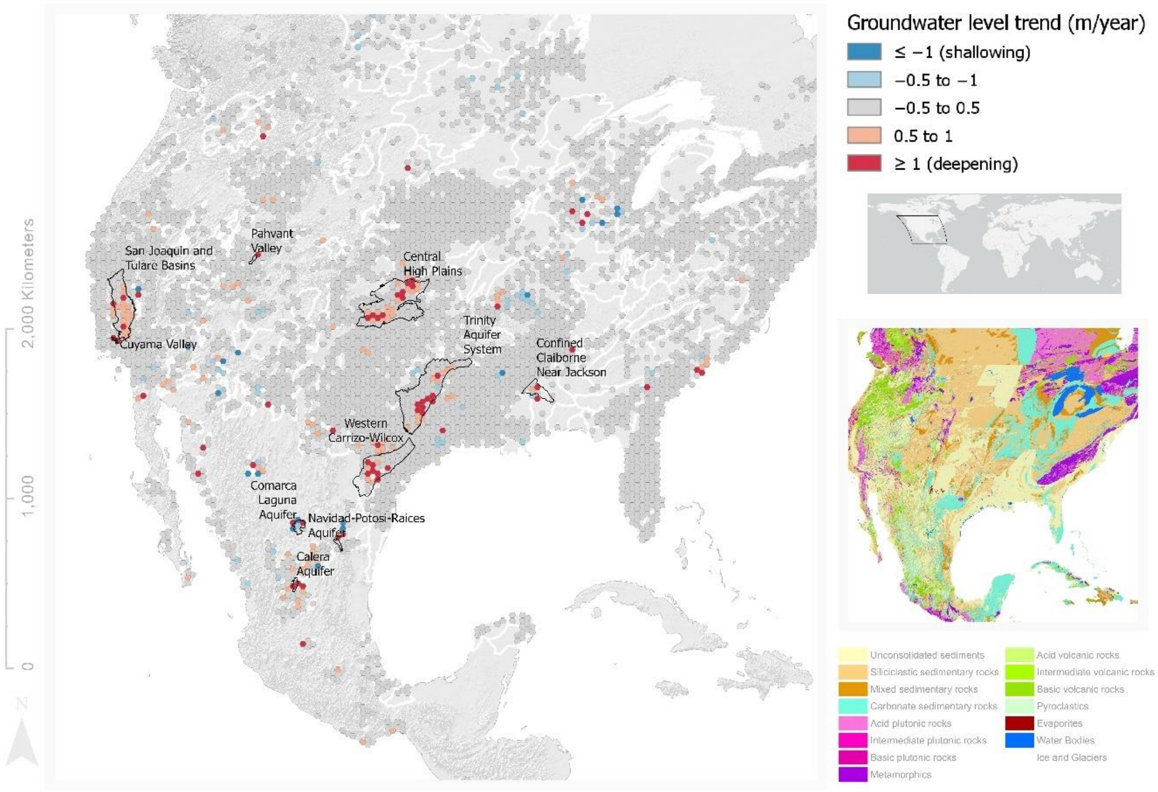

Supplementary Fig. 269. 21st-century trends in depth to groundwater in 1,000 km² areas across North America. Each hexagonal area represents one 1,000 km² area. The colour of the hexagon corresponds to the median Theil-Sen slope among all monitoring wells within the 1,000 km² area. Red and orange hexagons represent areas where the aquifer-scale trend in depth to groundwater exceeds 0.5 m/year (i.e., groundwater levels became deeper over time faster than 0.5 m/year). Blue hexagons represent areas where the aquifer-scale trend in depth to groundwater is less than -0.5 m/year (i.e., groundwater levels becoming shallower over time faster than -0.5 m/year; see legend in upper-right corner of figure). The grey map (right side of figure) shows the area covered by the main map (see dark grey polygon on global map). The map in the lower-right corner of the figure shows spatial variations in near-surface geology across the area depicted in the main map (geologic data from ref.[1360]: Hartmann, J., & Moosdorf, N. The new global lithological map database GLiM: A representation of rock properties at the Earth surface. *Geochemistry, Geophysics, Geosystems* **13,** doi.org/10.1029/2012GC004370 (2012)). Black polylines with labels outline some of our delineated aquifer systems. White polylines outline other aquifer systems (i.e., aquifer systems that are not specifically labelled in this figure).

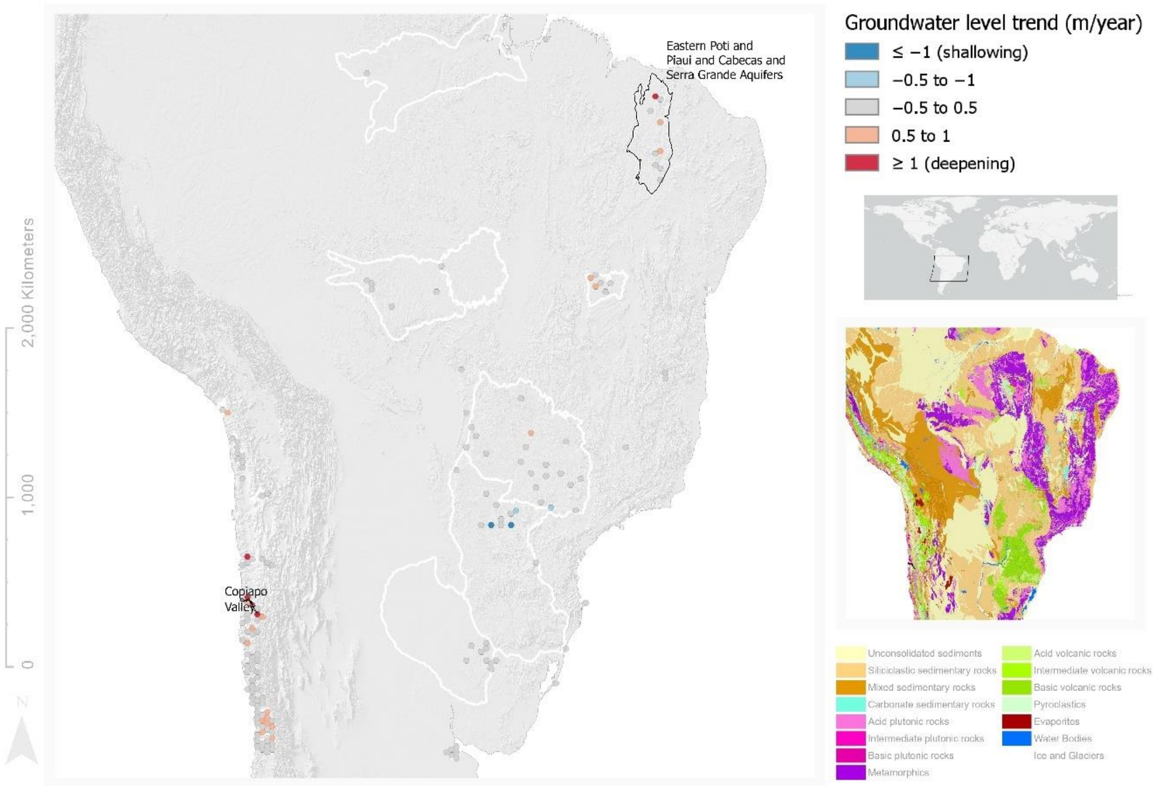

Supplementary Fig. 270. 21st-century trends in depth to groundwater in 1,000 km² areas across South America. Each hexagonal area represents one 1,000 km² area. The colour of the hexagon corresponds to the median Theil-Sen slope among all monitoring wells within the 1,000 km² area. Red and orange hexagons represent areas where the aquifer-scale trend in depth to groundwater exceeds 0.5 m/year (i.e., groundwater levels became deeper over time faster than 0.5 m/year). Blue hexagons represent areas where the aquifer-scale trend in depth to groundwater is less than -0.5 m/year (i.e., groundwater levels becoming shallower over time faster than -0.5 m/year; see legend in upper-right corner of figure). The grey map (right side of figure) shows the area covered by the main map (see dark grey polygon on global map). The map in the lower-right corner of the figure shows spatial variations in near-surface geology across the area depicted in the main map (geologic data from ref.[1360]: Hartmann, J., & Moosdorf, N. The new global lithological map database GLiM: A representation of rock properties at the Earth surface. *Geochemistry, Geophysics, Geosystems* **13,** doi.org/10.1029/2012GC004370 (2012)). Black polylines with labels outline some of our delineated aquifer systems. White polylines outline other aquifer systems (i.e., aquifer systems that are not specifically labelled in this figure).

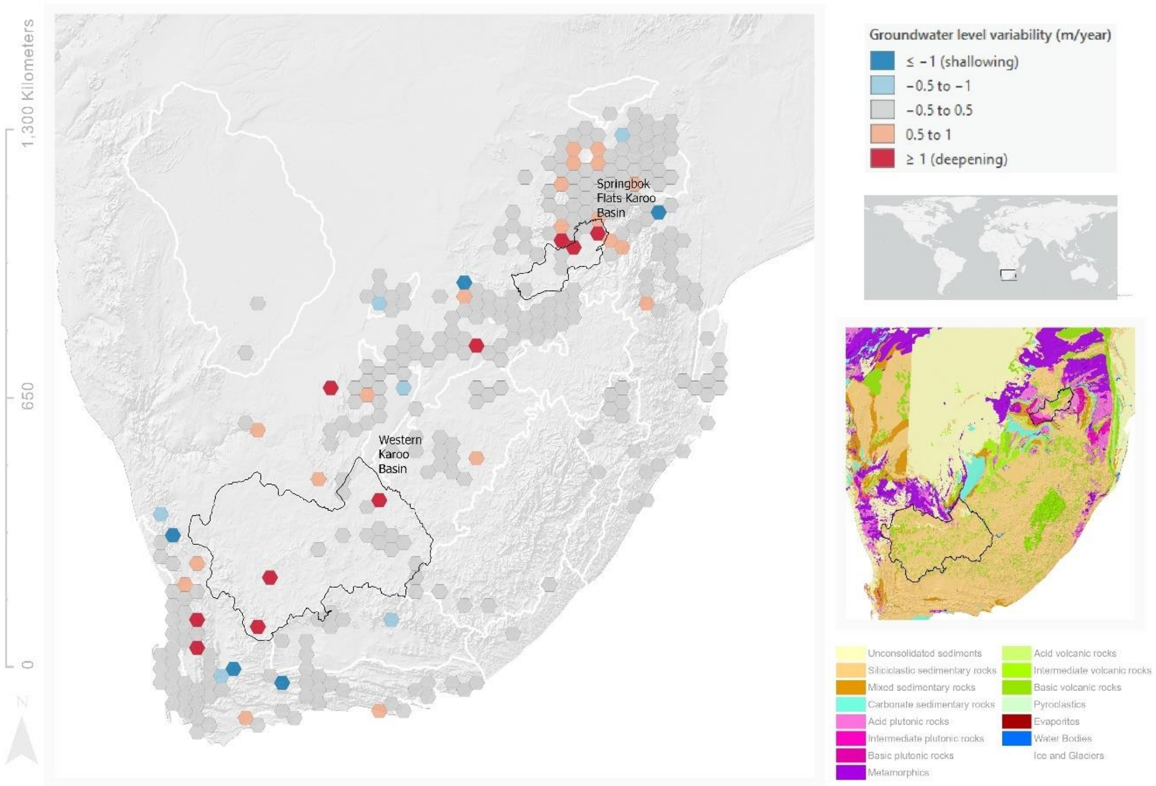

Supplementary Fig. 271. 21st-century trends in depth to groundwater in 1,000 km² areas across the southern portion of Africa. Each hexagonal area represents one 1,000 km² area. The colour of the hexagon corresponds to the median Theil-Sen slope among all monitoring wells within the 1,000 km² area. Red and orange hexagons represent areas where the aquifer-scale trend in depth to groundwater exceeds 0.5 m/year (i.e., groundwater levels became deeper over time faster than 0.5 m/year). Blue hexagons represent areas where the aquifer-scale trend in depth to groundwater is less than -0.5 m/year (i.e., groundwater levels becoming shallower over time faster than -0.5 m/year; see legend in upper-right corner of figure). The grey map (right side of figure) shows the area covered by the main map (see dark grey polygon on global map). The map in the lower-right corner of the figure shows spatial variations in near-surface geology across the area depicted in the main map (geologic data from ref.[1360]: Hartmann, J., & Moosdorf, N. The new global lithological map database GLiM: A representation of rock properties at the Earth surface. *Geochemistry, Geophysics, Geosystems* **13,** doi.org/10.1029/2012GC004370 (2012)). Black polylines with labels outline some of our delineated aquifer systems. White polylines outline other aquifer systems (i.e., aquifer systems that are not specifically labelled in this figure).

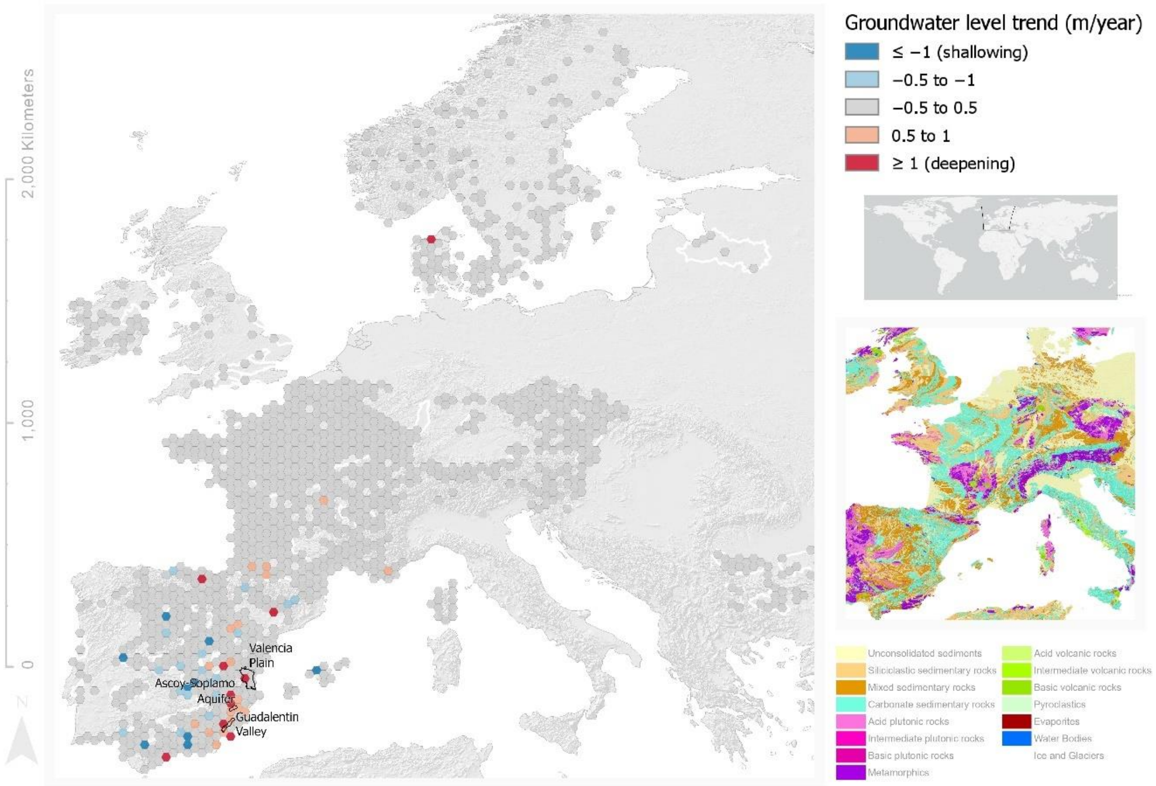

Supplementary Fig. 272. 21st-century trends in depth to groundwater in 1,000 km^2 areas across Europe. Each hexagonal area represents one 1,000 km^2 area. The colour of the hexagon corresponds to the median Theil-Sen slope among all monitoring wells within the 1,000 km^2 area. Red and orange hexagons represent areas where the aquifer-scale trend in depth to groundwater exceeds 0.5 m/year (i.e., groundwater levels became deeper over time faster than 0.5 m/year). Blue hexagons represent areas where the aquifer-scale trend in depth to groundwater is less than -0.5 m/year (i.e., groundwater levels becoming shallower over time faster than -0.5 m/year; see legend in upper-right corner of figure). The grey map (right side of figure) shows the area covered by the main map (see dark grey polygon on global map). The map in the lower-right corner of the figure shows spatial variations in near-surface geology across the area depicted in the main map (geologic data from ref.[1360]: Hartmann, J., & Moosdorf, N. The new global lithological map database GLiM: A representation of rock properties at the Earth surface. *Geochemistry, Geophysics, Geosystems* **13,** doi.org/10.1029/2012GC004370 (2012)). Black polylines with labels outline some of our delineated aquifer systems. White polylines outline other aquifer systems (i.e., aquifer systems that are not specifically labelled in this figure). Groundwater level shallowing (blue areas) are evident in several areas in central and south-central Spain, whereas groundwater level deepening is evident in some areas in southeastern Spain (red areas).

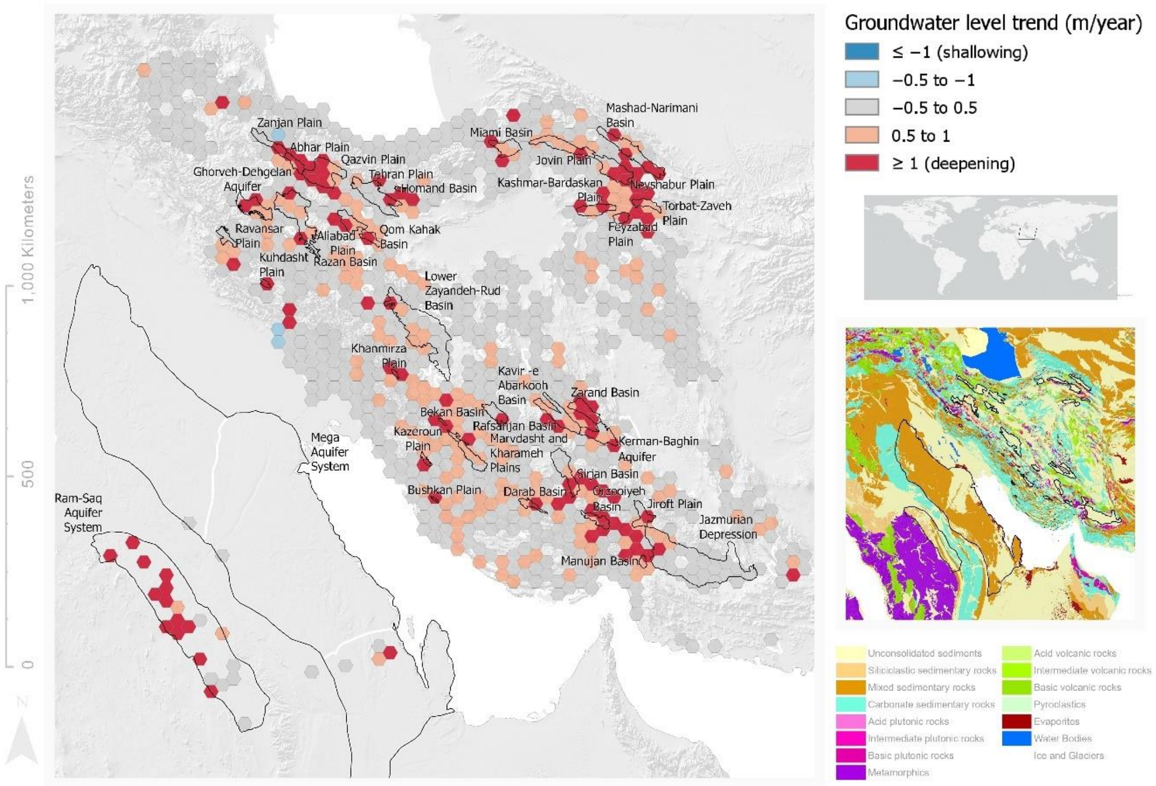

Supplementary Fig. 273. 21st-century trends in depth to groundwater in 1,000 km² areas in eastern Saudi Arabia and Iran. Each hexagonal area represents one 1,000 km² area. The colour of the hexagon corresponds to the median Theil-Sen slope among all monitoring wells within the 1,000 km² area. Red and orange hexagons represent areas where the aquifer-scale trend in depth to groundwater exceeds 0.5 m/year (i.e., groundwater levels became deeper over time faster than 0.5 m/year). Blue hexagons represent areas where the aquifer-scale trend in depth to groundwater is less than -0.5 m/year (i.e., groundwater levels becoming shallower over time faster than -0.5 m/year; see legend in upper-right corner of figure). The grey map (right side of figure) shows the area covered by the main map (see dark grey polygon on global map). The map in the lower-right corner of the figure shows spatial variations in near-surface geology across the area depicted in the main map (geologic data from ref.[1360]: Hartmann, J., & Moosdorf, N. The new global lithological map database GLiM: A representation of rock properties at the Earth surface. *Geochemistry, Geophysics, Geosystems* **13,** doi.org/10.1029/2012GC004370 (2012)). Black polylines with labels outline some of our delineated aquifer systems. White polylines outline other aquifer systems (i.e., aquifer systems that are not specifically labelled in this figure).

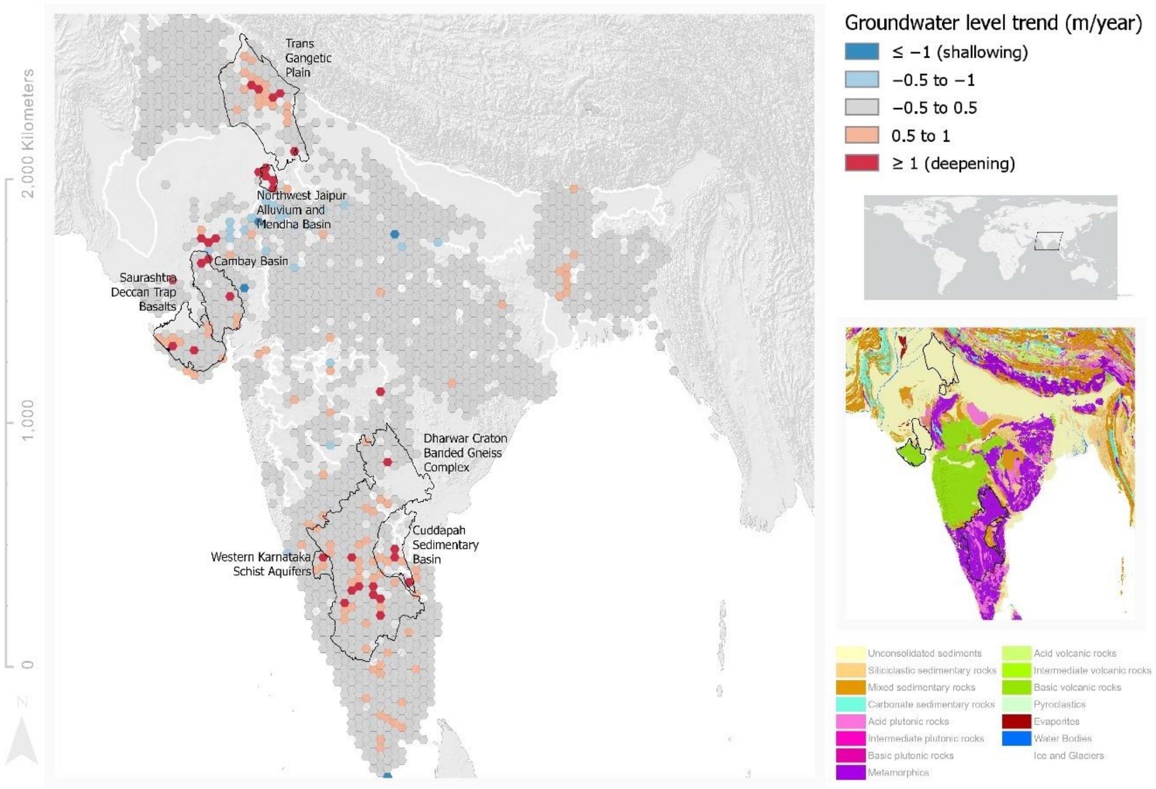

Supplementary Fig. 274. 21st-century trends in depth to groundwater in 1,000 km^2 areas across southern Asia. Each hexagonal area represents one 1,000 km^2 area. The colour of the hexagon corresponds to the median Theil-Sen slope among all monitoring wells within the 1,000 km^2 area. Red and orange hexagons represent areas where the aquifer-scale trend in depth to groundwater exceeds 0.5 m/year (i.e., groundwater levels became deeper over time faster than 0.5 m/year). Blue hexagons represent areas where the aquifer-scale trend in depth to groundwater is less than -0.5 m/year (i.e., groundwater levels becoming shallower over time faster than -0.5 m/year; see legend in upper-right corner of figure). The grey map (right side of figure) shows the area covered by the main map (see dark grey polygon on global map). The map in the lower-right corner of the figure shows spatial variations in near-surface geology across the area depicted in the main map (geologic data from ref.[1360]: Hartmann, J., & Moosdorf, N. The new global lithological map database GLiM: A representation of rock properties at the Earth surface. *Geochemistry, Geophysics, Geosystems* **13,** doi.org/10.1029/2012GC004370 (2012)). Black polylines with labels outline some of our delineated aquifer systems. White polylines outline other aquifer systems (i.e., aquifer systems that are not specifically labelled in this figure).

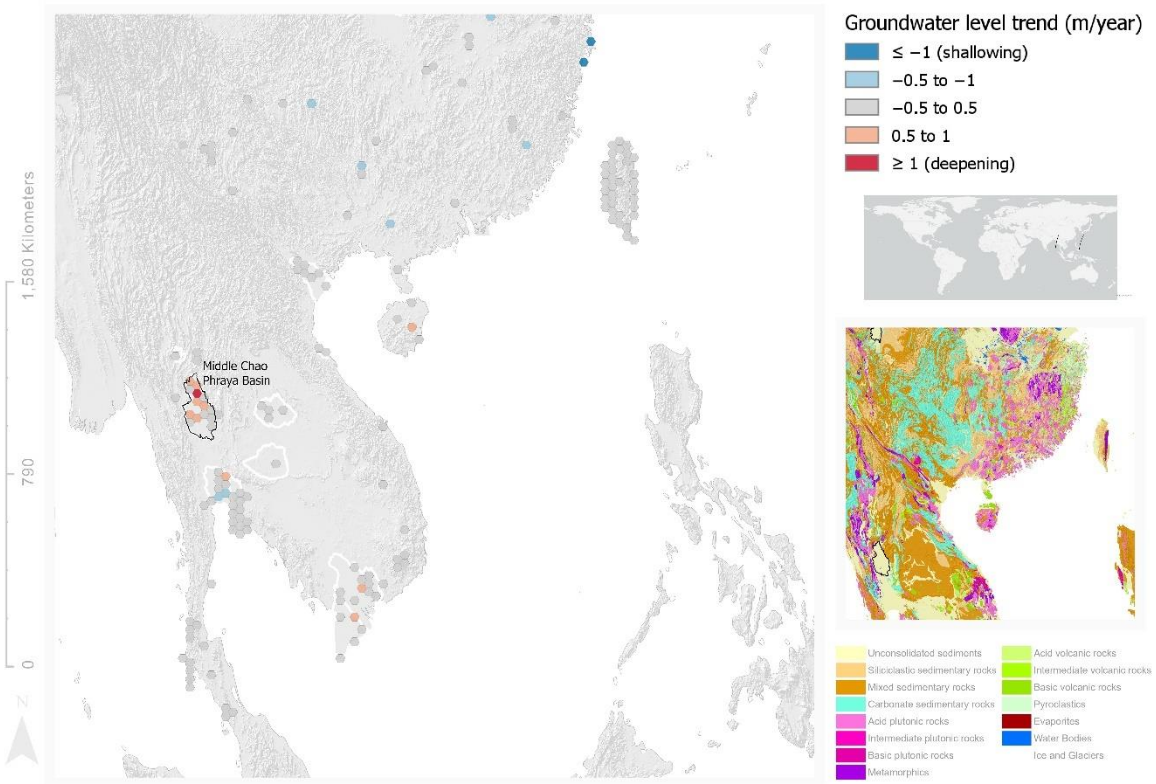

Supplementary Fig. 275. 21st-century trends in depth to groundwater in 1,000 km² areas across southeastern Asia. Each hexagonal area represents one 1,000 km² area. The colour of the hexagon corresponds to the median Theil-Sen slope among all monitoring wells within the 1,000 km² area (i.e., the median Theil-Sen slope among all monitoring wells within the 1,000 km² area). Red and orange hexagons represent areas where the aquifer-scale trend in depth to groundwater exceeds 0.5 m/year (i.e., groundwater levels became deeper over time faster than 0.5 m/year). Blue hexagons represent areas where the aquifer-scale trend in depth to groundwater is less than -0.5 m/year (i.e., groundwater levels becoming shallower over time faster than -0.5 m/year; see legend in upper-right corner of figure). The grey map (right side of figure) shows the area covered by the main map (see dark grey polygon on global map). The map in the lower-right corner of the figure shows spatial variations in near-surface geology across the area depicted in the main map (geologic data from ref.[1360]: Hartmann, J., & Moosdorf, N. The new global lithological map database GLiM: A representation of rock properties at the Earth surface. *Geochemistry, Geophysics, Geosystems* **13,** doi.org/10.1029/2012GC004370 (2012)). Black polylines with labels outline some of our delineated aquifer systems. White polylines outline other aquifer systems (i.e., aquifer systems that are not specifically labelled in this figure). Groundwater level shallowing trends are found in several regions in southern China (blue areas near, for example, the Oujiang River delta) and southern Bangkok (Thailand).

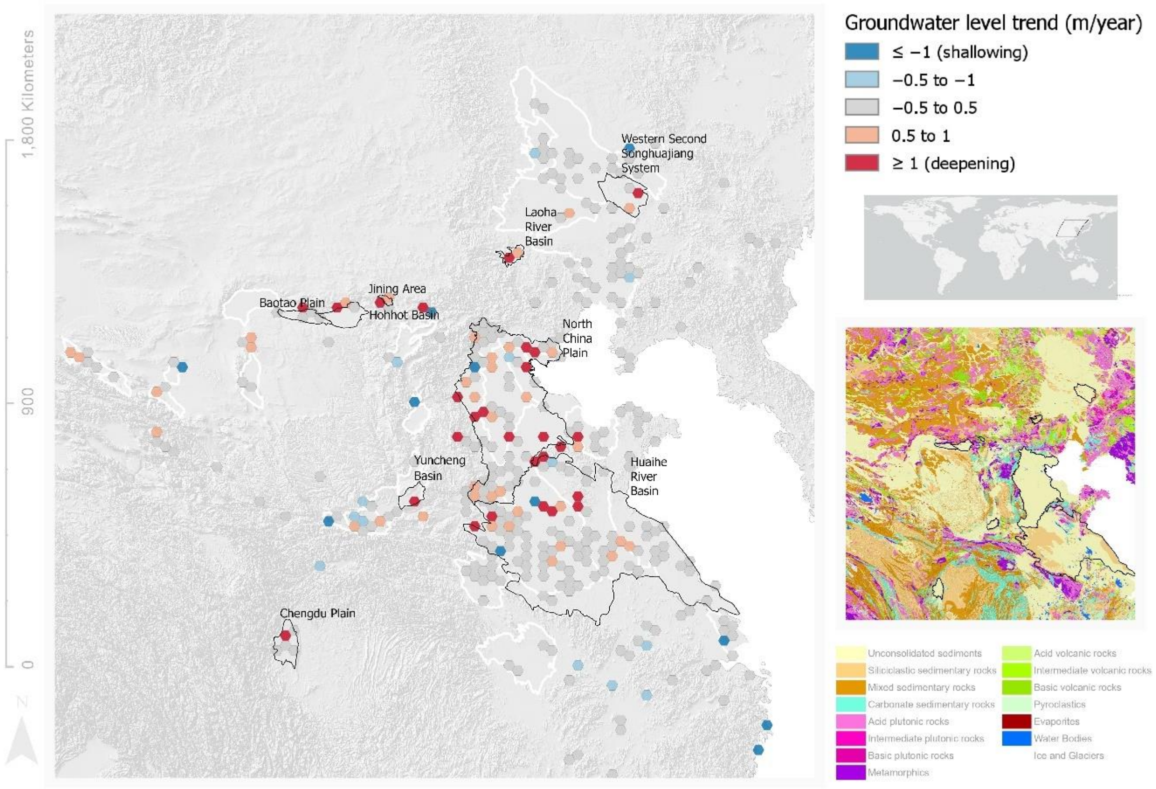

Supplementary Fig. 276. 21st-century trends in depth to groundwater in 1,000 km² areas across eastern China. Each hexagonal area represents one 1,000 km² area. The colour of the hexagon corresponds to the median Theil-Sen slope among all monitoring wells within the 1,000 km² area. Red and orange hexagons represent areas where the aquifer-scale trend in depth to groundwater exceeds 0.5 m/year (i.e., groundwater levels became deeper over time faster than 0.5 m/year). Blue hexagons represent areas where the aquifer-scale trend in depth to groundwater is less than -0.5 m/year (i.e., groundwater levels becoming shallower over time faster than -0.5 m/year; see legend in upper-right corner of figure). The grey map (right side of figure) shows the area covered by the main map (see dark grey polygon on global map). The map in the lower-right corner of the figure shows spatial variations in near-surface geology across the area depicted in the main map (geologic data from ref.[1360]: Hartmann, J., & Moosdorf, N. The new global lithological map database GLiM: A representation of rock properties at the Earth surface. *Geochemistry, Geophysics, Geosystems* **13,** doi.org/10.1029/2012GC004370 (2012)). Black polylines with labels outline some of our delineated aquifer systems. White polylines outline other aquifer systems (i.e., aquifer systems that are not specifically labelled in this figure).

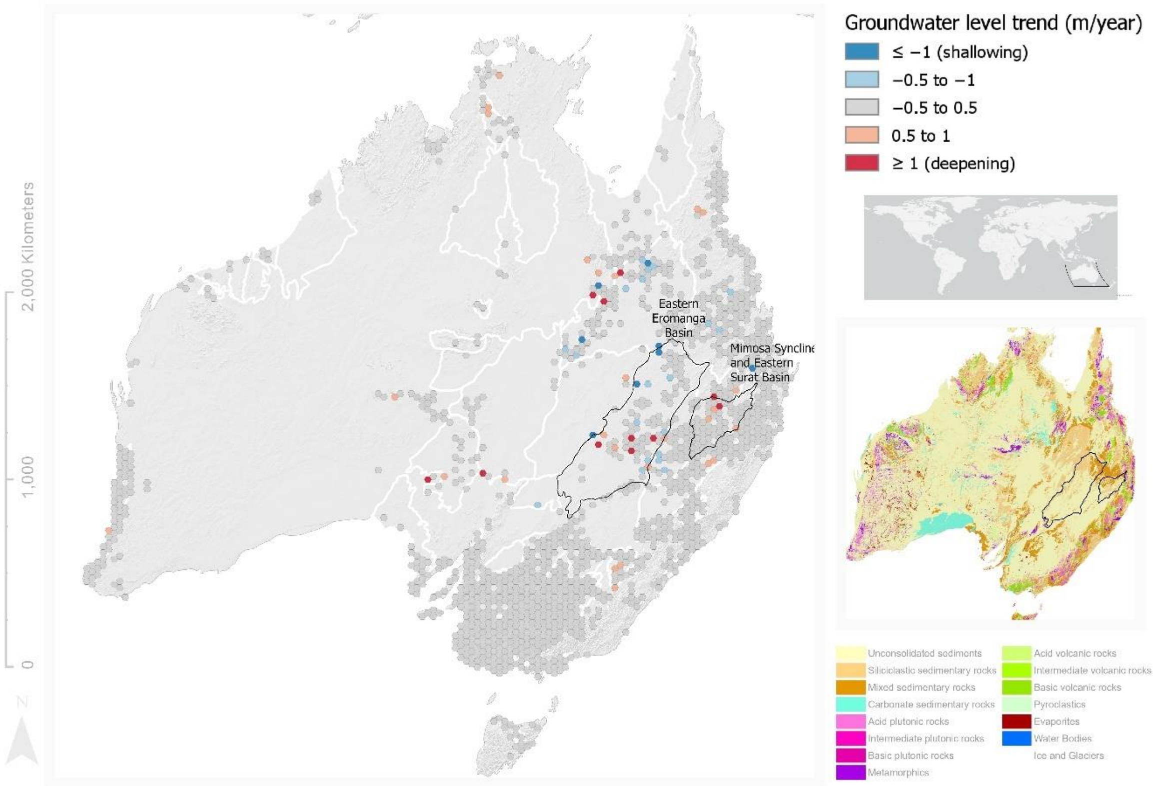

Supplementary Fig. 277. 21st-century trends in depth to groundwater in 1,000 km² areas across Australia. Each hexagonal area represents one 1,000 km² area. The colour of the hexagon corresponds to the median Theil-Sen slope among all monitoring wells within the 1,000 km² area. Red and orange hexagons represent areas where the aquifer-scale trend in depth to groundwater exceeds 0.5 m/year (i.e., groundwater levels became deeper over time faster than 0.5 m/year). Blue hexagons represent areas where the aquifer-scale trend in depth to groundwater is less than -0.5 m/year (i.e., groundwater levels becoming shallower over time faster than -0.5 m/year; see legend in upper-right corner of figure). The grey map (right side of figure) shows the area covered by the main map (see dark grey polygon on global map). The map in the lower-right corner of the figure shows spatial variations in near-surface geology across the area depicted in the main map (geologic data from ref.[1360]: Hartmann, J., & Moosdorf, N. The new global lithological map database GLiM: A representation of rock properties at the Earth surface. *Geochemistry, Geophysics, Geosystems* **13,** doi.org/10.1029/2012GC004370 (2012)). Black polylines with labels outline some of our delineated aquifer systems. White polylines outline other aquifer systems (i.e., aquifer systems that are not specifically labelled in this figure).

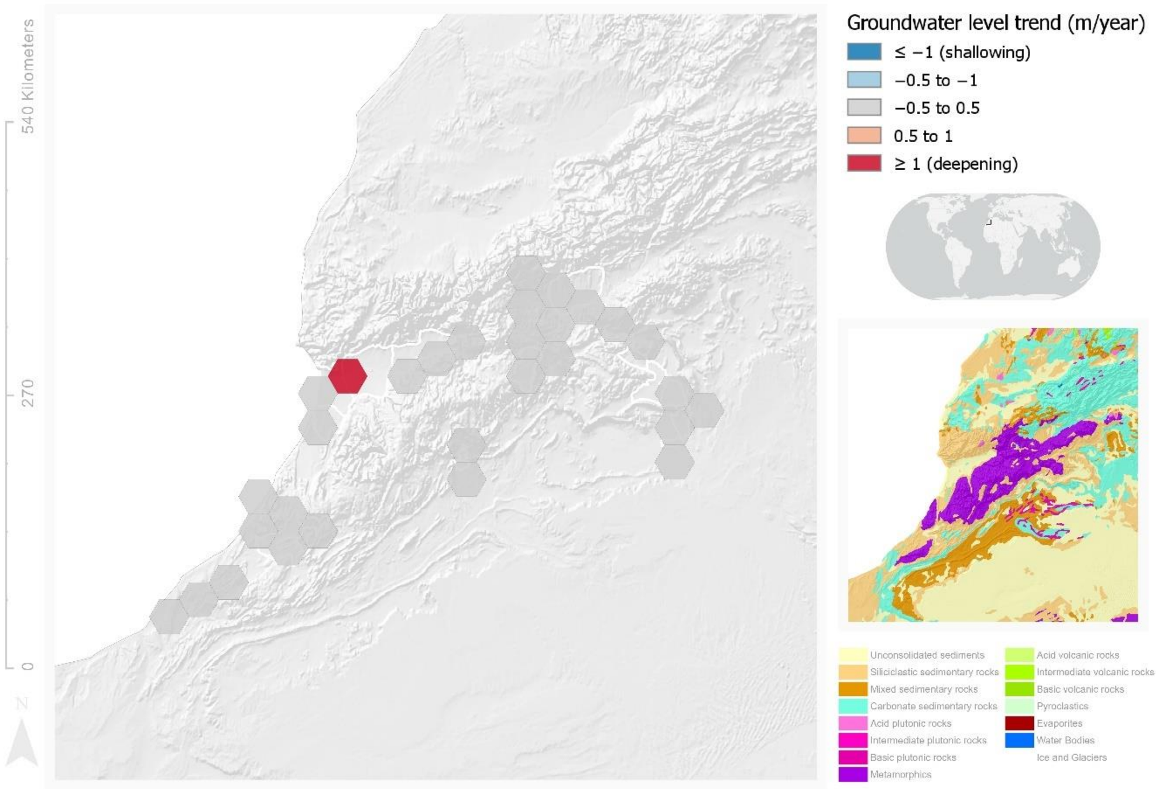

Supplementary Fig. 278. 21st-century trends in depth to groundwater in 1,000 km² areas in northwestern Africa. Each hexagonal area represents one 1,000 km² area. The colour of the hexagon corresponds to the median Theil-Sen slope among all monitoring wells within the 1,000 km² area. Red and orange hexagons represent areas where the aquifer-scale trend in depth to groundwater exceeds 0.5 m/year (i.e., groundwater levels became deeper over time faster than 0.5 m/year). Blue hexagons represent areas where the aquifer-scale trend in depth to groundwater is less than -0.5 m/year (i.e., groundwater levels becoming shallower over time faster than -0.5 m/year; see legend in upper-right corner of figure). The grey map (right side of figure) shows the area covered by the main map (see dark grey polygon on global map). The map in the lower-right corner of the figure shows spatial variations in near-surface geology across the area depicted in the main map (geologic data from ref.[1360]: Hartmann, J., & Moosdorf, N. The new global lithological map database GLiM: A representation of rock properties at the Earth surface. *Geochemistry, Geophysics, Geosystems* **13,** doi.org/10.1029/2012GC004370 (2012)). Black polylines with labels outline some of our delineated aquifer systems. White polylines outline other aquifer systems (i.e., aquifer systems that are not specifically labelled in this figure).

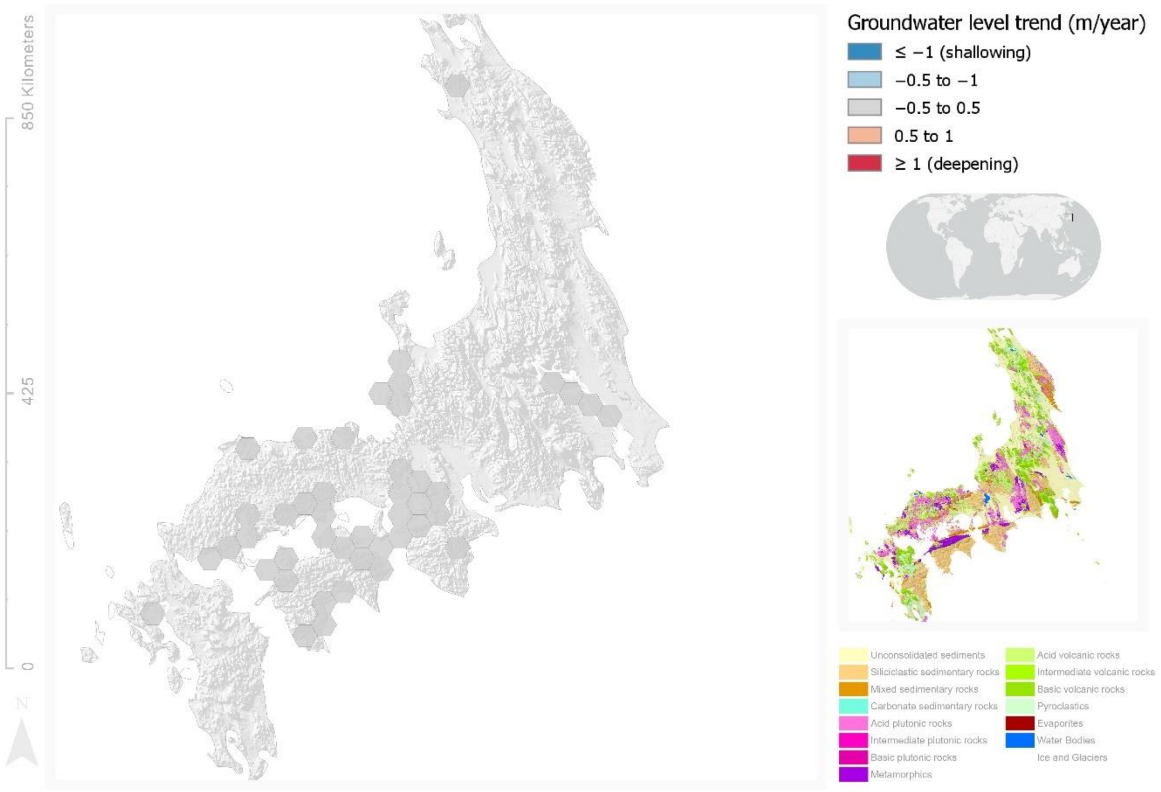

Supplementary Fig. 279. 21st-century trends in depth to groundwater in 1,000 km^2 areas in Japan. Each hexagonal area represents one 1,000 km^2 area. The colour of the hexagon corresponds to the median Theil-Sen slope among all monitoring wells within the 1,000 km^2 area. Red and orange hexagons represent areas where the aquifer-scale trend in depth to groundwater exceeds 0.5 m/year (i.e., groundwater levels became deeper over time faster than 0.5 m/year). Blue hexagons represent areas where the aquifer-scale trend in depth to groundwater is less than -0.5 m/year (i.e., groundwater levels becoming shallower over time faster than -0.5 m/year; see legend in upper-right corner of figure). The grey map (right side of figure) shows the area covered by the main map (see dark grey polygon on global map). The map in the lower-right corner of the figure shows spatial variations in near-surface geology across the area depicted in the main map (geologic data from ref.[1360]: Hartmann, J., & Moosdorf, N. The new global lithological map database GLiM: A representation of rock properties at the Earth surface. *Geochemistry, Geophysics, Geosystems* **13,** doi.org/10.1029/2012GC004370 (2012)). Black polylines with labels outline some of our delineated aquifer systems. White polylines outline other aquifer systems (i.e., aquifer systems that are not specifically labelled in this figure).

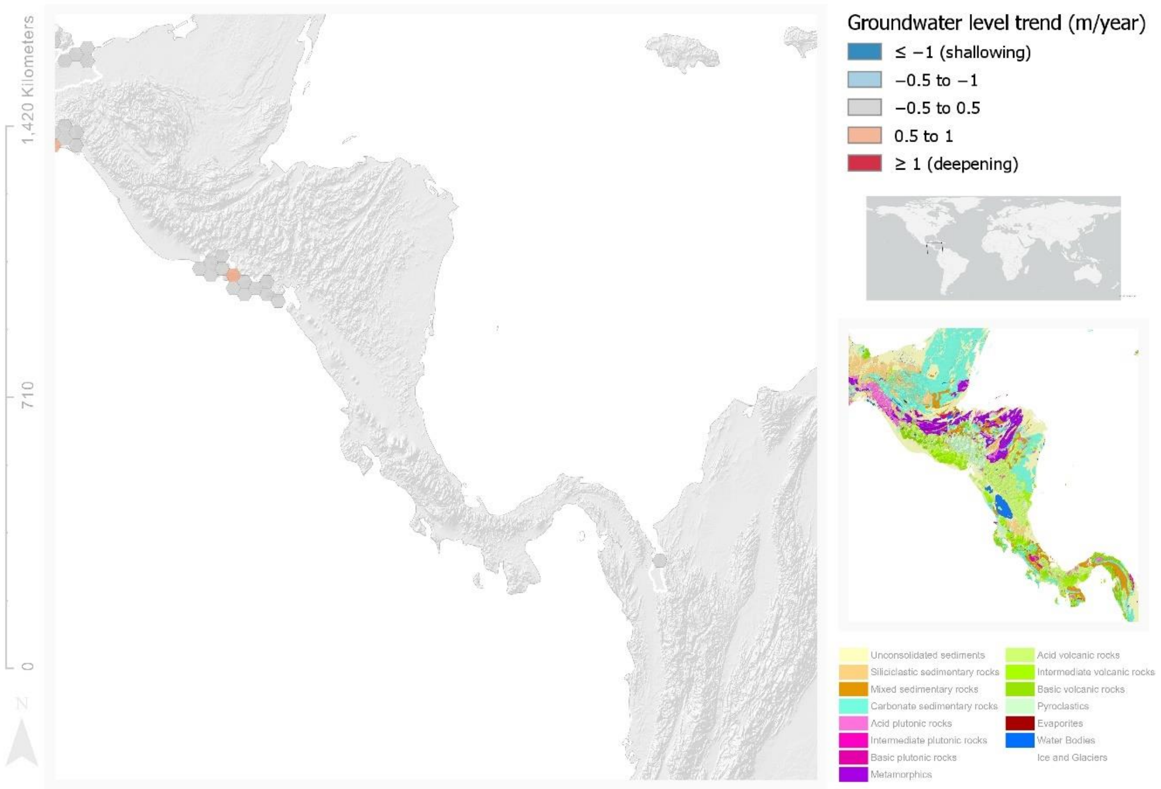

Supplementary Fig. 280. 21st-century trends in depth to groundwater in 1,000 km² areas in Central America. Each hexagonal area represents one 1,000 km² area. The colour of the hexagon corresponds to the median Theil-Sen slope among all monitoring wells within the 1,000 km² area. Red and orange hexagons represent areas where the aquifer-scale trend in depth to groundwater exceeds 0.5 m/year (i.e., groundwater levels became deeper over time faster than 0.5 m/year). Blue hexagons represent areas where the aquifer-scale trend in depth to groundwater is less than -0.5 m/year (i.e., groundwater levels becoming shallower over time faster than -0.5 m/year; see legend in upper-right corner of figure). The grey map (right side of figure) shows the area covered by the main map (see dark grey polygon on global map). The map in the lower-right corner of the figure shows spatial variations in near-surface geology across the area depicted in the main map (geologic data from ref.[1360]: Hartmann, J., & Moosdorf, N. The new global lithological map database GLiM: A representation of rock properties at the Earth surface. *Geochemistry, Geophysics, Geosystems* **13,** doi.org/10.1029/2012GC004370 (2012)). Black polylines with labels outline some of our delineated aquifer systems. White polylines outline other aquifer systems (i.e., aquifer systems that are not specifically labelled in this figure).

19 COMPARING GROUNDWATER-LEVEL TRENDS IN UNCONFINED AND CONFINED AQUIFERS

Trends in depth to groundwater can differ in shallower versus deeper monitoring wells (see ref.[1358]: Taylor, C.J., & Alley, W.M. Ground-water-level monitoring and the importance of long-term water-level data. US Geological Survey Circular 1217, 77 pp. (2001). Accessed March 9, 2023 via https://pubs.usgs.gov/circ/circ1217/pdf/circular1217.pdf). We completed two analyses to compare trends in depth to groundwater in deeper and shallower monitoring wells.

(i) We compare trends in depth to groundwater in deep versus shallow wells (see Supplementary Note 19.1).

(ii) We compare trends in depth to groundwater in monitoring wells classified as tapping unconfined aquifers versus those classified as tapping confined aquifers (see Supplementary Note 19.2).

19.1 GROUNDWATER LEVEL TRENDS IN DEEPER VERSUS SHALLOWER WELLS

We compare trends in depth to groundwater for deeper versus shallower monitoring wells. Specifically, the table below presents variations in trends in depth to groundwater (Theil-Sen slopes) among monitoring wells falling within one of the following groups:

- *(a) Monitoring well depth is less than or equal to 30 m; or,*
- *(b) Monitoring well depth is more than 30 m and less than or equal to 100 m; or,*
- *(c) Monitoring well depth is more than 100 m.*

High-magnitude trends in depth to groundwater (i.e., Theil-Sen slopes of <−1 m/year or >1 m/year) are more common in deeper monitoring wells than in shallower monitoring wells (see the following table and figure).

Supplementary Table 13. Percent of shallow (<30 m), moderately deep (30-100 m), and deep (>100 m) monitoring wells recording relatively rapid changes in groundwater levels

	**21st-century groundwater level trends* (Theil-Sen slope)**					
	**<−2 m/year**	**−2 to −1 m/year**	**−1 to 0 m/year**	**0 to 1 m/year**	**1 to 2 m/year**	**>2 m/year**
Monitoring wells with a depth of **less than or equal to 30 m**	<0.1%	<0.1%	43.9%	55.9%	0.2%	<0.1%
Monitoring wells with a depth of **greater than 30 m** but **less than or equal to 100 m**	0.1%	0.3%	38.0%	60.2%	1.3%	0.2%
Monitoring wells with a depth of **greater than 100 m**	1.2%	2.4%	33.8%	54.5%	5.9%	2.3%

*values expressed in units of percent, defined as the percent of monitoring wells with Theil-Sen slopes within a given range (e.g., the percent of monitoring wells with depths of greater than 100 m that have a trend in depth to groundwater of >2 m/year (Theil-Sen slope) is 2.3%, as stated in the bottom-right corner of table).

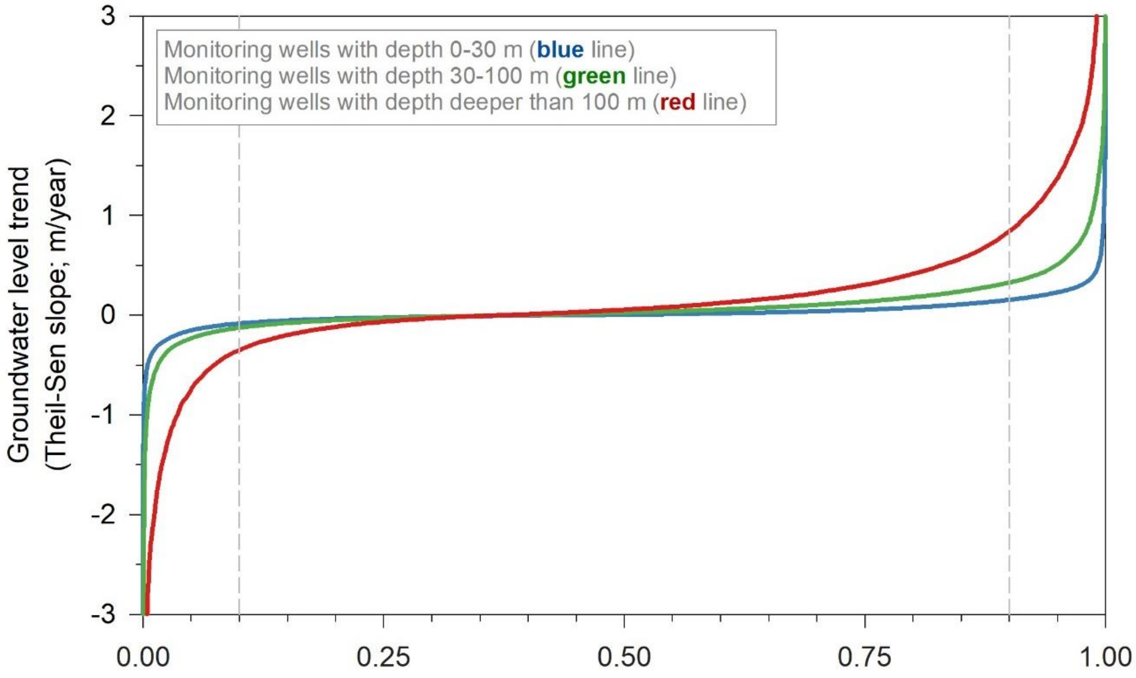

Supplementary Fig. 281. Cumulative distribution of 21st century trends in depth to groundwater among monitoring wells grouped by their total well depths (positive y-axis values indicate that groundwater levels became deeper over time). Each line presents a cumulative distribution of groundwater level trends (Theil-Sen slopes) during 2000-2022 for all monitoring wells within one of the following depth classes: (i) monitoring well depth is less than or equal to 30 m (blue line), (ii) monitoring well depth is greater than 30 m and less than or equal to 100 m (green line), or (iii) monitoring well depth is greater than 100 m. Vertical dashed lines correspond to x-axis values of 0.1 (i.e., 10% of monitoring wells) and 0.9 (i.e., 90% of monitoring wells). The deepest monitoring wells (red line) have a larger proportion of high-magnitude Theil-Sen slopes relative to the shallowest wells (i.e., the red line intersects the vertical grey dashed lines at higher magnitude values than the blue line does). This analysis highlights that high-magnitude groundwater shallowing trends and high-magnitude groundwater deepening trends are disproportionately common in deeper wells (relative to wells with shallower total depths). For example, ~10% of deep (>100 m depth) monitoring wells have Theil-Sen slopes exceeding 0.8 m/year (see intersection of red line with vertical grey dashed line located at an x-axis value of 0.9).

To further compare groundwater level trends in shallow versus deep wells, we identified aquifer systems with both deep (>100 m) and shallow (<30 m) monitoring wells.

Specifically, we identified n=232 aquifer systems with at least 4 shallow (<30 m deep) monitoring wells and at least 4 deep (>100 m) monitoring wells with sufficient data to calculate a 21st-century groundwater level trend (median Theil-Sen slope; see main text methods). For each of these n=232 aquifer systems, we calculated two aquifer-scale groundwater level trends: (i) an aquifer-scale groundwater level trend based solely on shallow (<30 m) monitoring wells (median Theil-Sen slope of all shallow monitoring wells), and (ii) an aquifer-scale groundwater level trend based solely on deep (>100 m) monitoring wells (median Theil-Sen slope of all shallow monitoring wells). We then compared these aquifer-scale groundwater level trends across all n=232 aquifer systems (figure below).

Among these n=232 aquifer systems, we show that aquifer-scale groundwater level trends based on deeper monitoring wells tend to have higher-magnitude values than trends based on shallow monitoring wells. Further, aquifer-scale groundwater level trends based on deep monitoring wells exceed trends based on shallow monitoring wells in a large number of aquifer systems (152 of 232 aquifer systems), implying that deepening groundwater level trends (or slower groundwater level shallowing trends) are more prevalent among deep monitoring wells compared to shallow monitoring wells (in these n=232 aquifer systems). Some of the aquifer systems where groundwater level deepening trends based on deep monitoring wells are outpacing deepening trends observed in shallow monitoring wells are in California's Central Valley (e.g., Tulare Lake Basin, Kaweah Basin, Kings Basin, Chowchilla Basin; see figure below). That groundwater levels are falling faster in deeper monitoring wells (relative to shallower monitoring wells) may imply a strengthening of downward-oriented hydraulic gradients in these areas, which can speed the movement of shallower groundwater to deeper aquifers, potentially impacting the quality of these deep groundwaters if the shallow groundwater contains mobile contaminants.

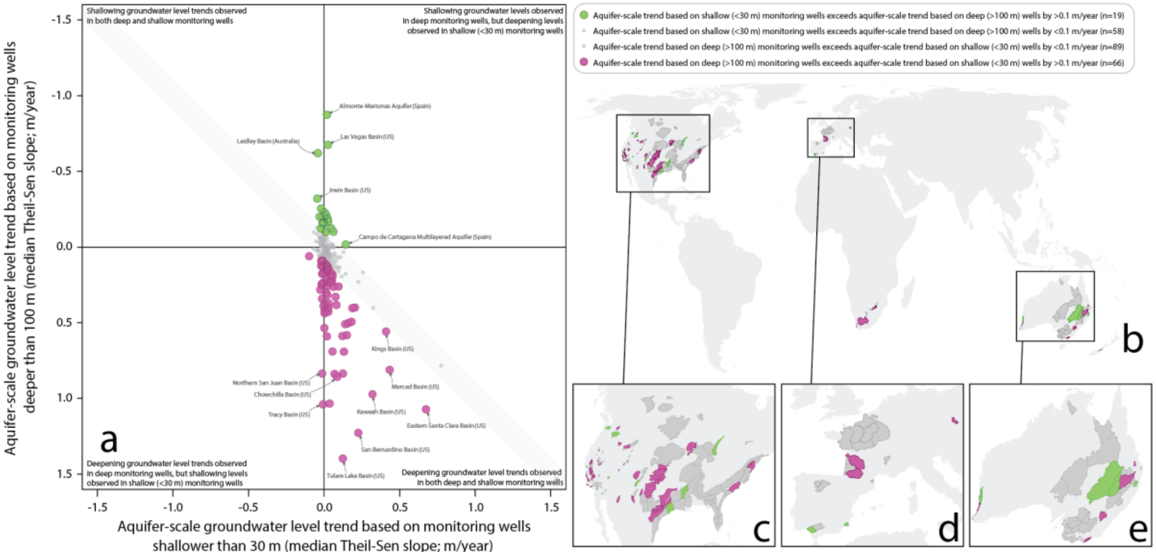

Supplementary Fig. 282. 21st-century aquifer-scale trends in depth to groundwater based on shallow versus deep monitoring wells. (a) Each point represents one aquifer system. The x-axis values are the aquifer-scale groundwater level trend based solely on monitoring wells that have total depths of no more than 30 m ("shallow" monitoring wells; aquifer-scale trend determined by the median of all shallow monitoring wells with sufficient data to determine a 21st-century groundwater level trend – see main text methods for details). The y-axis values represent aquifer-scale groundwater level trend based solely on monitoring wells that have total depths of greater than 100 m ("deep" monitoring wells; aquifer-scale trend determined by the median of all deep monitoring wells with sufficient data to determine a 21st-century groundwater level trend). Larger-sized points represent aquifer systems where the aquifer-scale groundwater level trend based on shallow (<30 m) monitoring wells differs from the aquifer-scale groundwater level trend based on deep (<100 m) monitoring wells by more than 0.1 m/year; among these points (i.e., large purple points and large green points), aquifer-scale trends in depth to groundwater that are based on deeper wells exceed aquifer-scale trends based on shallow monitoring wells for the majority of aquifer systems presented here. This analysis emphasizes that groundwater level trends in deep and shallow monitoring wells can differ even when these wells are located in the same aquifer system. However, we also highlight that in most of the aquifer systems shown here, the aquifer-scale groundwater level trend based on shallow monitoring wells differs from the aquifer-scale groundwater level trend based on deep monitoring wells by less than 0.1 m/year (147 of 232 aquifer systems). **(b)** Spatial distribution of aquifer systems and differences in aquifer-scale trends in depth to groundwater when based solely on shallow monitoring wells (total well depth is <30 m) versus deep monitoring wells (total well depth is >100 m). The colour of each aquifer system corresponds to the shades presented in panel a (and detailed in the legend in the top right corner of this figure). **(c-e)** Continental-scale maps for North America, western Europe, and Australia.

Previous works have compared trends in depth to groundwater among shallow versus deep monitoring wells in regions that are not included in the analysis presented in the Supplementary Fig. 282 shown above (e.g., ref.[1357]: Malakar, P., Mukherjee, A., Bhanja, S.N., Ganguly, A.R., Ray, R.K., Zahid, A., Sarkar, S., Saha, D., Chattopadhyay, S. Three decades of depth-dependent groundwater response to climate variability and human regime in the transboundary Indus-Ganges-Brahmaputra-Meghna mega river basin aquifers. *Advances in Water Resources* **149,** 103856 (2021)). We acknowledge that our data compilation may not include well depth data in all possible cases, and that the analyses presented here focus only on areas where we were able to compile well depth data (in addition to groundwater level time series).

19.2 GROUNDWATER LEVEL TRENDS IN WELLS THAT THE USGS HAS IDENTIFIED AS TAPPING UNCONFINED OR CONFINED STORAGE CONDITIONS

We compared trends in depth to groundwater in wells that the US Geological Survey has classified as tapping unconfined aquifers versus those classified as tapping confined aquifers. For each US Geological Survey monitoring well, we downloaded metadata from https://nwis.waterdata.usgs.gov/nwis (accessed November 9, 2022). We classified all US Geological Survey monitoring wells into one of three categories: (i) monitoring well taps confined conditions ("aqfr_type_cd" value in US Geological Survey database of either "C" or "M"); or, (ii) monitoring well taps unconfined conditions ("aqfr_type_cd" value in US Geological Survey database of either "N" or "U"); or, (iii) the conditions of the aquifer that the monitoring well taps are not specified ("aqfr_type_cd" value in US Geological Survey database of either "X" or no data). For further details, see the classifications presented in Supplementary Table 14.

We compared spatial patterns of trends in depth to groundwater level (Theil-Sen slopes) among wells classified as tapping unconfined aquifers versus those classified as tapping confined aquifers (see the supplementary figures on the following pages). The monitoring wells presented in Supplementary Figs. 283-286 demonstrate that there are some regions where confined and unconfined aquifer systems are experiencing different trends in depth to groundwater (e.g., in the northern portion of the North Atlantic Coastal Plain; see Supplementary Fig. 284).

Supplementary Table 14. US Geological Survey classifications for monitoring wells and the aquifers that they tap

**Unique value in US Geological Survey database*** **(field entitled "aqfr_type_cd")**	**Description from US Geological Survey "Search criteria and codes" website****	**Classification in this manuscript**
**C**	Confined single aquifer	Confined
**M**	Confined multiple aquifers	Confined
**N**	Unconfined multiple aquifer	Unconfined
**U**	Unconfined single aquifer	Unconfined
**X**	Mixed (confined and unconfined) multiple aquifers	Unknown
(no data)	n/a	Unknown

* https://nwis.waterdata.usgs.gov/nwis (accessed November 9, 2022)

** https://help.waterdata.usgs.gov/codes-and-parameters (accessed November 16, 2022)

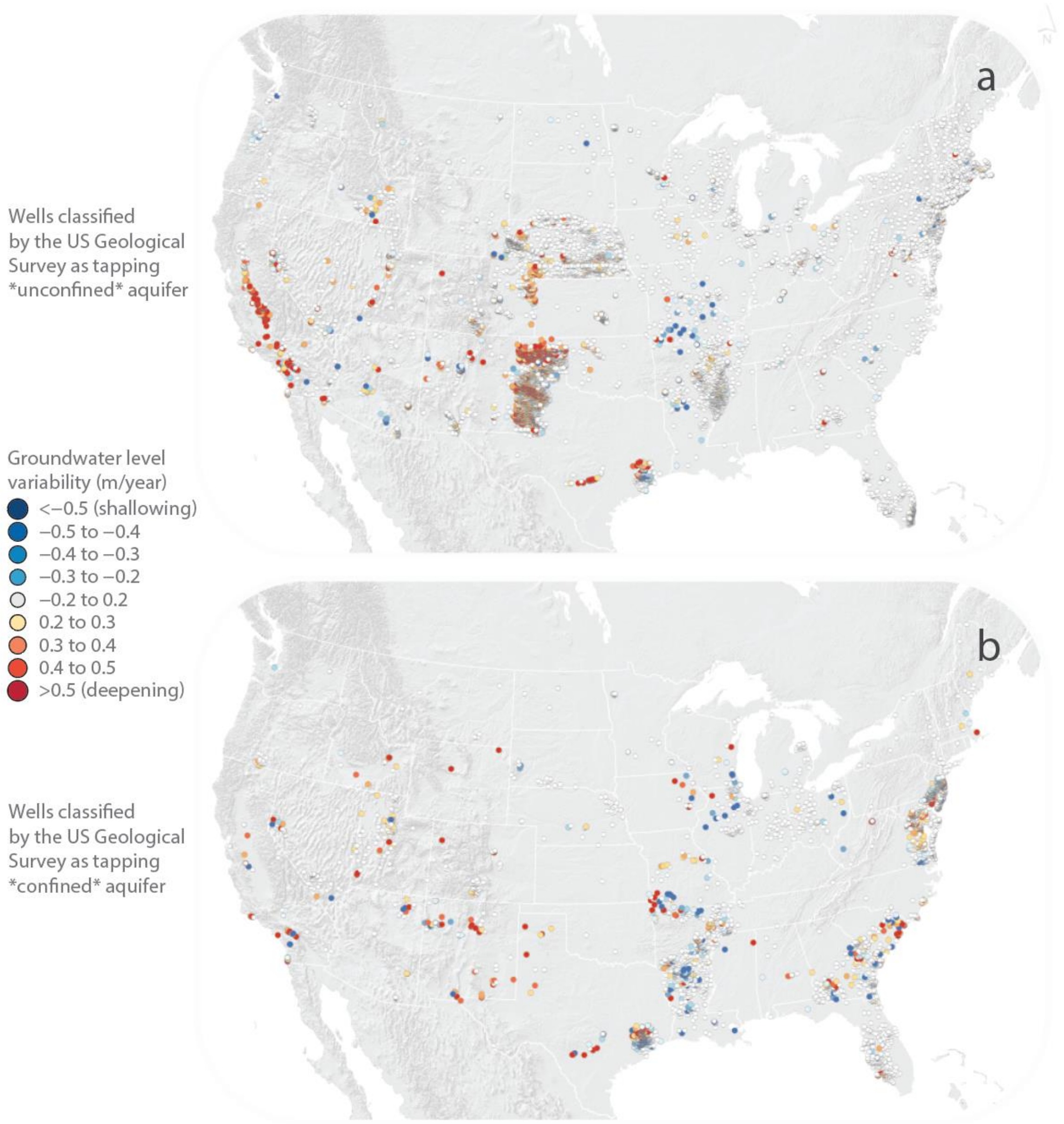

Supplementary Fig. 283. Trends in depth to groundwater in monitoring wells classified as tapping either unconfined or confined conditions. Each point represents one monitoring well with adequate data for analysis (see Methods). The colour of the point corresponds to the 21st-century trend in depth to groundwater (Theil-Sen slope). Light and dark blue points represent monitoring wells where groundwater levels have become shallower over time. Yellow and red points represent monitoring wells where groundwater levels have become deeper over time. **a)** Monitoring wells categorised by the US Geological Survey as tapping unconfined conditions (based on the field entitled "aqfr_type_cd"). **b)** Monitoring wells categorised by the US Geological Survey as tapping confined conditions (based on the field entitled "aqfr_type_cd"). This continent-wide analysis (i.e., of the contiguous US) demonstrates that there are some aquifer systems where unconfined and confined aquifers are experiencing different groundwater level trends over time. For example, in an aquifer system along the northeastern US coast known as the "North Atlantic Coastal Plain", monitoring wells tapping unconfined conditions have relatively stable groundwater levels over time (see many white circles in northeastern US in panel a, signifying near-constant groundwater levels over time), but monitoring wells tapping confined conditions show groundwater level deepening (see yellow and red circles in northeastern US in panel b). For a zoomed in version of the North Atlantic Coastal Plain see Supplementary Fig. 284 below.

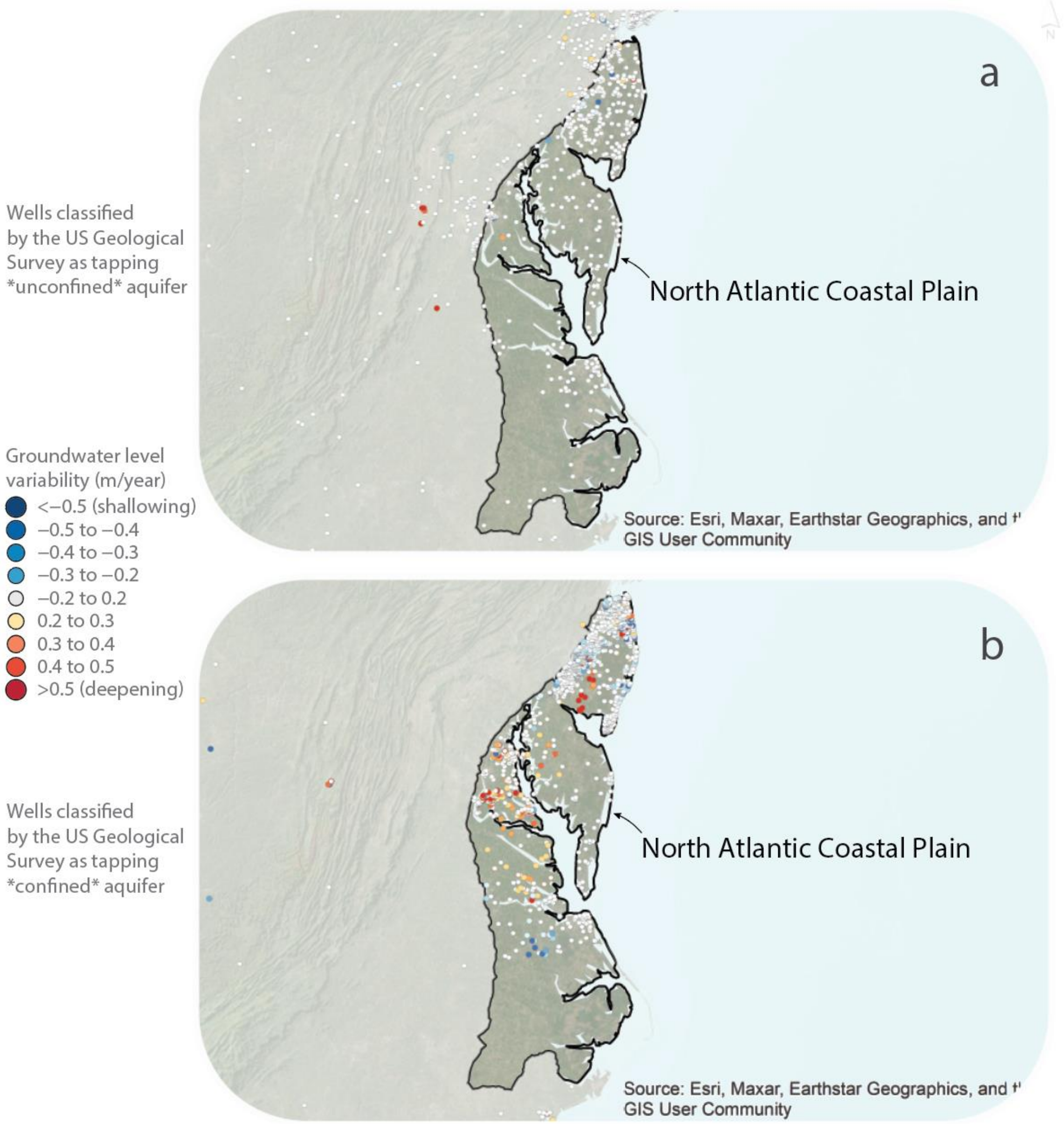

Supplementary Fig. 284. Groundwater level trends in the North Atlantic Coastal Plain among monitoring wells classified as tapping either unconfined or confined conditions. Each point represents one monitoring well with adequate data for analysis (see Methods). The colour of the point corresponds to the 21st-century trend in depth to groundwater (Theil-Sen slope). Light and dark blue points represent monitoring wells where groundwater levels have become shallower over time. Yellow and red points represent monitoring wells where groundwater levels have become deeper over time. **a)** Monitoring wells categorised by the US Geological Survey as tapping unconfined conditions (based on the field entitled "aqfr_type_cd"). **b)** Monitoring wells categorised by the US Geological Survey as tapping confined conditions (based on the field entitled "aqfr_type_cd"). Monitoring wells tapping unconfined conditions (upper panel) have relatively stable groundwater levels over time (see many white circles, signifying near-constant groundwater levels over time), but monitoring wells tapping confined conditions tend to exhibit groundwater level deepening in some areas (e.g., southern New Jersey; see yellow and red circles in panel b). Imagery from https://www.arcgis.com/home/item.html?id=10df2279f9684e4a9f6a7f08febac2a9

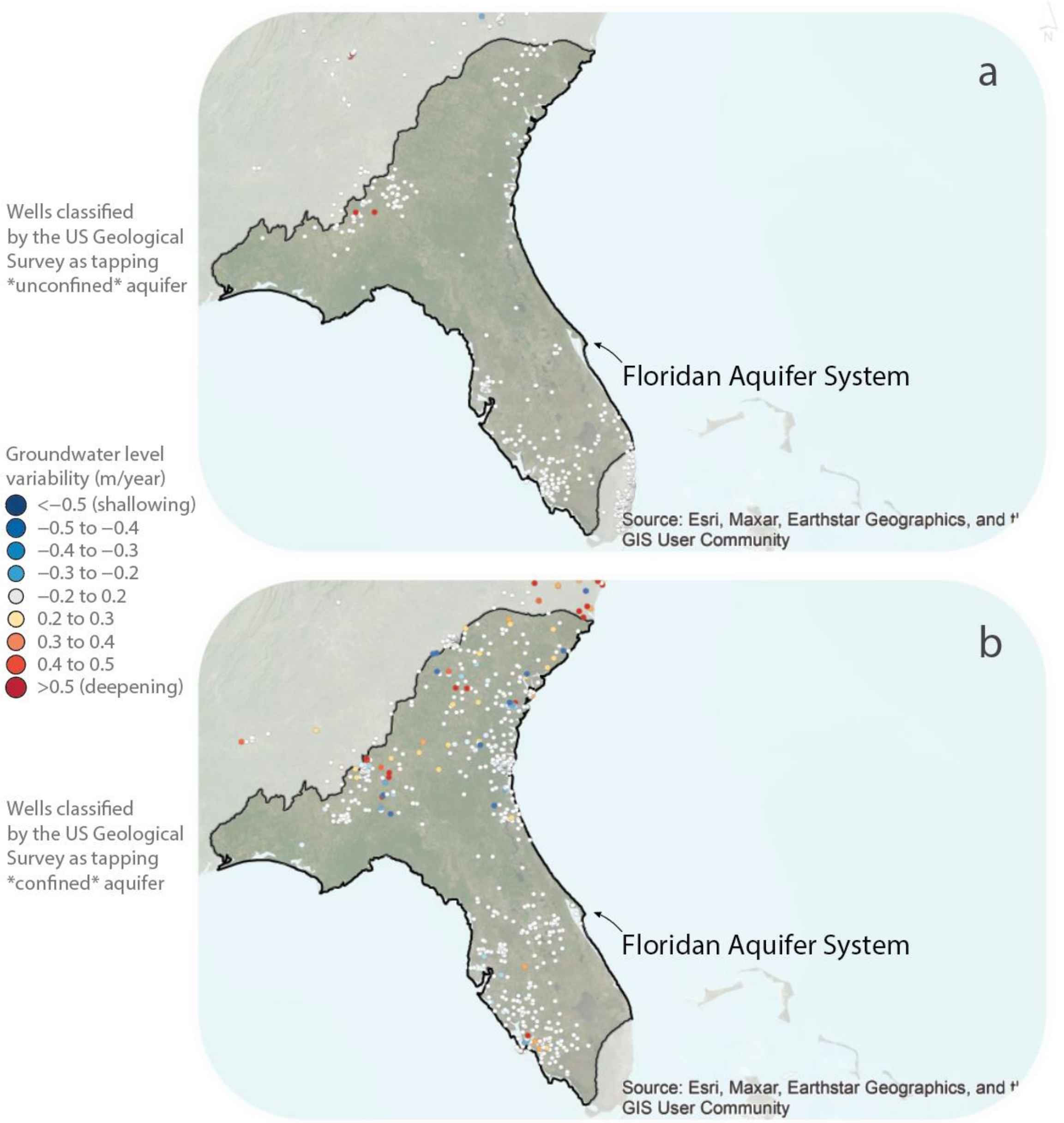

Supplementary Fig. 285. Groundwater level trends in the Floridan Aquifer System among monitoring wells classified as tapping either unconfined or confined conditions. Each point represents one monitoring well with adequate data for analysis (see Methods). The colour of the point corresponds to the 21st-century trend in depth to groundwater (Theil-Sen slope). Light and dark blue points represent monitoring wells where groundwater levels have become shallower over time. Yellow and red points represent monitoring wells where groundwater levels have become deeper over time. **a)** Monitoring wells categorised by the US Geological Survey as tapping unconfined conditions (based on the field entitled "aqfr_type_cd"). **b)** Monitoring wells categorised by the US Geological Survey as tapping confined conditions (based on the field entitled "aqfr_type_cd"). Imagery from https://www.arcgis.com/home/item.html?id=10df2279f9684e4a9f6a7f08febac2a9

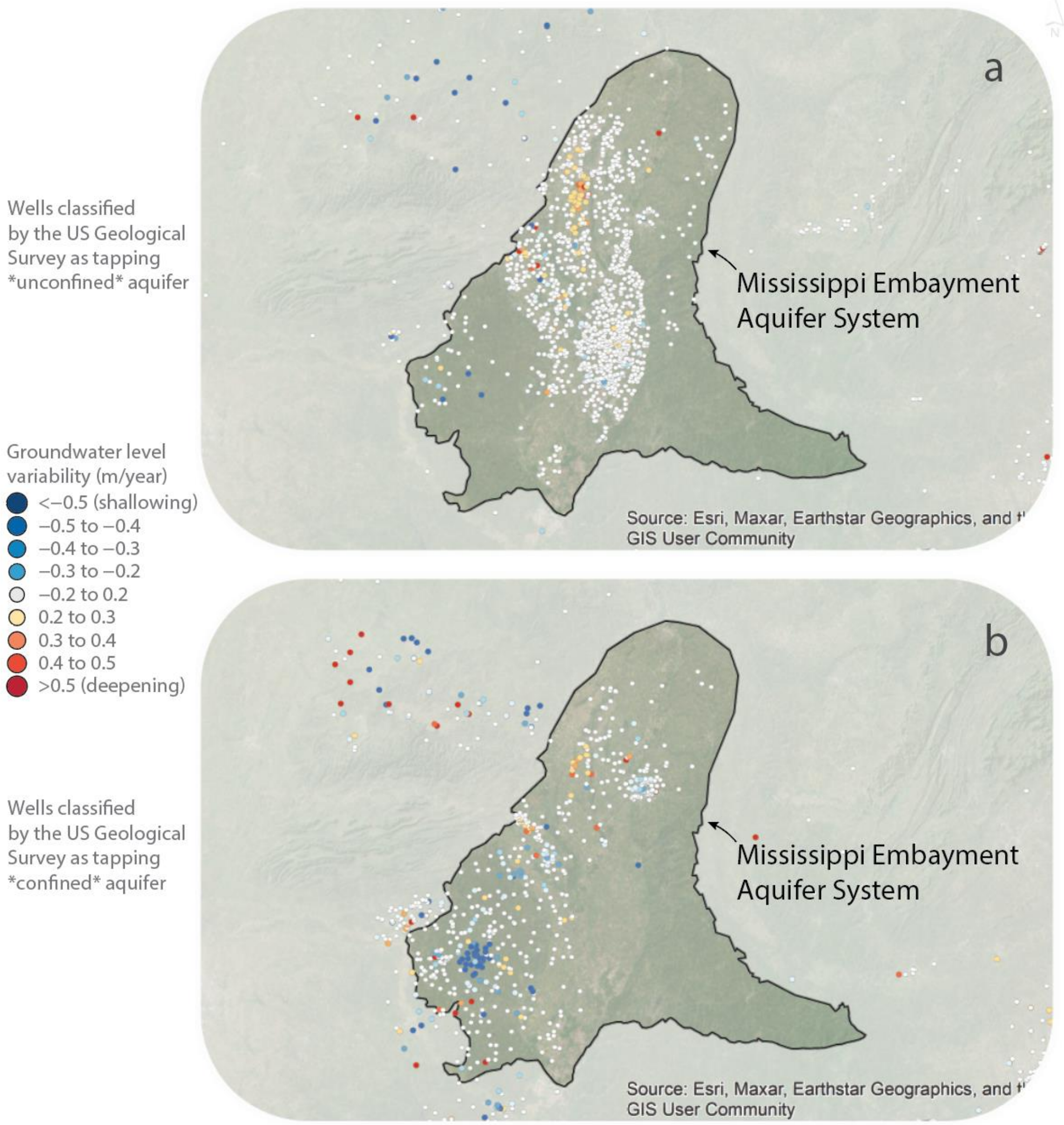

Supplementary Fig. 286. Groundwater level trends in the Mississippi Embayment Aquifer System among monitoring wells classified as tapping either unconfined or confined conditions. Each point represents one monitoring well with adequate data for analysis (see Methods). The colour of the point corresponds to the 21st-century trend in depth to groundwater (Theil-Sen slope). Light and dark blue points represent monitoring wells where groundwater levels have become shallower over time. Yellow and red points represent monitoring wells where groundwater levels have become deeper over time. **a)** Monitoring wells categorised by the US Geological Survey as tapping unconfined conditions (based on the field entitled "aqfr_type_cd"). **b)** Monitoring wells categorised by the US Geological Survey as tapping confined conditions (based on the field entitled "aqfr_type_cd"). The spatial patterns of trends in depth to groundwater are generally similar among monitoring wells tapping unconfined conditions (upper panel) and monitoring wells tapping confined conditions (lower panel). However, the spatial distributions of the monitoring wells differ, with unconfined monitoring wells being more prevalent in the central portion of the Mississippi Embayment Regional Aquifer System, and confined monitoring wells (many of which likely tap the Claiborne Formations) being more abundant in the southwestern portion of the study area. Our comparison of groundwater level trends in confined versus unconfined aquifers in the Mississippi Embayment Regional Aquifer System highlights that some of our aquifer systems are likely based mostly on monitoring wells tapping confined conditions (e.g., western Mississippi Embayment) whereas our results for other aquifer systems are likely based mostly on monitoring wells tapping unconfined conditions (e.g., central Mississippi Embayment). Imagery from https://www.arcgis.com/home/item.html?id=10df2279f9684e4a9f6a7f08febac2a9

20 POTENTIAL FOR MONITORING WELLS TO HAVE RUN DRY

It is possible that some monitoring well water level time series may be truncated because groundwater levels decline below the bottom of the well (see ref.[1361]: Hora, T., Srinivasan, V., & Basu, N.B. The groundwater recovery paradox in South India. *Geophysical Research Letters* **46,** 9602–9611 (2019)). To explore the potential that monitoring wells ran dry during the 21st century we (i) developed a map of monitoring well depths (72,403 of the 169,178 monitoring wells in Fig. 1 have a recorded well depth value), (ii) calculated the deepest 21st-century annual median groundwater level in each of these wells, and (iii) mapped the vertical offset between well bottoms and the deepest annual median groundwater level (i.e., [monitoring well depth] minus [deepest annual depth to groundwater]; see maps on the following pages).

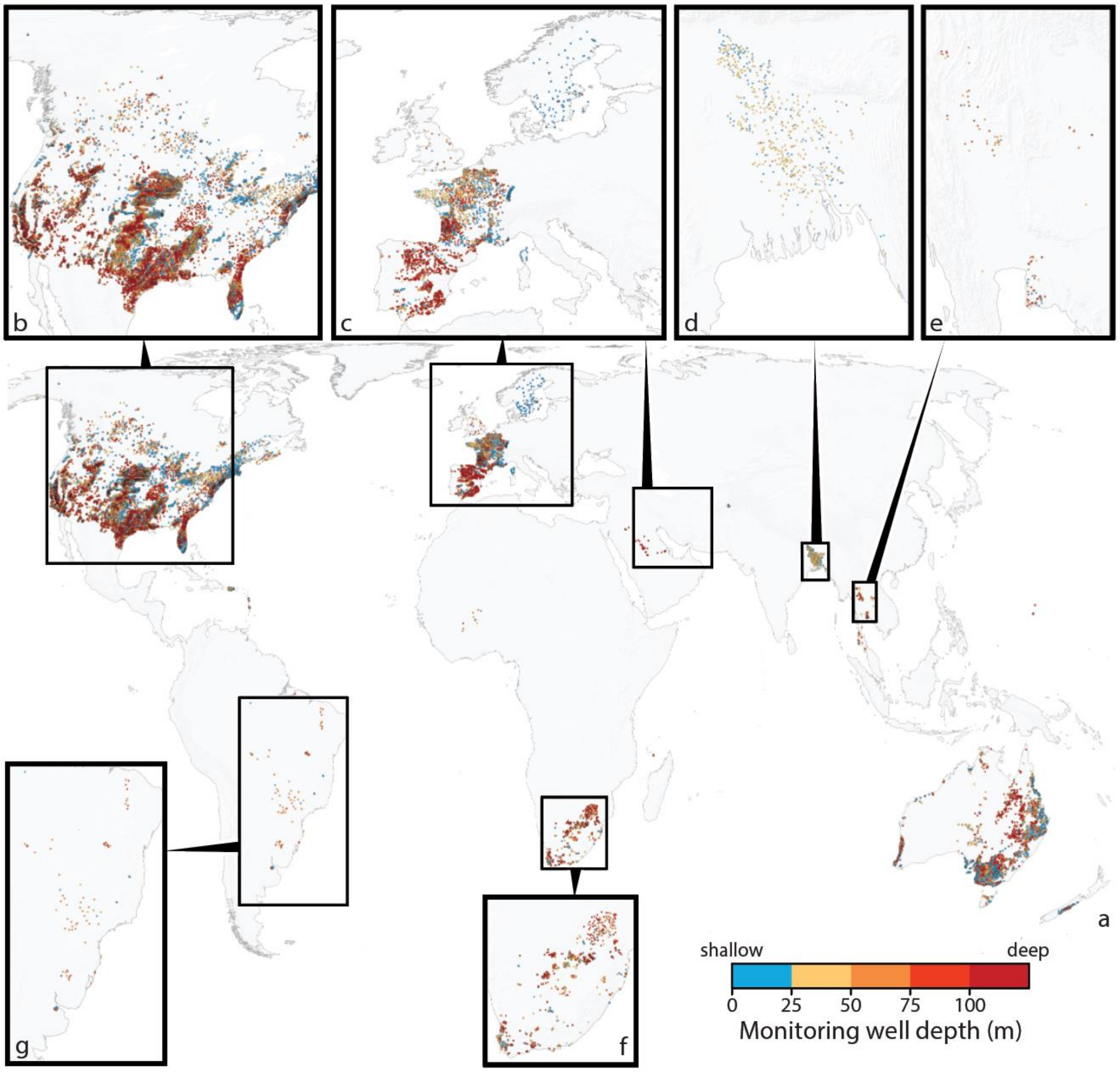

Supplementary Fig. 287. Recorded total depths of monitoring wells. Each circle represents one monitoring well (subset from the 170 thousand monitoring wells displayed in main text Fig. 1). Blue points represent monitoring wells that are shallower than 25 m, whereas orange and red points represent monitoring wells completed to deeper depths. (a) Global map of monitoring well depths. (b-g) Regional maps of monitoring well depths.

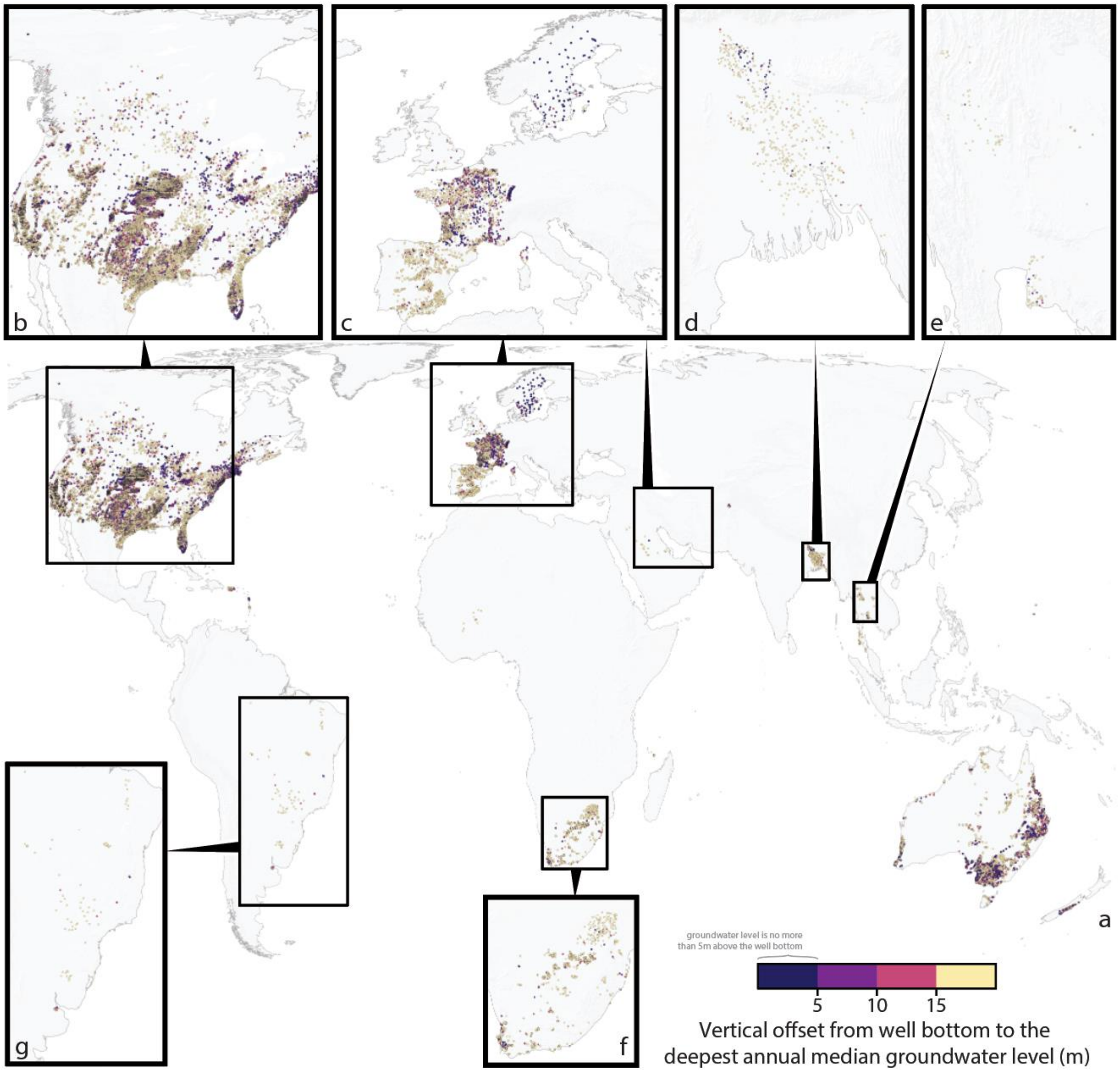

Supplementary Fig. 288. Vertical offset from the bottom of monitoring wells to the deepest annual median groundwater level observed during the 21st century. Each circle represents one monitoring well (subset from the 170 thousand monitoring wells displayed in main text Fig. 1). Dark purple points represent monitoring wells where the deepest annual median groundwater level fell to within 5 m of the well bottom for at least one year during the 21st century; the deepest annual median groundwater level fell to within 5 m of the well bottom for at least one year during the 21st century in just 11% of monitoring wells (among the 72,403 monitoring wells with depth data depicted here). Lighter purple and yellow points represent monitoring wells where even the deepest annual median groundwater level did not come within 5 m of the well bottom during the 21st century (implying a smaller likelihood that the monitoring well went dry during the 21st century). (a) Global map of monitoring wells and their vertical offsets between well bottoms and the deepest annual median groundwater levels. (b-g) Regional maps of monitoring wells and their vertical offsets between well bottoms and the deepest annual median groundwater levels.

The annual median groundwater level fell to within 5 m of the well bottom for at least one year during the 21st century in just 11% of monitoring wells (7,995 wells among the 72,403 monitoring wells – with depth data – depicted in the map on the preceding page are shaded dark purple). Therefore, it is unlikely that the great majority of the monitoring wells presented in main text Fig. 1 were at risk of experiencing an event where the groundwater fell below the well bottom (though we highlight that automated water level sensors reside above the bottom of the well, and thus we cannot rule out the possibility that these sensors ran dry for some of the monitoring wells we studied here). Furthermore, among these 7,995 wells, just 924 wells (i.e., 1.3% of the 72,403 monitoring wells displayed above) also have a 21st century trend in depth to groundwater exceeding 0.1 m/year (i.e., 924 wells have deepening groundwater levels and at least one year in the 21st century where the annual median groundwater level fell to within 5 m of the well bottom during the 21st century). One example of a monitoring well that likely ran dry is depicted below (see time series plot below).

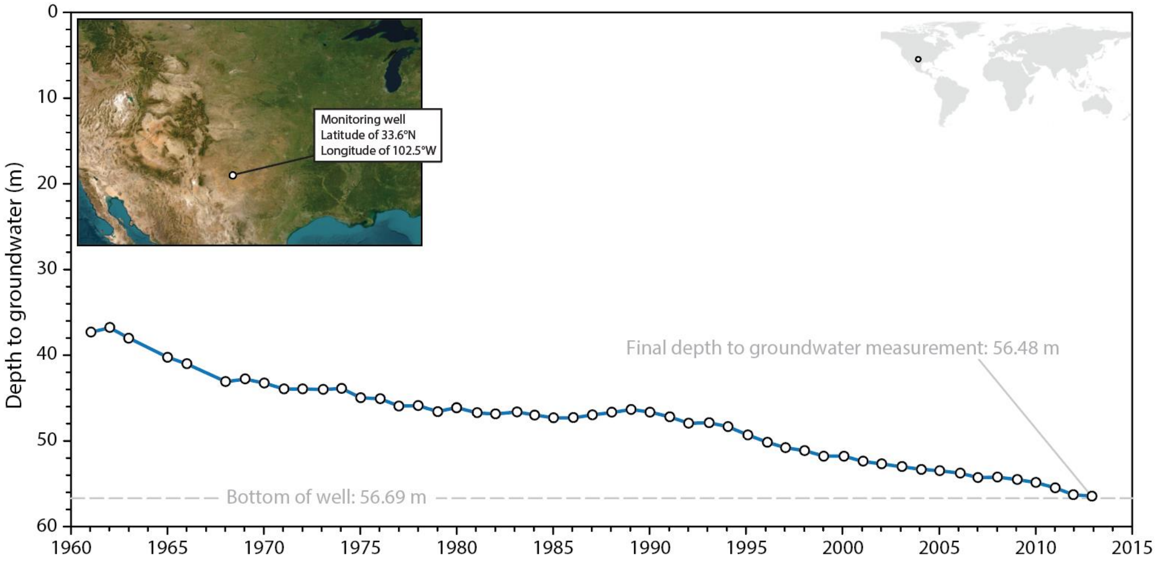

Supplementary Fig. 289. Groundwater level time series for a monitoring well located ~15 km west of Levelland, Texas (USA; see inset maps in top right (global) and top right (regional) for the location of the well, marked by a white circle). Each circle represents one groundwater level measurement. The grey horizontal dashed line near the bottom of the plot represents the recorded bottom of the monitoring well. Groundwater levels were recorded to be 37 m below the land surface in the early 1960s. However, groundwater levels declined from 1961-2012 at a rate of ~0.3 m/year. The final measurement of depth to groundwater in the time series is 56.48 m and, and the total depth of the monitoring well is just marginally deeper (56.69 m); given the chronic groundwater level deepening observed over this 50-year time series, there is a high likelihood that this monitoring well ran dry following the year 2012. Imagery from https://www.arcgis.com/home/item.html?id=10df2279f9684e4a9f6a7f08febac2a9

The time series presented in the figure above exemplifies the potential for monitoring wells to run dry. However, we stress that cases such as the time series depicted above (where the monitoring well may have run dry) are rare in our dataset. We find that there are only 924 wells (out of 72,403 wells with depth data) where (a) the deepest annual median groundwater level fell to within 5 m of the well bottom for at least one year during the 21st century, and (b) the 21st century trend in depth to groundwater exceeded 0.5 m/year. Given that such cases are rare, it is unlikely that a substantial share of monitoring wells were perennially desiccated at a point during the 21st century. Thus, although we lack data describing the exact proportion of monitoring wells that may have run dry, the groundwater level time series and well depth data available to us do not immediately suggest that monitoring well desiccation events are so widespread such that they may substantially alter our main conclusions.

Nevertheless, monitoring well desiccation events will expectedly mean that our results will, if anything, tend to under-represent groundwater level deepening (because monitoring wells that run dry are likely to be disproportionately common in areas where groundwater levels are declining). Therefore, our main conclusions surrounding the prevalence of rapid and accelerating groundwater level declines are likely to be, if anything, conservative with respect to monitoring well desiccation events.

21 INTRA-ANNUAL GROUNDWATER LEVEL VARIATIONS

Our main results are based on annual median groundwater levels. However, we stress that some aquifers—particularly shallow aquifers that have the potential to receive considerable seasonal recharge—may have water level time series that exhibit extensive drawdown during one season, only to be refilled (or partly refilled) during another season. To exemplify such a case, we present a groundwater level time series for a monitoring well located in the Bengal Basin (South Asia; figure below; data from ref.[21] (i.e., Shamsudduha, M. et. al. The Bengal Water Machine: Quantified freshwater capture in Bangladesh. Science **377,** 1315-1319 (2022))). The groundwater level time series (figure below) displays substantial intra-annual variability, reflecting seasonal (i.e., dry season vs. wet season) differences in groundwater withdrawals and recharge. From 1980-1990, intra-annual groundwater variations averaged 4.6 m (i.e., as determined by the difference between the shallowest and the deepest groundwater level measured for any given calendar year). After the year 2000, intra-annual groundwater variations averaged 10.7 m. The larger intra-annual variations after the year 2000 suggest (i) that it is likely that annual groundwater extractions have increased in more recent years, and (ii) that groundwater recharge rates have also likely increased over time, as evidenced by the high-magnitude groundwater rises that occur each year, leading groundwater levels to approach levels observed prior to the year 2000. Further, groundwater level trend analyses for the deepest groundwater levels observed in a given calendar year yield a steeper slope (light pink circles; 0.44 m/year) than a trend based on the shallowest groundwater levels observed in a given calendar year (light blue circles; 0.17 m/year), further highlighting the increasing

seasonal variability over time and how groundwater levels partly recover on a seasonal time step.

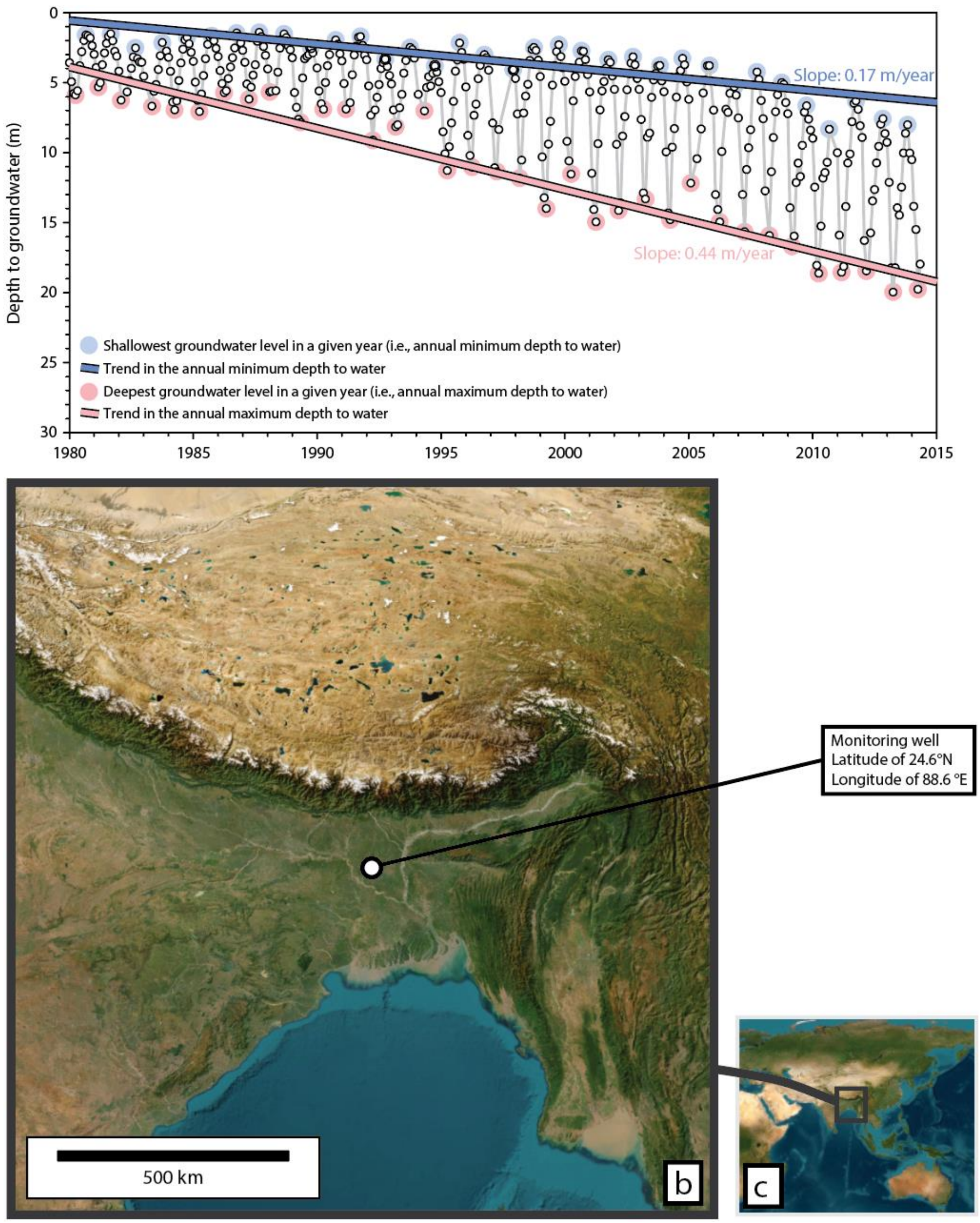

Supplementary Fig. 290. Groundwater level time series for a monitoring well in the Bengal Basin. (a) Observed depth to groundwater levels for a monitoring well from 1980-2015. White circles represent individual measurements. Circles surrounded by a blue halo are the annual minimum depth to water (i.e., the shallowest groundwater level for a given calendar year). Circles surrounded by a red halo are the annual maximum depth to water (i.e., the deepest groundwater level for a given calendar year). Two lines represent the trends in depth to groundwater; the red line represents the trend in the annual maximum depth to water, and the blue line represents the trend in the annual minimum depth to water. Overall, intra-annual groundwater level variability has increased over time. (b) The location of the monitoring well (white circle) in the Bengal Basin. (c) Inset map displaying the extent of the map in panel b (see map in lower right corner). Imagery from https://www.arcgis.com/home/item.html?id=10df2279f9684e4a9f6a7f08febac2a9

22 WELL DRILLING TIME SERIES IN AQUIFER SYSTEMS EXHIBITING RAPID GROUNDWATER LEVEL DEEPENING

Some previous studies have examined statistical relationships between time series of well completion or permitting events (i.e., the number of wells in a given year) and trends in depth to groundwater (see, for example, Fig. 9 within ref.[108] Babaee, S., Mousavi, Z., Masoumi, Z., Malekshah, A.H., Roostaei, M., Aflaki, M. Land subsidence from interferometric SAR and groundwater patterns in the Qazvin plain, Iran. *International Journal of Remote Sensing* **41,** 4780-4798 (2020)). Other works have evaluated changes over time in the number of wells (for example, refs.[1370,1371] (Mukherji, A., Rawat, S., & Shah, T. (2013). Major insights from India's minor irrigation censuses: 1986-87 to 2006-07. *Economic and Political Weekly* 115-124 (2013). and, Laghari, A. N., Vanham, D., & Rauch, W. The Indus basin in the framework of current and future water resources management. *Hydrology and Earth System Sciences* **16,** 1063-1083 (2012)) show that the number of groundwater structures has increased over time in parts of South Asia). Here we present time series of the number of recorded well completion events over time for a subset of aquifer systems where (i) aquifer-scale trends in depth to groundwater exceeding 0.5 m/year (i.e., deepening faster than 0.5 m/year), and (ii) a well completion event was recorded in at least 15 unique years during the 21st century in a database of global well completion events (database of well completion data described within the extensive Supplementary Materials of ref.[17]: Jasechko, S., Perrone, D. Global groundwater wells at risk of running dry. *Science* **372,** 418-421. doi.org/10.1126/science.abc2755 (2021)).

Twenty-four (n=24) aquifer systems meet both of these criteria (i.e., "(i)" and "(ii)" described in the preceding paragraph). The following figures present time series of the total number of recorded well completion events for each of these aquifer systems.

We stress that the absolute number of wells should not be compared directly across different aquifer systems presented in the plots presented in this supplementary note because the underlying databases have differing degrees of completeness (for example, a survey we completed of well construction databases among US state agencies suggests wide variability in the number of actual wells that are recorded in state databases, see responses from groundwater well completion database managers to the inquiry "How representative are the wells?" presented in Table S1 within the Supplementary Information of ref.[1368]: Perrone, D., Jasechko, S. Deeper well drilling an unsustainable stopgap to groundwater depletion. *Nature Sustainability* **2,** 773-782 (2019)). Therefore, we caution that changes in the recorded number of well completion events may be, to some degree, impacted by changes over time in the strategies and thoroughness of well construction data compilation (e.g., in Nebraska (US) domestic wells were exempt from inclusion in the state well completion database prior to the year 1993, see section S2.2 within the Supplementary Data of ref.[1367]: Perrone, D., Jasechko, S. Dry groundwater wells in the western United States. *Environmental Research Letters* **12,** 104002 (2017)). Nevertheless, these cumulative 21st-century well completion time series (supplementary figures on the following pages) highlight that there was demand for the construction of new wells designed to extract groundwater throughout the early 21st century in all n=24 of the aquifer systems exhibiting deepening aquifer-scale trends in depth to groundwater.

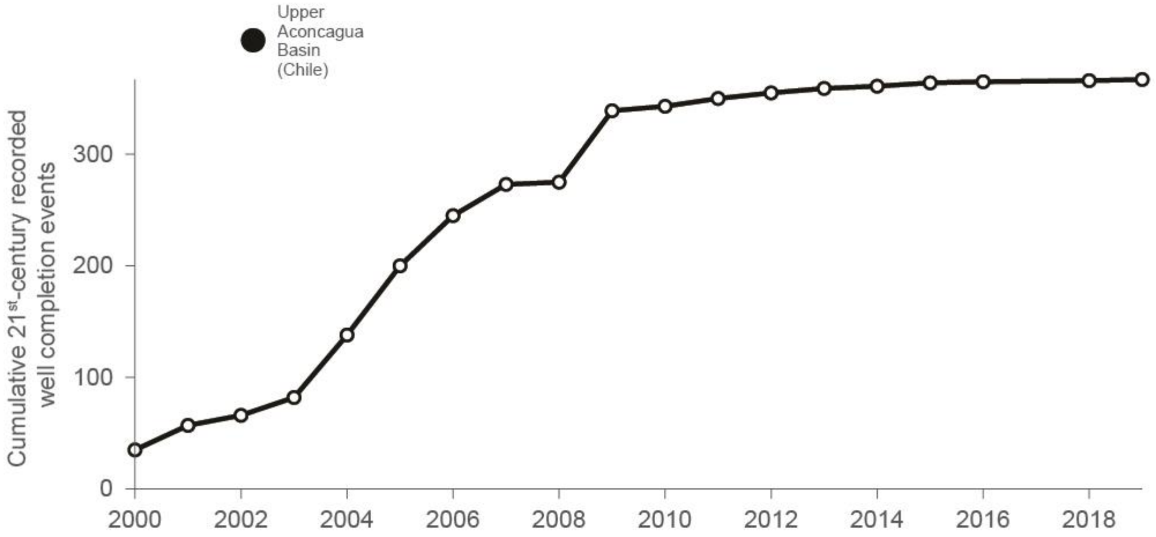

Supplementary Fig. 291. 21st-century recorded well completion events for the Upper Aconcagua Basin. Y-axis values are the cumulative number of recorded well completion events (during the 21st century). X-axis values represent calendar year. The title of the aquifer system is displayed at the top of the figure.

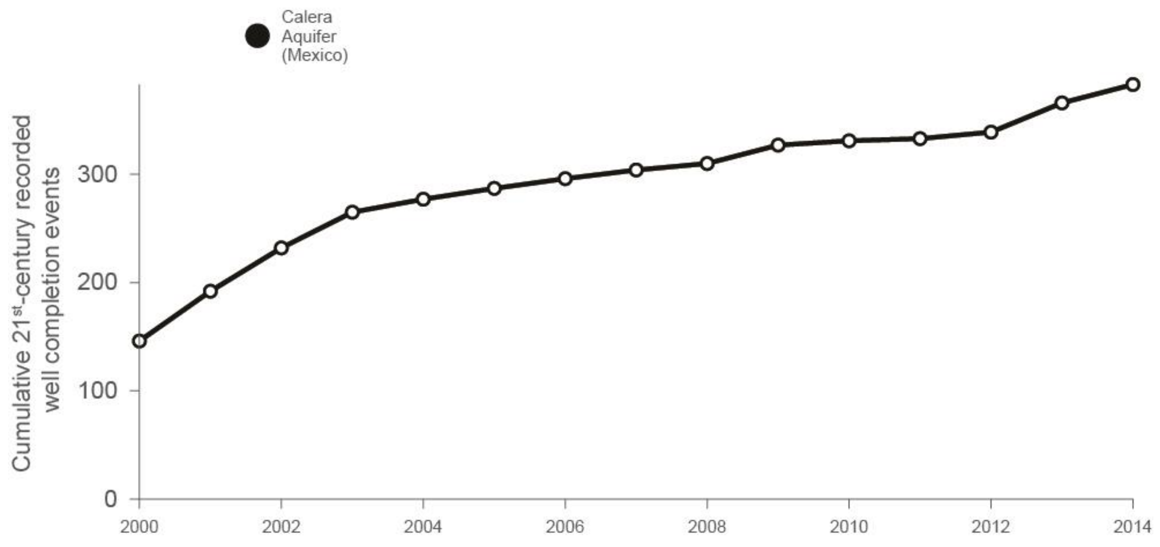

Supplementary Fig. 292. 21st-century recorded well completion events for the Calera Aquifer. Y-axis values are the cumulative number of recorded well completion events (during the 21st century). X-axis values represent calendar year. The title of the aquifer system is displayed at the top of the figure.

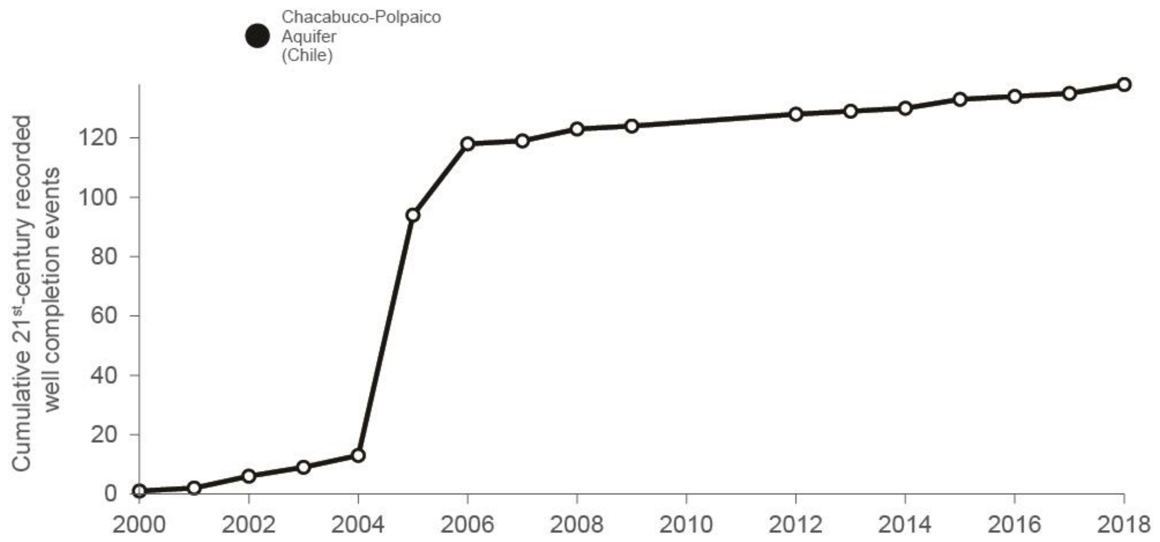

Supplementary Fig. 293. 21st-century recorded well completion events for the Chacabuco-Polpaico Aquifer. Y-axis values are the cumulative number of recorded well completion events (during the 21st century). X-axis values represent calendar year. The title of the aquifer system is displayed at the top of the figure.

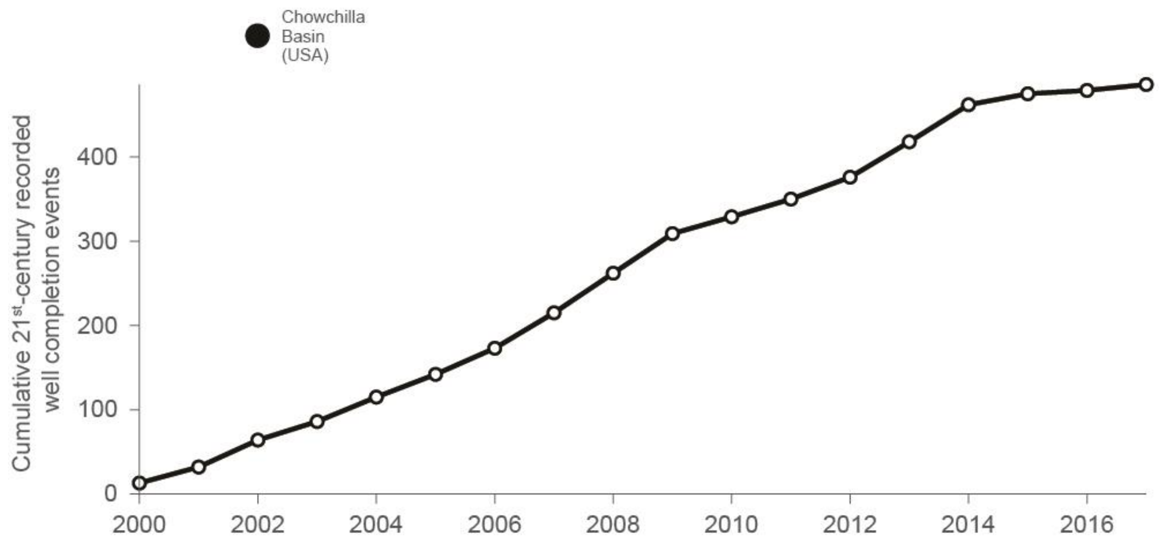

Supplementary Fig. 294. 21st-century recorded well completion events for the Chowchilla Basin (California Central Valley). Y-axis values are the cumulative number of recorded well completion events (during the 21st century). X-axis values represent calendar year. The title of the aquifer system is displayed at the top of the figure.

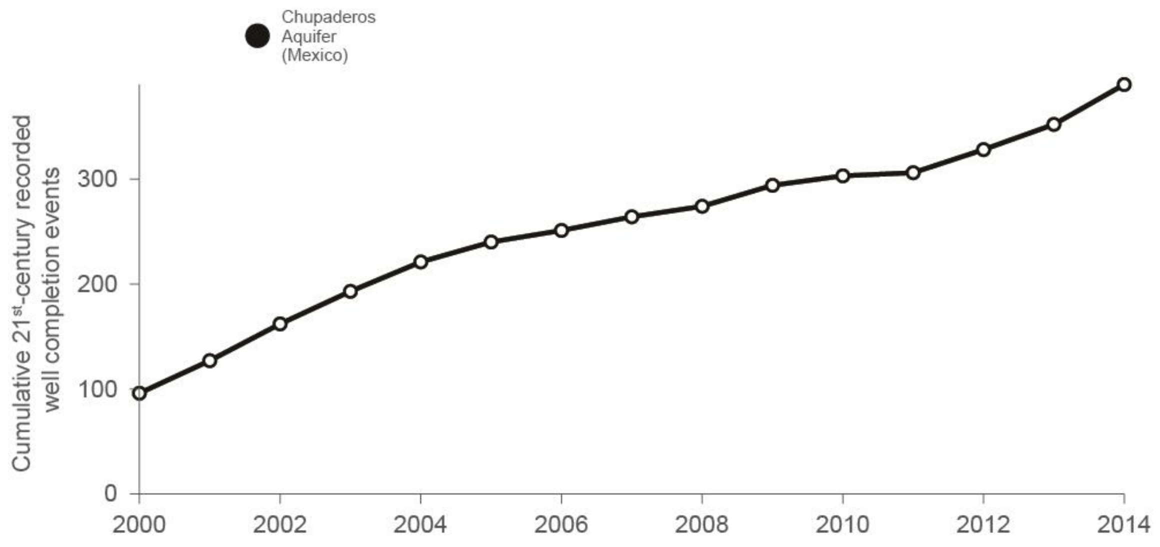

Supplementary Fig. 295. 21st-century recorded well completion events for the Chupaderos Aquifer. Y-axis values are the cumulative number of recorded well completion events (during the 21st century). X-axis values represent calendar year. The title of the aquifer system is displayed at the top of the figure.

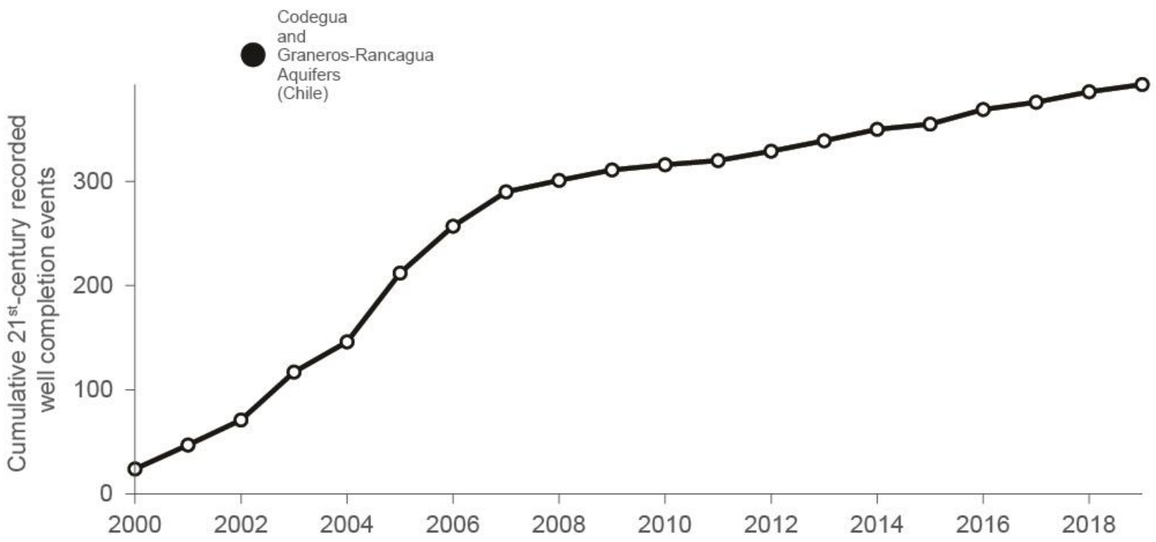

Supplementary Fig. 296. 21st-century recorded well completion events for the Codegua and Graneros-Rancagua Aquifers. Y-axis values are the cumulative number of recorded well completion events (during the 21st century). X-axis values represent calendar year. The title of the aquifer system is displayed at the top of the figure.

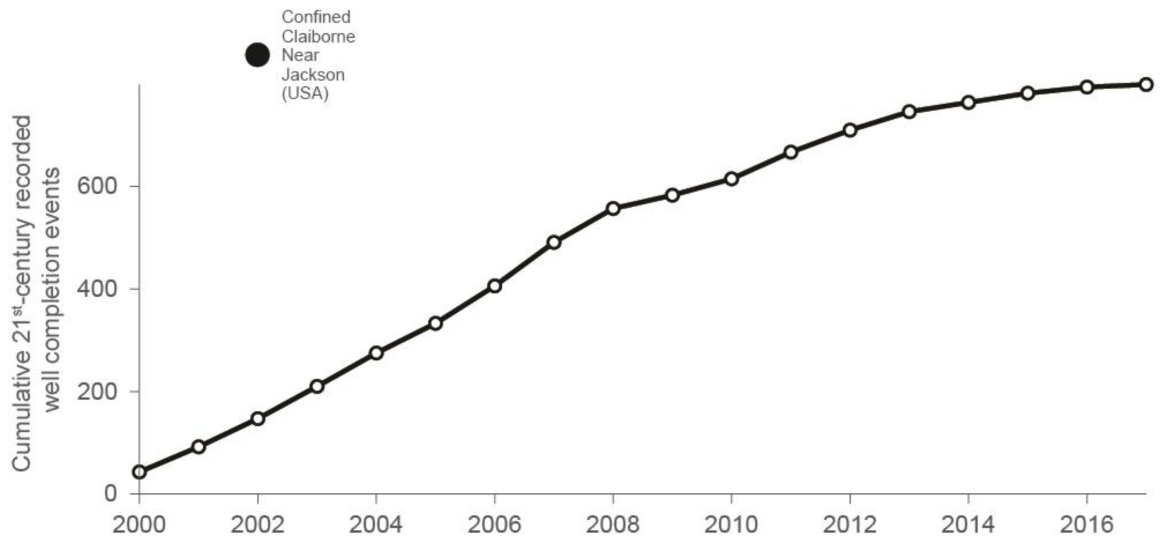

Supplementary Fig. 297. 21st-century recorded well completion events for the Confined Claiborne Near Jackson. Y-axis values are the cumulative number of recorded well completion events (during the 21st century). X-axis values represent calendar year. The title of the aquifer system is displayed at the top of the figure.

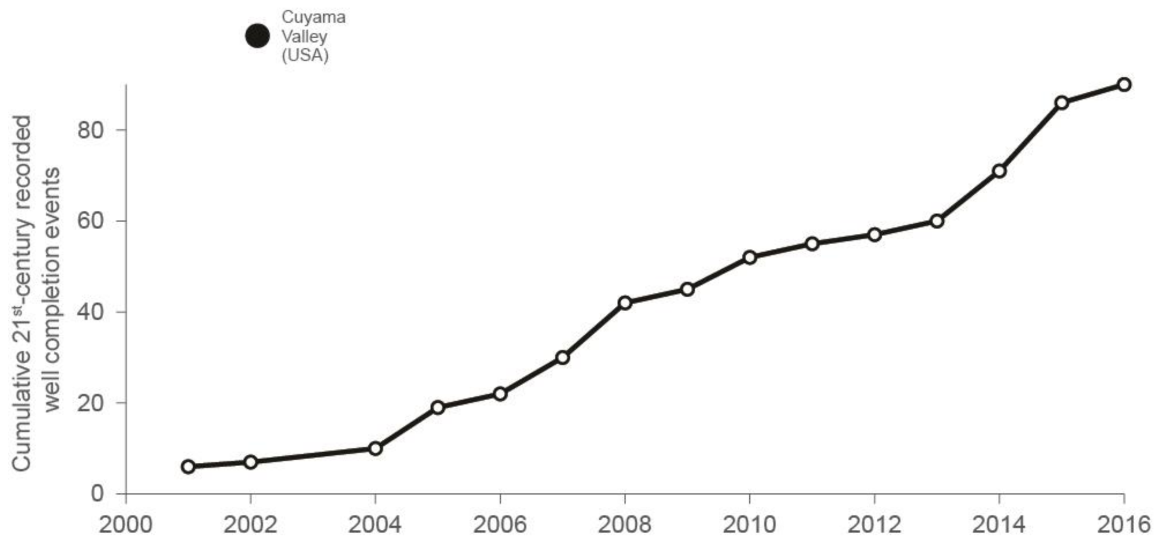

Supplementary Fig. 298. 21st-century recorded well completion events for the Cuyama Valley. Y-axis values are the cumulative number of recorded well completion events (during the 21st century). X-axis values represent calendar year. The title of the aquifer system is displayed at the top of the figure.

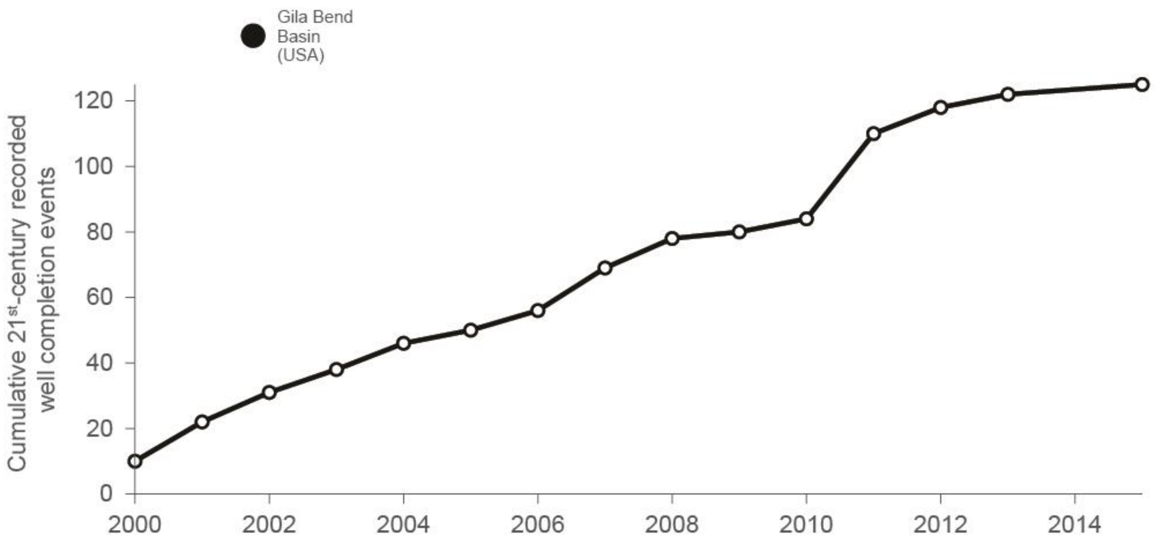

Supplementary Fig. 299. 21st-century recorded well completion events for the Gila Bend Basin. Y-axis values are the cumulative number of recorded well completion events (during the 21st century). X-axis values represent calendar year. The title of the aquifer system is displayed at the top of the figure.

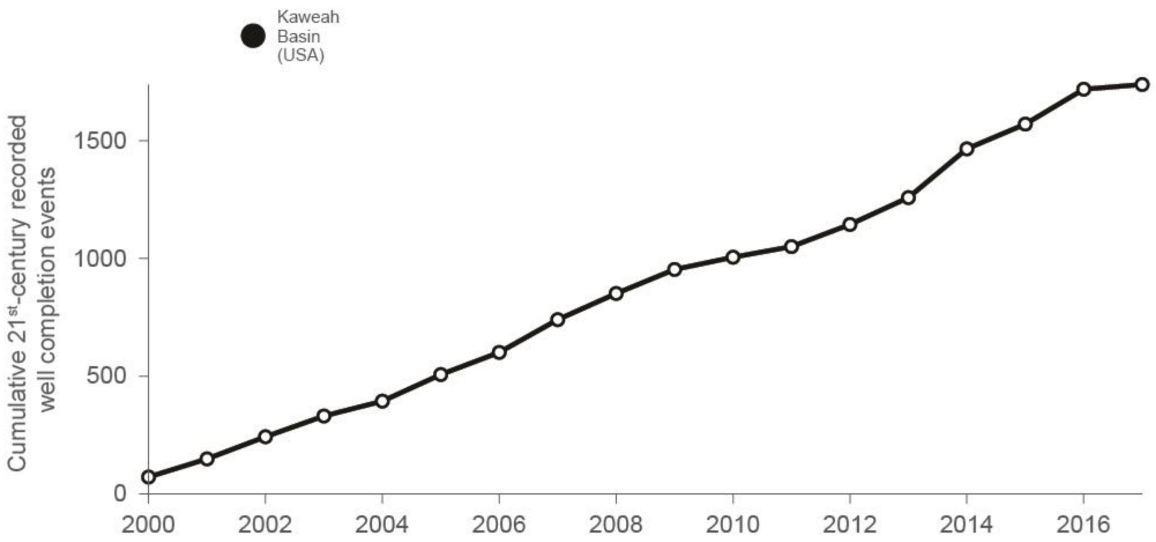

Supplementary Fig. 300. 21st-century recorded well completion events for the Kaweah Basin. Y-axis values are the cumulative number of recorded well completion events (during the 21st century). X-axis values represent calendar year. The title of the aquifer system is displayed at the top of the figure.

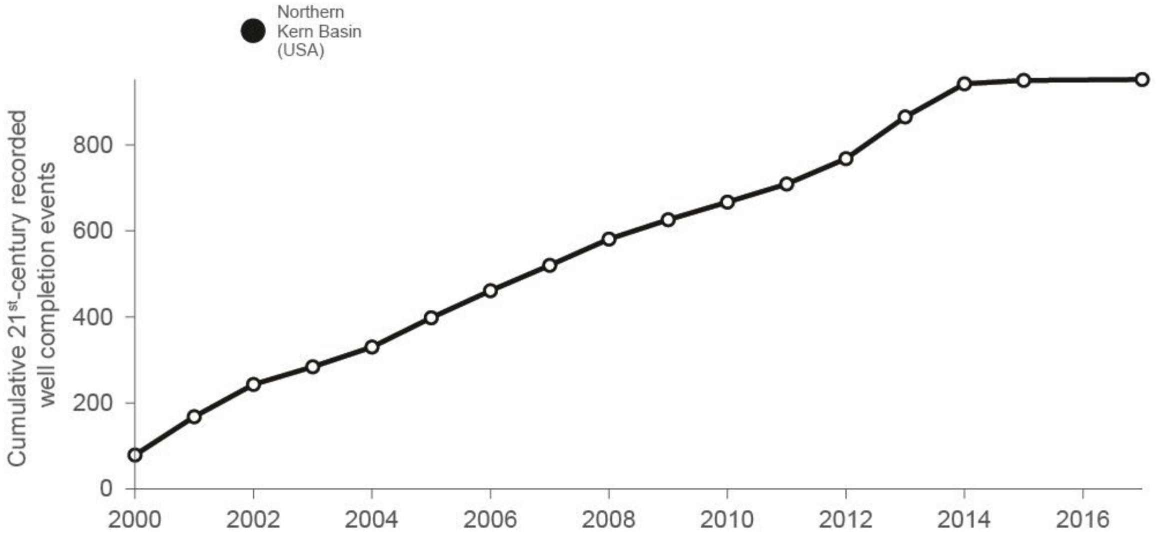

Supplementary Fig. 301. 21st-century recorded well completion events for the Northern Kern Basin. Y-axis values are the cumulative number of recorded well completion events (during the 21st century). X-axis values represent calendar year. The title of the aquifer system is displayed at the top of the figure.

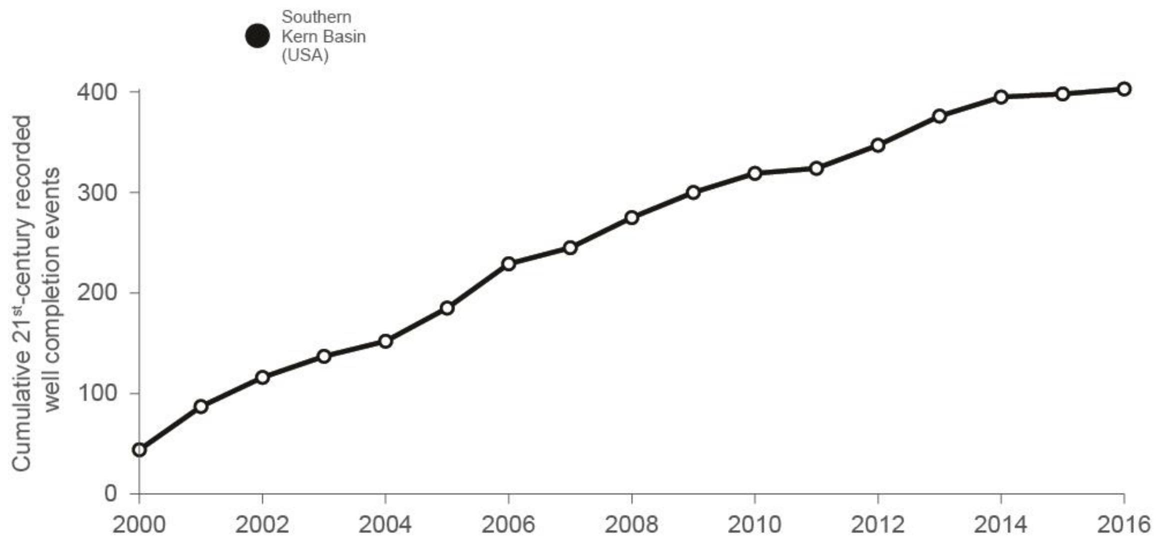

Supplementary Fig. 302. 21st-century recorded well completion events for the Southern Kern Basin. Y-axis values are the cumulative number of recorded well completion events (during the 21st century). X-axis values represent calendar year. The title of the aquifer system is displayed at the top of the figure.

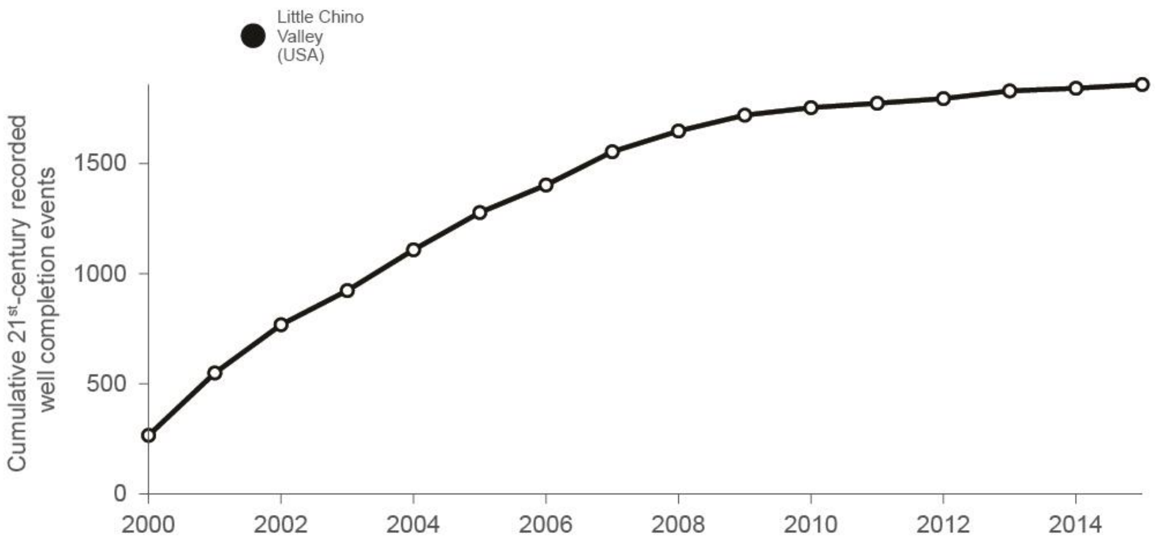

Supplementary Fig. 303. 21st-century recorded well completion events for the Little Chino Valley. Y-axis values are the cumulative number of recorded well completion events (during the 21st century). X-axis values represent calendar year. The title of the aquifer system is displayed at the top of the figure.

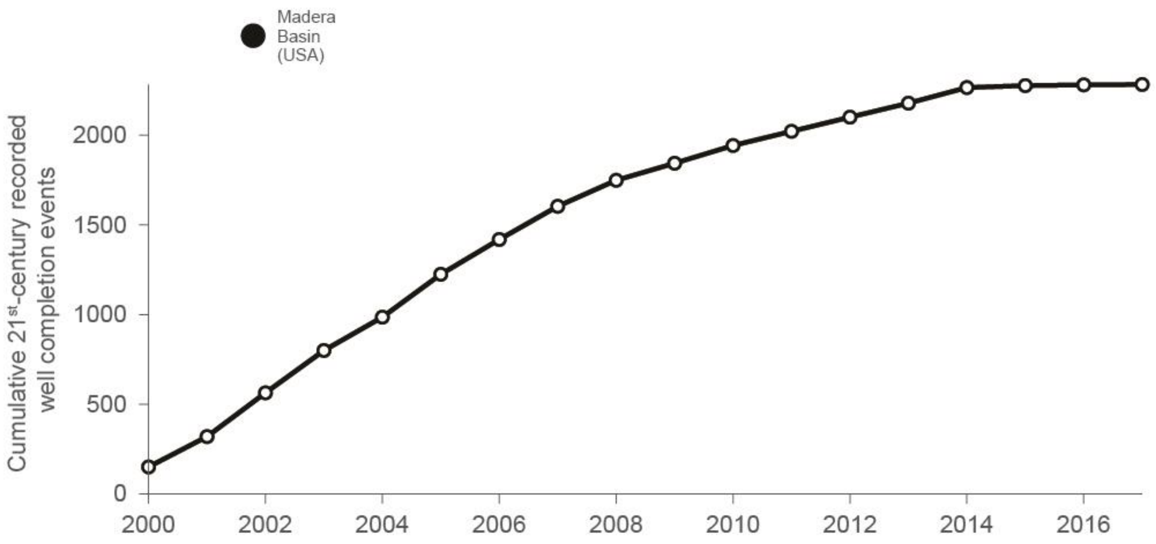

Supplementary Fig. 304. 21st-century recorded well completion events for the Madera Basin. Y-axis values are the cumulative number of recorded well completion events (during the 21st century). X-axis values represent calendar year. The title of the aquifer system is displayed at the top of the figure.

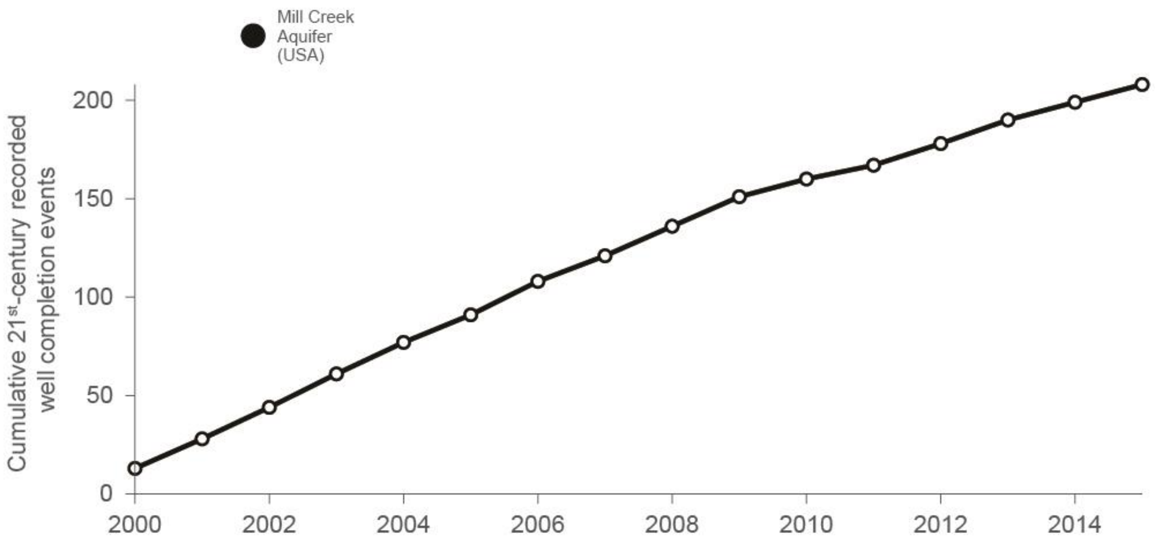

Supplementary Fig. 305. 21st-century recorded well completion events for the Mill Creek Aquifer. Y-axis values are the cumulative number of recorded well completion events (during the 21st century). X-axis values represent calendar year. The title of the aquifer system is displayed at the top of the figure.

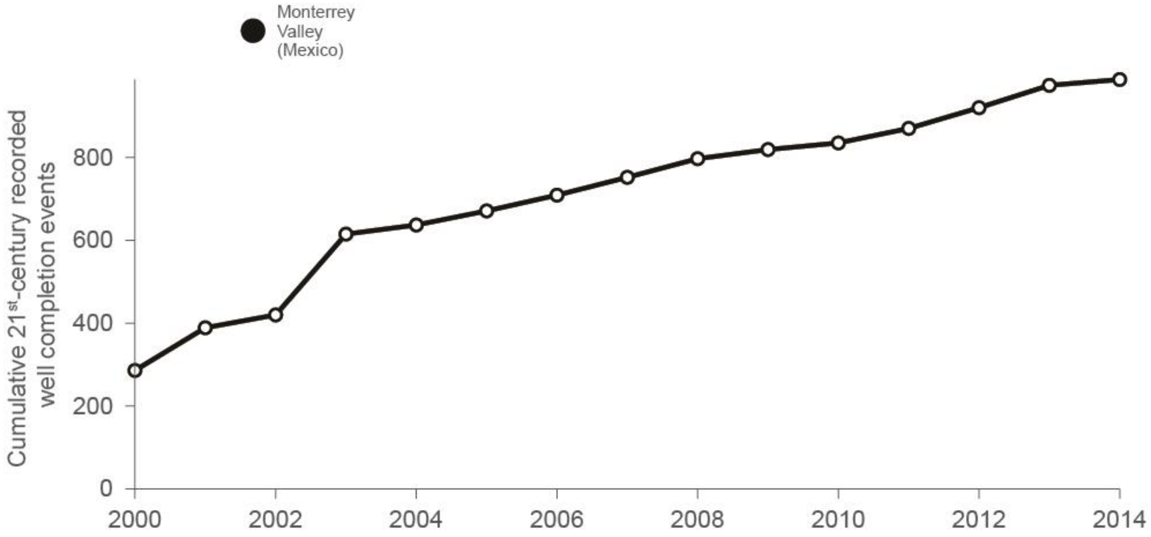

Supplementary Fig. 306. 21st-century recorded well completion events for the Monterrey Valley. Y-axis values are the cumulative number of recorded well completion events (during the 21st century). X-axis values represent calendar year. The title of the aquifer system is displayed at the top of the figure.

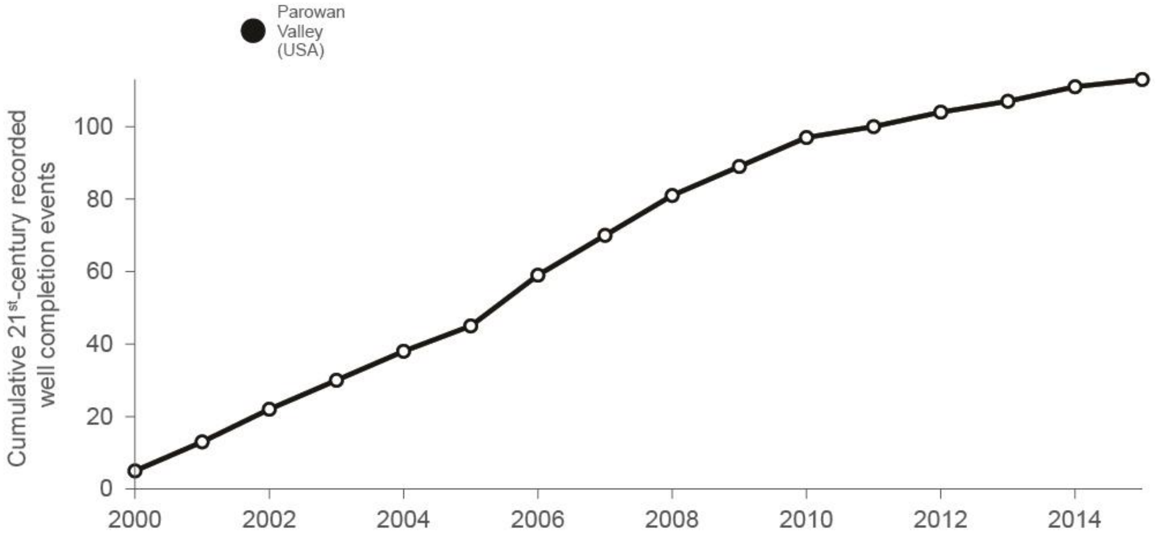

Supplementary Fig. 307. 21st-century recorded well completion events for the Parowan Valley. Y-axis values are the cumulative number of recorded well completion events (during the 21st century). X-axis values represent calendar year. The title of the aquifer system is displayed at the top of the figure.

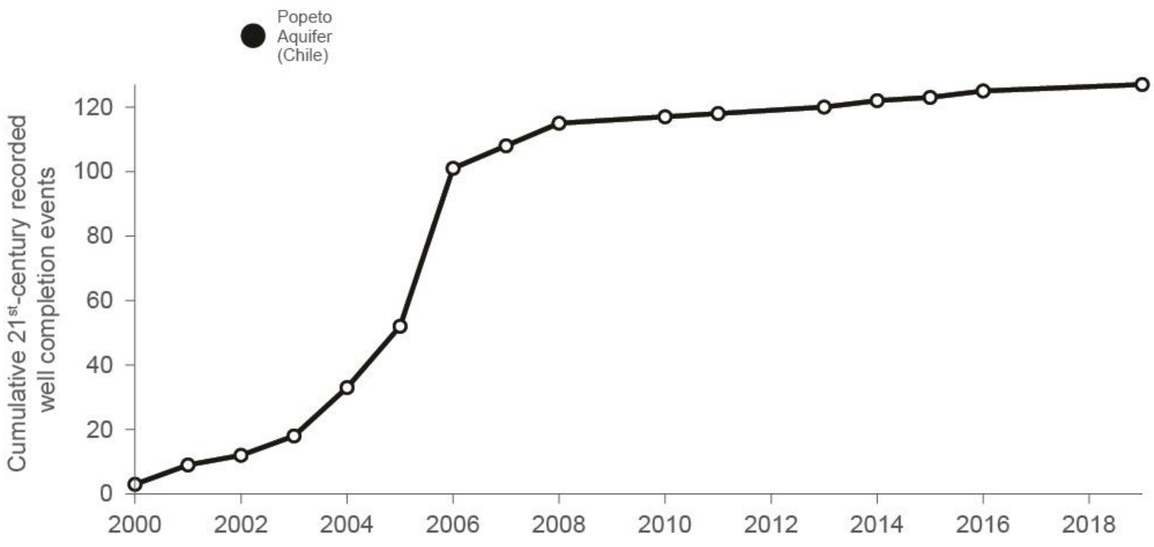

Supplementary Fig. 308. 21st-century recorded well completion events for the Popeto Aquifer. Y-axis values are the cumulative number of recorded well completion events (during the 21st century). X-axis values represent calendar year. The title of the aquifer system is displayed at the top of the figure.

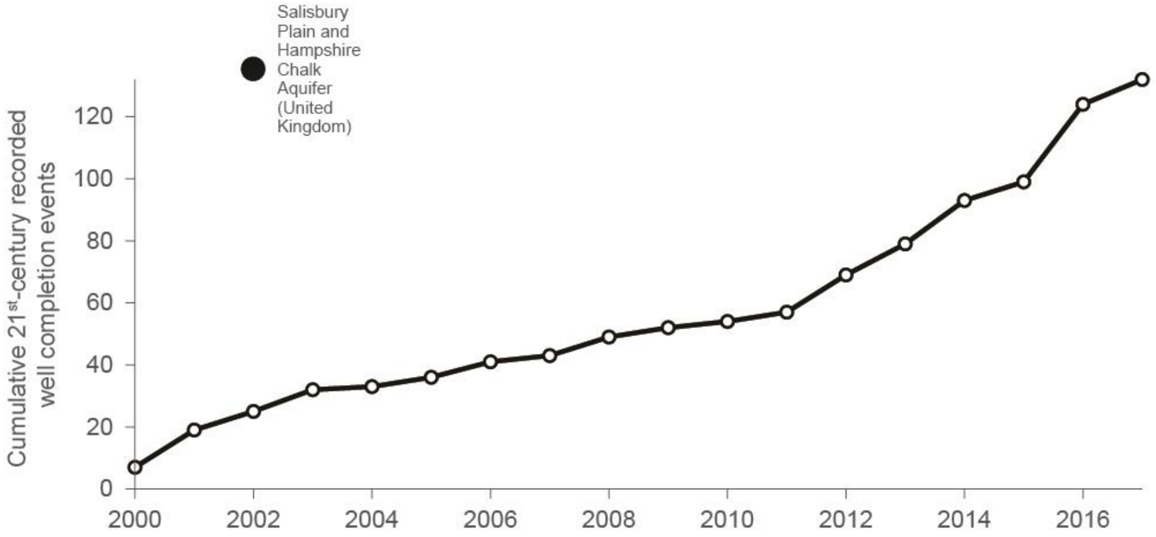

Supplementary Fig. 309. 21st-century recorded well completion events for the Salisbury Plain and Hampshire Chalk Aquifer. Y-axis values are the cumulative number of recorded well completion events (during the 21st century). X-axis values represent calendar year. The title of the aquifer system is displayed at the top of the figure.

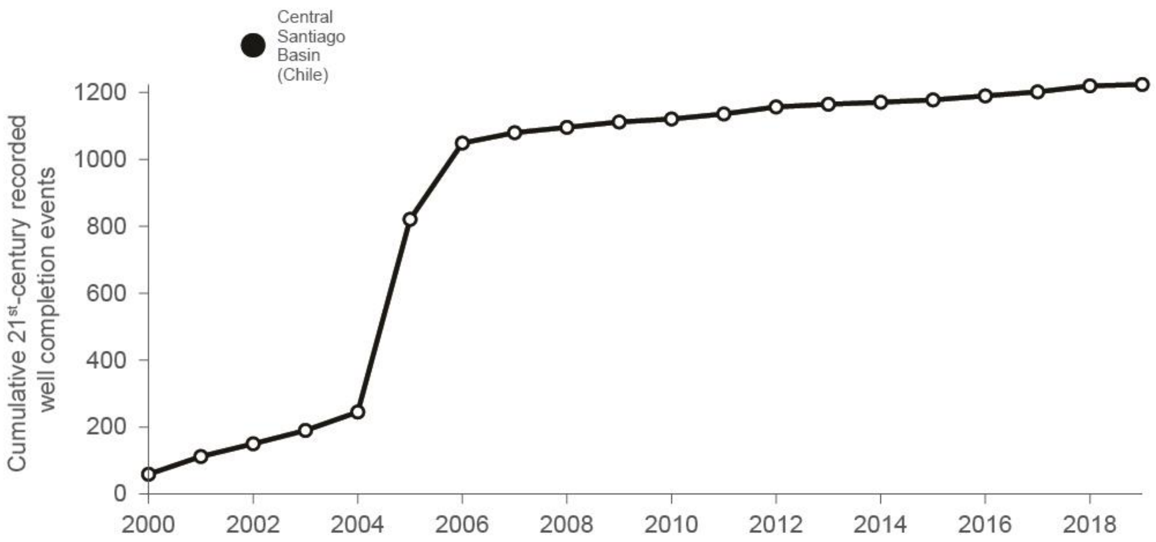

Supplementary Fig. 310. 21st-century recorded well completion events for the Central Santiago Basin. Y-axis values are the cumulative number of recorded well completion events (during the 21st century). X-axis values represent calendar year. The title of the aquifer system is displayed at the top of the figure.

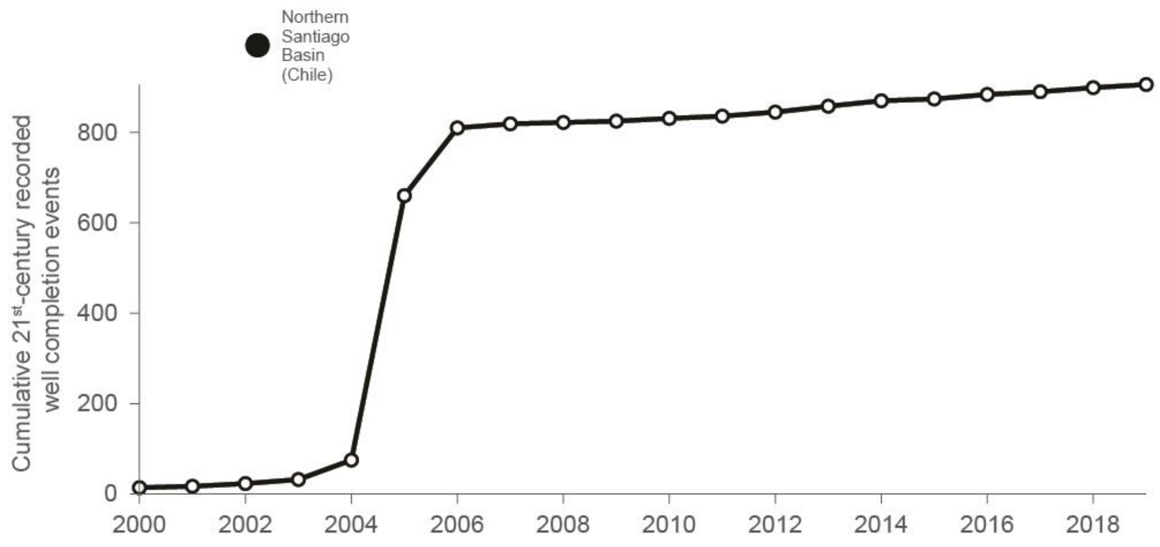

Supplementary Fig. 311. 21st-century recorded well completion events for the Northern Santiago Basin. Y-axis values are the cumulative number of recorded well completion events (during the 21st century). X-axis values represent calendar year. The title of the aquifer system is displayed at the top of the figure.

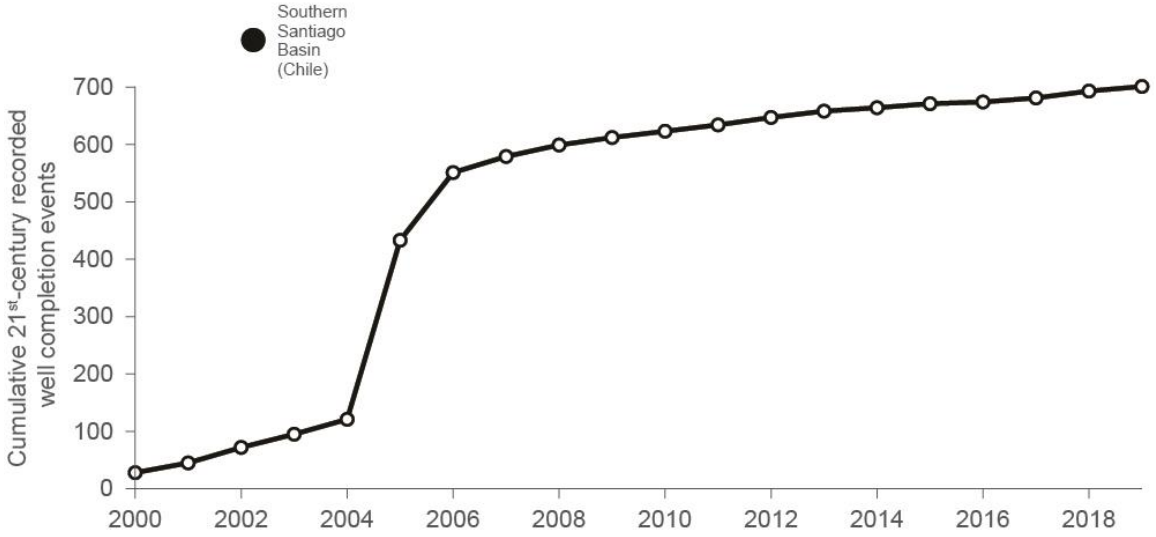

Supplementary Fig. 312. 21st-century recorded well completion events for the Southern Santiago Basin. Y-axis values are the cumulative number of recorded well completion events (during the 21st century). X-axis values represent calendar year. The title of the aquifer system is displayed at the top of the figure.

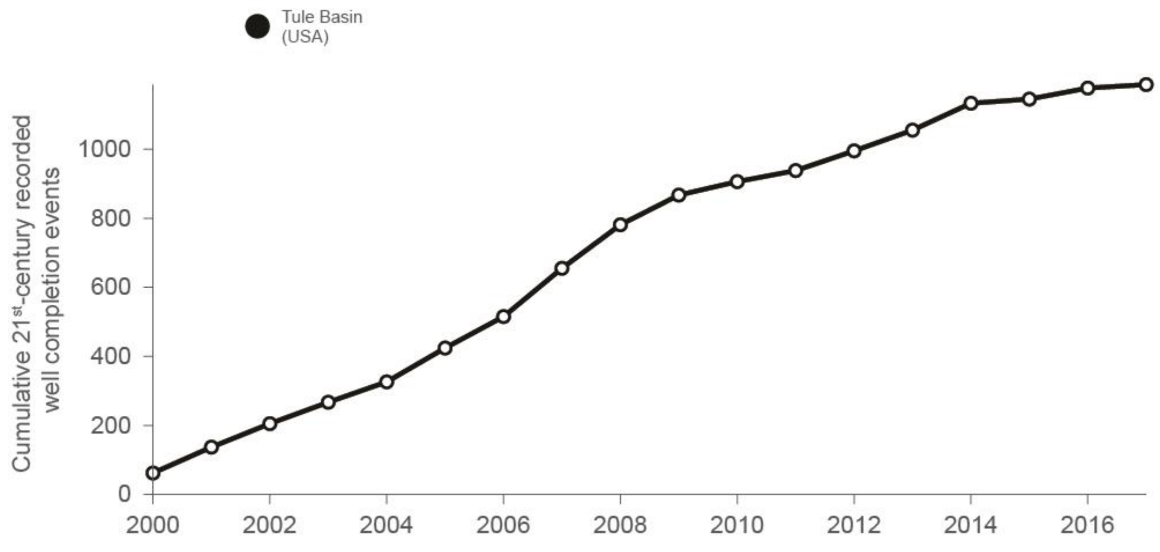

Supplementary Fig. 313. 21st-century recorded well completion events for the Tule Basin. Y-axis values are the cumulative number of recorded well completion events (during the 21st century). X-axis values represent calendar year. The title of the aquifer system is displayed at the top of the figure.

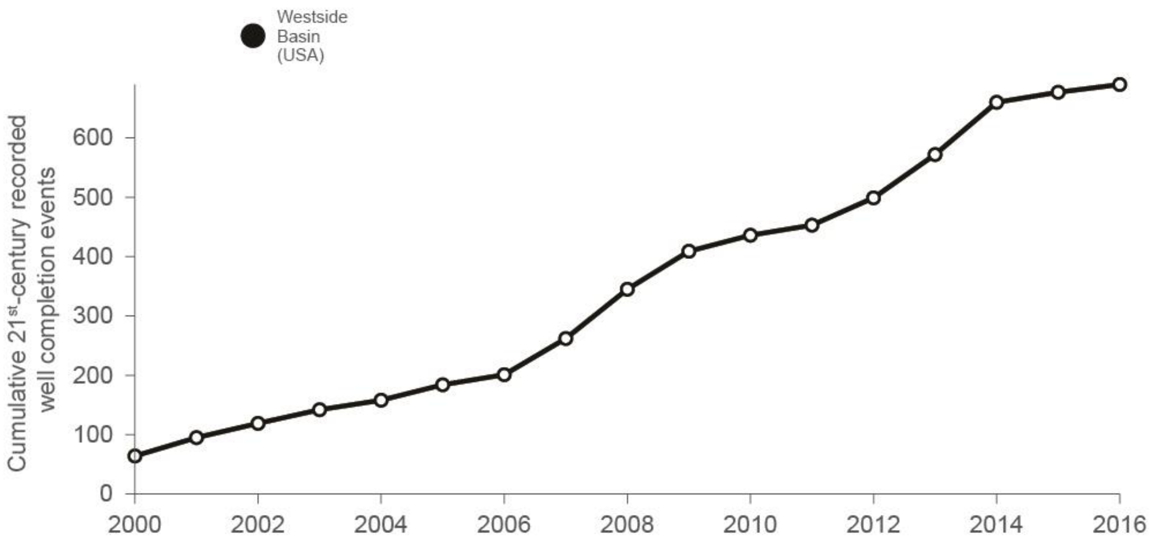

Supplementary Fig. 314. 21st-century recorded well completion events for the Westside Basin. Y-axis values are the cumulative number of recorded well completion events (during the 21st century). X-axis values represent calendar year. The title of the aquifer system is displayed at the top of the figure.

23 CLUSTERING OF GROUNDWATER-LEVEL TRENDS WITHIN INDIVIDUAL AQUIFER SYSTEMS

We examined calculated Moran's I to characterize the degree to which similar groundwater-level trends cluster in 2D space (within the boundaries of individual aquifer systems). First, we identified aquifer systems with at least 30 monitoring wells, which is the recommended minimum number of features (see "Best practice guidelines" available at https://pro.arcgis.com/en/pro-app/latest/tool-reference/spatial-statistics/h-how-spatial-autocorrelation-moran-s-i-spatial-st.htm (accessed August 3, 2023)). Next, we calculated Moran's I values for each aquifer and their associated P-values (see table below).

Supplementary Table 15. Statistical variability in Moran's I values among the 594 aquifer systems that contain at least 30 monitoring wells meeting our criteria for analysis

	**Moran's I < 0**	**Moran's I > 0**
**All aquifer systems with at least 30 wells (N=594 aquifer systems)**	N=101 of N=594<br>(17% of aquifer systems)	N=493 of N=594<br>(83% of aquifer systems)
**All aquifer systems with at least 30 wells *and* Moran's I P-value of less than 0.01 (N=336 aquifer systems)**	N=2 of N=336<br>(0.6% of aquifer systems)	N=334 of N=336<br>(99.4% of aquifer systems)

Our calculations suggest that groundwater-level trends exhibit some degree of clustering in many of our aquifer systems (e.g., Moran's I P-value of < 0.01 *and* Moran's I value of > 0 in 334 of 594 aquifer systems; see table above). In cases where the Moran's I P-value is less than zero (i.e., 334 of 594 aquifer systems), nearly all (99.4%) Moran's I values are greater than zero (consistent with a tendency towards clustering, rather than dispersion, of groundwater-level trends).

23.1 EXAMPLES OF AQUIFER SYSTEMS EXHIBITING CLUSTERING OF TRENDS IN DEPTH TO GROUNDWATER

The following maps present a series of aquifer systems exhibiting some clustering of groundwater-level trends (i.e., aquifer systems with significant (i.e., P-value < 0.01) Moran's I values that exceed zero).

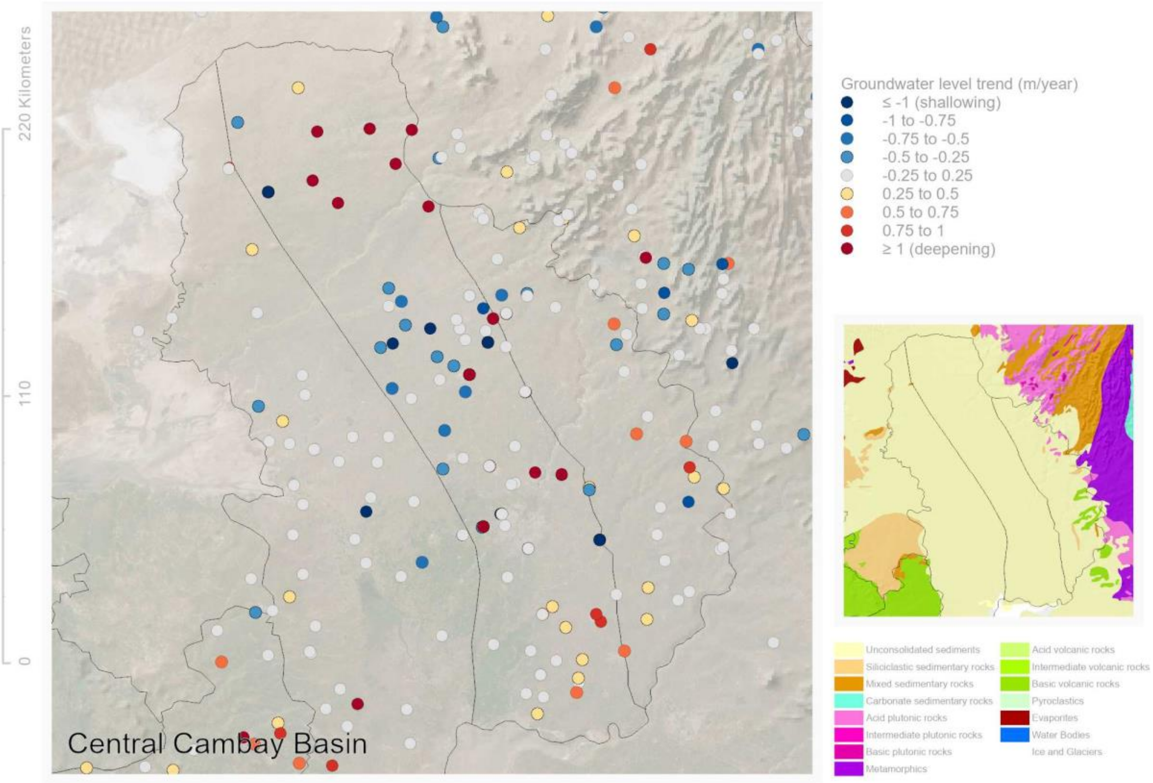

Supplementary Fig. 315. 21st-century trends in depth to groundwater in the Central Cambay Basin (South Asia). The groundwater-level trends within this aquifer system (i.e., center of the map above) exhibit some degree of spatial clustering (e.g., red-shaded points tend to be co-located). The largest map (on the left-hand side of the figure) depicts 21st-century trends in depth to groundwater. Each point (coloured circle) represents a monitoring well; the colour of the point corresponds to the 21st-century trend in depth to groundwater for the monitoring well (Theil-Sen slope). Orange and red points represent monitoring wells where groundwater levels became deeper over time (Theil-Sen slope >0.25 m/year). Blue points represent monitoring wells where groundwater levels became shallower over time (Theil-Sen slope <0.25 m/year; see legend in upper-right corner of figure). The boundaries of the aquifer system are outlined in black. The map in the lower-right corner of the figure presents spatial variations in near-surface geology (data from ref.[1360]: Hartmann, J., & Moosdorf, N. (2012). The new global lithological map database GLiM: A representation of rock properties at the Earth surface. Geochemistry, Geophysics, Geosystems 13, doi.org/10.1029/2012GC004370). Imagery from https://www.arcgis.com/home/item.html?id=10df2279f9684e4a9f6a7f08febac2a9

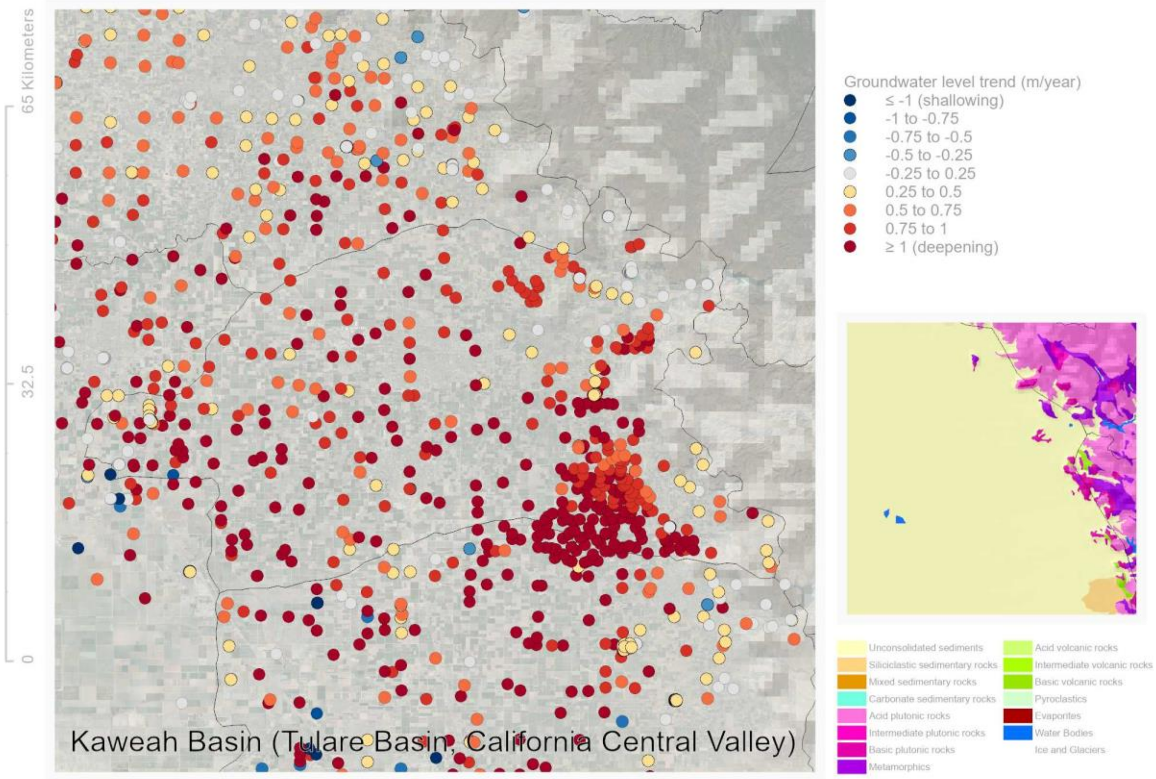

Supplementary Fig. 316. 21st-century trends in depth to groundwater in the Kaweah Basin (western US, within the broader Tulare Basin that is, itself, within the broader California Central Valley aquifer system). The groundwater-level trends within this aquifer system (i.e., center of the map above) exhibit some degree of spatial clustering (e.g., red-shaded points tend to be co-located). The largest map (on the left-hand side of the figure) depicts 21st-century trends in depth to groundwater. Each point (coloured circle) represents a monitoring well; the colour of the point corresponds to the 21st-century trend in depth to groundwater for the monitoring well (Theil-Sen slope). Orange and red points represent monitoring wells where groundwater levels became deeper over time (Theil-Sen slope >0.25 m/year). Blue points represent monitoring wells where groundwater levels became shallower over time (Theil-Sen slope <0.25 m/year; see legend in upper-right corner of figure). The boundaries of the aquifer system are outlined in black. The map in the lower-right corner of the figure presents spatial variations in near-surface geology (data from ref.[1360]: Hartmann, J., & Moosdorf, N. (2012). The new global lithological map database GLiM: A representation of rock properties at the Earth surface. Geochemistry, Geophysics, Geosystems 13, doi.org/10.1029/2012GC004370). Imagery from https://www.arcgis.com/home/item.html?id=10df2279f9684e4a9f6a7f08febac2a9

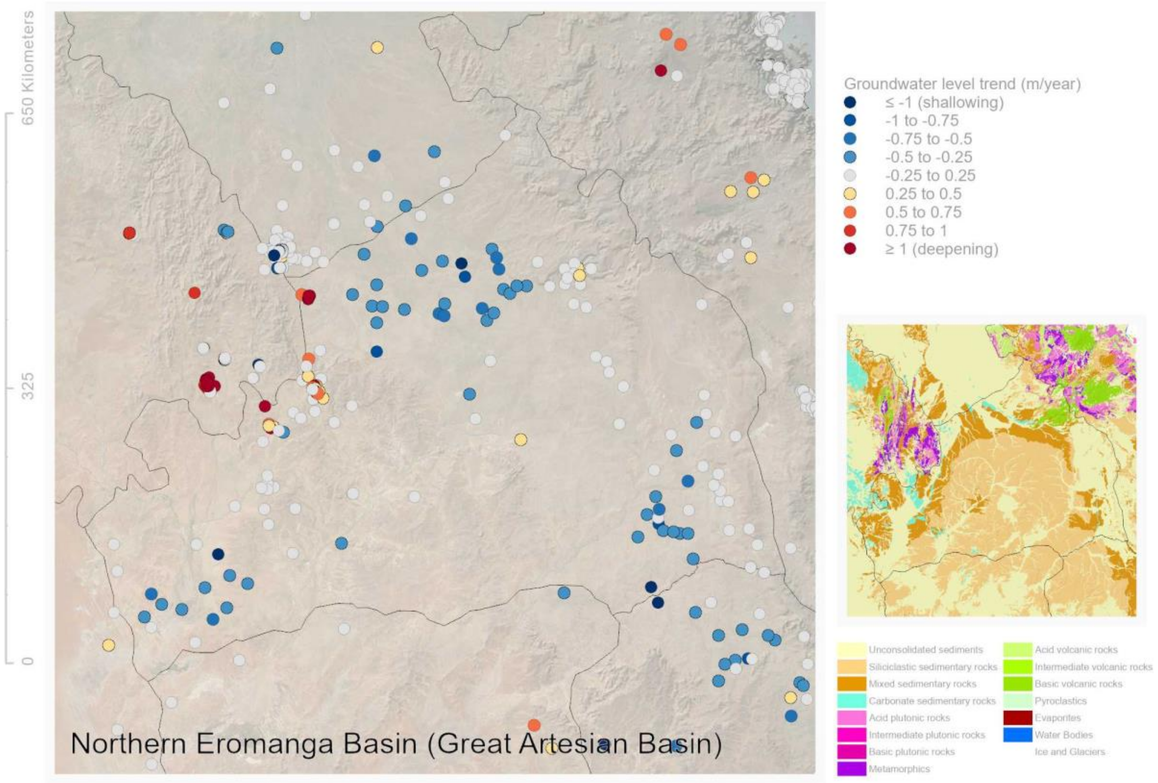

Supplementary Fig. 317. 21st-century trends in depth to groundwater in the Northern Eromanga Basin (Australia; within the broader Great Artesian Basin). The groundwater-level trends within this aquifer system (i.e., center of the map above) exhibit some degree of spatial clustering (e.g., red-shaded points tend to be co-located). The largest map (on the left-hand side of the figure) depicts 21st-century trends in depth to groundwater. Each point (coloured circle) represents a monitoring well; the colour of the point corresponds to the 21st-century trend in depth to groundwater for the monitoring well (Theil-Sen slope). Orange and red points represent monitoring wells where groundwater levels became deeper over time (Theil-Sen slope >0.25 m/year). Blue points represent monitoring wells where groundwater levels became shallower over time (Theil-Sen slope <0.25 m/year; see legend in upper-right corner of figure). The boundaries of the aquifer system are outlined in black. The map in the lower-right corner of the figure presents spatial variations in near-surface geology (data from ref.[1360]: Hartmann, J., & Moosdorf, N. (2012). The new global lithological map database GLiM: A representation of rock properties at the Earth surface. Geochemistry, Geophysics, Geosystems 13, doi.org/10.1029/2012GC004370). Imagery from https://www.arcgis.com/home/item.html?id=10df2279f9684e4a9f6a7f08febac2a9

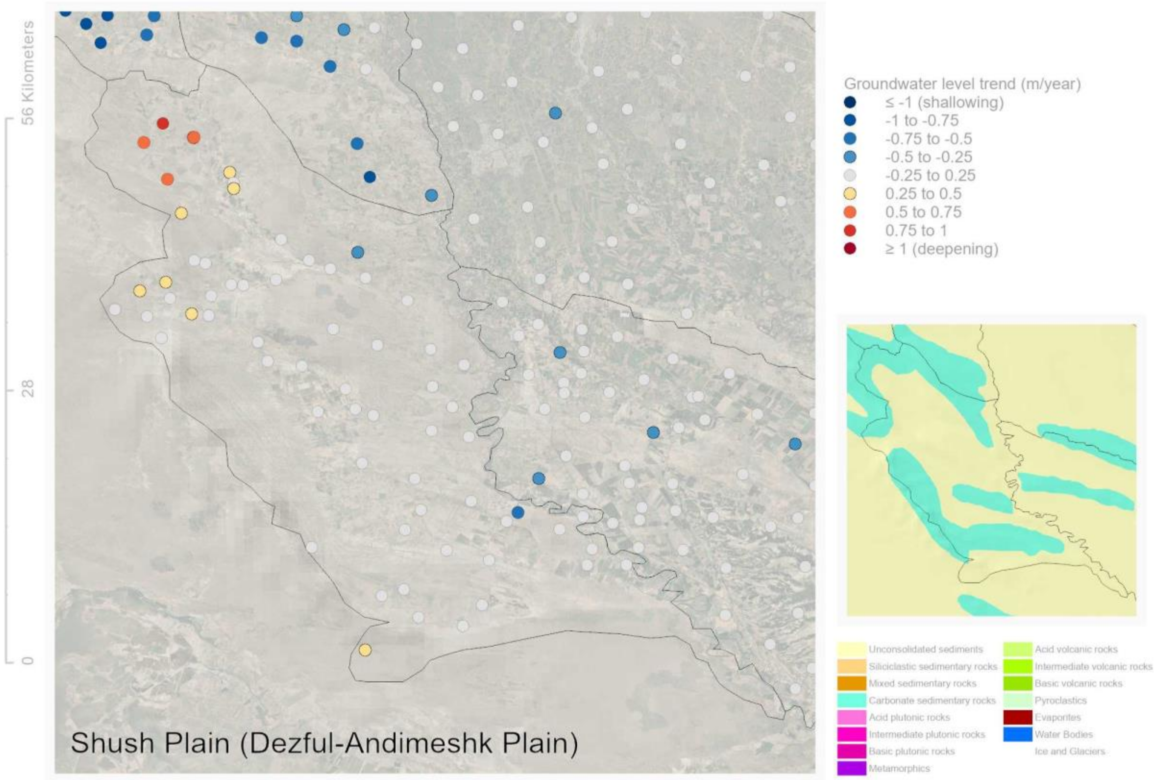

Supplementary Fig. 318. 21st-century trends in depth to groundwater in the Shush Plain (Iran; within the broader Dezful-Andimeshk Plain). The groundwater-level trends within this aquifer system (i.e., center of the map above) exhibit some degree of spatial clustering (e.g., red-shaded points tend to be co-located). The largest map (on the left-hand side of the figure) depicts 21st-century trends in depth to groundwater. Each point (coloured circle) represents a monitoring well; the colour of the point corresponds to the 21st-century trend in depth to groundwater for the monitoring well (Theil-Sen slope). Orange and red points represent monitoring wells where groundwater levels became deeper over time (Theil-Sen slope >0.25 m/year). Blue points represent monitoring wells where groundwater levels became shallower over time (Theil-Sen slope <0.25 m/year; see legend in upper-right corner of figure). The boundaries of the aquifer system are outlined in black. The map in the lower-right corner of the figure presents spatial variations in near-surface geology (data from ref.[1360]: Hartmann, J., & Moosdorf, N. (2012). The new global lithological map database GLiM: A representation of rock properties at the Earth surface. Geochemistry, Geophysics, Geosystems 13, doi.org/10.1029/2012GC004370). Imagery is from https://www.arcgis.com/home/item.html?id=10df2279f9684e4a9f6a7f08febac2a9

23.2 EXAMPLES OF AQUIFER SYSTEMS THAT DO NOT EXHIBIT CLUSTERING OF TRENDS IN DEPTH TO GROUNDWATER

The following maps present a series of aquifer systems where the Moran’s I values do not provide strong and significant evidence for clustering in trends in depth to groundwater (i.e., P-values > 0.05).

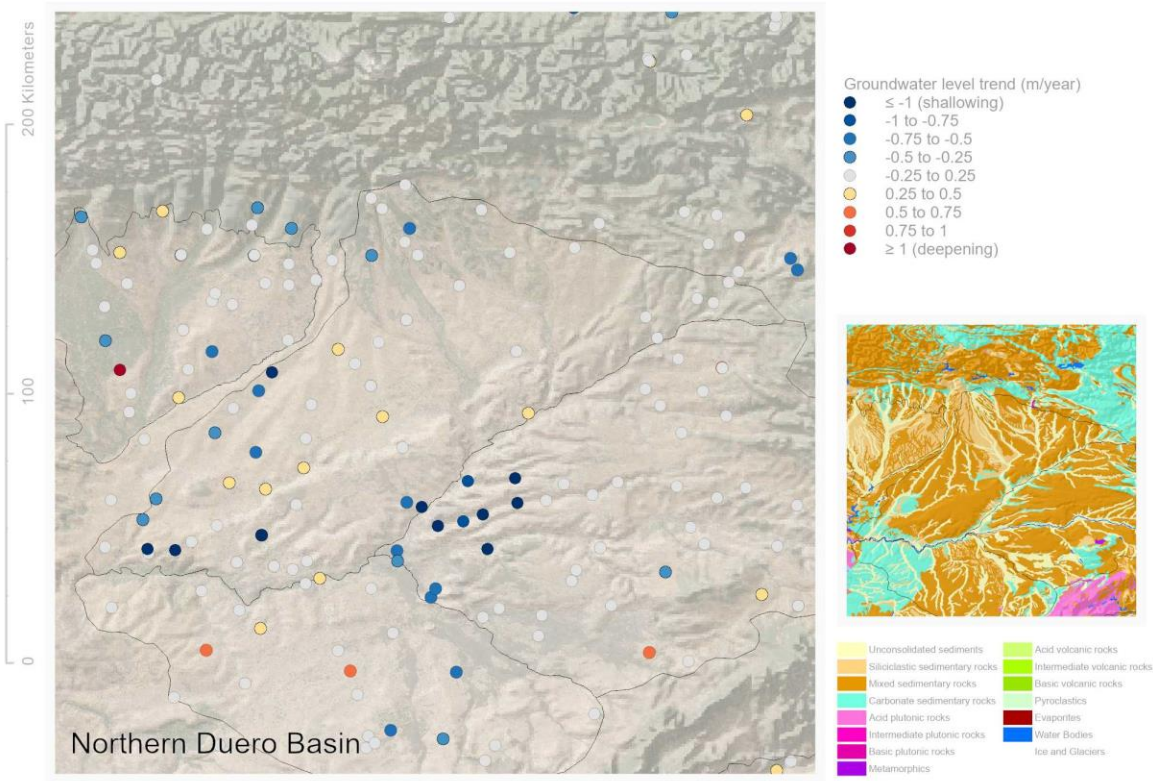

Supplementary Fig. 319. 21st-century trends in depth to groundwater in the Northern Duero Basin (Spain). The groundwater-level trends within this aquifer system (i.e., center of the map above) exhibit some degree of spatial clustering (e.g., red-shaded points tend to be co-located). The largest map (on the left-hand side of the figure) depicts 21st-century trends in depth to groundwater. Each point (coloured circle) represents a monitoring well; the colour of the point corresponds to the 21st-century trend in depth to groundwater for the monitoring well (Theil-Sen slope). Orange and red points represent monitoring wells where groundwater levels became deeper over time (Theil-Sen slope >0.25 m/year). Blue points represent monitoring wells where groundwater levels became shallower over time (Theil-Sen slope <0.25 m/year; see legend in upper-right corner of figure). The boundaries of the aquifer system are outlined in black. The map in the lower-right corner of the figure presents spatial variations in near-surface geology (data from ref.[1360]: Hartmann, J., & Moosdorf, N. (2012). The new global lithological map database GLiM: A representation of rock properties at the Earth surface. Geochemistry, Geophysics, Geosystems 13, doi.org/10.1029/2012GC004370). Imagery from https://www.arcgis.com/home/item.html?id=10df2279f9684e4a9f6a7f08febac2a9

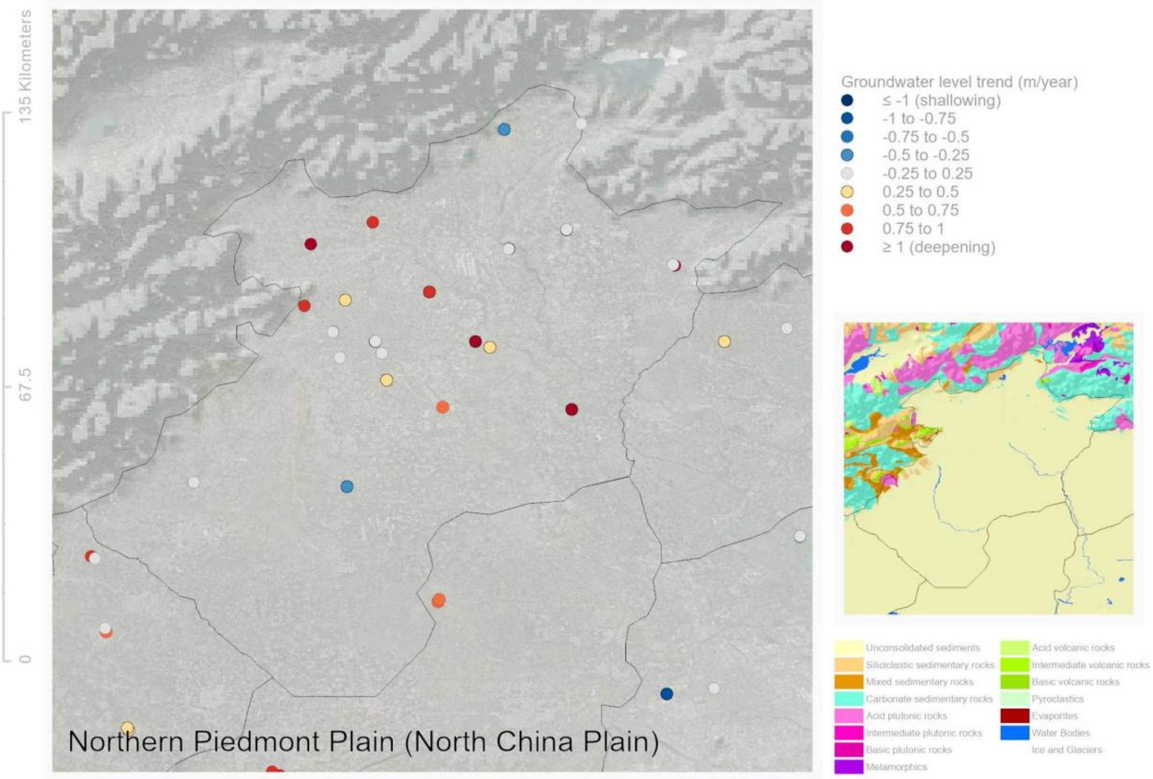

Supplementary Fig. 320. 21st-century trends in depth to groundwater in the Northern Piedmont Plain (North China Plain). The groundwater-level trends within this aquifer system (i.e., center of the map above) exhibit some degree of spatial clustering (e.g., red-shaded points tend to be co-located). The largest map (on the left-hand side of the figure) depicts 21st-century trends in depth to groundwater. Each point (coloured circle) represents a monitoring well; the colour of the point corresponds to the 21st-century trend in depth to groundwater for the monitoring well (Theil-Sen slope). Orange and red points represent monitoring wells where groundwater levels became deeper over time (Theil-Sen slope >0.25 m/year). Blue points represent monitoring wells where groundwater levels became shallower over time (Theil-Sen slope <0.25 m/year; see legend in upper-right corner of figure). The boundaries of the aquifer system are outlined in black. The map in the lower-right corner of the figure presents spatial variations in near-surface geology (data from ref.[1360]: Hartmann, J., & Moosdorf, N. (2012). The new global lithological map database GLiM: A representation of rock properties at the Earth surface. Geochemistry, Geophysics, Geosystems 13, doi.org/10.1029/2012GC004370). Imagery from https://www.arcgis.com/home/item.html?id=10df2279f9684e4a9f6a7f08febac2a9

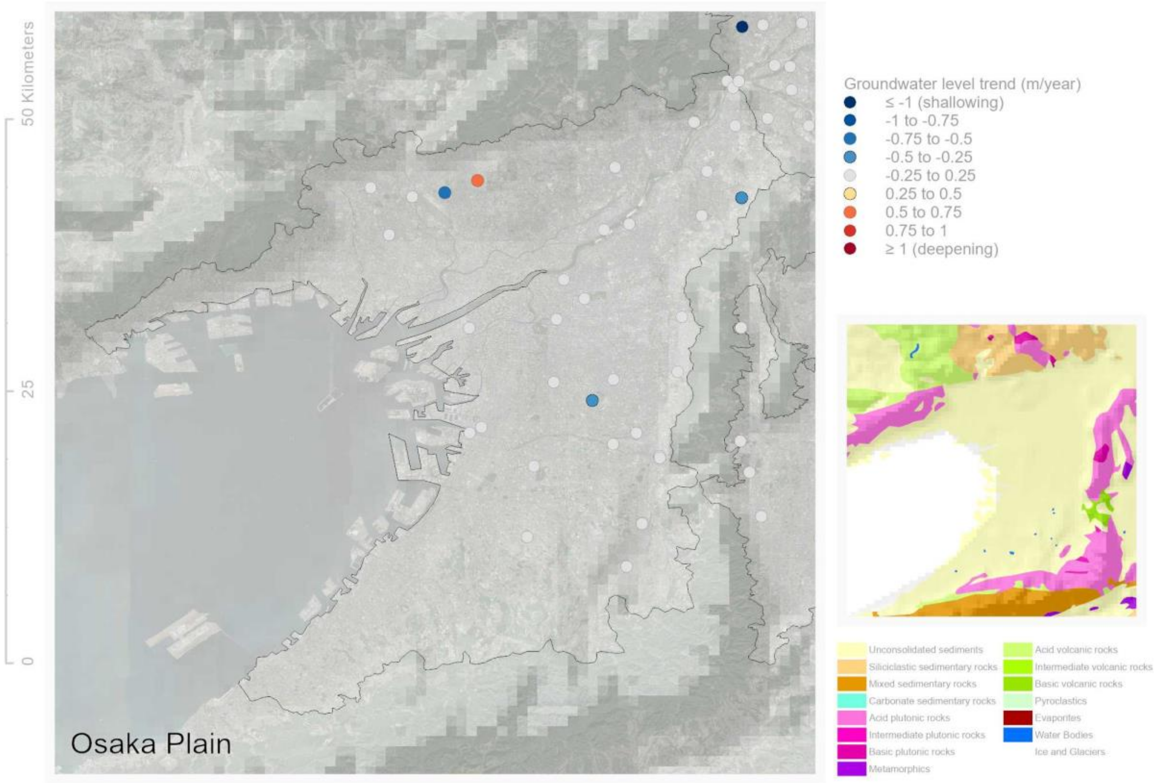

Supplementary Fig. 321. 21st-century trends in depth to groundwater in the Osaka Plain (Japan). The groundwater-level trends within this aquifer system (i.e., center of the map above) exhibit some degree of spatial clustering (e.g., red-shaded points tend to be co-located). The largest map (on the left-hand side of the figure) depicts 21st-century trends in depth to groundwater. Each point (coloured circle) represents a monitoring well; the colour of the point corresponds to the 21st-century trend in depth to groundwater for the monitoring well (Theil-Sen slope). Orange and red points represent monitoring wells where groundwater levels became deeper over time (Theil-Sen slope >0.25 m/year). Blue points represent monitoring wells where groundwater levels became shallower over time (Theil-Sen slope <0.25 m/year; see legend in upper-right corner of figure). The boundaries of the aquifer system are outlined in black. The map in the lower-right corner of the figure presents spatial variations in near-surface geology (data from ref.[1360]: Hartmann, J., & Moosdorf, N. (2012). The new global lithological map database GLiM: A representation of rock properties at the Earth surface. Geochemistry, Geophysics, Geosystems 13, doi.org/10.1029/2012GC004370). Imagery from https://www.arcgis.com/home/item.html?id=10df2279f9684e4a9f6a7f08febac2a9

24 TABLE SUMMARISING GROUNDWATER LEVEL TRENDS FOR 1,693 STUDY AQUIFER SYSTEMS

Supplementary Table 16. 21st-century trends in depth to groundwater for n=1,693 aquifer systems. Aquifer systems are ranked by aquifer-scale trends in depth to groundwater (i.e., ranked from highest to lowest median Theil-Sen slopes)

Study area	Area (km²)	Number of monitoring wells	Density of monitoring wells (with sufficient groundwater level observations for analyses) within the study area	Median Theil-Sen slope among monitoring wells *(positive values indicate deeper depths to water over time)*	Percent of wells with Theil-Sen slopes of >0 m per year	Percent of wells with Theil-Sen slopes of >0.5 m per year
Ascoy-Soplamo Aquifer	394	4	1 well for every 99 km²	2.95	75%	75%
Arsanjan Basin	208	12	1 well for every 17 km²	2.82	100%	83%
Baladeh Basin	47	7	1 well for every 7 km²	2.68	100%	100%
Rashtkhar Aquifer	714	12	1 well for every 60 km²	2.62	92%	83%
Yuncheng Basin	4659	1	1 well for every 4659 km²	2.49	100%	100%
Upper Copiapo Valley (Copiapo Valley)	42	2	1 well for every 21 km²	2.47	100%	100%
Fuente-de-Piedra Aquifer	197	3	1 well for every 66 km²	2.39	67%	67%
Kordi Shirazi Basin	67	6	1 well for every 11 km²	2.35	100%	100%
Monterrey Valley	1562	4	1 well for every 391 km²	2.33	100%	50%
Southern Shamil-Meymand Basin	93	10	1 well for every 9 km²	2.22	100%	90%
Mill Creek Aquifer	754	1	1 well for every 754 km²	2.17	100%	100%
Middle Copiapo Valley (Copiapo Valley)	176	4	1 well for every 44 km²	2.04	100%	75%
Soghan Basin	136	7	1 well for every 19 km²	1.97	100%	86%
Sierra Espuna Aquifer	617	8	1 well for every 77 km²	1.84	75%	63%
Bekan Basin	162	11	1 well for every 15 km²	1.82	91%	91%
Aledo Aquifer	51	1	1 well for every 51 km²	1.80	100%	100%
Eastern Kome Basin (Kome Basin)	101	10	1 well for every 10 km²	1.77	100%	70%
Western Qazvin Plain (Qazvin Plain)	1689	61	1 well for every 28 km²	1.74	97%	77%
Western Fakhrabad Basin	110	6	1 well for every 18 km²	1.73	100%	83%
Beram Basin	195	17	1 well for every 11 km²	1.73	100%	88%
Orzooiyeh Basin	1444	23	1 well for every 63 km²	1.72	96%	91%
Eyvanekey Basin (Tehran Plain)	280	17	1 well for every 16 km²	1.71	100%	88%
Jumilla-Villena Aquifer	334	3	1 well for every 111 km²	1.68	100%	100%

Study area	Area (km^2)	Number of monitoring wells	Density of monitoring wells (with sufficient groundwater level observations for analyses) within the study area	Median Theil-Sen slope among monitoring wells *(positive values indicate deeper depths to water over time)*	Percent of wells with Theil-Sen slopes of >0 m per year	Percent of wells with Theil-Sen slopes of >0.5 m per year
Northwest Jaipur Alluvium and Mendha Basin (Malwa Plateau)	5731	18	1 well for every 318 km^2	1.68	94%	89%
Southern Qazvin Plain (Qazvin Plain)	1579	46	1 well for every 34 km^2	1.64	100%	78%
Cingla-Cuchillo Aquifer	434	5	1 well for every 87 km^2	1.60	100%	100%
Jining Area	936	4	1 well for every 234 km^2	1.59	100%	75%
Hengam Basin	74	10	1 well for every 7 km^2	1.59	90%	70%
Seyedan-Farooq Aquifer	200	14	1 well for every 14 km^2	1.57	100%	93%
Dehrood -e Tangh -e Eram Basin	118	13	1 well for every 9 km^2	1.52	100%	62%
Khanmirza Plain	207	14	1 well for every 15 km^2	1.51	100%	86%
Kuhdasht Plain	362	13	1 well for every 28 km^2	1.47	100%	54%
Narimani Basin (Mashad-Narimani Basin)	1108	13	1 well for every 85 km^2	1.46	92%	92%
Cuyama Valley	483	77	1 well for every 6 km^2	1.45	91%	70%
Sierra de Crevillente Aquifer	52	4	1 well for every 13 km^2	1.43	75%	75%
Abhar Plain	1154	36	1 well for every 32 km^2	1.42	100%	92%
Homand Basin	237	22	1 well for every 11 km^2	1.42	86%	73%
Nodej Basin	144	6	1 well for every 24 km^2	1.40	100%	83%
Lower Nowbaran Basin	432	10	1 well for every 43 km^2	1.39	100%	80%
Faryab Basin	615	24	1 well for every 26 km^2	1.35	100%	96%
Chacabuco-Polpaico Aquifer	241	8	1 well for every 30 km^2	1.33	100%	88%
Eastern Neyshabur Plain (Neyshabur Plain)	1298	16	1 well for every 81 km^2	1.31	100%	81%
Eastern Saq Aquifer (Ram-Saq Aquifer System)	67601	29	1 well for every 2331 km^2	1.30	86%	72%
Southwestern Qaderabad Basin	151	15	1 well for every 10 km^2	1.26	100%	87%
Baghmalek Plain	81	10	1 well for every 8 km^2	1.23	90%	70%
Aliabad Plain	2056	45	1 well for every 46 km^2	1.21	100%	76%
Gila Bend Basin	1028	2	1 well for every 514 km^2	1.21	100%	100%
Eastern Lordegan Basin (Lordegan Basin)	116	3	1 well for every 39 km^2	1.20	100%	100%
Zarand Basin	2241	44	1 well for every 51 km^2	1.19	93%	70%
Imamzadeh Jafar Gachsaran Plain	126	20	1 well for every 6 km^2	1.19	100%	100%
Gharebagh Basin	231	13	1 well for every 18 km^2	1.18	100%	54%
White Wolf Basin (Tulare Basin of the Central Valley)	432	7	1 well for every 62 km^2	1.18	100%	100%
Deh Kahan Basin	27	3	1 well for every 9 km^2	1.17	100%	67%
Southern Nallamalai Basin (Cuddapah Sedimentary Basin)	2227	1	1 well for every 2227 km^2	1.15	100%	100%
San Pasqual Valley	22	9	1 well for every 2 km^2	1.14	67%	56%
Northern Littoral Plain (North China Plain)	4660	10	1 well for every 466 km^2	1.10	80%	70%
Damanehdaran Aquifer	338	15	1 well for every 23 km^2	1.10	100%	60%
Kashmar-Bardaskan Plain	2242	41	1 well for every 55 km^2	1.06	98%	76%
Boqueron Aquifer	294	5	1 well for every 59 km^2	1.05	100%	100%
Northern Laoha River Basin (Laoha River Basin)	3261	6	1 well for every 544 km^2	1.05	100%	100%
Central Souss Basin (Souss-Chtouka Basin)	2063	4	1 well for every 516 km^2	1.04	50%	50%
Manujan Basin	487	19	1 well for every 26 km^2	1.03	95%	84%
Dashtab Basin	236	13	1 well for every 18 km^2	1.02	100%	69%
Bushkan Plain	112	17	1 well for every 7 km^2	1.02	100%	71%
Chowchilla Basin (San Joaquin Basin of the Central Valley)	600	118	1 well for every 5 km^2	1.02	89%	65%

Study area	Area (km^2)	Number of monitoring wells	Density of monitoring wells (with sufficient groundwater level observations for analyses) within the study area	Median Theil-Sen slope among monitoring wells *(positive values indicate deeper depths to water over time)*	Percent of wells with Theil-Sen slopes of >0 m per year	Percent of wells with Theil-Sen slopes of >0.5 m per year
Southern Neyshabur Plain (Neyshabur Plain)	810	14	1 well for every 58 km^2	1.02	100%	86%
Eastern Torbat-Zaveh Plain (Torbat-Zaveh Plain)	575	9	1 well for every 64 km^2	1.02	100%	89%
Calera Aquifer	1742	18	1 well for every 97 km^2	1.01	94%	61%
Northern Kern Basin (Tulare Basin of the Central Valley)	3044	601	1 well for every 5 km^2	0.99	91%	73%
Golpayegan Aquifer	603	31	1 well for every 19 km^2	0.99	97%	71%
Upper La Ligua Aquifer (La Ligua Aquifer)	124	5	1 well for every 25 km^2	0.98	100%	80%
Khir Plain	98	5	1 well for every 20 km^2	0.98	100%	80%
Fahliyan Basin	132	14	1 well for every 9 km^2	0.97	100%	86%
Northern Qazvin Plain (Qazvin Plain)	1823	74	1 well for every 25 km^2	0.97	96%	68%
Sahre e- Bagh -e Lar Basin	667	17	1 well for every 39 km^2	0.97	100%	65%
Nurabad Mamasani Plain Aquifer	201	20	1 well for every 10 km^2	0.97	100%	80%
Azghand Basin	724	7	1 well for every 103 km^2	0.97	100%	100%
Ghorveh-Dehgelan Aquifer	3040	105	1 well for every 29 km^2	0.96	92%	71%
Zanjan Plain	1894	57	1 well for every 33 km^2	0.95	91%	65%
Dasht e- Nemdan Basin	1549	49	1 well for every 32 km^2	0.95	98%	76%
Qom Kahak Basin	1066	47	1 well for every 23 km^2	0.94	96%	83%
Cienega Prieta-Moroleon Aquifer	367	3	1 well for every 122 km^2	0.94	100%	67%
Ojos Negros Basin	215	9	1 well for every 24 km^2	0.94	100%	100%
Eastern Rezvan Basin	52	10	1 well for every 5 km^2	0.93	100%	100%
Dumak Basin	210	11	1 well for every 19 km^2	0.92	100%	82%
Sadegh Abad Basin	463	17	1 well for every 27 km^2	0.92	100%	82%
Feyzabad Plain	954	28	1 well for every 34 km^2	0.91	100%	71%
Upper Aconcagua Basin (Aconcagua Basin)	473	6	1 well for every 79 km^2	0.90	100%	67%
Jovin Plain	3468	61	1 well for every 57 km^2	0.90	98%	74%
Eij Basin	84	6	1 well for every 14 km^2	0.89	100%	67%
Western Torbat-Zaveh Plain (Torbat-Zaveh Plain)	681	13	1 well for every 52 km^2	0.88	100%	77%
Shahrekord Plain	575	34	1 well for every 17 km^2	0.88	97%	82%
Kaweah Basin (Tulare Basin of the Central Valley)	1825	499	1 well for every 4 km^2	0.88	95%	73%
Upper Morghab Basin	94	13	1 well for every 7 km^2	0.87	100%	77%
Harasm Basin	200	4	1 well for every 50 km^2	0.87	100%	75%
Miami Basin	2297	68	1 well for every 34 km^2	0.87	99%	65%
Sinclinal de la Higuera	703	8	1 well for every 88 km^2	0.86	88%	50%
Komijan Aquifer (Razan Basin)	1448	54	1 well for every 27 km^2	0.86	98%	70%
Northern Shamil-Meymand Basin	354	28	1 well for every 13 km^2	0.86	93%	64%
Kazeroun Plain	386	60	1 well for every 6 km^2	0.85	100%	72%
Qabr-e Kikha Basin	456	27	1 well for every 17 km^2	0.85	96%	85%
Lower Kabootar Ahang Basin (Razan Basin)	2237	50	1 well for every 45 km^2	0.84	94%	56%
Rayen Aquifer	750	11	1 well for every 68 km^2	0.83	91%	55%
Semirom Basin	59	4	1 well for every 15 km^2	0.83	100%	75%
Tule Basin (Tulare Basin of the Central Valley)	2003	266	1 well for every 8 km^2	0.80	88%	63%
Dariyan Basin	138	11	1 well for every 13 km^2	0.80	91%	64%
Marvdasht and Kharameh Plains	2556	68	1 well for every 38 km^2	0.80	100%	66%

Study area	Area (km²)	Number of monitoring wells	Density of monitoring wells (with sufficient groundwater level observations for analyses) within the study area	Median Theil-Sen slope among monitoring wells *(positive values indicate deeper depths to water over time)*	Percent of wells with Theil-Sen slopes of >0 m per year	Percent of wells with Theil-Sen slopes of >0.5 m per year
Estahban Basin	89	4	1 well for every 22 km²	0.78	100%	75%
Lo Ovalle Aquifer	57	1	1 well for every 57 km²	0.78	100%	100%
Chadegan Plain	238	12	1 well for every 20 km²	0.78	100%	58%
Popeto Aquifer	146	2	1 well for every 73 km²	0.78	100%	100%
Jaghin-Tokahor Plain	636	30	1 well for every 21 km²	0.78	90%	70%
Central Razan Plain (Razan Basin)	796	29	1 well for every 27 km²	0.78	97%	72%
Kavar and Kavar-Maharloo Aquifers	556	27	1 well for every 21 km²	0.77	100%	59%
La Vinilla Casablanca Aquifer	117	4	1 well for every 29 km²	0.77	100%	50%
Kerman-Baghin Aquifer	2950	56	1 well for every 53 km²	0.76	89%	59%
Kavir Qatruiyeh Basin	1120	24	1 well for every 47 km²	0.75	96%	75%
Northcentral Piedmont Plain (North China Plain)	18327	23	1 well for every 797 km²	0.75	91%	61%
Riz Basin	77	14	1 well for every 6 km²	0.75	100%	79%
Marveshkan Basin	408	22	1 well for every 19 km²	0.74	100%	68%
Reyosh Basin	234	6	1 well for every 39 km²	0.74	100%	67%
Eastern Imperial and Amos and Oligby Valleys	1934	30	1 well for every 64 km²	0.74	97%	70%
Jangal Plain	1275	8	1 well for every 159 km²	0.73	100%	63%
Central Kabul Basin (Kabul Basin)	110	14	1 well for every 8 km²	0.73	100%	57%
Kavir -e Abarkooh Basin	1820	30	1 well for every 61 km²	0.73	93%	60%
Esfandagheh Basin	130	7	1 well for every 19 km²	0.73	71%	57%
Orcheta Region covering the Cabeco dOr Aquifer	161	2	1 well for every 81 km²	0.72	100%	50%
Sib-Souran Basin	1602	28	1 well for every 57 km²	0.72	82%	61%
Confined Claiborne Near Jackson (Mississippi Embayment)	9606	16	1 well for every 600 km²	0.72	88%	56%
Jiroft Plain	1928	72	1 well for every 27 km²	0.71	85%	63%
Zaran -e Saveh Basin	1689	61	1 well for every 28 km²	0.70	90%	59%
Coastal Caborca Aquifer (Caborca Aquifer System)	2076	10	1 well for every 208 km²	0.69	100%	60%
Madera Basin (San Joaquin Basin of the Central Valley)	1662	169	1 well for every 10 km²	0.69	94%	64%
Saadat Abad Basin	173	15	1 well for every 12 km²	0.69	100%	80%
Cuberta de el Saltador	99	2	1 well for every 50 km²	0.69	100%	100%
Firuzabad Plain	333	47	1 well for every 7 km²	0.68	98%	66%
Darab Basin	889	42	1 well for every 21 km²	0.67	88%	57%
Sharif Abab and Masileh Aquifers	605	23	1 well for every 26 km²	0.67	87%	61%
Pino and Conejeros-Albatana and Tobarra-Tedera-Pinilla Aquifers	296	3	1 well for every 99 km²	0.66	100%	67%
Bad-Khaledabad Basin	663	14	1 well for every 47 km²	0.66	100%	57%
Central Nallamalai Basin (Cuddapah Sedimentary Basin)	4462	3	1 well for every 1487 km²	0.66	100%	100%
Mohammad Abad Area (Lower Zayandeh-Rud Basin)	2635	64	1 well for every 41 km²	0.65	89%	61%
Upper Mashad Plain (Mashad-Narimani Basin)	2749	49	1 well for every 56 km²	0.65	92%	59%
Kadkan Basin	1406	30	1 well for every 47 km²	0.65	100%	70%
Western Second Songhuajiang System (Songnen Plain)	14776	3	1 well for every 4925 km²	0.64	100%	67%
Malahat Basin	1018	4	1 well for every 255 km²	0.64	100%	75%
Westside Basin (Tulare Basin of the Central Valley)	2979	288	1 well for every 10 km²	0.64	78%	56%
Upper Qeydar Basin (Qeydar Basin)	691	17	1 well for every 41 km²	0.64	94%	65%

Study area	Area (km²)	Number of monitoring wells	Density of monitoring wells (with sufficient groundwater level observations for analyses) within the study area	Median Theil-Sen slope among monitoring wells *(positive values indicate deeper depths to water over time)*	Percent of wells with Theil-Sen slopes of >0 m per year	Percent of wells with Theil-Sen slopes of >0.5 m per year
Wuhai Basin	414	3	1 well for every 138 km^2	0.64	100%	67%
Kongque Piedmont Plain	4630	3	1 well for every 1543 km^2	0.63	100%	100%
Fasa Basin	402	23	1 well for every 17 km^2	0.63	96%	70%
Central Santiago Basin (Santiago Basin)	1534	18	1 well for every 85 km^2	0.63	94%	56%
Kamfruz Alluvial Aquifer	144	11	1 well for every 13 km^2	0.62	100%	55%
Kalacho Plain	159	28	1 well for every 6 km^2	0.62	100%	57%
Tavabeh Arsanjan Basin	178	9	1 well for every 20 km^2	0.61	100%	67%
Eastern Ghavbandi Basin	255	13	1 well for every 20 km^2	0.61	100%	77%
Asadabad Plain	411	23	1 well for every 18 km^2	0.61	96%	65%
Southern Kern Basin (Tulare Basin of the Central Valley)	2265	210	1 well for every 11 km^2	0.60	75%	55%
Kangavar Aquifer	351	20	1 well for every 18 km^2	0.60	85%	55%
Lobo Flat (Salt Basin Bolson)	389	9	1 well for every 43 km^2	0.60	89%	56%
Chahak -e Shahriari Basin	1153	23	1 well for every 50 km^2	0.60	96%	57%
Sarayan Plain	1219	22	1 well for every 55 km^2	0.60	100%	55%
Sirjan Basin	3617	80	1 well for every 45 km^2	0.60	100%	55%
Western Zayandeh-Rud Basin	1010	18	1 well for every 56 km^2	0.59	100%	56%
Rodbar Jiroft Plain (Jazmurian Depression)	6148	62	1 well for every 99 km^2	0.59	94%	56%
Mobarak Abad-Baros Basin	178	25	1 well for every 7 km^2	0.59	68%	52%
La Blanca Aquifer	383	5	1 well for every 77 km^2	0.59	100%	60%
Dezhgah-Kordeh Basin	205	14	1 well for every 15 km^2	0.59	100%	64%
Ghoharkoh Basin	1051	33	1 well for every 32 km^2	0.58	100%	58%
Murchehkhort Basin (Lower Zayandeh-Rud Basin)	1172	20	1 well for every 59 km^2	0.58	95%	55%
Codegua and Graneros-Rancagua Aquifers	632	11	1 well for every 57 km^2	0.57	82%	64%
Shahroud Plain	662	30	1 well for every 22 km^2	0.57	97%	50%
Shiraz Plain	401	41	1 well for every 10 km^2	0.57	90%	54%
Abadeh Tashk Basin	652	27	1 well for every 24 km^2	0.57	93%	56%
Sedeh Basin	170	7	1 well for every 24 km^2	0.57	100%	57%
Bahadoran Basin	1627	28	1 well for every 58 km^2	0.57	96%	54%
Pleasant Valley west of the San Joaquin Basin	201	41	1 well for every 5 km^2	0.57	88%	54%
Garmsar Basin	1287	56	1 well for every 23 km^2	0.57	100%	64%
Bosrouyeh Basin	1600	21	1 well for every 76 km^2	0.56	100%	67%
Marvast Basin	590	29	1 well for every 20 km^2	0.56	93%	52%
Eastern Quibas Aquifer	72	2	1 well for every 36 km^2	0.56	100%	50%
Hashtgerd Plain	518	22	1 well for every 24 km^2	0.56	100%	55%
Papaghni Basin (Cuddapah Sedimentary Basin)	5532	5	1 well for every 1106 km^2	0.56	100%	60%
Shihezi Area	825	2	1 well for every 413 km^2	0.56	50%	50%
Bahabad Plain	497	15	1 well for every 33 km^2	0.55	100%	73%
Pliocuaternario and Mioceno de Alfamen Aquifers	358	9	1 well for every 40 km^2	0.55	89%	56%
Western Lordegan Basin (Lordegan Basin)	80	11	1 well for every 7 km^2	0.55	91%	55%
Darkoye and Ghareblagh Basins	203	14	1 well for every 15 km^2	0.55	93%	50%
Abarkooh Plain	1664	43	1 well for every 39 km^2	0.55	100%	56%
Southern Santiago Basin (Santiago Basin)	692	7	1 well for every 99 km^2	0.54	100%	57%
Upper Beichuan Area (Huangshui Basin)	92	3	1 well for every 31 km^2	0.54	100%	67%

Study area	Area (km^2)	Number of monitoring wells	Density of monitoring wells (with sufficient groundwater level observations for analyses) within the study area	Median Theil-Sen slope among monitoring wells *(positive values indicate deeper depths to water over time)*	Percent of wells with Theil-Sen slopes of >0 m per year	Percent of wells with Theil-Sen slopes of >0.5 m per year
Valle de Chicalote	158	8	1 well for every 20 km^2	0.54	100%	63%
Parowan Valley	384	46	1 well for every 8 km^2	0.54	98%	61%
Aspas Plain	1040	51	1 well for every 20 km^2	0.53	96%	51%
Sikh Darengon Basin	417	20	1 well for every 21 km^2	0.53	90%	50%
Esfarayen Plain	1543	35	1 well for every 44 km^2	0.53	97%	57%
Rafsanjan Basin	3927	70	1 well for every 56 km^2	0.53	96%	54%
Salisbury Plain and Hampshire Chalk Aquifer (Chalk Aquifer System)	2904	1	1 well for every 2904 km^2	0.53	100%	100%
Vanegas-Catorce Aquifer	2631	2	1 well for every 1316 km^2	0.53	100%	50%
Galehdar Plain	324	10	1 well for every 32 km^2	0.52	100%	70%
Secret Valley	95	2	1 well for every 48 km^2	0.52	100%	50%
Khomein Basin	971	33	1 well for every 29 km^2	0.51	91%	52%
Little Chino Valley	472	8	1 well for every 59 km^2	0.51	100%	63%
Northern Santiago Basin (Santiago Basin)	617	18	1 well for every 34 km^2	0.51	94%	50%
Eastern Khash Basin	838	29	1 well for every 29 km^2	0.51	93%	52%
Eastern Goltapeh Zarin Abad Basin	1085	34	1 well for every 32 km^2	0.51	94%	50%
Chupaderos Aquifer	2000	8	1 well for every 250 km^2	0.51	88%	50%
Mahidasht Plain	1100	49	1 well for every 22 km^2	0.51	90%	51%
Lamerd Plain	1422	9	1 well for every 158 km^2	0.51	100%	56%
Baghan Basin	31	5	1 well for every 6 km^2	0.50	100%	60%
Malayer Aquifer	826	39	1 well for every 21 km^2	0.50	79%	49%
Ardestand Basin	1939	26	1 well for every 75 km^2	0.50	100%	50%
Upper Borrego Valley	263	22	1 well for every 12 km^2	0.50	86%	50%
Khosuye-Hajiabad Plain	496	15	1 well for every 33 km^2	0.50	87%	47%
Alturas Warm Springs Valley	253	7	1 well for every 36 km^2	0.49	86%	43%
Jam Basin	61	8	1 well for every 8 km^2	0.49	100%	50%
Kings Basin (Tulare Basin of the Central Valley)	3896	688	1 well for every 6 km^2	0.49	89%	49%
Vanrhynsdorp Aquifer	456	26	1 well for every 18 km^2	0.49	100%	50%
Shirvan Plain (Shirvan-Quchan Plain)	355	17	1 well for every 21 km^2	0.49	100%	41%
Kashan Plain	2116	52	1 well for every 41 km^2	0.48	94%	48%
Izeh Plain	206	36	1 well for every 6 km^2	0.48	89%	44%
Kahorestan Basin	543	22	1 well for every 25 km^2	0.48	100%	50%
San Bernardino Basin (Upper Santa Ana Basin)	312	157	1 well for every 2 km^2	0.48	87%	48%
Beryl-Enterprise Subarea (Escalante Valley)	1542	42	1 well for every 37 km^2	0.48	100%	45%
Bam and Rahmatabad Basins	4590	82	1 well for every 56 km^2	0.47	99%	43%
Jerez Aquifer	691	4	1 well for every 173 km^2	0.47	75%	50%
Sarchahan Aquifer	759	40	1 well for every 19 km^2	0.47	98%	43%
Khesht Plain	126	10	1 well for every 13 km^2	0.47	100%	50%
Darenjan Basin	148	12	1 well for every 12 km^2	0.47	100%	50%
Lo Orozco Aquifer	43	1	1 well for every 43 km^2	0.47	100%	0%
Farashband Plain	219	19	1 well for every 12 km^2	0.46	95%	42%
Abadeh Eghlid Basin	1611	32	1 well for every 50 km^2	0.46	94%	47%
Khash Aquifer	505	18	1 well for every 28 km^2	0.46	94%	39%
Hohhot Basin	8173	8	1 well for every 1022 km^2	0.45	88%	50%

Study area	Area (km^2)	Number of monitoring wells	Density of monitoring wells (with sufficient groundwater level observations for analyses) within the study area	Median Theil-Sen slope among monitoring wells *(positive values indicate deeper depths to water over time)*	Percent of wells with Theil-Sen slopes of >0 m per year	Percent of wells with Theil-Sen slopes of >0.5 m per year
Central High Plains (High Plains)	71803	1301	1 well for every 55 km^2	0.45	91%	47%
Roniz Basin	153	14	1 well for every 11 km^2	0.45	93%	50%
Harat Basin	902	29	1 well for every 31 km^2	0.45	97%	45%
Eastern Fakhrabad Basin	83	4	1 well for every 21 km^2	0.45	100%	50%
San Antonio Creek Valley	218	31	1 well for every 7 km^2	0.44	100%	35%
Southeastern System (Amadeus Basin)	59372	1	1 well for every 59372 km^2	0.44	100%	0%
Eastern Qaderabad Basin	617	23	1 well for every 27 km^2	0.44	91%	39%
Yazd-Ardakan Basin	6220	98	1 well for every 63 km^2	0.44	84%	42%
Tulare Lake Basin (Tulare Basin of the Central Valley)	2135	128	1 well for every 17 km^2	0.44	79%	45%
Eastern Sojas Basin (Sojas Basin)	786	39	1 well for every 20 km^2	0.44	87%	41%
Yengejeh Basin	125	3	1 well for every 42 km^2	0.44	100%	33%
Islam Abad Plain	251	19	1 well for every 13 km^2	0.44	100%	42%
Goshnekan Basin	27	5	1 well for every 5 km^2	0.44	100%	0%
Goshen Valley	331	16	1 well for every 21 km^2	0.43	88%	44%
Serranias de Cuenca Aquifer	2807	4	1 well for every 702 km^2	0.43	75%	50%
Southern Kettleman Plain	187	29	1 well for every 6 km^2	0.43	86%	38%
Western Quibas and Serral-Salinas Aquifers	200	6	1 well for every 33 km^2	0.43	50%	50%
Northwest Coimbatore Fractured Charnockite and Gneiss Aquifers (Coimbatore Upland)	6515	71	1 well for every 92 km^2	0.43	86%	48%
Hesaruiyeh Basin	82	7	1 well for every 12 km^2	0.43	100%	0%
Cedar Valley	580	48	1 well for every 12 km^2	0.42	92%	46%
Pahvant Valley	944	62	1 well for every 15 km^2	0.42	97%	37%
Aguanaval Aquifer	1493	22	1 well for every 68 km^2	0.42	86%	41%
Southern Piedmont Plain (North China Plain)	6809	19	1 well for every 358 km^2	0.42	95%	37%
Western Kome Basin (Kome Basin)	103	12	1 well for every 9 km^2	0.42	100%	33%
Central Mashad Plain (Mashad-Narimani Basin)	1202	31	1 well for every 39 km^2	0.42	74%	29%
Birjand Aquifer	1007	25	1 well for every 40 km^2	0.42	88%	44%
Ranegras Plain	984	2	1 well for every 492 km^2	0.42	100%	50%
Boroujen Aquifer	301	16	1 well for every 19 km^2	0.41	88%	50%
Basht Basin	57	12	1 well for every 5 km^2	0.41	92%	42%
Quinn River Valley in northern Nevada	1129	2	1 well for every 565 km^2	0.41	100%	0%
Srisailam Basin (Cuddapah Sedimentary Basin)	3720	2	1 well for every 1860 km^2	0.41	100%	50%
Beyza-Zarghan Plain	1119	36	1 well for every 31 km^2	0.41	100%	42%
Minab Plain	423	17	1 well for every 25 km^2	0.41	94%	41%
Gulgir Plain	36	15	1 well for every 2 km^2	0.41	100%	13%
Ravar Basin	512	16	1 well for every 32 km^2	0.41	94%	38%
Arak Plain	3047	52	1 well for every 59 km^2	0.41	100%	46%
Estancia Basin	4091	1	1 well for every 4091 km^2	0.40	100%	0%
Merced Basin (San Joaquin Basin of the Central Valley)	2101	429	1 well for every 5 km^2	0.40	96%	42%
Quchan Plain (Shirvan-Quchan Plain)	819	23	1 well for every 36 km^2	0.40	96%	43%
Shamil Aquifer	1791	54	1 well for every 33 km^2	0.40	100%	17%
Campos de Nijar Aquifer	322	2	1 well for every 161 km^2	0.39	100%	50%
Aguascalientes Valley	1201	20	1 well for every 60 km^2	0.39	80%	45%
Semnan and Sorkhe Aquifers	1076	46	1 well for every 23 km^2	0.39	89%	39%

Study area	Area (km^2)	Number of monitoring wells	Density of monitoring wells (with sufficient groundwater level observations for analyses) within the study area	Median Theil-Sen slope among monitoring wells *(positive values indicate deeper depths to water over time)*	Percent of wells with Theil-Sen slopes of >0 m per year	Percent of wells with Theil-Sen slopes of >0.5 m per year
Neyriz Plain	277	21	1 well for every 13 km^2	0.39	81%	19%
Daryache Parishan Basin	95	15	1 well for every 6 km^2	0.39	100%	33%
Linfen Basin	7489	1	1 well for every 7489 km^2	0.39	100%	0%
Damghan Sedimentary Plain	1710	55	1 well for every 31 km^2	0.39	100%	31%
Villena-Benejama Region covering the Solana Aquifer	556	10	1 well for every 56 km^2	0.38	80%	40%
Cholqui Aquifer	103	2	1 well for every 52 km^2	0.38	100%	50%
Western Neyshabur Plain (Neyshabur Plain)	1595	28	1 well for every 57 km^2	0.38	100%	39%
Esfahan Basin (Lower Zayandeh-Rud Basin)	2466	38	1 well for every 65 km^2	0.38	84%	45%
Upper Rio Elqui Basin (Elqui River Basin)	115	12	1 well for every 10 km^2	0.38	100%	33%
Wuwei Basin (Shiyang Basin)	4569	2	1 well for every 2285 km^2	0.38	100%	50%
Western Liaohe River Basin (Liaohe River Basin)	43494	8	1 well for every 5437 km^2	0.38	88%	25%
Mountain Home Plateau (Western Snake River Plain)	3505	39	1 well for every 90 km^2	0.38	90%	5%
Marand Plain	768	66	1 well for every 12 km^2	0.38	76%	42%
Sefid-Dasht Aquifer	201	15	1 well for every 13 km^2	0.38	100%	47%
Chehelkhaneh Aquifer	83	7	1 well for every 12 km^2	0.37	100%	43%
Isin Basin	270	18	1 well for every 15 km^2	0.37	89%	33%
Dindigul Fractured Charnockite and Gneiss Aquifers	1306	17	1 well for every 77 km^2	0.37	94%	35%
Northern Kettleman Plain	142	9	1 well for every 16 km^2	0.37	78%	44%
Deh Bid Basin	1538	41	1 well for every 38 km^2	0.37	100%	41%
Gila Valley	600	1	1 well for every 600 km^2	0.37	100%	0%
Ojocaliente Aquifer	526	2	1 well for every 263 km^2	0.36	100%	0%
Jajarm Plain	495	19	1 well for every 26 km^2	0.36	100%	37%
Gandoman-Boldaji Aquifer	156	14	1 well for every 11 km^2	0.36	100%	29%
San Jose del Cabo Aquifer	838	6	1 well for every 140 km^2	0.36	83%	17%
Cuauhtemoc Basin	2418	23	1 well for every 105 km^2	0.36	65%	39%
Southern Saurashtra Deccan Trap Basalts (Saurashtra Deccan Trap Basalts)	30217	144	1 well for every 210 km^2	0.36	92%	34%
Shah Gheyb Basin	186	4	1 well for every 47 km^2	0.35	100%	25%
Northern Darz Sayebn Basin	180	7	1 well for every 26 km^2	0.35	71%	29%
Southern Coromandel Fractured Charnockite and Gneiss Aquifers (Coromandel Coastal Plain)	13538	92	1 well for every 147 km^2	0.35	93%	33%
Valparaiso Aquifer	404	7	1 well for every 58 km^2	0.35	71%	43%
Southwestern Jazmurian Depression (Jazmurian Depression)	2613	12	1 well for every 218 km^2	0.35	100%	25%
Mokhtaran Basin	595	15	1 well for every 40 km^2	0.35	100%	13%
Juab Valley	393	28	1 well for every 14 km^2	0.34	100%	36%
Navidad-Potosi-Raices Aquifer	1255	61	1 well for every 21 km^2	0.34	89%	36%
Willcox-Douglas Basin	9486	1	1 well for every 9486 km^2	0.34	100%	0%
Bajestan Basin	1074	19	1 well for every 57 km^2	0.34	95%	26%
Javim Banaruyeh Basin	591	14	1 well for every 42 km^2	0.34	93%	21%
Lower La Ligua Aquifer (La Ligua Aquifer)	111	5	1 well for every 22 km^2	0.33	100%	0%
Middle Chao Phraya Basin	22178	47	1 well for every 472 km^2	0.33	87%	40%
Korin Basin	1140	27	1 well for every 42 km^2	0.33	100%	30%
Bardsir Plain	2363	55	1 well for every 43 km^2	0.33	96%	36%
Sabzevar Plain	3401	73	1 well for every 47 km^2	0.33	99%	32%

Study area	Area (km^2)	Number of monitoring wells	Density of monitoring wells (with sufficient groundwater level observations for analyses) within the study area	Median Theil-Sen slope among monitoring wells *(positive values indicate deeper depths to water over time)*	Percent of wells with Theil-Sen slopes of >0 m per year	Percent of wells with Theil-Sen slopes of >0.5 m per year
Mal-Khalife Basin	64	9	1 well for every 7 km^2	0.33	89%	33%
Western Huaihe Plain (Huaihe River Basin)	29254	23	1 well for every 1272 km^2	0.33	91%	22%
Antelope Plain and Lost Hills (Tulare Basin of the Central Valley)	1457	75	1 well for every 19 km^2	0.33	92%	21%
Raft River Valley (Central Snake River Plain)	1492	22	1 well for every 68 km^2	0.33	91%	18%
Ravansar Plain	1142	42	1 well for every 27 km^2	0.32	79%	40%
Corning Basin (Sacramento Valley of the Central Valley)	663	184	1 well for every 4 km^2	0.32	95%	24%
Dasht-e Rostam Basin	83	13	1 well for every 6 km^2	0.32	100%	31%
Dalgan Basin (Jazmurian Depression)	2178	22	1 well for every 99 km^2	0.32	95%	5%
Lash Basin	548	15	1 well for every 37 km^2	0.32	100%	27%
Biyarjomand Basin	539	25	1 well for every 22 km^2	0.32	96%	36%
Northern Nallamalai Basin (Cuddapah Sedimentary Basin)	5534	1	1 well for every 5534 km^2	0.32	100%	0%
Western Karnataka Schist Aquifers	6952	37	1 well for every 188 km^2	0.32	84%	35%
Rodan Basin	259	19	1 well for every 14 km^2	0.32	84%	5%
Northern Colusa Basin (Sacramento Valley of the Central Valley)	677	153	1 well for every 4 km^2	0.31	96%	31%
Coastal Saurashtra Miliolite Limestone Aquifer	3064	37	1 well for every 83 km^2	0.31	92%	35%
Central Caborca Aquifer (Caborca Aquifer System)	4074	10	1 well for every 407 km^2	0.31	80%	30%
Guerrero-Yepomera Aquifer	2203	3	1 well for every 734 km^2	0.31	67%	33%
Iranshahr Plain (Jazmurian Depression)	2276	46	1 well for every 49 km^2	0.31	89%	11%
Lower Souss Basin (Souss-Chtouka Basin)	1230	3	1 well for every 410 km^2	0.31	67%	33%
Rio Zanjon Basin	2339	8	1 well for every 292 km^2	0.30	88%	13%
Bone Spring-Victorio Peak Aquifer and Dell Valley	1469	29	1 well for every 51 km^2	0.30	100%	3%
Shazand Plain	430	23	1 well for every 19 km^2	0.30	87%	30%
Siriz Basin	707	11	1 well for every 64 km^2	0.30	91%	27%
Tashkoyeh Basin	74	5	1 well for every 15 km^2	0.30	60%	40%
Chahghah Basin	483	18	1 well for every 27 km^2	0.30	67%	22%
Northern Dezful-Andimeshk Plain (Dezful-Andimeshk Plain)	826	33	1 well for every 25 km^2	0.30	91%	18%
Campo de Belchite and Cubeta de Azuara Aquifers	999	3	1 well for every 333 km^2	0.29	67%	0%
Koohpayeh Basin (Lower Zayandeh-Rud Basin)	4756	107	1 well for every 44 km^2	0.29	90%	35%
Southern Tule Lake (Upper Klamath Basin)	145	21	1 well for every 7 km^2	0.29	95%	5%
Letaba Lowveld	15251	16	1 well for every 953 km^2	0.29	88%	6%
Chahak Musaviyeh Basin	1713	18	1 well for every 95 km^2	0.29	100%	22%
Shandan Basin and Middle Heihe Reaches (Heihe Basin)	4119	1	1 well for every 4119 km^2	0.29	100%	0%
Poza Rica Aquifer	1426	3	1 well for every 475 km^2	0.28	67%	0%
Pomfret-Vergelegen Dolomitic Aquifer	4607	43	1 well for every 107 km^2	0.28	65%	30%
Korron Basin	1850	64	1 well for every 29 km^2	0.28	92%	34%
Meymand Aquifer	170	16	1 well for every 11 km^2	0.28	94%	44%
Southeastern Divandareh-Bijar Basin	875	25	1 well for every 35 km^2	0.28	84%	16%
Nanyang Basin	21139	14	1 well for every 1510 km^2	0.28	86%	14%
Northcentral Coromandel Schist Aquifers (Coromandel Coastal Plain)	10679	13	1 well for every 821 km^2	0.28	100%	31%
Western Bengal Basin (Bengal Basin)	18026	119	1 well for every 151 km^2	0.28	90%	30%
Penoyer Valley	696	10	1 well for every 70 km^2	0.27	100%	0%

Study area	Area (km^2)	Number of monitoring wells	Density of monitoring wells (with sufficient groundwater level observations for analyses) within the study area	Median Theil-Sen slope among monitoring wells *(positive values indicate deeper depths to water over time)*	Percent of wells with Theil-Sen slopes of >0 m per year	Percent of wells with Theil-Sen slopes of >0.5 m per year
Milford-Blackrock Subarea (Escalante Valley)	1538	32	1 well for every 48 km^2	0.27	88%	3%
Southwestern Khorat Plateau (Khorat Plateau Aquifer System)	17185	2	1 well for every 8593 km^2	0.27	100%	0%
Northern Hosein Abad Basin	1382	17	1 well for every 81 km^2	0.27	100%	0%
Northwestern Karnataka Schist Aquifers	6578	23	1 well for every 286 km^2	0.27	83%	22%
Bimorgh Basin	1553	22	1 well for every 71 km^2	0.27	95%	18%
Hamedan-Bahar Plain	1548	29	1 well for every 53 km^2	0.27	76%	31%
Ataiyah Basin	1510	34	1 well for every 44 km^2	0.27	97%	3%
Varamin Aquifer (Tehran Plain)	1434	91	1 well for every 16 km^2	0.27	73%	44%
Shahr-Babak Plain	2943	71	1 well for every 41 km^2	0.27	97%	8%
Kahria Aquifer	133	14	1 well for every 10 km^2	0.27	93%	29%
Rashakan Basin	27	4	1 well for every 7 km^2	0.27	100%	25%
Eastern El Palmar Aquifer	1194	2	1 well for every 597 km^2	0.27	50%	50%
Grass Valley in northern Nevada	580	1	1 well for every 580 km^2	0.26	100%	0%
Takht Deraz Basin	32	5	1 well for every 6 km^2	0.26	100%	0%
Sarvestan Plain	1105	30	1 well for every 37 km^2	0.26	93%	30%
Theni Fractured Charnockite and Gneiss Aquifers	3027	13	1 well for every 233 km^2	0.26	69%	38%
Southern Darz Sayebn Basin	140	9	1 well for every 16 km^2	0.26	67%	22%
Meymeh Plain	589	17	1 well for every 35 km^2	0.26	94%	18%
Missionary Plains Syncline and Mereenie Anticline (Amadeus Basin)	22819	14	1 well for every 1630 km^2	0.26	93%	21%
Eastern Villa Garcia Aquifer	54	12	1 well for every 5 km^2	0.25	100%	17%
Ferdows Plains	604	16	1 well for every 38 km^2	0.25	100%	19%
Upper Santa Ynez Valley (Santa Ynez Valley)	369	115	1 well for every 3 km^2	0.25	97%	23%
Northern Piedmont Plain (North China Plain)	9339	50	1 well for every 187 km^2	0.25	76%	32%
Hertfordshire and Cambridgeshire Chalk Aquifer (Chalk Aquifer System)	2326	1	1 well for every 2326 km^2	0.25	100%	0%
Edwards Creek Valley	586	1	1 well for every 586 km^2	0.25	100%	0%
Khezri Plain	901	16	1 well for every 56 km^2	0.25	100%	38%
Central Culverden Basin (Culverden Basin)	343	7	1 well for every 49 km^2	0.24	86%	0%
Upper Cauvery Banded Gneiss Aquifers (Dharwar Craton Banded Gneiss Complex)	34251	220	1 well for every 156 km^2	0.24	79%	26%
Palakkad Gap Fractured Charnockite and Gneiss Aquifers	3022	38	1 well for every 80 km^2	0.24	95%	21%
Upper Rustaq-Fork Basin	96	7	1 well for every 14 km^2	0.24	57%	29%
Central Maputaland Coastal Plain (Maputaland Coastal Plain)	6950	4	1 well for every 1738 km^2	0.24	100%	0%
Playas Valley	1523	2	1 well for every 762 km^2	0.24	100%	0%
Salvatierra-Acambara and Eastern Cienega Prieta-Moroleon Aquifers	750	4	1 well for every 188 km^2	0.24	75%	25%
Shahreza Plain	1495	25	1 well for every 60 km^2	0.24	92%	36%
Northern Maputaland Coastal Plain (Maputaland Coastal Plain)	3670	1	1 well for every 3670 km^2	0.24	100%	0%
Southeastern Coastal Karoo Basin (Main Karoo Basin)	28053	2	1 well for every 14027 km^2	0.23	100%	0%
Piceance Basin Aquifer System	1773	4	1 well for every 443 km^2	0.23	50%	50%
Lower Qeydar Basin (Qeydar Basin)	702	17	1 well for every 41 km^2	0.23	88%	35%
Southern Guadalupe Garzaron Aquifer	465	4	1 well for every 116 km^2	0.23	75%	0%
Enid Isolated Terrace Aquifer	163	7	1 well for every 23 km^2	0.23	100%	0%
Crow Flats	429	6	1 well for every 72 km^2	0.23	100%	0%

Study area	Area (km²)	Number of monitoring wells	Density of monitoring wells (with sufficient groundwater level observations for analyses) within the study area	Median Theil-Sen slope among monitoring wells *(positive values indicate deeper depths to water over time)*	Percent of wells with Theil-Sen slopes of >0 m per year	Percent of wells with Theil-Sen slopes of >0.5 m per year
Dasht e- Afzar Basin	156	17	1 well for every 9 km²	0.23	71%	35%
Soufian-Shabestar Aquifer	308	27	1 well for every 11 km²	0.23	89%	30%
Azapa Aquifer	161	3	1 well for every 54 km²	0.23	67%	33%
Long Valley	570	1	1 well for every 570 km²	0.23	100%	0%
Northcentral Deccan Basalt Plateau (Deccan Plateau)	53372	48	1 well for every 1112 km²	0.23	73%	15%
Bastam Plain	533	32	1 well for every 17 km²	0.23	97%	38%
Central Alluvial-Laucustrine Plain (North China Plain)	38442	42	1 well for every 915 km²	0.23	71%	40%
Kurnool Basin (Cuddapah Sedimentary Basin)	11364	17	1 well for every 668 km²	0.23	82%	18%
Dastgerdan Basin	1082	20	1 well for every 54 km²	0.23	100%	10%
Thames Valley and London Basin (Chalk Aquifer System)	7326	2	1 well for every 3663 km²	0.22	100%	0%
Wadi As-Sirhan Basin	68037	4	1 well for every 17009 km²	0.22	100%	0%
Namakkal Fractured Charnockite and Gneiss Aquifers (Coimbatore Upland)	3663	22	1 well for every 167 km²	0.22	82%	23%
Gareh-Bygone Plain	466	20	1 well for every 23 km²	0.22	90%	20%
Abdan-Dayer Coastal Plain	446	48	1 well for every 9 km²	0.22	94%	6%
Southcentral Mega Aquifer System (Mega Aquifer System)	89916	9	1 well for every 9991 km²	0.22	89%	44%
Tuyserkan Plain	301	16	1 well for every 19 km²	0.22	81%	31%
Eastern Libres-Oriental Aquifer (Libres-Oriental Aquifer)	229	1	1 well for every 229 km²	0.22	100%	0%
Nehbandan Aquifer	634	12	1 well for every 53 km²	0.22	83%	17%
Mehrgerd Semirom	452	30	1 well for every 15 km²	0.21	77%	33%
Dehrom Basin	187	18	1 well for every 10 km²	0.21	89%	28%
Utah Lake Valley	384	35	1 well for every 11 km²	0.21	91%	9%
Butte Valley (Upper Klamath Basin)	557	30	1 well for every 19 km²	0.21	67%	0%
Bavanat Basin	278	12	1 well for every 23 km²	0.21	100%	25%
Sarbisheh Basin	392	9	1 well for every 44 km²	0.21	100%	0%
Bazman Basin (Jazmurian Depression)	2508	24	1 well for every 105 km²	0.21	100%	0%
Tehran-Karaj Plain (Tehran Plain)	3110	125	1 well for every 25 km²	0.21	69%	37%
Southcentral Coromandel Fractured Gneiss Aquifers (Coromandel Coastal Plain)	9508	56	1 well for every 170 km²	0.20	88%	18%
Porbander Coastal Region (Saurashtra Deccan Trap Basalts)	2271	18	1 well for every 126 km²	0.20	89%	39%
Northcentral Mega Aquifer System (Mega Aquifer System)	179365	1	1 well for every 179365 km²	0.20	100%	0%
Boein-Miandasht Aquifer	452	28	1 well for every 16 km²	0.20	93%	18%
North-central High Plains (High Plains)	18173	221	1 well for every 82 km²	0.20	89%	21%
Kordkandi-Duzduzan Plain	338	22	1 well for every 15 km²	0.20	86%	18%
Western Dehshir Basin	1019	17	1 well for every 60 km²	0.20	100%	12%
Northern Saurashtra Deccan Trap Basalts (Saurashtra Deccan Trap Basalts)	11532	1	1 well for every 11532 km²	0.20	100%	0%
Daryache Namak Basin	379	6	1 well for every 63 km²	0.20	100%	17%
Shoqan Plain	330	9	1 well for every 37 km²	0.20	100%	11%
Santo Domingo Coastal Aquifer	6050	47	1 well for every 129 km²	0.20	87%	17%
Central Urucuia Aquifer (Urucuia Aquifer System)	31045	8	1 well for every 3881 km²	0.20	100%	25%
Upper Rio Petorca Basin (Rio Petorca Basin)	181	6	1 well for every 30 km²	0.20	100%	17%
Valle de Escuinapa	202	2	1 well for every 101 km²	0.20	100%	0%

Study area	Area (km²)	Number of monitoring wells	Density of monitoring wells (with sufficient groundwater level observations for analyses) within the study area	Median Theil-Sen slope among monitoring wells *(positive values indicate deeper depths to water over time)*	Percent of wells with Theil-Sen slopes of >0 m per year	Percent of wells with Theil-Sen slopes of >0.5 m per year
Robat-Khorramabad Plain	346	10	1 well for every 35 km²	0.19	100%	10%
Western Karnataka Laterites	1106	8	1 well for every 138 km²	0.19	88%	0%
Railroad Valley	2142	2	1 well for every 1071 km²	0.19	100%	0%
Mid-Waitaki Valley	262	1	1 well for every 262 km²	0.19	100%	0%
Mobarakiyeh Basin (Tehran Plain)	310	6	1 well for every 52 km²	0.19	83%	0%
Wild Horse and Michigan Flats (Salt Basin Bolson)	989	14	1 well for every 71 km²	0.19	93%	7%
Loreto Aquifer	553	4	1 well for every 138 km²	0.19	75%	25%
Bostanabad Basin	426	18	1 well for every 24 km²	0.19	89%	22%
Southern Coimbatore Fractured Charnockite and Gneiss Aquifers (Coimbatore Upland)	6549	38	1 well for every 172 km²	0.19	89%	18%
Borazjan Aquifer	2266	52	1 well for every 44 km²	0.19	85%	25%
Roswell Basin	6539	166	1 well for every 39 km²	0.19	87%	10%
Southern Dharwar Craton Banded Gneiss Complex (Dharwar Craton Banded Gneiss Complex)	46406	158	1 well for every 294 km²	0.19	72%	31%
Valle de Juarez and Hueco Bolson (Tularosa-Huelco)	6161	112	1 well for every 55 km²	0.19	91%	24%
Toole Valley	575	46	1 well for every 13 km²	0.18	96%	11%
Villanueva Aquifer	501	19	1 well for every 26 km²	0.18	79%	21%
Urumqi Valley (Junggar Basin)	6794	4	1 well for every 1699 km²	0.18	75%	25%
Eastern Elsinore Valley	31	68	1 well for every 0 km²	0.18	94%	4%
Mazapil and Cedros Basin	2096	10	1 well for every 210 km²	0.18	90%	30%
Bandar Lengeh Basin	1768	7	1 well for every 253 km²	0.18	100%	14%
Castellon Plain	1038	21	1 well for every 49 km²	0.18	76%	24%
Ahmadi Basin	61	9	1 well for every 7 km²	0.18	89%	0%
Almanzora Basin	422	2	1 well for every 211 km²	0.18	50%	0%
Jahrom Basin	865	24	1 well for every 36 km²	0.18	92%	38%
Ghir Karzin Basin	141	12	1 well for every 12 km²	0.18	100%	33%
Northeastern Libres-Oriental Aquifer (Libres-Oriental Aquifer)	264	2	1 well for every 132 km²	0.18	100%	0%
Central Dharwar Craton Banded Gneiss Complex (Dharwar Craton Banded Gneiss Complex)	81286	303	1 well for every 268 km²	0.18	77%	22%
Pauma Valley	36	11	1 well for every 3 km²	0.17	73%	27%
Abrego Aquifer	410	16	1 well for every 26 km²	0.17	63%	38%
Lachlan Alluvium	21569	249	1 well for every 87 km²	0.17	86%	8%
Western Elsinore Valley	44	23	1 well for every 2 km²	0.17	96%	0%
Eastern Poti and Piaui and Cabecas and Serra Grande Aquifers (Parnaiba Basin)	117926	9	1 well for every 13103 km²	0.17	89%	33%
Central Coromandel Fractured Charnockite and Gneiss Aquifers (Coromandel Coastal Plain)	17492	110	1 well for every 159 km²	0.17	83%	12%
Western Rezvan Basin	27	6	1 well for every 5 km²	0.17	100%	0%
Central Kachchh Coastal Plain (Kachchh Coastal Plain)	3760	13	1 well for every 289 km²	0.17	85%	0%
Northcentral Coromandel Coastal Alluvial Aquifer (Coromandel Coastal Plain)	4924	14	1 well for every 352 km²	0.17	86%	0%
Central Plain (Central Snake River Plain)	9551	298	1 well for every 32 km²	0.17	84%	3%
Khane Ket Basin	326	11	1 well for every 30 km²	0.17	82%	18%
Western San Gabriel Valley (San Gabriel Valley)	191	131	1 well for every 1 km²	0.17	80%	24%
Aguilas Basin	158	3	1 well for every 53 km²	0.17	100%	33%

Study area	Area (km²)	Number of monitoring wells	Density of monitoring wells (with sufficient groundwater level observations for analyses) within the study area	Median Theil-Sen slope among monitoring wells *(positive values indicate deeper depths to water over time)*	Percent of wells with Theil-Sen slopes of >0 m per year	Percent of wells with Theil-Sen slopes of >0.5 m per year
Antelope Basin (Sacramento Valley of the Central Valley)	82	29	1 well for every 3 km²	0.17	97%	0%
Cadenas Marchigue y Yerbas Buenas	446	5	1 well for every 89 km²	0.17	100%	40%
Central Huaihe Plain (Huaihe River Basin)	79573	61	1 well for every 1304 km²	0.17	82%	21%
Gerash Basin	149	9	1 well for every 17 km²	0.17	78%	22%
Southern Cauvery Delta and Orathanadu Aquifer System (Coromandel Coastal Plain)	7850	37	1 well for every 212 km²	0.17	86%	5%
Southern Deccan Basalt Plateau (Deccan Plateau)	23112	78	1 well for every 296 km²	0.17	79%	14%
Hill Country (Edwards-Trinity Aquifer System)	14375	365	1 well for every 39 km²	0.17	83%	29%
Southcentral Deccan Basalt Plateau (Deccan Plateau)	57686	95	1 well for every 607 km²	0.17	82%	11%
Sevier Desert	2562	62	1 well for every 41 km²	0.17	97%	10%
Turlock Basin (San Joaquin Basin of the Central Valley)	1425	405	1 well for every 4 km²	0.17	91%	11%
Wiggertal Valley	62	1	1 well for every 62 km²	0.17	100%	0%
Western Carrizo-Wilcox (Carrizo-Wilcox)	43394	433	1 well for every 100 km²	0.17	71%	28%
Vina Basin (Sacramento Valley of the Central Valley)	828	190	1 well for every 4 km²	0.17	92%	2%
Trans Gangetic Plain (Indo-Gangetic Plain)	100322	422	1 well for every 238 km²	0.17	72%	27%
Azna-Aligudarz Basin	1090	37	1 well for every 29 km²	0.17	62%	38%
Darulaman-Pagh Basin (Kabul Basin)	207	9	1 well for every 23 km²	0.16	89%	11%
Khafr Basin	532	25	1 well for every 21 km²	0.16	92%	24%
Northern Sarkhoon Aquifer (Sarkhoon Aquifer)	243	16	1 well for every 15 km²	0.16	100%	13%
El Salvador Aquifer	159	4	1 well for every 40 km²	0.16	100%	0%
Chennai Coastal Aquifer (Coromandel Coastal Plain)	2686	20	1 well for every 134 km²	0.16	70%	5%
Northern Cauvery Delta and Cuddalore Sandstone Aquifer System (Coromandel Coastal Plain)	8539	67	1 well for every 127 km²	0.16	78%	15%
Southern Sarbisheh Basin	479	9	1 well for every 53 km²	0.16	100%	0%
Upper Kabootar Ahang Basin (Razan Basin)	650	15	1 well for every 43 km²	0.16	80%	40%
Southern Zona Citricola Aquifer (Gulf Coast Regional Aquifer System)	2921	70	1 well for every 42 km²	0.16	81%	13%
Bedford-Coldwater Valley	28	6	1 well for every 5 km²	0.16	83%	17%
Uvas Valley	225	4	1 well for every 56 km²	0.16	50%	25%
Lenjanat Basin	2658	42	1 well for every 63 km²	0.16	95%	7%
El Llano Basin	1026	8	1 well for every 128 km²	0.16	75%	13%
Mud Lake (Eastern Snake River Plain)	3202	104	1 well for every 31 km²	0.16	96%	4%
Indian Wells Valley	884	67	1 well for every 13 km²	0.16	69%	1%
Sidon Basin	36	8	1 well for every 5 km²	0.16	88%	0%
Warner Valley	121	1	1 well for every 121 km²	0.15	100%	0%
Jayezan Basin	236	18	1 well for every 13 km²	0.15	78%	0%
Agua Verde Aquifer	931	10	1 well for every 93 km²	0.15	60%	20%
Eastern Satpura Basalt Aquifers (Satpura Basalts)	10338	10	1 well for every 1034 km²	0.15	60%	10%
Eastern Santa Clara River Valley	196	145	1 well for every 1 km²	0.15	79%	22%
Semelghan Plain	288	17	1 well for every 17 km²	0.15	82%	24%
Ti Tree Basin	5368	3	1 well for every 1789 km²	0.15	100%	0%
Lhasa Valley	425	8	1 well for every 53 km²	0.15	100%	0%
Cahuilla Valley	87	49	1 well for every 2 km²	0.15	90%	2%

Study area	Area (km^2)	Number of monitoring wells	Density of monitoring wells (with sufficient groundwater level observations for analyses) within the study area	Median Theil-Sen slope among monitoring wells *(positive values indicate deeper depths to water over time)*	Percent of wells with Theil-Sen slopes of >0 m per year	Percent of wells with Theil-Sen slopes of >0.5 m per year
Temescal Basin (Upper Santa Ana Basin)	74	13	1 well for every 6 km^2	0.15	100%	0%
Xinachuan Area (Huangshui Basin)	44	1	1 well for every 44 km^2	0.15	100%	0%
Ladiz Basin	196	7	1 well for every 28 km^2	0.15	100%	0%
Lower Rio Elqui Basin (Elqui River Basin)	400	10	1 well for every 40 km^2	0.15	80%	30%
Southern Maputaland Coastal Plain (Maputaland Coastal Plain)	2628	14	1 well for every 188 km^2	0.15	79%	0%
North West Dolomite Aquifer System	8037	99	1 well for every 81 km^2	0.15	75%	20%
Mhamid Oasis (Middle Draa Basin)	126	4	1 well for every 32 km^2	0.14	100%	0%
Southern High Plains (High Plains)	74884	3143	1 well for every 24 km^2	0.14	81%	17%
Southeastern Jazmurian Depression (Jazmurian Depression)	4726	52	1 well for every 91 km^2	0.14	94%	4%
Saint Francis Coastal Aquifer	933	6	1 well for every 156 km^2	0.14	83%	17%
Balcones Fault Zone (Edwards-Trinity Aquifer System)	6663	160	1 well for every 42 km^2	0.14	67%	33%
Southern Goose Lake Valley	144	7	1 well for every 21 km^2	0.14	100%	0%
Darolmizan Basin	169	21	1 well for every 8 km^2	0.14	86%	0%
Cuchillos-Cabras and El Molar Aquifers	625	16	1 well for every 39 km^2	0.14	63%	25%
Southern Razan Plain (Razan Basin)	641	16	1 well for every 40 km^2	0.14	75%	31%
Myghan Basin	1134	13	1 well for every 87 km^2	0.14	100%	0%
Central Coastal Deccan Traps	2741	12	1 well for every 228 km^2	0.14	92%	8%
Northern Riverine Plains (Murray Basin)	25646	144	1 well for every 178 km^2	0.14	93%	5%
Central Pilbara Granite-Greenstone Terrane (Pilbara Craton)	18871	2	1 well for every 9436 km^2	0.14	100%	0%
Eastern Dehshir and Arnan Basins	968	17	1 well for every 57 km^2	0.14	94%	0%
Brahmani Delta and Northern Utkal Coastal Plain (Utkal Coastal Plain)	8137	6	1 well for every 1356 km^2	0.14	83%	0%
Southwest Rayong Basin (Rayong Basin)	218	3	1 well for every 73 km^2	0.14	100%	0%
Panoche Valley	71	39	1 well for every 2 km^2	0.14	74%	0%
Koriyan Basin	311	7	1 well for every 44 km^2	0.13	86%	14%
Western Tapi Valley Alluvial Aquifer (Tapi Valley Alluvial Aquifer)	8376	6	1 well for every 1396 km^2	0.13	83%	17%
Tularosa Basin (Tularosa-Huelco)	8748	25	1 well for every 350 km^2	0.13	76%	0%
Miandarband Plain	355	29	1 well for every 12 km^2	0.13	72%	10%
Izad Khast Basin	637	15	1 well for every 42 km^2	0.13	73%	20%
Mahanadi Delta (Utkal Coastal Plain)	8385	41	1 well for every 205 km^2	0.13	88%	0%
Barind Tract and Central Floodplains (Bengal Basin)	23481	135	1 well for every 174 km^2	0.13	92%	7%
Lonesome and Prescott Valleys	254	2	1 well for every 127 km^2	0.13	50%	0%
Pend Orielle East	328	1	1 well for every 328 km^2	0.13	100%	0%
Villa de Arriaga and Pino Suarez Aquifers	1163	9	1 well for every 129 km^2	0.13	100%	11%
Taipei Basin	292	28	1 well for every 10 km^2	0.13	79%	4%
San Simon Valley	1701	7	1 well for every 243 km^2	0.13	86%	0%
Red River Aquifer	5879	47	1 well for every 125 km^2	0.13	91%	9%
Eshtehard Basin	518	23	1 well for every 23 km^2	0.13	78%	26%
Tiruvadanai Aquifer System (Coromandel Coastal Plain)	8346	38	1 well for every 220 km^2	0.13	92%	5%
Zapotitan Aquifer	394	3	1 well for every 131 km^2	0.13	67%	33%
Mahanadi Basin (Son-Mahandai Valley Basins)	6162	21	1 well for every 293 km^2	0.13	76%	5%
Upper Nowbaran Basin	586	18	1 well for every 33 km^2	0.13	72%	11%

Study area	Area (km²)	Number of monitoring wells	Density of monitoring wells (with sufficient groundwater level observations for analyses) within the study area	Median Theil-Sen slope among monitoring wells *(positive values indicate deeper depths to water over time)*	Percent of wells with Theil-Sen slopes of >0 m per year	Percent of wells with Theil-Sen slopes of >0.5 m per year
Sirik Basin	77	10	1 well for every 8 km²	0.13	100%	0%
Qaen Aquifer	290	7	1 well for every 41 km²	0.13	86%	43%
Sarab Plain	937	73	1 well for every 13 km²	0.13	84%	18%
Western Nakambe Basin (Baoule-Mossi Domain of the southern West African Craton)	6022	5	1 well for every 1204 km²	0.13	60%	0%
Bari Doab (Indus Basin)	39776	405	1 well for every 98 km²	0.12	77%	1%
Central Basin (Los Angeles Basin)	747	4757	1 well for every 0 km²	0.12	91%	1%
Northern Karoo Basin (Main Karoo Basin)	89072	33	1 well for every 2699 km²	0.12	67%	9%
Limache Aquifer	197	2	1 well for every 99 km²	0.12	100%	0%
Central Malabar Khondalite and Charnockite Aquifers (Malabar Coastal Plain)	4052	108	1 well for every 38 km²	0.12	87%	5%
Eastern Mekong Delta (Mekong Delta)	35001	28	1 well for every 1250 km²	0.12	79%	18%
Eastern Tapi Valley Alluvial Aquifer (Tapi Valley Alluvial Aquifer)	9162	4	1 well for every 2291 km²	0.12	50%	50%
Naqadeh Plain	497	41	1 well for every 12 km²	0.12	88%	5%
Northern Razan Plain (Razan Basin)	1073	44	1 well for every 24 km²	0.12	80%	14%
Blok -e Sharra Basin (Razan Basin)	473	17	1 well for every 28 km²	0.12	82%	6%
Eastern Parecis Basin (Parecis Basin)	118059	1	1 well for every 118059 km²	0.12	100%	0%
Dehkyueh Basin	170	14	1 well for every 12 km²	0.12	93%	14%
Los Molinos Basin (Sacramento Valley of the Central Valley)	352	74	1 well for every 5 km²	0.12	97%	0%
Cosumnes Basin (San Joaquin Basin of the Central Valley)	643	74	1 well for every 9 km²	0.12	84%	1%
Teton Valley	1534	15	1 well for every 102 km²	0.12	53%	13%
Allah Marvdasht Basin	895	28	1 well for every 32 km²	0.12	96%	14%
Puangue Aquifer	553	6	1 well for every 92 km²	0.12	83%	0%
Southcentral Karoo Basin (Main Karoo Basin)	72357	56	1 well for every 1292 km²	0.12	70%	18%
Santiago Coastal Aquifer	675	7	1 well for every 96 km²	0.12	71%	14%
Ostuta Aquifer	1174	18	1 well for every 65 km²	0.12	83%	6%
Sahlabad Basin	1148	23	1 well for every 50 km²	0.11	96%	0%
Paradise Valley in northern Nevada	1121	3	1 well for every 374 km²	0.11	67%	0%
Eastern Cambay Basin (Cambay Basin)	8466	39	1 well for every 217 km²	0.11	56%	10%
Central Littoral Plain (North China Plain)	11974	20	1 well for every 599 km²	0.11	65%	35%
Tabas Basin	2723	27	1 well for every 101 km²	0.11	96%	7%
Pahrump Valley	1407	37	1 well for every 38 km²	0.11	78%	3%
Klamath Valley (Upper Klamath Basin)	284	15	1 well for every 19 km²	0.11	93%	0%
Daly Basin (Daly-Wiso-Georgina Basins)	26597	43	1 well for every 619 km²	0.11	79%	14%
Little Bitterroot Valley (Flathead Valley)	360	1	1 well for every 360 km²	0.11	100%	0%
Jackson Hole Aquifer	343	18	1 well for every 19 km²	0.11	94%	0%
Mangaluru Coastal Aquifer System	9331	130	1 well for every 72 km²	0.11	90%	3%
Northern Culverden Basin (Culverden Basin)	344	1	1 well for every 344 km²	0.11	100%	0%
Kozhikkode Coastal Aquifer (Malabar Coastal Plain)	137	7	1 well for every 20 km²	0.11	86%	0%
Northern Aconcagua Basin (Aconcagua Basin)	116	1	1 well for every 116 km²	0.11	100%	0%
Shahdad Basin	1982	9	1 well for every 220 km²	0.11	89%	11%
Coonamble Embayment (Great Artesian Basin)	65411	792	1 well for every 83 km²	0.11	80%	1%
Bukan Basin	957	30	1 well for every 32 km²	0.11	87%	3%

Study area	Area (km^2)	Number of monitoring wells	Density of monitoring wells (with sufficient groundwater level observations for analyses) within the study area	Median Theil-Sen slope among monitoring wells *(positive values indicate deeper depths to water over time)*	Percent of wells with Theil-Sen slopes of >0 m per year	Percent of wells with Theil-Sen slopes of >0.5 m per year
Kafehtaghestan Basin	1055	12	1 well for every 88 km^2	0.11	75%	8%
Puerto Madero Aquifer	1520	16	1 well for every 95 km^2	0.11	63%	13%
Chaleant Valley	172	4	1 well for every 43 km^2	0.11	100%	0%
Kern River Valley	131	12	1 well for every 11 km^2	0.11	83%	8%
Northern Dharwar Craton Banded Gneiss Complex (Dharwar Craton Banded Gneiss Complex)	61216	74	1 well for every 827 km^2	0.11	72%	20%
Requena-Utiel Aquifer	502	8	1 well for every 63 km^2	0.11	88%	13%
Tietar Valley Aquifer	2721	8	1 well for every 340 km^2	0.10	63%	13%
Northern Georgina Basin (Daly-Wiso-Georgina Basins)	116661	1	1 well for every 116661 km^2	0.10	100%	0%
Mesilla Valley (Rincon-Mesilla Valleys)	980	207	1 well for every 5 km^2	0.10	88%	5%
Eastern Kachchh Coastal Plain (Kachchh Coastal Plain)	1290	3	1 well for every 430 km^2	0.10	67%	0%
Lower Aconcagua Basin (Aconcagua Basin)	200	14	1 well for every 14 km^2	0.10	86%	0%
Searles Valley	627	3	1 well for every 209 km^2	0.10	67%	0%
Walker Basin Creek Valley	23	11	1 well for every 2 km^2	0.10	64%	18%
Southern Riverine Plains (Murray Basin)	51599	4073	1 well for every 13 km^2	0.10	80%	1%
Norfolk Chalk Aquifer (Chalk Aquifer System)	3402	1	1 well for every 3402 km^2	0.10	100%	0%
Lower Rio Petorca Basin (Rio Petorca Basin)	121	3	1 well for every 40 km^2	0.10	100%	0%
North Okanagan (Okanagan Basin)	202	4	1 well for every 51 km^2	0.10	100%	0%
Santa Maria Basin and Nipomo Valley	678	590	1 well for every 1 km^2	0.10	93%	15%
Urmia Aquifer	1110	66	1 well for every 17 km^2	0.10	86%	5%
Goose Creek and Golden Valley (Central Snake River Plain)	1422	44	1 well for every 32 km^2	0.10	68%	18%
Tabriz Plain	3080	214	1 well for every 14 km^2	0.10	72%	9%
Dezghan Basin	1458	21	1 well for every 69 km^2	0.10	71%	5%
Lower Fortescue Valley (Hamersley Basin)	5590	5	1 well for every 1118 km^2	0.10	100%	0%
Riverside-Arlington Basin (Upper Santa Ana Basin)	233	324	1 well for every 1 km^2	0.10	94%	0%
Northern Jalpa-Juchipila Aquifer	660	11	1 well for every 60 km^2	0.10	64%	27%
San Timoteo Basin	84	53	1 well for every 2 km^2	0.10	58%	34%
Guaymas Valley	1868	34	1 well for every 55 km^2	0.10	53%	15%
La Paz Coastal Aquifer (La Paz Basin)	200	58	1 well for every 3 km^2	0.10	90%	3%
Southern Hosein Abad Basin	873	4	1 well for every 218 km^2	0.10	100%	0%
Northern Hirri Basin (Chhattisgarh Basin)	14830	82	1 well for every 181 km^2	0.10	63%	9%
Limpopo Plateau	10209	35	1 well for every 292 km^2	0.09	77%	6%
Aqda Basin	1222	31	1 well for every 39 km^2	0.09	90%	6%
Northern Malabar Khondalite and Charnockite Aquifers (Malabar Coastal Plain)	1911	42	1 well for every 46 km^2	0.09	93%	0%
Lampang Basin	1847	10	1 well for every 185 km^2	0.09	60%	0%
Kenwood Valley	19	6	1 well for every 3 km^2	0.09	67%	0%
Eastern Plain (Eastern Snake River Plain)	8022	398	1 well for every 20 km^2	0.09	90%	2%
Tillman Terrace	761	22	1 well for every 35 km^2	0.09	82%	5%
Bojnourd Plain	152	12	1 well for every 13 km^2	0.09	75%	0%
South-central High Plains (High Plains)	14464	982	1 well for every 15 km^2	0.09	80%	11%
Nosrat Abad Basin	347	13	1 well for every 27 km^2	0.09	100%	0%
Fezouata Oasis (Middle Draa Basin)	492	4	1 well for every 123 km^2	0.09	75%	0%

Study area	Area (km²)	Number of monitoring wells	Density of monitoring wells (with sufficient groundwater level observations for analyses) within the study area	Median Theil-Sen slope among monitoring wells *(positive values indicate deeper depths to water over time)*	Percent of wells with Theil-Sen slopes of >0 m per year	Percent of wells with Theil-Sen slopes of >0.5 m per year
Pirie Basin	345	24	1 well for every 14 km²	0.09	79%	0%
North San Benito (Gilroy-Hollister Valley)	326	169	1 well for every 2 km²	0.09	76%	10%
Hasdo-Arand Basin (Son-Mahandai Valley Basins)	7733	32	1 well for every 242 km²	0.09	75%	6%
Kavir Namak Plain	1151	15	1 well for every 77 km²	0.09	87%	0%
Upper Deschutes Basin	12455	24	1 well for every 519 km²	0.09	88%	4%
Red River Delta	11968	24	1 well for every 499 km²	0.09	79%	0%
El Sahuaral Aquifer	346	18	1 well for every 19 km²	0.09	72%	11%
Cara Sucia-San Pedro Aquifer	473	3	1 well for every 158 km²	0.09	67%	0%
Tepalcingo-Axochiapan Aquifer	465	62	1 well for every 8 km²	0.09	63%	27%
Hetao Basin	13190	1	1 well for every 13190 km²	0.09	100%	0%
Gypsum Hills	13266	10	1 well for every 1327 km²	0.09	90%	10%
Willchra Basin	2243	24	1 well for every 93 km²	0.08	79%	0%
South Chianan Plain (Chianan Plain)	797	37	1 well for every 22 km²	0.08	78%	16%
Seymour-Blaine Aquifer System	38612	272	1 well for every 142 km²	0.08	77%	8%
Vidalia Upland (Floridan Aquifer System)	23831	55	1 well for every 433 km²	0.08	60%	9%
Northern Tule Lake (Upper Klamath Basin)	579	104	1 well for every 6 km²	0.08	93%	6%
Limari Basin	632	6	1 well for every 105 km²	0.08	83%	0%
Western Brahmaputra Basin Aquifer (Brahmaputra Basin Aquifer)	33634	10	1 well for every 3363 km²	0.08	90%	0%
Eastern Deccan Basalt Plateau (Deccan Plateau)	21575	48	1 well for every 449 km²	0.08	67%	13%
Gonabad Plain	1293	16	1 well for every 81 km²	0.08	81%	0%
Ivanhoe and Packsaddle Plains	502	58	1 well for every 9 km²	0.08	81%	0%
Shahindej Basin	757	45	1 well for every 17 km²	0.08	84%	11%
Lower Santa Ynez Valley (Santa Ynez Valley)	400	360	1 well for every 1 km²	0.08	89%	0%
Sylhet Basin (Bengal Basin)	12618	21	1 well for every 601 km²	0.08	100%	0%
San Luis Rey Valley	117	84	1 well for every 1 km²	0.08	92%	0%
Kavir Daranjir Basin	3692	47	1 well for every 79 km²	0.08	96%	2%
Western Blackwood Plateau	770	33	1 well for every 23 km²	0.08	94%	3%
Little Lost River Valley (Eastern Snake River Plain)	542	6	1 well for every 90 km²	0.08	83%	33%
Modesto Basin (San Joaquin Basin of the Central Valley)	1006	350	1 well for every 3 km²	0.08	76%	7%
Kouhbanan Basin	331	4	1 well for every 83 km²	0.08	100%	0%
Madhupur Tract and Eastern Floodplains (Bengal Basin)	19207	70	1 well for every 274 km²	0.08	97%	0%
Walla Walla Basin (Columbia Plateau Regional Aquifer System)	2288	2	1 well for every 1144 km²	0.08	50%	0%
Eastern Dakota Aquifer	39395	18	1 well for every 2189 km²	0.08	67%	0%
Sierra Valley	694	74	1 well for every 9 km²	0.08	69%	7%
Garber-Wellington Aquifer	8114	16	1 well for every 507 km²	0.08	63%	13%
Tlaltenango-Tepechitlan Aquifer	438	8	1 well for every 55 km²	0.08	63%	0%
Muteh Basin	960	26	1 well for every 37 km²	0.08	88%	0%
Brussels Sands	586	14	1 well for every 42 km²	0.08	100%	0%
Long Valley in eastern California	148	27	1 well for every 5 km²	0.08	85%	0%
Southcentral Floodplains (Bengal Basin)	30739	97	1 well for every 317 km²	0.08	94%	0%
Chino Basin (Upper Santa Ana Basin)	909	569	1 well for every 2 km²	0.08	63%	15%
Western St Vincent Basin (St Vincent Basin)	2058	19	1 well for every 108 km²	0.08	84%	0%

Study area	Area (km²)	Number of monitoring wells	Density of monitoring wells (with sufficient groundwater level observations for analyses) within the study area	Median Theil-Sen slope among monitoring wells *(positive values indicate deeper depths to water over time)*	Percent of wells with Theil-Sen slopes of >0 m per year	Percent of wells with Theil-Sen slopes of >0.5 m per year
Maryland Western Shores (North Atlantic Coastal Plain)	7014	402	1 well for every 17 km²	0.08	68%	7%
Eastern San Gabriel Valley (San Gabriel Valley)	202	85	1 well for every 2 km²	0.07	79%	22%
Central Kerala Coastal Aquifers (Malabar Coastal Plain)	975	33	1 well for every 30 km²	0.07	91%	0%
Northern Vienna Basin (Vienna Basin)	1481	1	1 well for every 1481 km²	0.07	100%	0%
Snake Valley	2043	25	1 well for every 82 km²	0.07	88%	0%
Esfandaran Basin (Lower Zayandeh-Rud Basin)	2850	26	1 well for every 110 km²	0.07	92%	0%
Anderson Valley	45	19	1 well for every 2 km²	0.07	74%	0%
Southern Coromandel Coastal Alluvial Aquifer (Coromandel Coastal Plain)	1439	11	1 well for every 131 km²	0.07	100%	27%
Maricopa-Stanfield Basin	1208	1	1 well for every 1208 km²	0.07	100%	0%
Red Bluff (Sacramento Valley of the Central Valley)	923	132	1 well for every 7 km²	0.07	81%	5%
Osage Plains (Osage Cuestas Aquifer System)	23975	2	1 well for every 11988 km²	0.07	100%	0%
Behbahan Plain	551	29	1 well for every 19 km²	0.07	72%	0%
Santa Clara-Calleguas Basin	852	782	1 well for every 1 km²	0.07	70%	15%
Eastern Huaihe Plain (Huaihe River Basin)	61827	33	1 well for every 1874 km²	0.07	61%	24%
Black Warrior River Aquifer System (Eutaw and McShan Formations and Tuscaloosa Group)	50216	39	1 well for every 1288 km²	0.07	64%	3%
Stilbaai Coastal Aquifer	3198	13	1 well for every 246 km²	0.07	69%	8%
Springbok Flats Karoo Basin	18345	19	1 well for every 966 km²	0.07	68%	16%
Hawthorne Whiskey Flat	582	10	1 well for every 58 km²	0.07	90%	0%
Aveiro Quaternary and Cretaceous Aquifer System	2473	3	1 well for every 824 km²	0.07	100%	0%
Miahuatlan Aquifer	585	16	1 well for every 37 km²	0.07	63%	6%
Willunga and Noarlunga Embayments (St Vincent Basin)	374	100	1 well for every 4 km²	0.07	72%	0%
Geer Basin and Hesbaye Chalk Aquifer	466	11	1 well for every 42 km²	0.07	91%	0%
Ktaoua Oasis (Middle Draa Basin)	363	5	1 well for every 73 km²	0.07	60%	0%
Canadian River Alluvial Aquifer	2829	26	1 well for every 109 km²	0.07	65%	0%
Sanpete Valley	677	42	1 well for every 16 km²	0.07	93%	0%
Antlers Aquifer	12607	24	1 well for every 525 km²	0.07	71%	4%
Lower Klamath Lake (Upper Klamath Basin)	382	8	1 well for every 48 km²	0.07	75%	0%
Crescent Valley in northern Nevada	1163	2	1 well for every 582 km²	0.07	100%	0%
Qatruiyeh Basin	1293	14	1 well for every 92 km²	0.07	71%	0%
Mississippi Delta (Gulf Coast Regional Aquifer System)	16784	4	1 well for every 4196 km²	0.07	50%	0%
Upper Thracian Plio-Quaternary Aquifer	6744	7	1 well for every 963 km²	0.07	71%	0%
Antelope Valley	8139	517	1 well for every 16 km²	0.07	66%	11%
Pampa del Tamarugal Aquifer	5065	16	1 well for every 317 km²	0.07	81%	0%
Maragheh-Bonab Plain	556	65	1 well for every 9 km²	0.07	71%	0%
Kerala Tertiary Coastal Aquifer (Malabar Coastal Plain)	2086	72	1 well for every 29 km²	0.07	85%	1%
Animas Valley	720	4	1 well for every 180 km²	0.07	75%	0%
Chittagong Coast Aquifer	4392	7	1 well for every 627 km²	0.07	71%	0%
Mason Valley	631	75	1 well for every 8 km²	0.07	76%	8%
Southern Malabar Khondalites (Malabar Coastal Plain)	2413	81	1 well for every 30 km²	0.07	75%	1%
Central Colusa Basin (Sacramento Valley of the Central Valley)	1153	152	1 well for every 8 km²	0.06	91%	3%

Study area	Area (km^2)	Number of monitoring wells	Density of monitoring wells (with sufficient groundwater level observations for analyses) within the study area	Median Theil-Sen slope among monitoring wells *(positive values indicate deeper depths to water over time)*	Percent of wells with Theil-Sen slopes of >0 m per year	Percent of wells with Theil-Sen slopes of >0.5 m per year
Eastern Riverine Plains (Murray Basin)	27505	247	1 well for every 111 km^2	0.06	74%	2%
Smith Valley	343	33	1 well for every 10 km^2	0.06	76%	24%
Central Vienna Basin (Vienna Basin)	1082	5	1 well for every 216 km^2	0.06	100%	0%
Scott Coastal Plain	1291	90	1 well for every 14 km^2	0.06	96%	1%
Southern Morongo Basin	1348	207	1 well for every 7 km^2	0.06	77%	2%
Baradwar Basin (Chhattisgarh Basin)	5494	38	1 well for every 145 km^2	0.06	74%	11%
Rio Canas Aquifer	143	3	1 well for every 48 km^2	0.06	100%	0%
Osoyoos Aquifer (Okanagan Basin)	83	5	1 well for every 17 km^2	0.06	100%	0%
Taznakht Plain	759	12	1 well for every 63 km^2	0.06	58%	8%
Southern San Juan Basin	7748	45	1 well for every 172 km^2	0.06	60%	9%
Volcanic Rift Zone (Eastern Snake River Plain)	5083	385	1 well for every 13 km^2	0.06	89%	1%
San Emidio Desert	303	1	1 well for every 303 km^2	0.06	100%	0%
Raymond Basin	108	14	1 well for every 8 km^2	0.06	71%	14%
Southern Railroad Valley	849	1	1 well for every 849 km^2	0.06	100%	0%
La Posa Plain	881	1	1 well for every 881 km^2	0.06	100%	0%
Wyandotte Creek Basin (Sacramento Valley of the Central Valley)	371	57	1 well for every 7 km^2	0.06	68%	0%
Covelo Round Valley	69	8	1 well for every 9 km^2	0.06	63%	0%
Elk City Aquifer	983	1	1 well for every 983 km^2	0.06	100%	0%
Goshute Valley	1711	1	1 well for every 1711 km^2	0.06	100%	0%
Gnangara Mound (Perth Basin)	2195	809	1 well for every 3 km^2	0.06	84%	3%
Lincolnshire Chalk Aquifer	1512	1	1 well for every 1512 km^2	0.06	100%	0%
Taoyuan-Chungli Tableland	707	28	1 well for every 25 km^2	0.06	64%	29%
East Shore Area	845	31	1 well for every 27 km^2	0.06	77%	0%
Chengdu Plain	8915	6	1 well for every 1486 km^2	0.06	83%	17%
Stampriet Kalahari Karoo Aquifer System	194772	4	1 well for every 48693 km^2	0.06	75%	0%
Great Plains Dakota Aquifer	23221	31	1 well for every 749 km^2	0.06	74%	0%
Choshui Alluvial Fan	2504	180	1 well for every 14 km^2	0.06	67%	3%
Montecito Basin	40	49	1 well for every 1 km^2	0.06	88%	0%
Papaloapan Basin (Coastal Plain of Veracruz)	9368	54	1 well for every 173 km^2	0.06	67%	4%
Northeastern Piedmont Plain (North China Plain)	8104	18	1 well for every 450 km^2	0.06	67%	11%
Cedar Valley near Utah Lake	366	33	1 well for every 11 km^2	0.06	70%	3%
Ramhormoz Alluvial Plain	967	71	1 well for every 14 km^2	0.06	82%	6%
Diamond Valley in northern Nevada	1292	6	1 well for every 215 km^2	0.06	100%	0%
Southern Hirri Basin (Chhattisgarh Basin)	10335	64	1 well for every 161 km^2	0.06	63%	2%
Eastern Second Songhuajiang System (Songnen Plain)	18076	4	1 well for every 4519 km^2	0.06	75%	0%
Central Sevier Valley	940	23	1 well for every 41 km^2	0.06	91%	0%
Curlew Valley	1431	20	1 well for every 72 km^2	0.06	85%	10%
Datong Basin	8944	1	1 well for every 8944 km^2	0.06	100%	0%
Santa Monica Coastal Plain (Los Angeles Basin)	108	514	1 well for every 0 km^2	0.05	65%	27%
Zhangye Basin (Heihe Basin)	6165	12	1 well for every 514 km^2	0.05	75%	17%
Cape Flats Aquifer	737	24	1 well for every 31 km^2	0.05	96%	8%
Northern Columbia River Basin (Columbia Plateau Regional Aquifer System)	12773	9	1 well for every 1419 km^2	0.05	67%	0%
Central Carrizo-Wilcox (Carrizo-Wilcox)	19641	313	1 well for every 63 km^2	0.05	60%	13%

Study area	Area (km^2)	Number of monitoring wells	Density of monitoring wells (with sufficient groundwater level observations for analyses) within the study area	Median Theil-Sen slope among monitoring wells *(positive values indicate deeper depths to water over time)*	Percent of wells with Theil-Sen slopes of >0 m per year	Percent of wells with Theil-Sen slopes of >0.5 m per year
Waipara Alluvial Basin	157	51	1 well for every 3 km^2	0.05	80%	20%
Eastern La Sabana Aquifer	76	3	1 well for every 25 km^2	0.05	100%	0%
Santa Barbara and Foothill Basin	55	934	1 well for every 0 km^2	0.05	80%	3%
San Fernando Valley	560	1014	1 well for every 1 km^2	0.05	70%	2%
Mission Valley and Irvine Flats (Flathead Valley)	1154	4	1 well for every 289 km^2	0.05	50%	0%
Middle Gangetic Plain (Indo-Gangetic Plain)	145648	101	1 well for every 1442 km^2	0.05	64%	1%
Forez Plain	956	5	1 well for every 191 km^2	0.05	80%	0%
Southern San Gabriel Valley (San Gabriel Valley)	114	349	1 well for every 0 km^2	0.05	70%	0%
Great Bend Prairie (High Plains)	18167	358	1 well for every 51 km^2	0.05	85%	0%
Yellow River Alluvial Plain (North China Plain)	37315	23	1 well for every 1622 km^2	0.05	61%	13%
Tinzouline Oasis (Middle Draa Basin)	425	4	1 well for every 106 km^2	0.05	75%	25%
Northcentral Swan Coastal Plain (Perth Basin)	4052	160	1 well for every 25 km^2	0.05	81%	0%
Delta-Mendota Basin (San Joaquin Basin of the Central Valley)	2581	839	1 well for every 3 km^2	0.05	71%	4%
Yolo Basin (Sacramento Valley of the Central Valley)	2049	1092	1 well for every 2 km^2	0.05	78%	7%
Southern Coastal Gujarat Alluvial Plain	7668	49	1 well for every 156 km^2	0.05	73%	4%
Mantinea Flats and Carlton Plain	314	27	1 well for every 12 km^2	0.05	81%	0%
Winkler Alluvial Aquifer	388	53	1 well for every 7 km^2	0.05	94%	0%
Western Karoo Basin (Main Karoo Basin)	146433	54	1 well for every 2712 km^2	0.05	80%	9%
Southern Miandoab Plain (Miandoab Plain)	970	59	1 well for every 16 km^2	0.05	66%	10%
Vinaroz Plain	492	6	1 well for every 82 km^2	0.05	100%	0%
Gunnedah Basin and Liverpool Plains	16086	649	1 well for every 25 km^2	0.05	76%	0%
Upper Gangetic Plain (Indo-Gangetic Plain)	199268	397	1 well for every 502 km^2	0.05	60%	3%
Sief Abad e- Laghar Basin	137	7	1 well for every 20 km^2	0.05	86%	0%
Central Cambay Basin (Cambay Basin)	16772	97	1 well for every 173 km^2	0.05	57%	24%
Gotvand Basin (Dezful-Andimeshk Plain)	579	36	1 well for every 16 km^2	0.05	78%	17%
Coastal Rayong Basin (Rayong Basin)	929	20	1 well for every 46 km^2	0.05	70%	0%
Yorkshire Chalk Aquifer	2101	2	1 well for every 1051 km^2	0.05	100%	0%
Trinity Aquifer System (Edwards-Trinity Aquifer System)	67070	622	1 well for every 108 km^2	0.05	59%	27%
Eastern Salton Sea	309	4	1 well for every 77 km^2	0.05	100%	0%
Gharesoo-Gorgan Rood Coastal Aquifer	6199	292	1 well for every 21 km^2	0.05	73%	8%
Salinas Valley	4318	890	1 well for every 5 km^2	0.05	74%	3%
Western Villa Garcia Aquifer	38	16	1 well for every 2 km^2	0.05	63%	0%
Mahabad Plain	236	24	1 well for every 10 km^2	0.05	88%	4%
Beaver Valley	325	23	1 well for every 14 km^2	0.05	74%	0%
Santa Maria Valley	97	116	1 well for every 1 km^2	0.05	87%	0%
Khormoj Basin	818	29	1 well for every 28 km^2	0.04	93%	0%
Hollywood Basin (Los Angeles Basin)	83	650	1 well for every 0 km^2	0.04	83%	0%
Rio Elota Aquifer	948	3	1 well for every 316 km^2	0.04	67%	0%
Mojave Basin	5973	848	1 well for every 7 km^2	0.04	64%	5%
Yuma Basin	3507	21	1 well for every 167 km^2	0.04	81%	14%
Chicago Valley	186	1	1 well for every 186 km^2	0.04	100%	0%
Lemhi Valley	997	2	1 well for every 499 km^2	0.04	50%	0%

Study area	Area (km^2)	Number of monitoring wells	Density of monitoring wells (with sufficient groundwater level observations for analyses) within the study area	Median Theil-Sen slope among monitoring wells *(positive values indicate deeper depths to water over time)*	Percent of wells with Theil-Sen slopes of >0 m per year	Percent of wells with Theil-Sen slopes of >0.5 m per year
Western Port Basin	1727	57	1 well for every 30 km^2	0.04	68%	0%
Northern St Vincent Basin (St Vincent Basin)	2276	11	1 well for every 207 km^2	0.04	91%	0%
Steptoe Valley	2396	4	1 well for every 599 km^2	0.04	75%	0%
Booborowie Valley	260	30	1 well for every 9 km^2	0.04	63%	3%
Eastern C Aquifer (The C Aquifer)	29532	7	1 well for every 4219 km^2	0.04	57%	0%
Kavir -e Siyahkoh Basin	655	5	1 well for every 131 km^2	0.04	100%	0%
Northern Piedmont and Tista Fan (Bengal Basin)	31898	199	1 well for every 160 km^2	0.04	81%	1%
Cumberland Basin (Maritimes Basin)	2853	3	1 well for every 951 km^2	0.04	100%	0%
Khor-Fakhri Basin	1346	25	1 well for every 54 km^2	0.04	60%	0%
Southeastern Surat Basin (Great Artesian Basin)	29141	208	1 well for every 140 km^2	0.04	67%	4%
Lund Subarea (Escalante Valley)	989	8	1 well for every 124 km^2	0.04	100%	0%
Goleta Basin	58	392	1 well for every 0 km^2	0.04	64%	9%
California Wash	452	1	1 well for every 452 km^2	0.04	100%	0%
North Chianan Plain (Chianan Plain)	1607	117	1 well for every 14 km^2	0.04	63%	21%
Southern Malabar Fractured Charnockite Aquifers (Malabar Coastal Plain)	3136	83	1 well for every 38 km^2	0.04	75%	0%
Bajo Guadalhorce Valley Alluvial Aquifer	261	4	1 well for every 65 km^2	0.04	50%	0%
San Luis Valley (Middle Rio Grande)	12667	305	1 well for every 42 km^2	0.04	82%	1%
Imperial Valley	2656	137	1 well for every 19 km^2	0.04	91%	0%
Kermanshah Plain	370	20	1 well for every 19 km^2	0.04	55%	15%
Southern Nuomin-Taoer System (Songnen Plain)	15583	9	1 well for every 1731 km^2	0.04	56%	0%
Mond Basin	734	24	1 well for every 31 km^2	0.04	79%	17%
Xian Depression (Weihe Basin)	9379	17	1 well for every 552 km^2	0.04	53%	18%
Eastern Salvadoran Coastal Plain (Salvadoran Coastal Plain)	1090	11	1 well for every 99 km^2	0.04	55%	36%
Conejo Basin	74	290	1 well for every 0 km^2	0.04	76%	0%
Baotao Plain	5310	8	1 well for every 664 km^2	0.04	50%	38%
Sain Alto Aquifer	561	15	1 well for every 37 km^2	0.04	60%	0%
Halvan Basin	656	9	1 well for every 73 km^2	0.04	100%	0%
Southern Utah Valley	417	17	1 well for every 25 km^2	0.04	82%	6%
Kristianstad Plain	1403	6	1 well for every 234 km^2	0.04	100%	0%
Espanola Basin (Middle Rio Grande)	4014	58	1 well for every 69 km^2	0.04	71%	10%
Djursland Limestone and Sand Aquifer System	1123	19	1 well for every 59 km^2	0.04	74%	0%
Borujerd -Doroud Plain	821	35	1 well for every 23 km^2	0.04	60%	14%
Perote-Zalayeta Aquifer	407	6	1 well for every 68 km^2	0.04	83%	17%
Rio Grande Delta (Gulf Coast Regional Aquifer System)	62984	159	1 well for every 396 km^2	0.04	60%	3%
Southeastern Bengal Basin (Bengal Basin)	10016	29	1 well for every 345 km^2	0.04	90%	0%
Central Otway Basin (Otway Basin)	9543	72	1 well for every 133 km^2	0.04	78%	0%
Jacumba Valley	69	7	1 well for every 10 km^2	0.04	86%	0%
Northern Guadalupe Garzaron Aquifer	754	15	1 well for every 50 km^2	0.04	67%	7%
Blue Creek Valley	285	2	1 well for every 143 km^2	0.04	100%	0%
Marbella-Estepona Coastal Aquifer	129	4	1 well for every 32 km^2	0.04	75%	0%
Valencia Plain	3272	49	1 well for every 67 km^2	0.04	73%	18%
Carson Valley (Carson River Basin)	730	52	1 well for every 14 km^2	0.04	69%	2%

Study area	Area (km^2)	Number of monitoring wells	Density of monitoring wells (with sufficient groundwater level observations for analyses) within the study area	Median Theil-Sen slope among monitoring wells *(positive values indicate deeper depths to water over time)*	Percent of wells with Theil-Sen slopes of >0 m per year	Percent of wells with Theil-Sen slopes of >0.5 m per year
Ivanpah Valley	1070	15	1 well for every 71 km^2	0.04	60%	0%
Rincon Valley (Rincon-Mesilla Valleys)	264	38	1 well for every 7 km^2	0.04	79%	0%
Cubeta de Oliete and Aliaga-Calanda Aquifers	4422	18	1 well for every 246 km^2	0.04	61%	0%
Las Vegas Basin of northeast New Mexico	5322	12	1 well for every 444 km^2	0.04	58%	25%
Solano Basin (Sacramento Valley of the Central Valley)	1467	708	1 well for every 2 km^2	0.04	76%	2%
Zaachila Valley (Central Valleys of Oaxaca)	1003	43	1 well for every 23 km^2	0.04	60%	12%
San Juan Valley	37	110	1 well for every 0 km^2	0.04	69%	0%
Presidio-Redford Bolson and Del Peso Valley	2931	3	1 well for every 977 km^2	0.04	67%	0%
La Yarada Aquifer	1232	3	1 well for every 411 km^2	0.04	100%	0%
Wuyuer-Shuangyang System (Songnen Plain)	63713	19	1 well for every 3353 km^2	0.04	63%	11%
Poolowanna Trough and Western Eromanga Basin (Great Artesian Basin)	292840	23	1 well for every 12732 km^2	0.04	65%	9%
Half Moon Bay Coastal Terraces	39	40	1 well for every 1 km^2	0.03	80%	0%
Western Riverine Plains (Murray Basin)	23144	144	1 well for every 161 km^2	0.03	83%	0%
Rush Valley	1257	10	1 well for every 126 km^2	0.03	90%	10%
Hamlin Valley	1862	12	1 well for every 155 km^2	0.03	100%	0%
Ghaap Plateau Dolomite Aquifer System	20319	56	1 well for every 363 km^2	0.03	64%	2%
Western Uinta Basin (Uinta Basin)	2823	2	1 well for every 1412 km^2	0.03	100%	0%
Western Kachchh Coastal Plain (Kachchh Coastal Plain)	416	1	1 well for every 416 km^2	0.03	100%	0%
Muddy River Springs Area	45	1	1 well for every 45 km^2	0.03	100%	0%
Central Perth Basin (Perth Basin)	1941	413	1 well for every 5 km^2	0.03	82%	5%
Cimarron Basin	5070	39	1 well for every 130 km^2	0.03	74%	0%
Northeastern Deccan Basalt Plateau (Deccan Plateau)	47763	24	1 well for every 1990 km^2	0.03	67%	0%
Lower Rio Huasco Basin (Rio Huasco Basin)	109	4	1 well for every 27 km^2	0.03	100%	0%
Dauphine Miocene Molasses Aquifer System	4845	36	1 well for every 135 km^2	0.03	83%	0%
Northern Paris Basin (Paris Basin)	23814	116	1 well for every 205 km^2	0.03	72%	1%
Southern Mekong Delta (Mekong Delta)	20929	8	1 well for every 2616 km^2	0.03	88%	13%
Bandan Basin	360	15	1 well for every 24 km^2	0.03	60%	7%
Llagas Area (Gilroy-Hollister Valley)	206	209	1 well for every 1 km^2	0.03	56%	1%
Western C Aquifer (The C Aquifer)	13082	17	1 well for every 770 km^2	0.03	94%	0%
Tyner Valley Aquifer (Bearpaw-Judith River Aquifer System)	4024	1	1 well for every 4024 km^2	0.03	100%	0%
Upper Salmon Basin	195	1	1 well for every 195 km^2	0.03	100%	0%
Eastern Pilbara Granite-Greenstone Terrane (Pilbara Craton)	23972	1	1 well for every 23972 km^2	0.03	100%	0%
Boise Valley and Homedale Murphy Area (Western Snake River Plain)	4578	88	1 well for every 52 km^2	0.03	58%	2%
Kobeh Valley	1573	6	1 well for every 262 km^2	0.03	83%	0%
Upper Coastal Plain (Floridan Aquifer System)	5562	52	1 well for every 107 km^2	0.03	69%	0%
Eastern Otway Basin (Otway Basin)	17980	304	1 well for every 59 km^2	0.03	72%	1%
Moor Limestone Aquifer	2543	16	1 well for every 159 km^2	0.03	75%	0%
Rio Sinaloa Coastal Aquifer	3204	52	1 well for every 62 km^2	0.03	62%	2%
Malekan Aquifer (Miandoab Plain)	857	76	1 well for every 11 km^2	0.03	66%	1%
Hirakud Command Area Endogenous Rock Aquifers	5570	76	1 well for every 73 km^2	0.03	61%	0%
Behshar Basin	656	45	1 well for every 15 km^2	0.03	58%	4%

Study area	Area (km^2)	Number of monitoring wells	Density of monitoring wells (with sufficient groundwater level observations for analyses) within the study area	Median Theil-Sen slope among monitoring wells *(positive values indicate deeper depths to water over time)*	Percent of wells with Theil-Sen slopes of >0 m per year	Percent of wells with Theil-Sen slopes of >0.5 m per year
Central Amarkantak Plateau (Amarkantak Plateau)	7924	34	1 well for every 233 km^2	0.03	59%	0%
Lake Valley	1702	3	1 well for every 567 km^2	0.03	67%	0%
Santa Margarita Valley	25	56	1 well for every 0 km^2	0.03	64%	0%
Northern Hickory Aquifer	5487	99	1 well for every 55 km^2	0.03	61%	6%
Donihue-Coinco-Coltauco Aquifer	340	4	1 well for every 85 km^2	0.03	75%	0%
Temecula Valley	233	175	1 well for every 1 km^2	0.03	63%	10%
Eastern Sutter Basin (Sacramento Valley of the Central Valley)	547	194	1 well for every 3 km^2	0.03	65%	0%
Atlantis Aquifer	326	5	1 well for every 65 km^2	0.03	80%	0%
Seaspray Region in Southwestern Gippsland Basin (Gippsland Basin)	2079	45	1 well for every 46 km^2	0.03	93%	4%
North Rewa Basin (Son-Mahandai Valley Basins)	13718	38	1 well for every 361 km^2	0.03	61%	3%
Surprise Valley	1059	47	1 well for every 23 km^2	0.03	68%	4%
Mississippian-Silurian-Devonian Carbonates (Northern Midwest Aquifer System)	87887	91	1 well for every 966 km^2	0.03	62%	5%
Marsh Creek (Eastern Snake River Plain)	433	8	1 well for every 54 km^2	0.03	75%	0%
Kavir Haj Aligholi Aquifer	1495	38	1 well for every 39 km^2	0.03	79%	0%
Stone Cabin Valley	932	6	1 well for every 155 km^2	0.03	83%	0%
Hualapai Valley Basin	2119	1	1 well for every 2119 km^2	0.03	100%	0%
Upper Lost River (Upper Klamath Basin)	523	30	1 well for every 17 km^2	0.03	70%	0%
Southern Swan Coastal Plain (Perth Basin)	1441	240	1 well for every 6 km^2	0.03	72%	1%
Roussillon Plain	911	25	1 well for every 36 km^2	0.03	68%	4%
Malm Valangnian Aquifer	17275	16	1 well for every 1080 km^2	0.03	75%	0%
Marana-Casinca Plain	141	2	1 well for every 71 km^2	0.03	100%	0%
Northern Swan Coastal Plain (Perth Basin)	3977	104	1 well for every 38 km^2	0.03	65%	0%
Puerto Rico South Coast Aquifer System	389	26	1 well for every 15 km^2	0.03	85%	0%
Canning Basin	130597	23	1 well for every 5678 km^2	0.03	57%	0%
Salt Lake Valley	1252	97	1 well for every 13 km^2	0.03	65%	3%
Northern Valley and Ridge (Valley and Ridge Aquifer System)	43020	131	1 well for every 328 km^2	0.03	62%	7%
Carpinteria Basin	31	25	1 well for every 1 km^2	0.03	80%	8%
Southern Mallee (Murray Basin)	31573	247	1 well for every 128 km^2	0.03	72%	1%
Gambier Embayment (Otway Basin)	12773	758	1 well for every 17 km^2	0.03	75%	1%
Kaikoura Plain	76	9	1 well for every 8 km^2	0.03	89%	0%
Sprague Basin (Upper Klamath Basin)	1356	17	1 well for every 80 km^2	0.03	65%	0%
Twin Falls (Central Snake River Plain)	2081	54	1 well for every 39 km^2	0.03	65%	2%
Spring Valley	2436	6	1 well for every 406 km^2	0.03	83%	0%
Norabad Basin	508	14	1 well for every 36 km^2	0.03	64%	0%
Butte Basin (Sacramento Valley of the Central Valley)	1235	115	1 well for every 11 km^2	0.03	70%	0%
Churchill Valley (Carson River Basin)	234	41	1 well for every 6 km^2	0.03	71%	2%
Lower Athabasca (Lower Athabasca Basin)	15814	12	1 well for every 1318 km^2	0.03	58%	25%
Western Pilbara Granite-Greenstone Terrane (Pilbara Craton)	16960	9	1 well for every 1884 km^2	0.03	89%	0%
Cuautla-Yautepec Valley	505	47	1 well for every 11 km^2	0.03	55%	23%
Blue Mountains and Clearwater Embayment (Columbia Plateau Regional Aquifer System)	26297	13	1 well for every 2023 km^2	0.03	62%	23%
Lower Limestone Coast Aquifer System (Murray Basin)	25446	699	1 well for every 36 km^2	0.03	76%	1%

Study area	Area (km^2)	Number of monitoring wells	Density of monitoring wells (with sufficient groundwater level observations for analyses) within the study area	Median Theil-Sen slope among monitoring wells *(positive values indicate deeper depths to water over time)*	Percent of wells with Theil-Sen slopes of >0 m per year	Percent of wells with Theil-Sen slopes of >0.5 m per year
Alabama Coastal Lowlands (Gulf Coast Regional Aquifer System)	21894	2	1 well for every 10947 km^2	0.03	50%	0%
Eastern Gippsland Basin (Gippsland Basin)	1613	78	1 well for every 21 km^2	0.03	72%	3%
Matanza-Riachuelo Aquifer System	2657	90	1 well for every 30 km^2	0.03	64%	3%
Southern Carpentaria Basin (Great Artesian Basin)	124001	89	1 well for every 1393 km^2	0.03	56%	2%
Southern Sarkhoon Aquifer (Sarkhoon Aquifer)	320	3	1 well for every 107 km^2	0.03	100%	0%
Victoria Area (Gulf Coast Regional Aquifer System)	31242	376	1 well for every 83 km^2	0.02	58%	1%
Dale Valley	438	9	1 well for every 49 km^2	0.02	89%	0%
Benkersandstein Aquifer System	2437	4	1 well for every 609 km^2	0.02	75%	0%
Lower Mur Valley	300	2	1 well for every 150 km^2	0.02	100%	0%
Consuegra-Villacanas Aquifer	1538	2	1 well for every 769 km^2	0.02	50%	0%
Dagh-e-Sorkh Basin	1475	17	1 well for every 87 km^2	0.02	65%	0%
Truckee Meadows and Pleasant Valley	382	34	1 well for every 11 km^2	0.02	71%	18%
Essex Clay Plain (Southern Ontario Paleozoic Aquifer System)	2045	3	1 well for every 682 km^2	0.02	67%	0%
Western Parecis Basin (Parecis Basin)	213494	7	1 well for every 30499 km^2	0.02	57%	0%
Lake Mohave Basin	661	25	1 well for every 26 km^2	0.02	92%	0%
Deep Creek Valley	580	5	1 well for every 116 km^2	0.02	80%	0%
Cordoba Basin (Orizaba-Cordoba Basins)	233	11	1 well for every 21 km^2	0.02	64%	9%
Okanagan Lake Perimeter (west) (Okanagan Basin)	98	1	1 well for every 98 km^2	0.02	100%	0%
Dalon Basin	20	2	1 well for every 10 km^2	0.02	50%	0%
Great Sand Hills North Aquifer (Bearpaw-Judith River Aquifer System)	2347	1	1 well for every 2347 km^2	0.02	100%	0%
Bear River Bay	611	11	1 well for every 56 km^2	0.02	73%	0%
Tracy Basin (San Joaquin Basin of the Central Valley)	915	443	1 well for every 2 km^2	0.02	67%	4%
Edwards Plateau (Edwards-Trinity Aquifer System)	58050	342	1 well for every 170 km^2	0.02	64%	5%
Sofia Neogene Graben	1389	18	1 well for every 77 km^2	0.02	56%	0%
Clarence-Moreton Basin and Cecil Plains (Great Artesian Basin)	13238	488	1 well for every 27 km^2	0.02	61%	3%
Central Cape Breton (Maritimes Basin)	1671	1	1 well for every 1671 km^2	0.02	100%	0%
Tournaisis Region Carbonate Aquifers	1960	39	1 well for every 50 km^2	0.02	59%	8%
Western Outer Bluegrass (Interior Low Plateaus)	9031	58	1 well for every 156 km^2	0.02	62%	0%
Middle Aconcagua Basin (Aconcagua Basin)	385	8	1 well for every 48 km^2	0.02	75%	0%
Mehraban Plain	464	17	1 well for every 27 km^2	0.02	82%	0%
Tijuana-San Diego Basin	455	1483	1 well for every 0 km^2	0.02	68%	1%
Central Paris Basin (Paris Basin)	31880	127	1 well for every 251 km^2	0.02	66%	1%
Rush Springs Aquifer	13372	68	1 well for every 197 km^2	0.02	60%	0%
Main Basin (Puget Sound Lowland)	2250	1	1 well for every 2250 km^2	0.02	100%	0%
Clayton Basin	83	279	1 well for every 0 km^2	0.02	76%	0%
Mioplioquatenario de Elvas-Campo Maior and Vegas Baja Aquifers	3824	30	1 well for every 127 km^2	0.02	70%	0%
Smith Creek Valley	725	3	1 well for every 242 km^2	0.02	100%	0%
Detrital Valley Basin	1209	8	1 well for every 151 km^2	0.02	63%	13%
Arbon Valley (Eastern Snake River Plain)	943	20	1 well for every 47 km^2	0.02	55%	0%
Lochiel Lobe (St Vincent Basin)	260	10	1 well for every 26 km^2	0.02	90%	0%

Study area	Area (km^2)	Number of monitoring wells	Density of monitoring wells (with sufficient groundwater level observations for analyses) within the study area	Median Theil-Sen slope among monitoring wells *(positive values indicate deeper depths to water over time)*	Percent of wells with Theil-Sen slopes of >0 m per year	Percent of wells with Theil-Sen slopes of >0.5 m per year
Big Valley	98	78	1 well for every 1 km^2	0.02	65%	0%
Ivanhoe Plains (Murray Basin)	45266	436	1 well for every 104 km^2	0.02	76%	0%
Mesilla-Conejos-Medanos Basin	6528	23	1 well for every 284 km^2	0.02	78%	0%
Scott Valley and Mann Creek	613	2	1 well for every 307 km^2	0.02	50%	0%
Western Sutter Basin (Sacramento Valley of the Central Valley)	531	57	1 well for every 9 km^2	0.02	75%	0%
Long Valley - Round Valley	1341	4	1 well for every 335 km^2	0.02	50%	0%
Big Lost River Valley (Eastern Snake River Plain)	886	25	1 well for every 35 km^2	0.02	60%	4%
Middle Tata Alluvial Basin	178	10	1 well for every 18 km^2	0.02	50%	0%
Coyote Springs Valley	646	9	1 well for every 72 km^2	0.02	67%	0%
Western Saldanah and Langebaan Lagoon Aquifer System	645	18	1 well for every 36 km^2	0.02	78%	0%
Shasta Valley	1280	80	1 well for every 16 km^2	0.02	75%	0%
Taichung Coastal Alluvial Aquifer (Taichung Basin)	386	15	1 well for every 26 km^2	0.02	60%	0%
East Contra Basin (San Joaquin Basin of the Central Valley)	384	350	1 well for every 1 km^2	0.02	76%	0%
Don Bonito Region in the Middle Guadiana Basin	2903	27	1 well for every 108 km^2	0.02	78%	0%
Northwestern Kanto Plain (Kanto Plain)	1696	2	1 well for every 848 km^2	0.02	50%	0%
Western Amarkantak Plateau (Amarkantak Plateau)	9732	31	1 well for every 314 km^2	0.02	55%	13%
Lom- Pleven Depression	8849	28	1 well for every 316 km^2	0.02	57%	0%
Oropesa Plain	114	5	1 well for every 23 km^2	0.02	60%	0%
Pahsimeroi Valley	627	5	1 well for every 125 km^2	0.02	60%	0%
Southwestern Coastal Sandveld and Elandsfontein Aquifer	4272	163	1 well for every 26 km^2	0.02	66%	2%
Coyote Wells Valley	384	30	1 well for every 13 km^2	0.02	67%	0%
Leibnitzer Feld	150	1	1 well for every 150 km^2	0.02	100%	0%
Lower Morovian Basin	1438	9	1 well for every 160 km^2	0.02	89%	0%
Central Canterbury Plains	3167	212	1 well for every 15 km^2	0.02	56%	5%
Southern Colusa Basin (Sacramento Valley of the Central Valley)	841	136	1 well for every 6 km^2	0.02	72%	20%
Almonte-Marismas Aquifer (Guadalquivir Basin)	5351	31	1 well for every 173 km^2	0.02	58%	0%
Dixie Valley	1826	3	1 well for every 609 km^2	0.02	100%	0%
Saravan Basin	1189	25	1 well for every 48 km^2	0.02	56%	0%
Sundarbans and Southern Delta (Bengal Basin)	22556	4	1 well for every 5639 km^2	0.02	75%	0%
Kazanlak Kettle	662	4	1 well for every 166 km^2	0.02	75%	0%
Yinchuan Plain	8295	4	1 well for every 2074 km^2	0.02	75%	0%
Mons Sedimentary Basin	711	30	1 well for every 24 km^2	0.02	77%	0%
Willow Creek Basin	70	7	1 well for every 10 km^2	0.02	71%	0%
Eastern Karoo Basin (Main Karoo Basin)	41725	12	1 well for every 3477 km^2	0.02	50%	8%
Western Karstic Plain (Yucatan Peninsula)	4252	2	1 well for every 2126 km^2	0.02	50%	0%
Western Paris Basin (Paris Basin)	28758	154	1 well for every 187 km^2	0.02	69%	0%
Upper Peninsula Jacobsville Sandstone and Cambrian-Ordovician and Silurian-Devonian Aquifers (Northern Midwest Aquifer System)	25514	8	1 well for every 3189 km^2	0.02	75%	0%
Fountain Creek Alluvial Aquifer	445	33	1 well for every 13 km^2	0.02	67%	0%
Trebon Basin	1027	12	1 well for every 86 km^2	0.02	92%	0%
Sadich Basin	218	5	1 well for every 44 km^2	0.02	80%	0%

Study area	Area (km^2)	Number of monitoring wells	Density of monitoring wells (with sufficient groundwater level observations for analyses) within the study area	Median Theil-Sen slope among monitoring wells *(positive values indicate deeper depths to water over time)*	Percent of wells with Theil-Sen slopes of >0 m per year	Percent of wells with Theil-Sen slopes of >0.5 m per year
Eastern Amarkantak Plateau (Amarkantak Plateau)	9898	25	1 well for every 396 km^2	0.02	60%	0%
Asahikawa Basin	858	8	1 well for every 107 km^2	0.02	75%	0%
Wah Wah Valley	627	5	1 well for every 125 km^2	0.02	100%	0%
Western Doon Valley	444	12	1 well for every 37 km^2	0.02	50%	0%
South Yuba Basin (Sacramento Valley of the Central Valley)	511	219	1 well for every 2 km^2	0.02	57%	0%
Arckaringa Basin and Southwestern Great Artesian Basin (Great Artesian Basin)	107715	30	1 well for every 3591 km^2	0.02	63%	3%
Hermanus Coastal Aquifer	298	5	1 well for every 60 km^2	0.02	100%	0%
Landes Plain (Aquitaine Basin)	15042	381	1 well for every 39 km^2	0.02	61%	3%
Wood River Valley	511	75	1 well for every 7 km^2	0.02	71%	0%
Livermore Valley	247	286	1 well for every 1 km^2	0.02	70%	3%
Honey Lake Valley	2072	149	1 well for every 14 km^2	0.02	59%	1%
Bighorn Basin	20257	3	1 well for every 6752 km^2	0.02	100%	33%
Transboundary Judith River Aquifer (Bearpaw-Judith River Aquifer System)	22275	5	1 well for every 4455 km^2	0.02	80%	0%
Wet Mountain Valley	978	48	1 well for every 20 km^2	0.02	60%	6%
Mitchell Plateau (Interior Low Plateaus)	5985	2	1 well for every 2993 km^2	0.02	50%	0%
Campo de Carinena Aquifer	718	7	1 well for every 103 km^2	0.02	57%	29%
Bandar Ganaveh Basin	2115	47	1 well for every 45 km^2	0.02	62%	0%
Northern Khorat Plateau (Khorat Plateau Aquifer System)	14425	15	1 well for every 962 km^2	0.02	60%	0%
Sardis-Vedder Aquifer	56	2	1 well for every 28 km^2	0.01	50%	0%
Ebro Delta	433	4	1 well for every 108 km^2	0.01	100%	0%
Otava and Blanice Quaternary Aquifer System	93	8	1 well for every 12 km^2	0.01	100%	0%
Denver Basin	18104	185	1 well for every 98 km^2	0.01	56%	3%
Dublin Basin	5996	27	1 well for every 222 km^2	0.01	74%	4%
Sonoma Valley	157	197	1 well for every 1 km^2	0.01	62%	5%
Lower Gascoyne Alluvial Aquifer	1022	30	1 well for every 34 km^2	0.01	60%	3%
Mianrahan Aquifer	154	23	1 well for every 7 km^2	0.01	61%	0%
Longford Tertiary Basin	2808	15	1 well for every 187 km^2	0.01	53%	0%
Hot Creek Valley	1411	3	1 well for every 470 km^2	0.01	67%	0%
Northern Rayong Basin (Rayong Basin)	823	12	1 well for every 69 km^2	0.01	58%	0%
Cuernavaca Valley	271	47	1 well for every 6 km^2	0.01	53%	19%
Western Ouarzazate Basin	1712	4	1 well for every 428 km^2	0.01	50%	25%
Lyonnais and South Dombes Miocene Aquifer System	1346	27	1 well for every 50 km^2	0.01	74%	0%
Lower Waitaki South Coastal Canterbury Aquifer	447	11	1 well for every 41 km^2	0.01	64%	0%
Myalup and Yalgorup Plain (Perth Basin)	1847	255	1 well for every 7 km^2	0.01	69%	0%
Equus Beds (High Plains)	4993	675	1 well for every 7 km^2	0.01	61%	0%
Southern Bohemian Tertiary and Cretaceous Sedimentary Aquifers (Bohemian Cretaceous Basin)	441	2	1 well for every 221 km^2	0.01	100%	0%
New Brunswick Carboniferous Basin (Maritimes Basin)	19211	4	1 well for every 4803 km^2	0.01	75%	0%
Western Cambay Basin (Cambay Basin)	17184	47	1 well for every 366 km^2	0.01	57%	9%
Socorro Basin	1127	14	1 well for every 81 km^2	0.01	64%	0%
Quincy Subregion (Columbia Plateau Regional Aquifer System)	3819	26	1 well for every 147 km^2	0.01	58%	8%

Study area	Area (km^2)	Number of monitoring wells	Density of monitoring wells (with sufficient groundwater level observations for analyses) within the study area	Median Theil-Sen slope among monitoring wells *(positive values indicate deeper depths to water over time)*	Percent of wells with Theil-Sen slopes of >0 m per year	Percent of wells with Theil-Sen slopes of >0.5 m per year
Ashley Valley (Uinta Basin)	316	7	1 well for every 45 km^2	0.01	100%	0%
Southern Vienna Basin (Vienna Basin)	1435	4	1 well for every 359 km^2	0.01	100%	0%
Upper Butte Creek Basin (Sierra Nevada Foothills)	380	27	1 well for every 14 km^2	0.01	70%	0%
Bishop Basin	510	70	1 well for every 7 km^2	0.01	71%	0%
Swan Lake and Upper Klamath Lake Perimeter (Upper Klamath Basin)	1174	16	1 well for every 73 km^2	0.01	63%	6%
San Agustin Plains	2291	40	1 well for every 57 km^2	0.01	93%	0%
Eastern Bohemian Cretaceous Basin (Bohemian Cretaceous Basin)	3269	41	1 well for every 80 km^2	0.01	83%	0%
Ghorghi Ziyarat Plain	459	15	1 well for every 31 km^2	0.01	53%	0%
North Yuba Basin (Sacramento Valley of the Central Valley)	304	217	1 well for every 1 km^2	0.01	62%	0%
Southern Butte Valley	818	2	1 well for every 409 km^2	0.01	100%	0%
Northern San Juan Basin	20120	15	1 well for every 1341 km^2	0.01	53%	27%
Frenchman Flat	386	20	1 well for every 19 km^2	0.01	80%	0%
Zaboli Aquifer	1201	12	1 well for every 100 km^2	0.01	58%	8%
Southern Paris Basin (Paris Basin)	24635	116	1 well for every 212 km^2	0.01	66%	0%
Ebro Alluvial Aquifers	1476	7	1 well for every 211 km^2	0.01	71%	0%
Upper Beaverhead Basin	1280	2	1 well for every 640 km^2	0.01	50%	0%
Mexicali Valley	2542	108	1 well for every 24 km^2	0.01	69%	2%
Middle-Upper Devonian Aquifer System (Baltic Artesian Basin)	34615	15	1 well for every 2308 km^2	0.01	67%	0%
Wimmera Plain (Murray Basin)	20408	345	1 well for every 59 km^2	0.01	61%	0%
Cheb Basin	351	4	1 well for every 88 km^2	0.01	75%	0%
Santa Clara Valley	781	3561	1 well for every 0 km^2	0.01	61%	0%
Dugway-Government Creek Valley	1103	7	1 well for every 158 km^2	0.01	71%	0%
Upper Yuba Basin (Sierra Nevada Foothills)	1626	56	1 well for every 29 km^2	0.01	61%	0%
Central Mississippi Embayment (Mississippi Embayment)	89580	1844	1 well for every 49 km^2	0.01	55%	0%
Hassanabad-Dehchah Plain	466	10	1 well for every 47 km^2	0.01	60%	30%
Laguna de Santiaguillo Basin	1611	48	1 well for every 34 km^2	0.01	50%	31%
Little Lake Valley	48	89	1 well for every 1 km^2	0.01	69%	0%
Central Bohemian Cretaceuos Basin (Bohemian Cretaceuos Basin)	4274	40	1 well for every 107 km^2	0.01	90%	0%
Klamath River (Upper Klamath Basin)	139	3	1 well for every 46 km^2	0.01	67%	0%
Coachella Valley	1797	233	1 well for every 8 km^2	0.01	55%	15%
Nahavand Plain	814	36	1 well for every 23 km^2	0.01	58%	14%
Iroquois Till Plains (Central Lowland Till Plain)	4508	8	1 well for every 564 km^2	0.01	63%	0%
Pecos Valley	21799	170	1 well for every 128 km^2	0.01	55%	4%
Northwestern Basal Cretaceous Aquifer (Bohemian Cretaceous Basin)	1992	13	1 well for every 153 km^2	0.01	100%	0%
Niles Cone	191	1192	1 well for every 0 km^2	0.01	65%	0%
Middle Yellowstone Area	3573	11	1 well for every 325 km^2	0.01	91%	0%
San Mateo Plain	141	1089	1 well for every 0 km^2	0.01	66%	0%
Palouse Basin (Columbia Plateau Regional Aquifer System)	2341	6	1 well for every 390 km^2	0.01	67%	0%
Hansel and North Rozel Flat	333	1	1 well for every 333 km^2	0.01	100%	0%
Latrobe Valley (Gippsland Basin)	3495	152	1 well for every 23 km^2	0.01	62%	9%
Lower Walker Basin	723	43	1 well for every 17 km^2	0.01	70%	0%

Study area	Area (km^2)	Number of monitoring wells	Density of monitoring wells (with sufficient groundwater level observations for analyses) within the study area	Median Theil-Sen slope among monitoring wells *(positive values indicate deeper depths to water over time)*	Percent of wells with Theil-Sen slopes of >0 m per year	Percent of wells with Theil-Sen slopes of >0.5 m per year
Fall River Valley	267	17	1 well for every 16 km^2	0.01	53%	0%
Nayband Basin	1202	9	1 well for every 134 km^2	0.01	100%	0%
Alsatian Aquifer and Upper Rhine Valley	10659	183	1 well for every 58 km^2	0.01	76%	0%
Amol-Ghaemshahr Plain	2368	112	1 well for every 21 km^2	0.01	58%	1%
Ygnacio Valley	103	224	1 well for every 0 km^2	0.01	74%	0%
Sari-Neka Plain	1289	79	1 well for every 16 km^2	0.01	57%	1%
San Pedro Basin	6711	101	1 well for every 66 km^2	0.01	68%	1%
Tecolutla Aquifer	2012	4	1 well for every 503 km^2	0.01	50%	0%
Big Valley in northern California	494	36	1 well for every 14 km^2	0.01	53%	11%
Northern Aquitaine Basin (Aquitaine Basin)	25909	267	1 well for every 97 km^2	0.01	54%	2%
Lower Arkansas River Eastern Reach	1651	33	1 well for every 50 km^2	0.01	70%	0%
Aleshtar Plain	238	21	1 well for every 11 km^2	0.01	62%	0%
Naein Basin	2727	30	1 well for every 91 km^2	0.01	60%	3%
Astian Aquifer	438	33	1 well for every 13 km^2	0.01	52%	0%
Lajas Valley	177	3	1 well for every 59 km^2	0.01	100%	0%
Wind River Basin West (Wind River Basin)	8902	2	1 well for every 4451 km^2	0.01	50%	0%
Promontory Mountains Area	148	3	1 well for every 49 km^2	0.01	67%	0%
El Cajon Valley	54	508	1 well for every 0 km^2	0.01	60%	0%
Tule Valley	1204	5	1 well for every 241 km^2	0.01	80%	0%
Pilsen Basin	664	11	1 well for every 60 km^2	0.01	82%	0%
Muscatatuck Plateau and Dearborn Upland	7718	23	1 well for every 336 km^2	0.01	57%	9%
Antigonish Basin (Maritimes Basin)	2230	2	1 well for every 1115 km^2	0.01	50%	0%
Escondido Valley	40	304	1 well for every 0 km^2	0.01	63%	0%
Sink Valley	317	4	1 well for every 79 km^2	0.01	100%	0%
Upper Morovian Basin	2381	21	1 well for every 113 km^2	0.01	71%	0%
Grouse Creek Valley	172	3	1 well for every 57 km^2	0.01	100%	0%
Petaluma Valley	220	475	1 well for every 0 km^2	0.01	62%	0%
Scott Valley	710	19	1 well for every 37 km^2	0.01	68%	0%
Peumo-Pichidega-La Cabras and Pelequen-Malloa-San Vicente De Tagua Tagua and Laguna San Vicente Aquifers	621	3	1 well for every 207 km^2	0.01	67%	0%
South Fork Pitt River Basin	369	48	1 well for every 8 km^2	0.01	54%	0%
Southern St. Lawrence Lowlands (St. Lawrence Lowlands)	24965	91	1 well for every 274 km^2	0.01	60%	0%
Payette Valley	815	11	1 well for every 74 km^2	0.01	64%	0%
Central Mallee (Murray Basin)	27348	1610	1 well for every 17 km^2	0.01	58%	0%
Jiangsu Coastal Plain (Huaihe River Basin)	56925	28	1 well for every 2033 km^2	0.01	54%	7%
Long Valley near Crowley Lake	258	19	1 well for every 14 km^2	0.01	68%	0%
Amargosa Desert	1896	82	1 well for every 23 km^2	0.01	66%	4%
Connecticut Valley	3038	62	1 well for every 49 km^2	0.01	71%	0%
Ryan Flat (Salt Basin Bolson)	1428	6	1 well for every 238 km^2	0.01	50%	0%
Western Satpura Basalt Aquifers (Satpura Basalts)	25934	69	1 well for every 376 km^2	0.01	54%	9%
Thousand Oaks and Russell Valley	46	163	1 well for every 0 km^2	0.01	59%	0%
Southwestern Bengal Basin (Bengal Basin)	20073	3	1 well for every 6691 km^2	0.01	67%	0%
Central Cumberland Plateau and Sequatchie Valley (Appalachian Plateaus)	12392	2	1 well for every 6196 km^2	0.01	100%	0%

Study area	Area (km²)	Number of monitoring wells	Density of monitoring wells (with sufficient groundwater level observations for analyses) within the study area	Median Theil-Sen slope among monitoring wells *(positive values indicate deeper depths to water over time)*	Percent of wells with Theil-Sen slopes of >0 m per year	Percent of wells with Theil-Sen slopes of >0.5 m per year
Fish Springs Flat	791	2	1 well for every 396 km²	0.01	100%	0%
Antelope Valley near Topaz Lake	282	18	1 well for every 16 km²	0.01	56%	0%
Smoke Creek Desert	864	1	1 well for every 864 km²	0.01	100%	0%
Odra Gate	303	4	1 well for every 76 km²	0.01	75%	0%
Yakima Basin (Columbia Plateau Regional Aquifer System)	9981	7	1 well for every 1426 km²	0.01	57%	0%
Southeastern Eucla Basin (Eucla Basin)	6424	104	1 well for every 62 km²	0.01	53%	0%
Western Armorican Massif (Armorican Massif)	23180	42	1 well for every 552 km²	0.01	62%	0%
Eastern Paris Basin (Paris Basin)	41666	129	1 well for every 323 km²	0.00	57%	2%
Manetin Basin	234	1	1 well for every 234 km²	0.00	100%	0%
Sahneh-Bistoon Plain	573	25	1 well for every 23 km²	0.00	52%	4%
North American Basin (Sacramento Valley of the Central Valley)	1510	1315	1 well for every 1 km²	0.00	54%	1%
Gold Flat	797	2	1 well for every 399 km²	0.00	100%	0%
Western Champlain Valley Lowlands	1852	2	1 well for every 926 km²	0.00	100%	0%
Vitoria-Gasteiz Quaternary Aquifer	582	5	1 well for every 116 km²	0.00	80%	0%
Eastern Carrizo-Wilcox (Carrizo-Wilcox)	57217	438	1 well for every 131 km²	0.00	53%	8%
Viscaino Aquifer	4002	42	1 well for every 95 km²	0.00	52%	2%
Southern Zululand Primary Coastal Aquifer	567	11	1 well for every 52 km²	0.00	64%	0%
Elandsbaai and Strandfontein Coastal Aquifers	2648	65	1 well for every 41 km²	0.00	52%	8%
Rockland Valley (Eastern Snake River Plain)	435	15	1 well for every 29 km²	0.00	60%	0%
Sarcobatus Flat	368	4	1 well for every 92 km²	0.00	100%	0%
Northern High Plains (High Plains)	259376	5421	1 well for every 48 km²	0.00	52%	2%
Gallatin Valley	2162	4	1 well for every 541 km²	0.00	50%	0%
Castle Hayne Aquifer	9991	46	1 well for every 217 km²	0.00	54%	2%
San Ramon Valley	40	34	1 well for every 1 km²	0.00	59%	0%
Lower Rio Paz Aquifer (Rio Paz Aquifer)	405	3	1 well for every 135 km²	0.00	67%	0%
Simi Valley	64	136	1 well for every 0 km²	0.00	56%	1%
Eastern Malwa Plateau (Malwa Plateau)	25218	80	1 well for every 315 km²	0.00	51%	6%
East Bay Plain	346	1943	1 well for every 0 km²	0.00	56%	0%
Cache Valley	1641	25	1 well for every 66 km²	0.00	60%	0%
Ruby Valley	1670	2	1 well for every 835 km²	0.00	50%	0%
Upper Carbonate Aquifer (Northern Midwest Aquifer System)	11793	7	1 well for every 1685 km²	0.00	57%	0%
Tokio-Woodbine Aquifer	4710	80	1 well for every 59 km²	0.00	54%	1%
Plymouth-Carver-Kingston-Duxburya Aquifer System	978	17	1 well for every 58 km²	0.00	65%	6%
Upper Tuolumne Basin (Sierra Nevada Foothills)	1807	191	1 well for every 9 km²	0.00	55%	0%
Carman Sand and Southern Carbonate Rock Aquifers (Manitoba Carbonate Aquifer System)	5639	126	1 well for every 45 km²	0.00	51%	0%
Central Dezful-Andimeshk Plain (Dezful-Andimeshk Plain)	2064	69	1 well for every 30 km²	0.00	52%	0%
Tejo Alluvium	1337	2	1 well for every 669 km²	0.00	50%	0%
Northern St. Lawrence Lowlands (St. Lawrence Lowlands)	14318	27	1 well for every 530 km²	0.00	59%	0%
Cretaceous Limestone and Guadalajara Detrital Aquifers	3268	27	1 well for every 121 km²	0.00	52%	7%
Crawford Upland (Interior Low Plateaus)	5107	1	1 well for every 5107 km²	0.00	100%	0%
Bayazeh Basin	828	12	1 well for every 69 km²	0.00	50%	0%

Study area	Area (km^2)	Number of monitoring wells	Density of monitoring wells (with sufficient groundwater level observations for analyses) within the study area	Median Theil-Sen slope among monitoring wells *(positive values indicate deeper depths to water over time)*	Percent of wells with Theil-Sen slopes of >0 m per year	Percent of wells with Theil-Sen slopes of >0.5 m per year
Camargue Plain	1396	1	1 well for every 1396 km^2	0.00	100%	0%
Skull Valley	1298	6	1 well for every 216 km^2	0.00	50%	0%
Southwestern Bohemian Cretaceous Basin (Bohemian Cretaceous Basin)	852	4	1 well for every 213 km^2	0.00	50%	0%
Crau Plain	1137	27	1 well for every 42 km^2	0.00	59%	0%
Southwestern Aquitaine Basin (Aquitaine Basin)	9271	24	1 well for every 386 km^2	0.00	63%	0%
Bridgeport Valley	73	17	1 well for every 4 km^2	0.00	59%	0%
Calama Basin	2641	4	1 well for every 660 km^2	0.00	50%	0%
Cactus Flat	678	7	1 well for every 97 km^2	0.00	57%	0%
Chimbarongo and San Fernando Aquifers (Chilean Central Valley)	578	3	1 well for every 193 km^2	0.00	67%	0%
Polda Basin (Eucla Basin)	2507	83	1 well for every 30 km^2	0.00	54%	0%
Mimosa Syncline and Eastern Surat Basin (Great Artesian Basin)	64955	217	1 well for every 299 km^2	0.00	52%	8%
Lannemezan Plateau (Aquitaine Basin)	18348	94	1 well for every 195 km^2	0.00	52%	12%
Western Fomanat Plain	1643	55	1 well for every 30 km^2	0.00	53%	0%
Huerva-Perejiles and Monreal-Calamocha Aquifers	2489	16	1 well for every 156 km^2	0.00	50%	19%
Southern Owens Valley	1350	58	1 well for every 23 km^2	0.00	53%	0%
Ashland Flats	148	8	1 well for every 19 km^2	0.00	63%	0%
Southern Jalpa-Juchipila Aquifer	244	7	1 well for every 35 km^2	0.00	57%	29%
Odessa Subregion (Columbia Plateau Regional Aquifer System)	7346	25	1 well for every 294 km^2	0.00	52%	0%
Pilot Valley	826	1	1 well for every 826 km^2	0.00	100%	0%
Rio Piaxtla Aquifer	347	5	1 well for every 69 km^2	0.00	60%	0%
Lower Copiapo Valley (Copiapo Valley)	503	6	1 well for every 84 km^2	0.00	50%	33%
Orange County Coastal Plain (Los Angeles Basin)	966	5990	1 well for every 0 km^2	0.00	50%	1%
Deh Sabz Basin (Kabul Basin)	390	8	1 well for every 49 km^2	0.00	50%	13%
Lower Arkansas River Western Reach	2788	83	1 well for every 34 km^2	0.00	49%	0%
Southern Duero Basin (Duero Basin)	7969	25	1 well for every 319 km^2	0.00	48%	8%
Astanea-Kochesfahan Plain	1906	51	1 well for every 37 km^2	0.00	49%	0%
Ahar-Varzeghan Basin	1639	37	1 well for every 44 km^2	0.00	49%	0%
Roma Shelf and Northern Surat Basin (Great Artesian Basin)	33598	41	1 well for every 819 km^2	0.00	46%	5%
Sydney Basin	4143	21	1 well for every 197 km^2	0.00	48%	0%
Kladno-Rakovnik Basin	1514	3	1 well for every 505 km^2	0.00	33%	0%
Ralston Valley	1375	1	1 well for every 1375 km^2	0.00	0%	0%
Weiser River Valley	1111	6	1 well for every 185 km^2	0.00	50%	0%
Columbus Salt Marsh Valley	276	1	1 well for every 276 km^2	0.00	0%	0%
Mud Meadows	253	2	1 well for every 127 km^2	0.00	50%	0%
Garden Basin	683	1	1 well for every 683 km^2	0.00	0%	0%
Leadville Limestone Aquifer	9807	5	1 well for every 1961 km^2	0.00	40%	0%
Eastern Champlain Valley Lowlands	8869	6	1 well for every 1478 km^2	0.00	50%	0%
Lahontan Valley	1730	102	1 well for every 17 km^2	0.00	49%	0%
Hengchun Plain Karst Aquifer System	80	5	1 well for every 16 km^2	0.00	40%	0%
Northern Canterbury Plains	3811	327	1 well for every 12 km^2	0.00	49%	1%
Ukiah Valley	145	145	1 well for every 1 km^2	0.00	50%	0%

Study area	Area (km^2)	Number of monitoring wells	Density of monitoring wells (with sufficient groundwater level observations for analyses) within the study area	Median Theil-Sen slope among monitoring wells *(positive values indicate deeper depths to water over time)*	Percent of wells with Theil-Sen slopes of >0 m per year	Percent of wells with Theil-Sen slopes of >0.5 m per year
Chicxulub Sedimentary Basin (Yucatan Peninsula)	14250	10	1 well for every 1425 km^2	0.00	50%	0%
Upper Arkansas River Basin	549	12	1 well for every 46 km^2	0.00	50%	0%
Lida Valley	506	1	1 well for every 506 km^2	0.00	0%	0%
Uley Basin (Eucla Basin)	279	68	1 well for every 4 km^2	0.00	49%	0%
Roosevelt Valley and Uinta River Basin and Pelican Lake region (Uinta Basin)	1914	5	1 well for every 383 km^2	0.00	40%	0%
Tedori Alluvial Fan	274	3	1 well for every 91 km^2	0.00	33%	0%
Halilan Basin	222	6	1 well for every 37 km^2	0.00	33%	17%
Saint Marys Basin (Maritimes Basin)	2457	4	1 well for every 614 km^2	0.00	50%	0%
Allier Basin	4579	35	1 well for every 131 km^2	0.00	49%	3%
Long Valley in central Nevada	578	5	1 well for every 116 km^2	0.00	40%	0%
Pittsburg Plain	59	222	1 well for every 0 km^2	0.00	45%	0%
Stellarton Basin (Maritimes Basin)	1290	1	1 well for every 1290 km^2	0.00	0%	0%
North Seeland Aquifer	60	2	1 well for every 30 km^2	0.00	50%	0%
Lillo-Quintanar Aquifer	999	3	1 well for every 333 km^2	0.00	33%	0%
Biscayne Aquifer	9935	261	1 well for every 38 km^2	0.00	42%	0%
Northcentral Piedmont Upland (Piedmont Plateau)	24072	76	1 well for every 317 km^2	0.00	45%	1%
Long Island	3972	609	1 well for every 7 km^2	0.00	46%	0%
Bluffton Till Plain (Central Lowland Till Plain)	23952	26	1 well for every 921 km^2	0.00	42%	0%
Shigenobu-gawa Alluvial Fan	191	8	1 well for every 24 km^2	0.00	38%	0%
Western Prince Edward Island (Prince Edward Island)	1549	3	1 well for every 516 km^2	0.00	33%	0%
Southeastern Allegheny Plateau (Appalachian Plateaus)	23152	6	1 well for every 3859 km^2	0.00	50%	0%
Ogden Valley	115	4	1 well for every 29 km^2	0.00	50%	0%
New Hampshire Seacoast	618	3	1 well for every 206 km^2	0.00	33%	0%
Uitenhage Artesian Basin	1680	7	1 well for every 240 km^2	0.00	43%	0%
Stockton Plateau (Edwards-Trinity Aquifer System)	33245	127	1 well for every 262 km^2	0.00	49%	5%
Mount Vernon Hill County (Central Lowland Till Plain)	27711	6	1 well for every 4619 km^2	0.00	50%	0%
Grand Forks Aquifer	63	1	1 well for every 63 km^2	0.00	0%	0%
Lanyang Plain	355	37	1 well for every 10 km^2	0.00	41%	0%
Peedee and Black Creek and Cape Fear Aquifers	34016	76	1 well for every 448 km^2	0.00	47%	12%
Scotts Valley	33	32	1 well for every 1 km^2	0.00	44%	0%
Northern Massa Basin (Souss-Chtouka Basin)	741	3	1 well for every 247 km^2	0.00	33%	0%
Newcastle Till Plain (Central Lowland Till Plain)	20988	144	1 well for every 146 km^2	0.00	47%	0%
South Rewa Basin (Son-Mahandai Valley Basins)	7704	26	1 well for every 296 km^2	0.00	50%	0%
Northern Guam Lens Aquifer	259	16	1 well for every 16 km^2	0.00	38%	0%
Upper Souss Basin (Souss-Chtouka Basin)	1292	5	1 well for every 258 km^2	0.00	40%	0%
Southwestern Flatwoods (Floridan Aquifer System)	12393	207	1 well for every 60 km^2	0.00	39%	1%
Northern Guarani Aquifer (Guarani Aquifer System)	419237	18	1 well for every 23291 km^2	0.00	44%	6%
Santa Rosa Valley	487	1660	1 well for every 0 km^2	0.00	48%	0%
Northwestern Deccan Basalt Plateau (Deccan Plateau)	18659	4	1 well for every 4665 km^2	0.00	50%	0%
West Jutland Quaternary and Tertiary Sand Aquifer System	14018	180	1 well for every 78 km^2	0.00	40%	0%

Study area	Area (km²)	Number of monitoring wells	Density of monitoring wells (with sufficient groundwater level observations for analyses) within the study area	Median Theil-Sen slope among monitoring wells *(positive values indicate deeper depths to water over time)*	Percent of wells with Theil-Sen slopes of >0 m per year	Percent of wells with Theil-Sen slopes of >0.5 m per year
Rudsar Coastal Area	482	50	1 well for every 10 km^2	0.00	36%	0%
Lower Amargosa Valley	473	8	1 well for every 59 km^2	0.00	38%	0%
Salzburg Basin	499	5	1 well for every 100 km^2	0.00	40%	0%
Smith River Plain	125	88	1 well for every 1 km^2	0.00	45%	0%
Lolo-Bitterroot Valley (Lolo-Bitterroot-Missoula Valleys)	1381	2	1 well for every 691 km^2	0.00	0%	0%
Upper Poso Basin (Sierra Nevada Foothills)	670	5	1 well for every 134 km^2	0.00	40%	0%
Pearl and Chattahoochee Aquifer System	35594	50	1 well for every 712 km^2	0.00	48%	2%
Lower Liaohe River Plain (Liaohe River Basin)	69968	25	1 well for every 2799 km^2	0.00	44%	4%
Northern Piedmont Upland (Piedmont Plateau)	15989	261	1 well for every 61 km^2	0.00	42%	0%
Central Karoo Basin (Main Karoo Basin)	128536	9	1 well for every 14282 km^2	0.00	44%	0%
Xinzo de Limia Aquifer	369	2	1 well for every 185 km^2	0.00	0%	0%
Springfield Plain (Central Lowland Till Plain)	39234	23	1 well for every 1706 km^2	0.00	48%	0%
Santa Cruz Coastal Terraces	81	370	1 well for every 0 km^2	0.00	45%	0%
Flint Hills Upland (Osage Cuestas Aquifer System)	22649	21	1 well for every 1079 km^2	0.00	38%	0%
Houlong Alluvial Plain Aquifer (Hsinchu-Miaoli Coastal Aquifer System)	110	13	1 well for every 8 km^2	0.00	46%	0%
Kavir Allahabad Basin	376	9	1 well for every 42 km^2	0.00	33%	0%
Transboundary Odanah Shale	14918	32	1 well for every 466 km^2	−0.01	38%	0%
Lower Fraser Valley (Puget Sound Lowland)	547	7	1 well for every 78 km^2	−0.01	43%	0%
Avra Valley (Tucson Basin and Avra Valley)	5681	26	1 well for every 219 km^2	−0.01	35%	0%
Oja Alluvial Aquifers	274	4	1 well for every 69 km^2	−0.01	25%	0%
Ranas Orbigo-Esla and Esla-Cea Aquifers	4581	36	1 well for every 127 km^2	−0.01	50%	6%
Palouse Slope (Columbia Plateau Regional Aquifer System)	15260	3	1 well for every 5087 km^2	−0.01	33%	0%
Dakota Aquifer System	110336	49	1 well for every 2252 km^2	−0.01	37%	0%
Uncompahgre Basin	598	13	1 well for every 46 km^2	−0.01	46%	0%
Hiroshima Plain	119	9	1 well for every 13 km^2	−0.01	11%	0%
Southern Taichung Basin (Taichung Basin)	162	6	1 well for every 27 km^2	−0.01	50%	0%
Delmarva Peninsula (North Atlantic Coastal Plain)	18153	294	1 well for every 62 km^2	−0.01	42%	0%
Camas Prairie	1107	7	1 well for every 158 km^2	−0.01	43%	0%
Powder River Basin (Northern Great Plains)	63642	12	1 well for every 5304 km^2	−0.01	33%	8%
Eureka and Eel River and Mad River Plains	663	728	1 well for every 1 km^2	−0.01	36%	0%
Sandlands Aquifer	3150	57	1 well for every 55 km^2	−0.01	44%	0%
Upper American and Auburn Basins (Sierra Nevada Foothills)	2553	401	1 well for every 6 km^2	−0.01	40%	0%
Central Adelaide Plains (St Vincent Basin)	390	169	1 well for every 2 km^2	−0.01	42%	2%
Central Minnesota Surficial and Buried Sand and Gravel Aquifers	75252	256	1 well for every 294 km^2	−0.01	21%	0%
Dagh -e Talleh Hamid Basin	846	8	1 well for every 106 km^2	−0.01	25%	0%
Dry Lake Valley and Dreamer Valley	1359	2	1 well for every 680 km^2	−0.01	0%	0%
Central Wabash and Bloomington Ridged Plain (Central Lowland Till Plain)	25827	50	1 well for every 517 km^2	−0.01	46%	0%
Southern Canterbury Plains	2060	149	1 well for every 14 km^2	−0.01	37%	1%
Western Washoe Valley	41	6	1 well for every 7 km^2	−0.01	50%	0%
Lower Gila Basin	6409	2	1 well for every 3205 km^2	−0.01	50%	0%
Poway Valley	27	141	1 well for every 0 km^2	−0.01	35%	0%

Study area	Area (km^2)	Number of monitoring wells	Density of monitoring wells (with sufficient groundwater level observations for analyses) within the study area	Median Theil-Sen slope among monitoring wells *(positive values indicate deeper depths to water over time)*	Percent of wells with Theil-Sen slopes of >0 m per year	Percent of wells with Theil-Sen slopes of >0.5 m per year
Sierra Grossa Karst Aquifer System	600	12	1 well for every 50 km^2	−0.01	42%	25%
Milk River	22986	9	1 well for every 2554 km^2	−0.01	33%	0%
Mulgrave Aquifer	341	38	1 well for every 9 km^2	−0.01	24%	0%
Ha Tinh Coastal Quaternary Aquifers	959	5	1 well for every 192 km^2	−0.01	40%	0%
Fort Bragg Terrace	9	35	1 well for every 0 km^2	−0.01	40%	0%
Bhuj Sandstone Aquifer System	6139	16	1 well for every 384 km^2	−0.01	50%	13%
Eastern Kankakee Plains	3485	5	1 well for every 697 km^2	−0.01	40%	0%
Garden Valley	101	1	1 well for every 101 km^2	−0.01	0%	0%
West-central Kanto Plain (Kanto Plain)	1473	7	1 well for every 210 km^2	−0.01	14%	0%
Western Surat Basin (Great Artesian Basin)	65684	109	1 well for every 603 km^2	−0.01	28%	0%
Heber Valley	123	12	1 well for every 10 km^2	−0.01	25%	0%
North Carolina and Virginia Coastal Plain (North Atlantic Coastal Plain)	49143	295	1 well for every 167 km^2	−0.01	38%	0%
Lower Coastal Plain (Floridan Aquifer System)	18560	47	1 well for every 395 km^2	−0.01	38%	2%
Upper Dry and King Basins (Sierra Nevada Foothills)	1078	8	1 well for every 135 km^2	−0.01	50%	0%
Eastern Armorican Massif (Armorican Massif)	36418	96	1 well for every 379 km^2	−0.01	41%	0%
Western Cambrian-Ordovician Aquifers (Northern Midwest Aquifer System)	59488	82	1 well for every 725 km^2	−0.01	38%	1%
Grazer Feld	233	1	1 well for every 233 km^2	−0.01	0%	0%
Shiramin Basin	57	19	1 well for every 3 km^2	−0.01	32%	5%
Tipton Till Plain (Central Lowland Till Plain)	11793	15	1 well for every 786 km^2	−0.01	27%	0%
Upper Mokelumne Basin (Sierra Nevada Foothills)	1280	167	1 well for every 8 km^2	−0.01	42%	1%
Guelmim Basin	1307	18	1 well for every 73 km^2	−0.01	39%	0%
Madison Aquifer	2010	1	1 well for every 2010 km^2	−0.01	0%	0%
Shush Plain (Dezful-Andimeshk Plain)	1473	67	1 well for every 22 km^2	−0.01	46%	9%
Anarak Basin	1404	9	1 well for every 156 km^2	−0.01	11%	0%
Upper Rio Fuerte Aquifer	529	23	1 well for every 23 km^2	−0.01	30%	0%
Southern Carbonate Rock Aquifers (Manitoba Carbonate Aquifer System)	6031	95	1 well for every 63 km^2	−0.01	38%	0%
Sydney Maritimes Basin (Maritimes Basin)	890	1	1 well for every 890 km^2	−0.01	0%	0%
Lower Herbert Aquifer	1440	77	1 well for every 19 km^2	−0.01	21%	0%
Intermediate Aquifer (Floridan Aquifer System)	17405	388	1 well for every 45 km^2	−0.01	44%	1%
Paskapoo-Scollard	65768	26	1 well for every 2530 km^2	−0.01	19%	0%
Upper Bear Basin (Sierra Nevada Foothills)	943	79	1 well for every 12 km^2	−0.01	41%	0%
Tafresh Basin	53	12	1 well for every 4 km^2	−0.01	33%	0%
Columbus Lowland and Darby Plain (Central Lowland Till Plain)	5713	76	1 well for every 75 km^2	−0.01	38%	0%
Yoshino Basin	332	11	1 well for every 30 km^2	−0.01	9%	0%
Bellefontaine Upland and Mad River Interlobate Plain (Central Lowland Till Plain)	1902	23	1 well for every 83 km^2	−0.01	43%	0%
Northern Mallee (Murray Basin)	21895	1	1 well for every 21895 km^2	−0.01	0%	0%
Napa Valley	231	118	1 well for every 2 km^2	−0.01	35%	1%
Westside Middle Indus Plain (Indus Basin)	24478	113	1 well for every 217 km^2	−0.01	43%	0%
Palen and Chuckwalla Valleys	1774	6	1 well for every 296 km^2	−0.01	33%	0%
Helena Valley FIll	826	1	1 well for every 826 km^2	−0.01	0%	0%
Pockmarked Terrain and Northern Peninsula (Yucatan Peninsula)	6257	9	1 well for every 695 km^2	−0.01	22%	0%

Study area	Area (km^2)	Number of monitoring wells	Density of monitoring wells (with sufficient groundwater level observations for analyses) within the study area	Median Theil-Sen slope among monitoring wells *(positive values indicate deeper depths to water over time)*	Percent of wells with Theil-Sen slopes of >0 m per year	Percent of wells with Theil-Sen slopes of >0.5 m per year
Kinokawa Valley	143	7	1 well for every 20 km^2	−0.01	0%	0%
Upper Tule Basin (Sierra Nevada Foothills)	1200	11	1 well for every 109 km^2	−0.01	27%	0%
West Zealand Quaternary Sand Aquifer System	1942	24	1 well for every 81 km^2	−0.01	42%	0%
San Jose de Guaymas Aquifer	468	7	1 well for every 67 km^2	−0.01	43%	0%
Western El Palmar Aquifer	2179	23	1 well for every 95 km^2	−0.01	43%	17%
Pingtung Plain	1164	119	1 well for every 10 km^2	−0.01	46%	1%
Gandia-Denia Coastal Plain	213	13	1 well for every 16 km^2	−0.01	46%	8%
Assiniboine Delta	3934	124	1 well for every 32 km^2	−0.01	27%	0%
Dayton Valley (Carson River Basin)	348	90	1 well for every 4 km^2	−0.01	46%	1%
Coatzacoalcos Coastal Aquifer (Coastal Plain of Veracruz)	11209	24	1 well for every 467 km^2	−0.01	50%	0%
Rio Fuerte Coastal Aquifer	3098	8	1 well for every 387 km^2	−0.01	25%	0%
Parker Valley (Blythe Basin)	716	7	1 well for every 102 km^2	−0.01	29%	0%
Rio Mocorito Coastal Aquifer	1261	24	1 well for every 53 km^2	−0.01	46%	0%
Eastern Flatwoods Southshores (Floridan Aquifer System)	25461	171	1 well for every 149 km^2	−0.01	22%	0%
Sonsonate Aquifer	558	4	1 well for every 140 km^2	−0.01	25%	0%
Narbonne Basin	895	11	1 well for every 81 km^2	−0.01	36%	0%
Cape Cod Aquifer	1379	236	1 well for every 6 km^2	−0.01	29%	0%
Rio Presido Aquifer	1071	7	1 well for every 153 km^2	−0.02	14%	0%
Grande Terre Limestone Aquifer	595	15	1 well for every 40 km^2	−0.02	20%	0%
Northern Allegheny Plateau (Appalachian Plateaus)	27131	53	1 well for every 512 km^2	−0.02	25%	0%
Eastern Flatwoods Northshores (Floridan Aquifer System)	4965	42	1 well for every 118 km^2	−0.02	40%	0%
Ocala Uplift (Floridan Aquifer System)	35660	393	1 well for every 91 km^2	−0.02	35%	0%
Campo de Cartagena Multilayered Aquifer	1529	22	1 well for every 70 km^2	−0.02	50%	18%
Silurian and Overlying Unconsolidated Aquifers (Southern Ontario Paleozoic Aquifer System)	13360	69	1 well for every 194 km^2	−0.02	19%	0%
Michigan Basin	62295	56	1 well for every 1112 km^2	−0.02	27%	0%
Southern Dezful-Andimeshk Plain (Dezful-Andimeshk Plain)	4113	101	1 well for every 41 km^2	−0.02	40%	0%
Coastal Maine Crystalline Aquifers	6045	1	1 well for every 6045 km^2	−0.02	0%	0%
Angas-Bremer Plains and Murray Estuary Aquifer System (Murray Basin)	1278	182	1 well for every 7 km^2	−0.02	35%	0%
Tehuantepec Aquifer	1835	20	1 well for every 92 km^2	−0.02	50%	5%
Indian Springs Valley	472	9	1 well for every 52 km^2	−0.02	11%	0%
Toyooka Basin	66	7	1 well for every 9 km^2	−0.02	29%	0%
Moulton Valley (Interior Low Plateaus)	6842	21	1 well for every 326 km^2	−0.02	29%	0%
Tifton Upland (Floridan Aquifer System)	14658	76	1 well for every 193 km^2	−0.02	43%	5%
San Bruno Aquifer	81	12	1 well for every 7 km^2	−0.02	33%	0%
Williston Basin (Northern Great Plains)	242272	77	1 well for every 3146 km^2	−0.02	34%	0%
Carmel Valley	34	40	1 well for every 1 km^2	−0.02	35%	0%
Mercury Valley	122	2	1 well for every 61 km^2	−0.02	0%	0%
Southern Hills (Gulf Coast Regional Aquifer System)	34191	224	1 well for every 153 km^2	−0.02	43%	4%
Malad Valley	526	8	1 well for every 66 km^2	−0.02	38%	0%
Eastern Prince Edward Island (Prince Edward Island)	2625	6	1 well for every 438 km^2	−0.02	33%	0%
Eastern Eromanga Basin (Great Artesian Basin)	290867	184	1 well for every 1581 km^2	−0.02	40%	7%

Study area	Area (km²)	Number of monitoring wells	Density of monitoring wells (with sufficient groundwater level observations for analyses) within the study area	Median Theil-Sen slope among monitoring wells *(positive values indicate deeper depths to water over time)*	Percent of wells with Theil-Sen slopes of >0 m per year	Percent of wells with Theil-Sen slopes of >0.5 m per year
Central Allegheny Plateau (Appalachian Plateaus)	52939	17	1 well for every 3114 km²	−0.02	24%	0%
Limmat Valley	98	2	1 well for every 49 km²	−0.02	0%	0%
Seville-Carmona and Aljarafe and Guadalquivir Alluvial Aquifers (Guadalquivir Basin)	8851	30	1 well for every 295 km²	−0.02	40%	3%
West Coast Basin (Los Angeles Basin)	426	4597	1 well for every 0 km²	−0.02	39%	0%
Western Allegheny Plateau (Appalachian Plateaus)	17977	24	1 well for every 749 km²	−0.02	25%	0%
Central Piedmont Upland (Piedmont Plateau)	57273	21	1 well for every 2727 km²	−0.02	33%	0%
Holbox Fracture Zone (Yucatan Peninsula)	6071	14	1 well for every 434 km²	−0.02	0%	0%
Umatilla Basin and Horse Heaven Hills (Columbia Plateau Regional Aquifer System)	15408	16	1 well for every 963 km²	−0.02	31%	6%
Niagara Peninsula (Southern Ontario Paleozoic Aquifer System)	3532	17	1 well for every 208 km²	−0.02	12%	0%
Desert Valley in northern Nevada	2142	1	1 well for every 2142 km²	−0.02	0%	0%
Tualatin and Portland Basins (Willamette Valley)	4640	33	1 well for every 141 km²	−0.02	15%	0%
Nanaimo Lowlands	133	4	1 well for every 33 km²	−0.02	50%	0%
Terwilliger Valley	33	8	1 well for every 4 km²	−0.02	50%	13%
Northern Nuomin-Taoer System (Songnen Plain)	10909	1	1 well for every 10909 km²	−0.02	0%	0%
Winfield-Wood Lake and Kalamalka Lake Aquifers (Okanagan Basin)	94	5	1 well for every 19 km²	−0.02	40%	0%
Yucca Flat	365	12	1 well for every 30 km²	−0.02	8%	0%
Ojai Basin	39	73	1 well for every 1 km²	−0.02	48%	14%
Silurian-Devonian Aquifers in Northern Michigan	6289	2	1 well for every 3145 km²	−0.02	0%	0%
Northeast Missouri Carbonates (Northern Midwest Aquifer System)	29764	30	1 well for every 992 km²	−0.02	30%	7%
Thal Doab (Indus Basin)	33296	177	1 well for every 188 km²	−0.02	37%	0%
Central Eucla Basin (Eucla Basin)	63315	87	1 well for every 728 km²	−0.02	21%	0%
Northeast Zealand Limestone and Sand Aquifer System	1818	49	1 well for every 37 km²	−0.02	31%	0%
Engle Basin	836	4	1 well for every 209 km²	−0.02	25%	0%
Pajaro Valley	222	354	1 well for every 1 km²	−0.02	31%	1%
Eastern Silurian-Devonian Aquifers (Northern Midwest Aquifer System)	30268	229	1 well for every 132 km²	−0.02	19%	0%
Arecibo-Manati and Vega Baja-Toa Baja Regions (Puerto Rico North Coast Karst Aquifer)	899	25	1 well for every 36 km²	−0.02	20%	0%
Southcentral Piedmont Upland (Piedmont Plateau)	44094	39	1 well for every 1131 km²	−0.02	31%	0%
Southern Guadiana Menor Basin	1430	19	1 well for every 75 km²	−0.02	47%	11%
Aguadilla-Hatillo Region (Puerto Rico North Coast Karst Aquifer)	598	6	1 well for every 100 km²	−0.02	50%	0%
South Platte Basin	13779	15	1 well for every 919 km²	−0.02	27%	0%
Palomas Basin	634	3	1 well for every 211 km²	−0.02	33%	0%
Central Eromanga Depocenter (Great Artesian Basin)	214471	7	1 well for every 30639 km²	−0.02	43%	14%
Abbotsford-Sumas Aquifer (Puget Sound Lowland)	530	15	1 well for every 35 km²	−0.02	7%	0%
MiyanAb Shushtar Basin (Dezful-Andimeshk Plain)	1299	78	1 well for every 17 km²	−0.02	36%	4%
Wisconsin Precambrian Aquifer	65963	45	1 well for every 1466 km²	−0.02	18%	0%
Western Kankakee Plains	2820	2	1 well for every 1410 km²	−0.02	0%	0%
Dougherty Plain and Marianna Lowlands (Floridan Aquifer System)	18608	246	1 well for every 76 km²	−0.02	36%	2%
Albuquerque Basin (Middle Rio Grande)	7056	247	1 well for every 29 km²	−0.02	33%	3%
Western Cape Breton (Maritimes Basin)	2151	1	1 well for every 2151 km²	−0.03	0%	0%

Study area	Area (km^2)	Number of monitoring wells	Density of monitoring wells (with sufficient groundwater level observations for analyses) within the study area	Median Theil-Sen slope among monitoring wells *(positive values indicate deeper depths to water over time)*	Percent of wells with Theil-Sen slopes of >0 m per year	Percent of wells with Theil-Sen slopes of >0.5 m per year
Illinoian Till Plain (Central Lowland Till Plain)	4203	9	1 well for every 467 km^2	−0.03	33%	0%
Eagle Valley (Carson River Basin)	179	63	1 well for every 3 km^2	−0.03	30%	10%
Palo Verde Valley (Blythe Basin)	1022	198	1 well for every 5 km^2	−0.03	12%	0%
Northern Mega Aquifer System (Mega Aquifer System)	143434	1	1 well for every 143434 km^2	−0.03	0%	0%
White River Valley	2159	2	1 well for every 1080 km^2	−0.03	50%	0%
Northern Adelaide Plains (St Vincent Basin)	2105	255	1 well for every 8 km^2	−0.03	37%	0%
Bear Lake Valley	1207	5	1 well for every 241 km^2	−0.03	20%	0%
Southern Namaqualand Coastal Aquifer (Namaqualand Coastal Aquifers)	4336	1	1 well for every 4336 km^2	−0.03	0%	0%
Devonian and Overlying Unconsolidated Aquifers (Southern Ontario Paleozoic Aquifer System)	21440	70	1 well for every 306 km^2	−0.03	24%	0%
Mendez - San Fernando Aquifer	5913	1	1 well for every 5913 km^2	−0.03	0%	0%
Three Lakes Valley	755	6	1 well for every 126 km^2	−0.03	33%	0%
Zacatepec Aquifer	652	37	1 well for every 18 km^2	−0.03	41%	14%
Mimbres Basin	8365	65	1 well for every 129 km^2	−0.03	45%	9%
Galion Glaciated Low Plateau (Central Lowland Till Plain)	4432	5	1 well for every 886 km^2	−0.03	20%	0%
Nara Basin	426	14	1 well for every 30 km^2	−0.03	0%	0%
Toucian-Fongshan Alluvial Plain (Hsinchu-Miaoli Coastal Aquifer System)	175	11	1 well for every 16 km^2	−0.03	36%	0%
Southwestern Allegheny Plateau (Appalachian Plateaus)	19131	28	1 well for every 683 km^2	−0.03	11%	0%
Southern Highland Rim (Interior Low Plateaus)	4055	4	1 well for every 1014 km^2	−0.03	25%	0%
Jhonggang Alluvial Plain (Hsinchu-Miaoli Coastal Aquifer System)	66	5	1 well for every 13 km^2	−0.03	20%	0%
San Lucas Aquifer	38	6	1 well for every 6 km^2	−0.03	0%	0%
San Felipe-Punta Estrella Aquifer	451	2	1 well for every 226 km^2	−0.03	50%	0%
Upper Stanislaus and Rock Creek Basins (Sierra Nevada Foothills)	1356	40	1 well for every 34 km^2	−0.03	35%	0%
Southern Malwa Plateau (Malwa Plateau)	38079	137	1 well for every 278 km^2	−0.03	43%	5%
Culiacan Coastal Plain	5164	48	1 well for every 108 km^2	−0.03	35%	0%
Santa Ana Aquifer	154	3	1 well for every 51 km^2	−0.03	0%	0%
Interlake Carbonate Rock Aquifers (Manitoba Carbonate Aquifer System)	9031	18	1 well for every 502 km^2	−0.03	11%	0%
Proserpine Basin	819	77	1 well for every 11 km^2	−0.03	25%	0%
Northern Cumberland Plateau and Mountains (Appalachian Plateaus)	28114	1	1 well for every 28114 km^2	−0.03	0%	0%
Interlake Carbonate Rock and Winnipeg Aquifers (Manitoba Carbonate Aquifer System)	9940	9	1 well for every 1104 km^2	−0.03	11%	0%
Napanee Limestone Plain	3121	17	1 well for every 184 km^2	−0.03	12%	0%
Saanich Peninsula	108	6	1 well for every 18 km^2	−0.03	0%	0%
Velez Aquifer	54	1	1 well for every 54 km^2	−0.03	0%	0%
Arbuckle-Simpson Aquifer	2017	10	1 well for every 202 km^2	−0.03	40%	0%
Upper Cosumnes Basin (Sierra Nevada Foothills)	1411	70	1 well for every 20 km^2	−0.03	23%	0%
Tahoe Valley South Basin	70	163	1 well for every 0 km^2	−0.03	14%	0%
Bruneau-Grandview Area (Western Snake River Plain)	4456	19	1 well for every 235 km^2	−0.03	47%	16%
Actopan Aquifer (Coastal Plain of Veracruz)	501	30	1 well for every 17 km^2	−0.03	37%	0%
Southern Odanah Shale	10541	78	1 well for every 135 km^2	−0.03	13%	0%
Ribstone Creek and Northern Judith River Aquifer (Bearpaw-Judith River Aquifer System)	47412	17	1 well for every 2789 km^2	−0.03	24%	0%

Study area	Area (km^2)	Number of monitoring wells	Density of monitoring wells (with sufficient groundwater level observations for analyses) within the study area	Median Theil-Sen slope among monitoring wells *(positive values indicate deeper depths to water over time)*	Percent of wells with Theil-Sen slopes of >0 m per year	Percent of wells with Theil-Sen slopes of >0.5 m per year
Eastern Celje Basin (Celje Basin)	87	2	1 well for every 44 km^2	−0.03	0%	0%
Central Prince Edward Island (Prince Edward Island)	1767	5	1 well for every 353 km^2	−0.03	20%	0%
Upper La Paz Basin (La Paz Basin)	674	2	1 well for every 337 km^2	−0.03	50%	0%
Cypress-Eastend Aquifer (Bearpaw-Judith River Aquifer System)	8875	3	1 well for every 2958 km^2	−0.03	33%	0%
Eastern Cambrian-Ordovician Aquifers (Northern Midwest Aquifer System)	55195	59	1 well for every 936 km^2	−0.03	24%	5%
Laramie Basin	4401	2	1 well for every 2201 km^2	−0.03	0%	0%
Central Lake Area (Floridan Aquifer System)	9464	111	1 well for every 85 km^2	−0.03	23%	0%
Sawtooth Valley and Bear Valley	232	2	1 well for every 116 km^2	−0.03	0%	0%
Eastside Lower Indus Valley (Indus Basin)	57275	2	1 well for every 28638 km^2	−0.03	50%	0%
Southern Cumberland Plateau - Pottsville Aquifer (Appalachian Plateaus)	12662	2	1 well for every 6331 km^2	−0.04	0%	0%
Virgin River Valley	954	3	1 well for every 318 km^2	−0.04	33%	0%
Eastern Duero Basin (Duero Basin)	16257	63	1 well for every 258 km^2	−0.04	40%	3%
Northern Duero Basin (Duero Basin)	12093	63	1 well for every 192 km^2	−0.04	46%	0%
Bundaberg Aquifer	2056	444	1 well for every 5 km^2	−0.04	17%	0%
Osaka Plain	1323	34	1 well for every 39 km^2	−0.04	15%	3%
Upper Humboldt Basin	5871	9	1 well for every 652 km^2	−0.04	44%	0%
Hatfield Valley	10899	4	1 well for every 2725 km^2	−0.04	0%	0%
Pine Valley	904	4	1 well for every 226 km^2	−0.04	0%	0%
Kyoto Basin	332	23	1 well for every 14 km^2	−0.04	0%	0%
Riding Mountain Odanah Shale	7583	8	1 well for every 948 km^2	−0.04	13%	0%
Fedami Basin	84	11	1 well for every 8 km^2	−0.04	36%	0%
Northcentral Valley and Ridge (Valley and Ridge Aquifer System)	45635	44	1 well for every 1037 km^2	−0.04	23%	9%
Big Bear Valley	99	110	1 well for every 1 km^2	−0.04	40%	5%
Thau Lagoon and Coastal Karstic System	332	8	1 well for every 42 km^2	−0.04	38%	25%
Round Valley and Pine Creek Basin	124	4	1 well for every 31 km^2	−0.04	50%	0%
Shawnee Hills (Interior Low Plateaus)	22911	37	1 well for every 619 km^2	−0.04	24%	3%
Orizaba Basin (Orizaba-Cordoba Basins)	87	22	1 well for every 4 km^2	−0.04	45%	0%
Ljubljana Barje (Ljubljana Alluvial Aquifer)	135	2	1 well for every 68 km^2	−0.04	0%	0%
Ljubljana Polje (Ljubljana Alluvial Aquifer)	113	6	1 well for every 19 km^2	−0.04	17%	0%
La Plana de la Galera and Tortosa Alluvial Aquifers	494	3	1 well for every 165 km^2	−0.04	33%	0%
Lafayette Area (Gulf Coast Regional Aquifer System)	65824	431	1 well for every 153 km^2	−0.04	27%	1%
Los Naranjos Aquifer (Coastal Plain of Veracruz)	5339	58	1 well for every 92 km^2	−0.04	29%	0%
El Cardito Aquifer	2940	18	1 well for every 163 km^2	−0.04	44%	17%
San Marcos-Palo Verde Aquifer	236	7	1 well for every 34 km^2	−0.04	14%	0%
Southern Chalk Aquifer (Chalk Aquifer System)	3494	2	1 well for every 1747 km^2	−0.04	0%	0%
Northcentral Hualien-Taitung Valley (Hualien-Taitung Valley)	212	3	1 well for every 71 km^2	−0.04	0%	0%
Guadalentin Valley	795	13	1 well for every 61 km^2	−0.05	46%	46%
Chaj Doab (Indus Basin)	13536	254	1 well for every 53 km^2	−0.05	22%	0%
Campo De Montiel Aquifer (Upper Guadiana Basin)	3612	41	1 well for every 88 km^2	−0.05	39%	20%
Northern Pitted Karst Plain (Yucatan Peninsula)	19270	4	1 well for every 4818 km^2	−0.05	0%	0%
Upper Calaveras Basin (Sierra Nevada Foothills)	1054	32	1 well for every 33 km^2	−0.05	25%	0%

Study area	Area (km²)	Number of monitoring wells	Density of monitoring wells (with sufficient groundwater level observations for analyses) within the study area	Median Theil-Sen slope among monitoring wells *(positive values indicate deeper depths to water over time)*	Percent of wells with Theil-Sen slopes of >0 m per year	Percent of wells with Theil-Sen slopes of >0.5 m per year
Oliver Sub-basin (Okanagan Basin)	106	4	1 well for every 27 km²	−0.05	50%	0%
Weipa Depression and Northern Carpentaria Basin (Great Artesian Basin)	43769	2	1 well for every 21885 km²	−0.05	50%	0%
Northeastern Karoo Basin (Main Karoo Basin)	52358	30	1 well for every 1745 km²	−0.05	30%	10%
Southern Tikapoo Valley	445	1	1 well for every 445 km²	−0.05	0%	0%
Big Sandy Valley	1561	1	1 well for every 1561 km²	−0.05	0%	0%
Northern Plane Basin	1733	283	1 well for every 6 km²	−0.05	14%	0%
Baza Basin	236	10	1 well for every 24 km²	−0.05	30%	0%
Upper Fresno Basin (Sierra Nevada Foothills)	783	64	1 well for every 12 km²	−0.05	23%	2%
Huatulco Aquifer	155	8	1 well for every 19 km²	−0.05	25%	0%
Eastern Arroyo Seco Valley	371	2	1 well for every 186 km²	−0.05	0%	0%
Bacon Terrace (Floridan Aquifer System)	7000	12	1 well for every 583 km²	−0.05	33%	0%
Howard East Aquifer	1385	21	1 well for every 66 km²	−0.05	29%	0%
Etla Valley (Central Valleys of Oaxaca)	497	22	1 well for every 23 km²	−0.05	32%	0%
Eastern San Joaquin Basin (San Joaquin Basin of the Central Valley)	3106	2482	1 well for every 1 km²	−0.05	31%	1%
Southern Valley and Ridge (Valley and Ridge Aquifer System)	23782	11	1 well for every 2162 km²	−0.05	18%	0%
Laidley Sub-basin (Great Artesian Basin)	6283	707	1 well for every 9 km²	−0.05	32%	4%
Dauphin Lake Area (Manitoba Carbonate Aquifer System)	7659	3	1 well for every 2553 km²	−0.05	0%	0%
Eastern Mississippi Embayment (Mississippi Embayment)	60634	170	1 well for every 357 km²	−0.05	20%	1%
Jamiltepec Aquifer	441	22	1 well for every 20 km²	−0.05	36%	0%
Southeast Lower Chao Phraya Basin (Lower Chao Phraya Basin)	4186	11	1 well for every 381 km²	−0.05	36%	0%
South Sound and Commencement Bay (Puget Sound Lowland)	5082	80	1 well for every 64 km²	−0.05	13%	0%
Meacham Aquifer	2512	2	1 well for every 1256 km²	−0.05	0%	0%
Okefenokee Basin (Floridan Aquifer System)	6801	11	1 well for every 618 km²	−0.05	36%	0%
Cold Lake-Beaver River Basin	5664	21	1 well for every 270 km²	−0.05	5%	0%
New Jersey Coastal Plain (North Atlantic Coastal Plain)	12554	1007	1 well for every 12 km²	−0.05	27%	2%
Keep River Plain	335	5	1 well for every 67 km²	−0.05	0%	0%
Oak Ridges Moraine	3948	45	1 well for every 88 km²	−0.05	22%	0%
Spanish Springs Valley	173	9	1 well for every 19 km²	−0.06	44%	0%
South American Basin (Sacramento Valley of the Central Valley)	1021	1374	1 well for every 1 km²	−0.06	27%	0%
Galisteo Aquifer	596	2	1 well for every 298 km²	−0.06	0%	0%
Dakota Aquifer in southeast Nebraska	5089	156	1 well for every 33 km²	−0.06	21%	0%
Apalachicola Delta (Floridan Aquifer System)	9858	1	1 well for every 9858 km²	−0.06	0%	0%
Duck Lake Valley	204	1	1 well for every 204 km²	−0.06	0%	0%
Eastern Vindhyan Basin	23103	84	1 well for every 275 km²	−0.06	43%	6%
Southern Piedmont Upland (Piedmont Plateau)	61856	112	1 well for every 552 km²	−0.06	21%	3%
Bajo Rio Bravo	6657	4	1 well for every 1664 km²	−0.06	25%	0%
Southern Willamette Valley (Willamette Valley)	6616	11	1 well for every 601 km²	−0.06	0%	0%
Northern Cambrian-Ordovician Aquifers (Northern Midwest Aquifer System)	20460	80	1 well for every 256 km²	−0.06	10%	1%
Gem Valley	674	12	1 well for every 56 km²	−0.06	25%	0%
Eastern Guadiana Menor Basin	1656	22	1 well for every 75 km²	−0.06	41%	0%
Eastside Middle Indus Plain (Indus Basin)	53045	355	1 well for every 149 km²	−0.06	25%	0%

Study area	Area (km^2)	Number of monitoring wells	Density of monitoring wells (with sufficient groundwater level observations for analyses) within the study area	Median Theil-Sen slope among monitoring wells *(positive values indicate deeper depths to water over time)*	Percent of wells with Theil-Sen slopes of >0 m per year	Percent of wells with Theil-Sen slopes of >0.5 m per year
Eastern Isonzo-Soca High Plain (Isonzo-Soca High Plain)	70	6	1 well for every 12 km^2	−0.06	17%	0%
Ozark Plateaus Aquifer System	172612	283	1 well for every 610 km^2	−0.06	27%	6%
Western Salvadoran Coastal Plain (Salvadoran Coastal Plain)	1098	16	1 well for every 69 km^2	−0.06	25%	0%
Upper Chowchilla Basin (Sierra Nevada Foothills)	1508	50	1 well for every 30 km^2	−0.06	22%	2%
Burdekin Delta	3324	818	1 well for every 4 km^2	−0.06	18%	0%
Rechna Doab (Indus Basin)	32700	430	1 well for every 76 km^2	−0.06	32%	0%
Rheintal Aquifer	223	8	1 well for every 28 km^2	−0.06	0%	0%
Sendai River Basin	71	10	1 well for every 7 km^2	−0.06	0%	0%
Laizhou Coastal Plain	17871	10	1 well for every 1787 km^2	−0.06	30%	20%
Guadalupe de Las Corrientes Aquifer	2353	8	1 well for every 294 km^2	−0.06	38%	38%
Upper Merced Basin (Sierra Nevada Foothills)	1361	7	1 well for every 194 km^2	−0.06	14%	0%
Dumfries Basin Aquifer	265	1	1 well for every 265 km^2	−0.07	0%	0%
Gushi Depression (Weihe Basin)	9965	6	1 well for every 1661 km^2	−0.07	33%	0%
Western Mississippi Embayment (Mississippi Embayment)	34240	325	1 well for every 105 km^2	−0.07	29%	1%
Nanchuan Area (Huangshui Basin)	53	3	1 well for every 18 km^2	−0.07	33%	0%
Central Chikugo-Saga Plain (Chikugo-Saga Plain)	408	2	1 well for every 204 km^2	−0.07	0%	0%
Broome-Wallal Aquifer System in West Canning Basin	9348	5	1 well for every 1870 km^2	−0.07	0%	0%
Northeastern Judith River (Bearpaw-Judith River Aquifer System)	23407	6	1 well for every 3901 km^2	−0.07	0%	0%
Marsh Valley (Central Snake River Plain)	205	6	1 well for every 34 km^2	−0.07	50%	0%
Norman Upland (Interior Low Plateaus)	4118	11	1 well for every 374 km^2	−0.07	0%	0%
Sea Island (Floridan Aquifer System)	22877	244	1 well for every 94 km^2	−0.07	17%	0%
Big Chino Valley	522	13	1 well for every 40 km^2	−0.07	46%	0%
San Jacinto Basin	1008	442	1 well for every 2 km^2	−0.07	39%	5%
Canatlan Valley	587	23	1 well for every 26 km^2	−0.07	43%	4%
Southcentral Piedmont Plain (North China Plain)	4215	11	1 well for every 383 km^2	−0.07	36%	0%
Cave Valley	454	1	1 well for every 454 km^2	−0.08	0%	0%
Mulege Aquifer	99	29	1 well for every 3 km^2	−0.08	3%	0%
Lanzhou Valley Basin	133	7	1 well for every 19 km^2	−0.08	29%	14%
Staten River Embayment and Central Carpentaria Basin (Great Artesian Basin)	81429	3	1 well for every 27143 km^2	−0.08	0%	0%
Tuli Karoo Aquifer	12618	2	1 well for every 6309 km^2	−0.08	50%	0%
Krsko-Brezice Polje Aquifer	152	2	1 well for every 76 km^2	−0.08	0%	0%
Northern Tikapoo Valley	611	3	1 well for every 204 km^2	−0.08	33%	0%
Biloela Basin	3207	344	1 well for every 9 km^2	−0.08	17%	1%
Guadiana Valley	1499	46	1 well for every 33 km^2	−0.08	41%	17%
Black Hills Uplift	18954	23	1 well for every 824 km^2	−0.08	22%	4%
Northern Eromanga Basin (Great Artesian Basin)	281571	154	1 well for every 1828 km^2	−0.09	36%	11%
Uraba Aquifer	2482	6	1 well for every 414 km^2	−0.09	33%	0%
Don River Delta Aquifer	363	190	1 well for every 2 km^2	−0.09	2%	0%
Tinguiririca Superior Aquifer (Tinguiririca Aquifer)	624	1	1 well for every 624 km^2	−0.09	0%	0%
Catahoula Area (Gulf Coast Regional Aquifer System)	23047	36	1 well for every 640 km^2	−0.09	19%	3%

Study area	Area (km^2)	Number of monitoring wells	Density of monitoring wells (with sufficient groundwater level observations for analyses) within the study area	Median Theil-Sen slope among monitoring wells *(positive values indicate deeper depths to water over time)*	Percent of wells with Theil-Sen slopes of >0 m per year	Percent of wells with Theil-Sen slopes of >0.5 m per year
Arriga Flats	181	93	1 well for every 2 km^2	−0.09	19%	0%
Temata Oasis (Middle Draa Basin)	717	1	1 well for every 717 km^2	−0.09	0%	0%
Kootenai Valley	1556	2	1 well for every 778 km^2	−0.09	0%	0%
Upper Guadalquivir Basin (Guadalquivir Basin)	3351	6	1 well for every 559 km^2	−0.09	33%	0%
Rio Baluarte Aquifer	1133	7	1 well for every 162 km^2	−0.09	0%	0%
Eastern Highland Rim (Interior Low Plateaus)	6817	31	1 well for every 220 km^2	−0.09	16%	0%
Coal Valley	747	1	1 well for every 747 km^2	−0.09	0%	0%
Kamyaran Plain	452	62	1 well for every 7 km^2	−0.09	31%	3%
Yaqui Valley	7471	20	1 well for every 374 km^2	−0.09	35%	10%
Golmud Alluvial Fan	2045	3	1 well for every 682 km^2	−0.09	33%	0%
Northwestern Malwa Plateau (Malwa Plateau)	22557	79	1 well for every 286 km^2	−0.10	24%	0%
Tinguiririca Inferior Aquifer (Tinguiririca Aquifer)	643	1	1 well for every 643 km^2	−0.10	0%	0%
Spokane Valley-Rathdrum Prairie Aquifer (Columbia Plateau Regional Aquifer System)	1750	6	1 well for every 292 km^2	−0.10	17%	0%
Southern Culverden Basin (Culverden Basin)	295	1	1 well for every 295 km^2	−0.10	0%	0%
Bonifacio Coastal Aquifer	30	1	1 well for every 30 km^2	−0.10	0%	0%
Eastern Pennyroyal Plateau (Interior Low Plateaus)	16219	31	1 well for every 523 km^2	−0.10	0%	0%
Blue River Basin	217	1	1 well for every 217 km^2	−0.10	0%	0%
Central Guadalquivir Basin (Guadalquivir Basin)	6540	22	1 well for every 297 km^2	−0.11	23%	14%
Kuzuryu Basin	554	11	1 well for every 50 km^2	−0.11	18%	0%
Southcentral Valley and Ridge (Valley and Ridge Aquifer System)	18344	1	1 well for every 18344 km^2	−0.11	0%	0%
Vamoosa Ada Aquifer	7278	8	1 well for every 910 km^2	−0.11	25%	0%
Arrowsmith and Dandaragan Plateau (Perth Basin)	12802	131	1 well for every 98 km^2	−0.11	23%	2%
Tajuna-Montes Universales Aquifer	3434	9	1 well for every 382 km^2	−0.11	33%	0%
Northern Taichung Basin (Taichung Basin)	290	13	1 well for every 22 km^2	−0.11	8%	0%
Rio Verde Basin	12	1	1 well for every 12 km^2	−0.11	0%	0%
Northern Shuicheng Basin	39	3	1 well for every 13 km^2	−0.11	33%	0%
Sabinas Aquifer	702	10	1 well for every 70 km^2	−0.11	40%	0%
Pend Orielle West	567	4	1 well for every 142 km^2	−0.12	25%	0%
Somontano del Moncayo Aquifer	648	13	1 well for every 50 km^2	−0.12	31%	8%
Lower Meadow Valley Wash	556	1	1 well for every 556 km^2	−0.12	0%	0%
Houston-Galveston Area (Gulf Coast Regional Aquifer System)	29004	863	1 well for every 34 km^2	−0.12	38%	15%
Southern Downs Chalk Aquifer (Chalk Aquifer System)	1403	1	1 well for every 1403 km^2	−0.12	0%	0%
Northern Hualien-Taitung Valley (Hualien-Taitung Valley)	344	10	1 well for every 34 km^2	−0.12	30%	0%
Sabinas Paras Aquifer	4339	1	1 well for every 4339 km^2	−0.12	0%	0%
Kunming Urban Area	630	7	1 well for every 90 km^2	−0.13	29%	14%
Bierzo Basin	306	2	1 well for every 153 km^2	−0.13	0%	0%
Little Spokane Basin (Columbia Plateau Regional Aquifer System)	1647	3	1 well for every 549 km^2	−0.13	33%	0%
Southern Maryborough Basin	973	28	1 well for every 35 km^2	−0.14	4%	0%
Lishtar Basin	77	13	1 well for every 6 km^2	−0.14	31%	0%
Vega Baja and Media of the Segura River Basin	1331	11	1 well for every 121 km^2	−0.14	18%	0%
Southern Hualien-Taitung Valley (Hualien-Taitung Valley)	179	4	1 well for every 45 km^2	−0.14	0%	0%

Study area	Area (km^2)	Number of monitoring wells	Density of monitoring wells (with sufficient groundwater level observations for analyses) within the study area	Median Theil-Sen slope among monitoring wells *(positive values indicate deeper depths to water over time)*	Percent of wells with Theil-Sen slopes of >0 m per year	Percent of wells with Theil-Sen slopes of >0.5 m per year
Madrid-Talavera Detrital Aquifer	6065	65	1 well for every 93 km^2	−0.14	32%	3%
Logar Aquifer (Kabul Basin)	148	10	1 well for every 15 km^2	−0.15	30%	0%
Lipan Flats	3112	97	1 well for every 32 km^2	−0.15	22%	1%
Hualapai Flat	184	1	1 well for every 184 km^2	−0.15	0%	0%
Bangkok Basin (Lower Chao Phraya Basin)	13316	72	1 well for every 185 km^2	−0.16	39%	10%
Buena Vista Valley in northern Nevada	1439	1	1 well for every 1439 km^2	−0.16	0%	0%
Comarca Laguna Aquifer	4012	18	1 well for every 223 km^2	−0.16	44%	28%
Vega de Granada Aquifer	670	31	1 well for every 22 km^2	−0.16	13%	0%
Tlacolula Valley (Central Valleys of Oaxaca)	757	33	1 well for every 23 km^2	−0.17	21%	0%
Mezguita Oasis (Middle Draa Basin)	100	2	1 well for every 50 km^2	−0.17	0%	0%
Lower Borrego and Ocotillo-Clark Valleys	665	4	1 well for every 166 km^2	−0.17	25%	0%
West Salt River Basin (Salt Basin)	3005	25	1 well for every 120 km^2	−0.18	44%	0%
Rose Valley	118	11	1 well for every 11 km^2	−0.18	27%	0%
Dalmeny Aquifer	665	1	1 well for every 665 km^2	−0.18	0%	0%
Campo de Dalias Coastal Aquifer	368	14	1 well for every 26 km^2	−0.18	36%	14%
Western Galilee Basin	302	2	1 well for every 151 km^2	−0.18	0%	0%
Southwestern Chaco-Lower Parana Basin (Guarani Aquifer System)	330826	9	1 well for every 36758 km^2	−0.18	0%	0%
Vicente Guerrero-Poanas Aquifer	1544	1	1 well for every 1544 km^2	−0.19	0%	0%
Motril-Salobrena Aquifer	68	3	1 well for every 23 km^2	−0.19	0%	0%
Amazonas Basin and Alter de Chao Aquifer System (Amazon Aquifer System)	318913	1	1 well for every 318913 km^2	−0.19	0%	0%
Hidalgo Aquifer	826	10	1 well for every 83 km^2	−0.19	20%	0%
Southcentral Hualien-Taitung Valley (Hualien-Taitung Valley)	143	4	1 well for every 36 km^2	−0.20	25%	0%
Central Willamette Valley (Willamette Valley)	3947	8	1 well for every 493 km^2	−0.20	0%	0%
Yucaipa Basin	68	119	1 well for every 1 km^2	−0.20	30%	8%
Western Chikugo-Saga Plain (Chikugo-Saga Plain)	126	8	1 well for every 16 km^2	−0.21	0%	0%
Campina de Faro and Sao Joao da Venda Aquifers	331	2	1 well for every 166 km^2	−0.21	0%	0%
Wiso Basin (Daly-Wiso-Georgina Basins)	116966	1	1 well for every 116966 km^2	−0.22	0%	0%
Madeline Plains	768	1	1 well for every 768 km^2	−0.22	0%	0%
Malwa Plateau Gneiss Complex (Malwa Plateau)	23958	60	1 well for every 399 km^2	−0.23	30%	8%
Mulgildie Outlier (Great Artesian Basin)	1712	104	1 well for every 16 km^2	−0.24	5%	0%
Southern Huaihe River Basin (Huaihe River Basin)	29395	1	1 well for every 29395 km^2	−0.24	0%	0%
Western La Sabana Aquifer	154	2	1 well for every 77 km^2	−0.24	0%	0%
Gonzales-New Orleans Aquifer (Gulf Coast Regional Aquifer System)	1913	12	1 well for every 159 km^2	−0.25	0%	0%
Irwin Basin	47	59	1 well for every 1 km^2	−0.25	14%	0%
Lampazos Villaldama Aquifer	1033	40	1 well for every 26 km^2	−0.26	20%	5%
Pir-e-Sohrab Basin	828	5	1 well for every 166 km^2	−0.26	20%	0%
Chachaua Aquifer	125	3	1 well for every 42 km^2	−0.26	0%	0%
Jianghan Plain	34644	5	1 well for every 6929 km^2	−0.27	40%	0%
Sierra de Altomira Aquifer (Upper Guadiana Basin)	2672	11	1 well for every 243 km^2	−0.27	27%	9%
Weaber Plain	172	13	1 well for every 13 km^2	−0.27	0%	0%
Coastal Aquifer	3558	18	1 well for every 198 km^2	−0.27	6%	0%

Study area	Area (km^2)	Number of monitoring wells	Density of monitoring wells (with sufficient groundwater level observations for analyses) within the study area	Median Theil-Sen slope among monitoring wells *(positive values indicate deeper depths to water over time)*	Percent of wells with Theil-Sen slopes of >0 m per year	Percent of wells with Theil-Sen slopes of >0.5 m per year
Yasuj Basin	133	9	1 well for every 15 km^2	−0.28	33%	22%
Upper Feather River Basin (Sierra Nevada Foothills)	1257	7	1 well for every 180 km^2	−0.28	14%	0%
Mancha Oriental Aquifer System	5927	37	1 well for every 160 km^2	−0.28	24%	5%
Central Parana Basin and Torres Syncline (Guarani Aquifer System)	494871	12	1 well for every 41239 km^2	−0.29	8%	0%
Upper Kaweah Basin (Sierra Nevada Foothills)	1182	2	1 well for every 591 km^2	−0.29	0%	0%
Yangtze Delta at Shanghai	9101	25	1 well for every 364 km^2	−0.29	4%	0%
Costa de Hermosillo aquifer	4746	28	1 well for every 170 km^2	−0.29	29%	4%
Las Vegas Basin	1681	58	1 well for every 29 km^2	−0.31	28%	0%
Evan Plain (Dezful-Andimeshk Plain)	442	17	1 well for every 26 km^2	−0.32	6%	0%
Shomali Basin (Kabul Basin)	733	13	1 well for every 56 km^2	−0.32	8%	0%
Upper Santa Cruz Basin (Tucson Basin and Avra Valley)	4163	47	1 well for every 89 km^2	−0.32	11%	6%
Round Valley	196	1	1 well for every 196 km^2	−0.33	0%	0%
Knox Creek Plain	253	14	1 well for every 18 km^2	−0.35	0%	0%
Ayamonte-Huelva Coastal Aquifer	264	7	1 well for every 38 km^2	−0.38	0%	0%
Eastern Malwa Plateau Alluvial Aquifer (Malwa Plateau)	12262	26	1 well for every 472 km^2	−0.40	42%	15%
Nochixtlan Aquifer	223	13	1 well for every 17 km^2	−0.40	15%	8%
Lake Frome Embayment (Great Artesian Basin)	34046	1	1 well for every 34046 km^2	−0.55	0%	0%
Moraleja Aquifer	227	2	1 well for every 114 km^2	−0.58	0%	0%
Sinclinal de Calasparra Aquifer	317	5	1 well for every 63 km^2	−0.58	0%	0%
Lordsburg Valley	839	4	1 well for every 210 km^2	−0.62	0%	0%
Baoju Uplift (Weihe Basin)	2959	6	1 well for every 493 km^2	−0.64	33%	0%
Eastern Washoe Valley	25	1	1 well for every 25 km^2	−0.71	0%	0%
East Salt River Basin (Salt Basin)	4156	7	1 well for every 594 km^2	−0.78	29%	14%
Abbas -e Sharghi Basin	385	28	1 well for every 14 km^2	−0.84	4%	0%
Mancha Occidental Aquifer (Upper Guadiana Basin)	5625	53	1 well for every 106 km^2	−0.92	2%	2%
Minqin Basin (Shiyang Basin)	3326	2	1 well for every 1663 km^2	−1.29	50%	0%
Campo Mina Aquifer	304	13	1 well for every 23 km^2	−1.59	0%	0%
Ganuelas-Mazarron Basin	153	3	1 well for every 51 km^2	−1.99	0%	0%
Taiyuan Basin	6012	5	1 well for every 1202 km^2	−2.58	0%	0%
